# Supplementary material for: Transcriptomic-Metabolomic Profiling in Mouse Lung Tissues Reveals Sex- and Strain-Based Differences
Source: Metabolites. 2022 Sep 30;12(10):932. doi: 10.3390/metabo12100932 (PMC9612261; doi:10.3390/metabo12100932)
Supplement: Supplementary file 1 [file metabolites-12-00932-s001.zip › metabolites-1924425-supplementary.pdf]

Table S1a. Significant features for strain (2 way limma anova test, limma decide test, P<0.05)

P.value	Adjusted.P.value	Decide test	m/z	RT(sec)
<0.01	0.019	-1	91.9624	197.7
<0.01	0.046	-1	98.9614	259.7
<0.01	0.041	-1	101.0599	33.5
<0.01	0.038	-1	105.9780	196.8
<0.01	0.038	-1	114.0318	52.2
<0.01	<0.01	-1	116.0621	27.6
<0.01	0.049	-1	117.0021	34.1
<0.01	<0.01	-1	117.0659	59.4
<0.01	0.014	-1	120.0196	45.0
<0.01	<0.01	-1	122.0142	197.5
<0.01	<0.01	-1	125.0152	26.7
<0.01	0.037	-1	126.5009	111.6
<0.01	0.043	-1	127.5088	91.5
<0.01	0.021	-1	128.5101	34.0
<0.01	0.044	-1	129.0925	186.4
<0.01	0.026	-1	131.0416	160.1
<0.01	<0.01	-1	132.4987	53.0
<0.01	0.026	-1	132.5222	84.8
<0.01	<0.01	-1	133.0148	31.9
<0.01	<0.01	-1	133.0591	290.6
<0.01	0.046	-1	133.9269	61.7
<0.01	0.020	-1	136.4964	34.3
<0.01	0.036	-1	136.5140	91.9
<0.01	<0.01	-1	137.0157	91.2
<0.01	0.037	-1	138.0429	131.9
<0.01	0.044	-1	139.5310	85.3
<0.01	<0.01	-1	140.0256	26.4
<0.01	0.033	-1	140.0330	82.9
<0.01	0.041	-1	140.5127	87.3
<0.01	<0.01	-1	140.5222	100.1
<0.01	0.025	-1	140.5332	88.8
<0.01	0.020	-1	141.5275	88.0
<0.01	0.019	-1	144.0307	100.1
<0.01	0.016	-1	145.0151	26.1
<0.01	<0.01	-1	146.1176	39.2
<0.01	<0.01	-1	147.0228	57.2
<0.01	0.027	-1	147.0257	197.0
<0.01	0.036	-1	147.0304	37.7
<0.01	<0.01	-1	147.1210	39.7
<0.01	0.029	-1	148.0221	92.2
<0.01	0.036	-1	148.0229	35.2
<0.01	<0.01	-1	148.0276	47.2
<0.01	<0.01	-1	148.5237	91.5
<0.01	<0.01	-1	148.5363	90.2
<0.01	<0.01	-1	151.0296	18.3
<0.01	0.031	-1	151.0390	84.4
<0.01	<0.01	-1	151.0696	289.0
<0.01	0.017	-1	151.5407	87.8
<0.01	0.031	-1	152.0566	179.6
<0.01	<0.01	-1	153.0277	17.9
<0.01	0.028	-1	153.0541	180.4
<0.01	<0.01	-1	154.0229	43.7
<0.01	0.019	-1	154.0377	38.3
<0.01	0.035	-1	155.0025	194.8
<0.01	0.024	-1	155.0912	172.6
<0.01	0.036	-1	157.0096	34.6
<0.01	0.030	-1	159.0436	54.5
<0.01	0.017	-1	160.5222	198.5
<0.01	0.020	-1	160.9998	99.6
<0.01	<0.01	-1	161.1285	83.6
<0.01	0.011	-1	162.0375	54.0
<0.01	<0.01	-1	162.0762	60.0
<0.01	0.012	-1	162.1319	82.4

<0.01	0.020	-1	163.0155	192.0						
<0.01	<0.01	-1	163.0866	33.2						
<0.01	<0.01	-1	164.0342	24.6						
<0.01	<0.01	-1	164.0561	61.1						
<0.01	<0.01	-1	169.0844	32.2						
<0.01	0.033	-1	169.5274	197.0						
<0.01	<0.01	-1	170.0051	100.3						
<0.01	0.016	-1	175.0076	33.2						
<0.01	<0.01	-1	175.1440	88.0						
<0.01	0.012	-1	175.1481	160.6						
<0.01	0.037	-1	176.4972	92.0						
<0.01	<0.01	-1	177.0409	30.0						
<0.01	0.027	-1	177.0466	192.2						
<0.01	<0.01	-1	178.0444	33.0						
<0.01	0.024	-1	179.0025	196.1						
<0.01	0.018	-1	179.0044	57.5						
<0.01	0.033	-1	179.5142	88.6						
<0.01	<0.01	-1	180.5168	75.3						
<0.01	<0.01	-1	180.5220	96.7						
<0.01	0.023	-1	181.0443	198.2						
<0.01	<0.01	-1	181.0794	24.6						
<0.01	0.027	-1	182.9814	76.6						
<0.01	0.044	-1	183.5386	197.1						
<0.01	0.037	-1	186.0855	268.0						
<0.01	0.038	-1	187.0536	197.9						
<0.01	<0.01	-1	187.1441	244.1						
<0.01	0.010	-1	188.0376	198.3						
<0.01	0.020	-1	189.0315	24.9						
<0.01	0.025	-1	190.5119	136.5						
<0.01	0.043	-1	191.5406	86.1						
<0.01	0.041	-1	191.5502	196.3						
<0.01	0.036	-1	196.0130	34.2						
<0.01	0.030	-1	196.0194	92.0						
<0.01	0.011	-1	198.0184	99.2						
<0.01	<0.01	-1	198.1222	178.5						
<0.01	<0.01	-1	200.0442	79.5						
<0.01	<0.01	-1	200.5459	82.2						
<0.01	0.015	-1	201.5456	98.1						
<0.01	<0.01	-1	202.0200	138.0						
<0.01	0.020	-1	202.0407	86.5						
<0.01	<0.01	-1	205.0731	144.2						
<0.01	0.012	-1	205.1192	168.9						
<0.01	0.029	-1	205.5476	197.8						
<0.01	0.036	-1	206.0395	196.6						
<0.01	0.025	-1	206.0400	59.8						
<0.01	<0.01	-1	207.0386	27.2						
<0.01	0.027	-1	209.0781	43.6						
<0.01	0.027	-1	210.1270	21.8						
<0.01	0.042	-1	211.5172	117.9						
<0.01	0.022	-1	211.5523	90.0						
<0.01	0.015	-1	212.0537	89.3						
<0.01	0.015	-1	212.5048	101.4						
<0.01	0.044	-1	213.5487	87.2						
<0.01	0.021	-1	214.5528	198.3						
<0.01	0.023	-1	215.0543	196.3						
<0.01	0.020	-1	215.1137	84.0						
<0.01	0.031	-1	215.5507	196.1						
<0.01	0.027	-1	216.0567	197.8						
<0.01	0.024	-1	216.5141	131.1						
<0.01	0.015	-1	216.5506	197.7						
<0.01	0.029	-1	217.1065	190.2						
<0.01	<0.01	-1	217.5404	89.5						
<0.01	<0.01	-1	218.0421	89.6						
<0.01	0.024	-1	219.0176	85.1						
<0.01	0.039	-1	219.0417	100.1						
<0.01	0.028	-1	219.5434	99.9						

<0.01	0.015	-1	220.0440	100.1						
<0.01	0.037	-1	220.0482	81.4						
<0.01	0.033	-1	220.1373	135.6						
<0.01	0.024	-1	221.0095	192.8						
<0.01	<0.01	-1	221.0384	81.9						
<0.01	0.038	-1	221.9712	52.6						
<0.01	<0.01	-1	222.1077	24.4						
<0.01	0.034	-1	223.0254	168.8						
<0.01	0.015	-1	223.5634	100.6						
<0.01	0.031	-1	223.5643	155.8						
<0.01	0.039	-1	223.9644	56.0						
<0.01	<0.01	-1	224.0835	24.8						
<0.01	<0.01	-1	225.0869	26.6						
<0.01	<0.01	-1	225.0870	193.9						
<0.01	0.042	-1	225.3789	160.1						
<0.01	<0.01	-1	225.9797	31.1						
<0.01	<0.01	-1	226.0810	202.1						
<0.01	<0.01	-1	226.1017	85.5						
<0.01	<0.01	-1	226.1437	28.0						
<0.01	0.049	-1	228.3906	280.7						
<0.01	0.023	-1	231.0092	170.3						
<0.01	0.049	-1	231.0250	106.0						
<0.01	0.037	-1	231.3825	159.1						
<0.01	0.031	-1	231.9366	271.6						
<0.01	0.023	-1	232.1025	124.0						
<0.01	0.026	-1	233.0376	87.0						
<0.01	0.013	-1	233.0670	138.5						
<0.01	0.011	-1	234.7112	156.8						
<0.01	0.045	-1	235.1310	184.7						
<0.01	0.049	-1	235.1652	52.9						
<0.01	0.029	-1	235.5385	90.3						
<0.01	0.029	-1	235.5584	88.9						
<0.01	0.020	-1	236.0239	201.7						
<0.01	<0.01	-1	236.0837	200.1						
<0.01	0.028	-1	238.0193	193.3						
<0.01	0.031	-1	238.0395	197.6						
<0.01	<0.01	-1	238.0634	28.5						
<0.01	<0.01	-1	238.0991	21.7						
<0.01	0.046	-1	238.5639	96.6						
<0.01	0.035	-1	239.0656	96.2						
<0.01	<0.01	-1	239.1029	185.1						
<0.01	0.021	-1	239.5310	89.6						
<0.01	<0.01	-1	241.0295	64.5						
<0.01	0.043	-1	243.9441	57.1						
<0.01	0.029	-1	244.0412	178.4						
<0.01	0.045	-1	245.9682	77.0						
<0.01	0.018	-1	249.0327	88.2						
<0.01	0.026	-1	249.1057	191.5						
<0.01	<0.01	-1	250.4831	100.2						
<0.01	0.023	-1	253.6311	29.6						
<0.01	<0.01	-1	253.9937	81.3						
<0.01	0.023	-1	255.0649	115.5						
<0.01	0.014	-1	255.5531	127.6						
<0.01	0.044	-1	257.5380	96.3						
<0.01	0.016	-1	260.0442	89.2						
<0.01	0.015	-1	260.4811	104.2						
<0.01	0.046	-1	261.1809	74.3						
<0.01	<0.01	-1	261.8896	50.6						
<0.01	<0.01	-1	262.0373	156.6						
<0.01	<0.01	-1	262.0781	52.8						
<0.01	0.041	-1	263.0753	156.3						
<0.01	<0.01	-1	265.0230	76.9						
<0.01	0.031	-1	265.1401	71.2						
<0.01	0.020	-1	265.5689	89.3						
<0.01	0.012	-1	266.5289	89.4						
<0.01	0.037	-1	268.3438	168.4						

<0.01	0.036	-1	271.0673	211.0						
<0.01	<0.01	-1	271.0678	105.1						
<0.01	0.041	-1	271.1055	219.0						
<0.01	0.034	-1	271.9852	34.3						
<0.01	0.017	-1	273.1201	73.2						
<0.01	0.043	-1	274.1032	210.4						
<0.01	0.012	-1	275.0165	206.4						
<0.01	0.031	-1	276.0118	127.8						
<0.01	<0.01	-1	278.0308	88.7						
<0.01	<0.01	-1	278.0706	271.9						
<0.01	0.030	-1	279.0646	86.7						
<0.01	<0.01	-1	279.1919	52.5						
<0.01	0.037	-1	279.5664	88.0						
<0.01	<0.01	-1	280.0070	135.3						
<0.01	0.023	-1	280.0688	87.7						
<0.01	0.010	-1	280.5642	87.6						
<0.01	0.037	-1	280.5687	87.6						
<0.01	<0.01	-1	281.0706	88.0						
<0.01	<0.01	-1	281.5724	88.0						
<0.01	0.026	-1	281.9455	63.3						
<0.01	0.012	-1	283.0455	99.8						
<0.01	0.034	-1	283.5440	127.1						
<0.01	<0.01	-1	284.1026	155.0						
<0.01	<0.01	-1	285.1207	242.1						
<0.01	0.044	-1	285.9412	70.0						
<0.01	0.017	-1	287.0763	118.8						
<0.01	<0.01	-1	287.5924	90.2						
<0.01	0.049	-1	288.0696	266.3						
<0.01	0.045	-1	288.5711	271.8						
<0.01	0.017	-1	288.5920	109.1						
<0.01	0.031	-1	289.0874	80.3						
<0.01	<0.01	-1	289.5514	100.2						
<0.01	0.030	-1	290.0343	196.4						
<0.01	0.011	-1	290.0763	84.2						
<0.01	<0.01	-1	290.1347	64.3						
<0.01	<0.01	-1	290.1597	31.9						
<0.01	0.012	-1	291.0477	189.2						
<0.01	<0.01	-1	291.1381	64.4						
<0.01	0.021	-1	293.5525	183.9						
<0.01	0.013	-1	296.0494	76.1						
<0.01	0.029	-1	298.0966	28.5						
<0.01	<0.01	-1	298.2011	15.7						
<0.01	<0.01	-1	299.0820	108.2						
<0.01	<0.01	-1	299.0832	41.9						
<0.01	0.036	-1	300.0156	93.4						
<0.01	0.011	-1	300.0546	198.1						
<0.01	0.040	-1	300.0594	196.4						
<0.01	0.028	-1	300.1555	55.6						
<0.01	<0.01	-1	300.5333	126.1						
<0.01	0.017	-1	301.1144	64.7						
<0.01	0.013	-1	301.5769	88.6						
<0.01	<0.01	-1	303.5463	241.1						
<0.01	0.010	-1	304.9132	284.4						
<0.01	0.012	-1	304.9816	293.5						
<0.01	0.017	-1	305.8579	42.7						
<0.01	0.020	-1	305.9530	169.8						
<0.01	0.027	-1	306.1297	78.3						
<0.01	0.017	-1	308.5241	100.8						
<0.01	<0.01	-1	308.9626	31.2						
<0.01	<0.01	-1	309.0738	98.9						
<0.01	0.046	-1	309.5823	221.1						
<0.01	<0.01	-1	312.1164	63.6						
<0.01	<0.01	-1	312.2166	23.7						
<0.01	<0.01	-1	313.0171	135.0						
<0.01	0.037	-1	315.0804	246.8						
<0.01	0.020	-1	315.1741	255.7						

<0.01	0.029	-1	316.0418	212.9							
<0.01	0.035	-1	317.9978	89.0							
<0.01	<0.01	-1	320.9921	101.2							
<0.01	0.023	-1	321.5046	128.3							
<0.01	0.041	-1	321.5374	167.9							
<0.01	0.038	-1	321.5638	203.6							
<0.01	<0.01	-1	321.9970	101.9							
<0.01	0.024	-1	323.0330	199.5							
<0.01	0.030	-1	323.9729	75.5							
<0.01	0.015	-1	324.0222	98.1							
<0.01	0.016	-1	324.9964	293.0							
<0.01	0.030	-1	325.0430	117.6							
<0.01	0.041	-1	326.0636	141.2							
<0.01	0.041	-1	326.9944	63.8							
<0.01	0.016	-1	327.0591	268.9							
<0.01	0.015	-1	327.2893	186.6							
<0.01	0.017	-1	327.5609	258.5							
<0.01	0.016	-1	327.5964	158.4							
<0.01	0.046	-1	328.0984	158.9							
<0.01	0.015	-1	328.5943	165.3							
<0.01	0.035	-1	329.0951	166.5							
<0.01	0.028	-1	329.1398	24.4							
<0.01	<0.01	-1	330.0592	75.4							
<0.01	0.025	-1	331.9781	80.3							
<0.01	<0.01	-1	332.5895	161.6							
<0.01	<0.01	-1	334.0269	276.1							
<0.01	0.039	-1	334.0355	248.8							
<0.01	0.027	-1	334.5187	273.5							
<0.01	0.044	-1	334.5729	141.2							
<0.01	<0.01	-1	334.5863	138.5							
<0.01	0.016	-1	336.0479	185.8							
<0.01	0.034	-1	338.5815	159.6							
<0.01	0.029	-1	339.0554	92.3							
<0.01	0.014	-1	339.0701	120.7							
<0.01	0.042	-1	340.0522	198.0							
<0.01	0.049	-1	340.0585	205.3							
<0.01	0.014	-1	341.0023	286.0							
<0.01	<0.01	-1	342.6017	242.5							
<0.01	0.029	-1	342.9961	284.8							
<0.01	<0.01	-1	344.4302	107.8							
<0.01	0.048	-1	346.0317	266.1							
<0.01	0.030	-1	346.5300	182.0							
<0.01	0.030	-1	346.5700	158.9							
<0.01	0.018	-1	347.0716	160.3							
<0.01	0.030	-1	349.1176	145.3							
<0.01	0.028	-1	351.0590	133.7							
<0.01	0.032	-1	351.5626	273.3							
<0.01	0.011	-1	351.5630	155.9							
<0.01	<0.01	-1	351.9680	74.0							
<0.01	0.038	-1	352.0637	184.8							
<0.01	0.043	-1	353.0664	196.9							
<0.01	<0.01	-1	355.0377	271.6							
<0.01	0.011	-1	355.5301	126.3							
<0.01	0.016	-1	356.0316	120.2							
<0.01	<0.01	-1	357.0371	89.4							
<0.01	<0.01	-1	357.0747	135.7							
<0.01	0.016	-1	358.0988	221.1							
<0.01	<0.01	-1	358.6010	215.6							
<0.01	0.018	-1	359.5472	128.3							
<0.01	0.017	-1	360.0367	98.0							
<0.01	0.037	-1	360.0482	128.4							
<0.01	<0.01	-1	360.1184	242.9							
<0.01	0.036	-1	364.9707	90.7							
<0.01	0.012	-1	365.0010	62.3							
<0.01	0.011	-1	366.0008	73.8							
<0.01	0.020	-1	367.0623	197.0							

<0.01	0.023	-1	367.5359	127.3							
<0.01	0.013	-1	368.0279	129.9							
<0.01	0.014	-1	368.0376	127.6							
<0.01	0.011	-1	368.0603	183.5							
<0.01	0.029	-1	368.9558	189.0							
<0.01	<0.01	-1	369.2245	65.1							
<0.01	0.011	-1	369.5412	196.6							
<0.01	0.043	-1	370.5319	129.5							
<0.01	<0.01	-1	370.9686	58.1							
<0.01	0.018	-1	372.1060	258.7							
<0.01	0.037	-1	372.6074	263.7							
<0.01	0.034	-1	373.0107	97.2							
<0.01	0.011	-1	373.0208	90.0							
<0.01	0.020	-1	373.1036	258.5							
<0.01	<0.01	-1	374.0677	77.0							
<0.01	<0.01	-1	375.5334	167.8							
<0.01	0.037	-1	377.1226	89.8							
<0.01	0.038	-1	378.5207	128.5							
<0.01	<0.01	-1	378.5464	196.8							
<0.01	0.016	-1	378.9497	292.9							
<0.01	<0.01	-1	379.0343	100.4							
<0.01	0.023	-1	379.1044	252.3							
<0.01	0.038	-1	379.6060	252.6							
<0.01	0.016	-1	380.0582	197.2							
<0.01	0.043	-1	382.9816	91.8							
<0.01	0.012	-1	383.0389	168.7							
<0.01	<0.01	-1	383.5403	169.0							
<0.01	0.041	-1	384.0987	284.1							
<0.01	0.035	-1	386.5195	119.6							
<0.01	0.019	-1	386.9425	53.4							
<0.01	0.041	-1	387.0551	126.1							
<0.01	<0.01	-1	387.2636	25.9							
<0.01	0.011	-1	388.0274	59.9							
<0.01	0.024	-1	388.1095	251.9							
<0.01	0.024	-1	388.6111	252.8							
<0.01	0.044	-1	390.5777	256.0							
<0.01	<0.01	-1	391.0315	89.8							
<0.01	0.041	-1	391.1198	252.4							
<0.01	<0.01	-1	392.0349	89.5							
<0.01	<0.01	-1	392.1177	261.8							
<0.01	0.019	-1	394.5063	167.6							
<0.01	0.042	-1	395.0268	76.7							
<0.01	<0.01	-1	396.0551	171.2							
<0.01	0.016	-1	397.9337	67.8							
<0.01	0.012	-1	402.5138	168.3							
<0.01	0.027	-1	406.1457	84.2							
<0.01	0.018	-1	407.2497	22.2							
<0.01	0.023	-1	409.5513	258.0							
<0.01	0.012	-1	412.0754	197.8							
<0.01	0.037	-1	413.0684	197.7							
<0.01	0.041	-1	414.9717	88.5							
<0.01	0.016	-1	415.5174	88.9							
<0.01	<0.01	-1	417.1415	88.6							
<0.01	<0.01	-1	418.1564	70.9							
<0.01	0.048	-1	418.7478	23.8							
<0.01	<0.01	-1	419.7557	20.7							
<0.01	0.015	-1	422.3261	21.4							
<0.01	0.020	-1	423.3296	21.6							
<0.01	<0.01	-1	424.0647	100.1							
<0.01	0.011	-1	424.3419	16.8							
<0.01	0.015	-1	425.6029	140.5							
<0.01	0.012	-1	426.0238	91.0							
<0.01	0.020	-1	427.0951	196.3							
<0.01	0.038	-1	427.5091	128.7							
<0.01	0.042	-1	429.0824	85.6							
<0.01	0.045	-1	429.1018	196.1							

<0.01	0.038	-1	430.1031	196.5						
<0.01	0.021	-1	430.4040	21.5						
<0.01	0.045	-1	430.7479	22.6						
<0.01	0.015	-1	431.0978	196.3						
<0.01	0.025	-1	431.2489	23.5						
<0.01	0.027	-1	434.1916	113.6						
<0.01	0.039	-1	437.0763	99.8						
<0.01	0.036	-1	438.0797	99.9						
<0.01	<0.01	-1	445.3714	21.8						
<0.01	0.027	-1	446.0891	137.0						
<0.01	0.025	-1	446.1052	244.9						
<0.01	<0.01	-1	448.9609	76.5						
<0.01	0.012	-1	449.5440	100.3						
<0.01	<0.01	-1	449.9452	76.1						
<0.01	0.019	-1	455.0902	79.8						
<0.01	0.043	-1	456.0936	79.4						
<0.01	0.047	-1	456.9162	99.8						
<0.01	0.017	-1	457.0732	136.1						
<0.01	0.012	-1	457.1671	65.8						
<0.01	<0.01	-1	462.0117	100.1						
<0.01	<0.01	-1	462.1021	136.3						
<0.01	0.048	-1	462.1945	122.5						
<0.01	0.015	-1	463.1765	23.4						
<0.01	<0.01	-1	463.3247	22.5						
<0.01	<0.01	-1	463.6083	269.8						
<0.01	0.032	-1	464.0809	114.2						
<0.01	0.031	-1	465.0849	114.3						
<0.01	<0.01	-1	466.0863	114.4						
<0.01	0.024	-1	467.9266	52.2						
<0.01	<0.01	-1	469.0672	91.6						
<0.01	0.017	-1	469.3119	28.5						
<0.01	0.033	-1	470.1440	147.8						
<0.01	0.031	-1	471.0773	227.9						
<0.01	0.047	-1	479.1112	144.2						
<0.01	0.046	-1	480.6988	45.2						
<0.01	<0.01	-1	484.0115	168.8						
<0.01	0.029	-1	485.9559	58.3						
<0.01	0.019	-1	486.0865	92.6						
<0.01	0.017	-1	487.1657	141.6						
<0.01	0.033	-1	490.0736	129.7						
<0.01	0.046	-1	490.0993	282.5						
<0.01	<0.01	-1	493.0062	97.5						
<0.01	<0.01	-1	498.5343	99.6						
<0.01	<0.01	-1	500.1204	74.2						
<0.01	0.043	-1	500.9519	59.0						
<0.01	0.040	-1	504.0096	114.4						
<0.01	0.012	-1	504.0575	109.6						
<0.01	0.046	-1	505.0857	169.3						
<0.01	0.018	-1	507.5860	100.5						
<0.01	<0.01	-1	508.1108	115.3						
<0.01	0.024	-1	510.4513	21.3						
<0.01	0.036	-1	512.6036	286.0						
<0.01	0.012	-1	525.1445	159.5						
<0.01	<0.01	-1	526.5600	100.1						
<0.01	0.016	-1	530.8375	43.4						
<0.01	0.013	-1	532.0525	90.2						
<0.01	0.014	-1	545.0666	191.2						
<0.01	0.035	-1	548.4584	289.8						
<0.01	0.038	-1	548.9588	289.4						
<0.01	0.036	-1	549.1271	289.8						
<0.01	0.019	-1	549.5896	239.5						
<0.01	0.036	-1	552.9647	58.8						
<0.01	0.029	-1	557.1222	87.2						
<0.01	0.026	-1	558.1268	87.6						
<0.01	0.031	-1	559.1291	87.9						
<0.01	<0.01	-1	564.9666	57.7						

<0.01	0.029	-1	565.6298	101.4						
<0.01	0.041	-1	575.5012	293.9						
<0.01	0.019	-1	575.5015	24.2						
<0.01	0.029	-1	578.2521	110.6						
<0.01	<0.01	-1	579.0758	131.3						
<0.01	0.031	-1	579.0982	99.9						
<0.01	<0.01	-1	603.0292	100.0						
<0.01	0.011	-1	604.6292	43.7						
<0.01	0.038	-1	606.1794	117.0						
<0.01	<0.01	-1	617.3169	152.5						
<0.01	<0.01	-1	618.1095	112.9						
<0.01	0.011	-1	627.0836	99.8						
<0.01	<0.01	-1	630.0825	272.6						
<0.01	<0.01	-1	630.0867	274.4						
<0.01	0.017	-1	640.6846	264.4						
<0.01	<0.01	-1	653.1079	249.7						
<0.01	<0.01	-1	654.1060	188.5						
<0.01	<0.01	-1	654.1858	160.9						
<0.01	<0.01	-1	657.9460	290.4						
<0.01	0.020	-1	658.3494	289.4						
<0.01	<0.01	-1	658.5535	289.8						
<0.01	<0.01	-1	664.1635	155.2						
<0.01	0.035	-1	664.1728	160.7						
<0.01	<0.01	-1	672.1035	131.7						
<0.01	<0.01	-1	678.5030	38.7						
<0.01	0.015	-1	680.4986	47.4						
<0.01	<0.01	-1	696.1158	130.8						
<0.01	0.048	-1	706.5379	17.9						
<0.01	0.018	-1	707.5411	20.5						
<0.01	0.027	-1	712.0319	275.3						
<0.01	0.020	-1	712.1111	168.6						
<0.01	0.029	-1	718.1393	196.4						
<0.01	0.011	-1	719.1139	100.2						
<0.01	0.027	-1	722.5109	22.7						
<0.01	<0.01	-1	727.1224	169.2						
<0.01	0.035	-1	728.1285	169.3						
<0.01	<0.01	-1	738.5080	26.6						
<0.01	0.045	-1	740.5554	274.9						
<0.01	<0.01	-1	742.5386	34.0						
<0.01	0.027	-1	742.5753	23.4						
<0.01	0.037	-1	743.2057	246.4						
<0.01	<0.01	-1	744.5851	23.4						
<0.01	<0.01	-1	745.5578	23.3						
<0.01	<0.01	-1	770.7838	281.9						
<0.01	0.049	-1	796.6599	287.8						
<0.01	<0.01	-1	796.8018	288.1						
<0.01	0.012	-1	828.5750	291.9						
<0.01	0.036	-1	852.5503	22.7						
<0.01	0.040	1	86.0965	40.0						
<0.01	<0.01	1	87.0040	226.7						
<0.01	0.042	1	87.0998	38.4						
<0.01	0.012	1	88.0043	260.5						
<0.01	0.029	1	88.0216	54.0						
<0.01	0.037	1	88.9931	35.6						
<0.01	<0.01	1	91.0059	79.3						
<0.01	0.046	1	91.5075	72.8						
<0.01	0.046	1	92.0165	65.8						
<0.01	0.028	1	96.5149	178.5						
<0.01	0.032	1	98.0344	123.8						
<0.01	0.037	1	99.5122	259.7						
<0.01	0.040	1	100.0474	194.0						
<0.01	0.041	1	100.5099	256.8						
<0.01	0.016	1	102.0130	282.0						
<0.01	<0.01	1	104.0087	283.8						
<0.01	0.018	1	104.5264	84.1						
<0.01	0.016	1	105.9538	175.3						

<0.01	0.043	1	111.0305	66.5							
<0.01	0.020	1	111.0684	89.9							
<0.01	0.029	1	112.0870	83.8							
<0.01	<0.01	1	113.0904	86.1							
<0.01	0.038	1	114.0662	45.0							
<0.01	0.019	1	115.0867	77.7							
<0.01	0.015	1	115.5787	93.2							
<0.01	0.020	1	116.0707	70.2							
<0.01	0.015	1	117.0740	68.3							
<0.01	<0.01	1	118.0612	56.2							
<0.01	0.028	1	118.9674	265.7							
<0.01	0.016	1	120.0236	276.0							
<0.01	<0.01	1	122.0192	274.6							
<0.01	0.037	1	122.0714	61.0							
<0.01	<0.01	1	124.9564	15.5							
<0.01	0.024	1	125.9643	110.6							
<0.01	<0.01	1	125.9643	16.6							
<0.01	0.047	1	126.9720	267.2							
<0.01	<0.01	1	127.0326	61.5							
<0.01	0.036	1	127.0867	185.1							
<0.01	<0.01	1	127.9722	15.6							
<0.01	0.028	1	129.1024	85.9							
<0.01	0.030	1	129.5818	93.4							
<0.01	0.016	1	130.0863	73.4							
<0.01	0.020	1	131.0895	80.0							
<0.01	<0.01	1	131.9743	236.7							
<0.01	<0.01	1	132.0036	280.8							
<0.01	0.048	1	132.0272	111.6							
<0.01	0.019	1	132.4752	124.3							
<0.01	0.043	1	132.5288	111.7							
<0.01	0.012	1	133.0972	94.4							
<0.01	0.044	1	133.1053	43.1							
<0.01	0.038	1	133.5440	123.6							
<0.01	<0.01	1	133.5612	61.4							
<0.01	0.018	1	133.9302	100.3							
<0.01	0.042	1	134.0601	228.1							
<0.01	0.020	1	136.0487	55.5							
<0.01	0.047	1	137.9643	16.5							
<0.01	0.038	1	138.0275	88.1							
<0.01	0.041	1	138.9590	140.9							
<0.01	0.032	1	139.0974	189.2							
<0.01	<0.01	1	139.5122	85.9							
<0.01	0.047	1	139.9879	253.8							
<0.01	<0.01	1	140.0133	116.8							
<0.01	0.011	1	140.5099	87.7							
<0.01	<0.01	1	140.9513	110.0							
<0.01	<0.01	1	141.9514	88.1							
<0.01	0.032	1	142.9670	15.5							
<0.01	0.037	1	144.0631	52.5							
<0.01	0.024	1	144.0712	104.6							
<0.01	<0.01	1	145.0351	279.6							
<0.01	0.012	1	146.1652	122.3							
<0.01	<0.01	1	148.5174	87.8							
<0.01	<0.01	1	149.0186	114.1							
<0.01	0.014	1	149.5151	88.0							
<0.01	<0.01	1	150.0141	279.0							
<0.01	0.020	1	150.0187	111.2							
<0.01	0.049	1	152.9884	121.5							
<0.01	0.038	1	153.9592	17.5							
<0.01	0.037	1	156.0507	125.7							
<0.01	0.039	1	156.5184	104.3							
<0.01	<0.01	1	157.0608	59.9							
<0.01	0.033	1	157.1084	86.3							
<0.01	<0.01	1	157.5187	102.2							
<0.01	<0.01	1	158.0028	273.6							
<0.01	0.037	1	158.0060	89.2							

<0.01	0.023	1	158.0924	86.0							
<0.01	<0.01	1	158.5163	101.2							
<0.01	<0.01	1	160.0254	86.1							
<0.01	<0.01	1	160.5270	91.1							
<0.01	0.011	1	161.0230	87.8							
<0.01	0.020	1	161.5188	123.3							
<0.01	0.020	1	162.0502	53.4							
<0.01	0.041	1	166.0661	167.1							
<0.01	0.035	1	166.9545	16.1							
<0.01	0.011	1	167.5696	69.2							
<0.01	0.014	1	168.0438	55.3							
<0.01	0.010	1	170.0925	86.6							
<0.01	0.011	1	171.0105	256.4							
<0.01	<0.01	1	171.0958	87.5							
<0.01	0.016	1	171.9904	69.3							
<0.01	0.030	1	173.0210	178.6							
<0.01	0.046	1	174.9915	267.3							
<0.01	0.013	1	176.0658	52.2							
<0.01	0.029	1	176.0706	247.2							
<0.01	0.020	1	176.1224	83.8							
<0.01	0.038	1	176.1281	193.9							
<0.01	0.018	1	177.1232	86.3							
<0.01	0.029	1	177.5093	124.0							
<0.01	<0.01	1	178.0240	46.3							
<0.01	0.012	1	178.0327	113.1							
<0.01	0.036	1	178.0896	15.4							
<0.01	0.036	1	178.0983	265.1							
<0.01	0.018	1	178.5335	101.1							
<0.01	0.038	1	178.9750	34.9							
<0.01	<0.01	1	179.0295	104.8							
<0.01	0.045	1	179.9813	142.1							
<0.01	<0.01	1	180.0034	61.6							
<0.01	0.035	1	181.0089	51.0							
<0.01	<0.01	1	186.1060	62.8							
<0.01	<0.01	1	186.1489	181.8							
<0.01	0.021	1	186.9563	165.9							
<0.01	0.044	1	187.0974	16.6							
<0.01	0.029	1	187.5471	127.7							
<0.01	<0.01	1	190.0894	238.8							
<0.01	0.020	1	190.1437	248.2							
<0.01	<0.01	1	191.0485	31.9							
<0.01	0.049	1	191.0976	32.4							
<0.01	0.011	1	192.0397	45.2							
<0.01	0.034	1	193.0019	268.3							
<0.01	0.036	1	193.0971	190.1							
<0.01	<0.01	1	193.9980	44.5							
<0.01	0.034	1	194.0224	50.5							
<0.01	<0.01	1	195.0877	22.5							
<0.01	<0.01	1	195.9305	100.3							
<0.01	0.016	1	195.9962	44.3							
<0.01	0.011	1	197.9259	102.4							
<0.01	0.047	1	197.9317	75.5							
<0.01	0.016	1	197.9775	85.9							
<0.01	0.045	1	198.0761	226.1							
<0.01	<0.01	1	198.1236	75.3							
<0.01	<0.01	1	198.9262	79.4							
<0.01	0.036	1	199.1350	145.2							
<0.01	0.038	1	199.6366	158.7							
<0.01	<0.01	1	199.9235	78.3							
<0.01	0.034	1	200.1645	88.2							
<0.01	0.045	1	203.4950	124.0							
<0.01	<0.01	1	206.0456	104.1							
<0.01	<0.01	1	206.5071	100.6							
<0.01	<0.01	1	207.0419	197.9							
<0.01	0.024	1	207.9463	80.7							
<0.01	0.041	1	208.0970	169.1							

<0.01	<0.01	1	208.1400	282.4						
<0.01	0.034	1	210.0118	42.8						
<0.01	0.011	1	210.0503	47.0						
<0.01	<0.01	1	210.0913	176.7						
<0.01	0.047	1	210.8993	158.9						
<0.01	0.045	1	212.0333	98.0						
<0.01	<0.01	1	212.0917	210.7						
<0.01	0.011	1	212.1610	129.4						
<0.01	<0.01	1	213.9410	118.0						
<0.01	<0.01	1	215.9365	94.8						
<0.01	0.044	1	216.0179	267.3						
<0.01	0.032	1	217.1949	251.9						
<0.01	<0.01	1	217.5179	261.9						
<0.01	0.037	1	222.0204	113.2						
<0.01	0.038	1	223.9886	142.5						
<0.01	<0.01	1	224.0895	52.1						
<0.01	0.018	1	224.1281	180.0						
<0.01	0.014	1	226.0241	45.1						
<0.01	0.029	1	226.1187	70.4						
<0.01	0.049	1	228.0281	102.8						
<0.01	0.011	1	228.0801	61.3						
<0.01	0.012	1	228.1018	261.0						
<0.01	0.020	1	229.1053	188.7						
<0.01	<0.01	1	229.5897	154.2						
<0.01	<0.01	1	230.0958	62.5						
<0.01	<0.01	1	230.5390	169.6						
<0.01	<0.01	1	230.9220	54.3						
<0.01	0.036	1	230.9623	78.8						
<0.01	0.014	1	231.0988	62.8						
<0.01	<0.01	1	232.0916	62.4						
<0.01	<0.01	1	232.1727	132.1						
<0.01	0.040	1	234.9333	80.3						
<0.01	0.019	1	234.9818	48.3						
<0.01	<0.01	1	235.9621	271.8						
<0.01	0.046	1	236.0556	259.9						
<0.01	0.024	1	236.6476	68.6						
<0.01	0.029	1	236.9411	125.6						
<0.01	0.043	1	236.9568	246.1						
<0.01	<0.01	1	236.9570	103.0						
<0.01	0.038	1	237.3293	278.9						
<0.01	0.030	1	237.6373	69.7						
<0.01	0.023	1	237.9551	84.5						
<0.01	0.048	1	238.2246	184.8						
<0.01	0.016	1	238.9525	104.5						
<0.01	0.024	1	238.9742	75.1						
<0.01	<0.01	1	239.1638	22.0						
<0.01	<0.01	1	244.1542	29.7						
<0.01	0.030	1	245.1383	188.2						
<0.01	<0.01	1	246.1698	28.9						
<0.01	0.025	1	247.1005	232.7						
<0.01	<0.01	1	247.1731	28.9						
<0.01	0.034	1	251.9410	112.5						
<0.01	0.037	1	252.0482	123.7						
<0.01	<0.01	1	252.0778	55.1						
<0.01	<0.01	1	252.1442	67.0						
<0.01	<0.01	1	253.0811	60.0						
<0.01	0.049	1	253.8786	78.8						
<0.01	<0.01	1	254.0737	58.6						
<0.01	<0.01	1	256.0968	165.8						
<0.01	0.025	1	256.9047	270.2						
<0.01	0.014	1	263.1224	171.7						
<0.01	<0.01	1	265.1116	60.3						
<0.01	<0.01	1	266.1150	61.0						
<0.01	0.041	1	267.0779	160.9						
<0.01	<0.01	1	268.0518	62.2						
<0.01	<0.01	1	269.0550	61.6						

<0.01	0.034	1	269.1613	60.5						
<0.01	<0.01	1	270.0497	61.6						
<0.01	<0.01	1	270.8913	78.1						
<0.01	0.015	1	270.9581	48.5						
<0.01	0.038	1	270.9776	57.4						
<0.01	<0.01	1	272.1253	89.7						
<0.01	<0.01	1	272.9551	48.5						
<0.01	<0.01	1	274.0597	59.7						
<0.01	0.039	1	274.0854	66.3						
<0.01	0.013	1	276.9226	47.5						
<0.01	0.029	1	278.0169	87.8						
<0.01	0.030	1	280.2350	242.0						
<0.01	0.041	1	280.6546	26.6						
<0.01	0.047	1	280.9734	181.9						
<0.01	0.039	1	281.1562	26.8						
<0.01	0.011	1	284.5899	127.4						
<0.01	<0.01	1	285.0911	128.9						
<0.01	<0.01	1	286.0697	102.1						
<0.01	0.030	1	286.1510	92.0						
<0.01	0.025	1	286.9320	49.0						
<0.01	0.026	1	288.9291	48.9						
<0.01	0.011	1	289.0403	47.9						
<0.01	<0.01	1	290.0336	60.5						
<0.01	0.011	1	290.6682	113.5						
<0.01	<0.01	1	292.5786	128.3						
<0.01	0.029	1	292.9400	48.6						
<0.01	0.034	1	293.0804	128.0						
<0.01	<0.01	1	293.9073	76.7						
<0.01	0.011	1	295.0305	53.4						
<0.01	<0.01	1	295.8292	259.9						
<0.01	<0.01	1	295.9028	77.5						
<0.01	0.015	1	297.9038	123.8						
<0.01	0.021	1	299.9371	126.1						
<0.01	0.041	1	301.9711	72.4						
<0.01	0.034	1	306.9979	279.6						
<0.01	0.022	1	309.4842	256.1						
<0.01	0.038	1	313.2732	286.8						
<0.01	<0.01	1	314.1711	256.5						
<0.01	0.038	1	315.9145	125.5						
<0.01	0.011	1	318.1679	74.2						
<0.01	0.025	1	319.0614	264.8						
<0.01	0.019	1	319.2245	47.2						
<0.01	<0.01	1	320.1022	272.9						
<0.01	0.011	1	322.2432	52.2						
<0.01	0.012	1	326.9176	89.0						
<0.01	<0.01	1	327.0487	73.5						
<0.01	0.012	1	328.9165	48.5						
<0.01	0.020	1	328.9581	165.3						
<0.01	0.029	1	330.6873	76.3						
<0.01	<0.01	1	332.5616	253.3						
<0.01	<0.01	1	333.5640	254.8						
<0.01	0.011	1	334.9673	86.5						
<0.01	0.041	1	335.1982	47.8						
<0.01	<0.01	1	336.9632	88.7						
<0.01	0.030	1	337.2138	47.9						
<0.01	0.019	1	341.8632	44.2						
<0.01	0.015	1	343.3116	195.4						
<0.01	0.013	1	347.1423	188.9						
<0.01	0.044	1	348.0074	279.7						
<0.01	0.020	1	349.1355	168.6						
<0.01	0.043	1	350.1388	168.9						
<0.01	0.011	1	350.8579	76.2						
<0.01	0.031	1	351.8557	75.0						
<0.01	0.011	1	352.8533	78.0						
<0.01	0.037	1	353.2668	42.9						
<0.01	0.022	1	354.3364	190.9						

<0.01	<0.01	1	355.1105	63.6							
<0.01	<0.01	1	366.1396	77.5							
<0.01	0.020	1	366.8723	48.7							
<0.01	0.046	1	367.2454	51.8							
<0.01	0.050	1	369.2632	257.9							
<0.01	0.018	1	369.4879	278.8							
<0.01	0.024	1	369.9600	88.4							
<0.01	0.031	1	370.3031	267.3							
<0.01	<0.01	1	372.2825	197.6							
<0.01	0.038	1	377.0926	63.0							
<0.01	0.027	1	378.8124	20.4							
<0.01	0.030	1	380.9834	165.7							
<0.01	0.044	1	381.1881	31.8							
<0.01	0.025	1	381.2979	39.1							
<0.01	0.036	1	382.3012	42.1							
<0.01	0.033	1	383.3155	171.1							
<0.01	0.021	1	383.3305	21.6							
<0.01	<0.01	1	384.1152	37.5							
<0.01	<0.01	1	385.1185	37.5							
<0.01	0.025	1	385.2947	192.2							
<0.01	0.011	1	386.0822	280.9							
<0.01	0.027	1	387.0070	261.1							
<0.01	0.024	1	387.0188	261.7							
<0.01	0.038	1	391.8602	43.5							
<0.01	0.021	1	395.5513	117.4							
<0.01	<0.01	1	397.2712	45.3							
<0.01	0.040	1	398.7613	283.8							
<0.01	<0.01	1	399.0740	61.8							
<0.01	0.028	1	402.4694	278.2							
<0.01	<0.01	1	411.2507	42.9							
<0.01	<0.01	1	414.5240	261.3							
<0.01	<0.01	1	415.0485	63.9							
<0.01	0.028	1	421.0562	60.0							
<0.01	<0.01	1	424.7321	67.7							
<0.01	0.037	1	424.7322	59.9							
<0.01	<0.01	1	429.2401	45.9							
<0.01	<0.01	1	431.0025	260.9							
<0.01	0.027	1	431.0727	202.1							
<0.01	0.020	1	431.3883	21.1							
<0.01	<0.01	1	431.7297	78.5							
<0.01	0.012	1	432.9443	86.5							
<0.01	<0.01	1	434.6907	47.7							
<0.01	0.025	1	444.7194	47.7							
<0.01	0.043	1	444.9604	279.6							
<0.01	0.045	1	449.2559	73.4							
<0.01	<0.01	1	449.7626	52.0							
<0.01	<0.01	1	457.0201	51.4							
<0.01	0.045	1	457.1679	149.5							
<0.01	<0.01	1	460.8078	48.5							
<0.01	0.030	1	462.9706	279.5							
<0.01	<0.01	1	471.1039	259.7							
<0.01	0.041	1	473.1781	39.7							
<0.01	0.013	1	478.0737	63.4							
<0.01	0.015	1	478.2355	266.2							
<0.01	0.040	1	478.4014	283.2							
<0.01	0.016	1	482.3604	27.9							
<0.01	<0.01	1	483.3638	28.4							
<0.01	0.042	1	484.2766	92.8							
<0.01	0.011	1	496.3393	18.6							
<0.01	<0.01	1	497.3428	19.3							
<0.01	0.022	1	498.6844	47.7							
<0.01	<0.01	1	499.3482	27.3							
<0.01	0.047	1	499.9716	57.4							
<0.01	0.020	1	506.6568	252.8							
<0.01	<0.01	1	507.1565	257.7							
<0.01	0.035	1	508.3396	26.0							

<0.01	<0.01	1	510.3552	24.6						
<0.01	0.029	1	510.6204	47.5						
<0.01	0.035	1	510.9931	57.1						
<0.01	<0.01	1	511.3585	25.0						
<0.01	<0.01	1	515.3132	27.3						
<0.01	0.012	1	515.8153	27.8						
<0.01	0.023	1	521.3431	25.7						
<0.01	0.022	1	522.3552	21.2						
<0.01	<0.01	1	523.2731	285.4						
<0.01	0.015	1	523.2995	26.5						
<0.01	0.021	1	523.3608	25.1						
<0.01	0.036	1	524.3706	26.0						
<0.01	0.039	1	526.3786	25.9						
<0.01	<0.01	1	527.3165	29.9						
<0.01	0.020	1	527.3806	26.8						
<0.01	0.026	1	529.3283	27.2						
<0.01	0.027	1	529.8307	27.4						
<0.01	<0.01	1	530.2177	70.6						
<0.01	<0.01	1	530.2865	23.4						
<0.01	<0.01	1	530.7692	47.9						
<0.01	<0.01	1	533.2767	259.4						
<0.01	<0.01	1	534.8024	253.7						
<0.01	<0.01	1	538.3864	25.5						
<0.01	0.028	1	540.3669	28.8						
<0.01	0.027	1	545.3428	24.3						
<0.01	0.038	1	545.6387	50.6						
<0.01	0.011	1	546.0706	61.0						
<0.01	<0.01	1	546.3546	24.5						
<0.01	0.017	1	547.3591	25.9						
<0.01	<0.01	1	548.3709	24.1						
<0.01	0.029	1	550.3878	26.3						
<0.01	<0.01	1	551.3895	26.6						
<0.01	0.035	1	558.2956	37.8						
<0.01	0.020	1	559.2998	38.7						
<0.01	0.048	1	560.3112	34.7						
<0.01	0.034	1	561.2845	277.8						
<0.01	<0.01	1	562.3270	39.1						
<0.01	0.040	1	563.3304	39.4						
<0.01	0.049	1	570.4579	22.3						
<0.01	0.020	1	580.0165	254.1						
<0.01	0.020	1	582.2963	38.9						
<0.01	0.038	1	586.5351	279.7						
<0.01	0.017	1	586.5940	47.4						
<0.01	<0.01	1	599.4412	30.4						
<0.01	0.021	1	605.3180	20.1						
<0.01	<0.01	1	606.3276	267.6						
<0.01	0.012	1	610.7415	274.8						
<0.01	0.041	1	613.1592	288.4						
<0.01	0.039	1	618.2569	42.9						
<0.01	<0.01	1	627.1216	61.7						
<0.01	<0.01	1	642.4707	284.6						
<0.01	0.045	1	662.5706	32.2						
<0.01	<0.01	1	664.1154	253.4						
<0.01	0.012	1	665.1168	253.9						
<0.01	0.038	1	673.6282	269.4						
<0.01	0.050	1	694.4736	47.0						
<0.01	0.045	1	695.0042	52.7						
<0.01	0.044	1	708.2556	221.4						
<0.01	<0.01	1	711.7312	256.3						
<0.01	0.044	1	719.5774	24.8						
<0.01	0.012	1	731.1622	280.1						
<0.01	0.038	1	749.1144	97.5						
<0.01	0.043	1	764.5501	22.4						
<0.01	0.031	1	832.5715	23.8						
<0.01	0.038	1	888.3588	280.9						
<0.01	0.017	1	957.4769	278.3						

**Note:** Decide test value "-1" or "1" indicates the t-statistic is classified as significantly negative or significantly positive respectively										

Table S1b. Significant features for gender (2 way limma anova test, limma decide test, P<0.05)

P.value	Adjusted.P.value	Decide test	m/z	RT(sec)
<0.01	0.049	1	86.06011	63.2
<0.01	0.040	1	86.0965	40.0
<0.01	<0.01	1	87.00403	226.7
<0.01	0.029	-1	88.02159	54.0
<0.01	0.015	-1	89.02494	49.9
<0.01	<0.01	-1	89.03864	29.2
<0.01	0.040	1	100.04742	194.0
<0.01	0.016	1	102.01299	282.0
<0.01	0.042	-1	102.0914	46.4
<0.01	0.049	-1	103.03905	50.1
<0.01	<0.01	-1	104.00871	283.8
<0.01	0.029	1	112.087	83.8
<0.01	0.049	1	113.02265	234.8
<0.01	<0.01	1	113.09038	86.1
<0.01	<0.01	-1	115.05425	28.4
<0.01	0.019	1	115.08668	77.7
<0.01	0.046	-1	116.03694	37.9
<0.01	<0.01	-1	116.05762	27.5
<0.01	<0.01	-1	116.06208	27.6
<0.01	0.020	1	116.07068	70.2
<0.01	0.047	-1	117.50434	80.5
<0.01	0.032	1	118.08628	56.0
<0.01	0.028	-1	120.05582	90.6
<0.01	0.036	1	121.01479	195.8
<0.01	<0.01	1	122.01419	197.5
<0.01	<0.01	1	122.01921	274.6
<0.01	<0.01	-1	125.01524	26.7
<0.01	0.049	-1	125.08356	189.4
<0.01	<0.01	-1	126.01862	25.3
<0.01	<0.01	-1	126.04698	48.6
<0.01	0.011	1	126.04745	129.8
<0.01	0.038	-1	126.10262	175.8
<0.01	<0.01	-1	126.10265	84.9
<0.01	0.037	1	126.50094	111.6
<0.01	<0.01	-1	127.01232	24.8
<0.01	0.017	-1	127.02148	44.6
<0.01	<0.01	-1	127.03255	61.5
<0.01	0.047	-1	127.05425	27.9
<0.01	0.043	-1	127.50878	91.5
<0.01	0.049	1	127.51743	28.3
<0.01	<0.01	-1	128.06204	24.3
<0.01	<0.01	-1	129.06987	24.2
<0.01	0.016	1	130.08633	73.4
<0.01	<0.01	1	130.12268	22.3
<0.01	<0.01	1	131.97428	236.7
<0.01	<0.01	1	132.00355	280.8
<0.01	<0.01	-1	132.49871	53.0
<0.01	<0.01	1	133.01476	31.9

<0.01	0.038	-1	133.08741	189.7						
<0.01	0.012	1	133.09718	94.4						
<0.01	0.027	-1	134.02707	58.0						
<0.01	0.032	-1	135.0304	52.5						
<0.01	0.036	-1	136.51402	91.9						
<0.01	<0.01	-1	137.0157	91.2						
<0.01	<0.01	-1	138.99425	89.5						
<0.01	<0.01	-1	139.0309	26.6						
<0.01	<0.01	1	140.01329	116.8						
<0.01	<0.01	-1	140.02557	26.4						
<0.01	<0.01	-1	140.52219	100.1						
<0.01	<0.01	-1	141.01023	24.5						
<0.01	0.032	1	141.01832	196.7						
<0.01	<0.01	-1	141.07081	43.2						
<0.01	<0.01	1	141.95144	88.1						
<0.01	<0.01	-1	142.07259	50.4						
<0.01	<0.01	-1	142.07769	24.3						
<0.01	<0.01	-1	143.08553	23.0						
<0.01	0.048	1	143.1066	23.9						
<0.01	<0.01	-1	144.05701	24.8						
<0.01	0.024	1	144.07117	104.6						
<0.01	0.030	1	144.08406	24.8						
<0.01	<0.01	-1	144.09338	24.5						
<0.01	<0.01	1	145.03514	279.6						
<0.01	<0.01	-1	145.06475	25.3						
<0.01	0.021	-1	145.07602	17.3						
<0.01	0.032	-1	145.09689	18.0						
<0.01	0.012	-1	147.09167	22.8						
<0.01	0.032	1	148.0427	42.3						
<0.01	<0.01	-1	148.52369	91.5						
<0.01	<0.01	1	149.01856	114.1						
<0.01	<0.01	1	150.01407	279.0						
<0.01	<0.01	-1	151.01228	26.1						
<0.01	<0.01	-1	151.02961	18.3						
<0.01	<0.01	-1	151.06957	289.0						
<0.01	<0.01	-1	152.02619	25.4						
<0.01	<0.01	-1	152.06226	31.7						
<0.01	0.040	1	152.58446	103.7						
<0.01	<0.01	-1	153.02774	17.9						
<0.01	<0.01	-1	153.06989	26.0						
<0.01	<0.01	-1	154.02294	43.7						
<0.01	<0.01	-1	154.07771	25.7						
<0.01	<0.01	-1	155.0854	21.7						
<0.01	0.030	-1	156.08079	26.7						
<0.01	0.015	1	156.08431	28.6						
<0.01	0.039	1	156.51837	104.3						
<0.01	0.047	-1	156.84524	40.3						
<0.01	<0.01	1	157.06078	59.9						
<0.01	0.033	1	157.10839	86.3						
<0.01	<0.01	1	158.00276	273.6						
<0.01	0.023	1	158.09239	86.0						

<0.01	<0.01	1	158.09982	26.1						
<0.01	0.037	1	159.09575	86.6						
<0.01	<0.01	1	159.10311	26.6						
<0.01	0.011	-1	162.03753	54.0						
<0.01	0.020	1	162.05023	53.4						
<0.01	<0.01	-1	163.03093	24.9						
<0.01	<0.01	-1	163.08658	33.2						
<0.01	<0.01	-1	164.03423	24.6						
<0.01	<0.01	-1	164.05609	61.1						
<0.01	<0.01	-1	165.01016	27.5						
<0.01	<0.01	-1	165.02797	24.9						
<0.01	<0.01	-1	165.04655	25.5						
<0.01	0.048	-1	165.11985	45.2						
<0.01	<0.01	-1	166.04991	26.3						
<0.01	<0.01	-1	167.00726	24.8						
<0.01	<0.01	-1	167.02608	27.6						
<0.01	0.014	-1	168.04378	55.3						
<0.01	<0.01	-1	168.08072	26.9						
<0.01	<0.01	-1	169.06482	28.2						
<0.01	<0.01	-1	169.08444	32.2						
<0.01	<0.01	-1	170.00508	100.3						
<0.01	<0.01	-1	170.07261	28.8						
<0.01	0.010	1	170.09249	86.6						
<0.01	0.011	-1	171.0105	256.4						
<0.01	0.012	-1	171.0804	24.9						
<0.01	<0.01	1	171.09579	87.5						
<0.01	0.040	1	171.1008	28.4						
<0.01	<0.01	-1	172.08827	24.6						
<0.01	0.038	1	174.99777	56.8						
<0.01	0.026	1	175.11893	85.1						
<0.01	0.012	-1	175.14813	160.6						
<0.01	0.029	1	176.07057	247.2						
<0.01	0.020	1	176.12235	83.8						
<0.01	<0.01	-1	177.04655	26.6						
<0.01	0.027	-1	177.04662	192.2						
<0.01	0.029	1	177.1314	53.2						
<0.01	0.024	1	179.00254	196.1						
<0.01	0.018	-1	179.00443	57.5						
<0.01	<0.01	-1	179.04359	27.4						
<0.01	<0.01	-1	179.06258	25.3						
<0.01	<0.01	1	180.00336	61.6						
<0.01	<0.01	-1	180.06548	25.8						
<0.01	0.037	1	180.99409	125.4						
<0.01	<0.01	-1	181.02288	26.2						
<0.01	0.031	-1	181.02375	96.8						
<0.01	<0.01	-1	181.05947	25.6						
<0.01	<0.01	-1	181.06893	25.4						
<0.01	<0.01	-1	181.07939	24.6						
<0.01	0.019	-1	186.09139	24.4						
<0.01	<0.01	-1	186.10603	62.8						
<0.01	<0.01	1	186.14887	181.8						

<0.01	0.021	1	186.95628	165.9						
<0.01	0.010	1	188.03762	198.3						
<0.01	<0.01	-1	189.04664	29.4						
<0.01	<0.01	-1	190.04987	29.3						
<0.01	<0.01	1	190.08943	238.8						
<0.01	0.020	1	190.14369	248.2						
<0.01	<0.01	-1	191.04387	27.6						
<0.01	<0.01	-1	191.04846	31.9						
<0.01	<0.01	-1	191.06215	48.2						
<0.01	0.011	1	192.03971	45.2						
<0.01	<0.01	-1	192.04693	25.6						
<0.01	0.034	1	193.00189	268.3						
<0.01	0.034	1	194.02238	50.5						
<0.01	0.037	1	194.0578	96.2						
<0.01	0.020	1	197.04104	66.9						
<0.01	0.023	-1	200.06836	44.3						
<0.01	0.034	-1	200.12155	68.0						
<0.01	0.014	-1	201.07177	44.4						
<0.01	<0.01	-1	202.01995	138.0						
<0.01	0.015	-1	202.06649	44.4						
<0.01	0.045	-1	202.52166	142.1						
<0.01	<0.01	-1	205.0416	27.0						
<0.01	0.031	1	205.06403	28.6						
<0.01	0.035	1	205.50688	101.0						
<0.01	0.036	1	206.0395	196.6						
<0.01	<0.01	-1	206.05458	24.9						
<0.01	<0.01	-1	206.07304	27.4						
<0.01	0.030	1	206.08097	24.8						
<0.01	<0.01	-1	207.03858	27.2						
<0.01	<0.01	1	207.04191	197.9						
<0.01	0.029	1	207.04313	104.3						
<0.01	<0.01	-1	207.07618	24.9						
<0.01	0.038	-1	208.04174	46.8						
<0.01	0.044	-1	208.06042	146.0						
<0.01	<0.01	-1	208.07001	49.9						
<0.01	<0.01	-1	209.05412	25.7						
<0.01	0.027	1	209.0781	43.6						
<0.01	0.034	1	210.01179	42.8						
<0.01	0.011	1	210.05027	47.0						
<0.01	<0.01	-1	210.05814	33.8						
<0.01	0.038	-1	211.0612	26.9						
<0.01	0.042	-1	211.51723	117.9						
<0.01	0.038	-1	212.09173	140.3						
<0.01	0.015	-1	212.50484	101.4						
<0.01	0.034	-1	213.01598	122.0						
<0.01	0.021	1	214.55278	198.3						
<0.01	0.031	1	215.55067	196.1						
<0.01	<0.01	-1	216.61192	22.9						
<0.01	0.035	-1	217.15462	36.0						
<0.01	<0.01	1	217.51791	261.9						
<0.01	0.016	-1	219.09714	53.8						

<0.01	0.028	-1	219.54338	99.9						
<0.01	0.015	-1	220.04403	100.1						
<0.01	<0.01	-1	220.08881	25.7						
<0.01	0.034	-1	221.00312	46.4						
<0.01	<0.01	-1	221.03836	81.9						
<0.01	<0.01	-1	221.09251	30.7						
<0.01	<0.01	1	221.09569	25.3						
<0.01	<0.01	-1	221.11038	286.8						
<0.01	0.043	1	221.12038	25.2						
<0.01	<0.01	1	221.14948	100.6						
<0.01	<0.01	-1	222.06804	25.8						
<0.01	<0.01	-1	222.08581	24.8						
<0.01	<0.01	-1	222.10772	24.4						
<0.01	<0.01	-1	222.11345	24.6						
<0.01	<0.01	-1	223.05205	26.5						
<0.01	<0.01	-1	223.08901	24.2						
<0.01	<0.01	-1	223.10692	28.3						
<0.01	0.015	-1	223.56336	100.6						
<0.01	0.038	1	223.98861	142.5						
<0.01	<0.01	-1	224.06475	29.2						
<0.01	<0.01	-1	224.0783	25.6						
<0.01	<0.01	-1	224.08345	24.8						
<0.01	<0.01	1	224.08952	52.1						
<0.01	<0.01	-1	225.08686	26.6						
<0.01	<0.01	-1	225.08699	193.9						
<0.01	0.011	-1	225.11256	23.3						
<0.01	0.014	1	226.02407	45.1						
<0.01	<0.01	-1	226.08099	202.1						
<0.01	<0.01	-1	226.08158	52.1						
<0.01	<0.01	-1	226.08983	25.4						
<0.01	<0.01	1	226.14368	28.0						
<0.01	0.046	1	227.03162	41.9						
<0.01	<0.01	-1	227.0837	25.3						
<0.01	<0.01	-1	227.11056	19.9						
<0.01	0.046	1	228.09792	132.2						
<0.01	0.028	1	228.97339	49.4						
<0.01	0.012	1	229.51932	125.9						
<0.01	<0.01	-1	230.92201	54.3						
<0.01	0.014	-1	231.09877	62.8						
<0.01	0.031	-1	231.93655	271.6						
<0.01	<0.01	-1	232.09156	62.4						
<0.01	0.031	1	233.1535	22.3						
<0.01	<0.01	-1	234.56847	125.6						
<0.01	0.019	1	234.98181	48.3						
<0.01	0.038	-1	235.03094	131.4						
<0.01	<0.01	-1	235.092	63.7						
<0.01	<0.01	-1	236.08405	22.1						
<0.01	0.029	1	236.94107	125.6						
<0.01	0.043	1	236.95679	246.1						
<0.01	<0.01	-1	237.10576	22.9						
<0.01	<0.01	-1	238.06343	28.5						

<0.01	<0.01	-1	238.0929	27.0						
<0.01	<0.01	-1	238.09912	21.7						
<0.01	<0.01	-1	238.10803	23.6						
<0.01	0.035	1	238.12005	22.5						
<0.01	0.044	1	239.00831	71.4						
<0.01	<0.01	-1	239.09677	27.3						
<0.01	<0.01	-1	239.10292	185.1						
<0.01	<0.01	-1	239.10442	18.9						
<0.01	<0.01	-1	240.07868	26.4						
<0.01	<0.01	-1	240.0902	25.3						
<0.01	<0.01	-1	240.1063	33.4						
<0.01	0.017	1	240.98846	121.5						
<0.01	<0.01	-1	241.08115	53.3						
<0.01	<0.01	-1	241.10195	43.8						
<0.01	0.013	-1	242.09386	27.3						
<0.01	<0.01	-1	242.10292	25.1						
<0.01	<0.01	-1	243.07876	27.5						
<0.01	0.029	1	244.04123	178.4						
<0.01	<0.01	1	244.15423	29.7						
<0.01	0.030	1	245.13829	188.2						
<0.01	0.024	1	245.14966	43.0						
<0.01	<0.01	-1	246.06488	57.0						
<0.01	0.037	1	248.14876	36.4						
<0.01	<0.01	1	249.05374	198.2						
<0.01	0.024	-1	249.10735	55.6						
<0.01	0.037	1	250.0934	30.2						
<0.01	0.017	1	250.15327	43.1						
<0.01	<0.01	-1	250.48312	100.2						
<0.01	0.032	1	251.15667	40.6						
<0.01	<0.01	1	252.14418	67.0						
<0.01	<0.01	-1	253.08105	60.0						
<0.01	<0.01	-1	254.07371	58.6						
<0.01	<0.01	-1	254.09409	25.7						
<0.01	<0.01	-1	254.10347	25.1						
<0.01	<0.01	-1	255.09748	43.6						
<0.01	<0.01	-1	256.09107	25.2						
<0.01	0.041	1	259.14011	86.3						
<0.01	<0.01	-1	260.08101	52.9						
<0.01	<0.01	1	261.88961	50.6						
<0.01	<0.01	1	262.03725	156.6						
<0.01	0.038	-1	262.03945	44.2						
<0.01	<0.01	-1	262.07814	52.8						
<0.01	<0.01	1	262.17431	25.1						
<0.01	0.014	1	263.12242	171.7						
<0.01	0.041	-1	265.07956	74.5						
<0.01	<0.01	-1	266.18601	23.0						
<0.01	<0.01	-1	268.05176	62.2						
<0.01	0.017	1	268.64992	27.6						
<0.01	0.047	-1	268.94586	41.7						
<0.01	<0.01	-1	269.05503	61.6						
<0.01	0.025	-1	269.09523	27.1						

<0.01	<0.01	-1	270.04971	61.6						
<0.01	0.040	-1	270.94422	41.9						
<0.01	0.015	1	270.95808	48.5						
<0.01	0.038	1	270.9776	57.4						
<0.01	0.011	1	271.0814	87.7						
<0.01	<0.01	-1	272.09223	57.2						
<0.01	<0.01	-1	272.1253	89.7						
<0.01	0.012	1	272.5222	261.2						
<0.01	<0.01	1	272.95509	48.5						
<0.01	0.013	1	276.92263	47.5						
<0.01	<0.01	-1	277.10239	54.0						
<0.01	<0.01	-1	279.09475	17.5						
<0.01	<0.01	-1	279.19185	52.5						
<0.01	0.034	1	280.14854	26.7						
<0.01	0.040	-1	284.02927	34.0						
<0.01	0.022	1	285.96767	59.0						
<0.01	<0.01	1	286.20102	25.1						
<0.01	0.036	1	286.57393	248.3						
<0.01	0.025	1	286.93198	49.0						
<0.01	0.049	1	287.06383	176.2						
<0.01	0.046	-1	287.98018	38.4						
<0.01	<0.01	1	288.21666	24.5						
<0.01	0.011	-1	289.04031	47.9						
<0.01	<0.01	-1	289.55139	100.2						
<0.01	0.030	1	290.03433	196.4						
<0.01	0.029	1	292.93996	48.6						
<0.01	0.021	-1	293.55245	183.9						
<0.01	0.032	-1	293.58145	128.8						
<0.01	0.011	-1	295.03049	53.4						
<0.01	<0.01	-1	295.82923	259.9						
<0.01	0.015	1	297.90383	123.8						
<0.01	<0.01	1	298.20108	15.7						
<0.01	<0.01	-1	299.0832	41.9						
<0.01	0.021	1	299.93709	126.1						
<0.01	0.036	-1	300.01564	93.4						
<0.01	0.017	-1	301.11436	64.7						
<0.01	<0.01	-1	303.54626	241.1						
<0.01	0.037	1	303.64685	26.7						
<0.01	0.010	1	304.47928	243.4						
<0.01	0.012	-1	304.98162	293.5						
<0.01	0.048	-1	305.01554	43.9						
<0.01	0.017	-1	308.52408	100.8						
<0.01	0.033	-1	308.89995	43.4						
<0.01	<0.01	-1	309.07375	98.9						
<0.01	<0.01	1	312.21655	23.7						
<0.01	<0.01	-1	313.01712	135.0						
<0.01	<0.01	1	314.23234	23.6						
<0.01	<0.01	-1	315.0563	35.7						
<0.01	0.032	1	315.13368	24.1						
<0.01	0.013	1	316.21148	28.0						
<0.01	0.050	1	317.2512	22.7						

<0.01	0.011	1	318.16785	74.2						
<0.01	<0.01	-1	320.99209	101.2						
<0.01	0.023	-1	321.50462	128.3						
<0.01	0.016	-1	324.99636	293.0						
<0.01	0.030	-1	325.04299	117.6						
<0.01	0.038	-1	326.04623	119.6						
<0.01	0.041	-1	326.06363	141.2						
<0.01	<0.01	-1	327.0487	73.5						
<0.01	0.015	1	327.28932	186.6						
<0.01	0.040	-1	328.24785	22.9						
<0.01	0.012	1	328.91654	48.5						
<0.01	<0.01	1	330.22731	26.7						
<0.01	0.030	-1	330.56287	167.2						
<0.01	0.025	-1	331.97809	80.3						
<0.01	0.021	1	332.24281	25.8						
<0.01	<0.01	1	332.56158	253.3						
<0.01	0.014	1	333.24622	25.9						
<0.01	<0.01	1	333.56404	254.8						
<0.01	<0.01	-1	334.58629	138.5						
<0.01	0.014	1	339.07011	120.7						
<0.01	0.034	1	339.65092	136.4						
<0.01	0.014	-1	341.00228	286.0						
<0.01	0.031	-1	341.15325	21.8						
<0.01	0.047	-1	341.49376	115.3						
<0.01	<0.01	-1	344.43016	107.8						
<0.01	0.024	-1	347.02513	123.5						
<0.01	0.044	-1	348.00741	279.7						
<0.01	0.035	1	348.93016	53.4						
<0.01	0.021	-1	349.03456	167.6						
<0.01	0.020	1	349.13551	168.6						
<0.01	0.046	1	351.23891	78.7						
<0.01	0.038	-1	353.46441	91.4						
<0.01	0.011	1	353.9879	49.8						
<0.01	<0.01	-1	355.11049	63.6						
<0.01	0.011	-1	355.53006	126.3						
<0.01	0.016	-1	356.03163	120.2						
<0.01	<0.01	-1	357.03708	89.4						
<0.01	0.035	-1	357.60715	192.0						
<0.01	0.024	1	358.25836	24.1						
<0.01	0.019	1	360.27415	23.9						
<0.01	0.040	1	361.13919	25.7						
<0.01	<0.01	1	361.27545	23.7						
<0.01	<0.01	-1	362.01992	56.8						
<0.01	0.042	1	362.02445	114.4						
<0.01	0.037	1	366.26361	22.1						
<0.01	0.020	1	366.87233	48.7						
<0.01	0.011	-1	368.06025	183.5						
<0.01	0.015	1	368.27928	22.1						
<0.01	0.043	-1	369.11831	108.4						
<0.01	<0.01	-1	369.22452	65.1						
<0.01	0.020	1	369.28268	22.1						

<0.01	0.038	1	371.29821	21.8						
<0.01	0.033	-1	373.98831	128.1						
<0.01	0.013	1	375.9701	50.1						
<0.01	0.016	-1	378.94972	292.9						
<0.01	<0.01	-1	379.03428	100.4						
<0.01	0.024	1	380.95079	60.2						
<0.01	0.048	-1	381.80117	50.7						
<0.01	<0.01	1	382.29551	24.9						
<0.01	0.021	1	383.33045	21.6						
<0.01	0.041	-1	384.09874	284.1						
<0.01	<0.01	1	384.11524	37.5						
<0.01	<0.01	1	384.27409	23.3						
<0.01	<0.01	1	385.11853	37.5						
<0.01	0.011	-1	386.08215	280.9						
<0.01	0.036	1	386.28967	22.8						
<0.01	0.035	-1	386.51947	119.6						
<0.01	<0.01	-1	387.26357	25.9						
<0.01	0.039	1	387.29309	22.7						
<0.01	0.018	1	388.30549	22.8						
<0.01	0.046	1	389.10712	254.0						
<0.01	0.024	1	389.30877	22.7						
<0.01	<0.01	1	390.31169	22.9						
<0.01	0.038	1	391.86018	43.5						
<0.01	0.042	-1	395.02682	76.7						
<0.01	<0.01	-1	396.05505	171.2						
<0.01	0.020	1	396.31037	21.8						
<0.01	0.037	1	398.32619	17.5						
<0.01	<0.01	-1	399.07395	61.8						
<0.01	0.044	1	399.32912	20.1						
<0.01	0.012	-1	402.51378	168.3						
<0.01	0.040	1	406.88892	50.5						
<0.01	0.018	1	407.24967	22.2						
<0.01	0.043	1	410.28938	22.7						
<0.01	0.021	-1	410.52672	90.5						
<0.01	0.012	1	412.07543	197.8						
<0.01	0.024	1	412.3053	22.4						
<0.01	0.023	1	413.30887	22.6						
<0.01	0.016	1	414.32123	22.2						
<0.01	<0.01	-1	415.04852	63.9						
<0.01	0.012	1	415.32456	22.4						
<0.01	<0.01	-1	415.79569	44.3						
<0.01	0.022	1	416.33668	21.9						
<0.01	<0.01	-1	417.14154	88.6						
<0.01	0.029	1	417.34026	22.1						
<0.01	0.047	1	417.7938	48.9						
<0.01	<0.01	-1	418.15637	70.9						
<0.01	0.037	1	418.34315	22.1						
<0.01	<0.01	1	419.75574	20.7						
<0.01	0.015	1	422.32608	21.4						
<0.01	0.020	1	423.32955	21.6						
<0.01	0.011	1	424.34185	16.8						

<0.01	0.043	1	425.34512	17.5						
<0.01	0.015	-1	425.60289	140.5						
<0.01	0.012	-1	426.02376	91.0						
<0.01	0.020	1	427.09506	196.3						
<0.01	0.045	1	429.10175	196.1						
<0.01	0.010	1	430.35234	22.1						
<0.01	0.021	-1	430.40397	21.5						
<0.01	<0.01	1	431.00249	260.9						
<0.01	0.015	1	431.09781	196.3						
<0.01	0.020	-1	431.38827	21.1						
<0.01	0.038	1	432.9643	58.8						
<0.01	<0.01	1	434.69068	47.7						
<0.01	<0.01	1	436.3053	22.6						
<0.01	0.049	-1	436.5624	279.7						
<0.01	0.039	-1	437.0763	99.8						
<0.01	0.036	-1	438.07974	99.9						
<0.01	0.011	1	438.29762	22.4						
<0.01	<0.01	1	438.32106	22.2						
<0.01	0.037	-1	439.07257	91.6						
<0.01	0.021	1	439.30106	24.4						
<0.01	0.027	1	439.32429	22.2						
<0.01	<0.01	1	440.3367	22.0						
<0.01	0.049	-1	441.06865	100.3						
<0.01	0.015	1	441.34013	22.2						
<0.01	<0.01	1	442.35232	21.5						
<0.01	0.011	1	443.3558	21.3						
<0.01	0.016	1	444.35876	22.3						
<0.01	<0.01	1	444.36798	21.9						
<0.01	<0.01	1	445.37136	21.8						
<0.01	<0.01	1	446.3261	21.6						
<0.01	<0.01	1	447.32949	21.7						
<0.01	0.028	1	447.68814	168.7						
<0.01	0.035	1	448.18936	168.2						
<0.01	<0.01	1	449.76255	52.0						
<0.01	0.019	1	451.36086	21.6						
<0.01	<0.01	1	454.29258	24.3						
<0.01	<0.01	1	455.29609	25.1						
<0.01	0.047	1	456.4044	21.4						
<0.01	<0.01	1	457.02009	51.4						
<0.01	0.036	-1	460.14968	110.7						
<0.01	<0.01	1	460.80777	48.5						
<0.01	0.038	1	461.77595	50.6						
<0.01	<0.01	-1	462.01166	100.1						
<0.01	0.021	1	462.07169	209.9						
<0.01	0.032	1	462.29748	25.1						
<0.01	0.022	1	462.32101	22.2						
<0.01	0.015	1	463.17648	23.4						
<0.01	<0.01	1	463.32466	22.5						
<0.01	<0.01	-1	463.60828	269.8						
<0.01	0.013	1	463.96265	69.0						
<0.01	<0.01	1	464.31337	23.4						

<0.01	<0.01	1	464.33665	21.9						
<0.01	<0.01	1	465.34017	22.1						
<0.01	0.019	1	466.35237	21.9						
<0.01	0.017	1	467.3557	22.1						
<0.01	0.041	1	468.30828	28.4						
<0.01	0.046	1	468.36803	21.9						
<0.01	<0.01	-1	469.06723	91.6						
<0.01	0.017	1	469.31186	28.5						
<0.01	0.041	1	474.35756	21.5						
<0.01	<0.01	1	475.36112	21.6						
<0.01	0.014	1	476.78143	48.2						
<0.01	0.012	1	478.29257	24.8						
<0.01	<0.01	1	479.29597	25.2						
<0.01	<0.01	1	480.30836	23.8						
<0.01	0.016	1	482.36038	27.9						
<0.01	0.016	-1	482.57993	296.4						
<0.01	<0.01	1	483.32729	24.5						
<0.01	<0.01	1	483.36375	28.4						
<0.01	<0.01	-1	484.01145	168.8						
<0.01	0.019	-1	486.06489	100.1						
<0.01	0.037	-1	492.24215	69.2						
<0.01	0.038	1	492.30852	28.3						
<0.01	0.010	-1	492.58774	44.3						
<0.01	0.038	1	494.32414	26.5						
<0.01	0.011	1	496.33931	18.6						
<0.01	<0.01	1	497.34284	19.3						
<0.01	0.014	1	498.34561	24.0						
<0.01	<0.01	1	499.34818	27.3						
<0.01	0.043	1	500.95187	59.0						
<0.01	0.014	1	502.29236	21.3						
<0.01	0.040	-1	504.00963	114.4						
<0.01	0.012	-1	504.0575	109.6						
<0.01	0.011	1	506.3596	25.8						
<0.01	0.018	-1	507.58599	100.5						
<0.01	<0.01	-1	508.11075	115.3						
<0.01	0.035	1	508.33959	26.0						
<0.01	0.040	-1	509.13816	110.6						
<0.01	0.024	1	510.45132	21.3						
<0.01	<0.01	1	511.35847	25.0						
<0.01	0.036	1	512.33438	27.4						
<0.01	<0.01	1	515.31319	27.3						
<0.01	0.012	1	515.8153	27.8						
<0.01	<0.01	1	518.32168	24.1						
<0.01	0.023	1	520.33965	25.5						
<0.01	0.023	1	521.34308	25.7						
<0.01	0.022	1	522.3552	21.2						
<0.01	0.015	1	523.29945	26.5						
<0.01	0.030	-1	526.07721	108.4						
<0.01	0.037	1	526.29244	21.1						
<0.01	<0.01	-1	526.56003	100.1						
<0.01	0.012	1	527.29577	20.8						

<0.01	<0.01	1	527.31647	29.9						
<0.01	0.020	1	527.38058	26.8						
<0.01	<0.01	1	527.66917	50.2						
<0.01	<0.01	1	528.30843	20.9						
<0.01	0.026	1	529.32832	27.2						
<0.01	<0.01	1	530.28653	23.4						
<0.01	0.013	-1	532.05249	90.2						
<0.01	<0.01	1	534.80238	253.7						
<0.01	<0.01	-1	538.19887	101.1						
<0.01	0.039	1	538.34914	16.8						
<0.01	<0.01	1	538.38642	25.5						
<0.01	0.016	1	539.38887	25.8						
<0.01	0.018	-1	542.17334	56.9						
<0.01	0.048	1	542.32258	26.0						
<0.01	0.018	1	543.32609	27.2						
<0.01	0.033	-1	544.10715	100.2						
<0.01	<0.01	1	546.35457	24.5						
<0.01	<0.01	1	548.37092	24.1						
<0.01	<0.01	1	549.37445	23.2						
<0.01	<0.01	1	549.48708	23.2						
<0.01	0.029	1	550.38781	26.3						
<0.01	<0.01	1	551.38948	26.6						
<0.01	0.020	1	551.68264	50.6						
<0.01	0.036	1	552.96471	58.8						
<0.01	0.049	-1	558.48632	44.0						
<0.01	0.020	1	559.2998	38.7						
<0.01	<0.01	1	564.96663	57.7						
<0.01	0.029	-1	565.62978	101.4						
<0.01	<0.01	1	570.35565	25.1						
<0.01	0.047	1	571.28887	29.8						
<0.01	0.033	1	571.36001	26.3						
<0.01	<0.01	1	572.37068	24.4						
<0.01	0.029	1	574.31258	28.5						
<0.01	0.019	1	575.50148	24.2						
<0.01	<0.01	-1	576.12582	247.9						
<0.01	<0.01	-1	579.0758	131.3						
<0.01	0.031	-1	579.09823	99.9						
<0.01	0.020	1	580.01645	254.1						
<0.01	0.023	-1	585.62739	50.2						
<0.01	0.017	1	586.59398	47.4						
<0.01	0.039	1	589.58505	261.2						
<0.01	<0.01	-1	596.36602	23.0						
<0.01	<0.01	-1	597.96795	169.5						
<0.01	<0.01	1	599.44124	30.4						
<0.01	0.038	1	599.95084	172.4						
<0.01	<0.01	-1	603.02915	100.0						
<0.01	0.011	-1	604.62922	43.7						
<0.01	<0.01	-1	606.32758	267.6						
<0.01	0.014	-1	614.61609	100.2						
<0.01	0.011	1	615.20042	190.9						
<0.01	0.038	1	615.43878	290.3						

<0.01	0.039	1	620.95449	58.7						
<0.01	0.011	-1	621.1683	167.8						
<0.01	0.022	-1	624.18634	289.1						
<0.01	0.011	-1	627.08357	99.8						
<0.01	<0.01	-1	644.49475	22.6						
<0.01	0.049	1	649.04999	280.7						
<0.01	<0.01	1	649.06615	53.1						
<0.01	0.027	1	660.66195	47.7						
<0.01	0.035	-1	663.24117	100.7						
<0.01	<0.01	1	664.11536	253.4						
<0.01	0.012	1	665.11676	253.9						
<0.01	0.018	-1	671.1225	142.7						
<0.01	<0.01	-1	672.10352	131.7						
<0.01	<0.01	1	678.50297	38.7						
<0.01	0.015	1	680.49859	47.4						
<0.01	0.024	1	682.37712	36.5						
<0.01	0.045	-1	688.96207	289.0						
<0.01	0.047	1	688.96934	293.2						
<0.01	<0.01	-1	696.11581	130.8						
<0.01	0.030	1	701.55999	23.0						
<0.01	0.012	1	702.5011	36.5						
<0.01	<0.01	1	711.73116	256.3						
<0.01	0.012	1	716.51767	23.3						
<0.01	0.036	1	718.53317	34.8						
<0.01	0.046	1	718.53985	24.5						
<0.01	0.011	-1	719.1139	100.2						
<0.01	0.027	1	722.51086	22.7						
<0.01	<0.01	-1	727.12235	169.2						
<0.01	0.030	1	731.05074	279.9						
<0.01	0.012	1	731.16216	280.1						
<0.01	0.023	1	733.55055	19.1						
<0.01	0.034	1	734.57535	21.1						
<0.01	0.010	1	735.56244	23.5						
<0.01	<0.01	1	738.508	26.6						
<0.01	<0.01	1	742.5386	34.0						
<0.01	0.027	1	742.57533	23.4						
<0.01	<0.01	1	744.58512	23.4						
<0.01	<0.01	1	745.55782	23.3						
<0.01	0.026	1	758.57103	16.8						
<0.01	0.019	1	758.57674	285.1						
<0.01	0.010	1	758.95961	276.0						
<0.01	<0.01	1	796.525	28.4						
<0.01	0.046	-1	804.45066	49.8						
<0.01	0.018	1	806.55524	23.3						
<0.01	0.047	-1	809.57699	20.5						
<0.01	0.038	1	812.53626	28.3						
<0.01	<0.01	-1	814.55737	24.2						
<0.01	<0.01	1	830.55374	23.0						
<0.01	0.031	1	832.5715	23.8						
<0.01	0.012	1	833.57474	23.7						
<0.01	0.020	-1	845.9841	52.5						

**Note:** Decide test value "-1" or "1" indicates the t-statistic is classified as significantly negative or significantly positive respectively										

Supplement Table S1c. Significant features for interaction (2 way limma anova test, limma decide test, P<0.05)

P.value	Adjusted.P.value	Decide test	m/z	RT(sec)
<0.01	<0.01	-1	87.0040	226.7
<0.01	0.03	-1	88.0216	54.0
<0.01	0.02	-1	89.0249	49.9
<0.01	<0.01	1	91.0059	79.3
<0.01	0.05	1	91.5075	72.8
<0.01	0.02	1	102.0130	282.0
<0.01	0.01	-1	102.0338	265.2
<0.01	<0.01	1	104.0087	283.8
<0.01	0.03	1	108.9614	267.9
<0.01	<0.01	-1	116.0621	27.6
<0.01	0.02	1	120.0236	276.0
<0.01	0.03	-1	120.0558	90.6
<0.01	0.04	1	121.0285	117.2
<0.01	<0.01	1	122.0142	197.5
<0.01	<0.01	1	122.0192	274.6
<0.01	0.04	-1	124.0758	136.4
<0.01	<0.01	-1	125.0152	26.7
<0.01	0.02	1	125.9643	110.6
<0.01	<0.01	-1	127.0123	24.8
<0.01	0.04	-1	127.0867	185.1
<0.01	<0.01	1	127.9722	15.6
<0.01	<0.01	-1	131.9743	236.7
<0.01	<0.01	1	132.0036	280.8
<0.01	<0.01	-1	133.0148	31.9
<0.01	<0.01	-1	133.0591	290.6
<0.01	0.03	-1	134.0271	58.0
<0.01	0.03	-1	135.0304	52.5
<0.01	<0.01	-1	137.0157	91.2
<0.01	<0.01	-1	138.9943	89.5
<0.01	<0.01	-1	140.0256	26.4
<0.01	<0.01	-1	140.5222	100.1
<0.01	<0.01	1	140.9513	110.0
<0.01	<0.01	1	141.9514	88.1
<0.01	0.02	-1	145.0151	26.1
<0.01	<0.01	1	145.0351	279.6
<0.01	0.01	-1	146.1652	122.3
<0.01	0.04	-1	146.1654	66.5
<0.01	0.03	-1	148.0221	92.2
<0.01	<0.01	-1	148.5237	91.5
<0.01	<0.01	-1	148.5363	90.2
<0.01	<0.01	1	150.0141	279.0
<0.01	<0.01	-1	151.0296	18.3
<0.01	<0.01	-1	151.0696	289.0
<0.01	<0.01	-1	153.0277	17.9
<0.01	<0.01	-1	153.0699	26.0
<0.01	<0.01	-1	154.0229	43.7
<0.01	0.02	-1	154.5227	133.9
<0.01	<0.01	-1	155.0854	21.7
<0.01	0.02	-1	155.0912	172.6

<0.01	<0.01	1	158.0998	26.1						
<0.01	0.05	1	160.0311	37.0						
<0.01	<0.01	1	160.5270	91.1						
<0.01	0.04	1	162.0912	40.4						
<0.01	<0.01	-1	163.0309	24.9						
<0.01	<0.01	1	163.0866	33.2						
<0.01	<0.01	-1	164.0342	24.6						
<0.01	<0.01	-1	164.0561	61.1						
<0.01	0.04	-1	165.9664	58.8						
<0.01	0.05	1	166.9830	252.7						
<0.01	0.01	1	167.0338	21.9						
<0.01	<0.01	1	167.0340	102.7						
<0.01	0.01	-1	167.4876	87.5						
<0.01	0.01	-1	168.0438	55.3						
<0.01	0.05	-1	169.0356	51.8						
<0.01	<0.01	-1	170.0051	100.3						
<0.01	0.01	1	171.0105	256.4						
<0.01	0.02	-1	175.0076	33.2						
<0.01	0.01	-1	175.1481	160.6						
<0.01	<0.01	-1	178.0444	33.0						
<0.01	0.04	1	178.9750	34.9						
<0.01	0.02	1	179.0025	196.1						
<0.01	0.05	-1	179.9813	142.1						
<0.01	<0.01	-1	180.0034	61.6						
<0.01	<0.01	-1	180.5168	75.3						
<0.01	<0.01	-1	180.5220	96.7						
<0.01	0.05	-1	180.9748	130.2						
<0.01	0.03	-1	181.0089	51.0						
<0.01	<0.01	-1	181.0794	24.6						
<0.01	0.02	-1	186.0505	77.8						
<0.01	<0.01	1	186.1060	62.8						
<0.01	0.02	1	186.9563	165.9						
<0.01	0.01	1	188.0376	198.3						
<0.01	<0.01	-1	189.0466	29.4						
<0.01	<0.01	-1	190.0894	238.8						
<0.01	0.01	-1	190.1567	108.0						
<0.01	0.02	-1	190.9793	267.9						
<0.01	<0.01	-1	191.0439	27.6						
<0.01	<0.01	-1	191.0485	31.9						
<0.01	<0.01	-1	191.0622	48.2						
<0.01	<0.01	1	195.0877	22.5						
<0.01	<0.01	1	195.9305	100.3						
<0.01	0.03	-1	196.0194	92.0						
<0.01	<0.01	-1	198.1222	178.5						
<0.01	<0.01	1	199.9235	78.3						
<0.01	0.03	1	200.1216	68.0						
<0.01	0.04	-1	201.4865	293.2						
<0.01	0.01	-1	201.5456	98.1						
<0.01	<0.01	-1	202.0200	138.0						
<0.01	0.05	-1	203.9908	122.7						
<0.01	<0.01	1	206.0456	104.1						
<0.01	<0.01	-1	206.0730	27.4						

<0.01	<0.01	-1	207.0419	197.9						
<0.01	<0.01	-1	209.0541	25.7						
<0.01	0.03	-1	209.0781	43.6						
<0.01	<0.01	-1	210.0581	33.8						
<0.01	<0.01	1	210.0913	176.7						
<0.01	0.04	-1	212.0917	140.3						
<0.01	<0.01	1	212.0917	210.7						
<0.01	0.03	-1	215.0697	61.0						
<0.01	<0.01	1	215.9365	94.8						
<0.01	0.03	-1	217.1065	190.2						
<0.01	0.04	-1	217.1546	36.0						
<0.01	<0.01	-1	217.5179	261.9						
<0.01	<0.01	-1	217.5404	89.5						
<0.01	<0.01	-1	218.0421	89.6						
<0.01	<0.01	-1	222.1077	24.4						
<0.01	0.02	-1	223.5634	100.6						
<0.01	<0.01	-1	224.0835	24.8						
<0.01	<0.01	1	224.0895	52.1						
<0.01	<0.01	-1	225.0869	26.6						
<0.01	<0.01	-1	225.0870	193.9						
<0.01	<0.01	-1	226.0810	202.1						
<0.01	<0.01	1	226.1017	85.5						
<0.01	0.03	-1	226.1187	70.4						
<0.01	0.03	-1	228.9734	49.4						
<0.01	0.01	-1	229.5193	125.9						
<0.01	<0.01	1	230.0958	62.5						
<0.01	<0.01	1	230.5390	169.6						
<0.01	<0.01	-1	230.9220	54.3						
<0.01	0.02	-1	231.0092	170.3						
<0.01	<0.01	1	232.0916	62.4						
<0.01	0.02	-1	234.9818	48.3						
<0.01	0.04	-1	235.1188	268.4						
<0.01	0.03	-1	235.5385	90.3						
<0.01	<0.01	1	235.9621	271.8						
<0.01	<0.01	-1	236.0837	200.1						
<0.01	0.01	1	237.0851	47.3						
<0.01	<0.01	1	238.0634	28.5						
<0.01	<0.01	-1	238.0991	21.7						
<0.01	0.04	1	238.9278	78.7						
<0.01	0.04	-1	239.0656	96.2						
<0.01	<0.01	-1	239.1029	185.1						
<0.01	0.02	-1	239.5310	89.6						
<0.01	0.04	-1	239.5664	95.9						
<0.01	<0.01	-1	241.0295	64.5						
<0.01	0.03	1	244.0412	178.4						
<0.01	0.04	-1	245.9682	77.0						
<0.01	<0.01	-1	246.0649	57.0						
<0.01	<0.01	1	246.1698	28.9						
<0.01	<0.01	1	247.1731	28.9						
<0.01	<0.01	1	249.0537	198.2						
<0.01	0.02	-1	250.1533	43.1						
<0.01	0.05	-1	250.9553	49.1						

<0.01	<0.01	1	252.1442	67.0						
<0.01	0.05	-1	252.9967	114.9						
<0.01	<0.01	1	253.0811	60.0						
<0.01	0.04	1	253.5381	140.2						
<0.01	0.02	1	253.6311	29.6						
<0.01	<0.01	-1	253.9937	81.3						
<0.01	<0.01	-1	254.0941	25.7						
<0.01	0.01	-1	255.5531	127.6						
<0.01	<0.01	1	256.0968	165.8						
<0.01	0.04	-1	257.5380	96.3						
<0.01	<0.01	-1	261.8896	50.6						
<0.01	<0.01	1	262.0373	156.6						
<0.01	<0.01	-1	262.0781	52.8						
<0.01	<0.01	-1	265.0230	76.9						
<0.01	<0.01	1	265.1116	60.3						
<0.01	0.01	-1	266.5289	89.4						
<0.01	<0.01	1	268.0518	62.2						
<0.01	<0.01	1	270.8913	78.1						
<0.01	0.01	-1	270.9581	48.5						
<0.01	0.01	1	271.0814	87.7						
<0.01	<0.01	1	272.0922	57.2						
<0.01	<0.01	-1	272.9551	48.5						
<0.01	0.01	-1	276.9226	47.5						
<0.01	0.02	1	279.1588	21.7						
<0.01	<0.01	1	280.0070	135.3						
<0.01	0.05	-1	282.9732	79.1						
<0.01	0.03	-1	286.9320	49.0						
<0.01	<0.01	-1	287.5924	90.2						
<0.01	0.02	-1	288.5920	109.1						
<0.01	0.03	-1	288.9291	48.9						
<0.01	0.01	-1	289.0403	47.9						
<0.01	0.01	-1	291.0477	189.2						
<0.01	0.04	1	292.1485	26.8						
<0.01	0.03	-1	292.9400	48.6						
<0.01	0.03	1	293.5815	128.8						
<0.01	0.03	-1	294.0223	128.3						
<0.01	0.01	-1	295.0305	53.4						
<0.01	<0.01	1	295.9028	77.5						
<0.01	0.01	-1	296.0494	76.1						
<0.01	0.02	-1	297.9038	123.8						
<0.01	<0.01	-1	298.2011	15.7						
<0.01	<0.01	-1	299.0832	41.9						
<0.01	0.02	-1	299.9371	126.1						
<0.01	0.04	-1	300.0156	93.4						
<0.01	<0.01	-1	300.5333	126.1						
<0.01	0.04	-1	302.9058	49.6						
<0.01	<0.01	-1	303.5463	241.1						
<0.01	0.04	-1	303.8738	50.0						
<0.01	0.01	1	304.4793	243.4						
<0.01	0.03	-1	304.9032	49.3						
<0.01	0.01	-1	304.9132	284.4						
<0.01	0.01	-1	304.9816	293.5						

<0.01	0.04	1	305.0678	252.5						
<0.01	<0.01	-1	309.0738	98.9						
<0.01	<0.01	-1	313.0171	135.0						
<0.01	0.02	1	313.2735	152.5						
<0.01	<0.01	-1	314.8157	43.8						
<0.01	0.02	-1	315.1741	255.7						
<0.01	0.04	-1	319.5066	127.0						
<0.01	0.03	-1	323.9729	75.5						
<0.01	0.03	-1	324.8447	43.6						
<0.01	<0.01	-1	327.0487	73.5						
<0.01	0.04	-1	328.2479	22.9						
<0.01	0.01	-1	328.9165	48.5						
<0.01	0.02	-1	332.0060	51.2						
<0.01	<0.01	1	332.5616	253.3						
<0.01	<0.01	-1	332.5895	161.6						
<0.01	<0.01	-1	334.0269	276.1						
<0.01	<0.01	1	336.9632	88.7						
<0.01	0.01	-1	341.0023	286.0						
<0.01	<0.01	-1	344.4302	107.8						
<0.01	0.03	-1	346.8876	48.8						
<0.01	0.04	-1	350.9727	127.3						
<0.01	<0.01	-1	351.9680	74.0						
<0.01	0.01	1	352.8533	78.0						
<0.01	0.04	-1	353.4644	91.4						
<0.01	0.01	-1	353.9879	49.8						
<0.01	<0.01	1	355.1105	63.6						
<0.01	<0.01	-1	357.0371	89.4						
<0.01	<0.01	1	362.0199	56.8						
<0.01	0.01	-1	365.0010	62.3						
<0.01	0.02	-1	366.8723	48.7						
<0.01	0.04	-1	372.0185	124.3						
<0.01	0.04	-1	374.0375	60.3						
<0.01	0.01	-1	375.9701	50.1						
<0.01	0.03	1	377.8300	43.9						
<0.01	0.02	-1	378.9497	292.9						
<0.01	<0.01	-1	379.0343	100.4						
<0.01	0.04	-1	380.0694	110.8						
<0.01	0.03	-1	380.9834	165.7						
<0.01	0.05	-1	381.8012	50.7						
<0.01	0.03	-1	382.8465	48.5						
<0.01	0.02	-1	383.3305	21.6						
<0.01	<0.01	-1	384.1152	37.5						
<0.01	<0.01	-1	385.1185	37.5						
<0.01	<0.01	-1	387.2636	25.9						
<0.01	0.01	-1	388.0274	59.9						
<0.01	0.05	1	389.1071	254.0						
<0.01	<0.01	-1	390.3117	22.9						
<0.01	<0.01	-1	391.0315	89.8						
<0.01	0.02	1	391.2838	22.5						
<0.01	0.04	-1	391.8602	43.5						
<0.01	<0.01	1	392.1177	261.8						
<0.01	<0.01	1	392.2871	21.2						

<0.01	0.05	1	392.2876	148.0						
<0.01	0.02	1	394.5063	167.6						
<0.01	<0.01	-1	396.0551	171.2						
<0.01	0.01	-1	399.0427	111.3						
<0.01	<0.01	1	413.2662	46.6						
<0.01	<0.01	1	414.2694	41.9						
<0.01	<0.01	-1	414.5240	261.3						
<0.01	<0.01	1	415.0485	63.9						
<0.01	0.03	-1	415.7720	27.1						
<0.01	<0.01	1	415.7957	44.3						
<0.01	<0.01	-1	417.1415	88.6						
<0.01	<0.01	1	418.1564	70.9						
<0.01	<0.01	1	419.7557	20.7						
<0.01	0.01	-1	426.0238	91.0						
<0.01	0.02	1	427.0951	196.3						
<0.01	0.05	-1	429.0395	284.0						
<0.01	<0.01	1	429.2401	45.9						
<0.01	<0.01	-1	431.0025	260.9						
<0.01	0.02	1	431.0978	196.3						
<0.01	0.04	1	437.9483	261.1						
<0.01	0.02	-1	438.8560	51.4						
<0.01	0.03	-1	446.0546	77.7						
<0.01	<0.01	-1	448.9609	76.5						
<0.01	<0.01	1	449.7626	52.0						
<0.01	<0.01	-1	449.9452	76.1						
<0.01	<0.01	-1	457.0201	51.4						
<0.01	<0.01	-1	460.8078	48.5						
<0.01	<0.01	-1	463.6083	269.8						
<0.01	0.03	-1	464.0809	114.2						
<0.01	0.03	-1	465.0849	114.3						
<0.01	0.01	1	466.0095	121.1						
<0.01	<0.01	-1	466.0863	114.4						
<0.01	<0.01	-1	469.0672	91.6						
<0.01	0.05	1	471.3398	22.9						
<0.01	0.02	-1	472.9860	41.6						
<0.01	<0.01	-1	474.8051	50.4						
<0.01	<0.01	1	475.3611	21.6						
<0.01	0.01	-1	476.7814	48.2						
<0.01	0.01	-1	478.0737	63.4						
<0.01	0.02	1	482.3604	27.9						
<0.01	0.02	-1	482.5799	296.4						
<0.01	<0.01	1	483.3638	28.4						
<0.01	<0.01	-1	484.0115	168.8						
<0.01	0.01	1	484.3846	22.7						
<0.01	0.03	1	485.9559	58.3						
<0.01	0.02	-1	486.0649	100.1						
<0.01	0.04	1	492.2422	69.2						
<0.01	0.01	1	496.3393	18.6						
<0.01	0.01	1	498.3456	24.0						
<0.01	<0.01	1	499.3482	27.3						
<0.01	0.04	1	500.9519	59.0						
<0.01	0.02	-1	507.5860	100.5						

<0.01	<0.01	1	510.3552	24.6						
<0.01	<0.01	1	511.3585	25.0						
<0.01	<0.01	1	515.3132	27.3						
<0.01	0.01	1	515.8153	27.8						
<0.01	<0.01	1	518.3217	24.1						
<0.01	<0.01	-1	520.7630	48.9						
<0.01	0.04	-1	523.0484	53.0						
<0.01	<0.01	1	527.3165	29.9						
<0.01	0.02	1	527.3806	26.8						
<0.01	0.05	1	527.4221	280.8						
<0.01	<0.01	1	530.2865	23.4						
<0.01	0.02	-1	530.8375	43.4						
<0.01	0.01	-1	532.0525	90.2						
<0.01	<0.01	1	538.3864	25.5						
<0.01	0.03	1	540.3669	28.8						
<0.01	0.02	-1	549.5896	239.5						
<0.01	0.05	1	556.4398	22.6						
<0.01	<0.01	1	564.9666	57.7						
<0.01	<0.01	1	570.3557	25.1						
<0.01	0.04	1	574.7213	50.8						
<0.01	0.01	1	575.7680	150.5						
<0.01	<0.01	-1	576.1258	247.9						
<0.01	0.02	1	582.2963	38.9						
<0.01	0.01	1	592.8772	277.6						
<0.01	0.04	-1	596.4599	52.7						
<0.01	<0.01	-1	597.9680	169.5						
<0.01	0.01	-1	604.6292	43.7						
<0.01	0.02	1	605.3180	20.1						
<0.01	<0.01	-1	606.3276	267.6						
<0.01	0.02	1	609.0121	68.3						
<0.01	0.04	1	613.1592	288.4						
<0.01	0.01	-1	615.2004	190.9						
<0.01	0.02	-1	624.1863	289.1						
<0.01	0.02	1	640.6846	264.4						
<0.01	<0.01	1	644.4948	22.6						
<0.01	0.04	-1	647.1464	113.6						
<0.01	0.04	1	647.8496	274.9						
<0.01	0.03	-1	660.6620	47.7						
<0.01	<0.01	1	664.1154	253.4						
<0.01	<0.01	-1	664.1635	155.2						
<0.01	<0.01	1	678.5030	38.7						
<0.01	0.01	-1	680.4986	47.4						
<0.01	0.02	1	682.3582	76.5						
<0.01	<0.01	1	696.1158	130.8						
<0.01	0.05	1	702.5485	47.4						
<0.01	0.04	1	705.0290	275.3						
<0.01	0.01	1	716.5177	23.3						
<0.01	0.03	1	731.0507	279.9						
<0.01	0.01	1	731.1622	280.1						
<0.01	0.02	1	731.2763	280.1						
<0.01	0.02	1	733.5506	19.1						
<0.01	<0.01	1	744.5851	23.4						

<0.01	<0.01	1	745.5578	23.3
<0.01	0.05	1	746.5699	24.0
<0.01	0.04	1	752.5233	22.5
<0.01	0.03	1	758.5710	16.8
<0.01	0.05	-1	758.7924	291.4
<0.01	0.01	1	763.2154	270.6
<0.01	<0.01	1	796.5250	28.4
<0.01	<0.01	-1	796.8018	288.1
<0.01	0.02	1	806.5552	23.3
<0.01	0.05	1	818.6046	23.4
<0.01	0.02	1	820.5174	24.7
<0.01	0.05	1	836.5407	25.8
<0.01	0.02	1	845.9841	52.5

Note: Decide test value "-1" or "1" indicates the t-statistic is classified as significantly negative or significantly positive respectively

Table S2a. Metabolic pathway enrichment

Pathway	Strain								Sex								Interaction							
Name	Overlap size	Pathway_size	-logP	Overlap metablite	KEGG ID	m/z	Match_form	Overlap size	Pathway_size	-logP	Overlap metablite	KEGG ID	m/z	Match_form	Overlap size	Pathway_size	-logP	Overlap metablite	KEGG ID	m/z	Match_form
**Drug metabolism - other enzymes**	10	28	1.7785	6-Thioguanosine monophosphate	C16619	379.0343	M(Cl37)+H[1+]	10	28	1.94677581	6-Thioguanosine monophosphate	C16619	379.0343	M(Cl37)+H[1+]	8	24	3.40515	6-Thioguanosine monophosphate	C16619	379.0343	M+K[1+]
				N-Acetylisoniazid; (N)1-Acetylisoniazid	C07585	181.0794	M(Cl37)+H[1+]				N-Acetylisoniazid; (N)1-Acetylisoniazid	C07585	181.0794	M(Cl37)+H[1+]				N-Acetylisoniazid; (N)1-Acetylisoniazid	C07585	181.0794	M(C13)+H[1+]
				5-Fluorodeoxyuridine; Floxuridine	C11736	269.055	M(C13)+H[1+]				5-Fluorodeoxyuridine; Floxuridine	C11736	246.0649,135.0304,269.055	M[1+]				5-Fluorodeoxyuridine; Floxuridine	C11736	135.0304,246.0649	M(C13)+H[1+]
				6-Mercaptopurin; Mercaptopurine	C02380	221.0095	M+HCOONa[1+]				Thioguanine	C07648	167.0261	M(Cl37)+H[1+]				6-Mercaptopurin; Mercaptopurine	C02380	190.9793	M+K[1+]
				SN38 glucuronide; SN-38G	C11376	285.0911	M+2H[2+]				6-Methylthioguanosine monophosphate	C16620	396.055,396.055	M(Cl37)+H[1+]				Thioguanine	C07648	169.0356	M(C13)+H[1+]
				6-Methylthioguanosine monophosphate	C16620	396.055,396.055	M(Cl37)+H[1+]				N,N'-Diacetylhydrazine; Diacetylhydrazine	C16622	116.0576	M(S34)+H[1+]				6-Methylmercaptopurine	C16614	169.0356,169.0356	M+HCOOK[1+]
				NPC	C16543	271.1055	M+H+Na[2+]				5'-Deoxy-5-fluorouridine; Doxifluridine	C12739	246.0649,135.0304,269.055	M[1+]				6-Methylthioguanosine monophosphate	C16620	396.055,396.055	M(Cl37)+H[1+]
				N,N'-Diacetylhydrazine; Diacetylhydrazine	C16622	117.0659	M(S34)+H[1+]				Isoniazid	C07054	206.0546	M+H[1+]				5'-Deoxy-5-fluorouridine; Doxifluridine	C12739	135.0304,246.0649	M(C13)+H[1+]
				5'-Deoxy-5-fluorouridine; Doxifluridine	C12739	269.055	M(C13)+H[1+]				SN-38	C11173	431.0978	M+2H[2+]				SN-38	C11173	431.0978	M+K[1+]
				SN-38	C11173	431.0978	M+2H[2+]				5-Fluorodeoxyuridine monophosphate	C04242	395.0268	M(Cl37)+H[1+]							
				5-Fluorodeoxyuridine monophosphate	C04242	395.0268	M(Cl37)+H[1+]														
**Glycosphingolipid biosynthesis - ganglioseries**															4	13	2.895029	Adenosine 3',5'-bisphosphate; PAP; 3'-Phc	C00054	429.0395	M+H[1+]
																		Chondroitin; Chondroitin-D-glucuronate	C00401	464.0809	M+HCOOK[1+]
																		GDP; Guanosine 5'-diphosphate; Guanosir	C00035	466.0095	M+Na[1+]
																		GDP-L-fucose; GDP-beta-L-fucose	C00325	296.0494	M+Na[1+]
**Tryptophan metabolism**								19	69	1.72258292	5-Methoxyindoleacetate	C05660	206.081	M+H[1+]	10	55	2.812652	Dihydrobiopterin; 6,7-Dihydrobiopterin; Q	C00268	239.1029	M[1+]
											Dihydrobiopterin; 6,7-Dihydrobiopterin; Qu	C00268	239.1029,242.1029	M[1+]				5-Hydroxykynurenine; 5-Hydroxy-L-kynure	C05651	225.087,225.0869	M+H[1+]
											5-Hydroxykynurenine; 5-Hydroxy-L-kynuren	C05651	224.0783,225.0869,226.0898	M+K[1+]				3-Hydroxy-L-kynurenine	C03227	225.087,225.0869	M+H[1+]
											3-Hydroxy-L-kynurenine	C03227	224.0783,225.0869,226.0898	M+K[1+]				Melatonin; N-Acetyl-5-methoxytryptamin	C01598	271.0814	M+K[1+]
											Melatonin; N-Acetyl-5-methoxytryptamine	C01598	301.1144,271.0814	M(C13)+H[1+]				Xanthurenic acid; Xanthurenate	C02470	206.0456	M+H[1+]
											Indolepyruvate; Indolepyruvic acid; (Indol-3	C00331	103.0391	M(C13)+2H[2+]				N-Formyl-D-kynurenine	C15605	237.0851	M[1+]
											5-Hydroxyindoleacetaldehyde	C05634	176.0706	M+Na[1+]				4-(2-Aminophenyl)-2,4-dioxobutanoate	C01252	210.0581,210.0581	M(Cl37)+H[1+]
											5-Hydroxy-N-formylkynurenine; 5-Hydroxy-	C05648	253.0811	M[1+]				5-Hydroxy-N-formylkynurenine; 5-Hydroxy	C05648	253.0811	M+Na[1+]
											Xanthurenic acid; Xanthurenate	C02470	208.0417,208.0417	M(Cl37)+H[1+]					CE2152	195.0876	M(C13)+H[1+]
												CE2152	211.0612	M+K[1+]				Formylkynurenine	C02406	237.0851	M[1+]
												C00936	181.0689	M(S34)+H[1+]				L-Formylkynurenine; N-Formyl-L-kynurenir	C02700	237.0851	M[1+]
											N-Acetylserotonin; N-Acetyl-5-hydroxytrypt	C00978	221.1104,221.1104	M+K[1+]				2-Amino-3-carboxymuconate semialdehy	C04409	188.0376	M+HCOONa[1+]
											4-(2-Aminophenyl)-2,4-dioxobutanoate	C01252	208.0604,210.0581,210.0581	M+H+Na[2+]							
											Indole-3-acetate; Indole-3-acetic acid; (Indo	C00954	176.0706	M+Na[1+]							
											5-Hydroxy-L-tryptophan	C00643	223.089,223.089,221.0925	M(Cl37)+H[1+]							
											5-hydroxytryptophol	CE1918	200.0684	M(C13)+H[1+]							
											Formylanthranilate; N-Formylanthranilate;	C05653	166.0499	M+K[1+]							
											2-Amino-3-carboxymuconate semialdehyde	C04409	188.0376	M+HCOONa[1+]							
											4-Hydroxy-2-quinolinecarboxylic acid; Kynu	C01717	192.0469,190.0499,192.0469	M(Cl37)+H[1+]							
**Drug metabolism - cytochrome P450**								13	52	1.31910365	Demethylcitalopram	C16608	156.0808	M+Na[1+]	8	44	2.638286	Morphine-3-glucuronide	C16643	232.0916	M(C13)+2H[2+]
											Morphine-3-glucuronide	C16643	232.0916,463.1765	M(C13)+2H[2+]				Cyclophosphamide anhydrous	C07888	262.0372	M(C13)+H[1+]
											Cyclophosphamide anhydrous	C07888	262.0372	M(C13)+H[1+]				Citalopram aldehyde	C16612	380.0694	M+HCOOK[1+]
											2-Hydroxyfelbamate	C16582	255.0975	M+H[1+]				2-Hydroxyiminostilbene	C16604	210.0913	M+H[1+]
											2-Ethylidene-1,5-dimethyl-3,3-diphenylpyrr	C16659	279.1918	M[1+]				2-Phenyl-1,3-propanediol monocarbamat	C16586	195.0876	M+H[1+]
											p-Hydroxyfelbamate	C16584	255.0975	M+H[1+]				Morphine-6-glucuronide	C16578	232.0916	M(C13)+2H[2+]
											Felbamate	C07501	241.1019,239.1029,240.1063	M+K[1+]				Felbamate	C07501	239.1029,120.0558	M+H[1+]
											Carbamazepine	C06868	239.0968,238.108	M(S34)+H[1+]				Ifosfamide	C07047	262.0372	M(C13)+H[1+]
											Morphine-6-glucuronide	C16578	232.0916,463.1765	M(C13)+2H[2+]				Codeine-6-glucuronide	C16577	239.1029	M(C13)+2H[2+]
											Ifosfamide	C07047	262.0372	M(C13)+H[1+]				Alcophosphamide	C16551	140.0256	M+Na[1+]
											Alcophosphamide	C16551	140.0256	M+2H[2+]							
											Methadone	C07163	312.2165	M(C13)+H[1+]							
											Codeine-6-glucuronide	C16577	239.1029	M(C13)+2H[2+]							
**Carnitine shuttle**								16	28	2.22025801	L-Carnitine; L-gamma-Trimethyl-beta-hydro	C00318	200.0684	M(Cl37)+H[1+]	3	10	2.503931	docosa-4,7,10,13,16-pentaenoyl carnitine	dcsptn1cr	496.3393,475.3611	M+Na[1+]
											gamma-linolenyl carnitine	lnlncgcrn	423.3295,422.3261	M(C13)+H[1+]				clupanodonyl carnitine	clpndcrn	496.3393,475.3611	M+Na[1+]
											arachidyl carnitine	arachcrn	456.4044	M+H[1+]				arachidyl carnitine	arachcrn	540.3669	M+Na[1+]
											Elaidic carnitine	elaidcrn	464.3134	M(C13)+H[1+]							
											stearoylcarnitine	stcrn	512.3344	M+H[1+]							
											clupanodonyl carnitine	clpndcrn	474.3576,496.3393,475.3611	M+H[1+]							
											Linoleyl carnitine	lnlccrn	446.3261,462.2975,425.3451	M+H[1+]							
											cervonyl carnitine	c226crn	494.3241	M(C13)+H[1+]							
											docosa-4,7,10,13,16-pentaenoyl carnitine	dcsptn1crn	474.3576,496.3393,475.3611	M+H[1+]							
											octadecenoyl carnitine	odecrn	464.3134	M(C13)+H[1+]							
											Vaccenyl carnitine	vacccrn	464.3134	M(C13)+H[1+]							
											alpha-linolenyl carnitine	lnlncacrn	423.3295,422.3261	M(C13)+H[1+]							
											Hexadecenoyl carnitine	hdcecrn	398.3262,399.3291	M+H[1+]							
											Linoelaidyl carnitine	lneldccrn	446.3261,462.2975,425.3451	M+H[1+]							
											L-Palmitoylcarnitine	C02990	422.3261,438.2976	M+Na[1+]							
											trans-Hexadec-2-enoyl carnitine	hdd2crn	398.3262,399.3291	M+H[1+]							
**Purine metabolism**	21	64	1.9522	Deoxyinosine	C05512	291.0477	M+K[1+]								9	54	2.489219	Urate; Uric acid	C00366	169.0356,252.9967	M+H[1+]
				3',5'-Cyclic AMP; Cyclic adenylic acid; Cyclic	C00575	330.0592	M+2H[2+]											Deoxyinosine	C05512	291.0477,254.0941	M+K[1+]
				Xanthosine	C01762	287.0763	M+K[1+]											Inosine	C00294	271.0814	M(S34)+H[1+]
				alpha-D-Ribose 1-phosphate	C00442	231.025	M+Na[1+]											GDP; Guanosine 5'-diphosphate; Guanosir	C00035	466.0095	M+Na[1+]
				Guanine; 2-Amino-6-hydroxypurine	C00242	152.0566,220.044	M+HCOOK[1+]											dGDP; 2'-Deoxyguanosine 5'-diphosphate	C00361	429.0395	M+H[1+]
				Ascorbate; Ascorbic acid; L-Ascorbate; L-Asc	C00072	178.0444,177.0409	M(C13)+H[1+]											Ascorbate; Ascorbic acid; L-Ascorbate; L-A	C00072	178.0444	M(C13)+H[1+]
				2-(Formamido)-N1-(5'-phosphoribosyl)aceta	C04640	315.0804	M+HCOONa[1+]											Aminoimidazole ribotide; AIR; 1-(5'-Phosp	C03373	148.5363	M(Cl37)+H[1+]
				Aminoimidazole ribotide; AIR; 1-(5'-Phosph	C03373	148.5363	M(Cl37)+H[1+]											2'-Deoxyinosine 5'-phosphate; dIMP	C06196	167.034,167.0337	M+Na[1+]
				1-(5'-Phosphoribosyl)-5-amino-4-(N-succino	C04823	457.0732	M+H+Na[2+]											N6-(1,2-Dicarboxyethyl)-AMP; Adenylosuc	C03794	486.0649,465.0849,464.0809	M+H[1+]
				IMP; Inosinic acid; Inosine monophosphate;	C00130	387.007	M(S34)+H[1+]														
				3',5'-Cyclic GMP; Guanosine 3',5'-cyclic mon	C00942	368.0376	M[1+]														
				2'-Deoxyinosine 5'-phosphate; dIMP	C06196	178.024	M+Na[1+]														
				Deoxyguanosine; 2'-Deoxyguanosine	C00330	352.0636	M(S34)+H[1+]														
				1-(5'-Phosphoribosyl)-5-amino-4-imidazolec	C04677	339.0701	M(Cl37)+H[1+]														
				N6-(1,2-Dicarboxyethyl)-AMP; Adenylosucci	C03794	465.0849,464.0809	M(C13)+H[1+]														
				1-(5-Phospho-D-ribosyl)-5-amino-4-imidazo	C04751	340.0522	M(S34)+H[1+]														
				5'-Phosphoribosylglycinamide; GAR; N1-(5-I	C03838	288.0696	M(C13)+H[1+]														
				Pyruvate; Pyruvic acid; 2-Oxopropanoate; 2-	C00022	157.0096	M+HCOOK[1+]														
				Adenosine	C00212	352.0636	M(S34)+H[1+]														
				Guanosine	C00387	368.0602	M+H[1+]														
				1-(5'-Phosphoribosyl)-5-formamido-4-imida	C04734	367.0623	M[1+]														
**Butanoate metabolism**															4	22	2.024062	5-Acetamidopentanoate	C03087	162.0912	M+K[1+]
																		2-(alpha-Hydroxyethyl)thiamine diphosph	C05125	469.0672	M[1+]
																		6-Acetamido-2-oxohexanoate; 2-Oxo-6-ac	C05548	190.0894,190.0894	M(Cl37)+H[1+]
																		Succinate; Succinic acid; Butanedionic acid	C00042	121.0285	M(S34)+H[1+]
**Biopterin metabolism**															3	14	2.010379	5,10-Methylenetetrahydrofolate; (6R)-5,1	C00143	230.0958	M(C13)+2H[2+]
																		Dihydrobiopterin; 6,7-Dihydrobiopterin; Q	C00268	239.1029	M[1+]
																		6-Pyruvoyltetrahydropterin; 6-(1,2-Dioxop	C03684	237.0851	M[1+]

																		6-Lactoyl-5,6,7,8-tetrahydropterin	C04244	239.1029	M[1+]
**TCA cycle**															3	17	1.692566	Succinate; Succinic acid; Butanedionic acid	C00042	121.0285	M(S34)+H[1+]
																		GDP; Guanosine 5'-diphosphate; Guanosir	C00035	466.0095	M+Na[1+]
																		Oxalosuccinate; Oxalosuccinic acid	C05379	228.9734	M+K[1+]
**Squalene and cholesterol biosynthesis**								10	34	1.61595544	Desmosterol; 24-Dehydrocholesterol; Chole	C01802	469.3119	M+H[1+]	3	18	1.597608	4alpha-Methylzymosterol-4-carboxylate; 4	C15808	527.3165	M+HCOOK[1+]
											Isofucosterol	C08821	497.3428	M+HCOOK[1+]				7-Dehydrodesmosterol	C05107	383.3304	M+H[1+]
											4alpha-Methylzymosterol-4-carboxylate; 4a	C15808	444.3588,443.3558,527.3165	M(C13)+H[1+]				(S)-2,3-Epoxysqualene; Squalene 2,3-epox	C01054	511.3585	M+HCOOK[1+]
											7-Dehydrodesmosterol	C05107	383.3304	M+H[1+]				cholesta-5,7,24-trien-3beta-ol	CE2321	383.3304	M+H[1+]
											14-Demethyllanosterol; 4,4-Dimethyl-5alph	C05108	497.3428	M+HCOOK[1+]				Lanosterol; 4,4',14alpha-Trimethyl-5alpha	C01724	511.3585	M+HCOOK[1+]
											Acetoacetate; 3-Oxobutanoic acid; beta-Ket	C00164	103.0391	M+H[1+]							
											Cholesta-5,7-dien-3beta-ol; 7-Dehydrochole	C01164	469.3119	M+H[1+]							
											cholesta-5,7,24-trien-3beta-ol	CE2321	383.3304	M+H[1+]							
											Episterol	C15777	483.3273	M+HCOOK[1+]							
											(R)-5-Phosphomevalonate; (R)-5-Phosphom	C01107	126.0186	M(S34)+H[1+]							
											Lanosterol; 4,4',14alpha-Trimethyl-5alpha-c	C01724	511.3585	M+HCOOK[1+]							
											4,4-dimethylcholesta-8(9),14-dien-3beta-ol	CE2313	497.3428	M+HCOOK[1+]							
											5alpha-Cholesta-7,24-dien-3beta-ol	C05439	469.3119	M+H[1+]							
											Zymosterol; delta8,24-Cholestadien-3beta-c	C05437	469.3119	M+H[1+]							
											(S)-2,3-Epoxysqualene; Squalene 2,3-epoxic	C01054	511.3585	M+HCOOK[1+]							
											delta7-Avenasterol	C15782	497.3428	M+HCOOK[1+]							
											24-Methylenecholesterol	C15781	483.3273	M+HCOOK[1+]							
**Linoleate metabolism**	8	22	1.6865	(6Z,9Z,12Z)-Octadecatrienoic acid; 6,9,12-O	C06426	280.235	M+2H[2+]								3	18	1.597608	Ascorbate; Ascorbic acid; L-Ascorbate; L-A	C00072	178.0444	M(C13)+H[1+]
				Glutathione disulfide; GSSG; Oxiglutatione;	C00127	613.1592	M+Na[1+]												CE2061	253.0811	M+HCOOK[1+]
				Ascorbate; Ascorbic acid; L-Ascorbate; L-Asc	C00072	178.0444,177.0409	M(C13)+H[1+]											Glutathione disulfide; GSSG; Oxiglutatione	C00127	613.1592	M+Na[1+]
				13(S)-HODE; (13S)-Hydroxyoctadecadienoic	C14762	335.1982,319.2245	M+K[1+]														
				9(10)-EpOME; (9R,10S)-(12Z)-9,10-Epoxyoc	C14825	335.1982,319.2245	M+K[1+]														
				12(13)-EpOME; (12R,13S)-(9Z)-12,13-Epoxy	C14826	335.1982,319.2245	M+K[1+]														
				UDP-glucuronate; UDPglucuronate; UDP-D-;	C00167	603.0291	M(C13)+2H[2+]														
				NA	CE2061	253.0811	M(S34)+H[1+]														
**Methionine and cysteine metabolism**															6	45	1.576665	Taurine; 2-Aminoethanesulfonic acid; Ami	C00245	125.0152	M(C13)+H[1+]
																		Glutathione disulfide; GSSG; Oxiglutatione	C00127	613.1592	M+Na[1+]
																		Adenosine 3',5'-bisphosphate; PAP; 3'-Phc	C00054	429.0395	M+H[1+]
																			C08276	120.0236	M[1+]
																		L-Cystine; L-Dicysteine; L-alpha-Diamino-b	C00491	241.0295	M(Cl37)+H[1+]
																		N-Acetylmethionine	C02712	191.0622	M[1+]
**Fructose and mannose metabolism**															4	28	1.527684	GDP; Guanosine 5'-diphosphate; Guanosir	C00035	466.0095	M+Na[1+]
																		D-Fructose 1,6-bisphosphate	C00354	341.0023	M+H[1+]
																		GDP-L-fucose; GDP-beta-L-fucose	C00325	296.0494	M+Na[1+]
																		beta-D-Fructose 1,6-bisphosphate	C05378	341.0023	M+H[1+]
																		D-Mannose 1,6-bisphosphate	C03693	341.0023	M+H[1+]
																		beta-D-Fructose 2,6-bisphosphate; D-Fruc	C00665	341.0023	M+H[1+]
																		GDP-mannose; GDP-D-mannose	C00096	303.5463	M+2H[2+]
**N-Glycan biosynthesis**															3	19	1.508043	GDP; Guanosine 5'-diphosphate; Guanosir	C00035	466.0095	M+Na[1+]
																		GDP-mannose; GDP-D-mannose	C00096	303.5463	M+2H[2+]
																		GDP-L-fucose; GDP-beta-L-fucose	C00325	296.0494	M+Na[1+]
**Glycosphingolipid metabolism**															4	29	1.456035	Adenosine 3',5'-bisphosphate; PAP; 3'-Phc	C00054	429.0395	M+H[1+]
																		GDP; Guanosine 5'-diphosphate; Guanosir	C00035	466.0095	M+Na[1+]
																		Ethanolamine phosphate; O-Phosphorylet	C00346	179.9813	M(S34)+H[1+]
																		GDP-L-fucose; GDP-beta-L-fucose	C00325	296.0494	M+Na[1+]
**Tyrosine metabolism**															10	85	1.3279	5,6-Dihydroxyindole; DHI	C05578	218.0421	M+HCOONa[1+]
																		Dihydrobiopterin; 6,7-Dihydrobiopterin; Q	C00268	239.1029	M[1+]
																		Dopamine; 4-(2-Aminoethyl)-1,2-benzene	C03758	155.0911	M(Cl37)+H[1+]
																		salsolinol 1-carboxylate	CE5626	224.0895	M+K[1+]
																		Ascorbate; Ascorbic acid; L-Ascorbate; L-A	C00072	178.0444	M(C13)+H[1+]
																		Glutathione disulfide; GSSG; Oxiglutatione	C00127	613.1592	M+Na[1+]
																		3,4-Dihydroxyphenylacetaldehyde; Protoc	C04043	88.0216	M+H+Na[2+]
																		2-Hydroxyphenylacetate	C05852	88.0216	M+H+Na[2+]
																		2-Carboxy-2,3-dihydro-5,6-dihydroxyindol	C05604	218.0421	M+Na[1+]
																		Dopaquinone	C00822	218.0421	M+Na[1+]
																		Adenosine 3',5'-bisphosphate; PAP; 3'-Phc	C00054	429.0395	M+H[1+]
																		3,4-Dihydroxyphenylacetate; 3,4-Dihydro	C01161	168.0438	M(C13)+H[1+]
																		4-Hydroxyphenylacetate; 4-Hydroxypheny	C00642	88.0216	M+H+Na[2+]
																		3-O-methyldopa	CE2176	212.0917	M+H[1+]
																		Homogentisate; Homogentisic acid; 2,5-D	C00544	168.0438	M(C13)+H[1+]
																		dopaminochrome	CE4888	218.0421	M+HCOONa[1+]
																		3,4-Dihydroxymandelaldehyde	C05577	168.0438	M(C13)+H[1+]
**Parathio degradation**	4	5	1.9658	Diethylthiophosphoric acid; DETP	C06607	173.021,173.021	M(S34)+H[1+]														
				4-Nitrophenol; p-Nitrophenol; PNP; Niphen;	C00870	140.033	M+H[1+]														
				Parathion; O,O-Diethyl O-p-nitrophenyl pho	C06604	147.0257	M(C13)+H[1+]														
				Diethylphosphoric acid; O,O-Diethylphosph	C06608	193.0019,156.0506	M+K[1+]														
**Urea cycle/amino group metabolism**	18	55	1.894	Guanidinoacetate	gudac	118.0612	M+HCOONa[1+]														
				L-Proline; 2-Pyrrolidinecarboxylic acid	C00148	117.074,116.0707	M(C13)+H[1+]														
				Guanine; 2-Amino-6-hydroxypurine	C00242	152.0566,220.044	M+HCOOK[1+]														
				N-Acetyl-L-glutamate; N-Acetyl-L-glutamic a	C00624	228.0281,212.0537	M+H[1+]														
				5,10-Methylenetetrahydrofolate; (6R)-5,10-	C00143	457.1671,230.0958,457.1679	M[1+]														
				Thiopurine	C01756	221.0095	M+HCOONa[1+]														
				L-Arginine; (S)-2-Amino-5-guanidinovaleric a	C00062	176.1224	M+H[1+]														
				L-Citrulline; 2-Amino-5-ureidovaleric acid; Ci	C00327	178.0983	M+H[1+]														
				L-Glutamyl 5-phosphate; L-Glutamate 5-phc	C03287	228.0281	M+K[1+]														
				14	41	1.862962492	M(C13)+H[1+]														
				L-Glutamate 5-semialdehyde; L-Glutamate g	C01165	134.06	M(S34)+H[1+]														
				5'-Methylthioadenosine; Methylthioadenos	C00170	298.0966	M+K[1+]														
				N-Acetyl-L-glutamate 5-semialdehyde; 2-Ac	C01250	176.0706,212.0333	M(S34)+H[1+]														
				Spermidine; N-(3-Aminopropyl)-1,4-butane-	C00315	146.1652	M(C13)+H[1+]														
				L-Methionine; Methionine; L-2-Amino-4met	C00073	152.0566	M(Cl37)+H[1+]														
				Agmatine; (4-Aminobutyl) guanidine	C00179	169.0844	M+K[1+]														
				N-(L-Arginino)succinate; 2-(Nomega-L-Argin	C03406	157.0608	M(C13)+2H[2+]														
				N4-Acetylaminobutanal	C05936	131.0895,130.0863	M(C13)+H[1+]														
**Arginine and Proline Metabolism**	14	41	1.863	L-Proline; 2-Pyrrolidinecarboxylic acid	C00148	117.074,116.0707	M(C13)+H[1+]														
				L-erythro-4-Hydroxyglutamate	C05947	164.0561	M+K[1+]														
				Pyruvate; Pyruvic acid; 2-Oxopropanoate; 2-	C00022	157.0096	M+HCOOK[1+]														
				L-Methionine; Methionine; L-2-Amino-4met	C00073	152.0566	M(Cl37)+H[1+]														
				L-Arginine; (S)-2-Amino-5-guanidinovaleric a	C00062	176.1224	M+H[1+]														
				L-Citrulline; 2-Amino-5-ureidovaleric acid; Ci	C00327	178.0983	M+H[1+]														
				L-Glutamate 5-semialdehyde; L-Glutamate g	C01165	134.06	M(S34)+H[1+]														

				5'-Methylthioadenosine; Methylthioadenos	C00170	298.0966	M+K[1+]							
				trans-4-Hydroxy-L-proline	C01157	134.06	M(S34)+H[1+]							
				N-(L-Arginino)succinate; 2-(Nomega-L-Argin	C03406	157.0608	M(C13)+2H[2+]							
				D-Proline	C00763	117.074,116.0707	M(C13)+H[1+]							
				Agmatine; (4-Aminobutyl) guanidine	C00179	169.0844	M+K[1+]							
				4-Hydroxy-2-oxoglutarate; 4-Hydroxy-2-oxo	C01127	231.0092	M+HCOONa[1+]							
				N4-Acetylaminobutanal	C05936	131.0895,130.0863	M(C13)+H[1+]							
**Aspartate and asparagine metabolism**	21	71	1.743	L-Proline; 2-Pyrrolidinecarboxylic acid	C00148	117.074,116.0707	M(C13)+H[1+]							
				Glutathione disulfide; GSSG; Oxiglutatione; (	C00127	613.1592	M+Na[1+]							
				L-Arginine; (S)-2-Amino-5-guanidinovaleric a	C00062	176.1224	M+H[1+]							
				L-Citrulline; 2-Amino-5-ureidovaleric acid; Ci	C00327	178.0983	M+H[1+]							
				O-Acetylcarnitine; O-Acetyl-L-carnitine	C02571	273.1201	M(C13)+H[1+]							
				L-Glutamate 5-semialdehyde; L-Glutamate g	C01165	134.06	M(S34)+H[1+]							
				N-Formyl-L-aspartate	C01044	164.0342	M(C13)+H[1+]							
				spermic acid 2	CE1938	271.1055	M+K[1+]							
				Cys-Gly; L-Cysteinylglycine	C01419	181.0443,181.0443	M(C13)+H[1+]							
				L-erythro-4-Hydroxyglutamate	C05947	164.0561	M+K[1+]							
				D-4-Hydroxy-2-oxoglutarate	C05946	231.0092	M+HCOONa[1+]							
				cis-4-Hydroxy-D-proline	C03440	134.06	M(S34)+H[1+]							
				2-Hydroxyglutarate	C02630	151.039	M(Cl37)+H[1+]							
				Spermidine; N-(3-Aminopropyl)-1,4-butane-	C00315	146.1652	M(C13)+H[1+]							
				L-Ornithine; (S)-2,5-Diaminovaleric acid; (S)-	C00077	133.0972	M(C13)+H[1+]							
				dehydrospermidine	CE5586	146.1652	M(C13)+H[1+]							
				trans-4-Hydroxy-L-proline	C01157	134.06	M(S34)+H[1+]							
				L-Glutamyl 5-phosphate; L-Glutamate 5-pho	C03287	228.0281	M+K[1+]							
				acetamidopropanal	CE4788	117.074,116.0707	M(C13)+H[1+]							
				N-Succinyl-L-glutamate 5-semialdehyde; (2S	C05932	316.0418	M+HCOOK[1+]							
				N2-Succinyl-L-ornithine; (2S)-5-Amino-2-(3-c	C03415	271.0673,271.0678	M+H[1+]							
				N4-Acetylaminobutanal	C05936	131.0895,130.0863	M(C13)+H[1+]							
**Selenoamino acid metabolism**	7	21	1.4577	Se-Adenosylselenohomocysteine	C05692	217.5404	M(C13)+2H[2+]							
				gamma-Glutamyl-Se-methylselenocysteine;	C05695	157.5187	M(C13)+2H[2+]							
				5-Methyltetrahydrofolate	C00440	462.1945	M(Cl37)+H[1+]							
				Selenocystathionine	C05699	136.514	M(C13)+2H[2+]							
				Selenohomocysteine	C05698	182.9814	M(Cl37)+H[1+]							
				Se-Methylselenocysteine	C05689	182.9814	M(Cl37)+H[1+]							
				Oxidized dithiothreitol	C01119	155.0025	M+Na[1+]							
				Adenosine	C00212	352.0636	M(S34)+H[1+]							
**Carbon fixation**	4	10	1.3519	D-Xylulose 5-phosphate	C00231	231.025	M+H[1+]							
				D-Fructose 1,6-bisphosphate	C00354	341.0023,342.9961	M+H[1+]							
				D-Erythrose 4-phosphate	C00279	202.0199	M(Cl37)+H[1+]							
				Pyruvate; Pyruvic acid; 2-Oxopropanoate; 2-	C00022	157.0096	M+HCOOK[1+]							
**Vitamin D3 (cholecalciferol) metabolism**								5	10	1.94073583	Calcidiol; 25-Hydroxyvitamin D3; Calcifediol	C01561	439.3011	M+K[1+]
											Calcitriol	C01673	417.3403,418.3431,455.2961	M+H[1+]
											Vitamin D3; Cholecalciferol	C05443	469.3119	M+H[1+]
											Cholesta-5,7-dien-3beta-ol; 7-Dehydrochole	C01164	469.3119	M+H[1+]
											24,25-Dihydroxyvitamin D3	CE1337	417.3403,418.3431,455.2961	M+H[1+]
											23S,25-dihydroxyvitamin D3	CE2201	417.3403,418.3431,455.2961	M+H[1+]
**Pentose and Glucuronate Interconversions**								6	15	1.82829663	UMP; Uridylic acid; Uridine monophosphate	C00105	347.0251,326.0462,325.043	M+2H[2+]
											D-Xylulose; D-threo-Pentulose; D-Lyxulose	C00310	152.0623	M+HCOOK[1+]
											L-Arabitol; L-Arabinol; L-Arabinitol; L-Lyxitol	C00532	154.0777	M+HCOOK[1+]
											L-Arabinose; L-Arabinopyranose	C00259	152.0623	M+HCOOK[1+]
											Xylitol	C00379	154.0777	M+HCOOK[1+]
											L-Xylulose; L-threo-Pentulose; L-Lyxulose	C00312	152.0623	M+HCOOK[1+]
											UDP-glucuronate; UDPglucuronate; UDP-D-	C00167	603.0291	M(C13)+2H[2+]
											D-Ribulose; D-erythro-2-Pentulose; D-Arabi	C00309	152.0623	M+HCOOK[1+]
**Starch and Sucrose Metabolism**								6	18	1.57874726	UMP; Uridylic acid; Uridine monophosphate	C00105	347.0251,326.0462,325.043	M+2H[2+]
											D-Glucose 1-phosphate; alpha-D-Glucose 1-	C00103	262.0394	M+Na[1+]
											beta-D-Glucose	C00221	181.0689	M(S34)+H[1+]
											D-Glucose; Grape sugar; Dextrose	C00031	181.0689	M(S34)+H[1+]
											alpha-D-Glucose	C00267	181.0689	M(S34)+H[1+]
											UDP-glucuronate; UDPglucuronate; UDP-D-	C00167	603.0291	M(C13)+2H[2+]
**Limonene and pinene degradation**								3	6	1.55286554		C06078	221.0925,221.0957	M+H[1+]
												C06306	221.0925,221.0957	M+H[1+]
											Perillyl aldehyde; Perillaldehyde	C02576	219.0971	M+K[1+]
**Nucleotide Sugar Metabolism**								3	7	1.40617182	dTDP-glucose; dTDP-D-glucose; dTDP-alpha	C00842	649.05	M[1+]
											UDP-glucuronate; UDPglucuronate; UDP-D-	C00167	603.0291	M(C13)+2H[2+]
											D-Glucose 1-phosphate; alpha-D-Glucose 1-	C00103	262.0394	M+Na[1+]

Table S2b. Metabolic pathway enrichment-Venn Diagram

Pathway	Overlap_size	Pathway_size	*P* value	-logP	Factor
Parathio degradation	4	5	0.01	1.966	Strain
Purine metabolism	21	64	0.01	1.952	Strain
Urea cycle/amino group metabolism	18	55	0.01	1.894	Strain
Arginine and Proline Metabolism	14	41	0.01	1.863	Strain
Drug metabolism - other enzymes	10	28	0.02	1.779	Strain
Aspartate and asparagine metabolism	21	71	0.02	1.743	Strain
Linoleate metabolism	8	22	0.02	1.686	Strain
Selenoamino acid metabolism	7	21	0.03	1.458	Strain
Carbon fixation	4	10	0.04	1.352	Strain
Carnitine shuttle	16	28	<0.01	2.220	Sex
Drug metabolism - other enzymes	10	28	0.01	1.947	Sex
Vitamin D3 (cholecalciferol) metabolism	5	10	0.01	1.941	Sex
Pentose and Glucuronate Interconversions	6	15	0.01	1.828	Sex
Tryptophan metabolism	19	69	0.02	1.723	Sex
Squalene and cholesterol biosynthesis	10	34	0.02	1.616	Sex
Starch and Sucrose Metabolism	6	18	0.03	1.579	Sex
Limonene and pinene degradation	3	6	0.03	1.553	Sex
Nucleotide Sugar Metabolism	3	7	0.04	1.406	Sex
Vitamin K metabolism	2	3	0.05	1.339	Sex
Drug metabolism - cytochrome P450	13	52	0.05	1.319	Sex
Drug metabolism - other enzymes	8	24	<0.01	3.405	Interaction
Glycosphingolipid biosynthesis - ganglioseries	4	13	<0.01	2.895	Interaction
Tryptophan metabolism	10	55	<0.01	2.813	Interaction
Drug metabolism - cytochrome P450	8	44	<0.01	2.638	Interaction
Carnitine shuttle	3	10	<0.01	2.504	Interaction
Purine metabolism	9	54	<0.01	2.489	Interaction
Butanoate metabolism	4	22	<0.01	2.024	Interaction
Biopterin metabolism	3	14	<0.01	2.010	Interaction
Glycosphingolipid biosynthesis - lactoseries	2	7	0.02	1.815	Interaction
TCA cycle	3	17	0.02	1.693	Interaction
Glycosphingolipid biosynthesis - neolactoseries	2	8	0.02	1.669	Interaction
Blood Group Biosynthesis	2	8	0.02	1.669	Interaction
Squalene and cholesterol biosynthesis	3	18	0.03	1.598	Interaction
Linoleate metabolism	3	18	0.03	1.598	Interaction
Methionine and cysteine metabolism	6	45	0.03	1.577	Interaction
Carbon fixation	2	9	0.03	1.538	Interaction
Glycosphingolipid biosynthesis - globoseries	2	9	0.03	1.538	Interaction
Fructose and mannose metabolism	4	28	0.03	1.528	Interaction
N-Glycan biosynthesis	3	19	0.03	1.508	Interaction
Glycosphingolipid metabolism	4	29	0.03	1.456	Interaction
Porphyrin metabolism	2	10	0.04	1.420	Interaction
Vitamin B1 (thiamin) metabolism	2	10	0.04	1.420	Interaction
Tyrosine metabolism	10	85	0.05	1.328	Interaction

1. Top pathway (ranked by *P* values) for each factor
2. *P* value < 0.05 pathways were considered significant

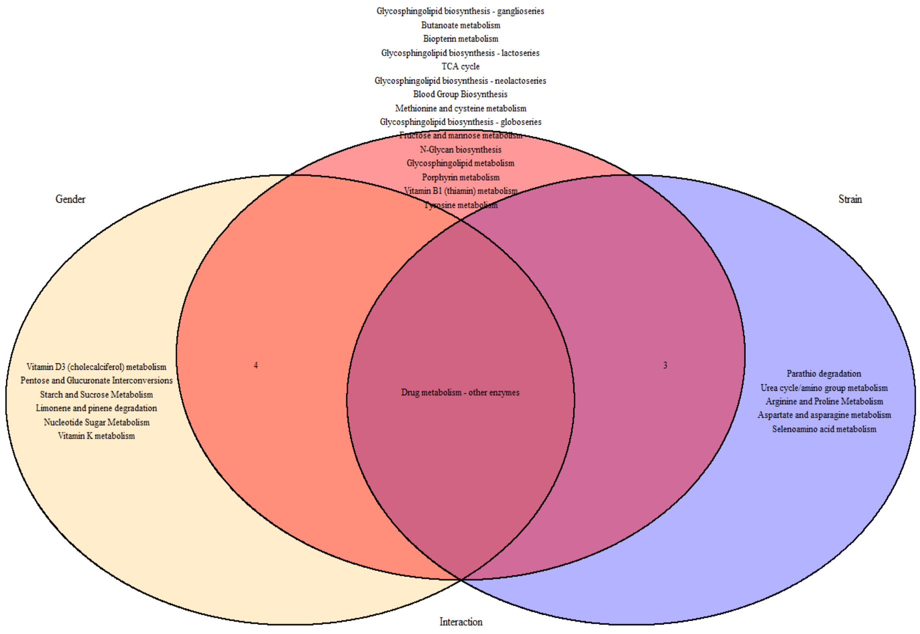

Table S3a. Differentially expressed transcripts for strain (2 way limma anova test, limma decide test, P<0.05)

Gene	Gene ID	Biotype	Description	P.value	Adjusted.P.value	Decide test
Igkv5-48	ENSMUSG00000076563	IG_V_gene	immunoglobulin kappa variable 5-48 [Source:MGI Symbol;Acc:MGI:3642817]	<0.01	0.020	-1
Apol10b	ENSMUSG00000050014	protein_coding	apolipoprotein L 10B [Source:MGI Symbol;Acc:MGI:3043522]	<0.01	<0.01	-1
Fcer1g	ENSMUSG00000058715	protein_coding	Fc receptor, IgE, high affinity I, gamma polypeptide [Source:MGI Symbol;Acc:MGI:95496]	<0.01	0.049	-1
Cd200	ENSMUSG00000022661	protein_coding	CD200 antigen [Source:MGI Symbol;Acc:MGI:1196990]	<0.01	<0.01	-1
Iglv2	ENSMUSG00000076940	IG_V_gene	immunoglobulin lambda variable 2 [Source:MGI Symbol;Acc:MGI:99548]	<0.01	0.011	-1
Pcdhgb2	ENSMUSG00000102748	protein_coding	protocadherin gamma subfamily B, 2 [Source:MGI Symbol;Acc:MGI:1935170]	<0.01	0.036	-1
Leo1	ENSMUSG00000042487	protein_coding	Leo1, Paf1/RNA polymerase II complex component [Source:MGI Symbol;Acc:MGI:2685031]	<0.01	<0.01	-1
Clu	ENSMUSG00000022037	protein_coding	clusterin [Source:MGI Symbol;Acc:MGI:88423]	<0.01	0.021	-1
Gm43254	ENSMUSG00000105957	lincRNA	predicted gene 43254 [Source:MGI Symbol;Acc:MGI:5663391]	<0.01	0.049	-1
Igkv4-59	ENSMUSG00000094006	IG_V_gene	immunoglobulin kappa variable 4-59 [Source:MGI Symbol;Acc:MGI:3646808]	<0.01	0.034	-1
Gm10874	ENSMUSG00000075591	lincRNA	predicted gene 10874 [Source:MGI Symbol;Acc:MGI:3704261]	<0.01	0.022	-1
Osgep	ENSMUSG00000006289	protein_coding	O-sialoglycoprotein endopeptidase [Source:MGI Symbol;Acc:MGI:1913496]	<0.01	0.027	-1
Itgb7	ENSMUSG00000001281	protein_coding	integrin beta 7 [Source:MGI Symbol;Acc:MGI:96616]	<0.01	<0.01	-1
Fam181a	ENSMUSG00000096753	protein_coding	family with sequence similarity 181, member A [Source:MGI Symbol;Acc:MGI:3647570]	<0.01	0.013	-1
Trat1	ENSMUSG00000030775	protein_coding	T cell receptor associated transmembrane adaptor 1 [Source:MGI Symbol;Acc:MGI:1924897]	<0.01	0.026	-1
Gm4955	ENSMUSG00000037849	protein_coding	interferon activated gene 206 [Source:MGI Symbol;Acc:MGI:3646410]	<0.01	<0.01	-1
Tm7sf2	ENSMUSG00000024799	protein_coding	transmembrane 7 superfamily member 2 [Source:MGI Symbol;Acc:MGI:1920416]	<0.01	0.040	-1
9530026P05Rik	ENSMUSG00000097462	antisense_RNA	RIKEN cDNA 9530026P05 gene [Source:MGI Symbol;Acc:MGI:1924659]	<0.01	0.049	-1
Gm4956	ENSMUSG00000025936	transcribed_unprocessed_pseudogene	predicted gene 4956 [Source:MGI Symbol;Acc:MGI:3647976]	<0.01	<0.01	-1
Ccl22	ENSMUSG00000031779	protein_coding	chemokine (C-C motif) ligand 22 [Source:MGI Symbol;Acc:MGI:1306779]	<0.01	0.016	-1
Adssl1	ENSMUSG00000011148	protein_coding	adenylosuccinate synthetase like 1 [Source:MGI Symbol;Acc:MGI:87947]	<0.01	0.016	-1
Ighv1-26	ENSMUSG00000094546	IG_V_gene	immunoglobulin heavy variable 1-26 [Source:MGI Symbol;Acc:MGI:4439641]	<0.01	<0.01	-1
Dapk1	ENSMUSG00000021559	protein_coding	death associated protein kinase 1 [Source:MGI Symbol;Acc:MGI:1916885]	<0.01	0.015	-1
Akt2-ps	ENSMUSG00000084347	processed_pseudogene	thymoma viral proto-oncogene 2, pseudogene [Source:MGI Symbol;Acc:MGI:108506]	<0.01	0.030	-1
Meox1	ENSMUSG00000001493	protein_coding	mesenchyme homeobox 1 [Source:MGI Symbol;Acc:MGI:103220]	<0.01	0.028	-1
Gm9826	ENSMUSG00000048538	processed_pseudogene	predicted gene 9826 [Source:MGI Symbol;Acc:MGI:3642725]	<0.01	<0.01	-1
Znhit1	ENSMUSG00000059518	protein_coding	zinc finger, HIT domain containing 1 [Source:MGI Symbol;Acc:MGI:1917353]	<0.01	0.041	-1
Pydc3	ENSMUSG00000066677	protein_coding	interferon activated gene 208 [Source:MGI Symbol;Acc:MGI:2442822]	<0.01	<0.01	-1
Polr1b	ENSMUSG00000027395	protein_coding	polymerase (RNA) I polypeptide B [Source:MGI Symbol;Acc:MGI:108014]	<0.01	0.039	-1
Zcchc11	ENSMUSG00000034610	protein_coding	zinc finger, CCHC domain containing 11 [Source:MGI Symbol;Acc:MGI:2445126]	<0.01	0.021	-1
Tmem159	ENSMUSG00000030917	protein_coding	transmembrane protein 159 [Source:MGI Symbol;Acc:MGI:1925752]	<0.01	<0.01	-1
Zfp446	ENSMUSG00000033961	protein_coding	zinc finger protein 446 [Source:MGI Symbol;Acc:MGI:2442185]	<0.01	0.038	-1
Atp1b1	ENSMUSG00000026576	protein_coding	ATPase, Na+/K+ transporting, beta 1 polypeptide [Source:MGI Symbol;Acc:MGI:88108]	<0.01	0.020	-1
Atp6v1c2	ENSMUSG00000020566	protein_coding	ATPase, H+ transporting, lysosomal V1 subunit C2 [Source:MGI Symbol;Acc:MGI:1916025]	<0.01	0.021	-1
Lpxn	ENSMUSG00000024696	protein_coding	leupaxin [Source:MGI Symbol;Acc:MGI:2147677]	<0.01	0.016	-1
Rplp0	ENSMUSG00000067274	protein_coding	ribosomal protein, large, P0 [Source:MGI Symbol;Acc:MGI:1927636]	<0.01	0.037	-1
Kmo	ENSMUSG00000039783	protein_coding	kynurenine 3-monooxygenase (kynurenine 3-hydroxylase) [Source:MGI Symbol;Acc:MGI:2138151]	<0.01	0.014	-1
Scnm1	ENSMUSG00000092607	protein_coding	sodium channel modifier 1 [Source:MGI Symbol;Acc:MGI:1341284]	<0.01	0.036	-1
Gm42501	ENSMUSG00000105609	TEC	predicted gene 42501 [Source:MGI Symbol;Acc:MGI:5662638]	<0.01	0.028	-1
Eci1	ENSMUSG00000024132	protein_coding	enoyl-Coenzyme A delta isomerase 1 [Source:MGI Symbol;Acc:MGI:94871]	<0.01	0.032	-1
F13a1	ENSMUSG00000039109	protein_coding	coagulation factor XIII, A1 subunit [Source:MGI Symbol;Acc:MGI:1921395]	<0.01	<0.01	-1
Fcgr2b	ENSMUSG00000026656	protein_coding	Fc receptor, IgG, low affinity IIb [Source:MGI Symbol;Acc:MGI:95499]	<0.01	0.021	-1
Tmco6	ENSMUSG00000006850	protein_coding	transmembrane and coiled-coil domains 6 [Source:MGI Symbol;Acc:MGI:1919233]	<0.01	0.019	-1
Gm6505	ENSMUSG00000070522	processed_pseudogene	predicted pseudogene 6505 [Source:MGI Symbol;Acc:MGI:3648080]	<0.01	0.034	-1
Rpl3	ENSMUSG00000060036	protein_coding	ribosomal protein L3 [Source:MGI Symbol;Acc:MGI:1351605]	<0.01	<0.01	-1
Gm13502	ENSMUSG00000083287	processed_pseudogene	predicted gene 13502 [Source:MGI Symbol;Acc:MGI:3649894]	<0.01	0.029	-1
Arl11	ENSMUSG00000043157	protein_coding	ADP-ribosylation factor-like 11 [Source:MGI Symbol;Acc:MGI:2444054]	<0.01	0.039	-1
Cacng7	ENSMUSG00000069806	protein_coding	calcium channel, voltage-dependent, gamma subunit 7 [Source:MGI Symbol;Acc:MGI:1932374]	<0.01	0.017	-1
Dcp2	ENSMUSG00000024472	protein_coding	decapping mRNA 2 [Source:MGI Symbol;Acc:MGI:1917890]	<0.01	0.016	-1
Gm13889	ENSMUSG00000087006	protein_coding	predicted gene 13889 [Source:MGI Symbol;Acc:MGI:3652053]	<0.01	<0.01	-1
1110059E24Rik	ENSMUSG00000035171	protein_coding	RIKEN cDNA 1110059E24 gene [Source:MGI Symbol;Acc:MGI:1913456]	<0.01	0.040	-1
Arhgdib	ENSMUSG00000030220	protein_coding	Rho, GDP dissociation inhibitor (GDI) beta [Source:MGI Symbol;Acc:MGI:101940]	<0.01	0.019	-1
BC048546	ENSMUSG00000047228	protein_coding	alpha-2-macroglobulin like 1 [Source:MGI Symbol;Acc:MGI:3039594]	<0.01	0.016	-1
Hrct1	ENSMUSG00000071001	protein_coding	histidine rich carboxyl terminus 1 [Source:MGI Symbol;Acc:MGI:1917945]	<0.01	<0.01	-1
Gm11826	ENSMUSG00000083328	processed_pseudogene	predicted gene 11826 [Source:MGI Symbol;Acc:MGI:3651142]	<0.01	<0.01	-1
Ighv5-4	ENSMUSG00000095612	IG_V_gene	immunoglobulin heavy variable 5-4 [Source:MGI Symbol;Acc:MGI:4439895]	<0.01	0.014	-1
Fcrla	ENSMUSG00000038421	protein_coding	Fc receptor-like A [Source:MGI Symbol;Acc:MGI:2138647]	<0.01	<0.01	-1
Igkv6-20	ENSMUSG00000076587	IG_V_gene	immunoglobulin kappa variable 6-20 [Source:MGI Symbol;Acc:MGI:1330836]	<0.01	0.012	-1
Epha7	ENSMUSG00000028289	protein_coding	Eph receptor A7 [Source:MGI Symbol;Acc:MGI:95276]	<0.01	0.028	-1
Ahsa1	ENSMUSG00000021037	protein_coding	AHA1, activator of heat shock protein ATPase 1 [Source:MGI Symbol;Acc:MGI:2387603]	<0.01	0.032	-1
Gm13139	ENSMUSG00000067916	protein_coding	zinc finger protein 991 [Source:MGI Symbol;Acc:MGI:3701604]	<0.01	0.015	-1
Abcg3	ENSMUSG00000029299	protein_coding	ATP-binding cassette, sub-family G (WHITE), member 3 [Source:MGI Symbol;Acc:MGI:1351624]	<0.01	<0.01	-1
Fam177a	ENSMUSG00000095595	protein_coding	family with sequence similarity 177, member A [Source:MGI Symbol;Acc:MGI:1920635]	<0.01	<0.01	-1
Tomm22	ENSMUSG00000022427	protein_coding	translocase of outer mitochondrial membrane 22 homolog (yeast) [Source:MGI Symbol;Acc:MGI:2450248]	<0.01	0.026	-1
Ttc7	ENSMUSG00000036918	protein_coding	tetratricopeptide repeat domain 7 [Source:MGI Symbol;Acc:MGI:1920999]	<0.01	0.029	-1
Slfn8	ENSMUSG00000035208	protein_coding	schlafen 8 [Source:MGI Symbol;Acc:MGI:2672859]	<0.01	<0.01	-1
Gm8203	ENSMUSG00000101878	processed_pseudogene	predicted pseudogene 8203 [Source:MGI Symbol;Acc:MGI:3646499]	<0.01	0.046	-1
Rprm	ENSMUSG00000075334	protein_coding	reprimo, TP53 dependent G2 arrest mediator candidate [Source:MGI Symbol;Acc:MGI:1915124]	<0.01	0.031	-1
Neurl3	ENSMUSG00000047180	protein_coding	neuralized E3 ubiquitin protein ligase 3 [Source:MGI Symbol;Acc:MGI:2429944]	<0.01	<0.01	-1
Kcns3	ENSMUSG00000043673	protein_coding	potassium voltage-gated channel, delayed-rectifier, subfamily S, member 3 [Source:MGI Symbol;Acc:MGI:1098804]	<0.01	0.027	-1
Rab32	ENSMUSG00000019832	protein_coding	RAB32, member RAS oncogene family [Source:MGI Symbol;Acc:MGI:1915094]	<0.01	0.012	-1
Rps3a1	ENSMUSG00000028081	protein_coding	ribosomal protein S3A1 [Source:MGI Symbol;Acc:MGI:1202063]	<0.01	<0.01	-1
Apobec3	ENSMUSG00000009585	protein_coding	apolipoprotein B mRNA editing enzyme, catalytic polypeptide 3 [Source:MGI Symbol;Acc:MGI:1933111]	<0.01	<0.01	-1
Fbln1	ENSMUSG00000006369	protein_coding	fibulin 1 [Source:MGI Symbol;Acc:MGI:95487]	<0.01	0.037	-1
Dusp23	ENSMUSG00000026544	protein_coding	dual specificity phosphatase 23 [Source:MGI Symbol;Acc:MGI:1915690]	<0.01	0.012	-1
Ms4a6c	ENSMUSG00000079419	protein_coding	membrane-spanning 4-domains, subfamily A, member 6C [Source:MGI Symbol;Acc:MGI:2385644]	<0.01	<0.01	-1
Ighv1-53	ENSMUSG00000093894	IG_V_gene	immunoglobulin heavy variable 1-53 [Source:MGI Symbol;Acc:MGI:3576502]	<0.01	<0.01	-1

Sirpb1a	ENSMUSG00000095788	protein_coding	signal-regulatory protein beta 1A [Source:MGI Symbol;Acc:MGI:2444824]	<0.01	0.015	-1
Tmem150c	ENSMUSG00000050640	protein_coding	transmembrane protein 150C [Source:MGI Symbol;Acc:MGI:3041258]	<0.01	0.013	-1
Gm9733	ENSMUSG00000078783	protein_coding	predicted gene 9733 [Source:MGI Symbol;Acc:MGI:3780136]	<0.01	0.045	-1
2610035D17Rik	ENSMUSG00000087259	lincRNA	RIKEN cDNA 2610035D17 gene [Source:MGI Symbol;Acc:MGI:1919636]	<0.01	<0.01	-1
Vnn3	ENSMUSG00000020010	protein_coding	vanin 3 [Source:MGI Symbol;Acc:MGI:1347055]	<0.01	<0.01	-1
Btf3l4	ENSMUSG00000028568	protein_coding	basic transcription factor 3-like 4 [Source:MGI Symbol;Acc:MGI:1915312]	<0.01	0.026	-1
Adamdec1	ENSMUSG00000022057	protein_coding	ADAM-like, decysin 1 [Source:MGI Symbol;Acc:MGI:1917650]	<0.01	0.012	-1
Themis2	ENSMUSG00000037731	protein_coding	thymocyte selection associated family member 2 [Source:MGI Symbol;Acc:MGI:2446213]	<0.01	0.012	-1
Lrrc48	ENSMUSG00000056598	protein_coding	dynein regulatory complex subunit 3 [Source:MGI Symbol;Acc:MGI:1921915]	<0.01	0.046	-1
Pctp	ENSMUSG00000020553	protein_coding	phosphatidylcholine transfer protein [Source:MGI Symbol;Acc:MGI:107375]	<0.01	0.031	-1
Cd52	ENSMUSG00000000682	protein_coding	CD52 antigen [Source:MGI Symbol;Acc:MGI:1346088]	<0.01	0.024	-1
Man2b1	ENSMUSG00000005142	protein_coding	mannosidase 2, alpha B1 [Source:MGI Symbol;Acc:MGI:107286]	<0.01	0.023	-1
Ms4a6b	ENSMUSG00000024677	protein_coding	membrane-spanning 4-domains, subfamily A, member 6B [Source:MGI Symbol;Acc:MGI:1917024]	<0.01	<0.01	-1
Sel1l3	ENSMUSG00000029189	protein_coding	sel-1 suppressor of lin-12-like 3 (C. elegans) [Source:MGI Symbol;Acc:MGI:1916941]	<0.01	<0.01	-1
Faap20	ENSMUSG00000073684	protein_coding	Fanconi anemia core complex associated protein 20 [Source:MGI Symbol;Acc:MGI:1914763]	<0.01	0.012	-1
C5ar2	ENSMUSG00000074361	protein_coding	complement component 5a receptor 2 [Source:MGI Symbol;Acc:MGI:2442013]	<0.01	0.019	-1
Mthfd1	ENSMUSG00000021048	protein_coding	methylenetetrahydrofolate dehydrogenase (NADP+ dependent), methenyltetrahydrofolate cyclohydrolase, formyltetrahydrofolate synthase [Source:MGI Symbol;Acc:MGI:1342005]	<0.01	<0.01	-1
Angptl4	ENSMUSG00000002289	protein_coding	angiopoietin-like 4 [Source:MGI Symbol;Acc:MGI:1888999]	<0.01	0.017	-1
Trim30d	ENSMUSG00000057596	protein_coding	tripartite motif-containing 30D [Source:MGI Symbol;Acc:MGI:3035181]	<0.01	0.017	-1
Fam47e	ENSMUSG00000057068	protein_coding	family with sequence similarity 47, member E [Source:MGI Symbol;Acc:MGI:2686227]	<0.01	<0.01	-1
Trpv6	ENSMUSG00000029868	protein_coding	transient receptor potential cation channel, subfamily V, member 6 [Source:MGI Symbol;Acc:MGI:1927259]	<0.01	<0.01	-1
6330403K07Rik	ENSMUSG00000018451	lincRNA	RIKEN cDNA 6330403K07 gene [Source:MGI Symbol;Acc:MGI:1918001]	<0.01	0.047	-1
Gm16118	ENSMUSG00000089797	antisense_RNA	predicted gene 16118 [Source:MGI Symbol;Acc:MGI:3801926]	<0.01	<0.01	-1
Ahcy	ENSMUSG00000027597	protein_coding	S-adenosylhomocysteine hydrolase [Source:MGI Symbol;Acc:MGI:87968]	<0.01	0.047	-1
Slfn1	ENSMUSG00000078763	protein_coding	schlafen 1 [Source:MGI Symbol;Acc:MGI:1313259]	<0.01	0.049	-1
Igkv4-50	ENSMUSG00000076562	IG_V_gene	immunoglobulin kappa variable 4-50 [Source:MGI Symbol;Acc:MGI:2685915]	<0.01	<0.01	-1
Ighv10-1	ENSMUSG00000095981	IG_V_gene	immunoglobulin heavy variable 10-1 [Source:MGI Symbol;Acc:MGI:4439620]	<0.01	0.020	-1
Foxq1	ENSMUSG00000038415	protein_coding	forkhead box Q1 [Source:MGI Symbol;Acc:MGI:1298228]	<0.01	0.044	-1
Cmtm7	ENSMUSG00000032436	protein_coding	CKLF-like MARVEL transmembrane domain containing 7 [Source:MGI Symbol;Acc:MGI:2447166]	<0.01	0.030	-1
Gm43653	ENSMUSG00000105447	antisense_RNA	predicted gene 43653 [Source:MGI Symbol;Acc:MGI:5663790]	<0.01	0.038	-1
Coro2a	ENSMUSG00000028337	protein_coding	coronin, actin binding protein 2A [Source:MGI Symbol;Acc:MGI:1345966]	<0.01	<0.01	-1
Rec8	ENSMUSG00000002324	protein_coding	REC8 meiotic recombination protein [Source:MGI Symbol;Acc:MGI:1929645]	<0.01	0.032	-1
Polr1c	ENSMUSG00000067148	protein_coding	polymerase (RNA) I polypeptide C [Source:MGI Symbol;Acc:MGI:103288]	<0.01	0.032	-1
Rab24	ENSMUSG00000034789	protein_coding	RAB24, member RAS oncogene family [Source:MGI Symbol;Acc:MGI:105065]	<0.01	0.028	-1
Hgsnat	ENSMUSG00000037260	protein_coding	heparan-alpha-glucosaminide N-acetyltransferase [Source:MGI Symbol;Acc:MGI:1196297]	<0.01	0.049	-1
Jchain	ENSMUSG00000067149	protein_coding	immunoglobulin joining chain [Source:MGI Symbol;Acc:MGI:96493]	<0.01	0.037	-1
Rab6b	ENSMUSG00000032549	protein_coding	RAB6B, member RAS oncogene family [Source:MGI Symbol;Acc:MGI:107283]	<0.01	<0.01	-1
Apol11b	ENSMUSG00000091694	protein_coding	apolipoprotein L 11b [Source:MGI Symbol;Acc:MGI:3036248]	<0.01	0.020	-1
Atp1a3	ENSMUSG00000040907	protein_coding	ATPase, Na+/K+ transporting, alpha 3 polypeptide [Source:MGI Symbol;Acc:MGI:88107]	<0.01	0.045	-1
Ighv1-69	ENSMUSG00000094502	IG_V_gene	immunoglobulin heavy variable 1-69 [Source:MGI Symbol;Acc:MGI:4439632]	<0.01	0.038	-1
Itga9	ENSMUSG00000039115	protein_coding	integrin alpha 9 [Source:MGI Symbol;Acc:MGI:104756]	<0.01	0.026	-1
Igkv6-15	ENSMUSG00000094797	IG_V_gene	immunoglobulin kappa variable 6-15 [Source:MGI Symbol;Acc:MGI:1330831]	<0.01	<0.01	-1
A830018L16Rik	ENSMUSG00000057715	protein_coding	RIKEN cDNA A830018L16 gene [Source:MGI Symbol;Acc:MGI:2444149]	<0.01	0.039	-1
Gas5	ENSMUSG00000053332	processed_transcript	growth arrest specific 5 [Source:MGI Symbol;Acc:MGI:95659]	<0.01	0.036	-1
Cep85	ENSMUSG00000037443	protein_coding	centrosomal protein 85 [Source:MGI Symbol;Acc:MGI:1917262]	<0.01	0.027	-1
I830127L07Rik	ENSMUSG00000102051	transcribed_unprocessed_pseudogene	RIKEN cDNA I830127L07 gene [Source:MGI Symbol;Acc:MGI:3612406]	<0.01	<0.01	-1
Selplg	ENSMUSG00000048163	protein_coding	selectin, platelet (p-selectin) ligand [Source:MGI Symbol;Acc:MGI:106689]	<0.01	0.011	-1
Blk	ENSMUSG00000014453	protein_coding	B lymphoid kinase [Source:MGI Symbol;Acc:MGI:88169]	<0.01	0.019	-1
Ighv1-75	ENSMUSG00000096020	IG_V_gene	immunoglobulin heavy variable 1-75 [Source:MGI Symbol;Acc:MGI:4439735]	<0.01	0.018	-1
Wnt10b	ENSMUSG00000022996	protein_coding	wingless-type MMTV integration site family, member 10B [Source:MGI Symbol;Acc:MGI:108061]	<0.01	<0.01	-1
Maff	ENSMUSG00000042622	protein_coding	v-maf musculoaponeurotic fibrosarcoma oncogene family, protein F (avian) [Source:MGI Symbol;Acc:MGI:96910]	<0.01	0.021	-1
Acot7	ENSMUSG00000028937	protein_coding	acyl-CoA thioesterase 7 [Source:MGI Symbol;Acc:MGI:1917275]	<0.01	<0.01	-1
Mplkip	ENSMUSG00000012429	protein_coding	M-phase specific PLK1 intereacting protein [Source:MGI Symbol;Acc:MGI:1913558]	<0.01	0.025	-1
Cxcr5	ENSMUSG00000047880	protein_coding	chemokine (C-X-C motif) receptor 5 [Source:MGI Symbol;Acc:MGI:103567]	<0.01	0.035	-1
Prmt8	ENSMUSG00000030350	protein_coding	protein arginine N-methyltransferase 8 [Source:MGI Symbol;Acc:MGI:3043083]	<0.01	<0.01	-1
Fcer2a	ENSMUSG00000005540	protein_coding	Fc receptor, IgE, low affinity II, alpha polypeptide [Source:MGI Symbol;Acc:MGI:95497]	<0.01	<0.01	-1
Adam28	ENSMUSG00000014725	protein_coding	a disintegrin and metallopeptidase domain 28 [Source:MGI Symbol;Acc:MGI:105988]	<0.01	0.013	-1
Fabp5	ENSMUSG00000027533	protein_coding	fatty acid binding protein 5, epidermal [Source:MGI Symbol;Acc:MGI:101790]	<0.01	<0.01	-1
Gzmb	ENSMUSG00000015437	protein_coding	granzyme B [Source:MGI Symbol;Acc:MGI:109267]	<0.01	0.021	-1
2610507I01Rik	ENSMUSG00000085882	processed_transcript	RIKEN cDNA 2610507I01 gene [Source:MGI Symbol;Acc:MGI:1919453]	<0.01	<0.01	-1
Smim7	ENSMUSG00000044600	protein_coding	small integral membrane protein 7 [Source:MGI Symbol;Acc:MGI:1914068]	<0.01	0.032	-1
Tpt1	ENSMUSG00000060126	protein_coding	tumor protein, translationally-controlled 1 [Source:MGI Symbol;Acc:MGI:104890]	<0.01	0.013	-1
Sirpb1b	ENSMUSG00000095028	protein_coding	signal-regulatory protein beta 1B [Source:MGI Symbol;Acc:MGI:3779828]	<0.01	<0.01	-1
Rpl29	ENSMUSG00000048758	protein_coding	ribosomal protein L29 [Source:MGI Symbol;Acc:MGI:99687]	<0.01	<0.01	-1
Mmp3	ENSMUSG00000043613	protein_coding	matrix metallopeptidase 3 [Source:MGI Symbol;Acc:MGI:97010]	<0.01	0.023	-1
Lpar2	ENSMUSG00000031861	protein_coding	lysophosphatidic acid receptor 2 [Source:MGI Symbol;Acc:MGI:1858422]	<0.01	0.037	-1
Lcmt2	ENSMUSG00000074890	protein_coding	leucine carboxyl methyltransferase 2 [Source:MGI Symbol;Acc:MGI:1353659]	<0.01	0.015	-1
Gm13268	ENSMUSG00000070271	processed_pseudogene	predicted gene 13268 [Source:MGI Symbol;Acc:MGI:3651947]	<0.01	0.048	-1
Iigp1	ENSMUSG00000054072	protein_coding	interferon inducible GTPase 1 [Source:MGI Symbol;Acc:MGI:1926259]	<0.01	0.041	-1
Megf6	ENSMUSG00000057751	protein_coding	multiple EGF-like-domains 6 [Source:MGI Symbol;Acc:MGI:1919351]	<0.01	0.011	-1
Igkv4-86	ENSMUSG00000076536	IG_V_gene	immunoglobulin kappa variable 4-86 [Source:MGI Symbol;Acc:MGI:2685305]	<0.01	0.011	-1
Ighv1-22	ENSMUSG00000094561	IG_V_gene	immunoglobulin heavy variable 1-22 [Source:MGI Symbol;Acc:MGI:4439784]	<0.01	0.015	-1
Ighv1-81	ENSMUSG00000094689	IG_V_gene	immunoglobulin heavy variable 1-81 [Source:MGI Symbol;Acc:MGI:4439635]	<0.01	<0.01	-1
Rab11b	ENSMUSG00000077450	protein_coding	RAB11B, member RAS oncogene family [Source:MGI Symbol;Acc:MGI:99425]	<0.01	<0.01	-1
Chsy1	ENSMUSG00000032640	protein_coding	chondroitin sulfate synthase 1 [Source:MGI Symbol;Acc:MGI:2681120]	<0.01	0.035	-1
Cdca7l	ENSMUSG00000021175	protein_coding	cell division cycle associated 7 like [Source:MGI Symbol;Acc:MGI:2384982]	<0.01	0.023	-1
Gm6158	ENSMUSG00000090381	processed_pseudogene	predicted gene 6158 [Source:MGI Symbol;Acc:MGI:3779562]	<0.01	0.022	-1
Bst2	ENSMUSG00000046718	protein_coding	bone marrow stromal cell antigen 2 [Source:MGI Symbol;Acc:MGI:1916800]	<0.01	0.018	-1
Wfdc17	ENSMUSG00000069792	protein_coding	WAP four-disulfide core domain 17 [Source:MGI Symbol;Acc:MGI:3649773]	<0.01	<0.01	-1
Gm20503	ENSMUSG00000092345	protein_coding	predicted gene 20503 [Source:MGI Symbol;Acc:MGI:5141968]	<0.01	0.010	-1

Rps7	ENSMUSG00000061477	protein_coding	ribosomal protein S7 [Source:MGI Symbol;Acc:MGI:1333818]	<0.01	<0.01	-1
Igtp	ENSMUSG00000078853	protein_coding	interferon gamma induced GTPase [Source:MGI Symbol;Acc:MGI:107729]	<0.01	0.021	-1
Smim6	ENSMUSG00000075420	protein_coding	small integral membrane protein 6 [Source:MGI Symbol;Acc:MGI:1915778]	<0.01	<0.01	-1
Fbxo2	ENSMUSG00000041556	protein_coding	F-box protein 2 [Source:MGI Symbol;Acc:MGI:2446216]	<0.01	0.040	-1
Rpl34	ENSMUSG00000062006	protein_coding	ribosomal protein L34 [Source:MGI Symbol;Acc:MGI:1915686]	<0.01	<0.01	-1
Cnpy2	ENSMUSG00000025381	protein_coding	canopy FGF signaling regulator 2 [Source:MGI Symbol;Acc:MGI:1928477]	<0.01	0.022	-1
Uba52	ENSMUSG00000090137	protein_coding	ubiquitin A-52 residue ribosomal protein fusion product 1 [Source:MGI Symbol;Acc:MGI:98887]	<0.01	0.031	-1
Kcnc3	ENSMUSG00000062785	protein_coding	potassium voltage gated channel, Shaw-related subfamily, member 3 [Source:MGI Symbol;Acc:MGI:96669]	<0.01	0.041	-1
Iglc3	ENSMUSG00000105547	IG_C_gene	immunoglobulin lambda constant 3 [Source:MGI Symbol;Acc:MGI:99886]	<0.01	0.040	-1
Gm11585	ENSMUSG00000082045	processed_pseudogene	predicted gene 11585 [Source:MGI Symbol;Acc:MGI:3651395]	<0.01	<0.01	-1
H2afj	ENSMUSG00000060032	protein_coding	H2A histone family, member J [Source:MGI Symbol;Acc:MGI:3606192]	<0.01	0.026	-1
Igkv8-27	ENSMUSG00000076580	IG_V_gene	immunoglobulin kappa chain variable 8-27 [Source:MGI Symbol;Acc:MGI:4439868]	<0.01	0.014	-1
Insig1	ENSMUSG00000045294	protein_coding	insulin induced gene 1 [Source:MGI Symbol;Acc:MGI:1916289]	<0.01	0.015	-1
Ighv3-6	ENSMUSG00000076672	IG_V_gene	immunoglobulin heavy variable 3-6 [Source:MGI Symbol;Acc:MGI:4439856]	<0.01	0.021	-1
Gjb6	ENSMUSG00000040055	protein_coding	gap junction protein, beta 6 [Source:MGI Symbol;Acc:MGI:107588]	<0.01	0.043	-1
Sox11	ENSMUSG00000063632	protein_coding	SRY (sex determining region Y)-box 11 [Source:MGI Symbol;Acc:MGI:98359]	<0.01	<0.01	-1
Igkv17-127	ENSMUSG00000076508	IG_V_gene	immunoglobulin kappa variable 17-127 [Source:MGI Symbol;Acc:MGI:3646891]	<0.01	<0.01	-1
Pcx	ENSMUSG00000024892	protein_coding	pyruvate carboxylase [Source:MGI Symbol;Acc:MGI:97520]	<0.01	0.027	-1
Acp1	ENSMUSG00000044573	protein_coding	acid phosphatase 1, soluble [Source:MGI Symbol;Acc:MGI:87881]	<0.01	<0.01	-1
Rdm1	ENSMUSG00000010362	protein_coding	RAD52 motif 1 [Source:MGI Symbol;Acc:MGI:1913849]	<0.01	<0.01	-1
Hist2h2be	ENSMUSG00000068854	protein_coding	histone cluster 2, H2be [Source:MGI Symbol;Acc:MGI:2448415]	<0.01	<0.01	-1
Pttg1	ENSMUSG00000020415	protein_coding	pituitary tumor-transforming gene 1 [Source:MGI Symbol;Acc:MGI:1353578]	<0.01	<0.01	-1
Nme2	ENSMUSG00000020857	protein_coding	NME/NM23 nucleoside diphosphate kinase 2 [Source:MGI Symbol;Acc:MGI:97356]	<0.01	<0.01	-1
Eif3f	ENSMUSG00000031029	protein_coding	eukaryotic translation initiation factor 3, subunit F [Source:MGI Symbol;Acc:MGI:1913335]	<0.01	0.012	-1
4833420G17Rik	ENSMUSG00000062822	protein_coding	RIKEN cDNA 4833420G17 gene [Source:MGI Symbol;Acc:MGI:1914642]	<0.01	<0.01	-1
D830050J10Rik	ENSMUSG00000107994	bidirectional_promoter_lncRNA	RIKEN cDNA D830050J10 gene [Source:MGI Symbol;Acc:MGI:2444203]	<0.01	0.031	-1
Snhg6	ENSMUSG00000098234	lincRNA	small nucleolar RNA host gene 6 [Source:MGI Symbol;Acc:MGI:1921074]	<0.01	<0.01	-1
9130008F23Rik	ENSMUSG00000054951	protein_coding	RIKEN cDNA 9130008F23 gene [Source:MGI Symbol;Acc:MGI:1918833]	<0.01	0.038	-1
Rps15a-ps4	ENSMUSG00000083757	processed_pseudogene	ribosomal protein S15A, pseudogene 4 [Source:MGI Symbol;Acc:MGI:3652187]	<0.01	<0.01	-1
Zfp619	ENSMUSG00000068959	protein_coding	zinc finger protein 619 [Source:MGI Symbol;Acc:MGI:1917477]	<0.01	0.015	-1
AI661453	ENSMUSG00000034382	protein_coding	expressed sequence AI661453 [Source:MGI Symbol;Acc:MGI:2146908]	<0.01	0.045	-1
Psmb8	ENSMUSG00000024338	protein_coding	proteasome (prosome, macropain) subunit, beta type 8 (large multifunctional peptidase 7) [Source:MGI Symbol;Acc:MGI:1346527]	<0.01	0.040	-1
Mvb12a	ENSMUSG00000031813	protein_coding	multivesicular body subunit 12A [Source:MGI Symbol;Acc:MGI:1920961]	<0.01	0.021	-1
Ap4b1	ENSMUSG00000032952	protein_coding	adaptor-related protein complex AP-4, beta 1 [Source:MGI Symbol;Acc:MGI:1337130]	<0.01	0.015	-1
Nabp1	ENSMUSG00000026107	protein_coding	nucleic acid binding protein 1 [Source:MGI Symbol;Acc:MGI:1923258]	<0.01	0.031	-1
Qpct	ENSMUSG00000024084	protein_coding	glutaminyl-peptide cyclotransferase (glutaminyl cyclase) [Source:MGI Symbol;Acc:MGI:1917786]	<0.01	<0.01	-1
Grk6	ENSMUSG00000074886	protein_coding	G protein-coupled receptor kinase 6 [Source:MGI Symbol;Acc:MGI:1347078]	<0.01	0.032	-1
RP23-378D16.5	ENSMUSG00000108950	antisense_RNA	RIKEN cDNA 9130015G15 gene [Source:MGI Symbol;Acc:MGI:1921817]	<0.01	0.042	-1
Glipr2	ENSMUSG00000028480	protein_coding	GLI pathogenesis-related 2 [Source:MGI Symbol;Acc:MGI:1917770]	<0.01	<0.01	-1
Thap6	ENSMUSG00000102644	transcribed_unitary_pseudogene	THAP domain containing 6 [Source:MGI Symbol;Acc:MGI:1922436]	<0.01	0.050	-1
Negr1	ENSMUSG00000040037	protein_coding	neuronal growth regulator 1 [Source:MGI Symbol;Acc:MGI:2444846]	<0.01	0.042	-1
Pyhin1	ENSMUSG00000043263	protein_coding	interferon activated gene 209 [Source:MGI Symbol;Acc:MGI:2138243]	<0.01	<0.01	-1
Slamf7	ENSMUSG00000038179	protein_coding	SLAM family member 7 [Source:MGI Symbol;Acc:MGI:1922595]	<0.01	0.026	-1
Rps2	ENSMUSG00000044533	protein_coding	ribosomal protein S2 [Source:MGI Symbol;Acc:MGI:105110]	<0.01	<0.01	-1
Cd82	ENSMUSG00000027215	protein_coding	CD82 antigen [Source:MGI Symbol;Acc:MGI:104651]	<0.01	0.034	-1
Tuft1	ENSMUSG00000005968	protein_coding	tuftelin 1 [Source:MGI Symbol;Acc:MGI:109572]	<0.01	0.034	-1
Gm13157	ENSMUSG00000078495	protein_coding	zinc finger protein 984 [Source:MGI Symbol;Acc:MGI:3651978]	<0.01	<0.01	-1
Pim1	ENSMUSG00000024014	protein_coding	proviral integration site 1 [Source:MGI Symbol;Acc:MGI:97584]	<0.01	0.035	-1
Adra1a	ENSMUSG00000045875	protein_coding	adrenergic receptor, alpha 1a [Source:MGI Symbol;Acc:MGI:104773]	<0.01	<0.01	-1
Pnpla2	ENSMUSG00000025509	protein_coding	patatin-like phospholipase domain containing 2 [Source:MGI Symbol;Acc:MGI:1914103]	<0.01	0.047	-1
Zfp125	ENSMUSG00000069755	transcribed_unprocessed_pseudogene	zinc finger protein 125 [Source:MGI Symbol;Acc:MGI:1336211]	<0.01	<0.01	-1
Klrd1	ENSMUSG00000030165	protein_coding	killer cell lectin-like receptor, subfamily D, member 1 [Source:MGI Symbol;Acc:MGI:1196275]	<0.01	0.029	-1
Acoxl	ENSMUSG00000027380	protein_coding	acyl-Coenzyme A oxidase-like [Source:MGI Symbol;Acc:MGI:1921371]	<0.01	0.037	-1
Ighv5-6	ENSMUSG00000094951	IG_V_gene	immunoglobulin heavy variable 5-6 [Source:MGI Symbol;Acc:MGI:4439815]	<0.01	<0.01	-1
Igkv6-23	ENSMUSG00000095630	IG_V_gene	immunoglobulin kappa variable 6-23 [Source:MGI Symbol;Acc:MGI:3711980]	<0.01	0.013	-1
Gm42573	ENSMUSG00000106736	processed_pseudogene	predicted gene 42573 [Source:MGI Symbol;Acc:MGI:5662710]	<0.01	<0.01	-1
Igkv1-88	ENSMUSG00000076535	IG_V_gene	immunoglobulin kappa chain variable 1-88 [Source:MGI Symbol;Acc:MGI:4439828]	<0.01	<0.01	-1
Malt1	ENSMUSG00000032688	protein_coding	MALT1 paracaspase [Source:MGI Symbol;Acc:MGI:2445027]	<0.01	0.020	-1
Susd3	ENSMUSG00000021384	protein_coding	sushi domain containing 3 [Source:MGI Symbol;Acc:MGI:1913579]	<0.01	0.018	-1
Sfxn2	ENSMUSG00000025036	protein_coding	sideroflexin 2 [Source:MGI Symbol;Acc:MGI:2137678]	<0.01	0.018	-1
Slfn2	ENSMUSG00000072620	protein_coding	schlafen 2 [Source:MGI Symbol;Acc:MGI:1313258]	<0.01	0.043	-1
Kpna2	ENSMUSG00000018362	protein_coding	karyopherin (importin) alpha 2 [Source:MGI Symbol;Acc:MGI:103561]	<0.01	0.013	-1
Polr1d	ENSMUSG00000029642	protein_coding	polymerase (RNA) I polypeptide D [Source:MGI Symbol;Acc:MGI:108403]	<0.01	<0.01	-1
Stat1	ENSMUSG00000026104	protein_coding	signal transducer and activator of transcription 1 [Source:MGI Symbol;Acc:MGI:103063]	<0.01	0.026	-1
Tec	ENSMUSG00000029217	protein_coding	tec protein tyrosine kinase [Source:MGI Symbol;Acc:MGI:98662]	<0.01	0.040	-1
Enpep	ENSMUSG00000028024	protein_coding	glutamyl aminopeptidase [Source:MGI Symbol;Acc:MGI:106645]	<0.01	0.042	-1
H2-Aa	ENSMUSG00000036594	protein_coding	histocompatibility 2, class II antigen A, alpha [Source:MGI Symbol;Acc:MGI:95895]	<0.01	0.024	-1
Elovl1	ENSMUSG00000006390	protein_coding	elongation of very long chain fatty acids (FEN1/Elo2, SUR4/Elo3, yeast)-like 1 [Source:MGI Symbol;Acc:MGI:1858959]	<0.01	0.027	-1
Iglv1	ENSMUSG00000076934	IG_V_gene	immunoglobulin lambda variable 1 [Source:MGI Symbol;Acc:MGI:96530]	<0.01	<0.01	-1
Ighv5-16	ENSMUSG00000094194	IG_V_gene	immunoglobulin heavy variable 5-16 [Source:MGI Symbol;Acc:MGI:4439556]	<0.01	0.035	-1
Ppp1r16a	ENSMUSG00000033819	protein_coding	protein phosphatase 1, regulatory (inhibitor) subunit 16A [Source:MGI Symbol;Acc:MGI:1920312]	<0.01	0.042	-1
Tmem51	ENSMUSG00000040616	protein_coding	transmembrane protein 51 [Source:MGI Symbol;Acc:MGI:2384874]	<0.01	0.035	-1
Ooep	ENSMUSG00000032346	protein_coding	oocyte expressed protein [Source:MGI Symbol;Acc:MGI:1915218]	<0.01	0.039	-1
Rtkn	ENSMUSG00000034930	protein_coding	rhotekin [Source:MGI Symbol;Acc:MGI:107371]	<0.01	0.032	-1
Gm26571	ENSMUSG00000096953	antisense_RNA	predicted gene, 26571 [Source:MGI Symbol;Acc:MGI:5477065]	<0.01	0.049	-1
Azgp1	ENSMUSG00000037053	protein_coding	alpha-2-glycoprotein 1, zinc [Source:MGI Symbol;Acc:MGI:103163]	<0.01	<0.01	-1
Igkv5-39	ENSMUSG00000076569	IG_V_gene	immunoglobulin kappa variable 5-39 [Source:MGI Symbol;Acc:MGI:2686255]	<0.01	0.027	-1
Gm15501	ENSMUSG00000087412	transcribed_processed_pseudogene	predicted pseudogene 15501 [Source:MGI Symbol;Acc:MGI:3704296]	<0.01	0.022	-1
H2-Eb1	ENSMUSG00000060586	protein_coding	histocompatibility 2, class II antigen E beta [Source:MGI Symbol;Acc:MGI:95901]	<0.01	<0.01	-1
Sparcl1	ENSMUSG00000029309	protein_coding	SPARC-like 1 [Source:MGI Symbol;Acc:MGI:108110]	<0.01	0.016	-1

Gm26894	ENSMUSG00000097453	lincRNA	predicted gene, 26894 [Source:MGI Symbol;Acc:MGI:5477388]	<0.01	0.025	-1
Creb3l1	ENSMUSG00000027230	protein_coding	cAMP responsive element binding protein 3-like 1 [Source:MGI Symbol;Acc:MGI:1347062]	<0.01	0.012	-1
Pisd-ps1	ENSMUSG00000082286	transcribed_unprocessed_pseudogene	phosphatidylserine decarboxylase, pseudogene 1 [Source:MGI Symbol;Acc:MGI:3842428]	<0.01	0.021	-1
1700047I17Rik2	ENSMUSG00000094103	protein_coding	RIKEN cDNA 1700047I17 gene 2 [Source:MGI Symbol;Acc:MGI:3714351]	<0.01	<0.01	-1
Pcna	ENSMUSG00000027342	protein_coding	proliferating cell nuclear antigen [Source:MGI Symbol;Acc:MGI:97503]	<0.01	<0.01	-1
Gm43198	ENSMUSG00000107222	TEC	predicted gene 43198 [Source:MGI Symbol;Acc:MGI:5663335]	<0.01	<0.01	-1
Syn2	ENSMUSG00000009394	protein_coding	synapsin II [Source:MGI Symbol;Acc:MGI:103020]	<0.01	0.012	-1
Psd4	ENSMUSG00000026979	protein_coding	pleckstrin and Sec7 domain containing 4 [Source:MGI Symbol;Acc:MGI:2674093]	<0.01	0.038	-1
Ighv1-7	ENSMUSG00000095200	IG_V_gene	immunoglobulin heavy variable V1-7 [Source:MGI Symbol;Acc:MGI:3704122]	<0.01	0.050	-1
Dixdc1	ENSMUSG00000032064	protein_coding	DIX domain containing 1 [Source:MGI Symbol;Acc:MGI:2679721]	<0.01	0.040	-1
Gm14964	ENSMUSG00000052188	antisense_RNA	predicted gene 14964 [Source:MGI Symbol;Acc:MGI:3641621]	<0.01	0.046	-1
Eif3k	ENSMUSG00000053565	protein_coding	eukaryotic translation initiation factor 3, subunit K [Source:MGI Symbol;Acc:MGI:1921080]	<0.01	0.043	-1
Tnfsf9	ENSMUSG00000035678	protein_coding	tumor necrosis factor (ligand) superfamily, member 9 [Source:MGI Symbol;Acc:MGI:1101058]	<0.01	<0.01	-1
Gm6652	ENSMUSG00000099858	processed_pseudogene	predicted gene 6652 [Source:MGI Symbol;Acc:MGI:3647244]	<0.01	<0.01	-1
Gm8730	ENSMUSG00000063696	processed_pseudogene	predicted pseudogene 8730 [Source:MGI Symbol;Acc:MGI:3644565]	<0.01	0.021	-1
Lpcat1	ENSMUSG00000021608	protein_coding	lysophosphatidylcholine acyltransferase 1 [Source:MGI Symbol;Acc:MGI:2384812]	<0.01	0.015	-1
Cyp2r1	ENSMUSG00000030670	protein_coding	cytochrome P450, family 2, subfamily r, polypeptide 1 [Source:MGI Symbol;Acc:MGI:2449771]	<0.01	0.016	-1
Ighg2c	ENSMUSG00000076612	IG_C_gene	immunoglobulin heavy constant gamma 2C [Source:MGI Symbol;Acc:MGI:2686979]	<0.01	<0.01	-1
Slc6a2	ENSMUSG00000055368	protein_coding	solute carrier family 6 (neurotransmitter transporter, noradrenalin), member 2 [Source:MGI Symbol;Acc:MGI:1270850]	<0.01	0.010	-1
Muc20	ENSMUSG00000035638	protein_coding	mucin 20 [Source:MGI Symbol;Acc:MGI:2385039]	<0.01	0.041	-1
Eif3j1	ENSMUSG00000027236	protein_coding	eukaryotic translation initiation factor 3, subunit J1 [Source:MGI Symbol;Acc:MGI:1925905]	<0.01	0.044	-1
Mmp12	ENSMUSG00000049723	protein_coding	matrix metallopeptidase 12 [Source:MGI Symbol;Acc:MGI:97005]	<0.01	0.017	-1
Pdia6	ENSMUSG00000020571	protein_coding	protein disulfide isomerase associated 6 [Source:MGI Symbol;Acc:MGI:1919103]	<0.01	0.015	-1
Sftpd	ENSMUSG00000021795	protein_coding	surfactant associated protein D [Source:MGI Symbol;Acc:MGI:109515]	<0.01	0.013	-1
Tpd52	ENSMUSG00000027506	protein_coding	tumor protein D52 [Source:MGI Symbol;Acc:MGI:107749]	<0.01	0.029	-1
Cep19	ENSMUSG00000035790	protein_coding	centrosomal protein 19 [Source:MGI Symbol;Acc:MGI:1914244]	<0.01	0.017	-1
Sfn	ENSMUSG00000047281	protein_coding	stratifin [Source:MGI Symbol;Acc:MGI:1891831]	<0.01	<0.01	-1
Acot1	ENSMUSG00000072949	protein_coding	acyl-CoA thioesterase 1 [Source:MGI Symbol;Acc:MGI:1349396]	<0.01	<0.01	-1
Asah1	ENSMUSG00000031591	protein_coding	N-acylsphingosine amidohydrolase 1 [Source:MGI Symbol;Acc:MGI:1277124]	<0.01	0.050	-1
Lamp3	ENSMUSG00000041247	protein_coding	lysosomal-associated membrane protein 3 [Source:MGI Symbol;Acc:MGI:2441659]	<0.01	0.037	-1
Cd79a	ENSMUSG00000003379	protein_coding	CD79A antigen (immunoglobulin-associated alpha) [Source:MGI Symbol;Acc:MGI:101774]	<0.01	0.013	-1
Pcf11	ENSMUSG00000041328	protein_coding	PCF11 cleavage and polyadenylation factor subunit [Source:MGI Symbol;Acc:MGI:1919579]	<0.01	0.050	-1
H2-Q6	ENSMUSG00000073409	protein_coding	histocompatibility 2, Q region locus 6 [Source:MGI Symbol;Acc:MGI:95935]	<0.01	<0.01	-1
Phldb2	ENSMUSG00000033149	protein_coding	pleckstrin homology like domain, family B, member 2 [Source:MGI Symbol;Acc:MGI:2444981]	<0.01	<0.01	-1
Ddhd1	ENSMUSG00000037697	protein_coding	DDHD domain containing 1 [Source:MGI Symbol;Acc:MGI:2150302]	<0.01	0.016	-1
Ttf2	ENSMUSG00000033222	protein_coding	transcription termination factor, RNA polymerase II [Source:MGI Symbol;Acc:MGI:1921294]	<0.01	0.025	-1
Tmprss4	ENSMUSG00000032091	protein_coding	transmembrane protease, serine 4 [Source:MGI Symbol;Acc:MGI:2384877]	<0.01	0.035	-1
Olfml2a	ENSMUSG00000046618	protein_coding	olfactomedin-like 2A [Source:MGI Symbol;Acc:MGI:2444741]	<0.01	0.042	-1
Nptx1	ENSMUSG00000025582	protein_coding	neuronal pentraxin 1 [Source:MGI Symbol;Acc:MGI:107811]	<0.01	<0.01	-1
Gm42644	ENSMUSG00000105843	TEC	predicted gene, 19439 [Source:MGI Symbol;Acc:MGI:5011624]	<0.01	<0.01	-1
Zbp1	ENSMUSG00000027514	protein_coding	Z-DNA binding protein 1 [Source:MGI Symbol;Acc:MGI:1927449]	<0.01	0.024	-1
Hmga1	ENSMUSG00000046711	protein_coding	high mobility group AT-hook 1 [Source:MGI Symbol;Acc:MGI:96160]	<0.01	<0.01	-1
Mif4gd	ENSMUSG00000020743	protein_coding	MIF4G domain containing [Source:MGI Symbol;Acc:MGI:1916924]	<0.01	0.037	-1
Dck	ENSMUSG00000029366	protein_coding	deoxycytidine kinase [Source:MGI Symbol;Acc:MGI:102726]	<0.01	<0.01	-1
N4bp2os	ENSMUSG00000106978	processed_transcript	NEDD4 binding protein 2, opposite strand [Source:MGI Symbol;Acc:MGI:1921484]	<0.01	<0.01	-1
Il4i1	ENSMUSG00000074141	protein_coding	interleukin 4 induced 1 [Source:MGI Symbol;Acc:MGI:109552]	<0.01	<0.01	-1
Khdrbs3	ENSMUSG00000022332	protein_coding	KH domain containing, RNA binding, signal transduction associated 3 [Source:MGI Symbol;Acc:MGI:1313312]	<0.01	0.015	-1
Klhl9	ENSMUSG00000070923	protein_coding	kelch-like 9 [Source:MGI Symbol;Acc:MGI:2180122]	<0.01	0.032	-1
Vnn1	ENSMUSG00000037440	protein_coding	vanin 1 [Source:MGI Symbol;Acc:MGI:108395]	<0.01	<0.01	-1
Spon2	ENSMUSG00000037379	protein_coding	spondin 2, extracellular matrix protein [Source:MGI Symbol;Acc:MGI:1923724]	<0.01	<0.01	-1
Ighv10-3	ENSMUSG00000095700	IG_V_gene	immunoglobulin heavy variable V10-3 [Source:MGI Symbol;Acc:MGI:3648785]	<0.01	0.030	-1
Ighv1-39	ENSMUSG00000095130	IG_V_gene	immunoglobulin heavy variable 1-39 [Source:MGI Symbol;Acc:MGI:4439888]	<0.01	0.039	-1
Abcb4	ENSMUSG00000042476	protein_coding	ATP-binding cassette, sub-family B (MDR/TAP), member 4 [Source:MGI Symbol;Acc:MGI:97569]	<0.01	0.024	-1
Snapc1	ENSMUSG00000021113	protein_coding	small nuclear RNA activating complex, polypeptide 1 [Source:MGI Symbol;Acc:MGI:1922877]	<0.01	0.011	-1
Gm11361	ENSMUSG00000061330	processed_pseudogene	predicted pseudogene 11361 [Source:MGI Symbol;Acc:MGI:3649931]	<0.01	<0.01	-1
Lyrm7	ENSMUSG00000020268	protein_coding	LYR motif containing 7 [Source:MGI Symbol;Acc:MGI:1922780]	<0.01	0.037	-1
Trp53i11	ENSMUSG00000068735	protein_coding	transformation related protein 53 inducible protein 11 [Source:MGI Symbol;Acc:MGI:2670995]	<0.01	0.031	-1
Gm10709	ENSMUSG00000074516	processed_pseudogene	predicted gene 10709 [Source:MGI Symbol;Acc:MGI:3642754]	<0.01	<0.01	-1
Slc28a2	ENSMUSG00000027219	protein_coding	solute carrier family 28 (sodium-coupled nucleoside transporter), member 2 [Source:MGI Symbol;Acc:MGI:1913105]	<0.01	0.040	-1
Apol7c	ENSMUSG00000044309	protein_coding	apolipoprotein L 7c [Source:MGI Symbol;Acc:MGI:1920912]	<0.01	<0.01	-1
Arl5c	ENSMUSG00000038352	protein_coding	ADP-ribosylation factor-like 5C [Source:MGI Symbol;Acc:MGI:3028577]	<0.01	0.022	-1
Cnp	ENSMUSG00000006782	protein_coding	2',3'-cyclic nucleotide 3' phosphodiesterase [Source:MGI Symbol;Acc:MGI:88437]	<0.01	0.028	-1
Myo1g	ENSMUSG00000020437	protein_coding	myosin IG [Source:MGI Symbol;Acc:MGI:1927091]	<0.01	0.049	-1
Ms4a1	ENSMUSG00000024673	protein_coding	membrane-spanning 4-domains, subfamily A, member 1 [Source:MGI Symbol;Acc:MGI:88321]	<0.01	0.034	-1
Zzz3	ENSMUSG00000039068	protein_coding	zinc finger, ZZ domain containing 3 [Source:MGI Symbol;Acc:MGI:1920453]	<0.01	0.014	-1
1110001J03Rik	ENSMUSG00000019689	protein_coding	formation of mitochondrial complex V assembly factor 1 [Source:MGI Symbol;Acc:MGI:1913367]	<0.01	<0.01	-1
Junos	ENSMUSG00000087366	antisense_RNA	jun proto-oncogene, opposite strand [Source:MGI Symbol;Acc:MGI:2652837]	<0.01	0.026	-1
Sftpa1	ENSMUSG00000021789	protein_coding	surfactant associated protein A1 [Source:MGI Symbol;Acc:MGI:109518]	<0.01	0.017	-1
Arf6	ENSMUSG00000044147	protein_coding	ADP-ribosylation factor 6 [Source:MGI Symbol;Acc:MGI:99435]	<0.01	<0.01	-1
Tgtp1	ENSMUSG00000078922	protein_coding	T cell specific GTPase 1 [Source:MGI Symbol;Acc:MGI:98734]	<0.01	0.027	-1
Haus8	ENSMUSG00000035439	protein_coding	4HAUS augmin-like complex, subunit 8 [Source:MGI Symbol;Acc:MGI:1923728]	<0.01	<0.01	-1
Tcea1	ENSMUSG00000033813	protein_coding	transcription elongation factor A (SII) 1 [Source:MGI Symbol;Acc:MGI:1196624]	<0.01	<0.01	-1
Hdac1	ENSMUSG00000028800	protein_coding	histone deacetylase 1 [Source:MGI Symbol;Acc:MGI:108086]	<0.01	<0.01	-1
Chil3	ENSMUSG00000040809	protein_coding	chitinase-like 3 [Source:MGI Symbol;Acc:MGI:1330860]	<0.01	<0.01	-1
Gm20458	ENSMUSG00000090996	protein_coding	predicted gene 20458 [Source:MGI Symbol;Acc:MGI:5141923]	<0.01	<0.01	-1
Ighv1-77	ENSMUSG00000096452	IG_V_gene	immunoglobulin heavy variable 1-77 [Source:MGI Symbol;Acc:MGI:4439670]	<0.01	0.012	-1
1700105P06Rik	ENSMUSG00000099923	antisense_RNA	RIKEN cDNA 1700105P06 gene [Source:MGI Symbol;Acc:MGI:1915479]	<0.01	0.012	-1
Cd79b	ENSMUSG00000040592	protein_coding	CD79B antigen [Source:MGI Symbol;Acc:MGI:96431]	<0.01	0.039	-1
Psmb5	ENSMUSG00000022193	protein_coding	proteasome (prosome, macropain) subunit, beta type 5 [Source:MGI Symbol;Acc:MGI:1194513]	<0.01	0.042	-1
Pigx	ENSMUSG00000023791	protein_coding	phosphatidylinositol glycan anchor biosynthesis, class X [Source:MGI Symbol;Acc:MGI:1919334]	<0.01	0.020	-1

Morf4l1	ENSMUSG00000062270	protein_coding	mortality factor 4 like 1 [Source:MGI Symbol;Acc:MGI:1096551]	<0.01	0.047	-1
Dtx4	ENSMUSG00000039982	protein_coding	deltex 4, E3 ubiquitin ligase [Source:MGI Symbol;Acc:MGI:2672905]	<0.01	0.017	-1
Gclc	ENSMUSG00000032350	protein_coding	glutamate-cysteine ligase, catalytic subunit [Source:MGI Symbol;Acc:MGI:104990]	<0.01	<0.01	-1
Zw10	ENSMUSG00000032264	protein_coding	zw10 kinetochore protein [Source:MGI Symbol;Acc:MGI:1349478]	<0.01	0.047	-1
Sema4d	ENSMUSG00000021451	protein_coding	sema domain, immunoglobulin domain (Ig), transmembrane domain (TM) and short cytoplasmic domain, (semaphorin) 4D [Source:MGI Symbol;Acc:MGI:109244]	<0.01	<0.01	-1
Mafb	ENSMUSG00000074622	protein_coding	v-maf musculoaponeurotic fibrosarcoma oncogene family, protein B (avian) [Source:MGI Symbol;Acc:MGI:104555]	<0.01	0.050	-1
Kazald1	ENSMUSG00000025213	protein_coding	Kazal-type serine peptidase inhibitor domain 1 [Source:MGI Symbol;Acc:MGI:2147606]	<0.01	<0.01	-1
Gm16984	ENSMUSG00000085962	antisense_RNA	predicted gene, 16984 [Source:MGI Symbol;Acc:MGI:4439908]	<0.01	<0.01	-1
Sirpb1c	ENSMUSG00000074677	protein_coding	signal-regulatory protein beta 1C [Source:MGI Symbol;Acc:MGI:3807521]	<0.01	<0.01	-1
Tyrobp	ENSMUSG00000030579	protein_coding	TYRO protein tyrosine kinase binding protein [Source:MGI Symbol;Acc:MGI:1277211]	<0.01	0.017	-1
Mcfd2	ENSMUSG00000024150	protein_coding	multiple coagulation factor deficiency 2 [Source:MGI Symbol;Acc:MGI:2183439]	<0.01	0.017	-1
Arg2	ENSMUSG00000021125	protein_coding	arginase type II [Source:MGI Symbol;Acc:MGI:1330806]	<0.01	<0.01	-1
Use1	ENSMUSG00000002395	protein_coding	unconventional SNARE in the ER 1 homolog (S. cerevisiae) [Source:MGI Symbol;Acc:MGI:1914273]	<0.01	0.029	-1
Cyp3a13	ENSMUSG00000029727	protein_coding	cytochrome P450, family 3, subfamily a, polypeptide 13 [Source:MGI Symbol;Acc:MGI:88610]	<0.01	<0.01	-1
Ifngr2	ENSMUSG00000022965	protein_coding	interferon gamma receptor 2 [Source:MGI Symbol;Acc:MGI:107654]	<0.01	0.039	-1
Ntan1	ENSMUSG00000022681	protein_coding	N-terminal Asn amidase [Source:MGI Symbol;Acc:MGI:108471]	<0.01	<0.01	-1
Igkv12-46	ENSMUSG00000076564	IG_V_gene	immunoglobulin kappa variable 12-46 [Source:MGI Symbol;Acc:MGI:4439773]	<0.01	<0.01	-1
BC051226	ENSMUSG00000092564	antisense_RNA	cDNA sequence BC051226 [Source:MGI Symbol;Acc:MGI:3039585]	<0.01	0.027	-1
Cd14	ENSMUSG00000051439	protein_coding	CD14 antigen [Source:MGI Symbol;Acc:MGI:88318]	<0.01	0.040	-1
Gm20033	ENSMUSG00000097640	processed_transcript	predicted gene, 20033 [Source:MGI Symbol;Acc:MGI:5012218]	<0.01	0.047	-1
Il1b	ENSMUSG00000027398	protein_coding	interleukin 1 beta [Source:MGI Symbol;Acc:MGI:96543]	<0.01	0.044	-1
Arhgap9	ENSMUSG00000040345	protein_coding	Rho GTPase activating protein 9 [Source:MGI Symbol;Acc:MGI:2143764]	<0.01	0.027	-1
Rnps1	ENSMUSG00000034681	protein_coding	ribonucleic acid binding protein S1 [Source:MGI Symbol;Acc:MGI:97960]	<0.01	<0.01	-1
Tmem221	ENSMUSG00000043664	protein_coding	transmembrane protein 221 [Source:MGI Symbol;Acc:MGI:3525074]	<0.01	<0.01	-1
H2-D1	ENSMUSG00000073411	protein_coding	histocompatibility 2, D region locus 1 [Source:MGI Symbol;Acc:MGI:95896]	<0.01	<0.01	-1
Kcnf1	ENSMUSG00000051726	protein_coding	potassium voltage-gated channel, subfamily F, member 1 [Source:MGI Symbol;Acc:MGI:2687399]	<0.01	0.026	-1
Lman2l	ENSMUSG00000001143	protein_coding	lectin, mannose-binding 2-like [Source:MGI Symbol;Acc:MGI:2443010]	<0.01	0.023	-1
Ly6e	ENSMUSG00000022587	protein_coding	lymphocyte antigen 6 complex, locus E [Source:MGI Symbol;Acc:MGI:106651]	<0.01	0.041	-1
Rps15a-ps7	ENSMUSG00000081087	processed_pseudogene	ribosomal protein S15A, pseudogene 7 [Source:MGI Symbol;Acc:MGI:3650538]	<0.01	<0.01	-1
Hnrnpa3	ENSMUSG00000059005	protein_coding	heterogeneous nuclear ribonucleoprotein A3 [Source:MGI Symbol;Acc:MGI:1917171]	<0.01	0.021	-1
Chst4	ENSMUSG00000035930	protein_coding	carbohydrate (chondroitin 6/keratan) sulfotransferase 4 [Source:MGI Symbol;Acc:MGI:1349479]	<0.01	0.043	-1
Rer1	ENSMUSG00000029048	protein_coding	retention in endoplasmic reticulum sorting receptor 1 [Source:MGI Symbol;Acc:MGI:1915080]	<0.01	0.040	-1
Arhgef38	ENSMUSG00000040969	protein_coding	Rho guanine nucleotide exchange factor (GEF) 38 [Source:MGI Symbol;Acc:MGI:1924919]	<0.01	0.019	-1
Gbp6	ENSMUSG00000104713	protein_coding	guanylate binding protein 6 [Source:MGI Symbol;Acc:MGI:2140937]	<0.01	<0.01	-1
Rpl9-ps7	ENSMUSG00000047965	processed_pseudogene	ribosomal protein L9, pseudogene 7 [Source:MGI Symbol;Acc:MGI:3642824]	<0.01	0.028	-1
I830077J02Rik	ENSMUSG00000074342	protein_coding	RIKEN cDNA I830077J02 gene [Source:MGI Symbol;Acc:MGI:3588284]	<0.01	<0.01	-1
Igkv14-100	ENSMUSG00000096515	IG_V_gene	immunoglobulin kappa chain variable 14-100 [Source:MGI Symbol;Acc:MGI:4439559]	<0.01	0.016	-1
Hba-a2	ENSMUSG00000069917	protein_coding	hemoglobin alpha, adult chain 2 [Source:MGI Symbol;Acc:MGI:96016]	<0.01	0.019	-1
Coro1a	ENSMUSG00000030707	protein_coding	coronin, actin binding protein 1A [Source:MGI Symbol;Acc:MGI:1345961]	<0.01	<0.01	-1
Cfh	ENSMUSG00000026365	protein_coding	complement component factor h [Source:MGI Symbol;Acc:MGI:88385]	<0.01	<0.01	-1
Ifit1bl1	ENSMUSG00000079339	protein_coding	interferon induced protein with tetratricpeptide repeats 1B like 1 [Source:MGI Symbol;Acc:MGI:3650685]	<0.01	0.011	-1
H2-Eb2	ENSMUSG00000067341	protein_coding	histocompatibility 2, class II antigen E beta2 [Source:MGI Symbol;Acc:MGI:95902]	<0.01	<0.01	-1
Ehf	ENSMUSG00000012350	protein_coding	ets homologous factor [Source:MGI Symbol;Acc:MGI:1270840]	<0.01	0.030	-1
Appbp2os	ENSMUSG00000085628	antisense_RNA	amyloid beta precursor protein (cytoplasmic tail) binding protein 2, opposite strand [Source:MGI Symbol;Acc:MGI:3603817]	<0.01	0.015	-1
Sdc4	ENSMUSG00000017009	protein_coding	syndecan 4 [Source:MGI Symbol;Acc:MGI:1349164]	<0.01	<0.01	-1
Mal	ENSMUSG00000027375	protein_coding	myelin and lymphocyte protein, T cell differentiation protein [Source:MGI Symbol;Acc:MGI:892970]	<0.01	0.023	-1
Cdc26	ENSMUSG00000066149	protein_coding	cell division cycle 26 [Source:MGI Symbol;Acc:MGI:1913690]	<0.01	0.033	-1
Rasl11a	ENSMUSG00000029641	protein_coding	RAS-like, family 11, member A [Source:MGI Symbol;Acc:MGI:1916145]	<0.01	<0.01	-1
Ptpn6	ENSMUSG00000004266	protein_coding	protein tyrosine phosphatase, non-receptor type 6 [Source:MGI Symbol;Acc:MGI:96055]	<0.01	0.013	-1
Rerg	ENSMUSG00000030222	protein_coding	RAS-like, estrogen-regulated, growth-inhibitor [Source:MGI Symbol;Acc:MGI:2665139]	<0.01	0.020	-1
Col26a1	ENSMUSG00000004415	protein_coding	collagen, type XXVI, alpha 1 [Source:MGI Symbol;Acc:MGI:2155345]	<0.01	<0.01	-1
Eno1	ENSMUSG00000063524	protein_coding	enolase 1, alpha non-neuron [Source:MGI Symbol;Acc:MGI:95393]	<0.01	<0.01	-1
4930481A15Rik	ENSMUSG00000086938	antisense_RNA	RIKEN cDNA 4930481A15 gene [Source:MGI Symbol;Acc:MGI:1922181]	<0.01	<0.01	-1
C1s1	ENSMUSG00000038521	protein_coding	complement component 1, s subcomponent 1 [Source:MGI Symbol;Acc:MGI:1355312]	<0.01	0.028	-1
Hdhd2	ENSMUSG00000025421	protein_coding	haloacid dehalogenase-like hydrolase domain containing 2 [Source:MGI Symbol;Acc:MGI:1924237]	<0.01	0.022	-1
Ahnak2	ENSMUSG00000072812	protein_coding	AHNAK nucleoprotein 2 [Source:MGI Symbol;Acc:MGI:2144831]	<0.01	0.014	-1
Chp1	ENSMUSG00000014077	protein_coding	calcineurin-like EF hand protein 1 [Source:MGI Symbol;Acc:MGI:1927185]	<0.01	0.041	-1
Msc	ENSMUSG00000025930	protein_coding	musculin [Source:MGI Symbol;Acc:MGI:1333884]	<0.01	<0.01	-1
Adm	ENSMUSG00000030790	protein_coding	adrenomedullin [Source:MGI Symbol;Acc:MGI:108058]	<0.01	<0.01	-1
Igkv19-93	ENSMUSG00000098814	IG_V_gene	immunoglobulin kappa chain variable 19-93 [Source:MGI Symbol;Acc:MGI:107617]	<0.01	0.013	-1
Ighv6-6	ENSMUSG00000076680	IG_V_gene	immunoglobulin heavy variable 6-6 [Source:MGI Symbol;Acc:MGI:4439619]	<0.01	0.022	-1
Arl6ip1	ENSMUSG00000030654	protein_coding	ADP-ribosylation factor-like 6 interacting protein 1 [Source:MGI Symbol;Acc:MGI:1858943]	<0.01	<0.01	-1
Spink5	ENSMUSG00000055561	protein_coding	serine peptidase inhibitor, Kazal type 5 [Source:MGI Symbol;Acc:MGI:1919682]	<0.01	<0.01	-1
A230020J21Rik	ENSMUSG00000097845	antisense_RNA	RIKEN cDNA A230020J21 gene [Source:MGI Symbol;Acc:MGI:3704207]	<0.01	0.048	-1
Cebpa	ENSMUSG00000034957	protein_coding	CCAAT/enhancer binding protein (C/EBP), alpha [Source:MGI Symbol;Acc:MGI:99480]	<0.01	<0.01	-1
Mtmr7	ENSMUSG00000039431	protein_coding	myotubularin related protein 7 [Source:MGI Symbol;Acc:MGI:1891693]	<0.01	<0.01	-1
Igkv12-44	ENSMUSG00000096422	IG_V_gene	immunoglobulin kappa variable 12-44 [Source:MGI Symbol;Acc:MGI:4439775]	<0.01	0.021	-1
Trim12a	ENSMUSG00000066258	protein_coding	tripartite motif-containing 12A [Source:MGI Symbol;Acc:MGI:1923931]	<0.01	<0.01	-1
Entpd1	ENSMUSG00000048120	protein_coding	ectonucleoside triphosphate diphosphohydrolase 1 [Source:MGI Symbol;Acc:MGI:102805]	<0.01	0.016	-1
Tmem171	ENSMUSG00000052485	protein_coding	transmembrane protein 171 [Source:MGI Symbol;Acc:MGI:2685751]	<0.01	0.040	-1
Decr2	ENSMUSG00000036775	protein_coding	2-4-dienoyl-Coenzyme A reductase 2, peroxisomal [Source:MGI Symbol;Acc:MGI:1347059]	<0.01	0.050	-1
Asprv1	ENSMUSG00000033508	protein_coding	aspartic peptidase, retroviral-like 1 [Source:MGI Symbol;Acc:MGI:1915105]	<0.01	<0.01	-1
Spop	ENSMUSG00000057522	protein_coding	speckle-type POZ protein [Source:MGI Symbol;Acc:MGI:1343085]	<0.01	0.019	-1
Gm9888	ENSMUSG00000052724	antisense_RNA	predicted gene 9888 [Source:MGI Symbol;Acc:MGI:3642202]	<0.01	0.020	-1
Rnase4	ENSMUSG00000021876	protein_coding	ribonuclease, RNase A family 4 [Source:MGI Symbol;Acc:MGI:1926217]	<0.01	<0.01	-1
Arntl	ENSMUSG00000055116	protein_coding	aryl hydrocarbon receptor nuclear translocator-like [Source:MGI Symbol;Acc:MGI:1096381]	<0.01	<0.01	-1
Plac8	ENSMUSG00000029322	protein_coding	placenta-specific 8 [Source:MGI Symbol;Acc:MGI:2445289]	<0.01	<0.01	-1
Rps15a-ps6	ENSMUSG00000083022	processed_pseudogene	ribosomal protein S15A, pseudogene 6 [Source:MGI Symbol;Acc:MGI:3650298]	<0.01	0.013	-1
Ighv1-76	ENSMUSG00000093896	IG_V_gene	immunoglobulin heavy variable 1-76 [Source:MGI Symbol;Acc:MGI:4439737]	<0.01	<0.01	-1
Spsb3	ENSMUSG00000024160	protein_coding	splA/ryanodine receptor domain and SOCS box containing 3 [Source:MGI Symbol;Acc:MGI:1891471]	<0.01	<0.01	-1

Ifi27l2a	ENSMUSG00000079017	protein_coding	interferon, alpha-inducible protein 27 like 2A [Source:MGI Symbol;Acc:MGI:1924183]	<0.01	0.017	-1
Fkbp4	ENSMUSG00000030357	protein_coding	FK506 binding protein 4 [Source:MGI Symbol;Acc:MGI:95543]	<0.01	0.013	-1
Klrb1c	ENSMUSG00000030325	protein_coding	killer cell lectin-like receptor subfamily B member 1C [Source:MGI Symbol;Acc:MGI:107538]	<0.01	0.028	-1
Serp1	ENSMUSG00000027808	protein_coding	stress-associated endoplasmic reticulum protein 1 [Source:MGI Symbol;Acc:MGI:92638]	<0.01	0.036	-1
Sspn	ENSMUSG00000030255	protein_coding	sarcospan [Source:MGI Symbol;Acc:MGI:1353511]	<0.01	0.012	-1
Bhlha15	ENSMUSG00000052271	protein_coding	basic helix-loop-helix family, member a15 [Source:MGI Symbol;Acc:MGI:891976]	<0.01	0.028	-1
Bcan	ENSMUSG00000004892	protein_coding	brevican [Source:MGI Symbol;Acc:MGI:1096385]	<0.01	0.017	-1
Gm14057	ENSMUSG00000089662	transcribed_processed_pseudogene	predicted gene 14057 [Source:MGI Symbol;Acc:MGI:3705433]	<0.01	0.027	-1
Cnbd2	ENSMUSG00000038085	protein_coding	cyclic nucleotide binding domain containing 2 [Source:MGI Symbol;Acc:MGI:1918123]	<0.01	<0.01	-1
Bco2	ENSMUSG00000032066	protein_coding	beta-carotene oxygenase 2 [Source:MGI Symbol;Acc:MGI:2177469]	<0.01	0.029	-1
Hist1h2bg	ENSMUSG00000058385	protein_coding	histone cluster 1, H2bg [Source:MGI Symbol;Acc:MGI:2448386]	<0.01	<0.01	-1
Apobec1	ENSMUSG00000040613	protein_coding	apolipoprotein B mRNA editing enzyme, catalytic polypeptide 1 [Source:MGI Symbol;Acc:MGI:103298]	<0.01	0.011	-1
Igkv14-111	ENSMUSG00000095771	IG_V_gene	immunoglobulin kappa variable 14-111 [Source:MGI Symbol;Acc:MGI:4439863]	<0.01	<0.01	-1
Arl3	ENSMUSG00000025035	protein_coding	ADP-ribosylation factor-like 3 [Source:MGI Symbol;Acc:MGI:1929699]	<0.01	0.018	-1
Serpina3c	ENSMUSG00000066361	protein_coding	serine (or cysteine) peptidase inhibitor, clade A, member 3C [Source:MGI Symbol;Acc:MGI:102848]	<0.01	<0.01	-1
Miip	ENSMUSG00000029022	protein_coding	migration and invasion inhibitory protein [Source:MGI Symbol;Acc:MGI:106506]	<0.01	0.018	-1
Gnpda1	ENSMUSG00000052102	protein_coding	glucosamine-6-phosphate deaminase 1 [Source:MGI Symbol;Acc:MGI:1347054]	<0.01	0.019	-1
Rab27a	ENSMUSG00000032202	protein_coding	RAB27A, member RAS oncogene family [Source:MGI Symbol;Acc:MGI:1861441]	<0.01	<0.01	-1
Hes2	ENSMUSG00000028940	protein_coding	hairy and enhancer of split 2 (Drosophila) [Source:MGI Symbol;Acc:MGI:1098624]	<0.01	0.019	-1
Rbpj	ENSMUSG00000039191	protein_coding	recombination signal binding protein for immunoglobulin kappa J region [Source:MGI Symbol;Acc:MGI:96522]	<0.01	<0.01	-1
Ms4a4c	ENSMUSG00000024675	protein_coding	membrane-spanning 4-domains, subfamily A, member 4C [Source:MGI Symbol;Acc:MGI:1927656]	<0.01	0.010	-1
Sord	ENSMUSG00000027227	protein_coding	sorbitol dehydrogenase [Source:MGI Symbol;Acc:MGI:98266]	<0.01	<0.01	-1
Apoc2	ENSMUSG00000002992	protein_coding	apolipoprotein C-II [Source:MGI Symbol;Acc:MGI:88054]	<0.01	<0.01	-1
Rplp1	ENSMUSG00000007892	protein_coding	ribosomal protein, large, P1 [Source:MGI Symbol;Acc:MGI:1927099]	<0.01	<0.01	-1
Nxph3	ENSMUSG00000046719	protein_coding	neurexophilin 3 [Source:MGI Symbol;Acc:MGI:1336188]	<0.01	0.013	-1
Igkv13-84	ENSMUSG00000076538	IG_V_gene	immunoglobulin kappa chain variable 13-84 [Source:MGI Symbol;Acc:MGI:96514]	<0.01	<0.01	-1
1810062G17Rik	ENSMUSG00000027713	protein_coding	RIKEN cDNA 1810062G17 gene [Source:MGI Symbol;Acc:MGI:1919532]	<0.01	<0.01	-1
4930550C14Rik	ENSMUSG00000005131	protein_coding	RIKEN cDNA 4930550C14 gene [Source:MGI Symbol;Acc:MGI:1922561]	<0.01	0.011	-1
Slc43a2	ENSMUSG00000038178	protein_coding	solute carrier family 43, member 2 [Source:MGI Symbol;Acc:MGI:2442746]	<0.01	0.030	-1
Ccr10	ENSMUSG00000044052	protein_coding	chemokine (C-C motif) receptor 10 [Source:MGI Symbol;Acc:MGI:1096320]	<0.01	0.010	-1
Tmem154	ENSMUSG00000056498	protein_coding	transmembrane protein 154 [Source:MGI Symbol;Acc:MGI:2444725]	<0.01	0.026	-1
Ighv1-59	ENSMUSG00000095197	IG_V_gene	immunoglobulin heavy variable V1-59 [Source:MGI Symbol;Acc:MGI:3644474]	<0.01	0.021	-1
Cyp4f18	ENSMUSG00000003484	protein_coding	cytochrome P450, family 4, subfamily f, polypeptide 18 [Source:MGI Symbol;Acc:MGI:1919304]	<0.01	0.050	-1
Tnfaip8	ENSMUSG00000062210	protein_coding	tumor necrosis factor, alpha-induced protein 8 [Source:MGI Symbol;Acc:MGI:2147191]	<0.01	0.025	-1
Tgfbi	ENSMUSG00000035493	protein_coding	transforming growth factor, beta induced [Source:MGI Symbol;Acc:MGI:99959]	<0.01	<0.01	-1
Cxcr6	ENSMUSG00000048521	protein_coding	chemokine (C-X-C motif) receptor 6 [Source:MGI Symbol;Acc:MGI:1934582]	<0.01	0.038	-1
Adcy7	ENSMUSG00000031659	protein_coding	adenylate cyclase 7 [Source:MGI Symbol;Acc:MGI:102891]	<0.01	0.022	-1
Igkv15-103	ENSMUSG00000076523	IG_V_gene	immunoglobulin kappa chain variable 15-103 [Source:MGI Symbol;Acc:MGI:96513]	<0.01	<0.01	-1
Amigo2	ENSMUSG00000048218	protein_coding	adhesion molecule with Ig like domain 2 [Source:MGI Symbol;Acc:MGI:2145995]	<0.01	0.010	-1
Acad11	ENSMUSG00000090150	protein_coding	acyl-Coenzyme A dehydrogenase family, member 11 [Source:MGI Symbol;Acc:MGI:2143169]	<0.01	0.032	-1
Wfdc3	ENSMUSG00000076434	protein_coding	WAP four-disulfide core domain 3 [Source:MGI Symbol;Acc:MGI:1923897]	<0.01	0.024	-1
Ccz1	ENSMUSG00000029617	protein_coding	CCZ1 vacuolar protein trafficking and biogenesis associated [Source:MGI Symbol;Acc:MGI:2141070]	<0.01	0.019	-1
Hmgn2	ENSMUSG00000003038	protein_coding	high mobility group nucleosomal binding domain 2 [Source:MGI Symbol;Acc:MGI:96136]	<0.01	<0.01	-1
Ywhaq	ENSMUSG00000076432	protein_coding	tyrosine 3-monooxygenase/tryptophan 5-monooxygenase activation protein theta [Source:MGI Symbol;Acc:MGI:891963]	<0.01	0.014	-1
Cyp26b1	ENSMUSG00000063415	protein_coding	cytochrome P450, family 26, subfamily b, polypeptide 1 [Source:MGI Symbol;Acc:MGI:2176159]	<0.01	0.016	-1
Atp6v0c	ENSMUSG00000024121	protein_coding	ATPase, H+ transporting, lysosomal V0 subunit C [Source:MGI Symbol;Acc:MGI:88116]	<0.01	<0.01	-1
Snhg3	ENSMUSG00000085241	processed_transcript	small nucleolar RNA host gene 3 [Source:MGI Symbol;Acc:MGI:2684817]	<0.01	0.028	-1
Ighv14-2	ENSMUSG00000095583	IG_V_gene	immunoglobulin heavy variable 14-2 [Source:MGI Symbol;Acc:MGI:4439607]	<0.01	<0.01	-1
Trim14	ENSMUSG00000039853	protein_coding	tripartite motif-containing 14 [Source:MGI Symbol;Acc:MGI:1921985]	<0.01	<0.01	-1
Fam84b	ENSMUSG00000072568	protein_coding	family with sequence similarity 84, member B [Source:MGI Symbol;Acc:MGI:3026924]	<0.01	0.031	-1
Nxpe4	ENSMUSG00000044229	protein_coding	neurexophilin and PC-esterase domain family, member 4 [Source:MGI Symbol;Acc:MGI:1924792]	<0.01	<0.01	-1
Rps18	ENSMUSG00000008668	protein_coding	ribosomal protein S18 [Source:MGI Symbol;Acc:MGI:98146]	<0.01	<0.01	-1
Areg	ENSMUSG00000029378	protein_coding	amphiregulin [Source:MGI Symbol;Acc:MGI:88068]	<0.01	<0.01	-1
Odc1	ENSMUSG00000011179	protein_coding	ornithine decarboxylase, structural 1 [Source:MGI Symbol;Acc:MGI:97402]	<0.01	<0.01	-1
Tecpr1	ENSMUSG00000066621	protein_coding	tectonin beta-propeller repeat containing 1 [Source:MGI Symbol;Acc:MGI:1917631]	<0.01	0.026	-1
Srp54b	ENSMUSG00000079108	protein_coding	signal recognition particle 54B [Source:MGI Symbol;Acc:MGI:3714357]	<0.01	0.045	-1
Tlr1	ENSMUSG00000044827	protein_coding	toll-like receptor 1 [Source:MGI Symbol;Acc:MGI:1341295]	<0.01	<0.01	-1
Ccr1	ENSMUSG00000025804	protein_coding	chemokine (C-C motif) receptor 1 [Source:MGI Symbol;Acc:MGI:104618]	<0.01	0.030	-1
Eif3m	ENSMUSG00000027170	protein_coding	eukaryotic translation initiation factor 3, subunit M [Source:MGI Symbol;Acc:MGI:1351744]	<0.01	0.017	-1
Hc	ENSMUSG00000026874	protein_coding	hemolytic complement [Source:MGI Symbol;Acc:MGI:96031]	<0.01	0.043	-1
Gapdh	ENSMUSG00000057666	protein_coding	glyceraldehyde-3-phosphate dehydrogenase [Source:MGI Symbol;Acc:MGI:95640]	<0.01	0.039	-1
Car8	ENSMUSG00000041261	protein_coding	carbonic anhydrase 8 [Source:MGI Symbol;Acc:MGI:88253]	<0.01	0.013	-1
Ppp1r14c	ENSMUSG00000040653	protein_coding	protein phosphatase 1, regulatory (inhibitor) subunit 14c [Source:MGI Symbol;Acc:MGI:1923392]	<0.01	<0.01	-1
Coa3	ENSMUSG00000017188	protein_coding	cytochrome C oxidase assembly factor 3 [Source:MGI Symbol;Acc:MGI:1098757]	<0.01	0.047	-1
H2-DMa	ENSMUSG00000037649	protein_coding	histocompatibility 2, class II, locus DMa [Source:MGI Symbol;Acc:MGI:95921]	<0.01	0.029	-1
Micall1	ENSMUSG00000033039	protein_coding	microtubule associated monooxygenase, calponin and LIM domain containing -like 1 [Source:MGI Symbol;Acc:MGI:105870]	<0.01	0.018	-1
Tdg	ENSMUSG00000034674	protein_coding	thymine DNA glycosylase [Source:MGI Symbol;Acc:MGI:108247]	<0.01	<0.01	-1
Fgfr2	ENSMUSG00000030849	protein_coding	fibroblast growth factor receptor 2 [Source:MGI Symbol;Acc:MGI:95523]	<0.01	0.023	-1
Mmp9	ENSMUSG00000017737	protein_coding	matrix metallopeptidase 9 [Source:MGI Symbol;Acc:MGI:97011]	<0.01	<0.01	-1
C1qb	ENSMUSG00000036905	protein_coding	complement component 1, q subcomponent, beta polypeptide [Source:MGI Symbol;Acc:MGI:88224]	<0.01	0.032	-1
Parp9	ENSMUSG00000022906	protein_coding	poly (ADP-ribose) polymerase family, member 9 [Source:MGI Symbol;Acc:MGI:1933117]	<0.01	0.018	-1
Ppfia4	ENSMUSG00000026458	protein_coding	protein tyrosine phosphatase, receptor type, f polypeptide (PTPRF), interacting protein (liprin), alpha 4 [Source:MGI Symbol;Acc:MGI:1915757]	<0.01	<0.01	-1
Gm5483	ENSMUSG00000079597	protein_coding	predicted gene 5483 [Source:MGI Symbol;Acc:MGI:3645124]	<0.01	0.046	-1
Slain2	ENSMUSG00000036087	protein_coding	SLAIN motif family, member 2 [Source:MGI Symbol;Acc:MGI:1923241]	<0.01	0.038	-1
Cst8	ENSMUSG00000027442	protein_coding	cystatin 8 (cystatin-related epididymal spermatogenic) [Source:MGI Symbol;Acc:MGI:107161]	<0.01	<0.01	-1
Grhl1	ENSMUSG00000020656	protein_coding	grainyhead-like 1 (Drosophila) [Source:MGI Symbol;Acc:MGI:2182540]	<0.01	0.042	-1
Gm13816	ENSMUSG00000085976	lincRNA	predicted gene 13816 [Source:MGI Symbol;Acc:MGI:3649317]	<0.01	0.021	-1
Adcy5	ENSMUSG00000022840	protein_coding	adenylate cyclase 5 [Source:MGI Symbol;Acc:MGI:99673]	<0.01	<0.01	-1
Cd96	ENSMUSG00000022657	protein_coding	CD96 antigen [Source:MGI Symbol;Acc:MGI:1934368]	<0.01	0.031	-1
Gm26782	ENSMUSG00000097431	lincRNA	predicted gene, 26782 [Source:MGI Symbol;Acc:MGI:5477276]	<0.01	<0.01	-1

Pcdhga10	ENSMUSG00000102222	protein_coding	protocadherin gamma subfamily A, 10 [Source:MGI Symbol;Acc:MGI:1935227]	<0.01	0.015	-1
Fgr	ENSMUSG00000028874	protein_coding	FGR proto-oncogene, Src family tyrosine kinase [Source:MGI Symbol;Acc:MGI:95527]	<0.01	0.030	-1
Sgcd	ENSMUSG00000020354	protein_coding	sarcoglycan, delta (dystrophin-associated glycoprotein) [Source:MGI Symbol;Acc:MGI:1346525]	<0.01	0.032	-1
Hspa4l	ENSMUSG00000025757	protein_coding	heat shock protein 4 like [Source:MGI Symbol;Acc:MGI:107422]	<0.01	<0.01	-1
Ccl17	ENSMUSG00000031780	protein_coding	chemokine (C-C motif) ligand 17 [Source:MGI Symbol;Acc:MGI:1329039]	<0.01	0.013	-1
Cd24a	ENSMUSG00000047139	protein_coding	CD24a antigen [Source:MGI Symbol;Acc:MGI:88323]	<0.01	0.039	-1
Gm9625	ENSMUSG00000097906	processed_pseudogene	predicted gene 9625 [Source:MGI Symbol;Acc:MGI:3780033]	<0.01	0.020	-1
Npc2	ENSMUSG00000021242	protein_coding	Niemann-Pick type C2 [Source:MGI Symbol;Acc:MGI:1915213]	<0.01	0.026	-1
Rpl13a-ps1	ENSMUSG00000062083	protein_coding	ribosomal protein 13A, pseudogene 1 [Source:MGI Symbol;Acc:MGI:3648883]	<0.01	<0.01	-1
Iah1	ENSMUSG00000062054	protein_coding	isoamyl acetate-hydrolyzing esterase 1 homolog [Source:MGI Symbol;Acc:MGI:1914982]	<0.01	<0.01	-1
Napsa	ENSMUSG00000002204	protein_coding	napsin A aspartic peptidase [Source:MGI Symbol;Acc:MGI:109365]	<0.01	0.029	-1
Adgrg3	ENSMUSG00000060470	protein_coding	adhesion G protein-coupled receptor G3 [Source:MGI Symbol;Acc:MGI:1859670]	<0.01	0.029	-1
Gaa	ENSMUSG00000025579	protein_coding	glucosidase, alpha, acid [Source:MGI Symbol;Acc:MGI:95609]	<0.01	0.024	-1
3110082I17Rik	ENSMUSG00000053553	protein_coding	RIKEN cDNA 3110082I17 gene [Source:MGI Symbol;Acc:MGI:1920462]	<0.01	0.036	-1
Nxpe2	ENSMUSG00000032028	protein_coding	neurexophilin and PC-esterase domain family, member 2 [Source:MGI Symbol;Acc:MGI:1925502]	<0.01	<0.01	-1
Eln	ENSMUSG00000029675	protein_coding	elastin [Source:MGI Symbol;Acc:MGI:95317]	<0.01	<0.01	-1
Tmem128	ENSMUSG00000067365	protein_coding	transmembrane protein 128 [Source:MGI Symbol;Acc:MGI:1913559]	<0.01	0.019	-1
Trim30a	ENSMUSG00000030921	protein_coding	tripartite motif-containing 30A [Source:MGI Symbol;Acc:MGI:98178]	<0.01	0.013	-1
Slco4c1	ENSMUSG00000040693	protein_coding	solute carrier organic anion transporter family, member 4C1 [Source:MGI Symbol;Acc:MGI:2442784]	<0.01	0.014	-1
Rpl21-ps8	ENSMUSG00000061684	processed_pseudogene	ribosomal protein L21, pseudogene 8 [Source:MGI Symbol;Acc:MGI:3648345]	<0.01	<0.01	-1
Alkbh7	ENSMUSG00000002661	protein_coding	alkB homolog 7 [Source:MGI Symbol;Acc:MGI:1913650]	<0.01	0.028	-1
Tgtp2	ENSMUSG00000078921	protein_coding	T cell specific GTPase 2 [Source:MGI Symbol;Acc:MGI:3710083]	<0.01	0.016	-1
Tc2n	ENSMUSG00000021187	protein_coding	tandem C2 domains, nuclear [Source:MGI Symbol;Acc:MGI:1921663]	<0.01	<0.01	-1
H2-Ob	ENSMUSG00000041538	protein_coding	histocompatibility 2, O region beta locus [Source:MGI Symbol;Acc:MGI:95925]	<0.01	0.018	-1
Maf1	ENSMUSG00000022553	protein_coding	MAF1 homolog, negative regulator of RNA polymerase III [Source:MGI Symbol;Acc:MGI:1916127]	<0.01	0.049	-1
Igkc	ENSMUSG00000076609	IG_C_gene	immunoglobulin kappa constant [Source:MGI Symbol;Acc:MGI:96495]	<0.01	<0.01	-1
Hdc	ENSMUSG00000027360	protein_coding	histidine decarboxylase [Source:MGI Symbol;Acc:MGI:96062]	<0.01	<0.01	-1
Tcea3	ENSMUSG00000001604	protein_coding	transcription elongation factor A (SII), 3 [Source:MGI Symbol;Acc:MGI:1196908]	<0.01	0.040	-1
C1s2	ENSMUSG00000107554	NA	NA	<0.01	<0.01	-1
Slc5a12	ENSMUSG00000041644	protein_coding	solute carrier family 5 (sodium/glucose cotransporter), member 12 [Source:MGI Symbol;Acc:MGI:2138890]	<0.01	<0.01	-1
Pira2	ENSMUSG00000089942	protein_coding	paired-Ig-like receptor A2 [Source:MGI Symbol;Acc:MGI:1195970]	<0.01	0.033	-1
Klre1	ENSMUSG00000050241	protein_coding	killer cell lectin-like receptor family E member 1 [Source:MGI Symbol;Acc:MGI:2662547]	<0.01	0.018	-1
Spata5l1	ENSMUSG00000074876	processed_transcript	spermatogenesis associated 5-like 1 [Source:MGI Symbol;Acc:MGI:3036261]	<0.01	0.032	-1
Tmem180	ENSMUSG00000025227	protein_coding	major facilitator superfamily domain containing 13a [Source:MGI Symbol;Acc:MGI:1922396]	<0.01	0.049	-1
Igkv1-135	ENSMUSG00000096336	IG_V_gene	immunoglobulin kappa variable 1-135 [Source:MGI Symbol;Acc:MGI:3819952]	<0.01	<0.01	-1
Atp8a1	ENSMUSG00000037685	protein_coding	ATPase, aminophospholipid transporter (APLT), class I, type 8A, member 1 [Source:MGI Symbol;Acc:MGI:1330848]	<0.01	0.014	-1
Tbc1d9	ENSMUSG00000031709	protein_coding	TBC1 domain family, member 9 [Source:MGI Symbol;Acc:MGI:1918560]	<0.01	<0.01	-1
Azin1	ENSMUSG00000037458	protein_coding	antizyme inhibitor 1 [Source:MGI Symbol;Acc:MGI:1859169]	<0.01	0.049	-1
Unc45b	ENSMUSG00000018845	protein_coding	unc-45 myosin chaperone B [Source:MGI Symbol;Acc:MGI:2443377]	<0.01	0.039	-1
Hyi	ENSMUSG00000006395	polymorphic_pseudogene	hydroxypyruvate isomerase (putative) [Source:MGI Symbol;Acc:MGI:1915430]	<0.01	0.049	-1
Lrrc25	ENSMUSG00000049988	protein_coding	leucine rich repeat containing 25 [Source:MGI Symbol;Acc:MGI:2445284]	<0.01	<0.01	-1
Trim5	ENSMUSG00000060441	protein_coding	tripartite motif-containing 5 [Source:MGI Symbol;Acc:MGI:3646853]	<0.01	<0.01	-1
Igkv3-12	ENSMUSG00000094117	IG_V_gene	immunoglobulin kappa variable 3-12 [Source:MGI Symbol;Acc:MGI:1330815]	<0.01	<0.01	-1
Cd300lg	ENSMUSG00000017309	protein_coding	CD300 molecule like family member G [Source:MGI Symbol;Acc:MGI:1289168]	<0.01	<0.01	-1
Ighv1-9	ENSMUSG00000094694	IG_V_gene	immunoglobulin heavy variable V1-9 [Source:MGI Symbol;Acc:MGI:4439621]	<0.01	<0.01	-1
C4b	ENSMUSG00000073418	protein_coding	complement component 4B (Chido blood group) [Source:MGI Symbol;Acc:MGI:88228]	<0.01	<0.01	-1
Epn3	ENSMUSG00000010080	protein_coding	epsin 3 [Source:MGI Symbol;Acc:MGI:1919139]	<0.01	0.016	-1
Sowahc	ENSMUSG00000098188	protein_coding	sosondowah ankyrin repeat domain family member C [Source:MGI Symbol;Acc:MGI:3606051]	<0.01	0.018	-1
Serpind1	ENSMUSG00000022766	protein_coding	serine (or cysteine) peptidase inhibitor, clade D, member 1 [Source:MGI Symbol;Acc:MGI:96051]	<0.01	0.048	-1
Slc15a2	ENSMUSG00000022899	protein_coding	solute carrier family 15 (H+/peptide transporter), member 2 [Source:MGI Symbol;Acc:MGI:1890457]	<0.01	<0.01	-1
Gvin1	ENSMUSG00000045868	protein_coding	GTPase, very large interferon inducible 1 [Source:MGI Symbol;Acc:MGI:1921808]	<0.01	0.037	-1
Pydc4	ENSMUSG00000073491	protein_coding	interferon activated gene 213 [Source:MGI Symbol;Acc:MGI:3695276]	<0.01	<0.01	-1
Gm9493	ENSMUSG00000044424	protein_coding	predicted gene 9493 [Source:MGI Symbol;Acc:MGI:3779903]	<0.01	<0.01	-1
Ighv1-64	ENSMUSG00000094088	IG_V_gene	immunoglobulin heavy variable 1-64 [Source:MGI Symbol;Acc:MGI:4439789]	<0.01	0.034	-1
Sqle	ENSMUSG00000022351	protein_coding	squalene epoxidase [Source:MGI Symbol;Acc:MGI:109296]	<0.01	0.048	-1
H1f0	ENSMUSG00000096210	protein_coding	H1 histone family, member 0 [Source:MGI Symbol;Acc:MGI:95893]	<0.01	0.025	-1
Gm43474	ENSMUSG00000105362	TEC	predicted gene 43474 [Source:MGI Symbol;Acc:MGI:5663611]	<0.01	0.019	-1
Cygb	ENSMUSG00000020810	protein_coding	cytoglobin [Source:MGI Symbol;Acc:MGI:2149481]	<0.01	<0.01	-1
Tmprss13	ENSMUSG00000037129	protein_coding	transmembrane protease, serine 13 [Source:MGI Symbol;Acc:MGI:2682935]	<0.01	0.026	-1
Etv5	ENSMUSG00000013089	protein_coding	ets variant 5 [Source:MGI Symbol;Acc:MGI:1096867]	<0.01	0.029	-1
Egfem1	ENSMUSG00000063600	protein_coding	EGF-like and EMI domain containing 1 [Source:MGI Symbol;Acc:MGI:1922990]	<0.01	<0.01	-1
Nadk	ENSMUSG00000029063	protein_coding	NAD kinase [Source:MGI Symbol;Acc:MGI:2183149]	<0.01	0.037	-1
Ighv1-15	ENSMUSG00000103254	IG_V_gene	immunoglobulin heavy variable 1-15 [Source:MGI Symbol;Acc:MGI:4439782]	<0.01	<0.01	-1
Slc34a2	ENSMUSG00000029188	protein_coding	solute carrier family 34 (sodium phosphate), member 2 [Source:MGI Symbol;Acc:MGI:1342284]	<0.01	0.030	-1
Rab27b	ENSMUSG00000024511	protein_coding	RAB27B, member RAS oncogene family [Source:MGI Symbol;Acc:MGI:1931295]	<0.01	0.023	-1
Gcdh	ENSMUSG00000003809	protein_coding	glutaryl-Coenzyme A dehydrogenase [Source:MGI Symbol;Acc:MGI:104541]	<0.01	0.015	-1
Srp54a	ENSMUSG00000073079	protein_coding	signal recognition particle 54A [Source:MGI Symbol;Acc:MGI:1346087]	<0.01	0.014	-1
Ifitm6	ENSMUSG00000059108	protein_coding	interferon induced transmembrane protein 6 [Source:MGI Symbol;Acc:MGI:2686976]	<0.01	0.021	-1
RP23-402A4.1	ENSMUSG00000108393	lincRNA	predicted gene, 32633 [Source:MGI Symbol;Acc:MGI:5591792]	<0.01	<0.01	-1
Cox7a2l	ENSMUSG00000024248	protein_coding	cytochrome c oxidase subunit VIIa polypeptide 2-like [Source:MGI Symbol;Acc:MGI:106015]	<0.01	<0.01	-1
Fam49b	ENSMUSG00000022378	protein_coding	family with sequence similarity 49, member B [Source:MGI Symbol;Acc:MGI:1923520]	<0.01	0.014	-1
Igkv16-104	ENSMUSG00000076522	IG_V_gene	immunoglobulin kappa variable 16-104 [Source:MGI Symbol;Acc:MGI:2685913]	<0.01	<0.01	-1
Cox7c	ENSMUSG00000017778	protein_coding	cytochrome c oxidase subunit VIIc [Source:MGI Symbol;Acc:MGI:103226]	<0.01	0.031	-1
Folr1	ENSMUSG00000001827	protein_coding	folate receptor 1 (adult) [Source:MGI Symbol;Acc:MGI:95568]	<0.01	<0.01	-1
Ighv1-78	ENSMUSG00000096326	IG_V_gene	immunoglobulin heavy variable 1-78 [Source:MGI Symbol;Acc:MGI:4439736]	<0.01	<0.01	-1
Tspan11	ENSMUSG00000030351	protein_coding	tetraspanin 11 [Source:MGI Symbol;Acc:MGI:1915748]	<0.01	0.015	-1
Atp7b	ENSMUSG00000006567	protein_coding	ATPase, Cu++ transporting, beta polypeptide [Source:MGI Symbol;Acc:MGI:103297]	<0.01	0.045	-1
Unc5a	ENSMUSG00000025876	protein_coding	unc-5 netrin receptor A [Source:MGI Symbol;Acc:MGI:894682]	<0.01	<0.01	-1
Igkv17-121	ENSMUSG00000076514	IG_V_gene	immunoglobulin kappa variable 17-121 [Source:MGI Symbol;Acc:MGI:3647671]	<0.01	<0.01	-1
Rps4l	ENSMUSG00000063171	transcribed_processed_pseudogene	ribosomal protein S4-like [Source:MGI Symbol;Acc:MGI:1913434]	<0.01	0.031	-1

Wars	ENSMUSG00000021266	protein_coding	tryptophanyl-tRNA synthetase [Source:MGI Symbol;Acc:MGI:104630]	<0.01	0.042	-1
Dnah11	ENSMUSG00000018581	protein_coding	dynein, axonemal, heavy chain 11 [Source:MGI Symbol;Acc:MGI:1100864]	<0.01	0.014	-1
Lsm4	ENSMUSG00000031848	protein_coding	LSM4 homolog, U6 small nuclear RNA and mRNA degradation associated [Source:MGI Symbol;Acc:MGI:1354692]	<0.01	<0.01	-1
Ccr7	ENSMUSG00000037944	protein_coding	chemokine (C-C motif) receptor 7 [Source:MGI Symbol;Acc:MGI:103011]	<0.01	0.034	-1
Prr16	ENSMUSG00000073565	protein_coding	proline rich 16 [Source:MGI Symbol;Acc:MGI:1918623]	<0.01	0.039	-1
Sbk1	ENSMUSG00000042978	protein_coding	SH3-binding kinase 1 [Source:MGI Symbol;Acc:MGI:2135937]	<0.01	0.018	-1
Cited2	ENSMUSG00000039910	protein_coding	Cbp/p300-interacting transactivator, with Glu/Asp-rich carboxy-terminal domain, 2 [Source:MGI Symbol;Acc:MGI:1306784]	<0.01	0.034	-1
Igkv10-94	ENSMUSG00000096490	IG_V_gene	immunoglobulin kappa variable 10-94 [Source:MGI Symbol;Acc:MGI:3646140]	<0.01	0.012	-1
Igha	ENSMUSG00000095079	IG_C_gene	immunoglobulin heavy constant alpha [Source:MGI Symbol;Acc:MGI:96444]	<0.01	<0.01	-1
Igkv1-110	ENSMUSG00000093861	IG_V_gene	immunoglobulin kappa variable 1-110 [Source:MGI Symbol;Acc:MGI:4439558]	<0.01	0.026	-1
Gstm6	ENSMUSG00000068762	protein_coding	glutathione S-transferase, mu 6 [Source:MGI Symbol;Acc:MGI:1309467]	<0.01	0.020	-1
Sftpb	ENSMUSG00000056370	protein_coding	surfactant associated protein B [Source:MGI Symbol;Acc:MGI:109516]	<0.01	0.025	-1
Igkv4-57	ENSMUSG00000076556	IG_V_gene	immunoglobulin kappa variable 4-57 [Source:MGI Symbol;Acc:MGI:2685035]	<0.01	<0.01	-1
B4galnt1	ENSMUSG00000006731	protein_coding	beta-1,4-N-acetyl-galactosaminyl transferase 1 [Source:MGI Symbol;Acc:MGI:1342057]	<0.01	0.040	-1
Gm20402	ENSMUSG00000092405	antisense_RNA	predicted gene 20402 [Source:MGI Symbol;Acc:MGI:5141867]	<0.01	0.023	-1
Ifi47	ENSMUSG00000078920	protein_coding	interferon gamma inducible protein 47 [Source:MGI Symbol;Acc:MGI:99448]	<0.01	0.044	-1
Ppp1r3e	ENSMUSG00000072494	protein_coding	protein phosphatase 1, regulatory (inhibitor) subunit 3E [Source:MGI Symbol;Acc:MGI:2145790]	<0.01	<0.01	-1
Ctsh	ENSMUSG00000032359	protein_coding	cathepsin H [Source:MGI Symbol;Acc:MGI:107285]	<0.01	0.017	-1
Cdv3	ENSMUSG00000032803	protein_coding	carnitine deficiency-associated gene expressed in ventricle 3 [Source:MGI Symbol;Acc:MGI:2448759]	<0.01	0.034	-1
Nfe2	ENSMUSG00000058794	protein_coding	nuclear factor, erythroid derived 2 [Source:MGI Symbol;Acc:MGI:97308]	<0.01	0.028	-1
Ppbp	ENSMUSG00000029372	protein_coding	pro-platelet basic protein [Source:MGI Symbol;Acc:MGI:1888712]	<0.01	0.026	-1
Ankrd63	ENSMUSG00000078137	protein_coding	ankyrin repeat domain 63 [Source:MGI Symbol;Acc:MGI:2686183]	<0.01	<0.01	-1
Cmtm8	ENSMUSG00000041012	protein_coding	CKLF-like MARVEL transmembrane domain containing 8 [Source:MGI Symbol;Acc:MGI:2447167]	<0.01	0.038	-1
6030419C18Rik	ENSMUSG00000066607	protein_coding	RIKEN cDNA 6030419C18 gene [Source:MGI Symbol;Acc:MGI:2442108]	<0.01	0.028	-1
Ighv1-66	ENSMUSG00000095519	IG_V_gene	immunoglobulin heavy variable 1-66 [Source:MGI Symbol;Acc:MGI:4439825]	<0.01	0.017	-1
Ly6i	ENSMUSG00000022586	protein_coding	lymphocyte antigen 6 complex, locus I [Source:MGI Symbol;Acc:MGI:1888480]	<0.01	<0.01	-1
Gm10645	ENSMUSG00000074228	protein_coding	predicted gene 10645 [Source:MGI Symbol;Acc:MGI:3704313]	<0.01	<0.01	-1
Gm830	ENSMUSG00000084939	processed_transcript	predicted gene 830 [Source:MGI Symbol;Acc:MGI:2685676]	<0.01	<0.01	-1
Ighv8-12	ENSMUSG00000076731	IG_V_gene	immunoglobulin heavy variable V8-12 [Source:MGI Symbol;Acc:MGI:3642873]	<0.01	<0.01	-1
C920006O11Rik	ENSMUSG00000097574	lincRNA	RIKEN cDNA C920006O11 gene [Source:MGI Symbol;Acc:MGI:2443759]	<0.01	0.012	-1
Arhgap20	ENSMUSG00000053199	protein_coding	Rho GTPase activating protein 20 [Source:MGI Symbol;Acc:MGI:2445175]	<0.01	0.016	-1
Npas2	ENSMUSG00000026077	protein_coding	neuronal PAS domain protein 2 [Source:MGI Symbol;Acc:MGI:109232]	<0.01	<0.01	-1
Rps26	ENSMUSG00000025362	protein_coding	ribosomal protein S26 [Source:MGI Symbol;Acc:MGI:1351628]	<0.01	<0.01	-1
Gm1966	ENSMUSG00000073902	unprocessed_pseudogene	predicted gene 1966 [Source:MGI Symbol;Acc:MGI:3584360]	<0.01	<0.01	-1
B430010I23Rik	ENSMUSG00000084960	antisense_RNA	RIKEN cDNA B430010I23 gene [Source:MGI Symbol;Acc:MGI:1926099]	<0.01	<0.01	-1
Igkv1-117	ENSMUSG00000094335	IG_V_gene	immunoglobulin kappa variable 1-117 [Source:MGI Symbol;Acc:MGI:4439721]	<0.01	<0.01	-1
Sprr1a	ENSMUSG00000050359	protein_coding	small proline-rich protein 1A [Source:MGI Symbol;Acc:MGI:106660]	<0.01	<0.01	-1
Plcb2	ENSMUSG00000040061	protein_coding	phospholipase C, beta 2 [Source:MGI Symbol;Acc:MGI:107465]	<0.01	0.045	-1
Cux2	ENSMUSG00000042589	protein_coding	cut-like homeobox 2 [Source:MGI Symbol;Acc:MGI:107321]	<0.01	0.041	-1
Capza1	ENSMUSG00000070372	protein_coding	capping protein (actin filament) muscle Z-line, alpha 1 [Source:MGI Symbol;Acc:MGI:106227]	<0.01	<0.01	-1
Cd300a	ENSMUSG00000034652	protein_coding	CD300A molecule [Source:MGI Symbol;Acc:MGI:2443411]	<0.01	<0.01	-1
Slc25a47	ENSMUSG00000048856	protein_coding	solute carrier family 25, member 47 [Source:MGI Symbol;Acc:MGI:2144766]	<0.01	0.013	-1
Pycard	ENSMUSG00000030793	protein_coding	PYD and CARD domain containing [Source:MGI Symbol;Acc:MGI:1931465]	<0.01	0.012	-1
H2-DMb1	ENSMUSG00000079547	protein_coding	histocompatibility 2, class II, locus Mb1 [Source:MGI Symbol;Acc:MGI:95922]	<0.01	0.015	-1
Rnaseh2c	ENSMUSG00000024925	protein_coding	ribonuclease H2, subunit C [Source:MGI Symbol;Acc:MGI:1915459]	<0.01	0.017	-1
Epsti1	ENSMUSG00000022014	protein_coding	epithelial stromal interaction 1 (breast) [Source:MGI Symbol;Acc:MGI:1915168]	<0.01	0.026	-1
Gm26510	ENSMUSG00000097154	lincRNA	predicted gene, 26510 [Source:MGI Symbol;Acc:MGI:5477004]	<0.01	0.040	-1
Gm27021	ENSMUSG00000097919	protein_coding	predicted gene, 27021 [Source:MGI Symbol;Acc:MGI:5504136]	<0.01	<0.01	-1
Nr2f6	ENSMUSG00000002393	protein_coding	nuclear receptor subfamily 2, group F, member 6 [Source:MGI Symbol;Acc:MGI:1352453]	<0.01	0.033	-1
Mmp13	ENSMUSG00000050578	protein_coding	matrix metallopeptidase 13 [Source:MGI Symbol;Acc:MGI:1340026]	<0.01	<0.01	-1
Rpl26	ENSMUSG00000060938	protein_coding	ribosomal protein L26 [Source:MGI Symbol;Acc:MGI:106022]	<0.01	<0.01	-1
Rps15a	ENSMUSG00000008683	protein_coding	ribosomal protein S15A [Source:MGI Symbol;Acc:MGI:2389091]	<0.01	<0.01	-1
Tinf2	ENSMUSG00000007589	protein_coding	Terf1 (TRF1)-interacting nuclear factor 2 [Source:MGI Symbol;Acc:MGI:107246]	<0.01	0.049	-1
Inhbb	ENSMUSG00000037035	protein_coding	inhibin beta-B [Source:MGI Symbol;Acc:MGI:96571]	<0.01	0.034	-1
Fibin	ENSMUSG00000074971	protein_coding	fin bud initiation factor homolog (zebrafish) [Source:MGI Symbol;Acc:MGI:1914856]	<0.01	0.040	-1
Ldha	ENSMUSG00000063229	protein_coding	lactate dehydrogenase A [Source:MGI Symbol;Acc:MGI:96759]	<0.01	0.034	-1
Dhx40	ENSMUSG00000018425	protein_coding	DEAH (Asp-Glu-Ala-His) box polypeptide 40 [Source:MGI Symbol;Acc:MGI:1914737]	<0.01	0.038	-1
Apobr	ENSMUSG00000042759	protein_coding	apolipoprotein B receptor [Source:MGI Symbol;Acc:MGI:2176230]	<0.01	<0.01	-1
Ccdc71l	ENSMUSG00000090946	protein_coding	coiled-coil domain containing 71 like [Source:MGI Symbol;Acc:MGI:1919373]	<0.01	0.050	-1
Myd88	ENSMUSG00000032508	protein_coding	myeloid differentiation primary response gene 88 [Source:MGI Symbol;Acc:MGI:108005]	<0.01	0.028	-1
Bcl2a1b	ENSMUSG00000089929	protein_coding	B cell leukemia/lymphoma 2 related protein A1b [Source:MGI Symbol;Acc:MGI:1278326]	<0.01	<0.01	-1
Ighv1-80	ENSMUSG00000094075	IG_V_gene	immunoglobulin heavy variable 1-80 [Source:MGI Symbol;Acc:MGI:4439738]	<0.01	<0.01	-1
Gm16174	ENSMUSG00000087593	antisense_RNA	predicted gene 16174 [Source:MGI Symbol;Acc:MGI:3801857]	<0.01	0.014	-1
Il1f9	ENSMUSG00000044103	protein_coding	interleukin 1 family, member 9 [Source:MGI Symbol;Acc:MGI:2449929]	<0.01	0.013	-1
Rab20	ENSMUSG00000031504	protein_coding	RAB20, member RAS oncogene family [Source:MGI Symbol;Acc:MGI:102789]	<0.01	0.045	-1
Trp53inp1	ENSMUSG00000028211	protein_coding	transformation related protein 53 inducible nuclear protein 1 [Source:MGI Symbol;Acc:MGI:1926609]	<0.01	0.015	-1
Tnfsf13	ENSMUSG00000089669	protein_coding	tumor necrosis factor (ligand) superfamily, member 13 [Source:MGI Symbol;Acc:MGI:1916833]	<0.01	<0.01	-1
Tmcc1	ENSMUSG00000030126	protein_coding	transmembrane and coiled coil domains 1 [Source:MGI Symbol;Acc:MGI:2442368]	<0.01	0.050	-1
Ogfr	ENSMUSG00000049401	protein_coding	opioid growth factor receptor [Source:MGI Symbol;Acc:MGI:1919325]	<0.01	<0.01	-1
Cradd	ENSMUSG00000045867	protein_coding	CASP2 and RIPK1 domain containing adaptor with death domain [Source:MGI Symbol;Acc:MGI:1336168]	<0.01	<0.01	-1
Sphk1	ENSMUSG00000061878	protein_coding	sphingosine kinase 1 [Source:MGI Symbol;Acc:MGI:1316649]	<0.01	<0.01	-1
Gm6204	ENSMUSG00000105879	processed_pseudogene	predicted gene 6204 [Source:MGI Symbol;Acc:MGI:3649038]	<0.01	0.040	-1
Zfp874a	ENSMUSG00000069206	protein_coding	zinc finger protein 874a [Source:MGI Symbol;Acc:MGI:3040703]	<0.01	<0.01	-1
Plbd1	ENSMUSG00000030214	protein_coding	phospholipase B domain containing 1 [Source:MGI Symbol;Acc:MGI:1914107]	<0.01	0.020	-1
Rap1gap	ENSMUSG00000041351	protein_coding	Rap1 GTPase-activating protein [Source:MGI Symbol;Acc:MGI:109338]	<0.01	0.012	-1
Ighv1-58	ENSMUSG00000095889	IG_V_gene	immunoglobulin heavy variable 1-58 [Source:MGI Symbol;Acc:MGI:4439557]	<0.01	0.010	-1
H2-Q7	ENSMUSG00000060550	protein_coding	histocompatibility 2, Q region locus 7 [Source:MGI Symbol;Acc:MGI:95936]	<0.01	<0.01	-1
Oat	ENSMUSG00000030934	protein_coding	ornithine aminotransferase [Source:MGI Symbol;Acc:MGI:97394]	<0.01	0.029	-1
Pgc	ENSMUSG00000023987	protein_coding	progastricsin (pepsinogen C) [Source:MGI Symbol;Acc:MGI:98909]	<0.01	<0.01	-1
Rpl10a-ps1	ENSMUSG00000084416	processed_pseudogene	ribosomal protein L10A, pseudogene 1 [Source:MGI Symbol;Acc:MGI:3705885]	<0.01	0.036	-1

Rps6ka1	ENSMUSG00000003644	protein_coding	ribosomal protein S6 kinase polypeptide 1 [Source:MGI Symbol;Acc:MGI:104558]	<0.01	0.013	-1
Ighv1-18	ENSMUSG00000076695	IG_V_gene	immunoglobulin heavy variable V1-18 [Source:MGI Symbol;Acc:MGI:4439780]	<0.01	<0.01	-1
Lyz2	ENSMUSG00000069516	protein_coding	lysozyme 2 [Source:MGI Symbol;Acc:MGI:96897]	<0.01	0.021	-1
Ang	ENSMUSG00000072115	protein_coding	angiogenin, ribonuclease, RNase A family, 5 [Source:MGI Symbol;Acc:MGI:88022]	<0.01	<0.01	-1
Best1	ENSMUSG00000037418	protein_coding	bestrophin 1 [Source:MGI Symbol;Acc:MGI:1346332]	<0.01	<0.01	-1
Eml2	ENSMUSG00000040811	protein_coding	echinoderm microtubule associated protein like 2 [Source:MGI Symbol;Acc:MGI:1919455]	<0.01	0.045	-1
Tap1	ENSMUSG00000037321	protein_coding	transporter 1, ATP-binding cassette, sub-family B (MDR/TAP) [Source:MGI Symbol;Acc:MGI:98483]	<0.01	0.029	-1
Dram1	ENSMUSG00000020057	protein_coding	DNA-damage regulated autophagy modulator 1 [Source:MGI Symbol;Acc:MGI:1918962]	<0.01	<0.01	-1
Pomt1	ENSMUSG00000039254	protein_coding	protein-O-mannosyltransferase 1 [Source:MGI Symbol;Acc:MGI:2138994]	<0.01	0.026	-1
Bank1	ENSMUSG00000037922	protein_coding	B cell scaffold protein with ankyrin repeats 1 [Source:MGI Symbol;Acc:MGI:2442120]	<0.01	0.038	-1
Ly6d	ENSMUSG00000034634	protein_coding	lymphocyte antigen 6 complex, locus D [Source:MGI Symbol;Acc:MGI:96881]	<0.01	<0.01	-1
Slc26a4	ENSMUSG00000020651	protein_coding	solute carrier family 26, member 4 [Source:MGI Symbol;Acc:MGI:1346029]	<0.01	<0.01	-1
Cd8b1	ENSMUSG00000053044	protein_coding	CD8 antigen, beta chain 1 [Source:MGI Symbol;Acc:MGI:88347]	<0.01	<0.01	-1
Nptxr	ENSMUSG00000022421	protein_coding	neuronal pentraxin receptor [Source:MGI Symbol;Acc:MGI:1920590]	<0.01	0.020	-1
Tfcp2l1	ENSMUSG00000026380	protein_coding	transcription factor CP2-like 1 [Source:MGI Symbol;Acc:MGI:2444691]	<0.01	0.014	-1
Cbx7	ENSMUSG00000053411	protein_coding	chromobox 7 [Source:MGI Symbol;Acc:MGI:1196439]	<0.01	<0.01	-1
Gm12942	ENSMUSG00000070737	protein_coding	transmembrane protein 35B [Source:MGI Symbol;Acc:MGI:3758095]	<0.01	0.020	-1
Cdcp1	ENSMUSG00000035498	protein_coding	CUB domain containing protein 1 [Source:MGI Symbol;Acc:MGI:2442010]	<0.01	0.041	-1
Gm8121	ENSMUSG00000051116	processed_pseudogene	predicted pseudogene 8121 [Source:MGI Symbol;Acc:MGI:3645325]	<0.01	0.040	-1
Pvrl2	ENSMUSG00000062300	protein_coding	nectin cell adhesion molecule 2 [Source:MGI Symbol;Acc:MGI:97822]	<0.01	0.011	-1
Trim30b	ENSMUSG00000052749	protein_coding	tripartite motif-containing 30B [Source:MGI Symbol;Acc:MGI:4821256]	<0.01	<0.01	-1
Gfer	ENSMUSG00000040888	protein_coding	growth factor, erv1 (S. cerevisiae)-like (augmenter of liver regeneration) [Source:MGI Symbol;Acc:MGI:107757]	<0.01	0.046	-1
Fam178b	ENSMUSG00000046337	protein_coding	family with sequence similarity 178, member B [Source:MGI Symbol;Acc:MGI:3026913]	<0.01	0.016	-1
Akr1e1	ENSMUSG00000045410	protein_coding	aldo-keto reductase family 1, member E1 [Source:MGI Symbol;Acc:MGI:1914758]	<0.01	<0.01	-1
Slc26a9	ENSMUSG00000042268	protein_coding	solute carrier family 26, member 9 [Source:MGI Symbol;Acc:MGI:2444594]	<0.01	<0.01	-1
Med8	ENSMUSG00000006392	protein_coding	mediator complex subunit 8 [Source:MGI Symbol;Acc:MGI:1915269]	<0.01	0.036	-1
Mapkapk3	ENSMUSG00000032577	protein_coding	mitogen-activated protein kinase-activated protein kinase 3 [Source:MGI Symbol;Acc:MGI:2143163]	<0.01	0.026	-1
Casc4	ENSMUSG00000060227	protein_coding	cancer susceptibility candidate 4 [Source:MGI Symbol;Acc:MGI:2443129]	<0.01	<0.01	-1
Klra7	ENSMUSG00000067599	protein_coding	killer cell lectin-like receptor, subfamily A, member 7 [Source:MGI Symbol;Acc:MGI:101901]	<0.01	0.037	-1
Klra2	ENSMUSG00000030187	protein_coding	killer cell lectin-like receptor, subfamily A, member 2 [Source:MGI Symbol;Acc:MGI:101906]	<0.01	<0.01	-1
Mmgt2	ENSMUSG00000048497	protein_coding	membrane magnesium transporter 2 [Source:MGI Symbol;Acc:MGI:2448491]	<0.01	0.029	-1
Cd300ld	ENSMUSG00000034641	protein_coding	CD300 molecule like family member d [Source:MGI Symbol;Acc:MGI:2442358]	<0.01	0.015	-1
Rap1gap2	ENSMUSG00000038807	protein_coding	RAP1 GTPase activating protein 2 [Source:MGI Symbol;Acc:MGI:3028623]	<0.01	0.015	-1
Trpv4	ENSMUSG00000014158	protein_coding	transient receptor potential cation channel, subfamily V, member 4 [Source:MGI Symbol;Acc:MGI:1926945]	<0.01	0.021	-1
Iglc1	ENSMUSG00000105906	IG_C_gene	immunoglobulin lambda constant 1 [Source:MGI Symbol;Acc:MGI:99546]	<0.01	0.011	-1
S100a9	ENSMUSG00000056071	protein_coding	S100 calcium binding protein A9 (calgranulin B) [Source:MGI Symbol;Acc:MGI:1338947]	<0.01	0.017	-1
Fbxo44	ENSMUSG00000029001	protein_coding	F-box protein 44 [Source:MGI Symbol;Acc:MGI:1354744]	<0.01	<0.01	-1
Igkv10-96	ENSMUSG00000094420	IG_V_gene	immunoglobulin kappa variable 10-96 [Source:MGI Symbol;Acc:MGI:4439561]	<0.01	<0.01	-1
Ccr2	ENSMUSG00000049103	protein_coding	chemokine (C-C motif) receptor 2 [Source:MGI Symbol;Acc:MGI:106185]	<0.01	0.028	-1
Nsmce1	ENSMUSG00000030750	protein_coding	NSE1 homolog, SMC5-SMC6 complex component [Source:MGI Symbol;Acc:MGI:1914961]	<0.01	0.027	-1
Ube2j2	ENSMUSG00000023286	protein_coding	ubiquitin-conjugating enzyme E2J 2 [Source:MGI Symbol;Acc:MGI:2153608]	<0.01	0.027	-1
Phospho1	ENSMUSG00000050860	protein_coding	phosphatase, orphan 1 [Source:MGI Symbol;Acc:MGI:2447348]	<0.01	0.042	-1
Smad3	ENSMUSG00000032402	protein_coding	SMAD family member 3 [Source:MGI Symbol;Acc:MGI:1201674]	<0.01	0.013	-1
Mlph	ENSMUSG00000026303	protein_coding	melanophilin [Source:MGI Symbol;Acc:MGI:2176380]	<0.01	0.034	-1
Cxcl14	ENSMUSG00000021508	protein_coding	chemokine (C-X-C motif) ligand 14 [Source:MGI Symbol;Acc:MGI:1888514]	<0.01	<0.01	-1
AA467197	ENSMUSG00000033213	protein_coding	expressed sequence AA467197 [Source:MGI Symbol;Acc:MGI:3034182]	<0.01	0.021	-1
Nnmt	ENSMUSG00000032271	protein_coding	nicotinamide N-methyltransferase [Source:MGI Symbol;Acc:MGI:1099443]	<0.01	<0.01	-1
Irgm2	ENSMUSG00000069874	protein_coding	immunity-related GTPase family M member 2 [Source:MGI Symbol;Acc:MGI:1926262]	<0.01	0.019	-1
H2-Ab1	ENSMUSG00000073421	protein_coding	histocompatibility 2, class II antigen A, beta 1 [Source:MGI Symbol;Acc:MGI:103070]	<0.01	<0.01	-1
Gjb2	ENSMUSG00000046352	protein_coding	gap junction protein, beta 2 [Source:MGI Symbol;Acc:MGI:95720]	<0.01	0.037	-1
Klra9	ENSMUSG00000033024	protein_coding	killer cell lectin-like receptor subfamily A, member 9 [Source:MGI Symbol;Acc:MGI:1321153]	<0.01	0.033	-1
Zfp219	ENSMUSG00000049295	protein_coding	zinc finger protein 219 [Source:MGI Symbol;Acc:MGI:1917140]	<0.01	0.043	-1
Sh2d1b1	ENSMUSG00000102418	protein_coding	SH2 domain containing 1B1 [Source:MGI Symbol;Acc:MGI:1349420]	<0.01	<0.01	-1
Rpl21	ENSMUSG00000041453	protein_coding	ribosomal protein L21 [Source:MGI Symbol;Acc:MGI:1278340]	<0.01	0.015	-1
9930014A18Rik	ENSMUSG00000097493	processed_transcript	RIKEN cDNA 9930014A18 gene [Source:MGI Symbol;Acc:MGI:2444091]	<0.01	0.025	-1
S100a8	ENSMUSG00000056054	protein_coding	S100 calcium binding protein A8 (calgranulin A) [Source:MGI Symbol;Acc:MGI:88244]	<0.01	0.032	-1
Ggps1	ENSMUSG00000021302	protein_coding	geranylgeranyl diphosphate synthase 1 [Source:MGI Symbol;Acc:MGI:1341724]	<0.01	0.015	-1
Tmprss2	ENSMUSG00000000385	protein_coding	transmembrane protease, serine 2 [Source:MGI Symbol;Acc:MGI:1354381]	<0.01	0.031	-1
Igkv4-68	ENSMUSG00000076549	IG_V_gene	immunoglobulin kappa variable 4-68 [Source:MGI Symbol;Acc:MGI:2686265]	<0.01	0.020	-1
Mrpl27	ENSMUSG00000024414	protein_coding	mitochondrial ribosomal protein L27 [Source:MGI Symbol;Acc:MGI:2137224]	<0.01	<0.01	-1
Rnaset2b	ENSMUSG00000094724	protein_coding	ribonuclease T2B [Source:MGI Symbol;Acc:MGI:3702087]	<0.01	<0.01	-1
Trim34a	ENSMUSG00000056144	protein_coding	tripartite motif-containing 34A [Source:MGI Symbol;Acc:MGI:2137359]	<0.01	<0.01	-1
Igkv4-58	ENSMUSG00000095633	IG_V_gene	immunoglobulin kappa variable 4-58 [Source:MGI Symbol;Acc:MGI:2685923]	<0.01	0.042	-1
B3gnt3	ENSMUSG00000031803	protein_coding	UDP-GlcNAc:betaGal beta-1,3-N-acetylglucosaminyltransferase 3 [Source:MGI Symbol;Acc:MGI:2152535]	<0.01	<0.01	-1
Gm26752	ENSMUSG00000097541	lincRNA	predicted gene, 26752 [Source:MGI Symbol;Acc:MGI:5477246]	<0.01	<0.01	-1
Ahcyl2	ENSMUSG00000029772	protein_coding	S-adenosylhomocysteine hydrolase-like 2 [Source:MGI Symbol;Acc:MGI:1921590]	<0.01	<0.01	-1
Gm12501	ENSMUSG00000083462	processed_pseudogene	predicted gene 12501 [Source:MGI Symbol;Acc:MGI:3649307]	<0.01	0.019	1
Gm4924	ENSMUSG00000073427	protein_coding	predicted gene 4924 [Source:MGI Symbol;Acc:MGI:3643133]	<0.01	0.045	1
Cytl1	ENSMUSG00000062329	protein_coding	cytokine-like 1 [Source:MGI Symbol;Acc:MGI:2684993]	<0.01	<0.01	1
Dio2	ENSMUSG00000007682	protein_coding	deiodinase, iodothyronine, type II [Source:MGI Symbol;Acc:MGI:1338833]	<0.01	0.015	1
Smad6	ENSMUSG00000036867	protein_coding	SMAD family member 6 [Source:MGI Symbol;Acc:MGI:1336883]	<0.01	0.021	1
Sfrp1	ENSMUSG00000031548	protein_coding	secreted frizzled-related protein 1 [Source:MGI Symbol;Acc:MGI:892014]	<0.01	0.042	1
Rab2b	ENSMUSG00000022159	protein_coding	RAB2B, member RAS oncogene family [Source:MGI Symbol;Acc:MGI:1923588]	<0.01	<0.01	1
Alpl	ENSMUSG00000028766	protein_coding	alkaline phosphatase, liver/bone/kidney [Source:MGI Symbol;Acc:MGI:87983]	<0.01	<0.01	1
Slc13a4	ENSMUSG00000029843	protein_coding	solute carrier family 13 (sodium/sulfate symporters), member 4 [Source:MGI Symbol;Acc:MGI:2442367]	<0.01	<0.01	1
Pglyrp1	ENSMUSG00000030413	protein_coding	peptidoglycan recognition protein 1 [Source:MGI Symbol;Acc:MGI:1345092]	<0.01	0.044	1
Hddc3	ENSMUSG00000030532	protein_coding	HD domain containing 3 [Source:MGI Symbol;Acc:MGI:1915945]	<0.01	<0.01	1
Slc6a6	ENSMUSG00000030096	protein_coding	solute carrier family 6 (neurotransmitter transporter, taurine), member 6 [Source:MGI Symbol;Acc:MGI:98488]	<0.01	0.017	1
Gdf10	ENSMUSG00000021943	protein_coding	growth differentiation factor 10 [Source:MGI Symbol;Acc:MGI:95684]	<0.01	0.016	1
Grik3	ENSMUSG00000001985	protein_coding	glutamate receptor, ionotropic, kainate 3 [Source:MGI Symbol;Acc:MGI:95816]	<0.01	0.049	1

Igf1	ENSMUSG00000020053	protein_coding	insulin-like growth factor 1 [Source:MGI Symbol;Acc:MGI:96432]	<0.01	0.042	1
Plekha6	ENSMUSG00000041757	protein_coding	pleckstrin homology domain containing, family A member 6 [Source:MGI Symbol;Acc:MGI:2388662]	<0.01	<0.01	1
Cpne8	ENSMUSG00000052560	protein_coding	copine VIII [Source:MGI Symbol;Acc:MGI:1914121]	<0.01	0.017	1
Prkag3	ENSMUSG00000006542	protein_coding	protein kinase, AMP-activated, gamma 3 non-catatlytic subunit [Source:MGI Symbol;Acc:MGI:1891343]	<0.01	<0.01	1
Nifk	ENSMUSG00000026377	protein_coding	nucleolar protein interacting with the FHA domain of MKI67 [Source:MGI Symbol;Acc:MGI:1915199]	<0.01	0.047	1
Scrn1	ENSMUSG00000019124	protein_coding	secernin 1 [Source:MGI Symbol;Acc:MGI:1917188]	<0.01	0.021	1
Gm12222	ENSMUSG00000060068	processed_pseudogene	predicted gene 12222 [Source:MGI Symbol;Acc:MGI:3649523]	<0.01	0.049	1
Vasn	ENSMUSG00000039646	protein_coding	vasorin [Source:MGI Symbol;Acc:MGI:2177651]	<0.01	0.033	1
Mmp24	ENSMUSG00000027612	protein_coding	matrix metallopeptidase 24 [Source:MGI Symbol;Acc:MGI:1341867]	<0.01	0.030	1
Btbd19	ENSMUSG00000073771	protein_coding	BTB (POZ) domain containing 19 [Source:MGI Symbol;Acc:MGI:1925861]	<0.01	0.017	1
Trp53bp2	ENSMUSG00000026510	protein_coding	transformation related protein 53 binding protein 2 [Source:MGI Symbol;Acc:MGI:2138319]	<0.01	0.011	1
Borcs8	ENSMUSG00000002345	protein_coding	BLOC-1 related complex subunit 8 [Source:MGI Symbol;Acc:MGI:1919618]	<0.01	0.014	1
Rbp4	ENSMUSG00000024990	protein_coding	retinol binding protein 4, plasma [Source:MGI Symbol;Acc:MGI:97879]	<0.01	0.042	1
Mpi	ENSMUSG00000032306	protein_coding	mannose phosphate isomerase [Source:MGI Symbol;Acc:MGI:97075]	<0.01	0.040	1
Fam118a	ENSMUSG00000022434	protein_coding	family with sequence similarity 118, member A [Source:MGI Symbol;Acc:MGI:1920475]	<0.01	<0.01	1
Lonrf1	ENSMUSG00000039633	protein_coding	LON peptidase N-terminal domain and ring finger 1 [Source:MGI Symbol;Acc:MGI:3609241]	<0.01	0.038	1
Bhlhe41	ENSMUSG00000030256	protein_coding	basic helix-loop-helix family, member e41 [Source:MGI Symbol;Acc:MGI:1930704]	<0.01	<0.01	1
Rpl21-ps10	ENSMUSG00000105359	processed_pseudogene	ribosomal protein L21, pseudogene 10 [Source:MGI Symbol;Acc:MGI:3643358]	<0.01	<0.01	1
Mustn1	ENSMUSG00000042485	protein_coding	musculoskeletal, embryonic nuclear protein 1 [Source:MGI Symbol;Acc:MGI:1913425]	<0.01	0.040	1
Pnliprp1	ENSMUSG00000042179	protein_coding	pancreatic lipase related protein 1 [Source:MGI Symbol;Acc:MGI:97723]	<0.01	0.024	1
1810011O10Rik	ENSMUSG00000056313	protein_coding	RIKEN cDNA 1810011O10 gene [Source:MGI Symbol;Acc:MGI:1916318]	<0.01	0.034	1
Nras	ENSMUSG00000027852	protein_coding	neuroblastoma ras oncogene [Source:MGI Symbol;Acc:MGI:97376]	<0.01	0.044	1
Atp6v0d2	ENSMUSG00000028238	protein_coding	ATPase, H+ transporting, lysosomal V0 subunit D2 [Source:MGI Symbol;Acc:MGI:1924415]	<0.01	0.030	1
Gm5575	ENSMUSG00000099891	processed_pseudogene	predicted gene 5575 [Source:MGI Symbol;Acc:MGI:3647974]	<0.01	0.028	1
Zfp322a	ENSMUSG00000046351	protein_coding	zinc finger protein 322A [Source:MGI Symbol;Acc:MGI:2442566]	<0.01	0.043	1
Aqp5	ENSMUSG00000044217	protein_coding	aquaporin 5 [Source:MGI Symbol;Acc:MGI:106215]	<0.01	0.040	1
Calr3	ENSMUSG00000019732	protein_coding	calreticulin 3 [Source:MGI Symbol;Acc:MGI:1920566]	<0.01	<0.01	1
Hspg2	ENSMUSG00000028763	protein_coding	perlecan (heparan sulfate proteoglycan 2) [Source:MGI Symbol;Acc:MGI:96257]	<0.01	0.019	1
Cryz	ENSMUSG00000028199	protein_coding	crystallin, zeta [Source:MGI Symbol;Acc:MGI:88527]	<0.01	0.040	1
Ntrk2	ENSMUSG00000055254	protein_coding	neurotrophic tyrosine kinase, receptor, type 2 [Source:MGI Symbol;Acc:MGI:97384]	<0.01	<0.01	1
Xkr6	ENSMUSG00000035067	protein_coding	X-linked Kx blood group related 6 [Source:MGI Symbol;Acc:MGI:2447765]	<0.01	0.031	1
Gm15337	ENSMUSG00000085289	antisense_RNA	predicted gene 15337 [Source:MGI Symbol;Acc:MGI:3705103]	<0.01	0.033	1
Nav1	ENSMUSG00000009418	protein_coding	neuron navigator 1 [Source:MGI Symbol;Acc:MGI:2183683]	<0.01	0.027	1
Abhd1	ENSMUSG00000006638	processed_transcript	abhydrolase domain containing 1 [Source:MGI Symbol;Acc:MGI:1931013]	<0.01	<0.01	1
Kctd6	ENSMUSG00000021752	protein_coding	potassium channel tetramerisation domain containing 6 [Source:MGI Symbol;Acc:MGI:1918643]	<0.01	0.028	1
Car11	ENSMUSG00000003273	protein_coding	carbonic anhydrase 11 [Source:MGI Symbol;Acc:MGI:1336193]	<0.01	<0.01	1
Myct1	ENSMUSG00000046916	protein_coding	myc target 1 [Source:MGI Symbol;Acc:MGI:1915882]	<0.01	<0.01	1
Rpl15	ENSMUSG00000012405	protein_coding	ribosomal protein L15 [Source:MGI Symbol;Acc:MGI:1913730]	<0.01	<0.01	1
Wnt9a	ENSMUSG00000000126	protein_coding	wingless-type MMTV integration site family, member 9A [Source:MGI Symbol;Acc:MGI:2446084]	<0.01	0.045	1
Polr2h	ENSMUSG00000021018	protein_coding	polymerase (RNA) II (DNA directed) polypeptide H [Source:MGI Symbol;Acc:MGI:2384309]	<0.01	0.044	1
Agmo	ENSMUSG00000050103	protein_coding	alkylglycerol monooxygenase [Source:MGI Symbol;Acc:MGI:2442495]	<0.01	<0.01	1
Lrrc4b	ENSMUSG00000047085	protein_coding	leucine rich repeat containing 4B [Source:MGI Symbol;Acc:MGI:3027390]	<0.01	<0.01	1
Rps3a3	ENSMUSG00000059751	processed_pseudogene	ribosomal protein S3A3 [Source:MGI Symbol;Acc:MGI:3643406]	<0.01	<0.01	1
Gm37516	ENSMUSG00000104376	TEC	predicted gene, 37516 [Source:MGI Symbol;Acc:MGI:5610744]	<0.01	0.034	1
Copg2	ENSMUSG00000025607	protein_coding	coatomer protein complex, subunit gamma 2 [Source:MGI Symbol;Acc:MGI:1858683]	<0.01	<0.01	1
Ebpl	ENSMUSG00000021928	protein_coding	emopamil binding protein-like [Source:MGI Symbol;Acc:MGI:1915427]	<0.01	0.023	1
Glo1	ENSMUSG00000024026	protein_coding	glyoxalase 1 [Source:MGI Symbol;Acc:MGI:95742]	<0.01	<0.01	1
Phf20	ENSMUSG00000038116	protein_coding	PHD finger protein 20 [Source:MGI Symbol;Acc:MGI:2444148]	<0.01	0.037	1
Myl12b	ENSMUSG00000034868	protein_coding	myosin, light chain 12B, regulatory [Source:MGI Symbol;Acc:MGI:107494]	<0.01	0.014	1
Ccdc34	ENSMUSG00000027160	protein_coding	coiled-coil domain containing 34 [Source:MGI Symbol;Acc:MGI:1915451]	<0.01	0.050	1
S100a14	ENSMUSG00000042306	protein_coding	S100 calcium binding protein A14 [Source:MGI Symbol;Acc:MGI:1913416]	<0.01	0.020	1
AA465934	ENSMUSG00000093483	processed_transcript	expressed sequence AA465934 [Source:MGI Symbol;Acc:MGI:2671018]	<0.01	<0.01	1
Fpr2	ENSMUSG00000052270	protein_coding	formyl peptide receptor 2 [Source:MGI Symbol;Acc:MGI:1278319]	<0.01	<0.01	1
Glrb	ENSMUSG00000028020	protein_coding	glycine receptor, beta subunit [Source:MGI Symbol;Acc:MGI:95751]	<0.01	<0.01	1
Gm3375	ENSMUSG00000107470	processed_pseudogene	predicted gene 3375 [Source:MGI Symbol;Acc:MGI:3781553]	<0.01	<0.01	1
RP24-74B24.4	ENSMUSG00000108658	TEC	predicted gene 45138 [Source:MGI Symbol;Acc:MGI:5753714]	<0.01	<0.01	1
Arhgef10	ENSMUSG00000071176	protein_coding	Rho guanine nucleotide exchange factor (GEF) 10 [Source:MGI Symbol;Acc:MGI:2444453]	<0.01	0.031	1
BC029722	ENSMUSG00000074649	protein_coding	cDNA sequence BC029722 [Source:MGI Symbol;Acc:MGI:3584273]	<0.01	0.015	1
Gm20478	ENSMUSG00000092474	antisense_RNA	predicted gene 20478 [Source:MGI Symbol;Acc:MGI:5141943]	<0.01	<0.01	1
Rps26-ps1	ENSMUSG00000059775	transcribed_processed_pseudogene	ribosomal protein S26, pseudogene 1 [Source:MGI Symbol;Acc:MGI:3704322]	<0.01	0.021	1
Lysmd2	ENSMUSG00000032184	protein_coding	LysM, putative peptidoglycan-binding, domain containing 2 [Source:MGI Symbol;Acc:MGI:1917332]	<0.01	0.038	1
Gm8420	ENSMUSG00000072324	processed_pseudogene	predicted gene 8420 [Source:MGI Symbol;Acc:MGI:3645594]	<0.01	<0.01	1
A930015D03Rik	ENSMUSG00000092368	antisense_RNA	RIKEN cDNA A930015D03 gene [Source:MGI Symbol;Acc:MGI:1925060]	<0.01	<0.01	1
Rtn4rl1	ENSMUSG00000045287	protein_coding	reticulon 4 receptor-like 1 [Source:MGI Symbol;Acc:MGI:2661375]	<0.01	0.024	1
Palmd	ENSMUSG00000033377	protein_coding	palmdelphin [Source:MGI Symbol;Acc:MGI:2148896]	<0.01	<0.01	1
Dnajc10	ENSMUSG00000027006	protein_coding	DnaJ heat shock protein family (Hsp40) member C10 [Source:MGI Symbol;Acc:MGI:1914111]	<0.01	0.028	1
Mtrf1l	ENSMUSG00000019774	protein_coding	mitochondrial translational release factor 1-like [Source:MGI Symbol;Acc:MGI:1918830]	<0.01	0.015	1
Gchfr	ENSMUSG00000046814	protein_coding	GTP cyclohydrolase I feedback regulator [Source:MGI Symbol;Acc:MGI:2443977]	<0.01	<0.01	1
Scel	ENSMUSG00000022123	protein_coding	sciellin [Source:MGI Symbol;Acc:MGI:1891228]	<0.01	0.030	1
Pigf	ENSMUSG00000024145	protein_coding	phosphatidylinositol glycan anchor biosynthesis, class F [Source:MGI Symbol;Acc:MGI:99462]	<0.01	0.025	1
Hdhd3	ENSMUSG00000038422	protein_coding	haloacid dehalogenase-like hydrolase domain containing 3 [Source:MGI Symbol;Acc:MGI:1919998]	<0.01	<0.01	1
Lrrk1	ENSMUSG00000015133	protein_coding	leucine-rich repeat kinase 1 [Source:MGI Symbol;Acc:MGI:2142227]	<0.01	0.038	1
Gm21596	ENSMUSG00000096006	processed_pseudogene	predicted gene, 21596 [Source:MGI Symbol;Acc:MGI:5434951]	<0.01	0.040	1
Abca17	ENSMUSG00000035435	protein_coding	ATP-binding cassette, sub-family A (ABC1), member 17 [Source:MGI Symbol;Acc:MGI:3625331]	<0.01	0.030	1
Tsen2	ENSMUSG00000042389	protein_coding	tRNA splicing endonuclease subunit 2 [Source:MGI Symbol;Acc:MGI:2141599]	<0.01	0.016	1
Tulp1	ENSMUSG00000037446	protein_coding	tubby like protein 1 [Source:MGI Symbol;Acc:MGI:109571]	<0.01	0.012	1
1500011B03Rik	ENSMUSG00000072694	protein_coding	RIKEN cDNA 1500011B03 gene [Source:MGI Symbol;Acc:MGI:1913486]	<0.01	<0.01	1
Cd207	ENSMUSG00000034783	protein_coding	CD207 antigen [Source:MGI Symbol;Acc:MGI:2180021]	<0.01	<0.01	1
Rarres2	ENSMUSG00000009281	protein_coding	retinoic acid receptor responder (tazarotene induced) 2 [Source:MGI Symbol;Acc:MGI:1918910]	<0.01	0.024	1
Ppp1r12b	ENSMUSG00000073557	protein_coding	protein phosphatase 1, regulatory (inhibitor) subunit 12B [Source:MGI Symbol;Acc:MGI:1916417]	<0.01	0.013	1

Lum	ENSMUSG00000036446	protein_coding	lumican [Source:MGI Symbol;Acc:MGI:109347]	<0.01	0.034	1
Rras2	ENSMUSG00000055723	protein_coding	related RAS viral (r-ras) oncogene 2 [Source:MGI Symbol;Acc:MGI:1914172]	<0.01	0.037	1
Synj2bp	ENSMUSG00000090935	protein_coding	synaptojanin 2 binding protein [Source:MGI Symbol;Acc:MGI:1344347]	<0.01	0.047	1
Syn3	ENSMUSG00000059602	protein_coding	synapsin III [Source:MGI Symbol;Acc:MGI:1351334]	<0.01	0.017	1
Ncaph	ENSMUSG00000034906	protein_coding	non-SMC condensin I complex, subunit H [Source:MGI Symbol;Acc:MGI:2444777]	<0.01	0.016	1
Atp6v0e2	ENSMUSG00000039347	protein_coding	ATPase, H+ transporting, lysosomal V0 subunit E2 [Source:MGI Symbol;Acc:MGI:1923502]	<0.01	<0.01	1
Tnn	ENSMUSG00000026725	protein_coding	tenascin N [Source:MGI Symbol;Acc:MGI:2665790]	<0.01	<0.01	1
Nppc	ENSMUSG00000026241	protein_coding	natriuretic peptide type C [Source:MGI Symbol;Acc:MGI:97369]	<0.01	0.013	1
Hpcal4	ENSMUSG00000046093	protein_coding	hippocalcin-like 4 [Source:MGI Symbol;Acc:MGI:2157521]	<0.01	<0.01	1
Clec3b	ENSMUSG00000025784	protein_coding	C-type lectin domain family 3, member b [Source:MGI Symbol;Acc:MGI:104540]	<0.01	0.010	1
Fam234b	ENSMUSG00000030207	protein_coding	family with sequence similarity 234, member B [Source:MGI Symbol;Acc:MGI:1921775]	<0.01	0.049	1
Mpc1	ENSMUSG00000023861	protein_coding	mitochondrial pyruvate carrier 1 [Source:MGI Symbol;Acc:MGI:1915240]	<0.01	<0.01	1
Pcsk5	ENSMUSG00000024713	protein_coding	proprotein convertase subtilisin/kexin type 5 [Source:MGI Symbol;Acc:MGI:97515]	<0.01	0.046	1
Taf4b	ENSMUSG00000054321	protein_coding	TATA-box binding protein associated factor 4b [Source:MGI Symbol;Acc:MGI:2152345]	<0.01	0.034	1
Gm8909	ENSMUSG00000073402	protein_coding	predicted gene 8909 [Source:MGI Symbol;Acc:MGI:3704134]	<0.01	<0.01	1
Gm42835	ENSMUSG00000104554	antisense_RNA	predicted gene 4610 [Source:MGI Symbol;Acc:MGI:3782793]	<0.01	<0.01	1
Rcan2	ENSMUSG00000039601	protein_coding	regulator of calcineurin 2 [Source:MGI Symbol;Acc:MGI:1858219]	<0.01	<0.01	1
Nr1h4	ENSMUSG00000047638	protein_coding	nuclear receptor subfamily 1, group H, member 4 [Source:MGI Symbol;Acc:MGI:1352464]	<0.01	0.011	1
Gm12892	ENSMUSG00000083679	processed_pseudogene	predicted gene 12892 [Source:MGI Symbol;Acc:MGI:3649649]	<0.01	0.013	1
Mfap1b	ENSMUSG00000048222	protein_coding	microfibrillar-associated protein 1B [Source:MGI Symbol;Acc:MGI:3694697]	<0.01	0.030	1
Gm6245	ENSMUSG00000090475	processed_pseudogene	predicted gene 6245 [Source:MGI Symbol;Acc:MGI:3646756]	<0.01	0.037	1
Rps15a-ps3	ENSMUSG00000084314	processed_pseudogene	ribosomal protein S15A, pseudogene 3 [Source:MGI Symbol;Acc:MGI:3650886]	<0.01	<0.01	1
6430573F11Rik	ENSMUSG00000039620	protein_coding	RIKEN cDNA 6430573F11 gene [Source:MGI Symbol;Acc:MGI:2442328]	<0.01	<0.01	1
Hmgb1	ENSMUSG00000066551	protein_coding	high mobility group box 1 [Source:MGI Symbol;Acc:MGI:96113]	<0.01	0.012	1
Dnase2a	ENSMUSG00000003812	protein_coding	deoxyribonuclease II alpha [Source:MGI Symbol;Acc:MGI:1329019]	<0.01	0.018	1
Plaur	ENSMUSG00000046223	protein_coding	plasminogen activator, urokinase receptor [Source:MGI Symbol;Acc:MGI:97612]	<0.01	0.046	1
Cntnap2	ENSMUSG00000039419	protein_coding	contactin associated protein-like 2 [Source:MGI Symbol;Acc:MGI:1914047]	<0.01	<0.01	1
Postn	ENSMUSG00000027750	protein_coding	periostin, osteoblast specific factor [Source:MGI Symbol;Acc:MGI:1926321]	<0.01	0.020	1
Dph6	ENSMUSG00000057147	protein_coding	diphthamine biosynthesis 6 [Source:MGI Symbol;Acc:MGI:1913882]	<0.01	<0.01	1
Lpar6	ENSMUSG00000033446	protein_coding	lysophosphatidic acid receptor 6 [Source:MGI Symbol;Acc:MGI:1914418]	<0.01	0.012	1
Gm5611	ENSMUSG00000090602	processed_pseudogene	predicted gene 5611 [Source:MGI Symbol;Acc:MGI:3647121]	<0.01	0.050	1
Tmem126b	ENSMUSG00000030614	protein_coding	transmembrane protein 126B [Source:MGI Symbol;Acc:MGI:1915722]	<0.01	0.026	1
Smim19	ENSMUSG00000031534	protein_coding	small integral membrane protein 19 [Source:MGI Symbol;Acc:MGI:2142501]	<0.01	0.030	1
RP24-330M21.1	ENSMUSG00000109244	lincRNA	predicted gene 44751 [Source:MGI Symbol;Acc:MGI:5753327]	<0.01	<0.01	1
Ccdc68	ENSMUSG00000038903	protein_coding	coiled-coil domain containing 68 [Source:MGI Symbol;Acc:MGI:3612676]	<0.01	0.015	1
Nr1d1	ENSMUSG00000020889	protein_coding	nuclear receptor subfamily 1, group D, member 1 [Source:MGI Symbol;Acc:MGI:2444210]	<0.01	<0.01	1
Insc	ENSMUSG00000048782	protein_coding	inscuteable homolog (Drosophila) [Source:MGI Symbol;Acc:MGI:1917942]	<0.01	0.034	1
Gm2904	ENSMUSG00000079101	processed_pseudogene	predicted pseudogene 2904 [Source:MGI Symbol;Acc:MGI:3781082]	<0.01	0.019	1
Gm15446	ENSMUSG00000090015	protein_coding	predicted gene 15446 [Source:MGI Symbol;Acc:MGI:3709333]	<0.01	<0.01	1
Fam118b	ENSMUSG00000050471	protein_coding	family with sequence similarity 118, member B [Source:MGI Symbol;Acc:MGI:1924483]	<0.01	0.049	1
Bckdhb	ENSMUSG00000032263	protein_coding	branched chain ketoacid dehydrogenase E1, beta polypeptide [Source:MGI Symbol;Acc:MGI:88137]	<0.01	<0.01	1
Hyal1	ENSMUSG00000010051	protein_coding	hyaluronoglucosaminidase 1 [Source:MGI Symbol;Acc:MGI:96298]	<0.01	<0.01	1
Gm15931	ENSMUSG00000081723	unprocessed_pseudogene	predicted gene 15931 [Source:MGI Symbol;Acc:MGI:3805553]	<0.01	0.040	1
44257	ENSMUSG00000079557	protein_coding	membrane-associated ring finger (C3HC4) 2 [Source:MGI Symbol;Acc:MGI:1925915]	<0.01	0.039	1
Pak6	ENSMUSG00000074923	protein_coding	p21 protein (Cdc42/Rac)-activated kinase 6 [Source:MGI Symbol;Acc:MGI:2679420]	<0.01	0.019	1
Agrn	ENSMUSG00000041936	protein_coding	agrin [Source:MGI Symbol;Acc:MGI:87961]	<0.01	<0.01	1
Krt78	ENSMUSG00000050463	protein_coding	keratin 78 [Source:MGI Symbol;Acc:MGI:1917529]	<0.01	<0.01	1
Mex3b	ENSMUSG00000057706	protein_coding	mex3 RNA binding family member B [Source:MGI Symbol;Acc:MGI:1918252]	<0.01	0.033	1
Kcna2	ENSMUSG00000040724	protein_coding	potassium voltage-gated channel, shaker-related subfamily, member 2 [Source:MGI Symbol;Acc:MGI:96659]	<0.01	0.019	1
Coq10b	ENSMUSG00000025981	protein_coding	coenzyme Q10B [Source:MGI Symbol;Acc:MGI:1915126]	<0.01	0.020	1
Inpp5f	ENSMUSG00000042105	protein_coding	inositol polyphosphate-5-phosphatase F [Source:MGI Symbol;Acc:MGI:2141867]	<0.01	0.045	1
Gm28044	ENSMUSG00000098781	protein_coding	predicted gene, 28044 [Source:MGI Symbol;Acc:MGI:5547780]	<0.01	<0.01	1
Gria1	ENSMUSG00000020524	protein_coding	glutamate receptor, ionotropic, AMPA1 (alpha 1) [Source:MGI Symbol;Acc:MGI:95808]	<0.01	0.039	1
Ubiad1	ENSMUSG00000047719	protein_coding	UbiA prenyltransferase domain containing 1 [Source:MGI Symbol;Acc:MGI:1918957]	<0.01	0.020	1
Pdgfrl	ENSMUSG00000031595	protein_coding	platelet-derived growth factor receptor-like [Source:MGI Symbol;Acc:MGI:1916047]	<0.01	0.048	1
Pianp	ENSMUSG00000030329	protein_coding	PILR alpha associated neural protein [Source:MGI Symbol;Acc:MGI:2441908]	<0.01	<0.01	1
Grin2c	ENSMUSG00000020734	protein_coding	glutamate receptor, ionotropic, NMDA2C (epsilon 3) [Source:MGI Symbol;Acc:MGI:95822]	<0.01	<0.01	1
Elp6	ENSMUSG00000054836	protein_coding	elongator acetyltransferase complex subunit 6 [Source:MGI Symbol;Acc:MGI:1919349]	<0.01	<0.01	1
Kcne3	ENSMUSG00000035165	protein_coding	potassium voltage-gated channel, Isk-related subfamily, gene 3 [Source:MGI Symbol;Acc:MGI:1891124]	<0.01	<0.01	1
Rprml	ENSMUSG00000046215	protein_coding	reprimo-like [Source:MGI Symbol;Acc:MGI:2144486]	<0.01	<0.01	1
Hist2h3c2	ENSMUSG00000081058	protein_coding	histone cluster 2, H3c2 [Source:MGI Symbol;Acc:MGI:2448357]	<0.01	0.034	1
Arl14ep	ENSMUSG00000027122	protein_coding	ADP-ribosylation factor-like 14 effector protein [Source:MGI Symbol;Acc:MGI:1926020]	<0.01	<0.01	1
Eif4g1	ENSMUSG00000045983	protein_coding	eukaryotic translation initiation factor 4, gamma 1 [Source:MGI Symbol;Acc:MGI:2384784]	<0.01	0.045	1
Sh2d4a	ENSMUSG00000053886	protein_coding	SH2 domain containing 4A [Source:MGI Symbol;Acc:MGI:1919531]	<0.01	<0.01	1
Plxdc1	ENSMUSG00000017417	protein_coding	plexin domain containing 1 [Source:MGI Symbol;Acc:MGI:1919574]	<0.01	<0.01	1
Cnn3	ENSMUSG00000053931	protein_coding	calponin 3, acidic [Source:MGI Symbol;Acc:MGI:1919244]	<0.01	<0.01	1
Rnmtl1	ENSMUSG00000038046	protein_coding	mitochondrial rRNA methyltransferase 3 [Source:MGI Symbol;Acc:MGI:1914640]	<0.01	0.042	1
Nol10	ENSMUSG00000061458	protein_coding	nucleolar protein 10 [Source:MGI Symbol;Acc:MGI:2684913]	<0.01	0.013	1
Ccdc190	ENSMUSG00000070532	protein_coding	coiled-coil domain containing 190 [Source:MGI Symbol;Acc:MGI:1925715]	<0.01	<0.01	1
Glrx	ENSMUSG00000021591	protein_coding	glutaredoxin [Source:MGI Symbol;Acc:MGI:2135625]	<0.01	0.048	1
Pus7	ENSMUSG00000057541	protein_coding	pseudouridylate synthase 7 [Source:MGI Symbol;Acc:MGI:1925947]	<0.01	<0.01	1
Tox2	ENSMUSG00000074607	protein_coding	TOX high mobility group box family member 2 [Source:MGI Symbol;Acc:MGI:3611233]	<0.01	<0.01	1
Gbp2b	ENSMUSG00000040264	protein_coding	guanylate binding protein 2b [Source:MGI Symbol;Acc:MGI:95666]	<0.01	<0.01	1
Ly6c1	ENSMUSG00000079018	protein_coding	lymphocyte antigen 6 complex, locus C1 [Source:MGI Symbol;Acc:MGI:96882]	<0.01	0.016	1
Ltbp1	ENSMUSG00000001870	protein_coding	latent transforming growth factor beta binding protein 1 [Source:MGI Symbol;Acc:MGI:109151]	<0.01	0.033	1
Mthfd1l	ENSMUSG00000040675	protein_coding	methylenetetrahydrofolate dehydrogenase (NADP+ dependent) 1-like [Source:MGI Symbol;Acc:MGI:1924836]	<0.01	<0.01	1
Ckmt1	ENSMUSG00000000308	protein_coding	creatine kinase, mitochondrial 1, ubiquitous [Source:MGI Symbol;Acc:MGI:99441]	<0.01	0.034	1
Adgrl1	ENSMUSG00000013033	protein_coding	adhesion G protein-coupled receptor L1 [Source:MGI Symbol;Acc:MGI:1929461]	<0.01	0.015	1
Gm15772	ENSMUSG00000062353	processed_pseudogene	predicted gene 15772 [Source:MGI Symbol;Acc:MGI:3805541]	<0.01	<0.01	1
Tifab	ENSMUSG00000049625	protein_coding	TRAF-interacting protein with forkhead-associated domain, family member B [Source:MGI Symbol;Acc:MGI:2385852]	<0.01	0.020	1

Col12a1	ENSMUSG00000032332	protein_coding	collagen, type XII, alpha 1 [Source:MGI Symbol;Acc:MGI:88448]	<0.01	<0.01	1
Eif3j2	ENSMUSG00000043424	protein_coding	eukaryotic translation initiation factor 3, subunit J2 [Source:MGI Symbol;Acc:MGI:3704486]	<0.01	<0.01	1
Eif2s2	ENSMUSG00000074656	protein_coding	eukaryotic translation initiation factor 2, subunit 2 (beta) [Source:MGI Symbol;Acc:MGI:1914454]	<0.01	<0.01	1
Lamc3	ENSMUSG00000026840	protein_coding	laminin gamma 3 [Source:MGI Symbol;Acc:MGI:1344394]	<0.01	0.020	1
Ackr4	ENSMUSG00000079355	protein_coding	atypical chemokine receptor 4 [Source:MGI Symbol;Acc:MGI:2181676]	<0.01	0.019	1
Pnmal2	ENSMUSG00000070802	protein_coding	PNMA-like 2 [Source:MGI Symbol;Acc:MGI:3645856]	<0.01	<0.01	1
Ttl	ENSMUSG00000027394	protein_coding	tubulin tyrosine ligase [Source:MGI Symbol;Acc:MGI:1916987]	<0.01	0.036	1
Smtn	ENSMUSG00000020439	protein_coding	smoothelin [Source:MGI Symbol;Acc:MGI:1354727]	<0.01	0.034	1
Inf2	ENSMUSG00000037679	protein_coding	inverted formin, FH2 and WH2 domain containing [Source:MGI Symbol;Acc:MGI:1917685]	<0.01	<0.01	1
B3gat2	ENSMUSG00000026156	protein_coding	beta-1,3-glucuronyltransferase 2 (glucuronosyltransferase S) [Source:MGI Symbol;Acc:MGI:2389490]	<0.01	0.043	1
Ept1	ENSMUSG00000075703	protein_coding	selenoprotein I [Source:MGI Symbol;Acc:MGI:107898]	<0.01	<0.01	1
Alad	ENSMUSG00000028393	protein_coding	aminolevulinate, delta-, dehydratase [Source:MGI Symbol;Acc:MGI:96853]	<0.01	<0.01	1
Adamts7	ENSMUSG00000032363	protein_coding	a disintegrin-like and metallopeptidase (reprolysin type) with thrombospondin type 1 motif, 7 [Source:MGI Symbol;Acc:MGI:1347346]	<0.01	0.014	1
Col6a5	ENSMUSG00000091345	protein_coding	collagen, type VI, alpha 5 [Source:MGI Symbol;Acc:MGI:3648134]	<0.01	<0.01	1
Ccdc85a	ENSMUSG00000032878	protein_coding	coiled-coil domain containing 85A [Source:MGI Symbol;Acc:MGI:2445069]	<0.01	<0.01	1
B3gnt7	ENSMUSG00000079445	protein_coding	UDP-GlcNAc:betaGal beta-1,3-N-acetylglucosaminyltransferase 7 [Source:MGI Symbol;Acc:MGI:2384394]	<0.01	<0.01	1
Hmga1-rs1	ENSMUSG00000078249	protein_coding	high mobility group AT-hook I, related sequence 1 [Source:MGI Symbol;Acc:MGI:96161]	<0.01	<0.01	1
Zdhhc2	ENSMUSG00000039470	protein_coding	zinc finger, DHHC domain containing 2 [Source:MGI Symbol;Acc:MGI:1923452]	<0.01	<0.01	1
Crym	ENSMUSG00000030905	protein_coding	crystallin, mu [Source:MGI Symbol;Acc:MGI:102675]	<0.01	<0.01	1
Dtna	ENSMUSG00000024302	protein_coding	dystrobrevin alpha [Source:MGI Symbol;Acc:MGI:106039]	<0.01	0.031	1
Piezo2	ENSMUSG00000041482	protein_coding	piezo-type mechanosensitive ion channel component 2 [Source:MGI Symbol;Acc:MGI:1918781]	<0.01	0.024	1
C130074G19Rik	ENSMUSG00000039349	protein_coding	RIKEN cDNA C130074G19 gene [Source:MGI Symbol;Acc:MGI:2444831]	<0.01	0.029	1
Jdp2	ENSMUSG00000034271	protein_coding	Jun dimerization protein 2 [Source:MGI Symbol;Acc:MGI:1932093]	<0.01	0.032	1
Rps2-ps10	ENSMUSG00000091957	transcribed_processed_pseudogene	ribosomal protein S2, pseudogene 10 [Source:MGI Symbol;Acc:MGI:3645604]	<0.01	<0.01	1
Ap3m2	ENSMUSG00000031539	protein_coding	adaptor-related protein complex 3, mu 2 subunit [Source:MGI Symbol;Acc:MGI:1929214]	<0.01	0.037	1
Crabp1	ENSMUSG00000032291	protein_coding	cellular retinoic acid binding protein I [Source:MGI Symbol;Acc:MGI:88490]	<0.01	0.011	1
Rsad2	ENSMUSG00000020641	protein_coding	radical S-adenosyl methionine domain containing 2 [Source:MGI Symbol;Acc:MGI:1929628]	<0.01	0.040	1
Gm13248	ENSMUSG00000063245	protein_coding	zinc finger protein 993 [Source:MGI Symbol;Acc:MGI:3713585]	<0.01	<0.01	1
Iqcc	ENSMUSG00000040795	protein_coding	IQ motif containing C [Source:MGI Symbol;Acc:MGI:2446212]	<0.01	0.024	1
Mycn	ENSMUSG00000037169	protein_coding	v-myc avian myelocytomatosis viral related oncogene, neuroblastoma derived [Source:MGI Symbol;Acc:MGI:97357]	<0.01	<0.01	1
Olr1	ENSMUSG00000030162	protein_coding	oxidized low density lipoprotein (lectin-like) receptor 1 [Source:MGI Symbol;Acc:MGI:1261434]	<0.01	0.043	1
Gm20513	ENSMUSG00000092415	lincRNA	predicted gene 20513 [Source:MGI Symbol;Acc:MGI:5141978]	<0.01	<0.01	1
Nudt5	ENSMUSG00000025817	protein_coding	nudix (nucleoside diphosphate linked moiety X)-type motif 5 [Source:MGI Symbol;Acc:MGI:1858232]	<0.01	0.027	1
Atxn7l1	ENSMUSG00000020564	protein_coding	ataxin 7-like 1 [Source:MGI Symbol;Acc:MGI:3584458]	<0.01	0.049	1
Rev1	ENSMUSG00000026082	protein_coding	REV1, DNA directed polymerase [Source:MGI Symbol;Acc:MGI:1929074]	<0.01	0.012	1
Coq7	ENSMUSG00000030652	protein_coding	demethyl-Q 7 [Source:MGI Symbol;Acc:MGI:107207]	<0.01	<0.01	1
Atp5g2	ENSMUSG00000062683	protein_coding	ATP synthase, H+ transporting, mitochondrial F0 complex, subunit C2 (subunit 9) [Source:MGI Symbol;Acc:MGI:1915192]	<0.01	<0.01	1
B3galt4	ENSMUSG00000067370	protein_coding	UDP-Gal:betaGlcNAc beta 1,3-galactosyltransferase, polypeptide 4 [Source:MGI Symbol;Acc:MGI:1859517]	<0.01	0.037	1
Ppm1m	ENSMUSG00000020253	protein_coding	protein phosphatase 1M [Source:MGI Symbol;Acc:MGI:1915155]	<0.01	0.027	1
Mettl7a3	ENSMUSG00000058057	protein_coding	methyltransferase like 7A3 [Source:MGI Symbol;Acc:MGI:3710670]	<0.01	0.031	1
Gm26821	ENSMUSG00000097038	lincRNA	predicted gene, 26821 [Source:MGI Symbol;Acc:MGI:5477315]	<0.01	0.042	1
Gm5292	ENSMUSG00000059565	processed_pseudogene	predicted gene 5292 [Source:MGI Symbol;Acc:MGI:3645663]	<0.01	0.029	1
Gpihbp1	ENSMUSG00000022579	protein_coding	GPI-anchored HDL-binding protein 1 [Source:MGI Symbol;Acc:MGI:1915703]	<0.01	0.012	1
Scn7a	ENSMUSG00000034810	protein_coding	sodium channel, voltage-gated, type VII, alpha [Source:MGI Symbol;Acc:MGI:102965]	<0.01	0.010	1
Plau	ENSMUSG00000021822	protein_coding	plasminogen activator, urokinase [Source:MGI Symbol;Acc:MGI:97611]	<0.01	0.017	1
Emcn	ENSMUSG00000054690	protein_coding	endomucin [Source:MGI Symbol;Acc:MGI:1891716]	<0.01	0.028	1
Trim65	ENSMUSG00000054517	protein_coding	tripartite motif-containing 65 [Source:MGI Symbol;Acc:MGI:2442815]	<0.01	0.043	1
Gm10020	ENSMUSG00000057262	protein_coding	predicted pseudogene 10020 [Source:MGI Symbol;Acc:MGI:3642192]	<0.01	<0.01	1
Sybu	ENSMUSG00000022340	protein_coding	syntabulin (syntaxin-interacting) [Source:MGI Symbol;Acc:MGI:2442392]	<0.01	0.019	1
Gm8935	ENSMUSG00000095661	transcribed_processed_pseudogene	predicted pseudogene 8935 [Source:MGI Symbol;Acc:MGI:3643401]	<0.01	0.049	1
Tmem252	ENSMUSG00000048572	protein_coding	transmembrane protein 252 [Source:MGI Symbol;Acc:MGI:3583948]	<0.01	<0.01	1
Gbp11	ENSMUSG00000092021	polymorphic_pseudogene	guanylate binding protein 11 [Source:MGI Symbol;Acc:MGI:3646307]	<0.01	<0.01	1
Stmn2	ENSMUSG00000027500	protein_coding	stathmin-like 2 [Source:MGI Symbol;Acc:MGI:98241]	<0.01	<0.01	1
Gm43766	ENSMUSG00000105868	TEC	predicted gene 43766 [Source:MGI Symbol;Acc:MGI:5663903]	<0.01	0.011	1
Mdk	ENSMUSG00000027239	protein_coding	midkine [Source:MGI Symbol;Acc:MGI:96949]	<0.01	0.047	1
Ramp2	ENSMUSG00000001240	protein_coding	receptor (calcitonin) activity modifying protein 2 [Source:MGI Symbol;Acc:MGI:1859650]	<0.01	<0.01	1
Pqlc3	ENSMUSG00000045679	protein_coding	PQ loop repeat containing [Source:MGI Symbol;Acc:MGI:2444067]	<0.01	0.049	1
Slc26a10	ENSMUSG00000040441	protein_coding	solute carrier family 26, member 10 [Source:MGI Symbol;Acc:MGI:2143920]	<0.01	<0.01	1
Ppih	ENSMUSG00000060288	protein_coding	peptidyl prolyl isomerase H [Source:MGI Symbol;Acc:MGI:106499]	<0.01	<0.01	1
Nphs2	ENSMUSG00000026602	protein_coding	nephrosis 2, podocin [Source:MGI Symbol;Acc:MGI:2157018]	<0.01	<0.01	1
Tmtc2	ENSMUSG00000036019	protein_coding	transmembrane and tetratricopeptide repeat containing 2 [Source:MGI Symbol;Acc:MGI:1914057]	<0.01	0.011	1
Rps15a-ps8	ENSMUSG00000084403	processed_pseudogene	ribosomal protein S15A, pseudogene 8 [Source:MGI Symbol;Acc:MGI:3652112]	<0.01	<0.01	1
Ocel1	ENSMUSG00000002396	protein_coding	occludin/ELL domain containing 1 [Source:MGI Symbol;Acc:MGI:1924340]	<0.01	<0.01	1
Cpd	ENSMUSG00000020841	protein_coding	carboxypeptidase D [Source:MGI Symbol;Acc:MGI:107265]	<0.01	0.026	1
Mroh2a	ENSMUSG00000079429	protein_coding	maestro heat-like repeat family member 2A [Source:MGI Symbol;Acc:MGI:3705228]	<0.01	0.018	1
H2-Q2	ENSMUSG00000091705	protein_coding	histocompatibility 2, Q region locus 2 [Source:MGI Symbol;Acc:MGI:95931]	<0.01	0.017	1
Tctex1d2	ENSMUSG00000014075	protein_coding	Tctex1 domain containing 2 [Source:MGI Symbol;Acc:MGI:1913311]	<0.01	0.047	1
Glb1l3	ENSMUSG00000031966	protein_coding	galactosidase, beta 1 like 3 [Source:MGI Symbol;Acc:MGI:1918143]	<0.01	0.037	1
Bvht	ENSMUSG00000098098	lincRNA	braveheart long non-coding RNA [Source:MGI Symbol;Acc:MGI:5434104]	<0.01	0.032	1
P2rx1	ENSMUSG00000020787	protein_coding	purinergic receptor P2X, ligand-gated ion channel, 1 [Source:MGI Symbol;Acc:MGI:1098235]	<0.01	0.049	1
Gm13251	ENSMUSG00000070605	protein_coding	zinc finger protein 992 [Source:MGI Symbol;Acc:MGI:3700963]	<0.01	<0.01	1
Armc6	ENSMUSG00000002343	protein_coding	armadillo repeat containing 6 [Source:MGI Symbol;Acc:MGI:1924063]	<0.01	<0.01	1
Clmp	ENSMUSG00000032024	protein_coding	CXADR-like membrane protein [Source:MGI Symbol;Acc:MGI:1918816]	<0.01	0.016	1
Ciapin1	ENSMUSG00000031781	protein_coding	cytokine induced apoptosis inhibitor 1 [Source:MGI Symbol;Acc:MGI:1922083]	<0.01	0.023	1
Col15a1	ENSMUSG00000028339	protein_coding	collagen, type XV, alpha 1 [Source:MGI Symbol;Acc:MGI:88449]	<0.01	0.034	1
Tmem2	ENSMUSG00000024754	protein_coding	transmembrane protein 2 [Source:MGI Symbol;Acc:MGI:1890373]	<0.01	0.050	1
Gbp8	ENSMUSG00000034438	protein_coding	guanylate-binding protein 8 [Source:MGI Symbol;Acc:MGI:1923324]	<0.01	<0.01	1
Gm7993	ENSMUSG00000107092	processed_pseudogene	predicted gene 7993 [Source:MGI Symbol;Acc:MGI:3647399]	<0.01	0.039	1
Gabbr1	ENSMUSG00000024462	protein_coding	gamma-aminobutyric acid (GABA) B receptor, 1 [Source:MGI Symbol;Acc:MGI:1860139]	<0.01	<0.01	1
Car15	ENSMUSG00000090236	protein_coding	carbonic anhydrase 15 [Source:MGI Symbol;Acc:MGI:1931324]	<0.01	0.041	1

Gprc5a	ENSMUSG00000046733	protein_coding	G protein-coupled receptor, family C, group 5, member A [Source:MGI Symbol;Acc:MGI:1891250]	<0.01	0.018	1
Slc1a1	ENSMUSG00000024935	protein_coding	solute carrier family 1 (neuronal/epithelial high affinity glutamate transporter, system Xag), member 1 [Source:MGI Symbol;Acc:MGI:105083]	<0.01	0.029	1
Hebp2	ENSMUSG00000019853	protein_coding	heme binding protein 2 [Source:MGI Symbol;Acc:MGI:1860084]	<0.01	<0.01	1
Cntln	ENSMUSG00000038070	protein_coding	centlein, centrosomal protein [Source:MGI Symbol;Acc:MGI:2443104]	<0.01	0.018	1
Gm6047	ENSMUSG00000091293	transcribed_processed_pseudogene	predicted gene 6047 [Source:MGI Symbol;Acc:MGI:3779547]	<0.01	0.015	1
Cdk19	ENSMUSG00000038481	protein_coding	cyclin-dependent kinase 19 [Source:MGI Symbol;Acc:MGI:1925584]	<0.01	0.027	1
Spta1	ENSMUSG00000026532	protein_coding	spectrin alpha, erythrocytic 1 [Source:MGI Symbol;Acc:MGI:98385]	<0.01	<0.01	1
1700112E06Rik	ENSMUSG00000063458	protein_coding	leucine rich melanocyte differentiation associated [Source:MGI Symbol;Acc:MGI:1923883]	<0.01	0.013	1
Iscu	ENSMUSG00000025825	protein_coding	iron-sulfur cluster assembly enzyme [Source:MGI Symbol;Acc:MGI:1913633]	<0.01	0.012	1
Gm11837	ENSMUSG00000086587	antisense_RNA	predicted gene 11837 [Source:MGI Symbol;Acc:MGI:3702175]	<0.01	0.012	1
Cryab	ENSMUSG00000032060	protein_coding	crystallin, alpha B [Source:MGI Symbol;Acc:MGI:88516]	<0.01	0.018	1
Colq	ENSMUSG00000057606	protein_coding	collagen-like tail subunit (single strand of homotrimer) of asymmetric acetylcholinesterase [Source:MGI Symbol;Acc:MGI:1338761]	<0.01	0.049	1
Klhl20	ENSMUSG00000026705	protein_coding	kelch-like 20 [Source:MGI Symbol;Acc:MGI:2444855]	<0.01	<0.01	1
Gm4759	ENSMUSG00000053541	unprocessed_pseudogene	predicted gene 4759 [Source:MGI Symbol;Acc:MGI:3647753]	<0.01	<0.01	1
Myh10	ENSMUSG00000020900	protein_coding	myosin, heavy polypeptide 10, non-muscle [Source:MGI Symbol;Acc:MGI:1930780]	<0.01	0.040	1
Hlf	ENSMUSG00000003949	protein_coding	hepatic leukemia factor [Source:MGI Symbol;Acc:MGI:96108]	<0.01	0.012	1
Nrgn	ENSMUSG00000053310	protein_coding	neurogranin [Source:MGI Symbol;Acc:MGI:1927184]	<0.01	0.020	1
Zfp862-ps	ENSMUSG00000107476	transcribed_unitary_pseudogene	zinc finger protein 862, pseudogene [Source:MGI Symbol;Acc:MGI:1889827]	<0.01	0.018	1
Pknox2	ENSMUSG00000035934	protein_coding	Pbx/knotted 1 homeobox 2 [Source:MGI Symbol;Acc:MGI:2445415]	<0.01	<0.01	1
Serinc5	ENSMUSG00000021703	protein_coding	serine incorporator 5 [Source:MGI Symbol;Acc:MGI:2444223]	<0.01	0.027	1
Impdh1	ENSMUSG00000003500	protein_coding	inosine monophosphate dehydrogenase 1 [Source:MGI Symbol;Acc:MGI:96567]	<0.01	0.019	1
Pmaip1	ENSMUSG00000024521	protein_coding	phorbol-12-myristate-13-acetate-induced protein 1 [Source:MGI Symbol;Acc:MGI:1930146]	<0.01	<0.01	1
Nol3	ENSMUSG00000014776	protein_coding	nucleolar protein 3 (apoptosis repressor with CARD domain) [Source:MGI Symbol;Acc:MGI:1925938]	<0.01	0.012	1
Pcdhb9	ENSMUSG00000051242	protein_coding	protocadherin beta 9 [Source:MGI Symbol;Acc:MGI:2136744]	<0.01	0.037	1
Gm12057	ENSMUSG00000084081	processed_pseudogene	predicted gene 12057 [Source:MGI Symbol;Acc:MGI:3650030]	<0.01	0.046	1
Fzd6	ENSMUSG00000022297	protein_coding	frizzled class receptor 6 [Source:MGI Symbol;Acc:MGI:108474]	<0.01	0.021	1
Gm20707	ENSMUSG00000093594	lincRNA	predicted gene 20707 [Source:MGI Symbol;Acc:MGI:5313154]	<0.01	0.024	1
Upp1	ENSMUSG00000020407	protein_coding	uridine phosphorylase 1 [Source:MGI Symbol;Acc:MGI:1097668]	<0.01	<0.01	1
A4galt	ENSMUSG00000047878	protein_coding	alpha 1,4-galactosyltransferase [Source:MGI Symbol;Acc:MGI:3512453]	<0.01	0.018	1
Gja4	ENSMUSG00000050234	protein_coding	gap junction protein, alpha 4 [Source:MGI Symbol;Acc:MGI:95715]	<0.01	0.030	1
Ankrd13b	ENSMUSG00000037907	protein_coding	ankyrin repeat domain 13b [Source:MGI Symbol;Acc:MGI:2144501]	<0.01	0.025	1
St8sia2	ENSMUSG00000025789	protein_coding	ST8 alpha-N-acetyl-neuraminide alpha-2,8-sialyltransferase 2 [Source:MGI Symbol;Acc:MGI:106020]	<0.01	0.016	1
Rps19-ps3	ENSMUSG00000080059	processed_pseudogene	ribosomal protein S19, pseudogene 3 [Source:MGI Symbol;Acc:MGI:3701125]	<0.01	0.036	1
Podxl2	ENSMUSG00000033152	protein_coding	podocalyxin-like 2 [Source:MGI Symbol;Acc:MGI:2442488]	<0.01	0.023	1
Prkg2	ENSMUSG00000029334	protein_coding	protein kinase, cGMP-dependent, type II [Source:MGI Symbol;Acc:MGI:108173]	<0.01	<0.01	1
Slc39a13	ENSMUSG00000002105	protein_coding	solute carrier family 39 (metal ion transporter), member 13 [Source:MGI Symbol;Acc:MGI:1915677]	<0.01	0.040	1
Cd34	ENSMUSG00000016494	protein_coding	CD34 antigen [Source:MGI Symbol;Acc:MGI:88329]	<0.01	0.044	1
Rpl38-ps2	ENSMUSG00000080921	processed_pseudogene	ribosomal protein L38, pseudogene 2 [Source:MGI Symbol;Acc:MGI:3646625]	<0.01	<0.01	1
Pdhx	ENSMUSG00000010914	protein_coding	pyruvate dehydrogenase complex, component X [Source:MGI Symbol;Acc:MGI:1351627]	<0.01	0.031	1
Enoph1	ENSMUSG00000029326	protein_coding	enolase-phosphatase 1 [Source:MGI Symbol;Acc:MGI:1915120]	<0.01	0.033	1
Gpr179	ENSMUSG00000070337	protein_coding	G protein-coupled receptor 179 [Source:MGI Symbol;Acc:MGI:2443409]	<0.01	0.012	1
Fam107a	ENSMUSG00000021750	protein_coding	family with sequence similarity 107, member A [Source:MGI Symbol;Acc:MGI:3041256]	<0.01	<0.01	1
Gsg1l	ENSMUSG00000046182	protein_coding	GSG1-like [Source:MGI Symbol;Acc:MGI:2685483]	<0.01	<0.01	1
Lrrc3b	ENSMUSG00000045201	protein_coding	leucine rich repeat containing 3B [Source:MGI Symbol;Acc:MGI:2384996]	<0.01	<0.01	1
Mgll	ENSMUSG00000033174	protein_coding	monoglyceride lipase [Source:MGI Symbol;Acc:MGI:1346042]	<0.01	<0.01	1
Chst1	ENSMUSG00000027221	protein_coding	carbohydrate (keratan sulfate Gal-6) sulfotransferase 1 [Source:MGI Symbol;Acc:MGI:1924219]	<0.01	0.028	1
E130311K13Rik	ENSMUSG00000048581	protein_coding	RIKEN cDNA E130311K13 gene [Source:MGI Symbol;Acc:MGI:3607716]	<0.01	<0.01	1
Il3ra	ENSMUSG00000068758	protein_coding	interleukin 3 receptor, alpha chain [Source:MGI Symbol;Acc:MGI:96553]	<0.01	0.013	1
S100a13	ENSMUSG00000042312	protein_coding	S100 calcium binding protein A13 [Source:MGI Symbol;Acc:MGI:109581]	<0.01	0.035	1
Sox7	ENSMUSG00000063060	protein_coding	SRY (sex determining region Y)-box 7 [Source:MGI Symbol;Acc:MGI:98369]	<0.01	0.017	1
Plb1	ENSMUSG00000029134	protein_coding	phospholipase B1 [Source:MGI Symbol;Acc:MGI:1922406]	<0.01	0.041	1
Samd8	ENSMUSG00000021770	protein_coding	sterile alpha motif domain containing 8 [Source:MGI Symbol;Acc:MGI:1914880]	<0.01	0.036	1
Krt79	ENSMUSG00000061397	protein_coding	keratin 79 [Source:MGI Symbol;Acc:MGI:2385030]	<0.01	<0.01	1
Ctsk	ENSMUSG00000028111	protein_coding	cathepsin K [Source:MGI Symbol;Acc:MGI:107823]	<0.01	0.022	1
Copz2	ENSMUSG00000018672	protein_coding	coatomer protein complex, subunit zeta 2 [Source:MGI Symbol;Acc:MGI:1929008]	<0.01	0.035	1
Tbx3os1	ENSMUSG00000087516	antisense_RNA	T-box 3, opposite strand 1 [Source:MGI Symbol;Acc:MGI:3780472]	<0.01	<0.01	1
Nrros	ENSMUSG00000052384	protein_coding	negative regulator of reactive oxygen species [Source:MGI Symbol;Acc:MGI:2445095]	<0.01	<0.01	1
Tvp23bos	ENSMUSG00000086677	antisense_RNA	trans-golgi network vesicle protein 23B, opposite strand [Source:MGI Symbol;Acc:MGI:3649886]	<0.01	0.030	1
Gm5566	ENSMUSG00000080002	processed_pseudogene	predicted pseudogene 5566 [Source:MGI Symbol;Acc:MGI:3648428]	<0.01	<0.01	1
Arhgap40	ENSMUSG00000074625	protein_coding	Rho GTPase activating protein 40 [Source:MGI Symbol;Acc:MGI:3649852]	<0.01	<0.01	1
Lilra5	ENSMUSG00000070873	protein_coding	leukocyte immunoglobulin-like receptor, subfamily A (with TM domain), member 5 [Source:MGI Symbol;Acc:MGI:3647196]	<0.01	0.015	1
Dpysl5	ENSMUSG00000029168	protein_coding	dihydropyrimidinase-like 5 [Source:MGI Symbol;Acc:MGI:1929772]	<0.01	0.028	1
Hemk1	ENSMUSG00000032579	protein_coding	HemK methyltransferase family member 1 [Source:MGI Symbol;Acc:MGI:1916786]	<0.01	0.022	1
Rpl15-ps2	ENSMUSG00000098915	processed_pseudogene	ribosomal protein L15, pseudogene 2 [Source:MGI Symbol;Acc:MGI:3648255]	<0.01	<0.01	1
Ubxn8	ENSMUSG00000052906	protein_coding	UBX domain protein 8 [Source:MGI Symbol;Acc:MGI:1337129]	<0.01	0.027	1
Nrcam	ENSMUSG00000020598	protein_coding	neuronal cell adhesion molecule [Source:MGI Symbol;Acc:MGI:104750]	<0.01	<0.01	1
Rpl38	ENSMUSG00000057322	protein_coding	ribosomal protein L38 [Source:MGI Symbol;Acc:MGI:1914921]	<0.01	<0.01	1
Ccnyl1	ENSMUSG00000070871	protein_coding	cyclin Y-like 1 [Source:MGI Symbol;Acc:MGI:2138614]	<0.01	0.016	1
Gatm	ENSMUSG00000027199	protein_coding	glycine amidinotransferase (L-arginine:glycine amidinotransferase) [Source:MGI Symbol;Acc:MGI:1914342]	<0.01	<0.01	1
C1s2	ENSMUSG00000079343	protein_coding	complement component 1, s subcomponent 2 [Source:MGI Symbol;Acc:MGI:3644269]	<0.01	<0.01	1
Dusp12	ENSMUSG00000026659	protein_coding	dual specificity phosphatase 12 [Source:MGI Symbol;Acc:MGI:1890614]	<0.01	0.010	1
S100a6	ENSMUSG00000001025	protein_coding	S100 calcium binding protein A6 (calcyclin) [Source:MGI Symbol;Acc:MGI:1339467]	<0.01	0.046	1
Cd300c	ENSMUSG00000058728	protein_coding	CD300C molecule [Source:MGI Symbol;Acc:MGI:3032626]	<0.01	<0.01	1
Ddi2	ENSMUSG00000078515	protein_coding	DNA-damage inducible protein 2 [Source:MGI Symbol;Acc:MGI:1917244]	<0.01	0.034	1
Gm8116	ENSMUSG00000059422	processed_pseudogene	predicted gene 8116 [Source:MGI Symbol;Acc:MGI:3648797]	<0.01	0.014	1
BC022687	ENSMUSG00000037594	protein_coding	cDNA sequence BC022687 [Source:MGI Symbol;Acc:MGI:2443738]	<0.01	<0.01	1
Nipa1	ENSMUSG00000047037	protein_coding	non imprinted in Prader-Willi/Angelman syndrome 1 homolog (human) [Source:MGI Symbol;Acc:MGI:2442058]	<0.01	<0.01	1
Ifi202b	ENSMUSG00000026535	protein_coding	interferon activated gene 202B [Source:MGI Symbol;Acc:MGI:1347083]	<0.01	<0.01	1
Smap1	ENSMUSG00000026155	protein_coding	small ArfGAP 1 [Source:MGI Symbol;Acc:MGI:2138261]	<0.01	0.035	1
Gm16340	ENSMUSG00000090222	unprocessed_pseudogene	interferon activated gene 203, pseudogene [Source:MGI Symbol;Acc:MGI:3840117]	<0.01	0.041	1

Bmp4	ENSMUSG00000021835	protein_coding	bone morphogenetic protein 4 [Source:MGI Symbol;Acc:MGI:88180]	<0.01	0.012	1
Creb3	ENSMUSG00000028466	protein_coding	cAMP responsive element binding protein 3 [Source:MGI Symbol;Acc:MGI:99946]	<0.01	0.026	1
2610305D13Rik	ENSMUSG00000066000	protein_coding	zinc finger protein 979 [Source:MGI Symbol;Acc:MGI:2148252]	<0.01	<0.01	1
Tmem117	ENSMUSG00000063296	protein_coding	transmembrane protein 117 [Source:MGI Symbol;Acc:MGI:2444580]	<0.01	<0.01	1
Cyp2b10	ENSMUSG00000030483	protein_coding	cytochrome P450, family 2, subfamily b, polypeptide 10 [Source:MGI Symbol;Acc:MGI:88598]	<0.01	0.026	1
Ptprd	ENSMUSG00000028399	protein_coding	protein tyrosine phosphatase, receptor type, D [Source:MGI Symbol;Acc:MGI:97812]	<0.01	0.026	1
Tor3a	ENSMUSG00000060519	protein_coding	torsin family 3, member A [Source:MGI Symbol;Acc:MGI:1353652]	<0.01	0.045	1
Tnxa	ENSMUSG00000092200	unprocessed_pseudogene	tenascin XA (pseudogene) [Source:MGI Symbol;Acc:MGI:2148489]	<0.01	0.013	1
Tcte2	ENSMUSG00000038347	protein_coding	t-complex-associated testis expressed 2 [Source:MGI Symbol;Acc:MGI:98641]	<0.01	0.014	1
Dynlt1b	ENSMUSG00000096255	protein_coding	dynein light chain Tctex-type 1B [Source:MGI Symbol;Acc:MGI:98643]	<0.01	<0.01	1
Gm26862	ENSMUSG00000097644	sense_overlapping	predicted gene, 26862 [Source:MGI Symbol;Acc:MGI:5477356]	<0.01	0.012	1
Adgrl2	ENSMUSG00000028184	protein_coding	adhesion G protein-coupled receptor L2 [Source:MGI Symbol;Acc:MGI:2139714]	<0.01	0.036	1
Gm5548	ENSMUSG00000045952	processed_pseudogene	predicted pseudogene 5548 [Source:MGI Symbol;Acc:MGI:3644344]	<0.01	0.029	1
Cpsf4	ENSMUSG00000029625	protein_coding	cleavage and polyadenylation specific factor 4 [Source:MGI Symbol;Acc:MGI:1861602]	<0.01	0.011	1
Gcgr	ENSMUSG00000025127	protein_coding	glucagon receptor [Source:MGI Symbol;Acc:MGI:99572]	<0.01	0.041	1
9330159F19Rik	ENSMUSG00000004360	protein_coding	RIKEN cDNA 9330159F19 gene [Source:MGI Symbol;Acc:MGI:3036239]	<0.01	<0.01	1
Ppp1r3a	ENSMUSG00000042717	protein_coding	protein phosphatase 1, regulatory (inhibitor) subunit 3A [Source:MGI Symbol;Acc:MGI:2153588]	<0.01	<0.01	1
Rab3gap2	ENSMUSG00000039318	protein_coding	RAB3 GTPase activating protein subunit 2 [Source:MGI Symbol;Acc:MGI:1916043]	<0.01	0.043	1
Gm13212	ENSMUSG00000078502	protein_coding	predicted gene 13212 [Source:MGI Symbol;Acc:MGI:3651014]	<0.01	<0.01	1
Gm13375	ENSMUSG00000075514	antisense_RNA	predicted gene 13375 [Source:MGI Symbol;Acc:MGI:3649913]	<0.01	0.019	1
Palm3	ENSMUSG00000047986	protein_coding	paralemmin 3 [Source:MGI Symbol;Acc:MGI:1921587]	<0.01	0.034	1
Add2	ENSMUSG00000030000	protein_coding	adducin 2 (beta) [Source:MGI Symbol;Acc:MGI:87919]	<0.01	<0.01	1
Cdkn2c	ENSMUSG00000028551	protein_coding	cyclin-dependent kinase inhibitor 2C (p18, inhibits CDK4) [Source:MGI Symbol;Acc:MGI:105388]	<0.01	<0.01	1
Per3	ENSMUSG00000028957	protein_coding	period circadian clock 3 [Source:MGI Symbol;Acc:MGI:1277134]	<0.01	<0.01	1
Gm13394	ENSMUSG00000083773	processed_pseudogene	predicted gene 13394 [Source:MGI Symbol;Acc:MGI:3651848]	<0.01	<0.01	1
Gm3716	ENSMUSG00000105402	lincRNA	predicted gene 3716 [Source:MGI Symbol;Acc:MGI:3781892]	<0.01	0.033	1
Rbpms2	ENSMUSG00000032387	protein_coding	RNA binding protein with multiple splicing 2 [Source:MGI Symbol;Acc:MGI:1919223]	<0.01	0.042	1
Emp1	ENSMUSG00000030208	protein_coding	epithelial membrane protein 1 [Source:MGI Symbol;Acc:MGI:107941]	<0.01	0.011	1
Hoxb2	ENSMUSG00000075588	protein_coding	homeobox B2 [Source:MGI Symbol;Acc:MGI:96183]	<0.01	0.042	1
Fam73a	ENSMUSG00000054942	protein_coding	mitoguardin 1 [Source:MGI Symbol;Acc:MGI:1924567]	<0.01	0.028	1
Serf2	ENSMUSG00000074884	protein_coding	small EDRK-rich factor 2 [Source:MGI Symbol;Acc:MGI:1337041]	<0.01	0.035	1
Plpp7	ENSMUSG00000051373	protein_coding	phospholipid phosphatase 7 (inactive) [Source:MGI Symbol;Acc:MGI:2445183]	<0.01	0.050	1
Nt5e	ENSMUSG00000032420	protein_coding	5' nucleotidase, ecto [Source:MGI Symbol;Acc:MGI:99782]	<0.01	0.037	1
Dynlt1-ps1	ENSMUSG00000082691	processed_pseudogene	dynein light chain Tctex-type 1, pseudogene 1 [Source:MGI Symbol;Acc:MGI:3642625]	<0.01	<0.01	1
Reep1	ENSMUSG00000052852	protein_coding	receptor accessory protein 1 [Source:MGI Symbol;Acc:MGI:1098827]	<0.01	0.028	1
Pla2g12b	ENSMUSG00000009646	protein_coding	phospholipase A2, group XIIB [Source:MGI Symbol;Acc:MGI:1917086]	<0.01	0.021	1
Atp10d	ENSMUSG00000046808	polymorphic_pseudogene	ATPase, class V, type 10D [Source:MGI Symbol;Acc:MGI:2450125]	<0.01	<0.01	1
Gm16556	ENSMUSG00000090192	processed_transcript	predicted gene 16556 [Source:MGI Symbol;Acc:MGI:4414976]	<0.01	<0.01	1
Dok1	ENSMUSG00000068335	protein_coding	docking protein 1 [Source:MGI Symbol;Acc:MGI:893587]	<0.01	0.023	1
Ctdspl	ENSMUSG00000047409	protein_coding	CTD (carboxy-terminal domain, RNA polymerase II, polypeptide A) small phosphatase-like [Source:MGI Symbol;Acc:MGI:1916524]	<0.01	0.014	1
Gm15703	ENSMUSG00000083890	processed_pseudogene	predicted gene 15703 [Source:MGI Symbol;Acc:MGI:3783143]	<0.01	0.023	1
Msx1	ENSMUSG00000048450	protein_coding	msh homeobox 1 [Source:MGI Symbol;Acc:MGI:97168]	<0.01	0.049	1
Efnb2	ENSMUSG00000001300	protein_coding	ephrin B2 [Source:MGI Symbol;Acc:MGI:105097]	<0.01	0.035	1
Slc25a34	ENSMUSG00000040740	protein_coding	solute carrier family 25, member 34 [Source:MGI Symbol;Acc:MGI:2686215]	<0.01	0.049	1
Igsf23	ENSMUSG00000040498	protein_coding	immunoglobulin superfamily, member 23 [Source:MGI Symbol;Acc:MGI:1917330]	<0.01	<0.01	1
Crip1	ENSMUSG00000006360	protein_coding	cysteine-rich protein 1 (intestinal) [Source:MGI Symbol;Acc:MGI:88501]	<0.01	0.030	1
Stc1	ENSMUSG00000014813	protein_coding	stanniocalcin 1 [Source:MGI Symbol;Acc:MGI:109131]	<0.01	0.016	1
Depdc7	ENSMUSG00000027173	protein_coding	DEP domain containing 7 [Source:MGI Symbol;Acc:MGI:2139258]	<0.01	0.035	1
Mettl21e	ENSMUSG00000046828	protein_coding	methyltransferase like 21E [Source:MGI Symbol;Acc:MGI:2685837]	<0.01	<0.01	1
Mapkbp1	ENSMUSG00000033902	protein_coding	mitogen-activated protein kinase binding protein 1 [Source:MGI Symbol;Acc:MGI:1347004]	<0.01	<0.01	1
Zfp652os	ENSMUSG00000086191	antisense_RNA	zinc finger protein 652, opposite strand [Source:MGI Symbol;Acc:MGI:3044900]	<0.01	0.021	1
Tnfrsf11b	ENSMUSG00000063727	protein_coding	tumor necrosis factor receptor superfamily, member 11b (osteoprotegerin) [Source:MGI Symbol;Acc:MGI:109587]	<0.01	0.043	1
Fbp2	ENSMUSG00000021456	protein_coding	fructose bisphosphatase 2 [Source:MGI Symbol;Acc:MGI:95491]	<0.01	<0.01	1
Gm6969	ENSMUSG00000066553	processed_pseudogene	predicted pseudogene 6969 [Source:MGI Symbol;Acc:MGI:3645320]	<0.01	0.020	1
Eya2	ENSMUSG00000017897	protein_coding	EYA transcriptional coactivator and phosphatase 2 [Source:MGI Symbol;Acc:MGI:109341]	<0.01	0.049	1
Agpat4	ENSMUSG00000023827	protein_coding	1-acylglycerol-3-phosphate O-acyltransferase 4 (lysophosphatidic acid acyltransferase, delta) [Source:MGI Symbol;Acc:MGI:1915512]	<0.01	0.043	1
Adrb3	ENSMUSG00000031489	protein_coding	adrenergic receptor, beta 3 [Source:MGI Symbol;Acc:MGI:87939]	<0.01	0.017	1
Hba-a1	ENSMUSG00000069919	protein_coding	hemoglobin alpha, adult chain 1 [Source:MGI Symbol;Acc:MGI:96015]	<0.01	<0.01	1
Ramp3	ENSMUSG00000041046	protein_coding	receptor (calcitonin) activity modifying protein 3 [Source:MGI Symbol;Acc:MGI:1860292]	<0.01	<0.01	1
Morn4	ENSMUSG00000049670	protein_coding	MORN repeat containing 4 [Source:MGI Symbol;Acc:MGI:2449568]	<0.01	<0.01	1
Hk3	ENSMUSG00000025877	protein_coding	hexokinase 3 [Source:MGI Symbol;Acc:MGI:2670962]	<0.01	0.028	1
Gm11128	ENSMUSG00000079495	protein_coding	N-acetyltransferase 8 (GCN5-related) family member 6 [Source:MGI Symbol;Acc:MGI:3779382]	<0.01	<0.01	1
Gm26619	ENSMUSG00000097346	lincRNA	predicted gene, 26619 [Source:MGI Symbol;Acc:MGI:5477113]	<0.01	<0.01	1
Pcna-ps2	ENSMUSG00000067608	protein_coding	proliferating cell nuclear antigen pseudogene 2 [Source:MGI Symbol;Acc:MGI:97505]	<0.01	<0.01	1
Gm8719	ENSMUSG00000107896	processed_pseudogene	predicted pseudogene 8719 [Source:MGI Symbol;Acc:MGI:3647551]	<0.01	<0.01	1
Stc2	ENSMUSG00000020303	protein_coding	stanniocalcin 2 [Source:MGI Symbol;Acc:MGI:1316731]	<0.01	0.031	1
Fkrp	ENSMUSG00000048920	protein_coding	fukutin related protein [Source:MGI Symbol;Acc:MGI:2447586]	<0.01	0.041	1
Smarca2	ENSMUSG00000024921	protein_coding	SWI/SNF related, matrix associated, actin dependent regulator of chromatin, subfamily a, member 2 [Source:MGI Symbol;Acc:MGI:99603]	<0.01	0.014	1
E330011M16Rik	ENSMUSG00000103030	TEC	RIKEN cDNA E330011M16 gene [Source:MGI Symbol;Acc:MGI:3704186]	<0.01	0.043	1
Cd1d2	ENSMUSG00000041750	polymorphic_pseudogene	CD1d2 antigen [Source:MGI Symbol;Acc:MGI:107675]	<0.01	<0.01	1
Gm43194	ENSMUSG00000106139	lincRNA	predicted gene, 30648 [Source:MGI Symbol;Acc:MGI:5589807]	<0.01	0.041	1
Gm26825	ENSMUSG00000097554	lincRNA	predicted gene, 26825 [Source:MGI Symbol;Acc:MGI:5477319]	<0.01	<0.01	1
Spp1	ENSMUSG00000029304	protein_coding	secreted phosphoprotein 1 [Source:MGI Symbol;Acc:MGI:98389]	<0.01	<0.01	1
Slc7a8	ENSMUSG00000022180	protein_coding	solute carrier family 7 (cationic amino acid transporter, y+ system), member 8 [Source:MGI Symbol;Acc:MGI:1355323]	<0.01	0.030	1
Acaa1b	ENSMUSG00000010651	protein_coding	acetyl-Coenzyme A acyltransferase 1B [Source:MGI Symbol;Acc:MGI:3605455]	<0.01	0.029	1
Reln	ENSMUSG00000042453	protein_coding	reelin [Source:MGI Symbol;Acc:MGI:103022]	<0.01	0.012	1
Fah	ENSMUSG00000030630	protein_coding	fumarylacetoacetate hydrolase [Source:MGI Symbol;Acc:MGI:95482]	<0.01	0.026	1
Hsd17b14	ENSMUSG00000030825	protein_coding	hydroxysteroid (17-beta) dehydrogenase 14 [Source:MGI Symbol;Acc:MGI:1913315]	<0.01	<0.01	1
Anxa3	ENSMUSG00000029484	protein_coding	annexin A3 [Source:MGI Symbol;Acc:MGI:1201378]	<0.01	<0.01	1
H2-K2	ENSMUSG00000067203	unprocessed_pseudogene	histocompatibility 2, K region locus 2 [Source:MGI Symbol;Acc:MGI:95906]	<0.01	<0.01	1

Dach1	ENSMUSG00000055639	protein_coding	dachshund 1 (Drosophila) [Source:MGI Symbol;Acc:MGI:1277991]	<0.01	0.040	1
Hapln3	ENSMUSG00000030606	protein_coding	hyaluronan and proteoglycan link protein 3 [Source:MGI Symbol;Acc:MGI:1914916]	<0.01	0.012	1
Gpr182	ENSMUSG00000058396	protein_coding	G protein-coupled receptor 182 [Source:MGI Symbol;Acc:MGI:109545]	<0.01	0.044	1
Fryl	ENSMUSG00000070733	protein_coding	FRY like transcription coactivator [Source:MGI Symbol;Acc:MGI:1919563]	<0.01	0.019	1
Coq5	ENSMUSG00000041733	protein_coding	coenzyme Q5 methyltransferase [Source:MGI Symbol;Acc:MGI:1098643]	<0.01	0.049	1
Rxfp4	ENSMUSG00000049741	protein_coding	relaxin family peptide receptor 4 [Source:MGI Symbol;Acc:MGI:2182926]	<0.01	0.026	1
Zfp69	ENSMUSG00000064141	protein_coding	zinc finger protein 69 [Source:MGI Symbol;Acc:MGI:107794]	<0.01	<0.01	1
Slx4	ENSMUSG00000039738	protein_coding	SLX4 structure-specific endonuclease subunit homolog (S. cerevisiae) [Source:MGI Symbol;Acc:MGI:106299]	<0.01	0.050	1
Ppp1r37	ENSMUSG00000051403	protein_coding	protein phosphatase 1, regulatory subunit 37 [Source:MGI Symbol;Acc:MGI:2687042]	<0.01	0.031	1
Rgs5	ENSMUSG00000026678	protein_coding	regulator of G-protein signaling 5 [Source:MGI Symbol;Acc:MGI:1098434]	<0.01	0.014	1
Nudt2	ENSMUSG00000028443	protein_coding	nudix (nucleoside diphosphate linked moiety X)-type motif 2 [Source:MGI Symbol;Acc:MGI:1913651]	<0.01	0.034	1
Ttyh2	ENSMUSG00000034714	protein_coding	tweety family member 2 [Source:MGI Symbol;Acc:MGI:2157091]	<0.01	<0.01	1
Gm13154	ENSMUSG00000065999	protein_coding	zinc finger protein 985 [Source:MGI Symbol;Acc:MGI:3651986]	<0.01	0.047	1
Gm17122	ENSMUSG00000090458	antisense_RNA	predicted gene 17122 [Source:MGI Symbol;Acc:MGI:4937949]	<0.01	0.029	1
H2-Q4	ENSMUSG00000035929	protein_coding	histocompatibility 2, Q region locus 4 [Source:MGI Symbol;Acc:MGI:95933]	<0.01	<0.01	1
Gm4477	ENSMUSG00000089694	protein_coding	N-acetyltransferase 8 (GCN5-related) family member 7 [Source:MGI Symbol;Acc:MGI:3782661]	<0.01	0.029	1
H2-T24	ENSMUSG00000053835	protein_coding	histocompatibility 2, T region locus 24 [Source:MGI Symbol;Acc:MGI:95958]	<0.01	0.013	1
Alpk1	ENSMUSG00000028028	protein_coding	alpha-kinase 1 [Source:MGI Symbol;Acc:MGI:1918731]	<0.01	0.037	1
Hopx	ENSMUSG00000059325	protein_coding	HOP homeobox [Source:MGI Symbol;Acc:MGI:1916782]	<0.01	0.011	1
Steap3	ENSMUSG00000026389	protein_coding	STEAP family member 3 [Source:MGI Symbol;Acc:MGI:1915678]	<0.01	<0.01	1
D330041H03Rik	ENSMUSG00000073437	processed_transcript	RIKEN cDNA D330041H03 gene [Source:MGI Symbol;Acc:MGI:3603827]	<0.01	0.013	1
Cdk11b	ENSMUSG00000029062	protein_coding	cyclin-dependent kinase 11B [Source:MGI Symbol;Acc:MGI:88353]	<0.01	0.021	1
4930526A20Rik	ENSMUSG00000085521	transcribed_processed_pseudogene	RIKEN cDNA 4930526A20 gene [Source:MGI Symbol;Acc:MGI:3612449]	<0.01	0.035	1
Gm9825	ENSMUSG00000096403	processed_pseudogene	predicted gene 9825 [Source:MGI Symbol;Acc:MGI:3708729]	<0.01	<0.01	1
Tacc2	ENSMUSG00000030852	protein_coding	transforming, acidic coiled-coil containing protein 2 [Source:MGI Symbol;Acc:MGI:1928899]	<0.01	0.020	1
G730003C15Rik	ENSMUSG00000097573	antisense_RNA	RIKEN cDNA G730003C15 gene [Source:MGI Symbol;Acc:MGI:3641700]	<0.01	0.019	1
A530016L24Rik	ENSMUSG00000043122	protein_coding	RIKEN cDNA A530016L24 gene [Source:MGI Symbol;Acc:MGI:2443020]	<0.01	<0.01	1
Slc25a29	ENSMUSG00000021265	protein_coding	solute carrier family 25 (mitochondrial carrier, palmitoylcarnitine transporter), member 29 [Source:MGI Symbol;Acc:MGI:2444911]	<0.01	<0.01	1
Lrrn3	ENSMUSG00000036295	protein_coding	leucine rich repeat protein 3, neuronal [Source:MGI Symbol;Acc:MGI:106036]	<0.01	<0.01	1
Gm15852	ENSMUSG00000089990	processed_transcript	predicted gene 15852 [Source:MGI Symbol;Acc:MGI:3801733]	<0.01	0.045	1
Ptprr	ENSMUSG00000020151	protein_coding	protein tyrosine phosphatase, receptor type, R [Source:MGI Symbol;Acc:MGI:109559]	<0.01	<0.01	1
Gm10116	ENSMUSG00000062382	protein_coding	ferritin light polypeptide 1, pseudogene 1 [Source:MGI Symbol;Acc:MGI:3779109]	<0.01	<0.01	1
1700020N18Rik	ENSMUSG00000100253	lincRNA	RIKEN cDNA 1700020N18 gene [Source:MGI Symbol;Acc:MGI:1914336]	<0.01	0.047	1
Gm37192	ENSMUSG00000102184	TEC	predicted gene, 37192 [Source:MGI Symbol;Acc:MGI:5610420]	<0.01	<0.01	1
Gm37660	ENSMUSG00000104467	TEC	predicted gene, 37660 [Source:MGI Symbol;Acc:MGI:5610888]	<0.01	0.017	1
Rnls	ENSMUSG00000071573	protein_coding	renalase, FAD-dependent amine oxidase [Source:MGI Symbol;Acc:MGI:1915045]	<0.01	0.019	1
Ccdc184	ENSMUSG00000029875	protein_coding	coiled-coil domain containing 184 [Source:MGI Symbol;Acc:MGI:2146066]	<0.01	<0.01	1
Sh3d21	ENSMUSG00000073758	protein_coding	SH3 domain containing 21 [Source:MGI Symbol;Acc:MGI:1914188]	<0.01	0.015	1
5930430L01Rik	ENSMUSG00000106951	lincRNA	RIKEN cDNA 5930430L01 gene [Source:MGI Symbol;Acc:MGI:2443110]	<0.01	<0.01	1
Prkar2a	ENSMUSG00000032601	protein_coding	protein kinase, cAMP dependent regulatory, type II alpha [Source:MGI Symbol;Acc:MGI:108025]	<0.01	0.023	1
Gm10093	ENSMUSG00000061062	protein_coding	predicted pseudogene 10093 [Source:MGI Symbol;Acc:MGI:3704479]	<0.01	<0.01	1
Slc25a42	ENSMUSG00000002346	protein_coding	solute carrier family 25, member 42 [Source:MGI Symbol;Acc:MGI:1920345]	<0.01	0.027	1
Cables1	ENSMUSG00000040957	protein_coding	CDK5 and Abl enzyme substrate 1 [Source:MGI Symbol;Acc:MGI:1927065]	<0.01	0.018	1
Magi1	ENSMUSG00000045095	protein_coding	membrane associated guanylate kinase, WW and PDZ domain containing 1 [Source:MGI Symbol;Acc:MGI:1203522]	<0.01	0.049	1
Gm43573	ENSMUSG00000104786	antisense_RNA	predicted gene 43573 [Source:MGI Symbol;Acc:MGI:5663710]	<0.01	0.022	1
Ubc	ENSMUSG00000008348	protein_coding	ubiquitin C [Source:MGI Symbol;Acc:MGI:98889]	<0.01	<0.01	1
Msl3l2	ENSMUSG00000047669	protein_coding	male-specific lethal 3-like 2 (Drosophila) [Source:MGI Symbol;Acc:MGI:1920640]	<0.01	0.020	1
Pgm5	ENSMUSG00000041731	protein_coding	phosphoglucomutase 5 [Source:MGI Symbol;Acc:MGI:1925668]	<0.01	<0.01	1
Nup35	ENSMUSG00000026999	protein_coding	nucleoporin 35 [Source:MGI Symbol;Acc:MGI:1916732]	<0.01	0.032	1
Cox4i2	ENSMUSG00000009876	protein_coding	cytochrome c oxidase subunit 4I2 [Source:MGI Symbol;Acc:MGI:2135755]	<0.01	<0.01	1
Gm17060	ENSMUSG00000090624	processed_pseudogene	predicted gene 17060 [Source:MGI Symbol;Acc:MGI:4937887]	<0.01	<0.01	1
Btbd9	ENSMUSG00000062202	protein_coding	BTB (POZ) domain containing 9 [Source:MGI Symbol;Acc:MGI:1916625]	<0.01	0.013	1
Clic3	ENSMUSG00000015093	protein_coding	chloride intracellular channel 3 [Source:MGI Symbol;Acc:MGI:1916704]	<0.01	0.046	1
Rock2	ENSMUSG00000020580	protein_coding	Rho-associated coiled-coil containing protein kinase 2 [Source:MGI Symbol;Acc:MGI:107926]	<0.01	0.032	1
Fabp1	ENSMUSG00000054422	protein_coding	fatty acid binding protein 1, liver [Source:MGI Symbol;Acc:MGI:95479]	<0.01	<0.01	1
Plk4	ENSMUSG00000025758	protein_coding	polo-like kinase 4 [Source:MGI Symbol;Acc:MGI:101783]	<0.01	<0.01	1
Pparg	ENSMUSG00000000440	protein_coding	peroxisome proliferator activated receptor gamma [Source:MGI Symbol;Acc:MGI:97747]	<0.01	0.042	1
Ech1	ENSMUSG00000053898	protein_coding	enoyl coenzyme A hydratase 1, peroxisomal [Source:MGI Symbol;Acc:MGI:1858208]	<0.01	0.043	1
Gpr155	ENSMUSG00000041762	protein_coding	G protein-coupled receptor 155 [Source:MGI Symbol;Acc:MGI:1915776]	<0.01	0.039	1
Gm7292	ENSMUSG00000104222	lincRNA	predicted gene 7292 [Source:MGI Symbol;Acc:MGI:3645786]	<0.01	<0.01	1
Tstd3	ENSMUSG00000028251	protein_coding	thiosulfate sulfurtransferase (rhodanese)-like domain containing 3 [Source:MGI Symbol;Acc:MGI:1924282]	<0.01	0.011	1
Pdpn	ENSMUSG00000028583	protein_coding	podoplanin [Source:MGI Symbol;Acc:MGI:103098]	<0.01	<0.01	1
Papln	ENSMUSG00000021223	protein_coding	papilin, proteoglycan-like sulfated glycoprotein [Source:MGI Symbol;Acc:MGI:2386139]	<0.01	0.039	1
Stk32c	ENSMUSG00000015981	protein_coding	serine/threonine kinase 32C [Source:MGI Symbol;Acc:MGI:2385336]	<0.01	<0.01	1
Spata20	ENSMUSG00000020867	protein_coding	spermatogenesis associated 20 [Source:MGI Symbol;Acc:MGI:2183449]	<0.01	0.012	1
Zfp451	ENSMUSG00000042197	protein_coding	zinc finger protein 451 [Source:MGI Symbol;Acc:MGI:2137896]	<0.01	0.026	1
Epha1	ENSMUSG00000029859	protein_coding	Eph receptor A1 [Source:MGI Symbol;Acc:MGI:107381]	<0.01	0.011	1
Pla1a	ENSMUSG00000002847	protein_coding	phospholipase A1 member A [Source:MGI Symbol;Acc:MGI:1934677]	<0.01	0.019	1
Catsper2	ENSMUSG00000033486	protein_coding	cation channel, sperm associated 2 [Source:MGI Symbol;Acc:MGI:2387404]	<0.01	0.049	1
Casp9	ENSMUSG00000028914	protein_coding	caspase 9 [Source:MGI Symbol;Acc:MGI:1277950]	<0.01	0.041	1
Pbx3	ENSMUSG00000038718	protein_coding	pre B cell leukemia homeobox 3 [Source:MGI Symbol;Acc:MGI:97496]	<0.01	0.026	1
Myh7	ENSMUSG00000053093	protein_coding	myosin, heavy polypeptide 7, cardiac muscle, beta [Source:MGI Symbol;Acc:MGI:2155600]	<0.01	0.026	1
Procr	ENSMUSG00000027611	protein_coding	protein C receptor, endothelial [Source:MGI Symbol;Acc:MGI:104596]	<0.01	<0.01	1
Ppp1r15a	ENSMUSG00000040435	protein_coding	protein phosphatase 1, regulatory (inhibitor) subunit 15A [Source:MGI Symbol;Acc:MGI:1927072]	<0.01	0.048	1
Lysmd1	ENSMUSG00000053769	protein_coding	LysM, putative peptidoglycan-binding, domain containing 1 [Source:MGI Symbol;Acc:MGI:1919409]	<0.01	0.019	1
Rspo4	ENSMUSG00000032852	protein_coding	R-spondin 4 [Source:MGI Symbol;Acc:MGI:1924467]	<0.01	<0.01	1
Wif1	ENSMUSG00000020218	protein_coding	Wnt inhibitory factor 1 [Source:MGI Symbol;Acc:MGI:1344332]	<0.01	0.015	1
Irs1	ENSMUSG00000055980	protein_coding	insulin receptor substrate 1 [Source:MGI Symbol;Acc:MGI:99454]	<0.01	0.033	1
Gm20632	ENSMUSG00000093577	antisense_RNA	predicted gene 20632 [Source:MGI Symbol;Acc:MGI:5313079]	<0.01	<0.01	1
Rgs4	ENSMUSG00000038530	protein_coding	regulator of G-protein signaling 4 [Source:MGI Symbol;Acc:MGI:108409]	<0.01	0.025	1

Adora3	ENSMUSG00000000562	protein_coding	adenosine A3 receptor [Source:MGI Symbol;Acc:MGI:104847]	<0.01	0.050	1
Plcb1	ENSMUSG00000051177	protein_coding	phospholipase C, beta 1 [Source:MGI Symbol;Acc:MGI:97613]	<0.01	0.026	1
Tmod1	ENSMUSG00000028328	protein_coding	tropomodulin 1 [Source:MGI Symbol;Acc:MGI:98775]	<0.01	0.012	1
Map2	ENSMUSG00000015222	protein_coding	microtubule-associated protein 2 [Source:MGI Symbol;Acc:MGI:97175]	<0.01	0.038	1
Sync	ENSMUSG00000001333	protein_coding	syncoilin [Source:MGI Symbol;Acc:MGI:1916078]	<0.01	0.046	1
Rtkn2	ENSMUSG00000037846	protein_coding	rhotekin 2 [Source:MGI Symbol;Acc:MGI:2158417]	<0.01	0.044	1
Rgs18	ENSMUSG00000026357	protein_coding	regulator of G-protein signaling 18 [Source:MGI Symbol;Acc:MGI:1927498]	<0.01	0.012	1
Col23a1	ENSMUSG00000063564	protein_coding	collagen, type XXIII, alpha 1 [Source:MGI Symbol;Acc:MGI:2653243]	<0.01	0.011	1
Hbq1b	ENSMUSG00000073063	protein_coding	hemoglobin, theta 1B [Source:MGI Symbol;Acc:MGI:3613460]	<0.01	0.034	1
Gm27219	ENSMUSG00000098985	processed_pseudogene	predicted gene 27219 [Source:MGI Symbol;Acc:MGI:5521062]	<0.01	0.027	1
A930005H10Rik	ENSMUSG00000054426	processed_transcript	RIKEN cDNA A930005H10 gene [Source:MGI Symbol;Acc:MGI:1915411]	<0.01	0.037	1
Tnnt1	ENSMUSG00000064179	protein_coding	troponin T1, skeletal, slow [Source:MGI Symbol;Acc:MGI:1333868]	<0.01	0.039	1
Borcs7	ENSMUSG00000062376	protein_coding	BLOC-1 related complex subunit 7 [Source:MGI Symbol;Acc:MGI:1913689]	<0.01	0.036	1
2310033P09Rik	ENSMUSG00000020441	protein_coding	RIKEN cDNA 2310033P09 gene [Source:MGI Symbol;Acc:MGI:1915112]	<0.01	0.028	1
Gm5303	ENSMUSG00000083107	processed_pseudogene	predicted gene 5303 [Source:MGI Symbol;Acc:MGI:3647388]	<0.01	0.021	1
Gm13443	ENSMUSG00000075391	processed_pseudogene	predicted gene 13443 [Source:MGI Symbol;Acc:MGI:3649615]	<0.01	<0.01	1
Edem2	ENSMUSG00000038312	protein_coding	ER degradation enhancer, mannosidase alpha-like 2 [Source:MGI Symbol;Acc:MGI:1915540]	<0.01	0.038	1
Poc1a	ENSMUSG00000023345	protein_coding	POC1 centriolar protein A [Source:MGI Symbol;Acc:MGI:1917485]	<0.01	<0.01	1
Hist1h4i	ENSMUSG00000060639	protein_coding	histone cluster 1, H4i [Source:MGI Symbol;Acc:MGI:2448432]	<0.01	<0.01	1
Rab6a	ENSMUSG00000030704	protein_coding	RAB6A, member RAS oncogene family [Source:MGI Symbol;Acc:MGI:894313]	<0.01	0.012	1
Rps6	ENSMUSG00000028495	protein_coding	ribosomal protein S6 [Source:MGI Symbol;Acc:MGI:98159]	<0.01	<0.01	1
Adgrf3	ENSMUSG00000067642	protein_coding	adhesion G protein-coupled receptor F3 [Source:MGI Symbol;Acc:MGI:2685887]	<0.01	<0.01	1
Dclre1a	ENSMUSG00000025077	protein_coding	DNA cross-link repair 1A [Source:MGI Symbol;Acc:MGI:1930042]	<0.01	0.016	1
Gm13225	ENSMUSG00000078503	protein_coding	zinc finger protein 990 [Source:MGI Symbol;Acc:MGI:3652161]	<0.01	0.015	1
Riok1	ENSMUSG00000021428	protein_coding	RIO kinase 1 (yeast) [Source:MGI Symbol;Acc:MGI:1918590]	<0.01	0.021	1
Apoh	ENSMUSG00000000049	protein_coding	apolipoprotein H [Source:MGI Symbol;Acc:MGI:88058]	<0.01	0.042	1
Sae1	ENSMUSG00000052833	protein_coding	SUMO1 activating enzyme subunit 1 [Source:MGI Symbol;Acc:MGI:1929264]	<0.01	0.026	1
1810058I24Rik	ENSMUSG00000073155	lincRNA	RIKEN cDNA 1810058I24 gene [Source:MGI Symbol;Acc:MGI:1914955]	<0.01	0.028	1
Mrc1	ENSMUSG00000026712	protein_coding	mannose receptor, C type 1 [Source:MGI Symbol;Acc:MGI:97142]	<0.01	0.039	1
Gpr4	ENSMUSG00000044317	protein_coding	G protein-coupled receptor 4 [Source:MGI Symbol;Acc:MGI:2441992]	<0.01	<0.01	1
Per2	ENSMUSG00000055866	protein_coding	period circadian clock 2 [Source:MGI Symbol;Acc:MGI:1195265]	<0.01	<0.01	1
Efhd1	ENSMUSG00000026255	protein_coding	EF hand domain containing 1 [Source:MGI Symbol;Acc:MGI:1921607]	<0.01	<0.01	1
Hist2h2aa2	ENSMUSG00000063954	protein_coding	histone cluster 2, H2aa2 [Source:MGI Symbol;Acc:MGI:2448283]	<0.01	<0.01	1
Mzt2	ENSMUSG00000022671	protein_coding	mitotic spindle organizing protein 2 [Source:MGI Symbol;Acc:MGI:1922845]	<0.01	0.019	1
Inafm2	ENSMUSG00000074918	protein_coding	InaF motif containing 2 [Source:MGI Symbol;Acc:MGI:1915354]	<0.01	0.018	1
Nacc2	ENSMUSG00000026932	protein_coding	nucleus accumbens associated 2, BEN and BTB (POZ) domain containing [Source:MGI Symbol;Acc:MGI:1915241]	<0.01	0.016	1
Gca	ENSMUSG00000026893	protein_coding	grancalcin [Source:MGI Symbol;Acc:MGI:1918521]	<0.01	0.030	1
Cuedc1	ENSMUSG00000018378	protein_coding	CUE domain containing 1 [Source:MGI Symbol;Acc:MGI:2144281]	<0.01	0.012	1
Pyroxd2	ENSMUSG00000060224	protein_coding	pyridine nucleotide-disulphide oxidoreductase domain 2 [Source:MGI Symbol;Acc:MGI:1921830]	<0.01	0.046	1
Ankrd1	ENSMUSG00000024803	protein_coding	ankyrin repeat domain 1 (cardiac muscle) [Source:MGI Symbol;Acc:MGI:1097717]	<0.01	<0.01	1
Dll4	ENSMUSG00000027314	protein_coding	delta-like 4 (Drosophila) [Source:MGI Symbol;Acc:MGI:1859388]	<0.01	0.023	1
Gm12312	ENSMUSG00000082145	processed_pseudogene	predicted gene 12312 [Source:MGI Symbol;Acc:MGI:3649321]	<0.01	<0.01	1
Chst2	ENSMUSG00000033350	protein_coding	carbohydrate sulfotransferase 2 [Source:MGI Symbol;Acc:MGI:1891160]	<0.01	<0.01	1
Man2a2	ENSMUSG00000038886	protein_coding	mannosidase 2, alpha 2 [Source:MGI Symbol;Acc:MGI:2150656]	<0.01	0.038	1
Calcr	ENSMUSG00000023964	protein_coding	calcitonin receptor [Source:MGI Symbol;Acc:MGI:101950]	<0.01	0.019	1
Ccdc42	ENSMUSG00000045915	protein_coding	coiled-coil domain containing 42 [Source:MGI Symbol;Acc:MGI:3045254]	<0.01	<0.01	1
Nhlrc1	ENSMUSG00000044231	protein_coding	NHL repeat containing 1 [Source:MGI Symbol;Acc:MGI:2145264]	<0.01	0.022	1
Angpt1	ENSMUSG00000022309	protein_coding	angiopoietin 1 [Source:MGI Symbol;Acc:MGI:108448]	<0.01	0.027	1
Thrb	ENSMUSG00000021779	protein_coding	thyroid hormone receptor beta [Source:MGI Symbol;Acc:MGI:98743]	<0.01	0.039	1
Inpp5k	ENSMUSG00000006127	protein_coding	inositol polyphosphate 5-phosphatase K [Source:MGI Symbol;Acc:MGI:1194899]	<0.01	0.021	1
Hadhb	ENSMUSG00000059447	protein_coding	hydroxyacyl-Coenzyme A dehydrogenase/3-ketoacyl-Coenzyme A thiolase/enoyl-Coenzyme A hydratase (trifunctional protein), beta subunit [Source:MGI Symbol;Acc:MGI:2136381]	<0.01	0.039	1
Vstm5	ENSMUSG00000031937	protein_coding	V-set and transmembrane domain containing 5 [Source:MGI Symbol;Acc:MGI:1916387]	<0.01	0.030	1
Cxcr1	ENSMUSG00000048480	protein_coding	chemokine (C-X-C motif) receptor 1 [Source:MGI Symbol;Acc:MGI:2448715]	<0.01	<0.01	1
Raver2	ENSMUSG00000035275	protein_coding	ribonucleoprotein, PTB-binding 2 [Source:MGI Symbol;Acc:MGI:2443623]	<0.01	0.014	1
Tlr11	ENSMUSG00000051969	protein_coding	toll-like receptor 11 [Source:MGI Symbol;Acc:MGI:3045226]	<0.01	<0.01	1
H2-Ea-ps	ENSMUSG00000036322	unprocessed_pseudogene	histocompatibility 2, class II antigen E alpha, pseudogene [Source:MGI Symbol;Acc:MGI:95900]	<0.01	<0.01	1
Camsap1	ENSMUSG00000026933	protein_coding	calmodulin regulated spectrin-associated protein 1 [Source:MGI Symbol;Acc:MGI:3036242]	<0.01	0.038	1
Cd59a	ENSMUSG00000032679	protein_coding	CD59a antigen [Source:MGI Symbol;Acc:MGI:109177]	<0.01	<0.01	1
Gm10499	ENSMUSG00000073403	transcribed_unprocessed_pseudogene	predicted gene 10499 [Source:MGI Symbol;Acc:MGI:3702919]	<0.01	<0.01	1
Hsd3b2	ENSMUSG00000063730	protein_coding	hydroxy-delta-5-steroid dehydrogenase, 3 beta- and steroid delta-isomerase 2 [Source:MGI Symbol;Acc:MGI:96234]	<0.01	0.028	1
Ackr2	ENSMUSG00000044534	protein_coding	atypical chemokine receptor 2 [Source:MGI Symbol;Acc:MGI:1891697]	<0.01	<0.01	1
Angptl7	ENSMUSG00000028989	protein_coding	angiopoietin-like 7 [Source:MGI Symbol;Acc:MGI:3605801]	<0.01	<0.01	1
Fbln5	ENSMUSG00000021186	protein_coding	fibulin 5 [Source:MGI Symbol;Acc:MGI:1346091]	<0.01	0.015	1
Ddc	ENSMUSG00000020182	protein_coding	dopa decarboxylase [Source:MGI Symbol;Acc:MGI:94876]	<0.01	0.049	1
Car4	ENSMUSG00000000805	protein_coding	carbonic anhydrase 4 [Source:MGI Symbol;Acc:MGI:1096574]	<0.01	<0.01	1
Abhd11os	ENSMUSG00000085042	transcribed_unitary_pseudogene	abhydrolase domain containing 11, opposite strand [Source:MGI Symbol;Acc:MGI:1917062]	<0.01	0.027	1
Cobll1	ENSMUSG00000034903	protein_coding	Cobl-like 1 [Source:MGI Symbol;Acc:MGI:2442894]	<0.01	0.026	1
Arsj	ENSMUSG00000046561	protein_coding	arylsulfatase J [Source:MGI Symbol;Acc:MGI:2443513]	<0.01	<0.01	1
Wdfy1	ENSMUSG00000073643	protein_coding	WD repeat and FYVE domain containing 1 [Source:MGI Symbol;Acc:MGI:1916618]	<0.01	0.033	1
Tfpi	ENSMUSG00000027082	protein_coding	tissue factor pathway inhibitor [Source:MGI Symbol;Acc:MGI:1095418]	<0.01	<0.01	1
Tspan4	ENSMUSG00000025511	protein_coding	tetraspanin 4 [Source:MGI Symbol;Acc:MGI:1928097]	<0.01	<0.01	1
Il18	ENSMUSG00000039217	protein_coding	interleukin 18 [Source:MGI Symbol;Acc:MGI:107936]	<0.01	<0.01	1
Ighv2-6-8	ENSMUSG00000076646	IG_V_gene	immunoglobulin heavy variable 2-6-8 [Source:MGI Symbol;Acc:MGI:4439811]	<0.01	0.012	1
Gm15487	ENSMUSG00000080242	processed_pseudogene	predicted gene 15487 [Source:MGI Symbol;Acc:MGI:3709610]	<0.01	0.048	1
Mfap4	ENSMUSG00000042436	protein_coding	microfibrillar-associated protein 4 [Source:MGI Symbol;Acc:MGI:1342276]	<0.01	0.032	1
Hbb-bs	ENSMUSG00000052305	protein_coding	hemoglobin, beta adult s chain [Source:MGI Symbol;Acc:MGI:5474852]	<0.01	<0.01	1
Upk3a	ENSMUSG00000022435	protein_coding	uroplakin 3A [Source:MGI Symbol;Acc:MGI:98914]	<0.01	<0.01	1
Prelid2	ENSMUSG00000056671	protein_coding	PRELI domain containing 2 [Source:MGI Symbol;Acc:MGI:1924869]	<0.01	<0.01	1
Kcnk3	ENSMUSG00000049265	protein_coding	potassium channel, subfamily K, member 3 [Source:MGI Symbol;Acc:MGI:1100509]	<0.01	<0.01	1
Gm5161	ENSMUSG00000061486	processed_pseudogene	predicted pseudogene 5161 [Source:MGI Symbol;Acc:MGI:3648529]	<0.01	0.023	1

Rassf1	ENSMUSG00000010067	protein_coding	Ras association (RalGDS/AF-6) domain family member 1 [Source:MGI Symbol;Acc:MGI:1928386]	<0.01	0.046	1
Tnxb	ENSMUSG00000033327	protein_coding	tenascin XB [Source:MGI Symbol;Acc:MGI:1932137]	<0.01	<0.01	1
Prkce	ENSMUSG00000045038	protein_coding	protein kinase C, epsilon [Source:MGI Symbol;Acc:MGI:97599]	<0.01	0.020	1
Hist2h2ac	ENSMUSG00000068855	protein_coding	histone cluster 2, H2ac [Source:MGI Symbol;Acc:MGI:2448316]	<0.01	<0.01	1
Baiap2l1	ENSMUSG00000038859	protein_coding	BAI1-associated protein 2-like 1 [Source:MGI Symbol;Acc:MGI:1914148]	<0.01	<0.01	1
Fer	ENSMUSG00000000127	protein_coding	fer (fms/fps related) protein kinase [Source:MGI Symbol;Acc:MGI:105917]	<0.01	0.026	1
Gnai1	ENSMUSG00000057614	protein_coding	guanine nucleotide binding protein (G protein), alpha inhibiting 1 [Source:MGI Symbol;Acc:MGI:95771]	<0.01	0.028	1
Pelo	ENSMUSG00000042275	protein_coding	pelota homolog (Drosophila) [Source:MGI Symbol;Acc:MGI:2145154]	<0.01	0.023	1
Serpine1	ENSMUSG00000037411	protein_coding	serine (or cysteine) peptidase inhibitor, clade E, member 1 [Source:MGI Symbol;Acc:MGI:97608]	<0.01	0.038	1
Sapcd1	ENSMUSG00000036185	protein_coding	suppressor APC domain containing 1 [Source:MGI Symbol;Acc:MGI:2388100]	<0.01	<0.01	1
Gm10260	ENSMUSG00000069117	protein_coding	predicted gene 10260 [Source:MGI Symbol;Acc:MGI:3642298]	<0.01	<0.01	1
Unc5b	ENSMUSG00000020099	protein_coding	unc-5 netrin receptor B [Source:MGI Symbol;Acc:MGI:894703]	<0.01	<0.01	1
BC064078	ENSMUSG00000087150	transcribed_unprocessed_pseudogene	cDNA sequence BC064078 [Source:MGI Symbol;Acc:MGI:3040692]	<0.01	<0.01	1
Rps25-ps1	ENSMUSG00000067344	processed_pseudogene	ribosomal protein S25, pseudogene 1 [Source:MGI Symbol;Acc:MGI:3642902]	<0.01	<0.01	1
Nbea	ENSMUSG00000027799	protein_coding	neurobeachin [Source:MGI Symbol;Acc:MGI:1347075]	<0.01	0.012	1
Usp13	ENSMUSG00000056900	protein_coding	ubiquitin specific peptidase 13 (isopeptidase T-3) [Source:MGI Symbol;Acc:MGI:1919857]	<0.01	0.022	1
Gm26733	ENSMUSG00000097469	lincRNA	predicted gene, 26733 [Source:MGI Symbol;Acc:MGI:5477227]	<0.01	0.033	1
Timeless	ENSMUSG00000039994	protein_coding	timeless circadian clock 1 [Source:MGI Symbol;Acc:MGI:1321393]	<0.01	0.044	1
Emid1	ENSMUSG00000034164	protein_coding	EMI domain containing 1 [Source:MGI Symbol;Acc:MGI:2155091]	<0.01	<0.01	1
Tmem237	ENSMUSG00000038079	protein_coding	transmembrane protein 237 [Source:MGI Symbol;Acc:MGI:2138365]	<0.01	0.020	1
Cntn4	ENSMUSG00000064293	protein_coding	contactin 4 [Source:MGI Symbol;Acc:MGI:1095737]	<0.01	<0.01	1
9230117E06Rik	ENSMUSG00000089874	antisense_RNA	RIKEN cDNA 9230117E06 gene [Source:MGI Symbol;Acc:MGI:1925332]	<0.01	0.042	1
Eno1b	ENSMUSG00000059040	protein_coding	enolase 1B, retrotransposed [Source:MGI Symbol;Acc:MGI:3648653]	<0.01	<0.01	1
Marveld2	ENSMUSG00000021636	protein_coding	MARVEL (membrane-associating) domain containing 2 [Source:MGI Symbol;Acc:MGI:2446166]	<0.01	0.044	1
Dbp	ENSMUSG00000059824	protein_coding	D site albumin promoter binding protein [Source:MGI Symbol;Acc:MGI:94866]	<0.01	<0.01	1
Dsp	ENSMUSG00000054889	protein_coding	desmoplakin [Source:MGI Symbol;Acc:MGI:109611]	<0.01	0.013	1
Cnot7	ENSMUSG00000031601	protein_coding	CCR4-NOT transcription complex, subunit 7 [Source:MGI Symbol;Acc:MGI:1298230]	<0.01	0.018	1
Ly6c2	ENSMUSG00000022584	protein_coding	lymphocyte antigen 6 complex, locus C2 [Source:MGI Symbol;Acc:MGI:3712069]	<0.01	0.029	1
Tshb	ENSMUSG00000027857	protein_coding	thyroid stimulating hormone, beta subunit [Source:MGI Symbol;Acc:MGI:98848]	<0.01	<0.01	1
Zcchc24	ENSMUSG00000055538	protein_coding	zinc finger, CCHC domain containing 24 [Source:MGI Symbol;Acc:MGI:1919168]	<0.01	0.036	1
Atp9a	ENSMUSG00000027546	protein_coding	ATPase, class II, type 9A [Source:MGI Symbol;Acc:MGI:1330826]	<0.01	<0.01	1
Atxn1	ENSMUSG00000046876	protein_coding	ataxin 1 [Source:MGI Symbol;Acc:MGI:104783]	<0.01	0.021	1
Map1b	ENSMUSG00000052727	protein_coding	microtubule-associated protein 1B [Source:MGI Symbol;Acc:MGI:1306778]	<0.01	0.016	1
Wfdc12	ENSMUSG00000042845	protein_coding	WAP four-disulfide core domain 12 [Source:MGI Symbol;Acc:MGI:2183434]	<0.01	0.022	1
Arsg	ENSMUSG00000020604	protein_coding	arylsulfatase G [Source:MGI Symbol;Acc:MGI:1921258]	<0.01	0.025	1
Nrp2	ENSMUSG00000025969	protein_coding	neuropilin 2 [Source:MGI Symbol;Acc:MGI:1100492]	<0.01	<0.01	1
H2-Q1	ENSMUSG00000079507	protein_coding	histocompatibility 2, Q region locus 1 [Source:MGI Symbol;Acc:MGI:95928]	<0.01	<0.01	1
Ednrb	ENSMUSG00000022122	protein_coding	endothelin receptor type B [Source:MGI Symbol;Acc:MGI:102720]	<0.01	0.031	1
2610203C22Rik	ENSMUSG00000079671	processed_transcript	RIKEN cDNA 2610203C22 gene [Source:MGI Symbol;Acc:MGI:1919731]	<0.01	<0.01	1
Hbb-bt	ENSMUSG00000073940	protein_coding	hemoglobin, beta adult t chain [Source:MGI Symbol;Acc:MGI:5474850]	<0.01	<0.01	1
Cxcl12	ENSMUSG00000061353	protein_coding	chemokine (C-X-C motif) ligand 12 [Source:MGI Symbol;Acc:MGI:103556]	<0.01	0.012	1
Mfap1a	ENSMUSG00000068479	protein_coding	microfibrillar-associated protein 1A [Source:MGI Symbol;Acc:MGI:1914782]	<0.01	<0.01	1
Fam167b	ENSMUSG00000050493	protein_coding	family with sequence similarity 167, member B [Source:MGI Symbol;Acc:MGI:2668032]	<0.01	<0.01	1
Il1a	ENSMUSG00000027399	protein_coding	interleukin 1 alpha [Source:MGI Symbol;Acc:MGI:96542]	<0.01	0.024	1
Rassf8	ENSMUSG00000030259	protein_coding	Ras association (RalGDS/AF-6) domain family (N-terminal) member 8 [Source:MGI Symbol;Acc:MGI:1918573]	<0.01	0.016	1
Tmeff1	ENSMUSG00000028347	protein_coding	transmembrane protein with EGF-like and two follistatin-like domains 1 [Source:MGI Symbol;Acc:MGI:1926810]	<0.01	<0.01	1
Rpl31-ps15	ENSMUSG00000097304	processed_pseudogene	ribosomal protein L31, pseudogene 15 [Source:MGI Symbol;Acc:MGI:3643738]	<0.01	<0.01	1
Prss22	ENSMUSG00000045027	protein_coding	protease, serine 22 [Source:MGI Symbol;Acc:MGI:1918085]	<0.01	0.037	1
Dnase1l3	ENSMUSG00000025279	protein_coding	deoxyribonuclease 1-like 3 [Source:MGI Symbol;Acc:MGI:1314633]	<0.01	<0.01	1
Tmc7	ENSMUSG00000042246	protein_coding	transmembrane channel-like gene family 7 [Source:MGI Symbol;Acc:MGI:2443317]	<0.01	<0.01	1
Cbfa2t3	ENSMUSG00000006362	protein_coding	core-binding factor, runt domain, alpha subunit 2, translocated to, 3 (human) [Source:MGI Symbol;Acc:MGI:1338013]	<0.01	0.025	1
Hist2h3c1	ENSMUSG00000093769	protein_coding	histone cluster 2, H3c1 [Source:MGI Symbol;Acc:MGI:2448355]	<0.01	0.016	1
Pvrl3	ENSMUSG00000022656	protein_coding	nectin cell adhesion molecule 3 [Source:MGI Symbol;Acc:MGI:1930171]	<0.01	0.014	1
Cipc	ENSMUSG00000034157	protein_coding	CLOCK interacting protein, circadian [Source:MGI Symbol;Acc:MGI:1919185]	<0.01	0.048	1
Opa3	ENSMUSG00000052214	protein_coding	optic atrophy 3 [Source:MGI Symbol;Acc:MGI:2686271]	<0.01	0.043	1
Commd9	ENSMUSG00000027163	protein_coding	COMM domain containing 9 [Source:MGI Symbol;Acc:MGI:1923751]	<0.01	0.042	1
Cybrd1	ENSMUSG00000027015	protein_coding	cytochrome b reductase 1 [Source:MGI Symbol;Acc:MGI:2654575]	<0.01	<0.01	1
2210011C24Rik	ENSMUSG00000074217	protein_coding	RIKEN cDNA 2210011C24 gene [Source:MGI Symbol;Acc:MGI:1917384]	<0.01	0.040	1
Sphk2	ENSMUSG00000057342	protein_coding	sphingosine kinase 2 [Source:MGI Symbol;Acc:MGI:1861380]	<0.01	<0.01	1
Eif2ak4	ENSMUSG00000005102	protein_coding	eukaryotic translation initiation factor 2 alpha kinase 4 [Source:MGI Symbol;Acc:MGI:1353427]	<0.01	0.025	1
Htr2b	ENSMUSG00000026228	protein_coding	5-hydroxytryptamine (serotonin) receptor 2B [Source:MGI Symbol;Acc:MGI:109323]	<0.01	<0.01	1
Pabpc4l	ENSMUSG00000090919	protein_coding	poly(A) binding protein, cytoplasmic 4-like [Source:MGI Symbol;Acc:MGI:3643087]	<0.01	0.028	1
RP23-137A24.3	ENSMUSG00000108555	TEC	predicted gene, 18310 [Source:MGI Symbol;Acc:MGI:5010495]	<0.01	0.016	1
Nudt17	ENSMUSG00000028100	protein_coding	nudix (nucleoside diphosphate linked moiety X)-type motif 17 [Source:MGI Symbol;Acc:MGI:1925623]	<0.01	0.038	1
Dapk2	ENSMUSG00000032380	protein_coding	death-associated protein kinase 2 [Source:MGI Symbol;Acc:MGI:1341297]	<0.01	0.030	1
Efr3b	ENSMUSG00000020658	protein_coding	EFR3 homolog B [Source:MGI Symbol;Acc:MGI:2444851]	<0.01	0.049	1
Rtp3	ENSMUSG00000066319	protein_coding	receptor transporter protein 3 [Source:MGI Symbol;Acc:MGI:2446841]	<0.01	<0.01	1
Sptbn1	ENSMUSG00000020315	protein_coding	spectrin beta, non-erythrocytic 1 [Source:MGI Symbol;Acc:MGI:98388]	<0.01	0.027	1
Scamp5	ENSMUSG00000040722	protein_coding	secretory carrier membrane protein 5 [Source:MGI Symbol;Acc:MGI:1928948]	<0.01	0.039	1
Gm11127	ENSMUSG00000079492	protein_coding	predicted gene 11127 [Source:MGI Symbol;Acc:MGI:3779381]	<0.01	0.015	1
Tef	ENSMUSG00000022389	protein_coding	thyrotroph embryonic factor [Source:MGI Symbol;Acc:MGI:98663]	<0.01	<0.01	1
Cldn1	ENSMUSG00000022512	protein_coding	claudin 1 [Source:MGI Symbol;Acc:MGI:1276109]	<0.01	0.012	1
2810428I15Rik	ENSMUSG00000058833	protein_coding	RIKEN cDNA 2810428I15 gene [Source:MGI Symbol;Acc:MGI:1913712]	<0.01	0.025	1
Sema3c	ENSMUSG00000028780	protein_coding	sema domain, immunoglobulin domain (Ig), short basic domain, secreted, (semaphorin) 3C [Source:MGI Symbol;Acc:MGI:107557]	<0.01	0.048	1
Igfbp2	ENSMUSG00000039323	protein_coding	insulin-like growth factor binding protein 2 [Source:MGI Symbol;Acc:MGI:96437]	<0.01	0.036	1
Fmo1	ENSMUSG00000040181	protein_coding	flavin containing monooxygenase 1 [Source:MGI Symbol;Acc:MGI:1310002]	<0.01	0.014	1
Pthlh	ENSMUSG00000048776	protein_coding	parathyroid hormone-like peptide [Source:MGI Symbol;Acc:MGI:97800]	<0.01	<0.01	1
Scarf1	ENSMUSG00000038188	protein_coding	scavenger receptor class F, member 1 [Source:MGI Symbol;Acc:MGI:2449455]	<0.01	<0.01	1
Pabpc1l	ENSMUSG00000054582	protein_coding	poly(A) binding protein, cytoplasmic 1-like [Source:MGI Symbol;Acc:MGI:1922908]	<0.01	<0.01	1
Wisp1	ENSMUSG00000005124	protein_coding	WNT1 inducible signaling pathway protein 1 [Source:MGI Symbol;Acc:MGI:1197008]	<0.01	<0.01	1

Clasp2	ENSMUSG00000033392	protein_coding	CLIP associating protein 2 [Source:MGI Symbol;Acc:MGI:1923749]	<0.01	0.030	1
Hebp1	ENSMUSG00000042770	protein_coding	heme binding protein 1 [Source:MGI Symbol;Acc:MGI:1333880]	<0.01	<0.01	1
Scube1	ENSMUSG00000016763	protein_coding	signal peptide, CUB domain, EGF-like 1 [Source:MGI Symbol;Acc:MGI:1890616]	<0.01	0.018	1
Zfp709	ENSMUSG00000056019	protein_coding	zinc finger protein 709 [Source:MGI Symbol;Acc:MGI:2384299]	<0.01	<0.01	1
Fgfrl1	ENSMUSG00000008090	protein_coding	fibroblast growth factor receptor-like 1 [Source:MGI Symbol;Acc:MGI:2150920]	<0.01	0.016	1
Fgfr4	ENSMUSG00000005320	protein_coding	fibroblast growth factor receptor 4 [Source:MGI Symbol;Acc:MGI:95525]	<0.01	0.028	1
Fbxw7	ENSMUSG00000028086	protein_coding	F-box and WD-40 domain protein 7 [Source:MGI Symbol;Acc:MGI:1354695]	<0.01	0.025	1
Vsnl1	ENSMUSG00000054459	protein_coding	visinin-like 1 [Source:MGI Symbol;Acc:MGI:1349453]	<0.01	0.018	1
Cpxm1	ENSMUSG00000027408	protein_coding	carboxypeptidase X 1 (M14 family) [Source:MGI Symbol;Acc:MGI:1934569]	<0.01	<0.01	1
Spata5	ENSMUSG00000027722	protein_coding	spermatogenesis associated 5 [Source:MGI Symbol;Acc:MGI:1927170]	<0.01	0.012	1
1500035N22Rik	ENSMUSG00000059631	lincRNA	RIKEN cDNA 1500035N22 gene [Source:MGI Symbol;Acc:MGI:1917508]	<0.01	<0.01	1
Lrrn2	ENSMUSG00000026443	protein_coding	leucine rich repeat protein 2, neuronal [Source:MGI Symbol;Acc:MGI:106037]	<0.01	0.015	1
1700052K11Rik	ENSMUSG00000099681	antisense_RNA	RIKEN cDNA 1700052K11 gene [Source:MGI Symbol;Acc:MGI:1920681]	<0.01	<0.01	1
Gabra4	ENSMUSG00000029211	protein_coding	gamma-aminobutyric acid (GABA) A receptor, subunit alpha 4 [Source:MGI Symbol;Acc:MGI:95616]	<0.01	0.013	1
Nkd2	ENSMUSG00000021567	protein_coding	naked cuticle 2 homolog (Drosophila) [Source:MGI Symbol;Acc:MGI:1919543]	<0.01	0.021	1
Colec12	ENSMUSG00000036103	protein_coding	collectin sub-family member 12 [Source:MGI Symbol;Acc:MGI:2152907]	<0.01	0.026	1
Tuba8	ENSMUSG00000030137	protein_coding	tubulin, alpha 8 [Source:MGI Symbol;Acc:MGI:1858225]	<0.01	0.021	1
Mrgprf	ENSMUSG00000031070	protein_coding	MAS-related GPR, member F [Source:MGI Symbol;Acc:MGI:2384823]	<0.01	0.041	1
Trim47	ENSMUSG00000020773	protein_coding	tripartite motif-containing 47 [Source:MGI Symbol;Acc:MGI:1917374]	<0.01	0.013	1
Scaper	ENSMUSG00000034007	protein_coding	S phase cyclin A-associated protein in the ER [Source:MGI Symbol;Acc:MGI:1925976]	<0.01	0.040	1
Rps6kc1	ENSMUSG00000089872	protein_coding	ribosomal protein S6 kinase polypeptide 1 [Source:MGI Symbol;Acc:MGI:2443419]	<0.01	0.015	1
Esam	ENSMUSG00000001946	protein_coding	endothelial cell-specific adhesion molecule [Source:MGI Symbol;Acc:MGI:1916774]	<0.01	0.038	1
Grin2d	ENSMUSG00000002771	protein_coding	glutamate receptor, ionotropic, NMDA2D (epsilon 4) [Source:MGI Symbol;Acc:MGI:95823]	<0.01	0.015	1
Mest	ENSMUSG00000051855	protein_coding	mesoderm specific transcript [Source:MGI Symbol;Acc:MGI:96968]	<0.01	<0.01	1
Gamt	ENSMUSG00000020150	protein_coding	guanidinoacetate methyltransferase [Source:MGI Symbol;Acc:MGI:1098221]	<0.01	0.021	1
Rad51ap1	ENSMUSG00000030346	protein_coding	RAD51 associated protein 1 [Source:MGI Symbol;Acc:MGI:1098224]	<0.01	<0.01	1
Gm13066	ENSMUSG00000086949	antisense_RNA	predicted gene 13066 [Source:MGI Symbol;Acc:MGI:3701131]	<0.01	0.015	1
Glb1l2	ENSMUSG00000036395	protein_coding	galactosidase, beta 1-like 2 [Source:MGI Symbol;Acc:MGI:2388283]	<0.01	<0.01	1
Rep15	ENSMUSG00000040121	protein_coding	RAB15 effector protein [Source:MGI Symbol;Acc:MGI:1913782]	<0.01	0.035	1
Ephx3	ENSMUSG00000037577	protein_coding	epoxide hydrolase 3 [Source:MGI Symbol;Acc:MGI:1919182]	<0.01	0.042	1
Dynlt1c	ENSMUSG00000000579	protein_coding	dynein light chain Tctex-type 1C [Source:MGI Symbol;Acc:MGI:3807476]	<0.01	<0.01	1
Tspan13	ENSMUSG00000020577	protein_coding	tetraspanin 13 [Source:MGI Symbol;Acc:MGI:1913359]	<0.01	0.013	1
Zfp369	ENSMUSG00000021514	protein_coding	zinc finger protein 369 [Source:MGI Symbol;Acc:MGI:2176229]	<0.01	0.024	1
Gm12022	ENSMUSG00000085675	lincRNA	predicted gene 12022 [Source:MGI Symbol;Acc:MGI:3650976]	<0.01	0.027	1
Klra3	ENSMUSG00000067591	protein_coding	killer cell lectin-like receptor, subfamily A, member 3 [Source:MGI Symbol;Acc:MGI:101905]	<0.01	<0.01	1
B230206L02Rik	ENSMUSG00000086003	processed_transcript	RIKEN cDNA B230206L02 gene [Source:MGI Symbol;Acc:MGI:1924598]	<0.01	<0.01	1
Tmem150a	ENSMUSG00000055912	protein_coding	transmembrane protein 150A [Source:MGI Symbol;Acc:MGI:2385244]	<0.01	<0.01	1
Fam150a	ENSMUSG00000087247	protein_coding	ALK and LTK ligand 1 [Source:MGI Symbol;Acc:MGI:3645495]	<0.01	<0.01	1
Tmem86a	ENSMUSG00000010307	protein_coding	transmembrane protein 86A [Source:MGI Symbol;Acc:MGI:1915143]	<0.01	0.031	1
Nr1d2	ENSMUSG00000021775	protein_coding	nuclear receptor subfamily 1, group D, member 2 [Source:MGI Symbol;Acc:MGI:2449205]	<0.01	<0.01	1
Gm5909	ENSMUSG00000082491	processed_pseudogene	predicted gene 5909 [Source:MGI Symbol;Acc:MGI:3646686]	<0.01	0.012	1
Mboat2	ENSMUSG00000020646	protein_coding	membrane bound O-acyltransferase domain containing 2 [Source:MGI Symbol;Acc:MGI:1914466]	<0.01	0.019	1
Lefty1	ENSMUSG00000038793	protein_coding	left right determination factor 1 [Source:MGI Symbol;Acc:MGI:107405]	<0.01	0.040	1
Slc6a4	ENSMUSG00000020838	protein_coding	solute carrier family 6 (neurotransmitter transporter, serotonin), member 4 [Source:MGI Symbol;Acc:MGI:96285]	<0.01	0.011	1
Adamtsl5	ENSMUSG00000043822	protein_coding	ADAMTS-like 5 [Source:MGI Symbol;Acc:MGI:1913798]	<0.01	0.037	1
Zfp605	ENSMUSG00000023284	protein_coding	zinc finger protein 605 [Source:MGI Symbol;Acc:MGI:2444933]	<0.01	<0.01	1
Gpnmb	ENSMUSG00000029816	protein_coding	glycoprotein (transmembrane) nmb [Source:MGI Symbol;Acc:MGI:1934765]	<0.01	<0.01	1
Thg1l	ENSMUSG00000011254	protein_coding	tRNA-histidine guanylyltransferase 1-like (S. cerevisiae) [Source:MGI Symbol;Acc:MGI:1913878]	<0.01	<0.01	1
Tlcd2	ENSMUSG00000038217	protein_coding	TLC domain containing 2 [Source:MGI Symbol;Acc:MGI:1917141]	<0.01	<0.01	1
Mettl24	ENSMUSG00000045555	protein_coding	methyltransferase like 24 [Source:MGI Symbol;Acc:MGI:3045338]	<0.01	<0.01	1
Stk19-ps1	ENSMUSG00000092202	unprocessed_pseudogene	serine/threonine kinase 19, pseudogene 1 [Source:MGI Symbol;Acc:MGI:2148500]	<0.01	<0.01	1
Gm15459	ENSMUSG00000100801	processed_pseudogene	predicted gene 15459 [Source:MGI Symbol;Acc:MGI:3705702]	<0.01	<0.01	1
Tlr6	ENSMUSG00000051498	protein_coding	toll-like receptor 6 [Source:MGI Symbol;Acc:MGI:1341296]	<0.01	0.037	1
Cramp1l	ENSMUSG00000038002	protein_coding	Crm, cramped-like (Drosophila) [Source:MGI Symbol;Acc:MGI:1930190]	<0.01	0.029	1
Cdkn2a	ENSMUSG00000044303	protein_coding	cyclin-dependent kinase inhibitor 2A [Source:MGI Symbol;Acc:MGI:104738]	<0.01	<0.01	1
Phf20-ps	ENSMUSG00000098506	processed_pseudogene	PHD finger protein 20, pseudogene [Source:MGI Symbol;Acc:MGI:5010233]	<0.01	0.024	1
Rnf43	ENSMUSG00000034177	protein_coding	ring finger protein 43 [Source:MGI Symbol;Acc:MGI:2442609]	<0.01	0.037	1
Gm43719	ENSMUSG00000105366	TEC	predicted gene 43719 [Source:MGI Symbol;Acc:MGI:5663856]	<0.01	0.013	1
Epb41l3	ENSMUSG00000024044	protein_coding	erythrocyte membrane protein band 4.1 like 3 [Source:MGI Symbol;Acc:MGI:103008]	<0.01	0.036	1
Gm11131	ENSMUSG00000079505	antisense_RNA	predicted gene 11131 [Source:MGI Symbol;Acc:MGI:3779386]	<0.01	<0.01	1
1700097N02Rik	ENSMUSG00000099474	antisense_RNA	RIKEN cDNA 1700097N02 gene [Source:MGI Symbol;Acc:MGI:1914772]	<0.01	<0.01	1

Note: Decide test value "-1" or "1" indicates the t-statistic is classified as significantly negative or significantly positive respectively

Table S3b. Differentially expressed transcripts for sex (2 way limma anova test, limma decide test, P<0.05)

Gene	Gene ID	Biotype	Description	P.value	Adjusted.P.value	Decide test
Cd200	ENSMUSG00000022661	protein_coding	CD200 antigen [Source:MGI Symbol;Acc:MGI:1196990]	<0.01	<0.01	-1
Clu	ENSMUSG00000022037	protein_coding	clusterin [Source:MGI Symbol;Acc:MGI:88423]	<0.01	0.021	-1
Gm10874	ENSMUSG00000075591	lincRNA	predicted gene 10874 [Source:MGI Symbol;Acc:MGI:3704261]	<0.01	0.022	-1
Atp1b1	ENSMUSG00000026576	protein_coding	ATPase, Na+/K+ transporting, beta 1 polypeptide [Source:MGI Symbol;Acc:MGI:88108]	<0.01	0.020	-1
Fbln1	ENSMUSG00000006369	protein_coding	fibulin 1 [Source:MGI Symbol;Acc:MGI:95487]	<0.01	0.037	-1
Maff	ENSMUSG00000042622	protein_coding	v-maf musculoaponeurotic fibrosarcoma oncogene family, protein F (avian) [Source:MGI Symbol;Acc:MGI:96910]	<0.01	0.021	-1
Gzmb	ENSMUSG00000015437	protein_coding	granzyme B [Source:MGI Symbol;Acc:MGI:109267]	<0.01	0.021	-1
Gm6158	ENSMUSG00000090381	processed_pseudogene	predicted gene 6158 [Source:MGI Symbol;Acc:MGI:3779562]	<0.01	0.022	-1
Gm20503	ENSMUSG00000092345	protein_coding	predicted gene 20503 [Source:MGI Symbol;Acc:MGI:5141968]	<0.01	0.010	-1
Gm15501	ENSMUSG00000087412	transcribed_processed_pseudogene	predicted pseudogene 15501 [Source:MGI Symbol;Acc:MGI:3704296]	<0.01	0.022	-1
Sparcl1	ENSMUSG00000029309	protein_coding	SPARC-like 1 [Source:MGI Symbol;Acc:MGI:108110]	<0.01	0.016	-1
Gm6652	ENSMUSG00000099858	processed_pseudogene	predicted gene 6652 [Source:MGI Symbol;Acc:MGI:3647244]	<0.01	<0.01	-1
Abcb4	ENSMUSG00000042476	protein_coding	ATP-binding cassette, sub-family B (MDR/TAP), member 4 [Source:MGI Symbol;Acc:MGI:97569]	<0.01	0.024	-1
1700105P06Rik	ENSMUSG00000099923	antisense_RNA	RIKEN cDNA 1700105P06 gene [Source:MGI Symbol;Acc:MGI:1915479]	<0.01	0.012	-1
Cfh	ENSMUSG00000026365	protein_coding	complement component factor h [Source:MGI Symbol;Acc:MGI:88385]	<0.01	<0.01	-1
C1s1	ENSMUSG00000038521	protein_coding	complement component 1, s subcomponent 1 [Source:MGI Symbol;Acc:MGI:1355312]	<0.01	0.028	-1
Ifi27l2a	ENSMUSG00000079017	protein_coding	interferon, alpha-inducible protein 27 like 2A [Source:MGI Symbol;Acc:MGI:1924183]	<0.01	0.017	-1
Bhlha15	ENSMUSG00000052271	protein_coding	basic helix-loop-helix family, member a15 [Source:MGI Symbol;Acc:MGI:891976]	<0.01	0.028	-1
Sord	ENSMUSG00000027227	protein_coding	sorbitol dehydrogenase [Source:MGI Symbol;Acc:MGI:98266]	<0.01	<0.01	-1
Areg	ENSMUSG00000029378	protein_coding	amphiregulin [Source:MGI Symbol;Acc:MGI:88068]	<0.01	<0.01	-1
Micall1	ENSMUSG00000033039	protein_coding	microtubule associated monooxygenase, calponin and LIM domain containing -like 1 [Source:MGI Symbol;Acc:MGI:105870]	<0.01	0.018	-1
Fgfr2	ENSMUSG00000030849	protein_coding	fibroblast growth factor receptor 2 [Source:MGI Symbol;Acc:MGI:95523]	<0.01	0.023	-1
Slain2	ENSMUSG00000036087	protein_coding	SLAIN motif family, member 2 [Source:MGI Symbol;Acc:MGI:1923241]	<0.01	0.038	-1
Adcy5	ENSMUSG00000022840	protein_coding	adenylate cyclase 5 [Source:MGI Symbol;Acc:MGI:99673]	<0.01	<0.01	-1
Cd96	ENSMUSG00000022657	protein_coding	CD96 antigen [Source:MGI Symbol;Acc:MGI:1934368]	<0.01	0.031	-1
Tbc1d9	ENSMUSG00000031709	protein_coding	TBC1 domain family, member 9 [Source:MGI Symbol;Acc:MGI:1918560]	<0.01	<0.01	-1
Igkv3-12	ENSMUSG00000094117	IG_V_gene	immunoglobulin kappa variable 3-12 [Source:MGI Symbol;Acc:MGI:1330815]	<0.01	<0.01	-1
Cygb	ENSMUSG00000020810	protein_coding	cytoglobin [Source:MGI Symbol;Acc:MGI:2149481]	<0.01	<0.01	-1
Ighv1-15	ENSMUSG00000103254	IG_V_gene	immunoglobulin heavy variable 1-15 [Source:MGI Symbol;Acc:MGI:4439782]	<0.01	<0.01	-1
Folr1	ENSMUSG00000001827	protein_coding	folate receptor 1 (adult) [Source:MGI Symbol;Acc:MGI:95568]	<0.01	<0.01	-1
Ighv1-78	ENSMUSG00000096326	IG_V_gene	immunoglobulin heavy variable 1-78 [Source:MGI Symbol;Acc:MGI:4439736]	<0.01	<0.01	-1
Unc5a	ENSMUSG00000025876	protein_coding	unc-5 netrin receptor A [Source:MGI Symbol;Acc:MGI:894682]	<0.01	<0.01	-1
Dnah11	ENSMUSG00000018581	protein_coding	dynein, axonemal, heavy chain 11 [Source:MGI Symbol;Acc:MGI:1100864]	<0.01	0.014	-1
Igkv4-57	ENSMUSG00000076556	IG_V_gene	immunoglobulin kappa variable 4-57 [Source:MGI Symbol;Acc:MGI:2685035]	<0.01	<0.01	-1
Gm20402	ENSMUSG00000092405	antisense_RNA	predicted gene 20402 [Source:MGI Symbol;Acc:MGI:5141867]	<0.01	0.023	-1
Cdv3	ENSMUSG00000032803	protein_coding	carnitine deficiency-associated gene expressed in ventricle 3 [Source:MGI Symbol;Acc:MGI:2448759]	<0.01	0.034	-1
Gm830	ENSMUSG00000084939	processed_transcript	predicted gene 830 [Source:MGI Symbol;Acc:MGI:2685676]	<0.01	<0.01	-1
Igkv1-117	ENSMUSG00000094335	IG_V_gene	immunoglobulin kappa variable 1-117 [Source:MGI Symbol;Acc:MGI:4439721]	<0.01	<0.01	-1
Cux2	ENSMUSG00000042589	protein_coding	cut-like homeobox 2 [Source:MGI Symbol;Acc:MGI:107321]	<0.01	0.041	-1
Gm27021	ENSMUSG00000097919	protein_coding	predicted gene, 27021 [Source:MGI Symbol;Acc:MGI:5504136]	<0.01	<0.01	-1
Ighv1-80	ENSMUSG00000094075	IG_V_gene	immunoglobulin heavy variable 1-80 [Source:MGI Symbol;Acc:MGI:4439738]	<0.01	<0.01	-1
Rap1gap	ENSMUSG00000041351	protein_coding	Rap1 GTPase-activating protein [Source:MGI Symbol;Acc:MGI:109338]	<0.01	0.012	-1
Slc26a4	ENSMUSG00000020651	protein_coding	solute carrier family 26, member 4 [Source:MGI Symbol;Acc:MGI:1346029]	<0.01	<0.01	-1
Cbx7	ENSMUSG00000053411	protein_coding	chromobox 7 [Source:MGI Symbol;Acc:MGI:1196439]	<0.01	<0.01	-1
Trpv4	ENSMUSG00000014158	protein_coding	transient receptor potential cation channel, subfamily V, member 4 [Source:MGI Symbol;Acc:MGI:1926945]	<0.01	0.021	-1
Ggps1	ENSMUSG00000021302	protein_coding	geranylgeranyl diphosphate synthase 1 [Source:MGI Symbol;Acc:MGI:1341724]	<0.01	0.015	-1
Kcnj15	ENSMUSG00000062609	protein_coding	potassium inwardly-rectifying channel, subfamily J, member 15 [Source:MGI Symbol;Acc:MGI:1310000]	<0.01	0.034	-1
Casc3	ENSMUSG00000078676	protein_coding	cancer susceptibility candidate 3 [Source:MGI Symbol;Acc:MGI:2179723]	<0.01	0.050	-1
Lama3	ENSMUSG00000024421	protein_coding	laminin, alpha 3 [Source:MGI Symbol;Acc:MGI:99909]	<0.01	0.021	-1
Otud3	ENSMUSG00000041161	protein_coding	OTU domain containing 3 [Source:MGI Symbol;Acc:MGI:1920412]	<0.01	0.030	-1
Dnajb4	ENSMUSG00000028035	protein_coding	DnaJ heat shock protein family (Hsp40) member B4 [Source:MGI Symbol;Acc:MGI:1914285]	<0.01	0.034	-1
Adnp	ENSMUSG00000051149	protein_coding	activity-dependent neuroprotective protein [Source:MGI Symbol;Acc:MGI:1338758]	<0.01	0.031	-1
44442	ENSMUSG00000022456	protein_coding	septin 3 [Source:MGI Symbol;Acc:MGI:1345148]	<0.01	0.050	-1
Arid5b	ENSMUSG00000019947	protein_coding	AT rich interactive domain 5B (MRF1-like) [Source:MGI Symbol;Acc:MGI:2175912]	<0.01	0.041	-1
Gga3	ENSMUSG00000020740	protein_coding	golgi associated, gamma adaptin ear containing, ARF binding protein 3 [Source:MGI Symbol;Acc:MGI:2384159]	<0.01	0.022	-1
Igkv8-24	ENSMUSG00000076583	IG_V_gene	immunoglobulin kappa chain variable 8-24 [Source:MGI Symbol;Acc:MGI:4947958]	<0.01	0.049	-1
Qsox1	ENSMUSG00000033684	protein_coding	quiescin Q6 sulfhydryl oxidase 1 [Source:MGI Symbol;Acc:MGI:1330818]	<0.01	0.033	-1
Timp3	ENSMUSG00000020044	protein_coding	tissue inhibitor of metalloproteinase 3 [Source:MGI Symbol;Acc:MGI:98754]	<0.01	0.030	-1
Klf4	ENSMUSG00000003032	protein_coding	Kruppel-like factor 4 (gut) [Source:MGI Symbol;Acc:MGI:1342287]	<0.01	0.027	-1
Man2b2	ENSMUSG00000029119	protein_coding	mannosidase 2, alpha B2 [Source:MGI Symbol;Acc:MGI:1195262]	<0.01	0.045	-1
Slc31a1	ENSMUSG00000066150	protein_coding	solute carrier family 31, member 1 [Source:MGI Symbol;Acc:MGI:1333843]	<0.01	0.043	-1
Spsb1	ENSMUSG00000039911	protein_coding	splA/ryanodine receptor domain and SOCS box containing 1 [Source:MGI Symbol;Acc:MGI:1921896]	<0.01	0.017	-1
Ammecr1l	ENSMUSG00000041915	protein_coding	AMME chromosomal region gene 1-like [Source:MGI Symbol;Acc:MGI:2442711]	<0.01	0.031	-1
Islr	ENSMUSG00000037206	protein_coding	immunoglobulin superfamily containing leucine-rich repeat [Source:MGI Symbol;Acc:MGI:1349645]	<0.01	0.022	-1
Gm11235	ENSMUSG00000083372	processed_pseudogene	predicted gene 11235 [Source:MGI Symbol;Acc:MGI:3651442]	<0.01	0.018	-1
Alas1	ENSMUSG00000032786	protein_coding	aminolevulinic acid synthase 1 [Source:MGI Symbol;Acc:MGI:87989]	<0.01	<0.01	-1
Dgat2	ENSMUSG00000030747	protein_coding	diacylglycerol O-acyltransferase 2 [Source:MGI Symbol;Acc:MGI:1915050]	<0.01	0.035	-1
Bag3	ENSMUSG00000030847	protein_coding	BCL2-associated athanogene 3 [Source:MGI Symbol;Acc:MGI:1352493]	<0.01	0.040	-1
G630022F23Rik	ENSMUSG00000106896	TEC	RIKEN cDNA G630022F23 gene [Source:MGI Symbol;Acc:MGI:3704248]	<0.01	0.049	-1
Dkk3	ENSMUSG00000030772	protein_coding	dickkopf WNT signaling pathway inhibitor 3 [Source:MGI Symbol;Acc:MGI:1354952]	<0.01	0.030	-1
Gm42457	ENSMUSG00000105339	antisense_RNA	predicted gene 42457 [Source:MGI Symbol;Acc:MGI:5662594]	<0.01	0.027	-1
Klf2	ENSMUSG00000055148	protein_coding	Kruppel-like factor 2 (lung) [Source:MGI Symbol;Acc:MGI:1342772]	<0.01	0.048	-1
Abi3bp	ENSMUSG00000035258	protein_coding	ABI gene family, member 3 (NESH) binding protein [Source:MGI Symbol;Acc:MGI:2444583]	<0.01	<0.01	-1
Gpr146	ENSMUSG00000044197	protein_coding	G protein-coupled receptor 146 [Source:MGI Symbol;Acc:MGI:1933113]	<0.01	0.014	-1
Aebp1	ENSMUSG00000020473	protein_coding	AE binding protein 1 [Source:MGI Symbol;Acc:MGI:1197012]	<0.01	0.042	-1
Icosl	ENSMUSG00000000732	protein_coding	icos ligand [Source:MGI Symbol;Acc:MGI:1354701]	<0.01	0.013	-1
Ube3b	ENSMUSG00000029577	protein_coding	ubiquitin protein ligase E3B [Source:MGI Symbol;Acc:MGI:1891295]	<0.01	0.028	-1

Nfe2l2	ENSMUSG00000015839	protein_coding	nuclear factor, erythroid derived 2, like 2 [Source:MGI Symbol;Acc:MGI:108420]	<0.01	0.027	-1
Adamts1	ENSMUSG00000022893	protein_coding	a disintegrin-like and metallopeptidase (reprolysin type) with thrombospondin type 1 motif, 1 [Source:MGI Symbol;Acc:MGI:109249]	<0.01	0.021	-1
Fam107b	ENSMUSG00000026655	protein_coding	family with sequence similarity 107, member B [Source:MGI Symbol;Acc:MGI:1913790]	<0.01	0.013	-1
Pamr1	ENSMUSG00000027188	protein_coding	peptidase domain containing associated with muscle regeneration 1 [Source:MGI Symbol;Acc:MGI:2445082]	<0.01	0.033	-1
Slc23a2	ENSMUSG00000027340	protein_coding	solute carrier family 23 (nucleobase transporters), member 2 [Source:MGI Symbol;Acc:MGI:1859682]	<0.01	0.041	-1
Papd5	ENSMUSG00000036779	protein_coding	PAP associated domain containing 5 [Source:MGI Symbol;Acc:MGI:1917820]	<0.01	0.040	-1
Dnah10	ENSMUSG00000038011	protein_coding	dynein, axonemal, heavy chain 10 [Source:MGI Symbol;Acc:MGI:1860299]	<0.01	0.021	-1
Herpud1	ENSMUSG00000031770	protein_coding	homocysteine-inducible, endoplasmic reticulum stress-inducible, ubiquitin-like domain member 1 [Source:MGI Symbol;Acc:MGI:1927406]	<0.01	0.014	-1
Cry2	ENSMUSG00000068742	protein_coding	cryptochrome 2 (photolyase-like) [Source:MGI Symbol;Acc:MGI:1270859]	<0.01	0.010	-1
Scnn1a	ENSMUSG00000030340	protein_coding	sodium channel, nonvoltage-gated 1 alpha [Source:MGI Symbol;Acc:MGI:101782]	<0.01	0.046	-1
8430408G22Rik	ENSMUSG00000048489	protein_coding	RIKEN cDNA 8430408G22 gene [Source:MGI Symbol;Acc:MGI:1918730]	<0.01	0.028	-1
Dusp2	ENSMUSG00000027368	protein_coding	dual specificity phosphatase 2 [Source:MGI Symbol;Acc:MGI:101911]	<0.01	0.017	-1
Per1	ENSMUSG00000020893	protein_coding	period circadian clock 1 [Source:MGI Symbol;Acc:MGI:1098283]	<0.01	<0.01	-1
Cst7	ENSMUSG00000068129	protein_coding	cystatin F (leukocystatin) [Source:MGI Symbol;Acc:MGI:1298217]	<0.01	0.015	-1
Zbtb16	ENSMUSG00000066687	protein_coding	zinc finger and BTB domain containing 16 [Source:MGI Symbol;Acc:MGI:103222]	<0.01	<0.01	-1
Gm2147	ENSMUSG00000103355	processed_pseudogene	predicted gene 2147 [Source:MGI Symbol;Acc:MGI:3780316]	<0.01	0.043	-1
Ltbp4	ENSMUSG00000040488	protein_coding	latent transforming growth factor beta binding protein 4 [Source:MGI Symbol;Acc:MGI:1321395]	<0.01	0.018	-1
Nrarp	ENSMUSG00000078202	protein_coding	Notch-regulated ankyrin repeat protein [Source:MGI Symbol;Acc:MGI:1914372]	<0.01	0.022	-1
Gm5319	ENSMUSG00000082782	processed_pseudogene	predicted gene 5319 [Source:MGI Symbol;Acc:MGI:3645267]	<0.01	0.044	-1
Trib1	ENSMUSG00000032501	protein_coding	tribbles pseudokinase 1 [Source:MGI Symbol;Acc:MGI:2443397]	<0.01	0.024	-1
Xpnpep3	ENSMUSG00000022401	protein_coding	X-prolyl aminopeptidase 3, mitochondrial [Source:MGI Symbol;Acc:MGI:2445217]	<0.01	0.030	-1
Usp2	ENSMUSG00000032010	protein_coding	ubiquitin specific peptidase 2 [Source:MGI Symbol;Acc:MGI:1858178]	<0.01	0.016	-1
Kif1b	ENSMUSG00000063077	protein_coding	kinesin family member 1B [Source:MGI Symbol;Acc:MGI:108426]	<0.01	0.038	-1
Nr4a1	ENSMUSG00000023034	protein_coding	nuclear receptor subfamily 4, group A, member 1 [Source:MGI Symbol;Acc:MGI:1352454]	<0.01	0.046	-1
Stbd1	ENSMUSG00000047963	protein_coding	starch binding domain 1 [Source:MGI Symbol;Acc:MGI:1261768]	<0.01	0.021	-1
Ank	ENSMUSG00000022265	protein_coding	progressive ankylosis [Source:MGI Symbol;Acc:MGI:3045421]	<0.01	0.046	-1
Gm10184	ENSMUSG00000066878	protein_coding	predicted pseudogene 10184 [Source:MGI Symbol;Acc:MGI:3704480]	<0.01	0.031	-1
Gpr171	ENSMUSG00000050075	protein_coding	G protein-coupled receptor 171 [Source:MGI Symbol;Acc:MGI:2442043]	<0.01	0.015	-1
Irx1	ENSMUSG00000060969	protein_coding	Iroquois related homeobox 1 (Drosophila) [Source:MGI Symbol;Acc:MGI:1197515]	<0.01	0.038	-1
Wee1	ENSMUSG00000031016	protein_coding	WEE 1 homolog 1 (S. pombe) [Source:MGI Symbol;Acc:MGI:103075]	<0.01	<0.01	-1
Klf5	ENSMUSG00000005148	protein_coding	Kruppel-like factor 5 [Source:MGI Symbol;Acc:MGI:1338056]	<0.01	0.016	-1
Ngp	ENSMUSG00000032484	protein_coding	neutrophilic granule protein [Source:MGI Symbol;Acc:MGI:105983]	<0.01	0.029	-1
Zfp36l2	ENSMUSG00000045817	protein_coding	zinc finger protein 36, C3H type-like 2 [Source:MGI Symbol;Acc:MGI:107945]	<0.01	0.041	-1
Fam46b	ENSMUSG00000046694	protein_coding	family with sequence similarity 46, member B [Source:MGI Symbol;Acc:MGI:2140500]	<0.01	0.041	-1
Arhgap27os2	ENSMUSG00000085360	antisense_RNA	Rho GTPase activating protein 27, opposite strand 2 [Source:MGI Symbol;Acc:MGI:3650160]	<0.01	0.029	-1
Etnk1	ENSMUSG00000030275	protein_coding	ethanolamine kinase 1 [Source:MGI Symbol;Acc:MGI:1922570]	<0.01	0.046	-1
Plat	ENSMUSG00000031538	protein_coding	plasminogen activator, tissue [Source:MGI Symbol;Acc:MGI:97610]	<0.01	0.020	-1
Gm3555	ENSMUSG00000089791	processed_pseudogene	predicted pseudogene 3555 [Source:MGI Symbol;Acc:MGI:3781732]	<0.01	0.034	-1
Rasl10b	ENSMUSG00000020684	protein_coding	RAS-like, family 10, member B [Source:MGI Symbol;Acc:MGI:2685575]	<0.01	0.029	-1
Gprc5b	ENSMUSG00000008734	protein_coding	G protein-coupled receptor, family C, group 5, member B [Source:MGI Symbol;Acc:MGI:1927596]	<0.01	<0.01	-1
Rgs1	ENSMUSG00000026358	protein_coding	regulator of G-protein signaling 1 [Source:MGI Symbol;Acc:MGI:1354694]	<0.01	0.015	-1
Grp	ENSMUSG00000024517	protein_coding	gastrin releasing peptide [Source:MGI Symbol;Acc:MGI:95833]	<0.01	0.039	-1
Slc38a4	ENSMUSG00000022464	protein_coding	solute carrier family 38, member 4 [Source:MGI Symbol;Acc:MGI:1916604]	<0.01	0.047	-1
Gm8812	ENSMUSG00000083289	processed_pseudogene	predicted gene 8812 [Source:MGI Symbol;Acc:MGI:3643091]	<0.01	<0.01	-1
Gm26527	ENSMUSG00000097582	lincRNA	predicted gene, 26527 [Source:MGI Symbol;Acc:MGI:5477021]	<0.01	0.041	-1
Bhlhe40	ENSMUSG00000030103	protein_coding	basic helix-loop-helix family, member e40 [Source:MGI Symbol;Acc:MGI:1097714]	<0.01	<0.01	-1
Slc38a2	ENSMUSG00000022462	protein_coding	solute carrier family 38, member 2 [Source:MGI Symbol;Acc:MGI:1915010]	<0.01	0.046	-1
Brd1	ENSMUSG00000022387	protein_coding	bromodomain containing 1 [Source:MGI Symbol;Acc:MGI:1924161]	<0.01	0.036	-1
Tmem200b	ENSMUSG00000070720	protein_coding	transmembrane protein 200B [Source:MGI Symbol;Acc:MGI:3646343]	<0.01	<0.01	-1
Ahctf1	ENSMUSG00000026491	protein_coding	AT hook containing transcription factor 1 [Source:MGI Symbol;Acc:MGI:1915033]	<0.01	0.024	-1
Letm1	ENSMUSG00000005299	protein_coding	leucine zipper-EF-hand containing transmembrane protein 1 [Source:MGI Symbol;Acc:MGI:1932557]	<0.01	0.029	-1
Dusp1	ENSMUSG00000024190	protein_coding	dual specificity phosphatase 1 [Source:MGI Symbol;Acc:MGI:105120]	<0.01	<0.01	-1
Sele	ENSMUSG00000026582	protein_coding	selectin, endothelial cell [Source:MGI Symbol;Acc:MGI:98278]	<0.01	0.016	-1
Nanos1	ENSMUSG00000072437	protein_coding	nanos homolog 1 (Drosophila) [Source:MGI Symbol;Acc:MGI:2669254]	<0.01	0.050	-1
Atp13a3	ENSMUSG00000022533	protein_coding	ATPase type 13A3 [Source:MGI Symbol;Acc:MGI:2685387]	<0.01	0.029	-1
Pdxk	ENSMUSG00000032788	protein_coding	pyridoxal (pyridoxine, vitamin B6) kinase [Source:MGI Symbol;Acc:MGI:1351869]	<0.01	0.017	-1
Angptl2	ENSMUSG00000004105	protein_coding	angiopoietin-like 2 [Source:MGI Symbol;Acc:MGI:1347002]	<0.01	0.011	-1
Col14a1	ENSMUSG00000022371	protein_coding	collagen, type XIV, alpha 1 [Source:MGI Symbol;Acc:MGI:1341272]	<0.01	0.036	-1
Dusp8	ENSMUSG00000037887	protein_coding	dual specificity phosphatase 8 [Source:MGI Symbol;Acc:MGI:106626]	<0.01	0.045	-1
Ampd2	ENSMUSG00000027889	protein_coding	adenosine monophosphate deaminase 2 [Source:MGI Symbol;Acc:MGI:88016]	<0.01	0.026	-1
Sik1	ENSMUSG00000024042	protein_coding	salt inducible kinase 1 [Source:MGI Symbol;Acc:MGI:104754]	<0.01	0.015	-1
Olfml2b	ENSMUSG00000038463	protein_coding	olfactomedin-like 2B [Source:MGI Symbol;Acc:MGI:2443310]	<0.01	0.026	-1
Mef2d	ENSMUSG00000001419	protein_coding	myocyte enhancer factor 2D [Source:MGI Symbol;Acc:MGI:99533]	<0.01	0.014	-1
RP23-445E20.7	ENSMUSG00000108391	TEC	predicted gene 9768 [Source:MGI Symbol;Acc:MGI:3704239]	<0.01	0.040	-1
Plet1	ENSMUSG00000032068	protein_coding	placenta expressed transcript 1 [Source:MGI Symbol;Acc:MGI:1923759]	<0.01	0.047	-1
Pdlim5	ENSMUSG00000028273	protein_coding	PDZ and LIM domain 5 [Source:MGI Symbol;Acc:MGI:1927489]	<0.01	0.049	-1
Fam13a	ENSMUSG00000037709	protein_coding	family with sequence similarity 13, member A [Source:MGI Symbol;Acc:MGI:1889842]	<0.01	0.041	-1
Arl8a	ENSMUSG00000026426	protein_coding	ADP-ribosylation factor-like 8A [Source:MGI Symbol;Acc:MGI:1915974]	<0.01	0.036	-1
Papd7	ENSMUSG00000034575	protein_coding	PAP associated domain containing 7 [Source:MGI Symbol;Acc:MGI:2682295]	<0.01	0.031	-1
Gm15406	ENSMUSG00000085237	lincRNA	predicted gene 15406 [Source:MGI Symbol;Acc:MGI:3705112]	<0.01	0.017	-1
Gm26699	ENSMUSG00000097206	lincRNA	predicted gene, 26699 [Source:MGI Symbol;Acc:MGI:5477193]	<0.01	0.032	-1
Slc3a2	ENSMUSG00000010095	protein_coding	solute carrier family 3 (activators of dibasic and neutral amino acid transport), member 2 [Source:MGI Symbol;Acc:MGI:96955]	<0.01	0.044	-1
Smad6	ENSMUSG00000036867	protein_coding	SMAD family member 6 [Source:MGI Symbol;Acc:MGI:1336883]	<0.01	0.021	-1
Gdf10	ENSMUSG00000021943	protein_coding	growth differentiation factor 10 [Source:MGI Symbol;Acc:MGI:95684]	<0.01	0.016	-1
Plekha6	ENSMUSG00000041757	protein_coding	pleckstrin homology domain containing, family A member 6 [Source:MGI Symbol;Acc:MGI:2388662]	<0.01	<0.01	-1
Vasn	ENSMUSG00000039646	protein_coding	vasorin [Source:MGI Symbol;Acc:MGI:2177651]	<0.01	0.033	-1
Bhlhe41	ENSMUSG00000030256	protein_coding	basic helix-loop-helix family, member e41 [Source:MGI Symbol;Acc:MGI:1930704]	<0.01	<0.01	-1
Agmo	ENSMUSG00000050103	protein_coding	alkylglycerol monooxygenase [Source:MGI Symbol;Acc:MGI:2442495]	<0.01	<0.01	-1
Scel	ENSMUSG00000022123	protein_coding	sciellin [Source:MGI Symbol;Acc:MGI:1891228]	<0.01	0.030	-1
Nr1d1	ENSMUSG00000020889	protein_coding	nuclear receptor subfamily 1, group D, member 1 [Source:MGI Symbol;Acc:MGI:2444210]	<0.01	<0.01	-1

Coq10b	ENSMUSG00000025981	protein_coding	coenzyme Q10B [Source:MGI Symbol;Acc:MGI:1915126]	<0.01	0.020	-1
Gm28044	ENSMUSG00000098781	protein_coding	predicted gene, 28044 [Source:MGI Symbol;Acc:MGI:5547780]	<0.01	<0.01	-1
Mthfd1l	ENSMUSG00000040675	protein_coding	methylenetetrahydrofolate dehydrogenase (NADP+ dependent) 1-like [Source:MGI Symbol;Acc:MGI:1924836]	<0.01	<0.01	-1
Jdp2	ENSMUSG00000034271	protein_coding	Jun dimerization protein 2 [Source:MGI Symbol;Acc:MGI:1932093]	<0.01	0.032	-1
Trim65	ENSMUSG00000054517	protein_coding	tripartite motif-containing 65 [Source:MGI Symbol;Acc:MGI:2442815]	<0.01	0.043	-1
Gm4759	ENSMUSG00000053541	unprocessed_pseudogene	predicted gene 4759 [Source:MGI Symbol;Acc:MGI:3647753]	<0.01	<0.01	-1
Hlf	ENSMUSG00000003949	protein_coding	hepatic leukemia factor [Source:MGI Symbol;Acc:MGI:96108]	<0.01	0.012	-1
Pknox2	ENSMUSG00000035934	protein_coding	Pbx/knotted 1 homeobox 2 [Source:MGI Symbol;Acc:MGI:2445415]	<0.01	<0.01	-1
St8sia2	ENSMUSG00000025789	protein_coding	ST8 alpha-N-acetyl-neuraminide alpha-2,8-sialyltransferase 2 [Source:MGI Symbol;Acc:MGI:106020]	<0.01	0.016	-1
Gsg1l	ENSMUSG00000046182	protein_coding	GSG1-like [Source:MGI Symbol;Acc:MGI:2685483]	<0.01	<0.01	-1
Cyp2b10	ENSMUSG00000030483	protein_coding	cytochrome P450, family 2, subfamily b, polypeptide 10 [Source:MGI Symbol;Acc:MGI:88598]	<0.01	0.026	-1
Ppp1r3a	ENSMUSG00000042717	protein_coding	protein phosphatase 1, regulatory (inhibitor) subunit 3A [Source:MGI Symbol;Acc:MGI:2153588]	<0.01	<0.01	-1
Per3	ENSMUSG00000028957	protein_coding	period circadian clock 3 [Source:MGI Symbol;Acc:MGI:1277134]	<0.01	<0.01	-1
Nt5e	ENSMUSG00000032420	protein_coding	5' nucleotidase, ecto [Source:MGI Symbol;Acc:MGI:99782]	<0.01	0.037	-1
Efnb2	ENSMUSG00000001300	protein_coding	ephrin B2 [Source:MGI Symbol;Acc:MGI:105097]	<0.01	0.035	-1
Gm26825	ENSMUSG00000097554	lincRNA	predicted gene, 26825 [Source:MGI Symbol;Acc:MGI:5477319]	<0.01	<0.01	-1
Cables1	ENSMUSG00000040957	protein_coding	CDK5 and Abl enzyme substrate 1 [Source:MGI Symbol;Acc:MGI:1927065]	<0.01	0.018	-1
Gpr155	ENSMUSG00000041762	protein_coding	G protein-coupled receptor 155 [Source:MGI Symbol;Acc:MGI:1915776]	<0.01	0.039	-1
Per2	ENSMUSG00000055866	protein_coding	period circadian clock 2 [Source:MGI Symbol;Acc:MGI:1195265]	<0.01	<0.01	-1
Efhd1	ENSMUSG00000026255	protein_coding	EF hand domain containing 1 [Source:MGI Symbol;Acc:MGI:1921607]	<0.01	<0.01	-1
Inafm2	ENSMUSG00000074918	protein_coding	InaF motif containing 2 [Source:MGI Symbol;Acc:MGI:1915354]	<0.01	0.018	-1
Dll4	ENSMUSG00000027314	protein_coding	delta-like 4 (Drosophila) [Source:MGI Symbol;Acc:MGI:1859388]	<0.01	0.023	-1
Man2a2	ENSMUSG00000038886	protein_coding	mannosidase 2, alpha 2 [Source:MGI Symbol;Acc:MGI:2150656]	<0.01	0.038	-1
Camsap1	ENSMUSG00000026933	protein_coding	calmodulin regulated spectrin-associated protein 1 [Source:MGI Symbol;Acc:MGI:3036242]	<0.01	0.038	-1
Tspan4	ENSMUSG00000025511	protein_coding	tetraspanin 4 [Source:MGI Symbol;Acc:MGI:1928097]	<0.01	<0.01	-1
Ighv2-6-8	ENSMUSG00000076646	IG_V_gene	immunoglobulin heavy variable 2-6-8 [Source:MGI Symbol;Acc:MGI:4439811]	<0.01	0.012	-1
Tnxb	ENSMUSG00000033327	protein_coding	tenascin XB [Source:MGI Symbol;Acc:MGI:1932137]	<0.01	<0.01	-1
Nbea	ENSMUSG00000027799	protein_coding	neurobeachin [Source:MGI Symbol;Acc:MGI:1347075]	<0.01	0.012	-1
Dbp	ENSMUSG00000059824	protein_coding	D site albumin promoter binding protein [Source:MGI Symbol;Acc:MGI:94866]	<0.01	<0.01	-1
Atxn1	ENSMUSG00000046876	protein_coding	ataxin 1 [Source:MGI Symbol;Acc:MGI:104783]	<0.01	0.021	-1
Sphk2	ENSMUSG00000057342	protein_coding	sphingosine kinase 2 [Source:MGI Symbol;Acc:MGI:1861380]	<0.01	<0.01	-1
Htr2b	ENSMUSG00000026228	protein_coding	5-hydroxytryptamine (serotonin) receptor 2B [Source:MGI Symbol;Acc:MGI:109323]	<0.01	<0.01	-1
Tef	ENSMUSG00000022389	protein_coding	thyrotroph embryonic factor [Source:MGI Symbol;Acc:MGI:98663]	<0.01	<0.01	-1
Cldn1	ENSMUSG00000022512	protein_coding	claudin 1 [Source:MGI Symbol;Acc:MGI:1276109]	<0.01	0.012	-1
Pthlh	ENSMUSG00000048776	protein_coding	parathyroid hormone-like peptide [Source:MGI Symbol;Acc:MGI:97800]	<0.01	<0.01	-1
Rps6kc1	ENSMUSG00000089872	protein_coding	ribosomal protein S6 kinase polypeptide 1 [Source:MGI Symbol;Acc:MGI:2443419]	<0.01	0.015	-1
Nr1d2	ENSMUSG00000021775	protein_coding	nuclear receptor subfamily 1, group D, member 2 [Source:MGI Symbol;Acc:MGI:2449205]	<0.01	<0.01	-1
Gm15459	ENSMUSG00000100801	processed_pseudogene	predicted gene 15459 [Source:MGI Symbol;Acc:MGI:3705702]	<0.01	<0.01	-1
Rnf43	ENSMUSG00000034177	protein_coding	ring finger protein 43 [Source:MGI Symbol;Acc:MGI:2442609]	<0.01	0.037	-1
Leo1	ENSMUSG00000042487	protein_coding	Leo1, Paf1/RNA polymerase II complex component [Source:MGI Symbol;Acc:MGI:2685031]	<0.01	<0.01	1
Gm4956	ENSMUSG00000025936	transcribed_unprocessed_pseudogene	predicted gene 4956 [Source:MGI Symbol;Acc:MGI:3647976]	<0.01	<0.01	1
F13a1	ENSMUSG00000039109	protein_coding	coagulation factor XIII, A1 subunit [Source:MGI Symbol;Acc:MGI:1921395]	<0.01	<0.01	1
Gm13889	ENSMUSG00000087006	protein_coding	predicted gene 13889 [Source:MGI Symbol;Acc:MGI:3652053]	<0.01	<0.01	1
Hrct1	ENSMUSG00000071001	protein_coding	histidine rich carboxyl terminus 1 [Source:MGI Symbol;Acc:MGI:1917945]	<0.01	<0.01	1
Neurl3	ENSMUSG00000047180	protein_coding	neuralized E3 ubiquitin protein ligase 3 [Source:MGI Symbol;Acc:MGI:2429944]	<0.01	<0.01	1
Dusp23	ENSMUSG00000026544	protein_coding	dual specificity phosphatase 23 [Source:MGI Symbol;Acc:MGI:1915690]	<0.01	0.012	1
Ms4a6c	ENSMUSG00000079419	protein_coding	membrane-spanning 4-domains, subfamily A, member 6C [Source:MGI Symbol;Acc:MGI:2385644]	<0.01	<0.01	1
Faap20	ENSMUSG00000073684	protein_coding	Fanconi anemia core complex associated protein 20 [Source:MGI Symbol;Acc:MGI:1914763]	<0.01	0.012	1
Mthfd1	ENSMUSG00000021048	protein_coding	methylenetetrahydrofolate dehydrogenase (NADP+ dependent), methenyltetrahydrofolate cyclohydrolase, formyltetrahydrofolate synthase [Source:MGI Symbol;Acc:MGI:1342005]	<0.01	<0.01	1
Angptl4	ENSMUSG00000002289	protein_coding	angiopoietin-like 4 [Source:MGI Symbol;Acc:MGI:1888999]	<0.01	0.017	1
Trpv6	ENSMUSG00000029868	protein_coding	transient receptor potential cation channel, subfamily V, member 6 [Source:MGI Symbol;Acc:MGI:1927259]	<0.01	<0.01	1
Rab24	ENSMUSG00000034789	protein_coding	RAB24, member RAS oncogene family [Source:MGI Symbol;Acc:MGI:105065]	<0.01	0.028	1
Apol11b	ENSMUSG00000091694	protein_coding	apolipoprotein L 11b [Source:MGI Symbol;Acc:MGI:3036248]	<0.01	0.020	1
I830127L07Rik	ENSMUSG00000102051	transcribed_unprocessed_pseudogene	RIKEN cDNA I830127L07 gene [Source:MGI Symbol;Acc:MGI:3612406]	<0.01	<0.01	1
Mplkip	ENSMUSG00000012429	protein_coding	M-phase specific PLK1 intereacting protein [Source:MGI Symbol;Acc:MGI:1913558]	<0.01	0.025	1
Sirpb1b	ENSMUSG00000095028	protein_coding	signal-regulatory protein beta 1B [Source:MGI Symbol;Acc:MGI:3779828]	<0.01	<0.01	1
Igkv4-86	ENSMUSG00000076536	IG_V_gene	immunoglobulin kappa variable 4-86 [Source:MGI Symbol;Acc:MGI:2685305]	<0.01	0.011	1
H2afj	ENSMUSG00000060032	protein_coding	H2A histone family, member J [Source:MGI Symbol;Acc:MGI:3606192]	<0.01	0.026	1
Mvb12a	ENSMUSG00000031813	protein_coding	multivesicular body subunit 12A [Source:MGI Symbol;Acc:MGI:1920961]	<0.01	0.021	1
Glipr2	ENSMUSG00000028480	protein_coding	GLI pathogenesis-related 2 [Source:MGI Symbol;Acc:MGI:1917770]	<0.01	<0.01	1
Tuft1	ENSMUSG00000005968	protein_coding	tuftelin 1 [Source:MGI Symbol;Acc:MGI:109572]	<0.01	0.034	1
Adra1a	ENSMUSG00000045875	protein_coding	adrenergic receptor, alpha 1a [Source:MGI Symbol;Acc:MGI:104773]	<0.01	<0.01	1
Susd3	ENSMUSG00000021384	protein_coding	sushi domain containing 3 [Source:MGI Symbol;Acc:MGI:1913579]	<0.01	0.018	1
Rtkn	ENSMUSG00000034930	protein_coding	rhotekin [Source:MGI Symbol;Acc:MGI:107371]	<0.01	0.032	1
Creb3l1	ENSMUSG00000027230	protein_coding	cAMP responsive element binding protein 3-like 1 [Source:MGI Symbol;Acc:MGI:1347062]	<0.01	0.012	1
Gm43198	ENSMUSG00000107222	TEC	predicted gene 43198 [Source:MGI Symbol;Acc:MGI:5663335]	<0.01	<0.01	1
Spon2	ENSMUSG00000037379	protein_coding	spondin 2, extracellular matrix protein [Source:MGI Symbol;Acc:MGI:1923724]	<0.01	<0.01	1
Arl5c	ENSMUSG00000038352	protein_coding	ADP-ribosylation factor-like 5C [Source:MGI Symbol;Acc:MGI:3028577]	<0.01	0.022	1
1110001J03Rik	ENSMUSG00000019689	protein_coding	formation of mitochondrial complex V assembly factor 1 [Source:MGI Symbol;Acc:MGI:1913367]	<0.01	<0.01	1
Dtx4	ENSMUSG00000039982	protein_coding	deltex 4, E3 ubiquitin ligase [Source:MGI Symbol;Acc:MGI:2672905]	<0.01	0.017	1
I830077J02Rik	ENSMUSG00000074342	protein_coding	RIKEN cDNA I830077J02 gene [Source:MGI Symbol;Acc:MGI:3588284]	<0.01	<0.01	1
Rasl11a	ENSMUSG00000029641	protein_coding	RAS-like, family 11, member A [Source:MGI Symbol;Acc:MGI:1916145]	<0.01	<0.01	1
Msc	ENSMUSG00000025930	protein_coding	musculin [Source:MGI Symbol;Acc:MGI:1333884]	<0.01	<0.01	1
Adm	ENSMUSG00000030790	protein_coding	adrenomedullin [Source:MGI Symbol;Acc:MGI:108058]	<0.01	<0.01	1
Asprv1	ENSMUSG00000033508	protein_coding	aspartic peptidase, retroviral-like 1 [Source:MGI Symbol;Acc:MGI:1915105]	<0.01	<0.01	1
Arntl	ENSMUSG00000055116	protein_coding	aryl hydrocarbon receptor nuclear translocator-like [Source:MGI Symbol;Acc:MGI:1096381]	<0.01	<0.01	1
Cnbd2	ENSMUSG00000038085	protein_coding	cyclic nucleotide binding domain containing 2 [Source:MGI Symbol;Acc:MGI:1918123]	<0.01	<0.01	1
Serpina3c	ENSMUSG00000066361	protein_coding	serine (or cysteine) peptidase inhibitor, clade A, member 3C [Source:MGI Symbol;Acc:MGI:102848]	<0.01	<0.01	1
Ms4a4c	ENSMUSG00000024675	protein_coding	membrane-spanning 4-domains, subfamily A, member 4C [Source:MGI Symbol;Acc:MGI:1927656]	<0.01	0.010	1
Apoc2	ENSMUSG00000002992	protein_coding	apolipoprotein C-II [Source:MGI Symbol;Acc:MGI:88054]	<0.01	<0.01	1

1810062G17Rik	ENSMUSG00000027713	protein_coding	RIKEN cDNA 1810062G17 gene [Source:MGI Symbol;Acc:MGI:1919532]	<0.01	<0.01	1
Amigo2	ENSMUSG00000048218	protein_coding	adhesion molecule with Ig like domain 2 [Source:MGI Symbol;Acc:MGI:2145995]	<0.01	0.010	1
Ccr1	ENSMUSG00000025804	protein_coding	chemokine (C-C motif) receptor 1 [Source:MGI Symbol;Acc:MGI:104618]	<0.01	0.030	1
Eif3m	ENSMUSG00000027170	protein_coding	eukaryotic translation initiation factor 3, subunit M [Source:MGI Symbol;Acc:MGI:1351744]	<0.01	0.017	1
Coa3	ENSMUSG00000017188	protein_coding	cytochrome C oxidase assembly factor 3 [Source:MGI Symbol;Acc:MGI:1098757]	<0.01	0.047	1
Mmp9	ENSMUSG00000017737	protein_coding	matrix metallopeptidase 9 [Source:MGI Symbol;Acc:MGI:97011]	<0.01	<0.01	1
Iah1	ENSMUSG00000062054	protein_coding	isoamyl acetate-hydrolyzing esterase 1 homolog [Source:MGI Symbol;Acc:MGI:1914982]	<0.01	<0.01	1
Eln	ENSMUSG00000029675	protein_coding	elastin [Source:MGI Symbol;Acc:MGI:95317]	<0.01	<0.01	1
Alkbh7	ENSMUSG00000002661	protein_coding	alkB homolog 7 [Source:MGI Symbol;Acc:MGI:1913650]	<0.01	0.028	1
H1f0	ENSMUSG00000096210	protein_coding	H1 histone family, member 0 [Source:MGI Symbol;Acc:MGI:95893]	<0.01	0.025	1
Egfem1	ENSMUSG00000063600	protein_coding	EGF-like and EMI domain containing 1 [Source:MGI Symbol;Acc:MGI:1922990]	<0.01	<0.01	1
Gcdh	ENSMUSG00000003809	protein_coding	glutaryl-Coenzyme A dehydrogenase [Source:MGI Symbol;Acc:MGI:104541]	<0.01	0.015	1
RP23-402A4.1	ENSMUSG00000108393	lincRNA	predicted gene, 32633 [Source:MGI Symbol;Acc:MGI:5591792]	<0.01	<0.01	1
Rps4l	ENSMUSG00000063171	transcribed_processed_pseudogene	ribosomal protein S4-like [Source:MGI Symbol;Acc:MGI:1913434]	<0.01	0.031	1
Lsm4	ENSMUSG00000031848	protein_coding	LSM4 homolog, U6 small nuclear RNA and mRNA degradation associated [Source:MGI Symbol;Acc:MGI:1354692]	<0.01	<0.01	1
Nfe2	ENSMUSG00000058794	protein_coding	nuclear factor, erythroid derived 2 [Source:MGI Symbol;Acc:MGI:97308]	<0.01	0.028	1
Ppbp	ENSMUSG00000029372	protein_coding	pro-platelet basic protein [Source:MGI Symbol;Acc:MGI:1888712]	<0.01	0.026	1
Npas2	ENSMUSG00000026077	protein_coding	neuronal PAS domain protein 2 [Source:MGI Symbol;Acc:MGI:109232]	<0.01	<0.01	1
Cd300a	ENSMUSG00000034652	protein_coding	CD300A molecule [Source:MGI Symbol;Acc:MGI:2443411]	<0.01	<0.01	1
Pycard	ENSMUSG00000030793	protein_coding	PYD and CARD domain containing [Source:MGI Symbol;Acc:MGI:1931465]	<0.01	0.012	1
Mmp13	ENSMUSG00000050578	protein_coding	matrix metallopeptidase 13 [Source:MGI Symbol;Acc:MGI:1340026]	<0.01	<0.01	1
Gm12942	ENSMUSG00000070737	protein_coding	transmembrane protein 35B [Source:MGI Symbol;Acc:MGI:3758095]	<0.01	0.020	1
Trim30b	ENSMUSG00000052749	protein_coding	tripartite motif-containing 30B [Source:MGI Symbol;Acc:MGI:4821256]	<0.01	<0.01	1
Klra2	ENSMUSG00000030187	protein_coding	killer cell lectin-like receptor, subfamily A, member 2 [Source:MGI Symbol;Acc:MGI:101906]	<0.01	<0.01	1
Nsmce1	ENSMUSG00000030750	protein_coding	NSE1 homolog, SMC5-SMC6 complex component [Source:MGI Symbol;Acc:MGI:1914961]	<0.01	0.027	1
Gjb2	ENSMUSG00000046352	protein_coding	gap junction protein, beta 2 [Source:MGI Symbol;Acc:MGI:95720]	<0.01	0.037	1
Igkv4-68	ENSMUSG00000076549	IG_V_gene	immunoglobulin kappa variable 4-68 [Source:MGI Symbol;Acc:MGI:2686265]	<0.01	0.020	1
Mrpl27	ENSMUSG00000024414	protein_coding	mitochondrial ribosomal protein L27 [Source:MGI Symbol;Acc:MGI:2137224]	<0.01	<0.01	1
Rpl27	ENSMUSG00000063316	protein_coding	ribosomal protein L27 [Source:MGI Symbol;Acc:MGI:98036]	<0.01	0.034	1
Rbm8a	ENSMUSG00000038374	protein_coding	RNA binding motif protein 8a [Source:MGI Symbol;Acc:MGI:1913129]	<0.01	0.037	1
Gm12582	ENSMUSG00000081705	processed_pseudogene	predicted gene 12582 [Source:MGI Symbol;Acc:MGI:3651489]	<0.01	0.026	1
2700046A07Rik	ENSMUSG00000041789	lincRNA	RIKEN cDNA 2700046A07 gene [Source:MGI Symbol;Acc:MGI:1919803]	<0.01	0.044	1
Gm7160	ENSMUSG00000099843	lincRNA	predicted gene 7160 [Source:MGI Symbol;Acc:MGI:3646078]	<0.01	0.038	1
Afap1l2	ENSMUSG00000025083	protein_coding	actin filament associated protein 1-like 2 [Source:MGI Symbol;Acc:MGI:2147658]	<0.01	0.035	1
Alkbh3	ENSMUSG00000040174	protein_coding	alkB homolog 3, alpha-ketoglutarate-dependent dioxygenase [Source:MGI Symbol;Acc:MGI:1916363]	<0.01	0.040	1
Schip1	ENSMUSG00000027777	protein_coding	schwannomin interacting protein 1 [Source:MGI Symbol;Acc:MGI:1353557]	<0.01	0.035	1
Vps53	ENSMUSG00000017288	protein_coding	VPS53 GARP complex subunit [Source:MGI Symbol;Acc:MGI:1915549]	<0.01	0.045	1
Trem3	ENSMUSG00000041754	protein_coding	triggering receptor expressed on myeloid cells 3 [Source:MGI Symbol;Acc:MGI:1930003]	<0.01	0.026	1
Uqcc3	ENSMUSG00000071654	protein_coding	ubiquinol-cytochrome c reductase complex assembly factor 3 [Source:MGI Symbol;Acc:MGI:2147553]	<0.01	0.042	1
Gm26735	ENSMUSG00000097461	lincRNA	predicted gene, 26735 [Source:MGI Symbol;Acc:MGI:5477229]	<0.01	0.038	1
Ccdc28b	ENSMUSG00000028795	protein_coding	coiled coil domain containing 28B [Source:MGI Symbol;Acc:MGI:1913514]	<0.01	0.050	1
Stx8	ENSMUSG00000020903	protein_coding	syntaxin 8 [Source:MGI Symbol;Acc:MGI:1890156]	<0.01	0.028	1
Lars2	ENSMUSG00000035202	protein_coding	leucyl-tRNA synthetase, mitochondrial [Source:MGI Symbol;Acc:MGI:2142973]	<0.01	0.025	1
Ikzf4	ENSMUSG00000002578	protein_coding	IKAROS family zinc finger 4 [Source:MGI Symbol;Acc:MGI:1343139]	<0.01	0.019	1
Ier3ip1	ENSMUSG00000090000	protein_coding	immediate early response 3 interacting protein 1 [Source:MGI Symbol;Acc:MGI:1913441]	<0.01	0.033	1
Serpina3h	ENSMUSG00000041449	transcribed_unprocessed_pseudogene	serine (or cysteine) peptidase inhibitor, clade A, member 3H [Source:MGI Symbol;Acc:MGI:2182839]	<0.01	0.034	1
Ccdc58	ENSMUSG00000075229	protein_coding	coiled-coil domain containing 58 [Source:MGI Symbol;Acc:MGI:2146423]	<0.01	<0.01	1
Lox	ENSMUSG00000024529	protein_coding	lysyl oxidase [Source:MGI Symbol;Acc:MGI:96817]	<0.01	0.013	1
Evi2a	ENSMUSG00000078771	protein_coding	ecotropic viral integration site 2a [Source:MGI Symbol;Acc:MGI:95458]	<0.01	0.037	1
Nudt16	ENSMUSG00000032565	protein_coding	nudix (nucleoside diphosphate linked moiety X)-type motif 16 [Source:MGI Symbol;Acc:MGI:1922936]	<0.01	0.014	1
Zfp719	ENSMUSG00000030469	protein_coding	zinc finger protein 719 [Source:MGI Symbol;Acc:MGI:2444708]	<0.01	0.019	1
Vstm4	ENSMUSG00000050666	protein_coding	V-set and transmembrane domain containing 4 [Source:MGI Symbol;Acc:MGI:2444633]	<0.01	0.017	1
Mthfs	ENSMUSG00000066442	protein_coding	5, 10-methenyltetrahydrofolate synthetase [Source:MGI Symbol;Acc:MGI:1340032]	<0.01	0.044	1
Gm42929	ENSMUSG00000106022	TEC	predicted gene 42929 [Source:MGI Symbol;Acc:MGI:5663066]	<0.01	0.040	1
Gtf3c6	ENSMUSG00000019837	protein_coding	general transcription factor IIIC, polypeptide 6, alpha [Source:MGI Symbol;Acc:MGI:1914621]	<0.01	0.040	1
Wfdc1	ENSMUSG00000023336	protein_coding	WAP four-disulfide core domain 1 [Source:MGI Symbol;Acc:MGI:1915116]	<0.01	0.045	1
Abt1	ENSMUSG00000036376	protein_coding	activator of basal transcription 1 [Source:MGI Symbol;Acc:MGI:1353636]	<0.01	0.041	1
Mfap2	ENSMUSG00000060572	protein_coding	microfibrillar-associated protein 2 [Source:MGI Symbol;Acc:MGI:99559]	<0.01	0.044	1
2010320M18Rik	ENSMUSG00000100691	lincRNA	RIKEN cDNA 2010320M18 gene [Source:MGI Symbol;Acc:MGI:1919343]	<0.01	0.029	1
Gm17046	ENSMUSG00000091613	processed_pseudogene	predicted gene 17046 [Source:MGI Symbol;Acc:MGI:4937873]	<0.01	0.025	1
Snhg18	ENSMUSG00000096956	lincRNA	small nucleolar RNA host gene 18 [Source:MGI Symbol;Acc:MGI:1914085]	<0.01	0.049	1
Emc9	ENSMUSG00000022217	protein_coding	ER membrane protein complex subunit 9 [Source:MGI Symbol;Acc:MGI:1934682]	<0.01	0.038	1
Dera	ENSMUSG00000030225	protein_coding	deoxyribose-phosphate aldolase (putative) [Source:MGI Symbol;Acc:MGI:1913762]	<0.01	0.043	1
Gap43	ENSMUSG00000047261	protein_coding	growth associated protein 43 [Source:MGI Symbol;Acc:MGI:95639]	<0.01	0.042	1
Socs1	ENSMUSG00000038037	protein_coding	suppressor of cytokine signaling 1 [Source:MGI Symbol;Acc:MGI:1354910]	<0.01	0.050	1
Alg9	ENSMUSG00000032059	protein_coding	asparagine-linked glycosylation 9 (alpha 1,2 mannosyltransferase) [Source:MGI Symbol;Acc:MGI:1924753]	<0.01	0.036	1
Nat2	ENSMUSG00000051147	protein_coding	N-acetyltransferase 2 (arylamine N-acetyltransferase) [Source:MGI Symbol;Acc:MGI:109201]	<0.01	0.047	1
Ccdc166	ENSMUSG00000098176	protein_coding	coiled-coil domain containing 166 [Source:MGI Symbol;Acc:MGI:1925902]	<0.01	0.039	1
Fam220a	ENSMUSG00000083012	protein_coding	family with sequence similarity 220, member A [Source:MGI Symbol;Acc:MGI:1914488]	<0.01	0.038	1
Slc25a38	ENSMUSG00000032519	protein_coding	solute carrier family 25, member 38 [Source:MGI Symbol;Acc:MGI:2384782]	<0.01	0.028	1
Ypel2	ENSMUSG00000018427	protein_coding	yippee-like 2 (Drosophila) [Source:MGI Symbol;Acc:MGI:1925114]	<0.01	0.017	1
Gm26549	ENSMUSG00000097217	lincRNA	predicted gene, 26549 [Source:MGI Symbol;Acc:MGI:5477043]	<0.01	0.029	1
Ccdc142os	ENSMUSG00000087578	antisense_RNA	coiled-coil domain containing 142, opposite strand [Source:MGI Symbol;Acc:MGI:3783052]	<0.01	0.035	1
Dusp22	ENSMUSG00000069255	protein_coding	dual specificity phosphatase 22 [Source:MGI Symbol;Acc:MGI:1915926]	<0.01	<0.01	1
Gm12732	ENSMUSG00000082646	processed_pseudogene	predicted gene 12732 [Source:MGI Symbol;Acc:MGI:3650211]	<0.01	0.030	1
Foxs1	ENSMUSG00000074676	protein_coding	forkhead box S1 [Source:MGI Symbol;Acc:MGI:95546]	<0.01	0.011	1
Ap2s1	ENSMUSG00000008036	protein_coding	adaptor-related protein complex 2, sigma 1 subunit [Source:MGI Symbol;Acc:MGI:2141861]	<0.01	0.045	1
Klk8	ENSMUSG00000064023	protein_coding	kallikrein related-peptidase 8 [Source:MGI Symbol;Acc:MGI:1343327]	<0.01	<0.01	1
4931428F04Rik	ENSMUSG00000014837	protein_coding	RIKEN cDNA 4931428F04 gene [Source:MGI Symbol;Acc:MGI:1921606]	<0.01	0.020	1
Rps16	ENSMUSG00000037563	protein_coding	ribosomal protein S16 [Source:MGI Symbol;Acc:MGI:98118]	<0.01	0.024	1

Zfp455	ENSMUSG00000051037	protein_coding	zinc finger protein 455 [Source:MGI Symbol;Acc:MGI:3040708]	<0.01	0.029	1
Mrpl21	ENSMUSG00000024829	protein_coding	mitochondrial ribosomal protein L21 [Source:MGI Symbol;Acc:MGI:2660674]	<0.01	0.032	1
Tmem42	ENSMUSG00000066233	protein_coding	transmembrane protein 42 [Source:MGI Symbol;Acc:MGI:1277176]	<0.01	0.036	1
Rftn2	ENSMUSG00000025978	protein_coding	raftlin family member 2 [Source:MGI Symbol;Acc:MGI:1921263]	<0.01	0.034	1
Slc40a1	ENSMUSG00000025993	protein_coding	solute carrier family 40 (iron-regulated transporter), member 1 [Source:MGI Symbol;Acc:MGI:1315204]	<0.01	<0.01	1
H3f3aos	ENSMUSG00000073485	antisense_RNA	H3 histone, family 3A, opposite strand [Source:MGI Symbol;Acc:MGI:3802006]	<0.01	0.047	1
Gng11	ENSMUSG00000032766	protein_coding	guanine nucleotide binding protein (G protein), gamma 11 [Source:MGI Symbol;Acc:MGI:1913316]	<0.01	0.030	1
Lipt1	ENSMUSG00000037216	protein_coding	lipoyltransferase 1 [Source:MGI Symbol;Acc:MGI:3645211]	<0.01	0.031	1
Gm43672	ENSMUSG00000106019	lincRNA	predicted gene 43672 [Source:MGI Symbol;Acc:MGI:5663809]	<0.01	<0.01	1
1500011K16Rik	ENSMUSG00000051319	protein_coding	RIKEN cDNA 1500011K16 gene [Source:MGI Symbol;Acc:MGI:1915135]	<0.01	0.042	1
Snca	ENSMUSG00000025889	protein_coding	synuclein, alpha [Source:MGI Symbol;Acc:MGI:1277151]	<0.01	0.029	1
Gm8242	ENSMUSG00000102827	transcribed_processed_pseudogene	predicted gene 8242 [Source:MGI Symbol;Acc:MGI:3647216]	<0.01	0.023	1
Gm26917	ENSMUSG00000097971	lincRNA	predicted gene, 26917 [Source:MGI Symbol;Acc:MGI:5504032]	<0.01	0.018	1
Gimap6	ENSMUSG00000047867	protein_coding	GTPase, IMAP family member 6 [Source:MGI Symbol;Acc:MGI:1918876]	<0.01	0.019	1
Coa4	ENSMUSG00000044881	protein_coding	cytochrome c oxidase assembly factor 4 [Source:MGI Symbol;Acc:MGI:1915435]	<0.01	0.016	1
Serpina3g	ENSMUSG00000041481	protein_coding	serine (or cysteine) peptidase inhibitor, clade A, member 3G [Source:MGI Symbol;Acc:MGI:105046]	<0.01	<0.01	1
Bloc1s2	ENSMUSG00000057506	protein_coding	biogenesis of lysosomal organelles complex-1, subunit 2 [Source:MGI Symbol;Acc:MGI:1920939]	<0.01	0.046	1
4930555A03Rik	ENSMUSG00000106157	lincRNA	RIKEN cDNA 4930555A03 gene [Source:MGI Symbol;Acc:MGI:1922587]	<0.01	0.024	1
Gm15512	ENSMUSG00000087639	antisense_RNA	predicted gene 15512 [Source:MGI Symbol;Acc:MGI:3782960]	<0.01	0.035	1
Scx	ENSMUSG00000034161	protein_coding	scleraxis [Source:MGI Symbol;Acc:MGI:102934]	<0.01	0.033	1
Arrdc4	ENSMUSG00000042659	protein_coding	arrestin domain containing 4 [Source:MGI Symbol;Acc:MGI:1913662]	<0.01	0.045	1
5430416O09Rik	ENSMUSG00000028475	protein_coding	small regulatory polypeptide of amino acid response [Source:MGI Symbol;Acc:MGI:1918656]	<0.01	0.012	1
Gm15420	ENSMUSG00000087387	antisense_RNA	predicted gene 15420 [Source:MGI Symbol;Acc:MGI:3705302]	<0.01	0.045	1
Phyhd1	ENSMUSG00000079484	protein_coding	phytanoyl-CoA dioxygenase domain containing 1 [Source:MGI Symbol;Acc:MGI:3612860]	<0.01	0.040	1
Gadd45a	ENSMUSG00000036390	protein_coding	growth arrest and DNA-damage-inducible 45 alpha [Source:MGI Symbol;Acc:MGI:107799]	<0.01	0.035	1
Fam181b	ENSMUSG00000051515	protein_coding	family with sequence similarity 181, member B [Source:MGI Symbol;Acc:MGI:1930951]	<0.01	0.046	1
Blvra	ENSMUSG00000001999	protein_coding	biliverdin reductase A [Source:MGI Symbol;Acc:MGI:88170]	<0.01	0.043	1
Tsr3	ENSMUSG00000015126	protein_coding	TSR3 20S rRNA accumulation [Source:MGI Symbol;Acc:MGI:1915577]	<0.01	0.035	1
Derl2	ENSMUSG00000018442	protein_coding	Der1-like domain family, member 2 [Source:MGI Symbol;Acc:MGI:2151483]	<0.01	0.038	1
Gm6257	ENSMUSG00000091866	processed_pseudogene	predicted gene 6257 [Source:MGI Symbol;Acc:MGI:3645370]	<0.01	0.018	1
Ckb	ENSMUSG00000001270	protein_coding	creatine kinase, brain [Source:MGI Symbol;Acc:MGI:88407]	<0.01	0.017	1
Cstad	ENSMUSG00000047363	protein_coding	CSA-conditional, T cell activation-dependent protein [Source:MGI Symbol;Acc:MGI:1925867]	<0.01	0.013	1
Vamp8	ENSMUSG00000050732	protein_coding	vesicle-associated membrane protein 8 [Source:MGI Symbol;Acc:MGI:1336882]	<0.01	0.047	1
2010315B03Rik	ENSMUSG00000074829	protein_coding	RIKEN cDNA 2010315B03 gene [Source:MGI Symbol;Acc:MGI:1919321]	<0.01	0.042	1
Zfp784	ENSMUSG00000043290	protein_coding	zinc finger protein 784 [Source:MGI Symbol;Acc:MGI:3606042]	<0.01	0.049	1
Cdkn2b	ENSMUSG00000073802	protein_coding	cyclin-dependent kinase inhibitor 2B (p15, inhibits CDK4) [Source:MGI Symbol;Acc:MGI:104737]	<0.01	0.028	1
Cass4	ENSMUSG00000074570	protein_coding	Cas scaffolding protein family member 4 [Source:MGI Symbol;Acc:MGI:2444482]	<0.01	0.049	1
Stambpl1	ENSMUSG00000024776	protein_coding	STAM binding protein like 1 [Source:MGI Symbol;Acc:MGI:1923880]	<0.01	0.043	1
Gtpbp10	ENSMUSG00000040464	protein_coding	GTP-binding protein 10 (putative) [Source:MGI Symbol;Acc:MGI:2385599]	<0.01	0.044	1
Gm4944	ENSMUSG00000096433	protein_coding	zinc finger protein 994 [Source:MGI Symbol;Acc:MGI:3643318]	<0.01	0.034	1
Cwc27	ENSMUSG00000021715	protein_coding	CWC27 spliceosome-associated protein [Source:MGI Symbol;Acc:MGI:1914535]	<0.01	0.015	1
Cyp1a1	ENSMUSG00000032315	protein_coding	cytochrome P450, family 1, subfamily a, polypeptide 1 [Source:MGI Symbol;Acc:MGI:88588]	<0.01	0.021	1
Gm27209	ENSMUSG00000098620	antisense_RNA	predicted gene 27209 [Source:MGI Symbol;Acc:MGI:5521052]	<0.01	0.050	1
Lekr1	ENSMUSG00000074579	protein_coding	leucine, glutamate and lysine rich 1 [Source:MGI Symbol;Acc:MGI:3645902]	<0.01	0.032	1
Zfp472	ENSMUSG00000053600	protein_coding	zinc finger protein 472 [Source:MGI Symbol;Acc:MGI:2385049]	<0.01	0.041	1
Rasgrp2	ENSMUSG00000032946	protein_coding	RAS, guanyl releasing protein 2 [Source:MGI Symbol;Acc:MGI:1333849]	<0.01	0.045	1
Gpatch11	ENSMUSG00000050668	protein_coding	G patch domain containing 11 [Source:MGI Symbol;Acc:MGI:1858435]	<0.01	0.035	1
2610524H06Rik	ENSMUSG00000092486	protein_coding	RIKEN cDNA 2610524H06 gene [Source:MGI Symbol;Acc:MGI:2447819]	<0.01	0.026	1
Rps27a	ENSMUSG00000020460	protein_coding	ribosomal protein S27A [Source:MGI Symbol;Acc:MGI:1925544]	<0.01	0.029	1
B4galt7	ENSMUSG00000021504	protein_coding	xylosylprotein beta1,4-galactosyltransferase, polypeptide 7 (galactosyltransferase I) [Source:MGI Symbol;Acc:MGI:2384987]	<0.01	<0.01	1
Gm28731	ENSMUSG00000101555	antisense_RNA	predicted gene 28731 [Source:MGI Symbol;Acc:MGI:5579437]	<0.01	0.015	1
Slamf1	ENSMUSG00000015316	protein_coding	signaling lymphocytic activation molecule family member 1 [Source:MGI Symbol;Acc:MGI:1351314]	<0.01	0.031	1
A930004D18Rik	ENSMUSG00000054057	protein_coding	RIKEN cDNA A930004D18 gene [Source:MGI Symbol;Acc:MGI:1925190]	<0.01	0.024	1
Thoc7	ENSMUSG00000053453	protein_coding	THO complex 7 [Source:MGI Symbol;Acc:MGI:1913481]	<0.01	0.046	1
Gm11537	ENSMUSG00000085242	antisense_RNA	predicted gene 11537 [Source:MGI Symbol;Acc:MGI:3705160]	<0.01	0.036	1
1700123O20Rik	ENSMUSG00000040822	protein_coding	RIKEN cDNA 1700123O20 gene [Source:MGI Symbol;Acc:MGI:1920893]	<0.01	0.031	1
Smim12	ENSMUSG00000042380	protein_coding	small integral membrane protein 12 [Source:MGI Symbol;Acc:MGI:1933141]	<0.01	0.026	1
Fam20a	ENSMUSG00000020614	protein_coding	family with sequence similarity 20, member A [Source:MGI Symbol;Acc:MGI:2388266]	<0.01	0.021	1
Zeb2os	ENSMUSG00000052248	antisense_RNA	zinc finger E-box binding homeobox 2, opposite strand [Source:MGI Symbol;Acc:MGI:3652108]	<0.01	0.042	1
Nnat	ENSMUSG00000067786	protein_coding	neuronatin [Source:MGI Symbol;Acc:MGI:104716]	<0.01	0.049	1
Pfdn6	ENSMUSG00000024309	protein_coding	prefoldin subunit 6 [Source:MGI Symbol;Acc:MGI:95908]	<0.01	0.034	1
Zfp593	ENSMUSG00000028840	protein_coding	zinc finger protein 593 [Source:MGI Symbol;Acc:MGI:1915290]	<0.01	0.035	1
S100a16	ENSMUSG00000074457	protein_coding	S100 calcium binding protein A16 [Source:MGI Symbol;Acc:MGI:1915110]	<0.01	0.015	1
Gmfg	ENSMUSG00000060791	protein_coding	glia maturation factor, gamma [Source:MGI Symbol;Acc:MGI:1927135]	<0.01	0.021	1
Isg15	ENSMUSG00000035692	protein_coding	ISG15 ubiquitin-like modifier [Source:MGI Symbol;Acc:MGI:1855694]	<0.01	0.040	1
Dhrs7b	ENSMUSG00000042569	protein_coding	dehydrogenase/reductase (SDR family) member 7B [Source:MGI Symbol;Acc:MGI:2384931]	<0.01	0.045	1
Rmrp	ENSMUSG00000088088	ribozyme	RNA component of mitochondrial RNAase P [Source:MGI Symbol;Acc:MGI:97937]	<0.01	<0.01	1
Ccdc112	ENSMUSG00000071855	protein_coding	coiled-coil domain containing 112 [Source:MGI Symbol;Acc:MGI:1918800]	<0.01	0.021	1
2610306M01Rik	ENSMUSG00000100164	bidirectional_promoter_lncRNA	RIKEN cDNA 2610306M01 gene [Source:MGI Symbol;Acc:MGI:1914420]	<0.01	0.029	1
Gadd45g	ENSMUSG00000021453	protein_coding	growth arrest and DNA-damage-inducible 45 gamma [Source:MGI Symbol;Acc:MGI:1346325]	<0.01	<0.01	1
Rpph1	ENSMUSG00000092837	ribozyme	ribonuclease P RNA component H1 [Source:MGI Symbol;Acc:MGI:1934664]	<0.01	0.032	1
Hoxa2	ENSMUSG00000014704	protein_coding	homeobox A2 [Source:MGI Symbol;Acc:MGI:96174]	<0.01	0.013	1
Tekt5	ENSMUSG00000039179	protein_coding	tektin 5 [Source:MGI Symbol;Acc:MGI:1917676]	<0.01	0.028	1
2310039H08Rik	ENSMUSG00000062619	protein_coding	RIKEN cDNA 2310039H08 gene [Source:MGI Symbol;Acc:MGI:1914351]	<0.01	0.028	1
Rbks	ENSMUSG00000029136	protein_coding	ribokinase [Source:MGI Symbol;Acc:MGI:1918586]	<0.01	0.029	1
Cfdp1	ENSMUSG00000031954	protein_coding	craniofacial development protein 1 [Source:MGI Symbol;Acc:MGI:1344403]	<0.01	0.041	1
Rpl19	ENSMUSG00000017404	protein_coding	ribosomal protein L19 [Source:MGI Symbol;Acc:MGI:98020]	<0.01	0.019	1
Nupr1	ENSMUSG00000030717	protein_coding	nuclear protein transcription regulator 1 [Source:MGI Symbol;Acc:MGI:1891834]	<0.01	0.023	1
Igsf6	ENSMUSG00000035004	protein_coding	immunoglobulin superfamily, member 6 [Source:MGI Symbol;Acc:MGI:1891393]	<0.01	0.036	1
Gadd45b	ENSMUSG00000015312	protein_coding	growth arrest and DNA-damage-inducible 45 beta [Source:MGI Symbol;Acc:MGI:107776]	<0.01	0.028	1

Ttk	ENSMUSG00000038379	protein_coding	Ttk protein kinase [Source:MGI Symbol;Acc:MGI:1194921]	<0.01	0.042	1
BC043934	ENSMUSG00000056418	lincRNA	cDNA sequence BC043934 [Source:MGI Symbol;Acc:MGI:2679715]	<0.01	0.036	1
Ccnjl	ENSMUSG00000044707	protein_coding	cyclin J-like [Source:MGI Symbol;Acc:MGI:2685723]	<0.01	0.020	1
Fjx1	ENSMUSG00000075012	protein_coding	four jointed box 1 (Drosophila) [Source:MGI Symbol;Acc:MGI:1341907]	<0.01	0.040	1
Chchd5	ENSMUSG00000037938	protein_coding	coiled-coil-helix-coiled-coil-helix domain containing 5 [Source:MGI Symbol;Acc:MGI:1913420]	<0.01	0.044	1
Nup37	ENSMUSG00000035351	protein_coding	nucleoporin 37 [Source:MGI Symbol;Acc:MGI:1919964]	<0.01	<0.01	1
Ifi204	ENSMUSG00000073489	protein_coding	interferon activated gene 204 [Source:MGI Symbol;Acc:MGI:96429]	<0.01	0.040	1
Cytl1	ENSMUSG00000062329	protein_coding	cytokine-like 1 [Source:MGI Symbol;Acc:MGI:2684993]	<0.01	<0.01	1
1810011O10Rik	ENSMUSG00000056313	protein_coding	RIKEN cDNA 1810011O10 gene [Source:MGI Symbol;Acc:MGI:1916318]	<0.01	0.034	1
Zfp322a	ENSMUSG00000046351	protein_coding	zinc finger protein 322A [Source:MGI Symbol;Acc:MGI:2442566]	<0.01	0.043	1
Ntrk2	ENSMUSG00000055254	protein_coding	neurotrophic tyrosine kinase, receptor, type 2 [Source:MGI Symbol;Acc:MGI:97384]	<0.01	<0.01	1
Myct1	ENSMUSG00000046916	protein_coding	myc target 1 [Source:MGI Symbol;Acc:MGI:1915882]	<0.01	<0.01	1
Rpl15	ENSMUSG00000012405	protein_coding	ribosomal protein L15 [Source:MGI Symbol;Acc:MGI:1913730]	<0.01	<0.01	1
Copg2	ENSMUSG00000025607	protein_coding	coatomer protein complex, subunit gamma 2 [Source:MGI Symbol;Acc:MGI:1858683]	<0.01	<0.01	1
Hdhd3	ENSMUSG00000038422	protein_coding	haloacid dehalogenase-like hydrolase domain containing 3 [Source:MGI Symbol;Acc:MGI:1919998]	<0.01	<0.01	1
Tulp1	ENSMUSG00000037446	protein_coding	tubby like protein 1 [Source:MGI Symbol;Acc:MGI:109571]	<0.01	0.012	1
Lpar6	ENSMUSG00000033446	protein_coding	lysophosphatidic acid receptor 6 [Source:MGI Symbol;Acc:MGI:1914418]	<0.01	0.012	1
Tmem126b	ENSMUSG00000030614	protein_coding	transmembrane protein 126B [Source:MGI Symbol;Acc:MGI:1915722]	<0.01	0.026	1
Kcna2	ENSMUSG00000040724	protein_coding	potassium voltage-gated channel, shaker-related subfamily, member 2 [Source:MGI Symbol;Acc:MGI:96659]	<0.01	0.019	1
Elp6	ENSMUSG00000054836	protein_coding	elongator acetyltransferase complex subunit 6 [Source:MGI Symbol;Acc:MGI:1919349]	<0.01	<0.01	1
Tifab	ENSMUSG00000049625	protein_coding	TRAF-interacting protein with forkhead-associated domain, family member B [Source:MGI Symbol;Acc:MGI:2385852]	<0.01	0.020	1
Crabp1	ENSMUSG00000032291	protein_coding	cellular retinoic acid binding protein I [Source:MGI Symbol;Acc:MGI:88490]	<0.01	0.011	1
Rsad2	ENSMUSG00000020641	protein_coding	radical S-adenosyl methionine domain containing 2 [Source:MGI Symbol;Acc:MGI:1929628]	<0.01	0.040	1
Mycn	ENSMUSG00000037169	protein_coding	v-myc avian myelocytomatosis viral related oncogene, neuroblastoma derived [Source:MGI Symbol;Acc:MGI:97357]	<0.01	<0.01	1
Plau	ENSMUSG00000021822	protein_coding	plasminogen activator, urokinase [Source:MGI Symbol;Acc:MGI:97611]	<0.01	0.017	1
Stmn2	ENSMUSG00000027500	protein_coding	stathmin-like 2 [Source:MGI Symbol;Acc:MGI:98241]	<0.01	<0.01	1
Slc26a10	ENSMUSG00000040441	protein_coding	solute carrier family 26, member 10 [Source:MGI Symbol;Acc:MGI:2143920]	<0.01	<0.01	1
Bvht	ENSMUSG00000098098	lincRNA	braveheart long non-coding RNA [Source:MGI Symbol;Acc:MGI:5434104]	<0.01	0.032	1
Nrgn	ENSMUSG00000053310	protein_coding	neurogranin [Source:MGI Symbol;Acc:MGI:1927184]	<0.01	0.020	1
Gja4	ENSMUSG00000050234	protein_coding	gap junction protein, alpha 4 [Source:MGI Symbol;Acc:MGI:95715]	<0.01	0.030	1
Prkg2	ENSMUSG00000029334	protein_coding	protein kinase, cGMP-dependent, type II [Source:MGI Symbol;Acc:MGI:108173]	<0.01	<0.01	1
E130311K13Rik	ENSMUSG00000048581	protein_coding	RIKEN cDNA E130311K13 gene [Source:MGI Symbol;Acc:MGI:3607716]	<0.01	<0.01	1
S100a13	ENSMUSG00000042312	protein_coding	S100 calcium binding protein A13 [Source:MGI Symbol;Acc:MGI:109581]	<0.01	0.035	1
Copz2	ENSMUSG00000018672	protein_coding	coatomer protein complex, subunit zeta 2 [Source:MGI Symbol;Acc:MGI:1929008]	<0.01	0.035	1
Tbx3os1	ENSMUSG00000087516	antisense_RNA	T-box 3, opposite strand 1 [Source:MGI Symbol;Acc:MGI:3780472]	<0.01	<0.01	1
Hemk1	ENSMUSG00000032579	protein_coding	HemK methyltransferase family member 1 [Source:MGI Symbol;Acc:MGI:1916786]	<0.01	0.022	1
Ubxn8	ENSMUSG00000052906	protein_coding	UBX domain protein 8 [Source:MGI Symbol;Acc:MGI:1337129]	<0.01	0.027	1
Dusp12	ENSMUSG00000026659	protein_coding	dual specificity phosphatase 12 [Source:MGI Symbol;Acc:MGI:1890614]	<0.01	0.010	1
Cd300c	ENSMUSG00000058728	protein_coding	CD300C molecule [Source:MGI Symbol;Acc:MGI:3032626]	<0.01	<0.01	1
Cdkn2c	ENSMUSG00000028551	protein_coding	cyclin-dependent kinase inhibitor 2C (p18, inhibits CDK4) [Source:MGI Symbol;Acc:MGI:105388]	<0.01	<0.01	1
Hoxb2	ENSMUSG00000075588	protein_coding	homeobox B2 [Source:MGI Symbol;Acc:MGI:96183]	<0.01	0.042	1
Serf2	ENSMUSG00000074884	protein_coding	small EDRK-rich factor 2 [Source:MGI Symbol;Acc:MGI:1337041]	<0.01	0.035	1
Gm16556	ENSMUSG00000090192	processed_transcript	predicted gene 16556 [Source:MGI Symbol;Acc:MGI:4414976]	<0.01	<0.01	1
Dok1	ENSMUSG00000068335	protein_coding	docking protein 1 [Source:MGI Symbol;Acc:MGI:893587]	<0.01	0.023	1
Adrb3	ENSMUSG00000031489	protein_coding	adrenergic receptor, beta 3 [Source:MGI Symbol;Acc:MGI:87939]	<0.01	0.017	1
Hba-a1	ENSMUSG00000069919	protein_coding	hemoglobin alpha, adult chain 1 [Source:MGI Symbol;Acc:MGI:96015]	<0.01	<0.01	1
Ramp3	ENSMUSG00000041046	protein_coding	receptor (calcitonin) activity modifying protein 3 [Source:MGI Symbol;Acc:MGI:1860292]	<0.01	<0.01	1
Acaa1b	ENSMUSG00000010651	protein_coding	acetyl-Coenzyme A acyltransferase 1B [Source:MGI Symbol;Acc:MGI:3605455]	<0.01	0.029	1
Gm37192	ENSMUSG00000102184	TEC	predicted gene, 37192 [Source:MGI Symbol;Acc:MGI:5610420]	<0.01	<0.01	1
Gm37660	ENSMUSG00000104467	TEC	predicted gene, 37660 [Source:MGI Symbol;Acc:MGI:5610888]	<0.01	0.017	1
Ccdc184	ENSMUSG00000029875	protein_coding	coiled-coil domain containing 184 [Source:MGI Symbol;Acc:MGI:2146066]	<0.01	<0.01	1
Gm43573	ENSMUSG00000104786	antisense_RNA	predicted gene 43573 [Source:MGI Symbol;Acc:MGI:5663710]	<0.01	0.022	1
Msl3l2	ENSMUSG00000047669	protein_coding	male-specific lethal 3-like 2 (Drosophila) [Source:MGI Symbol;Acc:MGI:1920640]	<0.01	0.020	1
Cox4i2	ENSMUSG00000009876	protein_coding	cytochrome c oxidase subunit 4I2 [Source:MGI Symbol;Acc:MGI:2135755]	<0.01	<0.01	1
Fabp1	ENSMUSG00000054422	protein_coding	fatty acid binding protein 1, liver [Source:MGI Symbol;Acc:MGI:95479]	<0.01	<0.01	1
Plk4	ENSMUSG00000025758	protein_coding	polo-like kinase 4 [Source:MGI Symbol;Acc:MGI:101783]	<0.01	<0.01	1
Tstd3	ENSMUSG00000028251	protein_coding	thiosulfate sulfurtransferase (rhodanese)-like domain containing 3 [Source:MGI Symbol;Acc:MGI:1924282]	<0.01	0.011	1
Pla1a	ENSMUSG00000002847	protein_coding	phospholipase A1 member A [Source:MGI Symbol;Acc:MGI:1934677]	<0.01	0.019	1
Lysmd1	ENSMUSG00000053769	protein_coding	LysM, putative peptidoglycan-binding, domain containing 1 [Source:MGI Symbol;Acc:MGI:1919409]	<0.01	0.019	1
Gm20632	ENSMUSG00000093577	antisense_RNA	predicted gene 20632 [Source:MGI Symbol;Acc:MGI:5313079]	<0.01	<0.01	1
Rgs18	ENSMUSG00000026357	protein_coding	regulator of G-protein signaling 18 [Source:MGI Symbol;Acc:MGI:1927498]	<0.01	0.012	1
Hbq1b	ENSMUSG00000073063	protein_coding	hemoglobin, theta 1B [Source:MGI Symbol;Acc:MGI:3613460]	<0.01	0.034	1
Hist1h4i	ENSMUSG00000060639	protein_coding	histone cluster 1, H4i [Source:MGI Symbol;Acc:MGI:2448432]	<0.01	<0.01	1
Riok1	ENSMUSG00000021428	protein_coding	RIO kinase 1 (yeast) [Source:MGI Symbol;Acc:MGI:1918590]	<0.01	0.021	1
1810058I24Rik	ENSMUSG00000073155	lincRNA	RIKEN cDNA 1810058I24 gene [Source:MGI Symbol;Acc:MGI:1914955]	<0.01	0.028	1
Mzt2	ENSMUSG00000022671	protein_coding	mitotic spindle organizing protein 2 [Source:MGI Symbol;Acc:MGI:1922845]	<0.01	0.019	1
Ccdc42	ENSMUSG00000045915	protein_coding	coiled-coil domain containing 42 [Source:MGI Symbol;Acc:MGI:3045254]	<0.01	<0.01	1
Vstm5	ENSMUSG00000031937	protein_coding	V-set and transmembrane domain containing 5 [Source:MGI Symbol;Acc:MGI:1916387]	<0.01	0.030	1
Hbb-bs	ENSMUSG00000052305	protein_coding	hemoglobin, beta adult s chain [Source:MGI Symbol;Acc:MGI:5474852]	<0.01	<0.01	1
Pelo	ENSMUSG00000042275	protein_coding	pelota homolog (Drosophila) [Source:MGI Symbol;Acc:MGI:2145154]	<0.01	0.023	1
Sapcd1	ENSMUSG00000036185	protein_coding	suppressor APC domain containing 1 [Source:MGI Symbol;Acc:MGI:2388100]	<0.01	<0.01	1
Emid1	ENSMUSG00000034164	protein_coding	EMI domain containing 1 [Source:MGI Symbol;Acc:MGI:2155091]	<0.01	<0.01	1
2610203C22Rik	ENSMUSG00000079671	processed_transcript	RIKEN cDNA 2610203C22 gene [Source:MGI Symbol;Acc:MGI:1919731]	<0.01	<0.01	1
Hbb-bt	ENSMUSG00000073940	protein_coding	hemoglobin, beta adult t chain [Source:MGI Symbol;Acc:MGI:5474850]	<0.01	<0.01	1
Fam167b	ENSMUSG00000050493	protein_coding	family with sequence similarity 167, member B [Source:MGI Symbol;Acc:MGI:2668032]	<0.01	<0.01	1
Commd9	ENSMUSG00000027163	protein_coding	COMM domain containing 9 [Source:MGI Symbol;Acc:MGI:1923751]	<0.01	0.042	1
RP23-137A24.3	ENSMUSG00000108555	TEC	predicted gene, 18310 [Source:MGI Symbol;Acc:MGI:5010495]	<0.01	0.016	1
Nudt17	ENSMUSG00000028100	protein_coding	nudix (nucleoside diphosphate linked moiety X)-type motif 17 [Source:MGI Symbol;Acc:MGI:1925623]	<0.01	0.038	1
Pabpc1l	ENSMUSG00000054582	protein_coding	poly(A) binding protein, cytoplasmic 1-like [Source:MGI Symbol;Acc:MGI:1922908]	<0.01	<0.01	1
Hebp1	ENSMUSG00000042770	protein_coding	heme binding protein 1 [Source:MGI Symbol;Acc:MGI:1333880]	<0.01	<0.01	1

Zfp709	ENSMUSG00000056019	protein_coding	zinc finger protein 709 [Source:MGI Symbol;Acc:MGI:2384299]	<0.01	<0.01	1
Vsnl1	ENSMUSG00000054459	protein_coding	visinin-like 1 [Source:MGI Symbol;Acc:MGI:1349453]	<0.01	0.018	1
1700052K11Rik	ENSMUSG00000099681	antisense_RNA	RIKEN cDNA 1700052K11 gene [Source:MGI Symbol;Acc:MGI:1920681]	<0.01	<0.01	1
Mest	ENSMUSG00000051855	protein_coding	mesoderm specific transcript [Source:MGI Symbol;Acc:MGI:96968]	<0.01	<0.01	1
Gm13066	ENSMUSG00000086949	antisense_RNA	predicted gene 13066 [Source:MGI Symbol;Acc:MGI:3701131]	<0.01	0.015	1
Tmem150a	ENSMUSG00000055912	protein_coding	transmembrane protein 150A [Source:MGI Symbol;Acc:MGI:2385244]	<0.01	<0.01	1
Thg1l	ENSMUSG00000011254	protein_coding	tRNA-histidine guanylyltransferase 1-like (S. cerevisiae) [Source:MGI Symbol;Acc:MGI:1913878]	<0.01	<0.01	1

Note: Decide test value "-1" or "1" indicates the t-statistic is classified as significantly negative or significantly positive respectively

Table S3c. Differentially expressed transcripts for interaction (2 way limma anova test, limma decide test, P<0.05)

Gene	Gene ID	Biotype	Description	P.value	Adjusted.P.value	Decide test
Gm10874	ENSMUSG00000075591	lincRNA	predicted gene 10874 [Source:MGI Symbol;Acc:MGI:3704261]	<0.01	0.022	-1
Maff	ENSMUSG00000042622	protein_coding	v-maf musculoaponeurotic fibrosarcoma oncogene family, protein F (avian) [Source:MGI Symbol;Acc:MGI:96910]	<0.01	0.021	-1
Gm6652	ENSMUSG00000099858	processed_pseudogene	predicted gene 6652 [Source:MGI Symbol;Acc:MGI:3647244]	<0.01	<0.01	-1
1700105P06Rik	ENSMUSG00000099923	antisense_RNA	RIKEN cDNA 1700105P06 gene [Source:MGI Symbol;Acc:MGI:1915479]	<0.01	0.012	-1
Ifi27l2a	ENSMUSG00000079017	protein_coding	interferon, alpha-inducible protein 27 like 2A [Source:MGI Symbol;Acc:MGI:1924183]	<0.01	0.017	-1
Sord	ENSMUSG00000027227	protein_coding	sorbitol dehydrogenase [Source:MGI Symbol;Acc:MGI:98266]	<0.01	<0.01	-1
Igkv1-117	ENSMUSG00000094335	IG_V_gene	immunoglobulin kappa variable 1-117 [Source:MGI Symbol;Acc:MGI:4439721]	<0.01	<0.01	-1
Ggps1	ENSMUSG00000021302	protein_coding	geranylgeranyl diphosphate synthase 1 [Source:MGI Symbol;Acc:MGI:1341724]	<0.01	0.015	-1
Gga3	ENSMUSG00000020740	protein_coding	golgi associated, gamma adaptin ear containing, ARF binding protein 3 [Source:MGI Symbol;Acc:MGI:2384159]	<0.01	0.022	-1
Qsox1	ENSMUSG00000033684	protein_coding	quiescin Q6 sulfhydryl oxidase 1 [Source:MGI Symbol;Acc:MGI:1330818]	<0.01	0.033	-1
Slc31a1	ENSMUSG00000066150	protein_coding	solute carrier family 31, member 1 [Source:MGI Symbol;Acc:MGI:1333843]	<0.01	0.043	-1
Alas1	ENSMUSG00000032786	protein_coding	aminolevulinic acid synthase 1 [Source:MGI Symbol;Acc:MGI:87989]	<0.01	<0.01	-1
8430408G22Rik	ENSMUSG00000048489	protein_coding	RIKEN cDNA 8430408G22 gene [Source:MGI Symbol;Acc:MGI:1918730]	<0.01	0.028	-1
Zbtb16	ENSMUSG00000066687	protein_coding	zinc finger and BTB domain containing 16 [Source:MGI Symbol;Acc:MGI:103222]	<0.01	<0.01	-1
Ank	ENSMUSG00000022265	protein_coding	progressive ankylosis [Source:MGI Symbol;Acc:MGI:3045421]	<0.01	0.046	-1
Gm10184	ENSMUSG00000066878	protein_coding	predicted pseudogene 10184 [Source:MGI Symbol;Acc:MGI:3704480]	<0.01	0.031	-1
Wee1	ENSMUSG00000031016	protein_coding	WEE 1 homolog 1 (S. pombe) [Source:MGI Symbol;Acc:MGI:103075]	<0.01	<0.01	-1
Ngp	ENSMUSG00000032484	protein_coding	neutrophilic granule protein [Source:MGI Symbol;Acc:MGI:105983]	<0.01	0.029	-1
Arhgap27os2	ENSMUSG00000085360	antisense_RNA	Rho GTPase activating protein 27, opposite strand 2 [Source:MGI Symbol;Acc:MGI:3650160]	<0.01	0.029	-1
Bhlhe40	ENSMUSG00000030103	protein_coding	basic helix-loop-helix family, member e40 [Source:MGI Symbol;Acc:MGI:1097714]	<0.01	<0.01	-1
Tmem200b	ENSMUSG00000070720	protein_coding	transmembrane protein 200B [Source:MGI Symbol;Acc:MGI:3646343]	<0.01	<0.01	-1
Letm1	ENSMUSG00000005299	protein_coding	leucine zipper-EF-hand containing transmembrane protein 1 [Source:MGI Symbol;Acc:MGI:1932557]	<0.01	0.029	-1
Sele	ENSMUSG00000026582	protein_coding	selectin, endothelial cell [Source:MGI Symbol;Acc:MGI:98278]	<0.01	0.016	-1
Gm26699	ENSMUSG00000097206	lincRNA	predicted gene, 26699 [Source:MGI Symbol;Acc:MGI:5477193]	<0.01	0.032	-1
Plekha6	ENSMUSG00000041757	protein_coding	pleckstrin homology domain containing, family A member 6 [Source:MGI Symbol;Acc:MGI:2388662]	<0.01	<0.01	-1
Bhlhe41	ENSMUSG00000030256	protein_coding	basic helix-loop-helix family, member e41 [Source:MGI Symbol;Acc:MGI:1930704]	<0.01	<0.01	-1
Agmo	ENSMUSG00000050103	protein_coding	alkylglycerol monooxygenase [Source:MGI Symbol;Acc:MGI:2442495]	<0.01	<0.01	-1
Gm28044	ENSMUSG00000098781	protein_coding	predicted gene, 28044 [Source:MGI Symbol;Acc:MGI:5547780]	<0.01	<0.01	-1
Mthfd1l	ENSMUSG00000040675	protein_coding	methylenetetrahydrofolate dehydrogenase (NADP+ dependent) 1-like [Source:MGI Symbol;Acc:MGI:1924836]	<0.01	<0.01	-1
Gm4759	ENSMUSG00000053541	unprocessed_pseudogene	predicted gene 4759 [Source:MGI Symbol;Acc:MGI:3647753]	<0.01	<0.01	-1
Per2	ENSMUSG00000055866	protein_coding	period circadian clock 2 [Source:MGI Symbol;Acc:MGI:1195265]	<0.01	<0.01	-1
Tspan4	ENSMUSG00000025511	protein_coding	tetraspanin 4 [Source:MGI Symbol;Acc:MGI:1928097]	<0.01	<0.01	-1
Ighv2-6-8	ENSMUSG00000076646	IG_V_gene	immunoglobulin heavy variable 2-6-8 [Source:MGI Symbol;Acc:MGI:4439811]	<0.01	0.012	-1
Htr2b	ENSMUSG00000026228	protein_coding	5-hydroxytryptamine (serotonin) receptor 2B [Source:MGI Symbol;Acc:MGI:109323]	<0.01	<0.01	-1
Tef	ENSMUSG00000022389	protein_coding	thyrotroph embryonic factor [Source:MGI Symbol;Acc:MGI:98663]	<0.01	<0.01	-1
Pthlh	ENSMUSG00000048776	protein_coding	parathyroid hormone-like peptide [Source:MGI Symbol;Acc:MGI:97800]	<0.01	<0.01	-1
Nr1d2	ENSMUSG00000021775	protein_coding	nuclear receptor subfamily 1, group D, member 2 [Source:MGI Symbol;Acc:MGI:2449205]	<0.01	<0.01	-1
Osgep	ENSMUSG00000006289	protein_coding	O-sialoglycoprotein endopeptidase [Source:MGI Symbol;Acc:MGI:1913496]	<0.01	0.027	-1
Trat1	ENSMUSG00000030775	protein_coding	T cell receptor associated transmembrane adaptor 1 [Source:MGI Symbol;Acc:MGI:1924897]	<0.01	0.026	-1
Tm7sf2	ENSMUSG00000024799	protein_coding	transmembrane 7 superfamily member 2 [Source:MGI Symbol;Acc:MGI:1920416]	<0.01	0.040	-1
Akt2-ps	ENSMUSG00000084347	processed_pseudogene	thymoma viral proto-oncogene 2, pseudogene [Source:MGI Symbol;Acc:MGI:108506]	<0.01	0.030	-1
Lpxn	ENSMUSG00000024696	protein_coding	leupaxin [Source:MGI Symbol;Acc:MGI:2147677]	<0.01	0.016	-1
Gm42501	ENSMUSG00000105609	TEC	predicted gene 42501 [Source:MGI Symbol;Acc:MGI:5662638]	<0.01	0.028	-1
Gm13502	ENSMUSG00000083287	processed_pseudogene	predicted gene 13502 [Source:MGI Symbol;Acc:MGI:3649894]	<0.01	0.029	-1
Arl11	ENSMUSG00000043157	protein_coding	ADP-ribosylation factor-like 11 [Source:MGI Symbol;Acc:MGI:2444054]	<0.01	0.039	-1
Ahsa1	ENSMUSG00000021037	protein_coding	AHA1, activator of heat shock protein ATPase 1 [Source:MGI Symbol;Acc:MGI:2387603]	<0.01	0.032	-1
Rab32	ENSMUSG00000019832	protein_coding	RAB32, member RAS oncogene family [Source:MGI Symbol;Acc:MGI:1915094]	<0.01	0.012	-1
Sirpb1a	ENSMUSG00000095788	protein_coding	signal-regulatory protein beta 1A [Source:MGI Symbol;Acc:MGI:2444824]	<0.01	0.015	-1
Themis2	ENSMUSG00000037731	protein_coding	thymocyte selection associated family member 2 [Source:MGI Symbol;Acc:MGI:2446213]	<0.01	0.012	-1
Ms4a6b	ENSMUSG00000024677	protein_coding	membrane-spanning 4-domains, subfamily A, member 6B [Source:MGI Symbol;Acc:MGI:1917024]	<0.01	<0.01	-1
Smim7	ENSMUSG00000044600	protein_coding	small integral membrane protein 7 [Source:MGI Symbol;Acc:MGI:1914068]	<0.01	0.032	-1
Tpt1	ENSMUSG00000060126	protein_coding	tumor protein, translationally-controlled 1 [Source:MGI Symbol;Acc:MGI:104890]	<0.01	0.013	-1
Wfdc17	ENSMUSG00000069792	protein_coding	WAP four-disulfide core domain 17 [Source:MGI Symbol;Acc:MGI:3649773]	<0.01	<0.01	-1
Igkv8-27	ENSMUSG00000076580	IG_V_gene	immunoglobulin kappa chain variable 8-27 [Source:MGI Symbol;Acc:MGI:4439868]	<0.01	0.014	-1
Acp1	ENSMUSG00000044573	protein_coding	acid phosphatase 1, soluble [Source:MGI Symbol;Acc:MGI:87881]	<0.01	<0.01	-1
Nme2	ENSMUSG00000020857	protein_coding	NME/NM23 nucleoside diphosphate kinase 2 [Source:MGI Symbol;Acc:MGI:97356]	<0.01	<0.01	-1
Ap4b1	ENSMUSG00000032952	protein_coding	adaptor-related protein complex AP-4, beta 1 [Source:MGI Symbol;Acc:MGI:1337130]	<0.01	0.015	-1
Nabp1	ENSMUSG00000026107	protein_coding	nucleic acid binding protein 1 [Source:MGI Symbol;Acc:MGI:1923258]	<0.01	0.031	-1
Pnpla2	ENSMUSG00000025509	protein_coding	patatin-like phospholipase domain containing 2 [Source:MGI Symbol;Acc:MGI:1914103]	<0.01	0.047	-1
Stat1	ENSMUSG00000026104	protein_coding	signal transducer and activator of transcription 1 [Source:MGI Symbol;Acc:MGI:103063]	<0.01	0.026	-1
Tpd52	ENSMUSG00000027506	protein_coding	tumor protein D52 [Source:MGI Symbol;Acc:MGI:107749]	<0.01	0.029	-1
Ttf2	ENSMUSG00000033222	protein_coding	transcription termination factor, RNA polymerase II [Source:MGI Symbol;Acc:MGI:1921294]	<0.01	0.025	-1
Dck	ENSMUSG00000029366	protein_coding	deoxycytidine kinase [Source:MGI Symbol;Acc:MGI:102726]	<0.01	<0.01	-1
Chil3	ENSMUSG00000040809	protein_coding	chitinase-like 3 [Source:MGI Symbol;Acc:MGI:1330860]	<0.01	<0.01	-1
Gm20458	ENSMUSG00000090996	protein_coding	predicted gene 20458 [Source:MGI Symbol;Acc:MGI:5141923]	<0.01	<0.01	-1
Sirpb1c	ENSMUSG00000074677	protein_coding	signal-regulatory protein beta 1C [Source:MGI Symbol;Acc:MGI:3807521]	<0.01	<0.01	-1
Tyrobp	ENSMUSG00000030579	protein_coding	TYRO protein tyrosine kinase binding protein [Source:MGI Symbol;Acc:MGI:1277211]	<0.01	0.017	-1
Rpl9-ps7	ENSMUSG00000047965	processed_pseudogene	ribosomal protein L9, pseudogene 7 [Source:MGI Symbol;Acc:MGI:3642824]	<0.01	0.028	-1
Coro1a	ENSMUSG00000030707	protein_coding	coronin, actin binding protein 1A [Source:MGI Symbol;Acc:MGI:1345961]	<0.01	<0.01	-1
Ifit1bl1	ENSMUSG00000079339	protein_coding	interferon induced protein with tetratricpeptide repeats 1B like 1 [Source:MGI Symbol;Acc:MGI:3650685]	<0.01	0.011	-1
Appbp2os	ENSMUSG00000085628	antisense_RNA	amyloid beta precursor protein (cytoplasmic tail) binding protein 2, opposite strand [Source:MGI Symbol;Acc:MGI:3603817]	<0.01	0.015	-1
Mal	ENSMUSG00000027375	protein_coding	myelin and lymphocyte protein, T cell differentiation protein [Source:MGI Symbol;Acc:MGI:892970]	<0.01	0.023	-1
Ptpn6	ENSMUSG00000004266	protein_coding	protein tyrosine phosphatase, non-receptor type 6 [Source:MGI Symbol;Acc:MGI:96055]	<0.01	0.013	-1
Col26a1	ENSMUSG00000004415	protein_coding	collagen, type XXVI, alpha 1 [Source:MGI Symbol;Acc:MGI:2155345]	<0.01	<0.01	-1
Gm9888	ENSMUSG00000052724	antisense_RNA	predicted gene 9888 [Source:MGI Symbol;Acc:MGI:3642202]	<0.01	0.020	-1
Plac8	ENSMUSG00000029322	protein_coding	placenta-specific 8 [Source:MGI Symbol;Acc:MGI:2445289]	<0.01	<0.01	-1
Fkbp4	ENSMUSG00000030357	protein_coding	FK506 binding protein 4 [Source:MGI Symbol;Acc:MGI:95543]	<0.01	0.013	-1

Sspn	ENSMUSG00000030255	protein_coding	sarcospan [Source:MGI Symbol;Acc:MGI:1353511]	<0.01	0.012	-1
Tmem154	ENSMUSG00000056498	protein_coding	transmembrane protein 154 [Source:MGI Symbol;Acc:MGI:2444725]	<0.01	0.026	-1
Acad11	ENSMUSG00000090150	protein_coding	acyl-Coenzyme A dehydrogenase family, member 11 [Source:MGI Symbol;Acc:MGI:2143169]	<0.01	0.032	-1
Car8	ENSMUSG00000041261	protein_coding	carbonic anhydrase 8 [Source:MGI Symbol;Acc:MGI:88253]	<0.01	0.013	-1
Hspa4l	ENSMUSG00000025757	protein_coding	heat shock protein 4 like [Source:MGI Symbol;Acc:MGI:107422]	<0.01	<0.01	-1
Trim30a	ENSMUSG00000030921	protein_coding	tripartite motif-containing 30A [Source:MGI Symbol;Acc:MGI:98178]	<0.01	0.013	-1
Tc2n	ENSMUSG00000021187	protein_coding	tandem C2 domains, nuclear [Source:MGI Symbol;Acc:MGI:1921663]	<0.01	<0.01	-1
Gm43474	ENSMUSG00000105362	TEC	predicted gene 43474 [Source:MGI Symbol;Acc:MGI:5663611]	<0.01	0.019	-1
Capza1	ENSMUSG00000070372	protein_coding	capping protein (actin filament) muscle Z-line, alpha 1 [Source:MGI Symbol;Acc:MGI:106227]	<0.01	<0.01	-1
Rps15a	ENSMUSG00000008683	protein_coding	ribosomal protein S15A [Source:MGI Symbol;Acc:MGI:2389091]	<0.01	<0.01	-1
Apobr	ENSMUSG00000042759	protein_coding	apolipoprotein B receptor [Source:MGI Symbol;Acc:MGI:2176230]	<0.01	<0.01	-1
Tnfsf13	ENSMUSG00000089669	protein_coding	tumor necrosis factor (ligand) superfamily, member 13 [Source:MGI Symbol;Acc:MGI:1916833]	<0.01	<0.01	-1
Zfp874a	ENSMUSG00000069206	protein_coding	zinc finger protein 874a [Source:MGI Symbol;Acc:MGI:3040703]	<0.01	<0.01	-1
Mmgt2	ENSMUSG00000048497	protein_coding	membrane magnesium transporter 2 [Source:MGI Symbol;Acc:MGI:2448491]	<0.01	0.029	-1
Sh2d1b1	ENSMUSG00000102418	protein_coding	SH2 domain containing 1B1 [Source:MGI Symbol;Acc:MGI:1349420]	<0.01	<0.01	-1
Sla	ENSMUSG00000022372	protein_coding	src-like adaptor [Source:MGI Symbol;Acc:MGI:104295]	<0.01	0.039	-1
Eci2	ENSMUSG00000021417	protein_coding	enoyl-Coenzyme A delta isomerase 2 [Source:MGI Symbol;Acc:MGI:1346064]	<0.01	0.049	-1
Gm7389	ENSMUSG00000097657	processed_pseudogene	predicted gene 7389 [Source:MGI Symbol;Acc:MGI:3645153]	<0.01	0.049	-1
Chil4	ENSMUSG00000063779	protein_coding	chitinase-like 4 [Source:MGI Symbol;Acc:MGI:1341098]	<0.01	0.048	-1
Clec4n	ENSMUSG00000023349	protein_coding	C-type lectin domain family 4, member n [Source:MGI Symbol;Acc:MGI:1861231]	<0.01	0.030	-1
Gm7478	ENSMUSG00000107327	processed_pseudogene	predicted gene 7478 [Source:MGI Symbol;Acc:MGI:3648572]	<0.01	0.044	-1
Ucp3	ENSMUSG00000032942	protein_coding	uncoupling protein 3 (mitochondrial, proton carrier) [Source:MGI Symbol;Acc:MGI:1099787]	<0.01	0.033	-1
Gm13991	ENSMUSG00000081787	processed_pseudogene	predicted gene 13991 [Source:MGI Symbol;Acc:MGI:3651689]	<0.01	0.048	-1
Gm13456	ENSMUSG00000082536	processed_pseudogene	predicted gene 13456 [Source:MGI Symbol;Acc:MGI:3651389]	<0.01	0.042	-1
Gm26566	ENSMUSG00000097078	protein_coding	predicted gene, 26566 [Source:MGI Symbol;Acc:MGI:5477060]	<0.01	0.033	-1
Pstpip2	ENSMUSG00000025429	protein_coding	proline-serine-threonine phosphatase-interacting protein 2 [Source:MGI Symbol;Acc:MGI:1335088]	<0.01	0.017	-1
Siglecf	ENSMUSG00000039013	protein_coding	sialic acid binding Ig-like lectin F [Source:MGI Symbol;Acc:MGI:2681107]	<0.01	0.022	-1
Usp3	ENSMUSG00000032376	protein_coding	ubiquitin specific peptidase 3 [Source:MGI Symbol;Acc:MGI:2152450]	<0.01	0.045	-1
Mrps5	ENSMUSG00000027374	protein_coding	mitochondrial ribosomal protein S5 [Source:MGI Symbol;Acc:MGI:1924971]	<0.01	0.038	-1
Klk13	ENSMUSG00000054046	protein_coding	kallikrein related-peptidase 13 [Source:MGI Symbol;Acc:MGI:3615275]	<0.01	0.037	-1
BC026585	ENSMUSG00000033488	protein_coding	cDNA sequence BC026585 [Source:MGI Symbol;Acc:MGI:2448516]	<0.01	0.049	-1
Hmbs	ENSMUSG00000032126	protein_coding	hydroxymethylbilane synthase [Source:MGI Symbol;Acc:MGI:96112]	<0.01	0.026	-1
Gm6649	ENSMUSG00000105645	processed_pseudogene	predicted gene 6649 [Source:MGI Symbol;Acc:MGI:3779621]	<0.01	0.049	-1
Gm14325	ENSMUSG00000095362	protein_coding	predicted gene 14325 [Source:MGI Symbol;Acc:MGI:3702875]	<0.01	0.028	-1
Trp53rka	ENSMUSG00000039725	protein_coding	transformation related protein 53 regulating kinase A [Source:MGI Symbol;Acc:MGI:1918294]	<0.01	0.044	-1
Scai	ENSMUSG00000035236	protein_coding	suppressor of cancer cell invasion [Source:MGI Symbol;Acc:MGI:2443716]	<0.01	0.017	-1
Mcm5	ENSMUSG00000005410	protein_coding	minichromosome maintenance complex component 5 [Source:MGI Symbol;Acc:MGI:103197]	<0.01	0.040	-1
Tnfrsf26	ENSMUSG00000045362	protein_coding	tumor necrosis factor receptor superfamily, member 26 [Source:MGI Symbol;Acc:MGI:2651928]	<0.01	0.039	-1
Fbxo6	ENSMUSG00000055401	protein_coding	F-box protein 6 [Source:MGI Symbol;Acc:MGI:1354743]	<0.01	0.043	-1
Gm18432	ENSMUSG00000102609	processed_pseudogene	predicted gene, 18432 [Source:MGI Symbol;Acc:MGI:5010617]	<0.01	0.040	-1
Cd302	ENSMUSG00000060703	protein_coding	CD302 antigen [Source:MGI Symbol;Acc:MGI:1913455]	<0.01	0.039	-1
Calr-ps	ENSMUSG00000081731	processed_pseudogene	calreticulin, pseudogene [Source:MGI Symbol;Acc:MGI:3651104]	<0.01	0.021	-1
Tmem54	ENSMUSG00000028786	protein_coding	transmembrane protein 54 [Source:MGI Symbol;Acc:MGI:1913510]	<0.01	0.045	-1
Wfdc21	ENSMUSG00000051748	protein_coding	WAP four-disulfide core domain 21 [Source:MGI Symbol;Acc:MGI:1913357]	<0.01	0.024	-1
Lipo1	ENSMUSG00000024766	protein_coding	lipase, member O3 [Source:MGI Symbol;Acc:MGI:2147592]	<0.01	0.027	-1
Fbxw5	ENSMUSG00000015095	protein_coding	F-box and WD-40 domain protein 5 [Source:MGI Symbol;Acc:MGI:1354731]	<0.01	0.047	-1
Gm4737	ENSMUSG00000048087	protein_coding	predicted gene 4737 [Source:MGI Symbol;Acc:MGI:3643647]	<0.01	0.043	-1
Il33	ENSMUSG00000024810	protein_coding	interleukin 33 [Source:MGI Symbol;Acc:MGI:1924375]	<0.01	0.048	-1
Cd84	ENSMUSG00000038147	protein_coding	CD84 antigen [Source:MGI Symbol;Acc:MGI:1336885]	<0.01	0.046	-1
Tcp1	ENSMUSG00000068039	protein_coding	t-complex protein 1 [Source:MGI Symbol;Acc:MGI:98535]	<0.01	0.031	-1
Klrg1	ENSMUSG00000030114	protein_coding	killer cell lectin-like receptor subfamily G, member 1 [Source:MGI Symbol;Acc:MGI:1355294]	<0.01	0.020	-1
H3f3c	ENSMUSG00000082029	processed_pseudogene	H3 histone, family 3C [Source:MGI Symbol;Acc:MGI:3650546]	<0.01	0.026	-1
Sdf2l1	ENSMUSG00000022769	protein_coding	stromal cell-derived factor 2-like 1 [Source:MGI Symbol;Acc:MGI:2149842]	<0.01	0.031	-1
Cbr3	ENSMUSG00000022947	protein_coding	carbonyl reductase 3 [Source:MGI Symbol;Acc:MGI:1309992]	<0.01	0.016	-1
Ap5m1	ENSMUSG00000036291	protein_coding	adaptor-related protein complex 5, mu 1 subunit [Source:MGI Symbol;Acc:MGI:1921635]	<0.01	0.047	-1
Mgl2	ENSMUSG00000040950	protein_coding	macrophage galactose N-acetyl-galactosamine specific lectin 2 [Source:MGI Symbol;Acc:MGI:2385729]	<0.01	0.050	-1
St7l	ENSMUSG00000045576	protein_coding	suppression of tumorigenicity 7-like [Source:MGI Symbol;Acc:MGI:2386964]	<0.01	0.044	-1
Gm37784	ENSMUSG00000102525	processed_pseudogene	predicted gene, 37784 [Source:MGI Symbol;Acc:MGI:5611012]	<0.01	0.030	-1
Gm5837	ENSMUSG00000104496	processed_pseudogene	predicted gene 5837 [Source:MGI Symbol;Acc:MGI:3648028]	<0.01	0.034	-1
4933428P19Rik	ENSMUSG00000105203	antisense_RNA	RIKEN cDNA 4933428P19 gene [Source:MGI Symbol;Acc:MGI:1918479]	<0.01	0.027	-1
Zfp946	ENSMUSG00000071266	protein_coding	zinc finger protein 946 [Source:MGI Symbol;Acc:MGI:1921399]	<0.01	0.029	-1
Gm2423	ENSMUSG00000061724	processed_pseudogene	predicted gene 2423 [Source:MGI Symbol;Acc:MGI:3805957]	<0.01	0.030	-1
Rpl7a	ENSMUSG00000062647	protein_coding	ribosomal protein L7A [Source:MGI Symbol;Acc:MGI:1353472]	<0.01	0.049	-1
Casp3	ENSMUSG00000031628	protein_coding	caspase 3 [Source:MGI Symbol;Acc:MGI:107739]	<0.01	0.044	-1
Polr3c	ENSMUSG00000028099	protein_coding	polymerase (RNA) III (DNA directed) polypeptide C [Source:MGI Symbol;Acc:MGI:1921664]	<0.01	0.037	-1
Rprd1a	ENSMUSG00000040446	protein_coding	regulation of nuclear pre-mRNA domain containing 1A [Source:MGI Symbol;Acc:MGI:2385066]	<0.01	0.050	-1
Atp6v0d2	ENSMUSG00000028238	protein_coding	ATPase, H+ transporting, lysosomal V0 subunit D2 [Source:MGI Symbol;Acc:MGI:1924415]	<0.01	0.030	-1
Gm20478	ENSMUSG00000092474	antisense_RNA	predicted gene 20478 [Source:MGI Symbol;Acc:MGI:5141943]	<0.01	<0.01	-1
Upp1	ENSMUSG00000020407	protein_coding	uridine phosphorylase 1 [Source:MGI Symbol;Acc:MGI:1097668]	<0.01	<0.01	-1
Fam107a	ENSMUSG00000021750	protein_coding	family with sequence similarity 107, member A [Source:MGI Symbol;Acc:MGI:3041256]	<0.01	<0.01	-1
Lilra5	ENSMUSG00000070873	protein_coding	leukocyte immunoglobulin-like receptor, subfamily A (with TM domain), member 5 [Source:MGI Symbol;Acc:MGI:3647196]	<0.01	0.015	-1
Cxcr1	ENSMUSG00000048480	protein_coding	chemokine (C-X-C motif) receptor 1 [Source:MGI Symbol;Acc:MGI:2448715]	<0.01	<0.01	-1
Il18	ENSMUSG00000039217	protein_coding	interleukin 18 [Source:MGI Symbol;Acc:MGI:107936]	<0.01	<0.01	-1
Dnase1l3	ENSMUSG00000025279	protein_coding	deoxyribonuclease 1-like 3 [Source:MGI Symbol;Acc:MGI:1314633]	<0.01	<0.01	-1
Gm4956	ENSMUSG00000025936	transcribed_unprocessed_pseudogene	predicted gene 4956 [Source:MGI Symbol;Acc:MGI:3647976]	<0.01	<0.01	-1
F13a1	ENSMUSG00000039109	protein_coding	coagulation factor XIII, A1 subunit [Source:MGI Symbol;Acc:MGI:1921395]	<0.01	<0.01	-1
Ms4a6c	ENSMUSG00000079419	protein_coding	membrane-spanning 4-domains, subfamily A, member 6C [Source:MGI Symbol;Acc:MGI:2385644]	<0.01	<0.01	-1
Apol11b	ENSMUSG00000091694	protein_coding	apolipoprotein L 11b [Source:MGI Symbol;Acc:MGI:3036248]	<0.01	0.020	-1
Sirpb1b	ENSMUSG00000095028	protein_coding	signal-regulatory protein beta 1B [Source:MGI Symbol;Acc:MGI:3779828]	<0.01	<0.01	-1
Igkv4-86	ENSMUSG00000076536	IG_V_gene	immunoglobulin kappa variable 4-86 [Source:MGI Symbol;Acc:MGI:2685305]	<0.01	0.011	-1

Susd3	ENSMUSG00000021384	protein_coding	sushi domain containing 3 [Source:MGI Symbol;Acc:MGI:1913579]	<0.01	0.018	-1
Asprv1	ENSMUSG00000033508	protein_coding	aspartic peptidase, retroviral-like 1 [Source:MGI Symbol;Acc:MGI:1915105]	<0.01	<0.01	-1
1810062G17Rik	ENSMUSG00000027713	protein_coding	RIKEN cDNA 1810062G17 gene [Source:MGI Symbol;Acc:MGI:1919532]	<0.01	<0.01	-1
Ccr1	ENSMUSG00000025804	protein_coding	chemokine (C-C motif) receptor 1 [Source:MGI Symbol;Acc:MGI:104618]	<0.01	0.030	-1
Egfem1	ENSMUSG00000063600	protein_coding	EGF-like and EMI domain containing 1 [Source:MGI Symbol;Acc:MGI:1922990]	<0.01	<0.01	-1
RP23-402A4.1	ENSMUSG00000108393	lincRNA	predicted gene, 32633 [Source:MGI Symbol;Acc:MGI:5591792]	<0.01	<0.01	-1
Cd300a	ENSMUSG00000034652	protein_coding	CD300A molecule [Source:MGI Symbol;Acc:MGI:2443411]	<0.01	<0.01	-1
Trim30b	ENSMUSG00000052749	protein_coding	tripartite motif-containing 30B [Source:MGI Symbol;Acc:MGI:4821256]	<0.01	<0.01	-1
Klra2	ENSMUSG00000030187	protein_coding	killer cell lectin-like receptor, subfamily A, member 2 [Source:MGI Symbol;Acc:MGI:101906]	<0.01	<0.01	-1
Gm12582	ENSMUSG00000081705	processed_pseudogene	predicted gene 12582 [Source:MGI Symbol;Acc:MGI:3651489]	<0.01	0.026	-1
Trem3	ENSMUSG00000041754	protein_coding	triggering receptor expressed on myeloid cells 3 [Source:MGI Symbol;Acc:MGI:1930003]	<0.01	0.026	-1
Evi2a	ENSMUSG00000078771	protein_coding	ecotropic viral integration site 2a [Source:MGI Symbol;Acc:MGI:95458]	<0.01	0.037	-1
Dera	ENSMUSG00000030225	protein_coding	deoxyribose-phosphate aldolase (putative) [Source:MGI Symbol;Acc:MGI:1913762]	<0.01	0.043	-1
Serpina3g	ENSMUSG00000041481	protein_coding	serine (or cysteine) peptidase inhibitor, clade A, member 3G [Source:MGI Symbol;Acc:MGI:105046]	<0.01	<0.01	-1
Gm28731	ENSMUSG00000101555	antisense_RNA	predicted gene 28731 [Source:MGI Symbol;Acc:MGI:5579437]	<0.01	0.015	-1
A930004D18Rik	ENSMUSG00000054057	protein_coding	RIKEN cDNA A930004D18 gene [Source:MGI Symbol;Acc:MGI:1925190]	<0.01	0.024	-1
Tifab	ENSMUSG00000049625	protein_coding	TRAF-interacting protein with forkhead-associated domain, family member B [Source:MGI Symbol;Acc:MGI:2385852]	<0.01	0.020	-1
Fabp1	ENSMUSG00000054422	protein_coding	fatty acid binding protein 1, liver [Source:MGI Symbol;Acc:MGI:95479]	<0.01	<0.01	-1
Hbb-bs	ENSMUSG00000052305	protein_coding	hemoglobin, beta adult s chain [Source:MGI Symbol;Acc:MGI:5474852]	<0.01	<0.01	-1
Hbb-bt	ENSMUSG00000073940	protein_coding	hemoglobin, beta adult t chain [Source:MGI Symbol;Acc:MGI:5474850]	<0.01	<0.01	-1
Cd200	ENSMUSG00000022661	protein_coding	CD200 antigen [Source:MGI Symbol;Acc:MGI:1196990]	<0.01	<0.01	1
Gm20503	ENSMUSG00000092345	protein_coding	predicted gene 20503 [Source:MGI Symbol;Acc:MGI:5141968]	<0.01	0.010	1
Abcb4	ENSMUSG00000042476	protein_coding	ATP-binding cassette, sub-family B (MDR/TAP), member 4 [Source:MGI Symbol;Acc:MGI:97569]	<0.01	0.024	1
Bhlha15	ENSMUSG00000052271	protein_coding	basic helix-loop-helix family, member a15 [Source:MGI Symbol;Acc:MGI:891976]	<0.01	0.028	1
Ighv1-15	ENSMUSG00000103254	IG_V_gene	immunoglobulin heavy variable 1-15 [Source:MGI Symbol;Acc:MGI:4439782]	<0.01	<0.01	1
Folr1	ENSMUSG00000001827	protein_coding	folate receptor 1 (adult) [Source:MGI Symbol;Acc:MGI:95568]	<0.01	<0.01	1
Dnah11	ENSMUSG00000018581	protein_coding	dynein, axonemal, heavy chain 11 [Source:MGI Symbol;Acc:MGI:1100864]	<0.01	0.014	1
Gm830	ENSMUSG00000084939	processed_transcript	predicted gene 830 [Source:MGI Symbol;Acc:MGI:2685676]	<0.01	<0.01	1
Gm27021	ENSMUSG00000097919	protein_coding	predicted gene, 27021 [Source:MGI Symbol;Acc:MGI:5504136]	<0.01	<0.01	1
Ltbp4	ENSMUSG00000040488	protein_coding	latent transforming growth factor beta binding protein 4 [Source:MGI Symbol;Acc:MGI:1321395]	<0.01	0.018	1
Nrarp	ENSMUSG00000078202	protein_coding	Notch-regulated ankyrin repeat protein [Source:MGI Symbol;Acc:MGI:1914372]	<0.01	0.022	1
Ahctf1	ENSMUSG00000026491	protein_coding	AT hook containing transcription factor 1 [Source:MGI Symbol;Acc:MGI:1915033]	<0.01	0.024	1
Col14a1	ENSMUSG00000022371	protein_coding	collagen, type XIV, alpha 1 [Source:MGI Symbol;Acc:MGI:1341272]	<0.01	0.036	1
Nbea	ENSMUSG00000027799	protein_coding	neurobeachin [Source:MGI Symbol;Acc:MGI:1347075]	<0.01	0.012	1
Atxn1	ENSMUSG00000046876	protein_coding	ataxin 1 [Source:MGI Symbol;Acc:MGI:104783]	<0.01	0.021	1
Snapc1	ENSMUSG00000021113	protein_coding	small nuclear RNA activating complex, polypeptide 1 [Source:MGI Symbol;Acc:MGI:1922877]	<0.01	0.011	1
Ahnak2	ENSMUSG00000072812	protein_coding	AHNAK nucleoprotein 2 [Source:MGI Symbol;Acc:MGI:2144831]	<0.01	0.014	1
Tecpr1	ENSMUSG00000066621	protein_coding	tectonin beta-propeller repeat containing 1 [Source:MGI Symbol;Acc:MGI:1917631]	<0.01	0.026	1
Arhgap20	ENSMUSG00000053199	protein_coding	Rho GTPase activating protein 20 [Source:MGI Symbol;Acc:MGI:2445175]	<0.01	0.016	1
Smad3	ENSMUSG00000032402	protein_coding	SMAD family member 3 [Source:MGI Symbol;Acc:MGI:1201674]	<0.01	0.013	1
Fat1	ENSMUSG00000070047	protein_coding	FAT atypical cadherin 1 [Source:MGI Symbol;Acc:MGI:109168]	<0.01	0.018	1
Gm28959	ENSMUSG00000100039	antisense_RNA	predicted gene 28959 [Source:MGI Symbol;Acc:MGI:5579665]	<0.01	0.040	1
Thra	ENSMUSG00000058756	protein_coding	thyroid hormone receptor alpha [Source:MGI Symbol;Acc:MGI:98742]	<0.01	0.034	1
Zfp523	ENSMUSG00000024220	protein_coding	zinc finger protein 523 [Source:MGI Symbol;Acc:MGI:2687278]	<0.01	0.050	1
D530018E20Rik	ENSMUSG00000107610	TEC	RIKEN cDNA D530018E20 gene [Source:MGI Symbol;Acc:MGI:1926117]	<0.01	0.046	1
Kcp	ENSMUSG00000059022	protein_coding	kielin/chordin-like protein [Source:MGI Symbol;Acc:MGI:2141640]	<0.01	0.015	1
Plxnb2	ENSMUSG00000036606	protein_coding	plexin B2 [Source:MGI Symbol;Acc:MGI:2154239]	<0.01	0.034	1
Vwf	ENSMUSG00000001930	protein_coding	Von Willebrand factor [Source:MGI Symbol;Acc:MGI:98941]	<0.01	0.027	1
Flii	ENSMUSG00000002812	protein_coding	flightless I actin binding protein [Source:MGI Symbol;Acc:MGI:1342286]	<0.01	0.050	1
Macf1	ENSMUSG00000028649	protein_coding	microtubule-actin crosslinking factor 1 [Source:MGI Symbol;Acc:MGI:108559]	<0.01	0.035	1
1810024B03Rik	ENSMUSG00000044145	protein_coding	RIKEN cDNA 1810024B03 gene [Source:MGI Symbol;Acc:MGI:1925560]	<0.01	0.026	1
Zfp354c	ENSMUSG00000044807	protein_coding	zinc finger protein 354C [Source:MGI Symbol;Acc:MGI:1353621]	<0.01	0.044	1
Mgat4b	ENSMUSG00000036620	protein_coding	mannoside acetylglucosaminyltransferase 4, isoenzyme B [Source:MGI Symbol;Acc:MGI:2143974]	<0.01	0.038	1
2810425M01Rik	ENSMUSG00000097811	lincRNA	RIKEN cDNA 2810425M01 gene [Source:MGI Symbol;Acc:MGI:1917222]	<0.01	0.035	1
Cpeb4	ENSMUSG00000020300	protein_coding	cytoplasmic polyadenylation element binding protein 4 [Source:MGI Symbol;Acc:MGI:1914829]	<0.01	0.025	1
Piezo1	ENSMUSG00000014444	protein_coding	piezo-type mechanosensitive ion channel component 1 [Source:MGI Symbol;Acc:MGI:3603204]	<0.01	0.035	1
Ppp2r5a	ENSMUSG00000026626	protein_coding	protein phosphatase 2, regulatory subunit B', alpha [Source:MGI Symbol;Acc:MGI:2388479]	<0.01	0.047	1
Tdp1	ENSMUSG00000021177	protein_coding	tyrosyl-DNA phosphodiesterase 1 [Source:MGI Symbol;Acc:MGI:1920036]	<0.01	0.045	1
Anpep	ENSMUSG00000039062	protein_coding	alanyl (membrane) aminopeptidase [Source:MGI Symbol;Acc:MGI:5000466]	<0.01	0.049	1
Smim3	ENSMUSG00000038059	protein_coding	small integral membrane protein 3 [Source:MGI Symbol;Acc:MGI:1917088]	<0.01	0.045	1
Gm19426	ENSMUSG00000097187	protein_coding	predicted gene, 19426 [Source:MGI Symbol;Acc:MGI:5011611]	<0.01	0.042	1
Syne2	ENSMUSG00000063450	protein_coding	spectrin repeat containing, nuclear envelope 2 [Source:MGI Symbol;Acc:MGI:2449316]	<0.01	0.025	1
2700046G09Rik	ENSMUSG00000097787	lincRNA	RIKEN cDNA 2700046G09 gene [Source:MGI Symbol;Acc:MGI:1914438]	<0.01	0.046	1
Rassf6	ENSMUSG00000029370	protein_coding	Ras association (RalGDS/AF-6) domain family member 6 [Source:MGI Symbol;Acc:MGI:1920496]	<0.01	0.023	1
Otulin	ENSMUSG00000046034	protein_coding	OTU deubiquitinase with linear linkage specificity [Source:MGI Symbol;Acc:MGI:3577015]	<0.01	0.029	1
Sppl3	ENSMUSG00000029550	protein_coding	signal peptide peptidase 3 [Source:MGI Symbol;Acc:MGI:1891433]	<0.01	0.026	1
Tada3	ENSMUSG00000048930	protein_coding	transcriptional adaptor 3 [Source:MGI Symbol;Acc:MGI:1915724]	<0.01	0.026	1
4930577N17Rik	ENSMUSG00000087440	TEC	RIKEN cDNA 4930577N17 gene [Source:MGI Symbol;Acc:MGI:1914996]	<0.01	0.042	1
Ephb3	ENSMUSG00000005958	protein_coding	Eph receptor B3 [Source:MGI Symbol;Acc:MGI:104770]	<0.01	0.033	1
R3hdm2	ENSMUSG00000025404	protein_coding	R3H domain containing 2 [Source:MGI Symbol;Acc:MGI:1919000]	<0.01	0.048	1
Arap1	ENSMUSG00000032812	protein_coding	ArfGAP with RhoGAP domain, ankyrin repeat and PH domain 1 [Source:MGI Symbol;Acc:MGI:1916960]	<0.01	0.029	1
Usp4	ENSMUSG00000032612	protein_coding	ubiquitin specific peptidase 4 (proto-oncogene) [Source:MGI Symbol;Acc:MGI:98905]	<0.01	0.045	1
Pde2a	ENSMUSG00000030653	protein_coding	predicted gene 45837 [Source:MGI Symbol;Acc:MGI:5804952]	<0.01	0.049	1
Myo5b	ENSMUSG00000025885	protein_coding	myosin VB [Source:MGI Symbol;Acc:MGI:106598]	<0.01	<0.01	1
Tpr	ENSMUSG00000006005	protein_coding	translocated promoter region, nuclear basket protein [Source:MGI Symbol;Acc:MGI:1922066]	<0.01	0.019	1
Myh13	ENSMUSG00000060180	protein_coding	myosin, heavy polypeptide 13, skeletal muscle [Source:MGI Symbol;Acc:MGI:1339967]	<0.01	0.043	1
Spry1	ENSMUSG00000037211	protein_coding	sprouty homolog 1 (Drosophila) [Source:MGI Symbol;Acc:MGI:1345139]	<0.01	<0.01	1
Msto1	ENSMUSG00000068922	protein_coding	misato 1, mitochondrial distribution and morphology regulator [Source:MGI Symbol;Acc:MGI:2385175]	<0.01	0.015	1
Eml1	ENSMUSG00000058070	protein_coding	echinoderm microtubule associated protein like 1 [Source:MGI Symbol;Acc:MGI:1915769]	<0.01	0.050	1
Pemt	ENSMUSG00000000301	protein_coding	phosphatidylethanolamine N-methyltransferase [Source:MGI Symbol;Acc:MGI:104535]	<0.01	0.033	1

Scmh1	ENSMUSG00000000085	protein_coding	sex comb on midleg homolog 1 [Source:MGI Symbol;Acc:MGI:1352762]	<0.01	0.034	1
Pkhd1l1	ENSMUSG00000038725	protein_coding	polycystic kidney and hepatic disease 1-like 1 [Source:MGI Symbol;Acc:MGI:2183153]	<0.01	0.047	1
Hace1	ENSMUSG00000038822	protein_coding	HECT domain and ankyrin repeat containing, E3 ubiquitin protein ligase 1 [Source:MGI Symbol;Acc:MGI:2446110]	<0.01	0.044	1
Gm38287	ENSMUSG00000103217	antisense_RNA	predicted gene, 38287 [Source:MGI Symbol;Acc:MGI:5611515]	<0.01	0.019	1
Ginm1	ENSMUSG00000040006	protein_coding	glycoprotein integral membrane 1 [Source:MGI Symbol;Acc:MGI:2384905]	<0.01	0.042	1
Sltm	ENSMUSG00000032212	protein_coding	SAFB-like, transcription modulator [Source:MGI Symbol;Acc:MGI:1913910]	<0.01	0.038	1
Cul7	ENSMUSG00000038545	protein_coding	cullin 7 [Source:MGI Symbol;Acc:MGI:1913765]	<0.01	<0.01	1
Tgm4	ENSMUSG00000025787	protein_coding	transglutaminase 4 (prostate) [Source:MGI Symbol;Acc:MGI:3027002]	<0.01	0.050	1
Lamc1	ENSMUSG00000026478	protein_coding	laminin, gamma 1 [Source:MGI Symbol;Acc:MGI:99914]	<0.01	0.034	1
Etl4	ENSMUSG00000036617	protein_coding	enhancer trap locus 4 [Source:MGI Symbol;Acc:MGI:95454]	<0.01	0.035	1
Pde8b	ENSMUSG00000021684	protein_coding	phosphodiesterase 8B [Source:MGI Symbol;Acc:MGI:2443999]	<0.01	0.026	1
Fam189a2	ENSMUSG00000071604	protein_coding	family with sequence similarity 189, member A2 [Source:MGI Symbol;Acc:MGI:2685813]	<0.01	0.021	1
Sepw1	ENSMUSG00000041571	protein_coding	selenoprotein W [Source:MGI Symbol;Acc:MGI:1100878]	<0.01	0.049	1
Gm43737	ENSMUSG00000105692	TEC	predicted gene 43737 [Source:MGI Symbol;Acc:MGI:5663874]	<0.01	0.024	1
Dhrs13os	ENSMUSG00000087050	antisense_RNA	dehydrogenase/reductase (SDR family) member 13, opposite strand [Source:MGI Symbol;Acc:MGI:1924478]	<0.01	0.042	1
Pcdhga8	ENSMUSG00000023036	protein_coding	protocadherin gamma subfamily C, 4 [Source:MGI Symbol;Acc:MGI:1935203]	<0.01	0.014	1
Mbd2	ENSMUSG00000024513	protein_coding	methyl-CpG binding domain protein 2 [Source:MGI Symbol;Acc:MGI:1333813]	<0.01	0.037	1
Gm4524	ENSMUSG00000090257	antisense_RNA	predicted gene 4524 [Source:MGI Symbol;Acc:MGI:3782709]	<0.01	0.050	1
Mroh1	ENSMUSG00000022558	protein_coding	maestro heat-like repeat family member 1 [Source:MGI Symbol;Acc:MGI:2442558]	<0.01	0.016	1
Mmp14	ENSMUSG00000000957	protein_coding	matrix metallopeptidase 14 (membrane-inserted) [Source:MGI Symbol;Acc:MGI:101900]	<0.01	0.042	1
Slc37a1	ENSMUSG00000024036	protein_coding	solute carrier family 37 (glycerol-3-phosphate transporter), member 1 [Source:MGI Symbol;Acc:MGI:2446181]	<0.01	0.040	1
Angptl8	ENSMUSG00000047822	protein_coding	angiopoietin-like 8 [Source:MGI Symbol;Acc:MGI:3643534]	<0.01	0.046	1
Esf1	ENSMUSG00000045624	protein_coding	ESF1 nucleolar pre-rRNA processing protein homolog [Source:MGI Symbol;Acc:MGI:1913830]	<0.01	0.040	1
Scrib	ENSMUSG00000022568	protein_coding	scribbled planar cell polarity [Source:MGI Symbol;Acc:MGI:2145950]	<0.01	0.038	1
Jup	ENSMUSG00000001552	protein_coding	junction plakoglobin [Source:MGI Symbol;Acc:MGI:96650]	<0.01	0.015	1
Leng1	ENSMUSG00000078813	protein_coding	leukocyte receptor cluster (LRC) member 1 [Source:MGI Symbol;Acc:MGI:1917007]	<0.01	0.044	1
Brd3	ENSMUSG00000026918	protein_coding	bromodomain containing 3 [Source:MGI Symbol;Acc:MGI:1914632]	<0.01	0.040	1
Nkx6-2	ENSMUSG00000041309	protein_coding	NK6 homeobox 2 [Source:MGI Symbol;Acc:MGI:1352738]	<0.01	0.028	1
Rgma	ENSMUSG00000070509	protein_coding	repulsive guidance molecule family member A [Source:MGI Symbol;Acc:MGI:2679262]	<0.01	0.049	1
0610009E02Rik	ENSMUSG00000086714	processed_transcript	RIKEN cDNA 0610009E02 gene [Source:MGI Symbol;Acc:MGI:3698435]	<0.01	0.021	1
Ing3	ENSMUSG00000029670	protein_coding	inhibitor of growth family, member 3 [Source:MGI Symbol;Acc:MGI:1919027]	<0.01	0.050	1
Osgin2	ENSMUSG00000041153	protein_coding	oxidative stress induced growth inhibitor family member 2 [Source:MGI Symbol;Acc:MGI:2384798]	<0.01	0.036	1
Tnk2	ENSMUSG00000022791	protein_coding	tyrosine kinase, non-receptor, 2 [Source:MGI Symbol;Acc:MGI:1858308]	<0.01	0.013	1
Sfrp1	ENSMUSG00000031548	protein_coding	secreted frizzled-related protein 1 [Source:MGI Symbol;Acc:MGI:892014]	<0.01	0.042	1
Trp53bp2	ENSMUSG00000026510	protein_coding	transformation related protein 53 binding protein 2 [Source:MGI Symbol;Acc:MGI:2138319]	<0.01	0.011	1
Hspg2	ENSMUSG00000028763	protein_coding	perlecan (heparan sulfate proteoglycan 2) [Source:MGI Symbol;Acc:MGI:96257]	<0.01	0.019	1
Kctd6	ENSMUSG00000021752	protein_coding	potassium channel tetramerisation domain containing 6 [Source:MGI Symbol;Acc:MGI:1918643]	<0.01	0.028	1
Rtn4rl1	ENSMUSG00000045287	protein_coding	reticulon 4 receptor-like 1 [Source:MGI Symbol;Acc:MGI:2661375]	<0.01	0.024	1
Mtrf1l	ENSMUSG00000019774	protein_coding	mitochondrial translational release factor 1-like [Source:MGI Symbol;Acc:MGI:1918830]	<0.01	0.015	1
Nppc	ENSMUSG00000026241	protein_coding	natriuretic peptide type C [Source:MGI Symbol;Acc:MGI:97369]	<0.01	0.013	1
6430573F11Rik	ENSMUSG00000039620	protein_coding	RIKEN cDNA 6430573F11 gene [Source:MGI Symbol;Acc:MGI:2442328]	<0.01	<0.01	1
Hyal1	ENSMUSG00000010051	protein_coding	hyaluronoglucosaminidase 1 [Source:MGI Symbol;Acc:MGI:96298]	<0.01	<0.01	1
Agrn	ENSMUSG00000041936	protein_coding	agrin [Source:MGI Symbol;Acc:MGI:87961]	<0.01	<0.01	1
Mex3b	ENSMUSG00000057706	protein_coding	mex3 RNA binding family member B [Source:MGI Symbol;Acc:MGI:1918252]	<0.01	0.033	1
Grin2c	ENSMUSG00000020734	protein_coding	glutamate receptor, ionotropic, NMDA2C (epsilon 3) [Source:MGI Symbol;Acc:MGI:95822]	<0.01	<0.01	1
Sh2d4a	ENSMUSG00000053886	protein_coding	SH2 domain containing 4A [Source:MGI Symbol;Acc:MGI:1919531]	<0.01	<0.01	1
Col12a1	ENSMUSG00000032332	protein_coding	collagen, type XII, alpha 1 [Source:MGI Symbol;Acc:MGI:88448]	<0.01	<0.01	1
Scn7a	ENSMUSG00000034810	protein_coding	sodium channel, voltage-gated, type VII, alpha [Source:MGI Symbol;Acc:MGI:102965]	<0.01	0.010	1
Emcn	ENSMUSG00000054690	protein_coding	endomucin [Source:MGI Symbol;Acc:MGI:1891716]	<0.01	0.028	1
Tmtc2	ENSMUSG00000036019	protein_coding	transmembrane and tetratricopeptide repeat containing 2 [Source:MGI Symbol;Acc:MGI:1914057]	<0.01	0.011	1
Cpd	ENSMUSG00000020841	protein_coding	carboxypeptidase D [Source:MGI Symbol;Acc:MGI:107265]	<0.01	0.026	1
Clmp	ENSMUSG00000032024	protein_coding	CXADR-like membrane protein [Source:MGI Symbol;Acc:MGI:1918816]	<0.01	0.016	1
Car15	ENSMUSG00000090236	protein_coding	carbonic anhydrase 15 [Source:MGI Symbol;Acc:MGI:1931324]	<0.01	0.041	1
Gprc5a	ENSMUSG00000046733	protein_coding	G protein-coupled receptor, family C, group 5, member A [Source:MGI Symbol;Acc:MGI:1891250]	<0.01	0.018	1
Cntln	ENSMUSG00000038070	protein_coding	centlein, centrosomal protein [Source:MGI Symbol;Acc:MGI:2443104]	<0.01	0.018	1
Myh10	ENSMUSG00000020900	protein_coding	myosin, heavy polypeptide 10, non-muscle [Source:MGI Symbol;Acc:MGI:1930780]	<0.01	0.040	1
Ankrd13b	ENSMUSG00000037907	protein_coding	ankyrin repeat domain 13b [Source:MGI Symbol;Acc:MGI:2144501]	<0.01	0.025	1
Podxl2	ENSMUSG00000033152	protein_coding	podocalyxin-like 2 [Source:MGI Symbol;Acc:MGI:2442488]	<0.01	0.023	1
Mgll	ENSMUSG00000033174	protein_coding	monoglyceride lipase [Source:MGI Symbol;Acc:MGI:1346042]	<0.01	<0.01	1
Chst1	ENSMUSG00000027221	protein_coding	carbohydrate (keratan sulfate Gal-6) sulfotransferase 1 [Source:MGI Symbol;Acc:MGI:1924219]	<0.01	0.028	1
Ptprd	ENSMUSG00000028399	protein_coding	protein tyrosine phosphatase, receptor type, D [Source:MGI Symbol;Acc:MGI:97812]	<0.01	0.026	1
Tcte2	ENSMUSG00000038347	protein_coding	t-complex-associated testis expressed 2 [Source:MGI Symbol;Acc:MGI:98641]	<0.01	0.014	1
Dynlt1b	ENSMUSG00000096255	protein_coding	dynein light chain Tctex-type 1B [Source:MGI Symbol;Acc:MGI:98643]	<0.01	<0.01	1
Gm13375	ENSMUSG00000075514	antisense_RNA	predicted gene 13375 [Source:MGI Symbol;Acc:MGI:3649913]	<0.01	0.019	1
Msx1	ENSMUSG00000048450	protein_coding	msh homeobox 1 [Source:MGI Symbol;Acc:MGI:97168]	<0.01	0.049	1
Zfp652os	ENSMUSG00000086191	antisense_RNA	zinc finger protein 652, opposite strand [Source:MGI Symbol;Acc:MGI:3044900]	<0.01	0.021	1
Smarca2	ENSMUSG00000024921	protein_coding	SWI/SNF related, matrix associated, actin dependent regulator of chromatin, subfamily a, member 2 [Source:MGI Symbol;Acc:MGI:99603]	<0.01	0.014	1
Reln	ENSMUSG00000042453	protein_coding	reelin [Source:MGI Symbol;Acc:MGI:103022]	<0.01	0.012	1
Anxa3	ENSMUSG00000029484	protein_coding	annexin A3 [Source:MGI Symbol;Acc:MGI:1201378]	<0.01	<0.01	1
Fryl	ENSMUSG00000070733	protein_coding	FRY like transcription coactivator [Source:MGI Symbol;Acc:MGI:1919563]	<0.01	0.019	1
Ppp1r37	ENSMUSG00000051403	protein_coding	protein phosphatase 1, regulatory subunit 37 [Source:MGI Symbol;Acc:MGI:2687042]	<0.01	0.031	1
Gm17122	ENSMUSG00000090458	antisense_RNA	predicted gene 17122 [Source:MGI Symbol;Acc:MGI:4937949]	<0.01	0.029	1
Hopx	ENSMUSG00000059325	protein_coding	HOP homeobox [Source:MGI Symbol;Acc:MGI:1916782]	<0.01	0.011	1
D330041H03Rik	ENSMUSG00000073437	processed_transcript	RIKEN cDNA D330041H03 gene [Source:MGI Symbol;Acc:MGI:3603827]	<0.01	0.013	1
Tacc2	ENSMUSG00000030852	protein_coding	transforming, acidic coiled-coil containing protein 2 [Source:MGI Symbol;Acc:MGI:1928899]	<0.01	0.020	1
Pbx3	ENSMUSG00000038718	protein_coding	pre B cell leukemia homeobox 3 [Source:MGI Symbol;Acc:MGI:97496]	<0.01	0.026	1
Irs1	ENSMUSG00000055980	protein_coding	insulin receptor substrate 1 [Source:MGI Symbol;Acc:MGI:99454]	<0.01	0.033	1
2310033P09Rik	ENSMUSG00000020441	protein_coding	RIKEN cDNA 2310033P09 gene [Source:MGI Symbol;Acc:MGI:1915112]	<0.01	0.028	1
Hist2h2aa2	ENSMUSG00000063954	protein_coding	histone cluster 2, H2aa2 [Source:MGI Symbol;Acc:MGI:2448283]	<0.01	<0.01	1
Gm5161	ENSMUSG00000061486	processed_pseudogene	predicted pseudogene 5161 [Source:MGI Symbol;Acc:MGI:3648529]	<0.01	0.023	1

Usp13	ENSMUSG00000056900	protein_coding	ubiquitin specific peptidase 13 (isopeptidase T-3) [Source:MGI Symbol;Acc:MGI:1919857]	<0.01	0.022	1
Gm26733	ENSMUSG00000097469	lincRNA	predicted gene, 26733 [Source:MGI Symbol;Acc:MGI:5477227]	<0.01	0.033	1
Map1b	ENSMUSG00000052727	protein_coding	microtubule-associated protein 1B [Source:MGI Symbol;Acc:MGI:1306778]	<0.01	0.016	1
Igfbp2	ENSMUSG00000039323	protein_coding	insulin-like growth factor binding protein 2 [Source:MGI Symbol;Acc:MGI:96437]	<0.01	0.036	1
Fgfrl1	ENSMUSG00000008090	protein_coding	fibroblast growth factor receptor-like 1 [Source:MGI Symbol;Acc:MGI:2150920]	<0.01	0.016	1
Fbxw7	ENSMUSG00000028086	protein_coding	F-box and WD-40 domain protein 7 [Source:MGI Symbol;Acc:MGI:1354695]	<0.01	0.025	1
Spata5	ENSMUSG00000027722	protein_coding	spermatogenesis associated 5 [Source:MGI Symbol;Acc:MGI:1927170]	<0.01	0.012	1
Tuba8	ENSMUSG00000030137	protein_coding	tubulin, alpha 8 [Source:MGI Symbol;Acc:MGI:1858225]	<0.01	0.021	1
Gm43719	ENSMUSG00000105366	TEC	predicted gene 43719 [Source:MGI Symbol;Acc:MGI:5663856]	<0.01	0.013	1
Leo1	ENSMUSG00000042487	protein_coding	Leo1, Paf1/RNA polymerase II complex component [Source:MGI Symbol;Acc:MGI:2685031]	<0.01	<0.01	1
Gm13889	ENSMUSG00000087006	protein_coding	predicted gene 13889 [Source:MGI Symbol;Acc:MGI:3652053]	<0.01	<0.01	1
Mthfd1	ENSMUSG00000021048	protein_coding	methylenetetrahydrofolate dehydrogenase (NADP+ dependent), methenyltetrahydrofolate cyclohydrolase, formyltetrahydrofolate synthase [Source:MGI Symbol;Acc:MGI:1342005]	<0.01	<0.01	1
Trpv6	ENSMUSG00000029868	protein_coding	transient receptor potential cation channel, subfamily V, member 6 [Source:MGI Symbol;Acc:MGI:1927259]	<0.01	<0.01	1
Glipr2	ENSMUSG00000028480	protein_coding	GLI pathogenesis-related 2 [Source:MGI Symbol;Acc:MGI:1917770]	<0.01	<0.01	1
Creb3l1	ENSMUSG00000027230	protein_coding	cAMP responsive element binding protein 3-like 1 [Source:MGI Symbol;Acc:MGI:1347062]	<0.01	0.012	1
Spon2	ENSMUSG00000037379	protein_coding	spondin 2, extracellular matrix protein [Source:MGI Symbol;Acc:MGI:1923724]	<0.01	<0.01	1
Rasl11a	ENSMUSG00000029641	protein_coding	RAS-like, family 11, member A [Source:MGI Symbol;Acc:MGI:1916145]	<0.01	<0.01	1
Adm	ENSMUSG00000030790	protein_coding	adrenomedullin [Source:MGI Symbol;Acc:MGI:108058]	<0.01	<0.01	1
Arntl	ENSMUSG00000055116	protein_coding	aryl hydrocarbon receptor nuclear translocator-like [Source:MGI Symbol;Acc:MGI:1096381]	<0.01	<0.01	1
Serpina3c	ENSMUSG00000066361	protein_coding	serine (or cysteine) peptidase inhibitor, clade A, member 3C [Source:MGI Symbol;Acc:MGI:102848]	<0.01	<0.01	1
Eln	ENSMUSG00000029675	protein_coding	elastin [Source:MGI Symbol;Acc:MGI:95317]	<0.01	<0.01	1
Npas2	ENSMUSG00000026077	protein_coding	neuronal PAS domain protein 2 [Source:MGI Symbol;Acc:MGI:109232]	<0.01	<0.01	1
Gm12942	ENSMUSG00000070737	protein_coding	transmembrane protein 35B [Source:MGI Symbol;Acc:MGI:3758095]	<0.01	0.020	1
Rbm8a	ENSMUSG00000038374	protein_coding	RNA binding motif protein 8a [Source:MGI Symbol;Acc:MGI:1913129]	<0.01	0.037	1
Afap1l2	ENSMUSG00000025083	protein_coding	actin filament associated protein 1-like 2 [Source:MGI Symbol;Acc:MGI:2147658]	<0.01	0.035	1
Alkbh3	ENSMUSG00000040174	protein_coding	alkB homolog 3, alpha-ketoglutarate-dependent dioxygenase [Source:MGI Symbol;Acc:MGI:1916363]	<0.01	0.040	1
Vps53	ENSMUSG00000017288	protein_coding	VPS53 GARP complex subunit [Source:MGI Symbol;Acc:MGI:1915549]	<0.01	0.045	1
Ikzf4	ENSMUSG00000002578	protein_coding	IKAROS family zinc finger 4 [Source:MGI Symbol;Acc:MGI:1343139]	<0.01	0.019	1
Alg9	ENSMUSG00000032059	protein_coding	asparagine-linked glycosylation 9 (alpha 1,2 mannosyltransferase) [Source:MGI Symbol;Acc:MGI:1924753]	<0.01	0.036	1
Ypel2	ENSMUSG00000018427	protein_coding	yippee-like 2 (Drosophila) [Source:MGI Symbol;Acc:MGI:1925114]	<0.01	0.017	1
Ccdc142os	ENSMUSG00000087578	antisense_RNA	coiled-coil domain containing 142, opposite strand [Source:MGI Symbol;Acc:MGI:3783052]	<0.01	0.035	1
Ap2s1	ENSMUSG00000008036	protein_coding	adaptor-related protein complex 2, sigma 1 subunit [Source:MGI Symbol;Acc:MGI:2141861]	<0.01	0.045	1
Gm15512	ENSMUSG00000087639	antisense_RNA	predicted gene 15512 [Source:MGI Symbol;Acc:MGI:3782960]	<0.01	0.035	1
Scx	ENSMUSG00000034161	protein_coding	scleraxis [Source:MGI Symbol;Acc:MGI:102934]	<0.01	0.033	1
5430416O09Rik	ENSMUSG00000028475	protein_coding	small regulatory polypeptide of amino acid response [Source:MGI Symbol;Acc:MGI:1918656]	<0.01	0.012	1
Gadd45a	ENSMUSG00000036390	protein_coding	growth arrest and DNA-damage-inducible 45 alpha [Source:MGI Symbol;Acc:MGI:107799]	<0.01	0.035	1
Ckb	ENSMUSG00000001270	protein_coding	creatine kinase, brain [Source:MGI Symbol;Acc:MGI:88407]	<0.01	0.017	1
Cwc27	ENSMUSG00000021715	protein_coding	CWC27 spliceosome-associated protein [Source:MGI Symbol;Acc:MGI:1914535]	<0.01	0.015	1
Gpatch11	ENSMUSG00000050668	protein_coding	G patch domain containing 11 [Source:MGI Symbol;Acc:MGI:1858435]	<0.01	0.035	1
Fam20a	ENSMUSG00000020614	protein_coding	family with sequence similarity 20, member A [Source:MGI Symbol;Acc:MGI:2388266]	<0.01	0.021	1
Pfdn6	ENSMUSG00000024309	protein_coding	prefoldin subunit 6 [Source:MGI Symbol;Acc:MGI:95908]	<0.01	0.034	1
Rmrp	ENSMUSG00000088088	ribozyme	RNA component of mitochondrial RNAase P [Source:MGI Symbol;Acc:MGI:97937]	<0.01	<0.01	1
Ccdc112	ENSMUSG00000071855	protein_coding	coiled-coil domain containing 112 [Source:MGI Symbol;Acc:MGI:1918800]	<0.01	0.021	1
Tekt5	ENSMUSG00000039179	protein_coding	tektin 5 [Source:MGI Symbol;Acc:MGI:1917676]	<0.01	0.028	1
Nupr1	ENSMUSG00000030717	protein_coding	nuclear protein transcription regulator 1 [Source:MGI Symbol;Acc:MGI:1891834]	<0.01	0.023	1
Ccnjl	ENSMUSG00000044707	protein_coding	cyclin J-like [Source:MGI Symbol;Acc:MGI:2685723]	<0.01	0.020	1
Fjx1	ENSMUSG00000075012	protein_coding	four jointed box 1 (Drosophila) [Source:MGI Symbol;Acc:MGI:1341907]	<0.01	0.040	1
Tulp1	ENSMUSG00000037446	protein_coding	tubby like protein 1 [Source:MGI Symbol;Acc:MGI:109571]	<0.01	0.012	1
Lpar6	ENSMUSG00000033446	protein_coding	lysophosphatidic acid receptor 6 [Source:MGI Symbol;Acc:MGI:1914418]	<0.01	0.012	1
Kcna2	ENSMUSG00000040724	protein_coding	potassium voltage-gated channel, shaker-related subfamily, member 2 [Source:MGI Symbol;Acc:MGI:96659]	<0.01	0.019	1
Prkg2	ENSMUSG00000029334	protein_coding	protein kinase, cGMP-dependent, type II [Source:MGI Symbol;Acc:MGI:108173]	<0.01	<0.01	1
Copz2	ENSMUSG00000018672	protein_coding	coatomer protein complex, subunit zeta 2 [Source:MGI Symbol;Acc:MGI:1929008]	<0.01	0.035	1
Hoxb2	ENSMUSG00000075588	protein_coding	homeobox B2 [Source:MGI Symbol;Acc:MGI:96183]	<0.01	0.042	1
Gm16556	ENSMUSG00000090192	processed_transcript	predicted gene 16556 [Source:MGI Symbol;Acc:MGI:4414976]	<0.01	<0.01	1
Gm37192	ENSMUSG00000102184	TEC	predicted gene, 37192 [Source:MGI Symbol;Acc:MGI:5610420]	<0.01	<0.01	1
Gm37660	ENSMUSG00000104467	TEC	predicted gene, 37660 [Source:MGI Symbol;Acc:MGI:5610888]	<0.01	0.017	1
Ccdc184	ENSMUSG00000029875	protein_coding	coiled-coil domain containing 184 [Source:MGI Symbol;Acc:MGI:2146066]	<0.01	<0.01	1
Gm43573	ENSMUSG00000104786	antisense_RNA	predicted gene 43573 [Source:MGI Symbol;Acc:MGI:5663710]	<0.01	0.022	1
Msl3l2	ENSMUSG00000047669	protein_coding	male-specific lethal 3-like 2 (Drosophila) [Source:MGI Symbol;Acc:MGI:1920640]	<0.01	0.020	1
Plk4	ENSMUSG00000025758	protein_coding	polo-like kinase 4 [Source:MGI Symbol;Acc:MGI:101783]	<0.01	<0.01	1
Pla1a	ENSMUSG00000002847	protein_coding	phospholipase A1 member A [Source:MGI Symbol;Acc:MGI:1934677]	<0.01	0.019	1
Vstm5	ENSMUSG00000031937	protein_coding	V-set and transmembrane domain containing 5 [Source:MGI Symbol;Acc:MGI:1916387]	<0.01	0.030	1
Sapcd1	ENSMUSG00000036185	protein_coding	suppressor APC domain containing 1 [Source:MGI Symbol;Acc:MGI:2388100]	<0.01	<0.01	1
Fam167b	ENSMUSG00000050493	protein_coding	family with sequence similarity 167, member B [Source:MGI Symbol;Acc:MGI:2668032]	<0.01	<0.01	1
Pabpc1l	ENSMUSG00000054582	protein_coding	poly(A) binding protein, cytoplasmic 1-like [Source:MGI Symbol;Acc:MGI:1922908]	<0.01	<0.01	1
Thg1l	ENSMUSG00000011254	protein_coding	tRNA-histidine guanylyltransferase 1-like (S. cerevisiae) [Source:MGI Symbol;Acc:MGI:1913878]	<0.01	<0.01	1

Note: Decide test value "-1" or "1" indicates the t-statistic is classified as significantly negative or significantly positive respectively

Table S4a. Gene Set Enrichment Analysis

Strain

Pathway Name	Pathway size	Enrichment Score	NOM.P.value	FDR.q.value	FWER.p.value	RANK.AT.MAX	Enriched Symbol	Title	Rank in Gene List	Rank Metric Score	Running Enrichment Score
**ANGIOGENESIS**	33	0.46061426	0.01237113	0.02116199	0.04	1713	SPP1	secreted phosphoprotein 1 [Source:HGNC Symbol;Acc:HGNC:11255]	185	1.418506622	0.044493295
							MSX1	msh homeobox 1 [Source:HGNC Symbol;Acc:HGNC:7391]	201	1.389016628	0.10012972
							PGLYRP1	peptidoglycan recognition protein 1 [Source:HGNC Symbol;Acc:HGNC:8904]	241	1.292899966	0.15009768
							OLR1	oxidized low density lipoprotein receptor 1 [Source:HGNC Symbol;Acc:HGNC:8133]	286	1.21445334	0.1964988
							ITGAV	integrin subunit alpha V [Source:HGNC Symbol;Acc:HGNC:6150]	346	1.149060011	0.23913985
							STC1	stanniocalcin 1 [Source:HGNC Symbol;Acc:HGNC:11373]	363	1.126273274	0.2839735
							CXCL6	C-X-C motif chemokine ligand 6 [Source:HGNC Symbol;Acc:HGNC:10643]	381	1.107283354	0.32795897
							PF4	platelet factor 4 [Source:HGNC Symbol;Acc:HGNC:8861]	454	1.049976707	0.36560935
							VTN	vitronectin [Source:HGNC Symbol;Acc:HGNC:12724]	592	0.959326684	0.3948366
							POSTN	periostin [Source:HGNC Symbol;Acc:HGNC:16953]	933	0.818616688	0.40357307
							PDGFA	platelet derived growth factor subunit A [Source:HGNC Symbol;Acc:HGNC:8799]	1170	0.757513344	0.41736788
							APOH	apolipoprotein H [Source:HGNC Symbol;Acc:HGNC:616]	1423	0.695333362	0.4274612
							COL5A2	collagen type V alpha 2 chain [Source:HGNC Symbol;Acc:HGNC:2210]	1504	0.68053335	0.44944286
							S100A4	S100 calcium binding protein A4 [Source:HGNC Symbol;Acc:HGNC:10494]	1713	0.643476665	0.46061426
**WNT BETA CATENIN SIGNALING**											
**BILE ACID METABOLISM**	95	-0.31486982	0.0139165	0.14943145	0.506	3449	SCP2	sterol carrier protein 2 [Source:HGNC Symbol;Acc:HGNC:10606]	10353	-0.427520007	-0.30629283
							CROT	carnitine O-octanoyltransferase [Source:HGNC Symbol;Acc:HGNC:2366]	10399	-0.43197	-0.30090982
							BMP6	bone morphogenetic protein 6 [Source:HGNC Symbol;Acc:HGNC:1073]	10400	-0.431973338	-0.2922435
							PEX6	peroxisomal biogenesis factor 6 [Source:HGNC Symbol;Acc:HGNC:8859]	10514	-0.442270011	-0.2916152
							HSD17B4	hydroxysteroid 17-beta dehydrogenase 4 [Source:HGNC Symbol;Acc:HGNC:5213]	10631	-0.453893334	-0.29097256
							RXRA	retinoid X receptor alpha [Source:HGNC Symbol;Acc:HGNC:10477]	10681	-0.459426671	-0.28533056
							ABCA3	ATP binding cassette subfamily A member 3 [Source:HGNC Symbol;Acc:HGNC:33]	10744	-0.465623319	-0.28051272
							GNPAT	glyceronephosphate O-acyltransferase [Source:HGNC Symbol;Acc:HGNC:4416]	11064	-0.499926656	-0.2937576
							PNPLA8	patatin like phospholipase domain containing 8 [Source:HGNC Symbol;Acc:HGNC:28900]	11077	-0.501820028	-0.28456554
							IDH1	isocitrate dehydrogenase (NADP(+)) 1 [Source:HGNC Symbol;Acc:HGNC:5382]	11269	-0.522189975	-0.28802478
							DHCR24	24-dehydrocholesterol reductase [Source:HGNC Symbol;Acc:HGNC:2859]	11382	-0.534493327	-0.2854733
							HACL1	2-hydroxyacyl-CoA lyase 1 [Source:HGNC Symbol;Acc:HGNC:17856]	11438	-0.541170001	-0.2786291
							ABCD3	ATP binding cassette subfamily D member 3 [Source:HGNC Symbol;Acc:HGNC:67]	11576	-0.558516681	-0.2774197
							NR3C2	nuclear receptor subfamily 3 group C member 2 [Source:HGNC Symbol;Acc:HGNC:7979]	11579	-0.558690012	-0.2663571
							LCK	LCK proto-oncogene, Src family tyrosine kinase [Source:HGNC Symbol;Acc:HGNC:6524]	11744	-0.582599998	-0.26663443
							ABCA1	ATP binding cassette subfamily A member 1 [Source:HGNC Symbol;Acc:HGNC:29]	11819	-0.59187001	-0.26015934
							ALDH9A1	aldehyde dehydrogenase 9 family member A1 [Source:HGNC Symbol;Acc:HGNC:412]	11834	-0.593946695	-0.24926494
							EFHC1	EF-hand domain containing 1 [Source:HGNC Symbol;Acc:HGNC:16406]	12040	-0.625696659	-0.25166908
							DIO1	iodothyronine deiodinase 1 [Source:HGNC Symbol;Acc:HGNC:2883]	12105	-0.637266695	-0.24355362
							SLCO1A2	solute carrier organic anion transporter family member 1A2 [Source:HGNC Symbol;Acc:HGNC:10956]	12147	-0.643769979	-0.2336296
							NPC1	NPC intracellular cholesterol transporter 1 [Source:HGNC Symbol;Acc:HGNC:7897]	12267	-0.665570021	-0.22895917
							PFKM	phosphofructokinase, muscle [Source:HGNC Symbol;Acc:HGNC:8877]	12405	-0.693333328	-0.22504504
							HSD3B7	hydroxy-delta-5-steroid dehydrogenase, 3 beta- and steroid delta-isomerase 7 [Source:HGNC Symbol;Acc:HGNC:18324]	12562	-0.727653325	-0.22182862
							NEDD4	NEDD4 E3 ubiquitin protein ligase [Source:HGNC Symbol;Acc:HGNC:7727]	12692	-0.760969996	-0.21597387
							PEX11A	peroxisomal biogenesis factor 11 alpha [Source:HGNC Symbol;Acc:HGNC:8852]	12712	-0.767356694	-0.20196529
							PEX7	peroxisomal biogenesis factor 7 [Source:HGNC Symbol;Acc:HGNC:8860]	12774	-0.784080029	-0.19068557
							BCAR3	BCAR3 adaptor protein, NSP family member [Source:HGNC Symbol;Acc:HGNC:973]	12962	-0.839783311	-0.18748136
							RETSAT	retinol saturase [Source:HGNC Symbol;Acc:HGNC:25991]	12968	-0.843850017	-0.17091672
							ALDH1A1	aldehyde dehydrogenase 1 family member A1 [Source:HGNC Symbol;Acc:HGNC:402]	13061	-0.889036655	-0.15979312
							FADS2	fatty acid desaturase 2 [Source:HGNC Symbol;Acc:HGNC:3575]	13193	-0.947350025	-0.1503451
							CYP39A1	cytochrome P450 family 39 subfamily A member 1 [Source:HGNC Symbol;Acc:HGNC:17449]	13245	-0.968376696	-0.13463838
							IDI1	isopentenyl-diphosphate delta isomerase 1 [Source:HGNC Symbol;Acc:HGNC:5387]	13268	-0.987006664	-0.11644204
							TFCP2L1	transcription factor CP2 like 1 [Source:HGNC Symbol;Acc:HGNC:17925]	13302	-1.003163338	-0.098724134
							EPHX2	epoxide hydrolase 2 [Source:HGNC Symbol;Acc:HGNC:3402]	13324	-1.019556642	-0.079801805
							ABCA5	ATP binding cassette subfamily A member 5 [Source:HGNC Symbol;Acc:HGNC:35]	13333	-1.02936995	-0.059734117
							GNMT	glycine N-methyltransferase [Source:HGNC Symbol;Acc:HGNC:4415]	13341	-1.039503336	-0.03939017
							ACSL1	acyl-CoA synthetase long chain family member 1 [Source:HGNC Symbol;Acc:HGNC:3569]	13473	-1.17513001	-0.025372392
							ABCA2	ATP binding cassette subfamily A member 2 [Source:HGNC Symbol;Acc:HGNC:32]	13521	-1.20796001	-0.004567271
							ISOC1	isochorismatase domain containing 1 [Source:HGNC Symbol;Acc:HGNC:24254]	13542	-1.238659978	0.018823698
**TNFA SIGNALING VIA NFKB**	180	-0.33760515	0	0.21819851	0.357	2595	IL12B	interleukin 12B [Source:HGNC Symbol;Acc:HGNC:5970]	11207	-0.515919983	-0.33281377
							RELA	RELA proto-oncogene, NF-kB subunit [Source:HGNC Symbol;Acc:HGNC:9955]	11228	-0.517629981	-0.32947487
							BTG1	BTG anti-proliferation factor 1 [Source:HGNC Symbol;Acc:HGNC:1130]	11236	-0.518503308	-0.3251734
							CCNL1	cyclin L1 [Source:HGNC Symbol;Acc:HGNC:20569]	11245	-0.519333363	-0.32093766
							KLF6	Kruppel like factor 6 [Source:HGNC Symbol;Acc:HGNC:2235]	11257	-0.520950019	-0.31690717
							KDM6B	lysine demethylase 6B [Source:HGNC Symbol;Acc:HGNC:29012]	11263	-0.521476686	-0.31243128
							CCND1	cyclin D1 [Source:HGNC Symbol;Acc:HGNC:1582]	11279	-0.523143351	-0.30867407
							SQSTM1	sequestosome 1 [Source:HGNC Symbol;Acc:HGNC:11280]	11341	-0.529603362	-0.308234
							TLR2	toll like receptor 2 [Source:HGNC Symbol;Acc:HGNC:11848]	11380	-0.534143329	-0.3060632
							ZBTB10	zinc finger and BTB domain containing 10 [Source:HGNC Symbol;Acc:HGNC:30953]	11409	-0.537329972	-0.30312866
							ICAM1	intercellular adhesion molecule 1 [Source:HGNC Symbol;Acc:HGNC:5344]	11436	-0.541023314	-0.30001298
							PLK2	polo like kinase 2 [Source:HGNC Symbol;Acc:HGNC:19699]	11467	-0.544340014	-0.29716015
							BCL3	BCL3 transcription coactivator [Source:HGNC Symbol;Acc:HGNC:998]	11560	-0.556949973	-0.29874203
							JUN	Jun proto-oncogene, AP-1 transcription factor subunit [Source:HGNC Symbol;Acc:HGNC:6204]	11591	-0.560363352	-0.2957404
							IFNGR2	interferon gamma receptor 2 [Source:HGNC Symbol;Acc:HGNC:5440]	11668	-0.572526693	-0.29600292
							FUT4	fucosyltransferase 4 [Source:HGNC Symbol;Acc:HGNC:4015]	11734	-0.58217001	-0.29536834
							BIRC3	baculoviral IAP repeat containing 3 [Source:HGNC Symbol;Acc:HGNC:591]	11748	-0.583530009	-0.29090348
							CD83	CD83 molecule [Source:HGNC Symbol;Acc:HGNC:1703]	11764	-0.585959971	-0.2865629
							CCL2	C-C motif chemokine ligand 2 [Source:HGNC Symbol;Acc:HGNC:10618]	11766	-0.586263359	-0.28119168
							F2RL1	F2R like trypsin receptor 1 [Source:HGNC Symbol;Acc:HGNC:3538]	11791	-0.588776648	-0.27748567
							ABCA1	ATP binding cassette subfamily A member 1 [Source:HGNC Symbol;Acc:HGNC:29]	11819	-0.59187001	-0.27397117
							F3	coagulation factor III, tissue factor [Source:HGNC Symbol;Acc:HGNC:3541]	11820	-0.591936648	-0.26847383
							CD69	CD69 molecule [Source:HGNC Symbol;Acc:HGNC:1694]	11859	-0.598246694	-0.26570773
							JAG1	jagged canonical Notch ligand 1 [Source:HGNC Symbol;Acc:HGNC:6188]	11942	-0.610096693	-0.26606184
							FOSB	FosB proto-oncogene, AP-1 transcription factor subunit [Source:HGNC Symbol;Acc:HGNC:3797]	11988	-0.617846668	-0.26362762
							PLPP3	phospholipid phosphatase 3 [Source:HGNC Symbol;Acc:HGNC:9229]	12014	-0.621843338	-0.25968793
							HES1	hes family bHLH transcription factor 1 [Source:HGNC Symbol;Acc:HGNC:5192]	12038	-0.625566661	-0.25556684
							IRS2	insulin receptor substrate 2 [Source:HGNC Symbol;Acc:HGNC:6126]	12085	-0.634119987	-0.2530549
							TNFRSF9	TNF receptor superfamily member 9 [Source:HGNC Symbol;Acc:HGNC:11924]	12103	-0.637040019	-0.24838674
							GPR183	G protein-coupled receptor 183 [Source:HGNC Symbol;Acc:HGNC:3128]	12149	-0.644206643	-0.24570769
							LDLR	low density lipoprotein receptor [Source:HGNC Symbol;Acc:HGNC:6547]	12150	-0.644280016	-0.23972425
							MAP2K3	mitogen-activated protein kinase kinase 3 [Source:HGNC Symbol;Acc:HGNC:6843]	12166	-0.647173345	-0.23481517
							MCL1	MCL1 apoptosis regulator, BCL2 family member [Source:HGNC Symbol;Acc:HGNC:6943]	12167	-0.647279978	-0.22880386
							SDC4	syndecan 4 [Source:HGNC Symbol;Acc:HGNC:10661]	12184	-0.650046647	-0.2239415
							TNFAIP8	TNF alpha induced protein 8 [Source:HGNC Symbol;Acc:HGNC:17260]	12191	-0.651413321	-0.2183323
							EDN1	endothelin 1 [Source:HGNC Symbol;Acc:HGNC:3176]	12205	-0.654429972	-0.213209
							IL7R	interleukin 7 receptor [Source:HGNC Symbol;Acc:HGNC:6024]	12262	-0.66470331	-0.21114719
							NR4A2	nuclear receptor subfamily 4 group A member 2 [Source:HGNC Symbol;Acc:HGNC:7981]	12411	-0.694113314	-0.2155665
							PPP1R15A	protein phosphatase 1 regulatory subunit 15A [Source:HGNC Symbol;Acc:HGNC:14375]	12429	-0.698536694	-0.21032725
							TNFAIP6	TNF alpha induced protein 6 [Source:HGNC Symbol;Acc:HGNC:11898]	12439	-0.700743318	-0.20448017
							B4GALT5	beta-1,4-galactosyltransferase 5 [Source:HGNC Symbol;Acc:HGNC:928]	12449	-0.702663362	-0.19861525
							RHOB	ras homolog family member B [Source:HGNC Symbol;Acc:HGNC:668]	12476	-0.708573341	-0.19394353
							TNFAIP2	TNF alpha induced protein 2 [Source:HGNC Symbol;Acc:HGNC:11895]	12488	-0.711963356	-0.18813908
							EGR1	early growth response 1 [Source:HGNC Symbol;Acc:HGNC:3238]	12527	-0.720200002	-0.18424036
							SMAD3	SMAD family member 3 [Source:HGNC Symbol;Acc:HGNC:6769]	12623	-0.744603336	-0.18429974
							CCL5	C-C motif chemokine ligand 5 [Source:HGNC Symbol;Acc:HGNC:10632]	12631	-0.746720016	-0.17787883
							JUNB	JunB proto-oncogene, AP-1 transcription factor subunit [Source:HGNC Symbol;Acc:HGNC:6205]	12646	-0.750383317	-0.17193782
							ZC3H12A	zinc finger CCCH-type containing 12A [Source:HGNC Symbol;Acc:HGNC:26259]	12655	-0.753356695	-0.1655287
							CCN1	cellular communication network factor 1 [Source:HGNC Symbol;Acc:HGNC:2654]	12667	-0.756123304	-0.15931414
							SOCS3	suppressor of cytokine signaling 3 [Source:HGNC Symbol;Acc:HGNC:19391]	12746	-0.776639998	-0.1578279
							KLF9	Kruppel like factor 9 [Source:HGNC Symbol;Acc:HGNC:1123]	12776	-0.784556687	-0.15267077
							YRDC	yrdC N6-threonylcarbamoyltransferase domain containing [Source:HGNC Symbol;Acc:HGNC:28905]	12806	-0.795023322	-0.14741643
							ACKR3	atypical chemokine receptor 3 [Source:HGNC Symbol;Acc:HGNC:23692]	12853	-0.806620002	-0.14330246
							LAMB3	laminin subunit beta 3 [Source:HGNC Symbol;Acc:HGNC:6490]	12990	-0.853929996	-0.14535657
							EGR3	early growth response 3 [Source:HGNC Symbol;Acc:HGNC:3240]	12998	-0.856859982	-0.1379128
							BTG2	BTG anti-proliferation factor 2 [Source:HGNC Symbol;Acc:HGNC:1131]	13095	-0.905489981	-0.13655141
							ZFP36	ZFP36 ring finger protein [Source:HGNC Symbol;Acc:HGNC:12862]	13099	-0.906736672	-0.12835076
							TAP1	transporter 1, ATP binding cassette subfamily B member [Source:HGNC Symbol;Acc:HGNC:43]	13178	-0.94168669	-0.12533174
							DRAM1	DNA damage regulated autophagy modulator 1 [Source:HGNC Symbol;Acc:HGNC:25645]	13197	-0.949296653	-0.11783709
							CEBPB	CCAAT enhancer binding protein beta [Source:HGNC Symbol;Acc:HGNC:1834]	13297	-0.998733342	-0.115830004
							PFKFB3	6-phosphofructo-2-kinase/fructose-2,6-biphosphatase 3 [Source:HGNC Symbol;Acc:HGNC:8874]	13308	-1.006226659	-0.10721931
							ATF3	activating transcription factor 3 [Source:HGNC Symbol;Acc:HGNC:785]	13327	-1.022729993	-0.09904267
							GFPT2	glutamine-fructose-6-phosphate transaminase 2 [Source:HGNC Symbol;Acc:HGNC:4242]	13345	-1.040956616	-0.09062334
							CEBPD	CCAAT enhancer binding protein delta [Source:HGNC Symbol;Acc:HGNC:1835]	13379	-1.072893381	-0.08308208
							DUSP4	dual specificity phosphatase 4 [Source:HGNC Symbol;Acc:HGNC:3070]	13439	-1.132186651	-0.07689897
							FOS	Fos proto-oncogene, AP-1 transcription factor subunit [Source:HGNC Symbol;Acc:HGNC:3796]	13466	-1.162256718	-0.06801387
							PLAUR	plasminogen activator, urokinase receptor [Source:HGNC Symbol;Acc:HGNC:9053]	13472	-1.174236655	-0.057475775
							SERPINE1	serpin family E member 1 [Source:HGNC Symbol;Acc:HGNC:8583]	13513	-1.200383306	-0.04926442
							PDE4B	phosphodiesterase 4B [Source:HGNC Symbol;Acc:HGNC:8781]	13580	-1.308416724	-0.04195857
							MAFF	MAF bZIP transcription factor F [Source:HGNC Symbol;Acc:HGNC:6780]	13618	-1.382410049	-0.031836476
							NR4A3	nuclear receptor subfamily 4 group A member 3 [Source:HGNC Symbol;Acc:HGNC:7982]	13626	-1.399856687	-0.019349873
							TNFSF9	TNF superfamily member 9 [Source:HGNC Symbol;Acc:HGNC:11939]	13664	-1.49502337	-0.008181935
							AREG	amphiregulin [Source:HGNC Symbol;Acc:HGNC:651]	13742	-1.94825995	0.004258584
**CHOLESTEROL HOMEOSTASIS**	67	-0.31557238	0.02024292	0.17111285	0.499	1349	SREBF2	sterol regulatory element binding transcription factor 2 [Source:HGNC Symbol;Acc:HGNC:11290]	12453	-0.703379989	-0.29850334
							SQLE	squalene epoxidase [Source:HGNC Symbol;Acc:HGNC:11279]	12513	-0.71704334	-0.2853986
							CPEB2	cytoplasmic polyadenylation element binding protein 2 [Source:HGNC Symbol;Acc:HGNC:21745]	12610	-0.74047333	-0.27441934
							HMGCR	3-hydroxy-3-methylglutaryl-CoA reductase [Source:HGNC Symbol;Acc:HGNC:5006]	12632	-0.746763349	-0.25782657
							LSS	lanosterol synthase [Source:HGNC Symbol;Acc:HGNC:6708]	12797	-0.791806638	-0.2505528
							NIBAN1	niban apoptosis regulator 1 [Source:HGNC Symbol;Acc:HGNC:16784]	12939	-0.833473325	-0.24059324
							FADS2	fatty acid desaturase 2 [Source:HGNC Symbol;Acc:HGNC:3575]	13193	-0.947350025	-0.23602515
							CLU	clusterin [Source:HGNC Symbol;Acc:HGNC:2095]	13249	-0.971543312	-0.21645318
							ALCAM	activated leukocyte cell adhesion molecule [Source:HGNC Symbol;Acc:HGNC:400]	13253	-0.97554332	-0.19299792
							IDI1	isopentenyl-diphosphate delta isomerase 1 [Source:HGNC Symbol;Acc:HGNC:5387]	13268	-0.987006664	-0.17006542
							TM7SF2	transmembrane 7 superfamily member 2 [Source:HGNC Symbol;Acc:HGNC:11863]	13273	-0.988759995	-0.14636223
							ATF3	activating transcription factor 3 [Source:HGNC Symbol;Acc:HGNC:785]	13327	-1.022729993	-0.12540248
							PLAUR	plasminogen activator, urokinase receptor [Source:HGNC Symbol;Acc:HGNC:9053]	13472	-1.174236655	-0.10739198
							ABCA2	ATP binding cassette subfamily A member 2 [Source:HGNC Symbol;Acc:HGNC:32]	13521	-1.20796001	-0.081573166
							ACTG1	actin gamma 1 [Source:HGNC Symbol;Acc:HGNC:144]	13581	-1.309723377	-0.054085765
							FASN	fatty acid synthase [Source:HGNC Symbol;Acc:HGNC:3594]	13637	-1.428286672	-0.023429919
							ERRFI1	ERBB receptor feedback inhibitor 1 [Source:HGNC Symbol;Acc:HGNC:18185]	13649	-1.451570034	0.010994675
**FATTY ACID METABOLISM**											

Sex

Pathway Name	Pathway size	Enrichment Score	NOM.P.value	FDR.q.value	FWER.p.value	RANK.AT.MAX	Enriched Symbol	Title	Rank in Gene List	Rank Metric Score	Running Enrichment Score
**ANGIOGENESIS**											
**WNT BETA CATENIN SIGNALING**	36	-0.3551733	0.04930966	0.1550339	0.286	3533	WNT6	Wnt family member 6 [Source:HGNC Symbol;Acc:HGNC:12785]	9591	-0.396303326	-0.33141705
							HEY1	hes related family bHLH transcription factor with YRPW motif 1 [Source:HGNC Symbol;Acc:HGNC:4880]	9648	-0.400339991	-0.3116979
							MYC	MYC proto-oncogene, bHLH transcription factor [Source:HGNC Symbol;Acc:HGNC:7553]	9683	-0.402550012	-0.2901652
							NCSTN	nicastrin [Source:HGNC Symbol;Acc:HGNC:17091]	10050	-0.433456659	-0.2921485
							WNT5B	Wnt family member 5B [Source:HGNC Symbol;Acc:HGNC:16265]	10086	-0.436613321	-0.2686503
							JAG2	jagged canonical Notch ligand 2 [Source:HGNC Symbol;Acc:HGNC:6189]	10220	-0.448803335	-0.25190973
							FZD1	frizzled class receptor 1 [Source:HGNC Symbol;Acc:HGNC:4038]	10588	-0.488633335	-0.25066188
							NUMB	NUMB endocytic adaptor protein [Source:HGNC Symbol;Acc:HGNC:8060]	10742	-0.504410028	-0.23211624
							MAML1	mastermind like transcriptional coactivator 1 [Source:HGNC Symbol;Acc:HGNC:13632]	10794	-0.510256648	-0.2054261
							HDAC11	histone deacetylase 11 [Source:HGNC Symbol;Acc:HGNC:19086]	11058	-0.539680004	-0.19317149
							NCOR2	nuclear receptor corepressor 2 [Source:HGNC Symbol;Acc:HGNC:7673]	11275	-0.569563329	-0.17553417
							CCND2	cyclin D2 [Source:HGNC Symbol;Acc:HGNC:1583]	11512	-0.602416694	-0.15745571
							JAG1	jagged canonical Notch ligand 1 [Source:HGNC Symbol;Acc:HGNC:6188]	11561	-0.610096693	-0.124551475
							AXIN2	axin 2 [Source:HGNC Symbol;Acc:HGNC:904]	11825	-0.657100022	-0.10525816
							NOTCH4	notch receptor 4 [Source:HGNC Symbol;Acc:HGNC:7884]	11890	-0.670513332	-0.069954865
							AXIN1	axin 1 [Source:HGNC Symbol;Acc:HGNC:903]	12279	-0.76922667	-0.053491626
							NOTCH1	notch receptor 1 [Source:HGNC Symbol;Acc:HGNC:7881]	12297	-0.773273349	-0.008437067
							DLL1	delta like canonical Notch ligand 1 [Source:HGNC Symbol;Acc:HGNC:2908]	12922	-1.191106677	0.015282432
**BILE ACID METABOLISM**	93	-0.3475921	0	0.1343311	0.325	3135	SCP2	sterol carrier protein 2 [Source:HGNC Symbol;Acc:HGNC:10606]	9989	-0.427520007	-0.33878648
							CROT	carnitine O-octanoyltransferase [Source:HGNC Symbol;Acc:HGNC:2366]	10035	-0.43197	-0.3333428
							BMP6	bone morphogenetic protein 6 [Source:HGNC Symbol;Acc:HGNC:1073]	10036	-0.431973338	-0.3244455
							PEX6	peroxisomal biogenesis factor 6 [Source:HGNC Symbol;Acc:HGNC:8859]	10150	-0.442270011	-0.32400838
							HSD17B4	hydroxysteroid 17-beta dehydrogenase 4 [Source:HGNC Symbol;Acc:HGNC:5213]	10267	-0.453893334	-0.32356212
							RXRA	retinoid X receptor alpha [Source:HGNC Symbol;Acc:HGNC:10477]	10317	-0.459426671	-0.3178599
							ABCA3	ATP binding cassette subfamily A member 3 [Source:HGNC Symbol;Acc:HGNC:33]	10380	-0.465623319	-0.3130277
							GNPAT	glyceronephosphate O-acyltransferase [Source:HGNC Symbol;Acc:HGNC:4416]	10700	-0.499926656	-0.32721275
							PNPLA8	patatin like phospholipase domain containing 8 [Source:HGNC Symbol;Acc:HGNC:28900]	10713	-0.501820028	-0.31779775
							IDH1	isocitrate dehydrogenase (NADP(+)) 1 [Source:HGNC Symbol;Acc:HGNC:5382]	10906	-0.522189975	-0.32177746
							DHCR24	24-dehydrocholesterol reductase [Source:HGNC Symbol;Acc:HGNC:2859]	11014	-0.534493327	-0.31898037
							HACL1	2-hydroxyacyl-CoA lyase 1 [Source:HGNC Symbol;Acc:HGNC:17856]	11068	-0.541170001	-0.31190148
							ABCD3	ATP binding cassette subfamily D member 3 [Source:HGNC Symbol;Acc:HGNC:67]	11205	-0.558516681	-0.3108352
							NR3C2	nuclear receptor subfamily 3 group C member 2 [Source:HGNC Symbol;Acc:HGNC:7979]	11208	-0.558690012	-0.2994814
							LCK	LCK proto-oncogene, Src family tyrosine kinase [Source:HGNC Symbol;Acc:HGNC:6524]	11372	-0.582599998	-0.29999122
							ABCA1	ATP binding cassette subfamily A member 1 [Source:HGNC Symbol;Acc:HGNC:29]	11444	-0.59187001	-0.2932495
							ALDH9A1	aldehyde dehydrogenase 9 family member A1 [Source:HGNC Symbol;Acc:HGNC:412]	11459	-0.593946695	-0.28209046
							EFHC1	EF-hand domain containing 1 [Source:HGNC Symbol;Acc:HGNC:16406]	11653	-0.625696659	-0.284015
							DIO1	iodothyronine deiodinase 1 [Source:HGNC Symbol;Acc:HGNC:2883]	11711	-0.637266695	-0.2752638
							SLCO1A2	solute carrier organic anion transporter family member 1A2 [Source:HGNC Symbol;Acc:HGNC:10956]	11750	-0.643769979	-0.26492047
							NPC1	NPC intracellular cholesterol transporter 1 [Source:HGNC Symbol;Acc:HGNC:7897]	11868	-0.665570021	-0.26019105
							PFKM	phosphofructokinase, muscle [Source:HGNC Symbol;Acc:HGNC:8877]	11988	-0.693333328	-0.2550433
							HSD3B7	hydroxy-delta-5-steroid dehydrogenase, 3 beta- and steroid delta-isomerase 7 [Source:HGNC Symbol;Acc:HGNC:18324]	12131	-0.727653325	-0.2509538
							NEDD4	NEDD4 E3 ubiquitin protein ligase [Source:HGNC Symbol;Acc:HGNC:7727]	12252	-0.760969996	-0.24448968
							PEX11A	peroxisomal biogenesis factor 11 alpha [Source:HGNC Symbol;Acc:HGNC:8852]	12271	-0.767356694	-0.23006593
							PEX7	peroxisomal biogenesis factor 7 [Source:HGNC Symbol;Acc:HGNC:8860]	12323	-0.784080029	-0.21783035
							BCAR3	BCAR3 adaptor protein, NSP family member [Source:HGNC Symbol;Acc:HGNC:973]	12487	-0.839783311	-0.21304299
							RETSAT	retinol saturase [Source:HGNC Symbol;Acc:HGNC:25991]	12493	-0.843850017	-0.19604601
							ALDH1A1	aldehyde dehydrogenase 1 family member A1 [Source:HGNC Symbol;Acc:HGNC:402]	12577	-0.889036655	-0.1841045
							FADS2	fatty acid desaturase 2 [Source:HGNC Symbol;Acc:HGNC:3575]	12682	-0.947350025	-0.1725736
							CYP39A1	cytochrome P450 family 39 subfamily A member 1 [Source:HGNC Symbol;Acc:HGNC:17449]	12722	-0.968376696	-0.15562111
							IDI1	isopentenyl-diphosphate delta isomerase 1 [Source:HGNC Symbol;Acc:HGNC:5387]	12743	-0.987006664	-0.13682672
							EPHX2	epoxide hydrolase 2 [Source:HGNC Symbol;Acc:HGNC:3402]	12794	-1.019556642	-0.119664304
							ABCA5	ATP binding cassette subfamily A member 5 [Source:HGNC Symbol;Acc:HGNC:35]	12800	-1.02936995	-0.09884618
							GNMT	glycine N-methyltransferase [Source:HGNC Symbol;Acc:HGNC:4415]	12810	-1.039503336	-0.078126326
							ACSL1	acyl-CoA synthetase long chain family member 1 [Source:HGNC Symbol;Acc:HGNC:3569]	12905	-1.17513001	-0.06113639
							ABCA2	ATP binding cassette subfamily A member 2 [Source:HGNC Symbol;Acc:HGNC:32]	12937	-1.20796001	-0.03863526
							ISOC1	isochorismatase domain containing 1 [Source:HGNC Symbol;Acc:HGNC:24254]	12952	-1.238659978	-0.014197125
							SLC23A2	solute carrier family 23 member 2 [Source:HGNC Symbol;Acc:HGNC:10973]	12989	-1.318983316	0.010207014
**TNFA SIGNALING VIA NFKB**	180	-0.3975118	0	0.1742628	0.117	2280	IL12B	interleukin 12B [Source:HGNC Symbol;Acc:HGNC:5970]	10844	-0.515919983	-0.39279875
							RELA	RELA proto-oncogene, NF-kB subunit [Source:HGNC Symbol;Acc:HGNC:9955]	10865	-0.517629981	-0.3896153
							BTG1	BTG anti-proliferation factor 1 [Source:HGNC Symbol;Acc:HGNC:1130]	10873	-0.518503308	-0.3854195
							CCNL1	cyclin L1 [Source:HGNC Symbol;Acc:HGNC:20569]	10882	-0.519333363	-0.38129333
							KLF6	Kruppel like factor 6 [Source:HGNC Symbol;Acc:HGNC:2235]	10894	-0.520950019	-0.37738422
							KDM6B	lysine demethylase 6B [Source:HGNC Symbol;Acc:HGNC:29012]	10900	-0.521476686	-0.3730067
							CCND1	cyclin D1 [Source:HGNC Symbol;Acc:HGNC:1582]	10916	-0.523143351	-0.36938658
							SQSTM1	sequestosome 1 [Source:HGNC Symbol;Acc:HGNC:11280]	10975	-0.529603362	-0.3690297
							TLR2	toll like receptor 2 [Source:HGNC Symbol;Acc:HGNC:11848]	11012	-0.534143329	-0.3669316
							ZBTB10	zinc finger and BTB domain containing 10 [Source:HGNC Symbol;Acc:HGNC:30953]	11039	-0.537329972	-0.36403173
							ICAM1	intercellular adhesion molecule 1 [Source:HGNC Symbol;Acc:HGNC:5344]	11066	-0.541023314	-0.36109817
							PLK2	polo like kinase 2 [Source:HGNC Symbol;Acc:HGNC:19699]	11097	-0.544340014	-0.35844335
							BCL3	BCL3 transcription coactivator [Source:HGNC Symbol;Acc:HGNC:998]	11189	-0.556949973	-0.36038628
							JUN	Jun proto-oncogene, AP-1 transcription factor subunit [Source:HGNC Symbol;Acc:HGNC:6204]	11220	-0.560363352	-0.35758507
							FUT4	fucosyltransferase 4 [Source:HGNC Symbol;Acc:HGNC:4015]	11362	-0.58217001	-0.36316073
							BIRC3	baculoviral IAP repeat containing 3 [Source:HGNC Symbol;Acc:HGNC:591]	11376	-0.583530009	-0.35883445
							CD83	CD83 molecule [Source:HGNC Symbol;Acc:HGNC:1703]	11390	-0.585959971	-0.35448596
							CCL2	C-C motif chemokine ligand 2 [Source:HGNC Symbol;Acc:HGNC:10618]	11392	-0.586263359	-0.34920755
							PDLIM5	PDZ and LIM domain 5 [Source:HGNC Symbol;Acc:HGNC:17468]	11393	-0.586333334	-0.34385124
							F2RL1	F2R like trypsin receptor 1 [Source:HGNC Symbol;Acc:HGNC:3538]	11417	-0.588776648	-0.34024963
							ABCA1	ATP binding cassette subfamily A member 1 [Source:HGNC Symbol;Acc:HGNC:29]	11444	-0.59187001	-0.33685157
							F3	coagulation factor III, tissue factor [Source:HGNC Symbol;Acc:HGNC:3541]	11445	-0.591936648	-0.33144405
							CD69	CD69 molecule [Source:HGNC Symbol;Acc:HGNC:1694]	11484	-0.598246694	-0.32891488
							JAG1	jagged canonical Notch ligand 1 [Source:HGNC Symbol;Acc:HGNC:6188]	11561	-0.610096693	-0.32921338
							FOSB	FosB proto-oncogene, AP-1 transcription factor subunit [Source:HGNC Symbol;Acc:HGNC:3797]	11604	-0.617846668	-0.3268142
							TRIB1	tribbles pseudokinase 1 [Source:HGNC Symbol;Acc:HGNC:16891]	11605	-0.618019998	-0.32116842
							PLPP3	phospholipid phosphatase 3 [Source:HGNC Symbol;Acc:HGNC:9229]	11629	-0.621843338	-0.31726477
							HES1	hes family bHLH transcription factor 1 [Source:HGNC Symbol;Acc:HGNC:5192]	11651	-0.625566661	-0.31317255
							IRS2	insulin receptor substrate 2 [Source:HGNC Symbol;Acc:HGNC:6126]	11695	-0.634119987	-0.31070197
							TNFRSF9	TNF receptor superfamily member 9 [Source:HGNC Symbol;Acc:HGNC:11924]	11709	-0.637040019	-0.30588683
							GPR183	G protein-coupled receptor 183 [Source:HGNC Symbol;Acc:HGNC:3128]	11752	-0.644206643	-0.30324686
							LDLR	low density lipoprotein receptor [Source:HGNC Symbol;Acc:HGNC:6547]	11753	-0.644280016	-0.29736117
							MAP2K3	mitogen-activated protein kinase kinase 3 [Source:HGNC Symbol;Acc:HGNC:6843]	11770	-0.647173345	-0.29268527
							MCL1	MCL1 apoptosis regulator, BCL2 family member [Source:HGNC Symbol;Acc:HGNC:6943]	11771	-0.647279978	-0.28677222
							EDN1	endothelin 1 [Source:HGNC Symbol;Acc:HGNC:3176]	11807	-0.654429972	-0.283498
							IL7R	interleukin 7 receptor [Source:HGNC Symbol;Acc:HGNC:6024]	11863	-0.66470331	-0.28167516
							NR4A2	nuclear receptor subfamily 4 group A member 2 [Source:HGNC Symbol;Acc:HGNC:7981]	11994	-0.694113314	-0.28537828
							TNFAIP6	TNF alpha induced protein 6 [Source:HGNC Symbol;Acc:HGNC:11898]	12021	-0.700743318	-0.28098562
							B4GALT5	beta-1,4-galactosyltransferase 5 [Source:HGNC Symbol;Acc:HGNC:928]	12031	-0.702663362	-0.27526197
							RHOB	ras homolog family member B [Source:HGNC Symbol;Acc:HGNC:668]	12055	-0.708573341	-0.270566
							TNFAIP2	TNF alpha induced protein 2 [Source:HGNC Symbol;Acc:HGNC:11895]	12063	-0.711963356	-0.26460287
							DUSP2	dual specificity phosphatase 2 [Source:HGNC Symbol;Acc:HGNC:3068]	12067	-0.712339997	-0.25832725
							EGR1	early growth response 1 [Source:HGNC Symbol;Acc:HGNC:3238]	12100	-0.720200002	-0.25422043
							CCL5	C-C motif chemokine ligand 5 [Source:HGNC Symbol;Acc:HGNC:10632]	12196	-0.746720016	-0.2547388
							JUNB	JunB proto-oncogene, AP-1 transcription factor subunit [Source:HGNC Symbol;Acc:HGNC:6205]	12210	-0.750383317	-0.24888827
							DNAJB4	DnaJ heat shock protein family (Hsp40) member B4 [Source:HGNC Symbol;Acc:HGNC:14886]	12216	-0.753170013	-0.24239418
							ZC3H12A	zinc finger CCCH-type containing 12A [Source:HGNC Symbol;Acc:HGNC:26259]	12218	-0.753356695	-0.23558934
							CCN1	cellular communication network factor 1 [Source:HGNC Symbol;Acc:HGNC:2654]	12230	-0.756123304	-0.22953184
							SOCS3	suppressor of cytokine signaling 3 [Source:HGNC Symbol;Acc:HGNC:19391]	12301	-0.776639998	-0.22784536
							KLF9	Kruppel like factor 9 [Source:HGNC Symbol;Acc:HGNC:1123]	12325	-0.784556687	-0.22245526
							YRDC	yrdC N6-threonylcarbamoyltransferase domain containing [Source:HGNC Symbol;Acc:HGNC:28905]	12351	-0.795023322	-0.21712406
							NFE2L2	nuclear factor, erythroid 2 like 2 [Source:HGNC Symbol;Acc:HGNC:7782]	12389	-0.80509001	-0.21262805
							ACKR3	atypical chemokine receptor 3 [Source:HGNC Symbol;Acc:HGNC:23692]	12396	-0.806620002	-0.20572294
							LAMB3	laminin subunit beta 3 [Source:HGNC Symbol;Acc:HGNC:6490]	12514	-0.853929996	-0.2069617
							EGR3	early growth response 3 [Source:HGNC Symbol;Acc:HGNC:3240]	12520	-0.856859982	-0.19952038
							BTG2	BTG anti-proliferation factor 2 [Source:HGNC Symbol;Acc:HGNC:1131]	12606	-0.905489981	-0.19781576
							ZFP36	ZFP36 ring finger protein [Source:HGNC Symbol;Acc:HGNC:12862]	12610	-0.906736672	-0.18976428
							ICOSLG	inducible T cell costimulator ligand [Source:HGNC Symbol;Acc:HGNC:17087]	12617	-0.911503315	-0.18190102
							SPSB1	splA/ryanodine receptor domain and SOCS box containing 1 [Source:HGNC Symbol;Acc:HGNC:30628]	12727	-0.971893311	-0.18144406
							CEBPB	CCAAT enhancer binding protein beta [Source:HGNC Symbol;Acc:HGNC:1834]	12768	-0.998733342	-0.17541087
							PFKFB3	6-phosphofructo-2-kinase/fructose-2,6-biphosphatase 3 [Source:HGNC Symbol;Acc:HGNC:8874]	12776	-1.006226659	-0.16675955
							ATF3	activating transcription factor 3 [Source:HGNC Symbol;Acc:HGNC:785]	12796	-1.022729993	-0.15888463
							GFPT2	glutamine-fructose-6-phosphate transaminase 2 [Source:HGNC Symbol;Acc:HGNC:4242]	12814	-1.040956616	-0.15068868
							CEBPD	CCAAT enhancer binding protein delta [Source:HGNC Symbol;Acc:HGNC:1835]	12838	-1.072893381	-0.14266455
							DUSP4	dual specificity phosphatase 4 [Source:HGNC Symbol;Acc:HGNC:3070]	12881	-1.132186651	-0.13556673
							FOS	Fos proto-oncogene, AP-1 transcription factor subunit [Source:HGNC Symbol;Acc:HGNC:3796]	12902	-1.162256718	-0.12649445
							KLF2	Kruppel like factor 2 [Source:HGNC Symbol;Acc:HGNC:6347]	12953	-1.24162662	-0.11901497
							PDE4B	phosphodiesterase 4B [Source:HGNC Symbol;Acc:HGNC:8781]	12984	-1.308416724	-0.1093801
							MAFF	MAF bZIP transcription factor F [Source:HGNC Symbol;Acc:HGNC:6780]	13011	-1.382410049	-0.09876025
							NR4A3	nuclear receptor subfamily 4 group A member 3 [Source:HGNC Symbol;Acc:HGNC:7982]	13016	-1.399856687	-0.08628125
							KLF4	Kruppel like factor 4 [Source:HGNC Symbol;Acc:HGNC:6348]	13074	-1.718729973	-0.07498413
							DUSP1	dual specificity phosphatase 1 [Source:HGNC Symbol;Acc:HGNC:3064]	13089	-1.87762332	-0.05891323
							AREG	amphiregulin [Source:HGNC Symbol;Acc:HGNC:651]	13094	-1.94825995	-0.041424423
							NR4A1	nuclear receptor subfamily 4 group A member 1 [Source:HGNC Symbol;Acc:HGNC:7980]	13105	-2.348623276	-0.02074177
							PER1	period circadian regulator 1 [Source:HGNC Symbol;Acc:HGNC:8845]	13107	-2.40588665	0.001159356
**CHOLESTEROL HOMEOSTASIS**	59	-0.3259802	0.03059273	0.1685724	0.457	1563	JAG1	jagged canonical Notch ligand 1 [Source:HGNC Symbol;Acc:HGNC:6188]	11561	-0.610096693	-0.30834562
							LDLR	low density lipoprotein receptor [Source:HGNC Symbol;Acc:HGNC:6547]	11753	-0.644280016	-0.30434328
							SREBF2	sterol regulatory element binding transcription factor 2 [Source:HGNC Symbol;Acc:HGNC:11290]	12034	-0.703379989	-0.30544534
							CPEB2	cytoplasmic polyadenylation element binding protein 2 [Source:HGNC Symbol;Acc:HGNC:21745]	12176	-0.74047333	-0.29483527
							HMGCR	3-hydroxy-3-methylglutaryl-CoA reductase [Source:HGNC Symbol;Acc:HGNC:5006]	12197	-0.746763349	-0.27478132
							LSS	lanosterol synthase [Source:HGNC Symbol;Acc:HGNC:6708]	12344	-0.791806638	-0.26307023
							NIBAN1	niban apoptosis regulator 1 [Source:HGNC Symbol;Acc:HGNC:16784]	12469	-0.833473325	-0.24847077
							FADS2	fatty acid desaturase 2 [Source:HGNC Symbol;Acc:HGNC:3575]	12682	-0.947350025	-0.23731582
							CLU	clusterin [Source:HGNC Symbol;Acc:HGNC:2095]	12725	-0.971543312	-0.2124487
							ALCAM	activated leukocyte cell adhesion molecule [Source:HGNC Symbol;Acc:HGNC:400]	12730	-0.97554332	-0.18455721
							IDI1	isopentenyl-diphosphate delta isomerase 1 [Source:HGNC Symbol;Acc:HGNC:5387]	12743	-0.987006664	-0.15694676
							ATF3	activating transcription factor 3 [Source:HGNC Symbol;Acc:HGNC:785]	12796	-1.022729993	-0.13136558
							ABCA2	ATP binding cassette subfamily A member 2 [Source:HGNC Symbol;Acc:HGNC:32]	12937	-1.20796001	-0.10716648
							ACTG1	actin gamma 1 [Source:HGNC Symbol;Acc:HGNC:144]	12985	-1.309723377	-0.07290714
							FASN	fatty acid synthase [Source:HGNC Symbol;Acc:HGNC:3594]	13024	-1.428286672	-0.03453187
							ERRFI1	ERBB receptor feedback inhibitor 1 [Source:HGNC Symbol;Acc:HGNC:18185]	13030	-1.451570034	0.007042426
**FATTY ACID METABOLISM**	124	-0.2600672	0.0037244	0.1694138	0.897	3356	CRAT	carnitine O-acetyltransferase [Source:HGNC Symbol;Acc:HGNC:2342]	9768	-0.409333318	-0.25400966
							ACO2	aconitase 2 [Source:HGNC Symbol;Acc:HGNC:118]	9826	-0.41421333	-0.2522649
							ELOVL5	ELOVL fatty acid elongase 5 [Source:HGNC Symbol;Acc:HGNC:21308]	9841	-0.414933324	-0.24720149
							NBN	nibrin [Source:HGNC Symbol;Acc:HGNC:7652]	9896	-0.420556664	-0.24513204
							HMGCS2	3-hydroxy-3-methylglutaryl-CoA synthase 2 [Source:HGNC Symbol;Acc:HGNC:5008]	10020	-0.430126667	-0.24822907
							HSDL2	hydroxysteroid dehydrogenase like 2 [Source:HGNC Symbol;Acc:HGNC:18572]	10060	-0.43489334	-0.24479353
							YWHAH	tyrosine 3-monooxygenase/tryptophan 5-monooxygenase activation protein eta [Source:HGNC Symbol;Acc:HGNC:12853]	10101	-0.438210011	-0.24138583
							PPARA	peroxisome proliferator activated receptor alpha [Source:HGNC Symbol;Acc:HGNC:9232]	10133	-0.44108668	-0.23724322
							DECR1	2,4-dienoyl-CoA reductase 1 [Source:HGNC Symbol;Acc:HGNC:2753]	10140	-0.441503346	-0.23117119
							IDH3B	isocitrate dehydrogenase (NAD(+)) 3 non-catalytic subunit beta [Source:HGNC Symbol;Acc:HGNC:5385]	10227	-0.449386656	-0.23113684
							GRHPR	glyoxylate and hydroxypyruvate reductase [Source:HGNC Symbol;Acc:HGNC:4570]	10243	-0.450899988	-0.22561812
							CPT1A	carnitine palmitoyltransferase 1A [Source:HGNC Symbol;Acc:HGNC:2328]	10250	-0.451773345	-0.21939413
							HSD17B4	hydroxysteroid 17-beta dehydrogenase 4 [Source:HGNC Symbol;Acc:HGNC:5213]	10267	-0.453893334	-0.21390805
							GPD2	glycerol-3-phosphate dehydrogenase 2 [Source:HGNC Symbol;Acc:HGNC:4456]	10361	-0.463666677	-0.21420087
							ETFDH	electron transfer flavoprotein dehydrogenase [Source:HGNC Symbol;Acc:HGNC:3483]	10366	-0.46419999	-0.20763911

Interaction

Pathway Name	Pathway size	Enrichment Score	NOM.P.value	FDR.q.value	FWER.p.value	RANK.AT.MAX	Enriched Symbol	Title	Rank in Gene List	Rank Metric Score	Running Enrichment Score
**ANGIOGENESIS**	26	0.37208638	0.05102041	0.0697961	0.144	1691	MSX1	msh homeobox 1 [Source:HGNC Symbol;Acc:HGNC:7391]	81	1.389016628	0.07748481
							ITGAV	integrin subunit alpha V [Source:HGNC Symbol;Acc:HGNC:6150]	172	1.149060011	0.1398167
							CXCL6	C-X-C motif chemokine ligand 6 [Source:HGNC Symbol;Acc:HGNC:10643]	191	1.107283354	0.20516467
							PF4	platelet factor 4 [Source:HGNC Symbol;Acc:HGNC:8861]	247	1.049976707	0.26421526
							VTN	vitronectin [Source:HGNC Symbol;Acc:HGNC:12724]	360	0.959326684	0.31342182
							PDGFA	platelet derived growth factor subunit A [Source:HGNC Symbol;Acc:HGNC:8799]	830	0.757513344	0.3230276
							COL5A2	collagen type V alpha 2 chain [Source:HGNC Symbol;Acc:HGNC:2210]	1142	0.68053335	0.3401377
							S100A4	S100 calcium binding protein A4 [Source:HGNC Symbol;Acc:HGNC:10494]	1342	0.643476665	0.36362264
							NRP1	neuropilin 1 [Source:HGNC Symbol;Acc:HGNC:8004]	1691	0.584249973	0.37208638
**WNT BETA CATENIN SIGNALING**	36	-0.35662764	0.02443609	0.1998441	0.239	3491	WNT6	Wnt family member 6 [Source:HGNC Symbol;Acc:HGNC:12785]	9547	-0.396303326	-0.3328714
							HEY1	hes related family bHLH transcription factor with YRPW motif 1 [Source:HGNC Symbol;Acc:HGNC:4880]	9604	-0.400339991	-0.31318057
							MYC	MYC proto-oncogene, bHLH transcription factor [Source:HGNC Symbol;Acc:HGNC:7553]	9639	-0.402550012	-0.29166505
							NCSTN	nicastrin [Source:HGNC Symbol;Acc:HGNC:17091]	10005	-0.433456659	-0.29375643
							WNT5B	Wnt family member 5B [Source:HGNC Symbol;Acc:HGNC:16265]	10041	-0.436613321	-0.27027592
							JAG2	jagged canonical Notch ligand 2 [Source:HGNC Symbol;Acc:HGNC:6189]	10175	-0.448803335	-0.25360256
							FZD1	frizzled class receptor 1 [Source:HGNC Symbol;Acc:HGNC:4038]	10546	-0.488633335	-0.252771
							NUMB	NUMB endocytic adaptor protein [Source:HGNC Symbol;Acc:HGNC:8060]	10699	-0.504410028	-0.23422574
							MAML1	mastermind like transcriptional coactivator 1 [Source:HGNC Symbol;Acc:HGNC:13632]	10750	-0.510256648	-0.20748447
							HDAC11	histone deacetylase 11 [Source:HGNC Symbol;Acc:HGNC:19086]	11012	-0.539680004	-0.19520895
							NCOR2	nuclear receptor corepressor 2 [Source:HGNC Symbol;Acc:HGNC:7673]	11228	-0.569563329	-0.1776039
							CCND2	cyclin D2 [Source:HGNC Symbol;Acc:HGNC:1583]	11465	-0.602416694	-0.15964472
							JAG1	jagged canonical Notch ligand 1 [Source:HGNC Symbol;Acc:HGNC:6188]	11514	-0.610096693	-0.12676474
							AXIN2	axin 2 [Source:HGNC Symbol;Acc:HGNC:904]	11774	-0.657100022	-0.107296705
							NOTCH4	notch receptor 4 [Source:HGNC Symbol;Acc:HGNC:7884]	11837	-0.670513332	-0.07187192
							AXIN1	axin 1 [Source:HGNC Symbol;Acc:HGNC:903]	12223	-0.76922667	-0.05537405
							NOTCH1	notch receptor 1 [Source:HGNC Symbol;Acc:HGNC:7881]	12240	-0.773273349	-0.010251165
							DLL1	delta like canonical Notch ligand 1 [Source:HGNC Symbol;Acc:HGNC:2908]	12856	-1.191106677	0.013845185
**BILE ACID METABOLISM**	92	-0.33753663	0	0.2070451	0.35	3094	SCP2	sterol carrier protein 2 [Source:HGNC Symbol;Acc:HGNC:10606]	9944	-0.427520007	-0.32848513
							CROT	carnitine O-octanoyltransferase [Source:HGNC Symbol;Acc:HGNC:2366]	9990	-0.43197	-0.32281566
							BMP6	bone morphogenetic protein 6 [Source:HGNC Symbol;Acc:HGNC:1073]	9991	-0.431973338	-0.31366986
							PEX6	peroxisomal biogenesis factor 6 [Source:HGNC Symbol;Acc:HGNC:8859]	10105	-0.442270011	-0.3130353
							HSD17B4	hydroxysteroid 17-beta dehydrogenase 4 [Source:HGNC Symbol;Acc:HGNC:5213]	10222	-0.453893334	-0.31238642
							RXRA	retinoid X receptor alpha [Source:HGNC Symbol;Acc:HGNC:10477]	10272	-0.459426671	-0.30644464
							ABCA3	ATP binding cassette subfamily A member 3 [Source:HGNC Symbol;Acc:HGNC:33]	10335	-0.465623319	-0.30137593
							GNPAT	glyceronephosphate O-acyltransferase [Source:HGNC Symbol;Acc:HGNC:4416]	10657	-0.499926656	-0.31558865
							PNPLA8	patatin like phospholipase domain containing 8 [Source:HGNC Symbol;Acc:HGNC:28900]	10670	-0.501820028	-0.30589107
							IDH1	isocitrate dehydrogenase (NADP(+)) 1 [Source:HGNC Symbol;Acc:HGNC:5382]	10862	-0.522189975	-0.30958992
							DHCR24	24-dehydrocholesterol reductase [Source:HGNC Symbol;Acc:HGNC:2859]	10968	-0.534493327	-0.30638483
							HACL1	2-hydroxyacyl-CoA lyase 1 [Source:HGNC Symbol;Acc:HGNC:17856]	11022	-0.541170001	-0.29902136
							ABCD3	ATP binding cassette subfamily D member 3 [Source:HGNC Symbol;Acc:HGNC:67]	11158	-0.558516681	-0.29762512
							NR3C2	nuclear receptor subfamily 3 group C member 2 [Source:HGNC Symbol;Acc:HGNC:7979]	11161	-0.558690012	-0.285951
							LCK	LCK proto-oncogene, Src family tyrosine kinase [Source:HGNC Symbol;Acc:HGNC:6524]	11325	-0.582599998	-0.28620785
							ABCA1	ATP binding cassette subfamily A member 1 [Source:HGNC Symbol;Acc:HGNC:29]	11395	-0.59187001	-0.27900696
							ALDH9A1	aldehyde dehydrogenase 9 family member A1 [Source:HGNC Symbol;Acc:HGNC:412]	11410	-0.593946695	-0.26751336
							EFHC1	EF-hand domain containing 1 [Source:HGNC Symbol;Acc:HGNC:16406]	11607	-0.625696659	-0.26940703
							DIO1	iodothyronine deiodinase 1 [Source:HGNC Symbol;Acc:HGNC:2883]	11665	-0.637266695	-0.26031798
							SLCO1A2	solute carrier organic anion transporter family member 1A2 [Source:HGNC Symbol;Acc:HGNC:10956]	11703	-0.643769979	-0.24954627
							NPC1	NPC intracellular cholesterol transporter 1 [Source:HGNC Symbol;Acc:HGNC:7897]	11816	-0.665570021	-0.24410674
							PFKM	phosphofructokinase, muscle [Source:HGNC Symbol;Acc:HGNC:8877]	11933	-0.693333328	-0.2383884
							HSD3B7	hydroxy-delta-5-steroid dehydrogenase, 3 beta- and steroid delta-isomerase 7 [Source:HGNC Symbol;Acc:HGNC:18324]	12077	-0.727653325	-0.23402919
							NEDD4	NEDD4 E3 ubiquitin protein ligase [Source:HGNC Symbol;Acc:HGNC:7727]	12198	-0.760969996	-0.22718784
							PEX11A	peroxisomal biogenesis factor 11 alpha [Source:HGNC Symbol;Acc:HGNC:8852]	12216	-0.767356694	-0.21225454
							PEX7	peroxisomal biogenesis factor 7 [Source:HGNC Symbol;Acc:HGNC:8860]	12268	-0.784080029	-0.19959366
							BCAR3	BCAR3 adaptor protein, NSP family member [Source:HGNC Symbol;Acc:HGNC:973]	12430	-0.839783311	-0.19425093
							RETSAT	retinol saturase [Source:HGNC Symbol;Acc:HGNC:25991]	12436	-0.843850017	-0.17677109
							ALDH1A1	aldehyde dehydrogenase 1 family member A1 [Source:HGNC Symbol;Acc:HGNC:402]	12518	-0.889036655	-0.16420555
							FADS2	fatty acid desaturase 2 [Source:HGNC Symbol;Acc:HGNC:3575]	12622	-0.947350025	-0.1521049
							CYP39A1	cytochrome P450 family 39 subfamily A member 1 [Source:HGNC Symbol;Acc:HGNC:17449]	12661	-0.968376696	-0.13453783
							IDI1	isopentenyl-diphosphate delta isomerase 1 [Source:HGNC Symbol;Acc:HGNC:5387]	12680	-0.987006664	-0.115031324
							EPHX2	epoxide hydrolase 2 [Source:HGNC Symbol;Acc:HGNC:3402]	12729	-1.019556642	-0.09715316
							ABCA5	ATP binding cassette subfamily A member 5 [Source:HGNC Symbol;Acc:HGNC:35]	12735	-1.02936995	-0.075745486
							GNMT	glycine N-methyltransferase [Source:HGNC Symbol;Acc:HGNC:4415]	12744	-1.039503336	-0.05435501
							ACSL1	acyl-CoA synthetase long chain family member 1 [Source:HGNC Symbol;Acc:HGNC:3569]	12838	-1.17513001	-0.036659278
							ABCA2	ATP binding cassette subfamily A member 2 [Source:HGNC Symbol;Acc:HGNC:32]	12872	-1.20796001	-0.013633473
							ISOC1	isochorismatase domain containing 1 [Source:HGNC Symbol;Acc:HGNC:24254]	12884	-1.238659978	0.011741814
**TNFA SIGNALING VIA NFKB**	166	-0.3361857	0	0.1587702	0.358	2238	IL12B	interleukin 12B [Source:HGNC Symbol;Acc:HGNC:5970]	10800	-0.515919983	-0.33052075
							RELA	RELA proto-oncogene, NF-kB subunit [Source:HGNC Symbol;Acc:HGNC:9955]	10821	-0.517629981	-0.32639092
							BTG1	BTG anti-proliferation factor 1 [Source:HGNC Symbol;Acc:HGNC:1130]	10829	-0.518503308	-0.32124147
							CCNL1	cyclin L1 [Source:HGNC Symbol;Acc:HGNC:20569]	10838	-0.519333363	-0.3161606
							KLF6	Kruppel like factor 6 [Source:HGNC Symbol;Acc:HGNC:2235]	10850	-0.520950019	-0.31129506
							KDM6B	lysine demethylase 6B [Source:HGNC Symbol;Acc:HGNC:29012]	10856	-0.521476686	-0.3059576
							CCND1	cyclin D1 [Source:HGNC Symbol;Acc:HGNC:1582]	10872	-0.523143351	-0.30137873
							SQSTM1	sequestosome 1 [Source:HGNC Symbol;Acc:HGNC:11280]	10929	-0.529603362	-0.29991442
							TLR2	toll like receptor 2 [Source:HGNC Symbol;Acc:HGNC:11848]	10966	-0.534143329	-0.29684636
							ZBTB10	zinc finger and BTB domain containing 10 [Source:HGNC Symbol;Acc:HGNC:30953]	10993	-0.537329972	-0.29296637
							ICAM1	intercellular adhesion molecule 1 [Source:HGNC Symbol;Acc:HGNC:5344]	11020	-0.541023314	-0.28904584
							PLK2	polo like kinase 2 [Source:HGNC Symbol;Acc:HGNC:19699]	11051	-0.544340014	-0.28539965
							BCL3	BCL3 transcription coactivator [Source:HGNC Symbol;Acc:HGNC:998]	11143	-0.556949973	-0.28635436
							JUN	Jun proto-oncogene, AP-1 transcription factor subunit [Source:HGNC Symbol;Acc:HGNC:6204]	11173	-0.560363352	-0.28245455
							FUT4	fucosyltransferase 4 [Source:HGNC Symbol;Acc:HGNC:4015]	11315	-0.58217001	-0.28701702
							BIRC3	baculoviral IAP repeat containing 3 [Source:HGNC Symbol;Acc:HGNC:591]	11329	-0.583530009	-0.28161973
							CD83	CD83 molecule [Source:HGNC Symbol;Acc:HGNC:1703]	11343	-0.585959971	-0.27619573
							CCL2	C-C motif chemokine ligand 2 [Source:HGNC Symbol;Acc:HGNC:10618]	11345	-0.586263359	-0.2698361
							F2RL1	F2R like trypsin receptor 1 [Source:HGNC Symbol;Acc:HGNC:3538]	11369	-0.588776648	-0.26515815
							ABCA1	ATP binding cassette subfamily A member 1 [Source:HGNC Symbol;Acc:HGNC:29]	11395	-0.59187001	-0.2606016
							F3	coagulation factor III, tissue factor [Source:HGNC Symbol;Acc:HGNC:3541]	11396	-0.591936648	-0.254102
							CD69	CD69 molecule [Source:HGNC Symbol;Acc:HGNC:1694]	11436	-0.598246694	-0.25056314
							JAG1	jagged canonical Notch ligand 1 [Source:HGNC Symbol;Acc:HGNC:6188]	11514	-0.610096693	-0.24984655
							FOSB	FosB proto-oncogene, AP-1 transcription factor subunit [Source:HGNC Symbol;Acc:HGNC:3797]	11558	-0.617846668	-0.24640328
							PLPP3	phospholipid phosphatase 3 [Source:HGNC Symbol;Acc:HGNC:9229]	11582	-0.621843338	-0.24136223
							HES1	hes family bHLH transcription factor 1 [Source:HGNC Symbol;Acc:HGNC:5192]	11605	-0.625566661	-0.23620261
							IRS2	insulin receptor substrate 2 [Source:HGNC Symbol;Acc:HGNC:6126]	11648	-0.634119987	-0.23250295
							TNFRSF9	TNF receptor superfamily member 9 [Source:HGNC Symbol;Acc:HGNC:11924]	11663	-0.637040019	-0.22659579
							GPR183	G protein-coupled receptor 183 [Source:HGNC Symbol;Acc:HGNC:3128]	11705	-0.644206643	-0.22270769
							LDLR	low density lipoprotein receptor [Source:HGNC Symbol;Acc:HGNC:6547]	11706	-0.644280016	-0.21563332
							MAP2K3	mitogen-activated protein kinase kinase 3 [Source:HGNC Symbol;Acc:HGNC:6843]	11722	-0.647173345	-0.20969258
							MCL1	MCL1 apoptosis regulator, BCL2 family member [Source:HGNC Symbol;Acc:HGNC:6943]	11723	-0.647279978	-0.20258528
							EDN1	endothelin 1 [Source:HGNC Symbol;Acc:HGNC:3176]	11757	-0.654429972	-0.19796337
							IL7R	interleukin 7 receptor [Source:HGNC Symbol;Acc:HGNC:6024]	11811	-0.66470331	-0.19478253
							NR4A2	nuclear receptor subfamily 4 group A member 2 [Source:HGNC Symbol;Acc:HGNC:7981]	11939	-0.694113314	-0.19702812
							TNFAIP6	TNF alpha induced protein 6 [Source:HGNC Symbol;Acc:HGNC:11898]	11965	-0.700743318	-0.19127612
							B4GALT5	beta-1,4-galactosyltransferase 5 [Source:HGNC Symbol;Acc:HGNC:928]	11975	-0.702663362	-0.18425994
							RHOB	ras homolog family member B [Source:HGNC Symbol;Acc:HGNC:668]	12000	-0.708573341	-0.17834426
							TNFAIP2	TNF alpha induced protein 2 [Source:HGNC Symbol;Acc:HGNC:11895]	12009	-0.711963356	-0.17114827
							EGR1	early growth response 1 [Source:HGNC Symbol;Acc:HGNC:3238]	12046	-0.720200002	-0.16603726
							SMAD3	SMAD family member 3 [Source:HGNC Symbol;Acc:HGNC:6769]	12133	-0.744603336	-0.164543
							CCL5	C-C motif chemokine ligand 5 [Source:HGNC Symbol;Acc:HGNC:10632]	12143	-0.746720016	-0.15704307
							JUNB	JunB proto-oncogene, AP-1 transcription factor subunit [Source:HGNC Symbol;Acc:HGNC:6205]	12158	-0.750383317	-0.14989136
							ZC3H12A	zinc finger CCCH-type containing 12A [Source:HGNC Symbol;Acc:HGNC:26259]	12164	-0.753356695	-0.14200778
							CCN1	cellular communication network factor 1 [Source:HGNC Symbol;Acc:HGNC:2654]	12176	-0.756123304	-0.13455997
							SOCS3	suppressor of cytokine signaling 3 [Source:HGNC Symbol;Acc:HGNC:19391]	12246	-0.776639998	-0.13139315
							KLF9	Kruppel like factor 9 [Source:HGNC Symbol;Acc:HGNC:1123]	12270	-0.784556687	-0.12456547
							YRDC	yrdC N6-threonylcarbamoyltransferase domain containing [Source:HGNC Symbol;Acc:HGNC:28905]	12295	-0.795023322	-0.117700554
							ACKR3	atypical chemokine receptor 3 [Source:HGNC Symbol;Acc:HGNC:23692]	12338	-0.806620002	-0.11210679
							LAMB3	laminin subunit beta 3 [Source:HGNC Symbol;Acc:HGNC:6490]	12459	-0.853929996	-0.1120537
							EGR3	early growth response 3 [Source:HGNC Symbol;Acc:HGNC:3240]	12463	-0.856859982	-0.10287823
							BTG2	BTG anti-proliferation factor 2 [Source:HGNC Symbol;Acc:HGNC:1131]	12547	-0.905489981	-0.09938431
							ZFP36	ZFP36 ring finger protein [Source:HGNC Symbol;Acc:HGNC:12862]	12551	-0.906736672	-0.08966117
							CEBPB	CCAAT enhancer binding protein beta [Source:HGNC Symbol;Acc:HGNC:1834]	12705	-0.998733342	-0.090582006
							PFKFB3	6-phosphofructo-2-kinase/fructose-2,6-biphosphatase 3 [Source:HGNC Symbol;Acc:HGNC:8874]	12712	-1.006226659	-0.07999953
							ATF3	activating transcription factor 3 [Source:HGNC Symbol;Acc:HGNC:785]	12731	-1.022729993	-0.07016817
							GFPT2	glutamine-fructose-6-phosphate transaminase 2 [Source:HGNC Symbol;Acc:HGNC:4242]	12748	-1.040956616	-0.05998129
							CEBPD	CCAAT enhancer binding protein delta [Source:HGNC Symbol;Acc:HGNC:1835]	12771	-1.072893381	-0.0499099
							DUSP4	dual specificity phosphatase 4 [Source:HGNC Symbol;Acc:HGNC:3070]	12813	-1.132186651	-0.04066364
							FOS	Fos proto-oncogene, AP-1 transcription factor subunit [Source:HGNC Symbol;Acc:HGNC:3796]	12834	-1.162256718	-0.029455628
							PDE4B	phosphodiesterase 4B [Source:HGNC Symbol;Acc:HGNC:8781]	12911	-1.308416724	-0.020993605
							MAFF	MAF bZIP transcription factor F [Source:HGNC Symbol;Acc:HGNC:6780]	12936	-1.382410049	-0.007679027
							NR4A3	nuclear receptor subfamily 4 group A member 3 [Source:HGNC Symbol;Acc:HGNC:7982]	12941	-1.399856687	0.007381002
**CHOLESTEROL HOMEOSTASIS**	60	-0.3228966	0.01532567	0.1713991	0.446	1524	JAG1	jagged canonical Notch ligand 1 [Source:HGNC Symbol;Acc:HGNC:6188]	11514	-0.610096693	-0.3054917
							LDLR	low density lipoprotein receptor [Source:HGNC Symbol;Acc:HGNC:6547]	11706	-0.644280016	-0.3018299
							SREBF2	sterol regulatory element binding transcription factor 2 [Source:HGNC Symbol;Acc:HGNC:11290]	11978	-0.703379989	-0.3026469
							CPEB2	cytoplasmic polyadenylation element binding protein 2 [Source:HGNC Symbol;Acc:HGNC:21745]	12120	-0.74047333	-0.29238796
							HMGCR	3-hydroxy-3-methylglutaryl-CoA reductase [Source:HGNC Symbol;Acc:HGNC:5006]	12144	-0.746763349	-0.27285656
							LSS	lanosterol synthase [Source:HGNC Symbol;Acc:HGNC:6708]	12288	-0.791806638	-0.26128727
							NIBAN1	niban apoptosis regulator 1 [Source:HGNC Symbol;Acc:HGNC:16784]	12412	-0.833473325	-0.24698815
							FADS2	fatty acid desaturase 2 [Source:HGNC Symbol;Acc:HGNC:3575]	12622	-0.947350025	-0.23606744
							ALCAM	activated leukocyte cell adhesion molecule [Source:HGNC Symbol;Acc:HGNC:400]	12667	-0.97554332	-0.21162762
							IDI1	isopentenyl-diphosphate delta isomerase 1 [Source:HGNC Symbol;Acc:HGNC:5387]	12680	-0.987006664	-0.18439488
							TM7SF2	transmembrane 7 superfamily member 2 [Source:HGNC Symbol;Acc:HGNC:11863]	12685	-0.988759995	-0.15649565
							ATF3	activating transcription factor 3 [Source:HGNC Symbol;Acc:HGNC:785]	12731	-1.022729993	-0.13078673
							ABCA2	ATP binding cassette subfamily A member 2 [Source:HGNC Symbol;Acc:HGNC:32]	12872	-1.20796001	-0.1071142
							ACTG1	actin gamma 1 [Source:HGNC Symbol;Acc:HGNC:144]	12912	-1.309723377	-0.07275556
							FASN	fatty acid synthase [Source:HGNC Symbol;Acc:HGNC:3594]	12952	-1.428286672	-0.03501452
							ERRFI1	ERBB receptor feedback inhibitor 1 [Source:HGNC Symbol;Acc:HGNC:18185]	12958	-1.451570034	0.006010766
**FATTY ACID METABOLISM**	123	-0.31497097	0.00197239	0.1641912	0.493	3314	CRAT	carnitine O-acetyltransferase [Source:HGNC Symbol;Acc:HGNC:2342]	9724	-0.409333318	-0.30876094
							ACO2	aconitase 2 [Source:HGNC Symbol;Acc:HGNC:118]	9782	-0.41421333	-0.30689067
							ELOVL5	ELOVL fatty acid elongase 5 [Source:HGNC Symbol;Acc:HGNC:21308]	9797	-0.414933324	-0.30167976
							NBN	nibrin [Source:HGNC Symbol;Acc:HGNC:7652]	9852	-0.420556664	-0.29948097
							HMGCS2	3-hydroxy-3-methylglutaryl-CoA synthase 2 [Source:HGNC Symbol;Acc:HGNC:5008]	9975	-0.430126667	-0.3024026
							HSDL2	hydroxysteroid dehydrogenase like 2 [Source:HGNC Symbol;Acc:HGNC:18572]	10015	-0.43489334	-0.29882473
							YWHAH	tyrosine 3-monooxygenase/tryptophan 5-monooxygenase activation protein eta [Source:HGNC Symbol;Acc:HGNC:12853]	10056	-0.438210011	-0.29527402
							PPARA	peroxisome proliferator activated receptor alpha [Source:HGNC Symbol;Acc:HGNC:9232]	10088	-0.44108668	-0.29098272
							DECR1	2,4-dienoyl-CoA reductase 1 [Source:HGNC Symbol;Acc:HGNC:2753]	10095	-0.441503346	-0.28474924
							IDH3B	isocitrate dehydrogenase (NAD(+)) 3 non-catalytic subunit beta [Source:HGNC Symbol;Acc:HGNC:5385]	10182	-0.449386656	-0.284591
							GRHPR	glyoxylate and hydroxypyruvate reductase [Source:HGNC Symbol;Acc:HGNC:4570]	10198	-0.450899988	-0.27891186
							CPT1A	carnitine palmitoyltransferase 1A [Source:HGNC Symbol;Acc:HGNC:2328]	10205	-0.451773345	-0.27252257
							HSD17B4	hydroxysteroid 17-beta dehydrogenase 4 [Source:HGNC Symbol;Acc:HGNC:5213]	10222	-0.453893334	-0.26687548
							GPD2	glycerol-3-phosphate dehydrogenase 2 [Source:HGNC Symbol;Acc:HGNC:4456]	10316	-0.463666677	-0.26704264
							ETFDH	electron transfer flavoprotein dehydrogenase [Source:HGNC Symbol;Acc:HGNC:3483]	10321	-0.46419999	-0.26030993

APICAL SURFACE	34	-0.32160196	0.13849287	0.17939587	0.447	2076	THY1	Thy-1 cell surface antigen [Source:HGNC Symbol;Acc:HGNC:11801]	11726	-0.581216693	-0.29506359
							APP	amyloid beta precursor protein [Source:HGNC Symbol;Acc:HGNC:620]	12052	-0.627916694	-0.29000008
							ADIPOR2	adiponectin receptor 2 [Source:HGNC Symbol;Acc:HGNC:24041]	12109	-0.638066649	-0.26493365
							TMEM8B	transmembrane protein 8B [Source:HGNC Symbol;Acc:HGNC:21427]	12142	-0.643113315	-0.23789346
							MDGA1	MAM domain containing glycosylphosphatidylinositol anchor 1 [Source:HGNC Symbol;Acc:HGNC:19267]	12335	-0.678786695	-0.22084644
							GAS1	growth arrest specific 1 [Source:HGNC Symbol;Acc:HGNC:4165]	12596	-0.736490011	-0.20610404
							ATP6V0A4	ATPase H+ transporting V0 subunit a4 [Source:HGNC Symbol;Acc:HGNC:866]	12629	-0.745500028	-0.17438889
							SLC2A4	solute carrier family 2 member 4 [Source:HGNC Symbol;Acc:HGNC:11009]	13208	-0.953143358	-0.17285278
							IL2RB	interleukin 2 receptor subunit beta [Source:HGNC Symbol;Acc:HGNC:6009]	13434	-1.126933336	-0.13774042
							MAL	mal, T cell differentiation protein [Source:HGNC Symbol;Acc:HGNC:6817]	13440	-1.13411665	-0.086319804
							PLAUR	plasminogen activator, urokinase receptor [Source:HGNC Symbol;Acc:HGNC:9053]	13472	-1.174236655	-0.03495589
							HSPB1	heat shock protein family B (small) member 1 [Source:HGNC Symbol;Acc:HGNC:5246]	13577	-1.285770059	0.01619811
INFLAMMATORY RESPONSE	177	-0.3017759	0	0.17906211	0.624	2905	CX3CL1	C-X3-C motif chemokine ligand 1 [Source:HGNC Symbol;Acc:HGNC:10647]	10897	-0.482749999	-0.29745236
							BDKRB1	bradykinin receptor B1 [Source:HGNC Symbol;Acc:HGNC:1029]	10900	-0.48312667	-0.29327223
							EBI3	Epstein-Barr virus induced 3 [Source:HGNC Symbol;Acc:HGNC:3129]	10902	-0.483213335	-0.28901795
							RASGRP1	RAS guanyl releasing protein 1 [Source:HGNC Symbol;Acc:HGNC:9878]	10982	-0.491793334	-0.29041198
							KCNMB2	potassium calcium-activated channel subfamily M regulatory beta subunit 2 [Source:HGNC Symbol;Acc:HGNC:6286]	10988	-0.492723346	-0.2863661
							OSM	oncostatin M [Source:HGNC Symbol;Acc:HGNC:8506]	11005	-0.495119989	-0.28310618
							ACVR2A	activin A receptor type 2A [Source:HGNC Symbol;Acc:HGNC:173]	11055	-0.499253333	-0.28223142
							ATP2C1	ATPase secretory pathway Ca2+ transporting 1 [Source:HGNC Symbol;Acc:HGNC:13211]	11116	-0.505393326	-0.28210905
							IL12B	interleukin 12B [Source:HGNC Symbol;Acc:HGNC:5970]	11207	-0.515919983	-0.28409442
							RELA	RELA proto-oncogene, NF-kB subunit [Source:HGNC Symbol;Acc:HGNC:9955]	11228	-0.517629981	-0.2809265
							KLF6	Kruppel like factor 6 [Source:HGNC Symbol;Acc:HGNC:2235]	11257	-0.520950019	-0.27831602
							IRF7	interferon regulatory factor 7 [Source:HGNC Symbol;Acc:HGNC:6122]	11352	-0.530796647	-0.28046176
							FZD5	frizzled class receptor 5 [Source:HGNC Symbol;Acc:HGNC:4043]	11355	-0.530913353	-0.27585363
							TLR2	toll like receptor 2 [Source:HGNC Symbol;Acc:HGNC:11848]	11380	-0.534143329	-0.2728314
							SLC7A2	solute carrier family 7 member 2 [Source:HGNC Symbol;Acc:HGNC:11060]	11422	-0.539099991	-0.27101257
							ICAM1	intercellular adhesion molecule 1 [Source:HGNC Symbol;Acc:HGNC:5344]	11436	-0.541023314	-0.26712132
							ACVR1B	activin A receptor type 1B [Source:HGNC Symbol;Acc:HGNC:172]	11447	-0.542333305	-0.26299813
							CD82	CD82 molecule [Source:HGNC Symbol;Acc:HGNC:6210]	11470	-0.544423342	-0.25973704
							PSEN1	presenilin 1 [Source:HGNC Symbol;Acc:HGNC:9508]	11555	-0.556500018	-0.26091856
							CLEC5A	C-type lectin domain containing 5A [Source:HGNC Symbol;Acc:HGNC:2054]	11557	-0.556683362	-0.25600627
							OSMR	oncostatin M receptor [Source:HGNC Symbol;Acc:HGNC:8507]	11614	-0.564320028	-0.25506255
							IRAK2	interleukin 1 receptor associated kinase 2 [Source:HGNC Symbol;Acc:HGNC:6113]	11651	-0.570003331	-0.25259995
							IFNGR2	interferon gamma receptor 2 [Source:HGNC Symbol;Acc:HGNC:5440]	11668	-0.572526693	-0.24864675
							TNFRSF1B	TNF receptor superfamily member 1B [Source:HGNC Symbol;Acc:HGNC:11917]	11671	-0.572976649	-0.24366193
							MET	MET proto-oncogene, receptor tyrosine kinase [Source:HGNC Symbol;Acc:HGNC:7029]	11681	-0.573976696	-0.23918194
							LCK	LCK proto-oncogene, Src family tyrosine kinase [Source:HGNC Symbol;Acc:HGNC:6524]	11744	-0.582599998	-0.23851492
							GNAI3	G protein subunit alpha i3 [Source:HGNC Symbol;Acc:HGNC:4387]	11746	-0.582729995	-0.23336934
							CCL2	C-C motif chemokine ligand 2 [Source:HGNC Symbol;Acc:HGNC:10618]	11766	-0.586263359	-0.22951332
							ABCA1	ATP binding cassette subfamily A member 1 [Source:HGNC Symbol;Acc:HGNC:29]	11819	-0.59187001	-0.22802927
							F3	coagulation factor III, tissue factor [Source:HGNC Symbol;Acc:HGNC:3541]	11820	-0.591936648	-0.22272784
							CD69	CD69 molecule [Source:HGNC Symbol;Acc:HGNC:1694]	11859	-0.598246694	-0.22015908
							ADORA2B	adenosine A2b receptor [Source:HGNC Symbol;Acc:HGNC:264]	11906	-0.604849994	-0.21811838
							SLC1A2	solute carrier family 1 member 2 [Source:HGNC Symbol;Acc:HGNC:10940]	11925	-0.607476652	-0.21399897
							TNFRSF9	TNF receptor superfamily member 9 [Source:HGNC Symbol;Acc:HGNC:11924]	12103	-0.637040019	-0.22128537
							GPR183	G protein-coupled receptor 183 [Source:HGNC Symbol;Acc:HGNC:3128]	12149	-0.644206643	-0.2188188
							LDLR	low density lipoprotein receptor [Source:HGNC Symbol;Acc:HGNC:6547]	12150	-0.644280016	-0.21304858
							RGS16	regulator of G protein signaling 16 [Source:HGNC Symbol;Acc:HGNC:9997]	12152	-0.644573331	-0.20734912
							CCL7	C-C motif chemokine ligand 7 [Source:HGNC Symbol;Acc:HGNC:10634]	12180	-0.649633348	-0.20351274
							EDN1	endothelin 1 [Source:HGNC Symbol;Acc:HGNC:3176]	12205	-0.654429972	-0.19941321
							RTP4	receptor transporter protein 4 [Source:HGNC Symbol;Acc:HGNC:23992]	12222	-0.657199979	-0.19470167
							SCN1B	sodium voltage-gated channel beta subunit 1 [Source:HGNC Symbol;Acc:HGNC:10586]	12232	-0.66030997	-0.18944848
							CD48	CD48 molecule [Source:HGNC Symbol;Acc:HGNC:1683]	12248	-0.662580013	-0.18461536
							CMKLR1	chemerin chemokine-like receptor 1 [Source:HGNC Symbol;Acc:HGNC:2121]	12260	-0.664596677	-0.17947057
							IL7R	interleukin 7 receptor [Source:HGNC Symbol;Acc:HGNC:6024]	12262	-0.66470331	-0.17359082
							TNFAIP6	TNF alpha induced protein 6 [Source:HGNC Symbol;Acc:HGNC:11898]	12439	-0.700743318	-0.18023328
							PCDH7	protocadherin 7 [Source:HGNC Symbol;Acc:HGNC:8659]	12504	-0.715433359	-0.1785234
							GPR132	G protein-coupled receptor 132 [Source:HGNC Symbol;Acc:HGNC:17482]	12516	-0.718116641	-0.17289929
							KCNJ2	potassium inwardly rectifying channel subfamily J member 2 [Source:HGNC Symbol;Acc:HGNC:6263]	12620	-0.744173348	-0.1737946
							ITGA5	integrin subunit alpha 5 [Source:HGNC Symbol;Acc:HGNC:6141]	12630	-0.746190012	-0.16777225
							CCL5	C-C motif chemokine ligand 5 [Source:HGNC Symbol;Acc:HGNC:10632]	12631	-0.746720016	-0.16108456
							AXL	AXL receptor tyrosine kinase [Source:HGNC Symbol;Acc:HGNC:905]	12714	-0.768093348	-0.16022424
							SLC11A2	solute carrier family 11 member 2 [Source:HGNC Symbol;Acc:HGNC:10908]	12794	-0.791100025	-0.15893768
							NLRP3	NLR family pyrin domain containing 3 [Source:HGNC Symbol;Acc:HGNC:16400]	12905	-0.822486639	-0.15964541
							BTG2	BTG anti-proliferation factor 2 [Source:HGNC Symbol;Acc:HGNC:1131]	13095	-0.905489981	-0.16540834
							ADGRE1	adhesion G protein-coupled receptor E1 [Source:HGNC Symbol;Acc:HGNC:3336]	13126	-0.919470012	-0.15937549
							IL10RA	interleukin 10 receptor subunit alpha [Source:HGNC Symbol;Acc:HGNC:5964]	13133	-0.921263337	-0.15156497
							CCR7	C-C motif chemokine receptor 7 [Source:HGNC Symbol;Acc:HGNC:1608]	13259	-0.981283307	-0.1519515
							IFITM1	interferon induced transmembrane protein 1 [Source:HGNC Symbol;Acc:HGNC:5412]	13262	-0.983173311	-0.14329292
							SEMA4D	semaphorin 4D [Source:HGNC Symbol;Acc:HGNC:10732]	13328	-1.023953319	-0.13889329
							LAMP3	lysosomal associated membrane protein 3 [Source:HGNC Symbol;Acc:HGNC:14582]	13354	-1.048290014	-0.13133971
							BEST1	bestrophin 1 [Source:HGNC Symbol;Acc:HGNC:12703]	13411	-1.097833276	-0.12561783
							IL2RB	interleukin 2 receptor subunit beta [Source:HGNC Symbol;Acc:HGNC:6009]	13434	-1.126933336	-0.11713971
							PLAUR	plasminogen activator, urokinase receptor [Source:HGNC Symbol;Acc:HGNC:9053]	13472	-1.174236655	-0.10933895
							CD14	CD14 molecule [Source:HGNC Symbol;Acc:HGNC:1628]	13476	-1.176620007	-0.09902124
							CXCR6	C-X-C motif chemokine receptor 6 [Source:HGNC Symbol;Acc:HGNC:16647]	13500	-1.194630027	-0.090010226
							SERPINE1	serpin family E member 1 [Source:HGNC Symbol;Acc:HGNC:8583]	13513	-1.200383306	-0.08014029
							SELL	selectin L [Source:HGNC Symbol;Acc:HGNC:10720]	13518	-1.203663349	-0.06965378
							PDE4B	phosphodiesterase 4B [Source:HGNC Symbol;Acc:HGNC:8781]	13580	-1.308416724	-0.062412884
							CXCL9	C-X-C motif chemokine ligand 9 [Source:HGNC Symbol;Acc:HGNC:7098]	13591	-1.330023289	-0.051235083
							ATP2A2	ATPase sarcoplasmic/endoplasmic reticulum Ca2+ transporting 2 [Source:HGNC Symbol;Acc:HGNC:812]	13603	-1.343680024	-0.04000837
							TLR1	toll like receptor 1 [Source:HGNC Symbol;Acc:HGNC:11847]	13604	-1.34801662	-0.027935417
							TNFSF9	TNF superfamily member 9 [Source:HGNC Symbol;Acc:HGNC:11939]	13664	-1.49502337	-0.018876456
							LY6E	lymphocyte antigen 6 family member E [Source:HGNC Symbol;Acc:HGNC:6727]	13691	-1.602270007	-0.006434788
							CCL17	C-C motif chemokine ligand 17 [Source:HGNC Symbol;Acc:HGNC:10615]	13694	-1.603579998	0.007780211
ALLOGRAFT REJECTION	174	-0.28876418	0.00201613	0.20412254	0.717	2242	BCL3	BCL3 transcription coactivator [Source:HGNC Symbol;Acc:HGNC:998]	11560	-0.556949973	-0.28425762
							DYRK3	dual specificity tyrosine phosphorylation regulated kinase 3 [Source:HGNC Symbol;Acc:HGNC:3094]	11571	-0.558030009	-0.28047615
							IFNGR2	interferon gamma receptor 2 [Source:HGNC Symbol;Acc:HGNC:5440]	11668	-0.572526693	-0.28288835
							CTSS	cathepsin S [Source:HGNC Symbol;Acc:HGNC:2545]	11705	-0.578629971	-0.28084818
							THY1	Thy-1 cell surface antigen [Source:HGNC Symbol;Acc:HGNC:11801]	11726	-0.581216693	-0.27761292
							ZAP70	zeta chain of T cell receptor associated protein kinase 70 [Source:HGNC Symbol;Acc:HGNC:12858]	11730	-0.581623316	-0.27312684
							LCK	LCK proto-oncogene, Src family tyrosine kinase [Source:HGNC Symbol;Acc:HGNC:6524]	11744	-0.582599998	-0.2693667
							CCL2	C-C motif chemokine ligand 2 [Source:HGNC Symbol;Acc:HGNC:10618]	11766	-0.586263359	-0.266164
							CCND2	cyclin D2 [Source:HGNC Symbol;Acc:HGNC:1583]	11889	-0.602416694	-0.27024236
							WARS1	tryptophanyl-tRNA synthetase 1 [Source:HGNC Symbol;Acc:HGNC:12729]	11958	-0.613456666	-0.27026865
							CD247	CD247 molecule [Source:HGNC Symbol;Acc:HGNC:1677]	12041	-0.625753343	-0.2712228
							FASLG	Fas ligand [Source:HGNC Symbol;Acc:HGNC:11936]	12171	-0.647996664	-0.27544603
							CCL7	C-C motif chemokine ligand 7 [Source:HGNC Symbol;Acc:HGNC:10634]	12180	-0.649633348	-0.27077657
							ITK	IL2 inducible T cell kinase [Source:HGNC Symbol;Acc:HGNC:6171]	12231	-0.660243332	-0.26910338
							CD28	CD28 molecule [Source:HGNC Symbol;Acc:HGNC:1653]	12241	-0.66111666	-0.2644144
							CD3E	CD3e molecule [Source:HGNC Symbol;Acc:HGNC:1674]	12253	-0.663453341	-0.25985327
							NOS2	nitric oxide synthase 2 [Source:HGNC Symbol;Acc:HGNC:7873]	12274	-0.666809976	-0.25592542
							ITGAL	integrin subunit alpha L [Source:HGNC Symbol;Acc:HGNC:6148]	12288	-0.669430017	-0.2514627
							RPL3L	ribosomal protein L3 like [Source:HGNC Symbol;Acc:HGNC:10351]	12332	-0.677779973	-0.24913393
							C2	complement C2 [Source:HGNC Symbol;Acc:HGNC:1248]	12436	-0.700576663	-0.2510237
							CAPG	capping actin protein, gelsolin like [Source:HGNC Symbol;Acc:HGNC:1474]	12444	-0.701780021	-0.24585892
							PRKCB	protein kinase C beta [Source:HGNC Symbol;Acc:HGNC:9395]	12521	-0.719506681	-0.24561419
							LY86	lymphocyte antigen 86 [Source:HGNC Symbol;Acc:HGNC:16837]	12568	-0.728389978	-0.24309605
							CCL5	C-C motif chemokine ligand 5 [Source:HGNC Symbol;Acc:HGNC:10632]	12631	-0.746720016	-0.24160373
							HLA-DQA1	major histocompatibility complex, class II, DQ alpha 1 [Source:HGNC Symbol;Acc:HGNC:4942]	12633	-0.747590005	-0.23562796
							HLA-DMA	major histocompatibility complex, class II, DM alpha [Source:HGNC Symbol;Acc:HGNC:4934]	12687	-0.760209978	-0.23336604
							F2R	coagulation factor II thrombin receptor [Source:HGNC Symbol;Acc:HGNC:3537]	12713	-0.767446637	-0.22899082
							B2M	beta-2-microglobulin [Source:HGNC Symbol;Acc:HGNC:914]	12860	-0.809759974	-0.23315266
							FYB1	FYN binding protein 1 [Source:HGNC Symbol;Acc:HGNC:4036]	12867	-0.81262666	-0.22701757
							NLRP3	NLR family pyrin domain containing 3 [Source:HGNC Symbol;Acc:HGNC:16400]	12905	-0.822486639	-0.2230776
							CD96	CD96 molecule [Source:HGNC Symbol;Acc:HGNC:16892]	12950	-0.836556673	-0.21953745
							RPL9	ribosomal protein L9 [Source:HGNC Symbol;Acc:HGNC:10369]	12960	-0.839636683	-0.21340397
							MRPL3	mitochondrial ribosomal protein L3 [Source:HGNC Symbol;Acc:HGNC:10379]	12999	-0.856913328	-0.20925881
							HCLS1	hematopoietic cell-specific Lyn substrate 1 [Source:HGNC Symbol;Acc:HGNC:4844]	13017	-0.867826641	-0.2034843
							INHBB	inhibin subunit beta B [Source:HGNC Symbol;Acc:HGNC:6067]	13034	-0.871930003	-0.19760318
							PTPRC	protein tyrosine phosphatase receptor type C [Source:HGNC Symbol;Acc:HGNC:9666]	13076	-0.892926693	-0.19338678
							ITGB2	integrin subunit beta 2 [Source:HGNC Symbol;Acc:HGNC:6155]	13085	-0.90079999	-0.186685
							TPD52	tumor protein D52 [Source:HGNC Symbol;Acc:HGNC:12005]	13102	-0.907633305	-0.180515
							STAT1	signal transducer and activator of transcription 1 [Source:HGNC Symbol;Acc:HGNC:11362]	13149	-0.930766642	-0.17635933
							GZMA	granzyme A [Source:HGNC Symbol;Acc:HGNC:4708]	13154	-0.932073355	-0.16911097
							CCR1	C-C motif chemokine receptor 1 [Source:HGNC Symbol;Acc:HGNC:1602]	13166	-0.938040018	-0.162328
							TAP1	transporter 1, ATP binding cassette subfamily B member [Source:HGNC Symbol;Acc:HGNC:43]	13178	-0.94168669	-0.15551555
							CD74	CD74 molecule [Source:HGNC Symbol;Acc:HGNC:1697]	13339	-1.037240028	-0.15886408
							FCGR2B	Fc fragment of IgG receptor IIb [Source:HGNC Symbol;Acc:HGNC:3618]	13369	-1.063583374	-0.1523862
							KLRD1	killer cell lectin like receptor D1 [Source:HGNC Symbol;Acc:HGNC:6378]	13378	-1.071910024	-0.14429988
							IL2RB	interleukin 2 receptor subunit beta [Source:HGNC Symbol;Acc:HGNC:6009]	13434	-1.126933336	-0.13921738
							PTPN6	protein tyrosine phosphatase non-receptor type 6 [Source:HGNC Symbol;Acc:HGNC:9658]	13467	-1.167333364	-0.13212015
							TRAT1	T cell receptor associated transmembrane adaptor 1 [Source:HGNC Symbol;Acc:HGNC:30698]	13583	-1.316996694	-0.12990277
							CXCL9	C-X-C motif chemokine ligand 9 [Source:HGNC Symbol;Acc:HGNC:7098]	13591	-1.330023289	-0.11965453
							TLR1	toll like receptor 1 [Source:HGNC Symbol;Acc:HGNC:11847]	13604	-1.34801662	-0.10962762
							PRF1	perforin 1 [Source:HGNC Symbol;Acc:HGNC:9360]	13609	-1.361283302	-0.09890629
							CD8B	CD8b molecule [Source:HGNC Symbol;Acc:HGNC:1707]	13645	-1.444059968	-0.08979007
							HLA-DOB	major histocompatibility complex, class II, DO beta [Source:HGNC Symbol;Acc:HGNC:4937]	13659	-1.480736613	-0.078762636
							GZMB	granzyme B [Source:HGNC Symbol;Acc:HGNC:4709]	13699	-1.649469972	-0.06827787
							NCR1	natural cytotoxicity triggering receptor 1 [Source:HGNC Symbol;Acc:HGNC:6731]	13705	-1.693636656	-0.054940686

							CBR1	carbonyl reductase 1 [Source:HGNC Symbol;Acc:HGNC:1548]	10497	-0.47832334	-0.21056141
							METAP1	methionyl aminopeptidase 1 [Source:HGNC Symbol;Acc:HGNC:15789]	10504	-0.479070008	-0.20393346
							ACOX1	acyl-CoA oxidase 1 [Source:HGNC Symbol;Acc:HGNC:119]	10571	-0.486626655	-0.20180944
							SDHA	succinate dehydrogenase complex flavoprotein subunit A [Source:HGNC Symbol;Acc:HGNC:10680]	10602	-0.49011001	-0.19686441
							CPOX	coproporphyrinogen oxidase [Source:HGNC Symbol;Acc:HGNC:2321]	10637	-0.494230002	-0.19216612
							HIBCH	3-hydroxyisobutyryl-CoA hydrolase [Source:HGNC Symbol;Acc:HGNC:4908]	10650	-0.495730013	-0.18575321
							CD1D	CD1d molecule [Source:HGNC Symbol;Acc:HGNC:1637]	10721	-0.502539992	-0.1837014
							IDH1	isocitrate dehydrogenase (NADP(+)) 1 [Source:HGNC Symbol;Acc:HGNC:5382]	10906	-0.522189975	-0.1901287
							DHCR24	24-dehydrocholesterol reductase [Source:HGNC Symbol;Acc:HGNC:2859]	11014	-0.534493327	-0.1904504
							ADH7	alcohol dehydrogenase 7 (class IV), mu or sigma polypeptide [Source:HGNC Symbol;Acc:HGNC:256]	11071	-0.541766644	-0.18674108
							GLUL	glutamate-ammonia ligase [Source:HGNC Symbol;Acc:HGNC:4341]	11093	-0.54387331	-0.18030807
							GPD1	glycerol-3-phosphate dehydrogenase 1 [Source:HGNC Symbol;Acc:HGNC:4455]	11223	-0.560663342	-0.18193494
							ENO2	enolase 2 [Source:HGNC Symbol;Acc:HGNC:3353]	11234	-0.562526643	-0.17437968
							REEP6	receptor accessory protein 6 [Source:HGNC Symbol;Acc:HGNC:30078]	11249	-0.56480664	-0.16709839
							LGALS1	galectin 1 [Source:HGNC Symbol;Acc:HGNC:6561]	11388	-0.585603356	-0.16904853
							SDHD	succinate dehydrogenase complex subunit D [Source:HGNC Symbol;Acc:HGNC:10683]	11412	-0.588620007	-0.1621072
							ECHS1	enoyl-CoA hydratase, short chain 1 [Source:HGNC Symbol;Acc:HGNC:3151]	11451	-0.592966676	-0.15625548
							ALDH9A1	aldehyde dehydrogenase 9 family member A1 [Source:HGNC Symbol;Acc:HGNC:412]	11459	-0.593946695	-0.14800446
							ACADL	acyl-CoA dehydrogenase long chain [Source:HGNC Symbol;Acc:HGNC:88]	11539	-0.606243312	-0.14511035
							ADIPOR2	adiponectin receptor 2 [Source:HGNC Symbol;Acc:HGNC:24041]	11715	-0.638066649	-0.1491305
							SERINC1	serine incorporator 1 [Source:HGNC Symbol;Acc:HGNC:13464]	11896	-0.671490014	-0.15304066
							EHHADH	enoyl-CoA hydratase and 3-hydroxyacyl CoA dehydrogenase [Source:HGNC Symbol;Acc:HGNC:3247]	12084	-0.716523349	-0.1568229
							ENO3	enolase 3 [Source:HGNC Symbol;Acc:HGNC:3354]	12150	-0.732990026	-0.15097614
							CD36	CD36 molecule [Source:HGNC Symbol;Acc:HGNC:1663]	12250	-0.760673344	-0.14733528
							EPHX1	epoxide hydrolase 1 [Source:HGNC Symbol;Acc:HGNC:3401]	12317	-0.782016695	-0.1408399
							HMGCL	3-hydroxy-3-methylglutaryl-CoA lyase [Source:HGNC Symbol;Acc:HGNC:5005]	12359	-0.796599984	-0.1322055
							RETSAT	retinol saturase [Source:HGNC Symbol;Acc:HGNC:25991]	12493	-0.843850017	-0.12994933
							ACSS1	acyl-CoA synthetase short chain family member 1 [Source:HGNC Symbol;Acc:HGNC:16091]	12509	-0.851783335	-0.118498154
							ALDH1A1	aldehyde dehydrogenase 1 family member A1 [Source:HGNC Symbol;Acc:HGNC:402]	12577	-0.889036655	-0.11049598
							D2HGDH	D-2-hydroxyglutarate dehydrogenase [Source:HGNC Symbol;Acc:HGNC:28358]	12615	-0.910223305	-0.0998724
							IDI1	isopentenyl-diphosphate delta isomerase 1 [Source:HGNC Symbol;Acc:HGNC:5387]	12743	-0.987006664	-0.095036164
							SUCLG1	succinate-CoA ligase GDP/ADP-forming subunit alpha [Source:HGNC Symbol;Acc:HGNC:11449]	12772	-1.002529979	-0.082354225
							HSPH1	heat shock protein family H (Hsp110) member 1 [Source:HGNC Symbol;Acc:HGNC:16969]	12858	-1.09310329	-0.07271689
							ALDH3A1	aldehyde dehydrogenase 3 family member A1 [Source:HGNC Symbol;Acc:HGNC:405]	12862	-1.102040052	-0.05663913
							ACSL1	acyl-CoA synthetase long chain family member 1 [Source:HGNC Symbol;Acc:HGNC:3569]	12905	-1.17513001	-0.042479977
							ADSL	adenylosuccinate lyase [Source:HGNC Symbol;Acc:HGNC:291]	12942	-1.215639949	-0.027259763
							CYP4A22	cytochrome P450 family 4 subfamily A member 22 [Source:HGNC Symbol;Acc:HGNC:20575]	12999	-1.339093328	-0.011751204
							FASN	fatty acid synthase [Source:HGNC Symbol;Acc:HGNC:3594]	13024	-1.428286672	0.007539011
APICAL SURFACE	31	-0.2879237	0.2734531	0.1748431	0.722	1770	THY1	Thy-1 cell surface antigen [Source:HGNC Symbol;Acc:HGNC:11801]	11354	-0.581216693	-0.25730896
							APP	amyloid beta precursor protein [Source:HGNC Symbol;Acc:HGNC:620]	11664	-0.627916694	-0.24783655
							ADIPOR2	adiponectin receptor 2 [Source:HGNC Symbol;Acc:HGNC:24041]	11715	-0.638066649	-0.21804641
							TMEM8B	transmembrane protein 8B [Source:HGNC Symbol;Acc:HGNC:21427]	11746	-0.643113315	-0.18646282
							MDGA1	MAM domain containing glycosylphosphatidylinositol anchor 1 [Source:HGNC Symbol;Acc:HGNC:19267]	11930	-0.678786695	-0.16468671
							GAS1	growth arrest specific 1 [Source:HGNC Symbol;Acc:HGNC:4165]	12164	-0.736490011	-0.14369029
							ATP6V0A4	ATPase H+ transporting V0 subunit a4 [Source:HGNC Symbol;Acc:HGNC:866]	12194	-0.745500028	-0.10663724
							SLC2A4	solute carrier family 2 member 4 [Source:HGNC Symbol;Acc:HGNC:11009]	12694	-0.953143358	-0.09454666
							IL2RB	interleukin 2 receptor subunit beta [Source:HGNC Symbol;Acc:HGNC:6009]	12876	-1.126933336	-0.0490123
							HSPB1	heat shock protein family B (small) member 1 [Source:HGNC Symbol;Acc:HGNC:5246]	12981	-1.285770059	0.010770009
INFLAMMATORY RESPONSE	158	-0.32278055	0	0.1464325	0.466	2592	CX3CL1	C-X3-C motif chemokine ligand 1 [Source:HGNC Symbol;Acc:HGNC:10647]	10532	-0.482749999	-0.31741098
							BDKRB1	bradykinin receptor B1 [Source:HGNC Symbol;Acc:HGNC:1029]	10535	-0.48312667	-0.31219146
							EBI3	Epstein-Barr virus induced 3 [Source:HGNC Symbol;Acc:HGNC:3129]	10537	-0.483213335	-0.30689386
							RASGRP1	RAS guanyl releasing protein 1 [Source:HGNC Symbol;Acc:HGNC:9878]	10618	-0.491793334	-0.30759415
							KCNMB2	potassium calcium-activated channel subfamily M regulatory beta subunit 2 [Source:HGNC Symbol;Acc:HGNC:6286]	10624	-0.492723346	-0.3024993
							OSM	oncostatin M [Source:HGNC Symbol;Acc:HGNC:8506]	10641	-0.495119989	-0.2982262
							ACVR2A	activin A receptor type 2A [Source:HGNC Symbol;Acc:HGNC:173]	10690	-0.499253333	-0.29637533
							ATP2C1	ATPase secretory pathway Ca2+ transporting 1 [Source:HGNC Symbol;Acc:HGNC:13211]	10752	-0.505393326	-0.29545888
							IL12B	interleukin 12B [Source:HGNC Symbol;Acc:HGNC:5970]	10844	-0.515919983	-0.29673925
							RELA	RELA proto-oncogene, NF-kB subunit [Source:HGNC Symbol;Acc:HGNC:9955]	10865	-0.517629981	-0.2925243
							KLF6	Kruppel like factor 6 [Source:HGNC Symbol;Acc:HGNC:2235]	10894	-0.520950019	-0.2888895
							IRF7	interferon regulatory factor 7 [Source:HGNC Symbol;Acc:HGNC:6122]	10986	-0.530796647	-0.2900044
							FZD5	frizzled class receptor 5 [Source:HGNC Symbol;Acc:HGNC:4043]	10988	-0.530913353	-0.28417623
							TLR2	toll like receptor 2 [Source:HGNC Symbol;Acc:HGNC:11848]	11012	-0.534143329	-0.28000903
							SLC7A2	solute carrier family 7 member 2 [Source:HGNC Symbol;Acc:HGNC:11060]	11052	-0.539099991	-0.27702078
							ICAM1	intercellular adhesion molecule 1 [Source:HGNC Symbol;Acc:HGNC:5344]	11066	-0.541023314	-0.27200574
							ACVR1B	activin A receptor type 1B [Source:HGNC Symbol;Acc:HGNC:172]	11077	-0.542333305	-0.2667447
							PSEN1	presenilin 1 [Source:HGNC Symbol;Acc:HGNC:9508]	11184	-0.556500018	-0.26873067
							CLEC5A	C-type lectin domain containing 5A [Source:HGNC Symbol;Acc:HGNC:2054]	11186	-0.556683362	-0.2626159
							OSMR	oncostatin M receptor [Source:HGNC Symbol;Acc:HGNC:8507]	11243	-0.564320028	-0.26065832
							IRAK2	interleukin 1 receptor associated kinase 2 [Source:HGNC Symbol;Acc:HGNC:6113]	11280	-0.570003331	-0.25709495
							TNFRSF1B	TNF receptor superfamily member 1B [Source:HGNC Symbol;Acc:HGNC:11917]	11299	-0.572976649	-0.25211012
							MET	MET proto-oncogene, receptor tyrosine kinase [Source:HGNC Symbol;Acc:HGNC:7029]	11309	-0.573976696	-0.24642003
							LCK	LCK proto-oncogene, Src family tyrosine kinase [Source:HGNC Symbol;Acc:HGNC:6524]	11372	-0.582599998	-0.24472193
							GNAI3	G protein subunit alpha i3 [Source:HGNC Symbol;Acc:HGNC:4387]	11374	-0.582729995	-0.23831742
							CCL2	C-C motif chemokine ligand 2 [Source:HGNC Symbol;Acc:HGNC:10618]	11392	-0.586263359	-0.23310769
							ABCA1	ATP binding cassette subfamily A member 1 [Source:HGNC Symbol;Acc:HGNC:29]	11444	-0.59187001	-0.23045805
							F3	coagulation factor III, tissue factor [Source:HGNC Symbol;Acc:HGNC:3541]	11445	-0.591936648	-0.223874
							CD69	CD69 molecule [Source:HGNC Symbol;Acc:HGNC:1694]	11484	-0.598246694	-0.22015072
							ADORA2B	adenosine A2b receptor [Source:HGNC Symbol;Acc:HGNC:264]	11526	-0.604849994	-0.2165854
							SLC1A2	solute carrier family 1 member 2 [Source:HGNC Symbol;Acc:HGNC:10940]	11546	-0.607476652	-0.211294
							KIF1B	kinesin family member 1B [Source:HGNC Symbol;Acc:HGNC:16636]	11689	-0.63198334	-0.21521707
							TNFRSF9	TNF receptor superfamily member 9 [Source:HGNC Symbol;Acc:HGNC:11924]	11709	-0.637040019	-0.20959681
							GPR183	G protein-coupled receptor 183 [Source:HGNC Symbol;Acc:HGNC:3128]	11752	-0.644206643	-0.20567086
							LDLR	low density lipoprotein receptor [Source:HGNC Symbol;Acc:HGNC:6547]	11753	-0.644280016	-0.1985046
							RGS16	regulator of G protein signaling 16 [Source:HGNC Symbol;Acc:HGNC:9997]	11755	-0.644573331	-0.19141221
							CCL7	C-C motif chemokine ligand 7 [Source:HGNC Symbol;Acc:HGNC:10634]	11784	-0.649633348	-0.18634607
							EDN1	endothelin 1 [Source:HGNC Symbol;Acc:HGNC:3176]	11807	-0.654429972	-0.18076378
							RTP4	receptor transporter protein 4 [Source:HGNC Symbol;Acc:HGNC:23992]	11826	-0.657199979	-0.17484216
							SCN1B	sodium voltage-gated channel beta subunit 1 [Source:HGNC Symbol;Acc:HGNC:10586]	11836	-0.66030997	-0.16819179
							CD48	CD48 molecule [Source:HGNC Symbol;Acc:HGNC:1683]	11851	-0.662580013	-0.1619018
							CMKLR1	chemerin chemokine-like receptor 1 [Source:HGNC Symbol;Acc:HGNC:2121]	11861	-0.664596677	-0.15520374
							IL7R	interleukin 7 receptor [Source:HGNC Symbol;Acc:HGNC:6024]	11863	-0.66470331	-0.14788744
							TNFAIP6	TNF alpha induced protein 6 [Source:HGNC Symbol;Acc:HGNC:11898]	12021	-0.700743318	-0.15220267
							PCDH7	protocadherin 7 [Source:HGNC Symbol;Acc:HGNC:8659]	12078	-0.715433359	-0.14856431
							GPR132	G protein-coupled receptor 132 [Source:HGNC Symbol;Acc:HGNC:17482]	12089	-0.718116641	-0.14134806
							KCNJ2	potassium inwardly rectifying channel subfamily J member 2 [Source:HGNC Symbol;Acc:HGNC:6263]	12186	-0.744173348	-0.14047526
							ITGA5	integrin subunit alpha 5 [Source:HGNC Symbol;Acc:HGNC:6141]	12195	-0.746190012	-0.1327925
							CCL5	C-C motif chemokine ligand 5 [Source:HGNC Symbol;Acc:HGNC:10632]	12196	-0.746720016	-0.12448682
							SLC31A1	solute carrier family 31 member 1 [Source:HGNC Symbol;Acc:HGNC:11016]	12261	-0.766009986	-0.12090293
							AXL	AXL receptor tyrosine kinase [Source:HGNC Symbol;Acc:HGNC:905]	12273	-0.768093348	-0.113207944
							SLC11A2	solute carrier family 11 member 2 [Source:HGNC Symbol;Acc:HGNC:10908]	12341	-0.791100025	-0.10957638
							NLRP3	NLR family pyrin domain containing 3 [Source:HGNC Symbol;Acc:HGNC:16400]	12441	-0.822486639	-0.10806389
							BTG2	BTG anti-proliferation factor 2 [Source:HGNC Symbol;Acc:HGNC:1131]	12606	-0.905489981	-0.11064166
							ICOSLG	inducible T cell costimulator ligand [Source:HGNC Symbol;Acc:HGNC:17087]	12617	-0.911503315	-0.10127441
							ADGRE1	adhesion G protein-coupled receptor E1 [Source:HGNC Symbol;Acc:HGNC:3336]	12628	-0.919470012	-0.09181854
							IL10RA	interleukin 10 receptor subunit alpha [Source:HGNC Symbol;Acc:HGNC:5964]	12633	-0.921263337	-0.08187994
							IFITM1	interferon induced transmembrane protein 1 [Source:HGNC Symbol;Acc:HGNC:5412]	12738	-0.983173311	-0.078965805
							SELE	selectin E [Source:HGNC Symbol;Acc:HGNC:10718]	12793	-1.018596649	-0.07180112
							IL2RB	interleukin 2 receptor subunit beta [Source:HGNC Symbol;Acc:HGNC:6009]	12876	-1.126933336	-0.065591075
							SELL	selectin L [Source:HGNC Symbol;Acc:HGNC:10720]	12935	-1.203663349	-0.056676432
							PDE4B	phosphodiesterase 4B [Source:HGNC Symbol;Acc:HGNC:8781]	12984	-1.308416724	-0.04582532
							CXCL9	C-X-C motif chemokine ligand 9 [Source:HGNC Symbol;Acc:HGNC:7098]	12994	-1.330023289	-0.031725787
							ATP2A2	ATPase sarcoplasmic/endoplasmic reticulum Ca2+ transporting 2 [Source:HGNC Symbol;Acc:HGNC:812]	13002	-1.343680024	-0.017320085
							RGS1	regulator of G protein signaling 1 [Source:HGNC Symbol;Acc:HGNC:9991]	13106	-2.382339954	0.001233974
ALLOGRAFT REJECTION	152	-0.26753882	0.00571429	0.1627772	0.86	2138	IRF7	interferon regulatory factor 7 [Source:HGNC Symbol;Acc:HGNC:6122]	10986	-0.530796647	-0.26120505
							TLR2	toll like receptor 2 [Source:HGNC Symbol;Acc:HGNC:11848]	11012	-0.534143329	-0.25675872
							ICAM1	intercellular adhesion molecule 1 [Source:HGNC Symbol;Acc:HGNC:5344]	11066	-0.541023314	-0.25438896
							BCL3	BCL3 transcription coactivator [Source:HGNC Symbol;Acc:HGNC:998]	11189	-0.556949973	-0.25714868
							DYRK3	dual specificity tyrosine phosphorylation regulated kinase 3 [Source:HGNC Symbol;Acc:HGNC:3094]	11200	-0.558030009	-0.2512609
							CTSS	cathepsin S [Source:HGNC Symbol;Acc:HGNC:2545]	11332	-0.578629971	-0.2544558
							THY1	Thy-1 cell surface antigen [Source:HGNC Symbol;Acc:HGNC:11801]	11354	-0.581216693	-0.24913938
							ZAP70	zeta chain of T cell receptor associated protein kinase 70 [Source:HGNC Symbol;Acc:HGNC:12858]	11358	-0.581623316	-0.2424304
							LCK	LCK proto-oncogene, Src family tyrosine kinase [Source:HGNC Symbol;Acc:HGNC:6524]	11372	-0.582599998	-0.23648071
							CCL2	C-C motif chemokine ligand 2 [Source:HGNC Symbol;Acc:HGNC:10618]	11392	-0.586263359	-0.23094988
							CCND2	cyclin D2 [Source:HGNC Symbol;Acc:HGNC:1583]	11512	-0.602416694	-0.23293582
							CD247	CD247 molecule [Source:HGNC Symbol;Acc:HGNC:1677]	11654	-0.625753343	-0.23633936
							FASLG	Fas ligand [Source:HGNC Symbol;Acc:HGNC:11936]	11775	-0.647996664	-0.23785849
							CCL7	C-C motif chemokine ligand 7 [Source:HGNC Symbol;Acc:HGNC:10634]	11784	-0.649633348	-0.23072344
							ITK	IL2 inducible T cell kinase [Source:HGNC Symbol;Acc:HGNC:6171]	11835	-0.660243332	-0.22669978
							CD28	CD28 molecule [Source:HGNC Symbol;Acc:HGNC:1653]	11844	-0.66111666	-0.21942772
							CD3E	CD3e molecule [Source:HGNC Symbol;Acc:HGNC:1674]	11856	-0.663453341	-0.21235906
							NOS2	nitric oxide synthase 2 [Source:HGNC Symbol;Acc:HGNC:7873]	11874	-0.666809976	-0.2057129
							ITGAL	integrin subunit alpha L [Source:HGNC Symbol;Acc:HGNC:6148]	11885	-0.669430017	-0.19849582
							RPL3L	ribosomal protein L3 like [Source:HGNC Symbol;Acc:HGNC:10351]	11928	-0.677779973	-0.19364615
							C2	complement C2 [Source:HGNC Symbol;Acc:HGNC:1248]	12019	-0.700576663	-0.19222501
							CAPG	capping actin protein, gelsolin like [Source:HGNC Symbol;Acc:HGNC:1474]	12026	-0.701780021	-0.18431354
							PRKCB	protein kinase C beta [Source:HGNC Symbol;Acc:HGNC:9395]	12094	-0.719506681	-0.18089333
							LY86	lymphocyte antigen 86 [Source:HGNC Symbol;Acc:HGNC:16837]	12137	-0.728389978	-0.17543975
							CCL5	C-C motif chemokine ligand 5 [Source:HGNC Symbol;Acc:HGNC:10632]	12196	-0.746720016	-0.17100096
							F2R	coagulation factor II thrombin receptor [Source:HGNC Symbol;Acc:HGNC:3537]	12272	-0.767446637	-0.16762547
							GBP2	guanylate binding protein 2 [Source:HGNC Symbol;Acc:HGNC:4183]	12361	-0.796963334	-0.1649
							B2M	beta-2-microglobulin [Source:HGNC Symbol;Acc:HGNC:914]	12403	-0.809759974	-0.15839837
							FYB1	FYN binding protein 1 [Source:HGNC Symbol;Acc:HGNC:4036]	12410	-0.81262666	-0.14916421
							NLRP3	NLR family pyrin domain containing 3 [Source:HGNC Symbol;Acc:HGNC:16400]	12441	-0.822486639	-0.14166267
							CD96	CD96 molecule [Source:HGNC Symbol;Acc:HGNC:16892]	12480	-0.836556673	-0.13461
							RPL9	ribosomal protein L9 [Source:HGNC Symbol;Acc:HGNC:10369]	12485	-0.839636683	-0.12489934
							MRPL3	mitochondrial ribosomal protein L3 [Source:HGNC Symbol;Acc:HGNC:10379]	12521	-0.856913328	-0.11737248
							HCLS1	hematopoietic cell-specific Lyn substrate 1 [Source:HGNC Symbol;Acc:HGNC:4844]	12537	-0.867826641	-0.1081735
							PTPRC	protein tyrosine phosphatase receptor type C [Source:HGNC Symbol;Acc:HGNC:9666]	12591	-0.892926693	-0.10160461
							ITGB2	integrin subunit beta 2 [Source:HGNC Symbol;Acc:HGNC:6155]	12598	-0.90079999	-0.09131831
							ICOSLG	inducible T cell costimulator ligand [Source:HGNC Symbol;Acc:HGNC:17087]	12617	-0.911503315	-0.08182943
							GZMA	granzyme A [Source:HGNC Symbol;Acc:HGNC:4708]	12651	-0.932073355	-0.07325152
							CCR1	C-C motif chemokine receptor 1 [Source:HGNC Symbol;Acc:HGNC:1602]	12661	-0.938040018	-0.062752135
							CD74	CD74 molecule [Source:HGNC Symbol;Acc:HGNC:1697]	12808	-1.037240028	-0.06163106
							IL2RB	interleukin 2 receptor subunit beta [Source:HGNC Symbol;Acc:HGNC:6009]	12876	-1.126933336	-0.053349204
							CXCL9	C-X-C motif chemokine ligand 9 [Source:HGNC Symbol;Acc:HGNC:7098]	12994	-1.330023289	-0.046498712
							PRF1	perforin 1 [Source:HGNC Symbol;Acc:HGNC:9360]	13005	-1.361283302	-0.031026036
							GZMB	granzyme B [Source:HGNC Symbol;Acc:HGNC:4709]	13066	-1.649469972	-0.015969299
							NCR1	natural cytotoxicity triggering receptor 1 [Source:HGNC Symbol;Acc:HGNC:6731]	13070	-1.693636656	0.004008877

							CBR1	carbonyl reductase 1 [Source:HGNC Symbol;Acc:HGNC:1548]	10454	-0.47832334	-0.2632747
							METAP1	methionyl aminopeptidase 1 [Source:HGNC Symbol;Acc:HGNC:15789]	10461	-0.479070008	-0.2564713
							ACAA1	acetyl-CoA acyltransferase 1 [Source:HGNC Symbol;Acc:HGNC:82]	10503	-0.484629989	-0.25229377
							ACOX1	acyl-CoA oxidase 1 [Source:HGNC Symbol;Acc:HGNC:119]	10529	-0.486626655	-0.24684699
							SDHA	succinate dehydrogenase complex flavoprotein subunit A [Source:HGNC Symbol;Acc:HGNC:10680]	10560	-0.49011001	-0.24173453
							CPOX	coproporphyrinogen oxidase [Source:HGNC Symbol;Acc:HGNC:2321]	10595	-0.494230002	-0.23686932
							HIBCH	3-hydroxyisobutyryl-CoA hydrolase [Source:HGNC Symbol;Acc:HGNC:4908]	10608	-0.495730013	-0.23027776
							CD1D	CD1d molecule [Source:HGNC Symbol;Acc:HGNC:1637]	10678	-0.502539992	-0.2279967
							IDH1	isocitrate dehydrogenase (NADP(+)) 1 [Source:HGNC Symbol;Acc:HGNC:5382]	10862	-0.522189975	-0.23424518
							DHCR24	24-dehydrocholesterol reductase [Source:HGNC Symbol;Acc:HGNC:2859]	10968	-0.534493327	-0.23426703
							ADH7	alcohol dehydrogenase 7 (class IV), mu or sigma polypeptide [Source:HGNC Symbol;Acc:HGNC:256]	11025	-0.541766644	-0.2303842
							GLUL	glutamate-ammonia ligase [Source:HGNC Symbol;Acc:HGNC:4341]	11047	-0.54387331	-0.22375919
							GPD1	glycerol-3-phosphate dehydrogenase 1 [Source:HGNC Symbol;Acc:HGNC:4455]	11176	-0.560663342	-0.22516502
							ENO2	enolase 2 [Source:HGNC Symbol;Acc:HGNC:3353]	11187	-0.562526643	-0.21740523
							REEP6	receptor accessory protein 6 [Source:HGNC Symbol;Acc:HGNC:30078]	11202	-0.56480664	-0.20992057
							LGALS1	galectin 1 [Source:HGNC Symbol;Acc:HGNC:6561]	11341	-0.585603356	-0.21172239
							SDHD	succinate dehydrogenase complex subunit D [Source:HGNC Symbol;Acc:HGNC:10683]	11364	-0.588620007	-0.20449595
							ECHS1	enoyl-CoA hydratase, short chain 1 [Source:HGNC Symbol;Acc:HGNC:3151]	11402	-0.592966676	-0.19836509
							ALDH9A1	aldehyde dehydrogenase 9 family member A1 [Source:HGNC Symbol;Acc:HGNC:412]	11410	-0.593946695	-0.18989632
							ACADL	acyl-CoA dehydrogenase long chain [Source:HGNC Symbol;Acc:HGNC:88]	11492	-0.606243312	-0.18697119
							ADIPOR2	adiponectin receptor 2 [Source:HGNC Symbol;Acc:HGNC:24041]	11669	-0.638066649	-0.19091964
							SERINC1	serine incorporator 1 [Source:HGNC Symbol;Acc:HGNC:13464]	11842	-0.671490014	-0.19405127
							EHHADH	enoyl-CoA hydratase and 3-hydroxyacyl CoA dehydrogenase [Source:HGNC Symbol;Acc:HGNC:3247]	12030	-0.716523349	-0.1976612
							ENO3	enolase 3 [Source:HGNC Symbol;Acc:HGNC:3354]	12095	-0.732990026	-0.1914968
							ECI2	enoyl-CoA delta isomerase 2 [Source:HGNC Symbol;Acc:HGNC:14601]	12193	-0.760309994	-0.18747328
							CD36	CD36 molecule [Source:HGNC Symbol;Acc:HGNC:1663]	12196	-0.760673344	-0.17608789
							EPHX1	epoxide hydrolase 1 [Source:HGNC Symbol;Acc:HGNC:3401]	12261	-0.782016695	-0.1691797
							HMGCL	3-hydroxy-3-methylglutaryl-CoA lyase [Source:HGNC Symbol;Acc:HGNC:5005]	12303	-0.796599984	-0.16026923
							RETSAT	retinol saturase [Source:HGNC Symbol;Acc:HGNC:25991]	12436	-0.843850017	-0.15768856
							ACSS1	acyl-CoA synthetase short chain family member 1 [Source:HGNC Symbol;Acc:HGNC:16091]	12455	-0.851783335	-0.14615989
							ALDH1A1	aldehyde dehydrogenase 1 family member A1 [Source:HGNC Symbol;Acc:HGNC:402]	12518	-0.889036655	-0.13747321
							D2HGDH	D-2-hydroxyglutarate dehydrogenase [Source:HGNC Symbol;Acc:HGNC:28358]	12556	-0.910223305	-0.12652922
							IDI1	isopentenyl-diphosphate delta isomerase 1 [Source:HGNC Symbol;Acc:HGNC:5387]	12680	-0.987006664	-0.12107977
							SUCLG1	succinate-CoA ligase GDP/ADP-forming subunit alpha [Source:HGNC Symbol;Acc:HGNC:11449]	12708	-1.002529979	-0.107961036
							HSPH1	heat shock protein family H (Hsp110) member 1 [Source:HGNC Symbol;Acc:HGNC:16969]	12793	-1.09310329	-0.09788201
							ALDH3A1	aldehyde dehydrogenase 3 family member A1 [Source:HGNC Symbol;Acc:HGNC:405]	12795	-1.102040052	-0.081240274
							ACSL1	acyl-CoA synthetase long chain family member 1 [Source:HGNC Symbol;Acc:HGNC:3569]	12838	-1.17513001	-0.06666453
							ADSL	adenylosuccinate lyase [Source:HGNC Symbol;Acc:HGNC:291]	12877	-1.215639949	-0.051164463
							CYP4A22	cytochrome P450 family 4 subfamily A member 22 [Source:HGNC Symbol;Acc:HGNC:20575]	12924	-1.339093328	-0.034410957
							CBR3	carbonyl reductase 3 [Source:HGNC Symbol;Acc:HGNC:1549]	12942	-1.401373386	-0.014466966
							FASN	fatty acid synthase [Source:HGNC Symbol;Acc:HGNC:3594]	12952	-1.428286672	0.006504813
APICAL SURFACE	33	-0.30815038	0.15324165	0.1600091	0.533	1731	THY1	Thy-1 cell surface antigen [Source:HGNC Symbol;Acc:HGNC:11801]	11307	-0.581216693	-0.28018275
							APP	amyloid beta precursor protein [Source:HGNC Symbol;Acc:HGNC:620]	11618	-0.627916694	-0.27380678
							ADIPOR2	adiponectin receptor 2 [Source:HGNC Symbol;Acc:HGNC:24041]	11669	-0.638066649	-0.24694857
							TMEM8B	transmembrane protein 8B [Source:HGNC Symbol;Acc:HGNC:21427]	11699	-0.643113315	-0.21823262
							MDGA1	MAM domain containing glycosylphosphatidylinositol anchor 1 [Source:HGNC Symbol;Acc:HGNC:19267]	11875	-0.678786695	-0.1990274
							GAS1	growth arrest specific 1 [Source:HGNC Symbol;Acc:HGNC:4165]	12108	-0.736490011	-0.18142883
							ATP6V0A4	ATPase H+ transporting V0 subunit a4 [Source:HGNC Symbol;Acc:HGNC:866]	12139	-0.745500028	-0.14786302
							SLC2A4	solute carrier family 2 member 4 [Source:HGNC Symbol;Acc:HGNC:11009]	12634	-0.953143358	-0.13998692
							IL2RB	interleukin 2 receptor subunit beta [Source:HGNC Symbol;Acc:HGNC:6009]	12809	-1.126933336	-0.0991404
							MAL	mal, T cell differentiation protein [Source:HGNC Symbol;Acc:HGNC:6817]	12814	-1.13411665	-0.044875324
							HSPB1	heat shock protein family B (small) member 1 [Source:HGNC Symbol;Acc:HGNC:5246]	12908	-1.285770059	0.009843133
INFLAMMATORY RESPONSE	156	-0.29793[illegible]	[illegible]	[illegible]	0.621	2549	CX3CL1	C-X3-C motif chemokine ligand 1 [Source:HGNC Symbol;Acc:HGNC:10647]	10489	-0.482749999	-0.29237294
							BDKRB1	bradykinin receptor B1 [Source:HGNC Symbol;Acc:HGNC:1029]	10492	-0.48312667	-0.28696182
							EBI3	Epstein-Barr virus induced 3 [Source:HGNC Symbol;Acc:HGNC:3129]	10494	-0.483213335	-0.28147212
							RASGRP1	RAS guanyl releasing protein 1 [Source:HGNC Symbol;Acc:HGNC:9878]	10576	-0.491793334	-0.2820942
							KCNMB2	potassium calcium-activated channel subfamily M regulatory beta subunit 2 [Source:HGNC Symbol;Acc:HGNC:6286]	10582	-0.492723346	-0.27680546
							OSM	oncostatin M [Source:HGNC Symbol;Acc:HGNC:8506]	10599	-0.495119989	-0.27234304
							ACVR2A	activin A receptor type 2A [Source:HGNC Symbol;Acc:HGNC:173]	10648	-0.499253333	-0.27031732
							ATP2C1	ATPase secretory pathway Ca2+ transporting 1 [Source:HGNC Symbol;Acc:HGNC:13211]	10709	-0.505393326	-0.2691524
							IL12B	interleukin 12B [Source:HGNC Symbol;Acc:HGNC:5970]	10800	-0.515919983	-0.27019525
							RELA	RELA proto-oncogene, NF-kB subunit [Source:HGNC Symbol;Acc:HGNC:9955]	10821	-0.517629981	-0.26578403
							KLF6	Kruppel like factor 6 [Source:HGNC Symbol;Acc:HGNC:2235]	10850	-0.520950019	-0.26195562
							IRF7	interferon regulatory factor 7 [Source:HGNC Symbol;Acc:HGNC:6122]	10940	-0.530796647	-0.26274943
							FZD5	frizzled class receptor 5 [Source:HGNC Symbol;Acc:HGNC:4043]	10942	-0.530913353	-0.25671014
							TLR2	toll like receptor 2 [Source:HGNC Symbol;Acc:HGNC:11848]	10966	-0.534143329	-0.25234154
							SLC7A2	solute carrier family 7 member 2 [Source:HGNC Symbol;Acc:HGNC:11060]	11006	-0.539099991	-0.24915801
							ICAM1	intercellular adhesion molecule 1 [Source:HGNC Symbol;Acc:HGNC:5344]	11020	-0.541023314	-0.24393381
							ACVR1B	activin A receptor type 1B [Source:HGNC Symbol;Acc:HGNC:172]	11031	-0.542333305	-0.23846164
							PSEN1	presenilin 1 [Source:HGNC Symbol;Acc:HGNC:9508]	11138	-0.556500018	-0.24027908
							CLEC5A	C-type lectin domain containing 5A [Source:HGNC Symbol;Acc:HGNC:2054]	11140	-0.556683362	-0.23394287
							OSMR	oncostatin M receptor [Source:HGNC Symbol;Acc:HGNC:8507]	11196	-0.564320028	-0.23171088
							IRAK2	interleukin 1 receptor associated kinase 2 [Source:HGNC Symbol;Acc:HGNC:6113]	11233	-0.570003331	-0.22793837
							TNFRSF1B	TNF receptor superfamily member 1B [Source:HGNC Symbol;Acc:HGNC:11917]	11252	-0.572976649	-0.22273421
							MET	MET proto-oncogene, receptor tyrosine kinase [Source:HGNC Symbol;Acc:HGNC:7029]	11262	-0.573976696	-0.21681981
							LCK	LCK proto-oncogene, Src family tyrosine kinase [Source:HGNC Symbol;Acc:HGNC:6524]	11325	-0.582599998	-0.21492065
							GNAI3	G protein subunit alpha i3 [Source:HGNC Symbol;Acc:HGNC:4387]	11327	-0.582729995	-0.20828433
							CCL2	C-C motif chemokine ligand 2 [Source:HGNC Symbol;Acc:HGNC:10618]	11345	-0.586263359	-0.20284946
							ABCA1	ATP binding cassette subfamily A member 1 [Source:HGNC Symbol;Acc:HGNC:29]	11395	-0.59187001	-0.19983426
							F3	coagulation factor III, tissue factor [Source:HGNC Symbol;Acc:HGNC:3541]	11396	-0.591936648	-0.19301423
							CD69	CD69 molecule [Source:HGNC Symbol;Acc:HGNC:1694]	11436	-0.598246694	-0.18914922
							ADORA2B	adenosine A2b receptor [Source:HGNC Symbol;Acc:HGNC:264]	11480	-0.604849994	-0.18551867
							SLC1A2	solute carrier family 1 member 2 [Source:HGNC Symbol;Acc:HGNC:10940]	11499	-0.607476652	-0.17991701
							TNFRSF9	TNF receptor superfamily member 9 [Source:HGNC Symbol;Acc:HGNC:11924]	11663	-0.637040019	-0.18523163
							GPR183	G protein-coupled receptor 183 [Source:HGNC Symbol;Acc:HGNC:3128]	11705	-0.644206643	-0.18099235
							LDLR	low density lipoprotein receptor [Source:HGNC Symbol;Acc:HGNC:6547]	11706	-0.644280016	-0.17356925
							RGS16	regulator of G protein signaling 16 [Source:HGNC Symbol;Acc:HGNC:9997]	11708	-0.644573331	-0.1662204
							CCL7	C-C motif chemokine ligand 7 [Source:HGNC Symbol;Acc:HGNC:10634]	11735	-0.649633348	-0.1607541
							EDN1	endothelin 1 [Source:HGNC Symbol;Acc:HGNC:3176]	11757	-0.654429972	-0.15484437
							RTP4	receptor transporter protein 4 [Source:HGNC Symbol;Acc:HGNC:23992]	11775	-0.657199979	-0.14859219
							SCN1B	sodium voltage-gated channel beta subunit 1 [Source:HGNC Symbol;Acc:HGNC:10586]	11784	-0.66030997	-0.14160547
							CD48	CD48 molecule [Source:HGNC Symbol;Acc:HGNC:1683]	11799	-0.662580013	-0.13505839
							CMKLR1	chemerin chemokine-like receptor 1 [Source:HGNC Symbol;Acc:HGNC:2121]	11809	-0.664596677	-0.12809992
							IL7R	interleukin 7 receptor [Source:HGNC Symbol;Acc:HGNC:6024]	11811	-0.66470331	-0.12051915
							TNFAIP6	TNF alpha induced protein 6 [Source:HGNC Symbol;Acc:HGNC:11898]	11965	-0.700743318	-0.124323465
							PCDH7	protocadherin 7 [Source:HGNC Symbol;Acc:HGNC:8659]	12024	-0.715433359	-0.120583326
							GPR132	G protein-coupled receptor 132 [Source:HGNC Symbol;Acc:HGNC:17482]	12035	-0.718116641	-0.11308585
							KCNJ2	potassium inwardly rectifying channel subfamily J member 2 [Source:HGNC Symbol;Acc:HGNC:6263]	12130	-0.744173348	-0.111809395
							ITGA5	integrin subunit alpha 5 [Source:HGNC Symbol;Acc:HGNC:6141]	12140	-0.746190012	-0.10391084
							CCL5	C-C motif chemokine ligand 5 [Source:HGNC Symbol;Acc:HGNC:10632]	12143	-0.746720016	-0.09546274
							SLC31A1	solute carrier family 31 member 1 [Source:HGNC Symbol;Acc:HGNC:11016]	12207	-0.766009986	-0.09152805
							AXL	AXL receptor tyrosine kinase [Source:HGNC Symbol;Acc:HGNC:905]	12218	-0.768093348	-0.083454765
							SLC11A2	solute carrier family 11 member 2 [Source:HGNC Symbol;Acc:HGNC:10908]	12286	-0.791100025	-0.079541534
							NLRP3	NLR family pyrin domain containing 3 [Source:HGNC Symbol;Acc:HGNC:16400]	12386	-0.822486639	-0.07775096
							BTG2	BTG anti-proliferation factor 2 [Source:HGNC Symbol;Acc:HGNC:1131]	12547	-0.905489981	-0.07973971
							ADGRE1	adhesion G protein-coupled receptor E1 [Source:HGNC Symbol;Acc:HGNC:3336]	12569	-0.919470012	-0.070776306
							IL10RA	interleukin 10 receptor subunit alpha [Source:HGNC Symbol;Acc:HGNC:5964]	12574	-0.921263337	-0.06047247
							IFITM1	interferon induced transmembrane protein 1 [Source:HGNC Symbol;Acc:HGNC:5412]	12674	-0.983173311	-0.056830537
							SELE	selectin E [Source:HGNC Symbol;Acc:HGNC:10718]	12728	-1.018596649	-0.04920932
							IL2RB	interleukin 2 receptor subunit beta [Source:HGNC Symbol;Acc:HGNC:6009]	12809	-1.126933336	-0.042436007
							SELL	selectin L [Source:HGNC Symbol;Acc:HGNC:10720]	12870	-1.203663349	-0.033225972
							PDE4B	phosphodiesterase 4B [Source:HGNC Symbol;Acc:HGNC:8781]	12911	-1.308416724	-0.021256343
							CXCL9	C-X-C motif chemokine ligand 9 [Source:HGNC Symbol;Acc:HGNC:7098]	12919	-1.330023289	-0.006475859
							ATP2A2	ATPase sarcoplasmic/endoplasmic reticulum Ca2+ transporting 2 [Source:HGNC Symbol;Acc:HGNC:812]	12927	-1.343680024	0.008461971
ALLOGRAFT REJECTION	151	-0.29441887	0.00182149	0.1646998	0.65	2098	IRF7	interferon regulatory factor 7 [Source:HGNC Symbol;Acc:HGNC:6122]	10940	-0.530796647	-0.28788748
							TLR2	toll like receptor 2 [Source:HGNC Symbol;Acc:HGNC:11848]	10966	-0.534143329	-0.28325504
							ICAM1	intercellular adhesion molecule 1 [Source:HGNC Symbol;Acc:HGNC:5344]	11020	-0.541023314	-0.28071082
							BCL3	BCL3 transcription coactivator [Source:HGNC Symbol;Acc:HGNC:998]	11143	-0.556949973	-0.28332528
							DYRK3	dual specificity tyrosine phosphorylation regulated kinase 3 [Source:HGNC Symbol;Acc:HGNC:3094]	11153	-0.558030009	-0.27715725
							CTSS	cathepsin S [Source:HGNC Symbol;Acc:HGNC:2545]	11285	-0.578629971	-0.28020337
							THY1	Thy-1 cell surface antigen [Source:HGNC Symbol;Acc:HGNC:11801]	11307	-0.581216693	-0.27468127
							ZAP70	zeta chain of T cell receptor associated protein kinase 70 [Source:HGNC Symbol;Acc:HGNC:12858]	11311	-0.581623316	-0.2677573
							LCK	LCK proto-oncogene, Src family tyrosine kinase [Source:HGNC Symbol;Acc:HGNC:6524]	11325	-0.582599998	-0.26159734
							CCL2	C-C motif chemokine ligand 2 [Source:HGNC Symbol;Acc:HGNC:10618]	11345	-0.586263359	-0.2558579
							CCND2	cyclin D2 [Source:HGNC Symbol;Acc:HGNC:1583]	11465	-0.602416694	-0.25768012
							CD247	CD247 molecule [Source:HGNC Symbol;Acc:HGNC:1677]	11608	-0.625753343	-0.26100004
							FASLG	Fas ligand [Source:HGNC Symbol;Acc:HGNC:11936]	11727	-0.647996664	-0.26218376
							CCL7	C-C motif chemokine ligand 7 [Source:HGNC Symbol;Acc:HGNC:10634]	11735	-0.649633348	-0.25473335
							ITK	IL2 inducible T cell kinase [Source:HGNC Symbol;Acc:HGNC:6171]	11783	-0.660243332	-0.25025654
							CD28	CD28 molecule [Source:HGNC Symbol;Acc:HGNC:1653]	11792	-0.66111666	-0.24274242
							CD3E	CD3e molecule [Source:HGNC Symbol;Acc:HGNC:1674]	11804	-0.663453341	-0.23543237
							NOS2	nitric oxide synthase 2 [Source:HGNC Symbol;Acc:HGNC:7873]	11822	-0.666809976	-0.22854665
							ITGAL	integrin subunit alpha L [Source:HGNC Symbol;Acc:HGNC:6148]	11833	-0.669430017	-0.22108544
							RPL3L	ribosomal protein L3 like [Source:HGNC Symbol;Acc:HGNC:10351]	11873	-0.677779973	-0.21577202
							C2	complement C2 [Source:HGNC Symbol;Acc:HGNC:1248]	11963	-0.700576663	-0.21405825
							CAPG	capping actin protein, gelsolin like [Source:HGNC Symbol;Acc:HGNC:1474]	11970	-0.701780021	-0.20588858
							PRKCB	protein kinase C beta [Source:HGNC Symbol;Acc:HGNC:9395]	12040	-0.719506681	-0.20238982
							LY86	lymphocyte antigen 86 [Source:HGNC Symbol;Acc:HGNC:16837]	12083	-0.728389978	-0.19668645
							CCL5	C-C motif chemokine ligand 5 [Source:HGNC Symbol;Acc:HGNC:10632]	12143	-0.746720016	-0.19207679
							F2R	coagulation factor II thrombin receptor [Source:HGNC Symbol;Acc:HGNC:3537]	12217	-0.767446637	-0.18829854
							GBP2	guanylate binding protein 2 [Source:HGNC Symbol;Acc:HGNC:4183]	12305	-0.796963334	-0.18524355
							B2M	beta-2-microglobulin [Source:HGNC Symbol;Acc:HGNC:914]	12345	-0.809759974	-0.17830612
							FYB1	FYN binding protein 1 [Source:HGNC Symbol;Acc:HGNC:4036]	12352	-0.81262666	-0.1687725
							NLRP3	NLR family pyrin domain containing 3 [Source:HGNC Symbol;Acc:HGNC:16400]	12386	-0.822486639	-0.16121285
							RPL9	ribosomal protein L9 [Source:HGNC Symbol;Acc:HGNC:10369]	12428	-0.839636683	-0.154063
							MRPL3	mitochondrial ribosomal protein L3 [Source:HGNC Symbol;Acc:HGNC:10379]	12464	-0.856913328	-0.14623494
							HCLS1	hematopoietic cell-specific Lyn substrate 1 [Source:HGNC Symbol;Acc:HGNC:4844]	12479	-0.867826641	-0.13664293
							PTPRC	protein tyrosine phosphatase receptor type C [Source:HGNC Symbol;Acc:HGNC:9666]	12533	-0.892926693	-0.1297686
							ITGB2	integrin subunit beta 2 [Source:HGNC Symbol;Acc:HGNC:6155]	12540	-0.90079999	-0.11915002
							TPD52	tumor protein D52 [Source:HGNC Symbol;Acc:HGNC:12005]	12554	-0.907633305	-0.10899059
							STAT1	signal transducer and activator of transcription 1 [Source:HGNC Symbol;Acc:HGNC:11362]	12589	-0.930766642	-0.10017617
							GZMA	granzyme A [Source:HGNC Symbol;Acc:HGNC:4708]	12593	-0.932073355	-0.08893997
							CCR1	C-C motif chemokine receptor 1 [Source:HGNC Symbol;Acc:HGNC:1602]	12604	-0.938040018	-0.07817357
							CD74	CD74 molecule [Source:HGNC Symbol;Acc:HGNC:1697]	12742	-1.037240028	-0.07604219
							IL2RB	interleukin 2 receptor subunit beta [Source:HGNC Symbol;Acc:HGNC:6009]	12809	-1.126933336	-0.067297295
							PTPN6	protein tyrosine phosphatase non-receptor type 6 [Source:HGNC Symbol;Acc:HGNC:9658]	12835	-1.167333364	-0.054873534
							TRAT1	T cell receptor associated transmembrane adaptor 1 [Source:HGNC Symbol;Acc:HGNC:30698]	12914	-1.316996694	-0.044721182
							CXCL9	C-X-C motif chemokine ligand 9 [Source:HGNC Symbol;Acc:HGNC:7098]	12919	-1.330023289	-0.02866587
							PRF1	perforin 1 [Source:HGNC Symbol;Acc:HGNC:9360]	12930	-1.361283302	-0.01269153
							NCR1	natural cytotoxicity triggering receptor 1 [Source:HGNC Symbol;Acc:HGNC:6731]	12995	-1.693636656	0.003181768
							ALDH1A3	aldehyde dehydrogenase 1 family member A3 [Source:HGNC Symbol;Acc:HGNC:409]	8514	-0.311340004	-0.27190375

							HLA-DRA	major histocompatibility complex, class II, DR alpha [Source:HGNC Symbol;Acc:HGNC:4947]	13800	-7.642416477	6.62E-08
**KRAS SIGNALING UP**											

KRAS SIGNALING UP	163	-0.27986336	0.00408163	0.1717892	0.793	2261	LAPTM5	lysosomal protein transmembrane 5 [Source:HGNC Symbol;Acc:HGNC:29612]	10863	-0.516906679	-0.2747164
							TMEM176A	transmembrane protein 176A [Source:HGNC Symbol;Acc:HGNC:24930]	10888	-0.520089984	-0.2713896
							SPRY2	sprouty RTK signaling antagonist 2 [Source:HGNC Symbol;Acc:HGNC:11270]	10889	-0.520353317	-0.26620835
							AKAP12	A-kinase anchoring protein 12 [Source:HGNC Symbol;Acc:HGNC:370]	11027	-0.535669982	-0.27144557
							IGFBP3	insulin like growth factor binding protein 3 [Source:HGNC Symbol;Acc:HGNC:5472]	11045	-0.53840667	-0.26739627
							ETV4	ETS variant transcription factor 4 [Source:HGNC Symbol;Acc:HGNC:3493]	11087	-0.543200016	-0.26515108
							TRIB2	tribbles pseudokinase 2 [Source:HGNC Symbol;Acc:HGNC:30809]	11124	-0.546686649	-0.26248538
							LY96	lymphocyte antigen 96 [Source:HGNC Symbol;Acc:HGNC:17156]	11142	-0.549326658	-0.25832736
							TMEM100	transmembrane protein 100 [Source:HGNC Symbol;Acc:HGNC:25607]	11197	-0.55769664	-0.25694093
							GALNT3	polypeptide N-acetylgalactosaminyltransferase 3 [Source:HGNC Symbol;Acc:HGNC:4125]	11253	-0.565366685	-0.25555527
							BTBD3	BTB domain containing 3 [Source:HGNC Symbol;Acc:HGNC:15854]	11279	-0.569809973	-0.25181058
							TNFRSF1B	TNF receptor superfamily member 1B [Source:HGNC Symbol;Acc:HGNC:11917]	11299	-0.572976649	-0.24757136
							DOCK2	dedicator of cytokinesis 2 [Source:HGNC Symbol;Acc:HGNC:2988]	11301	-0.57311666	-0.24194188
							CTSS	cathepsin S [Source:HGNC Symbol;Acc:HGNC:2545]	11332	-0.578629971	-0.23849516
							ANKH	ANKH inorganic pyrophosphate transport regulator [Source:HGNC Symbol;Acc:HGNC:15492]	11352	-0.581049979	-0.23417558
							BIRC3	baculoviral IAP repeat containing 3 [Source:HGNC Symbol;Acc:HGNC:591]	11376	-0.583530009	-0.23013994
							F2RL1	F2R like trypsin receptor 1 [Source:HGNC Symbol;Acc:HGNC:3538]	11417	-0.588776648	-0.22736378
							USP12	ubiquitin specific peptidase 12 [Source:HGNC Symbol;Acc:HGNC:20485]	11431	-0.589913309	-0.22249298
							CCND2	cyclin D2 [Source:HGNC Symbol;Acc:HGNC:1583]	11512	-0.602416694	-0.22266743
							TRIB1	tribbles pseudokinase 1 [Source:HGNC Symbol;Acc:HGNC:16891]	11605	-0.618019998	-0.22361244
							SPON1	spondin 1 [Source:HGNC Symbol;Acc:HGNC:11252]	11617	-0.619910002	-0.21828863
							ENG	endoglin [Source:HGNC Symbol;Acc:HGNC:3349]	11687	-0.631760001	-0.21732214
							PTBP2	polypyrimidine tract binding protein 2 [Source:HGNC Symbol;Acc:HGNC:17662]	11741	-0.642313361	-0.21501599
							RGS16	regulator of G protein signaling 16 [Source:HGNC Symbol;Acc:HGNC:9997]	11755	-0.644573331	-0.20960093
							SCN1B	sodium voltage-gated channel beta subunit 1 [Source:HGNC Symbol;Acc:HGNC:10586]	11836	-0.66030997	-0.20919892
							CMKLR1	chemerin chemokine-like receptor 1 [Source:HGNC Symbol;Acc:HGNC:2121]	11861	-0.664596677	-0.20443325
							IL7R	interleukin 7 receptor [Source:HGNC Symbol;Acc:HGNC:6024]	11863	-0.66470331	-0.19789182
							CFB	complement factor B [Source:HGNC Symbol;Acc:HGNC:1037]	11903	-0.672839999	-0.19420147
							MPZL2	myelin protein zero like 2 [Source:HGNC Symbol;Acc:HGNC:3496]	11913	-0.676046669	-0.18816438
							RBM4	RNA binding motif protein 4 [Source:HGNC Symbol;Acc:HGNC:9901]	11948	-0.68378669	-0.18397923
							KCNN4	potassium calcium-activated channel subfamily N member 4 [Source:HGNC Symbol;Acc:HGNC:6293]	12011	-0.698050022	-0.18181254
							ADGRA2	adhesion G protein-coupled receptor A2 [Source:HGNC Symbol;Acc:HGNC:17849]	12053	-0.708230019	-0.17792413
							ADGRL4	adhesion G protein-coupled receptor L4 [Source:HGNC Symbol;Acc:HGNC:20822]	12075	-0.714940012	-0.1724257
							DNMBP	dynamin binding protein [Source:HGNC Symbol;Acc:HGNC:30373]	12087	-0.717069983	-0.16613445
							HKDC1	hexokinase domain containing 1 [Source:HGNC Symbol;Acc:HGNC:23302]	12205	-0.749279976	-0.16770148
							LCP1	lymphocyte cytosolic protein 1 [Source:HGNC Symbol;Acc:HGNC:6528]	12338	-0.788820028	-0.17003222
							YRDC	yrdC N6-threonylcarbamoyltransferase domain containing [Source:HGNC Symbol;Acc:HGNC:28905]	12351	-0.795023322	-0.16304193
							ALDH1A2	aldehyde dehydrogenase 1 family member A2 [Source:HGNC Symbol;Acc:HGNC:15472]	12364	-0.798510015	-0.15601693
							ITGA2	integrin subunit alpha 2 [Source:HGNC Symbol;Acc:HGNC:6137]	12375	-0.801103354	-0.14881179
							CFH	complement factor H [Source:HGNC Symbol;Acc:HGNC:4883]	12398	-0.807113349	-0.14247271
							MALL	mal, T cell differentiation protein like [Source:HGNC Symbol;Acc:HGNC:6818]	12406	-0.810386658	-0.13494365
							IKZF1	IKAROS family zinc finger 1 [Source:HGNC Symbol;Acc:HGNC:13176]	12419	-0.814866662	-0.12775578
							CDADC1	cytidine and dCMP deaminase domain containing 1 [Source:HGNC Symbol;Acc:HGNC:20299]	12467	-0.832913339	-0.12308882
							ACE	angiotensin I converting enzyme [Source:HGNC Symbol;Acc:HGNC:2707]	12523	-0.858659983	-0.118782796
							CD37	CD37 molecule [Source:HGNC Symbol;Acc:HGNC:1666]	12533	-0.864106655	-0.11087315
							ITGB2	integrin subunit beta 2 [Source:HGNC Symbol;Acc:HGNC:6155]	12598	-0.90079999	-0.10684197
							IL10RA	interleukin 10 receptor subunit alpha [Source:HGNC Symbol;Acc:HGNC:5964]	12633	-0.921263337	-0.10029221
							ADAM8	ADAM metallopeptidase domain 8 [Source:HGNC Symbol;Acc:HGNC:215]	12696	-0.953986645	-0.09557711
							PLAT	plasminogen activator, tissue type [Source:HGNC Symbol;Acc:HGNC:9051]	12717	-0.966520011	-0.08749648
							GPRC5B	G protein-coupled receptor class C group 5 member B [Source:HGNC Symbol;Acc:HGNC:13308]	12807	-1.03695333	-0.08403861
							GFPT2	glutamine-fructose-6-phosphate transaminase 2 [Source:HGNC Symbol;Acc:HGNC:4242]	12814	-1.040956616	-0.07413655
							ANO1	anoctamin 1 [Source:HGNC Symbol;Acc:HGNC:21625]	12848	-1.084030032	-0.06588893
							PIGR	polymeric immunoglobulin receptor [Source:HGNC Symbol;Acc:HGNC:8968]	12849	-1.084843278	-0.055086914
							F13A1	coagulation factor XIII A chain [Source:HGNC Symbol;Acc:HGNC:3531]	12908	-1.181319952	-0.047799572
							RETN	resistin [Source:HGNC Symbol;Acc:HGNC:20389]	12986	-1.311153293	-0.040685505
							SPARCL1	SPARC like 1 [Source:HGNC Symbol;Acc:HGNC:11220]	12988	-1.318863392	-0.027630465
							KLF4	Kruppel like factor 4 [Source:HGNC Symbol;Acc:HGNC:6348]	13074	-1.718729973	-0.017075352
							TSPAN1	tetraspanin 1 [Source:HGNC Symbol;Acc:HGNC:20657]	13098	-2.079050064	0.001851494

KRAS SIGNALING UP	153	-0.28798077	0.00186567	0.1673344	0.691	2219	LAPTM5	lysosomal protein transmembrane 5 [Source:HGNC Symbol;Acc:HGNC:29612]	10819	-0.516906679	-0.28203896
							TMEM176A	transmembrane protein 176A [Source:HGNC Symbol;Acc:HGNC:24930]	10844	-0.520089984	-0.27792332
							SPRY2	sprouty RTK signaling antagonist 2 [Source:HGNC Symbol;Acc:HGNC:11270]	10845	-0.520353317	-0.2719419
							AKAP12	A-kinase anchoring protein 12 [Source:HGNC Symbol;Acc:HGNC:370]	10981	-0.535669982	-0.2762625
							IGFBP3	insulin like growth factor binding protein 3 [Source:HGNC Symbol;Acc:HGNC:5472]	10999	-0.53840667	-0.27139303
							ETV4	ETS variant transcription factor 4 [Source:HGNC Symbol;Acc:HGNC:3493]	11041	-0.543200016	-0.2683312
							TRIB2	tribbles pseudokinase 2 [Source:HGNC Symbol;Acc:HGNC:30809]	11078	-0.546686649	-0.26484123
							LY96	lymphocyte antigen 96 [Source:HGNC Symbol;Acc:HGNC:17156]	11095	-0.549326658	-0.2597686
							TMEM100	transmembrane protein 100 [Source:HGNC Symbol;Acc:HGNC:25607]	11150	-0.55769664	-0.25754917
							GALNT3	polypeptide N-acetylgalactosaminyltransferase 3 [Source:HGNC Symbol;Acc:HGNC:4125]	11206	-0.565366685	-0.25531915
							BTBD3	BTB domain containing 3 [Source:HGNC Symbol;Acc:HGNC:15854]	11232	-0.569809973	-0.25070962
							TNFRSF1B	TNF receptor superfamily member 1B [Source:HGNC Symbol;Acc:HGNC:11917]	11252	-0.572976649	-0.24559799
							DOCK2	dedicator of cytokinesis 2 [Source:HGNC Symbol;Acc:HGNC:2988]	11254	-0.57311666	-0.23908766
							CTSS	cathepsin S [Source:HGNC Symbol;Acc:HGNC:2545]	11285	-0.578629971	-0.2347648
							ANKH	ANKH inorganic pyrophosphate transport regulator [Source:HGNC Symbol;Acc:HGNC:15492]	11305	-0.581049979	-0.22956036
							BIRC3	baculoviral IAP repeat containing 3 [Source:HGNC Symbol;Acc:HGNC:591]	11329	-0.583530009	-0.22463788
							F2RL1	F2R like trypsin receptor 1 [Source:HGNC Symbol;Acc:HGNC:3538]	11369	-0.588776648	-0.22089694
							USP12	ubiquitin specific peptidase 12 [Source:HGNC Symbol;Acc:HGNC:20485]	11383	-0.589913309	-0.21512492
							CCND2	cyclin D2 [Source:HGNC Symbol;Acc:HGNC:1583]	11465	-0.602416694	-0.21448705
							SPON1	spondin 1 [Source:HGNC Symbol;Acc:HGNC:11252]	11570	-0.619910002	-0.21543324
							ENG	endoglin [Source:HGNC Symbol;Acc:HGNC:3349]	11641	-0.631760001	-0.21360429
							PTBP2	polypyrimidine tract binding protein 2 [Source:HGNC Symbol;Acc:HGNC:17662]	11695	-0.642313361	-0.21033457
							RGS16	regulator of G protein signaling 16 [Source:HGNC Symbol;Acc:HGNC:9997]	11708	-0.644573331	-0.20385662
							SCN1B	sodium voltage-gated channel beta subunit 1 [Source:HGNC Symbol;Acc:HGNC:10586]	11784	-0.66030997	-0.20208757
							CMKLR1	chemerin chemokine-like receptor 1 [Source:HGNC Symbol;Acc:HGNC:2121]	11809	-0.664596677	-0.19631083
							IL7R	interleukin 7 receptor [Source:HGNC Symbol;Acc:HGNC:6024]	11811	-0.66470331	-0.18874773
							CFB	complement factor B [Source:HGNC Symbol;Acc:HGNC:1037]	11849	-0.672839999	-0.18388525
							MPZL2	myelin protein zero like 2 [Source:HGNC Symbol;Acc:HGNC:3496]	11859	-0.676046669	-0.17681268
							RBM4	RNA binding motif protein 4 [Source:HGNC Symbol;Acc:HGNC:9901]	11893	-0.68378669	-0.1715139
							KCNN4	potassium calcium-activated channel subfamily N member 4 [Source:HGNC Symbol;Acc:HGNC:6293]	11954	-0.698050022	-0.16814679
							ADGRA2	adhesion G protein-coupled receptor A2 [Source:HGNC Symbol;Acc:HGNC:17849]	11998	-0.708230019	-0.1633432
							ADGRL4	adhesion G protein-coupled receptor L4 [Source:HGNC Symbol;Acc:HGNC:20822]	12020	-0.714940012	-0.15675493
							DNMBP	dynamin binding protein [Source:HGNC Symbol;Acc:HGNC:30373]	12033	-0.717069983	-0.14944364
							HKDC1	hexokinase domain containing 1 [Source:HGNC Symbol;Acc:HGNC:23302]	12153	-0.749279976	-0.15006697
							LCP1	lymphocyte cytosolic protein 1 [Source:HGNC Symbol;Acc:HGNC:6528]	12283	-0.788820028	-0.15101194
							YRDC	yrdC N6-threonylcarbamoyltransferase domain containing [Source:HGNC Symbol;Acc:HGNC:28905]	12295	-0.795023322	-0.14272697
							ALDH1A2	aldehyde dehydrogenase 1 family member A2 [Source:HGNC Symbol;Acc:HGNC:15472]	12308	-0.798510015	-0.13447954
							ITGA2	integrin subunit alpha 2 [Source:HGNC Symbol;Acc:HGNC:6137]	12319	-0.801103354	-0.12604706
							MALL	mal, T cell differentiation protein like [Source:HGNC Symbol;Acc:HGNC:6818]	12348	-0.810386658	-0.11890494
							IKZF1	IKAROS family zinc finger 1 [Source:HGNC Symbol;Acc:HGNC:13176]	12362	-0.814866662	-0.1105471
							CDADC1	cytidine and dCMP deaminase domain containing 1 [Source:HGNC Symbol;Acc:HGNC:20299]	12410	-0.832913339	-0.10462075
							ACE	angiotensin I converting enzyme [Source:HGNC Symbol;Acc:HGNC:2707]	12466	-0.858659983	-0.099019356
							CD37	CD37 molecule [Source:HGNC Symbol;Acc:HGNC:1666]	12475	-0.864106655	-0.08970743
							ITGB2	integrin subunit beta 2 [Source:HGNC Symbol;Acc:HGNC:6155]	12540	-0.90079999	-0.08432018
							IL10RA	interleukin 10 receptor subunit alpha [Source:HGNC Symbol;Acc:HGNC:5964]	12574	-0.921263337	-0.07629163
							ADAM8	ADAM metallopeptidase domain 8 [Source:HGNC Symbol;Acc:HGNC:215]	12636	-0.953986645	-0.07006016
							IL33	interleukin 33 [Source:HGNC Symbol;Acc:HGNC:16028]	12658	-0.967096686	-0.060573366
							GFPT2	glutamine-fructose-6-phosphate transaminase 2 [Source:HGNC Symbol;Acc:HGNC:4242]	12748	-1.040956616	-0.05551542
							ANO1	anoctamin 1 [Source:HGNC Symbol;Acc:HGNC:21625]	12783	-1.084030032	-0.04569349
							PIGR	polymeric immunoglobulin receptor [Source:HGNC Symbol;Acc:HGNC:8968]	12784	-1.084843278	-0.03322328
							F13A1	coagulation factor XIII A chain [Source:HGNC Symbol;Acc:HGNC:3531]	12842	-1.181319952	-0.024068164
							RETN	resistin [Source:HGNC Symbol;Acc:HGNC:20389]	12913	-1.311153293	-0.014429625
							TSPAN1	tetraspanin 1 [Source:HGNC Symbol;Acc:HGNC:20657]	13017	-2.079050064	0.001474526

**PROTEIN SECRETION**	86	-0.27819422	0.05148515	0.18844151	0.795	5000	YKT6	YKT6 v-SNARE homolog [Source:HGNC Symbol;Acc:HGNC:16959]	8802	-0.300020009	-0.27070114
							CLTA	clathrin light chain A [Source:HGNC Symbol;Acc:HGNC:2090]	8821	-0.302210003	-0.26446578
							VPS4B	vacuolar protein sorting 4 homolog B [Source:HGNC Symbol;Acc:HGNC:10895]	8884	-0.308429986	-0.26128325
							LMAN1	lectin, mannose binding 1 [Source:HGNC Symbol;Acc:HGNC:6631]	8960	-0.314173341	-0.25890517
							NAPA	NSF attachment protein alpha [Source:HGNC Symbol;Acc:HGNC:7641]	9038	-0.320593327	-0.25651255
							RAB22A	RAB22A, member RAS oncogene family [Source:HGNC Symbol;Acc:HGNC:9764]	9043	-0.320926666	-0.24878895
							SEC22B	SEC22 homolog B, vesicle trafficking protein [Source:HGNC Symbol;Acc:HGNC:10700]	9107	-0.325473338	-0.24525368
							MON2	MON2 homolog, regulator of endosome-to-Golgi trafficking [Source:HGNC Symbol;Acc:HGNC:29177]	9136	-0.327946663	-0.23910467
							CAV2	caveolin 2 [Source:HGNC Symbol;Acc:HGNC:1528]	9211	-0.334813327	-0.23613818
							BNIP3	BCL2 interacting protein 3 [Source:HGNC Symbol;Acc:HGNC:1084]	9249	-0.337833345	-0.23039846
							ARFGEF1	ADP ribosylation factor guanine nucleotide exchange factor 1 [Source:HGNC Symbol;Acc:HGNC:15772]	9370	-0.348003328	-0.23045653
							GALC	galactosylceramidase [Source:HGNC Symbol;Acc:HGNC:4115]	9389	-0.349556655	-0.22303869
							CLTC	clathrin heavy chain [Source:HGNC Symbol;Acc:HGNC:2092]	9426	-0.352356672	-0.21686333
							TMED10	transmembrane p24 trafficking protein 10 [Source:HGNC Symbol;Acc:HGNC:16998]	9573	-0.365869999	-0.21837091
							AP2B1	adaptor related protein complex 2 subunit beta 1 [Source:HGNC Symbol;Acc:HGNC:563]	9620	-0.369643331	-0.21249296
							ARFGEF2	ADP ribosylation factor guanine nucleotide exchange factor 2 [Source:HGNC Symbol;Acc:HGNC:15853]	9773	-0.381786674	-0.21404047
							STX12	syntaxin 12 [Source:HGNC Symbol;Acc:HGNC:11430]	10092	-0.405783325	-0.22709222
							SCAMP3	secretory carrier membrane protein 3 [Source:HGNC Symbol;Acc:HGNC:10565]	10144	-0.410103321	-0.22056833
							ARCN1	archain 1 [Source:HGNC Symbol;Acc:HGNC:649]	10293	-0.423036665	-0.22079396
							ARFIP1	ADP ribosylation factor interacting protein 1 [Source:HGNC Symbol;Acc:HGNC:21496]	10596	-0.449829996	-0.23157902
							TMX1	thioredoxin related transmembrane protein 1 [Source:HGNC Symbol;Acc:HGNC:15487]	10720	-0.46323666	-0.22897784
							ADAM10	ADAM metallopeptidase domain 10 [Source:HGNC Symbol;Acc:HGNC:188]	10964	-0.489833325	-0.23446193
							COG2	component of oligomeric golgi complex 2 [Source:HGNC Symbol;Acc:HGNC:6546]	11017	-0.49617666	-0.22586125
							AP2M1	adaptor related protein complex 2 subunit mu 1 [Source:HGNC Symbol;Acc:HGNC:564]	11300	-0.525129974	-0.2333074
							SNX2	sorting nexin 2 [Source:HGNC Symbol;Acc:HGNC:11173]	11384	-0.534953356	-0.22599857
							TOM1L1	target of myb1 like 1 membrane trafficking protein [Source:HGNC Symbol;Acc:HGNC:11983]	11407	-0.537186682	-0.21418627
							MAPK1	mitogen-activated protein kinase 1 [Source:HGNC Symbol;Acc:HGNC:6871]	11550	-0.555410028	-0.21066837
							COPB1	COPI coat complex subunit beta 1 [Source:HGNC Symbol;Acc:HGNC:2231]	11596	-0.561179996	-0.19993381
							KRT18	keratin 18 [Source:HGNC Symbol;Acc:HGNC:6430]	11793	-0.588786662	-0.19951962
							ABCA1	ATP binding cassette subfamily A member 1 [Source:HGNC Symbol;Acc:HGNC:29]	11819	-0.59187001	-0.18656032
							ARF1	ADP ribosylation factor 1 [Source:HGNC Symbol;Acc:HGNC:652]	12036	-0.625419974	-0.18668947
							PAM	peptidylglycine alpha-amidating monooxygenase [Source:HGNC Symbol;Acc:HGNC:8596]	12113	-0.638463318	-0.17628503
							IGF2R	insulin like growth factor 2 receptor [Source:HGNC Symbol;Acc:HGNC:5467]	12239	-0.66099	-0.16889073
							GBF1	golgi brefeldin A resistant guanine nucleotide exchange factor 1 [Source:HGNC Symbol;Acc:HGNC:4181]	12545	-0.724810004	-0.17302682
							SH3GL2	SH3 domain containing GRB2 like 2, endophilin A1 [Source:HGNC Symbol;Acc:HGNC:10831]	12649	-0.751066685	-0.16177873
							ATP1A1	ATPase Na+/K+ transporting subunit alpha 1 [Source:HGNC Symbol;Acc:HGNC:799]	12904	-0.822340012	-0.15976043
							SGMS1	sphingomyelin synthase 1 [Source:HGNC Symbol;Acc:HGNC:29799]	13060	-0.888493359	-0.14887156
							SNAP23	synaptosome associated protein 23 [Source:HGNC Symbol;Acc:HGNC:11131]	13096	-0.905903339	-0.1287983
							TPD52	tumor protein D52 [Source:HGNC Symbol;Acc:HGNC:12005]	13102	-0.907633305	-0.10649448
							CLN5	CLN5 intracellular trafficking protein [Source:HGNC Symbol;Acc:HGNC:2076]	13176	-0.940793335	-0.08832053
							SSPN	sarcospan [Source:HGNC Symbol;Acc:HGNC:11322]	13251	-0.975099981	-0.069362685
							DST	dystonin [Source:HGNC Symbol;Acc:HGNC:1090]	13263	-0.983423352	-0.045603454
							CTSC	cathepsin C [Source:HGNC Symbol;Acc:HGNC:2528]	13270	-0.987233341	-0.021384496
							ZW10	zw10 kinetochore protein [Source:HGNC Symbol;Acc:HGNC:13194]	13325	-1.020736694	0.000171397
							ATP6V1B1	ATPase H+ transporting V1 subunit B1 [Source:HGNC Symbol;Acc:HGNC:853]	13614	-1.376946688	0.013562125

PROTEIN SECRETION	83	-0.27376956	0.03921569	0.1779831	0.837	4688	YKT6	YKT6 v-SNARE homolog [Source:HGNC Symbol;Acc:HGNC:16959]	8436	-0.300020009	-0.26566908
							CLTA	clathrin light chain A [Source:HGNC Symbol;Acc:HGNC:2090]	8455	-0.302210003	-0.25888985
							VPS4B	vacuolar protein sorting 4 homolog B [Source:HGNC Symbol;Acc:HGNC:10895]	8518	-0.308429986	-0.25531694
							LMAN1	lectin, mannose binding 1 [Source:HGNC Symbol;Acc:HGNC:6631]	8594	-0.314173341	-0.25258586
							NAPA	NSF attachment protein alpha [Source:HGNC Symbol;Acc:HGNC:7641]	8672	-0.320593327	-0.24983484
							RAB22A	RAB22A, member RAS oncogene family [Source:HGNC Symbol;Acc:HGNC:9764]	8677	-0.320926666	-0.24147664
							SEC22B	SEC22 homolog B, vesicle trafficking protein [Source:HGNC Symbol;Acc:HGNC:10700]	8741	-0.325473338	-0.23752023
							MON2	MON2 homolog, regulator of endosome-to-Golgi trafficking [Source:HGNC Symbol;Acc:HGNC:29177]	8770	-0.327946663	-0.230813
							CAV2	caveolin 2 [Source:HGNC Symbol;Acc:HGNC:1528]	8845	-0.334813327	-0.22744796
							BNIP3	BCL2 interacting protein 3 [Source:HGNC Symbol;Acc:HGNC:1084]	8883	-0.337833345	-0.22116397
							ARFGEF1	ADP ribosylation factor guanine nucleotide exchange factor 1 [Source:HGNC Symbol;Acc:HGNC:15772]	9004	-0.348003328	-0.22097042
							GALC	galactosylceramidase [Source:HGNC Symbol;Acc:HGNC:4115]	9023	-0.349556655	-0.21291284
							CLTC	clathrin heavy chain [Source:HGNC Symbol;Acc:HGNC:2092]	9060	-0.352356672	-0.20616004
							TMED10	transmembrane p24 trafficking protein 10 [Source:HGNC Symbol;Acc:HGNC:16998]	9207	-0.365869999	-0.20747796
							AP2B1	adaptor related protein complex 2 subunit beta 1 [Source:HGNC Symbol;Acc:HGNC:563]	9254	-0.369643331	-0.20102529
							ARFGEF2	ADP ribosylation factor guanine nucleotide exchange factor 2 [Source:HGNC Symbol;Acc:HGNC:15853]	9407	-0.381786674	-0.2023736
							STX12	syntaxin 12 [Source:HGNC Symbol;Acc:HGNC:11430]	9726	-0.405783325	-0.21580404
							SCAMP3	secretory carrier membrane protein 3 [Source:HGNC Symbol;Acc:HGNC:10565]	9778	-0.410103321	-0.2086424
							ARCN1	archain 1 [Source:HGNC Symbol;Acc:HGNC:649]	9928	-0.423036665	-0.2086469
							ARFIP1	ADP ribosylation factor interacting protein 1 [Source:HGNC Symbol;Acc:HGNC:21496]	10232	-0.449829996	-0.2197378
							TMX1	thioredoxin related transmembrane protein 1 [Source:HGNC Symbol;Acc:HGNC:15487]	10356	-0.46323666	-0.21666303
							ADAM10	ADAM metallopeptidase domain 10 [Source:HGNC Symbol;Acc:HGNC:188]	10600	-0.489833325	-0.22207263
							COG2	component of oligomeric golgi complex 2 [Source:HGNC Symbol;Acc:HGNC:6546]	10652	-0.49617666	-0.21258703
							AP2M1	adaptor related protein complex 2 subunit mu 1 [Source:HGNC Symbol;Acc:HGNC:564]	10934	-0.525129974	-0.21995772
							SNX2	sorting nexin 2 [Source:HGNC Symbol;Acc:HGNC:11173]	11015	-0.534953356	-0.21164909
							TOM1L1	target of myb1 like 1 membrane trafficking protein [Source:HGNC Symbol;Acc:HGNC:11983]	11037	-0.537186682	-0.1987556
							MAPK1	mitogen-activated protein kinase 1 [Source:HGNC Symbol;Acc:HGNC:6871]	11179	-0.555410028	-0.19457257
							COPB1	COPI coat complex subunit beta 1 [Source:HGNC Symbol;Acc:HGNC:2231]	11225	-0.561179996	-0.18287176
							KRT18	keratin 18 [Source:HGNC Symbol;Acc:HGNC:6430]	11419	-0.588786662	-0.18177529
							ABCA1	ATP binding cassette subfamily A member 1 [Source:HGNC Symbol;Acc:HGNC:29]	11444	-0.59187001	-0.16763543
							ARF1	ADP ribosylation factor 1 [Source:HGNC Symbol;Acc:HGNC:652]	11649	-0.625419974	-0.16639343
							KIF1B	kinesin family member 1B [Source:HGNC Symbol;Acc:HGNC:16636]	11689	-0.63198334	-0.15232083
							PAM	peptidylglycine alpha-amidating monooxygenase [Source:HGNC Symbol;Acc:HGNC:8596]	11718	-0.638463318	-0.13722971
							IGF2R	insulin like growth factor 2 receptor [Source:HGNC Symbol;Acc:HGNC:5467]	11842	-0.66099	-0.12881567
							GBF1	golgi brefeldin A resistant guanine nucleotide exchange factor 1 [Source:HGNC Symbol;Acc:HGNC:4181]	12116	-0.724810004	-0.13018157
							SH3GL2	SH3 domain containing GRB2 like 2, endophilin A1 [Source:HGNC Symbol;Acc:HGNC:10831]	12213	-0.751066685	-0.11726491
							ATP1A1	ATPase Na+/K+ transporting subunit alpha 1 [Source:HGNC Symbol;Acc:HGNC:799]	12440	-0.822340012	-0.11239323
							SGMS1	sphingomyelin synthase 1 [Source:HGNC Symbol;Acc:HGNC:29799]	12576	-0.888493359	-0.09875689
							SNAP23	synaptosome associated protein 23 [Source:HGNC Symbol;Acc:HGNC:11131]	12607	-0.905903339	-0.07659834
							CLN5	CLN5 intracellular trafficking protein [Source:HGNC Symbol;Acc:HGNC:2076]	12669	-0.940793335	-0.05587507
							DST	dystonin [Source:HGNC Symbol;Acc:HGNC:1090]	12739	-0.983423352	-0.0346143
							CTSC	cathepsin C [Source:HGNC Symbol;Acc:HGNC:2528]	12744	-0.987233341	-0.008265992
							ATP6V1B1	ATPase H+ transporting V1 subunit B1 [Source:HGNC Symbol;Acc:HGNC:853]	13009	-1.376946688	0.008665825

PROTEIN SECRETION	84	-0.2867099	0.01897533	0.1562353	0.704	4647	YKT6	YKT6 v-SNARE homolog [Source:HGNC Symbol;Acc:HGNC:16959]	8391	-0.300020009	-0.27887407
							CLTA	clathrin light chain A [Source:HGNC Symbol;Acc:HGNC:2090]	8410	-0.302210003	-0.27237067
							VPS4B	vacuolar protein sorting 4 homolog B [Source:HGNC Symbol;Acc:HGNC:10895]	8473	-0.308429986	-0.26910168
							LMAN1	lectin, mannose binding 1 [Source:HGNC Symbol;Acc:HGNC:6631]	8549	-0.314173341	-0.26668635
							NAPA	NSF attachment protein alpha [Source:HGNC Symbol;Acc:HGNC:7641]	8627	-0.320593327	-0.26425773
							RAB22A	RAB22A, member RAS oncogene family [Source:HGNC Symbol;Acc:HGNC:9764]	8632	-0.320926666	-0.25618467
							SEC22B	SEC22 homolog B, vesicle trafficking protein [Source:HGNC Symbol;Acc:HGNC:10700]	8696	-0.325473338	-0.25254777
							MON2	MON2 homolog, regulator of endosome-to-Golgi trafficking [Source:HGNC Symbol;Acc:HGNC:29177]	8726	-0.327946663	-0.24622141
							CAV2	caveolin 2 [Source:HGNC Symbol;Acc:HGNC:1528]	8801	-0.334813327	-0.2431898
							BNIP3	BCL2 interacting protein 3 [Source:HGNC Symbol;Acc:HGNC:1084]	8839	-0.337833345	-0.23722284
							ARFGEF1	ADP ribosylation factor guanine nucleotide exchange factor 1 [Source:HGNC Symbol;Acc:HGNC:15772]	8960	-0.348003328	-0.23739803
							GALC	galactosylceramidase [Source:HGNC Symbol;Acc:HGNC:4115]	8979	-0.349556655	-0.22965804
							CLTC	clathrin heavy chain [Source:HGNC Symbol;Acc:HGNC:2092]	9016	-0.352356672	-0.22323455
							TMED10	transmembrane p24 trafficking protein 10 [Source:HGNC Symbol;Acc:HGNC:16998]	9163	-0.365869999	-0.22495036
							AP2B1	adaptor related protein complex 2 subunit beta 1 [Source:HGNC Symbol;Acc:HGNC:563]	9210	-0.369643331	-0.21884741
							ARFGEF2	ADP ribosylation factor guanine nucleotide exchange factor 2 [Source:HGNC Symbol;Acc:HGNC:15853]	9363	-0.381786674	-0.22061074
							STX12	syntaxin 12 [Source:HGNC Symbol;Acc:HGNC:11430]	9682	-0.405783325	-0.23456289
							SCAMP3	secretory carrier membrane protein 3 [Source:HGNC Symbol;Acc:HGNC:10565]	9734	-0.410103321	-0.22778922
							ARCN1	archain 1 [Source:HGNC Symbol;Acc:HGNC:649]	9884	-0.423036665	-0.22824357
							ARFIP1	ADP ribosylation factor interacting protein 1 [Source:HGNC Symbol;Acc:HGNC:21496]	10187	-0.449829996	-0.2398101
							TMX1	thioredoxin related transmembrane protein 1 [Source:HGNC Symbol;Acc:HGNC:15487]	10311	-0.46323666	-0.23720726
							ADAM10	ADAM metallopeptidase domain 10 [Source:HGNC Symbol;Acc:HGNC:188]	10558	-0.489833325	-0.24340566
							COG2	component of oligomeric golgi complex 2 [Source:HGNC Symbol;Acc:HGNC:6546]	10610	-0.49617666	-0.23438394
							AP2M1	adaptor related protein complex 2 subunit mu 1 [Source:HGNC Symbol;Acc:HGNC:564]	10890	-0.525129974	-0.24220814
							SNX2	sorting nexin 2 [Source:HGNC Symbol;Acc:HGNC:11173]	10969	-0.534953356	-0.23425813
							TOM1L1	target of myb1 like 1 membrane trafficking protein [Source:HGNC Symbol;Acc:HGNC:11983]	10991	-0.537186682	-0.22184928
							MAPK1	mitogen-activated protein kinase 1 [Source:HGNC Symbol;Acc:HGNC:6871]	11133	-0.555410028	-0.21822871
							COPB1	COPI coat complex subunit beta 1 [Source:HGNC Symbol;Acc:HGNC:2231]	11178	-0.561179996	-0.20696886
							KRT18	keratin 18 [Source:HGNC Symbol;Acc:HGNC:6430]	11371	-0.588786662	-0.2064139
							ABCA1	ATP binding cassette subfamily A member 1 [Source:HGNC Symbol;Acc:HGNC:29]	11395	-0.59187001	-0.19273123
							ARF1	ADP ribosylation factor 1 [Source:HGNC Symbol;Acc:HGNC:652]	11603	-0.625419974	-0.19237752
							PAM	peptidylglycine alpha-amidating monooxygenase [Source:HGNC Symbol;Acc:HGNC:8596]	11672	-0.638463318	-0.18095204
							IGF2R	insulin like growth factor 2 receptor [Source:HGNC Symbol;Acc:HGNC:5467]	11790	-0.66099	-0.17272112
							GBF1	golgi brefeldin A resistant guanine nucleotide exchange factor 1 [Source:HGNC Symbol;Acc:HGNC:4181]	12062	-0.724810004	-0.17471251
							SH3GL2	SH3 domain containing GRB2 like 2, endophilin A1 [Source:HGNC Symbol;Acc:HGNC:10831]	12160	-0.751066685	-0.16258495
							ATP1A1	ATPase Na+/K+ transporting subunit alpha 1 [Source:HGNC Symbol;Acc:HGNC:799]	12385	-0.822340012	-0.15840057
							SGMS1	sphingomyelin synthase 1 [Source:HGNC Symbol;Acc:HGNC:29799]	12517	-0.888493359	-0.1453086
							SNAP23	synaptosome associated protein 23 [Source:HGNC Symbol;Acc:HGNC:11131]	12548	-0.905903339	-0.1239645
							TPD52	tumor protein D52 [Source:HGNC Symbol;Acc:HGNC:12005]	12554	-0.907633305	-0.10064517
							CLN5	CLN5 intracellular trafficking protein [Source:HGNC Symbol;Acc:HGNC:2076]	12611	-0.940793335	-0.080397084
							SSPN	sarcospan [Source:HGNC Symbol;Acc:HGNC:11322]	12665	-0.975099981	-0.05902138
							DST	dystonin [Source:HGNC Symbol;Acc:HGNC:1090]	12675	-0.983423352	-0.034031387
							CTSC	cathepsin C [Source:HGNC Symbol;Acc:HGNC:2528]	12682	-0.987233341	-0.008710285
							ATP6V1B1	ATPase H+ transporting V1 subunit B1 [Source:HGNC Symbol;Acc:HGNC:853]	12934	-1.376946688	0.007874717

**PEROXISOME**	90	-0.28273052	0.03061225	0.1528909	0.729	4687	CAT	catalase [Source:HGNC Symbol;Acc:HGNC:1516]	8351	-0.297176659	-0.27555132
							PEX5	peroxisomal biogenesis factor 5 [Source:HGNC Symbol;Acc:HGNC:9719]	8399	-0.300976664	-0.27191052
							IDH2	isocitrate dehydrogenase (NADP(+)) 2 [Source:HGNC Symbol;Acc:HGNC:5383]	8405	-0.30133	-0.26501715
							NR1I2	nuclear receptor subfamily 1 group I member 2 [Source:HGNC Symbol;Acc:HGNC:7968]	8447	-0.30613333	-0.26078835
							VPS4B	vacuolar protein sorting 4 homolog B [Source:HGNC Symbol;Acc:HGNC:10895]	8473	-0.308429986	-0.25526825
							HSD17B11	hydroxysteroid 17-beta dehydrogenase 11 [Source:HGNC Symbol;Acc:HGNC:22960]	8522	-0.31189999	-0.25144076
							SLC25A19	solute carrier family 25 member 19 [Source:HGNC Symbol;Acc:HGNC:14409]	8560	-0.315079987	-0.24668686
							IDE	insulin degrading enzyme [Source:HGNC Symbol;Acc:HGNC:5381]	8635	-0.321170002	-0.24464364
							ACSL5	acyl-CoA synthetase long chain family member 5 [Source:HGNC Symbol;Acc:HGNC:16526]	8649	-0.322286665	-0.23786193
							SULT2B1	sulfotransferase family 2B member 1 [Source:HGNC Symbol;Acc:HGNC:11459]	8652	-0.32251668	-0.23022504
							ESR2	estrogen receptor 2 [Source:HGNC Symbol;Acc:HGNC:3468]	9060	-0.356576681	-0.25304672
							SLC25A4	solute carrier family 25 member 4 [Source:HGNC Symbol;Acc:HGNC:10990]	9253	-0.372666657	-0.25887352
							PRDX1	peroxiredoxin 1 [Source:HGNC Symbol;Acc:HGNC:9352]	9285	-0.375586659	-0.25219446
							PEX11B	peroxisomal biogenesis factor 11 beta [Source:HGNC Symbol;Acc:HGNC:8853]	9584	-0.398456663	-0.26558545
							CRAT	carnitine O-acetyltransferase [Source:HGNC Symbol;Acc:HGNC:2342]	9724	-0.409333318	-0.26643285
							ELOVL5	ELOVL fatty acid elongase 5 [Source:HGNC Symbol;Acc:HGNC:21308]	9797	-0.414933324	-0.26197
							CADM1	cell adhesion molecule 1 [Source:HGNC Symbol;Acc:HGNC:5951]	9866	-0.421646655	-0.25703606
							SCP2	sterol carrier protein 2 [Source:HGNC Symbol;Acc:HGNC:10606]	9944	-0.427520007	-0.25265533
							YWHAH	tyrosine 3-monooxygenase/tryptophan 5-monooxygenase activation protein eta [Source:HGNC Symbol;Acc:HGNC:12853]	10056	-0.438210011	-0.25064248
							PEX6	peroxisomal biogenesis factor 6 [Source:HGNC Symbol;Acc:HGNC:8859]	10105	-0.442270011	-0.24366552
							HSD17B4	hydroxysteroid 17-beta dehydrogenase 4 [Source:HGNC Symbol;Acc:HGNC:5213]	10222	-0.453893334	-0.24165997
							CACNA1B	calcium voltage-gated channel subunit alpha1 B [Source:HGNC Symbol;Acc:HGNC:1389]	10340	-0.466083348	-0.23943716
							ABCB9	ATP binding cassette subfamily B member 9 [Source:HGNC Symbol;Acc:HGNC:50]	10418	-0.474723339	-0.2339161
							ACAA1	acetyl-CoA acyltransferase 1 [Source:HGNC Symbol;Acc:HGNC:82]	10503	-0.484629989	-0.22869639
							ACOX1	acyl-CoA oxidase 1 [Source:HGNC Symbol;Acc:HGNC:119]	10529	-0.486626655	-0.21887141
							GNPAT	glyceronephosphate O-acyltransferase [Source:HGNC Symbol;Acc:HGNC:4416]	10657	-0.499926656	-0.2166034
							IDH1	isocitrate dehydrogenase (NADP(+)) 1 [Source:HGNC Symbol;Acc:HGNC:5382]	10862	-0.522189975	-0.21974486
							DHCR24	24-dehydrocholesterol reductase [Source:HGNC Symbol;Acc:HGNC:2859]	10968	-0.534493327	-0.21494254
							ABCD3	ATP binding cassette subfamily D member 3 [Source:HGNC Symbol;Acc:HGNC:67]	11158	-0.558516681	-0.21604787
							ALDH9A1	aldehyde dehydrogenase 9 family member A1 [Source:HGNC Symbol;Acc:HGNC:412]	11410	-0.593946695	-0.22108603
							CTPS1	CTP synthase 1 [Source:HGNC Symbol;Acc:HGNC:2519]	11518	-0.611543357	-0.21457681
							CLN6	CLN6 transmembrane ER protein [Source:HGNC Symbol;Acc:HGNC:2077]	11519	-0.611596644	-0.19980185
							DIO1	iodothyronine deiodinase 1 [Source:HGNC Symbol;Acc:HGNC:2883]	11665	-0.637266695	-0.19560625
							EHHADH	enoyl-CoA hydratase and 3-hydroxyacyl CoA dehydrogenase [Source:HGNC Symbol;Acc:HGNC:3247]	12030	-0.716523349	-0.2064111
							ABCB4	ATP binding cassette subfamily B member 4 [Source:HGNC Symbol;Acc:HGNC:45]	12057	-0.723596692	-0.19093862
							HSD3B7	hydroxy-delta-5-steroid dehydrogenase, 3 beta- and steroid delta-isomerase 7 [Source:HGNC Symbol;Acc:HGNC:18324]	12077	-0.727653325	-0.17482747
							ECI2	enoyl-CoA delta isomerase 2 [Source:HGNC Symbol;Acc:HGNC:14601]	12193	-0.760309994	-0.16534226
							PEX11A	peroxisomal biogenesis factor 11 alpha [Source:HGNC Symbol;Acc:HGNC:8852]	12216	-0.767356694	-0.14850368
							CLN8	CLN8 transmembrane ER and ERGIC protein [Source:HGNC Symbol;Acc:HGNC:2079]	12294	-0.79404664	-0.1352684
							HMGCL	3-hydroxy-3-methylglutaryl-CoA lyase [Source:HGNC Symbol;Acc:HGNC:5005]	12303	-0.796599984	-0.11664203
							RETSAT	retinol saturase [Source:HGNC Symbol;Acc:HGNC:25991]	12436	-0.843850017	-0.1064517
							ALDH1A1	aldehyde dehydrogenase 1 family member A1 [Source:HGNC Symbol;Acc:HGNC:402]	12518	-0.889036655	-0.09123061
							IDI1	isopentenyl-diphosphate delta isomerase 1 [Source:HGNC Symbol;Acc:HGNC:5387]	12680	-0.987006664	-0.0798218
							SCGB1A1	secretoglobin family 1A member 1 [Source:HGNC Symbol;Acc:HGNC:12523]	12696	-0.996216655	-0.05691375
							EPHX2	epoxide hydrolase 2 [Source:HGNC Symbol;Acc:HGNC:3402]	12729	-1.019556642	-0.034754895
							ACSL1	acyl-CoA synthetase long chain family member 1 [Source:HGNC Symbol;Acc:HGNC:3569]	12838	-1.17513001	-0.014707782
							ISOC1	isochorismatase domain containing 1 [Source:HGNC Symbol;Acc:HGNC:24254]	12884	-1.238659978	0.01174008

**ANDROGEN RESPONSE**	88	-0.25706634	0.10375276	0.23846436	0.909	2641	KRT8	keratin 8 [Source:HGNC Symbol;Acc:HGNC:6446]	11161	-0.510523319	-0.24585865
							CCND1	cyclin D1 [Source:HGNC Symbol;Acc:HGNC:1582]	11279	-0.523143351	-0.24290599
							DHCR24	24-dehydrocholesterol reductase [Source:HGNC Symbol;Acc:HGNC:2859]	11382	-0.534493327	-0.2386103
							AKAP12	A-kinase anchoring protein 12 [Source:HGNC Symbol;Acc:HGNC:370]	11396	-0.535669982	-0.22779858
							ZBTB10	zinc finger and BTB domain containing 10 [Source:HGNC Symbol;Acc:HGNC:30953]	11409	-0.537329972	-0.2168775
							HOMER2	homer scaffold protein 2 [Source:HGNC Symbol;Acc:HGNC:17513]	11531	-0.552370012	-0.21357492
							NCOA4	nuclear receptor coactivator 4 [Source:HGNC Symbol;Acc:HGNC:7671]	11621	-0.564856648	-0.20766464
							ACTN1	actinin alpha 1 [Source:HGNC Symbol;Acc:HGNC:163]	11622	-0.565066695	-0.19525957
							HERC3	HECT and RLD domain containing E3 ubiquitin protein ligase 3 [Source:HGNC Symbol;Acc:HGNC:4876]	11632	-0.566829979	-0.1834721
							GNAI3	G protein subunit alpha i3 [Source:HGNC Symbol;Acc:HGNC:4387]	11746	-0.582729995	-0.17891961
							XRCC6	X-ray repair cross complementing 6 [Source:HGNC Symbol;Acc:HGNC:4055]	11893	-0.603083313	-0.17632678
							UBE2I	ubiquitin conjugating enzyme E2 I [Source:HGNC Symbol;Acc:HGNC:12485]	11911	-0.605436683	-0.16427515
							TNFAIP8	TNF alpha induced protein 8 [Source:HGNC Symbol;Acc:HGNC:17260]	12191	-0.651413321	-0.17032015
							SRF	serum response factor [Source:HGNC Symbol;Acc:HGNC:11291]	12450	-0.70290333	-0.17370337
							MAK	male germ cell associated kinase [Source:HGNC Symbol;Acc:HGNC:6816]	12500	-0.714846671	-0.16158338
							HMGCR	3-hydroxy-3-methylglutaryl-CoA reductase [Source:HGNC Symbol;Acc:HGNC:5006]	12632	-0.746763349	-0.15474245
							B2M	beta-2-microglobulin [Source:HGNC Symbol;Acc:HGNC:914]	12860	-0.809759974	-0.15351918
							ABHD2	abhydrolase domain containing 2, acylglycerol lipase [Source:HGNC Symbol;Acc:HGNC:18717]	13064	-0.889653325	-0.14879185
							TPD52	tumor protein D52 [Source:HGNC Symbol;Acc:HGNC:12005]	13102	-0.907633305	-0.13156447
							TMPRSS2	transmembrane serine protease 2 [Source:HGNC Symbol;Acc:HGNC:11876]	13179	-0.942006648	-0.11642651
							IDI1	isopentenyl-diphosphate delta isomerase 1 [Source:HGNC Symbol;Acc:HGNC:5387]	13268	-0.987006664	-0.10117574
							INSIG1	insulin induced gene 1 [Source:HGNC Symbol;Acc:HGNC:6083]	13368	-1.063536644	-0.08504704
							FKBP5	FKBP prolyl isomerase 5 [Source:HGNC Symbol;Acc:HGNC:3721]	13423	-1.113193393	-0.064546645
							SORD	sorbitol dehydrogenase [Source:HGNC Symbol;Acc:HGNC:11184]	13432	-1.124303341	-0.040447876
							INPP4B	inositol polyphosphate-4-phosphatase type II B [Source:HGNC Symbol;Acc:HGNC:6075]	13574	-1.281023383	-0.022607412
							AZGP1	alpha-2-glycoprotein 1, zinc-binding [Source:HGNC Symbol;Acc:HGNC:910]	13720	-1.77718997	0.005833846

ANDROGEN RESPONSE	87	-0.29870087	0.01286765	0.2037243	0.637	2326	KRT8	keratin 8 [Source:HGNC Symbol;Acc:HGNC:6446]	10798	-0.510523319	-0.28685167
							CCND1	cyclin D1 [Source:HGNC Symbol;Acc:HGNC:1582]	10916	-0.523143351	-0.2836847
							DHCR24	24-dehydrocholesterol reductase [Source:HGNC Symbol;Acc:HGNC:2859]	11014	-0.534493327	-0.2787201
							AKAP12	A-kinase anchoring protein 12 [Source:HGNC Symbol;Acc:HGNC:370]	11027	-0.535669982	-0.2672078
							ZBTB10	zinc finger and BTB domain containing 10 [Source:HGNC Symbol;Acc:HGNC:30953]	11039	-0.537329972	-0.25558022
							HOMER2	homer scaffold protein 2 [Source:HGNC Symbol;Acc:HGNC:17513]	11160	-0.552370012	-0.25196505
							NCOA4	nuclear receptor coactivator 4 [Source:HGNC Symbol;Acc:HGNC:7671]	11250	-0.564856648	-0.24568203
							ACTN1	actinin alpha 1 [Source:HGNC Symbol;Acc:HGNC:163]	11251	-0.565066695	-0.2325669
							HERC3	HECT and RLD domain containing E3 ubiquitin protein ligase 3 [Source:HGNC Symbol;Acc:HGNC:4876]	11260	-0.566829979	-0.22002451
							ANKH	ANKH inorganic pyrophosphate transport regulator [Source:HGNC Symbol;Acc:HGNC:15492]	11352	-0.581049979	-0.21351907
							GNAI3	G protein subunit alpha i3 [Source:HGNC Symbol;Acc:HGNC:4387]	11374	-0.582729995	-0.20160489
							PDLIM5	PDZ and LIM domain 5 [Source:HGNC Symbol;Acc:HGNC:17468]	11393	-0.586333334	-0.18937695
							XRCC6	X-ray repair cross complementing 6 [Source:HGNC Symbol;Acc:HGNC:4055]	11516	-0.603083313	-0.18473814
							UBE2I	ubiquitin conjugating enzyme E2 I [Source:HGNC Symbol;Acc:HGNC:12485]	11531	-0.605436683	-0.17175996
							SLC38A2	solute carrier family 38 member 2 [Source:HGNC Symbol;Acc:HGNC:13448]	12005	-0.696269989	-0.19188374
							SRF	serum response factor [Source:HGNC Symbol;Acc:HGNC:11291]	12032	-0.70290333	-0.1775639
							MAK	male germ cell associated kinase [Source:HGNC Symbol;Acc:HGNC:6816]	12074	-0.714846671	-0.16411753
							HMGCR	3-hydroxy-3-methylglutaryl-CoA reductase [Source:HGNC Symbol;Acc:HGNC:5006]	12197	-0.746763349	-0.15614392
							ARID5B	AT-rich interaction domain 5B [Source:HGNC Symbol;Acc:HGNC:17362]	12304	-0.777956665	-0.14621896
							B2M	beta-2-microglobulin [Source:HGNC Symbol;Acc:HGNC:914]	12403	-0.809759974	-0.13494214
							ABHD2	abhydrolase domain containing 2, acylglycerol lipase [Source:HGNC Symbol;Acc:HGNC:18717]	12580	-0.889653325	-0.12779446
							IDI1	isopentenyl-diphosphate delta isomerase 1 [Source:HGNC Symbol;Acc:HGNC:5387]	12743	-0.987006664	-0.11731326
							MYL12A	myosin light chain 12A [Source:HGNC Symbol;Acc:HGNC:16701]	12804	-1.035836697	-0.09787423
							FKBP5	FKBP prolyl isomerase 5 [Source:HGNC Symbol;Acc:HGNC:3721]	12869	-1.113193393	-0.0769466
							SORD	sorbitol dehydrogenase [Source:HGNC Symbol;Acc:HGNC:11184]	12875	-1.124303341	-0.05123519
							INPP4B	inositol polyphosphate-4-phosphatase type II B [Source:HGNC Symbol;Acc:HGNC:6075]	12978	-1.281023383	-0.029327251
							ADAMTS1	ADAM metallopeptidase with thrombospondin type 1 motif 1 [Source:HGNC Symbol;Acc:HGNC:217]	13076	-1.736193299	0.003528687

ANDROGEN RESPONSE	83	-0.2798041	0.01587302	0.1508983	0.751	4524	ALDH1A3	aldehyde dehydrogenase 1 family member A3 [Source:HGNC Symbol;Acc:HGNC:409]	8514	-0.311340004	-0.27190375
							LMAN1	lectin, mannose binding 1 [Source:HGNC Symbol;Acc:HGNC:6631]	8549	-0.314173341	-0.2665562
							ZMIZ1	zinc finger MIZ-type containing 1 [Source:HGNC Symbol;Acc:HGNC:16493]	8715	-0.326646656	-0.27100483
							MAP7	microtubule associated protein 7 [Source:HGNC Symbol;Acc:HGNC:6869]	8728	-0.328189999	-0.26360327
							VAPA	VAMP associated protein A [Source:HGNC Symbol;Acc:HGNC:12648]	8915	-0.34398666	-0.26923302
							RRP12	ribosomal RNA processing 12 homolog [Source:HGNC Symbol;Acc:HGNC:29100]	8930	-0.345710009	-0.26154128
							B4GALT1	beta-1,4-galactosyltransferase 1 [Source:HGNC Symbol;Acc:HGNC:924]	8931	-0.345770001	-0.25276726
							UBE2J1	ubiquitin conjugating enzyme E2 J1 [Source:HGNC Symbol;Acc:HGNC:17598]	9027	-0.353983343	-0.25111848
							IQGAP2	IQ motif containing GTPase activating protein 2 [Source:HGNC Symbol;Acc:HGNC:6111]	9053	-0.356013328	-0.24401446
							PGM3	phosphoglucomutase 3 [Source:HGNC Symbol;Acc:HGNC:8907]	9098	-0.359956652	-0.2382771
							HPGD	15-hydroxyprostaglandin dehydrogenase [Source:HGNC Symbol;Acc:HGNC:5154]	9156	-0.365066677	-0.23341362
							PTK2B	protein tyrosine kinase 2 beta [Source:HGNC Symbol;Acc:HGNC:9612]	9569	-0.397606671	-0.25512907
							PLPP1	phospholipid phosphatase 1 [Source:HGNC Symbol;Acc:HGNC:9228]	9581	-0.398243338	-0.2458727
							SPCS3	signal peptidase complex subunit 3 [Source:HGNC Symbol;Acc:HGNC:26212]	9617	-0.401186675	-0.23839435
							SLC26A2	solute carrier family 26 member 2 [Source:HGNC Symbol;Acc:HGNC:10994]	9747	-0.411303341	-0.23791574
							ELOVL5	ELOVL fatty acid elongase 5 [Source:HGNC Symbol;Acc:HGNC:21308]	9797	-0.414933324	-0.2311693
							DNAJB9	DnaJ heat shock protein family (Hsp40) member B9 [Source:HGNC Symbol;Acc:HGNC:6968]	9932	-0.427046657	-0.23067717
							SELENOP	selenoprotein P [Source:HGNC Symbol;Acc:HGNC:10751]	9950	-0.427859992	-0.22113244
							PTPN21	protein tyrosine phosphatase non-receptor type 21 [Source:HGNC Symbol;Acc:HGNC:9651]	10199	-0.451099992	-0.22883032
							KRT8	keratin 8 [Source:HGNC Symbol;Acc:HGNC:6446]	10754	-0.510523319	-0.25864238
							CCND1	cyclin D1 [Source:HGNC Symbol;Acc:HGNC:1582]	10872	-0.523143351	-0.25439942
							DHCR24	24-dehydrocholesterol reductase [Source:HGNC Symbol;Acc:HGNC:2859]	10968	-0.534493327	-0.24817015
							AKAP12	A-kinase anchoring protein 12 [Source:HGNC Symbol;Acc:HGNC:370]	10981	-0.535669982	-0.23550373
							ZBTB10	zinc finger and BTB domain containing 10 [Source:HGNC Symbol;Acc:HGNC:30953]	10993	-0.537329972	-0.222718
							HOMER2	homer scaffold protein 2 [Source:HGNC Symbol;Acc:HGNC:17513]	11114	-0.552370012	-0.217965
							NCOA4	nuclear receptor coactivator 4 [Source:HGNC Symbol;Acc:HGNC:7671]	11203	-0.564856648	-0.21042489
							ACTN1	actinin alpha 1 [Source:HGNC Symbol;Acc:HGNC:163]	11204	-0.565066695	-0.19608615
							HERC3	HECT and RLD domain containing E3 ubiquitin protein ligase 3 [Source:HGNC Symbol;Acc:HGNC:4876]	11214	-0.566829979	-0.18239745
							ANKH	ANKH inorganic pyrophosphate transport regulator [Source:HGNC Symbol;Acc:HGNC:15492]	11305	-0.581049979	-0.17460082
							GNAI3	G protein subunit alpha i3 [Source:HGNC Symbol;Acc:HGNC:4387]	11327	-0.582729995	-0.16143501
							XRCC6	X-ray repair cross complementing 6 [Source:HGNC Symbol;Acc:HGNC:4055]	11469	-0.603083313	-0.15701628

						UBE2I	ubiquitin conjugating enzyme E2 I [Source:HGNC Symbol;Acc:HGNC:12485]	11485	-0.605436683	-0.14281109
						SRF	serum response factor [Source:HGNC Symbol;Acc:HGNC:11291]	11976	-0.70290333	-0.16280088
						MAK	male germ cell associated kinase [Source:HGNC Symbol;Acc:HGNC:6816]	12019	-0.714846671	-0.1479037
						HMGCR	3-hydroxy-3-methylglutaryl-CoA reductase [Source:HGNC Symbol;Acc:HGNC:5006]	12144	-0.746763349	-0.13852671
						B2M	beta-2-microglobulin [Source:HGNC Symbol;Acc:HGNC:914]	12345	-0.809759974	-0.13341807
						ABHD2	abhydrolase domain containing 2, acylglycerol lipase [Source:HGNC Symbol;Acc:HGNC:18717]	12521	-0.889653325	-0.12435222
						TPD52	tumor protein D52 [Source:HGNC Symbol;Acc:HGNC:12005]	12554	-0.907633305	-0.10379105
						IDI1	isopentenyl-diphosphate delta isomerase 1 [Source:HGNC Symbol;Acc:HGNC:5387]	12680	-0.987006664	-0.088395014
						MYL12A	myosin light chain 12A [Source:HGNC Symbol;Acc:HGNC:16701]	12739	-1.035836697	-0.06658776
						FKBP5	FKBP prolyl isomerase 5 [Source:HGNC Symbol;Acc:HGNC:3721]	12801	-1.113193393	-0.04304914
						SORD	sorbitol dehydrogenase [Source:HGNC Symbol;Acc:HGNC:11184]	12808	-1.124303341	-0.014982817
						INPP4B	inositol polyphosphate-4-phosphatase type II B [Source:HGNC Symbol;Acc:HGNC:6075]	12905	-1.281023383	0.010112662

NOTCH SIGNALING

31	-0.2758961	0.29583332	0.154018	0.784	3513	DTX2	deltex E3 ubiquitin ligase 2 [Source:HGNC Symbol;Acc:HGNC:15973]	9525	-0.394183338	-0.24993984
						NOTCH3	notch receptor 3 [Source:HGNC Symbol;Acc:HGNC:7883]	9781	-0.41407001	-0.24228044
						ST3GAL6	ST3 beta-galactoside alpha-2,3-sialyltransferase 6 [Source:HGNC Symbol;Acc:HGNC:18080]	10016	-0.43494001	-0.23163213
						FZD1	frizzled class receptor 1 [Source:HGNC Symbol;Acc:HGNC:4038]	10546	-0.488633335	-0.24013005
						NOTCH2	notch receptor 2 [Source:HGNC Symbol;Acc:HGNC:7882]	10570	-0.491163343	-0.20955625
						CCND1	cyclin D1 [Source:HGNC Symbol;Acc:HGNC:1582]	10872	-0.523143351	-0.1982514
						FZD5	frizzled class receptor 5 [Source:HGNC Symbol;Acc:HGNC:4043]	10942	-0.530913353	-0.16859695
						LFNG	LFNG O-fucosylpeptide 3-beta-N-acetylglucosaminyltransferase [Source:HGNC Symbol;Acc:HGNC:6560]	11240	-0.570596695	-0.15385982
						ARRB1	arrestin beta 1 [Source:HGNC Symbol;Acc:HGNC:711]	11456	-0.600260019	-0.13086464
						JAG1	jagged canonical Notch ligand 1 [Source:HGNC Symbol;Acc:HGNC:6188]	11514	-0.610096693	-0.09507348
						HES1	hes family bHLH transcription factor 1 [Source:HGNC Symbol;Acc:HGNC:5192]	11605	-0.625566661	-0.06080094
						FBXW11	F-box and WD repeat domain containing 11 [Source:HGNC Symbol;Acc:HGNC:13607]	11612	-0.626373351	-0.020016726
						NOTCH1	notch receptor 1 [Source:HGNC Symbol;Acc:HGNC:7881]	12240	-0.773273349	-0.017306611
						DLL1	delta like canonical Notch ligand 1 [Source:HGNC Symbol;Acc:HGNC:2908]	12856	-1.191106677	0.013839715

TGF BETA SIGNALING

46	-0.27350658	0.14643545	0.1658625	0.837	4725	TGFB1	transforming growth factor beta 1 [Source:HGNC Symbol;Acc:HGNC:11766]	8399	-0.297356665	-0.25975904
						PMEPA1	prostate transmembrane protein, androgen induced 1 [Source:HGNC Symbol;Acc:HGNC:14107]	8421	-0.29914999	-0.24753447
						TJP1	tight junction protein 1 [Source:HGNC Symbol;Acc:HGNC:11827]	8737	-0.324916661	-0.25660086
						FURIN	furin, paired basic amino acid cleaving enzyme [Source:HGNC Symbol;Acc:HGNC:8568]	8748	-0.325876653	-0.24229947
						SLC20A1	solute carrier family 20 member 1 [Source:HGNC Symbol;Acc:HGNC:10946]	8924	-0.341403335	-0.23989782
						SMAD7	SMAD family member 7 [Source:HGNC Symbol;Acc:HGNC:6773]	8939	-0.342806667	-0.22511959
						SMURF1	SMAD specific E3 ubiquitin protein ligase 1 [Source:HGNC Symbol;Acc:HGNC:16807]	9286	-0.371773332	-0.23439026
						TGFBR1	transforming growth factor beta receptor 1 [Source:HGNC Symbol;Acc:HGNC:11772]	9359	-0.378336668	-0.22240466
						SKIL	SKI like proto-oncogene [Source:HGNC Symbol;Acc:HGNC:10897]	9736	-0.406256676	-0.23237517
						SMAD1	SMAD family member 1 [Source:HGNC Symbol;Acc:HGNC:6767]	9830	-0.414389998	-0.22032863
						CDH1	cadherin 1 [Source:HGNC Symbol;Acc:HGNC:1748]	9847	-0.415773332	-0.20232992
						ACVR1	activin A receptor type 1 [Source:HGNC Symbol;Acc:HGNC:171]	10580	-0.487610012	-0.23576263
						SMURF2	SMAD specific E3 ubiquitin protein ligase 2 [Source:HGNC Symbol;Acc:HGNC:16809]	10755	-0.505516648	-0.22569714
						NCOR2	nuclear receptor corepressor 2 [Source:HGNC Symbol;Acc:HGNC:7673]	11275	-0.569563329	-0.23905282
						PPM1A	protein phosphatase, Mg2+/Mn2+ dependent 1A [Source:HGNC Symbol;Acc:HGNC:9275]	11326	-0.577009976	-0.21619973
						RHOA	ras homolog family member A [Source:HGNC Symbol;Acc:HGNC:667]	11377	-0.583936691	-0.19302638
						ENG	endoglin [Source:HGNC Symbol;Acc:HGNC:3349]	11687	-0.631760001	-0.18744782
						SKI	SKI proto-oncogene [Source:HGNC Symbol;Acc:HGNC:10896]	11937	-0.681240022	-0.17499347
						JUNB	JunB proto-oncogene, AP-1 transcription factor subunit [Source:HGNC Symbol;Acc:HGNC:6205]	12210	-0.750383317	-0.16110125
						BCAR3	BCAR3 adaptor protein, NSP family member [Source:HGNC Symbol;Acc:HGNC:973]	12487	-0.839783311	-0.14338174
						SMAD6	SMAD family member 6 [Source:HGNC Symbol;Acc:HGNC:6772]	12624	-0.916469991	-0.111411
						PPP1CA	protein phosphatase 1 catalytic subunit alpha [Source:HGNC Symbol;Acc:HGNC:9281]	12653	-0.93296665	-0.0704188
						UBE2D3	ubiquitin conjugating enzyme E2 D3 [Source:HGNC Symbol;Acc:HGNC:12476]	12699	-0.956529975	-0.029637193
						THBS1	thrombospondin 1 [Source:HGNC Symbol;Acc:HGNC:11785]	12998	-1.339050055	0.009482317

46	-0.27134314	0.15510204	0.1564498	0.818	4684	TGFB1	transforming growth factor beta 1 [Source:HGNC Symbol;Acc:HGNC:11766]	8354	-0.297356665	-0.25748548
						PMEPA1	prostate transmembrane protein, androgen induced 1 [Source:HGNC Symbol;Acc:HGNC:14107]	8376	-0.29914999	-0.24516076
						TJP1	tight junction protein 1 [Source:HGNC Symbol;Acc:HGNC:11827]	8692	-0.324916661	-0.2542663
						FURIN	furin, paired basic amino acid cleaving enzyme [Source:HGNC Symbol;Acc:HGNC:8568]	8703	-0.325876653	-0.2398493
						SLC20A1	solute carrier family 20 member 1 [Source:HGNC Symbol;Acc:HGNC:10946]	8880	-0.341403335	-0.2374868
						SMAD7	SMAD family member 7 [Source:HGNC Symbol;Acc:HGNC:6773]	8895	-0.342806667	-0.22258872
						SMURF1	SMAD specific E3 ubiquitin protein ligase 1 [Source:HGNC Symbol;Acc:HGNC:16807]	9242	-0.371773332	-0.23189686
						TGFBR1	transforming growth factor beta receptor 1 [Source:HGNC Symbol;Acc:HGNC:11772]	9315	-0.378336668	-0.21980762
						SKIL	SKI like proto-oncogene [Source:HGNC Symbol;Acc:HGNC:10897]	9692	-0.406256676	-0.22981805
						SMAD1	SMAD family member 1 [Source:HGNC Symbol;Acc:HGNC:6767]	9786	-0.414389998	-0.21766512
						CDH1	cadherin 1 [Source:HGNC Symbol;Acc:HGNC:1748]	9803	-0.415773332	-0.19952054
						ACVR1	activin A receptor type 1 [Source:HGNC Symbol;Acc:HGNC:171]	10539	-0.487610012	-0.2333742
						SMURF2	SMAD specific E3 ubiquitin protein ligase 2 [Source:HGNC Symbol;Acc:HGNC:16809]	10712	-0.505516648	-0.22305565
						NCOR2	nuclear receptor corepressor 2 [Source:HGNC Symbol;Acc:HGNC:7673]	11228	-0.569563329	-0.23615523
						PPM1A	protein phosphatase, Mg2+/Mn2+ dependent 1A [Source:HGNC Symbol;Acc:HGNC:9275]	11279	-0.577009976	-0.21311377
						RHOA	ras homolog family member A [Source:HGNC Symbol;Acc:HGNC:667]	11330	-0.583936691	-0.18974952
						ENG	endoglin [Source:HGNC Symbol;Acc:HGNC:3349]	11641	-0.631760001	-0.18417041
						SKI	SKI proto-oncogene [Source:HGNC Symbol;Acc:HGNC:10896]	11882	-0.681240022	-0.17089705
						SMAD3	SMAD family member 3 [Source:HGNC Symbol;Acc:HGNC:6769]	12133	-0.744603336	-0.15544055
						JUNB	JunB proto-oncogene, AP-1 transcription factor subunit [Source:HGNC Symbol;Acc:HGNC:6205]	12158	-0.750383317	-0.12231804
						BCAR3	BCAR3 adaptor protein, NSP family member [Source:HGNC Symbol;Acc:HGNC:973]	12430	-0.839783311	-0.10404239
						PPP1CA	protein phosphatase 1 catalytic subunit alpha [Source:HGNC Symbol;Acc:HGNC:9281]	12595	-0.93296665	-0.07318767
						UBE2D3	ubiquitin conjugating enzyme E2 D3 [Source:HGNC Symbol;Acc:HGNC:12476]	12639	-0.956529975	-0.031920694
						THBS1	thrombospondin 1 [Source:HGNC Symbol;Acc:HGNC:11785]	12923	-1.339050055	0.008698457

ESTROGEN RESPONSE EARLY

180	-0.2764127	0.0021322	0.1810923	0.809	3753	MYC	MYC proto-oncogene, bHLH transcription factor [Source:HGNC Symbol;Acc:HGNC:7553]	10049	-0.402550012	-0.27243876
						SEMA3B	semaphorin 3B [Source:HGNC Symbol;Acc:HGNC:10724]	10099	-0.406170011	-0.27202648
						CD44	CD44 molecule (Indian blood group) [Source:HGNC Symbol;Acc:HGNC:1681]	10110	-0.406966656	-0.2687431
						SLC26A2	solute carrier family 26 member 2 [Source:HGNC Symbol;Acc:HGNC:10994]	10157	-0.411303341	-0.2680599
						ELOVL5	ELOVL fatty acid elongase 5 [Source:HGNC Symbol;Acc:HGNC:21308]	10206	-0.414933324	-0.2674877
						JAK2	Janus kinase 2 [Source:HGNC Symbol;Acc:HGNC:6192]	10262	-0.420686662	-0.2673726
						UGCG	UDP-glucose ceramide glucosyltransferase [Source:HGNC Symbol;Acc:HGNC:12524]	10270	-0.421350002	-0.263727
						ITPK1	inositol-tetrakisphosphate 1-kinase [Source:HGNC Symbol;Acc:HGNC:6177]	10316	-0.425469995	-0.26283053
						SLC16A1	solute carrier family 16 member 1 [Source:HGNC Symbol;Acc:HGNC:10922]	10318	-0.425500005	-0.25870344
						SVIL	supervillin [Source:HGNC Symbol;Acc:HGNC:11480]	10370	-0.428900003	-0.2582136
						MED13L	mediator complex subunit 13L [Source:HGNC Symbol;Acc:HGNC:22962]	10404	-0.43228668	-0.25636885
						SYNGR1	synaptogyrin 1 [Source:HGNC Symbol;Acc:HGNC:11498]	10546	-0.445410013	-0.26232347
						SLC7A5	solute carrier family 7 member 5 [Source:HGNC Symbol;Acc:HGNC:11063]	10620	-0.45243001	-0.2632165
						ABCA3	ATP binding cassette subfamily A member 3 [Source:HGNC Symbol;Acc:HGNC:33]	10744	-0.465623319	-0.2676501
						XBP1	X-box binding protein 1 [Source:HGNC Symbol;Acc:HGNC:12801]	10809	-0.472189993	-0.26768732
						KRT13	keratin 13 [Source:HGNC Symbol;Acc:HGNC:6415]	10817	-0.473286659	-0.263529
						MUC1	mucin 1, cell surface associated [Source:HGNC Symbol;Acc:HGNC:7508]	10825	-0.474370003	-0.25936
						IL6ST	interleukin 6 signal transducer [Source:HGNC Symbol;Acc:HGNC:6021]	10881	-0.480626673	-0.25865316
						ASB13	ankyrin repeat and SOCS box containing 13 [Source:HGNC Symbol;Acc:HGNC:19765]	10904	-0.483839989	-0.25549188
						GJA1	gap junction protein alpha 1 [Source:HGNC Symbol;Acc:HGNC:4274]	10939	-0.48699668	-0.25318047
						AFF1	AF4/FMR2 family member 1 [Source:HGNC Symbol;Acc:HGNC:7135]	10948	-0.48791334	-0.24895117
						HSPB8	heat shock protein family B (small) member 8 [Source:HGNC Symbol;Acc:HGNC:30171]	10971	-0.490483344	-0.24572432
						RASGRP1	RAS guanyl releasing protein 1 [Source:HGNC Symbol;Acc:HGNC:9878]	10982	-0.491793334	-0.24160354
						PDLIM3	PDZ and LIM domain 3 [Source:HGNC Symbol;Acc:HGNC:20767]	10992	-0.493473321	-0.23739277
						ELF1	E74 like ETS transcription factor 1 [Source:HGNC Symbol;Acc:HGNC:3316]	11136	-0.50835669	-0.24287282
						KRT8	keratin 8 [Source:HGNC Symbol;Acc:HGNC:6446]	11161	-0.510523319	-0.23959498
						FLNB	filamin B [Source:HGNC Symbol;Acc:HGNC:3755]	11194	-0.514453351	-0.23686565
						ESRP2	epithelial splicing regulatory protein 2 [Source:HGNC Symbol;Acc:HGNC:26152]	11222	-0.516666651	-0.23374742
						CCND1	cyclin D1 [Source:HGNC Symbol;Acc:HGNC:1582]	11279	-0.523143351	-0.2326943
						CHPT1	choline phosphotransferase 1 [Source:HGNC Symbol;Acc:HGNC:17852]	11371	-0.533116639	-0.23411229
						SLC7A2	solute carrier family 7 member 2 [Source:HGNC Symbol;Acc:HGNC:11060]	11422	-0.539099991	-0.23246115
						CISH	cytokine inducible SH2 containing protein [Source:HGNC Symbol;Acc:HGNC:1984]	11496	-0.546786666	-0.23242271
						KCNK5	potassium two pore domain channel subfamily K member 5 [Source:HGNC Symbol;Acc:HGNC:6280]	11565	-0.557393312	-0.23191246
						ELF3	E74 like ETS transcription factor 3 [Source:HGNC Symbol;Acc:HGNC:3318]	11613	-0.564086676	-0.22979441
						ENDOD1	endonuclease domain containing 1 [Source:HGNC Symbol;Acc:HGNC:29129]	11639	-0.568186641	-0.22602074
						NCOR2	nuclear receptor corepressor 2 [Source:HGNC Symbol;Acc:HGNC:7673]	11646	-0.569563329	-0.22083858
						AMFR	autocrine motility factor receptor [Source:HGNC Symbol;Acc:HGNC:463]	11736	-0.582256675	-0.22162463
						KRT18	keratin 18 [Source:HGNC Symbol;Acc:HGNC:6430]	11793	-0.588786662	-0.21992348
						SCARB1	scavenger receptor class B member 1 [Source:HGNC Symbol;Acc:HGNC:1664]	11804	-0.58987999	-0.2148344
						OVOL2	ovo like zinc finger 2 [Source:HGNC Symbol;Acc:HGNC:15804]	11816	-0.591350019	-0.20980424
						TOB1	transducer of ERBB2, 1 [Source:HGNC Symbol;Acc:HGNC:11979]	11860	-0.598316669	-0.20705462
						WWC1	WW and C2 domain containing 1 [Source:HGNC Symbol;Acc:HGNC:29435]	11888	-0.602140009	-0.20309259
						PODXL	podocalyxin like [Source:HGNC Symbol;Acc:HGNC:9171]	11996	-0.619336665	-0.20483407
						HR	HR lysine demethylase and nuclear receptor corepressor [Source:HGNC Symbol;Acc:HGNC:5172]	12013	-0.621636689	-0.199872
						HES1	hes family bHLH transcription factor 1 [Source:HGNC Symbol;Acc:HGNC:5192]	12038	-0.625566661	-0.19545846
						CELSR1	cadherin EGF LAG seven-pass G-type receptor 1 [Source:HGNC Symbol;Acc:HGNC:1850]	12101	-0.636856675	-0.19372328
						SLC19A2	solute carrier family 19 member 2 [Source:HGNC Symbol;Acc:HGNC:10938]	12255	-0.663576663	-0.19840516
						TGM2	transglutaminase 2 [Source:HGNC Symbol;Acc:HGNC:11778]	12455	-0.703793347	-0.20606719
						TTC39A	tetratricopeptide repeat domain 39A [Source:HGNC Symbol;Acc:HGNC:18657]	12507	-0.715803325	-0.20274507
						TJP3	tight junction protein 3 [Source:HGNC Symbol;Acc:HGNC:11829]	12560	-0.72755003	-0.19938041
						MYOF	myoferlin [Source:HGNC Symbol;Acc:HGNC:3656]	12669	-0.756356657	-0.19984268
						TPD52L1	TPD52 like 1 [Source:HGNC Symbol;Acc:HGNC:12006]	12701	-0.76572001	-0.19455945
						PEX11A	peroxisomal biogenesis factor 11 alpha [Source:HGNC Symbol;Acc:HGNC:8852]	12712	-0.767356694	-0.18771836
						SEC14L2	SEC14 like lipid binding 2 [Source:HGNC Symbol;Acc:HGNC:10699]	12730	-0.771013319	-0.18135507
						MYB	MYB proto-oncogene, transcription factor [Source:HGNC Symbol;Acc:HGNC:7545]	12824	-0.798860013	-0.1802965
						AQP3	aquaporin 3 (Gill blood group) [Source:HGNC Symbol;Acc:HGNC:636]	12871	-0.813236654	-0.17564546
						FKBP4	FKBP prolyl isomerase 4 [Source:HGNC Symbol;Acc:HGNC:3720]	12954	-0.837339997	-0.17339943
						NPY1R	neuropeptide Y receptor Y1 [Source:HGNC Symbol;Acc:HGNC:7956]	12978	-0.84874332	-0.1667093
						EGR3	early growth response 3 [Source:HGNC Symbol;Acc:HGNC:3240]	12998	-0.856859982	-0.15964538
						INHBB	inhibin subunit beta B [Source:HGNC Symbol;Acc:HGNC:6067]	13034	-0.871930003	-0.15360734
						TSKU	tsukushi, small leucine rich proteoglycan [Source:HGNC Symbol;Acc:HGNC:28850]	13056	-0.884750009	-0.14641492
						ABHD2	abhydrolase domain containing 2, acylglycerol lipase [Source:HGNC Symbol;Acc:HGNC:18717]	13064	-0.889653325	-0.13814627
						ARL3	ADP ribosylation factor like GTPase 3 [Source:HGNC Symbol;Acc:HGNC:694]	13088	-0.903336644	-0.13091719
						MLPH	melanophilin [Source:HGNC Symbol;Acc:HGNC:29643]	13105	-0.909966648	-0.12310875
						CBFA2T3	CBFA2/RUNX1 partner transcriptional co-repressor 3 [Source:HGNC Symbol;Acc:HGNC:1537]	13181	-0.943426669	-0.11930155
						ADCY9	adenylate cyclase 9 [Source:HGNC Symbol;Acc:HGNC:240]	13205	-0.951063335	-0.11160132
						THSD4	thrombospondin type 1 domain containing 4 [Source:HGNC Symbol;Acc:HGNC:25835]	13254	-0.975826681	-0.10549203
						FCMR	Fc fragment of IgM receptor [Source:HGNC Symbol;Acc:HGNC:14315]	13303	-1.004036665	-0.099104255
						GFRA1	GDNF family receptor alpha 1 [Source:HGNC Symbol;Acc:HGNC:4243]	13416	-1.103899956	-0.09642927
						FKBP5	FKBP prolyl isomerase 5 [Source:HGNC Symbol;Acc:HGNC:3721]	13423	-1.113193393	-0.08588044
						FOS	Fos proto-oncogene, AP-1 transcription factor subunit [Source:HGNC Symbol;Acc:HGNC:3796]	13466	-1.162256718	-0.07749024
						FHL2	four and a half LIM domains 2 [Source:HGNC Symbol;Acc:HGNC:3703]	13557	-1.254879951	-0.07170964
						FASN	fatty acid synthase [Source:HGNC Symbol;Acc:HGNC:3594]	13637	-1.428286672	-0.06340962
						SFN	stratifin [Source:HGNC Symbol;Acc:HGNC:10773]	13738	-1.902033329	-0.05197455
						AREG	amphiregulin [Source:HGNC Symbol;Acc:HGNC:651]	13742	-1.94825995	-0.032961782
						CYP26B1	cytochrome P450 family 26 subfamily B member 1 [Source:HGNC Symbol;Acc:HGNC:20581]	13794	-3.762840033	0.00044036

166	-0.29509932	0	0.1908424	0.662	3441	MYC	MYC proto-oncogene, bHLH transcription factor [Source:HGNC Symbol;Acc:HGNC:7553]	9683	-0.402550012	-0.29046607
						SEMA3B	semaphorin 3B [Source:HGNC Symbol;Acc:HGNC:10724]	9733	-0.406170011	-0.2895729
						CD44	CD44 molecule (Indian blood group) [Source:HGNC Symbol;Acc:HGNC:1681]	9744	-0.406966656	-0.2856606
						SLC26A2	solute carrier family 26 member 2 [Source:HGNC Symbol;Acc:HGNC:10994]	9791	-0.411303341	-0.28447682
						ELOVL5	ELOVL fatty acid elongase 5 [Source:HGNC Symbol;Acc:HGNC:21308]	9841	-0.414933324	-0.28348276
						JAK2	Janus kinase 2 [Source:HGNC Symbol;Acc:HGNC:6192]	9897	-0.420686662	-0.28288558
						UGCG	UDP-glucose ceramide glucosyltransferase [Source:HGNC Symbol;Acc:HGNC:12524]	9905	-0.421350002	-0.2785762
						ITPK1	inositol-tetrakisphosphate 1-kinase [Source:HGNC Symbol;Acc:HGNC:6177]	9952	-0.425469995	-0.27722934
						SLC16A1	solute carrier family 16 member 1 [Source:HGNC Symbol;Acc:HGNC:10922]	9954	-0.425500005	-0.27240914
						SVIL	supervillin [Source:HGNC Symbol;Acc:HGNC:11480]	10006	-0.428900003	-0.2714087
						MED13L	mediator complex subunit 13L [Source:HGNC Symbol;Acc:HGNC:22962]	10040	-0.43228668	-0.2689801
						SYNGR1	synaptogyrin 1 [Source:HGNC Symbol;Acc:HGNC:11498]	10182	-0.445410013	-0.27473566
						SLC7A5	solute carrier family 7 member 5 [Source:HGNC Symbol;Acc:HGNC:11063]	10256	-0.45243001	-0.27516234
						ABCA3	ATP binding cassette subfamily A member 3 [Source:HGNC Symbol;Acc:HGNC:33]	10380	-0.465623319	-0.27929607
						XBP1	X-box binding protein 1 [Source:HGNC Symbol;Acc:HGNC:12801]	10444	-0.472189993	-0.2787235
						KRT13	keratin 13 [Source:HGNC Symbol;Acc:HGNC:6415]	10452	-0.473286659	-0.27381635
						MUC1	mucin 1, cell surface associated [Source:HGNC Symbol;Acc:HGNC:7508]	10460	-0.474370003	-0.26889673
						IL6ST	interleukin 6 signal transducer [Source:HGNC Symbol;Acc:HGNC:6021]	10516	-0.480626673	-0.26760966
						ASB13	ankyrin repeat and SOCS box containing 13 [Source:HGNC Symbol;Acc:HGNC:19765]	10539	-0.483839989	-0.2637387
						GJA1	gap junction protein alpha 1 [Source:HGNC Symbol;Acc:HGNC:4274]	10574	-0.48699668	-0.26075757
						AFF1	AF4/FMR2 family member 1 [Source:HGNC Symbol;Acc:HGNC:7135]	10583	-0.48791334	-0.2557592
						HSPB8	heat shock protein family B (small) member 8 [Source:HGNC Symbol;Acc:HGNC:30171]	10607	-0.490483344	-0.251889
						RASGRP1	RAS guanyl releasing protein 1 [Source:HGNC Symbol;Acc:HGNC:9878]	10618	-0.491793334	-0.24700035
						PDLIM3	PDZ and LIM domain 3 [Source:HGNC Symbol;Acc:HGNC:20767]	10628	-0.493473321	-0.2420152
						ELF1	E74 like ETS transcription factor 1 [Source:HGNC Symbol;Acc:HGNC:3316]	10773	-0.50835669	-0.24727783
						KRT8	keratin 8 [Source:HGNC Symbol;Acc:HGNC:6446]	10798	-0.510523319	-0.24325413
						FLNB	filamin B [Source:HGNC Symbol;Acc:HGNC:3755]	10831	-0.514453351	-0.2398026
						ESRP2	epithelial splicing regulatory protein 2 [Source:HGNC Symbol;Acc:HGNC:26152]	10859	-0.516666651	-0.23593971
						CCND1	cyclin D1 [Source:HGNC Symbol;Acc:HGNC:1582]	10916	-0.523143351	-0.23424046
						CHPT1	choline phosphotransferase 1 [Source:HGNC Symbol;Acc:HGNC:17852]	11003	-0.533116639	-0.23474176
						SLC7A2	solute carrier family 7 member 2 [Source:HGNC Symbol;Acc:HGNC:11060]	11052	-0.539099991	-0.23224142
						CISH	cytokine inducible SH2 containing protein [Source:HGNC Symbol;Acc:HGNC:1984]	11125	-0.546786666	-0.23150489
						KCNK5	potassium two pore domain channel subfamily K member 5 [Source:HGNC Symbol;Acc:HGNC:6280]	11194	-0.557393312	-0.23033756
						ELF3	E74 like ETS transcription factor 3 [Source:HGNC Symbol;Acc:HGNC:3318]	11242	-0.564086676	-0.22747244
						ENDOD1	endonuclease domain containing 1 [Source:HGNC Symbol;Acc:HGNC:29129]	11267	-0.568186641	-0.22278504
						NCOR2	nuclear receptor corepressor 2 [Source:HGNC Symbol;Acc:HGNC:7673]	11275	-0.569563329	-0.21676977
						AMFR	autocrine motility factor receptor [Source:HGNC Symbol;Acc:HGNC:463]	11364	-0.582256675	-0.21685983
						KRT18	keratin 18 [Source:HGNC Symbol;Acc:HGNC:6430]	11419	-0.588786662	-0.21425068
						SCARB1	scavenger receptor class B member 1 [Source:HGNC Symbol;Acc:HGNC:1664]	11430	-0.58987999	-0.20823309
						OVOL2	ovo like zinc finger 2 [Source:HGNC Symbol;Acc:HGNC:15804]	11441	-0.591350019	-0.2021986
						TOB1	transducer of ERBB2, 1 [Source:HGNC Symbol;Acc:HGNC:11979]	11485	-0.598316669	-0.19863078
						WWC1	WW and C2 domain containing 1 [Source:HGNC Symbol;Acc:HGNC:29435]	11511	-0.602140009	-0.19362977
						PODXL	podocalyxin like [Source:HGNC Symbol;Acc:HGNC:9171]	11612	-0.619336665	-0.1942192
						HR	HR lysine demethylase and nuclear receptor corepressor [Source:HGNC Symbol;Acc:HGNC:5172]	11628	-0.621636689	-0.18822199
						HES1	hes family bHLH transcription factor 1 [Source:HGNC Symbol;Acc:HGNC:5192]	11651	-0.625566661	-0.1827198
						CELSR1	cadherin EGF LAG seven-pass G-type receptor 1 [Source:HGNC Symbol;Acc:HGNC:1850]	11707	-0.636856675	-0.17963456
						SLC19A2	solute carrier family 19 member 2 [Source:HGNC Symbol;Acc:HGNC:10938]	11858	-0.663576663	-0.18357371
						TGM2	transglutaminase 2 [Source:HGNC Symbol;Acc:HGNC:11778]	12036	-0.703793347	-0.1891338
						TTC39A	tetratricopeptide repeat domain 39A [Source:HGNC Symbol;Acc:HGNC:18657]	12081	-0.715803325	-0.18429093
						TJP3	tight junction protein 3 [Source:HGNC Symbol;Acc:HGNC:11829]	12129	-0.72755003	-0.1795444
						MYOF	myoferlin [Source:HGNC Symbol;Acc:HGNC:3656]	12232	-0.756356657	-0.17871112
						TPD52L1	TPD52 like 1 [Source:HGNC Symbol;Acc:HGNC:12006]	12259	-0.76572001	-0.17190452
						PEX11A	peroxisomal biogenesis factor 11 alpha [Source:HGNC Symbol;Acc:HGNC:8852]	12271	-0.767356694	-0.1639214
						SEC14L2	SEC14 like lipid binding 2 [Source:HGNC Symbol;Acc:HGNC:10699]	12287	-0.771013319	-0.15620491
						MYB	MYB proto-oncogene, transcription factor [Source:HGNC Symbol;Acc:HGNC:7545]	12367	-0.798860013	-0.15310732
						AQP3	aquaporin 3 (Gill blood group) [Source:HGNC Symbol;Acc:HGNC:636]	12413	-0.813236654	-0.1472202
						NPY1R	neuropeptide Y receptor Y1 [Source:HGNC Symbol;Acc:HGNC:7956]	12502	-0.84874332	-0.14424308
						EGR3	early growth response 3 [Source:HGNC Symbol;Acc:HGNC:3240]	12520	-0.856859982	-0.13569286
						TSKU	tsukushi, small leucine rich proteoglycan [Source:HGNC Symbol;Acc:HGNC:28850]	12572	-0.884750009	-0.12944572
						ABHD2	abhydrolase domain containing 2, acylglycerol lipase [Source:HGNC Symbol;Acc:HGNC:18717]	12580	-0.889653325	-0.11974628
						ADCY9	adenylate cyclase 9 [Source:HGNC Symbol;Acc:HGNC:240]	12691	-0.951063335	-0.11728941
						THSD4	thrombospondin type 1 domain containing 4 [Source:HGNC Symbol;Acc:HGNC:25835]	12731	-0.975826681	-0.10906784
						FCMR	Fc fragment of IgM receptor [Source:HGNC Symbol;Acc:HGNC:14315]	12773	-1.004036665	-0.100675955
						GFRA1	GDNF family receptor alpha 1 [Source:HGNC Symbol;Acc:HGNC:4243]	12864	-1.103899956	-0.094916396
						FKBP5	FKBP prolyl isomerase 5 [Source:HGNC Symbol;Acc:HGNC:3721]	12869	-1.113193393	-0.08241254
						FOS	Fos proto-oncogene, AP-1 transcription factor subunit [Source:HGNC Symbol;Acc:HGNC:3796]	12902	-1.162256718	-0.071504965
						FHL2	four and a half LIM domains 2 [Source:HGNC Symbol;Acc:HGNC:3703]	12964	-1.254879951	-0.0617695
						SCNN1A	sodium channel epithelial 1 subunit alpha [Source:HGNC Symbol;Acc:HGNC:10599]	12983	-1.304049969	-0.048149426
						FASN	fatty acid synthase [Source:HGNC Symbol;Acc:HGNC:3594]	13024	-1.428286672	-0.034797344
						KLF4	Kruppel like factor 4 [Source:HGNC Symbol;Acc:HGNC:6348]	13074	-1.718729973	-0.01879694
						AREG	amphiregulin [Source:HGNC Symbol;Acc:HGNC:651]	13094	-1.94825995	0.002160647

165	-0.27100617	0	0.1484809	0.822	3399	MYC	MYC proto-oncogene, bHLH transcription factor [Source:HGNC Symbol;Acc:HGNC:7553]	9639	-0.402550012	-0.26617125
						SEMA3B	semaphorin 3B [Source:HGNC Symbol;Acc:HGNC:10724]	9689	-0.406170011	-0.26509953
						CD44	CD44 molecule (Indian blood group) [Source:HGNC Symbol;Acc:HGNC:1681]	9700	-0.406966656	-0.2609884
						SLC26A2	solute carrier family 26 member 2 [Source:HGNC Symbol;Acc:HGNC:10994]	9747	-0.411303341	-0.25962198
						ELOVL5	ELOVL fatty acid elongase 5 [Source:HGNC Symbol;Acc:HGNC:21308]	9797	-0.414933324	-0.25844502
						JAK2	Janus kinase 2 [Source:HGNC Symbol;Acc:HGNC:6192]	9853	-0.420686662	-0.25766507
						UGCG	UDP-glucose ceramide glucosyltransferase [Source:HGNC Symbol;Acc:HGNC:12524]	9861	-0.421350002	-0.25314814
						ITPK1	inositol-tetrakisphosphate 1-kinase [Source:HGNC Symbol;Acc:HGNC:6177]	9907	-0.425469995	-0.25153387
						SLC16A1	solute carrier family 16 member 1 [Source:HGNC Symbol;Acc:HGNC:10922]	9909	-0.425500005	-0.24650097
						SVIL	supervillin [Source:HGNC Symbol;Acc:HGNC:11480]	9961	-0.428900003	-0.24531163
						MED13L	mediator complex subunit 13L [Source:HGNC Symbol;Acc:HGNC:22962]	9995	-0.43228668	-0.24268323
						SYNGR1	synaptogyrin 1 [Source:HGNC Symbol;Acc:HGNC:11498]	10137	-0.445410013	-0.24828751
						SLC7A5	solute carrier family 7 member 5 [Source:HGNC Symbol;Acc:HGNC:11063]	10211	-0.45243001	-0.2485247
						ABCA3	ATP binding cassette subfamily A member 3 [Source:HGNC Symbol;Acc:HGNC:33]	10335	-0.465623319	-0.25248784
						XBP1	X-box binding protein 1 [Source:HGNC Symbol;Acc:HGNC:12801]	10400	-0.472189993	-0.25178847
						KRT13	keratin 13 [Source:HGNC Symbol;Acc:HGNC:6415]	10408	-0.473286659	-0.24664775
						MUC1	mucin 1, cell surface associated [Source:HGNC Symbol;Acc:HGNC:7508]	10416	-0.474370003	-0.24149401
						IL6ST	interleukin 6 signal transducer [Source:HGNC Symbol;Acc:HGNC:6021]	10473	-0.480626673	-0.24007183
						ASB13	ankyrin repeat and SOCS box containing 13 [Source:HGNC Symbol;Acc:HGNC:19765]	10496	-0.483839989	-0.23596966
						GJA1	gap junction protein alpha 1 [Source:HGNC Symbol;Acc:HGNC:4274]	10532	-0.48699668	-0.23283954
						AFF1	AF4/FMR2 family member 1 [Source:HGNC Symbol;Acc:HGNC:7135]	10542	-0.48791334	-0.22767851
						HSPB8	heat shock protein family B (small) member 8 [Source:HGNC Symbol;Acc:HGNC:30171]	10565	-0.490483344	-0.22349654
						RASGRP1	RAS guanyl releasing protein 1 [Source:HGNC Symbol;Acc:HGNC:9878]	10576	-0.491793334	-0.2183666
						PDLIM3	PDZ and LIM domain 3 [Source:HGNC Symbol;Acc:HGNC:20767]	10586	-0.493473321	-0.21313879
						ELF1	E74 like ETS transcription factor 1 [Source:HGNC Symbol;Acc:HGNC:3316]	10729	-0.50835669	-0.21806473
						KRT8	keratin 8 [Source:HGNC Symbol;Acc:HGNC:6446]	10754	-0.510523319	-0.21379745
						FLNB	filamin B [Source:HGNC Symbol;Acc:HGNC:3755]	10787	-0.514453351	-0.21010447
						ESRP2	epithelial splicing regulatory protein 2 [Source:HGNC Symbol;Acc:HGNC:26152]	10815	-0.516666651	-0.20599647
						CCND1	cyclin D1 [Source:HGNC Symbol;Acc:HGNC:1582]	10872	-0.523143351	-0.20406364
						CHPT1	choline phosphotransferase 1 [Source:HGNC Symbol;Acc:HGNC:17852]	10957	-0.533116639	-0.20418628
						SLC7A2	solute carrier family 7 member 2 [Source:HGNC Symbol;Acc:HGNC:11060]	11006	-0.539099991	-0.20144029
						CISH	cytokine inducible SH2 containing protein [Source:HGNC Symbol;Acc:HGNC:1984]	11079	-0.546786666	-0.20046648
						KCNK5	potassium two pore domain channel subfamily K member 5 [Source:HGNC Symbol;Acc:HGNC:6280]	11147	-0.557393312	-0.19897684
						ELF3	E74 like ETS transcription factor 3 [Source:HGNC Symbol;Acc:HGNC:3318]	11195	-0.564086676	-0.19585305
						ENDOD1	endonuclease domain containing 1 [Source:HGNC Symbol;Acc:HGNC:29129]	11221	-0.568186641	-0.19097088
						NCOR2	nuclear receptor corepressor 2 [Source:HGNC Symbol;Acc:HGNC:7673]	11228	-0.569563329	-0.1845961
						AMFR	autocrine motility factor receptor [Source:HGNC Symbol;Acc:HGNC:463]	11317	-0.582256675	-0.18443929
						KRT18	keratin 18 [Source:HGNC Symbol;Acc:HGNC:6430]	11371	-0.588786662	-0.18148495
						SCARB1	scavenger receptor class B member 1 [Source:HGNC Symbol;Acc:HGNC:1664]	11382	-0.58987999	-0.17517692
						OVOL2	ovo like zinc finger 2 [Source:HGNC Symbol;Acc:HGNC:15804]	11392	-0.591350019	-0.16877353
						TOB1	transducer of ERBB2, 1 [Source:HGNC Symbol;Acc:HGNC:11979]	11437	-0.598316669	-0.16500555
						WWC1	WW and C2 domain containing 1 [Source:HGNC Symbol;Acc:HGNC:29435]	11464	-0.602140009	-0.15979326
						PODXL	podocalyxin like [Source:HGNC Symbol;Acc:HGNC:9171]	11565	-0.619336665	-0.16012333
						HR	HR lysine demethylase and nuclear receptor corepressor [Source:HGNC Symbol;Acc:HGNC:5172]	11581	-0.621636689	-0.1538223
						HES1	hes family bHLH transcription factor 1 [Source:HGNC Symbol;Acc:HGNC:5192]	11605	-0.625566661	-0.14809558
						CELSR1	cadherin EGF LAG seven-pass G-type receptor 1 [Source:HGNC Symbol;Acc:HGNC:1850]	11661	-0.636856675	-0.14471927
						SLC19A2	solute carrier family 19 member 2 [Source:HGNC Symbol;Acc:HGNC:10938]	11806	-0.663576663	-0.14793627
						TGM2	transglutaminase 2 [Source:HGNC Symbol;Acc:HGNC:11778]	11981	-0.703793347	-0.15300086
						TTC39A	tetratricopeptide repeat domain 39A [Source:HGNC Symbol;Acc:HGNC:18657]	12027	-0.715803325	-0.14789946
						TJP3	tight junction protein 3 [Source:HGNC Symbol;Acc:HGNC:11829]	12075	-0.72755003	-0.14281234
						MYOF	myoferlin [Source:HGNC Symbol;Acc:HGNC:3656]	12178	-0.756356657	-0.14165208
						TPD52L1	TPD52 like 1 [Source:HGNC Symbol;Acc:HGNC:12006]	12205	-0.76572001	-0.13447507
						PEX11A	peroxisomal biogenesis factor 11 alpha [Source:HGNC Symbol;Acc:HGNC:8852]	12216	-0.767356694	-0.12603539
						SEC14L2	SEC14 like lipid binding 2 [Source:HGNC Symbol;Acc:HGNC:10699]	12231	-0.771013319	-0.117862545
						MYB	MYB proto-oncogene, transcription factor [Source:HGNC Symbol;Acc:HGNC:7545]	12311	-0.798860013	-0.11440496
						AQP3	aquaporin 3 (Gill blood group) [Source:HGNC Symbol;Acc:HGNC:636]	12356	-0.813236654	-0.108055614
						FKBP4	FKBP prolyl isomerase 4 [Source:HGNC Symbol;Acc:HGNC:3720]	12425	-0.837339997	-0.10328129
						NPY1R	neuropeptide Y receptor Y1 [Source:HGNC Symbol;Acc:HGNC:7956]	12447	-0.84874332	-0.09471866
						EGR3	early growth response 3 [Source:HGNC Symbol;Acc:HGNC:3240]	12463	-0.856859982	-0.08559241
						TSKU	tsukushi, small leucine rich proteoglycan [Source:HGNC Symbol;Acc:HGNC:28850]	12514	-0.884750009	-0.07885027
						ABHD2	abhydrolase domain containing 2, acylglycerol lipase [Source:HGNC Symbol;Acc:HGNC:18717]	12521	-0.889653325	-0.068630956
						ADCY9	adenylate cyclase 9 [Source:HGNC Symbol;Acc:HGNC:240]	12631	-0.951063335	-0.06567593
						THSD4	thrombospondin type 1 domain containing 4 [Source:HGNC Symbol;Acc:HGNC:25835]	12668	-0.975826681	-0.056752246
						FCMR	Fc fragment of IgM receptor [Source:HGNC Symbol;Acc:HGNC:14315]	12709	-1.004036665	-0.047800496
						GFRA1	GDNF family receptor alpha 1 [Source:HGNC Symbol;Acc:HGNC:4243]	12797	-1.103899956	-0.041300643
						FKBP5	FKBP prolyl isomerase 5 [Source:HGNC Symbol;Acc:HGNC:3721]	12801	-1.113193393	-0.028163375
						FOS	Fos proto-oncogene, AP-1 transcription factor subunit [Source:HGNC Symbol;Acc:HGNC:3796]	12834	-1.162256718	-0.01668977
						FHL2	four and a half LIM domains 2 [Source:HGNC Symbol;Acc:HGNC:3703]	12895	-1.254879951	-0.006278953
						FASN	fatty acid synthase [Source:HGNC Symbol;Acc:HGNC:3594]	12952	-1.428286672	0.006525369

ADIPOGENESIS

180	-0.24758242	0.00398406	0.200774	0.957	2607	CHUK	component of inhibitor of nuclear factor kappa B kinase complex [Source:HGNC Symbol;Acc:HGNC:1974]	10517	-0.480789989	-0.24229594
						ACOX1	acyl-CoA oxidase 1 [Source:HGNC Symbol;Acc:HGNC:119]	10571	-0.486626655	-0.24104016
						HSPB8	heat shock protein family B (small) member 8 [Source:HGNC Symbol;Acc:HGNC:30171]	10607	-0.490483344	-0.23835126
						ADCY6	adenylate cyclase 6 [Source:HGNC Symbol;Acc:HGNC:237]	10636	-0.494190007	-0.23508078
						HIBCH	3-hydroxyisobutyryl-CoA hydrolase [Source:HGNC Symbol;Acc:HGNC:4908]	10650	-0.495730013	-0.23063442
						STOM	stomatin [Source:HGNC Symbol;Acc:HGNC:3383]	10777	-0.50869	-0.23477617
						CAVIN2	caveolae associated protein 2 [Source:HGNC Symbol;Acc:HGNC:10690]	10890	-0.520386696	-0.23770763
						IDH1	isocitrate dehydrogenase (NADP(+)) 1 [Source:HGNC Symbol;Acc:HGNC:5382]	10906	-0.522189975	-0.23312487
						ALDH2	aldehyde dehydrogenase 2 family member [Source:HGNC Symbol;Acc:HGNC:404]	10909	-0.522289991	-0.2275366
						MIGA2	mitoguardin 2 [Source:HGNC Symbol;Acc:HGNC:23621]	10970	-0.529330015	-0.22635211
						DDT	D-dopachrome tautomerase [Source:HGNC Symbol;Acc:HGNC:2732]	11095	-0.54423666	-0.22994848
						SLC19A1	solute carrier family 19 member 1 [Source:HGNC Symbol;Acc:HGNC:10937]	11144	-0.549516678	-0.2276149
						YWHAG	tyrosine 3-monooxygenase/tryptophan 5-monooxygenase activation protein gamma [Source:HGNC Symbol;Acc:HGNC:12852]	11247	-0.564576685	-0.22928785
						REEP6	receptor accessory protein 6 [Source:HGNC Symbol;Acc:HGNC:30078]	11249	-0.56480664	-0.22315483
						SCARB1	scavenger receptor class B member 1 [Source:HGNC Symbol;Acc:HGNC:1664]	11430	-0.58987999	-0.230576
						ABCA1	ATP binding cassette subfamily A member 1 [Source:HGNC Symbol;Acc:HGNC:29]	11444	-0.59187001	-0.22507255
						ECHS1	enoyl-CoA hydratase, short chain 1 [Source:HGNC Symbol;Acc:HGNC:3151]	11451	-0.592966676	-0.21901621
						TOB1	transducer of ERBB2, 1 [Source:HGNC Symbol;Acc:HGNC:11979]	11485	-0.598316669	-0.21498711
						ACADL	acyl-CoA dehydrogenase long chain [Source:HGNC Symbol;Acc:HGNC:88]	11539	-0.606243312	-0.2124161
						CHCHD10	coiled-coil-helix-coiled-coil-helix domain containing 10 [Source:HGNC Symbol;Acc:HGNC:15559]	11592	-0.615906656	-0.20966157
						IDH3A	isocitrate dehydrogenase (NAD(+)) 3 catalytic subunit alpha [Source:HGNC Symbol;Acc:HGNC:5384]	11643	-0.624883354	-0.20665382
						ADIPOR2	adiponectin receptor 2 [Source:HGNC Symbol;Acc:HGNC:24041]	11715	-0.638066649	-0.20512362
						PIM3	Pim-3 proto-oncogene, serine/threonine kinase [Source:HGNC Symbol;Acc:HGNC:19310]	11765	-0.646336675	-0.20180272
						CYP4B1	cytochrome P450 family 4 subfamily B member 1 [Source:HGNC Symbol;Acc:HGNC:2644]	11810	-0.65490669	-0.19800127
						ITGA7	integrin subunit alpha 7 [Source:HGNC Symbol;Acc:HGNC:6143]	11862	-0.664619982	-0.19463386
						SLC5A6	solute carrier family 5 member 6 [Source:HGNC Symbol;Acc:HGNC:11041]	11875	-0.666823328	-0.18822901
						MRAP	melanocortin 2 receptor accessory protein [Source:HGNC Symbol;Acc:HGNC:1304]	11963	-0.687573314	-0.18739064
						CYC1	cytochrome c1 [Source:HGNC Symbol;Acc:HGNC:2579]	11965	-0.68871665	-0.17989518
						SLC1A5	solute carrier family 1 member 5 [Source:HGNC Symbol;Acc:HGNC:10943]	12048	-0.706960022	-0.17845735
						CD36	CD36 molecule [Source:HGNC Symbol;Acc:HGNC:1663]	12250	-0.760673344	-0.18562306
						UCK1	uridine-cytidine kinase 1 [Source:HGNC Symbol;Acc:HGNC:14859]	12282	-0.76976335	-0.17955431
						FZD4	frizzled class receptor 4 [Source:HGNC Symbol;Acc:HGNC:4042]	12305	-0.777960002	-0.17270009
						ESYT1	extended synaptotagmin 1 [Source:HGNC Symbol;Acc:HGNC:29534]	12388	-0.804939985	-0.17018493
						C3	complement C3 [Source:HGNC Symbol;Acc:HGNC:1318]	12424	-0.81736666	-0.1639018
						COQ9	coenzyme Q9 [Source:HGNC Symbol;Acc:HGNC:25302]	12444	-0.823176682	-0.15631863
						RETSAT	retinol saturase [Source:HGNC Symbol;Acc:HGNC:25991]	12493	-0.843850017	-0.15074873
						SLC27A1	solute carrier family 27 member 1 [Source:HGNC Symbol;Acc:HGNC:10995]	12535	-0.86596334	-0.14439484
						UCP2	uncoupling protein 2 [Source:HGNC Symbol;Acc:HGNC:12518]	12687	-0.949923337	-0.14561659
						BCL2L13	BCL2 like 13 [Source:HGNC Symbol;Acc:HGNC:17164]	12762	-0.997200012	-0.14036936
						SUCLG1	succinate-CoA ligase GDP/ADP-forming subunit alpha [Source:HGNC Symbol;Acc:HGNC:11449]	12772	-1.002529979	-0.13004148
						PFKFB3	6-phosphofructo-2-kinase/fructose-2,6-biphosphatase 3 [Source:HGNC Symbol;Acc:HGNC:8874]	12776	-1.006226659	-0.1192094
						EPHX2	epoxide hydrolase 2 [Source:HGNC Symbol;Acc:HGNC:3402]	12794	-1.019556642	-0.109312415
						SNCG	synuclein gamma [Source:HGNC Symbol;Acc:HGNC:11141]	12892	-1.149019957	-0.10417287
						SORBS1	sorbin and SH3 domain containing 1 [Source:HGNC Symbol;Acc:HGNC:14565]	12893	-1.149676681	-0.09153172
						FABP4	fatty acid binding protein 4 [Source:HGNC Symbol;Acc:HGNC:3559]	12897	-1.156383276	-0.0790486
						ADIPOQ	adiponectin, C1Q and collagen domain containing [Source:HGNC Symbol;Acc:HGNC:13633]	12982	-1.299676657	-0.07124812
						RETN	resistin [Source:HGNC Symbol;Acc:HGNC:20389]	12986	-1.311153293	-0.057063248
						SPARCL1	SPARC like 1 [Source:HGNC Symbol;Acc:HGNC:11220]	12988	-1.318863392	-0.042639077
						ACLY	ATP citrate lyase [Source:HGNC Symbol;Acc:HGNC:115]	13036	-1.472710013	-0.03007734
						GPAM	glycerol-3-phosphate acyltransferase, mitochondrial [Source:HGNC Symbol;Acc:HGNC:24865]	13047	-1.538003325	-0.01393899
						POR	cytochrome p450 oxidoreductase [Source:HGNC Symbol;Acc:HGNC:9208]	13083	-1.787703276	0.003013366

181	-0.26598808	0	0.1528119	0.846	2564	CHUK	component of inhibitor of nuclear factor kappa B kinase complex [Source:HGNC Symbol;Acc:HGNC:1974]	10474	-0.480789989	-0.26073352
						ACOX1	acyl-CoA oxidase 1 [Source:HGNC Symbol;Acc:HGNC:119]	10529	-0.486626655	-0.2596155
						HSPB8	heat shock protein family B (small) member 8 [Source:HGNC Symbol;Acc:HGNC:30171]	10565	-0.490483344	-0.25697747
						ADCY6	adenylate cyclase 6 [Source:HGNC Symbol;Acc:HGNC:237]	10594	-0.494190007	-0.2537544
						HIBCH	3-hydroxyisobutyryl-CoA hydrolase [Source:HGNC Symbol;Acc:HGNC:4908]	10608	-0.495730013	-0.24934776
						STOM	stomatin [Source:HGNC Symbol;Acc:HGNC:3383]	10733	-0.50869	-0.2534336
						CAVIN2	caveolae associated protein 2 [Source:HGNC Symbol;Acc:HGNC:10690]	10846	-0.520386696	-0.25645813
						IDH1	isocitrate dehydrogenase (NADP(+)) 1 [Source:HGNC Symbol;Acc:HGNC:5382]	10862	-0.522189975	-0.25191787
						ALDH2	aldehyde dehydrogenase 2 family member [Source:HGNC Symbol;Acc:HGNC:404]	10865	-0.522289991	-0.24636531
						MIGA2	mitoguardin 2 [Source:HGNC Symbol;Acc:HGNC:23621]	10926	-0.529330015	-0.24524732
						DDT	D-dopachrome tautomerase [Source:HGNC Symbol;Acc:HGNC:2732]	11049	-0.54423666	-0.24878906
						SLC19A1	solute carrier family 19 member 1 [Source:HGNC Symbol;Acc:HGNC:10937]	11097	-0.549516678	-0.24643925
						YWHAG	tyrosine 3-monooxygenase/tryptophan 5-monooxygenase activation protein gamma [Source:HGNC Symbol;Acc:HGNC:12852]	11200	-0.564576685	-0.24820301
						REEP6	receptor accessory protein 6 [Source:HGNC Symbol;Acc:HGNC:30078]	11202	-0.56480664	-0.242108
						SCARB1	scavenger receptor class B member 1 [Source:HGNC Symbol;Acc:HGNC:1664]	11382	-0.58987999	-0.24958463
						ABCA1	ATP binding cassette subfamily A member 1 [Source:HGNC Symbol;Acc:HGNC:29]	11395	-0.59187001	-0.24404947
						ECHS1	enoyl-CoA hydratase, short chain 1 [Source:HGNC Symbol;Acc:HGNC:3151]	11402	-0.592966676	-0.23803563
						TOB1	transducer of ERBB2, 1 [Source:HGNC Symbol;Acc:HGNC:11979]	11437	-0.598316669	-0.23414128
						ACADL	acyl-CoA dehydrogenase long chain [Source:HGNC Symbol;Acc:HGNC:88]	11492	-0.606243312	-0.23171599
						CHCHD10	coiled-coil-helix-coiled-coil-helix domain containing 10 [Source:HGNC Symbol;Acc:HGNC:15559]	11546	-0.615906656	-0.22910732
						IDH3A	isocitrate dehydrogenase (NAD(+)) 3 catalytic subunit alpha [Source:HGNC Symbol;Acc:HGNC:5384]	11596	-0.624883354	-0.22608939
						ADIPOR2	adiponectin receptor 2 [Source:HGNC Symbol;Acc:HGNC:24041]	11669	-0.638066649	-0.22471642
						PIM3	Pim-3 proto-oncogene, serine/threonine kinase [Source:HGNC Symbol;Acc:HGNC:19310]	11717	-0.646336675	-0.22130847
						CYP4B1	cytochrome P450 family 4 subfamily B member 1 [Source:HGNC Symbol;Acc:HGNC:2644]	11759	-0.65490669	-0.21734014
						ITGA7	integrin subunit alpha 7 [Source:HGNC Symbol;Acc:HGNC:6143]	11810	-0.664619982	-0.21396571
						SLC5A6	solute carrier family 5 member 6 [Source:HGNC Symbol;Acc:HGNC:11041]	11823	-0.666823328	-0.2076114
						MRAP	melanocortin 2 receptor accessory protein [Source:HGNC Symbol;Acc:HGNC:1304]	11908	-0.687573314	-0.2066308
						CYC1	cytochrome c1 [Source:HGNC Symbol;Acc:HGNC:2579]	11910	-0.68871665	-0.19918156
						SLC1A5	solute carrier family 1 member 5 [Source:HGNC Symbol;Acc:HGNC:10943]	11993	-0.706960022	-0.19783351
						CD36	CD36 molecule [Source:HGNC Symbol;Acc:HGNC:1663]	12196	-0.760673344	-0.20523259
						UCK1	uridine-cytidine kinase 1 [Source:HGNC Symbol;Acc:HGNC:14859]	12226	-0.76976335	-0.19907556
						FZD4	frizzled class receptor 4 [Source:HGNC Symbol;Acc:HGNC:4042]	12249	-0.777960002	-0.19228448
						ESYT1	extended synaptotagmin 1 [Source:HGNC Symbol;Acc:HGNC:29534]	12331	-0.804939985	-0.1897878
						C3	complement C3 [Source:HGNC Symbol;Acc:HGNC:1318]	12367	-0.81736666	-0.18357724
						NABP1	nucleic acid binding protein 1 [Source:HGNC Symbol;Acc:HGNC:26232]	12384	-0.82202667	-0.17583783
						COQ9	coenzyme Q9 [Source:HGNC Symbol;Acc:HGNC:25302]	12389	-0.823176682	-0.16715243
						RETSAT	retinol saturase [Source:HGNC Symbol;Acc:HGNC:25991]	12436	-0.843850017	-0.16150807
						SLC27A1	solute carrier family 27 member 1 [Source:HGNC Symbol;Acc:HGNC:10995]	12477	-0.86596334	-0.1551553
						UCP2	uncoupling protein 2 [Source:HGNC Symbol;Acc:HGNC:12518]	12627	-0.949923337	-0.15636347
						SSPN	sarcospan [Source:HGNC Symbol;Acc:HGNC:11322]	12665	-0.975099981	-0.14858459
						BCL2L13	BCL2 like 13 [Source:HGNC Symbol;Acc:HGNC:17164]	12699	-0.997200012	-0.14025305
						SUCLG1	succinate-CoA ligase GDP/ADP-forming subunit alpha [Source:HGNC Symbol;Acc:HGNC:11449]	12708	-1.002529979	-0.12991863
						PFKFB3	6-phosphofructo-2-kinase/fructose-2,6-biphosphatase 3 [Source:HGNC Symbol;Acc:HGNC:8874]	12712	-1.006226659	-0.11915489
						EPHX2	epoxide hydrolase 2 [Source:HGNC Symbol;Acc:HGNC:3402]	12729	-1.019556642	-0.10925667
						SNCG	synuclein gamma [Source:HGNC Symbol;Acc:HGNC:11141]	12825	-1.149019957	-0.10408853
						SORBS1	sorbin and SH3 domain containing 1 [Source:HGNC Symbol;Acc:HGNC:14565]	12826	-1.149676681	-0.09152366
						FABP4	fatty acid binding protein 4 [Source:HGNC Symbol;Acc:HGNC:3559]	12828	-1.156383276	-0.07896329
						CD302	CD302 molecule [Source:HGNC Symbol;Acc:HGNC:30843]	12894	-1.253546715	-0.07031923
						ADIPOQ	adiponectin, C1Q and collagen domain containing [Source:HGNC Symbol;Acc:HGNC:13633]	12909	-1.299676657	-0.057203993
						RETN	resistin [Source:HGNC Symbol;Acc:HGNC:20389]	12913	-1.311153293	-0.0431077
						ACLY	ATP citrate lyase [Source:HGNC Symbol;Acc:HGNC:115]	12963	-1.472710013	-0.03082384
						GPAM	glycerol-3-phosphate acyltransferase, mitochondrial [Source:HGNC Symbol;Acc:HGNC:24865]	12972	-1.538003325	-0.014637215
						POR	cytochrome p450 oxidoreductase [Source:HGNC Symbol;Acc:HGNC:9208]	13003	-1.787703276	0.002567129

COMPLEMENT

174	-0.28517455	0	0.19902052	0.749	2097	CTSS	cathepsin S [Source:HGNC Symbol;Acc:HGNC:2545]	11705	-0.578629971	-0.27947372
						LCK	LCK proto-oncogene, Src family tyrosine kinase [Source:HGNC Symbol;Acc:HGNC:6524]	11744	-0.582599998	-0.2765223
						GNAI3	G protein subunit alpha i3 [Source:HGNC Symbol;Acc:HGNC:4387]	11746	-0.582729995	-0.27085444

162	-0.2861657	0	0.1659564	0.741	2162	SIRT6	sirtuin 6 [Source:HGNC Symbol;Acc:HGNC:14934]	10962	-0.528283358	-0.2799817
						IRF7	interferon regulatory factor 7 [Source:HGNC Symbol;Acc:HGNC:6122]	10986	-0.530796647	-0.2755428
						DYRK2	dual specificity tyrosine phosphorylation regulated kinase 2 [Source:HGNC Symbol;Acc:HGNC:3093]	11102	-0.544650018	-0.27803996

157	-0.26357868	0.00750469	0.1497108	0.861	2120	SIRT6	sirtuin 6 [Source:HGNC Symbol;Acc:HGNC:14934]	10918	-0.528283358	-0.2570461
						IRF7	interferon regulatory factor 7 [Source:HGNC Symbol;Acc:HGNC:6122]	10940	-0.530796647	-0.2521129
						DYRK2	dual specificity tyrosine phosphorylation regulated kinase 2 [Source:HGNC Symbol;Acc:HGNC:3093]	11056	-0.544650018	-0.2543065

							F3	coagulation factor III, tissue factor [Source:HGNC Symbol;Acc:HGNC:3541]	11820	-0.591936648	-0.2703795
							LIPA	lipase A, lysosomal acid type [Source:HGNC Symbol;Acc:HGNC:6617]	11904	-0.604669988	-0.27051294
							LAP3	leucine aminopeptidase 3 [Source:HGNC Symbol;Acc:HGNC:18449]	12008	-0.620263338	-0.27196044
							BRPF3	bromodomain and PHD finger containing 3 [Source:HGNC Symbol;Acc:HGNC:14256]	12031	-0.624906659	-0.2674181
							SERPING1	serpin family G member 1 [Source:HGNC Symbol;Acc:HGNC:1228]	12098	-0.636123359	-0.2659941
							MMP15	matrix metallopeptidase 15 [Source:HGNC Symbol;Acc:HGNC:7161]	12236	-0.660920024	-0.26953608
							SH2B3	SH2B adaptor protein 3 [Source:HGNC Symbol;Acc:HGNC:29605]	12290	-0.669516683	-0.26682913
							NOTCH4	notch receptor 4 [Source:HGNC Symbol;Acc:HGNC:7884]	12293	-0.670513332	-0.26036978
							CFB	complement factor B [Source:HGNC Symbol;Acc:HGNC:1037]	12305	-0.672839999	-0.25454795
							C2	complement C2 [Source:HGNC Symbol;Acc:HGNC:1248]	12436	-0.700576663	-0.25718552
							CCL5	C-C motif chemokine ligand 5 [Source:HGNC Symbol;Acc:HGNC:10632]	12631	-0.746720016	-0.26406503
							PIM1	Pim-1 proto-oncogene, serine/threonine kinase [Source:HGNC Symbol;Acc:HGNC:8986]	12648	-0.750983357	-0.25784022
							CD36	CD36 molecule [Source:HGNC Symbol;Acc:HGNC:1663]	12690	-0.760673344	-0.25335455
							FCER1G	Fc fragment of IgE receptor Ig [Source:HGNC Symbol;Acc:HGNC:3611]	12792	-0.789573312	-0.25298718
							CALM3	calmodulin 3 [Source:HGNC Symbol;Acc:HGNC:1449]	12816	-0.796710014	-0.24682555
							CFH	complement factor H [Source:HGNC Symbol;Acc:HGNC:4883]	12855	-0.807113349	-0.24166217
							TIMP2	TIMP metallopeptidase inhibitor 2 [Source:HGNC Symbol;Acc:HGNC:11821]	12872	-0.814073324	-0.2348158
							C3	complement C3 [Source:HGNC Symbol;Acc:HGNC:1318]	12885	-0.81736666	-0.22764343
							ACTN2	actinin alpha 2 [Source:HGNC Symbol;Acc:HGNC:164]	12887	-0.817813337	-0.21965945
							CTSD	cathepsin D [Source:HGNC Symbol;Acc:HGNC:2529]	12917	-0.826723337	-0.21364243
							C1S	complement C1s [Source:HGNC Symbol;Acc:HGNC:1247]	13058	-0.888040006	-0.21516688
							CPM	carboxypeptidase M [Source:HGNC Symbol;Acc:HGNC:2311]	13071	-0.891390026	-0.20726521
							LRP1	LDL receptor related protein 1 [Source:HGNC Symbol;Acc:HGNC:6692]	13150	-0.931140006	-0.20381525
							GZMA	granzyme A [Source:HGNC Symbol;Acc:HGNC:4708]	13154	-0.932073355	-0.1948523
							FN1	fibronectin 1 [Source:HGNC Symbol;Acc:HGNC:3778]	13185	-0.944563329	-0.18774767
							CSRP1	cysteine and glycine rich protein 1 [Source:HGNC Symbol;Acc:HGNC:2469]	13230	-0.961633325	-0.18150222
							CLU	clusterin [Source:HGNC Symbol;Acc:HGNC:2095]	13249	-0.971543312	-0.17325115
							CTSC	cathepsin C [Source:HGNC Symbol;Acc:HGNC:2528]	13270	-0.987233341	-0.16499229
							HSPA5	heat shock protein family A (Hsp70) member 5 [Source:HGNC Symbol;Acc:HGNC:5238]	13277	-0.990736663	-0.15567152
							CEBPB	CCAAT enhancer binding protein beta [Source:HGNC Symbol;Acc:HGNC:1834]	13297	-0.998733342	-0.14722596
							SPOCK2	SPARC (osteonectin), cwcv and kazal like domains proteoglycan 2 [Source:HGNC Symbol;Acc:HGNC:13564]	13311	-1.009723306	-0.13823181
							CDH13	cadherin 13 [Source:HGNC Symbol;Acc:HGNC:1753]	13399	-1.085886717	-0.13391769
							PLAUR	plasminogen activator, urokinase receptor [Source:HGNC Symbol;Acc:HGNC:9053]	13472	-1.174236655	-0.12763235
							SERPINE1	serpin family E member 1 [Source:HGNC Symbol;Acc:HGNC:8583]	13513	-1.200383306	-0.11874112
							MMP12	matrix metallopeptidase 12 [Source:HGNC Symbol;Acc:HGNC:7158]	13544	-1.241629958	-0.10870968
							MAFF	MAF bZIP transcription factor F [Source:HGNC Symbol;Acc:HGNC:6780]	13618	-1.382410049	-0.10044673
							CTSH	cathepsin H [Source:HGNC Symbol;Acc:HGNC:2535]	13685	-1.587426662	-0.0896502
							GZMB	granzyme B [Source:HGNC Symbol;Acc:HGNC:4709]	13699	-1.649469972	-0.07435307
							ANG	angiogenin [Source:HGNC Symbol;Acc:HGNC:483]	13729	-1.846573353	-0.058288142
							HSPA1A	heat shock protein family A (Hsp70) member 1A [Source:HGNC Symbol;Acc:HGNC:5232]	13731	-1.850196719	-0.040132772
							S100A9	S100 calcium binding protein A9 [Source:HGNC Symbol;Acc:HGNC:10499]	13741	-1.944020033	-0.021640098
							LTF	lactotransferrin [Source:HGNC Symbol;Acc:HGNC:6720]	13779	-2.628453255	0.001541097
**HYPOXIA**											
**INTERFERON GAMMA RESPONSE**	185	-0.28179526	0	0.19359994	0.775	2099	BATF2	basic leucine zipper ATF-like transcription factor 2 [Source:HGNC Symbol;Acc:HGNC:25163]	11703	-0.578010023	-0.27642933
							CCL2	C-C motif chemokine ligand 2 [Source:HGNC Symbol;Acc:HGNC:10618]	11766	-0.586263359	-0.2755402
							SAMD9L	sterile alpha motif domain containing 9 like [Source:HGNC Symbol;Acc:HGNC:1349]	11780	-0.588073313	-0.2710356
							STAT3	signal transducer and activator of transcription 3 [Source:HGNC Symbol;Acc:HGNC:11364]	11792	-0.588779986	-0.26637757
							CIITA	class II major histocompatibility complex transactivator [Source:HGNC Symbol;Acc:HGNC:7067]	11839	-0.594936669	-0.26423284
							CD69	CD69 molecule [Source:HGNC Symbol;Acc:HGNC:1694]	11859	-0.598246694	-0.26007447
							WARS1	tryptophanyl-tRNA synthetase 1 [Source:HGNC Symbol;Acc:HGNC:12729]	11958	-0.613456666	-0.26157686
							HERC6	HECT and RLD domain containing E3 ubiquitin protein ligase family member 6 [Source:HGNC Symbol;Acc:HGNC:26072]	11990	-0.618149996	-0.25811502
							LAP3	leucine aminopeptidase 3 [Source:HGNC Symbol;Acc:HGNC:18449]	12008	-0.620263338	-0.25360534
							LATS2	large tumor suppressor kinase 2 [Source:HGNC Symbol;Acc:HGNC:6515]	12051	-0.627723336	-0.25086248
							SERPING1	serpin family G member 1 [Source:HGNC Symbol;Acc:HGNC:1228]	12098	-0.636123359	-0.24833544
							CCL7	C-C motif chemokine ligand 7 [Source:HGNC Symbol;Acc:HGNC:10634]	12180	-0.649633348	-0.24825345
							RTP4	receptor transporter protein 4 [Source:HGNC Symbol;Acc:HGNC:23992]	12222	-0.657199979	-0.24516352
							CMKLR1	chemerin chemokine-like receptor 1 [Source:HGNC Symbol;Acc:HGNC:2121]	12260	-0.664596677	-0.24171114
							CFB	complement factor B [Source:HGNC Symbol;Acc:HGNC:1037]	12305	-0.672839999	-0.23869634
							PSMB8	proteasome 20S subunit beta 8 [Source:HGNC Symbol;Acc:HGNC:9545]	12324	-0.676999986	-0.23373339
							CSF2RB	colony stimulating factor 2 receptor subunit beta [Source:HGNC Symbol;Acc:HGNC:2436]	12352	-0.682793319	-0.22937766
							CMTR1	cap methyltransferase 1 [Source:HGNC Symbol;Acc:HGNC:21077]	12369	-0.686343312	-0.22418109
							TRIM25	tripartite motif containing 25 [Source:HGNC Symbol;Acc:HGNC:12932]	12422	-0.696326673	-0.22153579
							TNFAIP6	TNF alpha induced protein 6 [Source:HGNC Symbol;Acc:HGNC:11898]	12439	-0.700743318	-0.21620554
							PSMA3	proteasome 20S subunit alpha 3 [Source:HGNC Symbol;Acc:HGNC:9532]	12463	-0.706449986	-0.21133642
							TNFAIP2	TNF alpha induced protein 2 [Source:HGNC Symbol;Acc:HGNC:11895]	12488	-0.711963356	-0.20648955
							CCL5	C-C motif chemokine ligand 5 [Source:HGNC Symbol;Acc:HGNC:10632]	12631	-0.746720016	-0.2099863
							HLA-DQA1	major histocompatibility complex, class II, DQ alpha 1 [Source:HGNC Symbol;Acc:HGNC:4942]	12633	-0.747590005	-0.2031195
							PIM1	Pim-1 proto-oncogene, serine/threonine kinase [Source:HGNC Symbol;Acc:HGNC:8986]	12648	-0.750983357	-0.19717596
							LGALS3BP	galectin 3 binding protein [Source:HGNC Symbol;Acc:HGNC:6564]	12677	-0.758363307	-0.19219212
							HLA-DMA	major histocompatibility complex, class II, DM alpha [Source:HGNC Symbol;Acc:HGNC:4934]	12687	-0.760209978	-0.18579572
							SOCS3	suppressor of cytokine signaling 3 [Source:HGNC Symbol;Acc:HGNC:19391]	12746	-0.776639998	-0.18284549
							NLRC5	NLR family CARD domain containing 5 [Source:HGNC Symbol;Acc:HGNC:29933]	12822	-0.798776686	-0.18093829
							CFH	complement factor H [Source:HGNC Symbol;Acc:HGNC:4883]	12855	-0.807113349	-0.17579564
							B2M	beta-2-microglobulin [Source:HGNC Symbol;Acc:HGNC:914]	12860	-0.809759974	-0.16857202
							C1S	complement C1s [Source:HGNC Symbol;Acc:HGNC:1247]	13058	-0.888040006	-0.1747962
							IL10RA	interleukin 10 receptor subunit alpha [Source:HGNC Symbol;Acc:HGNC:5964]	13133	-0.921263337	-0.17167845
							DHX58	DExH-box helicase 58 [Source:HGNC Symbol;Acc:HGNC:29517]	13144	-0.927336693	-0.16380396
							STAT1	signal transducer and activator of transcription 1 [Source:HGNC Symbol;Acc:HGNC:11362]	13149	-0.930766642	-0.15545699
							GZMA	granzyme A [Source:HGNC Symbol;Acc:HGNC:4708]	13154	-0.932073355	-0.14709787
							BANK1	B cell scaffold protein with ankyrin repeats 1 [Source:HGNC Symbol;Acc:HGNC:18233]	13155	-0.932773352	-0.1384385
							TAP1	transporter 1, ATP binding cassette subfamily B member [Source:HGNC Symbol;Acc:HGNC:43]	13178	-0.94168669	-0.13131212
							GPR18	G protein-coupled receptor 18 [Source:HGNC Symbol;Acc:HGNC:4472]	13200	-0.949670017	-0.124038175
							EPSTI1	epithelial stromal interaction 1 [Source:HGNC Symbol;Acc:HGNC:16465]	13227	-0.961393356	-0.11702261
							ITGB7	integrin subunit beta 7 [Source:HGNC Symbol;Acc:HGNC:6162]	13242	-0.967236638	-0.10907149
							SSPN	sarcospan [Source:HGNC Symbol;Acc:HGNC:11322]	13251	-0.975099981	-0.10060672
							CD74	CD74 molecule [Source:HGNC Symbol;Acc:HGNC:1697]	13339	-1.037240028	-0.09736706
							IL2RB	interleukin 2 receptor subunit beta [Source:HGNC Symbol;Acc:HGNC:6009]	13434	-1.126933336	-0.093808845
							PTPN6	protein tyrosine phosphatase non-receptor type 6 [Source:HGNC Symbol;Acc:HGNC:9658]	13467	-1.167333364	-0.085322104
							ZBP1	Z-DNA binding protein 1 [Source:HGNC Symbol;Acc:HGNC:16176]	13484	-1.183883309	-0.075506635
							SLAMF7	SLAM family member 7 [Source:HGNC Symbol;Acc:HGNC:21394]	13533	-1.228906631	-0.06762337
							ISOC1	isochorismatase domain containing 1 [Source:HGNC Symbol;Acc:HGNC:24254]	13542	-1.238659978	-0.05671184
							HLA-B	major histocompatibility complex, class I, B [Source:HGNC Symbol;Acc:HGNC:4932]	13543	-1.239956617	-0.04520073
							HLA-DRB1	major histocompatibility complex, class II, DR beta 1 [Source:HGNC Symbol;Acc:HGNC:4948]	13573	-1.278403282	-0.035462547
							PDE4B	phosphodiesterase 4B [Source:HGNC Symbol;Acc:HGNC:8781]	13580	-1.308416724	-0.023756545
							CXCL9	C-X-C motif chemokine ligand 9 [Source:HGNC Symbol;Acc:HGNC:7098]	13591	-1.330023289	-0.012143731
							TRIM14	tripartite motif containing 14 [Source:HGNC Symbol;Acc:HGNC:16283]	13601	-1.343443274	-0.00033289
							LY6E	lymphocyte antigen 6 family member E [Source:HGNC Symbol;Acc:HGNC:6727]	13691	-1.602270007	0.008005323
**MYOGENESIS**	173	-0.33637843	0	0.16738452	0.363	2008	RB1	RB transcriptional corepressor 1 [Source:HGNC Symbol;Acc:HGNC:9884]	11794	-0.589039981	-0.3319485
							GABARAPL2	GABA type A receptor associated protein like 2 [Source:HGNC Symbol;Acc:HGNC:13291]	11832	-0.593856692	-0.3301973
							COL6A2	collagen type VI alpha 2 chain [Source:HGNC Symbol;Acc:HGNC:2212]	11873	-0.599826694	-0.32862136
							MYO1C	myosin IC [Source:HGNC Symbol;Acc:HGNC:7597]	11952	-0.612446666	-0.3297389
							TPM2	tropomyosin 2 [Source:HGNC Symbol;Acc:HGNC:12011]	11957	-0.613373339	-0.32541943
							APP	amyloid beta precursor protein [Source:HGNC Symbol;Acc:HGNC:620]	12052	-0.627916694	-0.32759467
							GSN	gelsolin [Source:HGNC Symbol;Acc:HGNC:4620]	12116	-0.638993323	-0.32741186
							ADAM12	ADAM metallopeptidase domain 12 [Source:HGNC Symbol;Acc:HGNC:190]	12145	-0.643679976	-0.32462558
							MYOM2	myomesin 2 [Source:HGNC Symbol;Acc:HGNC:7614]	12190	-0.651229978	-0.32295656
							SORBS3	sorbin and SH3 domain containing 3 [Source:HGNC Symbol;Acc:HGNC:30907]	12216	-0.656196654	-0.31985602
							ITGA7	integrin subunit alpha 7 [Source:HGNC Symbol;Acc:HGNC:6143]	12261	-0.664619982	-0.3180863
							GNAO1	G protein subunit alpha o1 [Source:HGNC Symbol;Acc:HGNC:4389]	12392	-0.691479981	-0.3224251
							PFKM	phosphofructokinase, muscle [Source:HGNC Symbol;Acc:HGNC:8877]	12405	-0.693333328	-0.31809133
							ITGB4	integrin subunit beta 4 [Source:HGNC Symbol;Acc:HGNC:6158]	12457	-0.704233348	-0.31653735
							COL1A1	collagen type I alpha 1 chain [Source:HGNC Symbol;Acc:HGNC:2197]	12479	-0.709829986	-0.31273994
							ENO3	enolase 3 [Source:HGNC Symbol;Acc:HGNC:3354]	12583	-0.732990026	-0.31478536
							CD36	CD36 molecule [Source:HGNC Symbol;Acc:HGNC:1663]	12690	-0.760673344	-0.3168427
							TPD52L1	TPD52 like 1 [Source:HGNC Symbol;Acc:HGNC:12006]	12701	-0.76572001	-0.3118178
							NOTCH1	notch receptor 1 [Source:HGNC Symbol;Acc:HGNC:7881]	12741	-0.773273349	-0.30886403
							KCNH2	potassium voltage-gated channel subfamily H member 2 [Source:HGNC Symbol;Acc:HGNC:6251]	12748	-0.777793348	-0.30345482
							VIPR1	vasoactive intestinal peptide receptor 1 [Source:HGNC Symbol;Acc:HGNC:12694]	12757	-0.779659986	-0.2981783
							SGCG	sarcoglycan gamma [Source:HGNC Symbol;Acc:HGNC:10809]	12799	-0.792190015	-0.29522905
							SGCD	sarcoglycan delta [Source:HGNC Symbol;Acc:HGNC:10807]	12844	-0.80466336	-0.2924061
							CSRP3	cysteine and glycine rich protein 3 [Source:HGNC Symbol;Acc:HGNC:2472]	12858	-0.808903337	-0.28727657
							SGCA	sarcoglycan alpha [Source:HGNC Symbol;Acc:HGNC:10805]	12861	-0.810060024	-0.28133115

							SRC	SRC proto-oncogene, non-receptor tyrosine kinase [Source:HGNC Symbol;Acc:HGNC:11283]	11169	-0.554543316	-0.27664074
							PSEN1	presenilin 1 [Source:HGNC Symbol;Acc:HGNC:9508]	11184	-0.556500018	-0.2712066
							CTSS	cathepsin S [Source:HGNC Symbol;Acc:HGNC:2545]	11332	-0.578629971	-0.27577493
							LCK	LCK proto-oncogene, Src family tyrosine kinase [Source:HGNC Symbol;Acc:HGNC:6524]	11372	-0.582599998	-0.2719641
							GNAI3	G protein subunit alpha i3 [Source:HGNC Symbol;Acc:HGNC:4387]	11374	-0.582729995	-0.2652199
							F3	coagulation factor III, tissue factor [Source:HGNC Symbol;Acc:HGNC:3541]	11445	-0.591936648	-0.26369157
							LIPA	lipase A, lysosomal acid type [Source:HGNC Symbol;Acc:HGNC:6617]	11524	-0.604669988	-0.26263142
							LAP3	leucine aminopeptidase 3 [Source:HGNC Symbol;Acc:HGNC:18449]	11623	-0.620263338	-0.26293182
							BRPF3	bromodomain and PHD finger containing 3 [Source:HGNC Symbol;Acc:HGNC:14256]	11645	-0.624906659	-0.257237
							SERPING1	serpin family G member 1 [Source:HGNC Symbol;Acc:HGNC:1228]	11705	-0.636123359	-0.25434273
							MMP15	matrix metallopeptidase 15 [Source:HGNC Symbol;Acc:HGNC:7161]	11839	-0.660920024	-0.25686762
							SH2B3	SH2B adaptor protein 3 [Source:HGNC Symbol;Acc:HGNC:29605]	11887	-0.669516683	-0.2526566
							NOTCH4	notch receptor 4 [Source:HGNC Symbol;Acc:HGNC:7884]	11890	-0.670513332	-0.24496195
							CFB	complement factor B [Source:HGNC Symbol;Acc:HGNC:1037]	11903	-0.672839999	-0.23801161
							C2	complement C2 [Source:HGNC Symbol;Acc:HGNC:1248]	12019	-0.700576663	-0.23868352
							CCL5	C-C motif chemokine ligand 5 [Source:HGNC Symbol;Acc:HGNC:10632]	12196	-0.746720016	-0.2435217
							CD36	CD36 molecule [Source:HGNC Symbol;Acc:HGNC:1663]	12250	-0.760673344	-0.23870653
							CALM3	calmodulin 3 [Source:HGNC Symbol;Acc:HGNC:1449]	12360	-0.796710014	-0.23779018
							CFH	complement factor H [Source:HGNC Symbol;Acc:HGNC:4883]	12398	-0.807113349	-0.23119693
							TIMP2	TIMP metallopeptidase inhibitor 2 [Source:HGNC Symbol;Acc:HGNC:11821]	12414	-0.814073324	-0.2228248
							C3	complement C3 [Source:HGNC Symbol;Acc:HGNC:1318]	12424	-0.81736666	-0.21395119
							ACTN2	actinin alpha 2 [Source:HGNC Symbol;Acc:HGNC:164]	12426	-0.817813337	-0.2044551
							CTSD	cathepsin D [Source:HGNC Symbol;Acc:HGNC:2529]	12451	-0.826723337	-0.19662929
							C1S	complement C1s [Source:HGNC Symbol;Acc:HGNC:1247]	12574	-0.888040006	-0.19564685
							CPM	carboxypeptidase M [Source:HGNC Symbol;Acc:HGNC:2311]	12587	-0.891390026	-0.18613818
							LRP1	LDL receptor related protein 1 [Source:HGNC Symbol;Acc:HGNC:6692]	12648	-0.931140006	-0.17986764
							GZMA	granzyme A [Source:HGNC Symbol;Acc:HGNC:4708]	12651	-0.932073355	-0.1691112
							FN1	fibronectin 1 [Source:HGNC Symbol;Acc:HGNC:3778]	12674	-0.944563329	-0.15975165
							CSRP1	cysteine and glycine rich protein 1 [Source:HGNC Symbol;Acc:HGNC:2469]	12709	-0.961633325	-0.15111814
							PLAT	plasminogen activator, tissue type [Source:HGNC Symbol;Acc:HGNC:9051]	12717	-0.966520011	-0.14034425
							CLU	clusterin [Source:HGNC Symbol;Acc:HGNC:2095]	12725	-0.971543312	-0.12951155
							CTSC	cathepsin C [Source:HGNC Symbol;Acc:HGNC:2528]	12744	-0.987233341	-0.1193439
							HSPA5	heat shock protein family A (Hsp70) member 5 [Source:HGNC Symbol;Acc:HGNC:5238]	12750	-0.990736663	-0.10813222
							CEBPB	CCAAT enhancer binding protein beta [Source:HGNC Symbol;Acc:HGNC:1834]	12768	-0.998733342	-0.09775279
							SPOCK2	SPARC (osteonectin), cwcv and kazal like domains proteoglycan 2 [Source:HGNC Symbol;Acc:HGNC:13564]	12779	-1.009723306	-0.08670463
							CD59	CD59 molecule (CD59 blood group) [Source:HGNC Symbol;Acc:HGNC:1689]	12802	-1.031710029	-0.07632495
							CDH13	cadherin 13 [Source:HGNC Symbol;Acc:HGNC:1753]	12852	-1.085886717	-0.06739426
							MAFF	MAF bZIP transcription factor F [Source:HGNC Symbol;Acc:HGNC:6780]	13011	-1.382410049	-0.06340233
							GZMB	granzyme B [Source:HGNC Symbol;Acc:HGNC:4709]	13066	-1.649469972	-0.048260175
							HSPA1A	heat shock protein family A (Hsp70) member 1A [Source:HGNC Symbol;Acc:HGNC:5232]	13087	-1.850196719	-0.028145073
							LTF	lactotransferrin [Source:HGNC Symbol;Acc:HGNC:6720]	13112	-2.628453255	0.000771593
**HYPOXIA**											
**INTERFERON GAMMA RESPONSE**											
**MYOGENESIS**	160	-0.39431387	0	0.0950666	0.126	2105	MEF2A	myocyte enhancer factor 2A [Source:HGNC Symbol;Acc:HGNC:6993]	11019	-0.535266638	-0.38975513
							COL3A1	collagen type III alpha 1 chain [Source:HGNC Symbol;Acc:HGNC:2201]	11036	-0.537150025	-0.38641462
							IGFBP3	insulin like growth factor binding protein 3 [Source:HGNC Symbol;Acc:HGNC:5472]	11045	-0.53840667	-0.38244626
							EIF4A2	eukaryotic translation initiation factor 4A2 [Source:HGNC Symbol;Acc:HGNC:3284]	11078	-0.542406678	-0.38029525
							LPIN1	lipin 1 [Source:HGNC Symbol;Acc:HGNC:13345]	11117	-0.546193361	-0.37857488
							CASQ2	calsequestrin 2 [Source:HGNC Symbol;Acc:HGNC:1513]	11136	-0.548483312	-0.37529212
							RYR1	ryanodine receptor 1 [Source:HGNC Symbol;Acc:HGNC:10483]	11216	-0.560023308	-0.37661678
							MEF2D	myocyte enhancer factor 2D [Source:HGNC Symbol;Acc:HGNC:6997]	11272	-0.568923354	-0.37601423
							RB1	RB transcriptional corepressor 1 [Source:HGNC Symbol;Acc:HGNC:9884]	11420	-0.589039981	-0.38233748
							GABARAPL2	GABA type A receptor associated protein like 2 [Source:HGNC Symbol;Acc:HGNC:13291]	11457	-0.593856692	-0.38005686
							COL6A2	collagen type VI alpha 2 chain [Source:HGNC Symbol;Acc:HGNC:2212]	11498	-0.599826694	-0.37803397
							MYO1C	myosin IC [Source:HGNC Symbol;Acc:HGNC:7597]	11571	-0.612446666	-0.37837216
							TPM2	tropomyosin 2 [Source:HGNC Symbol;Acc:HGNC:12011]	11576	-0.613373339	-0.37345675
							APP	amyloid beta precursor protein [Source:HGNC Symbol;Acc:HGNC:620]	11664	-0.627916694	-0.37482032
							GSN	gelsolin [Source:HGNC Symbol;Acc:HGNC:4620]	11721	-0.638993323	-0.37369815
							ADAM12	ADAM metallopeptidase domain 12 [Source:HGNC Symbol;Acc:HGNC:190]	11748	-0.643679976	-0.37022176
							MYOM2	myomesin 2 [Source:HGNC Symbol;Acc:HGNC:7614]	11793	-0.651229978	-0.36806965
							SORBS3	sorbin and SH3 domain containing 3 [Source:HGNC Symbol;Acc:HGNC:30907]	11820	-0.656196654	-0.36448666
							ITGA7	integrin subunit alpha 7 [Source:HGNC Symbol;Acc:HGNC:6143]	11862	-0.664619982	-0.3619891
							GNAO1	G protein subunit alpha o1 [Source:HGNC Symbol;Acc:HGNC:4389]	11976	-0.691479981	-0.36481702
							PFKM	phosphofructokinase, muscle [Source:HGNC Symbol;Acc:HGNC:8877]	11988	-0.693333328	-0.3597606
							ITGB4	integrin subunit beta 4 [Source:HGNC Symbol;Acc:HGNC:6158]	12038	-0.704233348	-0.3575428
							COL1A1	collagen type I alpha 1 chain [Source:HGNC Symbol;Acc:HGNC:2197]	12056	-0.709829986	-0.35280874
							ENO3	enolase 3 [Source:HGNC Symbol;Acc:HGNC:3354]	12150	-0.732990026	-0.3537403
							CD36	CD36 molecule [Source:HGNC Symbol;Acc:HGNC:1663]	12250	-0.760673344	-0.35489893

							SRC	SRC proto-oncogene, non-receptor tyrosine kinase [Source:HGNC Symbol;Acc:HGNC:11283]	11123	-0.554543316	-0.25257343
							PSEN1	presenilin 1 [Source:HGNC Symbol;Acc:HGNC:9508]	11138	-0.556500018	-0.2467789
							CTSS	cathepsin S [Source:HGNC Symbol;Acc:HGNC:2545]	11285	-0.578629971	-0.25095916
							LCK	LCK proto-oncogene, Src family tyrosine kinase [Source:HGNC Symbol;Acc:HGNC:6524]	11325	-0.582599998	-0.2467829
							GNAI3	G protein subunit alpha i3 [Source:HGNC Symbol;Acc:HGNC:4387]	11327	-0.582729995	-0.23965469
							F3	coagulation factor III, tissue factor [Source:HGNC Symbol;Acc:HGNC:3541]	11396	-0.591936648	-0.23761451
							LIPA	lipase A, lysosomal acid type [Source:HGNC Symbol;Acc:HGNC:6617]	11478	-0.604669988	-0.23642619
							LAP3	leucine aminopeptidase 3 [Source:HGNC Symbol;Acc:HGNC:18449]	11576	-0.620263338	-0.23628728
							BRPF3	bromodomain and PHD finger containing 3 [Source:HGNC Symbol;Acc:HGNC:14256]	11598	-0.624906659	-0.23019034
							SERPING1	serpin family G member 1 [Source:HGNC Symbol;Acc:HGNC:1228]	11659	-0.636123359	-0.22698265
							MMP15	matrix metallopeptidase 15 [Source:HGNC Symbol;Acc:HGNC:7161]	11787	-0.660920024	-0.22867018
							SH2B3	SH2B adaptor protein 3 [Source:HGNC Symbol;Acc:HGNC:29605]	11835	-0.669516683	-0.22404024
							NOTCH4	notch receptor 4 [Source:HGNC Symbol;Acc:HGNC:7884]	11837	-0.670513332	-0.21582654
							CFB	complement factor B [Source:HGNC Symbol;Acc:HGNC:1037]	11849	-0.672839999	-0.20836048
							C2	complement C2 [Source:HGNC Symbol;Acc:HGNC:1248]	11963	-0.700576663	-0.20847069
							CCL5	C-C motif chemokine ligand 5 [Source:HGNC Symbol;Acc:HGNC:10632]	12143	-0.746720016	-0.21313451
							CD36	CD36 molecule [Source:HGNC Symbol;Acc:HGNC:1663]	12196	-0.760673344	-0.20776556
							CALM3	calmodulin 3 [Source:HGNC Symbol;Acc:HGNC:1449]	12304	-0.796710014	-0.20622118
							TIMP2	TIMP metallopeptidase inhibitor 2 [Source:HGNC Symbol;Acc:HGNC:11821]	12357	-0.814073324	-0.20019189
							C3	complement C3 [Source:HGNC Symbol;Acc:HGNC:1318]	12367	-0.81736666	-0.19078337
							ACTN2	actinin alpha 2 [Source:HGNC Symbol;Acc:HGNC:164]	12369	-0.817813337	-0.18074821
							CTSD	cathepsin D [Source:HGNC Symbol;Acc:HGNC:2529]	12395	-0.826723337	-0.17246623
							CPM	carboxypeptidase M [Source:HGNC Symbol;Acc:HGNC:2311]	12529	-0.891390026	-0.17176971
							LRP1	LDL receptor related protein 1 [Source:HGNC Symbol;Acc:HGNC:6692]	12590	-0.931140006	-0.16491394
							GZMA	granzyme A [Source:HGNC Symbol;Acc:HGNC:4708]	12593	-0.932073355	-0.15354352
							FN1	fibronectin 1 [Source:HGNC Symbol;Acc:HGNC:3778]	12615	-0.944563329	-0.1434938
							CSRP1	cysteine and glycine rich protein 1 [Source:HGNC Symbol;Acc:HGNC:2469]	12649	-0.961633325	-0.13416469
							CTSC	cathepsin C [Source:HGNC Symbol;Acc:HGNC:2528]	12682	-0.987233341	-0.12444138
							HSPA5	heat shock protein family A (Hsp70) member 5 [Source:HGNC Symbol;Acc:HGNC:5238]	12689	-0.990736663	-0.11265611
							CEBPB	CCAAT enhancer binding protein beta [Source:HGNC Symbol;Acc:HGNC:1834]	12705	-0.998733342	-0.10147072
							SPOCK2	SPARC (osteonectin), cwcv and kazal like domains proteoglycan 2 [Source:HGNC Symbol;Acc:HGNC:13564]	12715	-1.009723306	-0.089683585
							CD59	CD59 molecule (CD59 blood group) [Source:HGNC Symbol;Acc:HGNC:1689]	12737	-1.031710029	-0.07855625
							CDH13	cadherin 13 [Source:HGNC Symbol;Acc:HGNC:1753]	12787	-1.085886717	-0.0689329
							CASP3	caspase 3 [Source:HGNC Symbol;Acc:HGNC:1504]	12893	-1.251760006	-0.061606243
							MAFF	MAF bZIP transcription factor F [Source:HGNC Symbol;Acc:HGNC:6780]	12936	-1.382410049	-0.04777271
							HSPA1A	heat shock protein family A (Hsp70) member 1A [Source:HGNC Symbol;Acc:HGNC:5232]	13007	-1.850196719	-0.030328598
							LTF	lactotransferrin [Source:HGNC Symbol;Acc:HGNC:6720]	13029	-2.628453255	0.000543511
**HYPOXIA**	154	-0.24565412	0.02156863	0.2151158	0.953	2993	AMPD3	adenosine monophosphate deaminase 3 [Source:HGNC Symbol;Acc:HGNC:470]	10045	-0.436966658	-0.24063647
							CP	ceruloplasmin [Source:HGNC Symbol;Acc:HGNC:2295]	10077	-0.440180004	-0.23798817
							FOSL2	FOS like 2, AP-1 transcription factor subunit [Source:HGNC Symbol;Acc:HGNC:3798]	10104	-0.442253321	-0.23492798
							VLDLR	very low density lipoprotein receptor [Source:HGNC Symbol;Acc:HGNC:12698]	10120	-0.44388333	-0.23099524
							XPNPEP1	X-prolyl aminopeptidase 1 [Source:HGNC Symbol;Acc:HGNC:12822]	10144	-0.445803344	-0.2276614
							PKLR	pyruvate kinase L/R [Source:HGNC Symbol;Acc:HGNC:9020]	10163	-0.447023332	-0.22392546
							GRHPR	glyoxylate and hydroxypyruvate reductase [Source:HGNC Symbol;Acc:HGNC:4570]	10198	-0.450899988	-0.22138695
							TPI1	triosephosphate isomerase 1 [Source:HGNC Symbol;Acc:HGNC:12009]	10253	-0.457273334	-0.22032769
							P4HA2	prolyl 4-hydroxylase subunit alpha 2 [Source:HGNC Symbol;Acc:HGNC:8547]	10322	-0.464416653	-0.22027309
							DDIT4	DNA damage inducible transcript 4 [Source:HGNC Symbol;Acc:HGNC:24944]	10364	-0.469220012	-0.21806757
							ERO1A	endoplasmic reticulum oxidoreductase 1 alpha [Source:HGNC Symbol;Acc:HGNC:13280]	10444	-0.476643324	-0.21872643
							CSRP2	cysteine and glycine rich protein 2 [Source:HGNC Symbol;Acc:HGNC:2470]	10508	-0.484979987	-0.2180476
							CHST3	carbohydrate sulfotransferase 3 [Source:HGNC Symbol;Acc:HGNC:1971]	10563	-0.49022001	-0.21661001
							PGM2	phosphoglucomutase 2 [Source:HGNC Symbol;Acc:HGNC:8906]	10752	-0.510393322	-0.22534208
							BTG1	BTG anti-proliferation factor 1 [Source:HGNC Symbol;Acc:HGNC:1130]	10829	-0.518503308	-0.2252874
							P4HA1	prolyl 4-hydroxylase subunit alpha 1 [Source:HGNC Symbol;Acc:HGNC:8546]	10849	-0.520903349	-0.22078072
							KLF6	Kruppel like factor 6 [Source:HGNC Symbol;Acc:HGNC:2235]	10850	-0.520950019	-0.21479869
							GYS1	glycogen synthase 1 [Source:HGNC Symbol;Acc:HGNC:4706]	10974	-0.535269976	-0.21819969
							AKAP12	A-kinase anchoring protein 12 [Source:HGNC Symbol;Acc:HGNC:370]	10981	-0.535669982	-0.21251436
							IGFBP3	insulin like growth factor binding protein 3 [Source:HGNC Symbol;Acc:HGNC:5472]	10999	-0.53840667	-0.20765144
							PDK1	pyruvate dehydrogenase kinase 1 [Source:HGNC Symbol;Acc:HGNC:8809]	11130	-0.555096686	-0.21136813
							CAV1	caveolin 1 [Source:HGNC Symbol;Acc:HGNC:1527]	11162	-0.559509993	-0.20734958
							JUN	Jun proto-oncogene, AP-1 transcription factor subunit [Source:HGNC Symbol;Acc:HGNC:6204]	11173	-0.560363352	-0.2016912
							ENO2	enolase 2 [Source:HGNC Symbol;Acc:HGNC:3353]	11187	-0.562526643	-0.19624083
							HK2	hexokinase 2 [Source:HGNC Symbol;Acc:HGNC:4923]	11250	-0.572576642	-0.19447853
							PPARGC1A	PPARG coactivator 1 alpha [Source:HGNC Symbol;Acc:HGNC:9237]	11295	-0.58020997	-0.19123137
							SCARB1	scavenger receptor class B member 1 [Source:HGNC Symbol;Acc:HGNC:1664]	11382	-0.58987999	-0.19113329
							F3	coagulation factor III, tissue factor [Source:HGNC Symbol;Acc:HGNC:3541]	11396	-0.591936648	-0.1853452
							EXT1	exostosin glycosyltransferase 1 [Source:HGNC Symbol;Acc:HGNC:3512]	11401	-0.592823327	-0.17884834
							GPC1	glypican 1 [Source:HGNC Symbol;Acc:HGNC:4449]	11458	-0.600973308	-0.17629424
							SDC3	syndecan 3 [Source:HGNC Symbol;Acc:HGNC:10660]	11463	-0.601906657	-0.16969307
							ADORA2B	adenosine A2b receptor [Source:HGNC Symbol;Acc:HGNC:264]	11480	-0.604849994	-0.16398957
							IRS2	insulin receptor substrate 2 [Source:HGNC Symbol;Acc:HGNC:6126]	11648	-0.634119987	-0.16967084
							PAM	peptidylglycine alpha-amidating monooxygenase [Source:HGNC Symbol;Acc:HGNC:8596]	11672	-0.638463318	-0.16412471
							EFNA3	ephrin A3 [Source:HGNC Symbol;Acc:HGNC:3223]	11755	-0.653439999	-0.1629863
							HDLBP	high density lipoprotein binding protein [Source:HGNC Symbol;Acc:HGNC:4857]	11890	-0.682969987	-0.1655451
							TGFB3	transforming growth factor beta 3 [Source:HGNC Symbol;Acc:HGNC:11769]	11896	-0.684736669	-0.15807043
							RRAGD	Ras related GTP binding D [Source:HGNC Symbol;Acc:HGNC:19903]	11923	-0.691900015	-0.15214357
							TGM2	transglutaminase 2 [Source:HGNC Symbol;Acc:HGNC:11778]	11981	-0.703793347	-0.14848639
							PCK1	phosphoenolpyruvate carboxykinase 1 [Source:HGNC Symbol;Acc:HGNC:8724]	12058	-0.724096656	-0.1460709
							ENO3	enolase 3 [Source:HGNC Symbol;Acc:HGNC:3354]	12095	-0.732990026	-0.1404484
							CCN1	cellular communication network factor 1 [Source:HGNC Symbol;Acc:HGNC:2654]	12176	-0.756123304	-0.13797563
							KLHL24	kelch like family member 24 [Source:HGNC Symbol;Acc:HGNC:25947]	12277	-0.787199974	-0.13669844
							ACKR3	atypical chemokine receptor 3 [Source:HGNC Symbol;Acc:HGNC:23692]	12338	-0.806620002	-0.13209338
							MYH9	myosin heavy chain 9 [Source:HGNC Symbol;Acc:HGNC:7579]	12407	-0.831960022	-0.12781833
							DCN	decorin [Source:HGNC Symbol;Acc:HGNC:2705]	12431	-0.840266645	-0.119954914
							NDST1	N-deacetylase and N-sulfotransferase 1 [Source:HGNC Symbol;Acc:HGNC:7680]	12524	-0.890240014	-0.11687355
							FOXO3	forkhead box O3 [Source:HGNC Symbol;Acc:HGNC:3821]	12535	-0.894293308	-0.107380666
							ZFP36	ZFP36 ring finger protein [Source:HGNC Symbol;Acc:HGNC:12862]	12551	-0.906736672	-0.098133005
							TPD52	tumor protein D52 [Source:HGNC Symbol;Acc:HGNC:12005]	12554	-0.907633305	-0.08786596
							HSPA5	heat shock protein family A (Hsp70) member 5 [Source:HGNC Symbol;Acc:HGNC:5238]	12689	-0.990736663	-0.08689071
							PFKFB3	6-phosphofructo-2-kinase/fructose-2,6-biphosphatase 3 [Source:HGNC Symbol;Acc:HGNC:8874]	12712	-1.006226659	-0.077043965
							ATF3	activating transcription factor 3 [Source:HGNC Symbol;Acc:HGNC:785]	12731	-1.022729993	-0.06669723
							HAS1	hyaluronan synthase 1 [Source:HGNC Symbol;Acc:HGNC:4818]	12751	-1.044396639	-0.056179315
							FOS	Fos proto-oncogene, AP-1 transcription factor subunit [Source:HGNC Symbol;Acc:HGNC:3796]	12834	-1.162256718	-0.049198188
							CCN2	cellular communication network factor 2 [Source:HGNC Symbol;Acc:HGNC:2500]	12871	-1.207753301	-0.038124032
							MAFF	MAF bZIP transcription factor F [Source:HGNC Symbol;Acc:HGNC:6780]	12936	-1.382410049	-0.027217712
							ERRFI1	ERBB receptor feedback inhibitor 1 [Source:HGNC Symbol;Acc:HGNC:18185]	12958	-1.451570034	-0.012179501
							PLAC8	placenta associated 8 [Source:HGNC Symbol;Acc:HGNC:19254]	12984	-1.581143379	0.004036104
**INTERFERON GAMMA RESPONSE**	164	-0.24381383	0.0130597	0.212778	0.96	2838	IRF4	interferon regulatory factor 4 [Source:HGNC Symbol;Acc:HGNC:6119]	10200	-0.451163322	-0.23858617
							PTGS2	prostaglandin-endoperoxide synthase 2 [Source:HGNC Symbol;Acc:HGNC:9605]	10234	-0.455516666	-0.23587157
							IDO1	indoleamine 2,3-dioxygenase 1 [Source:HGNC Symbol;Acc:HGNC:6059]	10246	-0.456093341	-0.23144129
							NOD1	nucleotide binding oligomerization domain containing 1 [Source:HGNC Symbol;Acc:HGNC:16390]	10327	-0.464706659	-0.23227127
							GBP6	guanylate binding protein family member 6 [Source:HGNC Symbol;Acc:HGNC:25395]	10382	-0.470333338	-0.23101632
							USP18	ubiquitin specific peptidase 18 [Source:HGNC Symbol;Acc:HGNC:12616]	10387	-0.470816672	-0.22587167
							HIF1A	hypoxia inducible factor 1 subunit alpha [Source:HGNC Symbol;Acc:HGNC:4910]	10394	-0.471509993	-0.22087434
							ST3GAL5	ST3 beta-galactoside alpha-2,3-sialyltransferase 5 [Source:HGNC Symbol;Acc:HGNC:10872]	10440	-0.476266652	-0.2188515
							HLA-A	major histocompatibility complex, class I, A [Source:HGNC Symbol;Acc:HGNC:4931]	10481	-0.481803328	-0.2163761
							IFIH1	interferon induced with helicase C domain 1 [Source:HGNC Symbol;Acc:HGNC:18873]	10526	-0.486436665	-0.21415773
							PNP	purine nucleoside phosphorylase [Source:HGNC Symbol;Acc:HGNC:7892]	10553	-0.489326656	-0.21050762
							APOL6	apolipoprotein L6 [Source:HGNC Symbol;Acc:HGNC:14870]	10555	-0.489490002	-0.20491354
							FGL2	fibrinogen like 2 [Source:HGNC Symbol;Acc:HGNC:3696]	10796	-0.515339971	-0.21758595
							BTG1	BTG anti-proliferation factor 1 [Source:HGNC Symbol;Acc:HGNC:1130]	10829	-0.518503308	-0.21406384
							RAPGEF6	Rap guanine nucleotide exchange factor 6 [Source:HGNC Symbol;Acc:HGNC:20655]	10876	-0.524026692	-0.21156529
							IRF7	interferon regulatory factor 7 [Source:HGNC Symbol;Acc:HGNC:6122]	10940	-0.530796647	-0.21030888
							PARP12	poly(ADP-ribose) polymerase family member 12 [Source:HGNC Symbol;Acc:HGNC:21919]	11019	-0.540849984	-0.2101012
							ICAM1	intercellular adhesion molecule 1 [Source:HGNC Symbol;Acc:HGNC:5344]	11020	-0.541023314	-0.20383234
							BATF2	basic leucine zipper ATF-like transcription factor 2 [Source:HGNC Symbol;Acc:HGNC:25163]	11283	-0.578010023	-0.21748759
							CCL2	C-C motif chemokine ligand 2 [Source:HGNC Symbol;Acc:HGNC:10618]	11345	-0.586263359	-0.21543312
							SAMD9L	sterile alpha motif domain containing 9 like [Source:HGNC Symbol;Acc:HGNC:1349]	11358	-0.588073313	-0.20955126
							STAT3	signal transducer and activator of transcription 3 [Source:HGNC Symbol;Acc:HGNC:11364]	11370	-0.588779986	-0.20358354
							CIITA	class II major histocompatibility complex transactivator [Source:HGNC Symbol;Acc:HGNC:7067]	11415	-0.594936669	-0.20010798
							CD69	CD69 molecule [Source:HGNC Symbol;Acc:HGNC:1694]	11436	-0.598246694	-0.19472969
							HERC6	HECT and RLD domain containing E3 ubiquitin protein ligase family member 6 [Source:HGNC Symbol;Acc:HGNC:26072]	11560	-0.618149996	-0.19712204
							LAP3	leucine aminopeptidase 3 [Source:HGNC Symbol;Acc:HGNC:18449]	11576	-0.620263338	-0.19110024
							LATS2	large tumor suppressor kinase 2 [Source:HGNC Symbol;Acc:HGNC:6515]	11617	-0.627723336	-0.18693405
							SERPING1	serpin family G member 1 [Source:HGNC Symbol;Acc:HGNC:1228]	11659	-0.636123359	-0.18274821
							CCL7	C-C motif chemokine ligand 7 [Source:HGNC Symbol;Acc:HGNC:10634]	11735	-0.649633348	-0.18104702
							RTP4	receptor transporter protein 4 [Source:HGNC Symbol;Acc:HGNC:23992]	11775	-0.657199979	-0.1764616
							CMKLR1	chemerin chemokine-like receptor 1 [Source:HGNC Symbol;Acc:HGNC:2121]	11809	-0.664596677	-0.17132439
							CFB	complement factor B [Source:HGNC Symbol;Acc:HGNC:1037]	11849	-0.672839999	-0.16655774
							CSF2RB	colony stimulating factor 2 receptor subunit beta [Source:HGNC Symbol;Acc:HGNC:2436]	11889	-0.682793319	-0.16167578
							CMTR1	cap methyltransferase 1 [Source:HGNC Symbol;Acc:HGNC:21077]	11903	-0.686343312	-0.15473294
							TRIM25	tripartite motif containing 25 [Source:HGNC Symbol;Acc:HGNC:12932]	11950	-0.696326673	-0.15023795
							TNFAIP6	TNF alpha induced protein 6 [Source:HGNC Symbol;Acc:HGNC:11898]	11965	-0.700743318	-0.14320594
							PSMA3	proteasome 20S subunit alpha 3 [Source:HGNC Symbol;Acc:HGNC:9532]	11989	-0.706449986	-0.13680695
							TNFAIP2	TNF alpha induced protein 2 [Source:HGNC Symbol;Acc:HGNC:11895]	12009	-0.711963356	-0.13003334
							CCL5	C-C motif chemokine ligand 5 [Source:HGNC Symbol;Acc:HGNC:10632]	12143	-0.746720016	-0.13171275
							LGALS3BP	galectin 3 binding protein [Source:HGNC Symbol;Acc:HGNC:6564]	12186	-0.758363307	-0.1261882
							SOCS3	suppressor of cytokine signaling 3 [Source:HGNC Symbol;Acc:HGNC:19391]	12246	-0.776639998	-0.12177246
							NLRC5	NLR family CARD domain containing 5 [Source:HGNC Symbol;Acc:HGNC:29933]	12310	-0.798776686	-0.11741096
							B2M	beta-2-microglobulin [Source:HGNC Symbol;Acc:HGNC:914]	12345	-0.809759974	-0.110669404
							IL10RA	interleukin 10 receptor subunit alpha [Source:HGNC Symbol;Acc:HGNC:5964]	12574	-0.921263337	-0.117706165
							DHX58	DExH-box helicase 58 [Source:HGNC Symbol;Acc:HGNC:29517]	12584	-0.927336693	-0.1076602
							STAT1	signal transducer and activator of transcription 1 [Source:HGNC Symbol;Acc:HGNC:11362]	12589	-0.930766642	-0.09718608
							GZMA	granzyme A [Source:HGNC Symbol;Acc:HGNC:4708]	12593	-0.932073355	-0.08661914
							GPR18	G protein-coupled receptor 18 [Source:HGNC Symbol;Acc:HGNC:4472]	12626	-0.949670017	-0.078101076
							SSPN	sarcospan [Source:HGNC Symbol;Acc:HGNC:11322]	12665	-0.975099981	-0.06975445
							CD74	CD74 molecule [Source:HGNC Symbol;Acc:HGNC:1697]	12742	-1.037240028	-0.06363972
							IL2RB	interleukin 2 receptor subunit beta [Source:HGNC Symbol;Acc:HGNC:6009]	12809	-1.126933336	-0.05570889
							PTPN6	protein tyrosine phosphatase non-receptor type 6 [Source:HGNC Symbol;Acc:HGNC:9658]	12835	-1.167333364	-0.04412498
							ISOC1	isochorismatase domain containing 1 [Source:HGNC Symbol;Acc:HGNC:24254]	12884	-1.238659978	-0.03350129
							CASP3	caspase 3 [Source:HGNC Symbol;Acc:HGNC:1504]	12893	-1.251760006	-0.01961853
							PDE4B	phosphodiesterase 4B [Source:HGNC Symbol;Acc:HGNC:8781]	12911	-1.308416724	-0.005778423
							CXCL9	C-X-C motif chemokine ligand 9 [Source:HGNC Symbol;Acc:HGNC:7098]	12919	-1.330023289	0.00908886
**MYOGENESIS**	159	-0.37229708	0	0.2805936	0.18	2065	MEF2A	myocyte enhancer factor 2A [Source:HGNC Symbol;Acc:HGNC:6993]	10973	-0.535266638	-0.36775005
							COL3A1	collagen type III alpha 1 chain [Source:HGNC Symbol;Acc:HGNC:2201]	10990	-0.537150025	-0.36442944
							IGFBP3	insulin like growth factor binding protein 3 [Source:HGNC Symbol;Acc:HGNC:5472]	10999	-0.53840667	-0.36047694
							EIF4A2	eukaryotic translation initiation factor 4A2 [Source:HGNC Symbol;Acc:HGNC:3284]	11032	-0.542406678	-0.35835412
							LPIN1	lipin 1 [Source:HGNC Symbol;Acc:HGNC:13345]	11071	-0.546193361	-0.35666502
							CASQ2	calsequestrin 2 [Source:HGNC Symbol;Acc:HGNC:1513]	11089	-0.548483312	-0.35332578
							RYR1	ryanodine receptor 1 [Source:HGNC Symbol;Acc:HGNC:10483]	11169	-0.560023308	-0.35470295
							RB1	RB transcriptional corepressor 1 [Source:HGNC Symbol;Acc:HGNC:9884]	11372	-0.589039981	-0.3653848
							GABARAPL2	GABA type A receptor associated protein like 2 [Source:HGNC Symbol;Acc:HGNC:13291]	11408	-0.593856692	-0.36305785
							COL6A2	collagen type VI alpha 2 chain [Source:HGNC Symbol;Acc:HGNC:2212]	11450	-0.599826694	-0.3611461
							MYO1C	myosin IC [Source:HGNC Symbol;Acc:HGNC:7597]	11524	-0.612446666	-0.36161202
							TPM2	tropomyosin 2 [Source:HGNC Symbol;Acc:HGNC:12011]	11529	-0.613373339	-0.35671207
							APP	amyloid beta precursor protein [Source:HGNC Symbol;Acc:HGNC:620]	11618	-0.627916694	-0.35821134
							GSN	gelsolin [Source:HGNC Symbol;Acc:HGNC:4620]	11675	-0.638993323	-0.35713166
							ADAM12	ADAM metallopeptidase domain 12 [Source:HGNC Symbol;Acc:HGNC:190]	11701	-0.643679976	-0.35360497
							MYOM2	myomesin 2 [Source:HGNC Symbol;Acc:HGNC:7614]	11743	-0.651229978	-0.35125655
							SORBS3	sorbin and SH3 domain containing 3 [Source:HGNC Symbol;Acc:HGNC:30907]	11769	-0.656196654	-0.34762353
							ITGA7	integrin subunit alpha 7 [Source:HGNC Symbol;Acc:HGNC:6143]	11810	-0.664619982	-0.3450837
							GNAO1	G protein subunit alpha o1 [Source:HGNC Symbol;Acc:HGNC:4389]	11921	-0.691479981	-0.34775138
							PFKM	phosphofructokinase, muscle [Source:HGNC Symbol;Acc:HGNC:8877]	11933	-0.693333328	-0.34271574
							ITGB4	integrin subunit beta 4 [Source:HGNC Symbol;Acc:HGNC:6158]	11983	-0.704233348	-0.3405383
							COL1A1	collagen type I alpha 1 chain [Source:HGNC Symbol;Acc:HGNC:2197]	12002	-0.709829986	-0.3359061
							ENO3	enolase 3 [Source:HGNC Symbol;Acc:HGNC:3354]	12095	-0.732990026	-0.3368234
							CD36	CD36 molecule [Source:HGNC Symbol;Acc:HGNC:1663]	12196	-0.760673344	-0.33812672
							TPD52L1	TPD52 like 1 [Source:HGNC Symbol;Acc:HGNC:12006]	12205	-0.76572001	-0.33224323

PDE4DIP	phosphodiesterase 4D interacting protein [Source:HGNC Symbol;Acc:HGNC:15580]	12868	-0.812636673	-0.2756599	
ACTN2	actinin alpha 2 [Source:HGNC Symbol;Acc:HGNC:164]	12887	-0.817813337	-0.2708302	
MYH9	myosin heavy chain 9 [Source:HGNC Symbol;Acc:HGNC:7579]	12932	-0.831960022	-0.267802	
COX6A2	cytochrome c oxidase subunit 6A2 [Source:HGNC Symbol;Acc:HGNC:2279]	13053	-0.881786644	-0.2699758	
MYH11	myosin heavy chain 11 [Source:HGNC Symbol;Acc:HGNC:7569]	13100	-0.906806648	-0.26653144	
CKMT2	creatine kinase, mitochondrial 2 [Source:HGNC Symbol;Acc:HGNC:1996]	13109	-0.911583304	-0.2602628	
SLN	sarcolipin [Source:HGNC Symbol;Acc:HGNC:11089]	13203	-0.950680017	-0.25993726	
ADCY9	adenylate cyclase 9 [Source:HGNC Symbol;Acc:HGNC:240]	13205	-0.951063335	-0.25285804	
LDB3	LIM domain binding 3 [Source:HGNC Symbol;Acc:HGNC:15710]	13246	-0.968739986	-0.24850762	
CLU	clusterin [Source:HGNC Symbol;Acc:HGNC:2095]	13249	-0.971543312	-0.24134776	
SSPN	sarcospan [Source:HGNC Symbol;Acc:HGNC:11322]	13251	-0.975099981	-0.23408777	
MYBPC3	myosin binding protein C3 [Source:HGNC Symbol;Acc:HGNC:7551]	13252	-0.975499988	-0.22675139	
GAA	glucosidase alpha, acid [Source:HGNC Symbol;Acc:HGNC:4065]	13388	-1.079766631	-0.22853693	
CDH13	cadherin 13 [Source:HGNC Symbol;Acc:HGNC:1753]	13399	-1.085886717	-0.22110416	
CKM	creatine kinase, M-type [Source:HGNC Symbol;Acc:HGNC:1994]	13441	-1.134549975	-0.21558014	
SORBS1	sorbin and SH3 domain containing 1 [Source:HGNC Symbol;Acc:HGNC:14565]	13455	-1.149676681	-0.20788777	
ACSL1	acyl-CoA synthetase long chain family member 1 [Source:HGNC Symbol;Acc:HGNC:3569]	13473	-1.17513001	-0.20029747	
MYOZ1	myozenin 1 [Source:HGNC Symbol;Acc:HGNC:13752]	13482	-1.183253288	-0.19198568	
MYH7	myosin heavy chain 7 [Source:HGNC Symbol;Acc:HGNC:7577]	13516	-1.201509953	-0.18537106	
MYH1	myosin heavy chain 1 [Source:HGNC Symbol;Acc:HGNC:7567]	13539	-1.236310005	-0.17768754	
HRC	histidine rich calcium binding protein [Source:HGNC Symbol;Acc:HGNC:5178]	13570	-1.277106643	-0.17028424	
ACTC1	actin alpha cardiac muscle 1 [Source:HGNC Symbol;Acc:HGNC:143]	13572	-1.277343392	-0.1607512	
PC	pyruvate carboxylase [Source:HGNC Symbol;Acc:HGNC:8636]	13605	-1.348356724	-0.1529588	
MB	myoglobin [Source:HGNC Symbol;Acc:HGNC:6915]	13643	-1.441476703	-0.14483298	
TCAP	titin-cap [Source:HGNC Symbol;Acc:HGNC:11610]	13668	-1.515963316	-0.13519305	
MYLPF	myosin light chain, phosphorylatable, fast skeletal muscle [Source:HGNC Symbol;Acc:HGNC:29824]	13673	-1.544690013	-0.123869516	
BDKRB2	bradykinin receptor B2 [Source:HGNC Symbol;Acc:HGNC:1030]	13679	-1.572113276	-0.11241312	
MYL1	myosin light chain 1 [Source:HGNC Symbol;Acc:HGNC:7582]	13687	-1.592456698	-0.10095049	
ACTN3	actinin alpha 3 [Source:HGNC Symbol;Acc:HGNC:165]	13688	-1.593743324	-0.08896452	
CASQ1	calsequestrin 1 [Source:HGNC Symbol;Acc:HGNC:1512]	13703	-1.677936673	-0.07737268	
TNNI2	troponin I2, fast skeletal type [Source:HGNC Symbol;Acc:HGNC:11946]	13727	-1.839653373	-0.06522502	
CFD	complement factor D [Source:HGNC Symbol;Acc:HGNC:2771]	13755	-2.079580069	-0.05156647	
ATP2A1	ATPase sarcoplasmic/endoplasmic reticulum Ca2+ transporting 1 [Source:HGNC Symbol;Acc:HGNC:811]	13758	-2.153290033	-0.035519123	
TNNT3	troponin T3, fast skeletal type [Source:HGNC Symbol;Acc:HGNC:11950]	13760	-2.19936657	-0.019051868	
ACTA1	actin alpha 1, skeletal muscle [Source:HGNC Symbol;Acc:HGNC:129]	13785	-2.913803339	0.001100699	

TPD52L1	TPD52 like 1 [Source:HGNC Symbol;Acc:HGNC:12006]	12259	-0.76572001	-0.34899458
NOTCH1	notch receptor 1 [Source:HGNC Symbol;Acc:HGNC:7881]	12297	-0.773273349	-0.34526306
KCNH2	potassium voltage-gated channel subfamily H member 2 [Source:HGNC Symbol;Acc:HGNC:6251]	12303	-0.777793348	-0.33902448
VIPR1	vasoactive intestinal peptide receptor 1 [Source:HGNC Symbol;Acc:HGNC:12694]	12311	-0.779659986	-0.33292428
SGCG	sarcoglycan gamma [Source:HGNC Symbol;Acc:HGNC:10809]	12346	-0.792190015	-0.3288002
CSRP3	cysteine and glycine rich protein 3 [Source:HGNC Symbol;Acc:HGNC:2472]	12401	-0.808903337	-0.32607666
SGCA	sarcoglycan alpha [Source:HGNC Symbol;Acc:HGNC:10805]	12404	-0.810060024	-0.31933182
PDE4DIP	phosphodiesterase 4D interacting protein [Source:HGNC Symbol;Acc:HGNC:15580]	12411	-0.812636673	-0.31287363
ACTN2	actinin alpha 2 [Source:HGNC Symbol;Acc:HGNC:164]	12426	-0.817813337	-0.30698848
MYH9	myosin heavy chain 9 [Source:HGNC Symbol;Acc:HGNC:7579]	12464	-0.831960022	-0.30275714
COX6A2	cytochrome c oxidase subunit 6A2 [Source:HGNC Symbol;Acc:HGNC:2279]	12569	-0.881786644	-0.30326998
MYH11	myosin heavy chain 11 [Source:HGNC Symbol;Acc:HGNC:7569]	12611	-0.906806648	-0.29870975
CKMT2	creatine kinase, mitochondrial 2 [Source:HGNC Symbol;Acc:HGNC:1996]	12618	-0.911583304	-0.29140884
KLF5	Kruppel like factor 5 [Source:HGNC Symbol;Acc:HGNC:6349]	12638	-0.923900008	-0.2850059
SLN	sarcolipin [Source:HGNC Symbol;Acc:HGNC:11089]	12689	-0.950680017	-0.28076628
ADCY9	adenylate cyclase 9 [Source:HGNC Symbol;Acc:HGNC:240]	12691	-0.951063335	-0.27274343
LDB3	LIM domain binding 3 [Source:HGNC Symbol;Acc:HGNC:15710]	12723	-0.968739986	-0.2668843
CLU	clusterin [Source:HGNC Symbol;Acc:HGNC:2095]	12725	-0.971543312	-0.25868702
MYBPC3	myosin binding protein C3 [Source:HGNC Symbol;Acc:HGNC:7551]	12729	-0.975499988	-0.2506103
CDH13	cadherin 13 [Source:HGNC Symbol;Acc:HGNC:1753]	12852	-1.085886717	-0.25077343
CKM	creatine kinase, M-type [Source:HGNC Symbol;Acc:HGNC:1994]	12882	-1.134549975	-0.24334785
SORBS1	sorbin and SH3 domain containing 1 [Source:HGNC Symbol;Acc:HGNC:14565]	12893	-1.149676681	-0.23432773
AEBP1	AE binding protein 1 [Source:HGNC Symbol;Acc:HGNC:303]	12894	-1.151586652	-0.22451991
ACSL1	acyl-CoA synthetase long chain family member 1 [Source:HGNC Symbol;Acc:HGNC:3569]	12905	-1.17513001	-0.215283
MYOZ1	myozenin 1 [Source:HGNC Symbol;Acc:HGNC:13752]	12911	-1.183253288	-0.2055912
MYH1	myosin heavy chain 1 [Source:HGNC Symbol;Acc:HGNC:7567]	12951	-1.236310005	-0.19807038
HRC	histidine rich calcium binding protein [Source:HGNC Symbol;Acc:HGNC:5178]	12974	-1.277106643	-0.18889067
ACTC1	actin alpha cardiac muscle 1 [Source:HGNC Symbol;Acc:HGNC:143]	12976	-1.277343392	-0.17808895
MB	myoglobin [Source:HGNC Symbol;Acc:HGNC:6915]	13026	-1.441476703	-0.16959217
TCAP	titin-cap [Source:HGNC Symbol;Acc:HGNC:11610]	13044	-1.515963316	-0.15799247
MYLPF	myosin light chain, phosphorylatable, fast skeletal muscle [Source:HGNC Symbol;Acc:HGNC:29824]	13048	-1.544690013	-0.1450681
BDKRB2	bradykinin receptor B2 [Source:HGNC Symbol;Acc:HGNC:1030]	13055	-1.572113276	-0.13214159
MYL1	myosin light chain 1 [Source:HGNC Symbol;Acc:HGNC:7582]	13060	-1.592456698	-0.11888755
ACTN3	actinin alpha 3 [Source:HGNC Symbol;Acc:HGNC:165]	13061	-1.593743324	-0.10531397
CASQ1	calsequestrin 1 [Source:HGNC Symbol;Acc:HGNC:1512]	13068	-1.677936673	-0.0914862
TNNI2	troponin I2, fast skeletal type [Source:HGNC Symbol;Acc:HGNC:11946]	13085	-1.839653373	-0.07705254
CFD	complement factor D [Source:HGNC Symbol;Acc:HGNC:2771]	13099	-2.079580069	-0.060344055
ATP2A1	ATPase sarcoplasmic/endoplasmic reticulum Ca2+ transporting 1 [Source:HGNC Symbol;Acc:HGNC:811]	13100	-2.153290033	-0.04200494
TNNT3	troponin T3, fast skeletal type [Source:HGNC Symbol;Acc:HGNC:11950]	13101	-2.19936657	-0.023273397
ACTA1	actin alpha 1, skeletal muscle [Source:HGNC Symbol;Acc:HGNC:129]	13115	-2.913803339	0.000539994

NOTCH1	notch receptor 1 [Source:HGNC Symbol;Acc:HGNC:7881]	12240	-0.773273349	-0.3283145
KCNH2	potassium voltage-gated channel subfamily H member 2 [Source:HGNC Symbol;Acc:HGNC:6251]	12248	-0.777793348	-0.3222508
VIPR1	vasoactive intestinal peptide receptor 1 [Source:HGNC Symbol;Acc:HGNC:12694]	12255	-0.779659986	-0.3160936
SGCG	sarcoglycan gamma [Source:HGNC Symbol;Acc:HGNC:10809]	12290	-0.792190015	-0.31200418
CSRP3	cysteine and glycine rich protein 3 [Source:HGNC Symbol;Acc:HGNC:2472]	12343	-0.808903337	-0.3091705
SGCA	sarcoglycan alpha [Source:HGNC Symbol;Acc:HGNC:10805]	12346	-0.810060024	-0.30244446
PDE4DIP	phosphodiesterase 4D interacting protein [Source:HGNC Symbol;Acc:HGNC:15580]	12353	-0.812636673	-0.2960071
ACTN2	actinin alpha 2 [Source:HGNC Symbol;Acc:HGNC:164]	12369	-0.817813337	-0.29022464
MYH9	myosin heavy chain 9 [Source:HGNC Symbol;Acc:HGNC:7579]	12407	-0.831960022	-0.28603032
COX6A2	cytochrome c oxidase subunit 6A2 [Source:HGNC Symbol;Acc:HGNC:2279]	12511	-0.881786644	-0.28653777
MYH11	myosin heavy chain 11 [Source:HGNC Symbol;Acc:HGNC:7569]	12552	-0.906806648	-0.2819406
CKMT2	creatine kinase, mitochondrial 2 [Source:HGNC Symbol;Acc:HGNC:1996]	12559	-0.911583304	-0.27466273
SLN	sarcolipin [Source:HGNC Symbol;Acc:HGNC:11089]	12629	-0.950680017	-0.27194476
ADCY9	adenylate cyclase 9 [Source:HGNC Symbol;Acc:HGNC:240]	12631	-0.951063335	-0.26394325
LDB3	LIM domain binding 3 [Source:HGNC Symbol;Acc:HGNC:15710]	12662	-0.968739986	-0.25804344
SSPN	sarcospan [Source:HGNC Symbol;Acc:HGNC:11322]	12665	-0.975099981	-0.24991538
MYBPC3	myosin binding protein C3 [Source:HGNC Symbol;Acc:HGNC:7551]	12666	-0.975499988	-0.2416286
CDH13	cadherin 13 [Source:HGNC Symbol;Acc:HGNC:1753]	12787	-1.085886717	-0.24172233
CKM	creatine kinase, M-type [Source:HGNC Symbol;Acc:HGNC:1994]	12815	-1.134549975	-0.23418105
SORBS1	sorbin and SH3 domain containing 1 [Source:HGNC Symbol;Acc:HGNC:14565]	12826	-1.149676681	-0.22519118
ACSL1	acyl-CoA synthetase long chain family member 1 [Source:HGNC Symbol;Acc:HGNC:3569]	12838	-1.17513001	-0.21606274
MYOZ1	myozenin 1 [Source:HGNC Symbol;Acc:HGNC:13752]	12845	-1.183253288	-0.20647703
MYH1	myosin heavy chain 1 [Source:HGNC Symbol;Acc:HGNC:7567]	12883	-1.236310005	-0.19884783
HRC	histidine rich calcium binding protein [Source:HGNC Symbol;Acc:HGNC:5178]	12902	-1.277106643	-0.18939666
ACTC1	actin alpha cardiac muscle 1 [Source:HGNC Symbol;Acc:HGNC:143]	12904	-1.277343392	-0.17862342
MB	myoglobin [Source:HGNC Symbol;Acc:HGNC:6915]	12954	-1.441476703	-0.17018317
TCAP	titin-cap [Source:HGNC Symbol;Acc:HGNC:11610]	12969	-1.515963316	-0.15839234
MYLPF	myosin light chain, phosphorylatable, fast skeletal muscle [Source:HGNC Symbol;Acc:HGNC:29824]	12973	-1.544690013	-0.14550331
BDKRB2	bradykinin receptor B2 [Source:HGNC Symbol;Acc:HGNC:1030]	12980	-1.572113276	-0.13261428
MYL1	myosin light chain 1 [Source:HGNC Symbol;Acc:HGNC:7582]	12986	-1.592456698	-0.11947478
ACTN3	actinin alpha 3 [Source:HGNC Symbol;Acc:HGNC:165]	12987	-1.593743324	-0.1059361
CASQ1	calsequestrin 1 [Source:HGNC Symbol;Acc:HGNC:1512]	12994	-1.677936673	-0.09214811
TNNI2	troponin I2, fast skeletal type [Source:HGNC Symbol;Acc:HGNC:11946]	13005	-1.839653373	-0.077296965
CFD	complement factor D [Source:HGNC Symbol;Acc:HGNC:2771]	13018	-2.079580069	-0.06056297
ATP2A1	ATPase sarcoplasmic/endoplasmic reticulum Ca2+ transporting 1 [Source:HGNC Symbol;Acc:HGNC:811]	13020	-2.153290033	-0.042348646
TNNT3	troponin T3, fast skeletal type [Source:HGNC Symbol;Acc:HGNC:11950]	13021	-2.19936657	-0.023665251
ACTA1	actin alpha 1, skeletal muscle [Source:HGNC Symbol;Acc:HGNC:129]	13030	-2.913803339	0.000465992

XENOBIOTIC METABOLISM

166	-0.26663172	0.00403226	0.20912057	0.87	1112

CD36	CD36 molecule [Source:HGNC Symbol;Acc:HGNC:1663]	12690	-0.760673344	-0.2590618
ACOX2	acyl-CoA oxidase 2 [Source:HGNC Symbol;Acc:HGNC:120]	12734	-0.772010028	-0.25453275
CYP2S1	cytochrome P450 family 2 subfamily S member 1 [Source:HGNC Symbol;Acc:HGNC:15654]	12762	-0.781026661	-0.24874051
EPHX1	epoxide hydrolase 1 [Source:HGNC Symbol;Acc:HGNC:3401]	12766	-0.782016695	-0.24117823
MPP2	membrane palmitoylated protein 2 [Source:HGNC Symbol;Acc:HGNC:7220]	12831	-0.800566673	-0.23790513
CYFIP2	cytoplasmic FMR1 interacting protein 2 [Source:HGNC Symbol;Acc:HGNC:13760]	12895	-0.820623338	-0.2343591
RETSAT	retinol saturase [Source:HGNC Symbol;Acc:HGNC:25991]	12968	-0.843850017	-0.23124199
EPHA2	EPH receptor A2 [Source:HGNC Symbol;Acc:HGNC:3386]	13013	-0.865553319	-0.22585537
SLC6A6	solute carrier family 6 member 6 [Source:HGNC Symbol;Acc:HGNC:11052]	13078	-0.894156694	-0.2216509
COMT	catechol-O-methyltransferase [Source:HGNC Symbol;Acc:HGNC:2228]	13139	-0.925356686	-0.21684258
ENPEP	glutamyl aminopeptidase [Source:HGNC Symbol;Acc:HGNC:3355]	13151	-0.931503356	-0.2083794
FBLN1	fibulin 1 [Source:HGNC Symbol;Acc:HGNC:3600]	13289	-0.996543348	-0.20850989
GNMT	glycine N-methyltransferase [Source:HGNC Symbol;Acc:HGNC:4415]	13341	-1.039503336	-0.20190558
CYP2E1	cytochrome P450 family 2 subfamily E member 1 [Source:HGNC Symbol;Acc:HGNC:2631]	13382	-1.075736642	-0.19413392
ALDH3A1	aldehyde dehydrogenase 3 family member A1 [Source:HGNC Symbol;Acc:HGNC:405]	13413	-1.102040052	-0.1853671
ACSM1	acyl-CoA synthetase medium chain family member 1 [Source:HGNC Symbol;Acc:HGNC:18049]	13421	-1.110113382	-0.17483312
ARG2	arginase 2 [Source:HGNC Symbol;Acc:HGNC:664]	13431	-1.122380018	-0.16432373
XDH	xanthine dehydrogenase [Source:HGNC Symbol;Acc:HGNC:12805]	13492	-1.189026713	-0.1568915
SERPINE1	serpin family E member 1 [Source:HGNC Symbol;Acc:HGNC:8583]	13513	-1.200383306	-0.1464126
GSTA3	glutathione S-transferase alpha 3 [Source:HGNC Symbol;Acc:HGNC:4628]	13546	-1.244053364	-0.13637921
PDK4	pyruvate dehydrogenase kinase 4 [Source:HGNC Symbol;Acc:HGNC:8812]	13553	-1.250116706	-0.12437862
VNN1	vanin 1 [Source:HGNC Symbol;Acc:HGNC:12705]	13563	-1.266956687	-0.11243047
ATP2A2	ATPase sarcoplasmic/endoplasmic reticulum Ca2+ transporting 2 [Source:HGNC Symbol;Acc:HGNC:812]	13603	-1.343680024	-0.101919025
PC	pyruvate carboxylase [Source:HGNC Symbol;Acc:HGNC:8636]	13605	-1.348356724	-0.08857409
GCLC	glutamate-cysteine ligase catalytic subunit [Source:HGNC Symbol;Acc:HGNC:4311]	13616	-1.378973365	-0.07558454
AHCY	adenosylhomocysteinase [Source:HGNC Symbol;Acc:HGNC:343]	13700	-1.659579992	-0.06515638
RAP1GAP	RAP1 GTPase activating protein [Source:HGNC Symbol;Acc:HGNC:9858]	13704	-1.690206647	-0.048556186
POR	cytochrome p450 oxidoreductase [Source:HGNC Symbol;Acc:HGNC:9208]	13723	-1.787703276	-0.03208585
ACP1	acid phosphatase 1 [Source:HGNC Symbol;Acc:HGNC:122]	13734	-1.885653377	-0.014054037
FMO3	flavin containing dimethylaniline monoxygenase 3 [Source:HGNC Symbol;Acc:HGNC:3771]	13735	-1.891263366	0.004767013

P53 PATHWAY

181	-0.24378546	0.01898734	0.24048242	0.966	3121

RXRA	retinoid X receptor alpha [Source:HGNC Symbol;Acc:HGNC:10477]	10681	-0.459426671	-0.2391095
MDM2	MDM2 proto-oncogene [Source:HGNC Symbol;Acc:HGNC:6973]	10694	-0.460680008	-0.23530185
RAD51C	RAD51 paralog C [Source:HGNC Symbol;Acc:HGNC:9820]	10717	-0.462466657	-0.23221023
DDIT4	DNA damage inducible transcript 4 [Source:HGNC Symbol;Acc:HGNC:24944]	10772	-0.469220012	-0.23139936
RRP8	ribosomal RNA processing 8 [Source:HGNC Symbol;Acc:HGNC:29030]	10921	-0.485383332	-0.23732561
NUDT15	nudix hydrolase 15 [Source:HGNC Symbol;Acc:HGNC:23063]	10956	-0.488876671	-0.23484625
MXD4	MAX dimerization protein 4 [Source:HGNC Symbol;Acc:HGNC:13906]	11078	-0.501853347	-0.23862247
STOM	stomatin [Source:HGNC Symbol;Acc:HGNC:3383]	11140	-0.50869	-0.23792385
DCXR	dicarbonyl and L-xylulose reductase [Source:HGNC Symbol;Acc:HGNC:18985]	11154	-0.510169983	-0.23368591
CTSF	cathepsin F [Source:HGNC Symbol;Acc:HGNC:2531]	11199	-0.514896691	-0.23167594
BTG1	BTG anti-proliferation factor 1 [Source:HGNC Symbol;Acc:HGNC:1130]	11236	-0.518503308	-0.22904189
PERP	p53 apoptosis effector related to PMP22 [Source:HGNC Symbol;Acc:HGNC:17637]	11244	-0.519196689	-0.22427157
ZFP36L1	ZFP36 ring finger protein like 1 [Source:HGNC Symbol;Acc:HGNC:1107]	11323	-0.527779996	-0.2246268
ACVR1B	activin A receptor type 1B [Source:HGNC Symbol;Acc:HGNC:172]	11447	-0.542333305	-0.22813788
PLK2	polo like kinase 2 [Source:HGNC Symbol;Acc:HGNC:19699]	11467	-0.544340014	-0.2239927
CD82	CD82 molecule [Source:HGNC Symbol;Acc:HGNC:6210]	11470	-0.544423342	-0.21859851
JUN	Jun proto-oncogene, AP-1 transcription factor subunit [Source:HGNC Symbol;Acc:HGNC:6204]	11591	-0.560363352	-0.22170582
IRAG2	inositol 1,4,5-triphosphate receptor associated 2 [Source:HGNC Symbol;Acc:HGNC:6690]	11742	-0.582466662	-0.22679082
TGFA	transforming growth factor alpha [Source:HGNC Symbol;Acc:HGNC:11765]	11785	-0.58858335	-0.22388403
RB1	RB transcriptional corepressor 1 [Source:HGNC Symbol;Acc:HGNC:9884]	11794	-0.589039981	-0.21847627
TOB1	transducer of ERBB2, 1 [Source:HGNC Symbol;Acc:HGNC:11979]	11860	-0.598316669	-0.21715912
CCND2	cyclin D2 [Source:HGNC Symbol;Acc:HGNC:1583]	11889	-0.602416694	-0.21308364
TRIAP1	TP53 regulated inhibitor of apoptosis 1 [Source:HGNC Symbol;Acc:HGNC:26937]	11989	-0.618123353	-0.21406123
APP	amyloid beta precursor protein [Source:HGNC Symbol;Acc:HGNC:620]	12052	-0.627916694	-0.21222255
RGS16	regulator of G protein signaling 16 [Source:HGNC Symbol;Acc:HGNC:9997]	12152	-0.644573331	-0.21293093
SLC19A2	solute carrier family 19 member 2 [Source:HGNC Symbol;Acc:HGNC:10938]	12255	-0.663576663	-0.21366617
MKNK2	MAPK interacting serine/threonine kinase 2 [Source:HGNC Symbol;Acc:HGNC:7111]	12333	-0.67851001	-0.21241389
ZNF365	zinc finger protein 365 [Source:HGNC Symbol;Acc:HGNC:18194]	12370	-0.686726689	-0.2080677
PPP1R15A	protein phosphatase 1 regulatory subunit 15A [Source:HGNC Symbol;Acc:HGNC:14375]	12429	-0.698536694	-0.20521657
ITGB4	integrin subunit beta 4 [Source:HGNC Symbol;Acc:HGNC:6158]	12457	-0.704233348	-0.2000314
TPD52L1	TPD52 like 1 [Source:HGNC Symbol;Acc:HGNC:12006]	12701	-0.76572001	-0.21007948
F2R	coagulation factor II thrombin receptor [Source:HGNC Symbol;Acc:HGNC:3537]	12713	-0.767446637	-0.2030762
NOTCH1	notch receptor 1 [Source:HGNC Symbol;Acc:HGNC:7881]	12741	-0.773273349	-0.19718835
EPHX1	epoxide hydrolase 1 [Source:HGNC Symbol;Acc:HGNC:3401]	12766	-0.782016695	-0.19099125
MAPKAPK3	MAPK activated protein kinase 3 [Source:HGNC Symbol;Acc:HGNC:6888]	12803	-0.79327333	-0.18556066
KIF13B	kinesin family member 13B [Source:HGNC Symbol;Acc:HGNC:14405]	12847	-0.805689991	-0.18051763
CYFIP2	cytoplasmic FMR1 interacting protein 2 [Source:HGNC Symbol;Acc:HGNC:13760]	12895	-0.820623338	-0.1756163
CTSD	cathepsin D [Source:HGNC Symbol;Acc:HGNC:2529]	12917	-0.826723337	-0.16874392
RETSAT	retinol saturase [Source:HGNC Symbol;Acc:HGNC:25991]	12968	-0.843850017	-0.16382647
EPHA2	EPH receptor A2 [Source:HGNC Symbol;Acc:HGNC:3386]	13013	-0.865553319	-0.15824758
INHBB	inhibin subunit beta B [Source:HGNC Symbol;Acc:HGNC:6067]	13034	-0.871930003	-0.15084168
FOXO3	forkhead box O3 [Source:HGNC Symbol;Acc:HGNC:3821]	13079	-0.894293308	-0.14497028
BTG2	BTG anti-proliferation factor 2 [Source:HGNC Symbol;Acc:HGNC:1131]	13095	-0.905489981	-0.13685572
PCNA	proliferating cell nuclear antigen [Source:HGNC Symbol;Acc:HGNC:8729]	13127	-0.919856668	-0.12976967
CEBPA	CCAAT enhancer binding protein alpha [Source:HGNC Symbol;Acc:HGNC:1833]	13129	-0.920526683	-0.12047415
TCN2	transcobalamin 2 [Source:HGNC Symbol;Acc:HGNC:11653]	13153	-0.931710005	-0.112680085
SDC1	syndecan 1 [Source:HGNC Symbol;Acc:HGNC:10658]	13164	-0.936983347	-0.10387787
AEN	apoptosis enhancing nuclease [Source:HGNC Symbol;Acc:HGNC:25722]	13171	-0.93945998	-0.09475677
TAP1	transporter 1, ATP binding cassette subfamily B member [Source:HGNC Symbol;Acc:HGNC:43]	13178	-0.94168669	-0.085613
DRAM1	DNA damage regulated autophagy modulator 1 [Source:HGNC Symbol;Acc:HGNC:25645]	13197	-0.949296653	-0.07727284
TM4SF1	transmembrane 4 L six family member 1 [Source:HGNC Symbol;Acc:HGNC:11853]	13264	-0.984306693	-0.07210058
ATF3	activating transcription factor 3 [Source:HGNC Symbol;Acc:HGNC:785]	13327	-1.022729993	-0.06624357
CDH13	cadherin 13 [Source:HGNC Symbol;Acc:HGNC:1753]	13399	-1.085886717	-0.060404554
FOS	Fos proto-oncogene, AP-1 transcription factor subunit [Source:HGNC Symbol;Acc:HGNC:3796]	13466	-1.162256718	-0.05342116
HSPA4L	heat shock protein family A (Hsp70) member 4 like [Source:HGNC Symbol;Acc:HGNC:17041]	13477	-1.180513382	-0.042140346
GPX2	glutathione peroxidase 2 [Source:HGNC Symbol;Acc:HGNC:4554]	13552	-1.249480009	-0.034856576
TNFSF9	TNF superfamily member 9 [Source:HGNC Symbol;Acc:HGNC:11939]	13664	-1.49502337	-0.027790312
STEAP3	STEAP3 metalloreductase [Source:HGNC Symbol;Acc:HGNC:24592]	13725	-1.795106649	-0.013925366
SFN	stratifin [Source:HGNC Symbol;Acc:HGNC:10773]	13738	-1.902033329	0.004552086

UV RESPONSE DN

123	-0.28936574	0.00201207	0.1884113	0.712	2959

VLDLR	very low density lipoprotein receptor [Source:HGNC Symbol;Acc:HGNC:12698]	10165	-0.44388333	-0.2828258
PHF3	PHD finger protein 3 [Source:HGNC Symbol;Acc:HGNC:8921]	10173	-0.44495666	-0.27680853
PTPN21	protein tyrosine phosphatase non-receptor type 21 [Source:HGNC Symbol;Acc:HGNC:9651]	10244	-0.451099992	-0.27554688
RBPMS	RNA binding protein, mRNA processing factor [Source:HGNC Symbol;Acc:HGNC:19097]	10253	-0.452096671	-0.26950133
SLC7A1	solute carrier family 7 member 1 [Source:HGNC Symbol;Acc:HGNC:11057]	10297	-0.456926674	-0.26607692
PIK3CD	phosphatidylinositol-4,5-bisphosphate 3-kinase catalytic subunit delta [Source:HGNC Symbol;Acc:HGNC:8977]	10310	-0.458149999	-0.26024985
RXRA	retinoid X receptor alpha [Source:HGNC Symbol;Acc:HGNC:10477]	10317	-0.459426671	-0.25394246
TOGARAM1	TOG array regulator of axonemal microtubules 1 [Source:HGNC Symbol;Acc:HGNC:19959]	10385	-0.466186672	-0.25222778
ABCC1	ATP binding cassette subfamily C member 1 [Source:HGNC Symbol;Acc:HGNC:51]	10391	-0.466939986	-0.24573277
GJA1	gap junction protein alpha 1 [Source:HGNC Symbol;Acc:HGNC:4274]	10574	-0.48699668	-0.25255764
NOTCH2	notch receptor 2 [Source:HGNC Symbol;Acc:HGNC:7882]	10612	-0.491163343	-0.24816726
PTEN	phosphatase and tensin homolog [Source:HGNC Symbol;Acc:HGNC:9588]	10656	-0.496446669	-0.2441606
ACVR2A	activin A receptor type 2A [Source:HGNC Symbol;Acc:HGNC:173]	10690	-0.499253333	-0.23934333
ATP2C1	ATPase secretory pathway Ca2+ transporting 1 [Source:HGNC Symbol;Acc:HGNC:13211]	10752	-0.505393326	-0.23658946
TGFBR3	transforming growth factor beta receptor 3 [Source:HGNC Symbol;Acc:HGNC:11774]	10811	-0.511640012	-0.23351277
SFMBT1	Scm like with four mbt domains 1 [Source:HGNC Symbol;Acc:HGNC:20255]	10822	-0.512956679	-0.22672439
COL3A1	collagen type III alpha 1 chain [Source:HGNC Symbol;Acc:HGNC:2201]	11036	-0.537150025	-0.23519494
BDNF	brain derived neurotrophic factor [Source:HGNC Symbol;Acc:HGNC:1033]	11079	-0.542500019	-0.23043282
CAV1	caveolin 1 [Source:HGNC Symbol;Acc:HGNC:1527]	11209	-0.559509993	-0.2321124
MET	MET proto-oncogene, receptor tyrosine kinase [Source:HGNC Symbol;Acc:HGNC:7029]	11309	-0.573976696	-0.23127112
PLCB4	phospholipase C beta 4 [Source:HGNC Symbol;Acc:HGNC:9059]	11349	-0.580916643	-0.22571222
PDLIM5	PDZ and LIM domain 5 [Source:HGNC Symbol;Acc:HGNC:17468]	11393	-0.586333334	-0.22038122
TENT4A	terminal nucleotidyltransferase 4A [Source:HGNC Symbol;Acc:HGNC:16705]	11438	-0.59108001	-0.21505718
F3	coagulation factor III, tissue factor [Source:HGNC Symbol;Acc:HGNC:3541]	11445	-0.591936648	-0.20679747
ADORA2B	adenosine A2b receptor [Source:HGNC Symbol;Acc:HGNC:264]	11526	-0.604849994	-0.20403978
VAV2	vav guanine nucleotide exchange factor 2 [Source:HGNC Symbol;Acc:HGNC:12658]	11547	-0.607593358	-0.1966263
PLPP3	phospholipid phosphatase 3 [Source:HGNC Symbol;Acc:HGNC:9229]	11629	-0.621843338	-0.19369519
TGFBR2	transforming growth factor beta receptor 2 [Source:HGNC Symbol;Acc:HGNC:11773]	11631	-0.623083353	-0.18459195
LDLR	low density lipoprotein receptor [Source:HGNC Symbol;Acc:HGNC:6547]	11753	-0.644280016	-0.18440717
PTPRM	protein tyrosine phosphatase receptor type M [Source:HGNC Symbol;Acc:HGNC:9675]	11860	-0.663776696	-0.18278131
CDON	cell adhesion associated, oncogene regulated [Source:HGNC Symbol;Acc:HGNC:17104]	11934	-0.680186689	-0.17837521
COL1A1	collagen type I alpha 1 chain [Source:HGNC Symbol;Acc:HGNC:2197]	12056	-0.709829986	-0.17722467
CAP2	cyclase associated actin cytoskeleton regulatory protein 2 [Source:HGNC Symbol;Acc:HGNC:20039]	12183	-0.742680013	-0.17597474
MAPK14	mitogen-activated protein kinase 14 [Source:HGNC Symbol;Acc:HGNC:6876]	12223	-0.754863322	-0.16785301
CCN1	cellular communication network factor 1 [Source:HGNC Symbol;Acc:HGNC:2654]	12230	-0.756123304	-0.15717426
ATP2B4	ATPase plasma membrane Ca2+ transporting 4 [Source:HGNC Symbol;Acc:HGNC:817]	12460	-0.830656648	-0.16255121
PTGFR	prostaglandin F receptor [Source:HGNC Symbol;Acc:HGNC:9600]	12495	-0.846363306	-0.15269676
CACNA1A	calcium voltage-gated channel subunit alpha1 A [Source:HGNC Symbol;Acc:HGNC:1388]	12871	-1.113983393	-0.1651301
IGFBP5	insulin like growth factor binding protein 5 [Source:HGNC Symbol;Acc:HGNC:5474]	12946	-1.227583289	-0.15273589
FHL2	four and a half LIM domains 2 [Source:HGNC Symbol;Acc:HGNC:3703]	12964	-1.254879951	-0.13555488
INPP4B	inositol polyphosphate-4-phosphatase type II B [Source:HGNC Symbol;Acc:HGNC:6075]	12978	-1.281023383	-0.117681
DUSP1	dual specificity phosphatase 1 [Source:HGNC Symbol;Acc:HGNC:3064]	13089	-1.87762332	-0.09847869
NR1D2	nuclear receptor subfamily 1 group D member 2 [Source:HGNC Symbol;Acc:HGNC:7963]	13110	-2.558636665	-0.062319633
DBP	D-box binding PAR bZIP transcription factor [Source:HGNC Symbol;Acc:HGNC:2697]	13121	-4.287226677	7.67E-05

EPITHELIAL MESENCHYMAL TRANSITION

161	-0.27077866	0.00189394	0.1633067	0.853	2550

GJA1	gap junction protein alpha 1 [Source:HGNC Symbol;Acc:HGNC:4274]	10574	-0.48699668	-0.26580945
NOTCH2	notch receptor 2 [Source:HGNC Symbol;Acc:HGNC:7882]	10612	-0.491163343	-0.26365224
TPM1	tropomyosin 1 [Source:HGNC Symbol;Acc:HGNC:12010]	10655	-0.49643001	-0.26182705
COL4A2	collagen type IV alpha 2 chain [Source:HGNC Symbol;Acc:HGNC:2203]	10662	-0.496936679	-0.2572193
FSTL1	follistatin like 1 [Source:HGNC Symbol;Acc:HGNC:3972]	10793	-0.510223329	-0.26204243
TGFBR3	transforming growth factor beta receptor 3 [Source:HGNC Symbol;Acc:HGNC:11774]	10811	-0.511640012	-0.25813332
CRLF1	cytokine receptor like factor 1 [Source:HGNC Symbol;Acc:HGNC:2364]	10964	-0.528563321	-0.26446658
CALU	calumenin [Source:HGNC Symbol;Acc:HGNC:1458]	10965	-0.528673351	-0.25907212
COL3A1	collagen type III alpha 1 chain [Source:HGNC Symbol;Acc:HGNC:2201]	11036	-0.537150025	-0.25899157

										IGFBP3	insulin like growth factor binding protein 3 [Source:HGNC Symbol;Acc:HGNC:5472]	11045	-0.53840667	-0.254115
										BDNF	brain derived neurotrophic factor [Source:HGNC Symbol;Acc:HGNC:1033]	11079	-0.542500019	-0.25112537
										APLP1	amyloid beta precursor like protein 1 [Source:HGNC Symbol;Acc:HGNC:597]	11106	-0.545013309	-0.24757005
										GLIPR1	GLI pathogenesis related 1 [Source:HGNC Symbol;Acc:HGNC:17001]	11175	-0.555040002	-0.24715266
										FBN1	fibrillin 1 [Source:HGNC Symbol;Acc:HGNC:3603]	11180	-0.555773318	-0.24179028
										COL5A3	collagen type V alpha 3 chain [Source:HGNC Symbol;Acc:HGNC:14864]	11213	-0.559926689	-0.23854569
										JUN	Jun proto-oncogene, AP-1 transcription factor subunit [Source:HGNC Symbol;Acc:HGNC:6204]	11220	-0.560363352	-0.23329076
										ENO2	enolase 2 [Source:HGNC Symbol;Acc:HGNC:3353]	11234	-0.562526643	-0.22855382
										SERPINE2	serpin family E member 2 [Source:HGNC Symbol;Acc:HGNC:8951]	11328	-0.577820003	-0.22983271
										THY1	Thy-1 cell surface antigen [Source:HGNC Symbol;Acc:HGNC:11801]	11354	-0.581216693	-0.22583082
										LGALS1	galectin 1 [Source:HGNC Symbol;Acc:HGNC:6561]	11388	-0.585603356	-0.22240138
										COL6A2	collagen type VI alpha 2 chain [Source:HGNC Symbol;Acc:HGNC:2212]	11498	-0.599826694	-0.2246901
										GPC1	glypican 1 [Source:HGNC Symbol;Acc:HGNC:4449]	11505	-0.600973308	-0.2190208
										TPM2	tropomyosin 2 [Source:HGNC Symbol;Acc:HGNC:12011]	11576	-0.613373339	-0.21816249
										LAMC2	laminin subunit gamma 2 [Source:HGNC Symbol;Acc:HGNC:6493]	11730	-0.64095664	-0.22342607
										ADAM12	ADAM metallopeptidase domain 12 [Source:HGNC Symbol;Acc:HGNC:190]	11748	-0.643679976	-0.21816964
										SGCB	sarcoglycan beta [Source:HGNC Symbol;Acc:HGNC:10806]	11902	-0.672673345	-0.22310959
										NT5E	5'-nucleotidase ecto [Source:HGNC Symbol;Acc:HGNC:8021]	11923	-0.677296638	-0.2177416
										MMP2	matrix metallopeptidase 2 [Source:HGNC Symbol;Acc:HGNC:7166]	11939	-0.681293309	-0.21194707
										CAPG	capping actin protein, gelsolin like [Source:HGNC Symbol;Acc:HGNC:1474]	12026	-0.701780021	-0.21142106
										TGM2	transglutaminase 2 [Source:HGNC Symbol;Acc:HGNC:11778]	12036	-0.703793347	-0.20493406
										RHOB	ras homolog family member B [Source:HGNC Symbol;Acc:HGNC:668]	12055	-0.708573341	-0.19909264
										COL1A1	collagen type I alpha 1 chain [Source:HGNC Symbol;Acc:HGNC:2197]	12056	-0.709829986	-0.19184971
										COL8A2	collagen type VIII alpha 2 chain [Source:HGNC Symbol;Acc:HGNC:2216]	12139	-0.729986668	-0.1907273
										GAS1	growth arrest specific 1 [Source:HGNC Symbol;Acc:HGNC:4165]	12164	-0.736490011	-0.1850639
										CAP2	cyclase associated actin cytoskeleton regulatory protein 2 [Source:HGNC Symbol;Acc:HGNC:20039]	12183	-0.742680013	-0.17887445
										ITGA5	integrin subunit alpha 5 [Source:HGNC Symbol;Acc:HGNC:6141]	12195	-0.746190012	-0.17210914
										CCN1	cellular communication network factor 1 [Source:HGNC Symbol;Acc:HGNC:2654]	12230	-0.756123304	-0.16701691
										COL7A1	collagen type VII alpha 1 chain [Source:HGNC Symbol;Acc:HGNC:2214]	12269	-0.766943336	-0.16212285
										SGCG	sarcoglycan gamma [Source:HGNC Symbol;Acc:HGNC:10809]	12346	-0.792190015	-0.15990283
										ITGA2	integrin subunit alpha 2 [Source:HGNC Symbol;Acc:HGNC:6137]	12375	-0.801103354	-0.15388873
										VIM	vimentin [Source:HGNC Symbol;Acc:HGNC:12692]	12385	-0.80466336	-0.14637248
										QSOX1	quiescin sulfhydryl oxidase 1 [Source:HGNC Symbol;Acc:HGNC:9756]	12387	-0.804736674	-0.1382383
										DCN	decorin [Source:HGNC Symbol;Acc:HGNC:2705]	12488	-0.840266645	-0.13737929
										FMOD	fibromodulin [Source:HGNC Symbol;Acc:HGNC:3774]	12642	-0.925586641	-0.13973856
										LRP1	LDL receptor related protein 1 [Source:HGNC Symbol;Acc:HGNC:6692]	12648	-0.931140006	-0.13062319
										SDC1	syndecan 1 [Source:HGNC Symbol;Acc:HGNC:10658]	12659	-0.936983347	-0.12183393
										FN1	fibronectin 1 [Source:HGNC Symbol;Acc:HGNC:3778]	12674	-0.944563329	-0.11327592
										ABI3BP	ABI family member 3 binding protein [Source:HGNC Symbol;Acc:HGNC:17265]	12712	-0.964253306	-0.10629142
										DST	dystonin [Source:HGNC Symbol;Acc:HGNC:1090]	12739	-0.983423352	-0.098262675
										FBLN1	fibulin 1 [Source:HGNC Symbol;Acc:HGNC:3600]	12761	-0.996543348	-0.08971431
										CD59	CD59 molecule (CD59 blood group) [Source:HGNC Symbol;Acc:HGNC:1689]	12802	-1.031710029	-0.08227295
										BASP1	brain abundant membrane attached signal protein 1 [Source:HGNC Symbol;Acc:HGNC:957]	12824	-1.052016616	-0.07315855
										SNTB1	syntrophin beta 1 [Source:HGNC Symbol;Acc:HGNC:11168]	12861	-1.101426721	-0.06469721
										TIMP3	TIMP metallopeptidase inhibitor 3 [Source:HGNC Symbol;Acc:HGNC:11822]	12891	-1.144383311	-0.05525752
										CCN2	cellular communication network factor 2 [Source:HGNC Symbol;Acc:HGNC:2500]	12936	-1.207753301	-0.046328444
										LAMA3	laminin subunit alpha 3 [Source:HGNC Symbol;Acc:HGNC:6483]	12965	-1.25952661	-0.03563671
										THBS1	thrombospondin 1 [Source:HGNC Symbol;Acc:HGNC:11785]	12998	-1.339050055	-0.024442129
										PTHLH	parathyroid hormone like hormone [Source:HGNC Symbol;Acc:HGNC:9607]	13007	-1.36958003	-0.011084463
										AREG	amphiregulin [Source:HGNC Symbol;Acc:HGNC:651]	13094	-1.94825995	0.002160317
**KRAS SIGNALING DN**				125	-0.2624149	0.02103251	0.171854	0.889	1366	GP2	glycoprotein 2 [Source:HGNC Symbol;Acc:HGNC:4441]	11758	-0.645213306	-0.2536533
										EDN1	endothelin 1 [Source:HGNC Symbol;Acc:HGNC:3176]	11807	-0.654429972	-0.2484594
										GDNF	glial cell derived neurotrophic factor [Source:HGNC Symbol;Acc:HGNC:4232]	11845	-0.66165334	-0.24232113
										NR4A2	nuclear receptor subfamily 4 group A member 2 [Source:HGNC Symbol;Acc:HGNC:7981]	11994	-0.694113314	-0.24428186
										FGFR3	fibroblast growth factor receptor 3 [Source:HGNC Symbol;Acc:HGNC:3690]	11998	-0.694836676	-0.23507719
										SLC16A7	solute carrier family 16 member 7 [Source:HGNC Symbol;Acc:HGNC:10928]	12003	-0.696250021	-0.22593027
										CNTFR	ciliary neurotrophic factor receptor [Source:HGNC Symbol;Acc:HGNC:2170]	12092	-0.718933344	-0.22293785
										CDH16	cadherin 16 [Source:HGNC Symbol;Acc:HGNC:1755]	12476	-0.835163355	-0.24106291
										RGS11	regulator of G protein signaling 11 [Source:HGNC Symbol;Acc:HGNC:9993]	12483	-0.838620007	-0.23013656
										PTGFR	prostaglandin F receptor [Source:HGNC Symbol;Acc:HGNC:9600]	12495	-0.846363306	-0.21948972
										UPK3B	uroplakin 3B [Source:HGNC Symbol;Acc:HGNC:21444]	12584	-0.890309989	-0.21417013
										BTG2	BTG anti-proliferation factor 2 [Source:HGNC Symbol;Acc:HGNC:1131]	12606	-0.905489981	-0.20348974
										CYP39A1	cytochrome P450 family 39 subfamily A member 1 [Source:HGNC Symbol;Acc:HGNC:17449]	12722	-0.968376696	-0.19918728
										SPHK2	sphingosine kinase 2 [Source:HGNC Symbol;Acc:HGNC:18859]	12752	-0.992213309	-0.18794473
										SCGB1A1	secretoglobin family 1A member 1 [Source:HGNC Symbol;Acc:HGNC:12523]	12758	-0.996216655	-0.17480136
										KCNE2	potassium voltage-gated channel subfamily E regulatory subunit 2 [Source:HGNC Symbol;Acc:HGNC:6242]	12830	-1.059136629	-0.1658813
										CKM	creatine kinase, M-type [Source:HGNC Symbol;Acc:HGNC:1994]	12882	-1.134549975	-0.15439847
										LGALS7	galectin 7 [Source:HGNC Symbol;Acc:HGNC:6568]	12962	-1.251413345	-0.14348288
										ACTC1	actin alpha cardiac muscle 1 [Source:HGNC Symbol;Acc:HGNC:143]	12976	-1.277343392	-0.12713747
										ATP6V1B1	ATPase H+ transporting V1 subunit B1 [Source:HGNC Symbol;Acc:HGNC:853]	13009	-1.376946688	-0.11090127
										ZBTB16	zinc finger and BTB domain containing 16 [Source:HGNC Symbol;Acc:HGNC:12930]	13111	-2.569983244	-0.08377285
										EFHD1	EF-hand domain family member D1 [Source:HGNC Symbol;Acc:HGNC:29556]	13113	-2.697613239	-0.0472178
										TFF2	trefoil factor 2 [Source:HGNC Symbol;Acc:HGNC:11756]	13120	-3.522486687	0.000153859
**APICAL JUNCTION**				160	-0.2540586	0.01419878	0.1818225	0.929	3062	INPPL1	inositol polyphosphate phosphatase like 1 [Source:HGNC Symbol;Acc:HGNC:6080]	10062	-0.434980005	-0.24930201
										CAP1	cyclase associated actin cytoskeleton regulatory protein 1 [Source:HGNC Symbol;Acc:HGNC:20040]	10075	-0.435990006	-0.24546012
										YWHAH	tyrosine 3-monooxygenase/tryptophan 5-monooxygenase activation protein eta [Source:HGNC Symbol;Acc:HGNC:12853]	10101	-0.438210011	-0.24259679
										CLDN18	claudin 18 [Source:HGNC Symbol;Acc:HGNC:2039]	10106	-0.439006656	-0.23810476
										CERCAM	cerebral endothelial cell adhesion molecule [Source:HGNC Symbol;Acc:HGNC:23723]	10269	-0.454169989	-0.24563545
										PECAM1	platelet and endothelial cell adhesion molecule 1 [Source:HGNC Symbol;Acc:HGNC:8823]	10270	-0.454206675	-0.24066864
										EXOC4	exocyst complex component 4 [Source:HGNC Symbol;Acc:HGNC:30389]	10320	-0.45958668	-0.23942298
										GNAI2	G protein subunit alpha i2 [Source:HGNC Symbol;Acc:HGNC:4385]	10325	-0.460066676	-0.23470065
										ADAM9	ADAM metallopeptidase domain 9 [Source:HGNC Symbol;Acc:HGNC:216]	10463	-0.474779993	-0.2400774
										CX3CL1	C-X3-C motif chemokine ligand 1 [Source:HGNC Symbol;Acc:HGNC:10647]	10532	-0.482749999	-0.24004416
										AKT2	AKT serine/threonine kinase 2 [Source:HGNC Symbol;Acc:HGNC:392]	10538	-0.483333319	-0.23514456
										PTEN	phosphatase and tensin homolog [Source:HGNC Symbol;Acc:HGNC:9588]	10656	-0.496446669	-0.23874153
										PARVA	parvin alpha [Source:HGNC Symbol;Acc:HGNC:14652]	10722	-0.502633333	-0.23825943
										NECTIN1	nectin cell adhesion molecule 1 [Source:HGNC Symbol;Acc:HGNC:9706]	10747	-0.504683316	-0.23459208
										CDH4	cadherin 4 [Source:HGNC Symbol;Acc:HGNC:1763]	10842	-0.515553355	-0.23620583
										NRXN2	neurexin 2 [Source:HGNC Symbol;Acc:HGNC:8009]	10857	-0.516513348	-0.23163769
										TAOK2	TAO kinase 2 [Source:HGNC Symbol;Acc:HGNC:16835]	10858	-0.51655668	-0.22598906
										RSU1	Ras suppressor protein 1 [Source:HGNC Symbol;Acc:HGNC:10464]	10862	-0.516836643	-0.2205688
										SYK	spleen associated tyrosine kinase [Source:HGNC Symbol;Acc:HGNC:11491]	10951	-0.527143359	-0.22159296
										SYMPK	symplekin [Source:HGNC Symbol;Acc:HGNC:22935]	11044	-0.538173318	-0.22280508
										FLNC	filamin C [Source:HGNC Symbol;Acc:HGNC:3756]	11062	-0.54035002	-0.2182077
										ICAM1	intercellular adhesion molecule 1 [Source:HGNC Symbol;Acc:HGNC:5344]	11066	-0.541023314	-0.21252297
										PCDH1	protocadherin 1 [Source:HGNC Symbol;Acc:HGNC:8655]	11148	-0.55047667	-0.21275197
										SRC	SRC proto-oncogene, non-receptor tyrosine kinase [Source:HGNC Symbol;Acc:HGNC:11283]	11169	-0.554543316	-0.20823082
										FBN1	fibrillin 1 [Source:HGNC Symbol;Acc:HGNC:3603]	11180	-0.555773318	-0.20292479
										ACTN1	actinin alpha 1 [Source:HGNC Symbol;Acc:HGNC:163]	11251	-0.565066695	-0.20214568
										ACTN4	actinin alpha 4 [Source:HGNC Symbol;Acc:HGNC:166]	11333	-0.578786671	-0.20206513
										PKD1	polycystin 1, transient receptor potential channel interacting [Source:HGNC Symbol;Acc:HGNC:9008]	11334	-0.57888335	-0.19573495
										THY1	Thy-1 cell surface antigen [Source:HGNC Symbol;Acc:HGNC:11801]	11354	-0.581216693	-0.19084497
										NF1	neurofibromin 1 [Source:HGNC Symbol;Acc:HGNC:7765]	11433	-0.590483308	-0.19040507
										SDC3	syndecan 3 [Source:HGNC Symbol;Acc:HGNC:10660]	11510	-0.601906657	-0.18968597
										VAV2	vav guanine nucleotide exchange factor 2 [Source:HGNC Symbol;Acc:HGNC:12658]	11547	-0.607593358	-0.18581899
										EPB41L2	erythrocyte membrane protein band 4.1 like 2 [Source:HGNC Symbol;Acc:HGNC:3379]	11590	-0.615783334	-0.1823253
										PLCG1	phospholipase C gamma 1 [Source:HGNC Symbol;Acc:HGNC:9065]	11647	-0.625093341	-0.1798098
										LAMC2	laminin subunit gamma 2 [Source:HGNC Symbol;Acc:HGNC:6493]	11730	-0.64095664	-0.17912655
										TMEM8B	transmembrane protein 8B [Source:HGNC Symbol;Acc:HGNC:21427]	11746	-0.643113315	-0.17325115
										LIMA1	LIM domain and actin binding 1 [Source:HGNC Symbol;Acc:HGNC:24636]	11813	-0.655189991	-0.17117797
										SORBS3	sorbin and SH3 domain containing 3 [Source:HGNC Symbol;Acc:HGNC:30907]	11820	-0.656196654	-0.16446522
										MPZL2	myelin protein zero like 2 [Source:HGNC Symbol;Acc:HGNC:3496]	11913	-0.676046669	-0.16416967
										MMP2	matrix metallopeptidase 2 [Source:HGNC Symbol;Acc:HGNC:7166]	11939	-0.681293309	-0.1586482
										ITGB4	integrin subunit beta 4 [Source:HGNC Symbol;Acc:HGNC:6158]	12038	-0.704233348	-0.15850727
										CD276	CD276 molecule [Source:HGNC Symbol;Acc:HGNC:19137]	12144	-0.730916679	-0.15861458
										MAPK14	mitogen-activated protein kinase 14 [Source:HGNC Symbol;Acc:HGNC:6876]	12223	-0.754863322	-0.15637717
										ARPC2	actin related protein 2/3 complex subunit 2 [Source:HGNC Symbol;Acc:HGNC:705]	12278	-0.769093335	-0.15213272
										KCNH2	potassium voltage-gated channel subfamily H member 2 [Source:HGNC Symbol;Acc:HGNC:6251]	12303	-0.777793348	-0.14547886
										RAC2	Rac family small GTPase 2 [Source:HGNC Symbol;Acc:HGNC:9802]	12348	-0.792379975	-0.14020835
										ITGA2	integrin subunit alpha 2 [Source:HGNC Symbol;Acc:HGNC:6137]	12375	-0.801103354	-0.13345388
										FYB1	FYN binding protein 1 [Source:HGNC Symbol;Acc:HGNC:4036]	12410	-0.81262666	-0.12719053
										ACTN2	actinin alpha 2 [Source:HGNC Symbol;Acc:HGNC:164]	12426	-0.817813337	-0.11940476
										NRAP	nebulin related anchoring protein [Source:HGNC Symbol;Acc:HGNC:7988]	12443	-0.823073328	-0.11163862
										VCL	vinculin [Source:HGNC Symbol;Acc:HGNC:12665]	12452	-0.826849997	-0.10321403
										MYH9	myosin heavy chain 9 [Source:HGNC Symbol;Acc:HGNC:7579]	12464	-0.831960022	-0.094965
										LAMB3	laminin subunit beta 3 [Source:HGNC Symbol;Acc:HGNC:6490]	12514	-0.853929996	-0.08940714
										PTPRC	protein tyrosine phosphatase receptor type C [Source:HGNC Symbol;Acc:HGNC:9666]	12591	-0.892926693	-0.0855057
										ZYX	zyxin [Source:HGNC Symbol;Acc:HGNC:13200]	12620	-0.913276672	-0.07767888
										CLDN4	claudin 4 [Source:HGNC Symbol;Acc:HGNC:2046]	12756	-0.994286656	-0.07722047
										CLDN15	claudin 15 [Source:HGNC Symbol;Acc:HGNC:2036]	12958	-1.247196674	-0.07908786
										LAMA3	laminin subunit alpha 3 [Source:HGNC Symbol;Acc:HGNC:6483]	12965	-1.25952661	-0.065777615
										ACTC1	actin alpha cardiac muscle 1 [Source:HGNC Symbol;Acc:HGNC:143]	12976	-1.277343392	-0.052581105
										ACTG1	actin gamma 1 [Source:HGNC Symbol;Acc:HGNC:144]	12985	-1.309723377	-0.03887623
										ACTN3	actinin alpha 3 [Source:HGNC Symbol;Acc:HGNC:165]	13061	-1.593743324	-0.027234115
										ACTA1	actin alpha 1, skeletal muscle [Source:HGNC Symbol;Acc:HGNC:129]	13115	-2.913803339	0.000540188

Table S4b. Gene Set Enrichment Analysis- Venn Diagram **(p<0.01)**

Pathway	SIZE	Enrichment score	NOM.P.value	FDR.q.value	FWER.p.value	Factor
TNFA_SIGNALING_VIA_NFKB	180	-0.34	<0.01	0.22	0.36	Strain
MYOGENESIS	173	-0.34	<0.01	0.17	0.36	Strain
INFLAMMATORY_RESPONSE	177	-0.30	<0.01	0.18	0.62	Strain
ALLOGRAFT_REJECTION	174	-0.29	<0.01	0.20	0.72	Strain
COMPLEMENT	174	-0.29	<0.01	0.20	0.75	Strain
INTERFERON_GAMMA_RESPONSE	185	-0.28	<0.01	0.19	0.78	Strain
ESTROGEN_RESPONSE_EARLY	180	-0.28	<0.01	0.18	0.81	Strain
XENOBIOTIC_METABOLISM	166	-0.27	<0.01	0.21	0.87	Strain
MTORC1_SIGNALING	189	-0.25	<0.01	0.25	0.94	Strain
TNFA_SIGNALING_VIA_NFKB	180	-0.40	<0.01	0.17	0.12	Sex
MYOGENESIS	160	-0.39	<0.01	0.10	0.13	Sex
BILE_ACID_METABOLISM	93	-0.35	<0.01	0.13	0.33	Sex
INFLAMMATORY_RESPONSE	158	-0.32	<0.01	0.15	0.47	Sex
ESTROGEN_RESPONSE_EARLY	166	-0.30	<0.01	0.19	0.66	Sex
UV_RESPONSE_DN	123	-0.29	<0.01	0.19	0.71	Sex
COMPLEMENT	162	-0.29	<0.01	0.17	0.74	Sex
KRAS_SIGNALING_UP	163	-0.28	<0.01	0.17	0.79	Sex
EPITHELIAL_MESENCHYMAL_TRANSITIO	161	-0.27	<0.01	0.16	0.85	Sex
ALLOGRAFT_REJECTION	152	-0.27	<0.01	0.16	0.86	Sex
FATTY_ACID_METABOLISM	124	-0.26	<0.01	0.17	0.90	Sex
ADIPOGENESIS	180	-0.25	<0.01	0.20	0.96	Sex
MYOGENESIS	159	-0.37	<0.01	0.28	0.18	Interaction
BILE_ACID_METABOLISM	92	-0.34	<0.01	0.21	0.35	Interaction
TNFA_SIGNALING_VIA_NFKB	166	-0.34	<0.01	0.16	0.36	Interaction
FATTY_ACID_METABOLISM	123	-0.31	<0.01	0.16	0.49	Interaction
INFLAMMATORY_RESPONSE	156	-0.30	<0.01	0.17	0.62	Interaction
ALLOGRAFT_REJECTION	151	-0.29	<0.01	0.16	0.65	Interaction
KRAS_SIGNALING_UP	153	-0.29	<0.01	0.17	0.69	Interaction
ESTROGEN_RESPONSE_EARLY	165	-0.27	<0.01	0.15	0.82	Interaction
ADIPOGENESIS	181	-0.27	<0.01	0.15	0.85	Interaction
COMPLEMENT	157	-0.26	<0.01	0.15	0.86	Interaction
MTORC1_SIGNALING	178	-0.24	<0.01	0.20	0.96	Interaction

1. Top pathways (ranked by absolute enrichment score) for each factor
2. Positive or negative enrichment score indicates upregulation or downregulation respectively

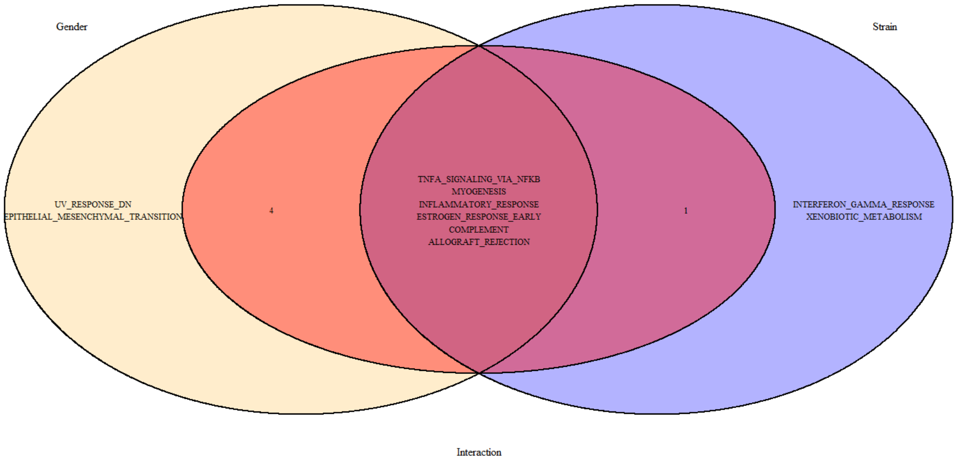

Table S5a. Metabolite feature name in each community for each factor							
Node	Community	m/z	centrality_vec	all_HMDBID	all_Name	all_Adduct	all_Formula
X120	1	163.087	0	HMDB01297 ; HMDB33504 ; HMDB34576 ; HMDB35357 ; HMDB35847 ; HMDB35848 ; HMDB04461 ; HMDB29737 ; HMDB33131 ; HMDB35281	Norcotinine ; AF Toxin II ; 3-Acetoxyscirpene-415-diol ; Blumealactone C ; 4-Acetoxyscirpene-315-diol ; Monoacetoxyscirpenol ; Benzamide ; 1H-Indole-3-carboxaldehyde ; 3-Acetylpyridine ; 2-Acetylpyridine	M+H ; M+2H ; M+2H ; M+2H ; M+2H ; M+2H ; M+ACN+H ; M+NH4 ; M+ACN+H ; M+ACN+H	C9H10N2O ; C17H24O6 ; C17H24O6 ; C17H24O6 ; C17H24O6 ; C17H24O6 ; C7H7NO ; C9H7NO ; C7H7NO ; C7H7NO
X521	1	131.974	0	Unknow	Unknow	Unknow	Unknow
X548	1	87.004	0	Unknow	Unknow	Unknow	Unknow
X554	1	431.002	0	HMDB41985	Phosalone	M+ACN+Na	C12H15ClNO4PS2
X576	1	190.089	0	HMDB12286 ; HMDB30657 ; HMDB34474 ; HMDB35053 ; HMDB36694 ; HMDB36897 ; HMDB36900 ; HMDB36945 ; HMDB41002 ; HMDB41231 ; HMDB41349 ; HMDB31717 ; HMDB32271 ; HMDB32410 ; HMDB32495 ; HMDB36193 ; HMDB37619 ; HMDB38228 ; HMDB38299 ; HMDB39653 ; HMDB40413 ; HMDB41208 ; HMDB41306 ; HMDB61100	S-Prenyl-L-cysteine ; Carinol ; Niveusin C ; Gibberellin A52 ; 15-Hydroxyleptocarpin ; Gibberellin A17 ; Gibberellin A66 ; Gibberellin A125 ; (1E4Z6a8b10a)-8-(2-Methylbutanoyloxy)-1015-dihydroxy-3-oxo-1411(13)-germacratrien-126-olide ; Gibberellin A99 ; Gibberellin A102 ; 3-(Methylthio)propyl acetate ; Ethyl 3-mercaptobutyrate ; Methyl 3-(methylthio)butanoate ; Propyl 2-mercaptopropionate ; 3-Mercapto-3-methylbutyl formate ; Methyl 4-(methylthio)butyrate ; 4-O-alpha-D-Galactopyranosylcalystegine B2 ; Methylthiomethyl butyrate ; 246-Trimethyl-135-dioxathiane ; Ethyl 3-(methylthio)propanoate ; De-O-methylsimmondsin ; Methyl 2-(methylthio)butyrate ; 6-oxo-famciclovir	M+H ; M+2H ; M+2H ; M+2H ; M+2H ; M+2H ; M+2H ; M+2H ; M+2H ; M+2H ; M+2H ; M+ACN+H ; M+ACN+H ; M+ACN+H ; M+ACN+H ; M+ACN+H ; M+ACN+H ; M+ACN+2H ; M+ACN+H ; M+ACN+H ; M+ACN+H ; M+H+NH4 ; M+ACN+H ; M+ACN+2H	C8H15NO2S ; C20H26O7 ; C20H26O7 ; C20H26O7 ; C20H26O7 ; C20H26O7 ; C20H26O7 ; C20H26O7 ; C20H26O7 ; C20H26O7 ; C20H26O7 ; C6H12O2S ; C6H12O2S ; C6H12O2S ; C6H12O2S ; C6H12O2S ; C6H12O2S ; C13H23NO9 ; C6H12O2S ; C6H12O2S ; C6H12O2S ; C15H23NO9 ; C6H12O2S ; C14H19N5O5
X578	1	207.042	0	HMDB04823 ; HMDB13636 ; HMDB15093 ; HMDB61116	Lanthionine ketimine ; Pyrroloquinoline quinone ; Carboplatin ; 2-aminophenol sulphate	M+NH4 ; M+2ACN+2H ; M+ACN+2H ; M+NH4	C6H7NO4S ; C14H6N2O8 ; C6H12N2O4Pt ; C6H7NO4S
X588	1	457.02	0	HMDB29735	5-Ethynyl-5-(1-propynyl)-22-bithiophene	2M+H	C13H8S2
X589	1	180.003	0	Unknow	Unknow	Unknow	Unknow
X599	1	272.955	0	Unknow	Unknow	Unknow	Unknow
X600	1	460.808	0	HMDB10166	PS(18:022:5(7Z10Z13Z16Z19Z))	M+2ACN+2H	C46H80NO10P
X636	1	217.518	0	Unknow	Unknow	Unknow	Unknow
X639	1	414.524	0	Unknow	Unknow	Unknow	Unknow
X664	1	328.917	0	Unknow	Unknow	Unknow	Unknow
X678	1	276.923	0	Unknow	Unknow	Unknow	Unknow
X687	1	270.958	0	Unknow	Unknow	Unknow	Unknow
X691	1	297.904	0	Unknow	Unknow	Unknow	Unknow
X712	1	234.982	0	Unknow	Unknow	Unknow	Unknow
X723	1	366.872	0	Unknow	Unknow	Unknow	Unknow
X760	1	286.932	0	HMDB39734	23579-Pentathiadecane 22-dioxide	M+Na	C5H12O2S5
X762	1	288.929	0	Unknow	Unknow	Unknow	Unknow
X776	1	292.94	0	Unknow	Unknow	Unknow	Unknow
X781	1	236.941	0	Unknow	Unknow	Unknow	Unknow
X837	1	270.978	0	Unknow	Unknow	Unknow	Unknow
X176	2	457.167	0	HMDB03747 ; HMDB15497 ; HMDB15575 ; HMDB29818 ; HMDB32217 ; HMDB34118 ; HMDB34257 ; HMDB34270 ; HMDB37667 ; HMDB41793	Resveratrol ; Oxybenzone ; Trioxsalen ; Benzyl salicylate ; 24-Difurfurylfuran ; (Z)-Resveratrol ; Seselin ; 56-Dihydro-5-hydroxy-6-methyl-2H-pyran-2-one ; o-Tolyl salicylate ; 1-Aminopyrene	2M+H ; 2M+H ; 2M+H ; 2M+H ; 2M+H ; 2M+H ; 2M+H ; 2M+H ; 2M+H ; 2M+Na	C14H12O3 ; C14H12O3 ; C14H12O3 ; C14H12O3 ; C14H12O3 ; C14H12O3 ; C14H12O3 ; C14H12O3 ; C14H12O3 ; C16H11N

X518	2	384.115	0.04	HMDB00912 ; HMDB01062 ; HMDB01121 ; HMDB01367 ; HMDB02817 ; HMDB06480 ; HMDB40546 ; HMDB59626	Succinyladenosine ; N-Acetyl-D-Glucosamine 6-Phosphate ; N-Acetyl-D-mannosamine 6-phosphate ; N-Acetyl-glucosamine 1-phosphate ; N-Acetylglucosamine 6-phosphate ; N-Acetyl-D-galactosamine 1-phosphate ; Pyriminobac-methyl ; N-acetyl-alpha-D-galactosamine 1-phosphate	M+H ; M+2ACN+H ; M+2ACN+H ; M+2ACN+H ; M+2ACN+H ; M+2ACN+H ; M+Na ; M+2ACN+H	C14H17N5O8 ; C8H16NO9P ; C8H16NO9P ; C8H16NO9P ; C8H16NO9P ; C8H16NO9P ; C17H19N3O6 ; C8H16NO9P
X556	2	385.119	0	HMDB29395 ; HMDB30391 ; HMDB40464 ; HMDB40786	L-L-Homoglutathione ; Cepharadione B ; Dowicide A ; Zanthobisquinolone	M+ACN+Na ; M+ACN+Na ; 2M+H ; M+Na	C11H19N3O6S ; C19H15NO4 ; C12H9NaO ; C21H18N2O4
X612	2	113.09	0.001	Unknow	Unknow	Unknow	Unknow
X638	2	434.691	0	Unknow	Unknow	Unknow	Unknow
X651	2	210.05	0	HMDB29261 ; HMDB29502 ; HMDB37466 ; HMDB41774 ; HMDB33249	Kaempferol 3-O-arabinoside ; Kaempferol 3-alpha-L-arabinofuranoside ; Scutellarein 6-xyloside ; Salvianolic acid G ; 6-Chloro-N-(1-methylethyl)-135-triazine-24-diamine	M+2H ; M+2H ; M+2H ; M+2H ; M+Na	C20H18O10 ; C20H18O10 ; C20H18O10 ; C20H18O10 ; C6H10ClN5
X657	2	322.243	0	HMDB06819 ; HMDB29598	2-Hexaprenyl-3-methyl-6-methoxy-14 benzoquinone ; Metenamine	M+2ACN+2H ; 2M+ACN+H	C38H56O3 ; C6H12N4
X676	2	176.066	0.041	HMDB03157 ; HMDB01425 ; HMDB32602 ; HMDB01005	Guanidinosuccinic acid ; Estrone sulfate ; 4-O-Methylkanzonol W ; (S)-Ureidoglycolic acid	M+H ; M+2H ; M+2H ; M+ACN+H	C5H9N3O4 ; C18H22O5S ; C21H18O5 ; C3H6N2O4
X68	2	696.116	0	HMDB35463 ; HMDB39208	Myricetin 7-(6-galloylglucoside) ; 8-C-Ascorbylepigallocatechin 3-gallate	M+ACN+Na ; M+ACN+Na	C28H24O17 ; C28H24O17
X100	3	148.536	0	HMDB01235 ; HMDB12250 ; HMDB14407	5-Aminoimidazole ribonucleotide ; L-Aspartyl-4-phosphate ; Carmustine	M+2H ; M+2ACN+2H ; M+2ACN+2H	C8H14N3O7P ; C4H8NO7P ; C5H9Cl2N3O2
X104	3	279.192	0	HMDB40904	Ipomeatetrahydrofuran	M+Na	C15H28O3
X108	3	383.54	0	Unknow	Unknow	Unknow	Unknow
X113	3	217.54	0	Unknow	Unknow	Unknow	Unknow
X115	3	180.522	0	Unknow	Unknow	Unknow	Unknow
X118	3	140.522	0	Unknow	Unknow	Unknow	Unknow
X127	3	320.992	0	Unknow	Unknow	Unknow	Unknow
X132	3	391.032	0	HMDB30436	Emblicanin B	M+2H	C34H20O22
X134	3	462.102	0	Unknow	Unknow	Unknow	Unknow
X137	3	287.592	0	HMDB29236 ; HMDB37971	Cyanidin 3-(6-acetyl-galactoside) ; Cyanidin 3-(4-acetylglucoside)	M+2ACN+2H ; M+2ACN+2H	C23H23O12 ; C23H23O12
X138	3	132.499	0	Unknow	Unknow	Unknow	Unknow
X139	3	334.027	0	HMDB38429 ; HMDB06343	Methyl glucosinolate ; Selenocystathionine	M+H ; M+ACN+Na	C8H15NO9S2 ; C7H14N2O4Se
X144	3	654.106	0	HMDB39269 ; HMDB39574 ; HMDB59651	Guavin A ; Guajavin A ; 2-(34-Dihydroxybenzoyloxy)-46-dihydroxybenzoate	M+2ACN+2H ; M+2ACN+2H ; 2M+ACN+H	C56H40O32 ; C56H40O32 ; C14H10O8
X145	3	424.065	0	HMDB15270 ; HMDB30451	Cephapirin ; Cefapirin	M+H ; M+H	C17H17N3O6S2 ; C17H17N3O6S2
X147	3	147.023	0	HMDB14024 ; HMDB14025 ; HMDB29633 ; HMDB29634 ; HMDB34448	2-Hydroxychlorpropamide ; 3-Hydroxychlorpropamide ; Phenylmethanethiol ; 2-Methylbenzenethiol ; Methyl phenyl sulfide	M+2H ; M+2H ; M+Na ; M+Na ; M+Na	C10H13ClN2O4S ; C10H13ClN2O4S ; C7H8S ; C7H8S ; C7H8S
X149	3	392.035	0	HMDB30437	Emblicanin A	M+2H	C34H22O22
X153	3	236.084	0	HMDB34007 ; HMDB38463	(EE)-1-Chloro-311-tridecadiene-579-triyn-2-ol ; 3-(Isothiocyanatomethyl)-1-methoxy-1H-indole	M+NH4 ; M+NH4	C13H11ClO ; C11H10N2OS
X155	3	280.564	0	Unknow	Unknow	Unknow	Unknow
X160	3	719.114	0	Unknow	Unknow	Unknow	Unknow
X161	3	388.027	0	HMDB01245	dCDP	M+H	C9H15N3O10P2
X162	3	627.084	0	HMDB60031	5-(35-Dihydroxyphenyl)-gamma-valerolactone-O-sulphate-O-methyl	2M+Na	C12H14O7S
X164	3	234.711	0	Unknow	Unknow	Unknow	Unknow
X165	3	351.563	0	Unknow	Unknow	Unknow	Unknow
X17	3	379.034	0	HMDB60481 ; HMDB31780	heparan sulfate alpha-D-glucosaminide ; Dithianon	M+2H ; M+2ACN+H	C18H32N2O24S3 ; C14H4N2O2S2

X170	3	373.021	0	HMDB32902	Sodium 6-hydroxy-5-(phenylazo)-2-naphthalenesulfoniate	M+Na	C16H11N2NaO4S
X174	3	426.024	0	Unknow	Unknow	Unknow	Unknow
X175	3	449.544	0	Unknow	Unknow	Unknow	Unknow
X178	3	291.048	0	HMDB01068 ; HMDB60509 ; HMDB40610 ; HMDB01915 ; HMDB15492	D-Sedoheptulose 7-phosphate ; Sedoheptulose 1-phosphate ; Epitheaflavic acid 3-gallate ; Alendronic acid ; Stepronin	M+H ; M+H ; M+2H ; M+ACN+H ; M+NH4	C7H15O10P ; C7H15O10P ; C28H20O14 ; C4H13NO7P2 ; C10H11NO4S2
X185	3	275.016	0	HMDB03976 ; HMDB00538 ; HMDB01440 ; HMDB32431 ; HMDB33133 ; HMDB33134 ; HMDB59593	D-Glucuronic acid 1-phosphate ; Adenosine triphosphate ; dGTP ; 5-Methyl-2-thiophenecarboxaldehyde ; 2-Acetylthiophene ; 3-Acetylthiophene ; 2-hydroxy-dATP	M+H ; M+ACN+2H ; M+ACN+2H ; 2M+Na ; 2M+Na ; 2M+Na ; M+ACN+2H	C6H11O10P ; C10H16N5O13P3 ; C10H16N5O13P3 ; C6H6OS ; C6H6OS ; C6H6OS ; C10H16N5O13P3
X186	3	402.514	0	Unknow	Unknow	Unknow	Unknow
X189	3	283.045	0	HMDB34034 ; HMDB40869 ; HMDB01328 ; HMDB06876 ; HMDB00224 ; HMDB29188 ; HMDB60151 ; HMDB60152	Bikojic acid ; 2-O-Caffeoyltartronic acid ; dTDP-D-glucose ; dTDP-D-galactose ; O-Phosphoethanolamine ; 5-Hydroxymethyl-2-furanoate ; O-Phosphonatoethanaminium ; Ethamp	M+H ; M+H ; M+2H ; M+2H ; 2M+H ; 2M+H ; 2M+H ; 2M+H	C12H10O8 ; C12H10O8 ; C16H26N2O16P2 ; C16H26N2O16P2 ; C2H8NO4P ; C6H5O4 ; C2H8NO4P ; C2H8NO4P
X19	3	198.122	0	HMDB29422 ; HMDB00162 ; HMDB03411 ; HMDB04076 ; HMDB12880 ; HMDB13319 ; HMDB30397 ; HMDB30409 ; HMDB32549 ; HMDB32565 ; HMDB34208 ; HMDB41109 ; HMDB60281	L-Histidine trimethylbetaine ; L-Proline ; D-Proline ; 5-Hydroxykynurenamine ; Acetamidopropanal ; Tyrosinamide ; L-2-Amino-3-(4-aminophenyl)propanoic acid ; 4-Amino-2-methylenebutanoic acid ; N-Undecylbenzenesulfonic acid ; (4-Ethoxyphenyl)urea ; Pterolactam ; (S)-3-Ethylidenehexahydropyrrolo12-apyrazine-14-dione ; 3-Hydroxykynurenamine	M+H ; M+2ACN+H ; M+2ACN+H ; M+NH4 ; M+2ACN+H ; M+NH4 ; M+NH4 ; M+2ACN+H ; M+2ACN+2H ; M+NH4 ; M+2ACN+H ; M+NH4 ; M+NH4	C9H15N3O2 ; C5H9NO2 ; C5H9NO2 ; C9H12N2O2 ; C5H9NO2 ; C9H12N2O2 ; C9H12N2O2 ; C5H9NO2 ; C17H28O3S ; C9H12N2O2 ; C5H9NO2 ; C9H12N2O2 ; C9H12N2O2
X198	3	545.067	0	HMDB06049 ; HMDB13926	O-Phosphotyrosine ; Dehydrogenated ticlopidine	2M+Na ; 2M+Na	C9H12NO6P ; C14H12ClNS
X200	3	120.02	0	Unknow	Unknow	Unknow	Unknow
X205	3	201.546	0	Unknow	Unknow	Unknow	Unknow
X216	3	260.481	0	Unknow	Unknow	Unknow	Unknow
X221	3	175.008	0	HMDB60015 ; HMDB34155	Phenol sulphate ; Thiourea	M+H ; 2M+Na	C6H6O4S ; CH4N2S
X222	3	356.032	0	HMDB60449 ; HMDB60687 ; HMDB60692	Carboxyphosphamide ; Carboxycyclophosphamide ; Carboxyifosfamide	M+ACN+Na ; M+ACN+Na ; M+ACN+Na	C7H15Cl2N2O4P ; C7H15Cl2N2O4P ; C7H15Cl2N2O4P
X232	3	273.12	0	HMDB11165 ; HMDB15443 ; HMDB15673 ; HMDB28753 ; HMDB28837 ; HMDB29876 ; HMDB33462 ; HMDB60742	L-beta-aspartyl-L-glycine ; Heptabarbital ; Carglumic acid ; Aspartyl-Glycine ; Glycyl-Aspartate ; Paucine ; 4-Coumaroyl-2-hydroxyputrescine ; 3-Hydroxymelatonin	M+2ACN+H ; M+Na ; M+2ACN+H ; M+2ACN+H ; M+2ACN+H ; M+Na ; M+Na ; M+Na	C6H10N2O5 ; C13H18N2O3 ; C6H10N2O5 ; C6H10N2O5 ; C6H10N2O5 ; C13H18N2O3 ; C13H18N2O3 ; C13H18N2O3
X237	3	287.076	0	HMDB29498 ; HMDB41272 ; HMDB59998 ; HMDB00576 ; HMDB00622 ; HMDB00661 ; HMDB01844 ; HMDB02001 ; HMDB06833 ; HMDB06855 ; HMDB14471 ; HMDB29884 ; HMDB30327 ; HMDB31805 ; HMDB33958 ; HMDB34681 ; HMDB40531	3-Methoxyfukiic acid ; Uralenneoside ; Diphenol glucuronide ; Monoethyl malonic acid ; Ethylmalonic acid ; Glutaric acid ; Methylsuccinic acid ; Dimethylmalonic acid ; 2-Acetolactate ; (S)-2-Acetolactate ; Calcium Gluceptate ; 2-C-Methyl-14-erythrono-D-lactone ; Perlolyrine ; (E)-Monocrotophos ; 2-Deoxy-L-ribono-14-lactone ; 3-Hydroxy-4-butanolide ; 2-Hydroxy-4-oxopentanoic acid	M+H ; M+H ; M+H ; 2M+Na ; 2M+Na ; 2M+Na ; 2M+Na ; 2M+Na ; 2M+Na ; 2M+Na ; M+2ACN+2H ; 2M+Na ; M+Na ; M+ACN+Na ; 2M+Na ; M+Na ; 2M+Na	C12H14O8 ; C12H14O8 ; C12H14O8 ; C5H8O4 ; C5H8O4 ; C5H8O4 ; C5H8O4 ; C5H8O4 ; C5H8O4 ; C5H8O4 ; C14H26CaO16 ; C5H8O4 ; C16H12N2O2 ; C7H14NO5P ; C5H8O4 ; C10H16O8 ; C5H8O4
X238	3	308.524	0	Unknow	Unknow	Unknow	Unknow
X239	3	288.592	0	HMDB35209 ; HMDB60391 ; HMDB60423 ; HMDB40862	Cartormin ; 45-Dihydro-4-hydroxy-5-S-glutathionyl-benzoapyrene ; 78-Dihydro-7-hydroxy-8-S-glutathionyl-benzoapyrene ; Piceatannol 4-galloylglucoside	M+2H ; M+2H ; M+2H ; M+H+NH4	C27H29NO13 ; C30H29N3O7S ; C30H29N3O7S ; C27H26O13

X24	3	239.103	0	HMDB15084 ; HMDB28853 ; HMDB29105 ; HMDB29451 ; HMDB40988 ; HMDB35635 ; HMDB00181 ; HMDB00609 ; HMDB01858 ; HMDB02048 ; HMDB02055 ; HMDB03119 ; HMDB29510 ; HMDB29610 ; HMDB33895 ; HMDB34436 ; HMDB38336 ; HMDB38750 ; HMDB38990 ; HMDB38991 ; HMDB40833 ; HMDB59731 ; HMDB60833	Felbamate ; Glycyl-Tyrosine ; Tyrosyl-Glycine ; gamma-Glutaminyl-4-hydroxybenzene ; Falimint ; Kanokoside A ; L-Dopa ; DL-Dopa ; p-Cresol ; m-Cresol ; o-Cresol ; Benzyl alcohol ; Garcinone B ; Ascladiol ; Anisole ; Rotenone ; 2-Hydroxy-3-(34-dihydroxyphenyl)propanamide ; N-Hydroxy-L-tyrosine ; Methyl 5-hydroxyoxindole-3-acetate ; Methyl dioxindole-3-acetate ; 3-4-Hydroxy-3-(3-methyl-2-butenyl)phenyl-2-propenal ; 23-Methyleneglutaric acid ; N-Acetylserotonin glucuronide	M+H ; M+H ; M+H ; M+H ; M+H ; M+2H ; M+ACN+H ; M+ACN+H ; 2M+Na ; 2M+Na ; 2M+Na ; 2M+Na ; M+2ACN+2H ; M+2ACN+H ; 2M+Na ; M+2ACN+2H ; M+ACN+H ; M+ACN+H ; M+NH4 ; M+NH4 ; M+Na ; M+2ACN+H ; M+2ACN+2H	C11H14N2O4 ; C11H14N2O4 ; C11H14N2O4 ; C11H14N2O4 ; C11H14N2O4 ; C21H32O12 ; C9H11NO4 ; C9H11NO4 ; C7H8O ; C7H8O ; C7H8O ; C7H8O ; C23H22O6 ; C7H8O4 ; C7H8O ; C23H22O6 ; C9H11NO4 ; C9H11NO4 ; C11H11NO4 ; C11H11NO4 ; C14H16O2 ; C7H8O4 ; C18H22N2O8
X242	3	301.114	0	HMDB00098 ; HMDB00283 ; HMDB00366 ; HMDB00621 ; HMDB00646 ; HMDB00751 ; HMDB01644 ; HMDB03162 ; HMDB03371 ; HMDB11112 ; HMDB11176 ; HMDB12153 ; HMDB12194 ; HMDB12325 ; HMDB13067 ; HMDB13141 ; HMDB28996 ; HMDB29029 ; HMDB29113 ; HMDB29578 ; HMDB29941 ; HMDB29942 ; HMDB31161 ; HMDB31472 ; HMDB31709 ; HMDB32180 ; HMDB32408 ; HMDB32775 ; HMDB33192 ; HMDB38174 ; HMDB40056 ; HMDB41934 ; HMDB59753 ; HMDB59856 ; HMDB60254	D-Xylose ; D-Ribose ; 2-Deoxyribonic acid ; D-Ribulose ; L-Arabinose ; L-Threo-2-pentulose ; D-Xylulose ; 7-Methylhypoxanthine ; L-Ribulose ; N1-(alpha-D-ribosyl)-56-dimethyl-benzimidazole ; L-phenylalanyl-L-hydroxyproline ; 34-Dihydroxybenzylamine ; Beta-D-ribopyranose ; Arabinofuranose ; Salsoline-1-carboxylate ; 1-Methylhypoxanthine ; Phenylalanyl-Hydroxyproline ; Prolyl-Tyrosine ; Tyrosyl-Proline ; Diisopropyl disulfide ; D-Apiose ; Arabinose ; Methyl pentyl disulfide ; Dipropyl disulfide ; 16-Hexanedithiol ; Butyl ethyl disulfide ; Methyl isopentyl disulfide ; 3-Hydroxy-carbofuran ; Neoacrimarine K ; 5-Acetyl-24-dimethyloxazole ; xi-1-(Propylthio)-1-propanethiol ; Mizoribine ; 2-Deoxypentonic acid ; Ethyl 2-pyrrolecarboxylate ; Aldehydo-D-xylose	2M+H ; 2M+H ; 2M+H ; 2M+H ; 2M+H ; 2M+H ; 2M+H ; 2M+H ; 2M+H ; M+Na ; M+Na ; 2M+Na ; 2M+H ; 2M+H ; M+ACN+Na ; 2M+H ; M+Na ; M+Na ; M+Na ; 2M+H ; 2M+H ; 2M+H ; 2M+H ; 2M+H ; 2M+H ; 2M+H ; 2M+H ; M+ACN+Na ; M+ACN+2H ; 2M+Na ; 2M+H ; M+ACN+H ; 2M+H ; 2M+Na ; 2M+H	C5H10O5 ; C5H10O5 ; C5H10O5 ; C5H10O5 ; C5H10O5 ; C5H10O5 ; C5H10O5 ; C6H6N4O ; C5H10O5 ; C14H18N2O4 ; C14H18N2O4 ; C7H9NO2 ; C5H10O5 ; C5H10O5 ; C12H15NO4 ; C6H6N4O ; C14H18N2O4 ; C14H18N2O4 ; C14H18N2O4 ; C6H14S2 ; C5H10O5 ; C5H10O5 ; C6H14S2 ; C6H14S2 ; C6H14S2 ; C6H14S2 ; C6H14S2 ; C12H15NO4 ; C31H29NO9 ; C7H9NO2 ; C6H14S2 ; C9H13N3O6 ; C5H10O5 ; C7H9NO2 ; C5H10O5
X254	3	455.09	0	HMDB01022 ; HMDB01269 ; HMDB02255 ; HMDB02310 ; HMDB06794	Succinyl-CoA ; Methylmalonyl-CoA ; R-Methylmalonyl-CoA ; S-Methylmalonyl-CoA ; 5-(2-Carboxyethyl)-46-Dihydroxypicolinate	M+ACN+2H ; M+ACN+2H ; M+ACN+2H ; M+ACN+2H ; 2M+H	C25H40N7O19P3S ; C25H40N7O19P3S ; C25H40N7O19P3S ; C25H40N7O19P3S ; C9H9NO6
X256	3	144.031	0	HMDB05800 ; HMDB05801 ; HMDB29711 ; HMDB30543 ; HMDB34014 ; HMDB34150 ; HMDB34444 ; HMDB41658 ; HMDB41688 ; HMDB41702	Luteolin ; Kaempferol ; Maritimetin ; Norartocarpetin ; 2-Hydroxygenistein ; Cernuine ; Citreorosein ; 3-Hydroxygenistein ; 5674-Tetrahydroxyisoflavone ; 7834-Tetrahydroxyisoflavone	M+2H ; M+2H ; M+2H ; M+2H ; M+2H ; M+2H ; M+2H ; M+2H ; M+2H ; M+2H	C15H10O6 ; C15H10O6 ; C15H10O6 ; C15H10O6 ; C15H10O6 ; C15H10O6 ; C15H10O6 ; C15H10O6 ; C15H10O6 ; C15H10O6
X258	3	154.038	0	Unknow	Unknow	Unknow	Unknow
X270	3	136.496	0	Unknow	Unknow	Unknow	Unknow
X276	3	305.953	0	HMDB39459	Di-2-propenyl pentasulfide	M+ACN+Na	C6H10S5
X284	3	128.51	0	HMDB14695	Lamotrigine	M+2H	C9H7Cl2N5
X286	3	231.009	0	HMDB00691 ; HMDB01352 ; HMDB06938 ; HMDB15373 ; HMDB31159	Malonic acid ; Hydroxypyruvic acid ; Tartronate semialdehyde ; Chloroxine ; Garcinia acid	2M+Na ; 2M+Na ; 2M+Na ; M+NH4 ; M+Na	C3H4O4 ; C3H4O4 ; C3H4O4 ; C9H5Cl2NO ; C6H8O8
X293	3	255.065	0	HMDB03312 ; HMDB04195 ; HMDB30670 ; HMDB30699 ; HMDB30874 ; HMDB33153 ; HMDB36619 ; HMDB38509 ; HMDB29418 ; HMDB30813 ; HMDB33330 ; HMDB38911 ; HMDB41325	Daidzein ; 5-L-Glutamyl-taurine ; Chrysophanol ; 57-Dihydroxyisoflavone ; Phomarin ; (Z)-46-Dihydroxyaurone ; 57-Dihydroxyflavone ; Rheidin B ; S-Cysteinosuccinic acid ; Cassiachromone ; Dihydrocoriandrin ; (2S3S4S)-57911-Tridecatetrayne-1234-tetrol ; Salfredin B11	M+H ; M+H ; M+H ; M+H ; M+H ; M+H ; M+H ; M+2H ; M+NH4 ; M+Na ; M+Na ; M+Na ; M+Na	C15H10O4 ; C7H14N2O6S ; C15H10O4 ; C15H10O4 ; C15H10O4 ; C15H10O4 ; C15H10O4 ; C30H20O8 ; C7H11NO6S ; C13H12O4 ; C13H12O4 ; C13H12O4 ; C13H12O4

X311	3	446.105	0	HMDB60723 ; HMDB29222 ; HMDB36377 ; HMDB40609 ; HMDB59663 ; HMDB60022	23-diene-Valproic acid-CoA ; Urolithin A-3-O-glucuronide ; 7-Hydroxy-2-methyl-4-oxo-4H-1-benzopyran-5-carboxylic acid 7-glucoside ; Theaflavic acid ; 5-hydroxy-2-oxo-4-ureido-25-dihydro-1H-imidazole-5-carboxylate ; Urolithin A-8-O-glucuronide	M+2H ; M+ACN+H ; M+ACN+Na ; M+NH4 ; 2M+ACN+H ; M+ACN+H	C29H47N7O17P3S ; C19H16O10 ; C17H18O10 ; C21H16O10 ; C5H6N4O5 ; C19H16O10
X32	3	250.483	0	Unknow	Unknow	Unknow	Unknow
X320	3	334.519	0	Unknow	Unknow	Unknow	Unknow
X323	3	406.146	0	HMDB32809 ; HMDB00118 ; HMDB00333 ; HMDB00423 ; HMDB00755 ; HMDB02643 ; HMDB04061 ; HMDB13682 ; HMDB14600 ; HMDB15024 ; HMDB29227 ; HMDB29232 ; HMDB29273 ; HMDB29545 ; HMDB29547 ; HMDB29646 ; HMDB29979 ; HMDB30095 ; HMDB30107 ; HMDB31955 ; HMDB32767 ; HMDB33275 ; HMDB33624 ; HMDB36335 ; HMDB37274 ; HMDB37552 ; HMDB37571 ; HMDB38630 ; HMDB39334 ; HMDB40663 ; HMDB40722 ; HMDB41270 ; HMDB41273 ; HMDB59763	4-Hydroxyphenylacetonitrile triacetylrhamnoside ; Homovanillic acid ; Isohomovanillic acid ; 34-Dihydroxyhydrocinnamic acid ; Hydroxyphenyllactic acid ; 3-(3-Hydroxyphenyl)-3-hydroxypropanoic acid ; 3-Methoxy-4-hydroxyphenylglycolaldehyde ; Coniferin ; Prazosin ; Bumetanide ; 5-Hydroxy-33478-pentamethoxyflavone ; 3-Hydroxyphenyllactate ; 26-Dimethoxybenzoic acid ; 3-Hydroxy-45678-pentamethoxyflavone ; 4-Hydroxy-35678-pentamethoxyflavone ; 26-Dihydroxy-4-methoxyacetophenone ; 2-Hydroxy-34578-pentamethoxyflavone ; Artemetin ; 3-Hydroxy-3-(34-dihydroxy 4-methylpentanoyl)-5-(3-methylbutyl)-124-cyclopentanetrione ; Dimethyl (1R2S3S)-2-carboxy-3-(34-dihydroxyphenyl)-23-dihydro-56-dihydroxy-1H-indene-1-acetate ; Sphalleroside A ; 5-Hydroxyauranetin ; ()-2-Hydroxy-3-(2-hydroxyphenyl)propanoic acid ; 246-Phenanthrenetriol 2-O-b-D-glucoside ;	M+H ; 2M+ACN+H ; 2M+ACN+H ; 2M+ACN+H ; 2M+ACN+H ; 2M+ACN+H ; 2M+ACN+H ; M+ACN+Na ; M+Na ; M+ACN+H ; M+NH4 ; 2M+ACN+H ; 2M+ACN+H ; M+NH4 ; M+NH4 ; 2M+ACN+H ; M+NH4 ; M+NH4 ; M+ACN+Na ; M+NH4 ; M+ACN+Na ; M+NH4 ; 2M+ACN+H ; M+NH4 ; 2M+ACN+H ; M+ACN+Na ; M+NH4 ; M+Na ; M+ACN+Na ; M+ACN+Na ; M+NH4 ; 2M+ACN+H ; M+ACN+Na ; 2M+ACN+H	C20H23NO8 ; C9H10O4 ; C9H10O4 ; C9H10O4 ; C9H10O4 ; C9H10O4 ; C9H10O4 ; C16H22O8 ; C19H21N5O4 ; C17H20N2O5S ; C20H20O8 ; C9H10O4 ; C9H10O4 ; C20H20O8 ; C20H20O8 ; C9H10O4 ; C20H20O8 ; C20H20O8 ; C16H22O8 ; C20H20O8 ; C16H22O8 ; C20H20O8 ; C9H10O4 ; C20H20O8 ; C9H10O4 ; C16H22O8 ; C20H20O8 ; C18H25NO8 ; C16H22O8 ; C16H22O8 ; C20H20O8 ; C9H10O4 ; C16H22O8 ; C9H10O4
X325	3	434.192	0	HMDB14937 ; HMDB15172 ; HMDB15196 ; HMDB33319 ; HMDB36524 ; HMDB41099 ; HMDB61008	Tazarotene ; Carphenazine ; Acetophenazine ; Licarin C ; 3b8b-Dihydroxy-6b-(3-chloro-2-hydroxy-2-methylbutanoyloxy)-7(11)-eremophilen-128-olide ; Kanzonol R ; m-chlorophenylpiperazine (m-CPP)	M+2ACN+H ; M+Na ; M+Na ; M+ACN+Na ; M+NH4 ; M+ACN+Na ; 2M+ACN+H	C21H21NO2S ; C23H29N3O2S ; C23H29N3O2S ; C22H26O5 ; C20H29ClO7 ; C22H26O5 ; C10H13ClN2
X328	3	210.127	0	HMDB15171 ; HMDB30929 ; HMDB31660 ; HMDB32610 ; HMDB38754 ; HMDB41477	Selegiline ; 2468-Tridecatetrayne ; Dibutyl sulfide ; 4-Methylbiphenyl ; (EEE)-13511-Tridecatetraene-79-diyne ; 2-Ethyl-1-hexanethiol	M+Na ; M+ACN+H ; M+ACN+Na ; M+ACN+H ; M+ACN+H ; M+ACN+Na	C13H17N ; C13H12 ; C8H18S ; C13H12 ; C13H12 ; C8H18S

X337	3	153.054	0	HMDB00020 ; HMDB00440 ; HMDB00669 ; HMDB00703 ; HMDB01101 ; HMDB02390 ; HMDB03791 ; HMDB04815 ; HMDB12308 ; HMDB29657 ; HMDB29658 ; HMDB29659 ; HMDB29660 ; HMDB29661 ; HMDB29662 ; HMDB31609 ; HMDB32399 ; HMDB32572 ; HMDB32594 ; HMDB32604 ; HMDB32606 ; HMDB32629 ; HMDB32919 ; HMDB33003 ; HMDB34172 ; HMDB36907 ; HMDB41283 ; HMDB60390 ; HMDB01067 ; HMDB29175 ; HMDB29179 ; HMDB29228 ; HMDB29231 ; HMDB30662 ; HMDB30745 ; HMDB33866 ; HMDB38519 ; HMDB41294 ; HMDB41507 ; HMDB29944	p-Hydroxyphenylacetic acid ; 3-Hydroxyphenylacetic acid ; Ortho-Hydroxyphenylacetic acid ; Mandelic acid ; p-Anisic acid ; 3-Cresotinic acid ; 34-Dihydroxyphenylacetaldehyde ; 4-Hydroxy-3-methylbenzoic acid ; Vanillin ; 24-Dihydroxyacetophenone ; 23-Dihydroxyacetophenone ; 24-Dihydroxyacetophenone ; 26-Dihydroxyacetophenone ; 34-Dihydroxyacetophenone ; 35-Dihydroxyacetophenone ; Phenoxyacetic acid ; Methyl furfuracrylate ; Methylparaben ; 2-(Hydroxymethyl)benzoic acid ; 2-Methoxybenzoic acid ; 3-Methoxybenzoic acid ; 25-Dihydroxyacetophenone ; Ethyl 2-furanyl diketone ; 1-(5-Methyl-2-furanyl)-12-propanedione ; Methyl 2-hydroxybenzoate ; 2-Propenyl 2-furancarboxylate ; 1-(2-Furanyl)-13-butanedione ; 4-Hydroxyphenyl acetate ; N-Acetylaspartylglutamic acid ; 3-O-methyl-(-)-epicatechin ; 4-O-methyl-(-)-epicatechin ; 4-Methyl-epicatechin ; 3-Methyl-epicatechin ; 4-	M+H ; M+2H ; M+2H ; M+2H ; M+2H ; M+2H ; M+2H ; M+2H ; M+2H ; M+2H ; M+2H ; M+2H ; M+ACN+2H	C8H8O3 ; C11H16N2O8 ; C16H16O6 ; C16H16O6 ; C16H16O6 ; C16H16O6 ; C16H16O6 ; C16H16O6 ; C16H16O6 ; C16H16O6 ; C16H16O6 ; C16H16O6 ; C9H13NO8
X35	3	170.005	0	Unknow	Unknow	Unknow	Unknow
X357	3	279.065	0	HMDB05760 ; HMDB29462 ; HMDB29519 ; HMDB30808 ; HMDB32577 ; HMDB33904 ; HMDB36457 ; HMDB37316 ; HMDB41647	Dihydrodaidzein ; (E)-244-Trihydroxychalcone ; (2S)-Liquiritigenin ; (S)-Pinocembrin ; 4-Methoxybenzophenone-2-carboxylic acid ; 7E-Mycosinyl acetate ; Emodinanthranol ; Isoliquiritigenin ; 2-Dehydro-O-desmethylangolensin	M+Na ; M+Na ; M+Na ; M+Na ; M+Na ; M+Na ; M+Na ; M+Na ; M+Na	C15H12O4 ; C15H12O4 ; C15H12O4 ; C15H12O4 ; C15H12O4 ; C15H12O4 ; C15H12O4 ; C15H12O4 ; C15H12O4
X363	3	159.044	0	HMDB33566 ; HMDB34453 ; HMDB60688	Ajoene ; 4-22-Bithiophen-5-yl-3-butyn-1-ol ; Nornitrogen mustard	M+2ACN+2H ; M+2ACN+2H ; M+NH4	C9H14OS3 ; C12H10OS2 ; C4H9Cl2N
X365	3	265.14	0	HMDB00107 ; HMDB00247 ; HMDB00765 ; HMDB02095 ; HMDB11632 ; HMDB30987 ; HMDB33699 ; HMDB38736	Galactitol ; Sorbitol ; Mannitol ; Malonylcarnitine ; L-Iditol ; 2-Carboxy-4-dodecanolide ; Linamarin ; (3S5R6R7E)-356-Trihydroxy-7-megastigmen-9-one	M+2ACN+H ; M+2ACN+H ; M+2ACN+H ; M+NH4 ; M+2ACN+H ; M+Na ; M+NH4 ; M+Na	C6H14O6 ; C6H14O6 ; C6H14O6 ; C10H17NO6 ; C6H14O6 ; C13H22O4 ; C10H17NO6 ; C13H22O4
X366	3	276.012	0	Unknow	Unknow	Unknow	Unknow
X368	3	471.077	0	HMDB14809	Cefixime	M+NH4	C16H15N5O7S2
X374	3	152.057	0	HMDB00132 ; HMDB00403 ; HMDB00542 ; HMDB00156 ; HMDB00744 ; HMDB31518 ; HMDB32872 ; HMDB38974 ; HMDB39789 ; HMDB39790	Guanine ; 2-Hydroxyadenine ; 8-Hydroxyadenine ; L-Malic acid ; Malic acid ; D-Malic acid ; Velcorin ; 33-Dimethyl-12-dithiolane ; Tetrahydro-2-methyl-2-thiophenethiol ; Tetrahydro-2-methyl-3-thiophenethiol	M+H ; M+H ; M+H ; M+NH4 ; M+NH4 ; M+NH4 ; M+NH4 ; M+NH4 ; M+NH4 ; M+NH4	C5H5N5O ; C5H5N5O ; C5H5N5O ; C4H6O5 ; C4H6O5 ; C4H6O5 ; C4H6O5 ; C5H10S2 ; C5H10S2 ; C5H10S2
X375	3	223.564	0	Unknow	Unknow	Unknow	Unknow
X378	3	351.563	0	Unknow	Unknow	Unknow	Unknow
X388	3	271.985	0	Unknow	Unknow	Unknow	Unknow
X392	3	728.129	0	HMDB00902	NAD	M+ACN+Na	C21H28N7O14P2
X399	3	196.013	0	HMDB31727	Alphachloralose	M+2ACN+2H	C8H11Cl3O6
X402	3	157.01	0	Unknow	Unknow	Unknow	Unknow
X407	3	512.604	0	Unknow	Unknow	Unknow	Unknow
X440	3	219.042	0	HMDB15025 ; HMDB30878	Mechlorethamine ; Methyl 2-propenyl selenide	M+ACN+Na ; M+2ACN+H	C5H11Cl2N ; C4H8Se
X441	3	334.036	0	Unknow	Unknow	Unknow	Unknow
X471	3	243.944	0	Unknow	Unknow	Unknow	Unknow
X48	3	330.059	0	HMDB00058 ; HMDB11616 ; HMDB14492 ; HMDB31800 ; HMDB10716 ; HMDB32956	Cyclic AMP ; Adenosine 23-cyclic phosphate ; Nitisinone ; Mecarbam ; Gamma-Glutamyl-Se-methylselenocysteine ; 2-O-p-Coumaroyltartronic acid	M+H ; M+H ; M+H ; M+H ; M+NH4 ; M+ACN+Na	C10H12N5O6P ; C10H12N5O6P ; C14H10F3NO5 ; C10H20NO5PS2 ; C9H16N2O5Se ; C12H10O7

X488	3	309.582	0	HMDB34707 ; HMDB37656 ; HMDB37657 ; HMDB38716 ; HMDB40678 ; HMDB59635	Epicatechin-(2beta-74beta-6)-catechin ; Proanthocyanidin A1 ; Proanthocyanidin A5 ; Pavetannin A2 ; Epicatechin-(2beta-54beta-6)-ent-epicatechin ; Lipoyl-AMP	M+ACN+2H ; M+ACN+2H ; M+ACN+2H ; M+ACN+2H ; M+ACN+2H ; M+2ACN+2H	C30H24O12 ; C30H24O12 ; C30H24O12 ; C30H24O12 ; C30H24O12 ; C18H26N5O8PS2
X496	3	456.916	0	Unknow	Unknow	Unknow	Unknow
X498	3	346.032	0	Unknow	Unknow	Unknow	Unknow
X514	3	150.014	0.08	Unknow	Unknow	Unknow	Unknow
X52	3	469.067	0	HMDB40628 ; HMDB00061 ; HMDB00960 ; HMDB01341 ; HMDB32937 ; HMDB33342 ; HMDB33352	3-(23-Digalloyl-46-hexahydroxydiphenoylglucosyl)-phloroacetophenone ; Adenosine 35-diphosphate ; dGDP ; ADP ; Methyl 4-chloro-1H-indole-3-acetate ; Mollicellin E ; Cyclobrassinin	M+2H ; M+ACN+H ; M+ACN+H ; M+ACN+H ; 2M+Na ; M+Na ; 2M+H	C42H32O25 ; C10H15N5O10P2 ; C10H15N5O10P2 ; C10H15N5O10P2 ; C11H10ClNO2 ; C22H19ClO8 ; C11H10N2S2
X520	3	141.951	0.085	Unknow	Unknow	Unknow	Unknow
X523	3	132.004	0.069	Unknow	Unknow	Unknow	Unknow
X526	3	235.962	0.13	Unknow	Unknow	Unknow	Unknow
X527	3	424.732	0.427	Unknow	Unknow	Unknow	Unknow
X529	3	548.371	0.147	HMDB10392 ; HMDB00138 ; HMDB00331 ; HMDB32596 ; HMDB33409 ; HMDB34516 ; HMDB35257	LysoPC(20:2(11Z14Z)) ; Glycocholic acid ; 3a7b12a-Trihydroxyoxocholanyl-Glycine ; Sodium glycocholate ; 26-Methyl nigranoate ; 3b18b-3-Methoxy-11-oxo-12-oleanen-30-oic acid ; Methyl 3b24-dihydroxy-1113(18)-oleanadien-30-oate	M+H ; M+2ACN+H ; M+2ACN+H ; M+2ACN+H ; M+ACN+Na ; M+ACN+Na ; M+ACN+Na	C28H54NO7P ; C26H43NO6 ; C26H43NO6 ; C26H43NO6 ; C31H48O4 ; C31H48O4 ; C31H48O4
X534	3	449.763	0.013	Unknow	Unknow	Unknow	Unknow
X536	3	252.144	0.058	HMDB00001 ; HMDB00479 ; HMDB00670 ; HMDB15238 ; HMDB29416	1-Methylhistidine ; 3-Methylhistidine ; Homo-L-arginine ; Levocabastine ; L-Targinine	M+2ACN+H ; M+2ACN+H ; M+ACN+Na ; M+2ACN+2H ; M+ACN+Na	C7H11N3O2 ; C7H11N3O2 ; C7H16N4O2 ; C26H29FN2O2 ; C7H16N4O2
X542	3	431.73	0.209	Unknow	Unknow	Unknow	Unknow
X551	3	140.013	0.005	HMDB42046 ; HMDB02287 ; HMDB31188	Treosulfan ; Homocysteine thiolactone ; Ethanethioic acid	M+2H ; M+Na ; M+ACN+Na	C6H14O8S2 ; C4H7NOS ; C2H4OS
X552	3	244.154	0.009	HMDB02366 ; HMDB00792 ; HMDB31510 ; HMDB40196 ; HMDB59708 ; HMDB59719 ; HMDB59754	Tiglylcarnitine ; Sebacic acid ; R-2-Hydroxy-3-methylbutanoic acid 3-Methylbutanoyl ; Oxalic acid dibutyl ester ; 2-Ethylsuberic acid ; Heptylmalonic acid ; 3-Methylazelaic acid	M+H ; M+ACN+H ; M+ACN+H ; M+ACN+H ; M+ACN+H ; M+ACN+H ; M+ACN+H	C12H21NO4 ; C10H18O4 ; C10H18O4 ; C10H18O4 ; C10H18O4 ; C10H18O4 ; C10H18O4
X553	3	122.019	0.013	Unknow	Unknow	Unknow	Unknow
X555	3	246.17	0.004	HMDB00378 ; HMDB00688 ; HMDB13128 ; HMDB41993 ; HMDB00718 ; HMDB00892 ; HMDB00933 ; HMDB02176 ; HMDB30058 ; HMDB30994 ; HMDB31175 ; HMDB31241 ; HMDB31247 ; HMDB31249 ; HMDB31516 ; HMDB32230 ; HMDB32263 ; HMDB33217 ; HMDB33479 ; HMDB33642 ; HMDB33742 ; HMDB33890 ; HMDB34237 ; HMDB35133 ; HMDB39053 ; HMDB39799 ; HMDB39974 ; HMDB40575 ; HMDB41992 ; HMDB59678	2-Methylbutyroylcarnitine ; Isovalerylcarnitine ; Valerylcarnitine ; Pivaloylcarnitine ; Isovaleric acid ; Valeric acid ; Traumatic acid ; Ethylmethylacetic acid ; Ethyl propionate ; 5-Heptyltetrahydro-2-oxo-3-furancarboxylic acid ; Tetrahydro-2-furanmethanol ; Isopropyl acetate ; 2-Methylpropyl formate ; Methyl isobutyrate ; 3-Hydroxy-2-pentanone ; 24-Dimethyl-13-dioxolane ; Ethylene oxidepropylene oxide copolymer ; (2xi6xi)-7-Methyl-3-methylene-1267-octanetetrol ; Sinapoylspermine ; (2xi3xi6E)-37-Dimethyl-6-octene-1238-tetrol ; (S)-2-Methylbutanoic acid ; Methyl butyrate ; Propyl acetate ; 37-Dimethyl-3-octene-1267-tetrol ; (1alpha2alpha4betaH6alpha8R)-p-Menthane-2689-tetrol ; Tetrahydro-2-methyl-3-furanol ; (1S2S4R8R)-p-Menthane-1289-tetrol ; Butyl formate ; Pivalic acid ; 1-Hydroxy-2-pentanone	M+H ; M+H ; M+H ; M+H ; 2M+ACN+H ; 2M+ACN+H ; M+NH4 ; 2M+ACN+H ; 2M+ACN+H ; M+NH4 ; 2M+ACN+H ; 2M+ACN+H ; 2M+ACN+H ; 2M+ACN+H ; 2M+ACN+H ; 2M+ACN+H ; 2M+ACN+H ; M+ACN+H ; M+2ACN+2H ; M+ACN+H ; 2M+ACN+H ; 2M+ACN+H ; 2M+ACN+H ; M+ACN+H ; M+ACN+H ; 2M+ACN+H ; M+ACN+H ; 2M+ACN+H ; 2M+ACN+H ; 2M+ACN+H	C12H23NO4 ; C12H23NO4 ; C12H23NO4 ; C12H23NO4 ; C5H10O2 ; C5H10O2 ; C12H20O4 ; C5H10O2 ; C5H10O2 ; C12H20O4 ; C5H10O2 ; C5H10O2 ; C5H10O2 ; C5H10O2 ; C5H10O2 ; C5H10O2 ; C5H10O2 ; C10H20O4 ; C21H36N4O4 ; C10H20O4 ; C5H10O2 ; C5H10O2 ; C5H10O2 ; C10H20O4 ; C10H20O4 ; C5H10O2 ; C10H20O4 ; C5H10O2 ; C5H10O2 ; C5H10O2
X558	3	145.035	0.059	Unknow	Unknow	Unknow	Unknow

X562	3	546.355	0.037	HMDB10393 ; HMDB10394 ; HMDB04888 ; HMDB10384 ; HMDB11128 ; HMDB15110 ; HMDB32111	LysoPC(20:3(5Z8Z11Z)) ; LysoPC(20:3(8Z11Z14Z)) ; Ganglioside GA2 (d18:112:0) ; LysoPC(18:0) ; LysoPC(0:018:0) ; Dipyridamole ; Adlupone	M+H ; M+H ; M+2ACN+2H ; M+Na ; M+Na ; M+ACN+H ; M+ACN+Na	C28H52NO7P ; C28H52NO7P ; C50H92N2O18 ; C26H54NO7P ; C26H54NO7P ; C24H40N8O4 ; C31H46O4
X567	3	314.171	0.021	HMDB00726 ; HMDB00953 ; HMDB15038 ; HMDB15114 ; HMDB15330 ; HMDB15341 ; HMDB15371 ; HMDB30917 ; HMDB31901 ; HMDB34721 ; HMDB35117 ; HMDB35137 ; HMDB35148 ; HMDB35358 ; HMDB35760 ; HMDB35798 ; HMDB36036 ; HMDB36037 ; HMDB36327 ; HMDB36550 ; HMDB36563 ; HMDB36664 ; HMDB37064 ; HMDB37529 ; HMDB37559 ; HMDB39156 ; HMDB39635 ; HMDB39644 ; HMDB40754	Isovalerylglutamic acid ; Suberylglycine ; Methdilazine ; Cyclopentolate ; Tubocurarine ; Levobunolol ; Gemfibrozil ; 1-Hydroxyacorenone ; Blennin A ; Procurcumadiol ; (3beta6beta)-Furanoeremophilane-36-diol ; Heliannuol D ; (6beta8alpha)-6-Hydroxy-7(11)-eremophilen-128-olide ; Ketosantalic acid ; 3-Hydroxytrichothecene ; Piperdial ; 3beta-Dihydroxymarasmene ; 13-Hydroxymarasmene ; Norcapsaicin ; 3-Ketoapotrichothecene ; Valerenolic acid ; Ketopelenolide a ; FS4 toxin ; Lactaronecatorin A ; Cadabicilone ; 3beta-Hydroxycinnamolide ; Abscisic alcohol ; Heliannuol A ; (3beta8beta)-3-Hydroxy-7(11)-eremophilen-128-olide	M+2ACN+H ; M+2ACN+H ; M+NH4 ; M+Na ; M+H+NH4 ; M+Na ; M+ACN+Na ; M+ACN+Na ; M+ACN+Na ; M+ACN+Na ; M+ACN+Na ; M+ACN+Na ; M+ACN+Na ; M+ACN+Na ; M+ACN+Na ; M+ACN+Na ; M+ACN+Na ; M+ACN+Na ; M+Na ; M+ACN+Na ; M+ACN+Na ; M+ACN+Na ; M+ACN+Na ; M+ACN+Na ; M+ACN+Na ; M+ACN+Na ; M+ACN+Na ; M+ACN+Na ; M+ACN+Na	C10H17NO5 ; C10H17NO5 ; C18H20N2S ; C17H25NO3 ; C37H41N2O6 ; C17H25NO3 ; C15H22O3 ; C15H22O3 ; C15H22O3 ; C15H22O3 ; C15H22O3 ; C15H22O3 ; C15H22O3 ; C15H22O3 ; C15H22O3 ; C15H22O3 ; C15H22O3 ; C15H22O3 ; C17H25NO3 ; C15H22O3 ; C15H22O3 ; C15H22O3 ; C15H22O3 ; C15H22O3 ; C15H22O3 ; C15H22O3 ; C15H22O3 ; C15H22O3 ; C15H22O3
X572	3	471.104	0.022	HMDB12158 ; HMDB60858	3-Oxopimelyl-CoA ; o-O-sulfate rosiglitazone	M+H+NH4 ; M+NH4	C28H44N7O20P3S ; C18H19N3O7S2
X579	3	523.273	0.234	HMDB41579 ; HMDB02248 ; HMDB04985 ; HMDB04987 ; HMDB28758 ; HMDB28947 ; HMDB34750	78-Dihydro-3b6a-dihydroxy-alpha-ionol 9-apiosyl-(1-6)-glucoside ; Gamma glutamyl ornithine ; Aspartylysine ; Alpha-Aspartyl-lysine ; Aspartyl-Lysine ; Lysyl-Aspartate ; Isopentyl beta-D-glucoside	M+H ; 2M+H ; 2M+H ; 2M+H ; 2M+H ; 2M+H ; 2M+Na	C24H42O12 ; C10H19N3O5 ; C10H19N3O5 ; C10H19N3O5 ; C10H19N3O5 ; C10H19N3O5 ; C11H22O6
X583	3	124.956	0.306	Unknow	Unknow	Unknow	Unknow
X590	3	193.998	0.239	HMDB15142 ; HMDB34852	Tioconazole ; Sulfentrazone	M+2H ; M+2H	C16H13Cl3N2OS ; C11H10Cl2F2N4O3S
X597	3	125.964	0.619	Unknow	Unknow	Unknow	Unknow
X598	3	158.003	0.004	HMDB01928 ; HMDB32930	Hydrochlorothiazide ; Benzothiazole	M+H+NH4 ; M+Na	C7H8ClN3O4S2 ; C7H5NS
X60	3	603.029	0	HMDB00935 ; HMDB12302 ; HMDB12304	Uridine diphosphate glucuronic acid ; UDP-D-galacturonate ; UDP-L-iduronate	M+Na ; M+Na ; M+Na	C15H22N2O18P2 ; C15H22N2O18P2 ; C15H22N2O18P2
X602	3	178.024	0.225	HMDB60173	N-Methylethanolaminium phosphate	M+Na	C3H10NO4P
X604	3	198.124	0.199	HMDB31964 ; HMDB40569 ; HMDB05805 ; HMDB32627 ; HMDB37013 ; HMDB37050 ; HMDB37051 ; HMDB59812 ; HMDB59821 ; HMDB59823 ; HMDB59832 ; HMDB59890	(3b6b8b12a)-812-Epoxy-7(11)-eremophilene-6-angeloyloxy-812-dimethoxy-3-ol ; 3-Methoxy-6-Gingerdiol 35-diacetate ; p-Cymene ; 1-Methyl-2-propylbenzene ; p-Mentha-138-triene ; 1-Isopropyl-2-methylbenzene ; 1-Isopropyl-3-methylbenzene ; Butylbenzene ; m-Propyltoluene ; Prehnitene ; 4-Ethyl-o-xylene ; 2-Ethyl-p-xylene	M+2H ; M+2H ; M+ACN+Na ; M+ACN+Na ; M+ACN+Na ; M+ACN+Na ; M+ACN+Na ; M+ACN+Na ; M+ACN+Na ; M+ACN+Na ; M+ACN+Na ; M+ACN+Na	C22H34O6 ; C22H34O6 ; C10H14 ; C10H14 ; C10H14 ; C10H14 ; C10H14 ; C10H14 ; C10H14 ; C10H14 ; C10H14 ; C10H14
X607	3	711.731	0.011	Unknow	Unknow	Unknow	Unknow
X616	3	149.019	0.005	HMDB14406	Anagrelide	M+ACN+2H	C10H7Cl2N3O
X618	3	265.112	0.013	HMDB14462 ; HMDB38748 ; HMDB60549 ; HMDB60624	Gefitinib ; Osmanthuside A ; Norketamine ; Ethionamide sulphoxide	M+2ACN+2H ; M+2ACN+2H ; M+ACN+H ; M+2ACN+H	C22H24ClFN4O3 ; C23H26O9 ; C12H14ClNO ; C8H10N2OS
X625	3	140.951	0.004	Unknow	Unknow	Unknow	Unknow
X631	3	160.527	0.008	HMDB15039	Ethacrynic acid	M+H+NH4	C13H12Cl2O4
X634	3	333.564	0.011	Unknow	Unknow	Unknow	Unknow

X646	3	318.168	0.002	HMDB15234 ; HMDB35587 ; HMDB36359 ; HMDB39765 ; HMDB41860 ; HMDB61077 ; HMDB61078 ; HMDB00279 ; HMDB04284 ; HMDB14333 ; HMDB29563 ; HMDB29671 ; HMDB29672 ; HMDB30755 ; HMDB30764 ; HMDB30922 ; HMDB32036 ; HMDB32136 ; HMDB32137 ; HMDB32139 ; HMDB32159 ; HMDB32290 ; HMDB34241 ; HMDB37245 ; HMDB37246 ; HMDB38325 ; HMDB39802 ; HMDB40174 ; HMDB40177 ; HMDB41607	Arbutamine ; Pandamarilactonine A ; Pandanamine ; Pandamarilactone 1 ; Cocaethylene ; alpha-oxycodol ; beta-oxycodol ; Saccharopine ; Tyrosol ; Esmolol ; 3-Acetyl-25-dimethylfuran ; 14-Dimethoxybenzene ; 13-Dimethoxybenzene ; Myrigalone E ; Acetylpterosin C ; 13-Diacetoxy-4612-tetradecatriene-810-diyne ; 4-Ethoxyphenol ; 2-Methoxy-4-methylphenol ; 26-Dimethyl-14-benzenediol ; 12-Dimethoxybenzene ; 2-Acetyl-35-dimethylfuran ; 1-(2-Furyl)butan-3-one ; 4-Methoxybenzyl alcohol ; Myrigalone A ; Myrigalon B ; Verimol C ; 1-(2-Furanyl)-1-butanone ; 4-Ethyl-12-benzenediol ; 3-Ethyl-12-benzenediol ; 2-Phenoxyethanol	M+H ; M+H ; M+H ; M+H ; M+H ; M+H ; M+H ; M+ACN+H ; 2M+ACN+H ; M+Na ; 2M+ACN+H ; 2M+ACN+H ; 2M+ACN+H ; M+NH4 ; M+ACN+H ; M+NH4 ; 2M+ACN+H ; 2M+ACN+H ; 2M+ACN+H ; 2M+ACN+H ; 2M+ACN+H ; 2M+ACN+H ; 2M+ACN+H ; M+NH4 ; M+NH4 ; M+NH4 ; 2M+ACN+H ; 2M+ACN+H ; 2M+ACN+H ; 2M+ACN+H	C18H23NO4 ; C18H23NO4 ; C18H23NO4 ; C18H23NO4 ; C18H23NO4 ; C18H23NO4 ; C18H23NO4 ; C11H20N2O6 ; C8H10O2 ; C16H25NO4 ; C8H10O2 ; C8H10O2 ; C8H10O2 ; C18H20O4 ; C16H20O4 ; C18H20O4 ; C8H10O2 ; C8H10O2 ; C8H10O2 ; C8H10O2 ; C8H10O2 ; C8H10O2 ; C8H10O2 ; C18H20O4 ; C18H20O4 ; C18H20O4 ; C8H10O2 ; C8H10O2 ; C8H10O2 ; C8H10O2
X65	3	271.068	0	Unknow	Unknow	Unknow	Unknow
X668	3	88.0043	0.068	Unknow	Unknow	Unknow	Unknow
X669	3	133.097	0.002	HMDB00214 ; HMDB03374 ; HMDB32455 ; HMDB28961 ; HMDB29066	Ornithine ; D-Ornithine ; L-Ornithine monochlorohydrateornithine ; Lysyl-Threonine ; Threoninyl-Lysine	M+H ; M+H ; M+H ; M+H+NH4 ; M+H+NH4	C5H12N2O2 ; C5H12N2O2 ; C5H12N2O2 ; C10H21N3O4 ; C10H21N3O4
X671	3	665.117	0.003	HMDB06555 ; HMDB30802 ; HMDB33648	dIMP ; Patuletin ; 45678-Pentahydroxy-3-methoxyflavone	2M+H ; 2M+H ; 2M+H	C10H13N4O7P ; C16H12O8 ; C16H12O8
X673	3	432.944	0.004	Unknow	Unknow	Unknow	Unknow
X688	3	117.074	0.009	HMDB32411 ; HMDB40244 ; HMDB06468 ; HMDB13704 ; HMDB29410 ; HMDB38513 ; HMDB39998 ; HMDB39999 ; HMDB41939	2-Methyl-1-methylthio-2-butene ; 1-Propenyl propyl sulfide ; 4-Hydroxydebrisoquine ; 6-Methylnicotinamide ; 2-Acetyl-3-ethylpyrazine ; 78-Dihydro-3-methylpyrrolo12-apyrimidin-2(6H)-one ; 2-Acetyl-36-dimethylpyrazine ; 2-Acetyl-35-dimethylpyrazine ; N-nitrosoanabasine	M+H ; M+H ; M+ACN+2H ; M+2ACN+2H ; M+2ACN+2H ; M+2ACN+2H ; M+2ACN+2H ; M+2ACN+2H ; M+ACN+2H	C6H12S ; C6H12S ; C10H13N3O ; C8H10N2O ; C8H10N2O ; C8H10N2O ; C8H10N2O ; C8H10N2O ; C10H13N3O
X692	3	130.086	0.002	HMDB00070 ; HMDB00716 ; HMDB04226 ; HMDB05960 ; HMDB15212 ; HMDB29435 ; HMDB29444 ; HMDB59649 ; HMDB00039 ; HMDB00824 ; HMDB00990 ; HMDB01164 ; HMDB01873 ; HMDB03243 ; HMDB29581 ; HMDB30062 ; HMDB31217 ; HMDB31305 ; HMDB31344 ; HMDB31507 ; HMDB31543 ; HMDB31759 ; HMDB32233 ; HMDB33569 ; HMDB34247 ; HMDB39778 ; HMDB40253 ; HMDB40260 ; HMDB40579	Pipecolic acid ; L-Pipecolic acid ; N4-Acetylaminobutanal ; D-Pipecolic acid ; Vigabatrin ; L-trans-4-Methyl-2-pyrrolidinecarboxylic acid ; 2-Pyrrolidineacetic acid ; 3-Acetamidobutanal ; Butyric acid ; Propionylcarnitine ; Acetaldehyde ; trans-12-Dihydrobenzene-12-diol ; Isobutyric acid ; Acetoin ; (2E4E)-24-Hexadienoic acid ; Methyl propionate ; Ethyl acetate ; Oxirane ; 12-Cyclohexanedione ; 1-Hydroxy-2-butanone ; 3-Methyl-12-cyclopentanedione ; (E)-4-Oxo-2-hexen-1-al ; 25-Dimethyl-3(2H)-furanone ; Syoyualdehyde ; 2-(Methoxymethyl)furan ; xi-35-Dimethyl-2(5H)-furanone ; Propyl formate ; 55-Dimethyl-2(5H)-furanone ; Isopropyl formate	M+H ; M+H ; M+H ; M+H ; M+H ; M+H ; M+H ; M+H ; M+ACN+H ; M+ACN+2H ; 2M+ACN+H ; M+NH4 ; M+ACN+H ; M+ACN+H ; M+NH4 ; M+ACN+H ; M+ACN+H ; 2M+ACN+H ; M+NH4 ; M+ACN+H ; M+NH4 ; M+NH4 ; M+NH4 ; M+NH4 ; M+NH4 ; M+NH4 ; M+ACN+H ; M+NH4 ; M+ACN+H	C6H11NO2 ; C6H11NO2 ; C6H11NO2 ; C6H11NO2 ; C6H11NO2 ; C6H11NO2 ; C6H11NO2 ; C6H11NO2 ; C4H8O2 ; C10H19NO4 ; C2H4O ; C6H8O2 ; C4H8O2 ; C4H8O2 ; C6H8O2 ; C4H8O2 ; C4H8O2 ; C2H4O ; C6H8O2 ; C4H8O2 ; C6H8O2 ; C6H8O2 ; C6H8O2 ; C6H8O2 ; C6H8O2 ; C6H8O2 ; C4H8O2 ; C6H8O2 ; C4H8O2
X695	3	195.996	0.096	Unknow	Unknow	Unknow	Unknow
X696	3	102.013	0.004	Unknow	Unknow	Unknow	Unknow
X701	3	957.477	0.075	HMDB31024 ; HMDB33406 ; HMDB37912	Achyranthoside C ; Quillaic acid 3-xylosyl-(1-3)-galactosyl-(1-2)-glucuronide ; Tragopogonsaponin C	M+H ; M+H ; M+H	C47H72O20 ; C47H72O20 ; C51H72O17
X702	3	547.359	0.018	HMDB41547	Hovenidulcigenin B	M+H	C32H50O7

X711	3	115.087	0.002	HMDB00323 ; HMDB61162 ; HMDB00182 ; HMDB01106 ; HMDB01875 ; HMDB01888 ; HMDB02134 ; HMDB03405 ; HMDB12114 ; HMDB12115 ; HMDB31339 ; HMDB32968 ; HMDB32969 ; HMDB32970 ; HMDB60177	3-Amino-2-piperidone ; N-Mononitrosopiperazine ; L-Lysine ; 3-Aminopropionaldehyde ; Methanol ; NN-Dimethylformamide ; Aminoacetone ; D-Lysine ; (3S)-36-Diaminohexanoate ; (3S5S)-35-Diaminohexanoate ; 1-Cyano-2-hydroxy-3-butene ; 24-Dimethyloxazole ; 25-Dimethyloxazole ; 45-Dimethyloxazole ; Ammonioacetone	M+H ; M+H ; M+2ACN+2H ; M+ACN+H ; M+2ACN+H ; M+ACN+H ; M+ACN+H ; M+2ACN+2H ; M+2ACN+2H ; M+2ACN+2H ; M+NH4 ; M+NH4 ; M+NH4 ; M+NH4 ; M+ACN+H	C5H10N2O ; C5H10N2O ; C6H14N2O2 ; C3H7NO ; CH4O ; C3H7NO ; C3H7NO ; C6H14N2O2 ; C6H14N2O2 ; C6H14N2O2 ; C5H7NO ; C5H7NO ; C5H7NO ; C5H7NO ; C3H7NO
X713	3	319.224	0.154	HMDB01337 ; HMDB05081 ; HMDB06027 ; HMDB10202 ; HMDB10205 ; HMDB10209 ; HMDB10210 ; HMDB10212 ; HMDB10217 ; HMDB12534 ; HMDB12611 ; HMDB13633 ; HMDB30101 ; HMDB35116 ; HMDB35311 ; HMDB36698 ; HMDB36706 ; HMDB36712 ; HMDB36713 ; HMDB36724 ; HMDB36752 ; HMDB36776 ; HMDB36806 ; HMDB36839 ; HMDB36930 ; HMDB37523 ; HMDB38521 ; HMDB39012 ; HMDB39716 ; HMDB41058 ; HMDB60053 ; HMDB61114 ; HMDB36928 ; HMDB04667 ; HMDB04670 ; HMDB04701 ; HMDB04702 ; HMDB10223 ; HMDB29796 ; HMDB29978 ; HMDB29998 ; HMDB34194 ; HMDB40057	Leukotriene A4 ; 5-HEPE ; Oxymesterone ; 12-HEPE ; 1415-EpETE ; 15-HEPE ; 15-KETE ; 1718-EpETE ; 5-KETE ; 11R-HEPE ; 18R-HEPE ; 12-KETE ; Methyl 8-Shogaol ; (ent-7alpha)-7-Hydroxy-8(14)15-pimaradien-19-oic acid ; Galanal A ; Ineketone ; Steviol ; Yucalexin B20 ; Yucalexin B22 ; ent-17-Hydroxy-15-kauren-19-oic acid ; Yucalexin P17 ; Yucalexin A19 ; Aframodial ; (ent-15beta)-15-Hydroxy-19-trachylobanoic acid ; Grandiflorolic acid ; Galanolactone ; 2-Hydroxy-6-(8-tridecenyl)benzoic acid ; (ent-15beta)-1519-Dihydroxy-7-trachylobanone ; 12alpha-12-Hydroxy-713-abietadien-18-oic acid ; Phytocassane C ; 9-HEPE ; Atractyligenin ; Mactraxanthin ; 13S-hydroxyoctadecadienoic acid ; Alpha-dimorphecolic acid ; 910-Epoxyoctadecenoic acid ; 1213-EpOME ; 9-HODE ; (Z)-13-Oxo-9-octadecenoic acid ; Avenoleic acid ; 12-Hydroxy-810-octadecadienoic acid ; Jurubine ; ()-2-Pentylthiazolidine	M+H ; M+2H ; M+Na ; M+Na ; M+Na ; M+Na ; M+Na ; M+Na ; M+Na ; M+Na ; M+ACN+2H ; 2M+H	C20H30O3 ; C40H60O6 ; C18H32O3 ; C18H32O3 ; C18H32O3 ; C18H32O3 ; C18H32O3 ; C18H32O3 ; C18H32O3 ; C18H32O3 ; C33H57NO8 ; C8H17NS
X714	3	341.863	0.181	Unknow	Unknow	Unknow	Unknow
X716	3	559.3	0.009	HMDB03141 ; HMDB11728 ; HMDB15266 ; HMDB28888 ; HMDB28889 ; HMDB28909 ; HMDB28931 ; HMDB29321 ; HMDB29560 ; HMDB30153 ; HMDB31802	Retinoyl b-glucuronide ; Bradykinin hydroxyproline ; Doxacurium chloride ; Histidinyl-Isoleucine ; Histidinyl-Leucine ; Isoleucyl-Histidine ; Leucyl-Histidine ; Asparagoside F ; Hesperaline ; Austalide H ; ()-Metalaxyl	M+2ACN+H ; M+ACN+2H ; M+2ACN+2H ; 2M+Na ; 2M+Na ; 2M+Na ; 2M+Na ; M+2ACN+2H ; 2M+Na ; M+2ACN+H ; 2M+H	C26H36O8 ; C50H73N15O12 ; C56H78N2O16 ; C12H20N4O3 ; C12H20N4O3 ; C12H20N4O3 ; C12H20N4O3 ; C50H82O22 ; C14H22NO4 ; C26H36O8 ; C15H21NO4
X717	3	580.016	0.008	HMDB29855 ; HMDB32885	Cochineal Red A ; Amaranth	M+ACN+H ; M+ACN+H	C20H14N2O10S3 ; C20H14N2O10S3
X722	3	162.05	0.002	HMDB37110	4-(34-Dihydroxyphenyl)-23-dihydro-23-dihydroxy-1H-phenalen-1-one	M+2H	C19H14O5
X725	3	131.089	0.092	HMDB31537 ; HMDB01240 ; HMDB01329 ; HMDB01497 ; HMDB15133 ; HMDB60679	3-Methylcyclohexanethiol ; Pseudooxynicotine ; 2-Hydroxynicotine ; Nicotine-1-N-oxide ; Frovatriptan ; Glycinexylidide	M+H ; M+2ACN+2H ; M+2ACN+2H ; M+2ACN+2H ; M+H+NH4 ; M+2ACN+2H	C7H14S ; C10H14N2O ; C10H14N2O ; C10H14N2O ; C14H17N3O ; C10H14N2O
X727	3	229.105	0.002	HMDB32496 ; HMDB14843 ; HMDB15327 ; HMDB60410	Propyl propane thiosulfonate ; Delavirdine ; Estramustine ; 6-Carboxy-5678-tetrahydropterin	M+H ; M+2H ; M+H+NH4 ; M+NH4	C11H16O5 ; C22H28N6O3S ; C23H31Cl2NO3 ; C7H9N5O3
X73	3	278.071	0	HMDB15274	Amifostine	M+ACN+Na	C5H15N2O3PS
X731	3	136.049	0.118	HMDB00562 ; HMDB06357 ; HMDB39506 ; HMDB60320	Creatinine ; cis-2-Methylaconitate ; N-Salicyloylaspartic acid ; (Z)-But-1-ene-124-tricarboxylate	M+Na ; M+2ACN+2H ; M+H+NH4 ; M+2ACN+2H	C4H7N3O ; C7H8O6 ; C11H11NO6 ; C7H8O6
X736	3	523.361	0.016	Unknow	Unknow	Unknow	Unknow
X737	3	395.551	0.142	Unknow	Unknow	Unknow	Unknow
X74	3	727.122	0	HMDB39720	a-L-threo-4-Hex-4-enopyranuronosyl-D-galacturonic acid	2M+Na	C12H16O12
X740	3	309.484	0.012	Unknow	Unknow	Unknow	Unknow
X753	3	238.974	0.038	Unknow	Unknow	Unknow	Unknow

X766	3	431.073	0.001	HMDB60067 ; HMDB61103 ; HMDB38486	CMP-2-aminoethylphosphonate ; lamotrigine-2-N-glucuronide ; Tetraphyllin B sulfate	M+H ; M+H ; M+ACN+Na	C11H20N4O10P2 ; C16H18Cl2N5O5 ; C12H17NO10S
X771	3	118.967	0.058	Unknow	Unknow	Unknow	Unknow
X772	3	129.102	0.011	HMDB00108 ; HMDB31163 ; HMDB31308 ; HMDB37837 ; HMDB37838 ; HMDB37858 ; HMDB37859 ; HMDB37863 ; HMDB39584 ; HMDB40148 ; HMDB60817	Ethanol ; N-Acetyl-23-dihydro-1H-pyrrole ; 5-Acetyl-34-dihydro-2H-pyrrole ; 2-Ethyl-4-methyloxazole ; 4-Ethyl-2-methyloxazole ; 2-Ethyl-5-methyloxazole ; 5-Ethyl-2-methyloxazole ; 5-Ethyl-4-methyloxazole ; 5-Imino-2-methyl-1-cyclopenten-1-ol ; Trimethyloxazole ; Diethylcarbamazine N-oxide	M+2ACN+H ; M+NH4 ; M+NH4 ; M+NH4 ; M+NH4 ; M+NH4 ; M+NH4 ; M+NH4 ; M+NH4 ; M+NH4 ; M+ACN+2H	C2H6O ; C6H9NO ; C6H9NO ; C6H9NO ; C6H9NO ; C6H9NO ; C6H9NO ; C6H9NO ; C6H9NO ; C6H9NO ; C10H21N3O2
X796	3	351.856	0.004	Unknow	Unknow	Unknow	Unknow
X804	3	200.165	0.04	HMDB00798 ; HMDB00847 ; HMDB11532 ; HMDB11563 ; HMDB30331 ; HMDB31008 ; HMDB31291 ; HMDB31480 ; HMDB31557 ; HMDB32042 ; HMDB32309 ; HMDB32406 ; HMDB32454 ; HMDB34162 ; HMDB34165 ; HMDB34606 ; HMDB36234 ; HMDB37143 ; HMDB37164 ; HMDB37288 ; HMDB38040 ; HMDB38603 ; HMDB39216 ; HMDB39219 ; HMDB40221 ; HMDB40295 ; HMDB40430 ; HMDB59633 ; HMDB59822	Oenanthic ether ; Pelargonic acid ; MG(0:015:00:0) ; MG(15:00:00:0) ; xi-2-Ethylheptanoic acid ; ()-1516-Dihydroxyoctadecanoic acid ; Methyl caprylate ; Heptyl acetate ; 4-Methyloctanoic acid ; Butyl 3-methylbutanoate ; 3-Heptyl acetate ; 5-Methylhexyl acetate ; 3-Octyl formate ; Pentyl butanoate ; Propyl hexanoate ; Hexyl propionate ; 3-Methylbutyl 2-methylpropanoate ; 1-Hydroxy-3-nonanone ; 3-(Hydroxymethyl)-2-octanone ; 4-Methyl-2-pentyl-13-dioxolane ; 2-Methylpropyl 3-methylbutanoate ; Octyl formate ; Butyl 2-methylbutanoate ; 1-Methylbutyl butanoate ; Isoamyl butyrate ; Butyl pentanoate ; Isopropyl hexanoate ; (9S10S)-910-dihydroxyoctadecanoate ; Ethyl 5-methylhexanoate	M+ACN+H ; M+ACN+H ; M+2ACN+2H ; M+2ACN+2H ; M+ACN+H ; M+2ACN+2H ; M+ACN+H ; M+2ACN+2H ; M+ACN+H	C9H18O2 ; C9H18O2 ; C18H36O4 ; C18H36O4 ; C9H18O2 ; C18H36O4 ; C9H18O2 ; C18H36O4 ; C9H18O2
X81	3	278.031	0	HMDB15343 ; HMDB41706 ; HMDB41708	Ceftriaxone ; Caffeic acid 3-sulfate ; Caffeic acid 4-sulfate	M+2H ; M+NH4 ; M+NH4	C18H18N8O7S3 ; C9H8O7S ; C9H8O7S
X816	3	558.296	0.012	HMDB10357 ; HMDB38029	Tetrahydroaldosterone-3-glucuronide ; Eriojaposide B	M+NH4 ; M+ACN+H	C27H40O11 ; C25H40O11
X818	3	199.135	0.002	HMDB30263	Rhazidigenine Nb-oxide	M+2ACN+2H	C19H26N2O2
X819	3	178.09	0.007	HMDB15548 ; HMDB60933	Valganciclovir ; Propofol glucuronide	M+2H ; M+2H	C14H22N6O5 ; C18H26O7
X82	3	463.608	0	Unknow	Unknow	Unknow	Unknow
X834	3	99.5122	0.073	HMDB00816 ; HMDB60148	Phosphoglycolic acid ; 2-Phosphonatoglycolate	M+ACN+2H ; M+ACN+2H	C2H5O6P ; C2H5O6P
X835	3	122.071	0.083	HMDB03361 ; HMDB34176 ; HMDB35170 ; HMDB39238	Pyrimidine ; Pyrazine ; 12-Dihydro-11-dimethyl-2-oxo-44-bipyridinium(1) ; Cytokinin B	M+ACN+H ; M+ACN+H ; M+ACN+2H ; M+H+NH4	C4H4N2 ; C4H4N2 ; C12H13N2O ; C12H11N5
X84	3	462.012	0	Unknow	Unknow	Unknow	Unknow
X859	3	281.156	0.114	HMDB33295 ; HMDB12230 ; HMDB28692 ; HMDB28944 ; HMDB60442	7-(4-Hydroxyphenyl)-1-phenyl-4-hepten-3-one ; Gamma-glutamyl-L-putrescine ; Alanyl-Lysine ; Lysyl-Alanine ; beta-Alanyl-L-lysine	M+H ; M+ACN+Na ; M+ACN+Na ; M+ACN+Na ; M+ACN+Na	C19H20O2 ; C9H19N3O3 ; C9H19N3O3 ; C9H19N3O3 ; C9H19N3O3
X869	3	100.51	0.02	HMDB32846	5-Chloro-6-methoxy-2(3H)-benzoxazolone	M+2H	C8H6ClNO3

X871	3	208.097	0.248	HMDB00512 ; HMDB00860 ; HMDB02042 ; HMDB60608 ; HMDB00375 ; HMDB00563 ; HMDB00748 ; HMDB00779 ; HMDB02072 ; HMDB02199 ; HMDB02229 ; HMDB05175 ; HMDB29665 ; HMDB29817 ; HMDB30435 ; HMDB30664 ; HMDB31132 ; HMDB32030 ; HMDB32138 ; HMDB32573 ; HMDB32605 ; HMDB32639 ; HMDB32991 ; HMDB33752 ; HMDB34243 ; HMDB34262 ; HMDB34993 ; HMDB39359 ; HMDB39571 ; HMDB40308 ; HMDB40326 ; HMDB40645 ; HMDB40731 ; HMDB40938 ; HMDB41122 ; HMDB41611 ; HMDB41683 ; HMDB59969	N-Acetyl-L-phenylalanine ; Phenylpropionylglycine ; 3-Phenylpropionylglycine ; N-isopropylterephthalamic acid ; 3-(3-Hydroxyphenyl)propanoic acid ; D-Phenyllactic acid ; L-3-Phenyllactic acid ; Phenyllactic acid ; 4-Methoxyphenylacetic acid ; Desaminotyrosine ; 3-Phenoxypropionic acid ; Homovanillin ; Ethyl vanillin ; Ethyl salicylate ; Simulansamide ; 33-Dihydroxy-457-trimethoxyflavan ; 34-Dihydroxyphenylacetone ; Guaicyl acetate ; 34-Dimethoxybenzaldehyde ; Ethylparaben ; Methyl 2-methoxybenzoate ; Methyl 4-methoxybenzoate ; 7-Methoxy-6-methyl-2H-1-benzopyran-2-one ; 3-(2-Hydroxyphenyl)propanoic acid ; Ipomeanine ; Byssochlamic acid ; 4-Methoxybenzyl formate ; Methyl (ZZ)-10-hydroxy-28-decadiene-46-diynoate ; 5-Hydroxy-3-methoxysativan ; 58-Dihydroxy-347-trimethoxyflavan ; 7-Hydroxy-25-dimethyl-4H-1-benzopyran-4-one ; 3-Hydroxy-1-(4-	M+H ; M+H ; M+H ; M+H ; M+ACN+H ; M+ACN+H ; M+ACN+H ; M+ACN+H ; M+ACN+H ; M+ACN+H ; M+ACN+H ; M+ACN+H ; M+ACN+H ; M+ACN+H ; M+H+NH4 ; M+2ACN+2H ; M+ACN+H ; M+ACN+H ; M+ACN+H ; M+ACN+H ; M+ACN+H ; M+ACN+H ; M+NH4 ; M+ACN+H ; M+ACN+H ; M+2ACN+2H ; M+ACN+H ; M+NH4 ; M+2ACN+2H ; M+2ACN+2H ; M+NH4 ; M+ACN+H ; M+NH4 ; M+NH4 ; M+NH4 ; M+ACN+H ; M+ACN+H ; M+ACN+H	C11H13NO3 ; C11H13NO3 ; C11H13NO3 ; C11H13NO3 ; C9H10O3 ; C9H10O3 ; C9H10O3 ; C9H10O3 ; C9H10O3 ; C9H10O3 ; C9H10O3 ; C9H10O3 ; C9H10O3 ; C9H10O3 ; C22H23NO6 ; C18H20O6 ; C9H10O3 ; C9H10O3 ; C9H10O3 ; C9H10O3 ; C9H10O3 ; C9H10O3 ; C11H10O3 ; C9H10O3 ; C9H10O3 ; C18H20O6 ; C9H10O3 ; C11H10O3 ; C18H20O6 ; C18H20O6 ; C11H10O3 ; C9H10O3 ; C11H10O3 ; C11H10O3 ; C11H10O3 ; C9H10O3 ; C9H10O3 ; C9H10O3
X873	3	280.655	0.051	HMDB10320 ; HMDB15499 ; HMDB31935	Cortolone-3-glucuronide ; Almitrine ; Blumenol C O-rhamnosyl-(1-6)-glucoside	M+H+NH4 ; M+2ACN+2H ; M+ACN+2H	C27H42O11 ; C26H29F2N7 ; C25H42O11
X878	3	87.0998	0.025	Unknow	Unknow	Unknow	Unknow
X90	3	334.586	0	HMDB37539	6-Caffeoylhyperin	M+ACN+2H	C30H26O15
X909	3	139.988	0.018	Unknow	Unknow	Unknow	Unknow
X91	3	289.551	0	Unknow	Unknow	Unknow	Unknow
X93	3	299.082	0	Unknow	Unknow	Unknow	Unknow
X95	3	117.066	0	HMDB60496 ; HMDB61163 ; HMDB39111 ; HMDB00123 ; HMDB14691 ; HMDB29384 ; HMDB30276 ; HMDB31239	NN-Diacetylhydrazine ; N-Nitroso-3-hydroxypyrrolidine ; L-Acetopine ; Glycine ; Acetohydroxamic Acid ; 4-Hydroxycitrulline ; (R)-Dihydromaleimide ; Ethyl nitrite	M+H ; M+H ; M+2H ; M+ACN+H ; M+ACN+H ; M+ACN+2H ; M+NH4 ; M+ACN+H	C4H8N2O2 ; C4H8N2O2 ; C8H16N4O4 ; C2H5NO2 ; C2H5NO2 ; C6H13N3O4 ; C4H5NO2 ; C2H5NO2
X97	3	218.042	0	HMDB00152 ; HMDB00397 ; HMDB00840 ; HMDB01229 ; HMDB01321 ; HMDB01856 ; HMDB02016 ; HMDB02404 ; HMDB04067 ; HMDB06116 ; HMDB13676 ; HMDB13677 ; HMDB13678 ; HMDB15366 ; HMDB29666 ; HMDB30726 ; HMDB34299 ; HMDB38055 ; HMDB39119 ; HMDB60602	Gentisic acid ; 2-Pyrocatechuic acid ; Salicyluric acid ; Dopaquinone ; D-Erythrose 4-phosphate ; Protocatechuic acid ; 4-Carboxyphenylglycine ; Alpha-Hydroxyhippuric acid ; Leucodopachrome ; 3-Hydroxyhippuric acid ; 26-Dihydroxybenzoic acid ; 35-Dihydroxybenzoic acid ; 4-Hydroxyhippuric acid ; Sevoflurane ; 24-Dihydroxybenzoic acid ; 2-Hydroxy-67-dimethoxybenzoxazole ; Patulin ; 2-Hydroxy-7-methoxy-2H-14-benzoxazin-3(4H)-one ; L-Dopaquinone ; N-acetyl-5-aminosalicylic acid	M+ACN+Na ; M+ACN+Na ; M+Na ; M+Na ; M+NH4 ; M+ACN+Na ; M+Na ; M+Na ; M+Na ; M+Na ; M+ACN+Na ; M+ACN+Na ; M+Na ; M+NH4 ; M+ACN+Na ; M+Na ; M+ACN+Na ; M+Na ; M+Na ; M+Na	C7H6O4 ; C7H6O4 ; C9H9NO4 ; C9H9NO4 ; C4H9O7P ; C7H6O4 ; C9H9NO4 ; C9H9NO4 ; C9H9NO4 ; C9H9NO4 ; C7H6O4 ; C7H6O4 ; C9H9NO4 ; C4H3F7O ; C7H6O4 ; C9H9NO4 ; C7H6O4 ; C9H9NO4 ; C9H9NO4 ; C9H9NO4
X103	4	265.023	0	HMDB38142 ; HMDB39791 ; HMDB39792 ; HMDB39796	58-Dihydro-6-(4-methyl-3-pentenyl)-1234-tetrathiocin ; 23-Dihydro-5-methyl-3-thiophenethiol ; 45-Dihydro-2-methyl-3-thiophenethiol ; 45-Dihydro-5-methyl-3-thiophenethiol	M+H ; 2M+H ; 2M+H ; 2M+H	C10H16S4 ; C5H8S2 ; C5H8S2 ; C5H8S2
X106	4	308.963	0	Unknow	Unknow	Unknow	Unknow
X107	4	312.116	0	HMDB05765 ; HMDB35204	Ophthalmic acid ; Dukunolide C	M+Na ; M+2ACN+2H	C11H19N3O6 ; C28H28O11
X109	4	493.006	0	HMDB29274	Sanguisorbic acid dilactone	M+Na	C21H10O13
X11	4	396.055	0	HMDB14667	Oxaliplatin	M+H	C8H12N2O4Pt
X112	4	133.015	0	Unknow	Unknow	Unknow	Unknow

X126	4	284.103	0	HMDB00472 ; HMDB13139 ; HMDB14774 ; HMDB15571 ; HMDB28693 ; HMDB28788 ; HMDB28852 ; HMDB28966 ; HMDB29083 ; HMDB29124 ; HMDB30584 ; HMDB39409 ; HMDB59600 ; HMDB60566 ; HMDB61174	5-Hydroxy-L-tryptophan ; 1-Hydroxypyrene ; Clofibrate ; Oxitriptan ; Alanyl-Methionine ; Cysteinyl-Valine ; Glycyl-Tryptophan ; Methionyl-Alanine ; Tryptophyl-Glycine ; Valyl-Cysteine ; Silidianin ; N-3-(b-D-Glucopyranosyloxy)-23-dihydro-2-oxo-1H-indol-3-ylacetylaspartic acid ; 5-phosphonooxy-L-lysine ; Carbamazepine-O-quinone ; p-Hydroxyl-ethotoin	M+ACN+Na ; M+ACN+Na ; M+ACN+H ; M+ACN+Na ; M+ACN+Na ; M+ACN+Na ; M+Na ; M+ACN+Na ; M+Na ; M+ACN+Na ; M+2ACN+2H ; M+2ACN+2H ; M+ACN+H ; M+NH4 ; M+ACN+Na	C11H12N2O3 ; C16H12O ; C12H15ClO3 ; C11H12N2O3 ; C8H16N2O3S ; C8H16N2O3S ; C13H15N3O3 ; C8H16N2O3S ; C13H15N3O3 ; C8H16N2O3S ; C25H24O10 ; C20H24N2O12 ; C6H15N2O6P ; C15H10N2O3 ; C11H12N2O3
X128	4	351.968	0	Unknow	Unknow	Unknow	Unknow
X148	4	200.546	0	HMDB15565 ; HMDB37200 ; HMDB37203	Etoricoxib ; 2-Galloyl-14-galactarolactone methyl ester ; 5-Methoxy-galloyl-14-galactarolactone	M+ACN+2H ; M+ACN+2H ; M+ACN+2H	C18H15ClN2O2S ; C14H14O11 ; C14H14O11
X150	4	449.945	0	Unknow	Unknow	Unknow	Unknow
X154	4	200.044	0	HMDB37269	Desacetycefapirin	M+H+NH4	C15H15N3O5S2
X166	4	366.001	0	Unknow	Unknow	Unknow	Unknow
X168	4	290.076	0	HMDB13690 ; HMDB29298 ; HMDB29754 ; HMDB32900 ; HMDB33973 ; HMDB33974 ; HMDB37347 ; HMDB37471 ; HMDB37968 ; HMDB38366 ; HMDB38367 ; HMDB38368 ; HMDB38369 ; HMDB38741 ; HMDB41239 ; HMDB01904 ; HMDB29678 ; HMDB32998 ; HMDB33925	Procyanidin ; 4-O-85-5-Dehydrotriferulic acid ; Procyanidin B1 ; Epicatechin-(6-8)-epicatechin ; Procyanidin B2 ; Procyanidin B3 ; Terniflorin ; 2-O-p-Coumaroylvitexin ; ent-Epicatechin(4alpha-8)catechin ; Procyanidin B5 ; Procyanidin B6 ; Procyanidin B7 ; Procyanidin B8 ; Genistein 7-O-(2-p-coumaroylglucoside) ; ent-Epicatechin-(4alpha-6)-ent-epicatechin ; 3-Nitrotyrosine ; Hibiscitrin ; 2-Methoxyxanthone ; Oroselone	M+2H ; M+2H ; M+2H ; M+2H ; M+2H ; M+2H ; M+2H ; M+2H ; M+2H ; M+2H ; M+2H ; M+2H ; M+2H ; M+2H ; M+2H ; M+ACN+Na ; M+2ACN+2H ; M+ACN+Na ; M+ACN+Na	C30H26O12 ; C30H26O12 ; C30H26O12 ; C30H26O12 ; C30H26O12 ; C30H26O12 ; C30H26O12 ; C30H26O12 ; C30H26O12 ; C30H26O12 ; C30H26O12 ; C30H26O12 ; C30H26O12 ; C30H26O12 ; C30H26O12 ; C9H10N2O5 ; C21H20O14 ; C14H10O3 ; C14H10O3
X169	4	198.018	0	Unknow	Unknow	Unknow	Unknow
X179	4	525.144	0	HMDB14837 ; HMDB29355 ; HMDB31762 ; HMDB38004 ; HMDB39102 ; HMDB59747	Nicergoline ; N-Phenylacetylaspartic acid ; Carbadox ; Delphinidin 3-(acetylglucoside) ; N-Carboxyacetyl-D-phenylalanine ; 4-Hydroxy-3-methoxy-cinnamoylglycine	M+ACN+H ; 2M+Na ; 2M+H ; M+NH4 ; 2M+Na ; 2M+Na	C24H26BrN3O3 ; C12H13NO5 ; C11H10N4O4 ; C23H23O13 ; C12H13NO5 ; C12H13NO5
X190	4	383.039	0	Unknow	Unknow	Unknow	Unknow
X20	4	178.044	0	HMDB01919	Famotidine	M+H+NH4	C8H15N7O2S3
X207	4	680.499	0	Unknow	Unknow	Unknow	Unknow
X212	4	324.022	0	Unknow	Unknow	Unknow	Unknow
X224	4	397.934	0	Unknow	Unknow	Unknow	Unknow
X226	4	260.044	0	HMDB00592 ; HMDB01481	Glucosamine 6-sulfate ; N-Sulfo-D-glucosamine	M+H ; M+H	C6H13NO8S ; C6H13NO8S
X227	4	327.059	0	HMDB00292 ; HMDB00786 ; HMDB01182 ; HMDB01876 ; HMDB02194 ; HMDB29614 ; HMDB60726 ; HMDB61102	Xanthine ; Oxypurinol ; 68-Dihydroxypurine ; Epinephrine sulfate ; N-acetyl-S-(3-oxo-3-carboxy-n-propyl)cysteine ; ()-Conen ; 28-bis-Trifluoromethyl-4-quinoline carboxylic acid ; 3-oxobrimonidine	2M+Na ; 2M+Na ; 2M+Na ; M+ACN+Na ; M+ACN+Na ; M+Na ; M+NH4 ; M+NH4	C5H4N4O2 ; C5H4N4O2 ; C5H4N4O2 ; C9H13NO6S ; C9H13NO6S ; C13H21O2PS2 ; C12H5F6NO2 ; C11H12BrN5O
X23	4	261.89	0	Unknow	Unknow	Unknow	Unknow
X234	4	360.037	0	Unknow	Unknow	Unknow	Unknow
X243	4	151.541	0	Unknow	Unknow	Unknow	Unknow
X249	4	359.547	0	Unknow	Unknow	Unknow	Unknow
X251	4	249.033	0	HMDB37204	35-Di-O-galloyl-14-galactarolactone	M+2H	C20H16O15
X263	4	265.569	0	Unknow	Unknow	Unknow	Unknow
X264	4	202.041	0	HMDB14332 ; HMDB31332 ; HMDB33075 ; HMDB60417 ; HMDB60601	Lorazepam ; Chloromethyl methyl ether ; Methyl 1-(methylsulfinyl)propyl disulfide ; 6-Thiourate ; 6-thiouric acid	M+2ACN+2H ; 2M+ACN+H ; M+NH4 ; M+NH4 ; M+NH4	C15H10Cl2N2O2 ; C2H5ClO ; C5H12OS3 ; C5H4N4O2S ; C5H4N4O2S

X265	4	215.114	0	HMDB00026 ; HMDB00168 ; HMDB01387 ; HMDB01942 ; HMDB03633 ; HMDB04225 ; HMDB11733 ; HMDB12265 ; HMDB15189 ; HMDB15227 ; HMDB32055 ; HMDB33222 ; HMDB33223 ; HMDB33780 ; HMDB35822 ; HMDB38149 ; HMDB39658 ; HMDB39659 ; HMDB39663 ; HMDB39674 ; HMDB39835 ; HMDB40037 ; HMDB40039 ; HMDB40044 ; HMDB60283 ; HMDB60284 ; HMDB60765 ; HMDB61172	Ureidopropionic acid ; L-Asparagine ; N-Methylphenylethanolamine ; Phenylpropanolamine ; N-Methyltyramine ; 2-Oxoarginine ; Glycyl-glycine ; N-Carbamoylsarcosine ; Tocainide ; Fluvastatin ; N-Acetylhistidine ; (1R3S4S6R)-69-Dihydroxyfenchone 6-O-b-D-glucoside ; (1S3R4R)-810-Dihydroxyfenchone 10-O-b-D-glucoside ; D-Asparagine ; Glucosyl 6-hydroxy-26-dimethyl-2E7-octadienoate ; Nepetaside ; 123456-Hexahydro-5-methyl-7H-cyclopentabpyridin-7-one ; 123456-Hexahydro-6-methyl-7H-cyclopentabpyridin-7-one ; 234567-Hexahydrocyclopentbazepin-8(1H)-one ; 2-(1-Pyrrolidinyl)-2-cyclopenten-1-one ; 2-(2-Furanyl)piperidine ; 1-Furfurylpyrrolidine ; 2-Acetyl-3-ethylidene-3456-tetrahydropyridine ; 2-(5-Methyl-2-furanyl)pyrrolidine ; N-Methyltyraminium ; Tyraminium ; 4-Hydroxyamphetamine ; O-Desmethylvenlafaxine glucuronide	M+2ACN+H ; M+2ACN+H ; M+ACN+Na ; M+ACN+Na ; M+ACN+Na ; M+ACN+H ; M+2ACN+H ; M+2ACN+H ; M+Na ; M+H+NH4 ; M+NH4 ; M+2ACN+2H ; M+2ACN+2H ; M+2ACN+H ; M+2ACN+2H ; M+2ACN+2H ; M+ACN+Na ; M+ACN+Na ; M+ACN+Na ; M+ACN+Na ; M+ACN+Na ; M+ACN+Na ; M+ACN+Na ; M+ACN+Na ; M+ACN+Na ; M+ACN+Na ; M+ACN+Na ; M+H+NH4	C4H8N2O3 ; C4H8N2O3 ; C9H13NO ; C9H13NO ; C9H13NO ; C6H11N3O3 ; C4H8N2O3 ; C4H8N2O3 ; C11H16N2O ; C24H26FNO4 ; C8H11N3O3 ; C16H26O8 ; C16H26O8 ; C4H8N2O3 ; C16H26O8 ; C16H26O8 ; C9H13NO ; C9H13NO ; C9H13NO ; C9H13NO ; C9H13NO ; C9H13NO ; C9H13NO ; C9H13NO ; C9H13NO ; C9H13NO ; C9H13NO ; C20H29NO8
X268	4	141.528	0	Unknow	Unknow	Unknow	Unknow
X273	4	161	0	Unknow	Unknow	Unknow	Unknow
X29	4	332.589	0	Unknow	Unknow	Unknow	Unknow
X291	4	280.069	0	HMDB14513 ; HMDB12208 ; HMDB14836	Cidofovir ; D-Erythro-imidazole-glycerol-phosphate ; Nitrofurantoin	M+H ; M+ACN+H ; M+ACN+H	C8H14N3O6P ; C6H11N2O6P ; C8H6N4O5
X300	4	221.01	0	Unknow	Unknow	Unknow	Unknow
X304	4	219.018	0	HMDB14394	Idoxuridine	M+2ACN+2H	C9H11IN2O5
X309	4	140.533	0	HMDB41060	Dehydro-4-methoxycyclobrassinin	M+H+NH4	C12H10N2OS2
X318	4	558.127	0	HMDB00652 ; HMDB38866	Chondroitin 4-sulfate ; Quercetagetin 3-methylether 7-glucoside	M+2ACN+H ; M+ACN+Na	C14H21NO15S ; C22H22O13
X329	4	182.981	0	Unknow	Unknow	Unknow	Unknow
X335	4	238.019	0	Unknow	Unknow	Unknow	Unknow
X339	4	316.042	0	Unknow	Unknow	Unknow	Unknow
X340	4	718.139	0	HMDB06032 ; HMDB41813	Imidazoleacetic acid ribotide ; 6-Demethylgriseofulvin	2M+ACN+H ; 2M+ACN+H	C10H15N2O9P ; C16H15ClO6
X342	4	235.558	0	HMDB38789	Torvanol A	M+H+NH4	C20H20O10S
X343	4	368.956	0	Unknow	Unknow	Unknow	Unknow
X344	4	557.122	0	HMDB14408 ; HMDB15688	Sulfisoxazole ; Sulfamoxole	2M+Na ; 2M+Na	C11H13N3O3S ; C11H13N3O3S
X358	4	346.53	0	Unknow	Unknow	Unknow	Unknow
X367	4	289.087	0	HMDB34449 ; HMDB40716	Hydroxyanigorufone ; Irenolone	M+H ; M+H	C19H12O3 ; C19H12O3
X369	4	151.039	0	HMDB01587 ; HMDB31515 ; HMDB32598 ; HMDB32612 ; HMDB40528 ; HMDB29492 ; HMDB29676 ; HMDB30617 ; HMDB30659 ; HMDB30667 ; HMDB32695 ; HMDB32696 ; HMDB33321 ; HMDB33720 ; HMDB33760 ; HMDB34443 ; HMDB37339 ; HMDB37441 ; HMDB42024	Phenylglyoxylic acid ; (E)-8-Hydroxy-2-octene-46-diynoic acid ; 4-Hydroxyphthalide ; 34-Methylenedioxybenzaldehyde ; 14-Benzodioxin-2(3H)-one ; 337-Trihydroxy-4-methoxyflavone ; Diosmetin ; Pratensein ; Takakin ; Chrysoeriol ; Barpisoflavone A ; Cajanin ; 35-Dihydroxy-67-methylenedioxyflavanone ; 6alpha-Hydroxymaackiain ; Santal ; Questinol ; Luteolin 7-methyl ether ; Kaempferide ; Tectorigenin	M+H ; M+H ; M+H ; M+H ; M+H ; M+2H ; M+2H ; M+2H ; M+2H ; M+2H ; M+2H ; M+2H ; M+2H ; M+2H ; M+2H ; M+2H ; M+2H ; M+2H ; M+2H ; M+2H	C8H6O3 ; C8H6O3 ; C8H6O3 ; C8H6O3 ; C8H6O3 ; C16H12O6 ; C16H12O6 ; C16H12O6 ; C16H12O6 ; C16H12O6 ; C16H12O6 ; C16H12O6 ; C16H12O6 ; C16H12O6 ; C16H12O6 ; C16H12O6 ; C16H12O6 ; C16H12O6 ; C16H12O6
X395	4	329.095	0	HMDB14342 ; HMDB29235	Fluconazole ; Mulberrofuran P	M+Na ; M+2ACN+2H	C13H12F2N6O ; C34H22O9

X417	4	377.123	0	HMDB30189 ; HMDB30384 ; HMDB30934 ; HMDB36989 ; HMDB37469 ; HMDB38633 ; HMDB40797 ; HMDB40898	Isogravacridonechlorine ; Gravacridonechlorine ; Triglochinin ; Romucosine B ; Spinacetin 3-gentiobioside ; (-)-3-Cyanomethyl-3-hydroxy-1H-indol-2(3H)-one ; Methyl helianthenoate F glucoside ; Methyl (R)-8-Hydroxy-9-decene-46-diynoate glucoside	M+NH4 ; M+NH4 ; M+NH4 ; M+NH4 ; M+2ACN+2H ; 2M+H ; M+Na ; M+Na	C19H18ClNO4 ; C19H18ClNO4 ; C14H17NO10 ; C19H18ClNO4 ; C29H34O18 ; C10H8N2O2 ; C17H22O8 ; C17H22O8
X42	4	579.076	0	Unknow	Unknow	Unknow	Unknow
X425	4	279.566	0	Unknow	Unknow	Unknow	Unknow
X432	4	427.509	0	Unknow	Unknow	Unknow	Unknow
X435	4	606.179	0	HMDB29260 ; HMDB30803 ; HMDB30843 ; HMDB33571 ; HMDB37569 ; HMDB37575 ; HMDB39319 ; HMDB39321	Apigenin 6-C-glucoside 8-C-arabinoside ; Corymboside ; Apiin ; 6-beta-D-Glucopyranosyl-8-beta-D-ribopyranosylapigenin ; Isovitexin 2-O-arabinoside ; Kaempferol 3-arabinofuranoside 7-rhamnofuranoside ; Kaempferol 3-rhamnoside 7-xyloside ; Kaempferol 3-rhamnoside 4-xyloside	M+ACN+H ; M+ACN+H ; M+ACN+H ; M+ACN+H ; M+ACN+H ; M+ACN+H ; M+ACN+H ; M+ACN+H	C26H28O14 ; C26H28O14 ; C26H28O14 ; C26H28O14 ; C26H28O14 ; C26H28O14 ; C26H28O14 ; C26H28O14
X44	4	241.03	0	HMDB00192 ; HMDB00965	L-Cystine ; Hypotaurine	M+H ; 2M+Na	C6H12N2O4S2 ; C2H7NO2S
X446	4	263.075	0	HMDB30376	(S)-Isowillardiine	M+ACN+Na	C7H9N3O4
X467	4	370.532	0	Unknow	Unknow	Unknow	Unknow
X47	4	653.108	0	HMDB37785 ; HMDB39168 ; HMDB39170 ; HMDB39193	2-C-Methylmyricetin 3-rhamnoside 5-gallate ; 2-O-p-Coumaroyl-16-digalloyl-beta-D-glucopyranoside ; 6-O-p-Coumaroyl-12-digalloylglucose ; 2-O-(4-Hydroxycinnamoyl)-16-di-O-galloyl-beta-D-glucopyranose	M+Na ; M+Na ; M+Na ; M+Na	C29H26O16 ; C29H26O16 ; C29H26O16 ; C29H26O16
X485	4	288.571	0	HMDB37434 ; HMDB37758 ; HMDB38266 ; HMDB39247	6-Malonylastragalin ; 45-Dihydroxy-33-dimethoxy-67-methylenedioxyflavone 4-glucuronide ; 35-Dihydroxy-34-dimethoxy-67-methylenedioxyflavone 3-glucuronide ; 2-Hydroxygenistein 7-(6-malonylglucoside)	M+ACN+2H ; M+ACN+2H ; M+ACN+2H ; M+ACN+2H	C24H22O14 ; C24H22O14 ; C24H22O14 ; C24H22O14
X491	4	328.098	0	HMDB37470 ; HMDB37579 ; HMDB38235 ; HMDB60764	Spinacetin 3-rutinoside ; Limocitrin 3-rutinoside ; Rhamnazin 3-sophoroside ; 4-Hydroxy-estazolam	M+2H ; M+2H ; M+2H ; M+NH4	C29H34O17 ; C29H34O17 ; C29H34O17 ; C16H11ClN4O
X503	4	228.391	0	Unknow	Unknow	Unknow	Unknow
X507	4	231.025	0	HMDB00618 ; HMDB00868 ; HMDB01489 ; HMDB01548 ; HMDB06534 ; HMDB11734 ; HMDB12195 ; HMDB60257	D-Ribulose 5-phosphate ; Xylulose 5-phosphate ; Ribose 1-phosphate ; D-Ribose 5-phosphate ; D-Xylulose 1-phosphate ; D-Arabinose 5-phosphate ; Beta-L-arabinose 1-phosphate ; Selenomethionine se-oxide	M+H ; M+H ; M+H ; M+H ; M+H ; M+H ; M+H ; M+NH4	C5H11O8P ; C5H11O8P ; C5H11O8P ; C5H11O8P ; C5H11O8P ; C5H11O8P ; C5H11O8P ; C5H11NO3Se

X508	4	256.097	0.676	HMDB14608 ; HMDB29322 ; HMDB30215 ; HMDB30320 ; HMDB38639 ; HMDB38640 ; HMDB41996 ; HMDB02399 ; HMDB30580 ; HMDB30752 ; HMDB31610 ; HMDB31623 ; HMDB31816 ; HMDB32018 ; HMDB32583 ; HMDB32591 ; HMDB35275 ; HMDB35873 ; HMDB35894 ; HMDB37277 ; HMDB37877 ; HMDB40268 ; HMDB40446 ; HMDB41007 ; HMDB41481 ; HMDB41807	Ketorolac ; 1-Hydroxy-3-methoxy-10-methylacridone ; Clausine L ; Mukonine ; Methyl 6-methoxy-9H-carbazole-3-carboxylate ; 16-Dimethoxy-9H-carbazole-3-carboxaldehyde ; Pranoprofen ; Cystathionine sulfoxide ; (R)-Shinanolone ; Methyl trans-p-methoxycinnamate ; Allyl phenoxyacetate ; Ethyl phenylglycidate ; 3-Hydroxyflavone ; Phenyl salicylate ; 4-Hydroxy-2-biphenylcarboxylic acid ; Dehydrozingerone ; Isomyristicin ; Myristicin ; (Z)-4-Methoxy-6-(1-propenyl)-13-benzodioxole ; Isoeugenol formate ; Eugenyl formate ; Ethyl 3-oxo-3-phenylpropanoate ; Benzyl acetoacetate ; 1-Hydroxychavicol acetate ; 4-(34-Methylenedioxyphenyl)-2-butanone ; 3-Phenoxybenzoic acid	M+H ; M+H ; M+H ; M+H ; M+H ; M+H ; M+H ; M+NH4 ; M+ACN+Na ; M+ACN+Na ; M+ACN+Na ; M+ACN+Na ; M+NH4 ; M+ACN+H ; M+ACN+H ; M+ACN+Na ; M+ACN+Na ; M+ACN+Na ; M+ACN+Na ; M+ACN+Na ; M+ACN+Na ; M+ACN+Na ; M+ACN+Na ; M+ACN+Na ; M+ACN+Na ; M+ACN+H	C15H13NO3 ; C15H13NO3 ; C15H13NO3 ; C15H13NO3 ; C15H13NO3 ; C15H13NO3 ; C15H13NO3 ; C7H14N2O5S ; C11H12O3 ; C11H12O3 ; C11H12O3 ; C11H12O3 ; C15H10O3 ; C13H10O3 ; C13H10O3 ; C11H12O3 ; C11H12O3 ; C11H12O3 ; C11H12O3 ; C11H12O3 ; C11H12O3 ; C11H12O3 ; C11H12O3 ; C11H12O3 ; C11H12O3 ; C13H10O3
X513	4	253.081	0.074	HMDB04086 ; HMDB35993 ; HMDB35486 ; HMDB01923 ; HMDB02335 ; HMDB13689 ; HMDB28761 ; HMDB29013 ; HMDB29756 ; HMDB31328 ; HMDB31701 ; HMDB32345 ; HMDB33184 ; HMDB34130 ; HMDB34212 ; HMDB35370 ; HMDB39097 ; HMDB39110 ; HMDB40331 ; HMDB40590 ; HMDB41751 ; HMDB41752	5-Hydroxy-N-formylkynurenine ; N5-(34-Dioxo-15-cyclohexadien-1-yl)-L-glutamine ; 6-Caffeoylsucrose ; Naproxen ; Aspartyl-L-proline ; Peonidin-3-glucoside ; Aspartyl-Proline ; Prolyl-Aspartate ; (1R2S3R)-2-Acetyl-4(5)-(1234-tetrahydroxybutyl)imidazole ; 1-Isothiocyanatobutane ; 1-(gamma-Glutamylamino)cyclopropanecarboxylic acid ; Isobutyl isothiocyanate ; Pondaplin ; Osthenol ; (R)-Kawain ; Pyranocyanin B ; Demethylbatatasin IV ; (2S3S)-alpha-Amino-2-carboxy-5-oxo-1-pyrrolidinebutanoic acid ; xi-25-Dihydro-24-dimethylthiazole ; Benzyl methyl disulfide ; Isopeonidin 3-galactoside ; Isopeonidin 3-glucoside	M+H ; M+H ; M+2H ; M+Na ; M+Na ; M+ACN+2H ; M+Na ; M+Na ; M+Na ; 2M+Na ; M+Na ; 2M+Na ; M+Na ; M+Na ; M+Na ; M+H+NH4 ; M+Na ; M+Na ; 2M+Na ; M+2ACN+H ; M+ACN+2H ; M+ACN+2H	C11H12N2O5 ; C11H12N2O5 ; C21H28O14 ; C14H14O3 ; C9H14N2O5 ; C22H23O11 ; C9H14N2O5 ; C9H14N2O5 ; C9H14N2O5 ; C5H9NS ; C9H14N2O5 ; C5H9NS ; C14H14O3 ; C14H14O3 ; C14H14O3 ; C24H23O11 ; C14H14O3 ; C9H14N2O5 ; C5H9NS ; C8H10S2 ; C22H23O11 ; C22H23O11
X515	4	270.891	0.322	HMDB12259	Methylarsonite	2M+Na	CH5AsO2
X519	4	399.074	0.229	HMDB14933	Sulfasalazine	M+H	C18H14N4O5S
X522	4	199.924	0.572	Unknow	Unknow	Unknow	Unknow
X524	4	295.829	0	Unknow	Unknow	Unknow	Unknow
X528	4	254.074	0.037	HMDB37997 ; HMDB39567	Delphinidin 3-glucoside ; BL V	M+ACN+2H ; M+2ACN+2H	C21H21O12 ; C22H16O9
X535	4	206.046	0.196	HMDB00881 ; HMDB32963 ; HMDB33528 ; HMDB60413	Xanthurenic acid ; Zeanic acid ; 46-Dihydroxy-2-quinolinecarboxylic acid ; 6-Methylthioguanosine monophosphate	M+H ; M+H ; M+H ; M+H+NH4	C10H7NO4 ; C10H7NO4 ; C10H7NO4 ; C11H16N5O7PS
X538	4	195.931	0.342	Unknow	Unknow	Unknow	Unknow
X539	4	215.936	0.327	Unknow	Unknow	Unknow	Unknow
X544	4	415.049	0	HMDB14952	Meloxicam	M+ACN+Na	C14H13N3O4S2
X546	4	210.091	0.004	HMDB32757 ; HMDB33000 ; HMDB60652 ; HMDB28684 ; HMDB28768 ; HMDB32867 ; HMDB33927 ; HMDB37621 ; HMDB39465 ; HMDB39468 ; HMDB40186	3-Methyl-9H-carbazole-9-carboxaldehyde ; 10-Methylacridone ; 2-Hydroxyiminostilbene ; Alanyl-Cysteine ; Cysteinyl-Alanine ; Capillin ; 3-Hydroxymugineic acid ; 4-Methyl-4-(methylthio)-2-pentanone ; S-Methyl hexanethioate ; S-Methyl 4-methylpentanethioate ; 3-(Methylthio)hexanal	M+H ; M+H ; M+H ; M+NH4 ; M+NH4 ; M+ACN+H ; M+2ACN+2H ; M+ACN+Na ; M+ACN+Na ; M+ACN+Na ; M+ACN+Na	C14H11NO ; C14H11NO ; C14H11NO ; C6H12N2O3S ; C6H12N2O3S ; C12H8O ; C12H20N2O9 ; C7H14OS ; C7H14OS ; C7H14OS ; C7H14OS
X549	4	293.907	0.493	Unknow	Unknow	Unknow	Unknow
X557	4	266.115	0.686	HMDB32834 ; HMDB15636 ; HMDB35134	7-Acetoxy-6-hydroxylimonin ; Agomelatine ; Armillaridin	M+2H ; M+Na ; M+2ACN+2H	C28H34O10 ; C15H17NO2 ; C24H29ClO6

X560	4	268.052	0	HMDB31793 ; HMDB32026 ; HMDB32884 ; HMDB34395	Heptenophos ; Sinalexin ; Allura red AC ; 2-Propenyl 3-(2-propenylsulfonyl)-1-propenyl disulfide	M+NH4 ; M+ACN+Na ; M+2ACN+2H ; M+NH4	C9H12ClO4P ; C10H8N2OS ; C18H16N2O8S2 ; C9H14O2S3
X563	4	355.11	0.004	HMDB34854 ; HMDB61000 ; HMDB11658 ; HMDB13189 ; HMDB28830 ; HMDB29082 ; HMDB29496 ; HMDB37356 ; HMDB37363 ; HMDB37537 ; HMDB38287 ; HMDB38824 ; HMDB39002 ; HMDB39878 ; HMDB39924 ; HMDB40371 ; HMDB40861 ; HMDB41358	Flumioxazin ; 4-(4-Chlorophenyl)-1-4-(4-fluorophenyl)-4-oxobutyl-pyridinium (HPP) ; 28-Dihydroxyquinoline-beta-D-glucuronide ; 3-Indole carboxylic acid glucuronide ; Glutamyl-Tryptophan ; Tryptophyl-Glutamate ; Fukiic acid ; Quercetin 47-diglucoside ; Quercetin 34-diglucoside ; Quercetin 3-glucosyl-(1-2)-galactoside ; Myricetin 3-neohesperidoside ; Hypoletin 8-gentiobioside ; Mytilin A ; Vicinin 2 ; 6-Hydroxykaempferol 67-diglucoside ; Herbacetin 38-diglucoside ; Myricetin 3-robinobioside ; Quercetin 3-beta-laminaribioside	M+H ; M+H ; M+NH4 ; M+NH4 ; M+Na ; M+Na ; M+2ACN+H ; M+2ACN+2H ; M+2ACN+2H ; M+2ACN+2H ; M+2ACN+2H ; M+2ACN+2H ; M+Na ; M+2ACN+2H ; M+2ACN+2H ; M+2ACN+2H ; M+2ACN+2H ; M+2ACN+2H	C19H15FN2O4 ; C21H18ClFNO ; C15H15NO8 ; C15H15NO8 ; C16H18N3O5 ; C16H18N3O5 ; C11H12O8 ; C27H30O17 ; C27H30O17 ; C27H30O17 ; C27H30O17 ; C27H30O17 ; C13H20N2O8 ; C27H30O17 ; C27H30O17 ; C27H30O17 ; C27H30O17 ; C27H30O17
X565	4	127.972	0.004	Unknow	Unknow	Unknow	Unknow
X566	4	295.903	0.214	Unknow	Unknow	Unknow	Unknow
X571	4	336.963	0.264	Unknow	Unknow	Unknow	Unknow
X577	4	252.078	0.038	HMDB03045 ; HMDB41887	Ergothioneine ; Endalin	M+Na ; M+Na	C9H15N3O2S ; C9H12FN3O3
X585	4	208.14	0.519	HMDB39883 ; HMDB02284 ; HMDB04224 ; HMDB12135 ; HMDB34326	Ascorbyl palmitate ; N-Acetylcadaverine ; N-(o)-Hydroxyarginine ; 1-(3-Aminopropyl)-4-aminobutanal ; L-erythro-4-Hydroxyarginine	M+2H ; M+ACN+Na ; M+NH4 ; M+ACN+Na ; M+NH4	C22H38O7 ; C7H16N2O ; C6H14N4O3 ; C7H16N2O ; C6H14N4O3
X587	4	127.033	0	HMDB33152 ; HMDB36186 ; HMDB29713	2-(Methylthio)pyrazine ; Pyrazinemethanethiol ; Thiazole	M+H ; M+H ; M+ACN+H	C5H6N2S ; C5H6N2S ; C3H3NS
X593	4	148.517	0.433	Unknow	Unknow	Unknow	Unknow
X596	4	198.926	0.057	Unknow	Unknow	Unknow	Unknow
X601	4	104.009	0	Unknow	Unknow	Unknow	Unknow
X605	4	139.512	0.465	Unknow	Unknow	Unknow	Unknow
X609	4	213.941	0.297	Unknow	Unknow	Unknow	Unknow
X611	4	286.07	0.054	HMDB14387 ; HMDB01265 ; HMDB02326 ; HMDB29218 ; HMDB29463 ; HMDB38634	Cladribine ; Fucose 1-phosphate ; Coumesterol ; Urolithin C ; Gentisein ; (-)-Dioxibrassinin	M+H ; M+ACN+H ; M+NH4 ; M+ACN+H ; M+ACN+H ; M+NH4	C10H12ClN5O3 ; C6H13O8P ; C15H8O5 ; C13H8O5 ; C13H8O5 ; C11H12N2O2S2
X613	4	320.102	0.004	HMDB14848 ; HMDB37462 ; HMDB37467 ; HMDB11669 ; HMDB33488 ; HMDB33497 ; HMDB34100 ; HMDB41200	Ibandronate ; Tricin 7-neohesperidoside ; Rhamnazin 3-rutinoside ; 6-(N-Acetyl-alpha-D-glucosaminyl)-1-phosphatidyl-1D-myo-inositol ; Aurasperone D ; 6-O-Demethylnigerone ; Dianhydroaurasperone C ; Dehydroxymethylflazine	M+H ; M+2H ; M+2H ; M+H+NH4 ; M+2ACN+2H ; M+2ACN+2H ; M+2ACN+2H ; M+ACN+H	C9H23NO7P2 ; C29H34O16 ; C29H34O16 ; C21H36NO18P ; C31H24O10 ; C31H24O10 ; C31H24O10 ; C16H10N2O3
X614	4	186.106	0.002	HMDB32841 ; HMDB32842 ; HMDB36822 ; HMDB31682 ; HMDB34913 ; HMDB37554	5-Megastigmen-7-yne-39-diol 9-glucoside ; 5-Megastigmen-7-yne-39-diol 3-glucoside ; (3S7E9S)-9-Hydroxy-47-megastigmadien-3-one 9-glucoside ; ()-2-Methylthiazolidine ; Imazamethabenz-methyl ; ()-Rollipyrrole	M+2H ; M+2H ; M+2H ; M+2ACN+H ; M+2ACN+2H ; M+2ACN+2H	C19H30O7 ; C19H30O7 ; C19H30O7 ; C4H9NS ; C16H20N2O3 ; C16H20N2O3
X619	4	562.327	0.118	HMDB28736 ; HMDB28946 ; HMDB38569	Asparaginyl-Lysine ; Lysyl-Asparagine ; PC-M5	2M+ACN+H ; 2M+ACN+H ; M+2ACN+H	C10H20N4O4 ; C10H20N4O4 ; C29H37NO5
X626	4	627.122	0.48	HMDB34258 ; HMDB60017	Amritoside ; Pyrogallol-2-O-glucuronide	M+H ; 2M+Na	C26H26O18 ; C12H14O9
X627	4	232.092	0	HMDB33001 ; HMDB33969 ; HMDB34273 ; HMDB34965 ; HMDB39817	2-Methylbenzothiazole ; Benzyl isothiocyanate ; Benzyl thiocyanate ; 1-Methyl-3-(2-thiazolyl)-1H-indole ; Dihydro-46-dimethyl-4H-135-dithiazine	M+2ACN+H ; M+2ACN+H ; M+2ACN+H ; M+NH4 ; M+2ACN+H	C8H7NS ; C8H7NS ; C8H7NS ; C12H10N2S ; C5H11NS2

X628	4	160.025	0.579	HMDB31576 ; HMDB41083 ; HMDB02755 ; HMDB37357 ; HMDB31522	(E)-4-Isothiocyanato-1-(methylthio)-1-butene ; (E)-Raphanusanin ; Myricetin ; 334558-Hexahydroxyflavone ; Mesoxalic acid	M+H ; M+H ; M+2H ; M+2H ; M+ACN+H	C6H9NS2 ; C6H9NS2 ; C15H10O8 ; C15H10O8 ; C3H2O5
X630	4	230.096	0.002	HMDB29909 ; HMDB37108 ; HMDB37603 ; HMDB39852 ; HMDB34294 ; HMDB38183 ; HMDB39104 ; HMDB59611	Gein ; 3-Hydroxychavicol 1-rhamnosyl-(1-6)-glucoside ; Eugenol O-a-L-Arabinofuranosyl-(1-6)-b-D-glucopyranoside ; Deoxynivalenol 3-glucoside ; 1311-Tridecatriene-579-triyne ; 3-(4-Methyl-3-pentenyl)thiophene ; (3E5Z)-135-Tridecatriene-7911-triyne ; Thiomorpholine 3-carboxylate	M+2H ; M+2H ; M+2H ; M+2H ; M+ACN+Na ; M+ACN+Na ; M+ACN+Na ; M+2ACN+H	C21H30O11 ; C21H30O11 ; C21H30O11 ; C21H30O11 ; C13H10 ; C10H14S ; C13H10 ; C5H9NO2S
X632	4	236.957	0.449	Unknow	Unknow	Unknow	Unknow
X647	4	197.926	0.074	HMDB39853	356-Trichloro-2-pyridinol	M+H	C5H2Cl3NO
X648	4	161.023	0.479	HMDB60386 ; HMDB60395 ; HMDB29465	4-Fluoromuconolactone ; 5-Fluoromuconolactone ; Erosnin	M+H ; M+H ; M+2H	C6H5FO4 ; C6H5FO4 ; C18H8O6
X653	4	171.011	0	Unknow	Unknow	Unknow	Unknow
X658	4	350.858	0.168	Unknow	Unknow	Unknow	Unknow
X660	4	386.082	0	HMDB38515	L-gamma-Glutamyl-S-allylthio-L-cysteine	M+ACN+Na	C11H18N2O5S2
X661	4	140.51	0.36	Unknow	Unknow	Unknow	Unknow
X662	4	334.967	0.238	Unknow	Unknow	Unknow	Unknow
X684	4	149.515	0.208	HMDB13922 ; HMDB60649	2-Chloroticlopidine ; Ascorbic acid-2-sulfate	M+2H ; M+ACN+2H	C14H13Cl2NS ; C6H8O9S
X698	4	238.952	0.299	Unknow	Unknow	Unknow	Unknow
X70	4	796.802	0	HMDB06669	O-6-deoxy-alpha-L-galactopyranosyl-(1-3)-O-O-6-deoxy-alpha-L-galactopyranosyl-(1-4)-O-O-6-deoxy-alpha-L-galactopyranosyl-(1-2)-beta-D-galactopyranosyl-(1-3)-O-2-(acetylamino)-2-deoxy-beta-D-glucopyranosyl-(1-3)-beta-D-galactopyranosyl-(1-4)-O-2-	M+2ACN+2H	C57H95N3O43
X707	4	178.534	0.054	Unknow	Unknow	Unknow	Unknow
X745	4	237.955	0.05	HMDB03474	35-Diiodo-L-tyrosine	M+ACN+2H	C9H9I2NO3
X748	4	369.96	0.194	Unknow	Unknow	Unknow	Unknow
X75	4	654.186	0	HMDB37420 ; HMDB38361 ; HMDB38365	Maysin 3-methyl ether ; (-)-Epigallocatechin ; ()-Gallocatechin	M+ACN+Na ; 2M+ACN+H ; 2M+ACN+H	C28H30O14 ; C15H14O7 ; C15H14O7
X782	4	278.017	0.004	Unknow	Unknow	Unknow	Unknow
X793	4	286.151	0.048	HMDB35988 ; HMDB12881 ; HMDB14524 ; HMDB14692 ; HMDB28865 ; HMDB28886 ; HMDB60678	Ganoderic acid F ; Acetylcarnosine ; Dexrazoxane ; Pentostatin ; Hydroxyprolyl-Histidine ; Histidinyl-Hydroxyproline ; 3-Hydroxymonoethylglycinexylidide	M+2H ; M+NH4 ; M+NH4 ; M+NH4 ; M+NH4 ; M+NH4 ; M+ACN+Na	C32H42O9 ; C11H16N4O4 ; C11H16N4O4 ; C11H16N4O4 ; C11H16N4O4 ; C11H16N4O4 ; C12H18N2O2
X85	4	225.98	0	HMDB04811 ; HMDB41800	24-Dichlorophenol ; 25-Dichlorophenol	M+ACN+Na ; M+ACN+Na	C6H4Cl2O ; C6H4Cl2O
X852	4	114.066	0.132	HMDB41941	NN-dinitrosopiperazine	M+2ACN+2H	C4H8N4O2
X853	4	199.637	0.024	HMDB12280 ; HMDB39880 ; HMDB60044	Phytosphingosine-1-P ; 1-Formylneogrifolin ; 14-HDoHE	M+2H ; M+ACN+2H ; M+ACN+2H	C18H40NO6P ; C23H32O3 ; C23H32O3
X856	4	526.379	0.432	Unknow	Unknow	Unknow	Unknow
X87	4	177.041	0	HMDB00044 ; HMDB06355 ; HMDB31193 ; HMDB00243 ; HMDB11111 ; HMDB31331 ; HMDB40261	Ascorbic acid ; D-Glucurono-63-lactone ; 123-Propanetricarboxylic acid ; Pyruvic acid ; Malonic semialdehyde ; Chloroacetic acid ; Glucosereductone	M+H ; M+H ; M+H ; 2M+H ; 2M+H ; M+2ACN+H ; 2M+H	C6H8O6 ; C6H8O6 ; C6H8O6 ; C3H4O3 ; C3H4O3 ; C2H3ClO2 ; C3H4O3
X870	4	138.959	0.004	HMDB31808	Tecnazene	M+H+NH4	C6HCl4NO2
X880	4	132.529	0.653	Unknow	Unknow	Unknow	Unknow
X917	4	228.028	0.06	HMDB31784	Halofuginone	M+ACN+2H	C16H17BrClN3O3
X125	5	280.007	0	Unknow	Unknow	Unknow	Unknow

X172	5	300.055	0	HMDB33062 ; HMDB36372 ; HMDB36373 ; HMDB38968	2-Propenyl 1-(2-propenylsulfinyl)propyl disulfide ; 1-Propenyl 1-(2-propenylsulfinyl)propyl disulfide ; 2-Propenyl 1-(1-propenylsulfinyl)propyl disulfide ; 1-Propenyl 1-(1-propenylsulfinyl)propyl disulfide	M+ACN+Na ; M+ACN+Na ; M+ACN+Na ; M+ACN+Na	C9H16OS3 ; C9H16OS3 ; C9H16OS3 ; C9H16OS3
X180	5	828.575	0	Unknow	Unknow	Unknow	Unknow
X181	5	412.075	0	Unknow	Unknow	Unknow	Unknow
X206	5	216.551	0	HMDB06953	1-Phosphatidyl-D-myo-inositol	M+ACN+2H	C11H19O13P
X214	5	431.098	0	HMDB33587 ; HMDB41717 ; HMDB41718 ; HMDB34757	Coumestrin ; Daidzein 4-O-glucuronide ; Daidzein 7-O-glucuronide ; 4-Methoxybenzenepropanol 1-(2-sulfoglucoside)	M+H ; M+H ; M+H ; M+Na	C21H18O10 ; C21H18O10 ; C21H18O10 ; C16H24O10S
X219	5	380.058	0	HMDB00370 ; HMDB15533	2-Amino-3-phosphonopropionic acid ; Cinolazepam	2M+ACN+H ; M+Na	C3H8NO5P ; C18H13ClFN3O2
X252	5	707.541	0	Unknow	Unknow	Unknow	Unknow
X255	5	486.087	0	Unknow	Unknow	Unknow	Unknow
X259	5	575.501	0	HMDB29795 ; HMDB32358	Montecristin ; Lauroyl diethanolamide	M+H ; 2M+H	C37H66O4 ; C16H33NO3
X260	5	91.9624	0	Unknow	Unknow	Unknow	Unknow
X277	5	427.095	0	HMDB00656 ; HMDB12248 ; HMDB01011 ; HMDB02009 ; HMDB32299 ; HMDB33343 ; HMDB37199 ; HMDB37201 ; HMDB37202 ; HMDB59627	Cysteineglutathione disulfide ; L-3-Aminobutyryl-CoA ; Methacrylyl-CoA ; Crotonoyl-CoA ; Glyceryl lactopalmitate ; Mollicellin D ; 2-O-Galloyl-14-galactarolactone ; 3-O-Galloyl-14-galactarolactone ; 5-O-Galloyl-14-galactarolactone ; (E)-but-2-enoyl-CoA	M+H ; M+2H ; M+H+NH4 ; M+H+NH4 ; M+Na ; M+Na ; M+2ACN+H ; M+2ACN+H ; M+2ACN+H ; M+H+NH4	C13H22N4O8S2 ; C25H43N8O17P3S ; C25H40N7O17P3S ; C25H40N7O17P3S ; C20H16N6O2S ; C21H21ClO6 ; C13H12O11 ; C13H12O11 ; C13H12O11 ; C25H40N7O17P3S
X278	5	367.062	0	HMDB01439 ; HMDB02377 ; HMDB02666 ; HMDB39287	Phosphoribosyl formamidocarboxamide ; Urothion ; Thiamine monophosphate ; Theogallin	M+H ; M+ACN+H ; M+Na ; M+Na	C10H15N4O9P ; C11H11N5O3S2 ; C12H17N4O4PS ; C14H16O10
X282	5	214.553	0	HMDB30560 ; HMDB34582	71112-Triacetoxycoumestan ; Shoyuflavone A	M+H+NH4 ; M+ACN+2H	C21H14O9 ; C19H14O9
X290	5	215.054	0	HMDB03072 ; HMDB03705 ; HMDB36354 ; HMDB38417	Quinic acid ; Phosphoguanidinoacetate ; 1-Pentenyl glucosinolate ; Glucobrassicanapin	M+Na ; M+NH4 ; M+ACN+2H ; M+ACN+2H	C7H12O6 ; C3H8N3O5P ; C12H21NO9S2 ; C12H21NO9S2
X301	5	179.003	0	HMDB31154	Allitridin	M+H	C6H10S3
X305	5	323.033	0	HMDB02708	Cyanidin	M+H	C15H11ClO6
X322	5	216.057	0	HMDB00175 ; HMDB04381 ; HMDB06268 ; HMDB06880 ; HMDB11681 ; HMDB12152 ; HMDB41565	Inosinic acid ; N-Acetylneuraminic acid 9-phosphate ; N-Acetylneuraminate 9-phosphate ; Acetyl adenylate ; Inosine 2-phosphate ; 2-O-(6-Phospho-alpha-mannosyl)-D-glycerate ; 57-Dihydro-2-methylthieno34-dpyrimidine	M+2ACN+2H ; M+ACN+2H ; M+ACN+2H ; M+ACN+2H ; M+2ACN+2H ; M+2ACN+2H ; M+ACN+Na	C10H13N4O8P ; C11H20NO12P ; C11H20NO12P ; C12H16N5O8P ; C10H13N4O8P ; C9H17O12P ; C7H8N2S
X324	5	147.026	0	Unknow	Unknow	Unknow	Unknow
X352	5	205.548	0	Unknow	Unknow	Unknow	Unknow
X360	5	290.034	0	HMDB15420 ; HMDB15677 ; HMDB41788	Quinethazone ; Dimercaprol ; Vanillic acid 4-sulfate	M+H ; 2M+ACN+H ; M+ACN+H	C10H12ClN3O3S ; C3H8OS2 ; C8H8O7S
X372	5	215.551	0	HMDB13855	Sulfamethoxazole N1-glucuronide	M+2H	C16H19N3O9S
X380	5	169.527	0	Unknow	Unknow	Unknow	Unknow
X408	5	206.04	0	Unknow	Unknow	Unknow	Unknow
X434	5	187.054	0	HMDB14533 ; HMDB29674 ; HMDB00905 ; HMDB14694 ; HMDB33582 ; HMDB39109	Carbimazole ; 2-Chloro-13-dimethoxy-5-methylbenzene ; Deoxyadenosine monophosphate ; Piroxicam ; (E)-111-Tridecadiene-3579-tetrayne ; (Z)-13-Tridecadiene-57911-tetrayne	M+H ; M+H ; M+ACN+2H ; M+ACN+2H ; M+Na ; M+Na	C7H10N2O2S ; C9H11ClO2 ; C10H14N5O6P ; C15H13N3O4S ; C13H8 ; C13H8
X438	5	105.978	0	HMDB29643	135-Trichloro-2-methoxybenzene	M+2H	C7H5Cl3O
X443	5	300.059	0	HMDB30568 ; HMDB33350	Trisjuglone ; Brassinin	M+2ACN+2H ; M+ACN+Na	C30H12O9 ; C11H12N2S2
X457	5	326.994	0	HMDB15679 ; HMDB33585	Niclosamide ; Acesulfame	M+H ; 2M+H	C13H8Cl2N2O4 ; C4H5NO4S
X465	5	353.066	0	HMDB14939 ; HMDB30756	Halazepam ; 13alpha-Hydroxydolineone	M+H ; M+H	C17H12ClF3N2O ; C19H12O7

X474	5	183.539	0	HMDB14665 ; HMDB14946 ; HMDB00288 ; HMDB01271 ; HMDB11641 ; HMDB60282	Metolazone ; Indapamide ; Uridine 5-monophosphate ; Pseudouridine 5-phosphate ; Uridine 2-phosphate ; 3-UMP	M+2H ; M+2H ; M+ACN+2H ; M+ACN+2H ; M+ACN+2H ; M+ACN+2H	C16H16ClN3O3S ; C16H16ClN3O3S ; C9H13N2O9P ; C9H13N2O9P ; C9H13N2O9P ; C9H13N2O9P
X497	5	418.748	0	Unknow	Unknow	Unknow	Unknow
X506	5	340.058	0	HMDB15484 ; HMDB30176 ; HMDB34923 ; HMDB60924 ; HMDB60925	Cefacetrile ; Norsanguinarine ; Lettowianthine ; Suprofen S-oxide ; Thiophene-45-epoxide	M+H ; M+Na ; M+Na ; M+ACN+Na ; M+ACN+Na	C13H13N3O6S ; C19H11NO4 ; C19H11NO4 ; C14H12O4S ; C14H12O4S
X56	5	678.503	0	HMDB07866 ; HMDB08890 ; HMDB08922 ; HMDB07032 ; HMDB07033 ; HMDB07060 ; HMDB07114 ; HMDB07141 ; HMDB07142 ; HMDB07193 ; HMDB07222 ; HMDB07249 ; HMDB07250 ; HMDB07277 ; HMDB07288 ; HMDB07306 ; HMDB07317 ; HMDB07333 ; HMDB07334 ; HMDB07344 ; HMDB07345 ; HMDB07353 ; HMDB07505 ; HMDB07512 ; HMDB07534 ; HMDB07541 ; HMDB07562 ; HMDB07568 ; HMDB07569 ; HMDB07578 ; HMDB07676 ; HMDB07704 ; HMDB07733 ; HMDB07773 ; HMDB31461 ; HMDB55974 ; HMDB55979 ; HMDB56033 ; HMDB56148 ; HMDB56167 ; HMDB56173 ; HMDB56192 ; HMDB56211 ; HMDB56306 ; HMDB56337 ; HMDB56369 ; HMDB56373 ; HMDB56376 ; HMDB56381	PC(14:014:0) ; PE(15:016:0) ; PE(16:015:0) ; DG(14:022:5(4Z7Z10Z13Z16Z)0:0) ; DG(14:022:5(7Z10Z13Z16Z19Z)0:0) ; DG(14:1(9Z)22:4(7Z10Z13Z16Z)0:0) ; DG(16:020:5(5Z8Z11Z14Z17Z)0:0) ; DG(16:1(9Z)20:4(5Z8Z11Z14Z)0:0) ; DG(16:1(9Z)20:4(8Z11Z14Z17Z)0:0) ; DG(18:1(11Z)18:4(6Z9Z12Z15Z)0:0) ; DG(18:1(9Z)18:4(6Z9Z12Z15Z)0:0) ; DG(18:2(9Z12Z)18:3(6Z9Z12Z)0:0) ; DG(18:2(9Z12Z)18:3(9Z12Z15Z)0:0) ; DG(18:3(6Z9Z12Z)18:2(9Z12Z)0:0) ; DG(18:3(6Z9Z12Z)20:5(5Z8Z11Z14Z17Z)0:0) ; DG(18:3(9Z12Z15Z)18:2(9Z12Z)0:0) ; DG(18:3(9Z12Z15Z)20:5(5Z8Z11Z14Z17Z)0:0) ; DG(18:4(6Z9Z12Z15Z)18:1(11Z)0:0) ; DG(18:4(6Z9Z12Z15Z)18:1(9Z)0:0) ; DG(18:4(6Z9Z12Z15Z)20:4(5Z8Z11Z14Z)0:0) ; DG(18:4(6Z9Z12Z15Z)20:4(8Z11Z14Z17Z)0:0) ; DG(18:4(6Z9Z12Z15Z)22:6(4Z7Z10Z13Z16Z19Z)0:0) ; DG(20:4(5Z8Z11Z14Z)16:1(9Z)0:0) ;	M+H ; M+H ; M+H ; M+ACN+Na ; M+ACN+Na ; M+ACN+Na ; M+ACN+Na ; M+ACN+Na ; M+ACN+Na ; M+ACN+Na ; M+ACN+Na ; M+ACN+Na ; M+ACN+Na ; M+ACN+Na ; M+ACN+H ; M+ACN+Na ; M+ACN+H ; M+ACN+Na ; M+ACN+Na ; M+ACN+H ; M+ACN+H ; M+NH4 ; M+ACN+Na ; M+ACN+H ; M+ACN+Na ; M+ACN+H ; M+ACN+Na ; M+ACN+H ; M+ACN+H ; M+NH4 ; M+ACN+Na ; M+ACN+Na ; M+ACN+Na ; M+NH4 ; 2M+ACN+H ; M+ACN+Na ; M+ACN+Na ; M+ACN+Na ; M+ACN+Na ; M+ACN+Na ; M+ACN+Na ; M+ACN+Na ; M+ACN+Na ; M+ACN+H ; M+ACN+H ; M+ACN+H ; M+ACN+H ; M+NH4 ; M+NH4	C36H72NO8P ; C36H72NO8P ; C36H72NO8P ; C39H66O5 ; C39H66O5 ; C39H66O5 ; C39H66O5 ; C39H66O5 ; C39H66O5 ; C39H66O5 ; C39H66O5 ; C39H66O5 ; C39H66O5 ; C39H66O5 ; C41H64O5 ; C39H66O5 ; C41H64O5 ; C39H66O5 ; C39H66O5 ; C41H64O5 ; C41H64O5 ; C43H64O5 ; C39H66O5 ; C41H64O5 ; C39H66O5 ; C41H64O5 ; C39H66O5 ; C41H64O5 ; C41H64O5 ; C43H64O5 ; C39H66O5 ; C39H66O5 ; C39H66O5 ; C43H64O5 ; C24H30 ; C39H66O5 ; C39H66O5 ; C39H66O5 ; C39H66O5 ; C39H66O5 ; C39H66O5 ; C39H66O5 ; C39H66O5 ; C41H64O5 ; C41H64O5 ; C41H64O5 ; C41H64O5 ; C43H64O5 ; C43H64O5
X62	5	122.014	0	Unknow	Unknow	Unknow	Unknow
X659	5	289.04	0	HMDB29191	5-(34-Dihydroxyphenyl)-gamma-valerolactone sulfate	M+H	C11H12O7S
X209	6	463.176	0	HMDB39233 ; HMDB14414 ; HMDB15686 ; HMDB32156 ; HMDB39914 ; HMDB39915 ; HMDB39916 ; HMDB39917	Verbasoside ; Chlorotrianisene ; Pipazethate ; alpha-Acetolactate decarboxylase (enzyme preparation from bacillus subtilis recombinant) ; 16-Dihydroxy-37-dimethoxy-2-(3-methyl-2-butenyl)-8-(3-hydroxy-3-methyl-1E-butenyl)-xanthone ; 16-Dihydroxy-37-dimethoxy-2-(2-hydroxy-3-methyl-3-butenyl)-8-(3-methyl-2-butenyl)-xanthone ; 16-Dihydroxy-37-dimethoxy-2-(3-methyl-2-butenyl)-8-(2-hydroxy-3-methyl-3-butenyl)-xanthone ; 13-Dihydroxy-67-dimethoxy-2-(2-hydroxy-3-methyl-3-butenyl)-8-(3-methyl-2-butenyl)-xanthone	M+H ; M+2ACN+H ; M+ACN+Na ; M+ACN+Na ; M+Na ; M+Na ; M+Na ; M+Na	C20H30O12 ; C23H21ClO3 ; C21H25N3O3S ; C21H25N3O3S ; C25H28O7 ; C25H28O7 ; C25H28O7 ; C25H28O7
X411	6	552.965	0	Unknow	Unknow	Unknow	Unknow
X101	7	342.602	0	Unknow	Unknow	Unknow	Unknow
X110	7	290.16	0	HMDB00552 ; HMDB02053 ; HMDB04181 ; HMDB11603 ; HMDB31852 ; HMDB31876 ; HMDB33064 ; HMDB33663 ; HMDB40141 ; HMDB40143	3-Methylglutarylcarnitine ; Histidylproline diketopiperazine ; Methylimidazole acetaldehyde ; 4-(Methylnitrosamino)-1-(3-pyridyl)-1-butanone ; 2-Methoxy-3-methylpyrazine ; Prenyl glucoside ; (E)-2-Methyl-2-buten-1-ol O-beta-D-Glucopyranoside ; Decarbamoylneosaxitoxin ; 2-Methoxy-5-methylpyrazine ; 2-Methoxy-6-methylpyrazine	M+H ; M+ACN+H ; 2M+ACN+H ; M+2ACN+H ; 2M+ACN+H ; M+ACN+H ; M+ACN+H ; M+NH4 ; 2M+ACN+H ; 2M+ACN+H	C13H23NO6 ; C12H16N4O2 ; C6H8N2O ; C10H13N3O2 ; C6H8N2O ; C11H20O6 ; C11H20O6 ; C9H16N6O4 ; C6H8N2O ; C6H8N2O
X111	7	321.997	0	Unknow	Unknow	Unknow	Unknow
X114	7	378.546	0	Unknow	Unknow	Unknow	Unknow

X117	7	500.12	0	HMDB03153 ; HMDB39624 ; HMDB39719 ; HMDB41147 ; HMDB41258 ; HMDB41418 ; HMDB41598	Epigallocatechin gallate ; Assamicain C ; Assamicain A ; Isochinomin ; Homomangiferin ; Taxifolin 3-arabinoside ; 3-Galloylgallocatechin	M+ACN+H ; M+2ACN+2H ; M+2ACN+2H ; M+ACN+Na ; M+ACN+Na ; M+ACN+Na ; M+ACN+H	C22H18O11 ; C44H36O22 ; C44H36O22 ; C20H20O11 ; C20H20O11 ; C20H20O11 ; C22H18O11
X119	7	498.534	0	Unknow	Unknow	Unknow	Unknow
X124	7	358.601	0	Unknow	Unknow	Unknow	Unknow
X129	7	162.076	0	HMDB00510 ; HMDB29423 ; HMDB33747 ; HMDB30416 ; HMDB33105 ; HMDB33106 ; HMDB00042 ; HMDB00337 ; HMDB00360 ; HMDB00393 ; HMDB00498 ; HMDB00522 ; HMDB01366 ; HMDB02266 ; HMDB02453 ; HMDB02601 ; HMDB02649 ; HMDB03331 ; HMDB03344 ; HMDB04044 ; HMDB04326 ; HMDB06023 ; HMDB06293 ; HMDB13311 ; HMDB29171 ; HMDB29954 ; HMDB31257 ; HMDB36232 ; HMDB37843 ; HMDB40207	Aminoadipic acid ; Acetylhomoserine ; ()-22-Iminobispropanoic acid ; Avenic acid A ; N2-Galacturonyl-L-lysine ; N6-Galacturonyl-L-lysine ; Acetic acid ; (S)-34-Dihydroxybutyric acid ; 24-Dihydroxybutanoic acid ; 3-Hexenedioic acid ; 4-Deoxyerythronic acid ; 3-Methylglutaconic acid ; Purine ; (E)-2-Methylglutaconic acid ; 4-Deoxythreonic acid ; Ab-Dihydroxyisobutyric acid ; Erythrose ; 1-Methyladenosine ; Glycolaldehyde ; N6-Methyladenosine ; 2-O-Methyladenosine ; 3-O-Methyladenosine ; L-Erythrulose ; trans-2-Hexenedioic acid ; 3-Hydroxyadipic acid 36-lactone ; D-glycero-L-galacto-Octulose ; Dimethyl fumarate ; Maleic acid homopolymer ; N-(1-Deoxy-1-fructosyl)threonine ; Ethyl hydrogen fumarate	M+H ; M+H ; M+H ; M+2H ; M+2H ; M+2H ; 2M+ACN+H ; M+ACN+H ; M+ACN+H ; M+NH4 ; M+ACN+H ; M+NH4 ; M+ACN+H ; M+NH4 ; M+ACN+H ; M+ACN+H ; M+ACN+H ; M+ACN+2H ; 2M+ACN+H ; M+ACN+2H ; M+ACN+2H ; M+ACN+2H ; M+ACN+H ; M+NH4 ; M+NH4 ; M+2ACN+2H ; M+NH4 ; M+NH4 ; M+ACN+2H ; M+NH4	C6H11NO4 ; C6H11NO4 ; C6H11NO4 ; C12H22N2O8 ; C12H22N2O8 ; C12H22N2O8 ; C2H4O2 ; C4H8O4 ; C4H8O4 ; C6H8O4 ; C4H8O4 ; C6H8O4 ; C5H4N4 ; C6H8O4 ; C4H8O4 ; C4H8O4 ; C4H8O4 ; C11H15N5O4 ; C2H4O2 ; C11H15N5O4 ; C11H15N5O4 ; C11H15N5O4 ; C4H8O4 ; C6H8O4 ; C6H8O4 ; C8H16O8 ; C6H8O4 ; C6H8O4 ; C10H19NO8 ; C6H8O4
X130	7	205.073	0	HMDB14555 ; HMDB30590 ; HMDB33100 ; HMDB35196 ; HMDB36343 ; HMDB37570	Carbachol ; Dihydrosterigmatocystin ; Atroviridin ; Harman ; O-Demethylforbexanthone ; Demethoxykanugin	M+Na ; M+2ACN+2H ; M+2ACN+2H ; M+Na ; M+2ACN+2H ; M+2ACN+2H	C6H15ClN2O2 ; C18H14O6 ; C18H14O6 ; C12H10N2 ; C18H14O6 ; C18H14O6
X133	7	161.128	0	HMDB02038 ; HMDB06009 ; HMDB04827 ; HMDB30410 ; HMDB37213 ; HMDB38949	N(6)-Methyllysine ; Isoputreanine ; Proline betaine ; L-2-Amino-3-methylenehexanoic acid ; xi-p-Menth-3-ene ; 3beta6beta-Dihydroxynortropane	M+H ; M+H ; M+NH4 ; M+NH4 ; M+Na ; M+NH4	C7H16N2O2 ; C7H16N2O2 ; C7H13NO2 ; C7H13NO2 ; C10H18 ; C7H13NO2
X136	7	375.533	0	Unknow	Unknow	Unknow	Unknow
X140	7	618.109	0	Unknow	Unknow	Unknow	Unknow
X142	7	148.028	0	Unknow	Unknow	Unknow	Unknow
X16	7	630.083	0	Unknow	Unknow	Unknow	Unknow
X167	7	369.541	0	Unknow	Unknow	Unknow	Unknow
X187	7	162.132	0	HMDB05045 ; HMDB36802 ; HMDB60052	15(S)-Hydroxyeicosatrienoic acid ; Austroinulin ; 8-HETrE	M+2H ; M+2H ; M+2H	C20H34O3 ; C20H34O3 ; C20H34O3

X188	7	205.119	0	HMDB29052 ; HMDB29136 ; HMDB33891 ; HMDB39948 ; HMDB00473 ; HMDB02994 ; HMDB04136 ; HMDB11727 ; HMDB12150 ; HMDB30006 ; HMDB30424 ; HMDB32050 ; HMDB32463 ; HMDB32862 ; HMDB34708 ; HMDB34730 ; HMDB35156 ; HMDB35359 ; HMDB36191 ; HMDB36215 ; HMDB37388 ; HMDB37829 ; HMDB38005 ; HMDB38068 ; HMDB38079 ; HMDB38245 ; HMDB38279 ; HMDB40595 ; HMDB59816	Serinyl-Valine ; Valyl-Serine ; N6-Acetyl-5S-hydroxy-L-lysine ; 34-Dihydroxy-2-hydroxymethyl-1-pyrrolidinepropanamide ; 6-Dimethylaminopurine ; Erythritol ; D-Threitol ; Bicine ; 2-Keto-6-acetamidocaproate ; 2277-Tetramethyl-16-dioxaspiro44non-3-ene ; Linalyl formate ; alpha-Terpineol formate ; 5-Pentyl-3h-furan-2-one ; 2-Amino-4-ethoxy-3-hydroxybutanoic acid ; Methyl 48-decadienoate ; Methyl (2E4Z)-decadienoate ; Neryl formate ; 1-Deoxynojirimycin ; 4-Hydroxy-4-methyl-7-decenoic acid gamma-lactone ; Ethyl octynecarboxylate ; Tsibulin 2 ; 6-(3-Hexenyl)tetrahydro-2H-pyran-2-one ; xi-2-Hexyl-5-methyl-3(2H)-furanone ; (3E6Z)-Nonadien-1-yl acetate ; (2E6Z)-26-Nonadien-1-Yl acetate ; Bornyl formate ; cis-3-Hexenyl tiglate ; Allyl cyclohexylacetate ; Methyl geranate	M+H ; M+H ; M+H ; M+H ; M+ACN+H ; M+2ACN+H ; M+2ACN+H ; M+ACN+H ; M+NH4 ; M+Na ; M+Na ; M+Na ; M+Na ; M+ACN+H ; M+Na ; M+Na ; M+Na ; M+ACN+H ; M+Na ; M+Na ; M+Na ; M+Na ; M+Na ; M+Na ; M+Na ; M+Na ; M+Na ; M+Na ; M+Na	C8H16N2O4 ; C8H16N2O4 ; C8H16N2O4 ; C8H16N2O4 ; C7H9N5 ; C4H10O4 ; C4H10O4 ; C6H13NO4 ; C8H13NO4 ; C11H18O2 ; C11H18O2 ; C11H18O2 ; C11H18O2 ; C6H13NO4 ; C11H18O2 ; C11H18O2 ; C11H18O2 ; C6H13NO4 ; C11H18O2 ; C11H18O2 ; C11H18O2 ; C11H18O2 ; C11H18O2 ; C11H18O2 ; C11H18O2 ; C11H18O2 ; C11H18O2 ; C11H18O2 ; C11H18O2
X194	7	301.577	0	Unknow	Unknow	Unknow	Unknow
X211	7	212.054	0	HMDB11639 ; HMDB29842 ; HMDB34864 ; HMDB59999 ; HMDB00426 ; HMDB00428 ; HMDB00590 ; HMDB00606 ; HMDB00694 ; HMDB01138 ; HMDB01900 ; HMDB05807 ; HMDB11676 ; HMDB29649 ; HMDB31574 ; HMDB32499 ; HMDB33912 ; HMDB38890 ; HMDB41392 ; HMDB59655 ; HMDB59964	Topaquinone ; Betalamic acid ; 24-Dihydroxy-7-methoxy-2H-14-benzoxazin-3(4H)-one ; Gentisuric acid ; Citramalic acid ; 3-Hydroxyglutaric acid ; Glutarylglycine ; D-2-Hydroxyglutaric acid ; L-2-Hydroxyglutaric acid ; N-Acetylglutamic acid ; Ribonolactone ; Gallic acid ; D-Xylono-15-lactone ; 246-Trihydroxybenzoic acid ; 1-Isothiocyanato-6-(methylthio)hexane ; Pyrrolidino-12E-4H-24-dimethyl-135-dithiazine ; 2-Propenyl propyl disulfide ; Methyl 3-methyl-1-butenyl disulfide ; 1-Propenyl propyl disulfide ; 2-Hydroxyglutarate ; 234-Trihydroxybenzoic acid	M+H ; M+H ; M+H ; M+H ; M+ACN+Na ; M+ACN+Na ; M+Na ; M+ACN+Na ; M+ACN+Na ; M+Na ; M+ACN+Na ; M+ACN+H ; M+ACN+Na ; M+ACN+H ; M+Na ; M+Na ; M+ACN+Na ; M+ACN+Na ; M+ACN+Na ; M+ACN+Na ; M+ACN+H	C9H9NO5 ; C9H9NO5 ; C9H9NO5 ; C9H9NO5 ; C5H8O5 ; C5H8O5 ; C7H11NO5 ; C5H8O5 ; C5H8O5 ; C7H11NO5 ; C5H8O5 ; C7H6O5 ; C5H8O5 ; C7H6O5 ; C8H15NS2 ; C8H15NS2 ; C6H12S2 ; C6H12S2 ; C6H12S2 ; C5H8O5 ; C7H6O5
X213	7	328.594	0	HMDB01176	Cytidine monophosphate N-acetylneuraminic acid	M+ACN+2H	C20H31N4O16P
X22	7	285.121	0	HMDB00089 ; HMDB11177 ; HMDB11179 ; HMDB14617 ; HMDB14798 ; HMDB15122 ; HMDB29436 ; HMDB31770 ; HMDB32706 ; HMDB32713 ; HMDB33701 ; HMDB34663 ; HMDB35475 ; HMDB38115 ; HMDB60478	Cytidine ; L-phenylalanyl-L-proline ; L-prolyl-L-phenylalanine ; Methohexital ; Metaxalone ; Cytarabine ; Penmacric acid ; Carbofuran ; (2-Naphthalenyloxy)acetic acid ; 345-Biphenyltriol ; Hydrocotarnine ; 5-Hydroxy-23-dimethyl-14-naphthoquinone ; Simmondsin 2-ferulate ; Setarin ; gamma-Glutamyl-beta-cyanoalanine	M+ACN+H ; M+Na ; M+Na ; M+Na ; M+ACN+Na ; M+ACN+H ; M+2ACN+H ; M+ACN+Na ; M+2ACN+H ; M+2ACN+H ; M+ACN+Na ; M+2ACN+H ; M+H+NH4 ; M+2ACN+H ; M+ACN+H	C9H13N3O5 ; C14H18N2O3 ; C14H18N2O3 ; C14H18N2O3 ; C12H15NO3 ; C9H13N3O5 ; C7H10N2O5 ; C12H15NO3 ; C12H10O3 ; C12H10O3 ; C12H15NO3 ; C12H10O3 ; C26H33NO12 ; C12H10O3 ; C9H13N3O5
X220	7	336.048	0	HMDB59993	5-(Hydroxyphenyl)-gamma-valerolactone-O-sulphate	M+ACN+Na	C11H12O6S
X223	7	327.596	0	HMDB03337	Oxidized glutathione	M+ACN+2H	C20H32N6O12S2
X225	7	358.099	0	HMDB29948 ; HMDB00528 ; HMDB03349 ; HMDB60006 ; HMDB60771 ; HMDB61153	Scleroglucan ; 45-Dihydroorotic acid ; L-Dihydroorotic acid ; N-(2-formyl-3-chlorophenyl)anthranilic acid ; 4-Hydroxyclobazam ; Lorcaserin sulfamate	M+2H ; 2M+ACN+H ; 2M+ACN+H ; M+2ACN+H ; M+ACN+H ; M+2ACN+H	C24H44O20P2 ; C5H6N2O4 ; C5H6N2O4 ; C14H10ClNO3 ; C16H13ClN2O3 ; C11H14ClNO3S
X229	7	415.517	0	Unknow	Unknow	Unknow	Unknow

X250	7	372.106	0	HMDB14854 ; HMDB41412 ; HMDB14258 ; HMDB14720 ; HMDB29263 ; HMDB30475 ; HMDB30480 ; HMDB30696 ; HMDB30811 ; HMDB31706 ; HMDB33268 ; HMDB34009 ; HMDB35598 ; HMDB37096 ; HMDB37351 ; HMDB37354 ; HMDB38659 ; HMDB40692 ; HMDB41690	Nedocromil ; Quercetin 3-(2Gal-apiosylrobinobioside) ; R-138727 ; Voriconazole ; 37-Dimethylquercetin ; Aflatoxin G2 ; Aflatoxin M2 ; Demethoxysudachitin ; Prudomestin ; 34-Dihydroxyphenacyl caffeate ; 135-Trihydroxy-67-dimethoxy-2-methylanthraquinone ; Trifolian ; Aflatoxin B2a ; Pilosin ; Caryatin ; 34-Di-O-methylquercetin ; Cicerin ; 4R5R6S-Trihydroxy-2-hydroxymethyl-2-cyclohexen-1-one 6-(2-hydroxy-6-methylbenzoate) ; 57-Dihydroxy-84-dimethoxyisoflavone	M+H ; M+2H ; M+Na ; M+Na ; M+ACN+H ; M+ACN+H ; M+ACN+H ; M+ACN+H ; M+ACN+H ; M+ACN+H ; M+ACN+H ; M+ACN+H ; M+ACN+H ; M+ACN+H ; M+ACN+H ; M+ACN+H ; M+ACN+H ; M+ACN+Na ; M+ACN+H	C19H17NO7 ; C32H38O20 ; C18H20FNO3S ; C16H14F3N5O ; C17H14O7 ; C17H14O7 ; C17H14O7 ; C17H14O7 ; C17H14O7 ; C17H14O7 ; C17H14O7 ; C17H14O7 ; C17H14O7 ; C17H14O7 ; C17H14O7 ; C17H14O7 ; C17H14O7 ; C15H16O7 ; C17H14O7
X253	7	347.072	0	HMDB32927 ; HMDB32984	Quindoxin ; Mahaleboside	2M+Na ; M+Na	C8H6N2O2 ; C15H16O8
X262	7	386.943	0	Unknow	Unknow	Unknow	Unknow
X27	7	770.784	0	Unknow	Unknow	Unknow	Unknow
X272	7	712.111	0	Unknow	Unknow	Unknow	Unknow
X274	7	189.032	0	HMDB00251	Taurine	M+ACN+Na	C2H7NO3S
X275	7	163.016	0	Unknow	Unknow	Unknow	Unknow
X279	7	658.349	0	HMDB34879	1-(5-Amino-5-carboxypentyl)amino-1-deoxyfructose	2M+ACN+H	C12H24N2O7
X285	7	211.552	0	Unknow	Unknow	Unknow	Unknow
X292	7	232.103	0	HMDB35864 ; HMDB38159 ; HMDB15164 ; HMDB29398	13-Hydroxy-5-O-methylmelledonal ; Retrocalamin ; Topotecan ; (3-Nitroamino)alanine	M+2H ; M+2H ; M+ACN+2H ; M+2ACN+H	C24H30O9 ; C24H30O9 ; C23H23N3O5 ; C3H7N3O4
X30	7	281.572	0	HMDB15574 ; HMDB40680	Latamoxef ; Melitric acid B	M+ACN+2H ; M+ACN+2H	C20H20N6O9S ; C27H20O11
X307	7	206.04	0	Unknow	Unknow	Unknow	Unknow
X313	7	131.042	0	HMDB33265 ; HMDB39616 ; HMDB40630	2-Methoxystypandrone ; Orientalone ; Pratenol A	M+2H ; M+2H ; M+2H	C14H12O5 ; C14H12O5 ; C14H12O5
X33	7	657.946	0	Unknow	Unknow	Unknow	Unknow
X347	7	298.097	0	HMDB01173 ; HMDB01885 ; HMDB14802 ; HMDB61037	5-Methylthioadenosine ; 3-Chlorotyrosine ; Sulfametopyrazine ; 44-methanol-bisbenzonitrile	M+H ; M+2ACN+H ; M+NH4 ; M+ACN+Na	C11H15N5O3S ; C9H10ClNO3 ; C11H12N4O3S ; C15H10N2O
X348	7	339.055	0	Unknow	Unknow	Unknow	Unknow
X359	7	346.57	0	HMDB60465	cyclic GMP-AMP	M+H+NH4	C20H24N10O13P2
X36	7	360.118	0	HMDB11685 ; HMDB28765 ; HMDB29101 ; HMDB29332 ; HMDB29827 ; HMDB30772 ; HMDB33524 ; HMDB38518 ; HMDB39333 ; HMDB40539 ; HMDB40873 ; HMDB40931 ; HMDB60885 ; HMDB60886 ; HMDB60887 ; HMDB60888	DHAP(8:0) ; Aspartyl-Tyrosine ; Tyrosyl-Aspartate ; 5-Methoxynoracronycine ; Junosidine ; Demethoxyegonol ; Lambertine ; Desmosflavone ; Quercetin 3-(4-acetylrhamnoside) 7-rhamnoside ; Kaempferol 3-(6-acetylgalactoside) 7-rhamnoside ; Dimethylstrobochrysin ; 1-(4-Hydroxy-3-methoxyphenyl)-5-(4-hydroxyphenyl)-14-pentadien-3-one ; 4-Hydroxy-R-phenprocoumon ; 6-Hydroxy-R-phenprocoumon ; 8-Hydroxy-R-phenprocoumon ; 7-Hydroxy-R-phenprocoumon	M+ACN+Na ; M+ACN+Na ; M+ACN+Na ; M+Na ; M+Na ; M+ACN+Na ; M+Na ; M+ACN+Na ; M+2ACN+2H ; M+2ACN+2H ; M+ACN+Na ; M+ACN+Na ; M+ACN+Na ; M+ACN+Na ; M+ACN+Na ; M+ACN+Na	C11H21O7P ; C13H16N2O6 ; C13H16N2O6 ; C20H19NO4 ; C20H19NO4 ; C18H16O4 ; C20H19NO4 ; C18H16O4 ; C29H32O16 ; C29H32O16 ; C18H16O4 ; C18H16O4 ; C18H16O4 ; C18H16O4 ; C18H16O4 ; C18H16O4
X361	7	349.118	0	HMDB01557 ; HMDB29299 ; HMDB00125 ; HMDB01424 ; HMDB04186 ; HMDB14507 ; HMDB15141 ; HMDB15166 ; HMDB29826 ; HMDB30177 ; HMDB33247 ; HMDB33351 ; HMDB34052 ; HMDB35435 ; HMDB38009 ; HMDB38033 ; HMDB40790 ; HMDB60610	Riboflavin reduced ; Sesamolinol 4-O-b-D-glucosyl (1-6)-O-b-D-glucoside ; Glutathione ; 4-(3-Pyridyl)-3-butenoic acid ; 3-Methyldioxyindole ; Clozapine ; Letrozole ; Probenecid ; Hallacridone ; Arborinine ; Aristolodione ; Methoxybrassinin ; Safflomin C ; Cinchonain Id 7-glucoside ; Malvin ; Malvidin 3-laminaribioside ; 4-Methoxybrassinin ; Hydroxyhexamide	M+H ; M+2H ; M+ACN+H ; 2M+Na ; 2M+Na ; M+Na ; M+ACN+Na ; M+ACN+Na ; M+ACN+H ; M+ACN+Na ; M+ACN+H ; M+2ACN+H ; M+2ACN+2H ; M+2ACN+2H ; M+ACN+2H ; M+ACN+2H ; M+2ACN+H ; M+Na	C15H16N4O6 ; C32H40O17 ; C10H17N3O6S ; C9H9NO2 ; C9H9NO2 ; C18H19ClN4 ; C17H11N5 ; C13H19NO4S ; C18H13NO4 ; C16H15NO4 ; C18H13NO4 ; C12H14N2OS2 ; C30H30O14 ; C30H30O14 ; C29H35O17 ; C29H35O17 ; C12H14N2OS2 ; C15H22N2O4S

X37	7	281.071	0	HMDB37687 ; HMDB40676 ; HMDB15059 ; HMDB28777 ; HMDB28882 ; HMDB29212 ; HMDB29214 ; HMDB33668 ; HMDB38384 ; HMDB41769	Prunus inhibitor b ; (2S2R3S3R4S)-3457-Tetrahydroxyflavan(2-74-8)-33557-pentahydroxyflavan ; Ceforanide ; Cysteinyl-Histidine ; Histidinyl-Cysteine ; Quercetin 3-O-glucuronide ; Quercetin-4-glucuronide ; Quercetin 4-glucuronide ; Neoglucobrassicin ; Quercetin 3-O-glucuronide	M+2H ; M+2H ; M+ACN+2H ; M+Na ; M+Na ; M+2ACN+2H ; M+2ACN+2H ; M+2ACN+2H ; M+2ACN+2H ; M+2ACN+2H	C30H24O11 ; C30H24O11 ; C20H21N7O6S2 ; C9H14N4O3S ; C9H14N4O3S ; C21H18O13 ; C21H18O13 ; C21H18O13 ; C17H22N2O10S2 ; C21H18O13
X38	7	291.138	0	HMDB40916 ; HMDB60517 ; HMDB60519 ; HMDB14400 ; HMDB15010 ; HMDB29639 ; HMDB29697 ; HMDB29698 ; HMDB30839 ; HMDB31626 ; HMDB32024 ; HMDB32142 ; HMDB32608 ; HMDB32623 ; HMDB33716 ; HMDB34107 ; HMDB34563 ; HMDB36441 ; HMDB59601 ; HMDB59895	2-(4-Methyl-3-pentenyl)anthraquinone ; trans-34-Dihydro-34-dihydroxy-712-dimethylbenzaanthracene ; trans-56-Dihydro-56-dihydroxy-712-dimethylbenzaanthracene ; Diethylstilbestrol ; Conivaptan ; (4-Methylphenyl)acetaldehyde ; Cinnamyl alcohol ; trans-Cinnamyl alcohol ; (Z)-4-(1-Propenyl)phenol ; 2-Phenylpropanal ; 4-Ethylbenzaldehyde ; 24-Dimethylbenzaldehyde ; 4-Methylacetophenone ; 1-Phenyl-1-propanone ; 3-Phenylpropanal ; Chavicol ; 7-Nonene-35-diyn-1-ol ; o-Vinylanisole ; Indan-1-ol ; m-Methylacetophenone	M+H ; M+H ; M+H ; M+Na ; M+2ACN+2H ; 2M+Na ; 2M+Na ; 2M+Na ; 2M+Na ; 2M+Na ; 2M+Na ; 2M+Na ; 2M+Na ; 2M+Na ; 2M+Na ; 2M+Na ; 2M+Na ; 2M+Na ; 2M+Na ; 2M+Na	C20H18O2 ; C20H18O2 ; C20H18O2 ; C18H20O2 ; C32H26N4O2 ; C9H10O ; C9H10O ; C9H10O ; C9H10O ; C9H10O ; C9H10O ; C9H10O ; C9H10O ; C9H10O ; C9H10O ; C9H10O ; C9H10O ; C9H10O ; C9H10O ; C9H10O
X39	7	355.038	0	Unknow	Unknow	Unknow	Unknow
X398	7	271.067	0	HMDB15229 ; HMDB60945 ; HMDB01128 ; HMDB04185 ; HMDB60537 ; HMDB60826	Leflunomide ; A771726 ; 5-Phosphoribosylamine ; 5-Hydroxyindoleacetylglycine ; p-Hydroxyphenobarbital ; Hydroxynalidixic acid	M+H ; M+H ; M+ACN+H ; M+Na ; M+Na ; M+Na	C12H9F3N2O2 ; C12H9F3N2O2 ; C5H12NO7P ; C12H12N2O4 ; C12H12N2O4 ; C12H12N2O4
X41	7	370.969	0	Unknow	Unknow	Unknow	Unknow
X416	7	231.382	0	Unknow	Unknow	Unknow	Unknow
X422	7	315.08	0	HMDB05033 ; HMDB00709 ; HMDB01241 ; HMDB14582 ; HMDB15109 ; HMDB35145 ; HMDB59808	Valdecoxib ; L-Cysteinylglycine disulfide ; 2-Aminomuconic acid ; Ceftazidime ; Edetic Acid ; Chrycolide ; Acetyl citrate	M+H ; M+NH4 ; 2M+H ; M+2ACN+2H ; M+Na ; M+2ACN+H ; M+2ACN+H	C16H14N2O3S ; C8H15N3O5S2 ; C6H7NO4 ; C22H22N6O7S2 ; C10H16N2O8 ; C12H8O3S ; C8H8O8
X427	7	352.064	0	HMDB29176 ; HMDB29182 ; HMDB29184 ; HMDB05790 ; HMDB30159 ; HMDB31778 ; HMDB41919 ; HMDB61161	3-O-Methyl-(-)-epicatechin-5-O-sulphate ; 4-O-Methyl-(-)-epicatechin-5-O-sulphate ; 4-O-Methyl-(-)-epicatechin-7-O-sulphate ; Tannin ; 3345678-Heptahydroxyflavone ; Diflubenzuron ; Lormetazepam ; N-Desalkyl flurazepam	M+H ; M+H ; M+H ; M+2ACN+2H ; M+NH4 ; M+ACN+H ; M+NH4 ; M+ACN+Na	C16H15O7S ; C16H15O7S ; C16H15O7S ; C26H20O18 ; C15H10O9 ; C14H9ClF2N2O2 ; C16H12Cl2N2O2 ; C15H10ClFN2O
X43	7	147.121	0	Unknow	Unknow	Unknow	Unknow
X448	7	391.12	0	HMDB34223 ; HMDB35608 ; HMDB14590 ; HMDB14815	23456-Penta-O-acetyl-D-glucose ; Monotropein ; Loracarbef ; Isoflurophate	M+H ; M+H ; M+ACN+H ; 2M+Na	C16H22O11 ; C16H22O11 ; C16H16ClN3O4 ; C6H14FO3P
X45	7	658.553	0	HMDB10367	CE(14:1(9Z))	M+ACN+Na	C41H70O2
X456	7	575.501	0	Unknow	Unknow	Unknow	Unknow
X459	7	225.379	0	Unknow	Unknow	Unknow	Unknow
X46	7	357.075	0	HMDB00401 ; HMDB02032 ; HMDB14975 ; HMDB15201 ; HMDB15403 ; HMDB29200 ; HMDB41748	28-Dihydroxyadenine ; 8-Hydroxyguanine ; Progabide ; Clonazepam ; Lumiracoxib ; Ferulic acid 4-sulfate ; Isoferulic acid 3-sulfate	2M+Na ; 2M+Na ; M+Na ; M+ACN+H ; M+ACN+Na ; M+2ACN+H ; M+2ACN+H	C5H5N5O2 ; C5H5N5O2 ; C17H16ClFN2O2 ; C15H10ClN3O3 ; C15H13ClFNO2 ; C10H10O7S ; C10H10O7S

X468	7	274.103	0	HMDB13070 ; HMDB15155 ; HMDB29187 ; HMDB29233 ; HMDB29616 ; HMDB33798 ; HMDB34047 ; HMDB36199 ; HMDB39428 ; HMDB41406 ; HMDB41900 ; HMDB60737	Sinapyl alcohol ; Isosorbide Mononitrate ; 5-(35)-Dihydroxyphenyl-gamma-valerolactone ; 34-Dihydroxyphenylvaleric acid ; Diazenedicarboxamide ; 3-Methyl-1-(246-trihydroxyphenyl)-1-butanone ; 2-Hydroxy-46-dimethoxy-3-methylacetophenone ; 2-Methoxy-4-(4-methyl-13-dioxolan-2-yl)phenol ; 2-Methoxy-3-(4-methoxyphenyl)propanoic acid ; Bancroftinone ; gamma-Carboxyglutamic acid ; 3-(4-Hydroxy-3-methoxyphenyl)-2-methylpropionic acid	M+ACN+Na ; M+2ACN+H ; M+ACN+Na ; M+ACN+Na ; 2M+ACN+H ; M+ACN+Na ; M+ACN+Na ; M+ACN+Na ; M+ACN+Na ; M+ACN+Na ; M+2ACN+H ; M+ACN+Na	C11H14O4 ; C6H9NO6 ; C11H14O4 ; C11H14O4 ; C2H4N4O2 ; C11H14O4 ; C11H14O4 ; C11H14O4 ; C11H14O4 ; C11H14O4 ; C6H9NO6 ; C11H14O4
X49	7	175.144	0	HMDB13287 ; HMDB01080 ; HMDB14447 ; HMDB31215 ; HMDB31581 ; HMDB32712 ; HMDB33433 ; HMDB33870 ; HMDB41218 ; HMDB60247	NeNe dimethyllysine ; 4-Aminobutyraldehyde ; Tranexamic Acid ; N-Ethylacetamide ; Morpholine ; 14-Undecadiene ; (S)-Homostachydrine ; Butyramide ; (Z)-15-Undecadiene ; 4-Ammoniobutanal	M+H ; 2M+H ; M+NH4 ; 2M+H ; 2M+H ; M+Na ; M+NH4 ; 2M+H ; M+Na ; 2M+H	C8H18N2O2 ; C4H9NO ; C8H15NO2 ; C4H9NO ; C4H9NO ; C11H20 ; C8H15NO2 ; C4H9NO ; C11H20 ; C4H9NO
X50	7	290.135	0	HMDB34061 ; HMDB00985 ; HMDB29939 ; HMDB40705 ; HMDB59729	Withaperuvin H ; Dihydrolipoamide ; 6-Amino-9H-purine-9-propanoic acid ; Allixin ; 34-Methylenesebacic acid	M+2H ; M+2ACN+H ; M+2ACN+H ; M+ACN+Na ; M+ACN+Na	C30H42O9S ; C8H17NOS2 ; C8H9N5O2 ; C12H18O4 ; C12H18O4
X509	7	366.14	0.709	HMDB02278 ; HMDB02282 ; HMDB12492 ; HMDB00321 ; HMDB00345 ; HMDB00355 ; HMDB00368 ; HMDB00640 ; HMDB15527 ; HMDB29934 ; HMDB32224 ; HMDB32873 ; HMDB34426 ; HMDB40985 ; HMDB41561 ; HMDB59758	2-(acetylamino)-15-anhydro-2-deoxy-3-O-b-D-galactopyranosyl-D-arabino-Hex-1-enitol ; 2-(acetylamino)-15-anhydro-2-deoxy-4-O-b-D-galactopyranosyl-D-arabino-Hex-1-enitol ; 1-(12345-Pentahydroxypent-1-yl)-1234-tetrahydro-beta-carboline-3-carboxylate ; 2-Hydroxyadipic acid ; 3-Hydroxyadipic acid ; 3-Hydroxymethylglutaric acid ; 2(R)-Hydroxyadipic acid ; Levoglucosan ; Prazepam ; D-1-Deoxy-erythro-hexo-23-diulose ; N1-(24-Dimethoxybenzyl)-n2-(2-(pyridin-2-yl) ethyl)oxalamide ; Diethyl dicarbonate ; (-)-1-Methylpropyl 1-propenyl disulfide ; Polixetonium chloride ; D-15-Anhydrofructose ; 2-Hydroxy-2-ethylsuccinic acid	M+H ; M+H ; M+H ; 2M+ACN+H ; 2M+ACN+H ; 2M+ACN+H ; 2M+ACN+H ; 2M+ACN+H ; M+ACN+H ; 2M+ACN+H ; M+Na ; 2M+ACN+H ; 2M+ACN+H ; M+ACN+H ; 2M+ACN+H ; 2M+ACN+H	C14H23NO10 ; C14H23NO10 ; C17H21N2O7 ; C6H10O5 ; C6H10O5 ; C6H10O5 ; C6H10O5 ; C6H10O5 ; C19H17ClN2O ; C6H10O5 ; C18H21N3O4 ; C6H10O5 ; C7H14S2 ; C10H28Cl2N2OP2 ; C6H10O5 ; C6H10O5
X51	7	374.068	0	Unknow	Unknow	Unknow	Unknow
X517	7	157.519	0.758	HMDB14543	Zoledronate	M+ACN+2H	C5H10N2O7P2
X525	7	179.03	0.782	Unknow	Unknow	Unknow	Unknow
X531	7	372.283	0.739	Unknow	Unknow	Unknow	Unknow
X532	7	397.271	0.768	HMDB00308 ; HMDB32995 ; HMDB38522 ; HMDB41453	3b-Hydroxy-5-cholenoic acid ; 2-Undecyl-4(1H)-quinolinone N-oxide ; 2-(10-Heptadecenyl)-6-hydroxybenzoic acid ; D8-Merulinic acid C	M+Na ; M+2ACN+H ; M+Na ; M+Na	C24H38O3 ; C20H28NO2 ; C24H38O3 ; C24H38O3
X533	7	133.561	0.686	HMDB30348 ; HMDB37594 ; HMDB40597 ; HMDB40694 ; HMDB14350 ; HMDB32584 ; HMDB60321	Anonaine ; Eduleine ; Eduline ; Agaritinal ; Pyrimethamine ; 4-Hydroxychalcone ; 1-Hydroxy-6-methoxypyrene	M+2H ; M+2H ; M+2H ; M+2H ; M+H+NH4 ; M+ACN+2H ; M+H+NH4	C17H15NO2 ; C17H15NO2 ; C17H15NO2 ; C12H15N3O4 ; C12H13ClN4 ; C15H12O2 ; C17H12O2
X540	7	206.507	0.664	Unknow	Unknow	Unknow	Unknow
X543	7	158.516	0.712	Unknow	Unknow	Unknow	Unknow
X547	7	118.061	0.655	HMDB00128 ; HMDB15140 ; HMDB28686 ; HMDB28812	Guanidoacetic acid ; Hydroxyurea ; Alanyl-Glutamate ; Glutamyl-Alanine	M+H ; M+ACN+H ; M+H+NH4 ; M+H+NH4	C3H7N3O2 ; CH4N2O2 ; C8H13N2O5 ; C8H13N2O5
X55	7	630.087	0	Unknow	Unknow	Unknow	Unknow
X561	7	530.218	0.374	HMDB14850	Flurbiprofen	2M+ACN+H	C15H13FO2

X564	7	533.277	0.559	HMDB35483 ; HMDB38312 ; HMDB38615 ; HMDB39397 ; HMDB39482	Tuberoside C (Allium tuberosum) ; Balanitoside ; Trigofoenoside F ; Tuberoside L ; Trigofoenoside D	M+2H ; M+2H ; M+2H ; M+2H ; M+2H	C51H84O23 ; C51H84O23 ; C51H84O23 ; C51H84O23 ; C51H84O23
X574	7	269.055	0.002	HMDB34035 ; HMDB00362 ; HMDB00807 ; HMDB01228 ; HMDB01488 ; HMDB02243 ; HMDB03391 ; HMDB06039 ; HMDB14467 ; HMDB14929 ; HMDB31713 ; HMDB41950 ; HMDB60180 ; HMDB60406 ; HMDB60665	Sagecoumarin ; 2-Phosphoglyceric acid ; 3-Phosphoglyceric acid ; L-Glutamic acid 5-phosphate ; Nicotinic acid ; Picolinic acid ; 2-Phospho-D-glyceric acid ; 8-Chloroxanthine ; Floxuridine ; Uracil mustard ; 2-Hydroxy-4-imino-25-cyclohexadienone ; Nitrobenzene ; (2R)-2-Hydroxy-3-(phosphonatooxy)propanoate ; 5-Deoxy-5-fluorouridine ; Isonicotinic acid	M+2H ; M+2ACN+H ; M+2ACN+H ; M+ACN+H ; 2M+Na ; 2M+Na ; M+2ACN+H ; M+2ACN+H ; M+Na ; M+NH4 ; 2M+Na ; 2M+Na ; M+2ACN+H ; M+Na ; 2M+Na	C27H20O12 ; C3H7O7P ; C3H7O7P ; C5H10NO7P ; C6H5NO2 ; C6H5NO2 ; C3H7O7P ; C5H3ClN4O2 ; C9H11FN2O5 ; C8H11Cl2N3O2 ; C6H5NO2 ; C6H5NO2 ; C3H7O7P ; C9H11FN2O5 ; C6H5NO2
X581	7	290.034	0.222	Unknow	Unknow	Unknow	Unknow
X582	7	411.251	0.142	HMDB07849 ; HMDB07853 ; HMDB14018 ; HMDB15297 ; HMDB30366 ; HMDB31840 ; HMDB32567 ; HMDB34450 ; HMDB34579 ; HMDB35126 ; HMDB35375 ; HMDB35376 ; HMDB35801 ; HMDB37018 ; HMDB37231 ; HMDB37389 ; HMDB38046 ; HMDB38292 ; HMDB39685 ; HMDB40439 ; HMDB41497 ; HMDB41610 ; HMDB61062	LPA(0:016:0) ; LPA(16:00:0) ; 4-Hydroxypropofol ; Cilostazol ; Cavipetin C ; (SZ)-Lyratol acetate ; Hexylresorcinol ; Neocnidilide ; 8-O-Methyloblongine ; ()-Myrtenyl acetate ; Ethyl (2E4E7Z)-Decatrienoate ; Ethyl (2E4Z7Z)-Decatrienoate ; (2S4R)-p-Mentha-1(7)5-dien-2-ol acetate ; (S)-p-Mentha-18-dien-10-yl acetate ; Perillyl acetate ; (R)-2-Acetoxy-p-mentha-18-diene ; Carvyl acetate ; Menthadienyl acetate ; 8-Ocimenyl acetate ; 1-(2-Furanyl)-1-octanone ; (S)-Neolyratyl acetate ; (22-Diethoxyethyl)benzene ; 7-hydroxygranisetron	M+H ; M+H ; 2M+Na ; M+ACN+H ; M+Na ; 2M+Na ; 2M+Na ; 2M+Na ; M+2ACN+H ; 2M+Na ; 2M+Na ; 2M+Na ; 2M+Na ; 2M+Na ; 2M+Na ; 2M+Na ; 2M+Na ; 2M+Na ; 2M+Na ; 2M+Na ; 2M+Na ; 2M+Na ; M+2ACN+H	C19H39O7P ; C19H39O7P ; C12H18O2 ; C20H27N5O2 ; C24H36O4 ; C12H18O2 ; C12H18O2 ; C12H18O2 ; C20H26NO3 ; C12H18O2 ; C12H18O2 ; C12H18O2 ; C12H18O2 ; C12H18O2 ; C12H18O2 ; C12H18O2 ; C12H18O2 ; C12H18O2 ; C12H18O2 ; C12H18O2 ; C12H18O2 ; C12H18O2 ; C18H24N4O2
X594	7	642.471	0.09	HMDB29957 ; HMDB29960 ; HMDB31887 ; HMDB32754 ; HMDB38037	Squamocin K ; Asimilobin ; Rollidecin C ; Isomurisolenin ; Corossolone	M+ACN+Na ; M+ACN+Na ; M+ACN+Na ; M+ACN+Na ; M+ACN+Na	C35H62O6 ; C35H62O6 ; C35H62O6 ; C35H62O6 ; C35H62O6
X595	7	274.06	0.251	HMDB39751	alpha-D-Galactopyranuronosyl-(1-4)-alpha-D-galactopyranuronosyl-(1-4)-D-galacturonic acid	M+2H	C18H26O19
X610	7	285.091	0.38	HMDB28787 ; HMDB29102 ; HMDB30135 ; HMDB60511 ; HMDB41668 ; HMDB60337	Cysteinyl-Tyrosine ; Tyrosyl-Cysteine ; Asticolorin C ; SN38 glucuronide ; 4-Methyl-(-)-epigallocatechin 3-(4-methyl-gallate) ; 16-Dimethoxypyrene	M+H ; M+H ; M+2H ; M+2H ; M+2ACN+2H ; M+Na	C12H16N2O4S ; C12H16N2O4S ; C33H28O9 ; C28H28N2O11 ; C24H22O11 ; C18H14O2
X621	7	229.59	0.438	Unknow	Unknow	Unknow	Unknow
X624	7	292.579	0.729	HMDB33762 ; HMDB33937 ; HMDB36739	Ginkgetin ; Isoginkgetin ; Yuccaol C	M+H+NH4 ; M+H+NH4 ; M+ACN+2H	C32H22O10 ; C32H22O10 ; C30H22O10
X629	7	232.173	0.264	HMDB34336 ; HMDB38499	Dolichosterone ; Polyporusterone F	M+2H ; M+2H	C28H46O5 ; C28H46O5
X633	7	239.164	0.371	HMDB38256 ; HMDB38496 ; HMDB38497 ; HMDB00387 ; HMDB02059 ; HMDB10728 ; HMDB12215 ; HMDB40169	Geranyl acetoacetate ; Polyporusterone B ; Polyporusterone C ; 3-Hydroxydodecanoic acid ; 12-Hydroxydodecanoic acid ; (R)-3-Hydroxydodecanoic acid ; Dihydrozeatin ; xi-5-Hydroxydodecanoic acid	M+H ; M+2H ; M+2H ; M+Na ; M+Na ; M+Na ; M+NH4 ; M+Na	C14H22O3 ; C28H44O6 ; C28H44O6 ; C12H24O3 ; C12H24O3 ; C12H24O3 ; C10H15N5O ; C12H24O3
X637	7	507.156	0.414	HMDB12470 ; HMDB32645	(2S6R10R)-Trimethyl-2E-hendecenoyl-CoA ; 3-Glucosyl-23446-pentahydroxybenzophenone	M+ACN+2H ; M+2ACN+H	C35H56N7O17P3S ; C19H20O11
X650	7	212.161	0.013	HMDB01921	11-Dimethylbiguanide	M+2ACN+H	C4H11N5
X655	7	284.59	0.044	HMDB29408	Betanin	M+H+NH4	C24H26N2O13
X656	7	167.57	0.156	Unknow	Unknow	Unknow	Unknow
X663	7	290.668	0.389	Unknow	Unknow	Unknow	Unknow
X665	7	326.918	0.264	Unknow	Unknow	Unknow	Unknow
X667	7	178.033	0.67	HMDB38591	Brassicanal A	M+H	C9H7NOS
X670	7	610.741	0.045	HMDB06611	Alpha-Heptasaccharide	M+H+NH4	C46H78N2O34

X674	7	228.102	0.413	HMDB33452 ; HMDB41444 ; HMDB00329 ; HMDB00543 ; HMDB01955 ; HMDB05802 ; HMDB05809 ; HMDB30060 ; HMDB30063 ; HMDB31761 ; HMDB32038 ; HMDB32336 ; HMDB32586 ; HMDB32618 ; HMDB32620 ; HMDB32708 ; HMDB32891 ; HMDB33723 ; HMDB33945 ; HMDB34471 ; HMDB34615 ; HMDB34732 ; HMDB35213 ; HMDB35268 ; HMDB35414 ; HMDB36098 ; HMDB37160 ; HMDB37174 ; HMDB38184 ; HMDB40046 ; HMDB40359 ; HMDB40460 ; HMDB41472 ; HMDB41473 ; HMDB41612	Koenoline ; Mukoline ; 2-Phenylbutyric acid ; Benzenebutanoic acid ; 3-Phenylbutyric acid ; Isoeugenol ; Eugenol ; Methyl 3-phenylpropanoate ; Benzyl propionate ; Propyl benzoate ; Isopropyl benzoate ; 3-Hydroxy-4-phenylbutan-2-one ; 3-(4-Methoxyphenyl)-2-propen-1-ol ; Ethyl phenylacetate ; 1-Phenylethyl acetate ; 1-Naphthaleneacetic acid ; 1-(4-Methoxyphenyl)-2-propanone ; 4-(4-Hydroxyphenyl)-2-butanone ; 2-Phenylethyl acetate ; 3-Phenylpropyl formate ; Epoxyanethole ; Thymoquinone ; beta-Thujaplicin ; 4-Isopropylbenzoic acid ; Lepalone ; Isoegomaketone ; 4-Methyl-2-phenyl-13-dioxolane ; p-Mentha-24(8)-dien-93-olide ; Egomaketone ; 1-(23-Dihydro-1H-pyrrolizin-5-yl)-14-pentanedione ; 12-Dimethoxy-4-vinylbenzene ; 4-Methylbenzyl alcohol acetate ; 2-Methylbenzyl alcohol acetate ; (3-Methylphenyl)methyl acetate ; Phenyl butyrate	M+H ; M+H ; M+ACN+Na ; M+ACN+Na ; M+ACN+Na ; M+ACN+Na ; M+ACN+Na ; M+ACN+Na ; M+ACN+Na ; M+ACN+Na ; M+ACN+Na ; M+ACN+Na ; M+ACN+Na ; M+ACN+Na ; M+ACN+Na ; M+ACN+H ; M+ACN+Na ; M+ACN+Na ; M+ACN+Na ; M+ACN+Na ; M+ACN+Na ; M+ACN+Na ; M+ACN+Na ; M+ACN+Na ; M+ACN+Na ; M+ACN+Na ; M+ACN+Na ; M+ACN+Na ; M+ACN+Na ; M+Na ; M+ACN+Na ; M+ACN+Na ; M+ACN+Na ; M+ACN+Na ; M+ACN+Na	C14H13NO2 ; C14H13NO2 ; C10H12O2 ; C10H12O2 ; C10H12O2 ; C10H12O2 ; C10H12O2 ; C10H12O2 ; C10H12O2 ; C10H12O2 ; C10H12O2 ; C10H12O2 ; C10H12O2 ; C10H12O2 ; C10H12O2 ; C12H10O2 ; C10H12O2 ; C10H12O2 ; C10H12O2 ; C10H12O2 ; C10H12O2 ; C10H12O2 ; C10H12O2 ; C10H12O2 ; C10H12O2 ; C10H12O2 ; C10H12O2 ; C10H12O2 ; C10H12O2 ; C12H15NO2 ; C10H12O2 ; C10H12O2 ; C10H12O2 ; C10H12O2 ; C10H12O2
X685	7	343.312	0.383	Unknow	Unknow	Unknow	Unknow
X689	7	478.236	0.223	Unknow	Unknow	Unknow	Unknow
X693	7	171.99	0.553	Unknow	Unknow	Unknow	Unknow
X704	7	104.526	0.059	HMDB33136 ; HMDB03974 ; HMDB42052	2-Amino-4-hydroxy-6-pteridinecarboxylic acid ; Oxalosuccinic acid ; Trimethylselenonium	M+2H ; M+H+NH4 ; M+2ACN+2H	C7H5N5O3 ; C6H6O7 ; C3H9Se
X705	7	177.123	0.434	HMDB00339 ; HMDB00678 ; HMDB00927 ; HMDB04043 ; HMDB04472 ; HMDB05812 ; HMDB11757 ; HMDB12154 ; HMDB12175 ; HMDB29230 ; HMDB29409 ; HMDB29412 ; HMDB30999 ; HMDB31345 ; HMDB31410 ; HMDB32118 ; HMDB32530 ; HMDB34848 ; HMDB34932 ; HMDB34969 ; HMDB34972 ; HMDB34976 ; HMDB35155 ; HMDB35162 ; HMDB35272 ; HMDB35783 ; HMDB35815 ; HMDB35818 ; HMDB35819 ; HMDB35820 ; HMDB35823 ; HMDB35825 ; HMDB35833 ; HMDB35838 ; HMDB35841 ; HMDB35842 ; HMDB35861 ; HMDB35954 ; HMDB35955 ; HMDB36041 ; HMDB36068 ; HMDB36077 ; HMDB36078 ; HMDB36086 ; HMDB36096 ; HMDB36097 ; HMDB36100 ; HMDB36101 ; HMDB36102 ; HMDB36107 ; HMDB36112 ; HMDB36126 ; HMDB36196 ; HMDB36384 ;	2-Methylbutyrylglycine ; Isovalerylglycine ; Valerylglycine ; Alpha-Terpineol ; Eucalyptol ; Geraniol ; N-Acetylvaline ; 3-Dehydrocarnitine ; 5-Acetamidovalerate ; 4-Hydroxystachydrine ; Turicine ; Betonicine ; 2-Decenal ; Calystegine A6 ; 3-Decen-2-one ; 26-Dimethyl-7-octen-3-one ; Thujyl alcohol ; 9-Decenal ; Fenchol ; (-)-Carvomenthone ; ()-Isomenthone ; (-)-Borneol ; beta-Geraniol ; (-)-Menthone ; p-Menthan-2-one ; ()-Menthone ; ()-Isoborneol ; ()-Borneol ; (-)-Isoborneol ; (R)-Citronellal ; (S)-p-Menth-1-en-4-ol ; (-)-Dihydrocarveol ; p-Menth-1-en-4-ol ; Piperitol ; Citronellal ; (S)-Citronellal ; trans-(-)-p-Menth-1-en-3-ol ; (1R3R6S)-3-Caranol ; (1R2S3S6S)-2-Caranol ; (R)-Lavandulol ; Campholenic alcohol ; p-Menth-8-en-3-ol ; (-)-Isopulegol ; (S)-alpha-Terpineol ; 14-Cineole ; Rose oxide ; 37-Dimethyl-16-octadien-3-ol ; (R)-37-Dimethyl-16-octadien-3-ol ; D-Linalool ; Myrcenol ; (1R4S5R)-4-Thujanol ; (-)-Pinocampheol ; (2E4E)-24-Decadien-1-ol ; Calystegine A7 ;	M+NH4 ; M+NH4 ; M+NH4 ; M+Na ; M+Na ; M+Na ; M+NH4 ; M+NH4 ; M+NH4 ; M+NH4 ; M+NH4 ; M+NH4 ; M+Na ; M+NH4 ; M+Na ; M+NH4 ; M+Na ; M+Na ; M+Na ; M+Na ; M+Na ; M+Na ; M+Na ; M+Na ; M+Na ; M+Na ; M+Na ; M+Na ; M+Na ; M+Na ; M+Na ; M+Na ; M+NH4 ; M+NH4 ; M+Na ; M+Na ; M+Na ; M+Na ; M+Na ; M+Na	C7H13NO3 ; C7H13NO3 ; C7H13NO3 ; C10H18O ; C10H18O ; C10H18O ; C7H13NO3 ; C7H13NO3 ; C7H13NO3 ; C7H13NO3 ; C7H13NO3 ; C7H13NO3 ; C10H18O ; C7H13NO3 ; C10H18O ; C7H13NO3 ; C10H18O ; C10H18O ; C10H18O ; C10H18O ; C10H18O ; C10H18O ; C10H18O ; C10H18O ; C10H18O ; C10H18O ; C10H18O ; C10H18O ; C10H18O ; C10H18O ; C10H18O ; C10H18O ; C7H13NO3 ; C7H13NO3 ; C10H18O ; C10H18O ; C10H18O ; C10H18O ; C10H18O ; C10H18O

X709	7	224.128	0.018	HMDB15168 ; HMDB13845 ; HMDB12550 ; HMDB29456 ; HMDB30762 ; HMDB30899 ; HMDB31729 ; HMDB31973 ; HMDB32348 ; HMDB32607 ; HMDB33394 ; HMDB33594 ; HMDB34122 ; HMDB34135 ; HMDB35071 ; HMDB35168 ; HMDB36522 ; HMDB36665 ; HMDB36666 ; HMDB36944 ; HMDB37641 ; HMDB37727 ; HMDB38181 ; HMDB38354 ; HMDB38746 ; HMDB39874 ; HMDB40757 ; HMDB40758 ; HMDB41226 ; HMDB41279 ; HMDB41348 ; HMDB41410 ; HMDB41496 ; HMDB59891 ; HMDB59992	Cerulenin ; SR 49498 ; 12-Oxo-20-carboxy-leukotriene B4 ; Amyl 2-furoate ; Ethyl trans-p-methoxycinnamate ; Eremopetasitenin A1 ; Phenylmethylglycidic ester ; (2RS5RS)-(E)-2-(2-Phenylethenyl)-13-dioxan-5-ol ; Isoeugenyl acetate ; ()-threo-Anethole glycol ; 4-Ethyl-26-dimethoxyphenol ; Cuelure ; Aceteugenol ; Isoeugenol acetate ; Verimol I ; Cinncassiol C2 ; Verimol J ; Blumealactone A ; Blumealactone B ; Gibberellin A123 ; 4-(Ethoxymethyl)-2-methoxyphenol ; Furfuryl pentanoate ; Peperinic acid ; 2-Ethoxy-4-(methoxymethyl)phenol ; Gibberellin A74 ; Furfuryl isovalerate ; 3b8a-Dihydroxy-6b-angeloyloxy-7(11)-eremophilen-128-olide ; 3b8b-Dihydroxy-6b-angeloyloxy-7(11)-eremophilen-128-olide ; 3b9b-Dihydroxy-6b-angeloyloxy-7(11)-eremophilen-128b-olide ; 3b-Hydroxy-6b-(23-epoxy-2-methylbutanoyloxy)-7(11)-eremophilen-128b-olide ; Gibberellin A100 ; 23-Dihydro-6-[illegible]	M+H ; M+2H ; M+2ACN+2H ; M+ACN+H ; M+NH4 ; M+2ACN+2H ; M+NH4 ; M+NH4 ; M+NH4 ; M+ACN+H ; M+ACN+H ; M+NH4 ; M+NH4 ; M+NH4 ; M+NH4 ; M+2ACN+2H ; M+ACN+H ; M+2ACN+2H ; M+2ACN+2H ; M+2ACN+2H ; M+ACN+H ; M+ACN+H ; M+ACN+H ; M+ACN+H ; M+2ACN+2H ; M+ACN+H ; M+2ACN+2H ; M+2ACN+2H ; M+2ACN+2H ; M+2ACN+2H ; M+2ACN+2H ; M+NH4 ; M+2ACN+2H ; M+ACN+H ; M+2ACN+2H	C12H17NO3 ; C25H30N6O2 ; C20H28O6 ; C10H14O3 ; C12H14O3 ; C20H28O6 ; C12H14O3 ; C12H14O3 ; C12H14O3 ; C10H14O3 ; C10H14O3 ; C12H14O3 ; C12H14O3 ; C12H14O3 ; C12H14O3 ; C20H28O6 ; C10H14O3 ; C20H28O6 ; C20H28O6 ; C20H28O6 ; C10H14O3 ; C10H14O3 ; C10H14O3 ; C10H14O3 ; C20H28O6 ; C10H14O3 ; C20H28O6 ; C20H28O6 ; C20H28O6 ; C20H28O6 ; C20H28O6 ; C12H14O3 ; C20H28O6 ; C10H14O3 ; C20H28O6
X71	7	146.118	0	HMDB06831 ; HMDB14347 ; HMDB13238 ; HMDB29165 ; HMDB29314 ; HMDB30330 ; HMDB31174 ; HMDB31342 ; HMDB31351 ; HMDB31476 ; HMDB31477 ; HMDB31484 ; HMDB31500 ; HMDB31504 ; HMDB31593 ; HMDB31603 ; HMDB31681 ; HMDB32391 ; HMDB32460 ; HMDB33793 ; HMDB38271 ; HMDB38962 ; HMDB39819 ; HMDB40214 ; HMDB40591	3-Dehydroxycarnitine ; Succinylcholine ; Heptanoylcarnitine ; 13-Diacetylpropane ; Isopentenyl acetate ; 1-Methoxy-3-methylene-2-pentanone ; 4-Methyl-5-hexanolide ; Cyclohexanecarboxylic acid ; Cyclohexyl formate ; 23-Heptanedione ; 34-Heptanedione ; 2-Heptenoic acid ; Methyl 2E-hexenoate ; Methyl (Z)-3-hexenoate ; 5-Methyl-23-hexanedione ; Ethyl 4-pentenoate ; Dihydro-5-propyl-2(3H)-furanone ; 3-Methyl-3-butenyl acetate ; 4-Pentenyl acetate ; 4-Heptenoic acid ; 2-Hexenyl formate ; Ethyl tiglate ; 24-Dimethyl-2-pentenoic acid ; cis-3-Hexenyl formate ; Allyl butyrate	M+H ; M+2H ; M+H+NH4 ; M+NH4	C7H15NO2 ; C14H30N2O4 ; C14H27NO4 ; C7H12O2
X718	7	506.657	0.173	Unknow	Unknow	Unknow	Unknow
X730	7	111.068	0.739	HMDB01189 ; HMDB02096 ; HMDB05199 ; HMDB15611 ; HMDB29223 ; HMDB29224 ; HMDB30372 ; HMDB31811 ; HMDB33763 ; HMDB39655 ; HMDB39656 ; HMDB40021 ; HMDB41526 ; HMDB41931 ; HMDB42012	2(N)-Methyl-norsalsolinol ; 3-Indolebutyric acid ; (R)-Salsolinol ; Methsuximide ; alpha-Methylphenylalanine ; N-methylphenylalanine ; Maltoxazine ; 35-Dimethylphenyl methylcarbamate ; Ethyl N-methylanthranilate ; 123456-Hexahydro-5-(1-hydroxyethylidene)-7H-cyclopentabpyridin-7-one ; 5-(2-Furanyl)-123456-hexahydro-7H-cyclopentabpyridin-7-one ; 23-Dihydro-5-(3-hydroxypropanoyl)-1H-pyrrolizine ; Isosalsolidine ; 34-Methylenedioxyamphetamine ; Salsolinol	M+ACN+2H ; M+H+NH4 ; M+ACN+2H ; M+H+NH4 ; M+ACN+2H ; M+ACN+2H ; M+ACN+2H ; M+ACN+2H ; M+ACN+2H ; M+ACN+2H ; M+H+NH4 ; M+ACN+2H ; M+H+NH4 ; M+ACN+2H ; M+ACN+2H	C10H13NO2 ; C12H13NO2 ; C10H13NO2 ; C12H13NO2 ; C10H13NO2 ; C10H13NO2 ; C10H13NO2 ; C10H13NO2 ; C10H13NO2 ; C10H13NO2 ; C12H13NO2 ; C10H13NO2 ; C12H13NO2 ; C10H13NO2 ; C10H13NO2
X742	7	498.684	0.021	Unknow	Unknow	Unknow	Unknow

X743	7	354.336	0.218	HMDB10372 ; HMDB00417 ; HMDB00553 ; HMDB00801 ; HMDB02212 ; HMDB03352 ; HMDB11623 ; HMDB13810 ; HMDB31318 ; HMDB31409 ; HMDB32206 ; HMDB32212 ; HMDB32241 ; HMDB32274 ; HMDB32532 ; HMDB34156 ; HMDB34717 ; HMDB35093 ; HMDB35094 ; HMDB35672 ; HMDB35673 ; HMDB35726 ; HMDB35763 ; HMDB35764 ; HMDB35765 ; HMDB37020 ; HMDB37171 ; HMDB37223 ; HMDB37806 ; HMDB39854 ; HMDB41628 ; HMDB59861	CE(22:1(13Z)) ; 3D7D11D-Phytanic acid ; 3L7D11D-Phytanic acid ; Phytanic acid ; Arachidic acid ; Menthol ; Decanal ; (E)-3-decen-1-ol ; 2261014-Pentamethylpentadecanoic acid ; 2-Decanone ; cis-4-Decenol ; 3-Decanone ; 37-Dimethyloctanal ; (-)-4-Ethyloctanal ; 2-DECENOL ; Ethyl stearate ; Menthanol ; D-Citronellol ; L-Citronellol ; 5-Hydroxy-7-eicosanone ; 4-Hydroxy-6-eicosanone ; p-Menthan-4-ol ; ()-Neomenthol ; (-)-Neoisomenthol ; p-Menthan-3-ol ; p-Menthan-1-ol ; alpha-Citronellol ; ()-Carvomenthol ; Dihydrocitronellal ; 1-Decen-3-ol ; ()-Neoisomenthol ; 9-Decenol	M+2H ; M+ACN+H ; M+ACN+H ; M+ACN+H ; M+ACN+H ; 2M+ACN+H ; 2M+ACN+H ; 2M+ACN+H ; M+ACN+H ; 2M+ACN+H ; 2M+ACN+H ; 2M+ACN+H ; 2M+ACN+H ; 2M+ACN+H ; 2M+ACN+H ; M+ACN+H ; 2M+ACN+H ; 2M+ACN+H ; 2M+ACN+H ; M+ACN+H ; M+ACN+H ; 2M+ACN+H ; 2M+ACN+H ; 2M+ACN+H ; 2M+ACN+H ; 2M+ACN+H ; 2M+ACN+H ; 2M+ACN+H ; 2M+ACN+H ; 2M+ACN+H ; 2M+ACN+H ; 2M+ACN+H	C49H86O2 ; C20H40O2 ; C20H40O2 ; C20H40O2 ; C20H40O2 ; C10H20O ; C10H20O ; C10H20O ; C20H40O2 ; C10H20O ; C10H20O ; C10H20O ; C10H20O ; C10H20O ; C10H20O ; C20H40O2 ; C10H20O ; C10H20O ; C10H20O ; C20H40O2 ; C20H40O2 ; C10H20O ; C10H20O ; C10H20O ; C10H20O ; C10H20O ; C10H20O ; C10H20O ; C10H20O ; C10H20O ; C10H20O ; C10H20O
X754	7	381.298	0.031	HMDB11545 ; HMDB11546 ; HMDB11547 ; HMDB11575 ; HMDB11576 ; HMDB11577 ; HMDB32735 ; HMDB39403 ; HMDB41103 ; HMDB11131 ; HMDB11535 ; HMDB31075 ; HMDB56295 ; HMDB56320	MG(0:020:3(11Z14Z17Z)0:0) ; MG(0:020:3(5Z8Z11Z)0:0) ; MG(0:020:3(8Z11Z14Z)0:0) ; MG(20:3(11Z14Z17Z)0:00:0) ; MG(20:3(5Z8Z11Z)0:00:0) ; MG(20:3(8Z11Z14Z)0:00:0) ; Isopersin ; 2-Hydroxy-4-oxo-512-heneicosadien-1-yl acetate ; Persin ; MG(18:00:00:0) ; MG(0:018:00:0) ; Glycerol 1-octadecanoate ; DG(18:2n60:022:6n3) ; DG(20:2n60:022:6n3)	M+H ; M+H ; M+H ; M+H ; M+H ; M+H ; M+H ; M+H ; M+H ; M+Na ; M+Na ; M+Na ; M+2ACN+2H ; M+2ACN+2H	C23H40O4 ; C23H40O4 ; C23H40O4 ; C23H40O4 ; C23H40O4 ; C23H40O4 ; C23H40O4 ; C23H40O4 ; C23H40O4 ; C21H42O4 ; C21H42O4 ; C21H42O4 ; C44H70O5 ; C44H70O5
X755	7	444.719	0.031	Unknow	Unknow	Unknow	Unknow
X756	7	247.101	0.009	HMDB34145 ; HMDB35828 ; HMDB39048	(-)-cis-Rotenolone ; Lactupicrin ; (5a6a8a11a)-8-Hydroxy-2-oxo-1(10)3-guaiadien-126-olide-15-al 8-(4-hydroxyphenylacetate)	M+2ACN+2H ; M+2ACN+2H ; M+2ACN+2H	C23H22O7 ; C23H22O7 ; C23H22O7
X759	7	256.905	0.371	Unknow	Unknow	Unknow	Unknow
X789	7	337.214	0.505	HMDB15324 ; HMDB01830 ; HMDB13121 ; HMDB14613 ; HMDB14746 ; HMDB31924 ; HMDB38533 ; HMDB41865	Acebutolol ; Progesterone ; 7-Dehydropregnenolone ; Dronabinol ; Chloroquine ; 4-Nerolidylcatechol ; Cardoltriene ; delta9-Tetrahydrocannabinol	M+H ; M+Na ; M+Na ; M+Na ; M+NH4 ; M+Na ; M+Na ; M+Na	C18H28N2O4 ; C21H30O2 ; C21H30O2 ; C21H30O2 ; C18H26ClN3 ; C21H30O2 ; C21H30O2 ; C21H30O2
X80	7	617.317	0	HMDB14950	Phenylbutazone	2M+H	C19H20N2O2
X801	7	217.195	0.009	HMDB36578 ; HMDB34731	Cyperotundone ; Tomatidine	M+H ; M+H+NH4	C16H24 ; C27H45NO2
X802	7	383.316	0.615	HMDB11544 ; HMDB11574 ; HMDB35959 ; HMDB36865	MG(0:020:2(11Z14Z)0:0) ; MG(20:2(11Z14Z)0:00:0) ; Persenone B ; Lepidiumterpenyl ester	M+H ; M+H ; M+H ; M+H	C23H42O4 ; C23H42O4 ; C23H42O4 ; C23H42O4
X805	7	561.284	0.002	HMDB37924 ; HMDB06765 ; HMDB10315 ; HMDB32423	Tragopogonsaponin J ; 2-Methoxy-estradiol-17b 3-glucuronide ; 4-Hydroxyandrostenedione glucuronide ; 2-(4-Methyl-5-thiazolyl)ethyl octanoate	M+2H ; M+2ACN+H ; M+2ACN+H ; 2M+Na	C57H84O22 ; C25H34O9 ; C25H34O9 ; C14H23NO2S
X820	7	178.098	0.067	HMDB41940 ; HMDB31877 ; HMDB03152 ; HMDB13751 ; HMDB30001 ; HMDB33947 ; HMDB36057 ; HMDB37819 ; HMDB59711	N-nitrosonornicotine ; Acetylsalvipisone ; N-Methylnicotinamide ; 2-Hydroxypyridine ; 2-Acetyl-3-methylpyrazine ; 2-Aminobenzamide ; 1H-Pyrrole-2-carboxaldehyde ; 4-Acetyl-2-methylpyrimidine ; 2-Methylnicotinamide	M+H ; M+2H ; M+ACN+H ; M+2ACN+H ; M+ACN+H ; M+ACN+H ; M+2ACN+H ; M+ACN+H ; M+ACN+H	C9H11N3O ; C22H26O4 ; C7H8N2O ; C5H5NO ; C7H8N2O ; C7H8N2O ; C5H5NO ; C7H8N2O ; C7H8N2O
X832	7	156.051	0.227	HMDB40357	611-Dihydroxy-22-dimethylpyrano32-cxanthen-7(2H)-one	M+2H	C18H14O5

X844	7	176.128	0.082	HMDB00451 ; HMDB00909 ; HMDB10721 ; HMDB13211 ; HMDB30303 ; HMDB31177 ; HMDB31307 ; HMDB32478 ; HMDB36230 ; HMDB36395 ; HMDB38305 ; HMDB40447 ; HMDB41616 ; HMDB59938 ; HMDB59939 ; HMDB60683 ; HMDB60685	cis-4-Hydroxycyclohexylacetic acid ; trans-4-Hydroxycyclohexylacetic acid ; 3-Oxooctanoic acid ; Alpha-Ketooctanoic acid ; 6-Ethyl-1-methyl-278-trioxabicyclo321octane ; Tetrahydrofurfuryl propionate ; Ethyl 3-oxohexanoate ; Polypropylene glycol (m w 1200-3000) ; 1-Methyl-2-oxopropyl butyrate ; 2-Methylpropyl 3-oxobutanoate ; 3-Methylbutyl 2-oxopropanoate ; Butyl acetoacetate ; Propyl levulinate ; 5-Butyl-14-dioxan-2-one ; 6-Butyl-14-dioxan-2-one ; 2-n-Propyl-4-oxopentanoic acid ; 3-Oxovalproic acid	M+NH4 ; M+NH4 ; M+NH4 ; M+NH4 ; M+NH4 ; M+NH4 ; M+NH4 ; M+ACN+H ; M+NH4 ; M+NH4 ; M+NH4 ; M+NH4 ; M+NH4 ; M+NH4 ; M+NH4 ; M+NH4 ; M+NH4	C8H14O3 ; C8H14O3 ; C8H14O3 ; C8H14O3 ; C8H14O3 ; C8H14O3 ; C8H14O3 ; C6H14O3 ; C8H14O3 ; C8H14O3 ; C8H14O3 ; C8H14O3 ; C8H14O3 ; C8H14O3 ; C8H14O3 ; C8H14O3 ; C8H14O3
X862	7	563.33	0.381	HMDB01936 ; HMDB34441	Doxylamine ; Americine	2M+Na ; M+NH4	C17H22N2O ; C31H39N5O4
X872	7	267.078	0.007	HMDB00030	Biotin	M+Na	C10H16N2O3S
X874	7	335.198	0.486	HMDB00022 ; HMDB00995 ; HMDB02182 ; HMDB04626 ; HMDB04826 ; HMDB12162 ; HMDB14511 ; HMDB14522 ; HMDB14748 ; HMDB15165 ; HMDB15348 ; HMDB32020 ; HMDB33197 ; HMDB36047 ; HMDB38869 ; HMDB39837 ; HMDB41888 ; HMDB59661 ; HMDB60580 ; HMDB60592 ; HMDB60807	3-Methoxytyramine ; 16-Dehydroprogesterone ; Phenylephrine ; Tetrahydrogestrinone ; p-Synephrine ; 4-Methoxytyramine ; Levonorgestrel ; Dydrogesterone ; Metaraminol ; Ethinamate ; Anastrozole ; 4-(beta-Methylaminoethyl)catechol ; 2244-Tetramethyl-6-(1-oxobutyl)-135-cyclohexanetrione ; 3alpha-Hydroxyoreadone ; Norpandamarilactonine A ; 2-(2-Furanyl)-3-piperidinol ; Epinine ; QH(2) ; Ethisterone ; 4-Hydroxynorephedrine ; a-Methyldopamine	2M+H ; M+Na ; 2M+H ; M+Na ; 2M+H ; 2M+H ; M+Na ; M+Na ; 2M+H ; 2M+H ; M+ACN+H ; 2M+H ; M+2ACN+H ; M+2ACN+H ; 2M+H ; 2M+H ; 2M+H ; M+2ACN+H ; M+Na ; 2M+H ; 2M+H	C9H13NO2 ; C21H28O2 ; C9H13NO2 ; C21H28O2 ; C9H13NO2 ; C9H13NO2 ; C21H28O2 ; C21H28O2 ; C9H13NO2 ; C9H13NO2 ; C17H19N5 ; C9H13NO2 ; C14H20O4 ; C14H20O4 ; C9H13NO2 ; C9H13NO2 ; C9H13NO2 ; C14H20O4 ; C21H28O2 ; C9H13NO2 ; C9H13NO2
X884	7	764.55	0.007	HMDB12356 ; HMDB12376 ; HMDB05096 ; HMDB08029 ; HMDB08030 ; HMDB08159 ; HMDB10570 ; HMDB10575 ; HMDB10588 ; HMDB10589 ; HMDB10616 ; HMDB10631 ; HMDB10645 ; HMDB11211 ; HMDB11273 ; HMDB11306 ; HMDB13413 ; HMDB39849 ; HMDB56974 ; HMDB57001 ; HMDB57052 ; HMDB57075 ; HMDB57202 ; HMDB57461	PS(16:018:0) ; PS(18:016:0) ; N-Arachidonoyl glycine ; PC(16:1(9Z)P-18:1(11Z)) ; PC(16:1(9Z)P-18:1(9Z)) ; PC(18:2(9Z12Z)P-16:0) ; PG(16:016:0) ; PG(16:018:2(9Z12Z)) ; PG(16:1(9Z)18:1(11Z)) ; PG(16:1(9Z)18:1(9Z)) ; PG(18:1(11Z)16:1(9Z)) ; PG(18:1(9Z)16:1(9Z)) ; PG(18:2(9Z12Z)16:0) ; PC(P-16:018:2(9Z12Z)) ; PC(P-18:1(11Z)16:1(9Z)) ; PC(P-18:1(9Z)16:1(9Z)) ; PC(o-16:1(9Z)18:2(9Z12Z)) ; 3-Oxohexadecanoic acid glycerides ; CL(18:018:016:1(9Z)18:2(9Z12Z)) ; CL(18:018:018:2(9Z12Z)16:1(9Z)) ; CL(18:016:1(9Z)18:018:2(9Z12Z)) ; CL(18:016:1(9Z)18:2(9Z12Z)18:0) ; CL(18:018:2(9Z12Z)16:1(9Z)18:0) ; CL(16:1(9Z)18:018:2(9Z12Z)18:0)	M+H ; M+H ; 2M+ACN+H ; M+Na ; M+Na ; M+Na ; M+ACN+H ; M+NH4 ; M+NH4 ; M+NH4 ; M+NH4 ; M+NH4 ; M+NH4 ; M+Na ; M+Na ; M+Na ; M+Na ; 2M+ACN+H ; M+2ACN+2H ; M+2ACN+2H ; M+2ACN+2H ; M+2ACN+2H ; M+2ACN+2H ; M+2ACN+2H	C40H78NO10P ; C40H78NO10P ; C22H35NO3 ; C42H80NO7P ; C42H80NO7P ; C42H80NO7P ; C38H75O10P ; C40H75O10P ; C40H75O10P ; C40H75O10P ; C40H75O10P ; C40H75O10P ; C40H75O10P ; C42H80NO7P ; C42H80NO7P ; C42H80NO7P ; C42H80NO7P ; C19H37O6 ; C80H150O17P2 ; C80H150O17P2 ; C80H150O17P2 ; C80H150O17P2 ; C80H150O17P2 ; C80H150O17P2
X899	7	212.033	0.019	HMDB41173 ; HMDB00715 ; HMDB01553 ; HMDB60331 ; HMDB60332	1-O-Caffeoyl-(b-D-glucose 6-O-sulfate) ; Kynurenic acid ; 2-Oxo-4-methylthiobutanoic acid ; 1-Nitronaphthalene-56-oxide ; 1-Nitronaphthalene-78-oxide	M+2H ; M+Na ; M+ACN+Na ; M+Na ; M+Na	C15H18O12S ; C10H7NO3 ; C5H8O3S ; C10H7NO3 ; C10H7NO3
X913	7	560.311	0.053	HMDB31919 ; HMDB37530	Chaetoglobosin N ; 1-O-Acetylpaxilline	M+NH4 ; M+2ACN+H	C33H38N2O5 ; C29H35NO5
X92	7	187.144	0	HMDB33553 ; HMDB31544 ; HMDB38321	3-(3-Methylbutyl)nitrosoamino-2-butanone ; 3-Methylcyclopentene ; Homoarecoline	M+H ; 2M+Na ; M+NH4	C9H18N2O2 ; C6H10 ; C9H15NO2
X102	8	202.02	0	HMDB01274 ; HMDB37112	dTDP ; Acifluorfen	M+2H ; M+ACN+2H	C10H16N2O11P2 ; C14H7ClF3NO5
X105	8	148.524	0	Unknow	Unknow	Unknow	Unknow

X122	8	466.086	0	HMDB38410 ; HMDB15045	Glucohesperalin ; Zonisamide	M+H ; 2M+ACN+H	C14H27NO10S3 ; C8H8N2O3S
X14	8	262.078	0	HMDB28752 ; HMDB28815 ; HMDB33705 ; HMDB37336 ; HMDB15188	Aspartyl-Glutamate ; Glutamyl-Aspartate ; Salviaflaside ; Sudachiin A ; Mimosine	M+H ; M+H ; M+2H ; M+2H ; M+ACN+Na	C9H13N2O7 ; C9H13N2O7 ; C24H26O13 ; C24H26O13 ; C8H10N2O4
X141	8	387.264	0	HMDB00309 ; HMDB00322 ; HMDB00324 ; HMDB00352 ; HMDB00388 ; HMDB03956 ; HMDB04611 ; HMDB04624 ; HMDB06031 ; HMDB06259 ; HMDB06769 ; HMDB12533 ; HMDB12654 ; HMDB15092 ; HMDB31464 ; HMDB39589 ; HMDB39735 ; HMDB60089 ; HMDB60339	3a16b-Dihydroxyandrostenone ; 16-Oxoandrostenediol ; 3a16a-Dihydroxyandrostenone ; 16a-Hydroxydehydroisoandrosterone ; 3a16-Dihydroxyandrostenone ; 7a-Hydroxytestosterone ; 7a-Hydroxydehydroepiandrosterone ; 7b-Hydroxydehydroepiandrosterone ; 11-Ketoetiocholanolone ; 6beta-Hydroxytestosterone ; 19-Hydroxytestosterone ; 11beta-Hydroxytestosterone ; 2beta-Hydroxytestosterone ; Norgestimate ; cis-8-Shogaol ; Ginsenoyne G ; 10-Acetoxy-8-heptadecene-46-diyn-3-ol ; w Hydroxy testosterone ; 11beta17beta-Dihydroxy-4-androsten-3-one	M+2ACN+H ; M+2ACN+H ; M+2ACN+H ; M+2ACN+H ; M+2ACN+H ; M+2ACN+H ; M+2ACN+H ; M+2ACN+H ; M+2ACN+H ; M+2ACN+H ; M+2ACN+H ; M+2ACN+H ; M+2ACN+H ; M+NH4 ; M+2ACN+H ; M+2ACN+H ; M+2ACN+H ; M+2ACN+H ; M+2ACN+H	C19H28O3 ; C19H28O3 ; C19H28O3 ; C19H28O3 ; C19H28O3 ; C19H28O3 ; C19H28O3 ; C19H28O3 ; C19H28O3 ; C19H28O3 ; C19H28O3 ; C19H28O3 ; C19H28O3 ; C23H31NO3 ; C19H28O3 ; C19H28O3 ; C19H28O3 ; C19H28O3 ; C19H28O3
X143	8	417.142	0	HMDB34157 ; HMDB03920 ; HMDB15011 ; HMDB30265 ; HMDB30322 ; HMDB30375 ; HMDB33445	2-Hydroxybenzaldehyde O-xylosyl-(1-6)-glucoside ; Protopine ; Loteprednol ; 22-Dimethyl(pyrano-56:3:4)-15-dihydroxy-6-methoxy-10-methylacridone ; Papaveraldine ; Citracridone I ; Honyumine	M+H ; M+ACN+Na ; M+Na ; M+ACN+Na ; M+ACN+Na ; M+ACN+Na ; M+ACN+Na	C18H24O11 ; C20H19NO5 ; C21H27ClO5 ; C20H19NO5 ; C20H19NO5 ; C20H19NO5 ; C20H19NO5
X146	8	137.016	0	HMDB14901 ; HMDB34106 ; HMDB60445 ; HMDB60447	Methimazole ; Methyl isothiocyanate ; Bromobenzene-23-dihydrodiol ; Bromobenzene-34-dihydrodiol	M+Na ; M+ACN+Na ; M+2ACN+2H ; M+2ACN+2H	C4H6N2S ; C2H3NS ; C6H7BrO2 ; C6H7BrO2
X177	8	304.982	0	Unknow	Unknow	Unknow	Unknow
X182	8	175.148	0	HMDB36683 ; HMDB59696 ; HMDB59826	57alpha-Dihydro-1447a-tetramethyl-4H-indene ; 1234Tetrahydro-157-trimethylnapthalene ; alpha-Ionene	M+H ; M+H ; M+H	C13H18 ; C13H18 ; C13H18
X201	8	341.002	0	HMDB00968 ; HMDB01047 ; HMDB01058 ; HMDB03514 ; HMDB06234 ; HMDB06235 ; HMDB06872 ; HMDB60269 ; HMDB60444 ; HMDB01112 ; HMDB01473	1D-Myo-inositol 14-bisphosphate ; D-Fructose 26-bisphosphate ; Fructose 16-bisphosphate ; Alpha-D-Glucose 16-bisphosphate ; 1D-Myo-inositol 13-bisphosphate ; 1D-Myo-inositol 34-bisphosphate ; D-Tagatose 16-bisphosphate ; D-Mannose 16-bisphosphate ; beta-D-Fructose 16-bisphosphate ; D-Glyceraldehyde 3-phosphate ; Dihydroxyacetone phosphate	M+H ; M+H ; M+H ; M+H ; M+H ; M+H ; M+H ; M+H ; M+H ; 2M+H ; 2M+H	C6H14O12P2 ; C6H14O12P2 ; C6H14O12P2 ; C6H14O12P2 ; C6H14O12P2 ; C6H14O12P2 ; C6H14O12P2 ; C6H14O12P2 ; C6H14O12P2 ; C3H7O6P ; C3H7O6P
X208	8	425.603	0	Unknow	Unknow	Unknow	Unknow
X210	8	223.563	0	HMDB01397 ; HMDB11670 ; HMDB59639	Guanosine monophosphate ; 8-Oxo-dGMP ; Cyclic pyranopterin monophosphate	M+2ACN+2H ; M+2ACN+2H ; M+2ACN+2H	C10H14N5O8P ; C10H14N5O8P ; C10H14N5O8P
X215	8	220.044	0	HMDB30000 ; HMDB37553 ; HMDB39940	Bissulfine ; SS-Ethylidene dithioacetate ; S-2-Propenyl 2-propene-1-sulfonothioate	M+ACN+H ; M+ACN+H ; M+ACN+H	C6H10O2S2 ; C6H10O2S2 ; C6H10O2S2
X228	8	378.95	0	Unknow	Unknow	Unknow	Unknow
X246	8	507.586	0	Unknow	Unknow	Unknow	Unknow

X25	8	164.056	0	HMDB00802 ; HMDB30393 ; HMDB31187 ; HMDB33581 ; HMDB36735 ; HMDB36936 ; HMDB39167 ; HMDB39169 ; HMDB39509 ; HMDB60077 ; HMDB00208 ; HMDB13701 ; HMDB29572 ; HMDB31157 ; HMDB31708 ; HMDB31872 ; HMDB33552 ; HMDB33966 ; HMDB41390 ; HMDB41393 ; HMDB41492	Pterin ; L-N-Carboxymethylserine ; Thialdine ; trans-o-Coumaric acid 2-glucoside ; Bilobalide A ; 1-O-p-Coumaroyl-beta-D-glucose ; 2-O-p-Coumaroyl-D-glucose ; 6-O-p-Coumaroyl-D-glucose ; trans-p-Coumaric acid 4-glucoside ; cis-beta-D-Glucosyl-2-hydroxycinnamate ; Oxoglutaric acid ; 3-Oxoglutaric acid ; Diethyl disulfide ; 23-Butanedithiol ; 12-Bis(methylthio)ethane ; Methyl propyl disulfide ; ()-13-Butanedithiol ; Di-2-propenyl disulfide 9CI ; (EE)-Di-1-propenyl disulfide ; (E)-1-Propenyl 2-propenyl disulfide ; 12-Butanedithiol	M+H ; M+H ; M+H ; M+2H ; M+2H ; M+2H ; M+2H ; M+2H ; M+2H ; M+2H ; M+NH4 ; M+NH4 ; M+ACN+H ; M+ACN+H ; M+ACN+H ; M+ACN+H ; M+ACN+H ; M+NH4 ; M+NH4 ; M+NH4 ; M+ACN+H	C6H5N5O ; C5H9NO5 ; C6H13NS2 ; C15H18O8 ; C15H18O8 ; C15H18O8 ; C15H18O8 ; C15H18O8 ; C15H18O8 ; C15H18O8 ; C5H6O5 ; C5H6O5 ; C4H10S2 ; C4H10S2 ; C4H10S2 ; C4H10S2 ; C4H10S2 ; C6H10S2 ; C6H10S2 ; C6H10S2 ; C4H10S2
X257	8	549.59	0	HMDB39203	Camelliatannin A	M+ACN+2H	C49H36O27
X26	8	344.43	0	Unknow	Unknow	Unknow	Unknow
X28	8	226.081	0	HMDB35067 ; HMDB15526 ; HMDB28682 ; HMDB28724 ; HMDB28797 ; HMDB28839 ; HMDB28850 ; HMDB28855 ; HMDB29039 ; HMDB29149 ; HMDB29424	Melleolide L ; Ketazolam ; Alanyl-Asparagine ; Asparaginyl-Alanine ; Glutaminyl-Glycine ; Glycyl-Glutamine ; Glycyl-Serine ; Glycyl-Gamma-glutamate ; Serinyl-Glycine ; Gamma-glutamyl-Glycine ; L-4-Hydroxyglutamine	M+2H ; M+2ACN+2H ; M+Na ; M+Na ; M+Na ; M+Na ; M+ACN+Na ; M+Na ; M+ACN+Na ; M+Na ; M+ACN+Na	C23H27ClO7 ; C20H17ClN2O3 ; C7H13N3O4 ; C7H13N3O4 ; C7H13N3O4 ; C7H13N3O4 ; C5H10N2O4 ; C7H13N3O4 ; C5H10N2O4 ; C7H13N3O4 ; C5H10N2O4
X280	8	293.552	0	Unknow	Unknow	Unknow	Unknow
X31	8	225.087	0	HMDB00732 ; HMDB11631 ; HMDB12819 ; HMDB14787 ; HMDB29397 ; HMDB00017 ; HMDB00978 ; HMDB02349 ; HMDB02432 ; HMDB06331 ; HMDB15249 ; HMDB29832 ; HMDB30080 ; HMDB30816 ; HMDB31663 ; HMDB32923 ; HMDB32988 ; HMDB33882 ; HMDB36556 ; HMDB38674 ; HMDB59762 ; HMDB60328	Hydroxykynurenine ; L-3-Hydroxykynurenine ; 5-Hydroxykynurenine ; Stavudine ; L-Nicotianine ; 4-Pyridoxic acid ; 4-(2-Aminophenyl)-24-dioxobutanoic acid ; trans-trans-Muconic acid ; Sumikis acid ; ciscis-Muconic acid ; Atovaquone ; 8-Carboxymethyldihydrochelerythrine ; Wampetin ; 6-Acetyl-22-dimethyl-2H-1-benzopyran ; 3-(3-Methylbutylidene)-1(3H)-isobenzofuranone ; Kojic acid ; 5-Hydroxymaltol ; Glycyrol ; Glycyrrhizaisoflavone B ; gamma-L-Glutamyl-gamma-L-glutamyl-L-methionine ; 23-Methylenesuccinic acid ; 1-Nitro-56-dihydroxy-dihydronaphthalene	M+H ; M+H ; M+H ; M+H ; M+H ; M+ACN+H ; M+NH4 ; M+2ACN+H ; M+2ACN+H ; M+2ACN+H ; M+2ACN+2H ; M+ACN+2H ; M+2ACN+2H ; M+Na ; M+Na ; M+2ACN+H ; M+2ACN+H ; M+2ACN+2H ; M+2ACN+2H ; M+ACN+2H ; M+2ACN+H ; M+NH4	C10H12N2O4 ; C10H12N2O4 ; C10H12N2O4 ; C10H12N2O4 ; C10H12N2O4 ; C8H9NO4 ; C10H9NO4 ; C6H6O4 ; C6H6O4 ; C6H6O4 ; C22H19ClO3 ; C23H21NO6 ; C21H18O6 ; C13H14O2 ; C13H14O2 ; C6H6O4 ; C6H6O4 ; C21H18O6 ; C21H18O6 ; C15H25N3O8S ; C6H6O4 ; C10H9NO4
X338	8	219.543	0	HMDB14599	Cefalotin	M+ACN+2H	C16H16N2O6S2
X373	8	231.937	0	Unknow	Unknow	Unknow	Unknow
X400	8	438.08	0	Unknow	Unknow	Unknow	Unknow
X409	8	300.016	0	HMDB02028 ; HMDB37131	DOPA sulfate ; ()-Furilazole	M+Na ; M+Na	C9H11NO7S ; C11H13Cl2NO3
X439	8	437.076	0	Unknow	Unknow	Unknow	Unknow
X464	8	127.509	0	Unknow	Unknow	Unknow	Unknow
X510	8	538.386	0	HMDB11490 ; HMDB11520 ; HMDB32228 ; HMDB34505 ; HMDB34528 ; HMDB34644 ; HMDB34683 ; HMDB35326 ; HMDB36221 ; HMDB36640 ; HMDB37713 ; HMDB37719 ; HMDB39692	LysoPE(0:022:0) ; LysoPE(22:00:0) ; Dimethylbenzyl carbinyl hexanoate ; Soyasapogenol A ; Camelliagenin A ; Priverogenin B ; (3alphaOH20S24S)-319:2024-Diepoxydammarane-325-diol ; Ganoderiol A ; 2-(Dimethoxymethyl)-1-heptenylbenzene ; Furanofukinin ; Octyl phenylacetate ; 2-Phenylethyl octanoate ; 2024-Epoxy-2526-dihydroxydammaran-3-one	M+H ; M+H ; 2M+ACN+H ; M+ACN+Na ; M+ACN+Na ; M+ACN+Na ; M+ACN+Na ; M+ACN+Na ; 2M+ACN+H ; 2M+ACN+H ; 2M+ACN+H ; 2M+ACN+H ; M+ACN+Na	C27H56NO7P ; C27H56NO7P ; C16H24O2 ; C30H50O4 ; C30H50O4 ; C30H50O4 ; C30H50O4 ; C30H50O4 ; C16H24O2 ; C16H24O2 ; C16H24O2 ; C16H24O2 ; C30H50O4
X511	8	530.287	0	HMDB40945 ; HMDB40963	Helianthoside A ; Matesaponin 2	M+2H ; M+2H	C53H86O21 ; C53H86O21

X516	8	195.088	0	HMDB01847 ; HMDB14962 ; HMDB29915 ; HMDB29916 ; HMDB29965 ; HMDB31437 ; HMDB31449 ; HMDB33816 ; HMDB34219 ; HMDB34221 ; HMDB34222 ; HMDB37295 ; HMDB14545 ; HMDB00300 ; HMDB00466 ; HMDB12488 ; HMDB30171 ; HMDB34236 ; HMDB39031 ; HMDB60760 ; HMDB60788	Caffeine ; Enprofylline ; D-4-O-Methyl-myo-inositol ; 4-O-Methyl-myo-inositol ; Methyl beta-D-glucopyranoside ; (-)-Bornesitol ; Sequoyitol ; Mytilitol ; D-Pinitol ; L-Quebrachitol ; L-Pinitol ; 3-(2-Mercapto-1-methylpropyl)thio-2-butanol ; Nisoldipine ; Uracil ; 3-Methylindole ; 1234-Tetrahydro-beta-carboline ; N-Methyl-14-O-demethylepiporphyroxine ; Benzenepropanenitrile ; trans-O-Methylgrandmarin ; 4-Carboxypyrazole ; 6-O-Desmethyl-mycophenolic acid	M+H ; M+H ; M+H ; M+H ; M+H ; M+H ; M+H ; M+H ; M+H ; M+H ; M+H ; M+H ; M+2H ; M+2ACN+H ; M+ACN+Na ; M+Na ; M+H+NH4 ; M+ACN+Na ; M+2ACN+2H ; M+2ACN+H ; M+2ACN+2H	C8H10N4O2 ; C8H10N4O2 ; C7H14O6 ; C7H14O6 ; C7H14O6 ; C7H14O6 ; C7H14O6 ; C7H14O6 ; C7H14O6 ; C7H14O6 ; C7H14O6 ; C8H18OS2 ; C20H24N2O6 ; C4H4N2O2 ; C9H9N ; C11H12N2 ; C20H21NO6 ; C9H9N ; C16H18O6 ; C4H4N2O2 ; C16H18O6
X53	8	313.017	0	HMDB04812	25-Furandicarboxylic acid	2M+H	C6H4O5
X537	8	515.313	0.003	HMDB39150 ; HMDB14393 ; HMDB29656 ; HMDB35493 ; HMDB35749 ; HMDB35778 ; HMDB36207 ; HMDB37705 ; HMDB39547 ; HMDB39597 ; HMDB40291 ; HMDB40390 ; HMDB41250	Flavidulol C ; Cabergoline ; Erinacine E ; Notoginsenoside K ; Ginsenoside B2 ; Ginsenoside Rd ; alpha-Amylcinnamyl acetate ; Heptyl cinnamate ; Gynosaponin S ; Colupdox a ; Erinacine A ; Erinacine B ; Demethoxyshogaol	M+H ; M+ACN+Na ; M+2ACN+H ; M+2ACN+2H ; M+2ACN+2H ; M+2ACN+2H ; 2M+Na ; 2M+Na ; M+2ACN+2H ; M+2ACN+H ; M+2ACN+H ; M+2ACN+H ; 2M+Na	C34H42O4 ; C26H37N5O2 ; C25H36O6 ; C48H82O18 ; C48H82O18 ; C48H82O18 ; C16H22O2 ; C16H22O2 ; C48H82O18 ; C25H36O6 ; C25H36O6 ; C25H36O6 ; C16H22O2
X545	8	224.09	0	HMDB00866 ; HMDB13068 ; HMDB00555 ; HMDB00857 ; HMDB02441 ; HMDB15400 ; HMDB29167 ; HMDB29466 ; HMDB29472 ; HMDB29573 ; HMDB29775 ; HMDB30818 ; HMDB32952 ; HMDB35303 ; HMDB36627 ; HMDB41605 ; HMDB59722 ; HMDB59738 ; HMDB59893	N-Acetyl-L-tyrosine ; Salsolinol 1-carboxylate ; 3-Methyladipic acid ; Pimelic acid ; 33-Dimethylglutaric acid ; Hydralazine ; 2-Methyladipic acid ; Eugenitol ; Isoeugenitol ; Diethyl malonate ; Vinyl caffeate ; Scoparone ; Citropten ; 3-Methoxy-45-methylenedioxycinnamaldehyde ; Eugenin ; Propyleneglycol diacetate ; Mono-methyl-adipate ; 2-Ethylglutaric acid ; Ethyl methylsuccinate	M+H ; M+H ; M+ACN+Na ; M+ACN+Na ; M+ACN+Na ; M+ACN+Na ; M+ACN+Na ; M+NH4 ; M+NH4 ; M+ACN+Na ; M+NH4 ; M+NH4 ; M+NH4 ; M+NH4 ; M+NH4 ; M+ACN+Na ; M+ACN+Na ; M+ACN+Na ; M+ACN+Na	C11H13NO4 ; C11H13NO4 ; C7H12O4 ; C7H12O4 ; C7H12O4 ; C8H8N4 ; C7H12O4 ; C11H10O4 ; C11H10O4 ; C7H12O4 ; C11H10O4 ; C11H10O4 ; C11H10O4 ; C11H10O4 ; C11H10O4 ; C7H12O4 ; C7H12O4 ; C7H12O4 ; C7H12O4
X57	8	151.07	0	HMDB14623 ; HMDB28783 ; HMDB29014 ; HMDB34911	Lenalidomide ; Cysteinyl-Proline ; Prolyl-Cysteine ; (S)-3-(Cyanophenylmethyl)amino-3-oxopropanoic acid	M+ACN+2H ; M+2ACN+2H ; M+2ACN+2H ; M+2ACN+2H	C13H13N3O3 ; C8H14N2O3S ; C8H14N2O3S ; C11H10N2O3
X573	8	429.24	0	HMDB15163 ; HMDB01038 ; HMDB15119 ; HMDB28846 ; HMDB28951 ; HMDB32819 ; HMDB32896 ; HMDB34605 ; HMDB35047 ; HMDB35812 ; HMDB36474 ; HMDB36661 ; HMDB36690 ; HMDB36894 ; HMDB36901 ; HMDB40795 ; HMDB41795	Irbesartan ; Angiotensin IV ; Nandrolone phenpropionate ; Glycyl-Lysine ; Lysyl-Glycine ; Gibberellin A113 ; 3-Oxo-6b-angeloyloxy-7(11)-eremophilen-128a-olide ; Annuolide G ; Gibberellin A37 ; Epirosmanol ; Verimol G ; Isorosmanol ; 3-Epinobilin ; Gibberellin A64 ; Gibberellin A44 ; 7-O-Methylmarmin ; 19-Noraldosterone	M+H ; M+2ACN+2H ; M+Na ; 2M+Na ; 2M+Na ; M+2ACN+H ; M+2ACN+H ; M+2ACN+H ; M+2ACN+H ; M+2ACN+H ; M+2ACN+H ; M+2ACN+H ; M+2ACN+H ; M+2ACN+H ; M+2ACN+H ; M+2ACN+H ; M+2ACN+H	C25H28N6O ; C40H54N8O8 ; C27H34O3 ; C8H17N3O3 ; C8H17N3O3 ; C20H26O5 ; C20H26O5 ; C20H26O5 ; C20H26O5 ; C20H26O5 ; C20H26O5 ; C20H26O5 ; C20H26O5 ; C20H26O5 ; C20H26O5 ; C20H26O5 ; C20H26O5
X575	8	527.316	0	HMDB09933 ; HMDB09969 ; HMDB09977 ; HMDB09988 ; HMDB09997 ; HMDB09999 ; HMDB10000 ; HMDB10002 ; HMDB10023 ; HMDB32305 ; HMDB33034	PIP(16:022:2(13Z16Z)) ; PIP(18:1(15Z)20:1(11Z)) ; PIP(18:1(9Z)20:1(11Z)) ; PIP(18:2(9Z12Z)20:0) ; PIP(20:018:2(9Z12Z)) ; PIP(20:1(11Z)18:1(11Z)) ; PIP(20:1(11Z)18:1(9Z)) ; PIP(20:2(11Z14Z)18:0) ; PIP(22:2(13Z16Z)16:0) ; N-(Heptan-4-yl)benzod13dioxole-5-carboxamide ; Antibiotic GR 95647X	M+2ACN+2H ; M+2ACN+2H ; M+2ACN+2H ; M+2ACN+2H ; M+2ACN+2H ; M+2ACN+2H ; M+2ACN+2H ; M+2ACN+2H ; M+2ACN+2H ; 2M+H ; M+2ACN+H	C47H88O16P2 ; C47H88O16P2 ; C47H88O16P2 ; C47H88O16P2 ; C47H88O16P2 ; C47H88O16P2 ; C47H88O16P2 ; C47H88O16P2 ; C47H88O16P2 ; C15H21NO3 ; C26H36O6
X580	8	499.348	0.003	HMDB10381 ; HMDB11129 ; HMDB11130 ; HMDB15004 ; HMDB30271 ; HMDB60852 ; HMDB60997 ; HMDB61007	LysoPC(15:0) ; LysoPE(0:018:0) ; LysoPE(18:00:0) ; Alprenolol ; Dehydrocarpaine I ; NO-Didesmethylvenlafaxine ; O-Desmethyltramadol ; N-Desmethyltramadol	M+NH4 ; M+NH4 ; M+NH4 ; 2M+H ; M+Na ; 2M+H ; 2M+H ; 2M+H	C23H48NO7P ; C23H48NO7P ; C23H48NO7P ; C15H23NO2 ; C28H48N2O4 ; C15H23NO2 ; C15H23NO2 ; C15H23NO2

X584	8	332.562	0.005	HMDB39421	Fenugreekine	M+2H	C21H27N7O14P2
X59	8	357.037	0	HMDB06462 ; HMDB15511 ; HMDB60636	Homocysteinesulfinic acid ; Bromazepam ; Malathion dicarboxylic acid	2M+Na ; M+ACN+H ; M+2ACN+H	C4H9NO4S ; C14H10BrN3O ; C6H11O6PS2
X591	8	497.343	0	Unknow	Unknow	Unknow	Unknow
X6	8	299.083	0	HMDB39922 ; HMDB11737 ; HMDB42036	Eriodictyol 7-(6-trans-p-coumaroylglucoside) ; Gamma Glutamylglutamic acid ; Thymidine glycol	M+2H ; M+Na ; M+Na	C30H28O13 ; C10H16N2O7 ; C10H16N2O7
X615	8	511.358	0	HMDB14930 ; HMDB31867 ; HMDB39588	Tripelennamine ; 4-Acetyl-6-tert-butyl-11-dimethylindane ; Panaxynol	2M+H ; 2M+Na ; 2M+Na	C16H21N3 ; C17H24O ; C17H24O
X641	8	664.115	0.003	Unknow	Unknow	Unknow	Unknow
X654	8	496.339	0	HMDB10382 ; HMDB06228 ; HMDB06321 ; HMDB06496 ; HMDB06710 ; HMDB12454 ; HMDB12556 ; HMDB30702 ; HMDB31402 ; HMDB33769 ; HMDB36850 ; HMDB60134	LysoPC(16:0) ; 24-Hydroxycalcitriol ; Docosa-47101316-pentaenoyl carnitine ; Clupanodonyl carnitine ; Ubiquinone-4 ; 3 beta7 alpha-Dihydroxy-5-cholestenoate ; 13-Carboxy-alpha-tocotrienol ; Neochlorogenin ; 23-Acetoxysoladulcidine ; Rockogenin ; Ceanothenic acid ; 23S2526-Trihydroxyvitamin D3	M+H ; M+ACN+Na ; M+Na ; M+Na ; M+ACN+H ; M+ACN+Na ; M+ACN+H ; M+ACN+Na ; M+Na ; M+ACN+Na ; M+ACN+H ; M+ACN+Na	C24H50NO7P ; C27H44O4 ; C29H47NO4 ; C29H47NO4 ; C29H42O4 ; C27H44O4 ; C29H42O4 ; C27H44O4 ; C29H47NO4 ; C27H44O4 ; C29H42O4 ; C27H44O4
X672	8	731.162	0	HMDB37965 ; HMDB37966 ; HMDB31981 ; HMDB39861	ent-Epicatechin-(4alpha-8)-ent-epicatechin 3-gallate ; ent-Epicatechin-(4alpha-8)-ent-epicatechin 3-gallate ; Niazidin ; 3-O-alpha-L-Arabinopyranosylproanthocyanidin A5	M+H ; M+H ; 2M+Na ; M+Na	C37H30O16 ; C37H30O16 ; C15H18N2O6S ; C35H32O16
X675	8	515.815	0.014	Unknow	Unknow	Unknow	Unknow
X69	8	303.546	0	HMDB01163 ; HMDB03351 ; HMDB33533	Guanosine diphosphate mannose ; GDP-glucose ; Ceftiofur	M+2H ; M+2H ; M+2ACN+2H	C16H25N5O16P2 ; C16H25N5O16P2 ; C19H17N5O7S3
X694	8	482.36	0	HMDB06280 ; HMDB06281 ; HMDB06763 ; HMDB06764 ; HMDB06886 ; HMDB06887 ; HMDB06894 ; HMDB11644 ; HMDB12560 ; HMDB35730 ; HMDB60136	7-a25-Dihydroxycholesterol ; 7-a27-Dihydroxycholesterol ; 20a22b-Dihydroxycholesterol ; 17a20a-Dihydroxycholesterol ; 7a12a-Dihydroxy-5a-cholestan-3-one ; 7a12a-Dihydroxy-5b-cholestan-3-one ; 3a7a-Dihydroxy-5b-cholestan-26-al ; (24R)-Cholest-5-ene-3-beta7-alpha24-triol ; 13-Hydroxy-alpha-tocotrienol ; Camelledionol ; (24S)-7alpha24-Dihydroxycholesterol	M+ACN+Na ; M+ACN+Na ; M+ACN+Na ; M+ACN+Na ; M+ACN+Na ; M+ACN+Na ; M+ACN+Na ; M+ACN+Na ; M+ACN+H ; M+ACN+H ; M+ACN+Na	C27H46O3 ; C27H46O3 ; C27H46O3 ; C27H46O3 ; C27H46O3 ; C27H46O3 ; C27H46O3 ; C27H46O3 ; C29H44O3 ; C29H44O3 ; C27H46O3
X72	8	133.059	0	Unknow	Unknow	Unknow	Unknow
X720	8	527.381	0	HMDB06227 ; HMDB11481 ; HMDB11511 ; HMDB12108 ; HMDB13892 ; HMDB14339 ; HMDB15646 ; HMDB29567 ; HMDB32667 ; HMDB32691 ; HMDB33647 ; HMDB38977 ; HMDB60532	1-a24R25-Trihydroxyvitamin D2 ; LysoPE(0:020:0) ; LysoPE(20:00:0) ; LysoPC(17:0) ; N-Desmethylvenlafaxine ; Tramadol ; Desvenlafaxine ; Hydroxy-alpha-sanshool ; 59-Epidioxy-3-hydroxyergost-7-en-6-one ; (3beta5alpha6alpha7beta14alpha22E24R)-56-Epoxyergosta-822-diene-3714-triol ; 56:89-Diepoxyergost-22-ene-37beta-diol ; (3beta5alpha6beta9alpha22E24R)-59-Epidioxyergosta-722-diene-36-diol ; O-Desmethylvenlafaxine	M+2ACN+H ; M+NH4 ; M+NH4 ; M+NH4 ; 2M+H ; 2M+H ; 2M+H ; 2M+H ; M+2ACN+H ; M+2ACN+H ; M+2ACN+H ; M+2ACN+H ; 2M+H	C28H44O4 ; C25H52NO7P ; C25H52NO7P ; C25H52NO7P ; C16H25NO2 ; C16H25NO2 ; C16H25NO2 ; C16H25NO2 ; C28H44O4 ; C28H44O4 ; C28H44O4 ; C28H44O4 ; C16H25NO2
X746	8	521.343	0	Unknow	Unknow	Unknow	Unknow
X765	8	545.343	0.016	HMDB41027	Hovenidulcigenin A	M+H	C32H48O7
X78	8	484.011	0	Unknow	Unknow	Unknow	Unknow

X79	8	309.074	0	HMDB29527 ; HMDB29870 ; HMDB29988 ; HMDB30090 ; HMDB30622 ; HMDB30793 ; HMDB30807 ; HMDB30852 ; HMDB31420 ; HMDB31620 ; HMDB32288 ; HMDB33291 ; HMDB33309 ; HMDB34011 ; HMDB34012 ; HMDB34114 ; HMDB34115 ; HMDB34438 ; HMDB37320 ; HMDB37322 ; HMDB37489 ; HMDB38448 ; HMDB40201 ; HMDB41726	Melilotocarpan B ; 37-Dihydroxy-4-methoxyisoflavanone ; Bis(2-furanylmethyl) disulfide ; Sakuranetin ; (R)-Oxypeucedanin ; Moracin B ; 34-Dihydro-8-hydroxy-3-(3-hydroxy-4-methoxyphenyl)-1H-2-benzopyran-1-one ; Asperxanthone ; Heliannone C ; Vestitone ; Furfuryl 2-methyl-3-furyl disulfide ; 38-Dihydroxy-9-methoxypterocarpan ; Moracin F ; 2-(24-Dihydroxyphenyl)-56-dimethoxybenzofuran ; 34-Dihydroxy-9-methoxypterocarpan ; 6alpha-Hydroxyisomedicarpin ; 6alpha-Hydroxymedicarpin ; (R)-Pabulenol ; Licochalcone B ; Dihydrooroxylin ; Dihydrowogonin ; (-)-57-Dihydroxy-3-(4-hydroxybenzyl)-4-chromanone ; 33-Dithiobis2-methylfuran ; Dihydroglycitein	M+Na ; M+Na ; M+2ACN+H ; M+Na ; M+Na ; M+Na ; M+Na ; M+Na ; M+Na ; M+Na ; M+2ACN+H ; M+Na ; M+Na ; M+Na ; M+Na ; M+Na ; M+Na ; M+Na ; M+Na ; M+Na ; M+Na ; M+Na ; M+2ACN+H ; M+Na	C16H14O5 ; C16H14O5 ; C10H10O2S2 ; C16H14O5 ; C16H14O5 ; C16H14O5 ; C16H14O5 ; C16H14O5 ; C16H14O5 ; C16H14O5 ; C10H10O2S2 ; C16H14O5 ; C16H14O5 ; C16H14O5 ; C16H14O5 ; C16H14O5 ; C16H14O5 ; C16H14O5 ; C16H14O5 ; C16H14O5 ; C16H14O5 ; C16H14O5 ; C10H10O2S2 ; C16H14O5
X863	8	86.0965	0	HMDB34301 ; HMDB31630	Piperidine ; Propane	M+H ; M+ACN+H	C5H11N ; C3H8

**Table S5b.** Metabolite feature name in each community for each factor							
Node	Community	m/z	centrality_vec	all_HMDBID	all_Name	all_Adduct	all_Formula
X18	1	164.05609	0	HMDB00802 ; HMDB30393 ; HMDB31187 ; HMDB33581 ; HMDB36735 ; HMDB36936 ; HMDB39167 ; HMDB39169 ; HMDB39509 ; HMDB60077 ; HMDB00208 ; HMDB13701 ; HMDB29572 ; HMDB31157 ; HMDB31708 ; HMDB31872 ; HMDB33552 ; HMDB33966 ; HMDB41390 ; HMDB41393 ; HMDB41492	Pterin ; L-N-Carboxymethylserine ; Thialdine ; trans-o-Coumaric acid 2-glucoside ; Bilobalide A ; 1-O-p-Coumaroyl-beta-D-glucose ; 2-O-p-Coumaroyl-D-glucose ; 6-O-p-Coumaroyl-D-glucose ; trans-p-Coumaric acid 4-glucoside ; cis-beta-D-Glucosyl-2-hydroxycinnamate ; Oxoglutaric acid ; 3-Oxoglutaric acid ; Diethyl disulfide ; 23-Butanedithiol ; 12-Bis(methylthio)ethane ; Methyl propyl disulfide ; ()-13-Butanedithiol ; Di-2-propenyl disulfide 9CI ; (EE)-Di-1-propenyl disulfide ; (E)-1-Propenyl 2-propenyl disulfide ; 12-Butanedithiol	M+H ; M+H ; M+H ; M+2H ; M+2H ; M+2H ; M+2H ; M+2H ; M+2H ; M+2H ; M+NH4 ; M+NH4 ; M+ACN+H ; M+ACN+H ; M+ACN+H ; M+ACN+H ; M+ACN+H ; M+NH4 ; M+NH4 ; M+NH4 ; M+ACN+H	C6H5N5O ; C5H9NO5 ; C6H13NS2 ; C15H18O8 ; C15H18O8 ; C15H18O8 ; C15H18O8 ; C15H18O8 ; C15H18O8 ; C15H18O8 ; C5H6O5 ; C5H6O5 ; C4H10S2 ; C4H10S2 ; C4H10S2 ; C4H10S2 ; C4H10S2 ; C6H10S2 ; C6H10S2 ; C6H10S2 ; C4H10S2
X19	1	344.43016	0	Unknow	Unknow	Unknow	Unknow
X248	1	221.00312	0	Unknow	Unknow	Unknow	Unknow
X25	1	469.06723	0	HMDB40628 ; HMDB00061 ; HMDB00960 ; HMDB01341 ; HMDB32937 ; HMDB33342 ; HMDB33352	3-(23-Digalloyl-46-hexahydroxydiphenoylglucosyl)-phloroacetophenone ; Adenosine 35-diphosphate ; dGDP ; ADP ; Methyl 4-chloro-1H-indole-3-acetate ; Mollicellin E ; Cyclobrassinin	M+2H ; M+ACN+H ; M+ACN+H ; M+ACN+H ; M+ACN+H ; 2M+Na ; M+Na ; 2M+H	C42H32O25 ; C10H15N5O10P2 ; C10H15N5O10P2 ; C10H15N5O10P2 ; C11H10ClNO2 ; C22H19ClO8 ; C11H10N2S2

X272	1	369.11831	0	HMDB30669 ; HMDB39959 ; HMDB39960 ; HMDB02022 ; HMDB12226 ; HMDB32401 ; HMDB32742 ; HMDB35057 ; HMDB36562 ; HMDB40601 ; HMDB40602 ; HMDB41189 ; HMDB41537 ; HMDB60343	3-O-Feruloylquinic acid ; 3-O-Caffeoyl-1-O-methylquinic acid ; 3-O-Caffeoyl-4-O-methylquinic acid ; Glycineamideribotide ; Entacapone ; (-)-3-(2-methyl-3-furyl)thio-2-butanone ; Di-O-methylcrenatin ; 1-(3-Hydroxy-4-Methoxyphenyl)-12-ethanediol 3-O-b-D-glucoside ; Aucubin ; (1xi2xi)-1-(4-Hydroxyphenyl)-123-propanetriol 2-O-beta-D-glucopyranoside ; (1xi2xi)-1-(4-Hydroxyphenyl)-123-propanetriol 3-O-beta-D-Glucopyranoside ; 345-Trimethoxyphenyl glucoside ; N-Acetyldehydroanonaine ; 2-(S-Glutathionyl)acetyl glutathione	M+H ; M+H ; M+H ; M+2ACN+H ; M+ACN+Na ; 2M+H ; M+Na ; M+Na ; M+Na ; M+Na ; M+Na ; M+Na ; M+ACN+Na ; M+2ACN+2H	C17H20O9 ; C17H20O9 ; C17H20O9 ; C7H15N2O8P ; C14H15N3O5 ; C9H12O2S ; C15H22O9 ; C15H22O9 ; C15H22O9 ; C15H22O9 ; C15H22O9 ; C15H22O9 ; C19H15NO3 ; C22H34N6O13S2
X31	1	526.56003	0	HMDB03424 ; HMDB30954 ; HMDB35278	1-Hexadecanol ; Grewinol ; 28-Hydroxy-6-methyl-5-tritriacontanone	2M+ACN+H ; M+NH4 ; M+NH4	C16H34O ; C34H68O2 ; C34H68O2
X329	1	449.76255	0.09	Unknow	Unknow	Unknow	Unknow
X331	1	515.31319	0.183	HMDB39150 ; HMDB14393 ; HMDB29656 ; HMDB35493 ; HMDB35749 ; HMDB35778 ; HMDB36207 ; HMDB37705 ; HMDB39547 ; HMDB39597 ; HMDB40291 ; HMDB40390 ; HMDB41250	Flavidulol C ; Cabergoline ; Erinacine E ; Notoginsenoside K ; Ginsenoside B2 ; Ginsenoside Rd ; alpha-Amylcinnamyl acetate ; Heptyl cinnamate ; Gynosaponin S ; Colupdox a ; Erinacine A ; Erinacine B ; Demethoxyshogaol	M+H ; M+ACN+Na ; M+2ACN+H ; M+2ACN+2H ; M+2ACN+2H ; M+2ACN+2H ; 2M+Na ; 2M+Na ; M+2ACN+2H ; M+2ACN+H ; M+2ACN+H ; M+2ACN+H ; 2M+Na	C34H42O4 ; C26H37N5O2 ; C25H36O6 ; C48H82O18 ; C48H82O18 ; C48H82O18 ; C16H22O2 ; C16H22O2 ; C48H82O18 ; C25H36O6 ; C25H36O6 ; C25H36O6 ; C16H22O2
X334	1	551.38948	0.126	HMDB32208 ; HMDB32837 ; HMDB34515 ; HMDB35434 ; HMDB35886 ; HMDB38797 ; HMDB39615 ; HMDB40711	Citronellyl anthranilate ; Ganoderic acid DM ; Glabrolide ; Isomasticadienonalic acid ; Isoglabrolide ; Uralenolide ; Colupone ; 16beta-16-Hydroxy-3-oxo-112-oleanadien-28-oic acid	2M+H ; M+2ACN+H ; M+2ACN+H ; M+2ACN+H ; M+2ACN+H ; M+2ACN+H ; M+2ACN+H ; M+2ACN+H	C17H25NO2 ; C30H44O4 ; C30H44O4 ; C30H44O4 ; C30H44O4 ; C30H44O4 ; C30H44O4 ; C30H44O4
X34	1	303.54626	0	HMDB01163 ; HMDB03351 ; HMDB33533	Guanosine diphosphate mannose ; GDP-glucose ; Ceftiofur	M+2H ; M+2H ; M+2ACN+2H	C16H25N5O16P2 ; C16H25N5O16P2 ; C19H17N5O7S3

X342	1	527.31647	0.095	HMDB09933 ; HMDB09969 ; HMDB09977 ; HMDB09988 ; HMDB09997 ; HMDB09999 ; HMDB10000 ; HMDB10002 ; HMDB10023 ; HMDB32305 ; HMDB33034	PIP(16:022:2(13Z16Z)) ; PIP(18:1(15Z)20:1(11Z)) ; PIP(18:1(9Z)20:1(11Z)) ; PIP(18:2(9Z12Z)20:0) ; PIP(20:018:2(9Z12Z)) ; PIP(20:1(11Z)18:1(11Z)) ; PIP(20:1(11Z)18:1(9Z)) ; PIP(20:2(11Z14Z)18:0) ; PIP(22:2(13Z16Z)16:0) ; N-(Heptan-4-yl)benzod13dioxole-5-carboxamide ; Antibiotic GR 95647X	M+2ACN+2H ; M+2ACN+2H ; M+2ACN+2H ; M+2ACN+2H ; M+2ACN+2H ; M+2ACN+2H ; M+2ACN+2H ; M+2ACN+2H ; M+2ACN+2H ; 2M+H ; M+2ACN+H	C47H88O16P2 ; C47H88O16P2 ; C47H88O16P2 ; C47H88O16P2 ; C47H88O16P2 ; C47H88O16P2 ; C47H88O16P2 ; C47H88O16P2 ; C47H88O16P2 ; C15H21NO3 ; C26H36O6
X346	1	332.56158	0.175	HMDB39421	Fenugreekine	M+2H	C21H27N7O14P2
X351	1	158.00276	0.315	HMDB01928 ; HMDB32930	Hydrochlorothiazide ; Benzothiazole	M+H+NH4 ; M+Na	C7H8ClN3O4S2 ; C7H5NS
X360	1	333.56404	0.118	Unknow	Unknow	Unknow	Unknow
X365	1	664.11536	0.095	Unknow	Unknow	Unknow	Unknow
X368	1	318.16785	0.078	HMDB15234 ; HMDB35587 ; HMDB36359 ; HMDB39765 ; HMDB41860 ; HMDB61077 ; HMDB61078 ; HMDB00279 ; HMDB04284 ; HMDB14333 ; HMDB29563 ; HMDB29671 ; HMDB29672 ; HMDB30755 ; HMDB30764 ; HMDB30922 ; HMDB32036 ; HMDB32136 ; HMDB32137 ; HMDB32139 ; HMDB32159 ; HMDB32290 ; HMDB34241 ; HMDB37245 ; HMDB37246 ; HMDB38325 ; HMDB39802 ; HMDB40174 ; HMDB40177 ; HMDB41607	Arbutamine ; Pandamarilactonine A ; Pandanamine ; Pandamarilactone 1 ; Cocaethylene ; alpha-oxycodol ; beta-oxycodol ; Saccharopine ; Tyrosol ; Esmolol ; 3-Acetyl-25-dimethylfuran ; 14-Dimethoxybenzene ; 13-Dimethoxybenzene ; Myrigalone E ; Acetylpterosin C ; 13-Diacetoxy-4612-tetradecatriene-810-diyne ; 4-Ethoxyphenol ; 2-Methoxy-4-methylphenol ; 26-Dimethyl-14-benzenediol ; 12-Dimethoxybenzene ; 2-Acetyl-35-dimethylfuran ; 1-(2-Furyl)butan-3-one ; 4-Methoxybenzyl alcohol ; Myrigalone A ; Myrigalon B ; Verimol C ; 1-(2-Furanyl)-1-butanone ; 4-Ethyl-12-benzenediol ; 3-Ethyl-12-benzenediol ; 2-Phenoxyethanol	M+H ; M+H ; M+H ; M+H ; M+H ; M+H ; M+H ; M+ACN+H ; 2M+ACN+H ; M+Na ; 2M+ACN+H ; 2M+ACN+H ; 2M+ACN+H ; M+NH4 ; M+ACN+H ; M+NH4 ; 2M+ACN+H ; 2M+ACN+H ; 2M+ACN+H ; 2M+ACN+H ; 2M+ACN+H ; 2M+ACN+H ; 2M+ACN+H ; M+NH4 ; M+NH4 ; M+NH4 ; 2M+ACN+H ; 2M+ACN+H ; 2M+ACN+H ; 2M+ACN+H	C18H23NO4 ; C18H23NO4 ; C18H23NO4 ; C18H23NO4 ; C18H23NO4 ; C18H23NO4 ; C18H23NO4 ; C11H20N2O6 ; C8H10O2 ; C16H25NO4 ; C8H10O2 ; C8H10O2 ; C8H10O2 ; C18H20O4 ; C16H20O4 ; C18H20O4 ; C8H10O2 ; C8H10O2 ; C8H10O2 ; C8H10O2 ; C8H10O2 ; C8H10O2 ; C8H10O2 ; C18H20O4 ; C18H20O4 ; C18H20O4 ; C8H10O2 ; C8H10O2 ; C8H10O2 ; C8H10O2
X372	1	133.09718	0.139	HMDB00214 ; HMDB03374 ; HMDB32455 ; HMDB00162 ; HMDB03411 ; HMDB12880 ; HMDB28961 ; HMDB29066 ; HMDB30409 ; HMDB34208	Ornithine ; D-Ornithine ; L-Ornithine monochlorohydrateornithine ; L-Proline ; D-Proline ; Acetamidopropanal ; Lysyl-Threonine ; Threoninyl-Lysine ; 4-Amino-2-methylenebutanoic acid ; Pterolactam	M+H ; M+H ; M+H ; M+NH4 ; M+NH4 ; M+NH4 ; M+H+NH4 ; M+H+NH4 ; M+NH4 ; M+NH4	C5H12N2O2 ; C5H12N2O2 ; C5H12N2O2 ; C5H9NO2 ; C5H9NO2 ; C5H9NO2 ; C10H21N3O4 ; C10H21N3O4 ; C5H9NO2 ; C5H9NO2

X373	1	665.11676	0.071	HMDB06555 ; HMDB30802 ; HMDB33648	dIMP ; Patuletin ; 45678-Pentahydroxy-3-methoxyflavone	2M+H ; 2M+H ; 2M+H	C10H13N4O7P ; C16H12O8 ; C16H12O8
X374	1	731.16216	0.001	HMDB37965 ; HMDB37966 ; HMDB31981 ; HMDB39861	ent-Epicatechin-(4alpha-8)-ent-epicatechin 3-gallate ; ent-Epicatechin-(4alpha-8)-ent-epicatechin 3-gallate ; Niazidin ; 3-O-alpha-L-Arabinopyranosylproanthocyanidin A5	M+H ; M+H ; 2M+Na ; M+Na	C37H30O16 ; C37H30O16 ; C15H18N2O6S ; C35H32O16
X375	1	515.8153	0.169	Unknow	Unknow	Unknow	Unknow
X379	1	523.29945	0.215	HMDB05770 ; HMDB05776 ; HMDB14913 ; HMDB15371 ; HMDB28718 ; HMDB29033 ; HMDB30917 ; HMDB31901 ; HMDB33234 ; HMDB34200 ; HMDB34721 ; HMDB35117 ; HMDB35137 ; HMDB35148 ; HMDB35299 ; HMDB35302 ; HMDB35358 ; HMDB35760 ; HMDB35798 ; HMDB36036 ; HMDB36037 ; HMDB36550 ; HMDB36563 ; HMDB36664 ; HMDB37064 ; HMDB37529 ; HMDB37559 ; HMDB39156 ; HMDB39635 ; HMDB39644 ; HMDB40754 ; HMDB61150	Tuftsin ; Kentsin ; Tirofiban ; Gemfibrozil ; Arginyl-Serine ; Serinyl-Arginine ; 1-Hydroxyacorenone ; Blennin A ; Ganoderic acid beta ; Physalolactone B ; Procurcumadiol ; (3beta6beta)-Furanoeremophilane-36-diol ; Heliannuol D ; (6beta8alpha)-6-Hydroxy-7(11)-eremophilen-128-olide ; Ganolucidic acid D ; Ganolucidic acid A ; Ketosantalic acid ; 3-Hydroxytrichothecene ; Piperdial ; 3beta-Dihydroxymarasmene ; 13-Hydroxymarasmene ; 3-Ketoapotrichothecene ; Valerenolic acid ; Ketopelenolide a ; FS4 toxin ; Lactaronecatorin A ; Cadabicilone ; 3beta-Hydroxycinnamolide ; Abscisic alcohol ; Heliannuol A ; (3beta8beta)-3-Hydroxy-7(11)-eremophilen-128-olide ; Hydroxyl frovatriptan	M+Na ; M+Na ; M+2ACN+H ; 2M+Na ; 2M+H ; 2M+H ; 2M+Na ; 2M+Na ; M+Na ; M+Na ; 2M+Na ; 2M+Na ; 2M+Na ; 2M+Na ; M+Na ; M+Na ; 2M+Na ; 2M+Na ; 2M+Na ; 2M+Na ; 2M+Na ; 2M+Na ; 2M+Na ; 2M+Na ; 2M+Na ; 2M+Na ; 2M+Na ; 2M+Na ; 2M+Na ; 2M+Na ; 2M+H	C21H40N8O6 ; C21H40N8O6 ; C22H36N2O5S ; C15H22O3 ; C9H19N5O4 ; C9H19N5O4 ; C15H22O3 ; C15H22O3 ; C30H44O6 ; C30H44O6 ; C15H22O3 ; C15H22O3 ; C15H22O3 ; C15H22O3 ; C30H44O6 ; C30H44O6 ; C15H22O3 ; C15H22O3 ; C15H22O3 ; C15H22O3 ; C15H22O3 ; C15H22O3 ; C15H22O3 ; C15H22O3 ; C15H22O3 ; C15H22O3 ; C15H22O3 ; C15H22O3 ; C15H22O3 ; C15H22O3 ; C15H22O3 ; C14H19N3O2
X38	1	484.01145	0	Unknow	Unknow	Unknow	Unknow

X382	1	130.08633	0.376	HMDB00070 ; HMDB00716 ; HMDB04226 ; HMDB05960 ; HMDB15212 ; HMDB29435 ; HMDB29444 ; HMDB59649 ; HMDB00039 ; HMDB00824 ; HMDB00990 ; HMDB01164 ; HMDB01873 ; HMDB03243 ; HMDB29581 ; HMDB30062 ; HMDB31217 ; HMDB31305 ; HMDB31344 ; HMDB31507 ; HMDB31543 ; HMDB31759 ; HMDB32233 ; HMDB33569 ; HMDB34247 ; HMDB39778 ; HMDB40253 ; HMDB40260 ; HMDB40579	Pipecolic acid ; L-Pipecolic acid ; N4-Acetylaminobutanal ; D-Pipecolic acid ; Vigabatrin ; L-trans-4-Methyl-2-pyrrolidinecarboxylic acid ; 2-Pyrrolidineacetic acid ; 3-Acetamidobutanal ; Butyric acid ; Propionylcarnitine ; Acetaldehyde ; trans-12-Dihydrobenzene-12-diol ; Isobutyric acid ; Acetoin ; (2E4E)-24-Hexadienoic acid ; Methyl propionate ; Ethyl acetate ; Oxirane ; 12-Cyclohexanedione ; 1-Hydroxy-2-butanone ; 3-Methyl-12-cyclopentanedione ; (E)-4-Oxo-2-hexen-1-al ; 25-Dimethyl-3(2H)-furanone ; Syoyualdehyde ; 2-(Methoxymethyl)furan ; xi-35-Dimethyl-2(5H)-furanone ; Propyl formate ; 55-Dimethyl-2(5H)-furanone ; Isopropyl formate	M+H ; M+H ; M+H ; M+H ; M+H ; M+H ; M+H ; M+H ; M+ACN+H ; M+ACN+2H ; 2M+ACN+H ; M+NH4 ; M+ACN+H ; M+ACN+H ; M+NH4 ; M+ACN+H ; M+ACN+H ; 2M+ACN+H ; M+NH4 ; M+ACN+H ; M+NH4 ; M+NH4 ; M+NH4 ; M+NH4 ; M+NH4 ; M+NH4 ; M+ACN+H ; M+NH4 ; M+ACN+H	C6H11NO2 ; C6H11NO2 ; C6H11NO2 ; C6H11NO2 ; C6H11NO2 ; C6H11NO2 ; C6H11NO2 ; C6H11NO2 ; C4H8O2 ; C10H19NO4 ; C2H4O ; C6H8O2 ; C4H8O2 ; C4H8O2 ; C6H8O2 ; C4H8O2 ; C4H8O2 ; C2H4O ; C6H8O2 ; C4H8O2 ; C6H8O2 ; C6H8O2 ; C6H8O2 ; C6H8O2 ; C6H8O2 ; C6H8O2 ; C4H8O2 ; C6H8O2 ; C4H8O2
X383	1	482.36038	0.038	HMDB06280 ; HMDB06281 ; HMDB06763 ; HMDB06764 ; HMDB06886 ; HMDB06887 ; HMDB06894 ; HMDB11644 ; HMDB12560 ; HMDB35730 ; HMDB60136	7-a25-Dihydroxycholesterol ; 7-a27-Dihydroxycholesterol ; 20a22b-Dihydroxycholesterol ; 17a20a-Dihydroxycholesterol ; 7a12a-Dihydroxy-5a-cholestan-3-one ; 7a12a-Dihydroxy-5b-cholestan-3-one ; 3a7a-Dihydroxy-5b-cholestan-26-al ; (24R)-Cholest-5-ene-3-beta7-alpha24-triol ; 13-Hydroxy-alpha-tocotrienol ; Camelledionol ; (24S)-7alpha24-Dihydroxycholesterol	M+ACN+Na ; M+ACN+Na ; M+ACN+Na ; M+ACN+Na ; M+ACN+Na ; M+ACN+Na ; M+ACN+Na ; M+ACN+Na ; M+ACN+H ; M+ACN+H ; M+ACN+Na	C27H46O3 ; C27H46O3 ; C27H46O3 ; C27H46O3 ; C27H46O3 ; C27H46O3 ; C27H46O3 ; C27H46O3 ; C29H44O3 ; C29H44O3 ; C27H46O3

X386	1	115.08668	0.147	HMDB00323 ; HMDB61162 ; HMDB00182 ; HMDB01106 ; HMDB01875 ; HMDB01888 ; HMDB02134 ; HMDB03405 ; HMDB12114 ; HMDB12115 ; HMDB31339 ; HMDB32968 ; HMDB32969 ; HMDB32970 ; HMDB60177	3-Amino-2-piperidone ; N-Mononitrosopiperazine ; L-Lysine ; 3-Aminopropionaldehyde ; Methanol ; NN-Dimethylformamide ; Aminoacetone ; D-Lysine ; (3S)-36-Diaminohexanoate ; (3S5S)-35-Diaminohexanoate ; 1-Cyano-2-hydroxy-3-butene ; 24-Dimethyloxazole ; 25-Dimethyloxazole ; 45-Dimethyloxazole ; Ammonioacetone	M+H ; M+H ; M+2ACN+2H ; M+ACN+H ; M+2ACN+H ; M+ACN+H ; M+ACN+H ; M+2ACN+2H ; M+2ACN+2H ; M+2ACN+2H ; M+NH4 ; M+NH4 ; M+NH4 ; M+NH4 ; M+ACN+H	C5H10N2O ; C5H10N2O ; C6H14N2O2 ; C3H7NO ; CH4O ; C3H7NO ; C3H7NO ; C6H14N2O2 ; C6H14N2O2 ; C6H14N2O2 ; C5H7NO ; C5H7NO ; C5H7NO ; C5H7NO ; C3H7NO
X389	1	559.2998	0.022	HMDB03141 ; HMDB11728 ; HMDB15266 ; HMDB28888 ; HMDB28889 ; HMDB28909 ; HMDB28931 ; HMDB29321 ; HMDB29560 ; HMDB30153 ; HMDB31802	Retinoyl b-glucuronide ; Bradykinin hydroxyproline ; Doxacurium chloride ; Histidinyl-Isoleucine ; Histidinyl-Leucine ; Isoleucyl-Histidine ; Leucyl-Histidine ; Asparagoside F ; Hesperaline ; Austalide H ; ()-Metalaxyl	M+2ACN+H ; M+ACN+2H ; M+2ACN+2H ; 2M+Na ; 2M+Na ; 2M+Na ; 2M+Na ; M+2ACN+2H ; 2M+Na ; M+2ACN+H ; 2M+H	C26H36O8 ; C50H73N15O12 ; C56H78N2O16 ; C12H20N4O3 ; C12H20N4O3 ; C12H20N4O3 ; C12H20N4O3 ; C50H82O22 ; C14H22NO4 ; C26H36O8 ; C15H21NO4
X39	1	309.07375	0	HMDB29527 ; HMDB29870 ; HMDB29988 ; HMDB30090 ; HMDB30622 ; HMDB30793 ; HMDB30807 ; HMDB30852 ; HMDB31420 ; HMDB31620 ; HMDB32288 ; HMDB33291 ; HMDB33309 ; HMDB34011 ; HMDB34012 ; HMDB34114 ; HMDB34115 ; HMDB34438 ; HMDB37320 ; HMDB37322 ; HMDB37489 ; HMDB38448 ; HMDB40201 ; HMDB41726	Melilotocarpan B ; 37-Dihydroxy-4-methoxyisoflavanone ; Bis(2-furanylmethyl) disulfide ; Sakuranetin ; (R)-Oxypeucedanin ; Moracin B ; 34-Dihydro-8-hydroxy-3-(3-hydroxy-4-methoxyphenyl)-1H-2-benzopyran-1-one ; Asperxanthone ; Heliannone C ; Vestitone ; Furfuryl 2-methyl-3-furyl disulfide ; 38-Dihydroxy-9-methoxypterocarpan ; Moracin F ; 2-(24-Dihydroxyphenyl)-56-dimethoxybenzofuran ; 34-Dihydroxy-9-methoxypterocarpan ; 6alpha-Hydroxyisomedicarpin ; 6alpha-Hydroxymedicarpin ; (R)-Pabulenol ; Licochalcone B ; Dihydrooroxylin ; Dihydrowogonin ; (-)-57-Dihydroxy-3-(4-hydroxybenzyl)-4-chromanone ; 33-Dithiobis2-methylfuran ; Dihydroglycitein	M+Na ; M+Na ; M+2ACN+H ; M+Na ; M+Na ; M+Na ; M+Na ; M+Na ; M+Na ; M+Na ; M+2ACN+H ; M+Na ; M+Na ; M+Na ; M+Na ; M+Na ; M+Na ; M+Na ; M+Na ; M+Na ; M+Na ; M+Na ; M+2ACN+H ; M+Na	C16H14O5 ; C16H14O5 ; C10H10O2S2 ; C16H14O5 ; C16H14O5 ; C16H14O5 ; C16H14O5 ; C16H14O5 ; C16H14O5 ; C16H14O5 ; C10H10O2S2 ; C16H14O5 ; C16H14O5 ; C16H14O5 ; C16H14O5 ; C16H14O5 ; C16H14O5 ; C16H14O5 ; C16H14O5 ; C16H14O5 ; C16H14O5 ; C16H14O5 ; C10H10O2S2 ; C16H14O5
X390	1	580.01645	0.147	HMDB29855 ; HMDB32885	Cochineal Red A ; Amaranth	M+ACN+H ; M+ACN+H	C20H14N2O10S3 ; C20H14N2O10S3

X391	1	527.38058	0.045	HMDB06227 ; HMDB11481 ; HMDB11511 ; HMDB12108 ; HMDB13892 ; HMDB14339 ; HMDB15646 ; HMDB29567 ; HMDB32667 ; HMDB32691 ; HMDB33647 ; HMDB38977 ; HMDB60532	1-a24R25-Trihydroxyvitamin D2 ; LysoPE(0:020:0) ; LysoPE(20:00:0) ; LysoPC(17:0) ; N-Desmethylvenlafaxine ; Tramadol ; Desvenlafaxine ; Hydroxy-alpha-sanshool ; 59-Epidioxy-3-hydroxyergost-7-en-6-one ; (3beta5alpha6alpha7beta14alpha22E24R)-56-Epoxyergosta-822-diene-3714-triol ; 56:89-Diepoxyergost-22-ene-37beta-diol ; (3beta5alpha6beta9alpha22E24R)-59-Epidioxyergosta-722-diene-36-diol ; O-Desmethylvenlafaxine	M+2ACN+H ; M+NH4 ; M+NH4 ; M+NH4 ; 2M+H ; 2M+H ; 2M+H ; 2M+H ; M+2ACN+H ; M+2ACN+H ; M+2ACN+H ; M+2ACN+H ; 2M+H	C28H44O4 ; C25H52NO7P ; C25H52NO7P ; C25H52NO7P ; C16H25NO2 ; C16H25NO2 ; C16H25NO2 ; C16H25NO2 ; C28H44O4 ; C28H44O4 ; C28H44O4 ; C28H44O4 ; C16H25NO2
X392	1	162.05023	0.228	HMDB37110	4-(34-Dihydroxyphenyl)-23-dihydro-23-dihydroxy-1H-phenalen-1-one	M+2H	C19H14O5
X394	1	190.14369	0.2	HMDB36188 ; HMDB36213 ; HMDB36396 ; HMDB40165 ; HMDB40202 ; HMDB40203 ; HMDB59936 ; HMDB59937 ; HMDB60287	Tetrahydrofurfuryl butyrate ; cis-3-Hexenyl lactate ; 3-Methylbutyl 3-oxobutanoate ; Butyl levulinate ; Butyl 3-hydroxy-2-methylidenebutanoate ; 2-Methylpropyl 3-hydroxy-2-methylidenebutanoate ; 5-Pentyl-14-dioxan-2-one ; 6-Pentyl-14-dioxan-2-one ; 4-Hydroperoxy-2-nonenal	M+NH4 ; M+NH4 ; M+NH4 ; M+NH4 ; M+NH4 ; M+NH4 ; M+NH4 ; M+NH4 ; M+NH4	C9H16O3 ; C9H16O3 ; C9H16O3 ; C9H16O3 ; C9H16O3 ; C9H16O3 ; C9H16O3 ; C9H16O3 ; C9H16O3
X395	1	349.13551	0.034	HMDB14359 ; HMDB32766 ; HMDB00854 ; HMDB05005 ; HMDB06028 ; HMDB13930 ; HMDB14011 ; HMDB15552 ; HMDB15553 ; HMDB28786 ; HMDB29080 ; HMDB29450	Torasemide ; N2-(2-Carboxymethyl-2-hydroxysuccinoyl)arginine ; Formiminoglutamic acid ; Tolnaftate ; N-Acetylasparagine ; R-95913 ; 5-O-Desmethyl omeprazole ; Acepromazine ; Aceprometazine ; Cysteinyl-Tryptophan ; Tryptophyl-Cysteine ; Aspergillomarasmine A	M+H ; M+H ; 2M+H ; M+ACN+H ; 2M+H ; M+NH4 ; M+NH4 ; M+Na ; M+Na ; M+ACN+H ; M+ACN+H ; M+ACN+H	C16H20N4O3S ; C12H20N4O8 ; C6H10N2O4 ; C19H17NOS ; C6H10N2O4 ; C18H18FNO2S ; C16H17N3O3S ; C19H22N2OS ; C19H22N2OS ; C14H17N3O3S ; C14H17N3O3S ; C10H17N3O8

X400	1	522.3552	0.133	HMDB02815 ; HMDB10385 ; HMDB15557 ; HMDB32669 ; HMDB35142 ; HMDB35995 ; HMDB39160	LysoPC(18:1(9Z)) ; LysoPC(18:1(11Z)) ; Pheniramine ; (3beta5alpha9alpha22E24R)-359-Trihydroxy-23-methylergosta-722-dien-6-one ; (3beta17alpha23S)-1723-Epoxy-329-dihydroxy-27-norlanost-8-en-24-one ; 2-(Methoxycarbonyl)-5-methyl-24-bis(3-methyl-2-butenyl)-6-(2-methyl-1-oxopropyl)-5-(4-methyl-3-pentenyl)cyclohexanone ; L-Gizzerosine	M+H ; M+H ; 2M+ACN+H ; M+ACN+Na ; M+ACN+Na ; M+ACN+Na ; 2M+ACN+H	C26H52NO7P ; C26H52NO7P ; C16H20N2 ; C29H46O4 ; C29H46O4 ; C29H46O4 ; C11H20N4O2
X401	1	158.09239	0.023	HMDB29848 ; HMDB29849 ; HMDB38706 ; HMDB00123 ; HMDB02144 ; HMDB02271 ; HMDB02820 ; HMDB04988 ; HMDB14691 ; HMDB31239 ; HMDB39111 ; HMDB60496 ; HMDB61163	(-)-trans-Carveol glucoside ; ()-trans-Carveol glucoside ; Perilloside A ; Glycine ; 13-Dimethyluracil ; Imidazolepropionic acid ; Methylimidazoleacetic acid ; Pi-Methylimidazoleacetic acid ; Acetohydroxamic Acid ; Ethyl nitrite ; L-Acetopine ; NN-Diacetylhydrazine ; N-Nitroso-3-hydroxypyrrolidine	M+2H ; M+2H ; M+2H ; M+2ACN+H ; M+NH4 ; M+NH4 ; M+NH4 ; M+NH4 ; M+2ACN+H ; M+2ACN+H ; M+2ACN+2H ; M+ACN+H ; M+ACN+H	C16H26O6 ; C16H26O6 ; C16H26O6 ; C2H5NO2 ; C6H8N2O2 ; C6H8N2O2 ; C6H8N2O2 ; C6H8N2O2 ; C2H5NO2 ; C2H5NO2 ; C8H16N4O4 ; C4H8N2O2 ; C4H8N2O2
X402	1	521.34308	0.106	Unknow	Unknow	Unknow	Unknow
X403	1	144.07117	0.023	HMDB11162 ; HMDB11667 ; HMDB14986 ; HMDB28683 ; HMDB28732 ; HMDB28746 ; HMDB28858	L-beta-aspartyl-L-alanine ; 5-L-Glutamylglycine ; Levamisole ; Alanyl-Aspartate ; Asparaginyl-Hydroxyproline ; Aspartyl-Alanine ; Hydroxyprolyl-Asparagine	M+2ACN+2H ; M+2ACN+2H ; M+2ACN+2H ; M+2ACN+2H ; M+ACN+2H ; M+2ACN+2H ; M+ACN+2H	C7H12N2O5 ; C7H12N2O5 ; C11H12N2S ; C7H12N2O5 ; C9H15N3O5 ; C7H12N2O5 ; C9H15N3O5
X405	1	529.32832	0.035	HMDB15620 ; HMDB35368	Mianserin ; Cytochalasin Ppho	2M+H ; M+NH4	C18H20N2 ; C30H41NO6
X410	1	550.38781	0.039	HMDB10391 ; HMDB11148 ; HMDB39362	LysoPC(20:1(11Z)) ; PC(18:1(9Z)e2:0) ; 37-Dihydroxy-25-methoxycucurbita-523-dien-19-al	M+H ; M+H ; M+ACN+Na	C28H56NO7P ; C28H56NO7P ; C31H50O4
X416	1	210.01179	0.039	Unknow	Unknow	Unknow	Unknow
X421	1	156.51837	0.023	Unknow	Unknow	Unknow	Unknow
X47	1	202.01995	0	HMDB01274 ; HMDB37112	dTDP ; Acifluorfen	M+2H ; M+ACN+2H	C10H16N2O11P2 ; C14H7ClF3NO5
X48	1	279.19185	0	HMDB40904	Ipomeatetrahydrofuran	M+Na	C15H28O3
X56	1	417.14154	0	HMDB34157 ; HMDB03920 ; HMDB15011 ; HMDB30265 ; HMDB30322 ; HMDB30375 ; HMDB33445	2-Hydroxybenzaldehyde O-xylosyl-(1-6)-glucoside ; Protopine ; Loteprednol ; 22-Dimethyl(pyrano-56:3:4)-15-dihydroxy-6-methoxy-10-methylacridone ; Papaveraldine ; Citracridone I ; Honyumine	M+H ; M+ACN+Na ; M+Na ; M+ACN+Na ; M+ACN+Na ; M+ACN+Na ; M+ACN+Na	C18H24O11 ; C20H19NO5 ; C21H27ClO5 ; C20H19NO5 ; C20H19NO5 ; C20H19NO5 ; C20H19NO5

X59	1	368.06025	0	HMDB01570 ; HMDB06095	Thymidine 35-cyclic monophosphate ; 3-Methylsulfinylpropyl isothiocyanate	M+ACN+Na ; 2M+ACN+H	C10H13N2O7P ; C5H9NOS2
X60	1	719.1139	0	Unknow	Unknow	Unknow	Unknow
X61	1	627.08357	0	HMDB60031	5-(35-Dihydroxyphenyl)-gamma-valerolactone-O-sulphate-O-methyl	2M+Na	C12H14O7S
X64	1	426.02376	0	Unknow	Unknow	Unknow	Unknow
X71	1	425.60289	0	Unknow	Unknow	Unknow	Unknow
X73	1	220.04403	0	HMDB30000 ; HMDB37553 ; HMDB39940	Bissulfine ; SS-Ethylidene dithioacetate ; S-2-Propenyl 2-propene-1-sulfonothioate	M+ACN+H ; M+ACN+H ; M+ACN+H	C6H10O2S2 ; C6H10O2S2 ; C6H10O2S2
X82	1	293.55245	0	Unknow	Unknow	Unknow	Unknow
X84	1	321.50462	0	Unknow	Unknow	Unknow	Unknow
X90	1	579.09823	0	Unknow	Unknow	Unknow	Unknow
X93	1	438.07974	0	Unknow	Unknow	Unknow	Unknow
X96	1	437.0763	0	Unknow	Unknow	Unknow	Unknow
X97	1	504.00963	0	HMDB45658 ; HMDB46233 ; HMDB46405 ; HMDB46981 ; HMDB47124	TG(20:024:0o-18:0) ; TG(20:0o-18:024:0) ; TG(22:022:0o-18:0) ; TG(22:0o-18:022:0) ; TG(24:020:0o-18:0)	M+H+NH4 ; M+H+NH4 ; M+H+NH4 ; M+H+NH4 ; M+H+NH4	C65H128O5 ; C65H128O5 ; C65H128O5 ; C65H128O5 ; C65H128O5
X173	2	576.12582	0	Unknow	Unknow	Unknow	Unknow
X207	2	597.96795	0	HMDB39116	Acid yellow 23	M+ACN+Na	C16H9N4Na3O9S2
X17	3	239.10292	0	HMDB15084 ; HMDB28853 ; HMDB29105 ; HMDB29451 ; HMDB40988 ; HMDB35635 ; HMDB00181 ; HMDB00609 ; HMDB01858 ; HMDB02048 ; HMDB02055 ; HMDB03119 ; HMDB29510 ; HMDB29610 ; HMDB33895 ; HMDB34436 ; HMDB38336 ; HMDB38750 ; HMDB38990 ; HMDB38991 ; HMDB40833 ; HMDB59731 ; HMDB60833	Felbamate ; Glycyl-Tyrosine ; Tyrosyl-Glycine ; gamma-Glutaminyl-4-hydroxybenzene ; Falimint ; Kanokoside A ; L-Dopa ; DL-Dopa ; p-Cresol ; m-Cresol ; o-Cresol ; Benzyl alcohol ; Garcinone B ; Ascladiol ; Anisole ; Rotenone ; 2-Hydroxy-3-(34-dihydroxyphenyl)propanamide ; N-Hydroxy-L-tyrosine ; Methyl 5-hydroxyoxindole-3-acetate ; Methyl dioxindole-3-acetate ; 3-4-Hydroxy-3-(3-methyl-2-butenyl)phenyl-2-propenal ; 23-Methyleneglutaric acid ; N-Acetylserotonin glucuronide	M+H ; M+H ; M+H ; M+H ; M+H ; M+2H ; M+ACN+H ; M+ACN+H ; 2M+Na ; 2M+Na ; 2M+Na ; 2M+Na ; M+2ACN+2H ; M+2ACN+H ; 2M+Na ; M+2ACN+2H ; M+ACN+H ; M+ACN+H ; M+NH4 ; M+NH4 ; M+Na ; M+2ACN+H ; M+2ACN+2H	C11H14N2O4 ; C11H14N2O4 ; C11H14N2O4 ; C11H14N2O4 ; C11H14N2O4 ; C21H32O12 ; C9H11NO4 ; C9H11NO4 ; C7H8O ; C7H8O ; C7H8O ; C7H8O ; C23H22O6 ; C7H8O4 ; C7H8O ; C23H22O6 ; C9H11NO4 ; C9H11NO4 ; C11H11NO4 ; C11H11NO4 ; C14H16O2 ; C7H8O4 ; C18H22N2O8
X26	3	313.01712	0	HMDB04812	25-Furandicarboxylic acid	2M+H	C6H4O5
X29	3	357.03708	0	HMDB06462 ; HMDB15511 ; HMDB60636	Homocysteinesulfinic acid ; Bromazepam ; Malathion dicarboxylic acid	2M+Na ; M+ACN+H ; M+2ACN+H	C4H9NO4S ; C14H10BrN3O ; C6H11O6PS2
X335	3	140.01329	0.241	HMDB42046 ; HMDB02287 ; HMDB31188	Treosulfan ; Homocysteine thiolactone ; Ethanethioic acid	M+2H ; M+Na ; M+ACN+Na	C6H14O8S2 ; C4H7NOS ; C2H4OS
X357	3	149.01856	0.133	HMDB14406	Anagrelide	M+ACN+2H	C10H7Cl2N3O
X422	3	86.0965	0.101	HMDB34301 ; HMDB31630	Piperidine ; Propane	M+H ; M+ACN+H	C5H11N ; C3H8

X49	3	148.52369	0	Unknow	Unknow	Unknow	Unknow
X50	3	140.52219	0	Unknow	Unknow	Unknow	Unknow
X62	3	162.03753	0	Unknow	Unknow	Unknow	Unknow
X63	3	604.62922	0	Unknow	Unknow	Unknow	Unknow
X68	3	402.51378	0	Unknow	Unknow	Unknow	Unknow
X79	3	301.11436	0	HMDB00098 ; HMDB00283 ; HMDB00366 ; HMDB00621 ; HMDB00646 ; HMDB00751 ; HMDB01644 ; HMDB03162 ; HMDB03371 ; HMDB11112 ; HMDB11176 ; HMDB12153 ; HMDB12194 ; HMDB12325 ; HMDB13067 ; HMDB13141 ; HMDB28996 ; HMDB29029 ; HMDB29113 ; HMDB29578 ; HMDB29941 ; HMDB29942 ; HMDB31161 ; HMDB31472 ; HMDB31709 ; HMDB32180 ; HMDB32408 ; HMDB32775 ; HMDB33192 ; HMDB38174 ; HMDB38463 ; HMDB40056 ; HMDB41934 ; HMDB59753 ; HMDB59856 ; HMDB60254	D-Xylose ; D-Ribose ; 2-Deoxyribonic acid ; D-Ribulose ; L-Arabinose ; L-Threo-2-pentulose ; D-Xylulose ; 7-Methylhypoxanthine ; L-Ribulose ; N1-(alpha-D-ribosyl)-56-dimethyl-benzimidazole ; L-phenylalanyl-L-hydroxyproline ; 34-Dihydroxybenzylamine ; Beta-D-ribopyranose ; Arabinofuranose ; Salsoline-1-carboxylate ; 1-Methylhypoxanthine ; Phenylalanyl-Hydroxyproline ; Prolyl-Tyrosine ; Tyrosyl-Proline ; Diisopropyl disulfide ; D-Apiose ; Arabinose ; Methyl pentyl disulfide ; Dipropyl disulfide ; 16-Hexanedithiol ; Butyl ethyl disulfide ; Methyl isopentyl disulfide ; 3-Hydroxy-carbofuran ; Neoacrimarine K ; 5-Acetyl-24-dimethyloxazole ; 3-(Isothiocyanatomethyl)-1-methoxy-1H-indole ; xi-1-(Propylthio)-1-propanethiol ; Mizoribine ; 2-Deoxypentonic acid ; Ethyl 2-pyrrolecarboxylate ; Aldehydo-D-xylose	2M+H ; 2M+H ; 2M+H ; 2M+H ; 2M+H ; 2M+H ; 2M+H ; 2M+H ; 2M+H ; M+Na ; M+Na ; 2M+Na ; 2M+H ; 2M+H ; M+ACN+Na ; 2M+H ; M+Na ; M+Na ; M+Na ; 2M+H ; 2M+H ; 2M+H ; 2M+H ; 2M+H ; 2M+H ; 2M+H ; 2M+H ; M+ACN+Na ; M+ACN+2H ; 2M+Na ; M+2ACN+H ; 2M+H ; M+ACN+H ; 2M+H ; 2M+Na ; 2M+H	C5H10O5 ; C5H10O5 ; C5H10O5 ; C5H10O5 ; C5H10O5 ; C5H10O5 ; C5H10O5 ; C6H6N4O ; C5H10O5 ; C14H18N2O4 ; C14H18N2O4 ; C7H9NO2 ; C5H10O5 ; C5H10O5 ; C12H15NO4 ; C6H6N4O ; C14H18N2O4 ; C14H18N2O4 ; C14H18N2O4 ; C6H14S2 ; C5H10O5 ; C5H10O5 ; C6H14S2 ; C6H14S2 ; C6H14S2 ; C6H14S2 ; C6H14S2 ; C12H15NO4 ; C31H29NO9 ; C7H9NO2 ; C11H10N2OS ; C6H14S2 ; C9H13N3O6 ; C5H10O5 ; C7H9NO2 ; C5H10O5
X85	3	331.97809	0	Unknow	Unknow	Unknow	Unknow
X91	3	231.93655	0	Unknow	Unknow	Unknow	Unknow

X1	4	225.08686	0	HMDB00732 ; HMDB11631 ; HMDB12819 ; HMDB14787 ; HMDB29397 ; HMDB00017 ; HMDB00978 ; HMDB02349 ; HMDB02432 ; HMDB06331 ; HMDB15249 ; HMDB29832 ; HMDB30080 ; HMDB30816 ; HMDB31663 ; HMDB32923 ; HMDB32988 ; HMDB33882 ; HMDB36556 ; HMDB38674 ; HMDB59762 ; HMDB60328	Hydroxykynurenine ; L-3-Hydroxykynurenine ; 5-Hydroxykynurenine ; Stavudine ; L-Nicotianine ; 4-Pyridoxic acid ; 4-(2-Aminophenyl)-24-dioxobutanoic acid ; trans-trans-Muconic acid ; Sumikis acid ; ciscis-Muconic acid ; Atovaquone ; 8-Carboxymethyldihydrochelerythrine ; Wampetin ; 6-Acetyl-22-dimethyl-2H-1-benzopyran ; 3-(3-Methylbutylidene)-1(3H)-isobenzofuranone ; Kojic acid ; 5-Hydroxymaltol ; Glycyrol ; Glycyrrhizaisoflavone B ; gamma-L-Glutamyl-gamma-L-glutamyl-L-methionine ; 23-Methylenesuccinic acid ; 1-Nitro-56-dihydroxy-dihydronaphthalene	M+H ; M+H ; M+H ; M+H ; M+H ; M+ACN+H ; M+NH4 ; M+2ACN+H ; M+2ACN+H ; M+2ACN+H ; M+2ACN+2H ; M+ACN+2H ; M+2ACN+2H ; M+Na ; M+Na ; M+2ACN+H ; M+2ACN+H ; M+2ACN+2H ; M+2ACN+2H ; M+ACN+2H ; M+2ACN+H ; M+NH4	C10H12N2O4 ; C10H12N2O4 ; C10H12N2O4 ; C10H12N2O4 ; C10H12N2O4 ; C8H9NO4 ; C10H9NO4 ; C6H6O4 ; C6H6O4 ; C6H6O4 ; C22H19ClO3 ; C23H21NO6 ; C21H18O6 ; C13H14O2 ; C13H14O2 ; C6H6O4 ; C6H6O4 ; C21H18O6 ; C21H18O6 ; C15H25N3O8S ; C6H6O4 ; C10H9NO4
X10	4	238.09912	0	HMDB15352 ; HMDB60711 ; HMDB13970 ; HMDB34249 ; HMDB35030 ; HMDB38475 ; HMDB40977 ; HMDB41484	Ketamine ; 2-Amino-5-benzoylbenzimidazole ; Dehydroxyzyleuton ; Caryoptosidic acid ; Amygdalin ; Mandelonitrile sophoroside ; Brassitin ; 5-Acetyl-24-dimethylthiazole	M+H ; M+H ; M+NH4 ; M+2ACN+2H ; M+H+NH4 ; M+H+NH4 ; M+NH4 ; M+2ACN+H	C13H16ClNO ; C14H11N3O ; C11H12N2OS ; C16H24O11 ; C20H27NO11 ; C20H27NO11 ; C11H12N2OS ; C7H9NOS
X12	4	181.07939	0	HMDB34626 ; HMDB34627 ; HMDB37028 ; HMDB38381 ; HMDB39473 ; HMDB28764 ; HMDB29079 ; HMDB33118 ; HMDB33119 ; HMDB34367 ; HMDB37285 ; HMDB38445 ; HMDB40034	1-(34-Dimethoxyphenyl)-12-ethanediol 1-O-b-D-glucoside ; 1-(34-Dimethoxyphenyl)-12-ethanediol 2-O-b-D-glucoside ; Deoxyloganic acid ; 2-(4-Hydroxy-35-dimethoxyphenyl)ethanol 4-glucoside ; 2-Methoxy-3-(24-dihydroxyphenyl)-12-propanediol 4-glucoside ; Aspartyl-Tryptophan ; Tryptophyl-Aspartate ; 2-Methylthiophene ; 3-Methylthiophene ; N-gamma-L-Glutamyl-L-methionine ; 24-Dimethyl-5-vinylthiazole ; 1-Isothiocyanato-2-phenylethane ; 2-Ethylbenzothiazole	M+2H ; M+2H ; M+2H ; M+2H ; M+2H ; M+ACN+2H ; M+ACN+2H ; M+2ACN+H ; M+2ACN+H ; M+2ACN+2H ; M+ACN+H ; M+NH4 ; M+NH4	C16H24O9 ; C16H24O9 ; C16H24O9 ; C16H24O9 ; C16H24O9 ; C15H17N3O5 ; C15H17N3O5 ; C5H6S ; C5H6S ; C10H18N2O5S ; C7H9NS ; C9H9NS ; C9H9NS
X13	4	125.01524	0	Unknow	Unknow	Unknow	Unknow

X14	4	262.07814	0	HMDB28752 ; HMDB28815 ; HMDB33705 ; HMDB37336 ; HMDB15188	Aspartyl-Glutamate ; Glutamyl-Aspartate ; Salviaflaside ; Sudachiin A ; Mimosine	M+H ; M+H ; M+2H ; M+2H ; M+ACN+Na	C9H13N2O7 ; C9H13N2O7 ; C24H26O13 ; C24H26O13 ; C8H10N2O4
X142	4	209.05412	0	HMDB00232 ; HMDB01552 ; HMDB33161 ; HMDB38421 ; HMDB38426 ; HMDB60260	Quinolinic acid ; 2-Keto-glutaramic acid ; 26-Pyridinedicarboxylic acid ; 2-Methylpropyl glucosinolate ; Butyl glucosinolate ; Urate radical	M+ACN+H ; M+ACN+Na ; M+ACN+H ; M+ACN+2H ; M+ACN+2H ; M+ACN+H	C7H5NO4 ; C5H7NO4 ; C7H5NO4 ; C11H21NO9S2 ; C11H21NO9S2 ; C5H3N4O3
X149	4	191.04387	0	HMDB15491 ; HMDB06273 ; HMDB12268 ; HMDB13159 ; HMDB34368 ; HMDB59778	Nitroxoline ; 5-amino-1-(5-phospho-D-ribosyl)imidazole-4-carboxylate ; N5-Carboxyaminoimidazole ribonucleotide ; 23-Diaminosalicylic acid ; Hydroxyminaline ; 2-Pyrroloylglycine	M+H ; M+ACN+2H ; M+ACN+2H ; M+Na ; M+ACN+Na ; M+Na	C9H6N2O3 ; C9H14N3O9P ; C9H14N3O9P ; C7H8N2O3 ; C5H5NO3 ; C7H8N2O3
X15	4	207.03858	0	HMDB01933 ; HMDB13636 ; HMDB14675	Furosemide ; Pyrroloquinoline quinone ; Cefdinir	M+2ACN+2H ; M+2ACN+2H ; M+H+NH4	C12H11ClN2O5S ; C14H6N2O8 ; C14H13N5O5S2
X155	4	152.06226	0	HMDB60116 ; HMDB30277 ; HMDB34857 ; HMDB35149 ; HMDB39127 ; HMDB59775	Dibenzoalpyrene ; Dihydromaleimide beta-D-glucoside ; 2-(2-Thienylmethylene)-16-dioxaspiro44non-3-ene ; Chrycorin ; Pisatoside ; 3-Hydroxy-3-carboxymethyl-adipic acid	M+2H ; M+ACN+2H ; M+2ACN+2H ; M+2ACN+2H ; M+ACN+2H ; M+2ACN+2H	C24H14 ; C10H15NO7 ; C12H12O2S ; C12H12O2S ; C10H15NO7 ; C8H12O7
X157	4	165.02797	0	HMDB00469 ; HMDB13974 ; HMDB13976 ; HMDB60542	5-Hydroxymethyluracil ; 4-Hydroxydiclofenac ; 3-Hydroxydiclofenac ; 5-Hydroxydiclofenac	M+Na ; M+H+NH4 ; M+H+NH4 ; M+H+NH4	C5H6N2O3 ; C14H11Cl2NO3 ; C14H11Cl2NO3 ; C14H11Cl2NO3
X159	4	165.01016	0	Unknow	Unknow	Unknow	Unknow
X163	4	154.07771	0	HMDB00235 ; HMDB38663	Thiamine ; D-1-(3-Carboxypropyl)amino-1-deoxyfructose	M+ACN+2H ; M+ACN+2H	C12H17N4OS ; C10H19NO7
X179	4	266.18601	0	HMDB14573 ; HMDB15095 ; HMDB15239 ; HMDB15584 ; HMDB35707 ; HMDB36416 ; HMDB36796 ; HMDB38125 ; HMDB59676 ; HMDB59857 ; HMDB59878 ; HMDB59910	Carboprost Tromethamine ; Pindolol ; Methyprylon ; Maraviroc ; (6alpha7alpha10alpha)-1(5)3-Aromadendradiene ; beta-Spathulene ; (1alpha6alpha7alphaH)-24(15)-Copadiene ; 149-Cadinatriene ; beta-Vatirenene ; beta-Vetivenene ; alpha-Curcumene ; (E)-Calamene	M+ACN+2H ; M+NH4 ; M+2ACN+H ; M+H+NH4 ; M+ACN+Na ; M+ACN+Na ; M+ACN+Na ; M+ACN+Na ; M+ACN+Na ; M+ACN+Na ; M+ACN+Na ; M+ACN+Na	C25H47NO8 ; C14H20N2O2 ; C10H17NO2 ; C29H41F2N5O ; C15H22 ; C15H22 ; C15H22 ; C15H22 ; C15H22 ; C15H22 ; C15H22 ; C15H22
X182	4	206.05458	0	Unknow	Unknow	Unknow	Unknow

X186	4	181.06893	0	HMDB00122 ; HMDB00143 ; HMDB00169 ; HMDB00211 ; HMDB00346 ; HMDB00516 ; HMDB00660 ; HMDB01151 ; HMDB01266 ; HMDB03345 ; HMDB03418 ; HMDB03449 ; HMDB06088 ; HMDB12326 ; HMDB32222 ; HMDB33704 ; HMDB34220 ; HMDB35051 ; HMDB40910 ; HMDB41196 ; HMDB00128 ; HMDB00190 ; HMDB00700 ; HMDB01051 ; HMDB01311 ; HMDB01882 ; HMDB14848 ; HMDB29580 ; HMDB31232 ; HMDB41200 ; HMDB41929 ; HMDB60980	D-Glucose ; D-Galactose ; D-Mannose ; Myoinositol ; 3-Deoxyarabinohexonic acid ; Beta-D-Glucose ; D-Fructose ; Allose ; L-Sorbose ; Alpha-D-Glucose ; D-Tagatose ; Beta-D-Galactose ; Scyllitol ; L-Gulose ; Dihydroxyacetone (dimer) ; L-Galactose ; Levoinositol ; Gibberellin A59 ; Heteroflavanone A ; Edulisin IV ; Guanidoacetic acid ; L-Lactic acid ; Hydroxypropionic acid ; Glyceraldehyde ; D-Lactic acid ; Dihydroxyacetone ; Ibandronate ; Dimethyl carbonate ; Monoethyl carbonate ; Dehydroxymethylflazine ; Methoxyacetic acid ; N-desmethylrosiglitazone	M+H ; M+H ; M+H ; M+H ; M+H ; M+H ; M+H ; M+H ; M+H ; M+H ; M+H ; M+H ; M+H ; M+H ; M+H ; M+H ; M+H ; M+2H ; M+2H ; M+2H ; M+ACN+Na ; 2M+H ; 2M+H ; 2M+H ; 2M+H ; 2M+H ; M+ACN+2H ; 2M+H ; 2M+H ; M+2ACN+2H ; 2M+H ; M+H+NH4	C6H12O6 ; C6H12O6 ; C6H12O6 ; C6H12O6 ; C6H12O6 ; C6H12O6 ; C6H12O6 ; C6H12O6 ; C6H12O6 ; C6H12O6 ; C6H12O6 ; C6H12O6 ; C6H12O6 ; C6H12O6 ; C6H12O6 ; C6H12O6 ; C6H12O6 ; C19H20O7 ; C19H20O7 ; C19H20O7 ; C3H7N3O2 ; C3H6O3 ; C3H6O3 ; C3H6O3 ; C3H6O3 ; C3H6O3 ; C9H23NO7P2 ; C3H6O3 ; C3H6O3 ; C16H10N2O3 ; C3H6O3 ; C17H17N3O3S
X189	4	138.99425	0	HMDB12974	Hypothiocyanite	M+ACN+Na	CHNOS
X190	4	126.10265	0	HMDB00898 ; HMDB01861 ; HMDB31849 ; HMDB32971 ; HMDB35248 ; HMDB35289	1-Methylhistamine ; 3-Methylhistamine ; 2-Ethylpyrazine ; 23-Dimethylpyrazine ; 26-Dimethylpyrazine ; 25-Dimethylpyrazine	M+H ; M+H ; M+NH4 ; M+NH4 ; M+NH4 ; M+NH4	C6H11N3 ; C6H11N3 ; C6H8N2 ; C6H8N2 ; C6H8N2 ; C6H8N2
X193	4	260.08101	0	HMDB38776 ; HMDB32658	Medicarpin 3-O-(6-malonylglucoside) ; 7-Chloro-334568-hexamethoxyflavone	M+2H ; M+2ACN+2H	C25H26O12 ; C21H21ClO8
X195	4	240.1063	0	HMDB32293 ; HMDB32771 ; HMDB32774 ; HMDB02706 ; HMDB10325 ; HMDB14761 ; HMDB36134 ; HMDB40565 ; HMDB40897 ; HMDB41936 ; HMDB41937 ; HMDB60824 ; HMDB60930 ; HMDB60934 ; HMDB60935	N-Gluconyl ethanolamine ; Eremopetasitenin C2 ; Eremopetasitenin D2 ; Canavanine ; Ethyl glucuronide ; Fluphenazine ; 4-(2-Furanylmethyl)thio-2-pentanone ; Aloesol 7-glucoside ; Methyl (3x4E10R)-310-dihydroxy-411-dodecadiene-68-diynoate 10-glucoside ; Morphine-3-glucuronide ; Morphine-6-glucuronide ; Hydromorphone-3-glucuronide ; Hydromorphone 3-beta-O-glucuronide ; 3-cis-Hydroxyglipizide ; 4-trans-Hydroxyglipizide	M+H ; M+2H ; M+2H ; M+ACN+Na ; M+NH4 ; M+ACN+2H ; M+ACN+H ; M+2ACN+2H ; M+2ACN+2H ; M+H+NH4 ; M+H+NH4 ; M+H+NH4 ; M+H+NH4 ; M+H+NH4 ; M+H+NH4	C8H17NO7 ; C25H34O7S ; C25H34O7S ; C5H12N4O3 ; C8H14O7 ; C22H26F3N3OS ; C10H14O2S ; C19H24O9 ; C19H24O9 ; C23H27NO9 ; C23H27NO9 ; C23H27NO9 ; C23H27NO9 ; C21H27N5O5S ; C21H27N5O5S
X2	4	164.03423	0	Unknow	Unknow	Unknow	Unknow

X20	4	226.08099	0	HMDB35067 ; HMDB15526 ; HMDB28682 ; HMDB28724 ; HMDB28797 ; HMDB28839 ; HMDB28850 ; HMDB28855 ; HMDB29039 ; HMDB29149 ; HMDB29424	Melleolide L ; Ketazolam ; Alanyl-Asparagine ; Asparaginyl-Alanine ; Glutaminyl-Glycine ; Glycyl-Glutamine ; Glycyl-Serine ; Glycyl-Gamma-glutamate ; Serinyl-Glycine ; Gamma-glutamyl-Glycine ; L-4-Hydroxyglutamine	M+2H ; M+2ACN+2H ; M+Na ; M+Na ; M+Na ; M+Na ; M+ACN+Na ; M+Na ; M+ACN+Na ; M+Na ; M+ACN+Na	C23H27ClO7 ; C20H17ClN2O3 ; C7H13N3O4 ; C7H13N3O4 ; C7H13N3O4 ; C7H13N3O4 ; C5H10N2O4 ; C7H13N3O4 ; C5H10N2O4 ; C7H13N3O4 ; C5H10N2O4
X205	4	277.10239	0	HMDB11737 ; HMDB42036 ; HMDB00127 ; HMDB02545 ; HMDB02704 ; HMDB03363 ; HMDB03402 ; HMDB06334 ; HMDB11731 ; HMDB11732 ; HMDB13204 ; HMDB32796 ; HMDB33245 ; HMDB38491 ; HMDB41503	Gamma Glutamylglutamic acid ; Thymidine glycol ; D-Glucuronic acid ; Galacturonic acid ; Iduronic acid ; Pectic acid ; Pectin ; 3-Dehydro-L-gulonate ; 5-Keto-D-gluconate ; 2-Keto-L-gluconate ; 6-Succinoaminopurine ; Methyl 3-(23-dihydroxy-3-methylbutyl)-4-hydroxybenzoate ; 6-(Hydroxymethyl)-24(1H3H)-pteridinedione ; L-Altruronic acid ; Bis(2-furanylmethyl) sulfide	M+H ; M+H ; M+2ACN+H ; M+2ACN+H ; M+2ACN+H ; M+2ACN+H ; M+2ACN+H ; M+2ACN+H ; M+2ACN+H ; M+2ACN+H ; M+ACN+H ; M+Na ; M+2ACN+H ; M+2ACN+H ; M+2ACN+H	C10H16N2O7 ; C10H16N2O7 ; C6H10O7 ; C6H10O7 ; C6H10O7 ; C6H10O7 ; C6H10O7 ; C6H10O7 ; C6H10O7 ; C6H10O7 ; C9H9N5O3 ; C13H18O5 ; C7H6N4O3 ; C6H10O7 ; C10H10O2S
X208	4	492.58774	0	Unknow	Unknow	Unknow	Unknow
X21	4	225.08699	0	Unknow	Unknow	Unknow	Unknow
X211	4	147.09167	0	HMDB03701 ; HMDB29740 ; HMDB30386 ; HMDB30906 ; HMDB30907 ; HMDB30908 ; HMDB34658 ; HMDB34738 ; HMDB34930 ; HMDB35687 ; HMDB36693 ; HMDB39275 ; HMDB41249 ; HMDB11664 ; HMDB15656 ; HMDB33731 ; HMDB34244 ; HMDB60956	Dimethylbenzimidazole ; 1H-Indole-3-methanamine ; Myosmine ; 14-Dimethylpyrrolo12-apyrazine ; 34-Dimethylpyrrolo12-apyrazine ; 13-Dimethylpyrrolo12-apyrazine ; 9-Acetoxyfukinanolide ; Isotrichodermin ; (3beta6beta)-Furanoeremophilane-36-diol 6-acetate ; Acetylvalerenolic acid ; Acetylbalchanolide ; 6-Gingerdione ; 6-Hydroxyshogaol ; 3-Methylene-indolenine ; Naphazoline ; Quinoline ; Isoquinoline ; N-desmethylmirtazapine	M+H ; M+H ; M+H ; M+H ; M+H ; M+H ; M+2H ; M+2H ; M+2H ; M+2H ; M+2H ; M+2H ; M+2H ; M+NH4 ; M+2ACN+2H ; M+NH4 ; M+NH4 ; M+ACN+2H	C9H10N2 ; C9H10N2 ; C9H10N2 ; C9H10N2 ; C9H10N2 ; C9H10N2 ; C17H24O4 ; C17H24O4 ; C17H24O4 ; C17H24O4 ; C17H24O4 ; C17H24O4 ; C17H24O4 ; C9H7N ; C14H14N2 ; C9H7N ; C9H7N ; C16H17N3
X214	4	614.61609	0	Unknow	Unknow	Unknow	Unknow
X223	4	486.06489	0	HMDB00536 ; HMDB41173 ; HMDB59653	Adenylsuccinic acid ; 1-O-Caffeoyl-(b-D-glucose 6-O-sulfate) ; N(6)-(12-dicarboxyethyl)AMP	M+Na ; M+ACN+Na ; M+Na	C14H18N5O11P ; C15H18O12S ; C14H18N5O11P
X228	4	349.03456	0	Unknow	Unknow	Unknow	Unknow
X238	4	330.56287	0	Unknow	Unknow	Unknow	Unknow

X245	4	308.89995	0	Unknow	Unknow	Unknow	Unknow
X254	4	460.14968	0	Unknow	Unknow	Unknow	Unknow
X255	4	439.07257	0	HMDB14580 ; HMDB34425 ; HMDB40242	Bendroflumethiazide ; ()-1-Methylpropyl 3-(methylthio)-2-propenyl disulfide ; 3-Methyl-5-pentyl-124-trithiolane	M+NH4 ; 2M+Na ; 2M+Na	C15H14F3N3O4S2 ; C8H16S3 ; C8H16S3
X266	4	270.94422	0	Unknow	Unknow	Unknow	Unknow
X267	4	284.02927	0	HMDB06049 ; HMDB13926	O-Phosphotyrosine ; Dehydrogenated ticlopidine	M+Na ; M+Na	C9H12NO6P ; C14H12ClNS
X283	4	268.94586	0	Unknow	Unknow	Unknow	Unknow
X3	4	116.06208	0	HMDB12286 ; HMDB31717 ; HMDB32271 ; HMDB32410 ; HMDB32418 ; HMDB32422 ; HMDB32495 ; HMDB36193 ; HMDB37619 ; HMDB38299 ; HMDB39653 ; HMDB40413 ; HMDB41306	S-Prenyl-L-cysteine ; 3-(Methylthio)propyl acetate ; Ethyl 3-mercaptobutyrate ; Methyl 3-(methylthio)butanoate ; 2-(4-Methyl-5-thiazolyl)ethyl butanoate ; 2-(4-Methyl-5-thiazolyl)ethyl isobutyrate ; Propyl 2-mercaptopropionate ; 3-Mercapto-3-methylbutyl formate ; Methyl 4-(methylthio)butyrate ; Methylthiomethyl butyrate ; 246-Trimethyl-135-dioxathiane ; Ethyl 3-(methylthio)propanoate ; Methyl 2-(methylthio)butyrate	M+ACN+2H ; M+2ACN+2H ; M+2ACN+2H ; M+2ACN+2H ; M+H+NH4 ; M+H+NH4 ; M+2ACN+2H ; M+2ACN+2H ; M+2ACN+2H ; M+2ACN+2H ; M+2ACN+2H ; M+2ACN+2H ; M+2ACN+2H	C8H15NO2S ; C6H12O2S ; C6H12O2S ; C6H12O2S ; C10H15NO2S ; C10H15NO2S ; C6H12O2S ; C6H12O2S ; C6H12O2S ; C6H12O2S ; C6H12O2S ; C6H12O2S ; C6H12O2S
X320	4	538.38642	0.091	HMDB11490 ; HMDB11520 ; HMDB32228 ; HMDB34505 ; HMDB34528 ; HMDB34644 ; HMDB34683 ; HMDB35326 ; HMDB36221 ; HMDB36640 ; HMDB37713 ; HMDB37719 ; HMDB39692	LysoPE(0:022:0) ; LysoPE(22:00:0) ; Dimethylbenzyl carbinyl hexanoate ; Soyasapogenol A ; Camelliagenin A ; Priverogenin B ; (3alphaOH20S24S)-319:2024-Diepoxydammarane-325-diol ; Ganoderiol A ; 2-(Dimethoxymethyl)-1-heptenylbenzene ; Furanofukinin ; Octyl phenylacetate ; 2-Phenylethyl octanoate ; 2024-Epoxy-2526-dihydroxydammaran-3-one	M+H ; M+H ; 2M+ACN+H ; M+ACN+Na ; M+ACN+Na ; M+ACN+Na ; M+ACN+Na ; M+ACN+Na ; 2M+ACN+H ; 2M+ACN+H ; 2M+ACN+H ; 2M+ACN+H ; M+ACN+Na	C27H56NO7P ; C27H56NO7P ; C16H24O2 ; C30H50O4 ; C30H50O4 ; C30H50O4 ; C30H50O4 ; C30H50O4 ; C16H24O2 ; C16H24O2 ; C16H24O2 ; C16H24O2 ; C30H50O4
X321	4	530.28653	0.086	HMDB40945 ; HMDB40963	Helianthoside A ; Matesaponin 2	M+2H ; M+2H	C53H86O21 ; C53H86O21
X345	4	499.34818	0.125	HMDB10381 ; HMDB11129 ; HMDB11130 ; HMDB15004 ; HMDB30271 ; HMDB60852 ; HMDB60997 ; HMDB61007	LysoPC(15:0) ; LysoPE(0:018:0) ; LysoPE(18:00:0) ; Alprenolol ; Dehydrocarpaine I ; NO-Didesmethylvenlafaxine ; O-Desmethyltramadol ; N-Desmethyltramadol	M+NH4 ; M+NH4 ; M+NH4 ; 2M+H ; M+Na ; 2M+H ; 2M+H ; 2M+H	C23H48NO7P ; C23H48NO7P ; C23H48NO7P ; C15H23NO2 ; C28H48N2O4 ; C15H23NO2 ; C15H23NO2 ; C15H23NO2

X350	4	497.34284	0.111	Unknow	Unknow	Unknow	Unknow
X356	4	511.35847	0.055	HMDB14930 ; HMDB31867 ; HMDB39588	Tripelennamine ; 4-Acetyl-6-tert-butyl-11-dimethylindane ; Panaxynol	2M+H ; 2M+Na ; 2M+Na	C16H21N3 ; C17H24O ; C17H24O
X358	4	171.09579	0.071	HMDB01091 ; HMDB40799 ; HMDB04995 ; HMDB15091 ; HMDB30208 ; HMDB30212 ; HMDB30248 ; HMDB30255 ; HMDB30420 ; HMDB31653 ; HMDB32341 ; HMDB32962 ; HMDB38433 ; HMDB38614 ; HMDB38647 ; HMDB38676 ; HMDB38725 ; HMDB38973 ; HMDB39767 ; HMDB39807 ; HMDB59843 ; HMDB60319 ; HMDB60642 ; HMDB60846 ; HMDB60960	3-Hydroxyquinine ; 11-Hydroxytubotaiwine ; Codeine ; Hydrocodone ; Koenigicine ; Mukonicine ; Neopine ; Erysodine ; Strobilurin A ; 3-(Methylthio)-1-propene ; Isoamyl isothiocyanate ; Secoclausenamide ; 1-Isothiocyanatopentane ; gamma-L-Glutamyl-L-pipecolic acid ; (2E6E)-Piperamide-C7:2 ; (2S2S)-Pyrosaccharopine ; (R)-Juziphine ; 25-Dihydro-245-trimethylthiazole ; Pandamarilactone 32 ; cis-23-Dimethylthiirane ; (1E)-1-(methylthio)prop-1-ene ; (S)-N-Methylcoclaurine ; N-depropylpropafenone ; N-Monodemethylolopatadine ; N-desalkylpropafenone	M+2H ; M+2H ; M+ACN+2H ; M+ACN+2H ; M+H+NH4 ; M+H+NH4 ; M+ACN+2H ; M+ACN+2H ; M+2ACN+2H ; M+2ACN+H ; M+ACN+H ; M+ACN+2H ; M+ACN+H ; M+2ACN+2H ; M+ACN+2H ; M+2ACN+2H ; M+ACN+2H ; M+ACN+H ; M+ACN+2H ; M+2ACN+H ; M+2ACN+H ; M+ACN+2H ; M+ACN+2H ; M+H+NH4 ; M+ACN+2H	C20H24N2O3 ; C20H24N2O3 ; C18H21NO3 ; C18H21NO3 ; C20H21NO3 ; C20H21NO3 ; C18H21NO3 ; C18H21NO3 ; C16H18O3 ; C4H8S ; C6H11NS ; C18H21NO3 ; C6H11NS ; C11H18N2O5 ; C18H21NO3 ; C11H18N2O5 ; C18H21NO3 ; C6H11NS ; C18H21NO3 ; C4H8S ; C4H8S ; C18H21NO3 ; C18H21NO3 ; C20H21NO3 ; C18H21NO3
X359	4	483.36375	0.016	HMDB36133 ; HMDB41947	3-5-Methyl-2-(1-methylethyl)cyclohexyloxy-12-propanediol ; N1N8-Diacetylspermidine	2M+Na ; 2M+Na	C13H26O3 ; C11H24N3O2
X366	4	170.09249	0.106	HMDB13032 ; HMDB00079 ; HMDB03609 ; HMDB04193 ; HMDB04194 ; HMDB11655 ; HMDB14570 ; HMDB28895 ; HMDB29063 ; HMDB29709 ; HMDB29874 ; HMDB30346 ; HMDB31547 ; HMDB39426 ; HMDB59810 ; HMDB59851 ; HMDB59905 ; HMDB59924	Omega-Carboxy-trinor-leukotriene B4 ; Dihydrothymine ; 2-Aminoacrylic acid ; N1-Methyl-2-pyridone-5-carboxamide ; N1-Methyl-4-pyridone-3-carboxamide ; 2-(3-Carboxy-3-aminopropyl)-L-histidine ; Famciclovir ; Histidinyl-Threonine ; Threoninyl-Histidine ; 5678-Tetrahydro-4-methylquinoline ; Squamolone ; (S)-Actinidine ; L-Cyclo(alanylglycyl) ; 3-Isoxazolidinone ; m-Xylene ; o-Xylene ; Ethylbenzene ; p-Xylene	M+2H ; M+ACN+H ; M+2ACN+H ; M+NH4 ; M+NH4 ; M+2ACN+2H ; M+H+NH4 ; M+2ACN+2H ; M+2ACN+2H ; M+Na ; M+ACN+H ; M+Na ; M+ACN+H ; M+2ACN+H ; M+ACN+Na ; M+ACN+Na ; M+ACN+Na ; M+ACN+Na	C18H26O6 ; C5H8N2O2 ; C3H5NO2 ; C7H8N2O2 ; C7H8N2O2 ; C10H16N4O4 ; C14H19N5O4 ; C10H16N4O4 ; C10H16N4O4 ; C10H13N ; C5H8N2O2 ; C10H13N ; C5H8N2O2 ; C3H5NO2 ; C8H10 ; C8H10 ; C8H10 ; C8H10
X37	4	140.02557	0	HMDB60432 ; HMDB60693 ; HMDB34155	Alcophosphamide ; Alcoifosfamide ; Thiourea	M+2H ; M+2H ; M+ACN+Na	C7H17Cl2N2O3P ; C7H17Cl2N2O3P ; CH4N2S

X370	4	496.33931	0.104	HMDB10382 ; HMDB06228 ; HMDB06321 ; HMDB06496 ; HMDB06710 ; HMDB12454 ; HMDB12556 ; HMDB30702 ; HMDB31402 ; HMDB33769 ; HMDB36850 ; HMDB60134	LysoPC(16:0) ; 24-Hydroxycalcitriol ; Docosa-47101316-pentaenoyl carnitine ; Clupanodonyl carnitine ; Ubiquinone-4 ; 3 beta7 alpha-Dihydroxy-5-cholestenoate ; 13-Carboxy-alpha-tocotrienol ; Neochlorogenin ; 23-Acetoxysoladulcidine ; Rockogenin ; Ceanothenic acid ; 23S2526-Trihydroxyvitamin D3	M+H ; M+ACN+Na ; M+Na ; M+Na ; M+ACN+H ; M+ACN+Na ; M+ACN+H ; M+ACN+Na ; M+Na ; M+ACN+Na ; M+ACN+H ; M+ACN+Na	C24H50NO7P ; C27H44O4 ; C29H47NO4 ; C29H47NO4 ; C29H42O4 ; C27H44O4 ; C29H42O4 ; C27H44O4 ; C29H47NO4 ; C27H44O4 ; C29H42O4 ; C27H44O4
X4	4	153.02774	0	HMDB14957	Acetazolamide	M+2ACN+2H	C4H6N4O3S2
X45	4	154.02294	0	HMDB03263	Pelargonidin	M+2H	C15H11ClO5
X46	4	169.08444	0	HMDB29680 ; HMDB30472 ; HMDB30473 ; HMDB32252 ; HMDB32921 ; HMDB37729 ; HMDB38925 ; HMDB59963 ; HMDB00132 ; HMDB00403 ; HMDB00542 ; HMDB02207 ; HMDB06790 ; HMDB14789 ; HMDB32269 ; HMDB36231 ; HMDB38664 ; HMDB39407 ; HMDB40254 ; HMDB59842	26-Dimethoxy-4-methylphenol ; 4-Ipomeanol ; 1-Ipomeanol ; Epoxyoxophorone ; Ethyl 2-furanpropionate ; 2-Furanylmethyl butanoate ; (4-Hydroxy-3-methoxyphenyl)ethanol ; 135-Trimethoxybenzene ; Guanine ; 2-Hydroxyadenine ; 8-Hydroxyadenine ; 3-Hydroxyisoheptanoic acid ; Galactosylglycerol ; Dyphylline ; (-)-Ethyl 2-hydroxy-2-methylbutyrate ; Methyl DL-Leucate ; (2R)-1-O-beta-D-Galactopyranosylglycerol ; Methyl ()-3-hydroxyhexanoate ; Butyl lactate ; Ethyl 2-hydroxyisovalerate	M+H ; M+H ; M+H ; M+H ; M+H ; M+H ; M+H ; M+H ; M+NH4 ; M+NH4 ; M+NH4 ; M+Na ; M+2ACN+2H ; M+2ACN+2H ; M+Na ; M+Na ; M+2ACN+2H ; M+Na ; M+Na ; M+Na	C9H12O3 ; C9H12O3 ; C9H12O3 ; C9H12O3 ; C9H12O3 ; C9H12O3 ; C9H12O3 ; C9H12O3 ; C5H5N5O ; C5H5N5O ; C5H5N5O ; C7H14O3 ; C9H18O8 ; C10H14N4O4 ; C7H14O3 ; C7H14O3 ; C9H18O8 ; C7H14O3 ; C7H14O3 ; C7H14O3
X5	4	222.10772	0	HMDB14363	Moxifloxacin	M+ACN+2H	C21H24FN3O4
X57	4	137.0157	0	HMDB14901 ; HMDB34106 ; HMDB60445 ; HMDB60447	Methimazole ; Methyl isothiocyanate ; Bromobenzene-23-dihydrodiol ; Bromobenzene-34-dihydrodiol	M+Na ; M+ACN+Na ; M+2ACN+2H ; M+2ACN+2H	C4H6N2S ; C2H3NS ; C6H7BrO2 ; C6H7BrO2
X6	4	299.0832	0	HMDB39922	Eriodictyol 7-(6-trans-p-coumaroylglucoside)	M+2H	C30H28O13
X7	4	224.08345	0	HMDB60549 ; HMDB14462 ; HMDB32414 ; HMDB36187 ; HMDB37168 ; HMDB60624	Norketamine ; Gefitinib ; 2-Methyl-3 or 5 or 6-(furfurylthio)pyrazine (mixture of isomers) ; 2-(2-Furanylmethyl)thio-6-methylpyrazine ; 2-Propanoylthiazole ; Ethionamide sulphoxide	M+H ; M+2H ; M+NH4 ; M+NH4 ; M+2ACN+H ; M+ACN+H	C12H14ClNO ; C22H24ClFN4O3 ; C10H10N2OS ; C10H10N2OS ; C6H7NOS ; C8H10N2OS
X74	4	212.50484	0	Unknow	Unknow	Unknow	Unknow

X8	4	151.02961	0	Unknow	Unknow	Unknow	Unknow
X326	5	131.97428	0	Unknow	Unknow	Unknow	Unknow
X333	5	87.00403	0.001	Unknow	Unknow	Unknow	Unknow
X339	5	385.11853	0.012	HMDB28684 ; HMDB28768 ; HMDB29395 ; HMDB30391 ; HMDB40464 ; HMDB40786	Alanyl-Cysteine ; Cysteinyl-Alanine ; L-L-Homoglutathione ; Cepharadione B ; Dowicide A ; Zanthobisquinolone	2M+H ; 2M+H ; M+ACN+Na ; M+ACN+Na ; 2M+H ; M+Na	C6H12N2O3S ; C6H12N2O3S ; C11H19N3O6S ; C19H15NO4 ; C12H9NaO ; C21H18N2O4
X343	5	190.08943	0.001	HMDB30657 ; HMDB34474 ; HMDB35053 ; HMDB36694 ; HMDB36897 ; HMDB36900 ; HMDB36945 ; HMDB41002 ; HMDB41231 ; HMDB41349 ; HMDB38228 ; HMDB41208 ; HMDB61100	Carinol ; Niveusin C ; Gibberellin A52 ; 15-Hydroxyleptocarpin ; Gibberellin A17 ; Gibberellin A66 ; Gibberellin A125 ; (1E4Z6a8b10a)-8-(2-Methylbutanoyloxy)-1015-dihydroxy-3-oxo-1411(13)-germacratrien-126-olide ; Gibberellin A99 ; Gibberellin A102 ; 4-O-alpha-D-Galactopyranosylcalystegine B2 ; De-O-methylsimmondsin ; 6-oxo-famciclovir	M+2H ; M+2H ; M+2H ; M+2H ; M+2H ; M+2H ; M+2H ; M+2H ; M+2H ; M+2H ; M+ACN+2H ; M+H+NH4 ; M+ACN+2H	C20H26O7 ; C20H26O7 ; C20H26O7 ; C20H26O7 ; C20H26O7 ; C20H26O7 ; C20H26O7 ; C20H26O7 ; C20H26O7 ; C20H26O7 ; C13H23NO9 ; C15H23NO9 ; C14H19N5O5
X344	5	207.04191	0	HMDB04823 ; HMDB15093 ; HMDB61116	Lanthionine ketimine ; Carboplatin ; 2-aminophenol sulphate	M+NH4 ; M+ACN+2H ; M+NH4	C6H7NO4S ; C6H12N2O4Pt ; C6H7NO4S
X348	5	457.02009	0	HMDB29735	5-Ethynyl-5-(1-propynyl)-22-bithiophene	2M+H	C13H8S2
X349	5	180.00336	0.001	Unknow	Unknow	Unknow	Unknow
X352	5	272.95509	0	Unknow	Unknow	Unknow	Unknow
X353	5	460.80777	0	HMDB10166	PS(18:022:5(7Z10Z13Z16Z19Z))	M+2ACN+2H	C46H80NO10P
X362	5	217.51791	0	Unknow	Unknow	Unknow	Unknow
X371	5	328.91654	0	Unknow	Unknow	Unknow	Unknow
X376	5	276.92263	0	Unknow	Unknow	Unknow	Unknow
X380	5	270.95808	0	Unknow	Unknow	Unknow	Unknow
X387	5	234.98181	0	Unknow	Unknow	Unknow	Unknow
X393	5	366.87233	0	Unknow	Unknow	Unknow	Unknow
X404	5	286.93198	0	HMDB39734	23579-Pentathiadecane 22-dioxide	M+Na	C5H12O2S5

X293	6	253.08105	0	HMDB04086 ; HMDB35993 ; HMDB35486 ; HMDB00590 ; HMDB01138 ; HMDB01923 ; HMDB02335 ; HMDB05807 ; HMDB11639 ; HMDB13689 ; HMDB28761 ; HMDB29013 ; HMDB29649 ; HMDB29756 ; HMDB29842 ; HMDB31328 ; HMDB31574 ; HMDB31701 ; HMDB32345 ; HMDB32499 ; HMDB33184 ; HMDB34130 ; HMDB34212 ; HMDB34864 ; HMDB35370 ; HMDB39097 ; HMDB39110 ; HMDB40331 ; HMDB40590 ; HMDB41751 ; HMDB41752 ; HMDB59964 ; HMDB59999	5-Hydroxy-N-formylkynurenine ; N5-(34-Dioxo-15-cyclohexadien-1-yl)-L-glutamine ; 6-Caffeoylsucrose ; Glutarylglycine ; N-Acetylglutamic acid ; Naproxen ; Aspartyl-L-proline ; Gallic acid ; Topaquinone ; Peonidin-3-glucoside ; Aspartyl-Proline ; Prolyl-Aspartate ; 246-Trihydroxybenzoic acid ; (1R2S3R)-2-Acetyl-4(5)-(1234-tetrahydroxybutyl)imidazole ; Betalamic acid ; 1-Isothiocyanatobutane ; 1-Isothiocyanato-6-(methylthio)hexane ; 1-(gamma-Glutamylamino)cyclopropanecarboxylic acid ; Isobutyl isothiocyanate ; Pyrrolidino-12E-4H-24-dimethyl-135-dithiazine ; Pondaplin ; Osthenol ; (R)-Kawain ; 24-Dihydroxy-7-methoxy-2H-14-benzoxazin-3(4H)-one ; Pyranocyanin B ; Demethylbatatasin IV ; (2S3S)-alpha-Amino-2-carboxy-5-oxo-1-pyrrolidinebutanoic acid ; xi-25-Dihydro-24-dimethylthiazole ;	M+H ; M+H ; M+2H ; M+ACN+Na ; M+ACN+Na ; M+Na ; M+Na ; M+2ACN+H ; M+ACN+H ; M+ACN+2H ; M+Na ; M+Na ; M+2ACN+H ; M+Na ; M+ACN+H ; 2M+Na ; M+ACN+Na ; M+Na ; 2M+Na ; M+ACN+Na ; M+Na ; M+Na ; M+Na ; M+ACN+H ; M+H+NH4 ; M+Na ; M+Na ; 2M+Na ; M+2ACN+H ; M+ACN+2H ; M+ACN+2H ; M+2ACN+H ; M+ACN+H	C11H12N2O5 ; C11H12N2O5 ; C21H28O14 ; C7H11NO5 ; C7H11NO5 ; C14H14O3 ; C9H14N2O5 ; C7H6O5 ; C9H9NO5 ; C22H23O11 ; C9H14N2O5 ; C9H14N2O5 ; C7H6O5 ; C9H14N2O5 ; C9H9NO5 ; C5H9NS ; C8H15NS2 ; C9H14N2O5 ; C5H9NS ; C8H15NS2 ; C14H14O3 ; C14H14O3 ; C14H14O3 ; C9H9NO5 ; C24H23O11 ; C14H14O3 ; C9H14N2O5 ; C5H9NS ; C8H10S2 ; C22H23O11 ; C22H23O11 ; C7H6O5 ; C9H9NO5
X299	6	415.04852	0	HMDB14952	Meloxicam	M+ACN+Na	C14H13N3O4S2
X302	6	355.11049	0	HMDB34854 ; HMDB61000 ; HMDB11658 ; HMDB13189 ; HMDB28830 ; HMDB29082 ; HMDB29496 ; HMDB37356 ; HMDB37363 ; HMDB37537 ; HMDB38287 ; HMDB38824 ; HMDB39002 ; HMDB39878 ; HMDB39924 ; HMDB40371 ; HMDB40861 ; HMDB41358	Flumioxazin ; 4-(4-Chlorophenyl)-1-4-(4-fluorophenyl)-4-oxobutyl-pyridinium (HPP) ; 28-Dihydroxyquinoline-beta-D-glucuronide ; 3-Indole carboxylic acid glucuronide ; Glutamyl-Tryptophan ; Tryptophyl-Glutamate ; Fukiic acid ; Quercetin 47-diglucoside ; Quercetin 34-diglucoside ; Quercetin 3-glucosyl-(1-2)-galactoside ; Myricetin 3-neohesperidoside ; Hypoletin 8-gentiobioside ; Mytilin A ; Vicinin 2 ; 6-Hydroxykaempferol 67-diglucoside ; Herbacetin 38-diglucoside ; Myricetin 3-robinobioside ; Quercetin 3-beta-laminaribioside	M+H ; M+H ; M+NH4 ; M+NH4 ; M+Na ; M+Na ; M+2ACN+H ; M+2ACN+2H ; M+2ACN+2H ; M+2ACN+2H ; M+2ACN+2H ; M+2ACN+2H ; M+Na ; M+2ACN+2H ; M+2ACN+2H ; M+2ACN+2H ; M+2ACN+2H ; M+2ACN+2H	C19H15FN2O4 ; C21H18ClFNO ; C15H15NO8 ; C15H15NO8 ; C16H18N3O5 ; C16H18N3O5 ; C11H12O8 ; C27H30O17 ; C27H30O17 ; C27H30O17 ; C27H30O17 ; C27H30O17 ; C13H20N2O8 ; C27H30O17 ; C27H30O17 ; C27H30O17 ; C27H30O17 ; C27H30O17

X308	6	186.10603	0	HMDB32841 ; HMDB32842 ; HMDB36822 ; HMDB31682 ; HMDB34913 ; HMDB37554	5-Megastigmen-7-yne-39-diol 9-glucoside ; 5-Megastigmen-7-yne-39-diol 3-glucoside ; (3S7E9S)-9-Hydroxy-47-megastigmadien-3-one 9-glucoside ; ()-2-Methylthiazolidine ; Imazamethabenz-methyl ; ()-Rollipyrrole	M+2H ; M+2H ; M+2H ; M+2ACN+H ; M+2ACN+2H ; M+2ACN+2H	C19H30O7 ; C19H30O7 ; C19H30O7 ; C4H9NS ; C16H20N2O3 ; C16H20N2O3
X16	7	379.03428	0	HMDB60481 ; HMDB31780	heparan sulfate alpha-D-glucosaminide ; Dithianon	M+2H ; M+2ACN+H	C18H32N2O24S3 ; C14H4N2O2S2
X323	7	150.01407	0.663	Unknow	Unknow	Unknow	Unknow
X325	7	141.95144	0.575	Unknow	Unknow	Unknow	Unknow
X327	7	132.00355	0.491	Unknow	Unknow	Unknow	Unknow
X330	7	252.14418	0.364	HMDB00001 ; HMDB00479 ; HMDB00670 ; HMDB15238 ; HMDB29416	1-Methylhistidine ; 3-Methylhistidine ; Homo-L-arginine ; Levocabastine ; L-Targinine	M+2ACN+H ; M+2ACN+H ; M+ACN+Na ; M+2ACN+2H ; M+ACN+Na	C7H11N3O2 ; C7H11N3O2 ; C7H16N4O2 ; C26H29FN2O2 ; C7H16N4O2
X336	7	244.15423	0.357	HMDB02366 ; HMDB00792 ; HMDB31510 ; HMDB40196 ; HMDB40705 ; HMDB59708 ; HMDB59719 ; HMDB59729 ; HMDB59754	Tiglylcarnitine ; Sebacic acid ; R-2-Hydroxy-3-methylbutanoic acid 3-Methylbutanoyl ; Oxalic acid dibutyl ester ; Allixin ; 2-Ethylsuberic acid ; Heptylmalonic acid ; 34-Methylenesebacic acid ; 3-Methylazelaic acid	M+H ; M+ACN+H ; M+ACN+H ; M+ACN+H ; M+NH4 ; M+ACN+H ; M+ACN+H ; M+NH4 ; M+ACN+H	C12H21NO4 ; C10H18O4 ; C10H18O4 ; C10H18O4 ; C12H18O4 ; C10H18O4 ; C10H18O4 ; C12H18O4 ; C10H18O4
X337	7	122.01921	0.159	Unknow	Unknow	Unknow	Unknow
X340	7	145.03514	0.492	Unknow	Unknow	Unknow	Unknow
X341	7	546.35457	0.571	HMDB10393 ; HMDB10394 ; HMDB04888 ; HMDB10384 ; HMDB11128 ; HMDB15110 ; HMDB32111	LysoPC(20:3(5Z8Z11Z)) ; LysoPC(20:3(8Z11Z14Z)) ; Ganglioside GA2 (d18:112:0) ; LysoPC(18:0) ; LysoPC(0:018:0) ; Dipyridamole ; Adlupone	M+H ; M+H ; M+2ACN+2H ; M+Na ; M+Na ; M+ACN+H ; M+ACN+Na	C28H52NO7P ; C28H52NO7P ; C50H92N2O18 ; C26H54NO7P ; C26H54NO7P ; C24H40N8O4 ; C31H46O4

X347	7	186.14887	0.105	HMDB00246 ; HMDB00474 ; HMDB00482 ; HMDB01877 ; HMDB03543 ; HMDB06202 ; HMDB13105 ; HMDB13321 ; HMDB15562 ; HMDB29980 ; HMDB30368 ; HMDB31156 ; HMDB31230 ; HMDB31243 ; HMDB31273 ; HMDB31290 ; HMDB31324 ; HMDB31478 ; HMDB31579 ; HMDB31587 ; HMDB31588 ; HMDB31596 ; HMDB31638 ; HMDB31703 ; HMDB32046 ; HMDB32047 ; HMDB32203 ; HMDB32219 ; HMDB32225 ; HMDB32272 ; HMDB32312 ; HMDB32313 ; HMDB32319 ; HMDB32335 ; HMDB32434 ; HMDB32551 ; HMDB33700 ; HMDB33858 ; HMDB34161 ; HMDB34164 ; HMDB34234 ; HMDB34431 ; HMDB34452 ; HMDB34459 ; HMDB34670 ; HMDB35457 ; HMDB35714 ; HMDB35766 ; HMDB35829 ; HMDB36067 ; HMDB36103 ; HMDB36165 ; HMDB37014 ; HMDB37175 ;	Tetrahydrofuran ; Butanone ; Caprylic acid ; Valproic acid ; Butanal ; 48 Dimethylnonanoyl carnitine ; trans-45-epoxy-2(E)-decenal ; Undecanoylcarnitine ; Isopropamide ; Hexyl acetate ; (3R5Z)-5-Octene-13-diol ; Ethyl (4Z)-47-octadienoate ; 2-Ethylhexanoic acid ; 2-Methylpropanal ; Methyl octynecarboxylate ; 1-Hydroxy-3-octanone ; 3-Buten-1-ol ; Methyl heptanoate ; Ethyl ()-2-methylpentanoate ; 2-Methylheptanoic acid ; 6-Methylheptanoic acid ; Methyl 5-methylhexanoate ; Pentyl propanoate ; 2-Ethylbutyl acetate ; Isopropyl 3-methylbutanoate ; Propyl 3-methylbutanoate ; cis- and trans-2-Isobutyl-4-methyl-13-dioxolane ; (-)-Dihydromintlactone ; 11-Dimethoxy-trans-2-hexene ; Ethyl 4-methylpentanoate ; 24-Hexadienyl butyrate ; 24-Hexadienyl isobutyrate ; cis-3-Hexenyl crotonate ; 3-Hydroxy-2-octanone ; (-)-2-(5-Methyl-5-	2M+ACN+H ; 2M+ACN+H ; M+ACN+H ; M+ACN+H ; 2M+ACN+H ; M+ACN+2H ; M+NH4 ; M+ACN+2H ; M+H+NH4 ; M+ACN+H ; M+ACN+H ; M+NH4 ; M+ACN+H ; 2M+ACN+H ; M+NH4 ; M+ACN+H ; 2M+ACN+H ; M+ACN+H ; M+ACN+H ; M+ACN+H ; M+ACN+H ; M+ACN+H ; M+ACN+H ; M+ACN+H ; M+ACN+H ; M+ACN+H ; M+ACN+H ; M+NH4 ; M+ACN+H ; M+ACN+H ; M+NH4 ; M+NH4 ; M+NH4 ; M+ACN+H ; M+NH4 ; M+ACN+H ; M+NH4 ; 2M+ACN+H ; M+ACN+H ; M+ACN+H ; M+ACN+H ; 2M+ACN+H ; M+NH4 ; M+ACN+H ; M+NH4 ; M+ACN+H ; M+NH4 ; M+NH4 ; M+NH4 ; M+NH4 ; M+NH4 ; M+ACN+H ; M+NH4 ; M+NH4 ; M+2ACN+2H ; M+NH4 ; M+ACN+H ; M+ACN+H ; M+ACN+H ; M+ACN+H ; M+ACN+H ; M+ACN+H ; M+ACN+H ; M+NH4	C4H8O ; C4H8O ; C8H16O2 ; C8H16O2 ; C4H8O ; C18H35NO4 ; C10H16O2 ; C18H35NO4 ; C23H33N2O ; C8H16O2 ; C8H16O2 ; C10H16O2 ; C8H16O2 ; C4H8O ; C10H16O2 ; C8H16O2 ; C4H8O ; C8H16O2 ; C8H16O2 ; C8H16O2 ; C8H16O2 ; C8H16O2 ; C8H16O2 ; C8H16O2 ; C8H16O2 ; C8H16O2 ; C8H16O2 ; C10H16O2 ; C8H16O2 ; C8H16O2 ; C10H16O2 ; C10H16O2 ; C10H16O2 ; C8H16O2 ; C10H16O2 ; C8H16O2 ; C10H16O2 ; C4H8O ; C8H16O2 ; C8H16O2 ; C8H16O2 ; C4H8O ; C10H16O2 ; C8H16O2 ; C10H16O2 ; C8H16O2 ; C10H16O2 ; C10H16O2 ; C10H16O2 ; C10H16O2 ; C10H16O2 ; C8H16O2 ; C10H16O2 ; C10H16O2 ; C16H32O4 ; C10H16O2 ; C8H16O2 ; C8H16O2 ; C8H16O2 ; C8H16O2 ; C8H16O2 ; C8H16O2 ; C8H16O2 ; C10H16O2
X354	7	711.73116	0.342	Unknow	Unknow	Unknow	Unknow
X355	7	113.09038	0.12	Unknow	Unknow	Unknow	Unknow
X367	7	192.03971	0.089	HMDB41833 ; HMDB60244	Barbituric acid ; Phosphodimethylethanolamine	M+ACN+Na ; M+Na	C4H4N2O3 ; C4H12NO4P
X369	7	210.05027	0.105	HMDB29261 ; HMDB29502 ; HMDB37466 ; HMDB41774 ; HMDB33249	Kaempferol 3-O-arabinoside ; Kaempferol 3-alpha-L-arabinofuranoside ; Scutellarein 6-xyloside ; Salvianolic acid G ; 6-Chloro-N-(1-methylethyl)-135-triazine-24-diamine	M+2H ; M+2H ; M+2H ; M+2H ; M+Na	C20H18O10 ; C20H18O10 ; C20H18O10 ; C20H18O10 ; C6H10ClN5
X377	7	226.02407	0.283	HMDB40192	N-Nitrosothiazolidine-4-carboxylic acid	M+ACN+Na	C4H6N2O3S
X388	7	176.12235	0.048	Unknow	Unknow	Unknow	Unknow
X40	7	463.60828	0	Unknow	Unknow	Unknow	Unknow
X408	7	112.087	0.04	HMDB00870 ; HMDB14417 ; HMDB60263 ; HMDB04101 ; HMDB04231 ; HMDB15050 ; HMDB29598 ; HMDB33112	Histamine ; Betazole ; Histamium ; Beta-Aminopropionitrile ; Pantothenol ; Phenformin ; Metenamine ; Methylpyrazine	M+H ; M+H ; M+H ; M+ACN+H ; M+H+NH4 ; M+H+NH4 ; M+2ACN+2H ; M+NH4	C5H9N3 ; C5H9N3 ; C5H9N3 ; C3H6N2 ; C9H19NO4 ; C10H15N5 ; C6H12N4 ; C5H6N2
X42	7	462.01166	0	Unknow	Unknow	Unknow	Unknow
X43	7	334.58629	0	HMDB37539	6-Caffeoylhyperin	M+ACN+2H	C30H26O15
X44	7	289.55139	0	Unknow	Unknow	Unknow	Unknow

X54	7	132.49871	0	Unknow	Unknow	Unknow	Unknow
X67	7	504.0575	0	Unknow	Unknow	Unknow	Unknow
X78	7	308.52408	0	Unknow	Unknow	Unknow	Unknow
X87	7	219.54338	0	HMDB14599	Cefalotin	M+ACN+2H	C16H16N2O6S2
X102	8	127.50878	0	Unknow	Unknow	Unknow	Unknow
X287	9	381.80117	0	HMDB07878 ; HMDB07970 ; HMDB08034 ; HMDB08262 ; HMDB08907 ; HMDB09483	PC(14:020:0) ; PC(16:018:0) ; PC(18:016:0) ; PC(20:014:0) ; PE(15:022:0) ; PE(22:015:0)	M+2H ; M+2H ; M+2H ; M+2H ; M+2H ; M+2H	C42H84NO8P ; C42H84NO8P ; C42H84NO8P ; C42H84NO8P ; C42H84NO8P ; C42H84NO8P
X11	10	396.05505	0	HMDB14667	Oxaliplatin	M+H	C8H12N2O4Pt
X22	10	250.48312	0	Unknow	Unknow	Unknow	Unknow
X23	10	170.00508	0	Unknow	Unknow	Unknow	Unknow
X24	10	579.0758	0	Unknow	Unknow	Unknow	Unknow
X294	10	399.07395	0.001	HMDB14933	Sulfasalazine	M+H	C18H14N4O5S
X30	10	603.02915	0	HMDB00935 ; HMDB12302 ; HMDB12304	Uridine diphosphate glucuronic acid ; UDP-D-galacturonate ; UDP-L-iduronate	M+Na ; M+Na ; M+Na	C15H22N2O18P2 ; C15H22N2O18P2 ; C15H22N2O18P2
X324	10	384.11524	0.197	HMDB00912 ; HMDB01062 ; HMDB01121 ; HMDB01367 ; HMDB02817 ; HMDB06480 ; HMDB40546 ; HMDB59626	Succinyladenosine ; N-Acetyl-D-Glucosamine 6-Phosphate ; N-Acetyl-D-mannosamine 6-phosphate ; N-Acetyl-glucosamine 1-phosphate ; N-Acetylglucosamine 6-phosphate ; N-Acetyl-D-galactosamine 1-phosphate ; Pyriminobac-methyl ; N-acetyl-alpha-D-galactosamine 1-phosphate	M+H ; M+2ACN+H ; M+2ACN+H ; M+2ACN+H ; M+2ACN+H ; M+2ACN+H ; M+Na ; M+2ACN+H	C14H17N5O8 ; C8H16NO9P ; C8H16NO9P ; C8H16NO9P ; C8H16NO9P ; C8H16NO9P ; C17H19N3O6 ; C8H16NO9P
X328	10	548.37092	1	HMDB10392 ; HMDB00138 ; HMDB00331 ; HMDB32596 ; HMDB33409 ; HMDB34516 ; HMDB35257	LysoPC(20:2(11Z14Z)) ; Glycocholic acid ; 3a7b12a-Trihydroxyoxocholanyl-Glycine ; Sodium glycocholate ; 26-Methyl nigranoate ; 3b18b-3-Methoxy-11-oxo-12-oleanen-30-oic acid ; Methyl 3b24-dihydroxy-1113(18)-oleanadien-30-oate	M+H ; M+2ACN+H ; M+2ACN+H ; M+2ACN+H ; M+ACN+Na ; M+ACN+Na ; M+ACN+Na	C28H54NO7P ; C26H43NO6 ; C26H43NO6 ; C26H43NO6 ; C31H48O4 ; C31H48O4 ; C31H48O4
X35	10	727.12235	0	HMDB01397 ; HMDB11670 ; HMDB39720 ; HMDB59639	Guanosine monophosphate ; 8-Oxo-dGMP ; a-L-threo-4-Hex-4-enopyranuronosyl-D-galacturonic acid ; Cyclic pyranopterin monophosphate	2M+H ; 2M+H ; 2M+Na ; 2M+H	C10H14N5O8P ; C10H14N5O8P ; C12H16O12 ; C10H14N5O8P
X363	10	434.69068	0.023	Unknow	Unknow	Unknow	Unknow
X52	10	320.99209	0	Unknow	Unknow	Unknow	Unknow

Table S5c. Metabolite feature name in each community for each factor

Node	Community	m/z	centrality_vec	all_HMDBID	all_Name	all_Adduct	all_Formula
X104	1	253.994	0.014	Unknow	Unknow	Unknow	Unknow
X111	1	266.529	0.016	Unknow	Unknow	Unknow	Unknow
X117	1	287.592	0.148	HMDB29236 ; HMDB37971	Cyanidin 3-(6-acetyl-galactoside) ; Cyanidin 3-(4-acetylglucoside)	M+2ACN+2H ; M+2ACN+2H	C23H23O12 ; C23H23O12
X121	1	291.048	0.088	HMDB01068 ; HMDB60509 ; HMDB40610 ; HMDB01915 ; HMDB15492	D-Sedoheptulose 7-phosphate ; Sedoheptulose 1-phosphate ; Epitheaflavic acid 3-gallate ; Alendronic acid ; Stepronin	M+H ; M+H ; M+2H ; M+ACN+H ; M+NH4	C7H15O10P ; C7H15O10P ; C28H20O14 ; C4H13NO7P2 ; C10H11NO4S2
X131	1	300.533	0.016	Unknow	Unknow	Unknow	Unknow
X136	1	304.913	0.027	Unknow	Unknow	Unknow	Unknow
X155	1	351.968	0.15	Unknow	Unknow	Unknow	Unknow
X165	1	379.034	0.238	HMDB60481 ; HMDB31780	heparan sulfate alpha-D-glucosaminide ; Dithianon	M+2H ; M+2ACN+H	C18H32N2O24S3 ; C14H4N2O2S2
X174	1	388.027	0.016	HMDB01245	dCDP	M+H	C9H15N3O10P2
X176	1	391.032	0.177	HMDB30436	Emblicanin B	M+2H	C34H20O22
X183	1	426.024	0.078	Unknow	Unknow	Unknow	Unknow
X188	1	448.961	0.016	Unknow	Unknow	Unknow	Unknow
X192	1	463.608	0.208	Unknow	Unknow	Unknow	Unknow
X196	1	469.067	0.127	HMDB40628 ; HMDB00061 ; HMDB00960 ; HMDB01341 ; HMDB32937 ; HMDB33342 ; HMDB33352	3-(23-Digalloyl-46-hexahydroxydiphenoylglucosyl)-phloroacetophenone ; Adenosine 35-diphosphate ; dGDP ; ADP ; Methyl 4-chloro-1H-indole-3-acetate ; Mollicellin E ; Cyclobrassinin	M+2H ; M+ACN+H ; M+ACN+H ; M+ACN+H ; 2M+Na ; M+Na ; 2M+H	C42H32O25 ; C10H15N5O10P2 ; C10H15N5O10P2 ; C10H15N5O10P2 ; C11H10ClNO2 ; C22H19ClO8 ; C11H10N2S2
X231	1	122.019	0	Unknow	Unknow	Unknow	Unknow
X234	1	132.004	0	Unknow	Unknow	Unknow	Unknow
X237	1	145.035	0	Unknow	Unknow	Unknow	Unknow
X238	1	150.014	0	Unknow	Unknow	Unknow	Unknow
X241	1	160.527	0	HMDB15039	Ethacrynic acid	M+H+NH4	C13H12Cl2O4
X25	1	148.536	0.115	HMDB01235 ; HMDB12250 ; HMDB14407	5-Aminoimidazole ribonucleotide ; L-Aspartyl-4-phosphate ; Carmustine	M+2H ; M+2ACN+2H ; M+2ACN+2H	C8H14N3O7P ; C4H8NO7P ; C5H9Cl2N3O2

X259	1	212.092	0	HMDB01434 ; HMDB11754 ; HMDB60747 ; HMDB29507 ; HMDB30710 ; HMDB30713 ; HMDB30861 ; HMDB30863 ; HMDB31927 ; HMDB33804 ; HMDB34098 ; HMDB38879 ; HMDB38905 ; HMDB00318 ; HMDB00954 ; HMDB00955 ; HMDB02120 ; HMDB29663 ; HMDB30678 ; HMDB30765 ; HMDB32614 ; HMDB32652 ; HMDB32809 ; HMDB32966 ; HMDB35441 ; HMDB37644 ; HMDB38760 ; HMDB38761 ; HMDB39606 ; HMDB59721 ; HMDB59756 ; HMDB60021	3-Methoxytyrosine ; Methyldopa ; 3-O-Methyl-a-methyldopa ; Mulberrin ; Cyclomammeisin ; Mammea AAB cyclo F ; Kuwanon D ; Kuwanon F ; Glyurallin B ; Cajanone ; Kuwanol C ; Albanin E ; Isoangustone A ; 34-Dihydroxyphenylglycol ; trans-Ferulic acid ; Isoferulic acid ; Monoethyl phthalate ; Vanillin acetate ; Linocinnamarin ; (-)-trans-34-Dihydro-48-dihydroxy-3-methyl-1H-2-benzopyran-1-one ; Piperonyl acetate ; Meconine ; 4-Hydroxyphenylacetonitrile triacetylrhamnoside ; Furaneol acetate ; Oenanthoside A ; 3-(2-Hydroxy-3-methoxyphenyl)-2-propenoic acid ; Herierin III ; Herierin IV ; Repandiol ; mono-Benzyl malonate ; 34-Methyleneadipic acid ; trans-isoeugenol-O-glucuronide	M+H ; M+H ; M+H ; M+2H ; M+2H ; M+2H ; M+2H ; M+2H ; M+2H ; M+2H ; M+2H ; M+2H ; M+2H ; M+ACN+H ; M+NH4 ; M+NH4 ; M+NH4 ; M+NH4 ; M+2ACN+2H ; M+NH4 ; M+NH4 ; M+NH4 ; M+H+NH4 ; M+ACN+H ; M+2ACN+2H ; M+NH4 ; M+ACN+H ; M+ACN+H ; M+NH4 ; M+NH4 ; M+ACN+H ; M+2ACN+2H	C10H13NO4 ; C10H13NO4 ; C10H13NO4 ; C25H26O6 ; C25H26O6 ; C25H26O6 ; C25H26O6 ; C25H26O6 ; C25H26O6 ; C25H26O6 ; C25H26O6 ; C25H26O6 ; C25H26O6 ; C8H10O4 ; C10H10O4 ; C10H10O4 ; C10H10O4 ; C10H10O4 ; C16H20O8 ; C10H10O4 ; C10H10O4 ; C10H10O4 ; C20H23NO8 ; C8H10O4 ; C16H20O8 ; C10H10O4 ; C8H10O4 ; C8H10O4 ; C10H10O4 ; C10H10O4 ; C8H10O4 ; C16H20O8
X264	1	230.539	0	Unknow	Unknow	Unknow	Unknow
X266	1	235.962	0	HMDB15373	Chloroxine	M+Na	C9H5Cl2NO

X271	1	246.17	0	HMDB00378 ; HMDB00688 ; HMDB13128 ; HMDB41993 ; HMDB00718 ; HMDB00892 ; HMDB00933 ; HMDB02176 ; HMDB30058 ; HMDB30994 ; HMDB31175 ; HMDB31241 ; HMDB31247 ; HMDB31249 ; HMDB31516 ; HMDB32230 ; HMDB32263 ; HMDB33217 ; HMDB33479 ; HMDB33642 ; HMDB33742 ; HMDB33890 ; HMDB34237 ; HMDB35133 ; HMDB39053 ; HMDB39799 ; HMDB39974 ; HMDB40575 ; HMDB41992 ; HMDB59678	2-Methylbutyroylcarnitine ; Isovalerylcarnitine ; Valerylcarnitine ; Pivaloylcarnitine ; Isovaleric acid ; Valeric acid ; Traumatic acid ; Ethylmethylacetic acid ; Ethyl propionate ; 5-Heptyltetrahydro-2-oxo-3-furancarboxylic acid ; Tetrahydro-2-furanmethanol ; Isopropyl acetate ; 2-Methylpropyl formate ; Methyl isobutyrate ; 3-Hydroxy-2-pentanone ; 24-Dimethyl-13-dioxolane ; Ethylene oxidepropylene oxide copolymer ; (2xi6xi)-7-Methyl-3-methylene-1267-octanetetrol ; Sinapoylspermine ; (2xi3xi6E)-37-Dimethyl-6-octene-1238-tetrol ; (S)-2-Methylbutanoic acid ; Methyl butyrate ; Propyl acetate ; 37-Dimethyl-3-octene-1267-tetrol ; (1alpha2alpha4betaH6alpha8R)-p-Menthane-2689-tetrol ; Tetrahydro-2-methyl-3-furanol ; (1S2S4R8R)-p-Menthane-1289-tetrol ; Butyl formate ; Pivalic acid ; 1-Hydroxy-2-pentanone	M+H ; M+H ; M+H ; M+H ; 2M+ACN+H ; 2M+ACN+H ; M+NH4 ; 2M+ACN+H ; 2M+ACN+H ; M+NH4 ; 2M+ACN+H ; 2M+ACN+H ; 2M+ACN+H ; 2M+ACN+H ; 2M+ACN+H ; 2M+ACN+H ; 2M+ACN+H ; M+ACN+H ; M+2ACN+2H ; M+ACN+H ; 2M+ACN+H ; 2M+ACN+H ; 2M+ACN+H ; M+ACN+H ; M+ACN+H ; 2M+ACN+H ; M+ACN+H ; 2M+ACN+H ; 2M+ACN+H ; 2M+ACN+H	C12H23NO4 ; C12H23NO4 ; C12H23NO4 ; C12H23NO4 ; C5H10O2 ; C5H10O2 ; C12H20O4 ; C5H10O2 ; C5H10O2 ; C12H20O4 ; C5H10O2 ; C5H10O2 ; C5H10O2 ; C5H10O2 ; C5H10O2 ; C5H10O2 ; C5H10O2 ; C10H20O4 ; C21H36N4O4 ; C10H20O4 ; C5H10O2 ; C5H10O2 ; C5H10O2 ; C10H20O4 ; C10H20O4 ; C5H10O2 ; C10H20O4 ; C5H10O2 ; C5H10O2 ; C5H10O2
X272	1	247.173	0	HMDB38947 ; HMDB15090 ; HMDB39020 ; HMDB39021	(S)-3-Methylthiohexyl hexanoate ; Netilmicin ; 58-Epoxy-58-dihydro-10-apo-by-carotene-310-diol ; 56-Epoxy-56-dihydro-10-apo-by-carotene-310-diol	M+H ; M+H+NH4 ; M+2ACN+2H ; M+2ACN+2H	C13H26O2S ; C21H41N5O7 ; C27H38O3 ; C27H38O3
X274	1	252.144	0	HMDB00001 ; HMDB00479 ; HMDB00670 ; HMDB15238 ; HMDB29416	1-Methylhistidine ; 3-Methylhistidine ; Homo-L-arginine ; Levocabastine ; L-Targinine	M+2ACN+H ; M+2ACN+H ; M+ACN+Na ; M+2ACN+2H ; M+ACN+Na	C7H11N3O2 ; C7H11N3O2 ; C7H16N4O2 ; C26H29FN2O2 ; C7H16N4O2
X280	1	265.112	0	HMDB14462 ; HMDB38748	Gefitinib ; Osmanthuside A	M+2ACN+2H ; M+2ACN+2H	C22H24ClFN4O3 ; C23H26O9
X315	1	449.763	0	Unknow	Unknow	Unknow	Unknow
X42	1	175.008	0.042	HMDB60015 ; HMDB34155	Phenol sulphate ; Thiourea	M+H ; 2M+Na	C6H6O4S ; CH4N2S
X48	1	180.522	0.366	Unknow	Unknow	Unknow	Unknow
X63	1	201.546	0.192	Unknow	Unknow	Unknow	Unknow
X76	1	217.54	0.256	Unknow	Unknow	Unknow	Unknow

X77	1	218.042	0.258	HMDB00152 ; HMDB00397 ; HMDB00840 ; HMDB01229 ; HMDB01321 ; HMDB01856 ; HMDB02016 ; HMDB02404 ; HMDB04067 ; HMDB06116 ; HMDB13676 ; HMDB13677 ; HMDB13678 ; HMDB15366 ; HMDB29666 ; HMDB30726 ; HMDB34299 ; HMDB38055 ; HMDB39119 ; HMDB60602	Gentisic acid ; 2-Pyrocatechuic acid ; Salicyluric acid ; Dopaquinone ; D-Erythrose 4-phosphate ; Protocatechuic acid ; 4-Carboxyphenylglycine ; Alpha-Hydroxyhippuric acid ; Leucodopachrome ; 3-Hydroxyhippuric acid ; 26-Dihydroxybenzoic acid ; 35-Dihydroxybenzoic acid ; 4-Hydroxyhippuric acid ; Sevoflurane ; 24-Dihydroxybenzoic acid ; 2-Hydroxy-67-dimethoxybenzoxazole ; Patulin ; 2-Hydroxy-7-methoxy-2H-14-benzoxazin-3(4H)-one ; L-Dopaquinone ; N-acetyl-5-aminosalicylic acid	M+ACN+Na ; M+ACN+Na ; M+Na ; M+Na ; M+NH4 ; M+ACN+Na ; M+Na ; M+Na ; M+Na ; M+Na ; M+ACN+Na ; M+ACN+Na ; M+Na ; M+NH4 ; M+ACN+Na ; M+Na ; M+ACN+Na ; M+Na ; M+Na ; M+Na	C7H6O4 ; C7H6O4 ; C9H9NO4 ; C9H9NO4 ; C4H9O7P ; C7H6O4 ; C9H9NO4 ; C9H9NO4 ; C9H9NO4 ; C9H9NO4 ; C7H6O4 ; C7H6O4 ; C9H9NO4 ; C4H3F7O ; C7H6O4 ; C9H9NO4 ; C7H6O4 ; C9H9NO4 ; C9H9NO4 ; C9H9NO4
X94	1	239.066	0.016	HMDB00812 ; HMDB03470 ; HMDB06955 ; HMDB13989 ; HMDB30647 ; HMDB31577 ; HMDB32035 ; HMDB32656 ; HMDB37723 ; HMDB37998 ; HMDB38017 ; HMDB39163 ; HMDB40939 ; HMDB60273	N-Acetyl-L-aspartic acid ; N-Formyl-L-glutamic acid ; 3-Hydroxy-2-methylpyridine-45-dicarboxylate ; O-Desmethylnaproxen ; Artemidinol ; Berteroin ; 6-(34-Methylenedioxyphenyl)-35-hexadien-2-one ; (Z)-6-(2-Methoxyvinyl)-7-methyl-2H-1-benzopyran-2-one ; 2-Phenylethyl 2-furancarboxylate ; Delphinidin 3-arabinoside ; Ethyl 2-phenyl-3-furancarboxylate ; D-N-(Carboxyacetyl)alanine ; Methyl phenyl disulfide ; 2-Amino-3-oxoadipate	M+ACN+Na ; M+ACN+Na ; M+ACN+H ; M+Na ; M+Na ; M+ACN+Na ; M+Na ; M+Na ; M+Na ; M+ACN+2H ; M+Na ; M+ACN+Na ; M+2ACN+H ; M+ACN+Na	C6H9NO5 ; C6H9NO5 ; C8H7NO5 ; C13H12O3 ; C13H12O3 ; C7H13NS2 ; C13H12O3 ; C13H12O3 ; C13H12O3 ; C20H19O11 ; C13H12O3 ; C6H9NO5 ; C7H8S2 ; C6H9NO5

X95	1	239.103	0.118	HMDB15084 ; HMDB28853 ; HMDB29105 ; HMDB29451 ; HMDB40988 ; HMDB35635 ; HMDB00181 ; HMDB00609 ; HMDB01858 ; HMDB02048 ; HMDB02055 ; HMDB03119 ; HMDB29510 ; HMDB29610 ; HMDB33895 ; HMDB34436 ; HMDB38336 ; HMDB38750 ; HMDB38990 ; HMDB38991 ; HMDB40833 ; HMDB59731 ; HMDB60833	Felbamate ; Glycyl-Tyrosine ; Tyrosyl-Glycine ; gamma-Glutaminyl-4-hydroxybenzene ; Falimint ; Kanokoside A ; L-Dopa ; DL-Dopa ; p-Cresol ; m-Cresol ; o-Cresol ; Benzyl alcohol ; Garcinone B ; Ascladiol ; Anisole ; Rotenone ; 2-Hydroxy-3-(34-dihydroxyphenyl)propanamide ; N-Hydroxy-L-tyrosine ; Methyl 5-hydroxyoxindole-3-acetate ; Methyl dioxindole-3-acetate ; 3-4-Hydroxy-3-(3-methyl-2-butenyl)phenyl-2-propenal ; 23-Methyleneglutaric acid ; N-Acetylserotonin glucuronide	M+H ; M+H ; M+H ; M+H ; M+H ; M+2H ; M+ACN+H ; M+ACN+H ; 2M+Na ; 2M+Na ; 2M+Na ; 2M+Na ; M+2ACN+2H ; M+2ACN+H ; 2M+Na ; M+2ACN+2H ; M+ACN+H ; M+ACN+H ; M+NH4 ; M+NH4 ; M+Na ; M+2ACN+H ; M+2ACN+2H	C11H14N2O4 ; C11H14N2O4 ; C11H14N2O4 ; C11H14N2O4 ; C11H14N2O4 ; C21H32O12 ; C9H11NO4 ; C9H11NO4 ; C7H8O ; C7H8O ; C7H8O ; C7H8O ; C23H22O6 ; C7H8O4 ; C7H8O ; C23H22O6 ; C9H11NO4 ; C9H11NO4 ; C11H11NO4 ; C11H11NO4 ; C14H16O2 ; C7H8O4 ; C18H22N2O8
X149	2	332.589	1	Unknow	Unknow	Unknow	Unknow
X178	2	396.055	0.983	HMDB14667	Oxaliplatin	M+H	C8H12N2O4Pt
X222	2	796.802	0.164	HMDB06669	O-6-deoxy-alpha-L-galactopyranosyl-(1-3)-O-O-6-deoxy-alpha-L-galactopyranosyl-(1-4)-O-O-6-deoxy-alpha-L-galactopyranosyl-(1-2)-beta-D-galactopyranosyl-(1-3)-O-2-(acetylamino)-2-deoxy-beta-D-glucopyranosyl-(1-3)-beta-D-galactopyranosyl-(1-4)-O-2-	M+2ACN+2H	C57H95N3O43
X236	2	141.951	0	Unknow	Unknow	Unknow	Unknow
X254	2	195.931	0	Unknow	Unknow	Unknow	Unknow
X255	2	199.924	0	Unknow	Unknow	Unknow	Unknow
X257	2	206.046	0	HMDB00881 ; HMDB32963 ; HMDB33528 ; HMDB60413	Xanthurenic acid ; Zeanic acid ; 46-Dihydroxy-2-quinolinecarboxylic acid ; 6-Methylthioguanosine monophosphate	M+H ; M+H ; M+H ; M+H+NH4	C10H7NO4 ; C10H7NO4 ; C10H7NO4 ; C11H16N5O7PS
X260	2	215.936	0	Unknow	Unknow	Unknow	Unknow

X278	2	256.097	0	HMDB14608 ; HMDB29322 ; HMDB30215 ; HMDB30320 ; HMDB38639 ; HMDB38640 ; HMDB41996 ; HMDB02399 ; HMDB30580 ; HMDB30752 ; HMDB31610 ; HMDB31623 ; HMDB31816 ; HMDB32018 ; HMDB32583 ; HMDB32591 ; HMDB35275 ; HMDB35873 ; HMDB35894 ; HMDB37277 ; HMDB37877 ; HMDB40268 ; HMDB40446 ; HMDB41007 ; HMDB41481 ; HMDB41807	Ketorolac ; 1-Hydroxy-3-methoxy-10-methylacridone ; Clausine L ; Mukonine ; Methyl 6-methoxy-9H-carbazole-3-carboxylate ; 16-Dimethoxy-9H-carbazole-3-carboxaldehyde ; Pranoprofen ; Cystathionine sulfoxide ; (R)-Shinanolone ; Methyl trans-p-methoxycinnamate ; Allyl phenoxyacetate ; Ethyl phenylglycidate ; 3-Hydroxyflavone ; Phenyl salicylate ; 4-Hydroxy-2-biphenylcarboxylic acid ; Dehydrozingerone ; Isomyristicin ; Myristicin ; (Z)-4-Methoxy-6-(1-propenyl)-13-benzodioxole ; Isoeugenol formate ; Eugenyl formate ; Ethyl 3-oxo-3-phenylpropanoate ; Benzyl acetoacetate ; 1-Hydroxychavicol acetate ; 4-(34-Methylenedioxyphenyl)-2-butanone ; 3-Phenoxybenzoic acid	M+H ; M+H ; M+H ; M+H ; M+H ; M+H ; M+H ; M+NH4 ; M+ACN+Na ; M+ACN+Na ; M+ACN+Na ; M+ACN+Na ; M+NH4 ; M+ACN+H ; M+ACN+H ; M+ACN+Na ; M+ACN+Na ; M+ACN+Na ; M+ACN+Na ; M+ACN+Na ; M+ACN+Na ; M+ACN+Na ; M+ACN+Na ; M+ACN+Na ; M+ACN+Na ; M+ACN+H	C15H13NO3 ; C15H13NO3 ; C15H13NO3 ; C15H13NO3 ; C15H13NO3 ; C15H13NO3 ; C15H13NO3 ; C7H14N2O5S ; C11H12O3 ; C11H12O3 ; C11H12O3 ; C11H12O3 ; C15H10O3 ; C13H10O3 ; C13H10O3 ; C11H12O3 ; C11H12O3 ; C11H12O3 ; C11H12O3 ; C11H12O3 ; C11H12O3 ; C11H12O3 ; C11H12O3 ; C11H12O3 ; C11H12O3 ; C13H10O3
X282	2	270.891	0	HMDB12259	Methylarsonite	2M+Na	CH5AsO2
X289	2	295.903	0	Unknow	Unknow	Unknow	Unknow
X294	2	336.963	0	Unknow	Unknow	Unknow	Unknow
X41	2	170.005	0.735	Unknow	Unknow	Unknow	Unknow
X44	2	178.044	0.537	HMDB01919	Famotidine	M+H+NH4	C8H15N7O2S3
X61	2	198.122	0.634	HMDB29422 ; HMDB00162 ; HMDB03411 ; HMDB04076 ; HMDB12880 ; HMDB13319 ; HMDB30397 ; HMDB30409 ; HMDB32549 ; HMDB32565 ; HMDB34208 ; HMDB41109 ; HMDB60281	L-Histidine trimethylbetaine ; L-Proline ; D-Proline ; 5-Hydroxykynurenamine ; Acetamidopropanal ; Tyrosinamide ; L-2-Amino-3-(4-aminophenyl)propanoic acid ; 4-Amino-2-methylenebutanoic acid ; N-Undecylbenzenesulfonic acid ; (4-Ethoxyphenyl)urea ; Pterolactam ; (S)-3-Ethylidenehexahydropyrrolo12-apyrazine-14-dione ; 3-Hydroxykynurenamine	M+H ; M+2ACN+H ; M+2ACN+H ; M+NH4 ; M+2ACN+H ; M+NH4 ; M+NH4 ; M+2ACN+H ; M+2ACN+2H ; M+NH4 ; M+2ACN+H ; M+NH4 ; M+NH4	C9H15N3O2 ; C5H9NO2 ; C5H9NO2 ; C9H12N2O2 ; C5H9NO2 ; C9H12N2O2 ; C9H12N2O2 ; C5H9NO2 ; C17H28O3S ; C9H12N2O2 ; C5H9NO2 ; C9H12N2O2 ; C9H12N2O2
X92	2	236.084	0.174	HMDB34007 ; HMDB38463	(EE)-1-Chloro-311-tridecadiene-579-triyn-2-ol ; 3-(Isothiocyanatomethyl)-1-methoxy-1H-indole	M+NH4 ; M+NH4	C13H11ClO ; C11H10N2OS
X98	2	241.03	0.561	HMDB00192 ; HMDB00965	L-Cystine ; Hypotaurine	M+H ; 2M+Na	C6H12N2O4S2 ; C2H7NO2S

X109	3	262.078	0.002	HMDB28752 ; HMDB28815 ; HMDB33705 ; HMDB37336 ; HMDB15188	Aspartyl-Glutamate ; Glutamyl-Aspartate ; Salviaflaside ; Sudachiin A ; Mimosine	M+H ; M+H ; M+2H ; M+2H ; M+ACN+Na	C9H13N2O7 ; C9H13N2O7 ; C24H26O13 ; C24H26O13 ; C8H10N2O4
X118	3	288.592	0.083	HMDB35209 ; HMDB60391 ; HMDB60423 ; HMDB40862	Cartormin ; 45-Dihydro-4-hydroxy-5-S-glutathionyl-benzoapyrene ; 78-Dihydro-7-hydroxy-8-S-glutathionyl-benzoapyrene ; Piceatannol 4-galloylglucoside	M+2H ; M+2H ; M+2H ; M+H+NH4	C27H29NO13 ; C30H29N3O7S ; C30H29N3O7S ; C27H26O13
X125	3	296.049	0.019	HMDB32964 ; HMDB42046 ; HMDB60257	2-Acetylthiazole ; Treosulfan ; Selenomethionine se-oxide	2M+ACN+H ; M+NH4 ; M+2ACN+H	C5H5NOS ; C6H14O8S2 ; C5H11NO3Se
X128	3	299.083	0	HMDB39922 ; HMDB11737 ; HMDB42036	Eriodictyol 7-(6-trans-p-coumaroylglucoside) ; Gamma Glutamylglutamic acid ; Thymidine glycol	M+2H ; M+Na ; M+Na	C30H28O13 ; C10H16N2O7 ; C10H16N2O7
X133	3	303.546	0.22	HMDB01163 ; HMDB03351 ; HMDB33533	Guanosine diphosphate mannose ; GDP-glucose ; Ceftiofur	M+2H ; M+2H ; M+2ACN+2H	C16H25N5O16P2 ; C16H25N5O16P2 ; C19H17N5O7S3
X138	3	309.074	0.104	HMDB29527 ; HMDB29870 ; HMDB29988 ; HMDB30090 ; HMDB30622 ; HMDB30793 ; HMDB30807 ; HMDB30852 ; HMDB31420 ; HMDB31620 ; HMDB32288 ; HMDB33291 ; HMDB33309 ; HMDB34011 ; HMDB34012 ; HMDB34114 ; HMDB34115 ; HMDB34438 ; HMDB37320 ; HMDB37322 ; HMDB37489 ; HMDB38448 ; HMDB40201 ; HMDB41726	Melilotocarpan B ; 37-Dihydroxy-4-methoxyisoflavanone ; Bis(2-furanylmethyl) disulfide ; Sakuranetin ; (R)-Oxypeucedanin ; Moracin B ; 34-Dihydro-8-hydroxy-3-(3-hydroxy-4-methoxyphenyl)-1H-2-benzopyran-1-one ; Asperxanthone ; Heliannone C ; Vestitone ; Furfuryl 2-methyl-3-furyl disulfide ; 38-Dihydroxy-9-methoxypterocarpan ; Moracin F ; 2-(24-Dihydroxyphenyl)-56-dimethoxybenzofuran ; 34-Dihydroxy-9-methoxypterocarpan ; 6alpha-Hydroxyisomedicarpin ; 6alpha-Hydroxymedicarpin ; (R)-Pabulenol ; Licochalcone B ; Dihydrooroxylin ; Dihydrowogonin ; (-)-57-Dihydroxy-3-(4-hydroxybenzyl)-4-chromanone ; 33-Dithiobis2-methylfuran ; Dihydroglycitein	M+Na ; M+Na ; M+2ACN+H ; M+Na ; M+Na ; M+Na ; M+Na ; M+Na ; M+Na ; M+Na ; M+2ACN+H ; M+Na ; M+Na ; M+Na ; M+Na ; M+Na ; M+Na ; M+Na ; M+Na ; M+Na ; M+Na ; M+Na ; M+2ACN+H ; M+Na	C16H14O5 ; C16H14O5 ; C10H10O2S2 ; C16H14O5 ; C16H14O5 ; C16H14O5 ; C16H14O5 ; C16H14O5 ; C16H14O5 ; C16H14O5 ; C10H10O2S2 ; C16H14O5 ; C16H14O5 ; C16H14O5 ; C16H14O5 ; C16H14O5 ; C16H14O5 ; C16H14O5 ; C16H14O5 ; C16H14O5 ; C16H14O5 ; C16H14O5 ; C10H10O2S2 ; C16H14O5
X139	3	313.017	0.014	HMDB04812	25-Furandicarboxylic acid	2M+H	C6H4O5
X141	3	315.174	0.011	HMDB37793	(3S5R6R6S)-67-Didehydro-56-dihydro-356-trihydroxy-131420-trinor-3-oxo-betaepsilon-caroten-1911-olide 3-acetate	M+2H	C39H48O7
X150	3	334.027	0.049	HMDB38429 ; HMDB06343	Methyl glucosinolate ; Selenocystathionine	M+H ; M+ACN+Na	C8H15NO9S2 ; C7H14N2O4Se

X151	3	341.002	0.011	HMDB00968 ; HMDB01047 ; HMDB01058 ; HMDB03514 ; HMDB06234 ; HMDB06235 ; HMDB06872 ; HMDB60269 ; HMDB60444 ; HMDB01112 ; HMDB01473	1D-Myo-inositol 14-bisphosphate ; D-Fructose 26-bisphosphate ; Fructose 16-bisphosphate ; Alpha-D-Glucose 16-bisphosphate ; 1D-Myo-inositol 13-bisphosphate ; 1D-Myo-inositol 34-bisphosphate ; D-Tagatose 16-bisphosphate ; D-Mannose 16-bisphosphate ; beta-D-Fructose 16-bisphosphate ; D-Glyceraldehyde 3-phosphate ; Dihydroxyacetone phosphate	M+H ; M+H ; M+H ; M+H ; M+H ; M+H ; M+H ; M+H ; M+H ; 2M+H ; 2M+H	C6H14O12P2 ; C6H14O12P2 ; C6H14O12P2 ; C6H14O12P2 ; C6H14O12P2 ; C6H14O12P2 ; C6H14O12P2 ; C6H14O12P2 ; C6H14O12P2 ; C3H7O6P ; C3H7O6P
X152	3	344.43	0.057	Unknow	Unknow	Unknow	Unknow
X158	3	357.037	0.003	HMDB06462 ; HMDB15511 ; HMDB60636	Homocysteinesulfinic acid ; Bromazepam ; Malathion dicarboxylic acid	2M+Na ; M+ACN+H ; M+2ACN+H	C4H9NO4S ; C14H10BrN3O ; C6H11O6PS2
X16	3	137.016	0.025	HMDB14901 ; HMDB34106 ; HMDB60445 ; HMDB60447	Methimazole ; Methyl isothiocyanate ; Bromobenzene-23-dihydrodiol ; Bromobenzene-34-dihydrodiol	M+Na ; M+ACN+Na ; M+2ACN+2H ; M+2ACN+2H	C4H6N2S ; C2H3NS ; C6H7BrO2 ; C6H7BrO2
X164	3	378.95	0.011	Unknow	Unknow	Unknow	Unknow
X17	3	138.994	0	HMDB12974	Hypothiocyanite	M+ACN+Na	CHNOS
X182	3	417.142	0.085	HMDB34157 ; HMDB03920 ; HMDB15011 ; HMDB30265 ; HMDB30322 ; HMDB30375 ; HMDB33445	2-Hydroxybenzaldehyde O-xylosyl-(1-6)-glucoside ; Protopine ; Loteprednol ; 22-Dimethyl(pyrano-56:3:4)-15-dihydroxy-6-methoxy-10-methylacridone ; Papaveraldine ; Citracridone I ; Honyumine	M+H ; M+ACN+Na ; M+Na ; M+ACN+Na ; M+ACN+Na ; M+ACN+Na ; M+ACN+Na	C18H24O11 ; C20H19NO5 ; C21H27ClO5 ; C20H19NO5 ; C20H19NO5 ; C20H19NO5 ; C20H19NO5
X19	3	140.522	0.054	Unknow	Unknow	Unknow	Unknow
X195	3	466.086	0.08	HMDB38410 ; HMDB15045	Glucohesperalin ; Zonisamide	M+H ; 2M+ACN+H	C14H27NO10S3 ; C8H8N2O3S
X201	3	482.58	0.001	Unknow	Unknow	Unknow	Unknow
X202	3	484.011	0.089	Unknow	Unknow	Unknow	Unknow
X203	3	486.065	0.017	HMDB00536 ; HMDB41173 ; HMDB59653	Adenylsuccinic acid ; 1-O-Caffeoyl-(b-D-glucose 6-O-sulfate) ; N(6)-(12-dicarboxyethyl)AMP	M+Na ; M+ACN+Na ; M+Na	C14H18N5O11P ; C15H18O12S ; C14H18N5O11P
X204	3	507.586	0.011	Unknow	Unknow	Unknow	Unknow
X208	3	532.052	0.011	Unknow	Unknow	Unknow	Unknow
X209	3	549.59	0.02	HMDB39203	Camelliatannin A	M+ACN+2H	C49H36O27
X210	3	576.126	0.001	Unknow	Unknow	Unknow	Unknow
X225	3	102.013	0	Unknow	Unknow	Unknow	Unknow
X228	3	120.024	0	HMDB14836	Nitrofurantoin	M+2H	C8H6N4O5
X23	3	148.022	0.011	HMDB37835	4-(24-Dihydroxyphenyl)azobenzenesulfonic acid	M+2H	C12H10N2O5S
X235	3	140.951	0	Unknow	Unknow	Unknow	Unknow
X24	3	148.524	0.02	Unknow	Unknow	Unknow	Unknow
X251	3	186.956	0	Unknow	Unknow	Unknow	Unknow

X261	3	224.09	0	HMDB00866 ; HMDB13068 ; HMDB00118 ; HMDB00333 ; HMDB00423 ; HMDB00555 ; HMDB00755 ; HMDB00857 ; HMDB02441 ; HMDB02643 ; HMDB04061 ; HMDB15024 ; HMDB15400 ; HMDB29167 ; HMDB29232 ; HMDB29273 ; HMDB29466 ; HMDB29472 ; HMDB29573 ; HMDB29646 ; HMDB29775 ; HMDB30818 ; HMDB32952 ; HMDB33624 ; HMDB35303 ; HMDB36627 ; HMDB37274 ; HMDB41270 ; HMDB41605 ; HMDB59722 ; HMDB59738 ; HMDB59763 ; HMDB59893	N-Acetyl-L-tyrosine ; Salsolinol 1-carboxylate ; Homovanillic acid ; Isohomovanillic acid ; 34-Dihydroxyhydrocinnamic acid ; 3-Methyladipic acid ; Hydroxyphenyllactic acid ; Pimelic acid ; 33-Dimethylglutaric acid ; 3-(3-Hydroxyphenyl)-3-hydroxypropanoic acid ; 3-Methoxy-4-hydroxyphenylglycolaldehyde ; Bumetanide ; Hydralazine ; 2-Methyladipic acid ; 3-Hydroxyphenyllactate ; 26-Dimethoxybenzoic acid ; Eugenitol ; Isoeugenitol ; Diethyl malonate ; 26-Dihydroxy-4-methoxyacetophenone ; Vinyl caffeate ; Scoparone ; Citropten ; ()-2-Hydroxy-3-(2-hydroxyphenyl)propanoic acid ; 3-Methoxy-45-methylenedioxycinnamaldehyde ; Eugenin ; Maltol propionate ; 24-Dihydroxy-6-methoxyacetophenone ; Propyleneglycol diacetate ; Mono-methyl-adipate ; 2-Ethylglutaric acid ; 34-Dimethoxybenzoic acid ; Ethyl methylsuccinate	M+H ; M+H ; M+ACN+H ; M+ACN+H ; M+ACN+H ; M+ACN+Na ; M+ACN+H ; M+ACN+Na ; M+ACN+Na ; M+ACN+H ; M+ACN+H ; M+2ACN+2H ; M+ACN+Na ; M+ACN+Na ; M+ACN+H ; M+ACN+H ; M+NH4 ; M+NH4 ; M+ACN+Na ; M+ACN+H ; M+NH4 ; M+NH4 ; M+NH4 ; M+ACN+H ; M+NH4 ; M+NH4 ; M+ACN+H ; M+ACN+H ; M+ACN+Na ; M+ACN+Na ; M+ACN+Na ; M+ACN+H ; M+ACN+Na	C11H13NO4 ; C11H13NO4 ; C9H10O4 ; C9H10O4 ; C9H10O4 ; C7H12O4 ; C9H10O4 ; C7H12O4 ; C7H12O4 ; C9H10O4 ; C9H10O4 ; C17H20N2O5S ; C8H8N4 ; C7H12O4 ; C9H10O4 ; C9H10O4 ; C11H10O4 ; C11H10O4 ; C7H12O4 ; C9H10O4 ; C11H10O4 ; C11H10O4 ; C11H10O4 ; C9H10O4 ; C11H10O4 ; C11H10O4 ; C9H10O4 ; C9H10O4 ; C7H12O4 ; C7H12O4 ; C7H12O4 ; C9H10O4 ; C7H12O4
X269	3	238.928	0	Unknow	Unknow	Unknow	Unknow
X287	3	292.148	0	HMDB38217 ; HMDB59788	Pterosin H ; Hemorphin-4	M+ACN+H ; M+H+NH4	C15H19ClO ; C29H35N5O7
X291	3	305.068	0	HMDB40631 ; HMDB36336 ; HMDB29694	Pratenol B ; Prodelphinidin A1 ; S-Methyl benzenecarbothioate	M+H ; M+2H ; 2M+H	C15H12O7 ; C30H24O14 ; C8H8OS
X293	3	332.562	0	HMDB39421	Fenugreekine	M+2H	C21H27N7O14P2
X295	3	352.853	0	Unknow	Unknow	Unknow	Unknow
X319	3	482.36	0	HMDB06280 ; HMDB06281 ; HMDB06763 ; HMDB06764 ; HMDB06886 ; HMDB06887 ; HMDB06894 ; HMDB11644 ; HMDB12560 ; HMDB35730 ; HMDB60136	7-a25-Dihydroxycholesterol ; 7-a27-Dihydroxycholesterol ; 20a22b-Dihydroxycholesterol ; 17a20a-Dihydroxycholesterol ; 7a12a-Dihydroxy-5a-cholestan-3-one ; 7a12a-Dihydroxy-5b-cholestan-3-one ; 3a7a-Dihydroxy-5b-cholestan-26-al ; (24R)-Cholest-5-ene-3-beta7-alpha24-triol ; 13-Hydroxy-alpha-tocotrienol ; Camelledionol ; (24S)-7alpha24-Dihydroxycholesterol	M+ACN+Na ; M+ACN+Na ; M+ACN+Na ; M+ACN+Na ; M+ACN+Na ; M+ACN+Na ; M+ACN+Na ; M+ACN+Na ; M+ACN+H ; M+ACN+H ; M+ACN+Na	C27H46O3 ; C27H46O3 ; C27H46O3 ; C27H46O3 ; C27H46O3 ; C27H46O3 ; C27H46O3 ; C27H46O3 ; C29H44O3 ; C29H44O3 ; C27H46O3
X320	3	483.364	0	HMDB36133 ; HMDB41947	3-5-Methyl-2-(1-methylethyl)cyclohexyloxy-12-propanediol ; N1N8-Diacetylspermidine	2M+Na ; 2M+Na	C13H26O3 ; C11H24N3O2

X324	3	496.339	0	HMDB10382 ; HMDB06228 ; HMDB06321 ; HMDB06496 ; HMDB06710 ; HMDB12454 ; HMDB12556 ; HMDB30702 ; HMDB31402 ; HMDB33769 ; HMDB36850 ; HMDB60134	LysoPC(16:0) ; 24-Hydroxycalcitriol ; Docosa-47101316-pentaenoyl carnitine ; Clupanodonyl carnitine ; Ubiquinone-4 ; 3 beta7 alpha-Dihydroxy-5-cholestenoate ; 13-Carboxy-alpha-tocotrienol ; Neochlorogenin ; 23-Acetoxysoladulcidine ; Rockogenin ; Ceanothenic acid ; 23S2526-Trihydroxyvitamin D3	M+H ; M+ACN+Na ; M+Na ; M+Na ; M+ACN+H ; M+ACN+Na ; M+ACN+H ; M+ACN+Na ; M+Na ; M+ACN+Na ; M+ACN+H ; M+ACN+Na	C24H50NO7P ; C27H44O4 ; C29H47NO4 ; C29H47NO4 ; C29H42O4 ; C27H44O4 ; C29H42O4 ; C27H44O4 ; C29H47NO4 ; C27H44O4 ; C29H42O4 ; C27H44O4
X325	3	498.346	0	HMDB30180	Crustecdysone	M+NH4	C27H44O7
X326	3	499.348	0	HMDB10381 ; HMDB11129 ; HMDB11130 ; HMDB15004 ; HMDB30271 ; HMDB60852 ; HMDB60997 ; HMDB61007	LysoPC(15:0) ; LysoPE(0:018:0) ; LysoPE(18:00:0) ; Alprenolol ; Dehydrocarpaine I ; NO-Didesmethylvenlafaxine ; O-Desmethyltramadol ; N-Desmethyltramadol	M+NH4 ; M+NH4 ; M+NH4 ; 2M+H ; M+Na ; 2M+H ; 2M+H ; 2M+H	C23H48NO7P ; C23H48NO7P ; C23H48NO7P ; C15H23NO2 ; C28H48N2O4 ; C15H23NO2 ; C15H23NO2 ; C15H23NO2
X328	3	510.355	0	HMDB11481 ; HMDB11511 ; HMDB12108 ; HMDB12557 ; HMDB30016 ; HMDB31349 ; HMDB32081 ; HMDB32107 ; HMDB32123 ; HMDB32837 ; HMDB32875 ; HMDB33252 ; HMDB33253 ; HMDB33960 ; HMDB34515 ; HMDB34562 ; HMDB34742 ; HMDB34956 ; HMDB35202 ; HMDB35407 ; HMDB35408 ; HMDB35413 ; HMDB35434 ; HMDB35639 ; HMDB35794 ; HMDB35869 ; HMDB35886 ; HMDB35889 ; HMDB35935 ; HMDB36035 ; HMDB36390 ; HMDB36392 ; HMDB36427 ; HMDB36449 ; HMDB36466 ; HMDB36647 ; HMDB37038 ; HMDB37391 ; HMDB37640 ; HMDB37710 ; HMDB37739 ; HMDB38190 ; HMDB38198 ; HMDB38698 ; HMDB38795 ; HMDB38796 ; HMDB38797 ; HMDB38914 ; HMDB39615 ; HMDB39621 ; HMDB40711 ; HMDB40999 ; HMDB41036 ; HMDB41527	LysoPE(0:020:0) ; LysoPE(20:00:0) ; LysoPC(17:0) ; 13-Carboxy-gamma-tocopherol ; Valerenic acid ; Epiacoronene ; 410-Longipinanedione ; (3beta5alpha6beta7alpha22E24R)-Ergosta-822-diene-3567-tetrol ; (3beta5alpha6alpha9alpha22E24R)-Ergosta-722-diene-3569-tetrol ; Ganoderic acid DM ; 359-Trihydroxyergost-7-en-6-one ; Curcumanolide A ; Curcumenone ; Curcumenol ; Glabrolide ; 9-Pentadecene-1214-diyne-111-diol ; Saussurea lactone ; Oxysolavetivone ; 7-Hydroxycostal ; Dehydrocurdione ; 110-Epoxygermacrone ; (4alpha5alpha)-11-Eremophilene-29-dione ; Isomasticadienonalic acid ; Petasalbin ; beta-Costic acid ; alpha-Rotunol ; Isoglabrolide ; Germacrone 45-epoxide ; Dihydroisoalantolactone ; Marasmene ; 3-Phenylpropyl hexanoate ; (Z)-alpha-Bergamotenoic acid ; Procurcumenol ; (1alpha4beta5beta)-4-Hydroxy-7(11)10(14)-guaiadien-8-one ; Zerumbone oxide ; Isocurcumenol ; Cyclodehydroisolubimin ; p-	M+H ; M+H ; M+H ; M+ACN+Na ; 2M+ACN+H ; 2M+ACN+H ; 2M+ACN+H ; M+ACN+Na ; M+ACN+Na ; M+ACN+H ; M+ACN+Na ; 2M+ACN+H ; 2M+ACN+H ; 2M+ACN+H ; M+ACN+H ; 2M+ACN+H ; 2M+ACN+H ; 2M+ACN+H ; 2M+ACN+H ; 2M+ACN+H ; 2M+ACN+H ; 2M+ACN+H ; M+ACN+H ; 2M+ACN+H ; 2M+ACN+H ; 2M+ACN+H ; M+ACN+H ; 2M+ACN+H ; 2M+ACN+H ; 2M+ACN+H ; 2M+ACN+H ; 2M+ACN+H ; 2M+ACN+H ; 2M+ACN+H ; 2M+ACN+H ; 2M+ACN+H ; 2M+ACN+H ; 2M+ACN+H ; 2M+ACN+H ; 2M+ACN+H ; 2M+ACN+H ; 2M+ACN+H ; 2M+ACN+H ; 2M+ACN+H ; 2M+ACN+H ; 2M+ACN+H ; M+ACN+H ; 2M+ACN+H ; M+ACN+H ; 2M+ACN+H ; M+ACN+H ; M+ACN+Na ; 2M+ACN+H ; M+ACN+Na	C25H52NO7P ; C25H52NO7P ; C25H52NO7P ; C28H46O4 ; C15H22O2 ; C15H22O2 ; C15H22O2 ; C28H46O4 ; C28H46O4 ; C30H44O4 ; C28H46O4 ; C15H22O2 ; C15H22O2 ; C15H22O2 ; C30H44O4 ; C15H22O2 ; C15H22O2 ; C15H22O2 ; C15H22O2 ; C15H22O2 ; C15H22O2 ; C15H22O2 ; C30H44O4 ; C15H22O2 ; C15H22O2 ; C15H22O2 ; C30H44O4 ; C15H22O2 ; C15H22O2 ; C15H22O2 ; C15H22O2 ; C15H22O2 ; C15H22O2 ; C15H22O2 ; C15H22O2 ; C15H22O2 ; C15H22O2 ; C15H22O2 ; C15H22O2 ; C15H22O2 ; C15H22O2 ; C15H22O2 ; C15H22O2 ; C15H22O2 ; C30H44O4 ; C15H22O2 ; C30H44O4 ; C15H22O2 ; C30H44O4 ; C28H46O4 ; C15H22O2 ; C28H46O4
X329	3	511.358	0	HMDB14930 ; HMDB31867 ; HMDB39588	Tripelennamine ; 4-Acetyl-6-tert-butyl-11-dimethylindane ; Panaxynol	2M+H ; 2M+Na ; 2M+Na	C16H21N3 ; C17H24O ; C17H24O

X330	3	515.313	0	HMDB39150 ; HMDB14393 ; HMDB29656 ; HMDB35493 ; HMDB35749 ; HMDB35778 ; HMDB36207 ; HMDB37705 ; HMDB39547 ; HMDB39597 ; HMDB40291 ; HMDB40390 ; HMDB41250	Flavidulol C ; Cabergoline ; Erinacine E ; Notoginsenoside K ; Ginsenoside B2 ; Ginsenoside Rd ; alpha-Amylcinnamyl acetate ; Heptyl cinnamate ; Gynosaponin S ; Colupdox a ; Erinacine A ; Erinacine B ; Demethoxyshogaol	M+H ; M+ACN+Na ; M+2ACN+H ; M+2ACN+2H ; M+2ACN+2H ; M+2ACN+2H ; 2M+Na ; 2M+Na ; M+2ACN+2H ; M+2ACN+H ; M+2ACN+H ; M+2ACN+H ; 2M+Na	C34H42O4 ; C26H37N5O2 ; C25H36O6 ; C48H82O18 ; C48H82O18 ; C48H82O18 ; C16H22O2 ; C16H22O2 ; C48H82O18 ; C25H36O6 ; C25H36O6 ; C25H36O6 ; C16H22O2
X331	3	515.815	0	Unknow	Unknow	Unknow	Unknow
X332	3	518.322	0	HMDB10387 ; HMDB10388	LysoPC(18:3(6Z9Z12Z)) ; LysoPC(18:3(9Z12Z15Z))	M+H ; M+H	C26H48NO7P ; C26H48NO7P
X333	3	527.316	0	HMDB09933 ; HMDB09969 ; HMDB09977 ; HMDB09988 ; HMDB09997 ; HMDB09999 ; HMDB10000 ; HMDB10002 ; HMDB10023 ; HMDB32305 ; HMDB33034	PIP(16:022:2(13Z16Z)) ; PIP(18:1(15Z)20:1(11Z)) ; PIP(18:1(9Z)20:1(11Z)) ; PIP(18:2(9Z12Z)20:0) ; PIP(20:018:2(9Z12Z)) ; PIP(20:1(11Z)18:1(11Z)) ; PIP(20:1(11Z)18:1(9Z)) ; PIP(20:2(11Z14Z)18:0) ; PIP(22:2(13Z16Z)16:0) ; N-(Heptan-4-yl)benzod13dioxole-5-carboxamide ; Antibiotic GR 95647X	M+2ACN+2H ; M+2ACN+2H ; M+2ACN+2H ; M+2ACN+2H ; M+2ACN+2H ; M+2ACN+2H ; M+2ACN+2H ; M+2ACN+2H ; M+2ACN+2H ; 2M+H ; M+2ACN+H	C47H88O16P2 ; C47H88O16P2 ; C47H88O16P2 ; C47H88O16P2 ; C47H88O16P2 ; C47H88O16P2 ; C47H88O16P2 ; C47H88O16P2 ; C47H88O16P2 ; C15H21NO3 ; C26H36O6
X336	3	530.287	0	HMDB40945 ; HMDB40963	Helianthoside A ; Matesaponin 2	M+2H ; M+2H	C53H86O21 ; C53H86O21
X337	3	538.386	0	HMDB11490 ; HMDB11520 ; HMDB32228 ; HMDB34505 ; HMDB34528 ; HMDB34644 ; HMDB34683 ; HMDB35326 ; HMDB36221 ; HMDB36640 ; HMDB37713 ; HMDB37719 ; HMDB39692	LysoPE(0:022:0) ; LysoPE(22:00:0) ; Dimethylbenzyl carbinyl hexanoate ; Soyasapogenol A ; Camelliagenin A ; Priverogenin B ; (3alphaOH20S24S)-319:2024-Diepoxydammarane-325-diol ; Ganoderiol A ; 2-(Dimethoxymethyl)-1-heptenylbenzene ; Furanofukinin ; Octyl phenylacetate ; 2-Phenylethyl octanoate ; 2024-Epoxy-2526-dihydroxydammaran-3-one	M+H ; M+H ; 2M+ACN+H ; M+ACN+Na ; M+ACN+Na ; M+ACN+Na ; M+ACN+Na ; M+ACN+Na ; 2M+ACN+H ; 2M+ACN+H ; 2M+ACN+H ; 2M+ACN+H ; M+ACN+Na	C27H56NO7P ; C27H56NO7P ; C16H24O2 ; C30H50O4 ; C30H50O4 ; C30H50O4 ; C30H50O4 ; C30H50O4 ; C16H24O2 ; C16H24O2 ; C16H24O2 ; C16H24O2 ; C30H50O4
X352	3	664.115	0	Unknow	Unknow	Unknow	Unknow

X36	3	164.056	0.103	HMDB00802 ; HMDB30393 ; HMDB31187 ; HMDB33581 ; HMDB36735 ; HMDB36936 ; HMDB39167 ; HMDB39169 ; HMDB39509 ; HMDB60077 ; HMDB00208 ; HMDB13701 ; HMDB29572 ; HMDB31157 ; HMDB31708 ; HMDB31872 ; HMDB33552 ; HMDB33966 ; HMDB41390 ; HMDB41393 ; HMDB41492	Pterin ; L-N-Carboxymethylserine ; Thialdine ; trans-o-Coumaric acid 2-glucoside ; Bilobalide A ; 1-O-p-Coumaroyl-beta-D-glucose ; 2-O-p-Coumaroyl-D-glucose ; 6-O-p-Coumaroyl-D-glucose ; trans-p-Coumaric acid 4-glucoside ; cis-beta-D-Glucosyl-2-hydroxycinnamate ; Oxoglutaric acid ; 3-Oxoglutaric acid ; Diethyl disulfide ; 23-Butanedithiol ; 12-Bis(methylthio)ethane ; Methyl propyl disulfide ; ()-13-Butanedithiol ; Di-2-propenyl disulfide 9CI ; (EE)-Di-1-propenyl disulfide ; (E)-1-Propenyl 2-propenyl disulfide ; 12-Butanedithiol	M+H ; M+H ; M+H ; M+2H ; M+2H ; M+2H ; M+2H ; M+2H ; M+2H ; M+2H ; M+NH4 ; M+NH4 ; M+ACN+H ; M+ACN+H ; M+ACN+H ; M+ACN+H ; M+ACN+H ; M+NH4 ; M+NH4 ; M+NH4 ; M+ACN+H	C6H5N5O ; C5H9NO5 ; C6H13NS2 ; C15H18O8 ; C15H18O8 ; C15H18O8 ; C15H18O8 ; C15H18O8 ; C15H18O8 ; C15H18O8 ; C5H6O5 ; C5H6O5 ; C4H10S2 ; C4H10S2 ; C4H10S2 ; C4H10S2 ; C4H10S2 ; C6H10S2 ; C6H10S2 ; C6H10S2 ; C4H10S2
X360	3	731.162	0	HMDB37965 ; HMDB37966 ; HMDB31981 ; HMDB39861	ent-Epicatechin-(4alpha-8)-ent-epicatechin 3-gallate ; ent-Epicatechin-(4alpha-8)-ent-epicatechin 3-gallate ; Niazidin ; 3-O-alpha-L-Arabinopyranosylproanthocyanidin A5	M+H ; M+H ; 2M+Na ; M+Na	C37H30O16 ; C37H30O16 ; C15H18N2O6S ; C35H32O16
X43	3	175.148	0.011	HMDB36683 ; HMDB59696 ; HMDB59826	57alpha-Dihydro-1447a-tetramethyl-4H-indene ; 1234Tetrahydro-157-trimethylnapthalene ; alpha-Ionene	M+H ; M+H ; M+H	C13H18 ; C13H18 ; C13H18
X64	3	202.02	0.025	HMDB01274 ; HMDB37112	dTDP ; Acifluorfen	M+2H ; M+ACN+2H	C10H16N2O11P2 ; C14H7ClF3NO5
X73	3	217.107	0.007	HMDB36131 ; HMDB60897 ; HMDB13253 ; HMDB61051	S-Furanopetasitin ; Diphenhydramine N-glucuronide ; N-Acetylhistamine ; N-desmethyltoremifene	M+2H ; M+2H ; M+ACN+Na ; M+ACN+2H	C24H32O5S ; C23H30NO7 ; C7H11N3O ; C25H26ClNO

X74	3	217.155	0.001	HMDB29140 ; HMDB00268 ; HMDB00449 ; HMDB05972 ; HMDB06406 ; HMDB11541 ; HMDB11571 ; HMDB15041 ; HMDB15073 ; HMDB31820 ; HMDB31846 ; HMDB32355 ; HMDB32478 ; HMDB32913 ; HMDB33616 ; HMDB36026 ; HMDB36184 ; HMDB36820 ; HMDB36823 ; HMDB38026 ; HMDB38216 ; HMDB38731 ; HMDB38897 ; HMDB41629 ; HMDB41997	Valyl-Valine ; Tetrahydrocorticosterone ; 5a-Tetrahydrocorticosterone ; Tetrahydrodeoxycortisol ; Ecgonine methyl ester ; MG(0:018:4(6Z9Z12Z15Z)0:0) ; MG(18:4(6Z9Z12Z15Z)0:00:0) ; Bimatoprost ; Salmeterol ; Neotussilagine ; Geranylacetone ; (-)-R-(E)-5-Isopropyl-8-methylnona-68-dien-2-one ; Polypropylene glycol (m w 1200-3000) ; 4-(266-Trimethyl-1-cyclohexen-1-yl)-2-butanone ; (S)-10-Gingerol ; Octahydro-6-isopropyl-2(1H)-naphthalenone ; 78-Dehydro-34-dihydro-beta-ionol ; beta-Ionol ; Theaspirane ; Dihydro-alpha-ionone ; 5-Isopropyl-2-(2-methylpropyl)-2-cyclohexen-1-one ; 47-Megastigmadien-9-ol ; (E)-2-(2-Octenyl)cyclopentanone ; (3S6E)-Nerolidol ; Pregnanetriolone	M+H ; M+2ACN+2H ; M+2ACN+2H ; M+2ACN+2H ; M+NH4 ; M+2ACN+2H ; M+2ACN+2H ; M+H+NH4 ; M+H+NH4 ; M+NH4 ; M+Na ; M+Na ; M+2ACN+H ; M+Na ; M+2ACN+2H ; M+Na ; M+Na ; M+Na ; M+Na ; M+Na ; M+Na ; M+Na ; M+Na ; M+Na ; M+2ACN+2H	C10H20N2O3 ; C21H34O4 ; C21H34O4 ; C21H34O4 ; C10H17NO3 ; C21H34O4 ; C21H34O4 ; C25H37NO4 ; C25H37NO4 ; C10H17NO3 ; C13H22O ; C13H22O ; C6H14O3 ; C13H22O ; C21H34O4 ; C13H22O ; C13H22O ; C13H22O ; C13H22O ; C13H22O ; C13H22O ; C13H22O ; C13H22O ; C13H22O ; C21H34O4
X79	3	223.563	0.003	HMDB01397 ; HMDB11670 ; HMDB59639	Guanosine monophosphate ; 8-Oxo-dGMP ; Cyclic pyranopterin monophosphate	M+2ACN+2H ; M+2ACN+2H ; M+2ACN+2H	C10H14N5O8P ; C10H14N5O8P ; C10H14N5O8P
X82	3	225.087	0.001	HMDB00732 ; HMDB11631 ; HMDB12819 ; HMDB14787 ; HMDB29397 ; HMDB00017 ; HMDB00978 ; HMDB02349 ; HMDB02432 ; HMDB06331 ; HMDB15249 ; HMDB29832 ; HMDB30080 ; HMDB30816 ; HMDB31663 ; HMDB32923 ; HMDB32988 ; HMDB33882 ; HMDB36556 ; HMDB38674 ; HMDB59762 ; HMDB60328	Hydroxykynurenine ; L-3-Hydroxykynurenine ; 5-Hydroxykynurenine ; Stavudine ; L-Nicotianine ; 4-Pyridoxic acid ; 4-(2-Aminophenyl)-24-dioxobutanoic acid ; trans-trans-Muconic acid ; Sumikis acid ; ciscis-Muconic acid ; Atovaquone ; 8-Carboxymethyldihydrochelerythrine ; Wampetin ; 6-Acetyl-22-dimethyl-2H-1-benzopyran ; 3-(3-Methylbutylidene)-1(3H)-isobenzofuranone ; Kojic acid ; 5-Hydroxymaltol ; Glycyrol ; Glycyrrhizaisoflavone B ; gamma-L-Glutamyl-gamma-L-glutamyl-L-methionine ; 23-Methylenesuccinic acid ; 1-Nitro-56-dihydroxy-dihydronaphthalene	M+H ; M+H ; M+H ; M+H ; M+H ; M+ACN+H ; M+NH4 ; M+2ACN+H ; M+2ACN+H ; M+2ACN+H ; M+2ACN+2H ; M+ACN+2H ; M+2ACN+2H ; M+Na ; M+Na ; M+2ACN+H ; M+2ACN+H ; M+2ACN+2H ; M+2ACN+2H ; M+ACN+2H ; M+2ACN+H ; M+NH4	C10H12N2O4 ; C10H12N2O4 ; C10H12N2O4 ; C10H12N2O4 ; C10H12N2O4 ; C8H9NO4 ; C10H9NO4 ; C6H6O4 ; C6H6O4 ; C6H6O4 ; C22H19ClO3 ; C23H21NO6 ; C21H18O6 ; C13H14O2 ; C13H14O2 ; C6H6O4 ; C6H6O4 ; C21H18O6 ; C21H18O6 ; C15H25N3O8S ; C6H6O4 ; C10H9NO4

X83	3	226.081	0.004	HMDB35067 ; HMDB15526 ; HMDB28682 ; HMDB28724 ; HMDB28797 ; HMDB28839 ; HMDB28850 ; HMDB28855 ; HMDB29039 ; HMDB29149 ; HMDB29424	Melleolide L ; Ketazolam ; Alanyl-Asparagine ; Asparaginyl-Alanine ; Glutaminyl-Glycine ; Glycyl-Glutamine ; Glycyl-Serine ; Glycyl-Gamma-glutamate ; Serinyl-Glycine ; Gamma-glutamyl-Glycine ; L-4-Hydroxyglutamine	M+2H ; M+2ACN+2H ; M+Na ; M+Na ; M+Na ; M+Na ; M+ACN+Na ; M+Na ; M+ACN+Na ; M+Na ; M+ACN+Na	C23H27ClO7 ; C20H17ClN2O3 ; C7H13N3O4 ; C7H13N3O4 ; C7H13N3O4 ; C7H13N3O4 ; C5H10N2O4 ; C7H13N3O4 ; C5H10N2O4 ; C7H13N3O4 ; C5H10N2O4
X88	3	231.009	0.019	HMDB00691 ; HMDB01352 ; HMDB06938 ; HMDB31159	Malonic acid ; Hydroxypyruvic acid ; Tartronate semialdehyde ; Garcinia acid	2M+Na ; 2M+Na ; 2M+Na ; M+Na	C3H4O4 ; C3H4O4 ; C3H4O4 ; C6H8O8
X99	3	245.968	0.008	Unknow	Unknow	Unknow	Unknow
X1	4	87.004	0	Unknow	Unknow	Unknow	Unknow
X102	4	250.955	0	HMDB41188	Bis(methylsulfonylmethyl) disulfide	M+H	C4H10O4S4
X11	4	131.974	0	Unknow	Unknow	Unknow	Unknow
X112	4	270.958	0	Unknow	Unknow	Unknow	Unknow
X113	4	272.955	0	Unknow	Unknow	Unknow	Unknow
X114	4	276.923	0	Unknow	Unknow	Unknow	Unknow
X116	4	286.932	0	HMDB39734	23579-Pentathiadecane 22-dioxide	M+Na	C5H12O2S5
X119	4	288.929	0	Unknow	Unknow	Unknow	Unknow
X122	4	292.94	0	Unknow	Unknow	Unknow	Unknow
X132	4	302.906	0	Unknow	Unknow	Unknow	Unknow
X135	4	304.903	0	Unknow	Unknow	Unknow	Unknow
X147	4	328.917	0	Unknow	Unknow	Unknow	Unknow
X160	4	366.872	0	Unknow	Unknow	Unknow	Unknow
X163	4	375.97	0	Unknow	Unknow	Unknow	Unknow
X169	4	382.847	0	Unknow	Unknow	Unknow	Unknow
X171	4	384.115	0	HMDB00912 ; HMDB01062 ; HMDB01121 ; HMDB01367 ; HMDB02817 ; HMDB06480 ; HMDB40546 ; HMDB59626	Succinyladenosine ; N-Acetyl-D-Glucosamine 6-Phosphate ; N-Acetyl-D-mannosamine 6-phosphate ; N-Acetyl-glucosamine 1-phosphate ; N-Acetylglucosamine 6-phosphate ; N-Acetyl-D-galactosamine 1-phosphate ; Pyriminobac-methyl ; N-acetyl-alpha-D-galactosamine 1-phosphate	M+H ; M+2ACN+H ; M+2ACN+H ; M+2ACN+H ; M+2ACN+H ; M+2ACN+H ; M+Na ; M+2ACN+H	C14H17N5O8 ; C8H16NO9P ; C8H16NO9P ; C8H16NO9P ; C8H16NO9P ; C8H16NO9P ; C17H19N3O6 ; C8H16NO9P
X181	4	415.772	0	HMDB15342	Clarithromycin	M+2ACN+2H	C38H69NO13
X185	4	431.002	0	HMDB41985	Phosalone	M+ACN+Na	C12H15ClNO4PS2
X191	4	460.808	0	HMDB10166	PS(18:022:5(7Z10Z13Z16Z19Z))	M+2ACN+2H	C46H80NO10P
X243	4	163.087	0	HMDB01297 ; HMDB33504 ; HMDB34576 ; HMDB35357 ; HMDB35847 ; HMDB35848 ; HMDB04461 ; HMDB29737 ; HMDB33131 ; HMDB35281	Norcotinine ; AF Toxin II ; 3-Acetoxyscirpene-415-diol ; Blumealactone C ; 4-Acetoxyscirpene-315-diol ; Monoacetoxyscirpenol ; Benzamide ; 1H-Indole-3-carboxaldehyde ; 3-Acetylpyridine ; 2-Acetylpyridine	M+H ; M+2H ; M+2H ; M+2H ; M+2H ; M+2H ; M+ACN+H ; M+NH4 ; M+ACN+H ; M+ACN+H	C9H10N2O ; C17H24O6 ; C17H24O6 ; C17H24O6 ; C17H24O6 ; C17H24O6 ; C7H7NO ; C9H7NO ; C7H7NO ; C7H7NO

X40	4	169.036	0	HMDB00289	Uric acid	M+H	C5H4N4O3
X54	4	190.089	0	HMDB12286 ; HMDB30657 ; HMDB34474 ; HMDB35053 ; HMDB36694 ; HMDB36897 ; HMDB36900 ; HMDB36945 ; HMDB41002 ; HMDB41231 ; HMDB41349 ; HMDB31717 ; HMDB32271 ; HMDB32410 ; HMDB32495 ; HMDB36193 ; HMDB37619 ; HMDB38228 ; HMDB38299 ; HMDB39653 ; HMDB40413 ; HMDB41208 ; HMDB41306 ; HMDB61100	S-Prenyl-L-cysteine ; Carinol ; Niveusin C ; Gibberellin A52 ; 15-Hydroxyleptocarpin ; Gibberellin A17 ; Gibberellin A66 ; Gibberellin A125 ; (1E4Z6a8b10a)-8-(2-Methylbutanoyloxy)-1015-dihydroxy-3-oxo-1411(13)-germacratrien-126-olide ; Gibberellin A99 ; Gibberellin A102 ; 3-(Methylthio)propyl acetate ; Ethyl 3-mercaptobutyrate ; Methyl 3-(methylthio)butanoate ; Propyl 2-mercaptopropionate ; 3-Mercapto-3-methylbutyl formate ; Methyl 4-(methylthio)butyrate ; 4-O-alpha-D-Galactopyranosylcalystegine B2 ; Methylthiomethyl butyrate ; 246-Trimethyl-135-dioxathiane ; Ethyl 3-(methylthio)propanoate ; De-O-methylsimmondsin ; Methyl 2-(methylthio)butyrate ; 6-oxo-famciclovir	M+H ; M+2H ; M+2H ; M+2H ; M+2H ; M+2H ; M+2H ; M+2H ; M+2H ; M+2H ; M+2H ; M+ACN+H ; M+ACN+H ; M+ACN+H ; M+ACN+H ; M+ACN+H ; M+ACN+H ; M+ACN+2H ; M+ACN+H ; M+ACN+H ; M+ACN+H ; M+H+NH4 ; M+ACN+H ; M+ACN+2H	C8H15NO2S ; C20H26O7 ; C20H26O7 ; C20H26O7 ; C20H26O7 ; C20H26O7 ; C20H26O7 ; C20H26O7 ; C20H26O7 ; C20H26O7 ; C20H26O7 ; C6H12O2S ; C6H12O2S ; C6H12O2S ; C6H12O2S ; C6H12O2S ; C6H12O2S ; C13H23NO9 ; C6H12O2S ; C6H12O2S ; C6H12O2S ; C15H23NO9 ; C6H12O2S ; C14H19N5O5
X67	4	207.042	0	HMDB04823 ; HMDB13636 ; HMDB15093 ; HMDB61116	Lanthionine ketimine ; Pyrroloquinoline quinone ; Carboplatin ; 2-aminophenol sulphate	M+NH4 ; M+2ACN+2H ; M+ACN+2H ; M+NH4	C6H7NO4S ; C14H6N2O8 ; C6H12N2O4Pt ; C6H7NO4S
X75	4	217.518	0	Unknow	Unknow	Unknow	Unknow
X85	4	228.973	0	Unknow	Unknow	Unknow	Unknow
X86	4	229.519	0	Unknow	Unknow	Unknow	Unknow
X89	4	234.982	0	Unknow	Unknow	Unknow	Unknow
X108	5	261.89	0	Unknow	Unknow	Unknow	Unknow
X110	5	265.023	0.05	HMDB38142 ; HMDB39791 ; HMDB39792 ; HMDB39796	58-Dihydro-6-(4-methyl-3-pentenyl)-1234-tetrathiocin ; 23-Dihydro-5-methyl-3-thiophenethiol ; 45-Dihydro-2-methyl-3-thiophenethiol ; 45-Dihydro-5-methyl-3-thiophenethiol	M+H ; 2M+H ; 2M+H ; 2M+H	C10H16S4 ; C5H8S2 ; C5H8S2 ; C5H8S2
X12	5	133.015	0.02	Unknow	Unknow	Unknow	Unknow
X159	5	365.001	0.006	Unknow	Unknow	Unknow	Unknow
X189	5	449.945	0.047	Unknow	Unknow	Unknow	Unknow
X233	5	127.972	0	Unknow	Unknow	Unknow	Unknow
X247	5	171.011	0	Unknow	Unknow	Unknow	Unknow
X250	5	186.106	0	HMDB32841 ; HMDB32842 ; HMDB36822 ; HMDB31682 ; HMDB34913 ; HMDB37554	5-Megastigmen-7-yne-39-diol 9-glucoside ; 5-Megastigmen-7-yne-39-diol 3-glucoside ; (3S7E9S)-9-Hydroxy-47-megastigmadien-3-one 9-glucoside ; ()-2-Methylthiazolidine ; Imazamethabenz-methyl ; ()-Rollipyrrole	M+2H ; M+2H ; M+2H ; M+2ACN+H ; M+2ACN+2H ; M+2ACN+2H	C19H30O7 ; C19H30O7 ; C19H30O7 ; C4H9NS ; C16H20N2O3 ; C16H20N2O3

X258	5	210.091	0	HMDB32757 ; HMDB33000 ; HMDB60652 ; HMDB28684 ; HMDB28768 ; HMDB32867 ; HMDB33927 ; HMDB37621 ; HMDB39465 ; HMDB39468 ; HMDB40186	3-Methyl-9H-carbazole-9-carboxaldehyde ; 10-Methylacridone ; 2-Hydroxyiminostilbene ; Alanyl-Cysteine ; Cysteinyl-Alanine ; Capillin ; 3-Hydroxymugineic acid ; 4-Methyl-4-(methylthio)-2-pentanone ; S-Methyl hexanethioate ; S-Methyl 4-methylpentanethioate ; 3-(Methylthio)hexanal	M+H ; M+H ; M+H ; M+NH4 ; M+NH4 ; M+ACN+H ; M+2ACN+2H ; M+ACN+Na ; M+ACN+Na ; M+ACN+Na ; M+ACN+Na	C14H11NO ; C14H11NO ; C14H11NO ; C6H12N2O3S ; C6H12N2O3S ; C12H8O ; C12H20N2O9 ; C7H14OS ; C7H14OS ; C7H14OS ; C7H14OS
X263	5	230.096	0	HMDB03045 ; HMDB41887 ; HMDB29909 ; HMDB37108 ; HMDB37603 ; HMDB39852 ; HMDB34294 ; HMDB38183 ; HMDB39104 ; HMDB59611	Ergothioneine ; Endalin ; Gein ; 3-Hydroxychavicol 1-rhamnosyl-(1-6)-glucoside ; Eugenol O-a-L-Arabinofuranosyl-(1-6)-b-D-glucopyranoside ; Deoxynivalenol 3-glucoside ; 1311-Tridecatriene-579-triyne ; 3-(4-Methyl-3-pentenyl)thiophene ; (3E5Z)-135-Tridecatriene-7911-triyne ; Thiomorpholine 3-carboxylate	M+H ; M+H ; M+2H ; M+2H ; M+2H ; M+2H ; M+ACN+Na ; M+ACN+Na ; M+ACN+Na ; M+2ACN+H	C9H15N3O2S ; C9H12FN3O3 ; C21H30O11 ; C21H30O11 ; C21H30O11 ; C21H30O11 ; C13H10 ; C10H14S ; C13H10 ; C5H9NO2S
X265	5	232.092	0	HMDB11165 ; HMDB14414 ; HMDB15673 ; HMDB28753 ; HMDB28837 ; HMDB33001 ; HMDB33969 ; HMDB34273 ; HMDB34965 ; HMDB39817	L-beta-aspartyl-L-glycine ; Chlorotrianisene ; Carglumic acid ; Aspartyl-Glycine ; Glycyl-Aspartate ; 2-Methylbenzothiazole ; Benzyl isothiocyanate ; Benzyl thiocyanate ; 1-Methyl-3-(2-thiazolyl)-1H-indole ; Dihydro-46-dimethyl-4H-135-dithiazine	M+ACN+H ; M+2ACN+2H ; M+ACN+H ; M+ACN+H ; M+ACN+H ; M+2ACN+H ; M+2ACN+H ; M+2ACN+H ; M+NH4 ; M+2ACN+H	C6H10N2O5 ; C23H21ClO3 ; C6H10N2O5 ; C6H10N2O5 ; C6H10N2O5 ; C8H7NS ; C8H7NS ; C8H7NS ; C12H10N2S ; C5H11NS2

X275	5	253.081	0	HMDB04086 ; HMDB35993 ; HMDB35486 ; HMDB00590 ; HMDB01138 ; HMDB01923 ; HMDB02335 ; HMDB05807 ; HMDB11639 ; HMDB13689 ; HMDB28761 ; HMDB29013 ; HMDB29649 ; HMDB29756 ; HMDB29842 ; HMDB31328 ; HMDB31574 ; HMDB31701 ; HMDB32345 ; HMDB32499 ; HMDB33184 ; HMDB34130 ; HMDB34212 ; HMDB34864 ; HMDB35370 ; HMDB39097 ; HMDB39110 ; HMDB40331 ; HMDB40590 ; HMDB41751 ; HMDB41752 ; HMDB59964 ; HMDB59999	5-Hydroxy-N-formylkynurenine ; N5-(34-Dioxo-15-cyclohexadien-1-yl)-L-glutamine ; 6-Caffeoylsucrose ; Glutarylglycine ; N-Acetylglutamic acid ; Naproxen ; Aspartyl-L-proline ; Gallic acid ; Topaquinone ; Peonidin-3-glucoside ; Aspartyl-Proline ; Prolyl-Aspartate ; 246-Trihydroxybenzoic acid ; (1R2S3R)-2-Acetyl-4(5)-(1234-tetrahydroxybutyl)imidazole ; Betalamic acid ; 1-Isothiocyanatobutane ; 1-Isothiocyanato-6-(methylthio)hexane ; 1-(gamma-Glutamylamino)cyclopropanecarboxylic acid ; Isobutyl isothiocyanate ; Pyrrolidino-12E-4H-24-dimethyl-135-dithiazine ; Pondaplin ; Osthenol ; (R)-Kawain ; 24-Dihydroxy-7-methoxy-2H-14-benzoxazin-3(4H)-one ; Pyranocyanin B ; Demethylbatatasin IV ; (2S3S)-alpha-Amino-2-carboxy-5-oxo-1-pyrrolidinebutanoic acid ; xi-25-Dihydro-24-dimethylthiazole ; Benzyl methyl disulfide ; Isopeonidin 3-galactoside ; Isopeonidin 3-glucoside ; 234-Trihydroxybenzoic acid ; Gentisuric acid	M+H ; M+H ; M+2H ; M+ACN+Na ; M+ACN+Na ; M+Na ; M+Na ; M+2ACN+H ; M+ACN+H ; M+ACN+2H ; M+Na ; M+Na ; M+2ACN+H ; M+Na ; M+ACN+H ; 2M+Na ; M+ACN+Na ; M+Na ; 2M+Na ; M+ACN+Na ; M+Na ; M+Na ; M+Na ; M+ACN+H ; M+H+NH4 ; M+Na ; M+Na ; 2M+Na ; M+2ACN+H ; M+ACN+2H ; M+ACN+2H ; M+2ACN+H ; M+ACN+H	C11H12N2O5 ; C11H12N2O5 ; C21H28O14 ; C7H11NO5 ; C7H11NO5 ; C14H14O3 ; C9H14N2O5 ; C7H6O5 ; C9H9NO5 ; C22H23O11 ; C9H14N2O5 ; C9H14N2O5 ; C7H6O5 ; C9H14N2O5 ; C9H9NO5 ; C5H9NS ; C8H15NS2 ; C9H14N2O5 ; C5H9NS ; C8H15NS2 ; C14H14O3 ; C14H14O3 ; C14H14O3 ; C9H9NO5 ; C24H23O11 ; C14H14O3 ; C9H14N2O5 ; C5H9NS ; C8H10S2 ; C22H23O11 ; C22H23O11 ; C7H6O5 ; C9H9NO5
X281	5	268.052	0	HMDB31793 ; HMDB32026 ; HMDB32884 ; HMDB34395	Heptenophos ; Sinalexin ; Allura red AC ; 2-Propenyl 3-(2-propenylsulfonyl)-1-propenyl disulfide	M+NH4 ; M+ACN+Na ; M+2ACN+2H ; M+NH4	C9H12ClO4P ; C10H8N2OS ; C18H16N2O8S2 ; C9H14O2S3
X296	5	355.11	0	HMDB34854 ; HMDB61000 ; HMDB11658 ; HMDB13189 ; HMDB28830 ; HMDB29082 ; HMDB29496 ; HMDB37356 ; HMDB37363 ; HMDB37537 ; HMDB38287 ; HMDB38824 ; HMDB39002 ; HMDB39878 ; HMDB39924 ; HMDB40371 ; HMDB40861 ; HMDB41358	Flumioxazin ; 4-(4-Chlorophenyl)-1-4-(4-fluorophenyl)-4-oxobutyl-pyridinium (HPP) ; 28-Dihydroxyquinoline-beta-D-glucuronide ; 3-Indole carboxylic acid glucuronide ; Glutamyl-Tryptophan ; Tryptophyl-Glutamate ; Fukiic acid ; Quercetin 47-diglucoside ; Quercetin 34-diglucoside ; Quercetin 3-glucosyl-(1-2)-galactoside ; Myricetin 3-neohesperidoside ; Hypoletin 8-gentiobioside ; Mytilin A ; Vicinin 2 ; 6-Hydroxykaempferol 67-diglucoside ; Herbacetin 38-diglucoside ; Myricetin 3-robinobioside ; Quercetin 3-beta-laminaribioside	M+H ; M+H ; M+NH4 ; M+NH4 ; M+Na ; M+Na ; M+2ACN+H ; M+2ACN+2H ; M+2ACN+2H ; M+2ACN+2H ; M+2ACN+2H ; M+2ACN+2H ; M+Na ; M+2ACN+2H ; M+2ACN+2H ; M+2ACN+2H ; M+2ACN+2H ; M+2ACN+2H	C19H15FN2O4 ; C21H18ClFNO ; C15H15NO8 ; C15H15NO8 ; C16H18N3O5 ; C16H18N3O5 ; C11H12O8 ; C27H30O17 ; C27H30O17 ; C27H30O17 ; C27H30O17 ; C27H30O17 ; C13H20N2O8 ; C27H30O17 ; C27H30O17 ; C27H30O17 ; C27H30O17 ; C27H30O17
X307	5	415.049	0	HMDB14952	Meloxicam	M+ACN+Na	C14H13N3O4S2
X374	5	845.984	0	HMDB40908	4-Methylnonacosane	2M+H	C30H62

X115	6	282.973	0	HMDB31853 ; HMDB32939 ; HMDB39787 ; HMDB39788	2-Thiophenemethanethiol ; 3-(Methylthio)thiophene ; 2-Methyl-3-thiophenethiol ; 5-Methyl-3-thiophenethiol	2M+Na ; 2M+Na ; 2M+Na ; 2M+Na	C5H6S2 ; C5H6S2 ; C5H6S2 ; C5H6S2
X13	6	133.059	0.027	HMDB60549 ; HMDB60624	Norketamine ; Ethionamide sulphoxide	M+ACN+2H ; M+2ACN+2H	C12H14ClNO ; C8H10N2OS
X187	6	446.055	0.001	HMDB15276 ; HMDB29202 ; HMDB38423	Sulfoxone ; hesperetin 3-O-sulfate ; Gluconasturtiin	M+ACN+H ; M+ACN+Na ; M+Na	C14H16N2O6S3 ; C16H14O9S ; C15H21NO9S2
X198	6	474.805	0	Unknow	Unknow	Unknow	Unknow
X22	6	146.165	0	HMDB01257 ; HMDB39492	Spermidine ; 117-Diamino-4913-triazaheptadecane	M+H ; M+H+NH4	C7H19N3 ; C14H35N5
X253	6	195.088	0	HMDB01847 ; HMDB14962 ; HMDB29915 ; HMDB29916 ; HMDB29965 ; HMDB31437 ; HMDB31449 ; HMDB33816 ; HMDB34219 ; HMDB34221 ; HMDB34222 ; HMDB37295 ; HMDB14545 ; HMDB00300 ; HMDB00466 ; HMDB12488 ; HMDB14342 ; HMDB30171 ; HMDB34236 ; HMDB39031 ; HMDB60760 ; HMDB60788	Caffeine ; Enprofylline ; D-4-O-Methyl-myo-inositol ; 4-O-Methyl-myo-inositol ; Methyl beta-D-glucopyranoside ; (-)-Bornesitol ; Sequoyitol ; Mytilitol ; D-Pinitol ; L-Quebrachitol ; L-Pinitol ; 3-(2-Mercapto-1-methylpropyl)thio-2-butanol ; Nisoldipine ; Uracil ; 3-Methylindole ; 1234-Tetrahydro-beta-carboline ; Fluconazole ; N-Methyl-14-O-demethylepiporphyroxine ; Benzenepropanenitrile ; trans-O-Methylgrandmarin ; 4-Carboxypyrazole ; 6-O-Desmethyl-mycophenolic acid	M+H ; M+H ; M+H ; M+H ; M+H ; M+H ; M+H ; M+H ; M+H ; M+H ; M+H ; M+H ; M+2H ; M+2ACN+H ; M+ACN+Na ; M+Na ; M+2ACN+2H ; M+H+NH4 ; M+ACN+Na ; M+2ACN+2H ; M+2ACN+H ; M+2ACN+2H	C8H10N4O2 ; C8H10N4O2 ; C7H14O6 ; C7H14O6 ; C7H14O6 ; C7H14O6 ; C7H14O6 ; C7H14O6 ; C7H14O6 ; C7H14O6 ; C7H14O6 ; C8H18OS2 ; C20H24N2O6 ; C4H4N2O2 ; C9H9N ; C11H12N2 ; C13H12F2N6O ; C20H21NO6 ; C9H9N ; C16H18O6 ; C4H4N2O2 ; C16H18O6
X27	6	151.07	0.012	HMDB14623 ; HMDB28783 ; HMDB29014 ; HMDB34911	Lenalidomide ; Cysteinyl-Proline ; Prolyl-Cysteine ; (S)-3-(Cyanophenylmethyl)amino-3-oxopropanoic acid	M+ACN+2H ; M+2ACN+2H ; M+2ACN+2H ; M+2ACN+2H	C13H13N3O3 ; C8H14N2O3S ; C8H14N2O3S ; C11H10N2O3
X285	6	279.159	0	HMDB01518 ; HMDB13248 ; HMDB13835 ; HMDB33244 ; HMDB12234 ; HMDB29165 ; HMDB29314 ; HMDB30330 ; HMDB30993 ; HMDB31174 ; HMDB31342 ; HMDB31351 ; HMDB31476 ; HMDB31477 ; HMDB31484 ; HMDB31500 ; HMDB31504 ; HMDB31593 ; HMDB31603 ; HMDB31681 ; HMDB32391 ; HMDB32460 ; HMDB33793 ; HMDB36143 ; HMDB38271 ; HMDB38962 ; HMDB39819 ; HMDB40214 ; HMDB40591	Alpha-CEHC ; Monoethylhexyl phthalic acid ; Diisobutyl phthalate ; Dibutyl phthalate ; Histidinal ; 13-Diacetylpropane ; Isopentenyl acetate ; 1-Methoxy-3-methylene-2-pentanone ; 5-Nonyltetrahydro-2-oxo-3-furancarboxylic acid ; 4-Methyl-5-hexanolide ; Cyclohexanecarboxylic acid ; Cyclohexyl formate ; 23-Heptanedione ; 34-Heptanedione ; 2-Heptenoic acid ; Methyl 2E-hexenoate ; Methyl (Z)-3-hexenoate ; 5-Methyl-23-hexanedione ; Ethyl 4-pentenoate ; Dihydro-5-propyl-2(3H)-furanone ; 3-Methyl-3-butenyl acetate ; 4-Pentenyl acetate ; 4-Heptenoic acid ; Monomenthyl succinate ; 2-Hexenyl formate ; Ethyl tiglate ; 24-Dimethyl-2-pentenoic acid ; cis-3-Hexenyl formate ; Allyl butyrate	M+H ; M+H ; M+H ; M+H ; 2M+H ; 2M+Na ; 2M+Na ; 2M+Na ; M+Na ; 2M+Na ; 2M+Na ; 2M+Na ; 2M+Na ; 2M+Na ; 2M+Na ; 2M+Na ; 2M+Na ; 2M+Na ; 2M+Na ; 2M+Na ; 2M+Na ; 2M+Na ; 2M+Na ; M+Na ; 2M+Na ; 2M+Na ; 2M+Na ; 2M+Na ; 2M+Na	C16H22O4 ; C16H22O4 ; C16H22O4 ; C16H22O4 ; C6H9N3O ; C7H12O2 ; C7H12O2 ; C7H12O2 ; C14H24O4 ; C7H12O2 ; C7H12O2 ; C7H12O2 ; C7H12O2 ; C7H12O2 ; C7H12O2 ; C7H12O2 ; C7H12O2 ; C7H12O2 ; C7H12O2 ; C7H12O2 ; C7H12O2 ; C7H12O2 ; C7H12O2 ; C14H24O4 ; C7H12O2 ; C7H12O2 ; C7H12O2 ; C7H12O2 ; C7H12O2

X292	6	313.273	0	HMDB02272 ; HMDB02871 ;<br>HMDB02936 ; HMDB13225 ;<br>HMDB39094	778811111212-Hexahydro-yy-Carotene ;<br>Phytofluene ; cis-77881112-Hexahydro-<br>Carotene ; Capryloylcholine ; (15Z9Z)-<br>77881112-Hexahydrolycopene	M+2ACN+2H ; M+2ACN+2H ;<br>M+2ACN+2H ; M+2ACN+H ;<br>M+2ACN+2H	C40H62 ; C40H62 ; C40H62 ; C13H28NO2 ; C40H62
X302	6	392.287	0	HMDB07861 ; HMDB07862 ;<br>HMDB07863 ; HMDB07864 ;<br>HMDB07865 ; HMDB07941 ;<br>HMDB07942 ; HMDB07951 ;<br>HMDB08165 ; HMDB08198 ;<br>HMDB08494 ; HMDB08876 ;<br>HMDB08935 ; HMDB08936 ;<br>HMDB08944 ; HMDB08945 ;<br>HMDB08967 ; HMDB08976 ;<br>HMDB08995 ; HMDB08996 ;<br>HMDB09005 ; HMDB09027 ;<br>HMDB09036 ; HMDB09037 ;<br>HMDB09060 ; HMDB09069 ;<br>HMDB09070 ; HMDB09091 ;<br>HMDB09092 ; HMDB09100 ;<br>HMDB09101 ; HMDB09123 ;<br>HMDB09132 ; HMDB09156 ;<br>HMDB09165 ; HMDB09197 ;<br>HMDB09261 ; HMDB09287 ;<br>HMDB09292 ; HMDB09293 ;<br>HMDB09319 ; HMDB09324 ;<br>HMDB09352 ; HMDB09357 ;<br>HMDB09388 ; HMDB09389 ;<br>HMDB09421 ; HMDB09422 ;<br>HMDB09453 ; HMDB09548 ;<br>HMDB09584 ; HMDB09616 ;<br>HMDB09649 ; HMDB31129 ;	PA(18:018:2(9Z12Z)) ;<br>PA(18:1(11Z)18:1(11Z)) ;<br>PA(18:1(11Z)18:1(9Z)) ;<br>PA(18:1(9Z)18:1(11Z)) ;<br>PA(18:1(9Z)18:1(9Z)) ;<br>PC(15:018:3(6Z9Z12Z)) ;<br>PC(15:018:3(9Z12Z15Z)) ;<br>PC(15:020:5(5Z8Z11Z14Z17Z)) ;<br>PC(18:3(6Z9Z12Z)15:0) ;<br>PC(18:3(9Z12Z15Z)15:0) ;<br>PC(20:5(5Z8Z11Z14Z17Z)15:0) ;<br>PE(14:1(9Z)22:2(13Z16Z)) ;<br>PE(16:020:3(5Z8Z11Z)) ;<br>PE(16:020:3(8Z11Z14Z)) ;<br>PE(16:022:5(4Z7Z10Z13Z16Z)) ;<br>PE(16:022:5(7Z10Z13Z16Z19Z)) ;<br>PE(16:1(9Z)20:2(11Z14Z)) ;<br>PE(16:1(9Z)22:4(7Z10Z13Z16Z)) ;<br>PE(18:018:3(6Z9Z12Z)) ;<br>PE(18:018:3(9Z12Z15Z)) ;<br>PE(18:020:5(5Z8Z11Z14Z17Z)) ;<br>PE(18:1(11Z)18:2(9Z12Z)) ;<br>PE(18:1(11Z)20:4(5Z8Z11Z14Z)) ;<br>PE(18:1(11Z)20:4(8Z11Z14Z17Z)) ;<br>PE(18:1(9Z)18:2(9Z12Z)) ;<br>PE(18:1(9Z)20:4(5Z8Z11Z14Z)) ;<br>PE(18:1(9Z)20:4(8Z11Z14Z17Z)) ;	M+2ACN+2H ; M+2ACN+2H ;<br>M+2ACN+2H ; M+2ACN+2H ;<br>M+2ACN+2H ; M+ACN+2H ;<br>M+ACN+2H ; M+H+NH4 ;<br>M+ACN+2H ; M+ACN+2H ;<br>M+H+NH4 ; M+ACN+2H ;<br>M+ACN+2H ; M+ACN+2H ;<br>M+H+NH4 ; M+H+NH4 ;<br>M+ACN+2H ; M+H+NH4 ;<br>M+ACN+2H ; M+ACN+2H ;<br>M+H+NH4 ; M+ACN+2H ;<br>M+H+NH4 ; M+H+NH4 ;<br>M+ACN+2H ; M+H+NH4 ;<br>M+H+NH4 ; M+ACN+2H ;<br>M+ACN+2H ; M+H+NH4 ;<br>M+H+NH4 ; M+ACN+2H ;<br>M+H+NH4 ; M+ACN+2H ;<br>M+H+NH4 ; M+H+NH4 ; M+H+NH4<br>; M+ACN+2H ; M+H+NH4 ;<br>M+H+NH4 ; M+ACN+2H ;<br>M+H+NH4 ; M+ACN+2H ;<br>M+H+NH4 ; M+H+NH4 ; M+H+NH4<br>; M+H+NH4 ; M+H+NH4 ;<br>M+H+NH4 ; M+ACN+2H ;<br>M+H+NH4 ; M+H+NH4 ; M+H+NH4<br>; M+2ACN+2H ; M+NH4	C39H73O8P ; C39H73O8P ; C39H73O8P ; C39H73O8P ;<br>C39H73O8P ; C41H76NO8P ; C41H76NO8P ; C43H76NO8P ;<br>C41H76NO8P ; C41H76NO8P ; C43H76NO8P ; C41H76NO8P<br>; C41H76NO8P ; C41H76NO8P ; C43H76NO8P ;<br>C43H76NO8P ; C41H76NO8P ; C43H76NO8P ; C41H76NO8P<br>; C41H76NO8P ; C43H76NO8P ; C41H76NO8P ;<br>C43H76NO8P ; C43H76NO8P ; C41H76NO8P ; C43H76NO8P<br>; C43H76NO8P ; C41H76NO8P ; C41H76NO8P ;<br>C43H76NO8P ; C43H76NO8P ; C41H76NO8P ; C43H76NO8P<br>; C41H76NO8P ; C43H76NO8P ; C43H76NO8P ;<br>C43H76NO8P ; C41H76NO8P ; C43H76NO8P ; C43H76NO8P<br>; C41H76NO8P ; C43H76NO8P ; C41H76NO8P ;<br>C43H76NO8P ; C43H76NO8P ; C43H76NO8P ; C43H76NO8P<br>; C43H76NO8P ; C43H76NO8P ; C41H76NO8P ;<br>C43H76NO8P ; C43H76NO8P ; C43H76NO8P ; C39H73O8P ;<br>C22H34N2O3

X305	6	413.266	0	HMDB30057 ; HMDB00328 ; HMDB00460 ; HMDB00467 ; HMDB00503 ; HMDB01925 ; HMDB09210 ; HMDB09467 ; HMDB09690 ; HMDB12866 ; HMDB29669 ; HMDB32176 ; HMDB33196 ; HMDB34469 ; HMDB34470 ; HMDB34472 ; HMDB34556 ; HMDB35008 ; HMDB35016 ; HMDB35017 ; HMDB35181 ; HMDB36025 ; HMDB36240 ; HMDB36386 ; HMDB36391 ; HMDB37194 ; HMDB37604 ; HMDB37623 ; HMDB37720 ; HMDB38605 ; HMDB39101 ; HMDB40431 ; HMDB41454	Boviquinone 4 ; 12-Ketodeoxycholic acid ; 7-Hydroxy-3-oxocholanoic acid ; Nutriacholic acid ; 7a-Hydroxy-3-oxo-5b-cholanoic acid ; Ibuprofen ; PE(18:4(6Z9Z12Z15Z)22:6(4Z7Z10Z13Z16Z19Z)) ; PE(20:5(5Z8Z11Z14Z17Z)20:5(5Z8Z11Z14Z17Z)) ; PE(22:6(4Z7Z10Z13Z16Z19Z)18:4(6Z9Z12Z15Z)) ; 9-Carboxy-alpha-chromanol ; Eremopetasinorone A ; Benzyl hexanoate ; (E)-6-Methyl-6-(5-methyl-2-furanyl)-3-hepten-2-one ; 2-Phenylpropyl butyrate ; 2-Phenylpropyl isobutyrate ; 3-Phenylpropyl 2-methylpropanoate ; 3-Acetoxy-3-methyl-1-phenylbutane ; 3-Methylbutyl phenylacetate ; 2-Phenylethyl pentanoate ; 2-Phenylethyl 3-methylbutanoate ; xi-89-Dehydrotheaspirone ; 246-Trimethyl-4-phenyl-13-dioxane ; Methyl 4-tert-butylphenylacetate ; 1-Phenylpropyl butyrate ; alpha-Methylphenethyl butyrate ; Ethyl ()-2-ethyl-3-phenylpropanoate ; 10beta-1213-Dinor-8-oxo-6-eremophilen-11-al ; 1-Methyl-1-phenylethyl isobutyrate ; 2-Phenylethyl 2-	M+H ; M+Na ; M+Na ; M+Na ; M+Na ; 2M+H ; M+ACN+2H ; M+ACN+2H ; M+ACN+2H ; M+Na ; 2M+H ; M+Na	C26H36O4 ; C24H38O4 ; C24H38O4 ; C24H38O4 ; C24H38O4 ; C13H18O2 ; C45H70NO8P ; C45H70NO8P ; C45H70NO8P ; C24H38O4 ; C13H18O2 ; C24H38O4
X316	6	466.009	0	HMDB01201 ; HMDB59648	Guanosine diphosphate ; 8-oxo-dGDP	M+Na ; M+Na	C10H15N5O11P2 ; C10H15N5O11P2
X343	6	575.768	0	Unknow	Unknow	Unknow	Unknow
X361	6	731.276	0	HMDB02278 ; HMDB02282 ; HMDB12492 ; HMDB29319 ; HMDB30716 ; HMDB30735 ; HMDB34025 ; HMDB34048 ; HMDB34264 ; HMDB34414 ; HMDB34838 ; HMDB35002 ; HMDB35371 ; HMDB35428 ; HMDB37479 ; HMDB38756 ; HMDB39067	2-(acetylamino)-15-anhydro-2-deoxy-3-O-b-D-galactopyranosyl-D-arabino-Hex-1-enitol ; 2-(acetylamino)-15-anhydro-2-deoxy-4-O-b-D-galactopyranosyl-D-arabino-Hex-1-enitol ; 1-(12345-Pentahydroxypent-1-yl)-1234-tetrahydro-beta-carboline-3-carboxylate ; 1-Methoxyphaseollidin ; 23-Dihydro-7-methoxy-2-(3-methoxy-45-methylenedioxyphenyl)-3-methyl-5-(1-propenyl)benzofuran ; Aurantiumal ; Cristacarpin ; Isoxanthohumol ; Glyceollin IV ; 3-Hydroxy-4-methoxyglabridin ; Lansiumarin C ; Xanthogalenol ; Licoagrochalcone D ; Licoagrochalcone C ; Xanthohumol ; Gancaonin I ; Epoxybergamottin	2M+H ; 2M+H ; 2M+H ; 2M+Na ; 2M+Na ; 2M+Na ; 2M+Na ; 2M+Na ; 2M+Na ; 2M+Na ; 2M+Na ; 2M+Na ; 2M+Na ; 2M+Na ; 2M+Na ; 2M+Na ; 2M+Na	C14H23NO10 ; C14H23NO10 ; C17H21N2O7 ; C21H22O5 ; C21H22O5 ; C21H22O5 ; C21H22O5 ; C21H22O5 ; C21H22O5 ; C21H22O5 ; C21H22O5 ; C21H22O5 ; C21H22O5 ; C21H22O5 ; C21H22O5 ; C21H22O5 ; C21H22O5
X4	6	102.034	0	Unknow	Unknow	Unknow	Unknow

X52	6	186.051	0	HMDB14553 ; HMDB39774 ; HMDB41733 ; HMDB41734 ; HMDB41747 ; HMDB41749	Remoxipride ; 5-Hydroxy-6-methoxycoumarin 7-glucoside ; Ferulic acid 4-O-glucuronide ; Feruloyl C1-glucuronide ; Isoferulic acid 3-O-glucuronide ; Isoferuloyl C1-glucuronide	M+2H ; M+2H ; M+2H ; M+2H ; M+2H ; M+2H	C16H23BrN2O3 ; C16H18O10 ; C16H18O10 ; C16H18O10 ; C16H18O10 ; C16H18O10
X168	7	381.801	0	HMDB07878 ; HMDB07970 ; HMDB08034 ; HMDB08262 ; HMDB08907 ; HMDB09483	PC(14:020:0) ; PC(16:018:0) ; PC(18:016:0) ; PC(20:014:0) ; PE(15:022:0) ; PE(22:015:0)	M+2H ; M+2H ; M+2H ; M+2H ; M+2H ; M+2H	C42H84NO8P ; C42H84NO8P ; C42H84NO8P ; C42H84NO8P ; C42H84NO8P ; C42H84NO8P
X15	8	135.03	0	HMDB38974 ; HMDB39789 ; HMDB39790 ; HMDB00362 ; HMDB00807 ; HMDB01228 ; HMDB03391 ; HMDB06039 ; HMDB60180	33-Dimethyl-12-dithiolane ; Tetrahydro-2-methyl-2-thiophenethiol ; Tetrahydro-2-methyl-3-thiophenethiol ; 2-Phosphoglyceric acid ; 3-Phosphoglyceric acid ; L-Glutamic acid 5-phosphate ; 2-Phospho-D-glyceric acid ; 8-Chloroxanthine ; (2R)-2-Hydroxy-3-(phosphonatooxy)propanoate	M+H ; M+H ; M+H ; M+2ACN+2H ; M+2ACN+2H ; M+ACN+2H ; M+2ACN+2H ; M+2ACN+2H ; M+2ACN+2H	C5H10S2 ; C5H10S2 ; C5H10S2 ; C3H7O7P ; C3H7O7P ; C5H10NO7P ; C3H7O7P ; C5H3ClN4O2 ; C3H7O7P
X252	8	188.038	0	Unknow	Unknow	Unknow	Unknow
X286	9	280.007	0	Unknow	Unknow	Unknow	Unknow
X120	10	289.04	0	HMDB29191	5-(34-Dihydroxyphenyl)-gamma-valerolactone sulfate	M+H	C11H12O7S

Table S6a. Genes associated with metabolites in network for strain Gene

Gene			Metabolite			
Node	Name	Community	Node	m/z	RT	Community
X1	*Ahcy*	1	Y10011	367.0622	197.0	1
X4	*Gclc*	1	Y10012	367.0724	176.6	1
X8	*Ifngr2*	1	Y10025	367.4781	279.5	1
			Y10028	367.5605	249.8	1
			Y10069	369.0392	196.8	1
			Y1007	125.5471	132.5	1
			Y10080	369.2632	257.9	1
			Y10100	370.0742	195.2	1
			Y10113	370.5319	129.5	1
			Y10117	370.7329	47.0	1
			Y10125	370.9686	58.1	1
			Y10158	371.9235	100.7	1
			Y10168	372.0612	76.9	1
			Y10170	372.1060	258.7	1
			Y10177	372.2825	197.6	1
			Y10183	372.6016	263.5	1
			Y10185	372.7302	47.0	1
			Y10199	373.0648	76.8	1
			Y10202	373.1036	258.5	1
			Y10226	374.0677	77.0	1
			Y10284	376.6781	45.7	1
			Y10297	377.0926	63.0	1
			Y10315	378.0451	196.6	1
			Y10332	378.5464	196.8	1
			Y10338	378.8124	20.4	1
			Y10372	380.0582	197.2	1
			Y10377	380.1189	87.6	1
			Y10386	380.6724	45.8	1
			Y10420	381.8463	43.7	1
			Y10439	382.3012	42.1	1
			Y10452	382.9816	91.8	1
			Y10459	383.0852	222.3	1
			Y10467	383.2790	274.6	1
			Y10470	383.3155	171.1	1
			Y10488	384.1152	37.5	1
			Y10513	385.0892	60.3	1
			Y10514	385.1185	37.5	1
			Y10529	385.5466	80.2	1
			Y10548	386.0471	169.8	1
			Y10571	386.9425	53.4	1
			Y10583	387.0551	126.1	1
			Y10663	390.0798	41.1	1
			Y10677	390.8812	45.3	1
			Y10687	391.1198	252.4	1
			Y10705	392.0349	89.5	1
			Y10810	396.0551	171.2	1
			Y10841	397.2712	45.3	1
			Y10860	398.0684	197.1	1
			Y10881	399.0739	61.8	1
			Y10956	402.0208	171.6	1
			Y10974	402.6817	45.1	1
			Y10990	403.0329	126.1	1
			Y11019	404.0950	238.4	1
			Y1105	128.5101	34.0	1
			Y11073	406.1457	84.2	1

Y11119	408.0134	84.5	1
Y11122	408.0973	261.4	1
Y1114	129.0142	101.2	1
Y11194	411.0802	214.8	1
Y11218	412.1385	102.5	1
Y11278	414.3575	21.2	1
Y113	91.9624	197.7	1
Y11304	415.3609	21.4	1
Y11305	415.5174	88.9	1
Y11350	417.1415	88.6	1
Y11380	418.7478	23.8	1
Y11398	419.4971	253.9	1
Y11416	420.1630	150.0	1
Y11435	421.0562	60.0	1
Y11459	422.1162	160.4	1
Y11495	424.0647	100.1	1
Y1152	130.0499	68.4	1
Y11575	427.5091	128.7	1
Y11591	428.0986	197.2	1
Y11600	428.6913	46.9	1
Y11613	428.9902	58.0	1
Y11624	429.1017	196.1	1
Y11642	430.0942	199.6	1
Y11643	430.1031	196.5	1
Y11655	430.6821	44.1	1
Y11673	431.0978	196.3	1
Y11674	431.1061	198.1	1
Y11681	431.2489	23.5	1
Y11707	432.0902	197.9	1
Y11719	432.6792	44.1	1
Y11720	432.6857	46.9	1
Y11726	432.8658	52.2	1
Y11756	434.0737	90.4	1
Y11761	434.1916	113.6	1
Y11814	436.6339	46.0	1
Y11856	438.1260	267.2	1
Y11914	441.1109	155.1	1
Y11943	442.7147	47.1	1
Y1199	131.0344	51.6	1
Y11991	444.6655	46.0	1
Y11995	444.7194	47.7	1
Y1200	131.0416	160.1	1
Y12005	444.9604	279.6	1
Y12013	445.1389	57.7	1
Y1202	131.0469	70.5	1
Y12027	446.1052	244.9	1
Y12037	446.6626	45.8	1
Y1204	131.0531	69.0	1
Y12046	446.9966	100.3	1
Y12082	448.6598	45.9	1
Y12086	448.7411	43.5	1
Y12100	449.0749	217.4	1
Y12127	450.6571	46.0	1
Y12129	450.7394	43.5	1
Y12214	455.0902	79.8	1
Y12236	456.5609	89.3	1
Y12242	456.6916	45.9	1
Y12280	458.6887	46.0	1
Y12353	462.1021	136.3	1

	Y12357	462.1945	122.5	1	
	Y12363	462.6368	45.2	1	
	Y12454	466.1062	219.3	1	
	Y12476	467.0900	208.4	1	
	Y12486	468.0935	216.3	1	
	Y12499	469.0007	129.2	1	
	Y12544	471.0773	227.9	1	
	Y12604	474.7504	44.7	1	
	Y12648	476.7475	44.5	1	
	Y12674	478.2355	266.2	1	
	Y12688	478.7603	275.4	1	
	Y12698	479.0477	88.2	1	
	Y12701	479.1112	144.2	1	
	Y12710	479.6505	272.8	1	
	Y12738	480.9568	67.6	1	
	Y12740	480.9835	129.3	1	
	Y12745	481.1660	61.5	1	
	Y12760	482.1982	163.6	1	
	Y12781	483.2022	162.2	1	
	Y12795	484.0966	276.8	1	
	Y12822	485.0704	68.7	1	
	Y12841	486.1123	218.1	1	
	Y12856	487.0898	92.4	1	
	Y12952	493.0062	97.5	1	
	Y1306	133.5612	61.4	1	
	Y13063	498.6756	47.0	1	
	Y13095	500.1204	74.2	1	
	Y13186	505.0857	169.3	1	
	Y1320	134.0448	71.8	1	
	Y13236	508.0003	59.2	1	
	Y13328	512.7707	286.9	1	
	Y13330	512.9032	143.1	1	
	Y13523	524.6743	44.4	1	
	Y1353	135.0425	59.9	1	
	Y13537	525.1445	159.5	1	
	Y13567	526.5399	45.7	1	
	Y1357	135.0482	69.8	1	
	Y1361	135.0634	190.5	1	
	Y1364	135.0804	21.9	1	
	Y13669	530.7692	47.9	1	
	Y13743	534.1288	101.3	1	
	Y1376	135.5062	102.2	1	
	Y13834	539.1313	211.8	1	
	Y13964	546.0706	61.0	1	
	Y13975	546.5109	44.9	1	
	Y13983	546.7784	290.3	1	
	Y13990	547.1285	176.3	1	
	Y14011	548.4584	289.8	1	
	Y14023	548.7925	289.6	1	
	Y14027	548.9588	289.4	1	
	Y14032	549.1271	289.8	1	
	Y14069	550.6809	44.5	1	
	Y1408	136.4964	34.3	1	
	Y14116	553.0854	88.9	1	
	Y14189	557.1222	87.2	1	
	Y14190	557.1415	213.0	1	
	Y14203	558.1268	87.6	1	
	Y14225	559.1291	87.9	1	
	Y14241	560.1211	88.5	1	

Y14260	561.3148	35.5	1
Y14272	562.3270	39.1	1
Y14277	562.5762	45.9	1
Y14291	563.3304	39.4	1
Y14330	565.9405	110.6	1
Y14380	568.1766	165.0	1
Y14511	575.5012	293.9	1
Y1454	137.9643	16.5	1
Y14572	578.2521	110.6	1
Y14591	579.0758	131.3	1
Y1471	138.0550	63.1	1
Y1474	138.0755	44.2	1
Y14882	596.0397	269.9	1
Y14917	597.9366	99.7	1
Y14982	601.0447	128.9	1
Y1501	139.0390	216.5	1
Y15015	603.0292	100.0	1
Y15094	606.1793	117.0	1
Y15135	608.2291	43.8	1
Y1514	139.1117	22.8	1
Y15293	613.6363	279.9	1
Y15333	615.3263	285.8	1
Y15338	615.5248	286.6	1
Y15352	616.1578	210.6	1
Y15377	617.1067	186.8	1
Y15383	617.3169	152.5	1
Y15399	618.1096	261.2	1
Y15402	618.2569	42.9	1
Y155	94.5152	157.7	1
Y15597	627.1216	61.7	1
Y15598	627.1996	280.5	1
Y15642	630.0825	272.6	1
Y15643	630.0867	274.4	1
Y15847	642.4707	284.6	1
Y15956	649.6858	176.6	1
Y15960	650.0192	179.1	1
Y16032	654.1060	188.5	1
Y16034	654.1858	160.9	1
Y1608	141.5635	141.5	1
Y16088	657.9460	290.4	1
Y16089	658.1476	289.8	1
Y16098	658.5535	289.8	1
Y1617	142.0264	98.8	1
Y16179	663.0005	254.3	1
Y16325	673.6282	269.4	1
Y16344	675.1512	100.9	1
Y16352	676.0726	99.6	1
Y1653	143.0234	100.4	1
Y1654	143.0295	100.8	1
Y16553	689.0470	293.9	1
Y16598	692.0472	186.0	1
Y16612	692.7832	290.2	1
Y16788	705.0216	277.8	1
Y16791	705.1184	63.4	1
Y16827	706.5379	17.9	1
Y16849	707.5411	20.5	1
Y16896	710.4535	47.3	1
Y1693	144.0114	198.9	1
Y1697	144.0307	100.1	1

Y17039	718.1393	196.4	1
Y17124	726.2259	281.3	1
Y17142	727.1224	169.2	1
Y17153	728.3369	72.4	1
Y17302	743.2057	246.4	1
Y17371	750.1244	253.2	1
Y17413	754.5426	19.5	1
Y1743	145.0438	236.1	1
Y1750	145.0688	242.8	1
Y17524	764.5501	22.4	1
Y176	96.0444	31.1	1
Y17608	772.5299	22.2	1
Y17698	782.8704	57.1	1
Y1782	146.0367	198.1	1
Y1794	146.1176	39.2	1
Y18063	828.5750	291.9	1
Y1814	147.0257	197.0	1
Y18166	848.3997	112.4	1
Y18323	957.0357	277.7	1
Y18326	957.3615	277.2	1
Y18337	971.2613	278.5	1
Y18344	971.9238	278.7	1
Y1836	147.1210	39.7	1
Y18380	1090.9195	274.4	1
Y18387	1093.0405	278.6	1
Y18388	1093.1715	278.0	1
Y1845	147.5226	196.6	1
Y1862	148.0604	67.7	1
Y188	97.0478	31.0	1
Y1900	149.0263	54.0	1
Y1909	149.0635	68.1	1
Y2029	152.0566	179.6	1
Y2061	153.0541	180.4	1
Y2063	153.0600	169.4	1
Y2067	153.0772	64.7	1
Y2078	153.5414	197.6	1
Y2098	154.0377	38.3	1
Y2101	154.0441	38.3	1
Y2133	155.0025	194.8	1
Y2134	155.0112	78.2	1
Y215	98.5121	34.5	1
Y2166	156.0422	54.7	1
Y2167	156.0506	125.7	1
Y2197	157.0096	34.6	1
Y2202	157.0392	54.7	1
Y2203	157.0455	53.4	1
Y221	98.9755	95.5	1
Y2225	157.5187	102.2	1
Y2238	158.0402	54.6	1
Y2260	158.5163	101.2	1
Y2269	158.9615	16.7	1
Y2287	159.0436	54.5	1
Y2327	160.0427	41.7	1
Y2334	160.0842	119.6	1
Y2346	160.5222	198.5	1
Y2352	160.9505	93.8	1
Y2360	160.9998	99.6	1
Y2386	161.1285	83.6	1
Y2425	162.0762	60.0	1

Y2432	162.1318	82.4	1
Y2453	163.0155	192.0	1
Y2468	163.0753	16.4	1
Y2498	164.0083	93.5	1
Y2529	164.5662	141.4	1
Y2661	168.0653	283.9	1
Y2702	169.1067	181.7	1
Y2709	169.5274	197.0	1
Y2815	172.0717	83.4	1
Y2840	172.9523	262.1	1
Y2916	174.9006	268.6	1
Y2955	175.1440	88.0	1
Y2971	176.0051	36.9	1
Y2997	176.1281	193.9	1
Y3013	176.9998	36.8	1
Y3018	177.0409	30.0	1
Y3065	178.0327	113.1	1
Y3068	178.0444	33.0	1
Y3080	178.0983	265.1	1
Y3102	178.9906	275.2	1
Y3112	179.0295	104.8	1
Y3201	181.0443	198.2	1
Y3263	182.9814	76.6	1
Y3270	183.0369	197.6	1
Y3277	183.0782	45.7	1
Y329	102.9483	16.4	1
Y3293	183.5331	203.3	1
Y3294	183.5386	197.1	1
Y3312	184.0816	44.6	1
Y3348	185.1188	275.0	1
Y3377	186.0165	62.0	1
Y3381	186.0526	92.7	1
Y3403	186.5163	187.5	1
Y3426	187.0366	229.0	1
Y3429	187.0536	197.9	1
Y3444	187.1441	244.1	1
Y3469	188.0915	25.9	1
Y3496	189.0410	33.8	1
Y3506	189.0949	26.0	1
Y353	103.5204	263.0	1
Y3578	191.0104	205.1	1
Y3584	191.0391	81.9	1
Y3587	191.0485	196.9	1
Y3611	191.4813	100.0	1
Y3615	191.5406	86.1	1
Y3684	193.1221	22.8	1
Y3693	193.4331	15.7	1
Y3757	195.0299	138.3	1
Y3789	195.9305	100.3	1
Y3800	196.0130	34.2	1
Y3866	197.9259	102.4	1
Y3889	198.1236	75.3	1
Y3909	198.9262	79.4	1
Y3925	199.0713	208.4	1
Y3947	199.6365	261.0	1
Y3949	199.9235	78.3	1
Y3958	200.0442	79.5	1
Y396	105.0447	32.0	1
Y3975	200.4866	100.5	1

Y3976	200.5459	82.2	1
Y4023	202.0407	86.5	1
Y415	105.9780	196.8	1
Y4176	205.0731	144.2	1
Y4187	205.1192	168.9	1
Y421	106.0287	30.8	1
Y4218	206.0400	59.8	1
Y4242	206.5071	100.6	1
Y4261	207.0058	102.2	1
Y4266	207.0289	116.7	1
Y429	106.0685	65.2	1
Y4312	207.9891	75.7	1
Y4328	208.0970	169.1	1
Y4454	211.5523	90.0	1
Y4467	212.0537	89.3	1
Y4475	212.1610	129.4	1
Y4539	214.0063	59.5	1
Y4563	214.5528	198.3	1
Y4584	215.0331	168.2	1
Y4588	215.0543	196.3	1
Y4613	215.5507	196.1	1
Y4614	215.5548	197.9	1
Y462	108.0114	52.2	1
Y4629	216.0323	197.9	1
Y4633	216.0523	196.8	1
Y4634	216.0567	197.8	1
Y4653	216.5506	197.7	1
Y4692	217.5404	89.5	1
Y4694	217.5977	145.4	1
Y4707	218.0421	89.6	1
Y471	108.5009	98.8	1
Y4723	218.3752	228.6	1
Y482	109.0635	45.1	1
Y4825	220.1497	272.0	1
Y4851	221.0590	154.9	1
Y49	88.0394	70.5	1
Y4964	223.5643	155.8	1
Y4969	223.9644	56.0	1
Y4997	224.1281	180.0	1
Y5007	224.6055	121.8	1
Y5029	225.0472	54.4	1
Y5068	225.9797	31.1	1
Y513	111.0036	101.6	1
Y5159	228.0219	58.9	1
Y5164	228.0801	61.3	1
Y5169	228.1018	261.0	1
Y5178	228.2320	21.3	1
Y5186	228.8128	46.7	1
Y5188	228.9066	42.8	1
Y5191	228.9525	40.8	1
Y5219	229.5897	154.2	1
Y5234	230.0788	78.6	1
Y5265	230.9588	34.5	1
Y527	111.0684	89.9	1
Y5272	231.0092	170.3	1
Y5274	231.0250	106.0	1
Y5276	231.0432	219.6	1
Y5297	231.3824	159.1	1
Y5301	231.6008	123.6	1

Y5303	231.7170	160.2	1
Y5308	231.9838	43.3	1
Y5315	232.0477	162.7	1
Y5324	232.1025	124.0	1
Y5385	233.5263	217.2	1
Y5404	234.0281	195.1	1
Y5429	234.5241	197.5	1
Y5437	234.9078	262.6	1
Y5444	234.9853	127.2	1
Y5446	235.0040	129.5	1
Y5455	235.0456	156.5	1
Y5466	235.1310	184.7	1
Y5482	235.5584	88.9	1
Y5487	235.8803	246.8	1
Y5503	236.0239	201.7	1
Y5545	236.9570	103.0	1
Y5549	236.9771	40.2	1
Y5550	236.9828	128.9	1
Y5565	237.1169	23.1	1
Y5596	238.0511	217.5	1
Y5620	238.8416	46.9	1
Y5629	238.9525	104.5	1
Y5643	239.1029	185.1	1
Y5651	239.1638	22.0	1
Y5698	240.8388	46.9	1
Y5714	241.0489	126.6	1
Y574	112.8958	44.3	1
Y5783	243.0155	45.5	1
Y5787	243.0432	208.6	1
Y5813	243.5597	219.2	1
Y5820	243.9441	57.1	1
Y5822	243.9902	30.9	1
Y5823	243.9960	146.5	1
Y5833	244.0485	92.1	1
Y5840	244.0946	81.4	1
Y5857	244.5501	93.0	1
Y5860	244.7868	45.6	1
Y5866	245.0074	241.3	1
Y5871	245.0400	203.7	1
Y5937	246.7839	45.3	1
Y5950	247.1005	232.7	1
Y5991	248.0876	99.9	1
Y6	85.0415	68.2	1
Y6070	250.4831	100.2	1
Y6073	250.4983	168.5	1
Y6130	252.0162	240.2	1
Y6139	252.0778	55.1	1
Y6141	252.1080	90.5	1
Y6151	252.4998	200.4	1
Y6173	253.0810	60.0	1
Y6187	253.5130	100.6	1
Y6208	254.0737	58.6	1
Y6225	254.8156	45.4	1
Y6240	255.0649	115.5	1
Y6257	255.5306	261.9	1
Y6279	256.0968	165.8	1
Y6298	256.8130	45.4	1
Y6303	256.9046	270.2	1
Y635	114.8930	44.2	1

Y6383	258.8104	45.5	1
Y6385	258.8992	53.9	1
Y6392	258.9899	44.4	1
Y6420	259.6493	28.1	1
Y6446	260.4810	104.2	1
Y6496	261.5557	212.5	1
Y6529	262.1393	122.3	1
Y6546	263.0149	209.5	1
Y6554	263.0753	156.3	1
Y6556	263.0873	74.3	1
Y6608	264.8443	45.3	1
Y6616	265.0229	234.3	1
Y6630	265.1401	71.2	1
Y6645	265.5689	89.3	1
Y6676	266.5617	209.2	1
Y6694	267.0669	101.5	1
Y6714	267.5556	214.5	1
Y6715	267.5685	101.8	1
Y6755	268.8398	45.5	1
Y6800	270.0497	61.6	1
Y6827	270.5608	217.8	1
Y6850	271.0672	211.0	1
Y6851	271.0678	105.1	1
Y6882	271.9852	34.3	1
Y6938	273.1201	73.2	1
Y6971	274.0597	59.7	1
Y6975	274.0854	66.3	1
Y6978	274.1032	210.4	1
Y7014	275.0165	206.4	1
Y7050	276.0118	127.8	1
Y7059	276.0845	83.2	1
Y706	116.8909	44.1	1
Y7090	277.0673	215.6	1
Y7123	277.9979	100.4	1
Y7132	278.0706	271.9	1
Y7165	279.0646	86.7	1
Y7166	279.0740	209.4	1
Y7183	279.5663	88.0	1
Y7185	279.5758	209.3	1
Y720	117.0377	69.9	1
Y7205	280.0688	87.7	1
Y7206	280.0717	215.1	1
Y7207	280.0763	220.2	1
Y7223	280.5642	87.6	1
Y7224	280.5687	87.6	1
Y7248	281.0706	88.0	1
Y7255	281.1562	26.8	1
Y7263	281.5724	88.0	1
Y7272	281.9936	58.6	1
Y7306	282.8160	43.7	1
Y7319	283.0455	99.8	1
Y735	117.5062	99.8	1
Y7399	285.0911	128.9	1
Y7403	285.1207	242.1	1
Y7435	286.0697	102.1	1
Y7440	286.1248	242.9	1
Y7445	286.1510	92.0	1
Y7465	286.9839	130.1	1
Y7470	287.0526	86.0	1

Y7473	287.0763	118.8	1
Y7498	287.9804	168.0	1
Y7503	288.0650	206.5	1
Y7505	288.0696	266.3	1
Y7511	288.1012	86.9	1
Y7565	289.5514	100.2	1
Y7566	289.5648	219.4	1
Y7584	290.0336	60.5	1
Y7590	290.0806	159.6	1
Y7595	290.0983	107.4	1
Y7596	290.1347	64.3	1
Y7627	290.9065	44.2	1
Y764	118.1023	54.1	1
Y7645	291.1026	98.6	1
Y7649	291.1381	64.4	1
Y7663	291.9735	168.1	1
Y7666	291.9895	151.6	1
Y7708	293.0917	83.4	1
Y7728	293.9072	76.7	1
Y7736	294.0950	83.3	1
Y7777	295.9231	100.7	1
Y7839	297.9673	58.0	1
Y7851	298.0966	28.5	1
Y7864	298.5668	89.5	1
Y7880	299.0820	108.2	1
Y79	90.0436	70.5	1
Y790	119.0615	28.8	1
Y7901	299.5840	109.6	1
Y7917	300.0546	198.1	1
Y7919	300.0594	196.4	1
Y7928	300.1554	55.6	1
Y796	119.0833	55.0	1
Y798	119.0896	55.8	1
Y7994	302.0635	155.4	1
Y8041	303.5463	241.1	1
Y8054	304.0792	167.5	1
Y8073	304.7426	46.7	1
Y8087	305.0984	281.1	1
Y8088	305.1092	111.1	1
Y8109	305.9530	169.8	1
Y8122	306.0776	208.0	1
Y8193	308.0909	197.2	1
Y8248	310.0214	189.1	1
Y8256	310.0949	195.9	1
Y8328	312.1164	63.6	1
Y834	120.5106	99.9	1
Y8361	313.0293	205.6	1
Y84	90.4903	100.2	1
Y841	120.9809	265.1	1
Y8414	314.2766	22.1	1
Y8424	314.7714	46.8	1
Y8441	315.0803	246.8	1
Y8474	316.0418	212.9	1
Y8477	316.0769	83.0	1
Y8561	318.8603	160.1	1
Y8594	319.5903	95.9	1
Y8640	321.5638	203.6	1
Y8645	321.9970	101.9	1
Y8654	322.1067	61.5	1

Y8707	324.0222	98.1	1
Y8740	325.0622	241.4	1
Y8742	325.0924	111.1	1
Y8791	326.9176	89.0	1
Y8797	326.9944	63.8	1
Y8803	327.0591	268.9	1
Y8820	327.5609	258.5	1
Y8821	327.5964	158.4	1
Y8844	328.0984	158.9	1
Y8846	328.1253	140.6	1
Y8859	328.5943	165.3	1
Y8863	328.7483	45.3	1
Y8872	328.9581	165.3	1
Y8881	329.0382	37.5	1
Y8886	329.0951	166.5	1
Y8904	329.5482	109.7	1
Y8909	329.9944	61.1	1
Y8916	330.0592	75.4	1
Y8929	330.6873	76.3	1
Y9021	333.0909	153.7	1
Y9042	333.5787	280.5	1
Y9063	334.1594	36.6	1
Y907	122.9135	95.3	1
Y9071	334.5187	273.5	1
Y9075	334.5863	138.5	1
Y908	122.9246	46.3	1
Y9115	335.5939	220.0	1
Y9189	338.0472	196.1	1
Y9213	339.0379	221.0	1
Y9214	339.0552	218.3	1
Y9215	339.0554	92.3	1
Y922	123.0553	33.4	1
Y9239	340.0522	198.0	1
Y9240	340.0585	205.3	1
Y9290	341.8632	44.2	1
Y930	123.1168	22.2	1
Y9312	342.6017	242.5	1
Y9324	342.9961	284.8	1
Y9333	343.1421	281.4	1
Y9348	343.3116	195.4	1
Y935	123.5219	197.1	1
Y9419	345.5429	96.5	1
Y9431	346.0317	266.1	1
Y9437	346.0814	182.2	1
Y9448	346.5300	182.0	1
Y9449	346.5700	158.9	1
Y9468	347.0716	160.3	1
Y9537	350.0748	120.6	1
Y9543	350.1560	119.0	1
Y957	124.0523	31.2	1
Y9583	351.5626	273.3	1
Y959	124.0585	33.4	1
Y9595	352.0014	285.9	1
Y9600	352.0637	184.8	1
Y9624	353.0129	42.1	1
Y9629	353.0664	196.9	1
Y9663	354.3364	190.9	1
Y9679	355.0377	271.6	1
Y9685	355.0741	211.9	1

			Y9694	355.5396	272.9	1
			Y9707	356.0579	196.9	1
			Y9709	356.0772	209.1	1
			Y9762	358.0988	221.1	1
			Y9779	358.6010	215.6	1
			Y9795	359.1668	20.0	1
			Y9820	360.1184	242.9	1
			Y9834	360.5461	128.5	1
			Y9859	361.3220	175.0	1
			Y9884	362.5988	86.8	1
			Y990	125.0594	30.6	1
			Y991	125.0618	30.6	1
			Y9932	364.6984	46.8	1
			Y9946	365.0675	183.7	1
			Y9957	365.5035	180.3	1
			Y9971	366.0008	73.8	1
			Y9976	366.0699	180.4	1
			Y9980	366.1396	77.5	1
X10	*Jun*	2	Y10	85.0779	92.1	2
X12	*Rela*	2	Y10020	367.2675	19.7	2
X7	*Icam1*	2	Y10053	368.2793	22.1	2
			Y10101	370.0988	254.2	2
			Y10107	370.2950	21.9	2
			Y10146	371.2982	21.8	2
			Y1019	126.0186	25.3	2
			Y10340	378.8991	57.2	2
			Y10494	384.3105	21.8	2
			Y1055	127.0123	24.8	2
			Y10747	393.7804	50.1	2
			Y10801	395.7779	50.2	2
			Y10815	396.3104	21.8	2
			Y1084	128.0157	25.6	2
			Y10907	400.0291	53.4	2
			Y1098	128.0620	24.3	2
			Y11058	405.5647	260.9	2
			Y11138	408.8860	50.5	2
			Y11222	412.3209	21.4	2
			Y1123	129.0699	24.2	2
			Y1134	129.5067	84.1	2
			Y1140	129.5945	70.3	2
			Y11433	421.0190	54.8	2
			Y11454	422.0108	54.9	2
			Y11472	422.8628	50.6	2
			Y11802	435.9890	52.7	2
			Y11808	436.3053	22.6	2
			Y11823	436.9931	52.8	2
			Y11834	437.3087	22.5	2
			Y11847	437.9855	52.7	2
			Y11862	438.3211	22.2	2
			Y11888	439.3011	24.4	2
			Y12031	446.3261	21.6	2
			Y12058	447.3295	21.7	2
			Y12078	448.3418	17.2	2
			Y12123	450.3574	21.5	2
			Y12141	451.3609	21.6	2
			Y12197	454.2926	24.3	2
			Y12218	455.2961	25.1	2
			Y12361	462.2975	25.1	2
			Y12414	464.8478	50.3	2

Y12460	466.3524	21.9	2
Y12470	466.8448	50.4	2
Y12516	469.9347	56.0	2
Y12563	472.3417	21.4	2
Y12581	473.3453	21.6	2
Y12590	473.9450	52.7	2
Y12598	474.3576	21.5	2
Y12624	475.3611	21.6	2
Y12632	475.9419	52.7	2
Y12667	477.7501	49.9	2
Y12671	477.9384	52.1	2
Y12724	480.3084	23.8	2
Y12735	480.8214	50.5	2
Y12775	482.8185	50.6	2
Y12895	489.9306	58.7	2
Y12962	493.7241	49.4	2
Y1302	133.4988	62.0	2
Y13131	502.0282	271.6	2
Y13303	511.6940	49.8	2
Y13350	513.8980	52.6	2
Y13352	514.1914	24.3	2
Y13769	535.5280	275.3	2
Y1386	135.9763	56.2	2
Y14041	549.4871	23.2	2
Y1407	136.4914	157.9	2
Y14127	553.6805	51.4	2
Y1422	137.0153	25.0	2
Y14273	562.3496	27.1	2
Y14283	563.0001	53.0	2
Y14424	570.3556	25.1	2
Y14556	577.4807	20.6	2
Y1460	138.0105	25.2	2
Y1464	138.0275	88.1	2
Y14687	585.3041	281.9	2
Y14930	598.7360	50.8	2
Y1494	139.0123	25.3	2
Y14980	600.9555	52.7	2
Y1500	139.0309	26.6	2
Y15047	604.7459	278.4	2
Y1529	140.0076	25.9	2
Y15368	616.7841	54.1	2
Y15520	623.3180	291.8	2
Y15619	628.5340	47.8	2
Y15672	631.9737	58.4	2
Y15743	636.9169	52.6	2
Y15778	638.9112	52.7	2
Y1582	141.0102	24.5	2
Y15946	649.0500	280.7	2
Y1616	142.0136	25.8	2
Y16275	669.4528	52.7	2
Y16369	676.8662	53.3	2
Y16435	681.4428	53.0	2
Y16508	685.1895	284.2	2
Y16527	687.0199	52.8	2
Y16552	688.9693	293.2	2
Y1666	143.0855	23.0	2
Y16726	701.5600	22.9	2
Y16743	702.5577	35.1	2
Y17019	716.5177	23.3	2

Y17080	721.4687	282.6	2
Y17106	723.9729	52.7	2
Y1712	144.0890	25.4	2
Y1713	144.0934	24.5	2
Y17140	726.8895	54.6	2
Y17167	729.8613	56.9	2
Y17378	750.9384	52.5	2
Y17455	758.5767	285.1	2
Y17575	769.9143	52.5	2
Y17765	790.4084	286.1	2
Y17878	801.8816	52.7	2
Y17904	806.5552	23.3	2
Y17937	812.5363	28.3	2
Y17957	814.5574	24.2	2
Y18129	837.6206	23.7	2
Y1816	147.0441	45.2	2
Y18271	886.9429	52.6	2
Y1852	148.0040	55.6	2
Y1890	149.0009	55.3	2
Y1891	149.0033	54.6	2
Y1984	151.0615	244.0	2
Y2023	152.0262	25.4	2
Y2030	152.0623	31.7	2
Y2066	153.0699	26.0	2
Y2109	154.0777	25.7	2
Y2146	155.0854	21.7	2
Y2170	156.0808	26.7	2
Y226	98.9984	123.4	2
Y227	98.9996	23.9	2
Y23	86.0601	63.2	2
Y2456	163.0309	24.9	2
Y2504	164.0342	24.6	2
Y2535	165.0102	27.5	2
Y2538	165.0280	24.9	2
Y254	100.0030	25.3	2
Y2542	165.0466	25.5	2
Y2575	166.0135	25.1	2
Y2579	166.0315	25.7	2
Y2583	166.0499	26.3	2
Y2608	167.0073	24.8	2
Y2617	167.0436	24.7	2
Y2651	168.0106	24.8	2
Y2665	168.0807	26.9	2
Y2688	169.0268	57.2	2
Y2759	170.9891	55.9	2
Y2819	172.0883	24.6	2
Y282	100.9967	24.7	2
Y2950	175.1116	21.0	2
Y3120	179.0626	25.3	2
Y3167	180.0653	76.7	2
Y3169	180.0655	25.8	2
Y3204	181.0595	25.6	2
Y3238	182.0626	24.6	2
Y3334	185.0006	61.0	2
Y3371	185.9597	52.6	2
Y3456	187.9579	52.8	2
Y3497	189.0466	29.4	2
Y3535	190.0499	29.3	2
Y3585	191.0439	27.6	2

Y3589	191.0622	48.2	2
Y3629	192.0469	25.6	2
Y3688	193.1585	22.8	2
Y3837	197.0410	66.9	2
Y3964	200.1017	27.0	2
Y3967	200.1280	32.6	2
Y4035	202.1439	97.8	2
Y4125	204.0783	37.0	2
Y4310	207.9805	37.3	2
Y4355	209.0541	25.7	2
Y4357	209.0635	24.6	2
Y4390	210.0581	33.8	2
Y4416	210.9407	41.7	2
Y4811	220.0888	25.7	2
Y4812	220.0928	76.2	2
Y4855	221.0925	30.7	2
Y4856	221.0942	263.1	2
Y4857	221.0957	25.3	2
Y4862	221.1204	25.2	2
Y4864	221.1495	100.6	2
Y4898	222.0858	24.8	2
Y4899	222.0955	25.2	2
Y4943	223.0890	24.2	2
Y4984	224.0738	30.0	2
Y5039	225.0908	22.4	2
Y5040	225.0909	169.2	2
Y5255	230.5753	94.3	2
Y5588	237.9731	56.7	2
Y5601	238.0991	21.7	2
Y5605	238.1200	22.5	2
Y5641	239.0913	36.3	2
Y5644	239.1044	18.9	2
Y5652	239.2004	17.9	2
Y5679	240.0995	111.4	2
Y5721	241.1019	43.8	2
Y5748	242.0789	40.9	2
Y5771	242.9253	57.0	2
Y5886	245.1497	43.0	2
Y5913	246.0631	260.1	2
Y5914	246.0649	57.0	2
Y6028	249.1074	55.6	2
Y6210	254.0941	25.6	2
Y6243	255.0975	43.6	2
Y6277	256.0911	25.2	2
Y628	114.1277	29.8	2
Y6324	257.0945	25.8	2
Y6444	260.1855	26.8	2
Y647	115.0466	267.3	2
Y6485	261.1887	27.0	2
Y6700	267.1162	28.4	2
Y6768	269.0550	61.6	2
Y68	89.0386	29.2	2
Y7027	275.1714	73.0	2
Y7067	276.1666	100.8	2
Y7158	278.8935	15.5	2
Y7367	284.1852	25.6	2
Y7437	286.0956	132.0	2
Y7448	286.2010	25.1	2
Y7517	288.2167	24.5	2

			Y7560	289.2200	24.6	2
			Y7612	290.6093	25.3	2
			Y78	90.0420	25.0	2
			Y80	90.0464	27.6	2
			Y8110	305.9592	56.4	2
			Y8283	310.9742	52.5	2
			Y8314	311.9058	53.3	2
			Y8374	313.2734	22.4	2
			Y8446	315.1337	24.1	2
			Y8519	317.2512	22.7	2
			Y8597	319.8480	50.2	2
			Y8922	330.2273	26.7	2
			Y9029	333.2462	25.9	2
			Y9393	344.9893	263.8	2
			Y9406	345.1056	179.8	2
			Y9445	346.2588	26.5	2
			Y9498	348.9302	53.4	2
			Y9561	350.9279	52.6	2
			Y977	125.0152	26.7	2
X3	*Fosb*	3	Y10044	368.0602	183.5	3
X9	*Il7r*	3	Y10238	374.5039	125.7	3
			Y10438	382.2955	24.9	3
			Y1046	126.9644	16.1	3
			Y1047	126.9675	262.5	3
			Y1052	126.9896	272.2	3
			Y1058	127.0215	44.6	3
			Y10634	388.9285	79.8	3
			Y11178	410.5267	90.5	3
			Y1129	129.1024	85.9	3
			Y1158	130.0863	73.4	3
			Y11816	436.6877	47.9	3
			Y11861	438.2976	22.4	3
			Y12077	448.3348	22.1	3
			Y1213	131.0895	80.0	3
			Y12338	460.9671	117.4	3
			Y12347	461.7760	50.6	3
			Y12402	464.3134	23.4	3
			Y12675	478.2926	24.8	3
			Y12751	481.3481	24.5	3
			Y12765	482.3604	27.9	3
			Y12784	483.3273	24.5	3
			Y12785	483.3637	28.4	3
			Y12939	492.5877	44.3	3
			Y1299	133.1012	217.2	3
			Y13014	496.3393	18.6	3
			Y13038	497.3428	19.3	3
			Y13080	499.3482	27.3	3
			Y13083	499.8935	56.7	3
			Y13119	500.9917	168.9	3
			Y13199	506.3596	25.8	3
			Y13239	508.1107	115.3	3
			Y13241	508.3396	26.0	3
			Y13317	512.3344	27.4	3
			Y13318	512.3619	25.9	3
			Y13371	515.3132	27.3	3
			Y13391	516.3167	28.5	3
			Y13496	523.2995	26.5	3
			Y13594	527.3806	26.8	3
			Y13606	527.8146	27.7	3

	Y13620	528.2978	25.0	3
	Y13756	534.8024	253.6	3
	Y13816	538.3864	25.5	3
	Y13844	539.3889	25.8	3
	Y13865	540.3669	28.8	3
	Y1398	136.0617	276.2	3
	Y14037	549.3152	29.5	3
	Y14038	549.3745	23.2	3
	Y14060	550.3878	26.3	3
	Y14085	551.3895	26.6	3
	Y14089	551.6826	50.6	3
	Y14140	554.2859	23.5	3
	Y14458	572.3707	24.4	3
	Y14568	578.0935	100.1	3
	Y14599	579.6671	281.0	3
	Y1475	138.0914	17.7	3
	Y1492	138.9942	89.5	3
	Y14925	598.5295	47.2	3
	Y14943	599.4412	30.4	3
	Y1499	139.0309	125.5	3
	Y1532	140.0133	116.8	3
	Y15477	621.1683	167.8	3
	Y1567	140.9035	47.7	3
	Y1592	141.0708	43.2	3
	Y16200	664.1154	253.4	3
	Y16221	665.1168	253.9	3
	Y16300	671.1225	142.7	3
	Y1635	142.5465	125.4	3
	Y16634	695.0042	52.7	3
	Y16739	702.5011	36.5	3
	Y1706	144.0712	104.6	3
	Y1710	144.0841	24.8	3
	Y17130	726.5070	23.2	3
	Y17164	729.5253	23.6	3
	Y1748	145.0647	25.3	3
	Y18100	833.5747	23.7	3
	Y1897	149.0186	114.1	3
	Y2043	152.5845	103.7	3
	Y2096	154.0229	43.7	3
	Y2171	156.0843	28.6	3
	Y2208	157.0608	59.9	3
	Y2219	157.1084	86.3	3
	Y2232	158.0028	273.6	3
	Y2247	158.0924	86.0	3
	Y2248	158.0998	26.1	3
	Y2297	159.0958	86.6	3
	Y2298	159.1031	26.6	3
	Y2393	161.5188	123.3	3
	Y2417	162.0502	53.4	3
	Y2537	165.0161	100.9	3
	Y2613	167.0261	27.6	3
	Y2654	168.0235	57.1	3
	Y266	100.0474	194.0	3
	Y2739	170.0925	86.6	3
	Y2779	171.0958	87.5	3
	Y2929	174.9978	56.8	3
	Y2980	176.0658	52.2	3
	Y2982	176.0706	247.2	3
	Y2995	176.1224	83.8	3

Y3019	177.0440	58.6	3
Y3023	177.0546	179.7	3
Y3099	178.9743	127.7	3
Y312	102.0461	48.1	3
Y3310	184.0731	22.8	3
Y3396	186.1489	181.8	3
Y3468	188.0821	28.8	3
Y3627	192.0397	45.2	3
Y3661	192.9806	37.6	3
Y3662	193.0019	268.3	3
Y3878	198.0417	101.7	3
Y3972	200.2010	15.9	3
Y3989	201.0537	58.5	3
Y4097	203.4950	124.0	3
Y4167	205.0041	92.3	3
Y4172	205.0446	195.3	3
Y4174	205.0640	28.6	3
Y4194	205.5069	101.0	3
Y4281	207.1029	31.4	3
Y4315	208.0136	42.8	3
Y4335	208.1400	22.8	3
Y4387	210.0118	42.8	3
Y4445	211.1692	20.7	3
Y4481	212.5048	101.4	3
Y4552	214.1438	148.6	3
Y4620	215.9575	119.4	3
Y4725	218.4691	115.2	3
Y4861	221.1104	286.8	3
Y4890	222.0228	263.0	3
Y4902	222.1077	24.4	3
Y4971	223.9886	142.5	3
Y5074	226.0241	45.1	3
Y5271	230.9871	118.1	3
Y5523	236.1726	17.9	3
Y5600	238.0929	27.0	3
Y5615	238.5169	100.5	3
Y5677	240.0902	25.3	3
Y5678	240.0992	24.4	3
Y5706	240.9885	121.5	3
Y5754	242.1029	25.1	3
Y5846	244.1542	29.7	3
Y607	113.0904	86.1	3
Y6075	250.5340	90.3	3
Y6180	253.1476	66.0	3
Y6437	260.0810	52.9	3
Y652	115.0542	227.0	3
Y6558	263.1224	171.7	3
Y6581	263.9991	168.1	3
Y6649	265.9760	126.9	3
Y6668	266.1860	23.0	3
Y6689	266.9686	113.4	3
Y671	115.5787	93.2	3
Y6718	267.6467	26.0	3
Y6742	268.1485	26.3	3
Y6752	268.6499	27.6	3
Y6853	271.0814	87.7	3
Y7094	277.1024	54.0	3
Y7100	277.1536	27.3	3
Y7136	278.1041	23.5	3

			Y7506	288.0723	28.5	3
			Y773	118.9674	265.7	3
			Y7737	294.1269	23.0	3
			Y7881	299.0832	41.9	3
			Y8306	311.4435	168.1	3
			Y8358	313.0171	135.0	3
			Y8544	318.1678	74.2	3
			Y8587	319.2245	47.2	3
			Y8736	325.0430	117.6	3
			Y8973	331.9781	80.3	3
			Y8995	332.5616	253.3	3
			Y90	90.9477	18.8	3
			Y9440	346.1680	28.5	3
			Y9506	349.0346	167.6	3
			Y9552	350.5718	111.3	3
			Y9855	361.2596	23.7	3
X5	*Gpx2*	4	Y10007	366.9996	58.9	4
X6	*Gsta3*	4	Y10065	368.9558	189.0	4
			Y10085	369.5412	196.6	4
			Y10095	370.0211	190.9	4
			Y10120	370.8320	159.8	4
			Y10195	373.0208	90.0	4
			Y10431	382.0930	221.0	4
			Y10457	383.0415	61.9	4
			Y10605	387.9966	119.4	4
			Y10778	394.8742	56.4	4
			Y1078	127.9722	15.6	4
			Y10846	397.5200	196.6	4
			Y10854	397.9337	67.8	4
			Y10877	399.0154	61.8	4
			Y11013	404.0250	284.0	4
			Y11145	409.0845	208.6	4
			Y11163	409.9927	108.9	4
			Y11170	410.0879	221.2	4
			Y11217	412.1132	255.6	4
			Y11268	414.1033	290.9	4
			Y11431	420.9298	42.9	4
			Y11457	422.0880	210.4	4
			Y11479	423.0803	209.5	4
			Y11497	424.1021	77.5	4
			Y11535	426.1112	217.2	4
			Y11557	426.9715	209.6	4
			Y11602	428.7258	275.1	4
			Y11616	429.0395	284.0	4
			Y11638	429.9705	52.8	4
			Y11979	444.0311	293.9	4
			Y120	92.0244	38.1	4
			Y12066	447.8805	56.1	4
			Y12095	448.9609	76.5	4
			Y12111	449.9452	76.1	4
			Y12272	458.0157	83.6	4
			Y12277	458.3471	22.4	4
			Y12310	459.9674	53.7	4
			Y12399	464.0809	114.2	4
			Y12423	465.0849	114.3	4
			Y12447	466.0435	217.9	4
			Y12453	466.0863	114.4	4
			Y12485	468.0709	96.9	4
			Y12506	469.0859	209.8	4

Y12618	475.0951	220.4	4
Y12794	484.0115	168.8	4
Y12857	487.1159	220.1	4
Y12858	487.1258	209.9	4
Y1294	133.0876	93.0	4
Y13261	509.1508	85.8	4
Y13598	527.4986	286.4	4
Y13672	530.8375	43.4	4
Y13792	536.9197	56.0	4
Y1389	136.0068	123.4	4
Y13943	545.0666	191.2	4
Y14058	550.2840	289.7	4
Y14104	552.6191	288.4	4
Y14197	557.8473	55.6	4
Y14803	592.6815	287.2	4
Y14806	592.8113	288.6	4
Y14889	596.4599	52.7	4
Y14994	601.9514	56.2	4
Y15055	604.9076	56.2	4
Y1506	139.0583	58.9	4
Y15107	606.6843	290.2	4
Y15114	606.9421	289.0	4
Y15131	608.0632	288.4	4
Y15133	608.0885	286.4	4
Y15170	609.8591	56.1	4
Y15259	612.5615	290.3	4
Y15305	614.1869	289.8	4
Y1533	140.0165	223.5	4
Y15343	615.8207	289.0	4
Y15375	617.0649	290.9	4
Y15396	617.9505	285.6	4
Y15401	618.2006	285.9	4
Y15411	618.5753	287.9	4
Y15436	619.4448	289.6	4
Y15466	620.8798	55.8	4
Y15480	621.3201	287.8	4
Y15483	621.5696	287.8	4
Y15485	621.8213	287.8	4
Y15488	621.9451	288.2	4
Y15492	622.1966	288.4	4
Y15572	626.1779	290.7	4
Y15582	626.5635	289.7	4
Y15788	639.3396	20.8	4
Y1579	140.9950	206.0	4
Y15848	642.4772	287.0	4
Y15977	650.9000	290.5	4
Y15989	651.4404	290.7	4
Y16118	659.6706	290.6	4
Y16152	661.5482	291.1	4
Y1618	142.0326	222.4	4
Y16203	664.1728	160.7	4
Y16377	677.6372	288.7	4
Y16413	679.7820	289.0	4
Y16476	683.7044	284.6	4
Y16639	695.0975	279.2	4
Y16707	699.9270	290.3	4
Y16754	703.3614	290.4	4
Y16758	703.6491	290.1	4
Y16890	709.9382	288.1	4

Y16892	710.0803	287.7	4
Y16893	710.2226	288.0	4
Y16924	711.9300	291.7	4
Y17004	716.0675	288.5	4
Y17240	736.3687	52.6	4
Y17290	742.2007	249.6	4
Y17292	742.4384	44.7	4
Y17650	777.8811	264.9	4
Y17840	796.8017	288.1	4
Y17842	796.9467	287.8	4
Y17993	820.9212	289.8	4
Y18064	828.5942	288.3	4
Y18105	834.4163	290.0	4
Y18108	834.5766	289.1	4
Y1882	148.5363	90.2	4
Y1918	149.1132	92.0	4
Y1958	150.1141	92.8	4
Y196	97.5144	31.6	4
Y1977	151.0353	118.4	4
Y1978	151.0390	232.6	4
Y213	98.4707	174.6	4
Y2188	156.9460	93.1	4
Y2239	158.0454	40.6	4
Y2281	159.0229	130.0	4
Y2284	159.0282	36.5	4
Y231	99.0137	26.2	4
Y2315	159.9471	94.2	4
Y2406	161.9801	103.6	4
Y2534	165.0009	211.8	4
Y2605	166.9832	15.6	4
Y2668	168.0963	189.0	4
Y2673	168.4776	103.3	4
Y2732	170.0414	72.0	4
Y2786	171.1167	21.8	4
Y2849	173.0212	99.0	4
Y2870	173.4923	208.7	4
Y2887	174.0316	198.0	4
Y2930	175.0017	36.6	4
Y2970	176.0026	188.7	4
Y3091	178.5484	99.4	4
Y3148	179.9537	159.8	4
Y3189	180.9748	130.2	4
Y3347	185.1170	195.1	4
Y3361	185.5141	205.8	4
Y3374	185.9968	106.1	4
Y3386	186.0855	268.0	4
Y3449	187.5106	188.0	4
Y3564	190.9119	49.8	4
Y3624	192.0244	60.9	4
Y3658	192.9573	94.0	4
Y3660	192.9748	119.5	4
Y3667	193.0278	61.4	4
Y3704	193.9298	285.7	4
Y3739	194.5006	191.5	4
Y3876	198.0184	99.2	4
Y3888	198.1222	178.5	4
Y4008	201.5456	98.1	4
Y4015	201.9769	94.8	4
Y4109	203.9908	122.7	4

Y4229	206.0932	178.6	4
Y4258	206.9904	128.3	4
Y4314	207.9983	62.1	4
Y4497	213.0010	125.1	4
Y4595	215.1137	84.0	4
Y4696	217.7068	224.0	4
Y4704	218.0063	126.6	4
Y4722	218.3721	222.6	4
Y4783	219.5371	89.2	4
Y4796	219.9682	146.6	4
Y4801	220.0087	200.2	4
Y4845	221.0095	192.8	4
Y4877	221.5608	157.0	4
Y4909	222.3681	124.7	4
Y4939	223.0637	184.2	4
Y4963	223.5634	100.6	4
Y4978	224.0433	55.4	4
Y5180	228.3906	280.7	4
Y5218	229.5586	100.1	4
Y5224	229.9744	37.0	4
Y5399	234.0013	127.9	4
Y5431	234.5391	106.3	4
Y5435	234.7112	156.8	4
Y5450	235.0207	222.1	4
Y5510	236.0837	200.1	4
Y5585	237.9551	84.5	4
Y5592	238.0193	193.3	4
Y5617	238.5639	96.6	4
Y5640	239.0656	96.2	4
Y5659	239.5664	95.9	4
Y5763	242.5112	184.7	4
Y5766	242.5619	210.9	4
Y5825	244.0133	241.2	4
Y590	113.0346	32.6	4
Y6032	249.1469	168.8	4
Y6160	252.9183	85.6	4
Y6165	252.9967	114.9	4
Y6193	253.9471	55.8	4
Y6197	253.9937	81.3	4
Y6258	255.5531	127.6	4
Y6272	256.0605	95.1	4
Y6284	256.1543	190.8	4
Y6295	256.5633	93.3	4
Y6342	257.5380	96.3	4
Y6404	259.1073	106.3	4
Y6448	260.5619	219.6	4
Y6455	260.8081	45.9	4
Y6732	268.0577	280.8	4
Y6788	269.5672	208.7	4
Y6801	270.0593	222.4	4
Y6804	270.0690	210.3	4
Y6828	270.5651	221.8	4
Y7016	275.0358	261.5	4
Y7057	276.0689	211.5	4
Y7066	276.1553	150.2	4
Y7073	276.5649	221.5	4
Y7074	276.5695	222.2	4
Y7151	278.5822	217.3	4
Y7355	284.0530	153.4	4

			Y7362	284.1026	155.0	4
			Y7493	287.8999	50.1	4
			Y7524	288.5711	271.8	4
			Y7637	291.0477	189.2	4
			Y7638	291.0544	210.4	4
			Y7702	293.0341	56.2	4
			Y7733	294.0223	128.3	4
			Y7776	295.9028	77.5	4
			Y7850	298.0780	208.6	4
			Y7879	299.0719	208.1	4
			Y788	119.0353	37.5	4
			Y7899	299.5681	208.0	4
			Y7900	299.5728	215.3	4
			Y7920	300.0700	222.5	4
			Y8135	306.5260	95.6	4
			Y8157	307.0756	209.8	4
			Y8159	307.0835	207.6	4
			Y817	120.0196	45.0	4
			Y8175	307.2880	211.0	4
			Y8180	307.5824	211.1	4
			Y8229	309.1294	102.3	4
			Y8264	310.1690	165.3	4
			Y8307	311.5180	127.8	4
			Y8357	313.0170	187.8	4
			Y8423	314.6051	280.8	4
			Y8444	315.1150	72.4	4
			Y8643	321.9346	57.3	4
			Y8698	323.9321	56.3	4
			Y872	121.9662	19.1	4
			Y8840	328.0466	98.8	4
			Y8998	332.5895	161.6	4
			Y9019	333.0771	280.2	4
			Y9052	334.0269	276.1	4
			Y9190	338.0764	259.1	4
			Y9221	339.0796	257.9	4
			Y9299	342.1391	280.6	4
			Y93	91.0059	79.3	4
			Y9331	343.0937	254.2	4
			Y9352	343.5739	152.8	4
			Y9558	350.8578	76.2	4
			Y9584	351.5630	155.9	4
			Y9592	351.9680	74.0	4
			Y9654	354.0772	64.3	4
			Y9687	355.1105	63.6	4
			Y9736	357.0823	95.9	4
			Y9807	359.8355	50.0	4
			Y9835	360.5851	237.5	4
			Y9845	361.0600	62.6	4
			Y9942	365.0010	62.3	4
X11	*Junb*	5	Y10136	371.0770	196.8	5
			Y10474	383.3668	22.4	5
			Y11333	416.7049	45.1	5
			Y11420	420.3186	273.6	5
			Y11421	420.3188	17.1	5
			Y15538	624.1863	289.1	5
			Y16630	694.8309	55.6	5
			Y17648	777.5623	22.7	5
			Y3054	177.9481	266.1	5
			Y324	102.5355	261.6	5

			Y7005	274.9331	44.6	5
			Y7277	282.0340	196.0	5
			Y7295	282.4973	241.5	5
			Y844	120.9931	61.0	5
			Y9274	341.1532	21.8	5
			Y946	123.9644	17.3	5
			Y9501	348.9763	69.6	5
X14	*Stat1*	6	Y10084	369.4879	278.8	6
X15	*Tlr2*	6	Y1013	125.9643	16.6	6
X16	*Tnfrsf1b*	6	Y10149	371.3269	25.1	6
X2	*Csrp1*	6	Y1021	126.0220	56.7	6
			Y1024	126.0373	217.8	6
			Y10298	377.1226	89.8	6
			Y10428	382.0570	59.6	6
			Y1048	126.9720	267.2	6
			Y10598	387.7092	73.2	6
			Y10618	388.2109	73.5	6
			Y10649	389.2684	22.3	6
			Y10660	390.0111	61.2	6
			Y10683	391.0315	89.8	6
			Y10690	391.2838	22.5	6
			Y1074	127.5088	91.5	6
			Y10796	395.5513	117.4	6
			Y108	91.5238	262.4	6
			Y10837	397.1642	156.8	6
			Y10968	402.3448	169.5	6
			Y10995	403.2624	22.5	6
			Y11134	408.7626	45.3	6
			Y11164	410.0053	84.9	6
			Y11165	410.0378	63.2	6
			Y11173	410.2239	79.4	6
			Y11249	413.2662	46.6	6
			Y11362	417.8919	46.5	6
			Y11386	418.9323	43.0	6
			Y11389	418.9696	99.3	6
			Y11392	419.1463	27.5	6
			Y11401	419.7665	25.7	6
			Y11502	424.5227	89.3	6
			Y11508	424.7321	67.7	6
			Y11532	426.0238	91.0	6
			Y11627	429.2401	45.9	6
			Y11729	432.9443	86.5	6
			Y11778	434.9358	58.1	6
			Y11797	435.8632	46.6	6
			Y1181	130.9330	152.1	6
			Y11897	440.3131	25.8	6
			Y12023	446.0546	77.7	6
			Y12044	446.9361	57.5	6
			Y12071	448.0519	59.2	6
			Y12102	449.2559	73.4	6
			Y12112	449.9642	89.5	6
			Y1223	131.5169	99.2	6
			Y12247	456.9162	99.8	6
			Y1233	132.0035	280.8	6
			Y1235	132.0186	100.7	6
			Y12384	463.6083	269.8	6
			Y12490	468.3891	22.2	6
			Y12503	469.0672	91.6	6
			Y12504	469.0695	287.7	6

Y12621	475.1542	273.6	6
Y12629	475.8506	46.9	6
Y12669	477.8478	46.7	6
Y12670	477.8806	79.2	6
Y12741	481.0178	265.6	6
Y12801	484.3297	28.8	6
Y12806	484.6938	44.8	6
Y1281	133.0339	234.8	6
Y12839	486.0649	100.1	6
Y1286	133.0591	290.6	6
Y12866	487.9118	92.8	6
Y12871	488.1548	30.2	6
Y12878	488.6936	47.6	6
Y1309	133.9302	100.3	6
Y13198	506.2683	270.1	6
Y13278	510.3914	26.1	6
Y13418	518.3217	24.1	6
Y13509	524.0561	256.7	6
Y13558	526.0465	53.2	6
Y13563	526.2925	279.8	6
Y13596	527.4221	280.8	6
Y13649	529.3283	27.2	6
Y13661	530.2865	23.4	6
Y13692	532.0525	90.2	6
Y13740	534.0492	259.3	6
Y13760	535.0534	98.7	6
Y1390	136.0143	38.4	6
Y13966	546.1056	111.6	6
Y14024	548.8600	277.0	6
Y14043	549.5896	239.5	6
Y14084	551.3121	27.0	6
Y1411	136.5140	91.9	6
Y14124	553.4026	25.5	6
Y14177	556.6116	289.8	6
Y1423	137.0157	91.2	6
Y1428	137.0458	37.4	6
Y146	94.0400	37.2	6
Y1465	138.0298	37.1	6
Y1466	138.0429	37.2	6
Y1468	138.0490	38.7	6
Y14700	586.5351	279.7	6
Y14927	598.6043	43.7	6
Y15163	609.4007	30.2	6
Y1557	140.4862	79.5	6
Y15596	627.0836	99.8	6
Y15751	637.4306	30.2	6
Y15926	647.9470	56.3	6
Y1611	141.9514	88.1	6
Y16122	659.8608	63.5	6
Y16238	666.7391	276.9	6
Y16466	683.2703	295.6	6
Y16571	689.9655	277.7	6
Y16645	695.3393	268.7	6
Y16649	695.5074	52.7	6
Y1680	143.9523	90.0	6
Y16836	706.9675	57.8	6
Y16937	712.3967	254.0	6
Y17151	728.1285	169.3	6
Y17173	730.5392	17.1	6

Y17182	731.1622	280.1	6
Y17190	731.5444	17.1	6
Y17333	746.4888	47.6	6
Y1741	145.0351	279.6	6
Y17607	772.4924	47.5	6
Y17685	780.5389	21.7	6
Y17836	796.5454	29.8	6
Y1797	146.1654	66.5	6
Y17970	817.0432	54.3	6
Y18027	824.4012	44.2	6
Y18184	854.3739	47.9	6
Y18324	957.1430	278.4	6
Y18325	957.2543	277.9	6
Y18327	957.4769	278.3	6
Y18338	971.3751	278.4	6
Y18339	971.4807	278.0	6
Y18340	971.5945	279.6	6
Y18343	971.8221	278.9	6
Y18373	1090.4120	275.3	6
Y18377	1090.7793	275.0	6
Y18378	1090.7986	278.3	6
Y18379	1090.9050	277.0	6
Y18382	1091.1661	275.0	6
Y18385	1092.7932	278.7	6
Y1854	148.0221	92.2	6
Y1879	148.5237	91.5	6
Y1911	149.0767	72.7	6
Y1943	150.0141	279.0	6
Y1962	150.5286	99.3	6
Y1987	151.0696	289.0	6
Y2032	152.0818	227.9	6
Y2059	153.0407	38.1	6
Y2126	154.9669	168.0	6
Y2159	155.9953	186.6	6
Y2307	159.4734	106.0	6
Y2314	159.9470	266.1	6
Y2328	160.0428	186.3	6
Y2329	160.0442	90.7	6
Y2348	160.5270	91.1	6
Y2402	161.9628	99.0	6
Y242	99.4956	99.0	6
Y243	99.5122	259.7	6
Y2437	162.4805	79.8	6
Y2488	163.9401	94.8	6
Y2507	164.0561	61.1	6
Y2566	165.5339	89.1	6
Y2572	165.9828	261.1	6
Y2600	166.9545	16.1	6
Y2712	169.5471	100.0	6
Y2718	169.9825	69.5	6
Y2724	170.0051	100.3	6
Y273	100.5099	256.8	6
Y2767	171.0419	99.5	6
Y2802	171.9904	69.3	6
Y2848	173.0210	178.6	6
Y2851	173.0301	280.5	6
Y2882	173.9961	35.7	6
Y2958	175.1481	160.6	6
Y3003	176.4972	92.0	6

Y3004	176.5091	191.7	6
Y3012	176.9989	90.8	6
Y303	102.0130	282.0	6
Y3056	177.9575	273.7	6
Y308	102.0338	265.2	6
Y3085	178.4631	285.5	6
Y3151	179.9734	97.8	6
Y3184	180.5220	96.7	6
Y336	103.0058	79.7	6
Y3360	185.4959	107.2	6
Y3380	186.0505	77.8	6
Y3387	186.0867	80.0	6
Y3412	186.9563	165.9	6
Y3480	188.5152	258.3	6
Y3544	190.0895	94.9	6
Y3548	190.1072	28.6	6
Y3565	190.9250	79.4	6
Y3630	192.0503	70.5	6
Y3700	193.5210	166.8	6
Y3706	193.9980	44.5	6
Y3768	195.0877	22.5	6
Y3804	196.0561	99.8	6
Y3851	197.1285	183.0	6
Y3857	197.5206	98.1	6
Y3873	197.9839	99.8	6
Y3877	198.0224	100.7	6
Y3948	199.6366	158.7	6
Y3952	199.9965	289.8	6
Y3955	200.0253	86.1	6
Y4059	202.9753	34.6	6
Y4060	202.9894	98.4	6
Y408	105.4921	267.8	6
Y4162	204.9666	95.1	6
Y4219	206.0456	104.1	6
Y4393	210.0913	176.7	6
Y44	88.0042	260.5	6
Y4400	210.1270	21.8	6
Y4412	210.8993	158.9	6
Y4418	210.9680	94.0	6
Y4531	213.9410	118.0	6
Y4605	215.4192	79.1	6
Y4619	215.9365	94.8	6
Y4728	218.5385	89.6	6
Y4734	218.8304	48.1	6
Y475	108.9614	267.9	6
Y4792	219.9312	79.0	6
Y4843	220.9997	89.1	6
Y490	109.9786	254.9	6
Y498	110.0293	35.0	6
Y4988	224.0895	52.1	6
Y500	110.0350	37.7	6
Y5112	226.9734	43.8	6
Y5198	229.0099	100.1	6
Y5230	230.0372	170.3	6
Y5305	231.9366	271.6	6
Y5397	233.9839	92.0	6
Y5493	235.9621	271.8	6
Y5531	236.5140	91.1	6
Y5539	236.9045	78.8	6

Y5575	237.3293	278.9	6
Y5593	238.0378	66.4	6
Y5622	238.8941	158.3	6
Y5657	239.5310	89.6	6
Y5666	240.0022	61.7	6
Y5716	241.0633	110.0	6
Y5725	241.1546	49.1	6
Y5758	242.1579	49.5	6
Y5955	247.1328	187.8	6
Y5999	248.1738	29.1	6
Y6056	250.0349	88.5	6
Y6127	251.9945	91.1	6
Y6145	252.1442	67.0	6
Y6192	253.8786	78.8	6
Y6200	254.0102	92.5	6
Y6215	254.1386	174.4	6
Y6307	256.9636	58.3	6
Y6394	258.9961	44.5	6
Y6507	261.9613	74.7	6
Y6600	264.2402	181.0	6
Y6629	265.1116	60.3	6
Y6648	265.9269	279.9	6
Y6662	266.1150	61.0	6
Y6674	266.5289	89.4	6
Y6825	270.5227	128.3	6
Y6833	270.8913	78.1	6
Y6919	272.8549	94.0	6
Y7120	277.9501	98.5	6
Y7311	282.9732	79.1	6
Y7339	283.5440	127.1	6
Y743	117.9698	78.1	6
Y7468	287.0037	99.2	6
Y7491	287.6507	82.3	6
Y7525	288.5920	109.1	6
Y7630	290.9345	94.0	6
Y771	118.9206	97.2	6
Y7731	293.9628	80.4	6
Y7779	295.9382	46.9	6
Y7808	296.9231	97.0	6
Y7912	300.0156	93.4	6
Y7948	300.9835	79.3	6
Y8000	302.1438	49.3	6
Y8070	304.5871	92.5	6
Y815	120.0089	100.4	6
Y8152	306.9979	279.6	6
Y8178	307.5726	89.3	6
Y8189	308.0406	44.4	6
Y8212	308.9449	91.2	6
Y825	120.0558	90.6	6
Y8251	310.0453	41.3	6
Y8352	312.9641	265.5	6
Y8451	315.1741	255.7	6
Y8483	316.1773	182.3	6
Y8498	316.9142	289.4	6
Y8522	317.4962	89.3	6
Y8531	317.9082	78.6	6
Y8570	318.9766	99.4	6
Y8580	319.0614	264.8	6
Y86	90.5258	278.1	6

			Y8622	320.9921	101.2	6
			Y8636	321.5046	128.3	6
			Y8638	321.5374	167.9	6
			Y8783	326.6601	27.2	6
			Y882	122.0192	274.6	6
			Y894	122.0714	61.0	6
			Y8964	331.5416	256.9	6
			Y901	122.5204	79.8	6
			Y9040	333.5640	254.8	6
			Y905	122.5739	105.2	6
			Y9069	334.4905	90.3	6
			Y9337	343.1919	88.5	6
			Y9345	343.2952	26.2	6
			Y9376	344.2431	26.5	6
			Y9394	344.9996	42.3	6
			Y9429	346.0031	42.4	6
			Y943	123.9547	272.2	6
			Y9458	346.9980	42.4	6
			Y9587	351.8557	75.0	6
			Y9594	351.9870	91.8	6
			Y961	124.0758	136.4	6
			Y9615	352.8533	78.0	6
			Y9635	353.4644	91.4	6
			Y9643	353.8976	49.2	6
			Y971	124.9564	15.5	6
			Y9749	357.5132	99.8	6
			Y9756	358.0149	100.2	6
			Y9800	359.3156	153.7	6
			Y9815	360.0251	281.4	6
			Y9816	360.0367	98.0	6
			Y9939	364.9707	90.7	6
X17	*Tnfrsf9*	7	Y10184	372.6074	263.7	7
			Y1059	127.0253	52.1	7
			Y10753	394.0341	100.8	7
			Y10784	395.0374	99.5	7
			Y10811	396.1201	100.3	7
			Y10972	402.5138	168.3	7
			Y11611	428.9612	42.3	7
			Y11912	441.0686	100.3	7
			Y14325	565.6298	101.4	7
			Y14704	586.6387	47.6	7
			Y16507	685.1244	99.9	7
			Y17026	716.7184	171.9	7
			Y17194	732.4029	44.1	7
			Y17757	789.6188	25.1	7
			Y2054	153.0010	121.4	7
			Y2191	156.9695	177.7	7
			Y2212	157.0738	89.8	7
			Y2226	157.5374	100.2	7
			Y2261	158.5355	100.5	7
			Y2311	159.5339	99.9	7
			Y2466	163.0714	72.8	7
			Y2559	165.1638	184.6	7
			Y2697	169.0844	32.2	7
			Y2733	170.0475	100.1	7
			Y2745	170.5151	37.5	7
			Y3794	195.9681	257.0	7
			Y4618	215.9270	270.6	7
			Y4753	219.0417	100.1	7

			Y4784	219.5434	99.9	7
			Y4807	220.0440	100.1	7
			Y4872	221.2264	185.4	7
			Y4913	222.5247	168.5	7
			Y5182	228.4642	113.4	7
			Y5591	238.0154	100.2	7
			Y5855	244.5073	99.4	7
			Y6671	266.3584	128.7	7
			Y728	117.0740	68.3	7
			Y734	117.5043	80.5	7
			Y8201	308.5241	100.8	7
			Y8764	326.0636	141.2	7
			Y9993	366.5668	100.3	7
X13	*Smad3*	8	Y10019	367.2454	51.8	8
			Y10026	367.5359	127.3	8
			Y10042	368.0376	127.6	8
			Y10075	369.2245	65.1	8
			Y10132	371.0361	240.8	8
			Y10134	371.0578	76.8	8
			Y10159	371.9326	167.7	8
			Y10259	375.5334	167.8	8
			Y10329	378.5207	128.5	8
			Y10348	379.0343	100.4	8
			Y10359	379.5360	100.1	8
			Y10400	380.9844	261.8	8
			Y10401	381.0006	116.7	8
			Y10432	382.0931	156.1	8
			Y10456	383.0389	168.7	8
			Y10476	383.5403	169.0	8
			Y10554	386.1368	28.3	8
			Y10560	386.5095	127.2	8
			Y10576	387.0001	266.0	8
			Y10646	389.1349	57.7	8
			Y10681	391.0195	35.5	8
			Y10708	392.1177	261.8	8
			Y10737	393.2349	25.1	8
			Y10768	394.5063	167.6	8
			Y10912	400.1157	28.6	8
			Y10940	401.1191	28.9	8
			Y10946	401.7267	22.9	8
			Y10955	402.0121	168.4	8
			Y10960	402.1127	28.5	8
			Y10989	403.0152	168.7	8
			Y11091	406.8550	44.3	8
			Y11096	406.9909	34.9	8
			Y11156	409.5513	258.0	8
			Y11186	410.8833	50.9	8
			Y11198	411.2506	42.9	8
			Y11266	414.0478	130.9	8
			Y11270	414.1410	50.2	8
			Y11280	414.5240	261.3	8
			Y11325	416.1106	68.8	8
			Y11349	417.1140	69.0	8
			Y11369	418.1078	68.6	8
			Y11370	418.1564	70.9	8
			Y11411	420.0226	167.8	8
			Y11599	428.6850	43.9	8
			Y11644	430.1264	43.8	8
			Y11653	430.4040	21.5	8

Y11664	431.0025	260.9	8
Y11669	431.0426	190.9	8
Y11757	434.0834	141.4	8
Y11770	434.6907	47.7	8
Y11782	435.0167	52.5	8
Y11870	438.7120	43.7	8
Y119	92.0165	65.8	8
Y11902	440.7106	43.7	8
Y11922	441.7378	68.6	8
Y11936	442.2396	68.6	8
Y11942	442.7080	44.0	8
Y11955	443.0387	254.2	8
Y11956	443.0387	168.4	8
Y12025	446.0891	137.0	8
Y12035	446.5906	137.9	8
Y12106	449.5440	100.3	8
Y12130	450.7462	46.9	8
Y12177	453.0906	272.1	8
Y12204	454.6944	45.9	8
Y12228	456.0508	88.3	8
Y12252	457.0732	136.1	8
Y12255	457.1671	65.8	8
Y12260	457.2715	47.8	8
Y12274	458.1619	75.9	8
Y12323	460.6864	46.1	8
Y12351	462.0117	100.1	8
Y12418	465.0420	197.9	8
Y12450	466.0626	115.3	8
Y12487	468.1218	140.2	8
Y125	92.0576	42.5	8
Y12522	470.1286	140.2	8
Y1256	132.4752	124.3	8
Y1258	132.4987	53.0	8
Y12816	484.9783	134.2	8
Y12840	486.0865	92.6	8
Y12859	487.1511	140.2	8
Y12902	490.0993	282.5	8
Y13059	498.5343	99.6	8
Y13087	499.9716	57.4	8
Y13092	500.0610	90.4	8
Y13171	504.0096	114.4	8
Y13172	504.0575	109.6	8
Y1321	134.0600	228.1	8
Y13283	510.6204	47.5	8
Y13293	510.9931	57.1	8
Y13312	512.0436	126.9	8
Y13340	513.2383	144.8	8
Y13479	522.6786	44.8	8
Y13569	526.5600	100.1	8
Y138	93.0448	85.9	8
Y13885	542.1733	56.9	8
Y14150	554.9518	290.4	8
Y14204	558.1445	231.6	8
Y1429	137.0459	131.2	8
Y1436	137.0718	45.5	8
Y1447	137.5153	34.5	8
Y1467	138.0429	131.9	8
Y14670	584.5527	47.5	8
Y1472	138.0661	18.2	8

Y14776	590.5765	44.5	8
Y15117	607.0787	280.5	8
Y15360	616.4565	25.6	8
Y15373	617.0217	129.2	8
Y154	94.5151	264.2	8
Y15584	626.6191	46.9	8
Y15606	627.8240	289.6	8
Y15636	629.5967	285.8	8
Y15650	630.5710	47.4	8
Y16048	655.1129	187.3	8
Y16082	657.6110	253.6	8
Y16177	662.8666	56.1	8
Y16210	664.5166	46.2	8
Y16306	672.1035	131.7	8
Y16309	672.5363	43.8	8
Y16332	674.5371	43.7	8
Y16391	678.4947	46.8	8
Y16452	682.4921	46.5	8
Y16655	695.9087	99.3	8
Y16659	696.1158	130.8	8
Y16670	697.1200	123.4	8
Y16688	698.5474	45.0	8
Y16720	701.0016	100.2	8
Y169	95.0855	249.5	8
Y16929	712.1111	168.6	8
Y1704	144.0631	52.5	8
Y17072	721.0615	110.1	8
Y17086	721.7297	108.2	8
Y17213	734.0655	124.7	8
Y17223	734.5661	18.2	8
Y17274	740.5554	274.9	8
Y17357	749.1246	252.4	8
Y17367	750.0638	168.3	8
Y1751	145.0760	17.3	8
Y17545	766.7733	52.1	8
Y17697	782.5752	18.4	8
Y1780	146.0271	37.3	8
Y1812	147.0228	57.2	8
Y1815	147.0304	37.7	8
Y18181	853.2221	139.3	8
Y1855	148.0229	35.2	8
Y1856	148.0276	47.2	8
Y1857	148.0300	45.9	8
Y1873	148.1159	87.1	8
Y1906	149.0540	194.9	8
Y207	98.0318	180.5	8
Y2169	156.0768	87.7	8
Y2185	156.8452	40.3	8
Y2214	157.0801	88.5	8
Y2237	158.0377	101.0	8
Y2264	158.8438	42.1	8
Y2341	160.1332	35.9	8
Y2387	161.1303	35.5	8
Y2390	161.1366	39.7	8
Y2433	162.1399	39.6	8
Y2471	163.0866	33.2	8
Y259	100.0216	43.3	8
Y291	101.0599	33.5	8
Y3021	177.0466	192.2	8

Y3040	177.1232	86.3	8
Y3063	178.0240	46.3	8
Y3274	183.0529	88.1	8
Y3453	187.5471	127.7	8
Y3543	190.0894	238.8	8
Y3620	191.9916	102.6	8
Y3669	193.0415	29.9	8
Y3707	194.0044	141.1	8
Y3815	196.5146	34.2	8
Y3822	196.8570	45.8	8
Y3960	200.0684	44.3	8
Y3990	201.0718	44.4	8
Y4007	201.5097	168.1	8
Y4178	205.0819	72.1	8
Y4228	206.0853	72.7	8
Y4389	210.0503	47.0	8
Y4421	211.0168	171.7	8
Y4451	211.5172	117.9	8
Y4482	212.5078	90.1	8
Y4485	212.5545	87.4	8
Y4499	213.0160	122.0	8
Y4527	213.5487	87.2	8
Y4544	214.0512	196.1	8
Y4547	214.1071	26.6	8
Y4557	214.5175	258.3	8
Y4579	215.0193	144.9	8
Y4586	215.0497	196.3	8
Y4610	215.5200	131.2	8
Y4686	217.1949	251.9	8
Y4691	217.5179	261.9	8
Y4708	218.0625	235.3	8
Y4750	219.0265	55.1	8
Y4769	219.1340	135.7	8
Y4786	219.5592	155.9	8
Y4804	220.0298	55.4	8
Y4808	220.0482	81.4	8
Y4823	220.1373	135.6	8
Y4849	221.0384	81.9	8
Y4934	223.0254	168.8	8
Y4936	223.0326	83.5	8
Y4946	223.1074	192.1	8
Y4958	223.5040	131.8	8
Y497	110.0271	65.3	8
Y5026	225.0355	58.3	8
Y503	110.0714	90.4	8
Y5053	225.3789	160.1	8
Y5075	226.0379	57.3	8
Y5114	226.9926	47.4	8
Y518	111.0305	66.5	8
Y5204	229.0639	26.1	8
Y5250	230.3558	256.6	8
Y5278	231.0435	139.1	8
Y528	111.0747	88.9	8
Y5333	232.1727	132.1	8
Y5355	233.0247	200.3	8
Y5356	233.0376	87.0	8
Y5361	233.0670	138.5	8
Y5449	235.0205	146.9	8
Y5480	235.5226	197.6	8

Y5483	235.5624	140.1	8
Y5526	236.4571	257.9	8
Y5544	236.9568	246.1	8
Y5598	238.0634	28.5	8
Y561	112.0870	83.8	8
Y570	112.5257	267.9	8
Y5764	242.5362	168.6	8
Y5767	242.5619	155.0	8
Y5776	242.9928	145.6	8
Y5780	243.0065	62.8	8
Y5812	243.5468	92.1	8
Y5815	243.5643	155.6	8
Y5834	244.0616	156.5	8
Y6085	250.9626	261.1	8
Y6098	251.0849	25.2	8
Y6136	252.0536	83.7	8
Y6157	252.8656	47.4	8
Y6162	252.9512	40.8	8
Y6229	254.9501	54.3	8
Y6237	255.0283	257.2	8
Y6274	256.0734	27.1	8
Y6293	256.5254	128.0	8
Y6353	257.9950	124.0	8
Y6436	260.0771	60.2	8
Y6482	261.1809	74.3	8
Y6635	265.2121	43.6	8
Y6681	266.8418	45.3	8
Y6747	268.3438	168.4	8
Y6771	269.0952	27.1	8
Y6802	270.0643	85.9	8
Y6876	271.5576	101.7	8
Y6877	271.5906	56.9	8
Y6892	272.0922	57.2	8
Y6935	273.0750	64.9	8
Y6951	273.5315	114.9	8
Y6959	273.9732	139.0	8
Y696	116.0707	70.2	8
Y6965	274.0184	261.0	8
Y6977	274.0921	66.5	8
Y7011	275.0025	130.8	8
Y7020	275.0955	66.4	8
Y7076	276.7822	44.1	8
Y7084	276.9626	130.7	8
Y7117	277.8826	260.3	8
Y7137	278.1064	70.3	8
Y7169	279.0934	167.6	8
Y7177	279.1919	52.5	8
Y7204	280.0627	87.4	8
Y7213	280.1540	28.3	8
Y7218	280.2350	242.0	8
Y7230	280.8183	43.8	8
Y724	117.0659	59.4	8
Y7398	285.0837	87.7	8
Y7535	288.9775	168.7	8
Y7536	288.9839	168.7	8
Y7544	289.0312	197.6	8
Y7551	289.1178	23.9	8
Y757	118.0612	56.2	8
Y7679	292.1026	67.3	8

	Y7681	292.1141	91.5	8
	Y7709	293.0951	24.2	8
	Y7726	293.5815	128.8	8
	Y7783	296.0092	57.4	8
	Y7786	296.0301	124.0	8
	Y7792	296.0806	160.0	8
	Y7836	297.9038	123.8	8
	Y7842	298.0189	134.6	8
	Y787	119.0353	128.4	8
	Y7877	299.0559	122.5	8
	Y7889	299.1365	54.4	8
	Y7902	299.5874	171.7	8
	Y7969	301.1734	57.7	8
	Y8019	302.9704	57.7	8
	Y8126	306.1297	78.3	8
	Y8185	307.9645	57.0	8
	Y8258	310.1128	66.0	8
	Y8272	310.5014	127.1	8
	Y8294	311.1168	66.5	8
	Y8466	315.9145	125.5	8
	Y8527	317.6742	82.6	8
	Y853	121.0626	66.1	8
	Y8628	321.0624	202.7	8
	Y8659	322.2432	52.2	8
	Y8760	326.0215	84.6	8
	Y8762	326.0462	119.6	8
	Y8813	327.2524	281.9	8
	Y8836	328.0298	264.6	8
	Y8860	328.5949	87.7	8
	Y887	122.0474	31.0	8
	Y8911	330.0255	112.9	8
	Y8936	330.9972	34.5	8
	Y8961	331.2841	201.7	8
	Y9008	332.9223	44.4	8
	Y9074	334.5729	141.2	8
	Y9104	335.1982	47.8	8
	Y9170	337.2138	47.9	8
	Y9220	339.0792	139.3	8
	Y9242	340.0807	264.6	8
	Y9271	341.0750	139.4	8
	Y9332	343.1035	246.3	8
	Y9365	344.0170	116.2	8
	Y9395	345.0186	125.1	8
	Y9462	347.0251	123.5	8
	Y9463	347.0263	255.3	8
	Y9471	347.1423	188.9	8
	Y9516	349.1175	145.3	8
	Y9535	350.0554	231.3	8
	Y9540	350.1197	72.4	8
	Y9569	351.0590	133.7	8
	Y9633	353.2668	42.9	8
	Y9680	355.0384	139.9	8
	Y9693	355.5301	126.3	8
	Y9802	359.5472	128.3	8
	Y9813	360.0033	46.7	8
	Y9817	360.0482	128.4	8
	Y9915	363.9928	116.1	8
	Y9922	364.0651	178.8	8

Table S6b. Metabolite tentative annotation in strain network								
m/z	RT	ID	match_for	mz_differe	name	pathway	community	
85.0415	68.2	CE3087	M+2H[2+]	-1.00E-04	beta-carbo	Tryptopha	1	
88.0394	70.5	C00439	M+2H[2+]	1.00E-04	N-Formimi	Histidine m	1	
98.9755	95.5	C00059	M+H[1+]	8.00E-04	Sulfate; Su	Glycosphir	1	
106.0685	65.2	C00334	M(Cl37)+H	7.00E-04	4-Aminobu	Butanoate	1	
108.0114	52.2	C00158	M+H+Na[2	-4.00E-04	Citrate; Cit	TCA cycle	1	
119.0615	28.8	C16622	M(S34)+H[	-2.00E-04	N,N'-Diace	Drug meta	1	
119.0896	55.8	C00183	M(C13)+H[	-1.00E-04	L-Valine; 2	Valine, leu	1	
123.0553	33.4	C00153	M+H[1+]	0	Nicotinam	Vitamin B3	1	
123.5219	197.1	C05645	M+H+Na[2	-4.00E-04	4-(2-Amino	Tryptopha	1	
124.0585	33.4	C00153	M(C13)+H[	-2.00E-04	Nicotinam	Vitamin B3	1	
125.0618	30.6	C00503	M(Cl37)+H	-6.00E-04	Erythritol; Erythrol; Pl		1	
130.0499	68.4	C01879	M+H[1+]	0	5-Oxoproli	Glutathion	1	
131.0344	51.6	C00490	M+H[1+]	5.00E-04	Itaconate;	C5-Branche	1	
131.0469	70.5	C07501	M+H+Na[2	9.00E-04	Felbamate	Drug meta	1	
131.0531	69.0	C01879	M(C13)+H[	-2.00E-04	5-Oxoproli	Glutathion	1	
134.0448	71.8	C00049	M+H[1+]	0	L-Aspartat	Purine met	1	
135.0425	59.9	C04221	M+Na[1+]	8.00E-04	trans-1,2-Dihydroben		1	
135.0482	69.8	C00049	M(C13)+H[	0	L-Aspartat	Purine met	1	
135.0634	190.5	CE2065	M(C13)+2H	-2.00E-04	acetylcarn	Histidine m	1	
135.0804	21.9	C06103	M(S34)+H[	-0.0013	6-Hydroxyhexanoic ac		1	
138.055	63.1	C00108	M+H[1+]	0	Anthranila	Tryptopha	1	
139.039	216.5	C00156	M+H[1+]	0	4-Hydroxy	Ubiquinon	1	
139.1117	22.8	C16300	M+2H[2+]	0	Stearidoni	Omega-3 f	1	
142.0264	98.8	C00334	M+K[1+]	3.00E-04	4-Aminobu	Butanoate	1	
143.0295	100.8	C00346	M(C13)+H[	-3.00E-04	Ethanolam	Glycosphir	1	
144.0114	198.9	CE0074	M(C13)+H[	-7.00E-04	alloxan	Purine met	1	
146.1176	39.2	C01181	M[1+]	-5.00E-04	4-Trimethy	Lysine met	1	
147.0257	197.0	C06604	M(C13)+2H	2.00E-04	Parathion;	Parathio de	1	
148.0604	67.7	C00025	M+H[1+]	-1.00E-04	L-Glutama	Purine met	1	
149.0263	54.0	C00026	M(Cl37)+H	3.00E-04	2-Oxogluta	Valine, leu	1	
149.0635	68.1	C00025	M(C13)+H[	-4.00E-04	L-Glutama	Purine met	1	
152.0566	179.6	C00073	M(Cl37)+H	0.0011	L-Methion	Glycine, se	1	
153.0541	180.4	C00642	M+H[1+]	-5.00E-04	4-Hydroxy	Tyrosine m	1	
153.06	169.4	C00242	M(C13)+H[	-1.00E-04	Guanine; 2	Urea cycle,	1	
153.0772	64.7	C00379	M+H[1+]	0.0014	Xylitol	Pentose ar	1	
154.0441	38.3	C00385	M(C13)+H[	0	Xanthine	Purine met	1	
155.0025	194.8	C01119	M(Cl37)+H	0.0014	Oxidized d	Selenoami	1	
155.0112	78.2	C00141	M+K[1+]	0.0011	3-Methyl-2	Valine, leu	1	
156.0422	54.7	C00183	M+K[1+]	4.00E-04	L-Valine; 2	Valine, leu	1	
156.0506	125.7	C06608	M(C13)+H[	4.00E-04	Diethylpho	Parathio de	1	
157.0096	34.6	C00022	M+HCOON	-0.0011	Pyruvate; I	Pyruvate M	1	
157.0455	53.4	C06608	M(Cl37)+H	0.0015	Diethylpho	Parathio de	1	
157.5187	102.2	C05695	M(C13)+2H	-0.0015	gamma-Gl	Selenoami	1	
158.0402	54.6	C00341	M+2H[2+]	-0.0013	Geranyl dip	Squalene a	1	

158.9615	16.7	C11036	M(Cl37)+H	-5.00E-04	Bromoben	Xenobiotic	1	
159.0436	54.5	C02617	M+H[1+]	-5.00E-04		Xenobiotic	1	
160.0842	119.6	C02909	M(C13)+H[	3.00E-04	(2-Naphthy	1- and 2-M	1	
160.9998	99.6	C00180	M+K[1+]	2.00E-04	Benzoate;	Alkaloid bi	1	
162.0762	60.0	C00956	M+H[1+]	1.00E-04	L-2-Amino	Lysine met	1	
163.0753	16.4	C06205	M+H[1+]	-1.00E-04	1,2-Dihydr	Xenobiotic	1	
164.0083	93.5	C00346	M+Na[1+]	-1.00E-04	Ethanolam	Glycosphir	1	
168.0653	283.9	C00250	M+H[1+]	-2.00E-04	Pyridoxal	Vitamin B6	1	
169.1067	181.7	C01181	M+Na[1+]	-7.00E-04	4-Trimethy	Lysine met	1	
176.1281	193.9	C06213	M(C13)+H[	0.0017	N-Methylt	Tryptopha	1	
176.9998	36.8	C00265	M+Na[1+]	-0.0017	Dithiothrei	Selenoami	1	
177.0409	30.0	C00072	M+H[1+]	0.0015	Ascorbate;	Prostaglan	1	
178.0444	33.0	C00072	M(C13)+H[	0.0016	Ascorbate;	Prostaglan	1	
178.0983	265.1	C00327	M(S34)+H[	-5.00E-04	L-Citrulline	Arginine ar	1	
181.0443	198.2	C01419	M(Cl37)+H	-0.0014	Cys-Gly; L-	Glycine, se	1	
182.9814	76.6	C05689	M[1+]	0.0015	Se-Methyl:	Selenoami	1	
183.0369	197.6	C00144	M(C13)+2H	-0.0011	GMP; Guar	Purine met	1	
183.0782	45.7	C06423	M+K[1+]	4.00E-04	Octanoic a	Fatty acid a	1	
185.1188	275.0	C05956	M+2H[2+]	0.0016	Prostaglan	Prostaglan	1	
186.0165	62.0	C00025	M+K[1+]	5.00E-04	L-Glutama	Purine met	1	
186.0526	92.7	C12455	M+HCOOK	-1.00E-04	5-Aminopentanal		1	
187.0366	229.0	C00166	M+Na[1+]	0	Phenylpyru	Tyrosine m	1	
188.0915	25.9	C05548	M+H[1+]	-3.00E-04	6-Acetami	Butanoate	1	
189.041	33.8	C01717	M[1+]	-0.0016	4-Hydroxy-	Tryptopha	1	
189.0949	26.0	C05548	M(C13)+H[	-3.00E-04	6-Acetami	Butanoate	1	
191.0104	205.1	C00642	M+K[1+]	3.00E-04	4-Hydroxy	Tyrosine m	1	
195.0299	138.3	C00158	M(Cl37)+H	-0.0016	Citrate; Cit	TCA cycle	1	
196.013	34.2	C00380	M+HCOOK	0.0011	Cytosine	Pyrimidine	1	
200.0442	79.5	C16550	M(C13)+H[	0.001	Dechloroe	Drug meta	1	
205.0731	144.2	C05660	M[1+]	-8.00E-04	5-Methoxy	Tryptopha	1	
205.1192	168.9	C00521	M+HCOON	-7.00E-04	(-)-Limonene; (-)-(S)-L		1	
207.0058	102.2	C00180	M+HCOOK	4.00E-04	Benzoate;	Alkaloid bi	1	
212.0537	89.3	C00624	M+Na[1+]	7.00E-04	N-Acetyl-L	Urea cycle,	1	
215.0331	168.2	C00233	M+HCOOK	0.0015	4-Methyl-2	Valine, leu	1	
215.0543	196.3	C01233	M[1+]	-0.0016	sn-glycero-	Glyceroph	1	
217.5404	89.5	C05692	M(C13)+2H	-0.0016	Se-Adenos	Selenoami	1	
218.0421	89.6	C00329	M+K[1+]	-1.00E-04	D-Glucosar	Aminosuga	1	
224.6055	121.8	C11133	M(C13)+2H	-5.00E-04	Estrone glu	Androgen a	1	
225.0472	54.4	C03680	M+HCOON	-0.001	4-Imidazol	Histidine m	1	
228.0801	61.3	C16365	M(C13)+H[	-8.00E-04	5-Acetylan	Caffeine m	1	
229.5897	154.2	C00445	M(C13)+2H	-9.00E-04	5,10-Meth	Vitamin B9	1	
231.0092	170.3	C01127	M+HCOON	-0.0019	4-Hydroxy-	Arginine ar	1	
231.025	106.0	C00117	M+H[1+]	-0.0015	D-Ribose 5	Pentose ph	1	
231.0432	219.6	C01107	M(Cl37)+H	-0.0012	(R)-5-Phos	Squalene a	1	
235.004	129.5	C00458	M(C13)+2H	2.00E-04	dCTP; Deo	Pyrimidine	1	
235.0456	156.5	C13690	M(C13)+H[	-9.00E-04	Dopamine	Tyrosine m	1	
236.957	103.0	C04309	M+K[1+]	0.0013	Phosphoenol-4-deoxy		1	

236.9771	40.2	C00074	M+HCOON	0	Phosphoer	Purine me	1	
236.9828	128.9	C01756	M+HCOOK	-0.0015	Thiopurine	Urea cycle,	1	
239.1029	185.1	C00268	M[1+]	0.0011	Dihydrobic	Tyrosine m	1	
243.0155	45.5	C16196	M(Cl37)+H	0.0018	1,2-Dihydroxynaphtha		1	
243.0432	208.6	C02909	M+HCOOK	0.0014	(2-Naphthy	1- and 2-M	1	
244.0946	81.4	C00475	M+H[1+]	0.0018	Cytidine	Pyrimidine	1	
245.0074	241.3	C00322	M+HCOOK	0.0016	2-Oxoadipa	Tryptopha	1	
245.04	203.7	C00438	M+HCOON	0.002	N-Carbamo	Pyrimidine	1	
248.0876	99.9	C00664	M+H+Na[2	-0.0016	5-Formimi	Vitamin B9	1	
252.108	90.5	C00559	M+H[1+]	-0.0011	Deoxyader	Purine me	1	
253.513	100.6	C03028	M+2H[2+]	1.00E-04	Thiamin tri	Vitamin B1	1	
254.8156	45.4	C01382	M+H[1+]	-6.00E-04	Iodine; I2	Tyrosine m	1	
255.0649	115.5	C05844	M+H[1+]	3.00E-04	5-L-Glutam	Methionin	1	
256.813	45.4	C01382	M(Cl37)+H	-4.00E-04	Iodine; I2	Tyrosine m	1	
263.0149	209.5	C00198	M+HCOOK	-0.0014	D-Glucono	Pentose ph	1	
267.5556	214.5	C15974	M+H+Na[2	-0.0017	3-Methyl-1	Valine, leu	1	
271.0678	105.1	C03415	M+K[1+]	-9.00E-04	N2-Succiny	Aspartate a	1	
273.1201	73.2	C02571	M+HCOON	0.0018	O-Acetylca	Aspartate a	1	
275.0165	206.4	C00944	M+HCOOK	2.00E-04	3-Dehydroquinate; 5-		1	
277.0673	215.6	C06606	M(C13)+H[	7.00E-04	Paraoxon;	Parathio de	1	
279.074	209.4	C05841	M+Na[1+]	0.0026	Nicotinate	Vitamin B3	1	
283.0455	99.8	C00842	M+2H[2+]	3.00E-04	dTDP-gluco	Nucleotide	1	
285.0911	128.9	C11376	M+2H[2+]	-8.00E-04	SN38 glucu	Drug meta	1	
286.151	92.0	CE5251	M(C13)+H[	-9.00E-04	estrone-3,4	Androgen a	1	
287.0763	118.8	C01762	M(S34)+H[	-0.0025	Xanthosine	Purine me	1	
288.065	206.5	C03838	M(C13)+H[	-0.0023	5'-Phospho	Purine me	1	
288.0696	266.3	C03838	M(C13)+H[	0.0023	5'-Phospho	Purine me	1	
290.0983	107.4	CE2065	M+Na[1+]	-3.00E-04	acetylcarn	Histidine m	1	
291.1026	98.6	C07030	M(C13)+2H	0.0015		Drug meta	1	
298.0966	28.5	C00170	M+H[1+]	-3.00E-04	5'-Methylt	Methionin	1	
299.584	109.6	C14855	M+H+Na[2	-6.00E-04	4,5-Dihydr	Xenobiotic	1	
302.0635	155.4	C00357	M+H[1+]	-1.00E-04	N-Acetyl-D	Aminosuga	1	
303.5463	241.1	C00096	M+2H[2+]	4.00E-04	GDP-mann	N-Glycan b	1	
304.0792	167.5	C00864	M+HCOOK	-1.00E-04	Pantothen	CoA Catab	1	
305.0984	281.1	C14853	M(Cl37)+H	-4.00E-04	Benzo[a]py	Xenobiotic	1	
308.0909	197.2	C00051	M+H[1+]	-2.00E-04	Glutathion	Pyruvate M	1	
310.0949	195.9	C01169	M(Cl37)+H	-8.00E-04	S-Succinylc	TCA cycle	1	
313.0293	205.6	C00281	M+Na[1+]	-3.00E-04	Sedoheptu	Pentose ph	1	
316.0418	212.9	C05932	M+HCOOK	-0.0011	N-Succinyl-	Aspartate a	1	
325.0622	241.4	C00055	M(C13)+H[	-4.00E-04	CMP; Cytic	Phosphatic	1	
325.0924	111.1	C01262	M+HCOOK	0.0016	beta-Alanyl-N(pi)-met		1	
327.0591	268.9	C00214	M+HCOOK	2.00E-04	Thymidine	Pyrimidine	1	
329.0382	37.5	C00299	M+HCOOK	1.00E-04	Uridine	Pyrimidine	1	
330.0592	75.4	C00575	M+H[1+]	-6.00E-04	3',5'-Cyclic	Purine me	1	
339.0552	218.3	C01185	M(Cl37)+H	0.0023	Nicotinate	Vitamin B3	1	
339.0554	92.3	C01185	M(Cl37)+H	0.0025	Nicotinate	Vitamin B3	1	
340.0522	198.0	C04751	M+H[1+]	-0.0019	1-(5-Phosp	Purine me	1	

342.9961	284.8	C00354	M(S34)+H[	-0.003	D-Fructose	Glycolysis a	1	
343.1421	281.4	C15658	M(C13)+H[	-8.00E-04	6-(alpha-D	Phosphatid	1	
353.0664	196.9	C06948	M+HCOON	1.00E-04	Diazepam		1	
358.0988	221.1	C01134	M[1+]	0.0024	Pantethein	CoA Catab	1	
365.0675	183.7	C00144	M(C13)+H[	-0.0012	GMP; Guan	Purine met	1	
369.0392	196.8	C06948	M+HCOOK	-0.001	Diazepam		1	
373.1036	258.5	CE2102	M+K[1+]	0.0023	lipoyllysine	Lipoate me	1	
386.0471	169.8	C00144	M+Na[1+]	-2.00E-04	GMP; Guan	Purine met	1	
387.0551	126.1	C05606	M+HCOON	-0.0036	Melanin	Tyrosine m	1	
397.2712	45.3	C01607	M+HCOOK	-2.00E-04	Phytanate;	Fatty acid a	1	
402.0208	171.6	C00144	M+K[1+]	0	GMP; Guan	Purine met	1	
403.0329	126.1	C00363	M+H[1+]	0.0027	dTDP; Deo	Pyrimidine	1	
404.095	238.4	C04352	M(C13)+H[	-0.0019	(R)-4'-Phos	Vitamin B5	1	
412.1385	102.5	CE6229	M+H[1+]	-0.0039	13E-tetran	Leukotrien	1	
414.3575	21.2	hpdcacrn	M+H[1+]	-3.00E-04	heptadeca	Carnitine s	1	
415.3609	21.4	CE7047	M[1+]	0.0033		Vitamin E r	1	
429.1017	196.1	C05399	M+HCOOK	0.0012	Melibiitol;	Galactose	1	
431.0978	196.3	C11173	M+K[1+]	-0.0022	SN-38	Drug meta	1	
432.8658	52.2	C01060	M[1+]	-0.0014	3,5-Diiodo	Tyrosine m	1	
434.0737	90.4	C05692	M(C13)+H[	-0.003	Se-Adenos	Selenoami	1	
434.1916	113.6	CE5139	M+HCOON	5.00E-04	12-oxo-20-	Leukotrien	1	
462.1945	122.5	C00440	M(Cl37)+H	0.0034	5-Methylte	Methionin	1	
469.0007	129.2	C00458	M(C13)+H[	4.00E-04	dCTP; Deo	Pyrimidine	1	
471.0773	227.9	C04352	M+HCOON	-0.0036	(R)-4'-Phos	Vitamin B5	1	
487.0898	92.4	C09820	M(C13)+2H	0.0021	Benzoylsuccinyl-CoA;		1	
508.0003	59.2	C00286	M+H[1+]	-0.0027	dGTP; 2'-D	Pyrimidine	1	
546.0706	61.0	C00687	M[1+]	0.0054	dTDP-4-de	Nucleotide	1	
616.1578	210.6	C00128	M(C13)+H[	-2.00E-04	CMP-N-ace	Glycosphin	1	
676.0726	99.6	C00043	M+HCOON	-0.0037	UDP-N-ace	Phosphatid	1	
692.0472	186.0	C00043	M+HCOOK	-0.003	UDP-N-ace	Phosphatid	1	
125.0152	26.7	C00245	M[1+]	5.00E-04	Taurine; 2-	Methionin	2	
129.0699	24.2	C00829	M+H[1+]	0	Naphthale	Xenobiotic	2	
148.004	55.6	C00245	M+Na[1+]	0	Taurine; 2-	Methionin	2	
149.0009	55.3	C00090	M+K[1+]	0.0013	Catechol; 1,2-Benzene		2	
149.0033	54.6	C01218	M+H+Na[2	5.00E-04	6-Phospho	Pentose ph	2	
151.0615	244.0	C00073	M(C13)+H[	-2.00E-04	L-Methion	Glycine, se	2	
164.0342	24.6	C01044	M(S34)+H[	-0.0013	N-Formyl-L	Aspartate a	2	
169.0268	57.2	C00233	M+K[1+]	0.001	4-Methyl-2	Valine, leu	2	
180.0653	76.7	C01586	M+H[1+]	-2.00E-04	Hippurate;	Urea cycle	2	
200.1017	27.0	C06114	M+H[1+]	-0.0013	gamma-Glutamyl-beta		2	
200.128	32.6	C12448	M+H[1+]	-1.00E-04	Ecgonine methyl este		2	
202.1439	97.8	C03626	M[1+]	9.00E-04	Nomega,Nomega'-Dir		2	
204.0783	37.0	C06199	M+K[1+]	1.00E-04	Hordenine	Tyrosine m	2	
246.0631	260.1	C11736	M[1+]	-0.0021	5-Fluorode	Drug meta	2	
269.055	61.6	C11736	M+Na[1+]	5.00E-04	5-Fluorode	Drug meta	2	
289.22	24.6	C02838	M(C13)+H[	-4.00E-04	L-Octanoylcarnitine		2	
344.9893	263.8	C02355	M+K[1+]	0.0012	2',3'-Cyclic	UMP	2	

350.9279	52.6	C00236	M+HCOOK	0	3-Phospho	Glycolysis a	2	
421.019	54.8	C01185	M+HCOOK	0.002	Nicotinate	Vitamin B3	2	
448.3418	17.2	elaidcrn	M+Na[1+]	0.002	Elaidic carn	Carnitine s	2	
450.3574	21.5	stcrn	M+Na[1+]	0.002	stearoylca	Carnitine s	2	
472.3417	21.4	c226crn	M+H[1+]	-4.00E-04	cervonyl ca	Carnitine s	2	
473.3453	21.6	c226crn	M(C13)+H[	-2.00E-04	cervonyl ca	Carnitine s	2	
502.0282	271.6	C14847	M+Na[1+]	0.0027	3,4-Dihydr	Xenobiotic	2	
113.0904	86.1	C00388	M(C13)+H[	1.00E-04	1H-Imidazo	Histidine m	3	
115.0542	227.0	C00881	M(C13)+2H	-1.00E-04	Deoxycytid	Pyrimidine	3	
130.0863	73.4	C00408	M+H[1+]	0	L-Pipecolat	Lysine met	3	
131.0895	80.0	C00408	M(C13)+H[	-2.00E-04	L-Pipecolat	Lysine met	3	
136.0617	276.2	C00147	M+H[1+]	-1.00E-04	Adenine; 6	Arginine ar	3	
138.0914	17.7	C00483	M+H[1+]	0	Tyramine;	Tyrosine m	3	
140.9035	47.7	C00023	M+HCOOK	0	Iron	Porphyrin	3	
157.0608	59.9	C03406	M+H+Na[2	0.0012	N-(L-Argini	Arginine ar	3	
165.0161	100.9	C04051	M+K[1+]	-9.00E-04	5-Amino-4	Purine met	3	
170.0925	86.6	C01152	M+H[1+]	1.00E-04	N(pi)-Methyl-L-histidi		3	
171.0958	87.5	C00534	M(Cl37)+H	0.0014	Pyridoxam	Vitamin B6	3	
176.0658	52.2	C02538	M+2H[2+]	-9.00E-04	Estrone 3-s	C21-steroi	3	
176.0706	247.2	C00954	M+H[1+]	0	Indole-3-ac	Tryptopha	3	
176.1224	83.8	C00062	M(C13)+H[	0	L-Arginine;	Alanine an	3	
184.0731	22.8	C00588	M[1+]	-8.00E-04	Choline ph	Glycosphir	3	
193.0019	268.3	C06608	M+K[1+]	-4.00E-04	Diethylpho	Parathio de	3	
201.0537	58.5	C14784	M+Na[1+]	0.0014	1,2-Dihydr	Xenobiotic	3	
210.0118	42.8	C00346	M+HCOON	-0.002	Ethanolam	Glycosphir	3	
266.9686	113.4	C00236	M+H[1+]	0.002	3-Phospho	Glycolysis a	3	
319.2245	47.2	12harachd	M[1+]	-0.0028	12 hydroxy	Arachidoni	3	
325.043	117.6	C00105	M+H[1+]	-2.00E-04	UMP; Urid	Pyrimidine	3	
368.0602	183.5	C00387	M+HCOOK	-1.00E-04	Guanosine	Purine met	3	
496.3393	18.6	clpndcrn	M+Na[1+]	-5.00E-04	clupanodo	Carnitine s	3	
497.3428	19.3	C05108	M+HCOOK	0.0037	14-Demeth	Squalene a	3	
540.3669	28.8	arachcrn	M+HCOOK	9.00E-04	arachidyl c	Carnitine s	3	
92.0244	38.1	C03012	M+H+Na[2	-1.00E-04	Naphthale	Xenobiotic	4	
113.0346	32.6	C00106	M+H[1+]	0	Uracil	Pyrimidine	4	
139.0583	58.9	C00108	M(C13)+H[	-1.00E-04	Anthranila	Tryptopha	4	
140.995	206.0	C00109	M+K[1+]	5.00E-04	2-Oxobuta	Methionin	4	
142.0326	222.4	C00870	M(Cl37)+H	0.0012	4-Nitrophe	Xenobiotic	4	
148.5363	90.2	C03373	M+2H[2+]	6.00E-04	Aminoimid	Purine met	4	
149.1132	92.0	C02797	M+H+Na[2	1.00E-04	3-Oxo-5beta-steroid		4	
151.039	232.6	C01087	M(S34)+H[	-0.0013	(R)-2-Hydroxyglutarat		4	
158.0454	40.6	C01551	M[1+]	0.0014	Allantoin; 5	Purine met	4	
159.0229	130.0	C00295	M(Cl37)+H	0.0013	Orotate; O	Pyrimidine	4	
159.0282	36.5	C00262	M+Na[1+]	4.00E-04	Hypoxanth	Purine met	4	
170.0414	72.0	C00025	M+Na[1+]	-0.0011	L-Glutama	Purine met	4	
171.1167	21.8	CE2061	M(S34)+H[	-0.0014		Linoleate r	4	
173.0212	99.0	C00093	M+H[1+]	2.00E-04	sn-Glycero	Glyceroph	4	
174.0316	198.0	C00942	M(C13)+2H	-0.0011	3',5'-Cyclic	Purine met	4	

175.0017	36.6	C00184	M+HCOOK	0.0014	Glycerone;	Glyceroph	4	
186.0855	268.0	C00588	M(C13)+H[	9.00E-04	Choline ph	Glycosphir	4	
192.9748	119.5	C00265	M+K[1+]	-2.00E-04	Dithiothre	Selenoami	4	
206.9904	128.3	C00366	M+K[1+]	-7.00E-04	Urate; Uric	Purine me	4	
207.9983	62.1	C01005	M+Na[1+]	1.00E-04	O-Phospho	Glycine, se	4	
213.001	125.1	C05379	M+Na[1+]	3.00E-04	Oxalosucci	TCA cycle	4	
215.1137	84.0	C01181	M+HCOON	9.00E-04	4-Trimethy	Lysine met	4	
218.0063	126.6	C00049	M+HCOOK	2.00E-04	L-Aspartat	Purine me	4	
221.0095	192.8	C01756	M+HCOON	-9.00E-04	Thiopurine	Urea cycle,	4	
244.0133	241.2	C15650	M(C13)+H[	0.0012	2,3-Diketo	Methionin	4	
259.1073	106.3	C06212	M+HCOON	0.002	N-Methyls	Tryptopha	4	
268.0577	280.8	C00788	M+HCOOK	-4.00E-04	L-Adrenali	Tyrosine m	4	
270.0593	222.4	C05931	M+Na[1+]	8.00E-04	N-Succinyl-	Aspartate a	4	
276.0689	211.5	C00579	M+HCOON	-0.001	Dihydrolip	Glycine, se	4	
284.053	153.4	C01233	M+HCOON	0.0024	sn-glycero-	Glyceroph	4	
291.0477	189.2	C00281	M+H[1+]	1.00E-04	Sedoheptu	Pentose ph	4	
291.0544	210.4	C07496	M+K[1+]	0.0017	Carbamaze	Drug meta	4	
300.07	222.5	C05932	M+HCOON	0.001	N-Succinyl-	Aspartate a	4	
307.0756	209.8	C14854	M+Na[1+]	0.0026	9-Hydroxy	Xenobiotic	4	
307.0835	207.6	C00051	M[1+]	-3.00E-04	Glutathion	Pyruvate M	4	
307.5824	211.1	C00127	M(C13)+2H	-0.0026	Glutathion	Tyrosine m	4	
339.0796	257.9	C14863	M+H+Na[2	1.00E-04	2-(S-Glutat	Xenobiotic	4	
342.1391	280.6	C15658	M+H[1+]	-4.00E-04	6-(alpha-D	Phosphatic	4	
373.0208	90.0	C00455	M+K[1+]	0.0014	Nicotinam	Vitamin B3	4	
404.025	284.0	C00112	M+H[1+]	-5.00E-04	CDP; Cytid	Pyrimidine	4	
423.0803	209.5	C00021	M+K[1+]	-0.0041	S-Adenosy	Ubiquinon	4	
426.9715	209.6	C01346	M+K[1+]	0.0014	dUDP; 2'-D	Pyrimidine	4	
444.0311	293.9	C00035	M+H[1+]	-5.00E-04	GDP; Guan	Glycosphir	4	
464.0809	114.2	C00401	M+HCOOK	9.00E-04	Chondroiti	Glycosphir	4	
465.0849	114.3	C03794	M(C13)+H[	2.00E-04	N6-(1,2-Di	Purine me	4	
469.0859	209.8	C00021	M+HCOOK	-0.0043	S-Adenosy	Ubiquinon	4	
608.0885	286.4	C00043	M+H[1+]	-4.00E-04	UDP-N-ace	Phosphatic	4	
614.1869	289.8	C04847	M+HCOON	-0.0034	alpha-D-Ga	lactosyl-1,	4	
102.5355	261.6	C00082	M+H+Na[2	3.00E-04	L-Tyrosine,	Tyrosine m	5	
274.9331	44.6	C14842	M+HCOOK	0.0016	Bromoben	Xenobiotic	5	
282.034	196.0	C00352	M+Na[1+]	-0.001	D-Glucosai	Aminosuga	5	
282.4973	241.5	C05922	M+H+Na[2	-0.0016	Formamide	Biopterin r	5	
110.035	37.7	C12205	M+H+Na[2	-1.00E-04	5-Hydroxy	coniferyl al	6	
126.022	56.7	C00245	M+H[1+]	0	Taurine; 2-	Methionin	6	
126.0373	217.8	C00253	M(Cl37)+H	8.00E-04	Nicotinate	Vitamin B3	6	
132.0035	280.8	C16196	M+H+Na[2	6.00E-04	1,2-Dihydr	oxynaphtha	6	
133.0339	234.8	C02362	M(C13)+H[	0.0013		Alanine an	6	
136.0143	38.4	C05699	M+2H[2+]	0.0011	Selenocyst	Selenoami	6	
136.514	91.9	C05699	M(C13)+2H	-9.00E-04	Selenocyst	Selenoami	6	
137.0458	37.4	C00155	M(C13)+H[	-3.00E-04	L-Homocys	Glycine, se	6	
138.0429	37.2	C00785	M[1+]	0	Urocanate	Histidine m	6	
138.049	38.7	C00262	M(C13)+H[	-2.00E-04	Hypoxanth	Purine me	6	

143.9523	90.0	C01755	M+HCOOK	7.00E-04	Thiocyanat	Methionin	6	
150.5286	99.3	C08249	M+H+Na[2	9.00E-04	Pyrimidine	5'-deoxynu	6	
153.0407	38.1	C00385	M+H[1+]	0	Xanthine	Purine me	6	
155.9953	186.6	C01962	M(Cl37)+H	-0.001	Thiocystei	Methionin	6	
160.0442	90.7	C00337	M(C13)+H[	7.00E-04	(S)-Dihydr	Pyrimidine	6	
164.0561	61.1	C05839	M+2H[2+]	-0.0013	cis-beta-D-	Hexose ph	6	
165.5339	89.1	C00575	M+2H[2+]	4.00E-04	3',5'-Cyclic	Purine me	6	
169.9825	69.5	C05528	M(C13)+H[	-0.0011	3-Sulfopyr	Methionin	6	
171.0419	99.5	C06103	M+K[1+]	5.00E-04	6-Hydroxyhexanoic ac		6	
173.021	178.6	C00093	M+H[1+]	0	sn-Glycero	Glyceroph	6	
190.0895	94.9	C05548	M(Cl37)+H	5.00E-04	6-Acetami	Butanoate	6	
190.1072	28.6	C05933	M[1+]	6.00E-04	N-(omega)	Arginine ar	6	
195.0876	22.5	C16586	M[1+]	-0.0019	2-Phenyl-1	Drug meta	6	
206.0456	104.1	C02470	M+H[1+]	8.00E-04	Xanthuren	Tryptopha	6	
210.0913	176.7	C02325	M[1+]	0.0021	Sinapyl alcohol; Sinap		6	
224.0895	52.1	CE5626	M(C13)+H[	0.0022	salsolinol 1	Tyrosine m	6	
229.0099	100.1	C00944	M+K[1+]	-6.00E-04	3-Dehydroquinate; 5-		6	
261.9613	74.7	C01272	M+H+Na[2	-0.0013	1D-myo-In	Phosphatic	6	
265.1116	60.3	C00378	M[1+]	-7.00E-04	Thiamin; T	Vitamin B1	6	
288.592	109.1	C14855	M+2H[2+]	-0.0016	4,5-Dihydr	Xenobiotic	6	
308.0406	44.4	C00365	M[1+]	-4.00E-04	dUMP; De	Pyrimidine	6	
312.9641	265.5	C07644	M+K[1+]	-0.0028	4-Ketocycl	Drug meta	6	
315.1741	255.7	C16300	M+K[1+]	0.0024	Stearidoni	Omega-3 f	6	
343.1919	88.5	C16548	M[1+]	-0.0017	N,N-Dides	Drug meta	6	
344.9996	42.3	C00085	M+HCOOK	0.0013	D-Fructose	Pentose ph	6	
469.0672	91.6	C05125	M[1+]	-0.004	2-(alpha-H	Butanoate	6	
469.0695	287.7	C05125	M[1+]	-0.0017	2-(alpha-H	Butanoate	6	
529.3283	27.2	C04840	M+HCOOK	-6.00E-04	3beta-Hydroxy-4beta-		6	
117.074	68.3	C00148	M(C13)+H[	0	L-Proline; 2	Arginine ar	7	
127.0253	52.1	C00245	M(C13)+H[	-1.00E-04	Taurine; 2-	Methionin	7	
158.5355	100.5	C04376	M(C13)+2H	8.00E-04	5'-Phosphc	Purine me	7	
169.0844	32.2	C00179	M+K[1+]	-2.00E-04	Agmatine;	Arginine ar	7	
219.0417	100.1	C00590	M+K[1+]	3.00E-04	Coniferyl alcohol; Cor		7	
220.044	100.1	C00242	M+HCOON	-1.00E-04	Guanine; 2	Urea cycle,	7	
92.0165	65.8	C00322	M+H+Na[2	-4.00E-04	2-Oxoadip	Tryptopha	8	
93.0448	85.9	C00588	M+2H[2+]	6.00E-04	Choline ph	Glycosphir	8	
110.0271	65.3	C00257	M+H+Na[2	-3.00E-04	D-Gluconic	Pentose ph	8	
111.0305	66.5	C00519	M(C13)+H[	1.00E-04	Hypotaurir	Methionin	8	
112.087	83.8	C00388	M+H[1+]	1.00E-04	1H-Imidaz	Histidine m	8	
116.0707	70.2	C00148	M+H[1+]	1.00E-04	L-Proline; 2	Arginine ar	8	
117.0659	59.4	C16622	M+H[1+]	0	N,N'-Diace	Drug meta	8	
118.0612	56.2	C00581	M+H[1+]	1.00E-04	Guanidino	Glycine, se	8	
121.0626	66.1	CE2705	M+2H[2+]	5.00E-04	quinonoid	Biopterin r	8	
122.0474	31.0	C00153	M[1+]	-6.00E-04	Nicotinam	Vitamin B3	8	
134.06	228.1	C00430	M(S34)+H[	-0.0013	5-Aminole	Glycine, se	8	
137.0459	131.2	C00155	M(C13)+H[	-2.00E-04	L-Homocys	Glycine, se	8	
137.0718	45.5	1mncam	M[1+]	3.00E-04	1-Methyln	Vitamin B3	8	

138.0429	131.9	C00785	M[1+]	0	Urocanate	Histidine m	8	
138.0661	18.2	C07054	M+H[1+]	-1.00E-04	Isoniazid	Drug meta	8	
146.0271	37.3	C00281	M+2H[2+]	-3.00E-04	Sedoheptu	Pentose pl	8	
148.1159	87.1	C00047	M(C13)+H[	-3.00E-04	L-Lysine; L'	Lysine met	8	
156.0768	87.7	C00135	M+H[1+]	0	L-Histidine	Beta-Alani	8	
157.0801	88.5	C00135	M(C13)+H[	-1.00E-04	L-Histidine	Beta-Alani	8	
158.0377	101.0	C02617	M[1+]	9.00E-04		Xenobiotic	8	
161.1366	39.7	C01601	M(Cl37)+H	0.0014		Linoleate r	8	
178.024	46.3	C06196	M+H+Na[2	-4.00E-04	2'-Deoxyin	Purine me	8	
190.0894	238.8	C05548	M(Cl37)+H	4.00E-04	6-Acetami	Butanoate	8	
214.0512	196.1	C02712	M+Na[1+]	3.00E-04	N-Acetylm	Methionin	8	
214.5175	258.3	C00224	M+2H[2+]	3.00E-04	Adenylyl su	Methionin	8	
215.0193	144.9	C00224	M(C13)+2H	4.00E-04	Adenylyl su	Methionin	8	
217.1949	251.9	C00751	M+H+Na[2	0.001	Squalene;	Squalene a	8	
218.0625	235.3	C01233	M(Cl37)+H	0.0021	sn-glycero-	Glycerophe	8	
219.0265	55.1	C00031	M+K[1+]	3.00E-04	D-Glucose;	Galactose	8	
220.1373	135.6	pcrn	M(Cl37)+H	0.0014	propionyl-	Carnitine s	8	
223.0326	83.5	C06608	M+HCOON	-0.0016	Diethylpho	Parathio de	8	
223.1074	192.1	C01239	M(S34)+H[	-0.0016	N-Acetyl-b	Aspartate a	8	
231.0435	139.1	C01107	M(Cl37)+H	-9.00E-04	(R)-5-Phos	Squalene a	8	
233.0247	200.3	C00117	M(Cl37)+H	0.001	D-Ribose 5	Pentose pl	8	
233.0376	87.0	C13690	M[1+]	0.0018	Dopamine	Tyrosine m	8	
235.0205	146.9	C00121	M+HCOOK	-9.00E-04	D-Ribose	Purine me	8	
236.9568	246.1	C04309	M+K[1+]	0.0011	Phosphoenol-4-deoxy		8	
242.9928	145.6	C00075	M+2H[2+]	0.0013	UTP; Uridi	Pyrimidine	8	
243.0065	62.8	C02617	M+HCOOK	0.0011		Xenobiotic	8	
250.9626	261.1	C14873	M+HCOOK	4.00E-04		Xenobiotic	8	
252.9512	40.8	C00074	M+HCOOK	2.00E-04	Phosphoer	Purine me	8	
255.0283	257.2	C05576	M+HCOOK	0.0018	3,4-Dihydr	Tyrosine m	8	
260.0771	60.2	C00835	M+Na[1+]	0.0016	Sepiapterin		8	
270.0643	85.9	C01817	M(C13)+H[	-0.0015		Methionin	8	
273.075	64.9	C08261	M+HCOOK	0.0015		Linoleate r	8	
274.0921	66.5	C00559	M+Na[1+]	0.001	Deoxyader	Purine me	8	
280.0627	87.4	C08249	M(Cl37)+H	-7.00E-04	Pyrimidine	5'-deoxynu	8	
280.235	242.0	C06426	M(C13)+H[	-3.00E-04	(6Z,9Z,12Z	Fatty acid a	8	
285.0837	87.7	C01762	M+H[1+]	7.00E-04	Xanthosine	Purine me	8	
288.9839	168.7	C16193	M+H[1+]	4.00E-04	1,6-Naphthalenedisul		8	
296.0806	160.0	C11376	M+H+Na[2	-0.0023	SN38 glucu	Drug meta	8	
299.1365	54.4	C16609	M(Cl37)+H	-5.00E-04	Didemethy	Drug meta	8	
299.5874	171.7	C14855	M+H+Na[2	0.0028	4,5-Dihydr	Xenobiotic	8	
310.1128	66.0	C00270	M+H[1+]	-5.00E-04	N-Acetylne	Keratan su	8	
311.1168	66.5	C00270	M(C13)+H[	1.00E-04	N-Acetylne	Keratan su	8	
322.2432	52.2	C04742	M(C13)+H[	-0.0026	(15S)-15-H	Arachidoni	8	
328.0298	264.6	C01075	M+HCOON	-0.0011	N-Sulfo-D-glucosamin		8	
330.9972	34.5	C01143	M+Na[1+]	0.0017	(R)-5-Diph	Squalene a	8	
335.1982	47.8	C14762	M+K[1+]	3.00E-04	13(S)-HOD	Linoleate r	8	
337.2138	47.9	C00410	M+Na[1+]	-1.00E-04	Progesterc	C21-steroi	8	

339.0792	139.3	C14863	M+H+Na[2	-3.00E-04	2-(S-Glutat	Xenobiotic	8	
343.1035	246.3	C05403	M(Cl37)+H	-0.0016	3-Ketolact	Galactose	8	
344.017	116.2	C00352	M+HCOOK	0.0027	D-Glucosa	Aminosuga	8	
345.0186	125.1	C00345	M+HCOON	-7.00E-04	6-Phospho	Pentose ph	8	
347.0263	255.3	C00105	M+Na[1+]	0.0011	UMP; Urid	Pyrimidine	8	
349.1175	145.3	C04886	M+H+Na[2	1.00E-04	alpha-N-Ac	Aminosuga	8	
350.1197	72.4	C02538	M[1+]	9.00E-04	Estrone 3-	C21-steroi	8	
353.2668	42.9	C01530	M+HCOON	6.00E-04	Octadecan	Fatty acid	8	
355.0384	139.9	C06196	M+Na[1+]	-0.0031	2'-Deoxyin	Purine me	8	
364.0651	178.8	C00144	M+H[1+]	-2.00E-04	GMP; Guai	Purine me	8	
367.2454	51.8	C05472	M+H[1+]	-0.0025	Urocortiso	C21-steroi	8	
368.0376	127.6	C00942	M+Na[1+]	9.00E-04	3',5'-Cyclic	Purine me	8	
369.2245	65.1	C05956	M+H[1+]	-0.0027	Prostaglan	Prostaglan	8	
371.0361	240.8	C00130	M+Na[1+]	-3.00E-04	IMP; Inosir	Purine me	8	
379.0343	100.4	C16619	M[1+]	-9.00E-04	6-Thioguar	Drug meta	8	
403.0152	168.7	C00112	M[1+]	-0.003	CDP; Cytid	Pyrimidine	8	
406.9909	34.9	C01103	M+K[1+]	0.0024	Orotidine !	Pyrimidine	8	
409.5513	258.0	C00798	M+H+Na[2	-0.002	Formyl-Co.	Phytanic a	8	
442.2396	68.6	C05952	M(Cl37)+H	-0.0041	Leukotrien	Leukotrien	8	
446.5906	137.9	C16471	M+2H[2+]	-5.00E-04	5-Methyl-3	Geraniol d	8	
457.0732	136.1	C04823	M(S34)+H[	-0.0036	1-(5'-Phos	Purine me	8	
457.1671	65.8	C00143	M[1+]	-0.0039	5,10-Meth	Glycine, se	8	
470.1286	140.2	C05264	M(C13)+2H	-0.0033	(S)-Hydrox	Saturated	8	
484.9783	134.2	C00075	M+H[1+]	0.0025	UTP; Uridi	Pyrimidine	8	
490.0993	282.5	C14791	M+K[1+]	-0.0048	(1R)-Hydro	Xenobiotic	8	
662.8666	56.1	C01204	M(Cl37)+H	7.00E-04	myo-Inosit	Phosphatic	8	

Table S6c. Genes associated with metabolites in network for sex

Gene			Metabolite			
Node	Cluster	Name	Node	Cluster	m/z	RT
X6	1	*Il7r*	Y10283	1	376.4868	99.93324
			Y10438	1	382.2955	24.92213
			Y1046	1	126.9644	16.07659
			Y1052	1	126.9896	272.2422
			Y10858	1	398.0231	167.3736
			Y10995	1	403.2624	22.50289
			Y11058	1	405.5647	260.9067
			Y1129	1	129.1024	85.87494
			Y11392	1	419.1463	27.46814
			Y1158	1	130.0863	73.36979
			Y11861	1	438.2976	22.38412
			Y12077	1	448.3348	22.07258
			Y1213	1	131.0895	79.99142
			Y12361	1	462.2975	25.14307
			Y12765	1	482.3604	27.87635
			Y12784	1	483.3273	24.47989
			Y12785	1	483.3637	28.35409
			Y12801	1	484.3297	28.81949
			Y12939	1	492.5877	44.31243
			Y13014	1	496.3393	18.5974
			Y13038	1	497.3428	19.29923
			Y13080	1	499.3482	27.31861
			Y13083	1	499.8935	56.66061
			Y13199	1	506.3596	25.82906
			Y13317	1	512.3344	27.38073
			Y13349	1	513.8146	57.15943
			Y13371	1	515.3132	27.29436
			Y13649	1	529.3283	27.15776
			Y13756	1	534.8024	253.6498
			Y13816	1	538.3864	25.52527
			Y1398	1	136.0617	276.2446
			Y14085	1	551.3895	26.6441
			Y14089	1	551.6826	50.56125
			Y14124	1	553.4026	25.46548
			Y14140	1	554.2859	23.48491
			Y14568	1	578.0935	100.1297
			Y14599	1	579.6671	280.9908
			Y1475	1	138.0914	17.72665
			Y1492	1	138.9942	89.51049
			Y1567	1	140.9035	47.71077
			Y16094	1	658.5059	22.67706
			Y1635	1	142.5465	125.3949

			Y16634	1	695.0042	52.68062		
			Y16739	1	702.5011	36.48399		
			Y1706	1	144.0712	104.638		
			Y1710	1	144.0841	24.79292		
			Y17130	1	726.507	23.19143		
			Y17164	1	729.5253	23.5538		
			Y1748	1	145.0647	25.27962		
			Y17634	1	775.4861	51.68949		
			Y18100	1	833.5747	23.68405		
			Y1860	1	148.0427	42.31289		
			Y2023	1	152.0262	25.44742		
			Y2032	1	152.0818	227.8718		
			Y2074	1	153.1274	88.69866		
			Y2096	1	154.0229	43.69815		
			Y2171	1	156.0843	28.5813		
			Y2208	1	157.0608	59.93977		
			Y2247	1	158.0924	86.00326		
			Y2248	1	158.0998	26.14468		
			Y2297	1	159.0958	86.57591		
			Y2298	1	159.1031	26.59286		
			Y2537	1	165.0161	100.8787		
			Y2654	1	168.0235	57.13369		
			Y266	1	100.0474	193.9999		
			Y2739	1	170.0925	86.62112		
			Y2779	1	171.0958	87.48991		
			Y3023	1	177.0546	179.7436		
			Y3310	1	184.0731	22.80612		
			Y3396	1	186.1489	181.817		
			Y3517	1	189.491	127.2212		
			Y3972	1	200.201	15.86842		
			Y4029	1	202.0897	195.4012		
			Y4167	1	205.0041	92.30072		
			Y4172	1	205.0446	195.2685		
			Y4174	1	205.064	28.62135		
			Y4194	1	205.5069	100.9801		
			Y4281	1	207.1029	31.42062		
			Y4315	1	208.0136	42.82374		
			Y4445	1	211.1692	20.696		
			Y4552	1	214.1438	148.6297		
			Y4725	1	218.4691	115.2063		
			Y4898	1	222.0858	24.77441		
			Y4943	1	223.089	24.22012		
			Y4971	1	223.9886	142.5345		
			Y5523	1	236.1726	17.89089		
			Y5601	1	238.0991	21.67942		
			Y5615	1	238.5169	100.4796		
			Y5644	1	239.1044	18.85865		

			Y5678	1	240.0992	24.3727	
			Y5721	1	241.1019	43.81865	
			Y5846	1	244.1542	29.69017	
			Y6437	1	260.081	52.85636	
			Y6558	1	263.1224	171.6916	
			Y6752	1	268.6499	27.62935	
			Y6853	1	271.0814	87.7459	
			Y7094	1	277.1024	53.99619	
			Y7737	1	294.1269	23.00301	
			Y7881	1	299.0832	41.9153	
			Y7933	1	300.2612	20.94449	
			Y8306	1	311.4435	168.0933	
			Y90	1	90.94773	18.83546	
			Y9440	1	346.168	28.51799	
			Y9552	1	350.5718	111.3003	
			Y9855	1	361.2596	23.71245	
X8	2	*Junb*	Y10254	2	375.1927	259.421	
			Y10474	2	383.3668	22.35291	
			Y11333	2	416.7049	45.1428	
			Y15538	2	624.1863	289.1168	
			Y3083	2	178.1338	25.70619	
			Y4481	2	212.5048	101.3634	
			Y7005	2	274.9331	44.57567	
			Y7048	2	275.9607	113.489	
			Y7251	2	281.0956	180.0456	
			Y8057	2	304.1492	45.04476	
			Y844	2	120.9931	61.04313	
			Y946	2	123.9644	17.25538	
			Y9833	2	360.5051	286.5889	
X10	3	*Rela*	Y10	3	85.07789	92.13098	
X11	3	*Tgfb1*	Y10494	3	384.3105	21.78682	
X5	3	*Icam1*	Y10510	3	384.9697	56.78118	
			Y11130	3	408.6738	45.24104	
			Y1134	3	129.5067	84.14342	
			Y11454	3	422.0108	54.90533	
			Y11808	3	436.3053	22.57517	
			Y11823	3	436.9931	52.83432	
			Y11834	3	437.3087	22.53787	
			Y11968	3	443.797	49.34984	
			Y1210	3	131.0834	92.26991	
			Y12470	3	466.8448	50.39409	
			Y12516	3	469.9347	56.04543	
			Y12590	3	473.945	52.70681	
			Y12671	3	477.9384	52.08609	
			Y1268	3	132.9028	155.0183	
			Y13745	3	534.2627	274.5422	

			Y13769	3	535.528	275.2987	
			Y13871	3	540.7784	50.47484	
			Y1464	3	138.0275	88.08543	
			Y14687	3	585.3041	281.8741	
			Y14980	3	600.9555	52.69276	
			Y15368	3	616.7841	54.14073	
			Y15520	3	623.318	291.778	
			Y15619	3	628.534	47.79333	
			Y15680	3	632.4728	46.0048	
			Y15743	3	636.9169	52.63839	
			Y15946	3	649.05	280.7179	
			Y15990	3	651.4739	52.56287	
			Y17568	3	769.241	290.0577	
			Y1816	3	147.0441	45.24878	
			Y18294	3	911.2263	279.6863	
			Y1852	3	148.004	55.61717	
			Y1890	3	149.0009	55.33413	
			Y1891	3	149.0033	54.63775	
			Y1942	3	150.0081	55.68949	
			Y226	3	98.99845	123.3542	
			Y2759	3	170.9891	55.86412	
			Y2950	3	175.1116	20.96484	
			Y2993	3	176.1181	19.2021	
			Y30	3	87.00407	30.18397	
			Y3105	3	179.0025	196.0598	
			Y3334	3	185.0006	61.04575	
			Y3451	3	187.5299	88.95085	
			Y3688	3	193.1585	22.77386	
			Y379	3	104.1373	39.3869	
			Y3837	3	197.041	66.85533	
			Y3964	3	200.1017	26.97332	
			Y4125	3	204.0783	36.98534	
			Y4147	3	204.4759	92.22729	
			Y4593	3	215.1026	99.81239	
			Y4761	3	219.1056	120.6203	
			Y5255	3	230.5753	94.33825	
			Y5331	3	232.1542	30.66235	
			Y5377	3	233.1576	30.92025	
			Y5588	3	237.9731	56.65092	
			Y5886	3	245.1497	42.98322	
			Y6444	3	260.1855	26.82716	
			Y6485	3	261.1887	26.96614	
			Y6768	3	269.055	61.62239	
			Y6852	3	271.0753	57.76606	
			Y6929	3	273.0184	67.26879	
			Y7367	3	284.1852	25.61388	
			Y7448	3	286.201	25.13965	

			Y7857	3	298.2011	15.74219	
			Y8110	3	305.9592	56.44234	
			Y8316	3	311.9776	52.77652	
			Y8488	3	316.2478	23.11028	
			Y8519	3	317.2512	22.70967	
			Y8922	3	330.2273	26.69255	
			Y9337	3	343.1919	88.54802	
			Y9700	3	355.8874	52.37498	
X1	4	*Csrp1*	Y10084	4	369.4879	278.7509	
X12	4	*Tlr2*	Y10103	4	370.1961	129.0348	
X13	4	*Tmx1*	Y10120	4	370.832	159.8474	
X4	4	*Dusp4*	Y10126	4	370.9827	241.0619	
			Y1013	4	125.9643	16.55913	
			Y10149	4	371.3269	25.09635	
			Y10428	4	382.057	59.57613	
			Y1048	4	126.972	267.2128	
			Y10649	4	389.2684	22.34947	
			Y10660	4	390.0111	61.23281	
			Y10690	4	391.2838	22.49414	
			Y1074	4	127.5088	91.46904	
			Y10784	4	395.0374	99.51426	
			Y108	4	91.52385	262.4388	
			Y10837	4	397.1642	156.8443	
			Y1101	4	128.0805	183.9466	
			Y11134	4	408.7626	45.30835	
			Y11137	4	408.8532	34.95116	
			Y11164	4	410.0053	84.94139	
			Y11165	4	410.0378	63.23281	
			Y11166	4	410.0445	249.9055	
			Y11173	4	410.2239	79.43848	
			Y11249	4	413.2662	46.55564	
			Y11276	4	414.2694	41.9276	
			Y11307	4	415.5554	99.43411	
			Y11312	4	415.8858	67.26429	
			Y11331	4	416.5576	100.9075	
			Y11362	4	417.8919	46.52635	
			Y11386	4	418.9323	42.96596	
			Y11401	4	419.7665	25.67388	
			Y11444	4	421.727	271.8166	
			Y11508	4	424.7321	67.6543	
			Y11509	4	424.7322	59.90118	
			Y11557	4	426.9715	209.599	
			Y11617	4	429.0444	50.47498	
			Y11627	4	429.2401	45.90552	
			Y11647	4	430.2433	43.15558	
			Y11729	4	432.9443	86.52027	
			Y11797	4	435.8632	46.57246	

			Y1181	4	130.933	152.1117		
			Y11816	4	436.6877	47.93969		
			Y11897	4	440.3131	25.82009		
			Y120	4	92.0244	38.08873		
			Y12023	4	446.0546	77.70078		
			Y12044	4	446.9361	57.4595		
			Y12071	4	448.0519	59.22744		
			Y1208	4	131.0717	190.8622		
			Y12102	4	449.2559	73.42758		
			Y1220	4	131.481	79.62172		
			Y12216	4	455.2163	75.9816		
			Y1223	4	131.5169	99.16969		
			Y1233	4	132.0035	280.8324		
			Y1235	4	132.0186	100.692		
			Y12490	4	468.3891	22.1955		
			Y12504	4	469.0695	287.6715		
			Y12621	4	475.1542	273.5548		
			Y12629	4	475.8506	46.87498		
			Y12636	4	476.0891	58.76336		
			Y12669	4	477.8478	46.71385		
			Y12670	4	477.8806	79.21283		
			Y12713	4	479.7907	44.37429		
			Y1274	4	132.9696	28.73949		
			Y12741	4	481.0178	265.5959		
			Y12756	4	481.8254	50.88586		
			Y1281	4	133.0339	234.8419		
			Y12839	4	486.0649	100.1459		
			Y1286	4	133.0591	290.5833		
			Y12866	4	487.9118	92.78584		
			Y12871	4	488.1548	30.22335		
			Y12878	4	488.6936	47.61426		
			Y12930	4	492.0109	96.74792		
			Y13074	4	499.0206	254.8498		
			Y1309	4	133.9302	100.3393		
			Y13184	4	504.8915	43.22484		
			Y13198	4	506.2683	270.1276		
			Y13278	4	510.3914	26.07918		
			Y13318	4	512.3619	25.93146		
			Y13341	4	513.2648	69.47656		
			Y13418	4	518.3217	24.09908		
			Y13458	4	521.1029	210.463		
			Y13509	4	524.0561	256.6843		
			Y13558	4	526.0465	53.24017		
			Y13596	4	527.4221	280.8135		
			Y13661	4	530.2865	23.36146		
			Y13740	4	534.0492	259.2565		
			Y13759	4	534.8318	78.86228		

			Y13760	4	535.0534	98.68091	
			Y1389	4	136.0068	123.4426	
			Y1390	4	136.0143	38.42132	
			Y13919	4	544.0323	77.2798	
			Y14024	4	548.86	277.0326	
			Y14031	4	549.0882	238.9024	
			Y14037	4	549.3152	29.48616	
			Y14043	4	549.5896	239.4576	
			Y14064	4	550.5462	251.8997	
			Y14084	4	551.3121	26.98712	
			Y14177	4	556.6116	289.8099	
			Y142	4	93.5038	264.5704	
			Y1423	4	137.0157	91.1506	
			Y1428	4	137.0458	37.38619	
			Y14411	4	569.6843	266.6598	
			Y1465	4	138.0298	37.10379	
			Y1466	4	138.0429	37.16868	
			Y1468	4	138.049	38.72642	
			Y14700	4	586.5351	279.7139	
			Y14802	4	592.6732	47.49957	
			Y14807	4	592.8772	277.5878	
			Y14889	4	596.4599	52.68893	
			Y1502	4	139.0467	40.22838	
			Y15163	4	609.4007	30.18308	
			Y1519	4	139.527	104.6066	
			Y1537	4	140.0287	88.37082	
			Y15444	4	619.9725	294.3029	
			Y1557	4	140.4862	79.50191	
			Y1562	4	140.5222	100.0998	
			Y15751	4	637.4306	30.18691	
			Y15767	4	638.4338	29.50295	
			Y15926	4	647.947	56.30254	
			Y1611	4	141.9514	88.13797	
			Y16122	4	659.8608	63.53273	
			Y16198	4	664.0238	255.8471	
			Y1655	4	143.0339	209.2944	
			Y16571	4	689.9655	277.7323	
			Y16645	4	695.3393	268.7114	
			Y1680	4	143.9523	89.99791	
			Y1681	4	143.959	262.0107	
			Y16836	4	706.9675	57.80233	
			Y17128	4	726.4649	47.30281	
			Y17165	4	729.5889	22.50184	
			Y17182	4	731.1622	280.1064	
			Y17333	4	746.4888	47.62173	
			Y17403	4	753.1315	267.2639	
			Y1741	4	145.0351	279.6261	

			Y17442	4	757.6353	68.72231	
			Y17607	4	772.4924	47.47952	
			Y17629	4	774.5668	16.01578	
			Y17662	4	778.5671	23.80275	
			Y17685	4	780.5389	21.7149	
			Y17836	4	796.5454	29.77174	
			Y17860	4	798.6855	279.8663	
			Y17889	4	803.6034	19.32993	
			Y1797	4	146.1654	66.49199	
			Y17970	4	817.0432	54.27296	
			Y18012	4	822.6821	279.766	
			Y18027	4	824.4012	44.22741	
			Y18184	4	854.3739	47.92457	
			Y1864	4	148.0734	71.94971	
			Y1879	4	148.5237	91.52672	
			Y1881	4	148.5322	100.2032	
			Y1902	4	149.0339	102.9137	
			Y1911	4	149.0767	72.70358	
			Y1930	4	149.5342	99.96391	
			Y1943	4	150.0141	278.9721	
			Y1962	4	150.5286	99.28435	
			Y1987	4	151.0696	289.0153	
			Y2126	4	154.9669	167.9665	
			Y2226	4	157.5374	100.1742	
			Y2261	4	158.5355	100.4944	
			Y2263	4	158.5395	100.009	
			Y2289	4	159.0513	39.25249	
			Y2307	4	159.4734	106.012	
			Y2314	4	159.947	266.081	
			Y2326	4	160.0401	99.79173	
			Y2348	4	160.527	91.11677	
			Y2406	4	161.9801	103.5663	
			Y2420	4	162.0584	37.0079	
			Y2437	4	162.4805	79.7642	
			Y2485	4	163.5279	135.0972	
			Y2488	4	163.9401	94.84676	
			Y2507	4	164.0561	61.10343	
			Y2566	4	165.5339	89.09741	
			Y2572	4	165.9828	261.1149	
			Y2600	4	166.9545	16.12863	
			Y2616	4	167.0339	102.6781	
			Y2692	4	169.0454	100.9399	
			Y2712	4	169.5471	99.96524	
			Y2718	4	169.9825	69.48443	
			Y2767	4	171.0419	99.46879	
			Y2851	4	173.0301	280.5046	
			Y2876	4	173.916	94.79266	

			Y2882	4	173.9961	35.71882		
			Y2896	4	174.0759	25.69819		
			Y2958	4	175.1481	160.5813		
			Y3004	4	176.5091	191.691		
			Y303	4	102.013	281.9958		
			Y3056	4	177.9575	273.6649		
			Y308	4	102.0338	265.2065		
			Y3085	4	178.4631	285.4678		
			Y3189	4	180.9748	130.2291		
			Y3258	4	182.9617	259.4954		
			Y3265	4	182.9966	97.19262		
			Y336	4	103.0058	79.6643		
			Y3360	4	185.4959	107.1901		
			Y3375	4	185.9994	78.31618		
			Y3380	4	186.0505	77.78637		
			Y3387	4	186.0867	80.03524		
			Y3412	4	186.9563	165.9415		
			Y3480	4	188.5152	258.277		
			Y3544	4	190.0895	94.93539		
			Y3548	4	190.1072	28.60267		
			Y3565	4	190.925	79.42072		
			Y3630	4	192.0503	70.50035		
			Y3660	4	192.9748	119.5432		
			Y3700	4	193.521	166.7802		
			Y3739	4	194.5006	191.4774		
			Y3768	4	195.0877	22.47124		
			Y3804	4	196.0561	99.8197		
			Y3851	4	197.1285	183.0122		
			Y3857	4	197.5206	98.05829		
			Y3866	4	197.9259	102.4193		
			Y3877	4	198.0224	100.7061		
			Y3933	4	199.135	145.206		
			Y3952	4	199.9965	289.8378		
			Y3955	4	200.0253	86.08596		
			Y3977	4	200.5783	79.60808		
			Y4059	4	202.9753	34.56778		
			Y408	4	105.4921	267.839		
			Y4108	4	203.9733	104.7248		
			Y4162	4	204.9666	95.11373		
			Y4393	4	210.0913	176.7211		
			Y44	4	88.00425	260.5333		
			Y4412	4	210.8993	158.891		
			Y4415	4	210.9328	79.12319		
			Y4418	4	210.968	93.96143		
			Y4531	4	213.941	118.0033		
			Y4605	4	215.4192	79.06265		
			Y4619	4	215.9365	94.76308		

			Y4620	4	215.9575	119.3541		
			Y4734	4	218.8304	48.11931		
			Y475	4	108.9614	267.8826		
			Y4792	4	219.9312	79.01423		
			Y4796	4	219.9682	146.5627		
			Y4877	4	221.5608	157.0103		
			Y490	4	109.9786	254.9273		
			Y4919	4	222.7022	125.6792		
			Y4963	4	223.5634	100.6485		
			Y498	4	110.0293	34.98544		
			Y4988	4	224.0895	52.07219		
			Y500	4	110.035	37.73777		
			Y5112	4	226.9734	43.82616		
			Y516	4	111.0202	260.568		
			Y5177	4	228.1956	20.79958		
			Y5230	4	230.0372	170.3478		
			Y5254	4	230.539	169.5907		
			Y5277	4	231.0435	72.90972		
			Y5493	4	235.9621	271.8129		
			Y5539	4	236.9045	78.83957		
			Y5593	4	238.0378	66.42242		
			Y5603	4	238.1073	176.3285		
			Y5622	4	238.8941	158.3387		
			Y5666	4	240.0022	61.73243		
			Y582	4	112.9911	99.66457		
			Y5999	4	248.1738	29.08201		
			Y6145	4	252.1442	66.95881		
			Y6180	4	253.1476	66.02655		
			Y6307	4	256.9636	58.33634		
			Y6352	4	257.9934	44.84911		
			Y6354	4	258.0013	126.8078		
			Y6394	4	258.9961	44.51295		
			Y6425	4	259.957	98.43021		
			Y6507	4	261.9613	74.70085		
			Y6596	4	264.1263	31.20972		
			Y6629	4	265.1116	60.30374		
			Y6648	4	265.9269	279.9436		
			Y6662	4	266.115	60.97739		
			Y6674	4	266.5289	89.39073		
			Y6687	4	266.9282	24.82237		
			Y6707	4	267.1952	21.90616		
			Y6721	4	267.9305	44.41643		
			Y6769	4	269.0879	39.99815		
			Y6825	4	270.5227	128.3451		
			Y6872	4	271.2633	16.58802		
			Y6919	4	272.8549	93.96283		
			Y7237	4	280.9848	178.7362		

			Y7311	4	282.9732	79.13564	
			Y743	4	117.9698	78.12367	
			Y7468	4	287.0037	99.24938	
			Y751	4	118.0289	77.47648	
			Y7525	4	288.592	109.1143	
			Y7630	4	290.9345	94.00606	
			Y771	4	118.9206	97.19323	
			Y7779	4	295.9382	46.87892	
			Y7808	4	296.9231	96.99099	
			Y7843	4	298.0219	261.0673	
			Y7948	4	300.9835	79.25621	
			Y8000	4	302.1438	49.32215	
			Y8086	4	305.0678	252.5085	
			Y8141	4	306.8289	47.48479	
			Y8152	4	306.9979	279.6197	
			Y8156	4	307.0438	42.39798	
			Y8175	4	307.288	211.0052	
			Y8189	4	308.0406	44.39817	
			Y8190	4	308.047	41.15348	
			Y8219	4	309.042	41.265	
			Y825	4	120.0558	90.58705	
			Y8251	4	310.0453	41.3446	
			Y8352	4	312.9641	265.5101	
			Y8483	4	316.1773	182.2637	
			Y8531	4	317.9082	78.59706	
			Y8565	4	318.9051	78.73891	
			Y8580	4	319.0614	264.8417	
			Y86	4	90.52583	278.1456	
			Y8638	4	321.5374	167.9228	
			Y8754	4	325.6106	101.9765	
			Y8757	4	325.9713	279.6264	
			Y8782	4	326.6125	101.8812	
			Y882	4	122.0192	274.596	
			Y894	4	122.0714	61.00587	
			Y8964	4	331.5416	256.8696	
			Y901	4	122.5204	79.7897	
			Y9065	4	334.1886	43.56035	
			Y9296	4	342.0626	105.8434	
			Y9329	4	343.0659	99.90017	
			Y9345	4	343.2952	26.16488	
			Y9376	4	344.2431	26.47808	
			Y9394	4	344.9996	42.31	
			Y9429	4	346.0031	42.44711	
			Y943	4	123.9547	272.2297	
			Y9458	4	346.998	42.36824	
			Y9531	4	349.9759	285.4996	
			Y9587	4	351.8557	74.98555	

			Y961	4	124.0758	136.3834		
			Y9615	4	352.8533	77.99886		
			Y9635	4	353.4644	91.41775		
			Y9643	4	353.8976	49.2269		
			Y971	4	124.9564	15.45339		
			Y9756	4	358.0149	100.2397		
			Y9800	4	359.3156	153.7243		
			Y9815	4	360.0251	281.4394		
			Y9816	4	360.0367	98.03781		
X9	5	*Nos2*	Y1033	5	126.1026	175.7562		
			Y10419	5	381.8012	50.67341		
			Y11216	5	412.0754	197.8476		
			Y11255	5	413.5245	262.7069		
			Y11366	5	417.9911	52.01983		
			Y11400	5	419.7557	20.68503		
			Y11673	5	431.0978	196.2579		
			Y12488	5	468.3083	28.39677		
			Y12510	5	469.3119	28.52498		
			Y12579	5	473.1781	39.65741		
			Y13066	5	498.7914	50.67681		
			Y14424	5	570.3556	25.13504		
			Y14512	5	575.5015	24.21279		
			Y15099	5	606.3276	267.6007		
			Y15635	5	629.5344	274.6905		
			Y16876	5	708.9086	53.01125		
			Y17019	5	716.5177	23.28104		
			Y17060	5	719.7173	51.11218		
			Y17207	5	733.5506	19.05637		
			Y17282	5	741.5255	22.72703		
			Y17313	5	744.5851	23.35888		
			Y17454	5	758.571	16.77236		
			Y17904	5	806.5552	23.27711		
			Y18126	5	836.5407	25.77061		
			Y18364	5	1059.818	65.71175		
			Y1910	5	149.071	97.26097		
			Y2044	5	152.9032	80.08368		
			Y3033	5	177.095	95.67248		
			Y3461	5	188.0376	198.3443		
			Y4035	5	202.1439	97.8006		
			Y4079	5	203.0928	113.0356		
			Y5261	5	230.922	54.32682		
			Y5453	5	235.0387	283.5956		
			Y6020	5	249.0537	198.1782		
			Y6026	5	249.0975	100.795		
			Y6155	5	252.5518	79.23755		
			Y6281	5	256.1098	26.20299		
			Y6427	5	259.9737	84.80245		

			Y70	5	89.04233	78.44261		
			Y7209	5	280.0831	33.26092		
			Y7545	5	289.0403	47.93183		
			Y7755	5	295.0305	53.4093		
			Y7926	5	300.1207	66.17846		
			Y846	5	121.0148	195.7637		
			Y9714	5	356.2869	287.4308		
			Y995	5	125.0836	189.4071		
X3	6	*Dusp1*	Y10020	6	367.2675	19.68427		
X7	6	*Jun*	Y10101	6	370.0988	254.1566		
			Y1019	6	126.0186	25.27579		
			Y1055	6	127.0123	24.80024		
			Y1058	6	127.0215	44.60402		
			Y1064	6	127.0542	27.86527		
			Y10747	6	393.7804	50.06955		
			Y10801	6	395.7779	50.19767		
			Y10815	6	396.3104	21.76523		
			Y1084	6	128.0157	25.59723		
			Y10907	6	400.0291	53.36189		
			Y1098	6	128.062	24.3269		
			Y11138	6	408.886	50.4802		
			Y11158	6	409.7541	39.61857		
			Y11222	6	412.3209	21.41015		
			Y1123	6	129.0699	24.15459		
			Y1140	6	129.5945	70.33764		
			Y11420	6	420.3186	273.5926		
			Y11433	6	421.019	54.78746		
			Y11469	6	422.7615	258.073		
			Y11472	6	422.8628	50.6333		
			Y11763	6	434.2878	21.82674		
			Y11802	6	435.989	52.73972		
			Y11847	6	437.9855	52.74366		
			Y11862	6	438.3211	22.24044		
			Y11888	6	439.3011	24.4346		
			Y12031	6	446.3261	21.61518		
			Y12058	6	447.3295	21.68301		
			Y12078	6	448.3418	17.23795		
			Y12105	6	449.3452	20.55744		
			Y12122	6	450.3482	21.54367		
			Y12141	6	451.3609	21.61479		
			Y12197	6	454.2926	24.26109		
			Y12218	6	455.2961	25.08793		
			Y12402	6	464.3134	23.40484		
			Y12414	6	464.8478	50.298		
			Y12429	6	465.3402	22.09198		
			Y12460	6	466.3524	21.8812		
			Y12581	6	473.3453	21.59564		

			Y12598	6	474.3576	21.52924		
			Y12624	6	475.3611	21.64843		
			Y12632	6	475.9419	52.69231		
			Y12642	6	476.3731	21.50824		
			Y12667	6	477.7501	49.85715		
			Y12675	6	478.2926	24.78169		
			Y12705	6	479.296	25.15684		
			Y12724	6	480.3084	23.79256		
			Y12735	6	480.8214	50.46283		
			Y12751	6	481.3481	24.50425		
			Y12775	6	482.8185	50.63636		
			Y12895	6	489.9306	58.73067		
			Y12962	6	493.7241	49.42422		
			Y12975	6	494.3241	26.49185		
			Y12996	6	495.3272	27.29103		
			Y13056	6	498.3456	24.03494		
			Y13303	6	511.694	49.80266		
			Y13350	6	513.898	52.59334		
			Y13352	6	514.1914	24.29014		
			Y13605	6	527.6692	50.21003		
			Y13620	6	528.2978	24.98499		
			Y13810	6	538.1989	101.0571		
			Y13890	6	542.5701	47.10958		
			Y13911	6	543.3261	27.21269		
			Y14038	6	549.3745	23.19512		
			Y14041	6	549.4871	23.16487		
			Y1422	6	137.0153	25.03309		
			Y14273	6	562.3496	27.12025		
			Y14458	6	572.3707	24.40532		
			Y14556	6	577.4807	20.56526		
			Y1460	6	138.0105	25.15466		
			Y14930	6	598.736	50.77622		
			Y1494	6	139.0123	25.31364		
			Y1500	6	139.0309	26.62077		
			Y1529	6	140.0076	25.94875		
			Y15639	6	629.8173	50.93501		
			Y15672	6	631.9737	58.44312		
			Y15778	6	638.9112	52.71288		
			Y1582	6	141.0102	24.54165		
			Y1616	6	142.0136	25.75599		
			Y16339	6	674.8736	52.37496		
			Y16369	6	676.8662	53.34575		
			Y16435	6	681.4428	52.99549		
			Y16552	6	688.9693	293.2237		
			Y1666	6	143.0855	23.02831		
			Y16726	6	701.56	22.94954		
			Y16743	6	702.5577	35.05751		

			Y1703	6	144.057	24.83595	
			Y17047	6	718.5332	34.78555	
			Y17106	6	723.9729	52.7141	
			Y1712	6	144.089	25.38193	
			Y1713	6	144.0934	24.52875	
			Y17224	6	734.5753	21.10892	
			Y17455	6	758.5767	285.0754	
			Y17495	6	761.9348	52.63417	
			Y17765	6	790.4084	286.0913	
			Y17878	6	801.8816	52.66441	
			Y1790	6	146.0923	16.39119	
			Y17937	6	812.5363	28.31364	
			Y17957	6	814.5574	24.22391	
			Y18023	6	823.8646	53.18251	
			Y18129	6	837.6206	23.69396	
			Y18214	6	860.6109	23.67285	
			Y18271	6	886.9429	52.58784	
			Y2030	6	152.0623	31.74855	
			Y2066	6	153.0699	26.01216	
			Y2109	6	154.0777	25.74511	
			Y2146	6	155.0854	21.70136	
			Y2170	6	156.0808	26.7369	
			Y227	6	98.99965	23.85995	
			Y2456	6	163.0309	24.94946	
			Y2504	6	164.0342	24.57938	
			Y2535	6	165.0102	27.47199	
			Y2538	6	165.028	24.87428	
			Y254	6	100.003	25.31962	
			Y2542	6	165.0466	25.5047	
			Y2575	6	166.0135	25.1057	
			Y2579	6	166.0315	25.65255	
			Y2583	6	166.0499	26.32747	
			Y2608	6	167.0073	24.77147	
			Y2617	6	167.0436	24.69195	
			Y2651	6	168.0106	24.78865	
			Y2665	6	168.0807	26.87643	
			Y2775	6	171.0804	24.90927	
			Y2819	6	172.0883	24.64441	
			Y282	6	100.9967	24.74502	
			Y3019	6	177.044	58.61296	
			Y312	6	102.0461	48.12381	
			Y3120	6	179.0626	25.29742	
			Y3167	6	180.0653	76.66213	
			Y3169	6	180.0655	25.84273	
			Y3204	6	181.0595	25.58344	
			Y3205	6	181.0606	210.9065	
			Y3238	6	182.0626	24.58256	

			Y3371	6	185.9597	52.64886	
			Y3456	6	187.9579	52.77903	
			Y3497	6	189.0466	29.44332	
			Y3535	6	190.0499	29.33943	
			Y3585	6	191.0439	27.56357	
			Y3589	6	191.0622	48.19288	
			Y3629	6	192.0469	25.55964	
			Y3638	6	192.0743	80.19313	
			Y3661	6	192.9806	37.59964	
			Y3793	6	195.9629	15.2705	
			Y4223	6	206.073	27.39555	
			Y4275	6	207.0762	24.8848	
			Y4355	6	209.0541	25.71219	
			Y4357	6	209.0635	24.6188	
			Y4390	6	210.0581	33.79389	
			Y4760	6	219.0971	53.80007	
			Y4811	6	220.0888	25.74679	
			Y4812	6	220.0928	76.20052	
			Y4855	6	221.0925	30.71676	
			Y4857	6	221.0957	25.26249	
			Y4899	6	222.0955	25.16509	
			Y4984	6	224.0738	29.96848	
			Y4987	6	224.0834	24.81989	
			Y5037	6	225.0869	26.56602	
			Y5039	6	225.0908	22.4223	
			Y5040	6	225.0909	169.1536	
			Y5076	6	226.0383	196.7848	
			Y5084	6	226.0816	52.0817	
			Y5085	6	226.0898	25.44099	
			Y5128	6	227.0837	25.28247	
			Y5140	6	227.2003	22.1175	
			Y5511	6	236.084	22.09868	
			Y5641	6	239.0913	36.29788	
			Y5679	6	240.0995	111.3863	
			Y5748	6	242.0789	40.86244	
			Y5749	6	242.079	89.51029	
			Y5914	6	246.0649	57.00323	
			Y5961	6	247.1652	60.20475	
			Y6210	6	254.0941	25.64987	
			Y6243	6	255.0975	43.60466	
			Y6277	6	256.0911	25.19223	
			Y6324	6	257.0945	25.811	
			Y6451	6	260.6388	27.60453	
			Y6515	6	262.0394	44.17289	
			Y652	6	115.0542	226.9883	
			Y6520	6	262.0781	52.84148	
			Y6668	6	266.186	23.01319	

			Y6776	6	269.1282	23.54331	
			Y68	6	89.03864	29.16497	
			Y7100	6	277.1536	27.34903	
			Y7437	6	286.0956	132.0263	
			Y7454	6	286.5739	248.3369	
			Y7506	6	288.0723	28.47584	
			Y7560	6	289.22	24.58457	
			Y7612	6	290.6093	25.27804	
			Y78	6	90.04197	25.0188	
			Y80	6	90.04643	27.64032	
			Y8066	6	304.4793	243.4406	
			Y807	6	119.9571	260.2042	
			Y8283	6	310.9742	52.4849	
			Y8314	6	311.9058	53.32578	
			Y8329	6	312.129	68.87417	
			Y8374	6	313.2734	22.3749	
			Y8403	6	314.1031	280.9967	
			Y8446	6	315.1337	24.14131	
			Y9274	6	341.1532	21.80288	
			Y9445	6	346.2588	26.52515	
			Y9466	6	347.0504	65.02424	
			Y9483	6	348.0445	65.06345	
			Y9498	6	348.9302	53.39291	
			Y9561	6	350.9279	52.6408	
			Y9683	6	355.0576	58.98211	
			Y977	6	125.0152	26.69958	
X14	7	*Tnfaip6*	Y10009	7	367.0439	119.47	
			Y10238	7	374.5039	125.7008	
			Y10251	7	375.0414	167.2386	
			Y10503	7	384.7565	47.42796	
			Y1103	7	128.107	248.6226	
			Y11360	7	417.7938	48.85035	
			Y11370	7	418.1564	70.94728	
			Y11770	7	434.6907	47.68014	
			Y12128	7	450.6647	47.51171	
			Y12137	7	451.0008	168.5955	
			Y12260	7	457.2715	47.79224	
			Y12338	7	460.9671	117.3736	
			Y13087	7	499.9716	57.43728	
			Y13119	7	500.9917	168.9207	
			Y13239	7	508.1107	115.2816	
			Y13568	7	526.5585	247.2072	
			Y13582	7	527.0373	118.0467	
			Y13591	7	527.2905	22.90744	
			Y14143	7	554.6373	47.14885	
			Y1447	7	137.5153	34.45787	
			Y14643	7	582.863	57.31501	

			Y14943	7	599.4412	30.44216		
			Y1499	7	139.0309	125.5059		
			Y15360	7	616.4565	25.55704		
			Y15647	7	630.4245	43.96991		
			Y15924	7	647.8786	55.37536		
			Y15937	7	648.5437	46.9521		
			Y16070	7	656.576	47.04821		
			Y16300	7	671.1225	142.6501		
			Y16332	7	674.5371	43.6598		
			Y16642	7	695.1335	122.6384		
			Y16799	7	705.3708	278.0121		
			Y17086	7	721.7297	108.2309		
			Y17253	7	738.1741	232.4997		
			Y17851	7	797.8472	57.05617		
			Y1873	7	148.1159	87.07595		
			Y2051	7	152.9884	121.4666		
			Y2219	7	157.1084	86.31781		
			Y2264	7	158.8438	42.14462		
			Y24	7	86.08123	93.87395		
			Y2433	7	162.1399	39.62915		
			Y2503	7	164.0294	60.73642		
			Y2613	7	167.0261	27.60495		
			Y2639	7	167.505	123.0597		
			Y28	7	86.98997	35.81534		
			Y2953	7	175.1189	85.09523		
			Y2980	7	176.0658	52.22134		
			Y2982	7	176.0706	247.1998		
			Y2995	7	176.1224	83.77834		
			Y3039	7	177.1193	87.25421		
			Y3099	7	178.9743	127.6552		
			Y3231	7	181.995	126.5029		
			Y3274	7	183.0529	88.14513		
			Y337	7	103.0208	35.48527		
			Y3633	7	192.0573	25.00441		
			Y3662	7	193.0019	268.3292		
			Y3680	7	193.0971	190.1219		
			Y3874	7	198.0013	127.5366		
			Y3989	7	201.0537	58.45911		
			Y4326	7	208.0966	24.24639		
			Y4358	7	209.0728	50.82281		
			Y4387	7	210.0118	42.76641		
			Y4404	7	210.5149	259.0948		
			Y4451	7	211.5172	117.9416		
			Y4861	7	221.1104	286.8162		
			Y4890	7	222.0228	262.984		
			Y5271	7	230.9871	118.0849		
			Y5543	7	236.9411	125.5968		

			Y5600	7	238.0929	27.02093	
			Y561	7	112.087	83.83632	
			Y5677	7	240.0902	25.27284	
			Y5706	7	240.9885	121.4821	
			Y5734	7	241.5738	234.4836	
			Y5754	7	242.1029	25.06899	
			Y5986	7	248.032	81.66939	
			Y607	7	113.0904	86.13736	
			Y6075	7	250.534	90.31311	
			Y6085	7	250.9626	261.1085	
			Y6134	7	252.0482	123.6874	
			Y6581	7	263.9991	168.0539	
			Y6689	7	266.9686	113.3854	
			Y6839	7	270.9776	57.40467	
			Y6876	7	271.5576	101.7148	
			Y7213	7	280.154	28.31856	
			Y7564	7	289.5264	113.8928	
			Y7836	7	297.9038	123.7712	
			Y83	7	90.09139	205.7319	
			Y8466	7	315.9145	125.4966	
			Y8587	7	319.2245	47.20437	
			Y8736	7	325.043	117.5995	
			Y8760	7	326.0215	84.56632	
			Y9462	7	347.0251	123.4915	
			Y9506	7	349.0346	167.5701	
			Y9571	7	351.0777	123.8733	
			Y9680	7	355.0384	139.8719	
			Y9693	7	355.5301	126.2584	
X15	8	*Tnfrsf9*	Y10026	8	367.5359	127.3357	
			Y10042	8	368.0376	127.5777	
			Y10044	8	368.0602	183.4529	
			Y10113	8	370.5319	129.4792	
			Y10184	8	372.6074	263.6614	
			Y10329	8	378.5207	128.515	
			Y10347	8	379.0216	128.109	
			Y10348	8	379.0343	100.3755	
			Y10359	8	379.536	100.0636	
			Y10386	8	380.6724	45.75095	
			Y10400	8	380.9844	261.756	
			Y10456	8	383.0389	168.7127	
			Y10476	8	383.5403	168.9596	
			Y10560	8	386.5095	127.1686	
			Y1059	8	127.0253	52.14304	
			Y10753	8	394.0341	100.8421	
			Y10811	8	396.1201	100.3123	
			Y10902	8	399.9577	262.4209	
			Y10955	8	402.0121	168.3933	

			Y10972	8	402.5138	168.3141		
			Y11290	8	414.9432	48.47144		
			Y11611	8	428.9612	42.28807		
			Y11664	8	431.0025	260.8875		
			Y11757	8	434.0834	141.4419		
			Y11825	8	437.0763	99.78134		
			Y11837	8	437.5778	100.2883		
			Y11853	8	438.0797	99.93758		
			Y119	8	92.01652	65.77824		
			Y11912	8	441.0686	100.2898		
			Y11942	8	442.708	43.97934		
			Y12274	8	458.1619	75.90782		
			Y1306	8	133.5612	61.38144		
			Y13130	8	502.0181	288.7051		
			Y1321	8	134.06	228.1295		
			Y13312	8	512.0436	126.8608		
			Y13340	8	513.2383	144.7699		
			Y13479	8	522.6786	44.81973		
			Y138	8	93.04477	85.91488		
			Y14150	8	554.9518	290.4433		
			Y14325	8	565.6298	101.4229		
			Y14333	8	566.1284	100.5184		
			Y14591	8	579.0758	131.3159		
			Y14704	8	586.6387	47.57907		
			Y1514	8	139.1117	22.76206		
			Y15477	8	621.1683	167.7851		
			Y15606	8	627.824	289.5874		
			Y15905	8	646.45	44.67564		
			Y1612	8	141.9587	21.14047		
			Y16210	8	664.5166	46.23185		
			Y16507	8	685.1244	99.9046		
			Y16929	8	712.1111	168.5598		
			Y17026	8	716.7184	171.9486		
			Y17142	8	727.1224	169.226		
			Y17194	8	732.4029	44.05633		
			Y17213	8	734.0655	124.6709		
			Y17757	8	789.6188	25.10327		
			Y1856	8	148.0276	47.16678		
			Y1900	8	149.0263	54.0224		
			Y2054	8	153.001	121.4282		
			Y2169	8	156.0768	87.73439		
			Y2191	8	156.9695	177.6979		
			Y2212	8	157.0738	89.78477		
			Y2214	8	157.0801	88.50503		
			Y2311	8	159.5339	99.87311		
			Y2341	8	160.1332	35.88149		
			Y2387	8	161.1303	35.47339		

			Y2390	8	161.1366	39.74627	
			Y243	8	99.51222	259.6991	
			Y2466	8	163.0714	72.80935	
			Y2555	8	165.1198	45.17086	
			Y2560	8	165.3505	101.7146	
			Y2697	8	169.0844	32.18229	
			Y2733	8	170.0475	100.1474	
			Y2745	8	170.5151	37.4792	
			Y291	8	101.0599	33.48412	
			Y2981	8	176.0705	26.7453	
			Y3063	8	178.024	46.30275	
			Y317	8	102.0914	46.44104	
			Y3620	8	191.9916	102.5628	
			Y3627	8	192.0397	45.19008	
			Y3757	8	195.0299	138.2702	
			Y3794	8	195.9681	256.9792	
			Y3822	8	196.857	45.84932	
			Y3878	8	198.0417	101.6821	
			Y3960	8	200.0684	44.30215	
			Y4007	8	201.5097	168.1027	
			Y4266	8	207.0289	116.6936	
			Y4271	8	207.0431	104.2926	
			Y4318	8	208.0417	46.79378	
			Y4389	8	210.0503	47.02396	
			Y4421	8	211.0168	171.7123	
			Y4547	8	214.1071	26.63012	
			Y4618	8	215.927	270.5959	
			Y4753	8	219.0417	100.1297	
			Y4784	8	219.5434	99.91241	
			Y4807	8	220.044	100.1434	
			Y4823	8	220.1373	135.6441	
			Y4849	8	221.0384	81.93908	
			Y4872	8	221.2264	185.4001	
			Y4913	8	222.5247	168.4916	
			Y4934	8	223.0254	168.8391	
			Y4946	8	223.1074	192.0613	
			Y497	8	110.0271	65.27735	
			Y5026	8	225.0355	58.30427	
			Y5029	8	225.0472	54.43594	
			Y5053	8	225.3789	160.1166	
			Y5074	8	226.0241	45.064	
			Y5075	8	226.0379	57.27588	
			Y5099	8	226.4937	261.518	
			Y5105	8	226.5586	99.23449	
			Y518	8	111.0305	66.53406	
			Y5182	8	228.4642	113.4397	
			Y5198	8	229.0099	100.0738	

			Y5250	8	230.3558	256.6123	
			Y528	8	111.0747	88.9413	
			Y5297	8	231.3824	159.1319	
			Y5315	8	232.0477	162.6578	
			Y5386	8	233.5311	101.1799	
			Y5544	8	236.9568	246.1465	
			Y5591	8	238.0154	100.1911	
			Y5610	8	238.2246	184.7756	
			Y5665	8	239.9955	16.903	
			Y5855	8	244.5073	99.39951	
			Y6293	8	256.5254	128.0373	
			Y6353	8	257.995	123.9675	
			Y6397	8	259.0211	229.2275	
			Y6671	8	266.3584	128.7366	
			Y6722	8	267.9509	112.784	
			Y6875	8	271.5217	260.9921	
			Y6935	8	273.075	64.93994	
			Y696	8	116.0707	70.24569	
			Y7076	8	276.7822	44.07644	
			Y7084	8	276.9626	130.7108	
			Y7117	8	277.8826	260.2738	
			Y7169	8	279.0934	167.5688	
			Y7177	8	279.1919	52.54849	
			Y7218	8	280.235	241.9822	
			Y724	8	117.0659	59.44788	
			Y728	8	117.074	68.30725	
			Y734	8	117.5043	80.50731	
			Y7398	8	285.0837	87.67701	
			Y7536	8	288.9839	168.6639	
			Y7565	8	289.5514	100.1777	
			Y7652	8	291.1932	49.62151	
			Y773	8	118.9674	265.6782	
			Y7783	8	296.0092	57.40424	
			Y7848	8	298.0528	120.302	
			Y7892	8	299.2003	22.99185	
			Y7969	8	301.1734	57.74684	
			Y8010	8	302.5745	155.825	
			Y8050	8	304.0191	118.6645	
			Y8126	8	306.1297	78.26124	
			Y8201	8	308.5241	100.7706	
			Y8272	8	310.5014	127.1317	
			Y8380	8	313.6705	103.5768	
			Y8762	8	326.0462	119.5602	
			Y8764	8	326.0636	141.1938	
			Y8813	8	327.2524	281.9462	
			Y8831	8	327.9876	128.8241	
			Y8917	8	330.0609	167.1546	

			Y8961	8	331.2841	201.6591	
			Y9183	8	338.007	261.3341	
			Y9242	8	340.0807	264.6369	
			Y9290	8	341.8632	44.21269	
			Y9395	8	345.0186	125.0858	
			Y9450	8	346.7196	44.00905	
			Y9463	8	347.0263	255.2723	
			Y9516	8	349.1175	145.3455	
			Y9569	8	351.059	133.6955	
			Y9583	8	351.5626	273.2877	
			Y9633	8	353.2668	42.94601	
			Y9751	8	357.8761	43.62716	
			Y9802	8	359.5472	128.3116	
			Y9817	8	360.0482	128.4016	
			Y9915	8	363.9928	116.1151	
			Y9922	8	364.0651	178.7949	
			Y9993	8	366.5668	100.3351	
X2	9	*Csrp3*	Y10267	9	375.9075	46.50003	
			Y10613	9	388.077	99.89375	
			Y11123	9	408.2353	60.31134	
			Y12193	9	454.0403	50.74845	
			Y13001	9	495.62	100.8871	
			Y13149	9	502.7857	51.16946	
			Y13150	9	502.7917	49.13369	
			Y13468	9	522.0635	156.8124	
			Y13683	9	531.4444	16.9901	
			Y13827	9	538.7993	48.09945	
			Y1395	9	136.0337	49.59869	
			Y13984	9	546.7939	287.6285	
			Y14000	9	547.5124	287.621	
			Y14091	9	551.7865	289.1869	
			Y14125	9	553.4516	53.97354	
			Y14914	9	597.6719	293.2437	
			Y14964	9	600.3376	61.04445	
			Y15044	9	604.6954	100.8706	
			Y15129	9	607.9719	54.72303	
			Y1515	9	139.1117	255.4218	
			Y15583	9	626.6077	44.03543	
			Y15837	9	641.5887	49.71768	
			Y16009	9	652.8239	55.16306	
			Y16318	9	672.78	56.02722	
			Y16360	9	676.4573	46.67034	
			Y16478	9	683.8412	268.1632	
			Y16556	9	689.053	280.9787	
			Y16609	9	692.6094	47.21806	
			Y17143	9	727.3355	72.21611	
			Y17248	9	737.3807	52.43178	

			Y17314	9	744.6243	47.98665	
			Y17420	9	756.424	46.98924	
			Y17743	9	788.3845	47.40718	
			Y17944	9	813.5192	22.29096	
			Y18116	9	835.1949	100.2964	
			Y18137	9	839.5653	26.55362	
			Y18275	9	888.5116	280.7551	
			Y1850	9	147.9897	267.5457	
			Y2528	9	164.5445	64.25696	
			Y253	9	99.99047	78.85809	
			Y2725	9	170.0092	113.4121	
			Y2959	9	175.3386	120.6126	
			Y3092	9	178.5524	101.3141	
			Y3750	9	194.967	38.75474	
			Y3875	9	198.0075	27.39764	
			Y3984	9	200.9921	72.51728	
			Y4436	9	211.0966	184.8118	
			Y4754	9	219.0475	60.82624	
			Y479	9	109.0285	86.7265	
			Y5113	9	226.9857	49.08068	
			Y5559	9	237.0423	84.79311	
			Y6552	9	263.0695	29.36419	
			Y6660	9	266.0631	62.88154	
			Y7181	9	279.3583	112.6436	
			Y7716	9	293.1456	72.2762	
			Y7729	9	293.9227	100.7191	
			Y8167	9	307.1827	210.8505	
			Y8661	9	322.5193	181.5506	
			Y9237	9	340.0245	61.48075	
			Y9270	9	341.0669	60.0175	
			Y9674	9	354.9413	44.11437	
			Y9767	9	358.2437	22.03736	
			Y9897	9	363.0911	248.2127	

Table S6d. Metabolite tentative annotation in sex network								
m.z	id	match_for	mz_differe	name	pathway	rt	community	
89.0423	C02218	M(C13)+H[	-4.00E-04	2-Aminoac	Glycine, se	78.44261	5	
92.0165	C00322	M+H+Na[2	-4.00E-04	2-Oxoadip	Tryptopha	65.77824	8	
92.0244	C03012	M+H+Na[2	-1.00E-04	Naphthale	Xenobiotic	38.08873	4	
93.0448	C00588	M+2H[2+]	6.00E-04	Choline ph	Glycosphir	85.91488	8	
102.0914	C00576	M[1+]	-5.00E-04	Betaine ald	Glycine, se	46.44104	8	
103.0208	C16355	M+H+Na[2	5.00E-04		Caffeine m	35.48527	7	
109.0285	C00472	M+H[1+]	1.00E-04	p-Benzoquinone; Quir		86.7265	9	
110.0271	C00257	M+H+Na[2	-3.00E-04	D-Gluconic	Pentose ph	65.27735	8	
110.035	C12205	M+H+Na[2	-1.00E-04	5-Hydroxyconiferyl al		37.73777	4	
112.087	C00388	M+H[1+]	1.00E-04	1H-Imidaz	Histidine m	83.83632	7	
113.0904	C00388	M(C13)+H[	1.00E-04	1H-Imidaz	Histidine m	86.13736	7	
115.0542	C00881	M(C13)+2H	-1.00E-04	Deoxycytic	Pyrimidine	226.9883	6	
116.0707	C00148	M+H[1+]	1.00E-04	L-Proline;	Arginine ar	70.24569	8	
120.0558	C00581	M(S34)+H[	-0.0011	Guanidino	Glycine, se	90.58705	4	
125.0152	C00245	M[1+]	5.00E-04	Taurine; 2-	Methionin	26.69958	6	
126.0186	C01107	M+H+Na[2	4.00E-04	(R)-5-Phos	Squalene a	25.27579	6	
126.1026	C05127	M+H[1+]	0	N-Methylh	Histidine m	175.7562	5	
127.0253	C00245	M(C13)+H[	-1.00E-04	Taurine; 2-	Methionin	52.14304	8	
127.0542	C07535	M+2H[2+]	0	Benzpyren	Xenobiotic	27.86527	6	
128.062	C00829	M[1+]	-6.00E-04	Naphthale	Xenobiotic	24.3269	6	
129.0699	C00829	M+H[1+]	0	Naphthale	Xenobiotic	24.15459	6	
130.0863	C00408	M+H[1+]	0	L-Pipecola	Lysine met	73.36979	1	
132.0035	C16196	M+H+Na[2	6.00E-04	1,2-Dihydroxynaphtha		280.8324	4	
133.0339	C02362	M(C13)+H[	0.0013		Alanine an	234.8419	4	
136.0143	C05699	M+2H[2+]	0.0011	Selenocyst	Selenoami	38.42132	4	
136.0617	C00147	M+H[1+]	-1.00E-04	Adenine; 6	Arginine ar	276.2446	1	
137.0458	C00155	M(C13)+H[	-3.00E-04	L-Homocys	Glycine, se	37.38619	4	
138.049	C00262	M(C13)+H[	-2.00E-04	Hypoxanth	Purine met	38.72642	4	
138.0914	C00483	M+H[1+]	0	Tyramine;	Tyrosine m	17.72665	1	
139.0467	C16622	M+Na[1+]	-0.0012	N,N'-Diace	Drug meta	40.22838	4	
139.1117	C16300	M+2H[2+]	0	Stearidoni	Omega-3 f	22.76206	8	
139.1117	C16300	M+2H[2+]	0	Stearidoni	Omega-3 f	255.4218	9	
140.9035	C00023	M+HCOOK	0	Iron	Porphyrin	47.71077	1	
143.0339	C15587	M+Na[1+]	0.001	Purine		209.2944	4	
143.9523	C01755	M+HCOOK	7.00E-04	Thiocyana	Methionin	89.99791	4	
144.057	C11713	M[1+]	-5.00E-04	beta-Naph	Xenobiotic	24.83595	6	
146.0923	C01035	M+H[1+]	-1.00E-04	4-Guanidir	Aspartate	16.39119	6	
148.004	C00245	M+Na[1+]	0	Taurine; 2-	Methionin	55.61717	3	
148.0427	C00940	M(Cl37)+H	7.00E-04	2-Oxogluta	Alanine an	42.31289	1	
148.0734	C00418	M[1+]	-2.00E-04	(R)-Mevalo	Squalene a	71.94971	4	
148.1159	C00047	M(C13)+H[	-3.00E-04	L-Lysine; L	Lysine met	87.07595	7	
149.0009	C00090	M+K[1+]	0.0013	Catechol; 1,2-Benzen		55.33413	3	
149.0033	C01218	M+H+Na[2	5.00E-04	6-Phospho	Pentose ph	54.63775	3	
149.0263	C00026	M(Cl37)+H	3.00E-04	2-Oxogluta	Valine, leu	54.0224	8	

149.071	C00064	M(S34)+H[	-0.0012	L-Glutamir	Purine me	97.26097	5	
150.0081	C07643	M+H+Na[2	0	4-Hydroxy	Drug meta	55.68949	3	
150.5286	C08249	M+H+Na[2	9.00E-04	Pyrimidine	5'-deoxynu	99.28435	4	
152.0623	C00121	M(C13)+H[	-0.0012	D-Ribose	Purine me	31.74855	6	
152.9032	C06790	M+Na[1+]	-5.00E-04	Trichloroe	Xenobiotic	80.08368	5	
153.0699	C02646	M(S34)+H[	-0.0013	4-Coumaryl alcohol; 4		26.01216	6	
153.1274	C00219	M+2H[2+]	0	(5Z,8Z,11Z	Prostaglan	88.69866	1	
154.0777	C00379	M(C13)+H[	-0.0015	Xylitol	Pentose ar	25.74511	6	
156.0768	C00135	M+H[1+]	0	L-Histidine	Beta-Alani	87.73439	8	
156.0808	C03758	M(S34)+H[	-0.0013	Dopamine	Tyrosine m	26.7369	6	
156.0843	C03758	M(Cl37)+H	8.00E-04	Dopamine	Tyrosine m	28.5813	1	
157.0608	C03406	M+H+Na[2	0.0012	N-(L-Argini	Arginine ar	59.93977	1	
157.0801	C00135	M(C13)+H[	-1.00E-04	L-Histidine	Beta-Alani	88.50503	8	
158.0998	CE5653	M(C13)+2H	6.00E-04	4-oxo-9-cis	Vitamin A	26.14468	1	
158.5355	C04376	M(C13)+2H	8.00E-04	5'-Phospho	Purine me	100.4944	4	
159.0513	C01551	M+H[1+]	0	Allantoin;	Purine me	39.25249	4	
160.0401	C02220	M(S34)+H[	-5.00E-04	2-Aminom	Tryptopha	99.79173	4	
161.1366	C01601	M(Cl37)+H	0.0014		Linoleate r	39.74627	8	
163.5279	C00105	M(C13)+2H	0.001	UMP; Urid	Pyrimidine	135.0972	4	
164.0342	C01044	M(S34)+H[	-0.0013	N-Formyl-l	Aspartate	24.57938	6	
164.0561	C05839	M+2H[2+]	-0.0013	cis-beta-D-	Hexose ph	61.10343	4	
165.0102	C02355	M+H+Na[2	-7.00E-04	2',3'-Cyclic	UMP	27.47199	6	
165.0161	C04051	M+K[1+]	-9.00E-04	5-Amino-4	Purine me	100.8787	1	
165.5339	C00575	M+2H[2+]	4.00E-04	3',5'-Cyclic	Purine me	89.09741	4	
166.0499	C05653	M+H[1+]	0	Formylant	Tryptopha	26.32747	6	
167.0261	C00829	M+K[1+]	7.00E-04	Naphthale	Xenobiotic	27.60495	7	
168.0807	C00079	M(S34)+H[	-0.0014	L-Phenylal	Tyrosine m	26.87643	6	
169.0844	C00179	M+K[1+]	-2.00E-04	Agmatine;	Arginine ar	32.18229	8	
169.9825	C05528	M(C13)+H[	-0.0011	3-Sulfopyr	Methionin	69.48443	4	
170.0925	C01152	M+H[1+]	1.00E-04	N(pi)-Methyl-L-histidi		86.62112	1	
171.0419	C06103	M+K[1+]	5.00E-04	6-Hydroxyhexanoic ac		99.46879	4	
171.0804	C06459	M(S34)+H[	-0.0017	N-Trimeth	Aminosuga	24.90927	6	
171.0958	C00534	M(Cl37)+H	0.0014	Pyridoxam	Vitamin B6	87.48991	1	
172.0883	C01152	M(Cl37)+H	-0.0013	N(pi)-Methyl-L-histidi		24.64441	6	
174.0759	C01250	M+H[1+]	-2.00E-04	N-Acetyl-L	Urea cycle	25.69819	4	
175.1189	C00062	M+H[1+]	-1.00E-04	L-Arginine;	Alanine an	85.09523	7	
176.0705	C00954	M+H[1+]	-1.00E-04	Indole-3-a	Tryptopha	26.7453	8	
176.0706	C00954	M+H[1+]	0	Indole-3-a	Tryptopha	247.1998	7	
176.1181	C06429	M+H+Na[2	-3.00E-04	(4Z,7Z,10Z	De novo fa	19.2021	3	
176.1224	C00062	M(C13)+H[	0	L-Arginine;	Alanine an	83.77834	7	
177.1193	C00427	M+2H[2+]	-5.00E-04	Prostaglan	Prostaglan	87.25421	7	
180.0653	C01586	M+H[1+]	-2.00E-04	Hippurate;	Urea cycle	76.66213	6	
180.0655	C01586	M+H[1+]	0	Hippurate;	Urea cycle	25.84273	6	
181.995	C00354	M+H+Na[2	-0.0013	D-Fructose	Glycolysis	126.5029	7	
184.0731	C00588	M[1+]	-8.00E-04	Choline ph	Glycosphir	22.80612	1	
188.0376	C04409	M(Cl37)+H	7.00E-04	2-Amino-3	Tryptopha	198.3443	5	
190.0499	C01717	M+H[1+]	0	4-Hydroxy-	Tryptopha	29.33943	6	

190.0895	C05548	M(Cl37)+H	5.00E-04	6-Acetami	Butanoate	94.93539	4	
190.1072	C05933	M[1+]	6.00E-04	N-(omega)	Arginine ar	28.60267	4	
191.0439	C00153	M+HCOON	0.0012	Nicotinam	Vitamin B3	27.56357	6	
191.0622	C02712	M[1+]	6.00E-04	N-Acetylm	Methionin	48.19288	6	
192.0469	C01717	M(Cl37)+H	-2.00E-04	4-Hydroxy-	Tryptopha	25.55964	6	
192.9748	C00265	M+K[1+]	-2.00E-04	Dithiothrei	Selenoami	119.5432	4	
193.0019	C06608	M+K[1+]	-4.00E-04	Diethylphc	Parathio de	268.3292	7	
195.0299	C00158	M(Cl37)+H	-0.0016	Citrate; Cit	TCA cycle	138.2702	8	
198.0013	C05335	M+H[1+]	-0.0015	Selenomet	Selenoami	127.5366	7	
200.0684	C00255	M+H+Na[2	0.001	Riboflavin;	Vitamin B2	44.30215	8	
200.1017	C06114	M+H[1+]	-0.0013	gamma-Glutamyl-beta		26.97332	3	
200.5783	C00019	M(C13)+2H	7.00E-04	S-Adenosy	Ubiquinon	79.60808	4	
201.0537	C14784	M+Na[1+]	0.0014	1,2-Dihydr	Xenobiotic	58.45911	7	
202.1439	C03626	M[1+]	9.00E-04	Nomega,Nomega'-Dir		97.8006	5	
203.0928	C16362	M(S34)+H[	-0.0012	3,6,8-Trim	Caffeine m	113.0356	5	
204.0783	C06199	M+K[1+]	1.00E-04	Hordenine	Tyrosine m	36.98534	3	
205.064	C11924	M+K[1+]	0.0018	Perillic acid		28.62135	1	
207.0431	C06459	M+K[1+]	0.0013	N-Trimethy	Aminosuga	104.2926	8	
207.0762	C00579	M[1+]	0.001	Dihydrolip	Glycine, se	24.8848	6	
208.0417	C02470	M(Cl37)+H	-3.00E-04	Xanthuren	Tryptopha	46.79378	8	
209.0541	C05828	M+HCOON	8.00E-04	Methylimi	Histidine m	25.71219	6	
210.0118	C00346	M+HCOON	-0.002	Ethanolam	Glycosphir	42.76641	7	
210.0581	C01252	M(Cl37)+H	4.00E-04	4-(2-Amino	Tryptopha	33.79389	6	
211.0966	C02325	M+H[1+]	1.00E-04	Sinapyl alcohol; Sinap		184.8118	9	
215.1026	C00756	M+HCOOK	-0.0018	1-Octanol	Glyceroph	99.81239	3	
219.0475	C00121	M+HCOON	0	D-Ribose	Purine me	60.82624	9	
219.0971	C02576	M+HCOON	-0.0021	Perillyl ald	Limonene	53.80007	6	
220.044	C00242	M+HCOON	-1.00E-04	Guanine; 2	Urea cycle,	100.1434	8	
221.0925	C00521	M+HCOOK	-0.0013	(-)-Limonene; (-)-(S)-L		30.71676	6	
221.0957	C00521	M+HCOOK	0.0019	(-)-Limonene; (-)-(S)-L		25.26249	6	
221.1104	C00978	M(Cl37)+H	4.00E-04	N-Acetylse	Tryptopha	286.8162	7	
222.0858	C06114	M+Na[1+]	8.00E-04	gamma-Glutamyl-beta		24.77441	1	
223.089	C00643	M(Cl37)+H	-3.00E-04	5-Hydroxy-	Tryptopha	24.22012	1	
223.1074	C01239	M(S34)+H[	-0.0016	N-Acetyl-b	Aspartate a	192.0613	8	
224.0834	C01674	M+H+Na[2	5.00E-04	Chitobiose	Sialic acid r	24.81989	6	
224.0895	CE5626	M(C13)+H[	0.0022	salsolinol 1	Tyrosine m	52.07219	4	
225.0472	C03680	M+HCOON	-0.001	4-Imidazol	Histidine m	54.43594	8	
225.0869	C03227	M+H[1+]	-1.00E-04	3-Hydroxy-	Tryptopha	26.56602	6	
226.0898	C03227	M(C13)+H[	-6.00E-04	3-Hydroxy-	Tryptopha	25.44099	6	
227.0837	C03227	M(Cl37)+H	-5.00E-04	3-Hydroxy-	Tryptopha	25.28247	6	
229.0099	C00944	M+K[1+]	-6.00E-04	3-Dehydroquinate; 5-		100.0738	8	
231.0435	C01107	M(Cl37)+H	-9.00E-04	(R)-5-Phos	Squalene a	72.90972	4	
235.0387	C02646	M+HCOOK	0.002	4-Coumaryl alcohol; 4		283.5956	5	
236.9568	C04309	M+K[1+]	0.0011	Phosphoenol-4-deoxy		246.1465	8	
238.0929	C00234	M(C13)+2H	0.001	10-Formylt	Methionin	27.02093	7	
238.1073	C06868	M(C13)+H[	0.0016	Carbamaze	Drug meta	176.3285	4	
239.1044	C07501	M+H[1+]	0.0017	Felbamate	Drug meta	18.85865	1	

240.0902	C00835	M(Cl37)+H	-5.00E-04	Sepiapterin		25.27284	7	
240.9885	C03494	M+K[1+]	0.0015	D-Erythritol 4-phosph		121.4821	7	
241.1019	C07501	M(Cl37)+H	0.002	Felbamate	Drug meta	43.81865	1	
242.1029	C00268	M(S34)+H[	-0.002	Dihydrobic	Tyrosine m	25.06899	7	
246.0649	C11736	M[1+]	-3.00E-04	5-Fluorode	Drug meta	57.00323	6	
248.032	C00018	M+H[1+]	1.00E-04	Pyridoxal p	Vitamin B6	81.66939	7	
249.0537	C02325	M+K[1+]	0.0017	Sinapyl alcohol; Sinap		198.1782	5	
250.9626	C14873	M+HCOOK	4.00E-04		Xenobiotic	261.1085	7	
254.0941	C01736	M(C13)+H|	-0.0025	Nebularine; Purine rib		25.64987	6	
255.0975	C03150	M[1+]	-6.00E-04	N-Ribosyln	Vitamin B3	43.60466	6	
259.0211	C01236	M+H[1+]	-3.00E-04	D-Glucono	Pentose ph	229.2275	8	
261.9613	C01272	M+H+Na[2	-0.0013	1D-myo-In	Phosphatic	74.70085	4	
262.0394	C00085	M(C13)+H|	-0.001	D-Fructose	Pentose ph	44.17289	6	
263.0695	C16633	M+H[1+]	0.0021	5-Fluorour	Drug meta	29.36419	9	
266.9686	C00236	M+H[1+]	0.002	3-Phospho	Glycolysis	113.3854	7	
267.9509	C05689	M+HCOOK	0.0024	Se-Methyl	Selenoami	112.784	8	
269.055	C11736	M+Na[1+]	5.00E-04	5-Fluorode	Drug meta	61.62239	3	
269.0879	C00294	M+H[1+]	-2.00E-04	Inosine	Purine me	39.99815	4	
271.0814	C00294	M(S34)+H[	-0.0025	Inosine	Purine me	87.7459	1	
273.075	C08261	M+HCOOK	0.0015		Linoleate r	64.93994	8	
274.9331	C14842	M+HCOOK	0.0016	Bromoben	Xenobiotic	44.57567	2	
285.0837	C01762	M+H[1+]	7.00E-04	Xanthosine	Purine me	87.67701	8	
288.9839	C16193	M+H[1+]	4.00E-04	1,6-Naphthalenedisul		168.6639	8	
289.22	C02838	M(C13)+H|	-4.00E-04	L-Octanoylcarnitine		24.58457	6	
293.1456	C05118	M(C13)+H|	0.0022	S-(2-Methy	Valine, leu	72.2762	9	
307.0438	C00294	M+K[1+]	2.00E-04	Inosine	Purine me	42.39798	4	
308.0406	C00365	M[1+]	-4.00E-04	dUMP; De	Pyrimidine	44.39817	4	
312.9641	C07644	M+K[1+]	-0.0028	4-Ketocycl	Drug meta	265.5101	4	
314.1031	C16610	M(Cl37)+H	0.0028	Citalopram	Drug meta	280.9967	6	
315.1337	chtn	M(C13)+2H	4.00E-04	chitin	Aminosuga	24.14131	6	
325.043	C00105	M+H[1+]	-2.00E-04	UMP; Urid	Pyrimidine	117.5995	7	
326.0462	C00105	M(C13)+H|	-4.00E-04	UMP; Urid	Pyrimidine	119.5602	8	
330.0609	C00575	M+H[1+]	0.0011	3',5'-Cyclic	Purine me	167.1546	8	
341.0669	C04677	M(Cl37)+H	-4.00E-04	1-(5'-Phos	Purine me	60.0175	9	
343.1919	C16548	M[1+]	-0.0017	N,N-Dides	Drug meta	88.54802	3	
344.9996	C00085	M+HCOOK	0.0013	D-Fructose	Pentose ph	42.31	4	
345.0186	C00345	M+HCOON	-7.00E-04	6-Phospho	Pentose ph	125.0858	8	
347.0251	C00105	M+Na[1+]	-1.00E-04	UMP; Urid	Pyrimidine	123.4915	7	
347.0263	C00105	M+Na[1+]	0.0011	UMP; Urid	Pyrimidine	255.2723	8	
348.0445	C00130	M[1+]	-0.0026	IMP; Inosi	Purine me	65.06345	6	
350.9279	C00236	M+HCOOK	0	3-Phospho	Glycolysis	52.6408	6	
355.0384	C06196	M+Na[1+]	-0.0031	2'-Deoxyin	Purine me	139.8719	7	
363.0911	C05403	M+Na[1+]	0.0012	3-Ketolact	Galactose	248.2127	9	
364.0651	C00144	M+H[1+]	-2.00E-04	GMP; Guai	Purine me	178.7949	8	
367.0439	C00655	M(Cl37)+H	-0.0026	Xanthosine	Purine me	119.47	7	
368.0602	C00387	M+HCOOK	-1.00E-04	Guanosine	Purine me	183.4529	8	
375.1927	C00523	M+HCOOK	-5.00E-04	Androstero	C21-steroi	259.421	2	

379.0343	C16619	M[1+]	-9.00E-04	6-Thioguar	Drug meta	100.3755	8	
412.0754	C00894	M(C13)+2H	0.0035	Propenoyl-	Beta-Alani	197.8476	5	
416.5576	C00024	M+H+Na[2	-0.0036	Acetyl-CoA	Prostaglan	100.9075	4	
421.019	C01185	M+HCOOK	0.002	Nicotinate	Vitamin B3	54.78746	6	
426.9715	C01346	M+K[1+]	0.0014	dUDP; 2'-D	Pyrimidine	209.599	4	
431.0978	C11173	M+K[1+]	-0.0022	SN-38	Drug meta	196.2579	5	
437.0763	C00512	M(C13)+2H	-0.0034	S-Benzoate coenzyme		99.78134	8	
438.2976	C02990	M+K[1+]	-1.00E-04	L-Palmitoy	Carnitine s	22.38412	1	
439.3011	C01561	M+K[1+]	0.0042	Calcidiol; 2	Vitamin D3	24.4346	6	
446.3261	lneldccrn	M+Na[1+]	0.002	Linoelaidyl	Carnitine s	21.61518	6	
447.3295	C06125	M(C13)+2H	-0.0029	Sulfatide; (	Glycosphir	21.68301	6	
448.3418	elaidcrn	M+Na[1+]	0.002	Elaidic carr	Carnitine s	17.23795	6	
450.3482	C01628	M[1+]	-0.0016	Vitamin K	Vitamin K r	21.54367	6	
451.0008	C00104	M+Na[1+]	-0.0019	IDP; Inosin	Galactose	168.5955	7	
451.3609	C01628	M+H[1+]	0.0038	Vitamin K	Vitamin K r	21.61479	6	
455.2163	CE5994	M(Cl37)+H	0.0011	20-oxo-leu	Leukotrien	75.9816	4	
455.2961	C01673	M+K[1+]	0.0043	Calcitriol	Vitamin D3	25.08793	6	
462.2975	lneldccrn	M+K[1+]	-1.00E-04	Linoelaidyl	Carnitine s	25.14307	1	
464.3134	elaidcrn	M+K[1+]	1.00E-04	Elaidic carr	Carnitine s	23.40484	6	
466.3524	C03640	M+H[1+]	-6.00E-04	Sphingosyl-phosphocl		21.8812	6	
468.3083	C01921	M(S34)+H[	-0.0038	Glycochola	Bile acid bi	28.39677	5	
469.0695	C05125	M[1+]	-0.0017	2-(alpha-H	Butanoate	287.6715	4	
469.3119	C00599	M+HCOOK	0.0041	Cholest-4-en-3-one; C		28.52498	5	
473.3453	c226crn	M(C13)+H[	-2.00E-04	cervonyl ca	Carnitine s	21.59564	6	
474.3576	clpndcrn	M+H[1+]	-2.00E-04	clupanodo	Carnitine s	21.52924	6	
475.3611	clpndcrn	M(C13)+H[	-1.00E-04	clupanodo	Carnitine s	21.64843	6	
479.296	CE7144	M+K[1+]	0.0042	13'-hydrox	Vitamin E r	25.15684	6	
483.3273	C04453	M+HCOOK	0.0038	4alpha-Methyl-5alpha		24.47989	1	
486.0649	C03794	M+Na[1+]	0.0016	N6-(1,2-Di	Purine met	100.1459	4	
492.0109	C00131	M+H[1+]	0.0028	dATP; 2'-D	Pyrimidine	96.74792	4	
494.3241	c226crn	M+Na[1+]	0	cervonyl ca	Carnitine s	26.49185	6	
495.3272	44mctr	M+HCOOK	0.0038	4,4-dimeth	Squalene a	27.29103	6	
496.3393	clpndcrn	M+Na[1+]	-5.00E-04	clupanodo	Carnitine s	18.5974	1	
497.3428	C05108	M+HCOOK	0.0037	14-Demeth	Squalene a	19.29923	1	
512.3344	stcrn	M+HCOOK	-3.00E-04	stearoylca	Carnitine s	27.38073	1	
529.3283	C04840	M+HCOOK	-6.00E-04	3beta-Hydroxy-4beta-		27.15776	1	
551.6826	C16173	M+H+Na[2	0.0041	(4Z,7Z,10Z	Polyunsatu	50.56125	1	
566.1284	CE6186	M+H+Na[2	0.003	18E-20-oxo	Leukotrien	100.5184	8	
649.05	C00842	M+HCOOK	0.0056	dTDP-gluco	Nucleotide	280.7179	3	
911.2263	C05266	M(C13)+H[	0.001	(S)-Hydrox	Saturated	279.6863	3	

Table S6e. Genes associated with metabolites in network for interaction

Gene			Metabolite			
Node	Cluster	Name	Node	Cluster	mz	rt
X16	1	*Tnfaip6*	Y10044	1	368.0602	183.4529
X8	1	*Il7r*	Y10238	1	374.5039	125.7008
			Y1046	1	126.9644	16.07659
			Y1047	1	126.9675	262.487
			Y1052	1	126.9896	272.2422
			Y1058	1	127.0215	44.60402
			Y11058	1	405.5647	260.9067
			Y1129	1	129.1024	85.87494
			Y1158	1	130.0863	73.36979
			Y11861	1	438.2976	22.38412
			Y12077	1	448.3348	22.07258
			Y12128	1	450.6647	47.51171
			Y1213	1	131.0895	79.99142
			Y12338	1	460.9671	117.3736
			Y12345	1	461.7448	66.8197
			Y12347	1	461.776	50.61678
			Y12402	1	464.3134	23.40484
			Y12765	1	482.3604	27.87635
			Y12784	1	483.3273	24.47989
			Y12785	1	483.3637	28.35409
			Y12939	1	492.5877	44.31243
			Y13014	1	496.3393	18.5974
			Y13038	1	497.3428	19.29923
			Y13080	1	499.3482	27.31861
			Y13083	1	499.8935	56.66061
			Y13087	1	499.9716	57.43728
			Y13119	1	500.9917	168.9207
			Y13199	1	506.3596	25.82906
			Y13239	1	508.1107	115.2816
			Y13317	1	512.3344	27.38073
			Y13371	1	515.3132	27.29436
			Y13606	1	527.8146	27.66991
			Y13620	1	528.2978	24.98499
			Y13649	1	529.3283	27.15776
			Y13756	1	534.8024	253.6498
			Y13816	1	538.3864	25.52527
			Y13844	1	539.3889	25.84289
			Y14038	1	549.3745	23.19512
			Y14085	1	551.3895	26.6441
			Y14089	1	551.6826	50.56125
			Y14124	1	553.4026	25.46548
			Y14140	1	554.2859	23.48491
			Y14143	1	554.6373	47.14885

Y1447	1	137.5153	34.45787
Y14568	1	578.0935	100.1297
Y14599	1	579.6671	280.9908
Y14643	1	582.863	57.31501
Y1475	1	138.0914	17.72665
Y1492	1	138.9942	89.51049
Y14925	1	598.5295	47.18989
Y14943	1	599.4412	30.44216
Y1499	1	139.0309	125.5059
Y1567	1	140.9035	47.71077
Y16070	1	656.576	47.04821
Y16200	1	664.1154	253.4334
Y16300	1	671.1225	142.6501
Y1635	1	142.5465	125.3949
Y16634	1	695.0042	52.68062
Y16739	1	702.5011	36.48399
Y1706	1	144.0712	104.638
Y17130	1	726.507	23.19143
Y17164	1	729.5253	23.5538
Y17173	1	730.5392	17.08043
Y17634	1	775.4861	51.68949
Y17851	1	797.8472	57.05617
Y2043	1	152.5845	103.6568
Y2051	1	152.9884	121.4666
Y2096	1	154.0229	43.69815
Y2171	1	156.0843	28.5813
Y2208	1	157.0608	59.93977
Y2219	1	157.1084	86.31781
Y2232	1	158.0028	273.601
Y2247	1	158.0924	86.00326
Y2248	1	158.0998	26.14468
Y2297	1	159.0958	86.57591
Y2298	1	159.1031	26.59286
Y2537	1	165.0161	100.8787
Y2613	1	167.0261	27.60495
Y2639	1	167.505	123.0597
Y2654	1	168.0235	57.13369
Y266	1	100.0474	193.9999
Y2739	1	170.0925	86.62112
Y2779	1	171.0958	87.48991
Y2953	1	175.1189	85.09523
Y2980	1	176.0658	52.22134
Y2982	1	176.0706	247.1998
Y2995	1	176.1224	83.77834
Y3019	1	177.044	58.61296
Y3039	1	177.1193	87.25421
Y3063	1	178.024	46.30275

Y3099	1	178.9743	127.6552
Y3310	1	184.0731	22.80612
Y3396	1	186.1489	181.817
Y3627	1	192.0397	45.19008
Y3662	1	193.0019	268.3292
Y3680	1	193.0971	190.1219
Y3874	1	198.0013	127.5366
Y3972	1	200.201	15.86842
Y3989	1	201.0537	58.45911
Y4029	1	202.0897	195.4012
Y4167	1	205.0041	92.30072
Y4172	1	205.0446	195.2685
Y4194	1	205.5069	100.9801
Y4315	1	208.0136	42.82374
Y4320	1	208.0604	146.038
Y4387	1	210.0118	42.76641
Y4445	1	211.1692	20.696
Y4552	1	214.1438	148.6297
Y4811	1	220.0888	25.74679
Y4861	1	221.1104	286.8162
Y4890	1	222.0228	262.984
Y4943	1	223.089	24.22012
Y4971	1	223.9886	142.5345
Y5074	1	226.0241	45.064
Y5271	1	230.9871	118.0849
Y5523	1	236.1726	17.89089
Y5600	1	238.0929	27.02093
Y5601	1	238.0991	21.67942
Y561	1	112.087	83.83632
Y5615	1	238.5169	100.4796
Y5644	1	239.1044	18.85865
Y5677	1	240.0902	25.27284
Y5678	1	240.0992	24.3727
Y5706	1	240.9885	121.4821
Y5721	1	241.1019	43.81865
Y5754	1	242.1029	25.06899
Y5846	1	244.1542	29.69017
Y5986	1	248.032	81.66939
Y607	1	113.0904	86.13736
Y6145	1	252.1442	66.95881
Y6148	1	252.2039	186.5762
Y6169	1	253.0488	123.4554
Y6437	1	260.081	52.85636
Y6558	1	263.1224	171.6916
Y6581	1	263.9991	168.0539
Y6668	1	266.186	23.01319
Y6689	1	266.9686	113.3854

			Y6752	1	268.6499	27.62935
			Y6853	1	271.0814	87.7459
			Y696	1	116.0707	70.24569
			Y7094	1	277.1024	53.99619
			Y7100	1	277.1536	27.34903
			Y7461	1	286.9061	58.90057
			Y7468	1	287.0037	99.24938
			Y7564	1	289.5264	113.8928
			Y7737	1	294.1269	23.00301
			Y7881	1	299.0832	41.9153
			Y7933	1	300.2612	20.94449
			Y8306	1	311.4435	168.0933
			Y8587	1	319.2245	47.20437
			Y8736	1	325.043	117.5995
			Y90	1	90.94773	18.83546
			Y9285	1	341.4938	115.3037
			Y9440	1	346.168	28.51799
			Y9462	1	347.0251	123.4915
			Y9506	1	349.0346	167.5701
			Y9552	1	350.5718	111.3003
			Y9855	1	361.2596	23.71245
X12	3	*Rela*	Y10	3	85.07789	92.13098
X14	3	*Tgfb1*	Y10494	3	384.3105	21.78682
X7	3	*Icam1*	Y10510	3	384.9697	56.78118
			Y10703	3	391.9829	110.4535
			Y10859	3	398.0274	75.48036
			Y11130	3	408.6738	45.24104
			Y1134	3	129.5067	84.14342
			Y11454	3	422.0108	54.90533
			Y11808	3	436.3053	22.57517
			Y11823	3	436.9931	52.83432
			Y11834	3	437.3087	22.53787
			Y12470	3	466.8448	50.39409
			Y12516	3	469.9347	56.04543
			Y12590	3	473.945	52.70681
			Y12671	3	477.9384	52.08609
			Y1268	3	132.9028	155.0183
			Y13745	3	534.2627	274.5422
			Y13769	3	535.528	275.2987
			Y1386	3	135.9763	56.16832
			Y13871	3	540.7784	50.47484
			Y1407	3	136.4914	157.8763
			Y14283	3	563.0001	52.9933
			Y14512	3	575.5015	24.21279
			Y1464	3	138.0275	88.08543
			Y14687	3	585.3041	281.8741
			Y14980	3	600.9555	52.69276

Y15368	3	616.7841	54.14073
Y15520	3	623.318	291.778
Y15619	3	628.534	47.79333
Y15743	3	636.9169	52.63839
Y15946	3	649.05	280.7179
Y15990	3	651.4739	52.56287
Y17202	3	732.9604	52.54842
Y17568	3	769.241	290.0577
Y1816	3	147.0441	45.24878
Y18294	3	911.2263	279.6863
Y1852	3	148.004	55.61717
Y1890	3	149.0009	55.33413
Y1891	3	149.0033	54.63775
Y1942	3	150.0081	55.68949
Y2034	3	152.107	250.242
Y226	3	98.99845	123.3542
Y23	3	86.06011	63.22627
Y2759	3	170.9891	55.86412
Y2950	3	175.1116	20.96484
Y2993	3	176.1181	19.2021
Y3334	3	185.0006	61.04575
Y3371	3	185.9597	52.64886
Y3451	3	187.5299	88.95085
Y3837	3	197.041	66.85533
Y3964	3	200.1017	26.97332
Y3967	3	200.128	32.58574
Y4035	3	202.1439	97.8006
Y4079	3	203.0928	113.0356
Y4125	3	204.0783	36.98534
Y4416	3	210.9407	41.73129
Y4593	3	215.1026	99.81239
Y4761	3	219.1056	120.6203
Y4864	3	221.1495	100.5512
Y4899	3	222.0955	25.16509
Y5255	3	230.5753	94.33825
Y5331	3	232.1542	30.66235
Y5377	3	233.1576	30.92025
Y5588	3	237.9731	56.65092
Y5652	3	239.2004	17.88378
Y5748	3	242.0789	40.86244
Y5771	3	242.9253	56.98653
Y5886	3	245.1497	42.98322
Y5913	3	246.0631	260.1081
Y5943	3	247.0286	56.20098
Y628	3	114.1277	29.84119
Y6444	3	260.1855	26.82716
Y647	3	115.0466	267.3126

		Y6485	3	261.1887	26.96614
		Y6768	3	269.055	61.62239
		Y6852	3	271.0753	57.76606
		Y6929	3	273.0184	67.26879
		Y7158	3	278.8935	15.54547
		Y7367	3	284.1852	25.61388
		Y7448	3	286.201	25.13965
		Y7517	3	288.2167	24.45339
		Y7857	3	298.2011	15.74219
		Y8002	3	302.1959	29.87719
		Y8110	3	305.9592	56.44234
		Y8316	3	311.9776	52.77652
		Y8488	3	316.2478	23.11028
		Y8519	3	317.2512	22.70967
		Y8922	3	330.2273	26.69255
		Y9029	3	333.2462	25.88667
		Y9406	3	345.1056	179.8497
		Y9828	3	360.2742	23.86249
X9	4 *Jun*	Y1019	4	126.0186	25.27579
		Y1055	4	127.0123	24.80024
		Y1084	4	128.0157	25.59723
		Y10907	4	400.0291	53.36189
		Y1098	4	128.062	24.3269
		Y11222	4	412.3209	21.41015
		Y1123	4	129.0699	24.15459
		Y11888	4	439.3011	24.4346
		Y12031	4	446.3261	21.61518
		Y12058	4	447.3295	21.68301
		Y12197	4	454.2926	24.26109
		Y12218	4	455.2961	25.08793
		Y12361	4	462.2975	25.14307
		Y12624	4	475.3611	21.64843
		Y12724	4	480.3084	23.79256
		Y12735	4	480.8214	50.46283
		Y1422	4	137.0153	25.03309
		Y14424	4	570.3556	25.13504
		Y1494	4	139.0123	25.31364
		Y1529	4	140.0076	25.94875
		Y15672	4	631.9737	58.44312
		Y1582	4	141.0102	24.54165
		Y16552	4	688.9693	293.2237
		Y1666	4	143.0855	23.02831
		Y16726	4	701.56	22.94954
		Y17106	4	723.9729	52.7141
		Y1713	4	144.0934	24.52875
		Y17878	4	801.8816	52.66441
		Y17904	4	806.5552	23.27711

			Y17937	4	812.5363	28.31364
			Y17957	4	814.5574	24.22391
			Y18271	4	886.9429	52.58784
			Y2023	4	152.0262	25.44742
			Y2030	4	152.0623	31.74855
			Y2066	4	153.0699	26.01216
			Y2109	4	154.0777	25.74511
			Y2146	4	155.0854	21.70136
			Y227	4	98.99965	23.85995
			Y2535	4	165.0102	27.47199
			Y254	4	100.003	25.31962
			Y2575	4	166.0135	25.1057
			Y2579	4	166.0315	25.65255
			Y2583	4	166.0499	26.32747
			Y2608	4	167.0073	24.77147
			Y2617	4	167.0436	24.69195
			Y2651	4	168.0106	24.78865
			Y282	4	100.9967	24.74502
			Y3120	4	179.0626	25.29742
			Y3167	4	180.0653	76.66213
			Y3169	4	180.0655	25.84273
			Y3204	4	181.0595	25.58344
			Y3497	4	189.0466	29.44332
			Y3535	4	190.0499	29.33943
			Y3585	4	191.0439	27.56357
			Y4357	4	209.0635	24.6188
			Y4390	4	210.0581	33.79389
			Y5039	4	225.0908	22.4223
			Y5679	4	240.0995	111.3863
			Y6210	4	254.0941	25.64987
			Y6243	4	255.0975	43.60466
			Y6277	4	256.0911	25.19223
			Y6324	4	257.0945	25.811
			Y68	4	89.03864	29.16497
			Y78	4	90.04197	25.0188
			Y80	4	90.04643	27.64032
			Y8374	4	313.2734	22.3749
			Y977	4	125.0152	26.69958
X1	5	*Cat*	Y14984	5	601.2972	284.2246
			Y2821	5	172.0967	27.79243
X11	6	*Prdx1*	Y10103	6	370.1961	129.0348
X18	6	*Sepp1*	Y10149	6	371.3269	25.09635
			Y10690	6	391.2838	22.49414
			Y10918	6	400.2538	22.20564
			Y11118	6	407.9179	52.27206
			Y11134	6	408.7626	45.30835

Y11234	6	412.8626	271.2442
Y11249	6	413.2662	46.55564
Y11276	6	414.2694	41.9276
Y11627	6	429.2401	45.90552
Y11647	6	430.2433	43.15558
Y1220	6	131.481	79.62172
Y12216	6	455.2163	75.9816
Y12225	6	455.9235	48.65456
Y12369	6	462.8615	56.65368
Y12641	6	476.3062	28.42496
Y1272	6	132.9551	263.9638
Y12930	6	492.0109	96.74792
Y13074	6	499.0206	254.8498
Y13324	6	512.6447	43.83531
Y13359	6	514.6408	43.72913
Y13392	6	516.5585	45.85521
Y13458	6	521.1029	210.463
Y13553	6	525.8975	56.65335
Y13673	6	530.8491	56.97663
Y13792	6	536.9197	55.96915
Y13855	6	539.9492	240.46
Y14197	6	557.8473	55.61854
Y148	6	94.04511	129.8554
Y14847	6	594.6041	248.9333
Y14889	6	596.4599	52.68893
Y15107	6	606.6843	290.2352
Y15147	6	608.6685	68.4674
Y1557	6	140.4862	79.50191
Y16099	6	658.5602	45.11399
Y16172	6	662.6295	51.4473
Y16206	6	664.3566	253.6836
Y16757	6	703.5746	23.27397
Y1680	6	143.9523	89.99791
Y17626	6	774.4914	47.62098
Y18027	6	824.4012	44.22741
Y1922	6	149.1325	204.4287
Y2307	6	159.4734	106.012
Y2314	6	159.947	266.081
Y2437	6	162.4805	79.7642
Y2495	6	163.9843	146.6991
Y2718	6	169.9825	69.48443
Y3004	6	176.5091	191.691
Y3189	6	180.9748	130.2291
Y3387	6	186.0867	80.03524
Y4061	6	202.9972	137.2855
Y4113	6	204.0045	129.5535
Y4164	6	204.9747	128.4978

			Y4168	6	205.0109	127.847
			Y4659	6	216.8974	90.49669
			Y475	6	108.9614	267.8826
			Y4796	6	219.9682	146.5627
			Y4957	6	223.4656	128.4627
			Y4978	6	224.0433	55.39837
			Y5148	6	227.6549	68.84301
			Y7237	6	280.9848	178.7362
			Y7573	6	289.8968	51.63349
			Y8236	6	309.206	180.2144
			Y8471	6	315.9814	115.4313
			Y9345	6	343.2952	26.16488
			Y941	6	123.5943	71.8921
			Y9461	6	347.0129	125.6036
X15	7	*Tlr2*	Y10084	7	369.4879	278.7509
X2	7	*Csrp1*	Y10126	7	370.9827	241.0619
X5	7	*Dusp4*	Y1013	7	125.9643	16.55913
			Y10428	7	382.057	59.57613
			Y1048	7	126.972	267.2128
			Y10604	7	387.996	275.7219
			Y10649	7	389.2684	22.34947
			Y10660	7	390.0111	61.23281
			Y10691	7	391.2839	210.6398
			Y1074	7	127.5088	91.46904
			Y108	7	91.52385	262.4388
			Y10837	7	397.1642	156.8443
			Y10995	7	403.2624	22.50289
			Y1101	7	128.0805	183.9466
			Y11127	7	408.2926	21.42637
			Y11137	7	408.8532	34.95116
			Y11164	7	410.0053	84.94139
			Y11165	7	410.0378	63.23281
			Y11166	7	410.0445	249.9055
			Y11173	7	410.2239	79.43848
			Y11312	7	415.8858	67.26429
			Y11362	7	417.8919	46.52635
			Y11392	7	419.1463	27.46814
			Y11401	7	419.7665	25.67388
			Y11444	7	421.727	271.8166
			Y11508	7	424.7321	67.6543
			Y11509	7	424.7322	59.90118
			Y11527	7	425.8246	44.61692
			Y11557	7	426.9715	209.599
			Y11561	7	427.0273	90.5741
			Y11714	7	432.2801	28.10498
			Y11797	7	435.8632	46.57246
			Y1181	7	130.933	152.1117

Y11897	7	440.3131	25.82009
Y120	7	92.0244	38.08873
Y12023	7	446.0546	77.70078
Y12044	7	446.9361	57.4595
Y12071	7	448.0519	59.22744
Y1208	7	131.0717	190.8622
Y12095	7	448.9609	76.45193
Y12102	7	449.2559	73.42758
Y12112	7	449.9642	89.51895
Y1223	7	131.5169	99.16969
Y1233	7	132.0035	280.8324
Y1235	7	132.0186	100.692
Y12490	7	468.3891	22.1955
Y12504	7	469.0695	287.6715
Y12621	7	475.1542	273.5548
Y12629	7	475.8506	46.87498
Y12636	7	476.0891	58.76336
Y12669	7	477.8478	46.71385
Y12670	7	477.8806	79.21283
Y1274	7	132.9696	28.73949
Y12741	7	481.0178	265.5959
Y12801	7	484.3297	28.81949
Y1281	7	133.0339	234.8419
Y1286	7	133.0591	290.5833
Y12866	7	487.9118	92.78584
Y12871	7	488.1548	30.22335
Y12878	7	488.6936	47.61426
Y13198	7	506.2683	270.1276
Y13256	7	508.965	57.80633
Y13311	7	512.022	253.5726
Y13341	7	513.2648	69.47656
Y13418	7	518.3217	24.09908
Y13509	7	524.0561	256.6843
Y13558	7	526.0465	53.24017
Y13565	7	526.3786	25.86707
Y13596	7	527.4221	280.8135
Y13740	7	534.0492	259.2565
Y1390	7	136.0143	38.42132
Y1398	7	136.0617	276.2446
Y14024	7	548.86	277.0326
Y14031	7	549.0882	238.9024
Y14043	7	549.5896	239.4576
Y14064	7	550.5462	251.8997
Y14084	7	551.3121	26.98712
Y1411	7	136.514	91.94164
Y1428	7	137.0458	37.38619
Y14301	7	564.3584	28.68445

Y14355	7	567.0442	90.71039
Y14411	7	569.6843	266.6598
Y14480	7	574.1197	112.6175
Y146	7	94.04004	37.2356
Y1465	7	138.0298	37.10379
Y1466	7	138.0429	37.16868
Y1468	7	138.049	38.72642
Y14700	7	586.5351	279.7139
Y14802	7	592.6732	47.49957
Y14807	7	592.8772	277.5878
Y14830	7	593.9766	54.34562
Y14921	7	598.0229	279.7004
Y15163	7	609.4007	30.18308
Y15278	7	612.9897	280.5089
Y1537	7	140.0287	88.37082
Y15444	7	619.9725	294.3029
Y15699	7	633.6508	49.19803
Y15767	7	638.4338	29.50295
Y15926	7	647.947	56.30254
Y16122	7	659.8608	63.53273
Y16186	7	663.4956	20.5527
Y16198	7	664.0238	255.8471
Y16238	7	666.7391	276.9137
Y16466	7	683.2703	295.6067
Y1655	7	143.0339	209.2944
Y16565	7	689.5135	20.63716
Y16571	7	689.9655	277.7323
Y16598	7	692.0472	186.0351
Y16645	7	695.3393	268.7114
Y16836	7	706.9675	57.80233
Y17165	7	729.5889	22.50184
Y17403	7	753.1315	267.2639
Y1741	7	145.0351	279.6261
Y17442	7	757.6353	68.72231
Y17685	7	780.5389	21.7149
Y17687	7	780.5886	23.02974
Y17748	7	788.5945	18.94842
Y17836	7	796.5454	29.77174
Y1784	7	146.0529	242.8781
Y17967	7	816.4715	47.70767
Y1797	7	146.1654	66.49199
Y17970	7	817.0432	54.27296
Y18184	7	854.3739	47.92457
Y1879	7	148.5237	91.52672
Y1911	7	149.0767	72.70358
Y1943	7	150.0141	278.9721
Y1962	7	150.5286	99.28435

Y1987	7	151.0696	289.0153
Y2032	7	152.0818	227.8718
Y2126	7	154.9669	167.9665
Y2147	7	155.0911	172.5793
Y2289	7	159.0513	39.25249
Y2328	7	160.0428	186.3176
Y2348	7	160.527	91.11677
Y2402	7	161.9628	99.01937
Y2420	7	162.0584	37.0079
Y2485	7	163.5279	135.0972
Y2488	7	163.9401	94.84676
Y2507	7	164.0561	61.10343
Y2566	7	165.5339	89.09741
Y2572	7	165.9828	261.1149
Y2600	7	166.9545	16.12863
Y2605	7	166.9832	15.61518
Y2616	7	167.0339	102.6781
Y2848	7	173.021	178.5527
Y2851	7	173.0301	280.5046
Y2876	7	173.916	94.79266
Y2882	7	173.9961	35.71882
Y2887	7	174.0316	198.0151
Y2896	7	174.0759	25.69819
Y2958	7	175.1481	160.5813
Y303	7	102.013	281.9958
Y3035	7	177.1014	186.0714
Y3056	7	177.9575	273.6649
Y3085	7	178.4631	285.4678
Y3100	7	178.975	34.9147
Y3265	7	182.9966	97.19262
Y336	7	103.0058	79.6643
Y3360	7	185.4959	107.1901
Y3380	7	186.0505	77.78637
Y3412	7	186.9563	165.9415
Y3544	7	190.0895	94.93539
Y3548	7	190.1072	28.60267
Y3565	7	190.925	79.42072
Y3567	7	190.9628	164.3617
Y3630	7	192.0503	70.50035
Y3649	7	192.5048	102.7104
Y3700	7	193.521	166.7802
Y3739	7	194.5006	191.4774
Y3768	7	195.0877	22.47124
Y3804	7	196.0561	99.8197
Y3851	7	197.1285	183.0122
Y3857	7	197.5206	98.05829
Y3877	7	198.0224	100.7061

Y3948	7	199.6366	158.6745
Y3952	7	199.9965	289.8378
Y408	7	105.4921	267.839
Y4162	7	204.9666	95.11373
Y4219	7	206.0456	104.1328
Y4305	7	207.6066	22.88546
Y4393	7	210.0913	176.7211
Y44	7	88.00425	260.5333
Y4412	7	210.8993	158.891
Y4418	7	210.968	93.96143
Y4531	7	213.941	118.0033
Y4535	7	213.9796	27.48043
Y4605	7	215.4192	79.06265
Y4715	7	218.1208	27.21488
Y4725	7	218.4691	115.2063
Y4734	7	218.8304	48.11931
Y4792	7	219.9312	79.01423
Y498	7	110.0293	34.98544
Y4988	7	224.0895	52.07219
Y500	7	110.035	37.73777
Y5112	7	226.9734	43.82616
Y5224	7	229.9744	36.99331
Y5230	7	230.0372	170.3478
Y5272	7	231.0092	170.2675
Y5277	7	231.0435	72.90972
Y546	7	112.0111	79.63643
Y5493	7	235.9621	271.8129
Y5539	7	236.9045	78.83957
Y5593	7	238.0378	66.42242
Y5603	7	238.1073	176.3285
Y5622	7	238.8941	158.3387
Y5666	7	240.0022	61.73243
Y5689	7	240.2319	25.61179
Y5707	7	240.9959	92.54559
Y5763	7	242.5112	184.7082
Y5999	7	248.1738	29.08201
Y6180	7	253.1476	66.02655
Y6200	7	254.0102	92.46312
Y6307	7	256.9636	58.33634
Y6352	7	257.9934	44.84911
Y6354	7	258.0013	126.8078
Y6394	7	258.9961	44.51295
Y6425	7	259.957	98.43021
Y6506	7	261.9545	98.66893
Y6507	7	261.9613	74.70085
Y6596	7	264.1263	31.20972
Y6629	7	265.1116	60.30374

Y6648	7	265.9269	279.9436
Y6662	7	266.115	60.97739
Y6674	7	266.5289	89.39073
Y6721	7	267.9305	44.41643
Y6825	7	270.5227	128.3451
Y6833	7	270.8913	78.06964
Y6919	7	272.8549	93.96283
Y7097	7	277.1302	29.36119
Y7311	7	282.9732	79.13564
Y7405	7	285.1386	105.99
Y743	7	117.9698	78.12367
Y751	7	118.0289	77.47648
Y7525	7	288.592	109.1143
Y7630	7	290.9345	94.00606
Y7688	7	292.5218	276.0095
Y771	7	118.9206	97.19323
Y7779	7	295.9382	46.87892
Y7788	7	296.0494	76.07999
Y7808	7	296.9231	96.99099
Y7948	7	300.9835	79.25621
Y8000	7	302.1438	49.32215
Y8078	7	304.9557	51.83034
Y8092	7	305.1569	46.59061
Y8115	7	306.0016	50.35336
Y8141	7	306.8289	47.48479
Y8152	7	306.9979	279.6197
Y8175	7	307.288	211.0052
Y8212	7	308.9449	91.23491
Y825	7	120.0558	90.58705
Y8394	7	314.0328	190.8115
Y8451	7	315.1741	255.7263
Y8531	7	317.9082	78.59706
Y8565	7	318.9051	78.73891
Y8580	7	319.0614	264.8417
Y86	7	90.52583	278.1456
Y882	7	122.0192	274.596
Y894	7	122.0714	61.00587
Y8964	7	331.5416	256.8696
Y901	7	122.5204	79.7897
Y9329	7	343.0659	99.90017
Y9376	7	344.2431	26.47808
Y9394	7	344.9996	42.31
Y9429	7	346.0031	42.44711
Y943	7	123.9547	272.2297
Y9500	7	348.9745	279.4011
Y9531	7	349.9759	285.4996
Y961	7	124.0758	136.3834

			Y9615	7	352.8533	77.99886
			Y9635	7	353.4644	91.41775
			Y9643	7	353.8976	49.2269
			Y971	7	124.9564	15.45339
			Y9756	7	358.0149	100.2397
			Y9800	7	359.3156	153.7243
			Y9816	7	360.0367	98.03781
			Y9857	7	361.31	21.51788
X17	8	*Tnfrsf9*	Y10026	8	367.5359	127.3357
X3	8	*Csrp2*	Y10042	8	368.0376	127.5777
			Y10065	8	368.9558	189.0045
			Y10113	8	370.5319	129.4792
			Y10120	8	370.832	159.8474
			Y10184	8	372.6074	263.6614
			Y10329	8	378.5207	128.515
			Y10347	8	379.0216	128.109
			Y10348	8	379.0343	100.3755
			Y10359	8	379.536	100.0636
			Y10386	8	380.6724	45.75095
			Y10400	8	380.9844	261.756
			Y10456	8	383.0389	168.7127
			Y10476	8	383.5403	168.9596
			Y10560	8	386.5095	127.1686
			Y1059	8	127.0253	52.14304
			Y10753	8	394.0341	100.8421
			Y10784	8	395.0374	99.51426
			Y10811	8	396.1201	100.3123
			Y10902	8	399.9577	262.4209
			Y10955	8	402.0121	168.3933
			Y10972	8	402.5138	168.3141
			Y11290	8	414.9432	48.47144
			Y11350	8	417.1415	88.60492
			Y11365	8	417.9732	114.6541
			Y11611	8	428.9612	42.28807
			Y11620	8	429.0824	85.58078
			Y11825	8	437.0763	99.78134
			Y11837	8	437.5778	100.2883
			Y11853	8	438.0797	99.93758
			Y11880	8	439.0647	260.5861
			Y119	8	92.01652	65.77824
			Y11912	8	441.0686	100.2898
			Y11914	8	441.1109	155.1427
			Y11956	8	443.0387	168.3591
			Y12274	8	458.1619	75.90782
			Y12363	8	462.6368	45.23667
			Y12453	8	466.0863	114.4004
			Y1247	8	132.0905	92.48056

	Y12485	8	468.0709	96.90508
	Y12818	8	485.0143	239.504
	Y13130	8	502.0181	288.7051
	Y1321	8	134.06	228.1295
	Y13312	8	512.0436	126.8608
	Y13340	8	513.2383	144.7699
	Y13416	8	518.219	100.3478
	Y138	8	93.04477	85.91488
	Y14325	8	565.6298	101.4229
	Y14591	8	579.0758	131.3159
	Y14592	8	579.0982	99.91212
	Y14704	8	586.6387	47.57907
	Y14739	8	588.5904	47.3351
	Y14982	8	601.0447	128.9067
	Y15477	8	621.1683	167.7851
	Y155	8	94.51518	157.7335
	Y15508	8	622.7657	49.82918
	Y1562	8	140.5222	100.0998
	Y15905	8	646.45	44.67564
	Y16032	8	654.106	188.4919
	Y16507	8	685.1244	99.9046
	Y16551	8	688.9621	289.0085
	Y16553	8	689.047	293.9456
	Y16929	8	712.1111	168.5598
	Y17026	8	716.7184	171.9486
	Y17142	8	727.1224	169.226
	Y17194	8	732.4029	44.05633
	Y17213	8	734.0655	124.6709
	Y17757	8	789.6188	25.10327
	Y1900	8	149.0263	54.0224
	Y2054	8	153.001	121.4282
	Y2134	8	155.0112	78.23482
	Y2169	8	156.0768	87.73439
	Y2191	8	156.9695	177.6979
	Y2197	8	157.0096	34.55355
	Y2212	8	157.0738	89.78477
	Y2214	8	157.0801	88.50503
	Y2226	8	157.5374	100.1742
	Y2311	8	159.5339	99.87311
	Y2315	8	159.9471	94.19409
	Y2341	8	160.1332	35.88149
	Y2352	8	160.9505	93.8467
	Y2387	8	161.1303	35.47339
	Y2390	8	161.1366	39.74627
	Y243	8	99.51222	259.6991
	Y2466	8	163.0714	72.80935
	Y2523	8	164.5089	125.9243

Y2555	8	165.1198	45.17086
Y2559	8	165.1638	184.5902
Y2692	8	169.0454	100.9399
Y2697	8	169.0844	32.18229
Y2698	8	169.0859	247.0734
Y2712	8	169.5471	99.96524
Y2733	8	170.0475	100.1474
Y2745	8	170.5151	37.4792
Y2767	8	171.0419	99.46879
Y291	8	101.0599	33.48412
Y3069	8	178.0507	100.3989
Y3091	8	178.5484	99.43263
Y3092	8	178.5524	101.3141
Y3620	8	191.9916	102.5628
Y3660	8	192.9748	119.5432
Y3757	8	195.0299	138.2702
Y3789	8	195.9305	100.25
Y3794	8	195.9681	256.9792
Y3866	8	197.9259	102.4193
Y3867	8	197.9317	75.53912
Y3933	8	199.135	145.206
Y3998	8	201.1234	68.0185
Y4007	8	201.5097	168.1027
Y4008	8	201.5456	98.06614
Y4258	8	206.9904	128.2644
Y4318	8	208.0417	46.79378
Y4389	8	210.0503	47.02396
Y4547	8	214.1071	26.63012
Y4618	8	215.927	270.5959
Y4619	8	215.9365	94.76308
Y4680	8	217.127	183.2382
Y4753	8	219.0417	100.1297
Y4784	8	219.5434	99.91241
Y4806	8	220.0392	92.36928
Y4807	8	220.044	100.1434
Y4872	8	221.2264	185.4001
Y4877	8	221.5608	157.0103
Y4913	8	222.5247	168.4916
Y4934	8	223.0254	168.8391
Y4963	8	223.5634	100.6485
Y5029	8	225.0472	54.43594
Y5075	8	226.0379	57.27588
Y5105	8	226.5586	99.23449
Y5125	8	227.0744	186.2414
Y5177	8	228.1956	20.79958
Y5182	8	228.4642	113.4397
Y5198	8	229.0099	100.0738

Y5218	8	229.5586	100.0633
Y5250	8	230.3558	256.6123
Y528	8	111.0747	88.9413
Y5315	8	232.0477	162.6578
Y5386	8	233.5311	101.1799
Y5544	8	236.9568	246.1465
Y5585	8	237.9551	84.53552
Y5591	8	238.0154	100.1911
Y5631	8	238.9742	75.12893
Y5665	8	239.9955	16.903
Y5820	8	243.9441	57.1034
Y5855	8	244.5073	99.39951
Y5905	8	245.9682	77.029
Y6075	8	250.534	90.31311
Y6189	8	253.5463	85.1018
Y6272	8	256.0605	95.08582
Y6293	8	256.5254	128.0373
Y6342	8	257.538	96.30068
Y6353	8	257.995	123.9675
Y6397	8	259.0211	229.2275
Y6404	8	259.1073	106.3363
Y6446	8	260.481	104.156
Y6671	8	266.3584	128.7366
Y6722	8	267.9509	112.784
Y6875	8	271.5217	260.9921
Y7076	8	276.7822	44.07644
Y7117	8	277.8826	260.2738
Y7124	8	278.0103	113.5019
Y7177	8	279.1919	52.54849
Y7218	8	280.235	241.9822
Y724	8	117.0659	59.44788
Y7275	8	282.0191	168.2218
Y728	8	117.074	68.30725
Y734	8	117.5043	80.50731
Y7445	8	286.151	91.98498
Y7536	8	288.9839	168.6639
Y7565	8	289.5514	100.1777
Y7652	8	291.1932	49.62151
Y7728	8	293.9072	76.73435
Y773	8	118.9674	265.6782
Y7776	8	295.9028	77.50989
Y7777	8	295.9231	100.6956
Y7783	8	296.0092	57.40424
Y7848	8	298.0528	120.302
Y7892	8	299.2003	22.99185
Y7969	8	301.1734	57.74684
Y8010	8	302.5745	155.825

		Y8041	8	303.5463	241.1295
		Y8201	8	308.5241	100.7706
		Y8272	8	310.5014	127.1317
		Y8380	8	313.6705	103.5768
		Y8754	8	325.6106	101.9765
		Y8764	8	326.0636	141.1938
		Y8803	8	327.0591	268.9265
		Y8813	8	327.2524	281.9462
		Y8831	8	327.9876	128.8241
		Y8886	8	329.0951	166.5266
		Y8917	8	330.0609	167.1546
		Y8971	8	331.9258	44.5822
		Y9052	8	334.0269	276.1428
		Y9063	8	334.1594	36.59048
		Y9242	8	340.0807	264.6369
		Y9296	8	342.0626	105.8434
		Y9342	8	343.2478	198.6055
		Y9395	8	345.0186	125.0858
		Y9463	8	347.0263	255.2723
		Y9558	8	350.8578	76.15943
		Y9583	8	351.5626	273.2877
		Y9633	8	353.2668	42.94601
		Y9802	8	359.5472	128.3116
		Y9817	8	360.0482	128.4016
		Y9915	8	363.9928	116.1151
		Y9922	8	364.0651	178.7949
		Y9993	8	366.5668	100.3351
X10	9 *Map2k3*	Y1026	9	126.047	48.57146
		Y11331	9	416.5576	100.9075
		Y13066	9	498.7914	50.67681
		Y13096	9	500.1755	24.73858
		Y14447	9	571.36	26.2684
		Y14690	9	585.6274	50.24542
		Y14888	9	596.366	22.95614
		Y15099	9	606.3276	267.6007
		Y15286	9	613.3543	23.66346
		Y16743	9	702.5577	35.05751
		Y1690	9	143.9969	155.5347
		Y17019	9	716.5177	23.28104
		Y17058	9	719.5411	23.96217
		Y17224	9	734.5753	21.10892
		Y17282	9	741.5255	22.72703
		Y17313	9	744.5851	23.35888
		Y17751	9	788.6758	279.1044
		Y18098	9	833.0128	53.45383
		Y18214	9	860.6109	23.67285
		Y3522	9	189.6193	264.7321

			Y5086	9	226.0988	25.21425
			Y5167	9	228.0962	25.62923
			Y5577	9	237.4995	107.9592
			Y5914	9	246.0649	57.00323
			Y6281	9	256.1098	26.20299
			Y7012	9	275.0093	94.79463
			Y7755	9	295.0305	53.4093
			Y8325	9	312.0502	198.4613
			Y8354	9	312.9805	79.66069
			Y8801	9	327.0487	73.5458
			Y9398	9	345.0455	99.93275
			Y9420	9	345.5472	100.4896
X4	10	*Csrp3*	Y10160	10	371.9385	70.32987
			Y10430	10	382.0737	198.9997
			Y10820	10	396.7002	49.70355
			Y10957	10	402.0545	63.00787
			Y10965	10	402.2666	27.16838
			Y11123	10	408.2353	60.31134
			Y11822	10	436.8935	53.10937
			Y12000	10	444.8533	50.41342
			Y12193	10	454.0403	50.74845
			Y12210	10	454.8377	48.12533
			Y12585	10	473.8016	52.41042
			Y12888	10	489.6153	264.7409
			Y13001	10	495.62	100.8871
			Y13134	10	502.1071	61.10858
			Y13149	10	502.7857	51.16946
			Y13150	10	502.7917	49.13369
			Y13240	10	508.3245	30.26367
			Y13417	10	518.3019	40.29371
			Y13468	10	522.0635	156.8124
			Y13676	10	530.9903	54.27355
			Y13683	10	531.4444	16.9901
			Y13791	10	536.8825	41.19551
			Y1395	10	136.0337	49.59869
			Y14125	10	553.4516	53.97354
			Y14218	10	558.8582	57.20764
			Y14743	10	588.7264	48.33733
			Y14872	10	595.5383	270.7631
			Y14914	10	597.6719	293.2437
			Y14942	10	599.336	24.38118
			Y14964	10	600.3376	61.04445
			Y15129	10	607.9719	54.72303
			Y15384	10	617.4156	56.91568
			Y15637	10	629.6074	50.84472
			Y15677	10	632.1527	221.5665
			Y15794	10	639.7606	293.3866

Y15805	10	640.1691	275.3742
Y15819	10	640.6499	48.6262
Y15837	10	641.5887	49.71768
Y16009	10	652.8239	55.16306
Y16360	10	676.4573	46.67034
Y16478	10	683.8412	268.1632
Y16556	10	689.053	280.9787
Y17045	10	718.4909	48.41855
Y17143	10	727.3355	72.21611
Y17314	10	744.6243	47.98665
Y17420	10	756.424	46.98924
Y17743	10	788.3845	47.40718
Y17902	10	806.4047	48.33222
Y17944	10	813.5192	22.29096
Y18116	10	835.1949	100.2964
Y18137	10	839.5653	26.55362
Y18196	10	856.4082	279.1661
Y18275	10	888.5116	280.7551
Y1837	10	147.1402	72.6449
Y2528	10	164.5445	64.25696
Y253	10	99.99047	78.85809
Y2725	10	170.0092	113.4121
Y2959	10	175.3386	120.6126
Y3059	10	177.9903	15.565
Y3860	10	197.5739	62.38973
Y3875	10	198.0075	27.39764
Y3984	10	200.9921	72.51728
Y4017	10	201.9986	16.03243
Y4381	10	209.9658	74.89175
Y441	10	107.047	73.11447
Y4436	10	211.0966	184.8118
Y5113	10	226.9857	49.08068
Y5559	10	237.0423	84.79311
Y5692	10	240.4641	91.8736
Y5841	10	244.0968	194.9471
Y6373	10	258.1759	101.2684
Y6500	10	261.9006	158.4287
Y6552	10	263.0695	29.36419
Y6660	10	266.0631	62.88154
Y6716	10	267.5755	64.64314
Y7181	10	279.3583	112.6436
Y7281	10	282.0682	70.81708
Y7572	10	289.6744	32.34715
Y7729	10	293.9227	100.7191
Y8661	10	322.5193	181.5506
Y9044	10	333.696	34.39881
Y9237	10	340.0245	61.48075

			Y9270	10	341.0669	60.0175
			Y9653	10	354.0699	197.4945
			Y9674	10	354.9413	44.11437
			Y9767	10	358.2437	22.03736
X13	11	*Smad3*	Y10019	11	367.2454	51.82602
			Y10075	11	369.2245	65.05714
			Y10134	11	371.0578	76.83536
			Y10401	11	381.0006	116.6869
			Y10503	11	384.7565	47.42796
			Y10554	11	386.1368	28.3257
			Y10646	11	389.1349	57.66185
			Y10681	11	391.0195	35.51182
			Y10708	11	392.1177	261.7971
			Y10912	11	400.1157	28.55312
			Y10940	11	401.1191	28.87623
			Y10946	11	401.7267	22.91075
			Y10960	11	402.1127	28.469
			Y10989	11	403.0152	168.7284
			Y11096	11	406.9909	34.92889
			Y11198	11	411.2506	42.91171
			Y11247	11	413.1375	48.5912
			Y11270	11	414.141	50.17197
			Y11280	11	414.524	261.2953
			Y11325	11	416.1106	68.82407
			Y11349	11	417.114	69.01977
			Y11369	11	418.1078	68.57361
			Y11370	11	418.1564	70.94728
			Y115	11	92.0019	85.61694
			Y11567	11	427.1173	31.02682
			Y11644	11	430.1264	43.83081
			Y11653	11	430.404	21.51784
			Y11669	11	431.0426	190.9245
			Y11757	11	434.0834	141.4419
			Y11764	11	434.4424	113.5048
			Y11770	11	434.6907	47.68014
			Y11782	11	435.0167	52.47205
			Y11922	11	441.7378	68.63284
			Y11936	11	442.2396	68.56657
			Y12106	11	449.544	100.2904
			Y12177	11	453.0906	272.0555
			Y12228	11	456.0508	88.30892
			Y12252	11	457.0732	136.0692
			Y12255	11	457.1671	65.75199
			Y12260	11	457.2715	47.79224
			Y12418	11	465.042	197.8846
			Y1256	11	132.4752	124.3291
			Y1258	11	132.4987	53.0108

Y12859	11	487.1511	140.1819
Y12902	11	490.0993	282.4631
Y13092	11	500.061	90.40849
Y13568	11	526.5585	247.2072
Y13885	11	542.1733	56.86584
Y14150	11	554.9518	290.4433
Y14175	11	556.5747	100.0895
Y1436	11	137.0718	45.46519
Y14670	11	584.5527	47.49085
Y1472	11	138.0661	18.23802
Y15220	11	611.4618	282.3914
Y15360	11	616.4565	25.55704
Y15584	11	626.6191	46.92009
Y15650	11	630.571	47.38193
Y16048	11	655.1129	187.291
Y16177	11	662.8666	56.12712
Y16332	11	674.5371	43.6598
Y16391	11	678.4947	46.75055
Y16452	11	682.4921	46.54709
Y16655	11	695.9087	99.33327
Y16720	11	701.0016	100.21
Y169	11	95.08552	249.4868
Y17072	11	721.0615	110.0676
Y17223	11	734.5661	18.16487
Y17367	11	750.0638	168.2905
Y1751	11	145.076	17.25434
Y17545	11	766.7733	52.13311
Y1780	11	146.0271	37.26014
Y1855	11	148.0229	35.21021
Y1856	11	148.0276	47.16678
Y1906	11	149.054	194.949
Y1966	11	150.9323	48.09366
Y2185	11	156.8452	40.34336
Y2264	11	158.8438	42.14462
Y2433	11	162.1399	39.62915
Y2471	11	163.0866	33.2362
Y259	11	100.0216	43.32936
Y2890	11	174.0408	79.12384
Y3040	11	177.1232	86.28149
Y3274	11	183.0529	88.14513
Y3543	11	190.0894	238.7627
Y3669	11	193.0415	29.91586
Y369	11	104.0579	37.27297
Y4087	11	203.1179	23.2939
Y4228	11	206.0853	72.72955
Y4482	11	212.5078	90.06638
Y4485	11	212.5545	87.43919

Y4527	11	213.5487	87.1965
Y4750	11	219.0265	55.14134
Y4808	11	220.0482	81.37006
Y4823	11	220.1373	135.6441
Y4849	11	221.0384	81.93908
Y4936	11	223.0326	83.47346
Y4946	11	223.1074	192.0613
Y497	11	110.0271	65.27735
Y5026	11	225.0355	58.30427
Y503	11	110.0714	90.4402
Y518	11	111.0305	66.53406
Y5333	11	232.1727	132.0589
Y5356	11	233.0376	86.99714
Y5480	11	235.5226	197.5677
Y5598	11	238.0634	28.46979
Y570	11	112.5257	267.8718
Y5815	11	243.5643	155.6241
Y6085	11	250.9626	261.1085
Y6098	11	251.0849	25.24145
Y6482	11	261.1809	74.31257
Y6635	11	265.2121	43.58333
Y6771	11	269.0952	27.13732
Y6802	11	270.0643	85.92524
Y6839	11	270.9776	57.40467
Y6876	11	271.5576	101.7148
Y6877	11	271.5906	56.91568
Y6892	11	272.0922	57.17043
Y6911	11	272.5883	56.93957
Y6977	11	274.0921	66.53554
Y7169	11	279.0934	167.5688
Y7204	11	280.0627	87.40019
Y7398	11	285.0837	87.67701
Y7679	11	292.1026	67.2772
Y7709	11	293.0951	24.20542
Y7726	11	293.5815	128.8258
Y7786	11	296.0301	124.0426
Y7836	11	297.9038	123.7712
Y7864	11	298.5668	89.51131
Y7902	11	299.5874	171.6849
Y8126	11	306.1297	78.26124
Y8258	11	310.1128	65.99285
Y8294	11	311.1168	66.48817
Y8429	11	314.9797	70.32857
Y8466	11	315.9145	125.4966
Y8527	11	317.6742	82.5821
Y8760	11	326.0215	84.56632
Y8836	11	328.0298	264.619

	Y8860	11	328.5949	87.70138
	Y8911	11	330.0255	112.8556
	Y8961	11	331.2841	201.6591
	Y9104	11	335.1982	47.80863
	Y9271	11	341.075	139.4313
	Y9356	11	343.861	48.47824
	Y9540	11	350.1197	72.3993
	Y9693	11	355.5301	126.2584
	Y9813	11	360.0033	46.65615

Table S6f. Metabolite tentative annotation in interaction network

m.z	rt	id	match_for	mz_differe	name	pathway	community
158.0998	26.14468	CE5653	M(C13)+2H	6.00E-04	4-oxo-9-cis	Vitamin A (	1
271.0814	87.7459	C00294	M(S34)+H[	-0.0025	Inosine	Purine met	1
496.3393	18.5974	clpndcrn	M+Na[1+]	-5.00E-04	clupanodo	Carnitine s	1
150.0081	55.68949	C07643	M+H+Na[2	0	4-Hydroxy	Drug meta	3
152.107	250.242	C02442	M+H[1+]	0	N-Methylt	Tyrosine m	3
125.0152	26.69958	C00245	M[1+]	5.00E-04	Taurine; 2-	Methionin	4
153.0699	26.01216	C02646	M(S34)+H[	-0.0013	4-Coumaryl alcohol; 4		4
191.0439	27.56357	C00153	M+HCOON	0.0012	Nicotinami	Vitamin B3	4
210.0581	33.79389	C01252	M(Cl37)+H	4.00E-04	4-(2-Amino	Tryptophan	4
254.0941	25.64987	C01736	M(C13)+H[	-0.0025	Nebularine;	Purine rib	4
475.3611	21.64843	clpndcrn	M(C13)+H[	-1.00E-04	clupanodo	Carnitine s	4
172.0967	27.79243	C00390	M+H+Na[2	-0.001	Ubiquinol;	Electron tr	5
143.9523	89.99791	C01755	M+HCOOK	7.00E-04	Thiocyanat	Methionin	6
169.9825	69.48443	C05528	M(C13)+H[	-0.0011	3-Sulfopyr	Methionin	6
413.2662	46.55564	CE5850	M[1+]	-0.003	11'-carbox	Vitamin E r	6
492.0109	96.74792	C00131	M+H[1+]	0.0028	dATP; 2'-D	Pyrimidine	6
120.0558	90.58705	C00581	M(S34)+H[	-0.0011	Guanidino	Glycine, se	7
132.0035	280.8324	C16196	M+H+Na[2	6.00E-04	1,2-Dihydroxynaphtha		7
146.1654	66.49199	C00315	M+H[1+]	2.00E-04	Spermidine	Beta-Alani	7
155.0911	172.5793	C03758	M(C13)+H[	0.0014	Dopamine;	Tyrosine m	7
164.0561	61.10343	C05839	M+2H[2+]	-0.0013	cis-beta-D-	Hexose ph	7
174.0759	25.69819	C01250	M+H[1+]	-2.00E-04	N-Acetyl-L-	Urea cycle,	7
190.0895	94.93539	C05548	M(Cl37)+H	5.00E-04	6-Acetami	Butanoate	7
190.1072	28.60267	C05933	M[1+]	6.00E-04	N-(omega)	Arginine ar	7
195.0876	22.47124	C16586	M[1+]	-0.0019	2-Phenyl-1	Drug meta	7
206.0456	104.1328	C02470	M+H[1+]	8.00E-04	Xanthuren	Tryptophan	7
210.0913	176.7211	C02325	M[1+]	0.0021	Sinapyl alcohol; Sinap		7
224.0895	52.07219	CE5626	M(C13)+H[	0.0022	salsolinol 1	Tyrosine m	7
231.0092	170.2675	C01127	M+HCOON	-0.0019	4-Hydroxy-	Arginine ar	7
265.1116	60.30374	C00378	M[1+]	-7.00E-04	Thiamin; T	Vitamin B1	7
288.592	109.1143	C14855	M+2H[2+]	-0.0016	4,5-Dihydr	Xenobiotic	7
296.0494	76.07999	C00325	M(C13)+2H	-7.00E-04	GDP-L-fuc	Glycosphir	7
315.1741	255.7263	C16300	M+K[1+]	0.0024	Stearidoni	Omega-3 f	7
344.9996	42.31	C00085	M+HCOOK	0.0013	D-Fructose	Pentose ph	7
127.0253	52.14304	C00245	M(C13)+H[	-1.00E-04	Taurine; 2-	Methionin	8
169.0859	247.0734	C00179	M+K[1+]	0.0013	Agmatine;	Arginine ar	8
225.0472	54.43594	C03680	M+HCOON	-0.001	4-Imidazol	Histidine m	8
303.5463	241.1295	C00096	M+2H[2+]	4.00E-04	GDP-mann	N-Glycan b	8
379.0343	100.3755	C16619	M[1+]	-9.00E-04	6-Thioguar	Drug meta	8
246.0649	57.00323	C11736	M[1+]	-3.00E-04	5-Fluorode	Drug meta	9
177.9903	15.565	C00870	M+K[1+]	6.00E-04	4-Nitrophe	Xenobiotic	10
211.0966	184.8118	C02325	M+H[1+]	1.00E-04	Sinapyl alcohol; Sinap		10
632.1527	221.5665	C03691	M(C13)+H[	-2.00E-04	CMP-N-gly	Aminosuga	10
137.0718	45.46519	1mncam	M[1+]	3.00E-04	1-Methyln	Vitamin B3	11

190.0894	238.7627	C05548	M(Cl37)+H	4.00E-04	6-Acetami	Butanoate	11
